# Microbiome composition modulates secondary metabolism in a multispecies bacterial community

**DOI:** 10.1073/pnas.2212930119

**Published:** 2022-10-10

**Authors:** Marc G. Chevrette, Chris S. Thomas, Amanda Hurley, Natalia Rosario-Meléndez, Kris Sankaran, Yixing Tu, Austin Hall, Shruthi Magesh, Jo Handelsman

**Affiliations:** ^a^Wisconsin Institute for Discovery, Madison, WI 53715;; ^b^Department of Plant Pathology, University of Wisconsin–Madison, Madison, WI 53706;; ^c^Microbiology Doctoral Training Program, University of Wisconsin–Madison, Madison, WI 53706;; ^d^Department of Statistics, University of Wisconsin–Madison, Madison, WI 53706

**Keywords:** metatranscriptomics, metametabolomics, microbiome, community interactions, secondary metabolism

## Abstract

Bacterial secondary metabolites are a major source of antibiotics and other bioactive compounds. In microbial communities, these molecules can mediate interspecies interactions and responses to environmental change. Despite the importance of secondary metabolites in human health and microbial ecology, little is known about their roles and regulation in the context of multispecies communities. In a simplified model of the rhizosphere composed of *Bacillus cereus*, *Flavobacterium johnsoniae*, and *Pseudomonas koreensis*, we show that the dynamics of secondary metabolism depend on community species composition and interspecies interactions. Comparative metatranscriptomics and metametabolomics reveal that the abundance of transcripts of biosynthetic gene clusters (BGCs) and metabolomic molecular features differ between monocultures or dual cultures and a tripartite community. In both two- and three-member cocultures, *P. koreensis* modified expression of BGCs for zwittermicin, petrobactin, and other secondary metabolites in *B. cereus* and *F. johnsoniae,* whereas the BGC transcriptional response to the community in *P. koreensis* itself was minimal. Pairwise and tripartite cocultures with *P. koreensis* displayed unique molecular features that appear to be derivatives of lokisin, suggesting metabolic handoffs between species. Deleting the BGC for koreenceine, another *P. koreensis* metabolite, altered transcript and metabolite profiles across the community, including substantial up-regulation of the petrobactin and bacillibactin BGCs in *B. cereus*, suggesting that koreenceine represses siderophore production. Results from this model community show that bacterial BGC expression and chemical output depend on the identity and biosynthetic capacity of coculture partners, suggesting community composition and microbiome interactions may shape the regulation of secondary metabolism in nature.

## Significance

Microbial communities have been implicated in human and plant disease and are essential to global biogeochemical cycles. However, our ability to reliably alter these communities is limited by insufficient understanding of the networks that drive community processes. The goal of this study was to understand how community membership alters secondary metabolism in a model microbial community. We found that community species composition affects expression of biosynthetic genes and abundance of metabolites. Dramatic changes were observed when the biosynthetic gene cluster of one metabolite, koreenceine, was deleted, suggesting that interspecies interaction networks may be driven by secondary metabolites. This work offers an approach to dissecting the flow of information through communities, which could lead to strategies for manipulating community function.

The composition of microbiomes, or microbial communities, influences the health of every ecosystem on Earth, exhibiting functions ranging from global nutrient cycling to determining the difference between host health and disease. Microbiomes are often highly complex, in part due to the organismal diversity and dynamic responses that they display. Microorganisms deploy correspondingly diverse gene products and metabolites, some of which mediate interactions between species or reveal community-specific, emergent phenotypes. Interspecies interaction networks shape ecosystem-level functions by facilitating competition, cooperation, and communication.

Organisms within microbiomes influence one another by producing, sensing, and responding to assorted metabolites (1). The chemical diversity of bacterial secondary metabolites generates a myriad of biological activities, including many exploited by humans as antibiotics, antivirals, anticancer agents, immunosuppressants (2), and pesticides (3). In nature, secondary metabolites play key roles in mediating microbial interactions, ranging from competitive to cooperative (e.g., antibiotics and quorum signals) and from general to specific (e.g., those with broad- and narrow-spectrum molecular targets) (4). The in situ roles of secondary metabolites remain difficult to characterize experimentally, although our emerging understanding suggests they may trigger coordinated responses among community members (5–12), including regulation of secondary metabolism (11–14). Moreover, little is known about the genes and metabolites important for bacterial fitness within multispecies communities, because most research in the history of microbiology has focused on organisms in isolation.

The genes involved in the assembly, transport, and regulation of secondary metabolites are often found adjacent to one another in bacterial genomes, forming biosynthetic gene clusters (BGCs) (15). Regulation of these gene clusters has been associated with bacterial developmental processes (16), sporulation (17, 18), responses to antibiotics (10, 18), and other signals and stressors (10). Many BGCs identified informatically are silent under traditional laboratory conditions, making them difficult to functionally characterize (19–22), especially since opportunities for organisms to interact are dependent on many factors (23, 24). Consequently, genome mining efforts suggest that the vast majority of BGC diversity has yet to be described functionally (19–22), and screens that manipulate stresses and nutrients to elicit silent BGCs are often untargeted (10, 18). Although information about the roles of BGCs and their products in pure culture is incomplete, knowledge of their roles in microbial communities is even more sparse. Microbial communities can span many orders of complexity, and each member may have dozens of BGCs in its genome. Secondary metabolites might, in turn, affect BGC expression in other species across the community interaction network. Thus, reductionist approaches are needed to unravel the complexity of BGC regulation in communities and their roles in community interactions (25).

We previously described THOR (the hitchhikers of the rhizosphere), a three-species model community comprising bacteria that interact in both the field and laboratory (12, 26). Two of THOR’s members, *Flavobacterium johnsoniae* UW101 (*Fj*) and *Pseudomonas koreensis* CI12 (*Pk*), were coisolated with the third member, *Bacillus cereus* UW85 (*Bc*), when it was cultured from the rhizospheres of field-grown soybean plants (26). The members are well studied individually and display several cooperative and competitive interactions. In the field, for example, treatment of seeds with *Bc* results in large increases of *Flavobacterium*–*Cytophaga* group bacteria [e.g., *Fj* (27)]. This may be due, in part, to the release of peptidoglycan fragments by *Bc*, which provides a necessary carbon source for *Fj* in root exudate (28). The three-member community exhibits emergent properties (i.e., those that could not be predicted from pure cultures or pairwise interactions), including augmented biofilm production that is decoupled from overall growth (26). THOR’s members are also prolific producers of secondary metabolites. *Bc* metabolites zwittermicin and kanosamine are antagonistic toward oomycete plant pathogens in both laboratory and field experiments (29–33). The genomes of THOR’s members contain several other BGCs, including the *Bc* siderophores petrobactin (34) and bacillibactin (34), the *Fj* antioxidant flexirubin (35), and the *Pk* antimicrobials lokisin (36) and koreenceine (37). *Pk* inhibits the growth of *Fj* with koreenceine (26, 37), but, when all three THOR members are cultured together (i.e., *Bc* + *Fj* + *Pk*), *Bc* protects *Fj* from *Pk* inhibition (26, 37). We previously reported that THOR community composition influences expression of many functional gene categories, including secondary metabolism, and deletion of *Pk* koreenceine can cause global changes in expression (12).

Despite the importance of metabolites in mediating community interactions, there remains a lack of governing principles regarding secondary metabolism in communities. We hypothesized that interactions between THOR strains modulate secondary metabolism in one another. Here, we characterize interspecies interactions in THOR that influence BGC expression and their corresponding metabolite products. A pathway-level metatranscriptomic analysis identified BGCs in THOR whose expression is modulated by other community members and showed that *Pk* koreenceine suppresses expression of *Bc* siderophore BGCs. Metametabolomic analyses quantified differences in metabolites, revealing a newly described molecular feature detected only when all three members are grown together. These results provide insight into how community metabolomes are shaped by interspecies interactions and provide a framework for studying secondary metabolism regulation across species in microbial communities.

## Results

### Genome Mining Reveals the THOR Community’s Biosynthetic Potential.

To define the THOR community’s BGC interaction network, we first sought to annotate the genome-wide biosynthetic potential of each member and to identify all BGC loci. We analyzed the genomes of *Bc*, *Fj*, and *Pk* via antiSMASH (antibiotics and secondary metabolite analysis shell) (38) and identified a total of 100 putative BGC loci (Table 1 and *SI Appendix*, Tables S1 and S2): 44 in *Bc*, 28 in *Fj*, and 28 in *Pk*. Manual review of the *Bc* genome revealed an additional four genomic regions involved in secondary metabolite biosynthesis: two putative bacillithiol biosynthetic regions (39, 40) and two putative nicotinate biosynthetic regions (41) (*SI Appendix*, Table S1). Of the 6,180 predicted protein-encoding genes in *Bc*, 2.3% were annotated as COG-Q (i.e., clusters of orthologous genes, category Q, secondary metabolism), and 7.0% were annotated as SMCOG (antiSMASH secondary metabolism COG; Table 1). For the 5,199 protein-encoding genes in *Fj* and the 5,865 in *Pk*, 2.0% and 2.8% were annotated as COG-Q and 4.1% and 4.6% as SMCOG, respectively (Table 1).

**Table 1.** Genomic summary of secondary metabolism in strains in this study

Strain	Abbreviation herein	Assembly accession (public database)	Protein- encoding genes	COG-Q annotated genes (%)	SMCOG annotated genes (%)	AntiSMASH regions	Previously described secondary metabolites (known functions) (reference)
*Bc*	*Bc*, B	Ga0417192 (Joint Genome Institue-Integrated Microbial Genomes and Microbiomes)	6,180	142 (2.3%)	433 (7.0%)	44	Zwittermicin (antibiotic) (33, 42) Kanosamine (antibiotic) (33) Petrobactin (siderophore) (34) Bacillibactin (siderophore) (34, 41) Pulcherriminic acid (siderophore) (43) Bacillithiol (redox balance/resistance) (39, 40) Kurstakin (antibiotic/antifungal) (44)
*Fj*	*Fj*, F	GCA_000016645.1 (National Center for Biotechnology Information-GenBank)	5,199	105 (2.0%)	215 (4.1%)	28	Flexirubin (antioxidant) (35)
*Pk*	*Pk.*, K	Ga0417193 (Joint Genome Institue-Integrated Microbial Genomes and Microbiomes)	5,865	163 (2.8%)	272 (4.6%)	28	*N*-acetylglutaminylglutamine amide (osmotic stress) (50) Lokisin (antibiotic/antifungal) (36, 49) Koreenceine (antibiotic) (37)

The *Bc* genome contained 10 previously described BGC loci, including those encoding biosynthetic machinery for zwittermicin/kanosamine (33, 42), petrobactin (34), bacillibactin (34, 41), pulcherriminic acid (43), bacillithiol (39, 40), and kurstakin (44) (Table 1 and *SI Appendix*, Tables S1 and S2). Of the remaining 38 BGC loci in *Bc*, 26 were putative saccharide and/or fatty acid BGCs and 12 were of other biosynthetic types, including nonribosomal peptide synthetases, polyketide synthases, ribosomally encoded and posttranslationally modified peptides (RiPPs), and others. We note this distinction because putative saccharide and fatty acid–type BGC identifications in antiSMASH are low-confidence annotations (45). Two *Bc* RiPP loci are distinct from, but bear resemblance to, heterocycloanthracin (46) and thermocellin (47), respectively, suggesting they may encode biosynthetic machinery for related molecules. Of the 28 BGC regions in the *Fj* genome, only flexirubin (35) was described previously. The remaining 27 include 21 putative saccharide and/or fatty acid BGCs and 6 other biosynthetic types coding for terpenes, polyketides, a putative fulvivirgamide-like siderophore (48), and others. The *Pk* genome contained BGCs that encode the biosynthetic machinery for lokisin (36, 49), koreenceine (37), and *N*-acetylglutaminylglutamine amide (50). Of the remaining 25 *Pk* BGCs, 17 were putative saccharide and/or fatty acid loci, and 8 were BGCs of other types. A putative maturation locus and two putative precursor loci in *Pk* are distinct from, but bear resemblance to, pyoverdine clusters (51), suggesting they may encode biosynthetic machinery for related molecules.

### Community Partners Shape the Interspecies Regulatory Dynamics of BGCs.

**Fig. 1.**
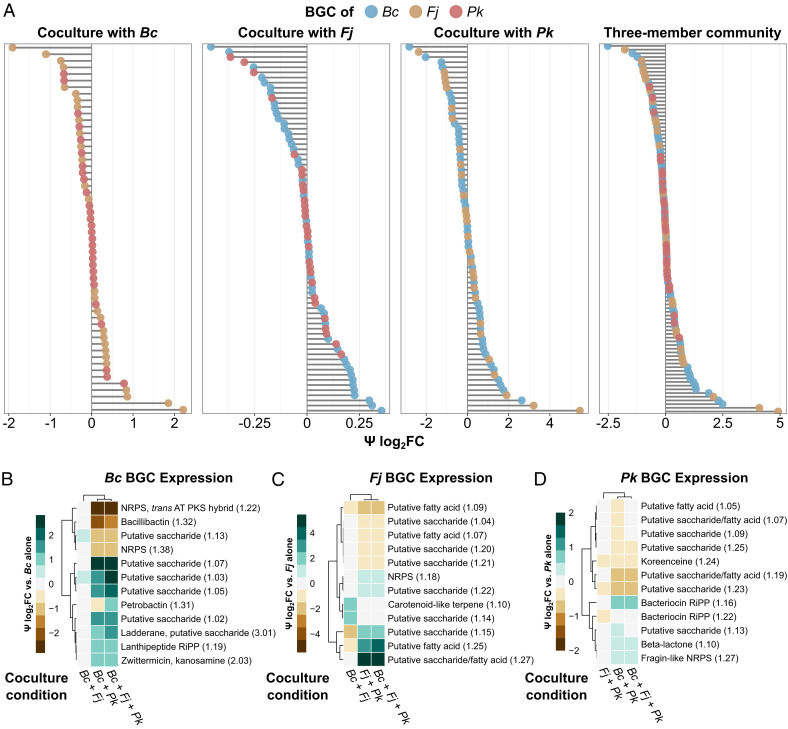
Interspecies coculture affects the expression of BGCs in a model community. (*A*) BGCs of *Bc*, *Fj*, and *Pk* are expressed at different levels when in pairwise or three-member coculture with each other. Psi log_2_(fold change) is calculated as the fold change of the coculture condition compared to the BGC-containing strain in monoculture. BGCs of *Bc*, *Fj*, and *Pk* are shown in blue, tan, and red, respectively. (*B*–*D*) The 12 most differentially expressed BGCs of *Bc* (*B*), *Fj* (*C*), and *Pk* (*D*) as heatmaps, with green indicating increased expression in coculture, brown indicating decreased expression in coculture, and light gray indicating no change. Columns denote coculture conditions, and rows denote individual BGCs and their antiSMASH region numbers in parentheses. *SI Appendix*, Fig. S2 shows all BGCs in all three strains.

We next sought to quantify the expression of BGCs in THOR and define how BGC expression changes in the presence of other THOR members. We generated transcriptomic profiles of *Bc*, *Fj*, and *Pk* in monoculture and compared them to profiles of pairwise cocultures and the three-member community (Fig. 1 and *SI Appendix*, Figs. S1 and S2 and Tables S3 and S4). Pairwise coculture with *Bc* or *Fj* generally did not show much difference in BGC expression in other species, except for a small modulation in expression of some *Fj* BGCs by *Bc* (Fig. 1 and *SI Appendix*, Fig. S2 and Table S4). In contrast, pairwise coculture with *Pk* substantially affected BGC expression for many loci in both *Bc* and *Fj* (Fig. 1 and *SI Appendix*, Fig. S2 and Table S4). *Bc* and *Fj* BGCs were differentially expressed in the three-member community compared to monoculture (Fig. 1 *A*–*C* and *SI Appendix*, Fig. S2 and Table S4), whereas *Pk* BGC expression was largely unchanged in the three-member coculture (Fig. 1 *A* and *D* and *SI Appendix*, Fig. S2 and Table S4). *Pk* reduced expression of the *Bc* bacillibactin BGC (−2.03 Ψ log_2_FC [effect size log_2_ fold change vs. monoculture]) and increased expression of the *Bc* zwittermicin/kanosamine locus (0.88 Ψ log_2_FC; Fig. 1*B* and *SI Appendix*, Fig. S2 and Table S4). For the majority of *Bc* BGCs, the expression changes observed in *Pk* coculture are also observed in the three-member community (Fig. 1*B* and *SI Appendix*, Fig. S2 and Table S4), suggesting that the presence of *Pk* is a major factor in *Bc* BGC expression, whether in pairwise coculture or in the three-member community. One exception is the BGC for petrobactin, which experiences little change in pairwise coculture with either *Fj* or *Pk* (0.15 Ψ log_2_FC and −0.27 Ψ log_2_FC, respectively) but exhibits a community-unique transcriptional signature with higher expression in the three-member coculture (1.22 Ψ log_2_FC; Fig. 1*B* and *SI Appendix*, Fig. S2 and Table S4).

**Fig. 2.**
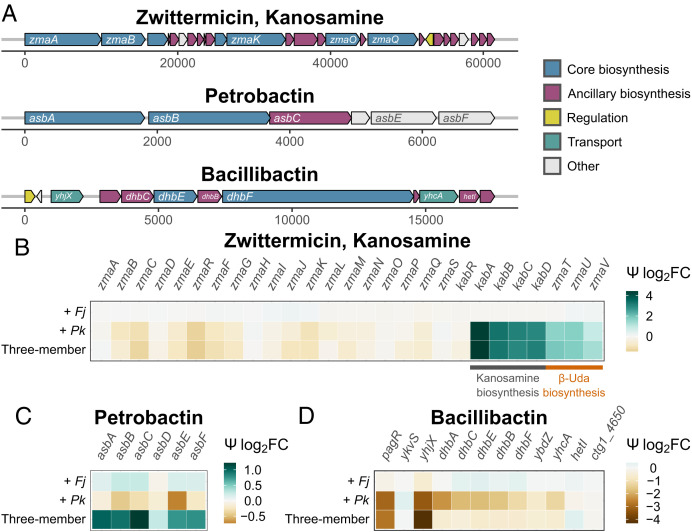
Several BGCs of *Bc* exhibit different expression patterns in coculture. (*A*) Gene maps of the BGCs of zwittermicin/kanosamine, petrobactin, and bacillibactin are colored based on antiSMASH annotations. (*B*–*D*) Gene expression for zwittermicin/kanosamine, petrobactin, and bacillibactin BGCs, with green indicating increased expression in coculture, brown indicating decreased expression in coculture, and light gray indicating no change. Rows indicate *Bc* coculture condition, and columns indicate individual *Bc* open reading frames.

At the individual gene level, the zwittermicin/kanosamine locus in *Bc* exhibits two distinct patterns (Fig. 2). First, most genes involved in zwittermicin biosynthesis did not differ among the coculture conditions tested (Fig. 2*B*), including all of the nonribosomal peptide synthetase and polyketide synthase genes (*zmaOKFABCQ*), whose products condense building blocks to assemble the zwittermicin protomolecule and the cleavage genes (*zmaLM*) required to generate mature zwittermicin A (33). In cocultures where *Pk* is present, the *kabABCD* biosynthetic genes for kanosamine (33) are up-regulated along with the *zmaTUV* genes involved in the biosynthesis of the unusual zwittermicin precursor β-ureidoalanine (β-Uda; Fig. 2*B*). In pairwise coculture with *Pk*, genes *kabA*,* kabB*,* kabC*, and* kabD* increase expression at 4.36, 3.29, 3.05, and 2.93 Ψ log_2_FC, respectively, and genes *zmaT*,* zmaU*, and* zmaV* increase expression at 1.60, 1.69, and 0.90 Ψ log_2_FC, respectively (Fig. 2*B* and *SI Appendix*, Table S3).

Genes involved in the biosynthesis of the *Bc* siderophore petrobactin are expressed relatively uniformly across its BGC (Fig. 2 *C* and *D*). When in pairwise coculture with either *Fj* or *Pk*, the levels of expression of petrobactin genes are similar to those in monoculture, whereas, in the three-member community, these genes are up-regulated (Fig. 2*C*). An exception is the *asbD* gene, which encodes a carrier protein, for which we detected little change in expression across conditions. This is likely due to technical difficulties of mapping transcripts to very short reading frames. The strongest up-regulation in the three-member coculture is in genes *asbABC* (Fig. 2*C*). The NRPS-independent siderophore synthases *asbA* and *asbB*, which condense spermidine and citric acid units, increase at 1.00 and 0.92 Ψ log_2_FC, respectively (Fig. 2*C* and *SI Appendix*, Table S3). The NRPS-like gene *asbC*, which installs a dihydroxybenzoate moiety on the citryl-spermidine, increases at 1.22 Ψ log_2_FC (Fig. 2*C* and *SI Appendix*, Table S3).

Expression of genes in the *Bc* BGC for bacillibactin is largely unchanged when *Bc* is cocultured with *Fj*, but is down-regulated by *Pk*, either in pairwise coculture or in the three-member community. Directly upstream of the bacillibactin NRPS is a gene annotated as a major facilitator superfamily (SMCOG1137) transporter. It has similarity to the *Escherichia coli* gene *yhjX* (44.8% protein identity). This gene exhibits the most change in expression at the bacillibactin locus, with −3.29 and −4.30 LFC Ψ log_2_FC in the *Pk* and three-member cocultures, respectively (Fig. 2*D* and *SI Appendix*, Table S3). *E. coli*’s *yhjX* regulates growth and adaptive resistance in the presence of subinhibitory concentrations of antibiotics (52). The bacillibactin biosynthesis genes are also down-regulated in the presence of *Pk* and in three-member cocultures (Fig. 2*D* and *SI Appendix*, Table S3).

### Metametabolome Profiles Are Determined by Community Composition.

**Fig. 3.**
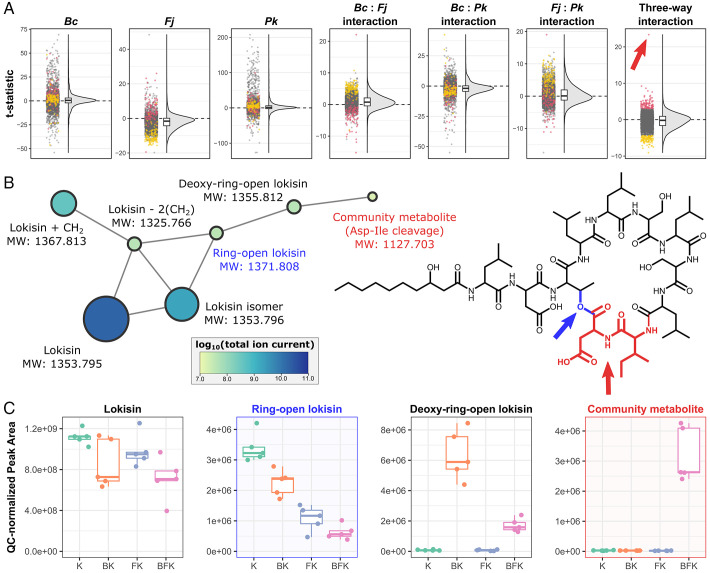
Coculture shapes the metabolomes of a model multispecies community. (*A*) The *t* statistics for each LC-MS molecular feature are shown for the effect of each species (*Bc*, *Fj*, or *Pk*), or pairwise or three-way interaction terms. Individual molecular features are shown as dots (left), and distributions are shown as frequency curves and boxes (right). Box heights denote the interquartile range, and the center line corresponds to the median. Molecular features in the top and bottom 2.5 percentile of the three-way interaction term are shown as red and yellow, respectively. A “community metabolite” with the highest estimated effect size in the three-way interaction is labeled with a red arrow. (*B*) LC-MS/MS subnetwork of *Pk*-produced lokisin and related molecular features is shown to the left. Molecular features are shown as nodes with edges connecting those with similar spectra. Nodes are sized and colored corresponding to their abundance across all sample conditions. Calculated MWs are shown. The structure of lokisin is shown to the right. Regions of lokisin corresponding to a putative ring-open form (blue atoms and arrow) or putative amino acid cleavages (community metabolite; red atoms and arrow) are highlighted. (*C*) Peak area of selected molecular features across *Pk*-containing conditions (B, *Bc*; F, *Fj*; K, *Pk*; combinations denote cocultures). The molecular features for the putative ring-open form of lokisin and the community metabolite are highlighted in blue and red, respectively.

To investigate the global metabolomic changes that result from these different coculture conditions, we performed untargeted liquid chromatography–mass spectrometry (LC-MS) metabolomics on *Bc*, *Fj*, and *Pk* monocultures and generated metametabolomic profiles of all pairwise and triple combinations (five replicates for each condition; Fig. 3 and *SI Appendix*, Figs. S3–S5 and Tables S5 and S6). A total of 4,904 molecular features (distinct from those found in media controls) were identified across all conditions tested. A principal component analysis of these LC-MS profiles grouped samples according to their monoculture or coculture condition (*SI Appendix*, Fig. S4). A loadings analysis revealed molecular features that discriminate across principal components, including features only seen in the three-member community (*SI Appendix*, Fig. S4*D*). Samples of the same coculture condition were highly correlated, and most hierarchically cluster together (*SI Appendix*, Fig. S5). Ninety-three molecular features are only seen when two or more members are cultured together (1.9% of total), 18 of which are only seen in the three-member community (0.37% of total; *SI Appendix*, Table S5). The *t* statistics were calculated to test whether *Bc*, *Fj*, *Pk*, or their pairwise and three-way interactions had effects on molecular feature abundances (higher absolute values of *t* indicate a stronger effect; Fig. 3*A*). Fig. 3*A* shows the *t* statistics for molecular features, highlighting the upper and lower 2.5 percentiles of the three-way interaction *t* statistics in red and yellow, respectively. Pairwise and three-way interaction terms have many molecular features with high *t* statistics, indicating that a model including pairwise and three-way interactions between species better explains the observed data for those features. Molecular features that have a large, positive, three-way interaction effect (red) tend to have a negative effect in the pairwise *Fj* interactions (i.e., *Bc*:*Fj* and *Fj*:*Pk*). Reciprocally, molecular features that have a large, negative, three-way interaction effect (yellow) tend to have a positive effect in the pairwise *Fj* interactions. Together, these results suggest that the impacts of *Fj* on metametabolomic profiles may be community dependent.

### A Metabolite Detected Only in the Three-Member Community.

One molecular feature (calculated molecular weight [MW] of 1,127.7028 Da) was strongly associated with the three-member community. It exhibited the highest three-way interaction effect, with a *t* statistic of 23.53 (Fig. 3*A*, red arrow). Further investigation of this feature (referred to henceforth as “community metabolite”) through molecular networking of tandem LC-MS (LC-MS/MS) profiles (Fig. 3*B*) showed that it was part of an LC-MS/MS subnetwork containing the *Pk* antibiotic lokisin (MW 1,353.795 Da) and related metabolites (Fig. 3*B*). Every condition containing *Pk* exhibited similar abundances for features with MWs corresponding to lokisin, a lokisin isomer, lokisin plus a methyl group, and lokisin minus two methyl groups (Fig. 3 *B* and *C* and *SI Appendix*, Tables S5 and S6). An MW of 1,371.808 Da corresponding to an open ring form of lokisin was detected in all *Pk* conditions, with the highest abundance in *Pk* monoculture, less in pairwise cocultures with *Bc* or *Fj*, and the least abundance in the three-member community coculture (Fig. 3 *B* and *C*, blue highlights). Networked to this ring-opened form is a feature of 1,355.812 Da, which corresponds to a deoxy version that is detected at its highest abundance in *Bc*–*Pk* pairwise coculture and at lower levels in the three-member community coculture. Networked to this is the community metabolite, whose MW is 228.11 Da less than the deoxy-ring-open lokisin, which is consistent with a cleavage of the terminal aspartic acid and isoleucine moieties (Fig. 3 *B* and *C*, red highlights).

### Metametabolome and BGC Expression Profiles Are Modulated by *Pk* Koreenceine.

Previous studies indicated that the antibiotic koreenceine produced by *Pk* could trigger transcriptional responses in THOR (12), so we sought to quantify the effect of koreenceine on BGC expression and metabolome profiles by comparing multispecies cocultures containing either *Pk* wild-type or a koreenceine BGC-deletion mutant [Δ*kecA-K*::*tetRA* (37)] (*SI Appendix*, Figs. S5 and S6 and Tables S5 and S7). In LC-MS metabolome profiles, 337 (71.9%) of nonmedia molecular features were shared between wild-type *Pk* and Δ*kecA-K*::*tetRA* monocultures, with 109 (23.2%) and 23 (4.9%) molecular features that were not detected in the media alone and unique to the wild type or mutant, respectively (*SI Appendix*, Table S5). In the *Pk* wild-type and mutant pairwise cocultures with *Bc*, there were 426 (78.2%) shared nonmedia molecular features, and 86 (15.8%) and 33 (6.1%) nonmedia molecular features unique to the wild type and mutant, respectively (*SI Appendix*, Table S5). Nonmedia molecular features from pairwise *Fj* cocultures were similarly distributed, with 357 (70.4%), 136 (26.8%), and 14 (2.8%) molecular features either shared, unique to wild type, or unique to mutant, respectively (*SI Appendix*, Table S5). In three-member communities with either wild-type or mutant *Pk*, 425 (72.9%), 119 (20.4%), and 39 (6.7%) were either shared, unique to wild type, or unique to mutant conditions, respectively (*SI Appendix*, Table S5). Overall, the profiles generated from communities containing the mutant are generally correlated with their cognate wild-type condition (e.g., *Bc* + *Pk* wild type hierarchically clusters with *Bc* + Δ*kec Pk*), yet still form distinct hierarchical groupings (*SI Appendix*, Fig. S5 and Table S5).

**Fig. 4.**
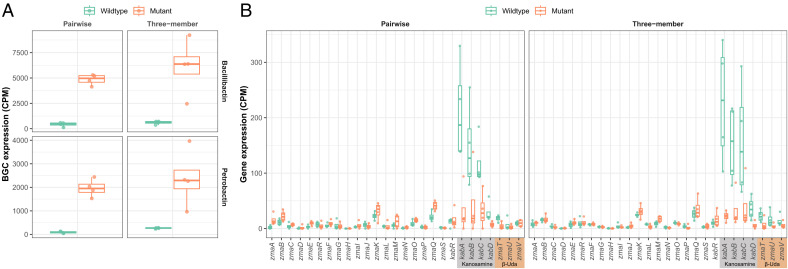
Koreenceine produced by *Pk* modifies BGC expression in *Bc*. (*A*) BGC expression (transcript CPM) is shown for *Bc* siderophores bacillibactin (*Top*) and petrobactin (*Bottom*) in either *Bc*–*Pk* pairwise (*Left*) or *Bc*–*Fj*–*Pk* three-member coculture (*Right*). Cocultures with wild-type *Pk* are shown in green, and cocultures with koreenceine-null mutant *Pk* are shown in orange. (*B*) At the zwittermicin/kanosamine BGC locus, genes involved in the biosynthesis of kanosamine (*kabABCD*) and β-Uda (*zmaTUV*) are up-regulated when *Bc* is cocultured with wild-type *Pk* (green). Up-regulation of *kabABCD* and *zmaTUV* is abolished when *Bc* is cocultured with koreenceine-null mutant *Pk* (orange).

*Bc* BGCs petrobactin and bacillibactin were more highly expressed in cocultures with the koreenceine mutant compared to those with *Pk* wild type (Fig. 4*A* and *SI Appendix*, Table S7). Bacillibactin and petrobactin expression was 1,041% and 2,123% higher, respectively, in coculture with the koreenceine mutant than with *Pk* wild type (Fig. 4 *A*, *Left*). In the three-member community cocultures, bacillibactin and petrobactin expression was 917% and 803% higher, respectively, in the mutant than in the wild type (Fig. 4 *A*, *Right*). When *Bc* was cocultured in pairwise or in the three-member cocultures with wild-type *Pk*, biosynthetic genes for kanosamine (*kabABCD*) and β-Uda (*zmaTUV*) are up-regulated (Figs. 2*B* and 4*B*), whereas cocultures containing the koreenceine mutant do not exhibit the same up-regulation (Fig. 4*B*), suggesting that the up-regulation of these *Bc* genes when in coculture with *Pk* may be linked to koreenceine. These results suggest that koreenceine may have major impacts on the regulation of BGC expression in other species, and future study is warranted to investigate mechanisms underlying community changes caused by koreenceine and other secondary metabolites.

## Discussion

We present a global metatranscriptional and metametabolomic view of secondary metabolism in a model microbial community and build on our previous study of transcriptional profiles of individual genes (12) by exploring changes in expression of entire gene clusters and production of metabolites. Several surprising results indicate that secondary metabolism is highly responsive to life in a multispecies community. We found that the three community members, *Bc*, *Fj*, and *Pk*, differ dramatically in both their effect on BGC expression in the other members and in their responses to others. *Fj* behaves as the “listener” of the community—mounting complex metabolic responses to the other members but producing few metabolites that influence them in turn. *Pk* is the “driver” of the community, producing a cascade of metabolites and eliciting large changes in BGC expression in *Bc* and *Fj*. *Bc* is the community “centrist” with moderate effects on and responses to the other members.

The three-member community has emergent properties that are not predicted from solitary or pairwise culture. For example, expression of the petrobactin BGC in *Bc* increases only in the three-member community; pairwise culture with either *Fj* or *Pk* does not affect petrobactin expression. Of note is the finding that one metabolite appears only in the complete community. Based on the molecular features found only in pairwise or three-member coculture conditions, we propose that the *Pk* antibiotic, lokisin, is modified through molecular handoffs between community members who each contribute a chemical modification. Alternatively, *Pk* may produce different versions of lokisin in response to its community but given the connectivity patterns of the lokisin LC-MS/MS subnetwork, we suggest the molecular handoff model in which lokisin is modified in the three-member community is more parsimonious.

Parts of pathways may be regulated differently from one another. This was illustrated by analysis of the zwittermicin biosynthetic pathway, which has long been of interest due the unusual features of its biosynthesis (33). Zwittermicin and kanosamine biosynthesis are linked, and the two antibiotics act synergistically against gram-negative bacteria and oomycete plant pathogens (29). The *Bc* strain UW85 used in this study as well as 43% of the 98 publicly available *Bacillus* genomes that contain the zwittermicin BGC also carry the kanosamine biosynthesis genes (*kabABCD*) flanked by *zmaS* and *zmaT*, parts of the zwittermicin BGC. In the present study, we found that most of the zwittermicin BGC is unaffected by community composition, but the kanosamine BGC and the neighboring downstream fragment of the zwittermicin BGC (*zmaTUV*) are strongly up-regulated in the presence of *Pk*. We hypothesize that *kabR*, which is in the kanosamine cluster and annotated as a *lacI* family negative regulator, represses expression of the kanosamine cluster and *zmaTUV*, and *Pk* derepresses their expression. Further work will identify the biological outcomes of coordinated regulation of these antibiotics.

Modeling metametabolomes revealed that three-way and pairwise interaction terms often have large effect sizes. This suggests that community metabolomics cannot be extrapolated from monoculture experiments alone. Differences between molecular feature *t* statistics in *Fj* pairwise and three-way interactions may be due to responses in *Fj* itself or changes elicited in other members. We hypothesize the former is more likely, as previous work has described large transcriptional shifts in *Fj* when in coculture with *Bc*, *Pk*, or both, with relatively minor transcriptional differences observed in *Bc* and *Pk* (12). Although lokisin itself is produced by *Pk*, we observe molecular features only in pairwise or three-member coculture conditions. This may suggest that *Pk* produces different versions of lokisin in response to its community or that there are molecular handoffs between community members to tailor the molecule. Given the connectivity patterns of the lokisin LC-MS/MS subnetwork, we hypothesize the latter is more parsimonious.

The presence or absence of the *Pk* BGC for koreenceine has large effects on BGC expression in the other community members. Compared to cocultures with wild-type *Pk*, the petrobactin and bacillibactin BGCs of *Bc* are strongly up-regulated in cocultures with a koreenceine-deficient mutant, suggesting that koreenceine represses the expression of these *Bc* siderophore BGCs. Previous studies in *Bacillus anthracis* show that expression of bacillibactin genes is highly sensitive to iron availability, and petrobactin can be regulated in response to both iron and oxygen variation (53), so these mechanisms may play a role in how koreenceine modulates their expression. However, since koreenceine affects the transcription of genes involved in many cellular processes (12), repression of *Bc* siderophores by koreenceine may be more complex or an indirect effect of koreenceine production. Similarly, the capacity of *Pk* to make koreenceine corresponds to the up-regulation of kanosamine biosynthetic genes *kabABCD* and β-Uda biosynthetic genes *zmaTUV*: wild-type *Pk* up-regulates these genes, and a koreenceine-deficient *Pk* mutant does not. This is particularly intriguing because β-Uda is a signature feature of zwittermicin biosynthesis that is rarely used in other pathways. Thus, further work to dissect the role of koreenceine induction of β-Uda expression and synthesis of zwittermicin will provide necessary answers to the puzzle of zwittermicin biosynthesis and its role in community behavior.

## Conclusions

THOR is a unique model community with at least 10 biological interactions among the members demonstrated in the field or in culture. The biological interactions, relevance to natural communities, and tools available for its study make THOR an exceptional model for exploring microbial interactions. Here we show that bacteria display dramatically different metabolic behavior in a community than in solitary culture. This is consistent with the hypothesis that, in microbial communities where organisms face complex chemical and environmental gradients, compounds designated as antibiotics by humans may have other functions. This seems especially likely for compounds produced at concentrations well below those required for growth inhibition (5). This work provides an important step in understanding the vast networks that unify communities and differentiate them from pure cultures. Research into these largely unstudied communities is likely to be rewarded with abundant discoveries that advance the understanding of microbial ecology.

## Materials and Methods

### DNA Extraction.

The publicly available, finished genome was used for *Fj* (accession GCA_000016645.1) (35). Since only fragmented public assemblies were available for *Bc* and *Pk*, complete genomes were generated via Pacific Biosciences DNA sequencing. Briefly, overnight cultures were washed and resuspended in 10 mL of TE (10 mM Tris; 1 mM (ethylenedinitrilo)tetraacetic acid; pH 8.0); 270 μL of lysozyme (100 mg/mL) was added for lysis and incubated on a rocker at 37 °C for 30 min; 540.5 μL of 10% sodium dodecyl sulfate and 108 μL of Proteinase K (10 mg/mL) was added to each and rocked to mix. Samples were incubated at 56 °C for 1 h. Then 1.35 mL of 5M NaCl and 1.35 mL of cetyl trimethylammonium bromide (heated to 56 °C) were added to each sample and mixed before incubation at 65 °C for 10 min. Four milliliters of chloroform was added and mixed. Samples were centrifuged (4,000 rpm, 30 min). Four milliliters of phenol:chloroform:isoamyl alcohol was added to new 50-mL tubes, and the aqueous layer from centrifuged tubes was added (approximately 8 mL) before mixing. After centrifugation (4,000 rpm, 30 min), 4 mL chloroform was added to new tubes, and the aqueous layer was transferred and mixed as before. After another centrifugation (4,000 rpm, 30 min), the aqueous layer was transferred to new 15-mL conical tubes, and 0.6 volume cold isopropanol was added and mixed gently. Samples were centrifuged (6,000 × *g*, 30 min, 4 °C), and the supernatant was discarded. Ten milliliters of 70% cold ethanol was added, the samples were centrifuged (4,000 rpm, 30 min), and the supernatant was discarded. Samples were air dried for ∼1 h. The dried pellet was resuspended in 170 μL of DNase-free water and gently mixed via pipette. Ten microliters of RNase A was added to each tube and gently mixed. Tubes were flash spun for 5 s and incubated at 37 °C for 1 h. Then 1/10 volume (17.25 μL) 3M sodium acetate was added and gently mixed; 2.5× volume (474.4 μL) 100% ethanol was added and gently mixed. Samples were stored at −20 °C overnight. Samples were centrifuged (12,000 rpm, 30 min, 4 °C) to pellet DNA, and the supernatant was discarded. Seven hundred microliters of 70% ethanol was added, and samples were centrifuged (12,000 rpm, 5 min, 4 °C). Excess ethanol was removed via aspiration, and samples were left to air dry for 30 min. The quality of genomic DNA was checked on a 1% agarose gel.

### Genome Sequencing, Assembly, and BGC Identification.

DNA sequencing was performed at the Joint Genome Institute by Pacific Biosciences Sequel II, and genomes were assembled via HGAP (hierarchical genome assembly process) 4 (54). *Bc* and *Pk* assemblies were deposited on JGI-IMG/M (Joint Genome Institute-Integrated Microbial Genomes and Microbiomes) as accessions Ga0417192 and Ga0417193, respectively. Open reading frames in *Bc*, *Fj*, and *Pk* genomes were called by prodigal v2.6.3 (55), and BGCs were identified with antiSMASH v5.2.0 (38) under “loose” detection strictness. Open reading frames were annotated with prokka v1.14.6 (56), and further classified into COG categories with eggNOG (evolutionary genealogy of genes: non-supervised orthologous groups) mapper v2.0.8.post2-80-g6e57065 (57). Cluster boundaries for known BGCs were manually curated (*SI Appendix*, Tables S1 and S2). An additional two loci were annotated as bacillithiol biosynthesis (39, 40), and two loci were annotated as nicotinate biosynthesis (41), after manual review of the *Bc* genome (*SI Appendix*, Table S1).

### Metatranscriptomics: RNA Harvest and Sequencing.

Metatranscriptomics was performed as in ref. 12. Briefly, strains were grown individually for 20 h at 28 °C with vigorous shaking (200 rpm). One-milliliter samples from each overnight culture were removed, and the cells were washed and resuspended in 10 mM NaCl. Total colony-forming units (CFUs) were estimated by optical density at 600 nm (OD_600_). Cultures (1/10-strength tryptic soy broth [TSB10]) were inoculated with 1 × 10^6^ *Fj* CFUs per milliliter (final OD_600_ = 0.0008), 1 × 10^6^ *Bc* CFUs per milliliter (final OD_600_ = 0.0167), and/or 1 × 10^6^ *Pk* CFUs per milliliter (final OD_600_ = 0.016) alone, pairwise (2 × 10^6^ total bacterial CFUs per milliliter), or in the three-member community (3 × 10^6^ total bacterial CFUs per milliliter). For *Bc*, *Fj*, and *Pk* monocultures, all pairwise cocultures, and the three-member coculture, five cultures of the same condition were grown statically at 19.5 h at 20 °C and pooled (5 mL total). Then 10 mL of RNAprotect (Qiagen, catalog no. 76526) was added, vortexed, and incubated for 15 min at room temperature. Samples were washed in 10 mM NaCl, and mechanically lysed via liquid nitrogen freezing and pestle grinding. Lysate was resuspended in 200 µL of phosphate-buffered saline. TRIzol (Fisher Scientific, catalog no. 15596026) was heated to 65 °C, and 1 mL was added to each lysate. Samples were heated at 65 °C for 2 min, frozen at −80 °C for 20 min, and thawed to room temperature. Samples were transferred to 240 µL of chloroform, mixed, and incubated for 3 min at room temperature. Samples were centrifuged at 4 °C for 30 min at 12,000 rpm, 600 µL of the aqueous layer was added to 600 µL of cold isopropanol, and the tubes were gently mixed and incubated at room temperature for 10 min. Pellets were air dried and resuspended in RNase-free water. RNA was digested with TurboDNase (Thermo Fisher, catalog no. AM2238) at 37 °C for 30 min and cleaned up via phase-lock tubes. The aqueous layer was subjected to ethanol precipitation and resuspended in RNase-free water. RNA samples were submitted to the University of Wisconsin Biotechnology Center for RNA sequencing. Five hundred nanograms of each sample was depleted of ribosomal RNA with RiboZero Plus before complementary DNA synthesis via the TruSeq Stranded Total RNA Library Prep. RNA sequencing was performed via Illumina NovaSeq 6000. The first dataset sequenced included seven samples (*Bc*, *Fj*, *Pk*, *Bc–Fj*, *B–Pk*, *Fj–Pk*, and *B–Fj–Pk*) in biological quadruplicate. The second dataset sequenced included nine samples (*Pk*, *Bc–Pk*, *Fj–Pk*, *Bc–Fj–Pk*, Δ*Pk*, *Bc–*Δ*Pk*, *Fj–*Δ*Pk*, and *Bc–Fj–*Δ*Pk*, where Δ*Pk* denotes the koreenceine mutant) in biological quadruplicate. All samples were sequenced at a depth of 5 million reads, except for samples in which *Fj* was inhibited by *Pk* (i.e., *Fj–Pk* and *Bc–Fj–Pk*), which were sequenced to 50 million or 20 million reads for cocultures with wild-type *Pk* or Δ*Pk*, respectively, to ensure adequate detection of *Fj* reads when *Fj* was at lower abundance.

### Metatranscriptomics: Data Analyses.

Full code for data processing and figure/table generation can be found at https://github.com/chevrm/thor_secmet (58). Briefly, reads were preprocessed with fastp v0.20.0 (59) and split to their respective source (i.e., either *Bc*, *Fj*, or *Pk*) with bbsplit (60), discarding ambiguously mapped reads. Bowtie2 v2.4.2 (61) was used to map reads to individually indexed genomes of *Bc*, *Fj*, or *Pk*. Resulting SAM (sequence alignment map) files were sorted with samtools v1.9 (62) and quantified by HTSeq (high throughput sequencing Python library) v0.12.4 (63) under mode “intersection-strict” against the GFF (general feature format) files previously annotated by prodigal and antiSMASH. Counts per million (CPM) were computed in edgeR (64). To reduce noise caused by genes with very low expression levels in the dataset, genes whose maximum expression across replicates fell below the 2.5 percentile for each reference genome were excluded from further analysis (*SI Appendix*, Figs. S1 and S6). Fold changes were calculated using the optimal effect size estimate as implemented in PsiLFC (Psi log fold change R library) (65). BGC-wide expression was calculated as the sum of all CPMs for a given BGC.

### Metametabolomics.

Single-isolate, pairwise, and three-member community cocultures (five replicates each) were grown as 1-mL static cultures in TSB10 for 19.5 h at 20 °C. Cultures were inoculated at 1 × 10^6^ CFU per milliliter. Cultures were pelleted, and supernatants were filtered through a 0.22-µm filter, flash frozen in liquid N_2_, and kept at −80 °C until analysis. Sterile media (TSB10) was also processed (five replicates, 1 mL) to identify media components in samples. Samples were thawed and transferred to a 96-well plate for use with a Gilson GX-271 liquid handler for solid-phase extraction (SPE) of secondary metabolites. In brief, 1 mL of SPE cartridges (ABN polymeric solid phase, Biotage) was conditioned with MeOH, equilibrated with MQ H_2_O, loaded with 700 µL of sample supernatant, washed with H_2_O, and eluted with 200 µL of MeOH. Five microliters from each sample was pooled to provide a quality control (QC) sample. Samples were randomized and analyzed on a Q-Exactive Orbitrap mass spectrometer (Thermo Scientific) operated in positive mode. Chromatography was conducted on a Phenomenex Kinetex C18 column (100 mm × 2.1 mm, 1.7 µm) using a 20-min gradient of MeCN (line B) and H_2_O (line A), each with 0.1% formic acid. The gradient method was as follows: 5% B for 0.5 min, linear gradient to 40% B at 10.5 min, linear gradient 97% B at 16.5 min and hold to 18.5 min, and return to 5% B at 19 min and hold to 20 min. The flow rate was kept constant for 0.3 mL/min; 5 µL was injected for each sample. The QC sample was injected every five samples to track instrument drift over time. Data-dependent acquisition was used to collect MS data over the course of the run. MS1 data were collected at 70,000 resolution with a scan range of 200 *m/z* to 2,000 *m/z*. MS2 data were collected at 17,500 resolution, and the top seven most intense ions were selected for each MS1 scan with an isolation window of 2 *m/z*. Dynamic exclusion was set for 7 s. A stepped collision energy (normalized collision energy [NCE]) was used at 20%, 30%, and 40% for MS2 fragmentation. Raw LC-MS data were processed using Compound Discoverer v3.3 (Thermo Scientific). The “Untargeted Metabolomics with Statistics Detect Unknowns with ID using Online Databases and mzLogic” preset workflow was used with some modifications. The minimum peak intensity in the “Detect Compounds” node was set to 1 × 10^6^. Mass spectral features were searched using the “Natural Product Atlas 2020_06” (66) mass list (MS1), and the “Bamba laboratory 598 polar metabolites stepped NCE 10 30 45” spectral library (MS2) in addition to the MzCloud (MS2) and ChemSpider (MS1- BioCyc, Human Metabolome Database, KEGG [Kyoto encyclopedia of genes and genomes], NPAtlas) search nodes already incorporated into the workflow. The “Generate Molecular Networks” node was also incorporated for examining molecular networks. The raw data table of molecular feature intensities was exported for further data processing.

### Metabolite-Level Statistical Analysis.

Full code for data processing and figure/table generation can be found at https://github.com/chevrm/thor_secmet (58). For Fig. 3*A*, samples were filtered to *Bc*-, *Fj*-, *Pk*-only and coculture conditions. For each molecular feature, two linear models were used to jointly analyze effects across all species present in the restricted sets of samples. The response variable in each model was log-transformed molecular feature abundance. This transformation accounts for skewness in the molecular feature abundance distributions. The three covariates for each model were ±1-coded indicators of whether the associated species were present. All main effects, two-way interactions, and three-way interactions were included. For example, for the *Bc*, *Fj*, and *Pk* analysis in Fig. 3*A*, we fit

$$\log\left({\text{y}}_{\text{i}}^{\text{m}}\right)={\text{β}}_{0}^{\text{m}}+\underset{\text{j}\in \{\text{B},\text{F},\text{K}\}}{\sum }{\text{β}}_{\text{j}}^{\text{m}}1\{\text{j}\in \text{sample}\;\text{i}\}+\underset{{\text{j}}^{\prime }\in \{\text{BF},\text{BK},\text{FK}\}}{\sum }{\text{β}}_{{\text{j}}^{\prime }}^{\text{m}}1\{{\text{j}}^{\prime }\subseteq \text{sample}\;\text{i}\}+{\text{β}}_{\text{BFK}}^{\text{m}}1\{\text{BFK}\subseteq \text{sample}\;\text{i}\},$$

where $1\{A\}=\{\begin{array}{c} 1\;if\;A\;is\;\mathrm{true}\\ -1\;\mathrm{otherwise} \end{array}$, and i ranges over all samples excluding media control ranges over all molecular features. Coefficients β and their *t* statistics were obtained using the lm function in R 4.2.0. For each effect ${\text{β}}_{j}^{m},{\text{β}}_{j^{\prime }}^{m},\;\mathrm{and}\;{\text{β}}_{\mathrm{BFK}}^{m}$, *t* statistics across the ensemble of molecular features *m* were visualized as histograms (67, 68). Molecular features at the tails of histograms have larger-than-typical absolute effect sizes. Interactive selection of metabolites with highly ranked effect sizes was implemented through linked brushing. This model includes all main effects, two-way interactions, and three-way interactions. Implementation of the above can be found at https://github.com/YixingTT/MTBrush (69). The *t* statistics for each term of the model were calculated for each molecular feature. A baseline for presence–absence intensities was calculated based on the peak area values for koreenceine A, B, and C for conditions with the koreenceine knockout *Pk*, as koreenceine is not made by the knockout strain. Principal component analyses with unit normalization and subsequent loadings analyses were carried out in base R (*SI Appendix*, Fig. S4), replicates were hierarchically clustered based on Spearman correlation (*SI Appendix*, Fig. S5), and overlapping molecular features between conditions were calculated.

### Koreenceine Mutant Studies.

Metabolomics and transcriptomics was performed in the same previously described conditions as the wild-type experiments, substituting *Pk* with the koreenceine mutant, *Pk* Δ*kecA-K*::*tetRA* (37).

## Supplementary Material

Supplementary File

## Data Availability

All study data are included in the article and/or supporting information. Data (including transcriptomics and metabolomics), code, and figures are openly available at https://github.com/chevrm/thor_secmet (70) and https://github.com/YixingTT/MTBrush (71). Raw transcriptomic reads can be found on National Center for Biotechnology Information's Sequence Read Archive under BioProject accession PRJNA885088.
