# Supplementary material for: Microbiome composition modulates secondary metabolism in a multispecies bacterial community
Source: Proc Natl Acad Sci U S A. 2022 Oct 10;119(42):e2212930119. doi: 10.1073/pnas.2212930119 (PMC9586298; doi:10.1073/pnas.2212930119)
Supplement: Supplementary File [file pnas.2212930119.sapp.pdf]

Enclosed in this file is the full supplementary appendix for

Chevrette et al. "Microbiome composition modulates secondary metabolism in a multispecies bacterial community." *Proceedings of the National Academy of Sciences*. 2022.

In compliance with *PNAS* publishing requirements, all supplemental figures and tables are included in this file. If readers would prefer individual, original figures and/or more easily human- and computer-readable table formats, you can find all materials related to this manuscript, at https://github.com/chevrm/thor_secmet/. Corresponding file names and locations can be found below.

This file includes:

Supplemental figures

Fig S1: https://github.com/chevrm/thor_secmet/blob/main/figures/sup/fs1_wt_max_cpm.pdf

Fig S2: https://github.com/chevrm/thor_secmet/blob/main/figures/sup/fs2_wt_transcriptomics.pdf

Fig S3: https://github.com/chevrm/thor_secmet/blob/main/figures/sup/fs3_lcms_presence_cutoffs.pdf

Fig S4: https://github.com/chevrm/thor_secmet/blob/main/figures/sup/fs4_wt_lcms_pca.pdf

Fig S5: https://github.com/chevrm/thor_secmet/blob/main/figures/sup/fs5_lcms_correlation.pdf

Fig S6: https://github.com/chevrm/thor_secmet/blob/main/figures/sup/fs6_mut_max_cpm.pdf

Supplemental table map

Table map: https://github.com/chevrm/thor_secmet/blob/main/figures/sup/supplimental_table_map.pdf

Supplemental tables

Table S1: https://github.com/chevrm/thor_secmet/blob/main/figures/sup/ts1_detailed_bgc_annotations.xlsx

Table S2: https://github.com/chevrm/thor_secmet/blob/main/figures/sup/ts2_select_bgc_gene_annotations.xlsx

Table S3: https://github.com/chevrm/thor_secmet/blob/main/figures/sup/ts3_wt_cpm.tsv

Table S4: https://github.com/chevrm/thor_secmet/blob/main/figures/sup/ts4_wt_BGC_expression_changes.tsv

Table S5: https://github.com/chevrm/thor_secmet/blob/main/figures/sup/ts5_lcms_abundance_matrix.tsv

Table S6: https://github.com/chevrm/thor_secmet/blob/main/figures/sup/ts6_lcms_annotations.tsv

Table S7: https://github.com/chevrm/thor_secmet/blob/main/figures/sup/ts7_mut_cpm.tsv

The figure following this page is:

Figure S1

for manuscript:

Chevrette et al. "Microbiome composition modulates secondary metabolism in a multispecies bacterial community." *Proceedings of the National Academy of Sciences*. 2022.

All supplemental materials for this manuscript can be found at:

https://github.com/chevrm/thor_secmet/tree/main/figures/sup

Please refer to the cover sheet of this PDF for the exact file location on GitHub.

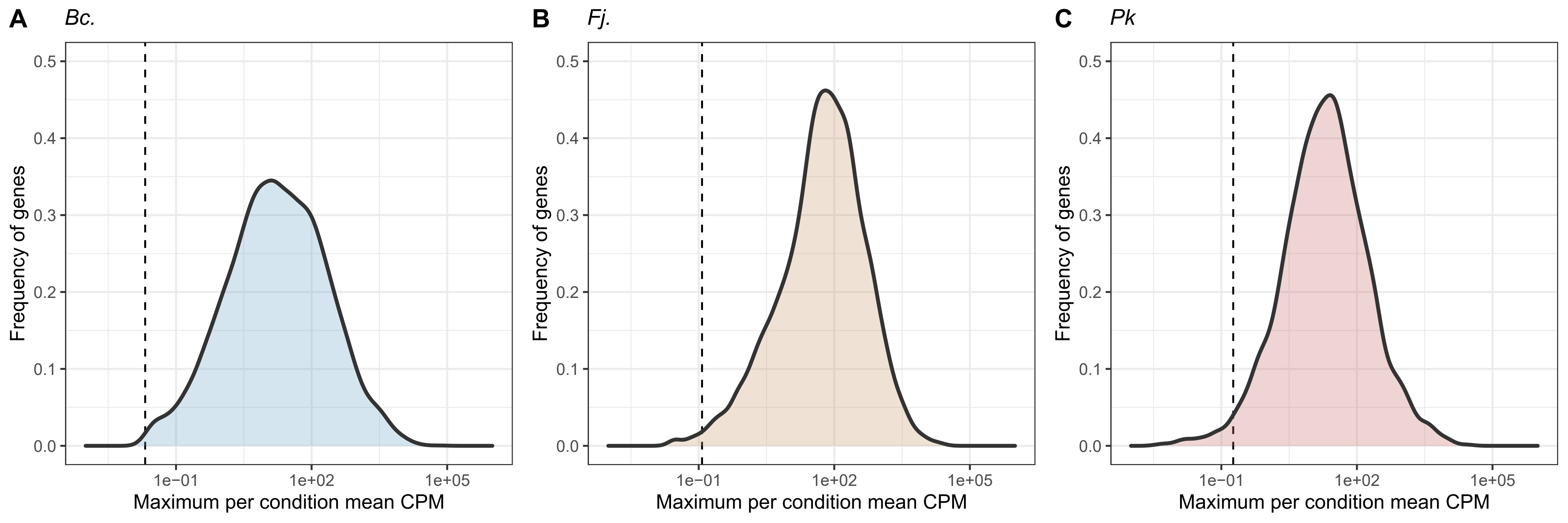
A
Bc.
B
Fj.
C
Pk
Frequency of genes
0.0
0.1
0.2
0.3
0.4
0.5
1e−01
1e+02
1e+05
Maximum per condition mean CPM

The figure following this page is:

Figure S2

for manuscript:

Chevrette et al. "Microbiome composition modulates secondary metabolism in a multispecies bacterial community." *Proceedings of the National Academy of Sciences*. 2022.

All supplemental materials for this manuscript can be found at:

https://github.com/chevrm/thor_secmet/tree/main/figures/sup

Please refer to the cover sheet of this PDF for the exact file location on GitHub.

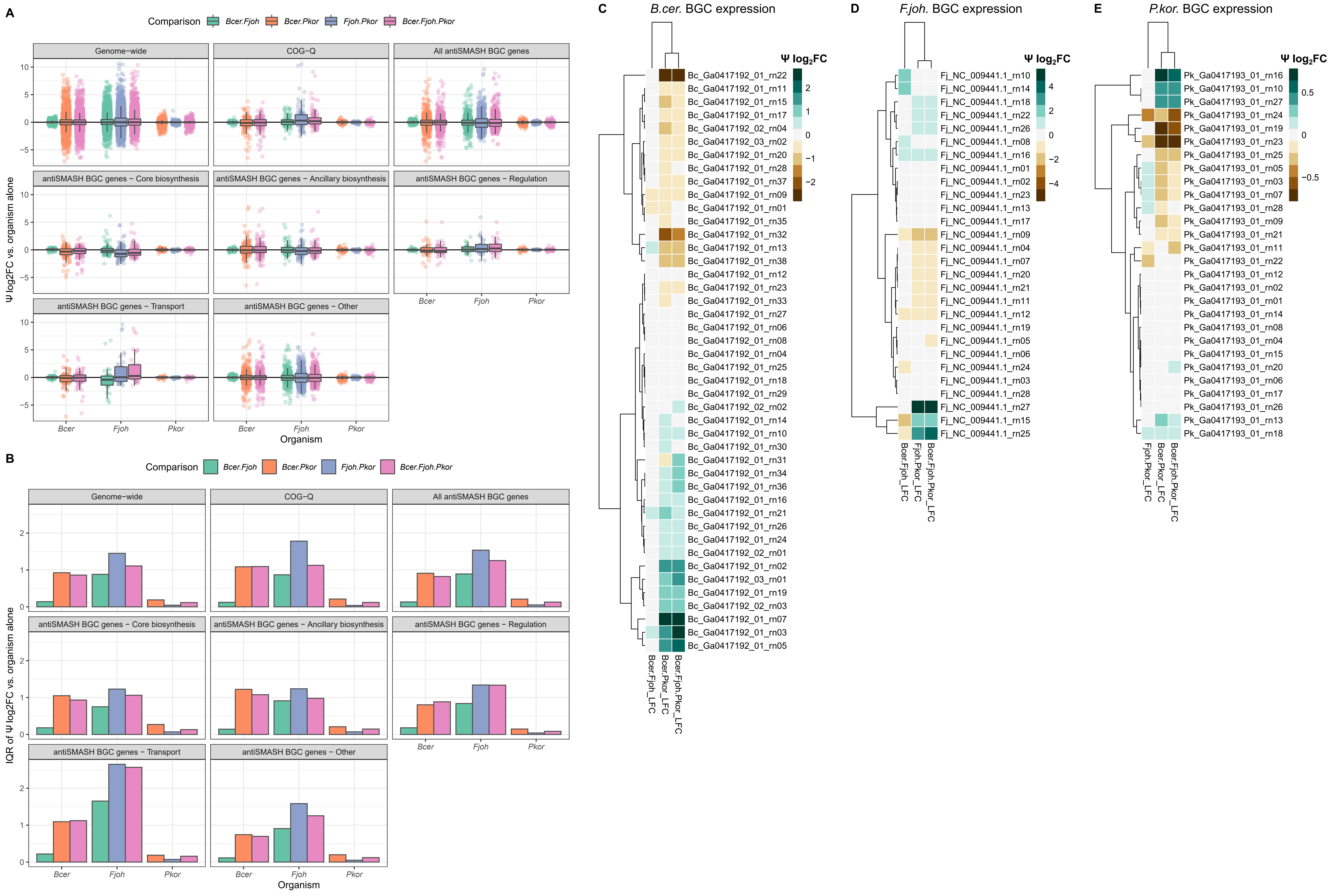
A
Comparison
Bcer.Fjoh
Bcer.Pkor
Fjoh.Pkor
Bcer.Fjoh.Pkor
Genome−wide
COG−Q
All antiSMASH BGC genes
antiSMASH BGC genes − Core biosynthesis
antiSMASH BGC genes − Ancillary biosynthesis
antiSMASH BGC genes − Regulation
antiSMASH BGC genes − Transport
antiSMASH BGC genes − Other
Ψ log2FC vs. organism alone
Bcer
Fjoh
Pkor
Organism
B
IQR of Ψ log2FC vs. organism alone
C
B.cer. BGC expression
Ψ log$_2$FC
Bc_Ga0417192_01_rn22
Bc_Ga0417192_01_rn11
Bc_Ga0417192_01_rn15
Bc_Ga0417192_01_rn17
Bc_Ga0417192_02_rn04
Bc_Ga0417192_03_rn02
Bc_Ga0417192_01_rn20
Bc_Ga0417192_01_rn28
Bc_Ga0417192_01_rn37
Bc_Ga0417192_01_rn09
Bc_Ga0417192_01_rn01
Bc_Ga0417192_01_rn35
Bc_Ga0417192_01_rn32
Bc_Ga0417192_01_rn13
Bc_Ga0417192_01_rn38
Bc_Ga0417192_01_rn12
Bc_Ga0417192_01_rn23
Bc_Ga0417192_01_rn33
Bc_Ga0417192_01_rn27
Bc_Ga0417192_01_rn06
Bc_Ga0417192_01_rn08
Bc_Ga0417192_01_rn04
Bc_Ga0417192_01_rn25
Bc_Ga0417192_01_rn18
Bc_Ga0417192_01_rn29
Bc_Ga0417192_02_rn02
Bc_Ga0417192_01_rn14
Bc_Ga0417192_01_rn10
Bc_Ga0417192_01_rn30
Bc_Ga0417192_01_rn31
Bc_Ga0417192_01_rn34
Bc_Ga0417192_01_rn36
Bc_Ga0417192_01_rn16
Bc_Ga0417192_01_rn21
Bc_Ga0417192_01_rn26
Bc_Ga0417192_01_rn24
Bc_Ga0417192_02_rn01
Bc_Ga0417192_01_rn02
Bc_Ga0417192_03_rn01
Bc_Ga0417192_01_rn19
Bc_Ga0417192_02_rn03
Bc_Ga0417192_01_rn07
Bc_Ga0417192_01_rn03
Bc_Ga0417192_01_rn05
Bcer.Fjoh_LFC
Bcer.Pkor_LFC
Bcer.Fjoh.Pkor_LFC
D
F.joh. BGC expression
Fj_NC_009441.1_rn10
Fj_NC_009441.1_rn14
Fj_NC_009441.1_rn18
Fj_NC_009441.1_rn22
Fj_NC_009441.1_rn26
Fj_NC_009441.1_rn08
Fj_NC_009441.1_rn16
Fj_NC_009441.1_rn01
Fj_NC_009441.1_rn02
Fj_NC_009441.1_rn23
Fj_NC_009441.1_rn13
Fj_NC_009441.1_rn17
Fj_NC_009441.1_rn09
Fj_NC_009441.1_rn04
Fj_NC_009441.1_rn07
Fj_NC_009441.1_rn20
Fj_NC_009441.1_rn21
Fj_NC_009441.1_rn11
Fj_NC_009441.1_rn12
Fj_NC_009441.1_rn19
Fj_NC_009441.1_rn05
Fj_NC_009441.1_rn06
Fj_NC_009441.1_rn24
Fj_NC_009441.1_rn03
Fj_NC_009441.1_rn28
Fj_NC_009441.1_rn27
Fj_NC_009441.1_rn15
Fj_NC_009441.1_rn25
Bcer.Fjoh_LFC
Fjoh.Pkor_LFC
Bcer.Fjoh.Pkor_LFC
E
P.kor. BGC expression
Pk_Ga0417193_01_rn16
Pk_Ga0417193_01_rn10
Pk_Ga0417193_01_rn27
Pk_Ga0417193_01_rn24
Pk_Ga0417193_01_rn19
Pk_Ga0417193_01_rn23
Pk_Ga0417193_01_rn25
Pk_Ga0417193_01_rn05
Pk_Ga0417193_01_rn03
Pk_Ga0417193_01_rn07
Pk_Ga0417193_01_rn28
Pk_Ga0417193_01_rn09
Pk_Ga0417193_01_rn21
Pk_Ga0417193_01_rn11
Pk_Ga0417193_01_rn22
Pk_Ga0417193_01_rn12
Pk_Ga0417193_01_rn02
Pk_Ga0417193_01_rn01
Pk_Ga0417193_01_rn14
Pk_Ga0417193_01_rn08
Pk_Ga0417193_01_rn04
Pk_Ga0417193_01_rn15
Pk_Ga0417193_01_rn20
Pk_Ga0417193_01_rn06
Pk_Ga0417193_01_rn17
Pk_Ga0417193_01_rn26
Pk_Ga0417193_01_rn13
Pk_Ga0417193_01_rn18
Fjoh.Pkor_LFC
Bcer.Pkor_LFC
Bcer.Fjoh.Pkor_LFC

The figure following this page is:

Figure S3

for manuscript:

Chevrette et al. "Microbiome composition modulates secondary metabolism in a multispecies bacterial community." *Proceedings of the National Academy of Sciences*. 2022.

All supplemental materials for this manuscript can be found at:

https://github.com/chevrm/thor_secmet/tree/main/figures/sup

Please refer to the cover sheet of this PDF for the exact file location on GitHub.

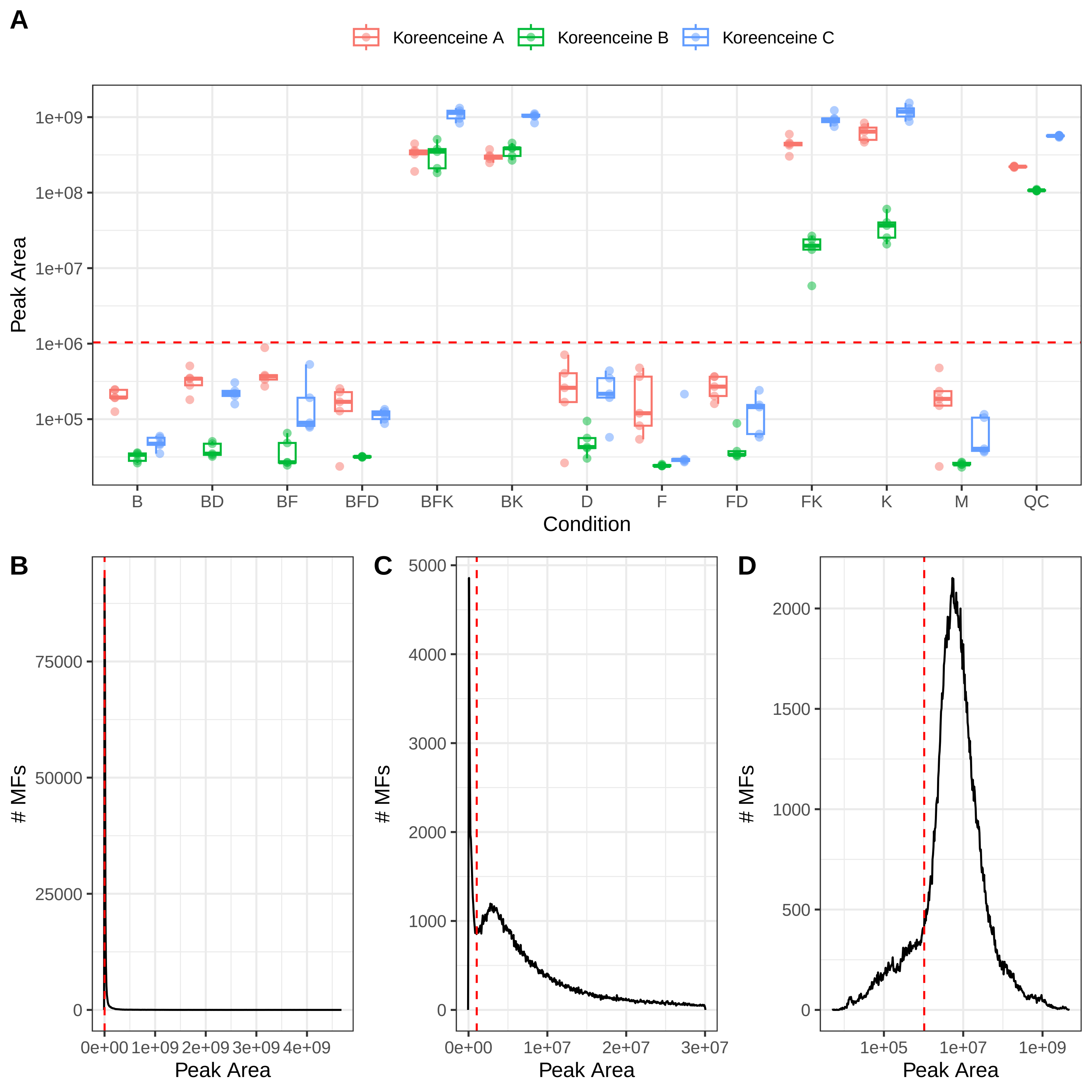
A
Koreenceine A
Koreenceine B
Koreenceine C
Peak Area
1e+09
1e+08
1e+07
1e+06
1e+05
B
BD
BF
BFD
BFK
BK
D
F
FD
FK
K
M
QC
Condition
B
MFs
75000
50000
25000
0
0e+00
1e+09
2e+09
3e+09
4e+09
Peak Area
C
MFs
5000
4000
3000
2000
1000
0
0e+00
1e+07
2e+07
3e+07
Peak Area
D
MFs
2000
1500
1000
500
0
1e+05
1e+07
1e+09
Peak Area

The figure following this page is:

Figure S4

for manuscript:

Chevrette et al. "Microbiome composition modulates secondary metabolism in a multispecies bacterial community." *Proceedings of the National Academy of Sciences*. 2022.

All supplemental materials for this manuscript can be found at:

https://github.com/chevrm/thor_secmet/tree/main/figures/sup

Please refer to the cover sheet of this PDF for the exact file location on GitHub.

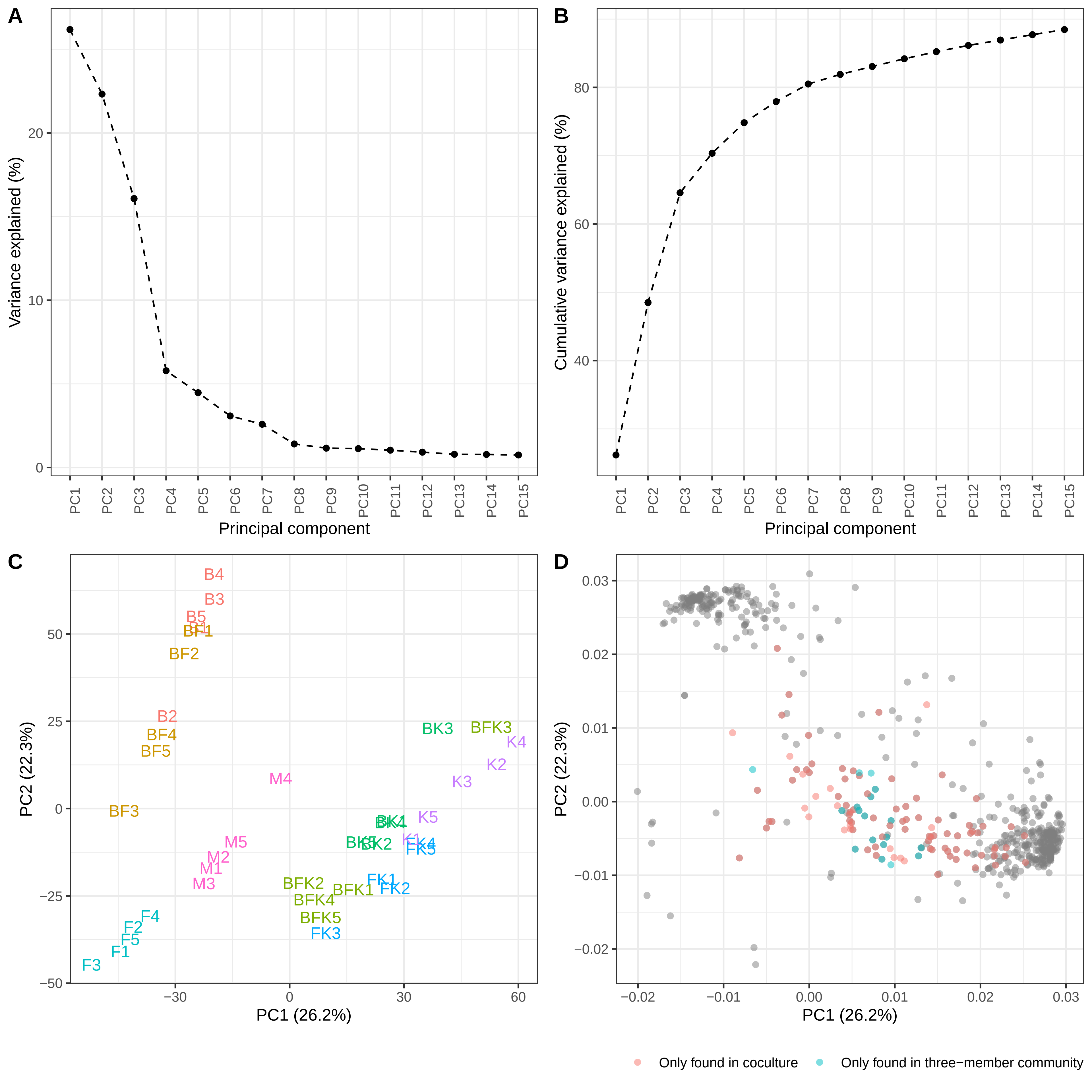
A
Variance explained (%)
Principal component
B
Cumulative variance explained (%)
Principal component
C
PC2 (22.3%)
PC1 (26.2%)
B4
B3
B5
BF1
BF2
B2
BF4
BF5
BF3
M4
M5
M2
M1
M3
F4
F2
F5
F1
F3
BK3
BFK3
K4
K2
K3
K5
BK1
BK4
BK5
BK2
K1
FK4
FK5
BFK2
BFK4
BFK1
BFK5
FK1
FK2
FK3
D
PC2 (22.3%)
PC1 (26.2%)
Only found in coculture
Only found in three−member community

The figure following this page is:

Figure S5

for manuscript:

Chevrette et al. “Microbiome composition modulates secondary metabolism in a multispecies bacterial community.” *Proceedings of the National Academy of Sciences*. 2022.

All supplemental materials for this manuscript can be found at:

https://github.com/chevrm/thor_secmet/tree/main/figures/sup

Please refer to the cover sheet of this PDF for the exact file location on GitHub.

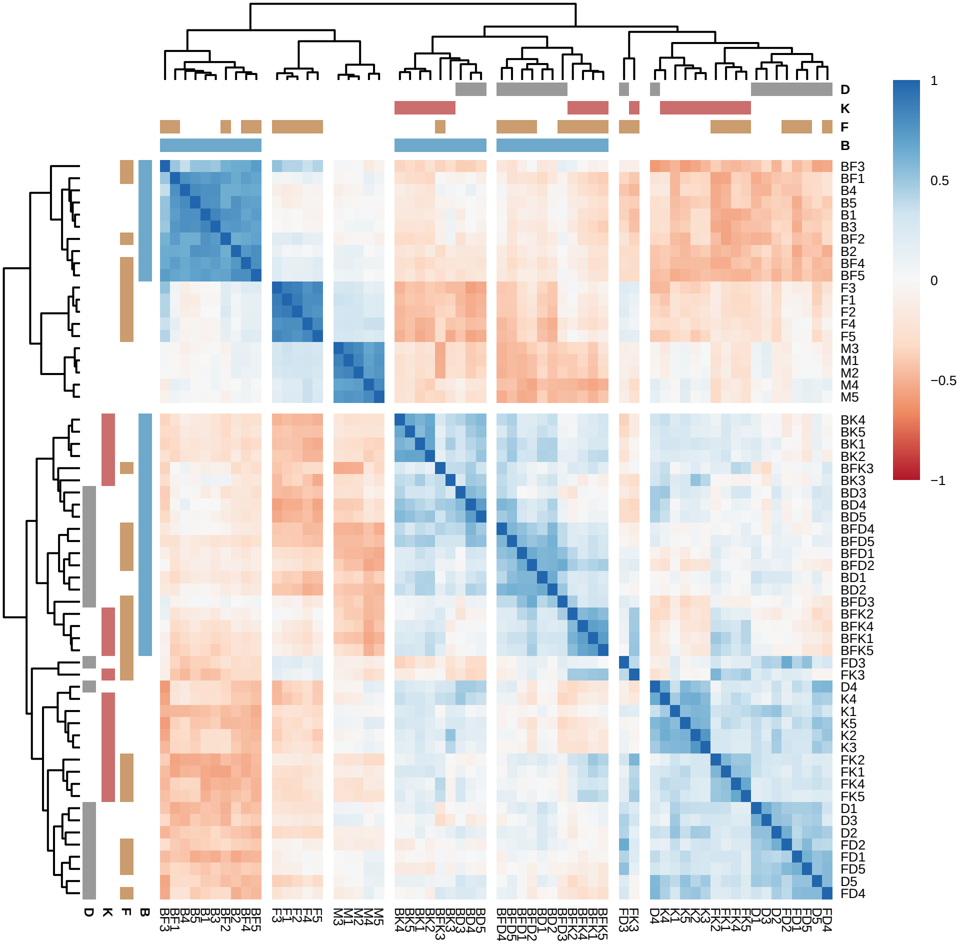
D
K
F
B
1
0.5
0
−0.5
−1
BF3
BF1
B4
B5
B1
B3
BF2
B2
BF4
BF5
F3
F1
F2
F4
F5
M3
M1
M2
M4
M5
BK4
BK5
BK1
BK2
BFK3
BK3
BD3
BD4
BD5
BFD4
BFD5
BFD1
BFD2
BD1
BD2
BFD3
BFK2
BFK4
BFK1
BFK5
FD3
FK3
D4
K4
K1
K5
K2
K3
FK2
FK1
FK4
FK5
D1
D3
D2
FD2
FD1
FD5
D5
FD4

The figure following this page is:

Figure S6

for manuscript:

Chevrette et al. "Microbiome composition modulates secondary metabolism in a multispecies bacterial community." *Proceedings of the National Academy of Sciences*. 2022.

All supplemental materials for this manuscript can be found at:

https://github.com/chevrm/thor_secmet/tree/main/figures/sup

Please refer to the cover sheet of this PDF for the exact file location on GitHub.

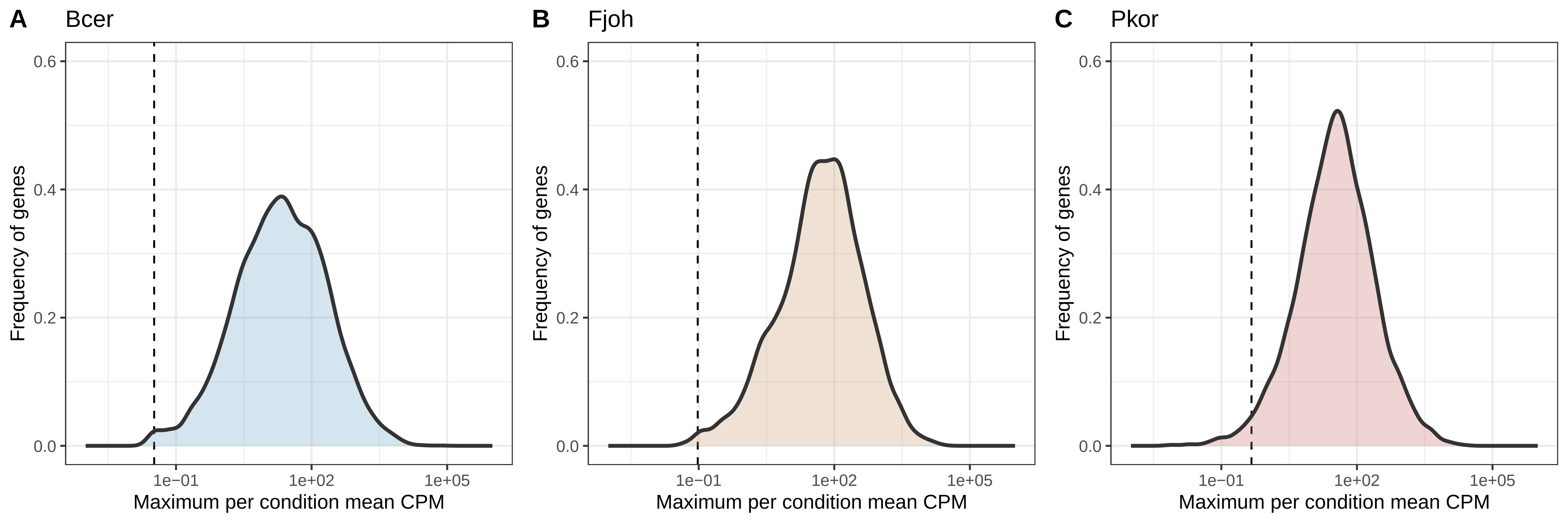
A
Bcer
B
Fjoh
C
Pkor
Frequency of genes
0.0
0.2
0.4
0.6
1e−01
1e+02
1e+05
Maximum per condition mean CPM

The text following this page is:

Supplemental table map

for manuscript:

Chevrette et al. "Microbiome composition modulates secondary metabolism in a multispecies bacterial community." *Proceedings of the National Academy of Sciences*. 2022.

All supplemental materials for this manuscript can be found at:

https://github.com/chevrm/thor_secmet/tree/main/figures/sup

Please refer to the cover sheet of this PDF for the exact file location on GitHub.

Supplemental table map for:

Chevrette et al. "Microbiome composition modulates secondary metabolism in a multispecies bacterial community." *Proceedings of the National Academy of Sciences*. 2022.

Table S1 – Detailed BGC annotations

Pathway-level annotations of all BGCs in this study.

- Rows = Individual BGCs
- Columns
    A. organism = Organism in THOR whose genome has this BGC. Either *Bacillus cereus* UW85, *Flavobacterium johnsoniae* UW101, or *Pseudomonas koreensis* CI12.
    B. bgc = Name of the BGC. For unknown BGCs, the antiSMASH category of biosynthesis is listed (e.g., putative saccharide, NRPS-like, etc.). For known BGCs, colloquial names are used (e.g., petrobactin, bacillibactin, etc.) and exactly match how they are referred to in the manuscript text.
    C. bgc_group = Type of biosynthesis (as defined by antiSMASH)
    D. contig = the accession number of the genomic contig that a BGC is found
    E. antiSMASH_bgc = antiSMASH-generated region numbers for each BGC
    F. Known = Is this BGC previously experimentally described, Y or N (yes or no)
    G. Manual curation = were the genes and/or start/end sites of this BGC been manually curated from the antiSMASH predictions, Y or N (yes or no)
    H. start = start location for the BGC on the contig
    I. end = end location for the BGC on the contig
    J. size = size of the BGC in basepairs
    K. ClusterBlast = top % gene identity in antiSMASH ClusterBlast against antiSMASH-db
    L. KnownClusterBlast = top % gene identity in antiSMASH ClusterBlast against MIBiG
    M. Lit_Activity = bioactivity as described in the literature
    N. Reference = DOI for references of the BGC and/or bioactivity
    O. Notes = Notes

Table S2 – Select BGC Annotations

Manually-curated, gene-level annotations and BGC start/end sites in THOR BGCs. In cases not seen here, cluster boundaries from antiSMASH predictions were used.

- Rows = Genes
- Columns
    A. organism = Organism in THOR whose genome has this gene. Either *Bacillus cereus* UW85, *Flavobacterium johnsoniae* UW101, or *Pseudomonas koreensis* CI12.
    B. bgc = Name of the BGC. For unknown BGCs, the antiSMASH category of biosynthesis is listed (e.g., putative saccharide, NRPS-like, etc.). For known BGCs, colloquial names are

used (e.g., petrobactin, bacillibactin, etc.) and exactly match how they are referred to in the manuscript text.

C. contig = the accession number of the genomic contig that a BGC is found
D. gene = unique gene name from prodigal gene calls in antiSMASH
E. ann = annotated gene name as found in the literature (e.g., zmaR for zwittermicin resistance gene)
F. replace_antismash_bgc = which BGC should this annotation replace. Perfectly matches column E from Table S1.
G. ann_verbose = verbose annotation of each gene
H. ann_acc = accession number for each annotation
I. ann_percent_prot_id = % protein identity to the gene used for annotation
J. notes = Notes

Table S3 – WT CPM

Gene-level counts per million reads (CPM) for wildtype mono- and co-culture transcriptomic studies.

- Rows = unique gene-replicate pair (e.g., Bc_ctg1_1 in replicate B1 is its own unique row)
- Columns
    A. gene = unique gene name from prodigal gene calls in antiSMASH. Note that antiSMASH calls genes with prodigal across the entire genome, not just in BGCs, so every gene in each THOR genome is represented here (not just those that are in BGCs).
    B. sample = replicate ID. B1 denotes *Bacillus* (B) monoculture, replicate 1. BFK4 denotes *Bacillus* (B), *Flavobacterium* (F), *Pseudomonas* (K), co-culture, replicate 4.
    C. cpm = Counts per million reads
    D. condition = mono- or co-culture condition of this replicate. Bcer denotes *Bacillus* monoculture. Bcer.Fjoh denotes *Bacillus-Flavobacterium* coculture.
    E. cog_category = COG category as annotated by eggNOG
    F. organism = Organism in THOR whose genome has this gene. Either Bcer *for Bacillus cereus* UW85, Fjoh for *Flavobacterium johnsoniae* UW101, or Pkor for *Pseudomonas koreensis* CI12.
    G. is.q = TRUE or FALSE, is this gene annotated as COG category Q (secondary metabolism)?
    H. bgc = BGC this gene belongs to. NA if outside a BGC.
    I. gene_kind = antiSMASH annotation of type of BGC gene
    J. smcog = antiSMASH SMCOG category

Table S4 – WT BGC Expression Changes

BGC-level changes in expression compared to monoculture.

- Rows = unique BGC-condition pair (e.g., antiSMASH BGC region Bc_Ga0417192_01_rn01 in condition “Pairwise with Fjoh” is its own unique row)
- Columns
    A. bgc = antiSMASH BGC region

B. BGC of = organism whose genome has this BGC. Either Bcer, Fjoh, or Pkor.
C. coculture = coculture condition to compare to monoculture
D. LFC = Psi log fold change (see methods) of coculture vs. monoculture. Positive values indicate higher expression in coculture, negative values indicate higher expression in monoculture.

Table S5 – LC-MS Abundance Matrix

LC-MS abundance of molecular features in each replicate (n=5 each condition).

- Rows = unique compound ID for each molecular feature
- Columns = replicate ID. B1 denotes *Bacillus* (B) monoculture, replicate 1. BFK4 denotes *Bacillus* (B), *Flavobacterium* (F), *Pseudomonas* (K), co-culture, replicate 4.
- Data in matrix = LC-MS abundance value (see methods)

Table S6 – LC-MS Annotations

Annotation information for LC-MS molecular features from Table S5.

- Rows = unique compound ID for each molecular feature. Perfectly matches row names in Table S5.
- Columns
  A. Compounds.ID = unique compound ID for each molecular feature. Perfectly matches row names in Table S5.
  B. Name = Compound Discoverer ™ Annotation
  C. Tags = Notes and/or manual annotation
  D. Formula = Compound Discoverer ™ predicted molecular formula
  E. RT_min = Compound Discoverer ™ retention time
  F. Calc_MW = Compound Discoverer ™ calculated molecular weight

Table S7 – Koreenceine Mutant CPM

Gene-level counts per million reads (CPM) for koreenceine deletion-mutant mono- and co-culture transcriptomic studies.

- Rows = unique gene-replicate pair (e.g., Bc_ctg1_1 in replicate B1 is its own unique row)
- Columns
  A. gene = unique gene name from prodigal gene calls in antiSMASH. Note that antiSMASH calls genes with prodigal across the entire genome, not just in BGCs, so every gene in each THOR genome is represented here (not just those that are in BGCs).
  B. sample = replicate ID. B1 denotes *Bacillus* (B) monoculture, replicate 1. BFK4 denotes *Bacillus* (B), *Flavobacterium* (F), *Pseudomonas* (K), co-culture, replicate 4. “delkecK” denotes the koreenceine deletion mutant of *Pseudomonas*.

C. cpm = Counts per million reads
D. condition = mono- or co-culture condition of this replicate. Bcer denotes *Bacillus* monoculture. Bcer.Fjoh denotes *Bacillus-Flavobacterium* coculture. Pkor denotes wildtype *Pseudomonas* and Pkor* denotes the *Pseudomonas* koreenceine deletion mutant.
E. cog_category = COG category as annotated by eggNOG
F. organism = Organism in THOR whose genome has this gene. Either Bcer *for Bacillus cereus* UW85, Fjoh for *Flavobacterium johnsoniae* UW101, or Pkor for *Pseudomonas koreensis* CI12.
G. is.q = TRUE or FALSE, is this gene annotated as COG category Q (secondary metabolism)?
H. bgc = BGC this gene belongs to. NA if outside a BGC.
I. gene_kind = antiSMASH annotation of type of BGC gene
J. smcog = antiSMASH SMCOG category

The table following this page is:

Table S1

for manuscript:

Chevrette et al. "Microbiome composition modulates secondary metabolism in a multispecies bacterial community." *Proceedings of the National Academy of Sciences*. 2022.

All supplemental materials for this manuscript can be found at:

https://github.com/chevrm/thor_secmet/tree/main/figures/sup

Please refer to the cover sheet of this PDF for the exact file location on GitHub.

organism	bgc	bgc_group	contig	antiSMASH_bgc	Known?	Manual curation?	start	end	size	ClusterBlast	KnownClusterBlast	Lit_Activity	Reference	Notes
Bacillus cereus UW85	Pulcherriminic acid	Siderophore	Ga0417192_01	Bc_Ga0417192_01_rn01	Y	Y	120529	125137	4609	93	0	Iron chelator; antibiotic	10.3390/molecules25235611	One molecule will bind 2 Fe
Bacillus cereus UW85	Putative saccharide	Putative saccharide and/or fatty acid	Ga0417192_01	Bc_Ga0417192_01_rn02	N	N	266653	289518	22866	22	0	NA	NA	
Bacillus cereus UW85	Putative saccharide	Putative saccharide and/or fatty acid	Ga0417192_01	Bc_Ga0417192_01_rn03	N	N	313671	339983	26313	50	5	NA	NA	
Bacillus cereus UW85	Putative saccharide	Putative saccharide and/or fatty acid	Ga0417192_01	Bc_Ga0417192_01_rn04	N	N	349362	389203	39842	27	0	NA	NA	Has bacillithiol gene bchB2 at Bc_ctg1_355
Bacillus cereus UW85	Putative saccharide	Putative saccharide and/or fatty acid	Ga0417192_01	Bc_Ga0417192_01_rn05	N	N	422984	442616	19633	37	0	NA	NA	
Bacillus cereus UW85	Putative cyclized triterpene	Terpene	Ga0417192_01	Bc_Ga0417192_01_rn06	N	N	491426	513279	21854	100	17	NA	NA	Has a squalene-hopene cyclase with similarity to the cyclase in Bsub sporulene, so likely a cyclized triterpene like sporulene or hopene; transporter is rarD (chloramphenicol family); right end has 3 molybdenum cofactor biosynthesis genes
Bacillus cereus UW85	Putative saccharide	Putative saccharide and/or fatty acid	Ga0417192_01	Bc_Ga0417192_01_rn07	N	N	682321	704039	21719	12	0	NA	NA	
Bacillus cereus UW85	Putative saccharide	Putative saccharide and/or fatty acid	Ga0417192_01	Bc_Ga0417192_01_rn08	N	N	900836	924494	23659	48	0	NA	NA	Has bacillithiol gene bchC at Bc_ctg1_895 and just outside the upstream Bc_ctg1_896 ylbQ
Bacillus cereus UW85	Putative saccharide	Putative saccharide and/or fatty acid	Ga0417192_01	Bc_Ga0417192_01_rn09	N	N	995040	1025853	30814	37	6	NA	NA	
Bacillus cereus UW85	Putative saccharide	Putative saccharide and/or fatty acid	Ga0417192_01	Bc_Ga0417192_01_rn10	N	N	1208160	1235182	27023	22	0	NA	NA	
Bacillus cereus UW85	Putative saccharide	Putative saccharide and/or fatty acid	Ga0417192_01	Bc_Ga0417192_01_rn11	N	N	1345810	1385028	39219	55	0	NA	NA	
Bacillus cereus UW85	Putative saccharide	Putative saccharide and/or fatty acid	Ga0417192_01	Bc_Ga0417192_01_rn12	N	N	1635665	1661642	25978	30	0	NA	NA	
Bacillus cereus UW85	Putative saccharide	Putative saccharide and/or fatty acid	Ga0417192_01	Bc_Ga0417192_01_rn13	N	N	1792168	1852775	60608	25	15	NA	NA	
Bacillus cereus UW85	Putative saccharide	Putative saccharide and/or fatty acid	Ga0417192_01	Bc_Ga0417192_01_rn14	N	N	2090937	2114937	24001	50	0	NA	NA	
Bacillus cereus UW85	Putative saccharide	Putative saccharide and/or fatty acid	Ga0417192_01	Bc_Ga0417192_01_rn15	N	N	2165528	2203865	38338	33	11	NA	NA	
Bacillus cereus UW85	Putative saccharide	Putative saccharide and/or fatty acid	Ga0417192_01	Bc_Ga0417192_01_rn16	N	N	2320516	2370380	49865	15	0	NA	NA	
Bacillus cereus UW85	Putative saccharide	Putative saccharide and/or fatty acid	Ga0417192_01	Bc_Ga0417192_01_rn17	N	N	2376939	2395774	18836	33	0	NA	NA	
Bacillus cereus UW85	Putative saccharide	Putative saccharide and/or fatty acid	Ga0417192_01	Bc_Ga0417192_01_rn18	N	N	2711232	2732132	20901	27	0	NA	NA	
Bacillus cereus UW85	Lanthipeptide	RiPP	Ga0417192_01	Bc_Ga0417192_01_rn19	N	N	2780943	2803555	22613	22	0	NA	NA	Lanthipeptide synthase type III; Many precursor peptide ORFs, all except one identical, all leader cleavages and crosslinks predicted
Bacillus cereus UW85	NRPS-like	NRPS and/or NRPS-like	Ga0417192_01	Bc_Ga0417192_01_rn20	N	N	2840470	2882888	42419	97	0	NA	NA	Shares two genes with similarity to those in thiamine biosynthesis
Bacillus cereus UW85	Putative saccharide	Putative saccharide and/or fatty acid	Ga0417192_01	Bc_Ga0417192_01_rn21	N	N	2914349	2944323	29975	23	0	NA	NA	
Bacillus cereus UW85	NRPS, trans-AT PKS hybrid	NRPS, trans-AT PKS	Ga0417192_01	Bc_Ga0417192_01_rn22	N	N	2971419	3048690	77272	57	0	NA	NA	Predicted substrate specificity for 7 of 7 A-domains, 3 of 3 trans-AT KS domains unknown specificity
Bacillus cereus UW85	Putative saccharide	Putative saccharide and/or fatty acid	Ga0417192_01	Bc_Ga0417192_01_rn23	N	N	3105707	3139068	33362	13	0	NA	NA	Subcluster of schizokinen (siderophore) transporters; these may be involved with citrate-containing siderophore uptake (there's one in Fj)
Bacillus cereus UW85	Putative fatty acid	Putative saccharide and/or fatty acid	Ga0417192_01	Bc_Ga0417192_01_rn24	N	N	3632568	3653783	21216	12	7	NA	NA	fabHa and fabF homologs
Bacillus cereus UW85	Putative saccharide	Putative saccharide and/or fatty acid	Ga0417192_01	Bc_Ga0417192_01_rn25	N	N	3667108	3695940	28833	40	26	NA	NA	4 genes homolgos to S-layer glycan biosynthesis
Bacillus cereus UW85	Heterocycloanthracin - Maturation locus	RiPP	Ga0417192_01	Bc_Ga0417192_01_rn26	N	N	3704644	3728150	23507	95	0	NA	10.1186/s12864-015-2008-0; 10.1186/1745-6150-4-15	Two putative precursor loci elsewhere in genome; Leader/core cleavages predicted in both; predicted to form heterocycles in core peptide Cys, Ser, & Thr
Bacillus cereus UW85	Bacillithiol - Locus 1	Saccharide	Ga0417192_01	Bc_Ga0417192_01_rn27	Y	Y	3957750	3967738	9989	37	11	NA	10.1073/pnas.1000928107; 10.1099/mic.0.070482-0	Bacillithiol, locus 1 of 3...the others noted below
Bacillus cereus UW85	Bacillithiol - Locus 2	Saccharide	Ga0417192_01	NA	Y	Y	359289	360317	1029	NA	NA	NA	10.1073/pnas.1000928107; 10.1099/mic.0.070482-0	Minor overlap with Bc_Ga0417192_01_rn04
Bacillus cereus UW85	Bacillithiol - Locus 3	Saccharide	Ga0417192_01	NA	Y	Y	922878	925458	2581	NA	NA	NA	10.1073/pnas.1000928107; 10.1099/mic.0.070482-0	Minor overlap with Bc_Ga0417192_01_rn08
Bacillus cereus UW85	Putative saccharide	Putative saccharide and/or fatty acid	Ga0417192_01	Bc_Ga0417192_01_rn28	N	N	4001601	4018374	16774	19	25	NA	NA	2 genes shares with teichuronic acid
Bacillus cereus UW85	Putative fatty acid	Putative saccharide and/or fatty acid	Ga0417192_01	Bc_Ga0417192_01_rn29	N	N	4184893	4205876	20984	26	0	NA	NA	
Bacillus cereus UW85	Putative saccharide	Putative saccharide and/or fatty acid	Ga0417192_01	Bc_Ga0417192_01_rn30	N	N	4304182	4326290	22109	17	0	NA	NA	
Bacillus cereus UW85	Petrobactin	Siderophore	Ga0417192_01	Bc_Ga0417192_01_rn31	Y	Y	4351398	4358489	7092	100	100	Iron chelator	10.1099/mic.0.039404-0	
Bacillus cereus UW85	Bacillibactin	Siderophore	Ga0417192_01	Bc_Ga0417192_01_rn32	Y	Y	4686550	4704132	17583	100	100	Iron chelator	10.1099/mic.0.039404-0; 10.1021/acs.jnatprod.0c01170	has upstream standalone A domain so nicotinic and benzoic acid containing derivatives are possible
Bacillus cereus UW85	Nicotinate - Locus 1	Precursor	Ga0417192_01	NA	Y	Y	1387339	1390859	3521	NA	NA	Precursor	10.1021/acs.jnatprod.0c01170	
Bacillus cereus UW85	Nicotinate - Locus 2	Precursor	Ga0417192_01	NA	Y	Y	3089063	3090527	1465	NA	NA	Precursor	10.1021/acs.jnatprod.0c01170	check in concert with bacillibactin
Bacillus cereus UW85	Kurstakin	Lipopeptide	Ga0417192_01	Bc_Ga0417192_01_rn33	Y	Y	4835730	4864650	28921	94	0	Antibiotic (antifungal)	10.1007/s00253-012-4181-2	
Bacillus cereus UW85	Beta-lactone	Beta-lactone	Ga0417192_01	Bc_Ga0417192_01_rn34	N	N	4954380	4979618	25239	92	40	NA	NA	Some tailoring genes similar to fengycin, mycosbtilin, plipastatin
Bacillus cereus UW85	Putative saccharide	Putative saccharide and/or fatty acid	Ga0417192_01	Bc_Ga0417192_01_rn35	N	N	5002259	5022143	19885	9	0	NA	NA	
Bacillus cereus UW85	Heterocycloanthracin - Precursor locus 1	RiPP	Ga0417192_01	Bc_Ga0417192_01_rn36	N	N	5033502	5042252	8751	100	0	NA	10.1021/jacs.5b04682	Precursor for Heterocycloanthracin; leader/core predicted Bc_ctg1_4945
Bacillus cereus UW85	Heterocycloanthracin - Precursor locus 2	RiPP	Ga0417192_01	Bc_Ga0417192_01_rn37	N	N	5144490	5154756	10267	100	0	NA	10.1021/jacs.5b04682	Precursor for Heterocycloanthracin; leader/core predicted Bc_ctg1_5070
Bacillus cereus UW85	NRPS	NRPS and/or NRPS-like	Ga0417192_01	Bc_Ga0417192_01_rn38	N	N	5175581	5222597	47017	100	0	NA	NA	Small saccharide subcluster upstream
Bacillus cereus UW85	Putative saccharide	Putative saccharide and/or fatty acid	Ga0417192_02	Bc_Ga0417192_02_rn01	N	N	223	17451	17229	24	0	NA	NA	
Bacillus cereus UW85	NRPS	NRPS and/or NRPS-like	Ga0417192_02	Bc_Ga0417192_02_rn02	N	N	116527	178205	61679	85	0	NA	NA	Small saccharide subcluster downstream
Bacillus cereus UW85	Zwittermicin/Kanosamine	NRPS, trans-AT PKS, T1PKS	Ga0417192_02	Bc_Ga0417192_02_rn03	Y	Y	460133	521618	61486	80	100	Antibiotic	10.1128/AEM.02518-08; 10.1016/S0378-1119(99)00315-7	
Bacillus cereus UW85	Lanthipeptide	RiPP	Ga0417192_02	Bc_Ga0417192_02_rn04	N	N	553858	576452	22595	18	0	NA	NA	Lanthipeptide synthase type III; Many precursor peptide ORFs, all except one identical, all leader cleavages and crosslinks predicted; similar (but not identical) core as Cyanothecamide B ThcE2)
Bacillus cereus UW85	Putative saccharide	Putative saccharide and/or fatty acid	Ga0417192_03	Bc_Ga0417192_03_rn01	N	N	8589	51045	42457	91	26	NA	NA	Has ladderane biosynthesis gene at Bc_ctg3_25 that shares some similarity to pimelate biosynthesis
Bacillus cereus UW85	Thermocellin-like ranthipeptide	RiPP	Ga0417192_03	Bc_Ga0417192_03_rn02	N	N	553858	576452	22595	50	0	Quorum sensing?	10.1002/biot.202000136; 10.1021/acschembio.9b00457; 10.1021/jacs.9b01519	Leader/core cleavage and crosslinks predicted
Flavobacterium johnsoniae UW101	Putative carotenoid	Terpene	NC_009441.1	Fj_	N	N	54263	73868	19606	100	28	NA	NA	Synthase has homology to phytoene synthase
Flavobacterium johnsoniae UW101	Putative saccharide	Putative saccharide and/or fatty acid	NC_009441.1	Fj_	N	N	218744	243572	24829	30	0	NA	NA	
Flavobacterium johnsoniae UW101	Putative fatty acid	Putative saccharide and/or fatty acid	NC_009441.1	Fj_	N	N	265251	285840	20590	8	0	NA	NA	
Flavobacterium johnsoniae UW101	Putative saccharide	Putative saccharide and/or fatty acid	NC_009441.1	Fj_	N	N	299475	395366	95892	58	20	NA	NA	
Flavobacterium johnsoniae UW101	Putative fatty acid	Putative saccharide and/or fatty acid	NC_009441.1	Fj_	N	N	455167	475294	20128	21	0	NA	NA	
Flavobacterium johnsoniae UW101	Putative saccharide	Putative saccharide and/or fatty acid	NC_009441.1	Fj_	N	N	565444	600145	34702	47	0	NA	NA	
Flavobacterium johnsoniae UW101	Putative fatty acid	Putative saccharide and/or fatty acid	NC_009441.1	Fj_	N	N	696808	717204	20397	20	8	NA	NA	
Flavobacterium johnsoniae UW101	Putative saccharide	Putative saccharide and/or fatty acid	NC_009441.1	Fj_	N	N	776274	795861	19588	31	0	NA	NA	
Flavobacterium johnsoniae UW101	Putative fatty acid	Putative saccharide and/or fatty acid	NC_009441.1	Fj_	N	N	907269	926979	19711	60	0	NA	NA	
Flavobacterium johnsoniae UW101	Putative carotenoid	Terpene	NC_009441.1	Fj_	N	N	1029292	1050467	21176	75	0	NA	NA	Cyclase is similar to lycopene biosynthesis
Flavobacterium johnsoniae UW101	Flexirubin	Arylpolyene resorcinol	NC_009441.1	Fj_	Y	Y	1218315	1253377	35063	96	100	Antioxidative pigment	10.1002/cbic.201500474; 10.1128/AEM.01495-09; 10.1111/1751-7915.12110	
Flavobacterium johnsoniae UW101	Putative fatty acid	Putative saccharide and/or fatty acid	NC_009441.1	Fj_	N	N	1275383	1295681	20299	26	0	NA	NA	
Flavobacterium johnsoniae UW101	Putative saccharide	Putative saccharide and/or fatty acid	NC_009441.1	Fj_	N	N	1355264	1390648	35385	15	0	NA	NA	
Flavobacterium johnsoniae UW101	Putative saccharide	Putative saccharide and/or fatty acid	NC_009441.1	Fj_	N	N	1568060	1621464	53405	28	0	NA	NA	
Flavobacterium johnsoniae UW101	Putative saccharide	Putative saccharide and/or fatty acid	NC_009441.1	Fj_	N	N	1706690	1734561	27872	8	6	NA	NA	
Flavobacterium johnsoniae UW101	Putative saccharide	Putative saccharide and/or fatty acid	NC_009441.1	Fj_	N	N	1974873	2030973	56101	17	0	NA	NA	
Flavobacterium johnsoniae UW101	Putative saccharide	Putative saccharide and/or fatty acid	NC_009441.1	Fj_	N	N	2078580	2103000	24421	30	0	NA	NA	Shares some genes with peptidoglycan biosynthesis
Flavobacterium johnsoniae UW101	NRPS	NRPS and/or NRPS-like	NC_009441.1	Fj_	N	N	2456740	2558460	101721	100	0	NA	10.1128/AEM.01495-09	Large NRPS, 14 A-domains; mentioned in McBride et al, but not characterized
Flavobacterium johnsoniae UW101	Putative saccharide	Putative saccharide and/or fatty acid	NC_009441.1	Fj_	N	N	2682330	2708526	26197	18	0	NA	NA	
Flavobacterium johnsoniae UW101	Putative saccharide	Putative saccharide and/or fatty acid	NC_009441.1	Fj_	N	N	2891670	2912245	20576	14	0	NA	NA	
Flavobacterium johnsoniae UW101	Putative saccharide	Putative saccharide and/or fatty acid	NC_009441.1	Fj_	N	N	2988419	3017976	29558	36	0	NA	NA	
Flavobacterium johnsoniae UW101	Putative saccharide	Putative saccharide and/or fatty acid	NC_009441.1	Fj_	N	N	3262273	3285664	23392	18	0	NA	NA	
Flavobacterium johnsoniae UW101	Beta-lactone	Beta-lactone	NC_009441.1	Fj_	N	N	3380701	3411335	30635	92	0	NA	NA	
Flavobacterium johnsoniae UW101	Fulvivirgamide-like siderophore	Siderophore	NC_009441.1	Fj_	N	Y	3771887	3785326	13440	100	50	Iron chelator	10.1021/acs.orglett.9b04490	Likely has citrate because of absA homolog; biosynthetic hypothesis in gene sheet
Flavobacterium johnsoniae UW101	Putative fatty acid	Putative saccharide and/or fatty acid	NC_009441.1	Fj_	N	N	3841210	3861004	19795	18	0	NA	NA	
Flavobacterium johnsoniae UW101	T3PKS	T3PKS, putative fatty acid	NC_009441.1	Fj_	N	N	4496769	4537857	41089	97	0	NA	NA	
Flavobacterium johnsoniae UW101	Putative saccharide/fatty acid	Putative saccharide and/or fatty acid	NC_009441.1	Fj_	N	N	5182819	5221398	38580	14	0	NA	NA	
Flavobacterium johnsoniae UW101	Putative saccharide	Putative saccharide and/or fatty acid	NC_009441.1	Fj_	N	N	6015393	6042013	26621	47	0	NA	NA	
Pseudomonas koreensis CI12	Putative saccharide	Putative saccharide and/or fatty acid	Ga0417193_01	Pk_	N	N	405367	425403	20037	30	0	NA	NA	
Pseudomonas koreensis CI12	Putative saccharide	Putative saccharide and/or fatty acid	Ga0417193_01	Pk_	N	N	798703	821914	23212	35	0	NA	NA	
Pseudomonas koreensis CI12	Putative saccharide	Putative saccharide and/or fatty acid	Ga0417193_01	Pk_	N	N	875897	910923	35027	81	1	NA	NA	
Pseudomonas koreensis CI12	Putative fatty acid	Putative saccharide and/or fatty acid	Ga0417193_01	Pk_	N	N	1370699	1390954	20256	20	0	NA	NA	3-oxoacyl-ACP synthase @ Pk_ctg1_1237, possible koreenceine precursor biosynthesis
Pseudomonas koreensis CI12	Putative fatty acid	Putative saccharide and/or fatty acid	Ga0417193_01	Pk_	N	N	1434994	1452893	17900	46	0	NA	NA	3-oxoacyl-ACP synthase @ Pk_ctg1_1296, possible koreenceine precursor biosynthesis
Pseudomonas koreensis CI12	N-acetylglutaminylglutamine amide	N-acetylglutaminylglutamine amide	Ga0417193_01	Pk_	Y	Y	1494162	1499067	4906	100	0	Osmoprotectant	10.1073/pnas.1003063107; 10.3390/ijms22083982	* note, no marR reg like pa
Pseudomonas koreensis CI12	Putative saccharide/fatty acid	Putative saccharide and/or fatty acid	Ga0417193_01	Pk_	N	N	1531499	1576015	44517	28	35	NA	NA	3-oxoacyl-ACP synthase @ Pk_ctg1_1407, possible koreenceine precursor biosynthesis
Pseudomonas koreensis CI12	Pyoverdine-like siderophore - chromophor	Siderophore	Ga0417193_01	Pk_	N	Y	1707518	1740915	33398	100	19	Iron chelator	10.15698/mic2018.10.649; 10.1007/s10534-009-9247-y	
Pseudomonas koreensis CI12	Putative saccharide	Putative saccharide and/or fatty acid	Ga0417193_01	Pk_	N	N	1825093	1861531	36439	26	0	NA	NA	
Pseudomonas koreensis CI12	Beta-lactone	Beta-lactone	Ga0417193_01	Pk_	N	N	2065543	2088807	23265	100	20	NA	NA	
Pseudomonas koreensis CI12	Lokisin	NRPS and/or NRPS-like	Ga0417193_01	Pk_	Y	Y	2403548	2449906	46359	93	100	Antibiotic (antifungal)	10.1016/S0040-4039(02)00856-0; 10.3389/fpls.2019.00901	
Pseudomonas koreensis CI12	Putative saccharide	Putative saccharide and/or fatty acid	Ga0417193_01	Pk_	N	N	2758498	2802581	44084	47	43	NA	10.1002/cbic.201500413	Shares some genes with the pseudopyronine BGC in MIBiG, but these are just flanking pseudopyronine and not involved in the biosynthesis
Pseudomonas koreensis CI12	Putative saccharide	Putative saccharide and/or fatty acid	Ga0417193_01	Pk_	N	N	2901089	2921561	20473	17	0	Putative biofilm promotion	10.1021/acs.biochem.8b00149	arnB and arnC homologs, might be involved with Lipid A remodeling to promote biofilm formation
Pseudomonas koreensis CI12	Putative saccharide	Putative saccharide and/or fatty acid	Ga0417193_01	Pk_	N	N	2955130	2980127	24998	40	0	NA	NA	
Pseudomonas koreensis CI12	Putative siderophore/saccharide	Siderophore	Ga0417193_01	Pk_	N	N	3548386	3585714	37329	100	0	Iron chelator	NA	Likely citrate based because has a iucA/iucC homolog
Pseudomonas koreensis CI12	Putative bacteriocin	Putative bacteriocin/RiPP	Ga0417193_01	Pk_	N	N	3758172	3768363	10192	100	0	NA	NA	
Pseudomonas koreensis CI12	Putative saccharide	Putative saccharide and/or fatty acid	Ga0417193_01	Pk_	N	N	3800402	3839735	39334	15	6	NA	NA	
Pseudomonas koreensis CI12	Pyoverdine-like siderophore - peptide bac	Siderophore	Ga0417193_01	Pk_	N	Y	4089121	4156632	67512	96	31	Iron chelator	10.15698/mic2018.10.649; 10.1007/s10534-009-9247-y	
Pseudomonas koreensis CI12	Putative saccharide/fatty acid	Putative saccharide and/or fatty acid	Ga0417193_01	Pk_	N	N	4513920	4552536	38617	45	22	NA	NA	
Pseudomonas koreensis CI12	Putative fatty acid	Putative saccharide and/or fatty acid	Ga0417193_01	Pk_	N	N	4651199	4671271	20073	22	0	NA	NA	3-oxoacyl-ACP synthase @ Pk_ctg1_4125, possible koreenceine precursor biosynthesis
Pseudomonas koreensis CI12	Putative saccharide	Putative saccharide and/or fatty acid	Ga0417193_01	Pk_	N	N	4697802	4721744	23943	24	0	NA	NA	
Pseudomonas koreensis CI12	Putative bacteriocin	Putative bacteriocin/RiPP	Ga0417193_01	Pk_	N	N	4848664	4859551	10888	100	0	NA	NA	
Pseudomonas koreensis CI12	Putative saccharide	Putative saccharide and/or fatty acid	Ga0417193_01	Pk_	N	N	5247917	5282357	34441	18	6	NA	NA	
Pseudomonas koreensis CI12	Koreenceine	Alkaloid fatty acid	Ga0417193_01	Pk_	Y	Y	5479976	5494664	14689	21	100	Antibiotic (antibacterial)	10.1128/AEM.03058-18; 10.1128/mBio.02846-18	
Pseudomonas koreensis CI12	Putative saccharide	Putative saccharide and/or fatty acid	Ga0417193_01	Pk_	N	N	5759644	5803786	44143	24	40	NA	NA	
Pseudomonas koreensis CI12	Putative arylpolyene	Arylpolyene	Ga0417193_01	Pk_	N	N	5831776	5875380	43605	97	40	NA	NA	Possible koreenceine precursor, but unlikely unless -enes are reduced
Pseudomonas koreensis CI12	Fragin-like	NRPS and/or NRPS-like	Ga0417193_01	Pk_	N	N	6169113	6212505	43393	100	60	NA	NA	
Pseudomonas koreensis CI12	Putative saccharide	Putative saccharide and/or fatty acid	Ga0417193_01	Pk_	N	N	6346810	6379143	32334	57	12	NA	NA	

The table following this page is:

Table S2

for manuscript:

Chevrette et al. "Microbiome composition modulates secondary metabolism in a multispecies bacterial community." *Proceedings of the National Academy of Sciences*. 2022.

All supplemental materials for this manuscript can be found at:

https://github.com/chevrm/thor_secmet/tree/main/figures/sup

Please refer to the cover sheet of this PDF for the exact file location on GitHub.

organism	bgc	contig	gene	ann	replace_antismash_bgc	ann_verbose	ann_acc	ann_percent_prot_id	notes
Bacillus cereus UW85	Pulcherriminic acid	Ga0417192_01	Bc_ctg1_118	pchE	Bc_Ga0417192_01_rn01	pulcherriminic acid efflux transporter	CAB15514.1	52.8	
Bacillus cereus UW85	Pulcherriminic acid	Ga0417192_01	Bc_ctg1_119	pchR	Bc_Ga0417192_01_rn01	marR-like regulator	CAB15513.1	53.3	
Bacillus cereus UW85	Pulcherriminic acid	Ga0417192_01	Bc_ctg1_120	yvnA	Bc_Ga0417192_01_rn01	marR-like regulator	NP_391385.1	42.2	
Bacillus cereus UW85	Pulcherriminic acid	Ga0417192_01	Bc_ctg1_121	cypX	Bc_Ga0417192_01_rn01	cytochrome p450	CAB15511.1	73.9	
Bacillus cereus UW85	Pulcherriminic acid	Ga0417192_01	Bc_ctg1_122	pchC	Bc_Ga0417192_01_rn01	cyclodipeptide synthase	CAB15512.1	63.3	
Bacillus cereus UW85	Bacillithiol - Locus 1	Ga0417192_01	Bc_ctg1_3901	ypjC	Bc_Ga0417192_01_rn27	inner membrane protein	CAB14167.2	73	
Bacillus cereus UW85	Bacillithiol - Locus 1	Ga0417192_01	Bc_ctg1_3902	ypjD	Bc_Ga0417192_01_rn27	oxidized nucleotide pyrophosphohydrolase	CAB14166.1	71	
Bacillus cereus UW85	Bacillithiol - Locus 1	Ga0417192_01	Bc_ctg1_3903	dapB	Bc_Ga0417192_01_rn27	(4S)-4-hydroxy-2,3,4, 5-tetrahydro-(2S)-dipicolinic acid (HTPA) dehydratase reductase	CAB14165.1	70.6	
Bacillus cereus UW85	Bacillithiol - Locus 1	Ga0417192_01	Bc_ctg1_3904	mgsA	Bc_Ga0417192_01_rn27	methylglyoxal synthase	CAB14164.1	67.3	
Bacillus cereus UW85	Bacillithiol - Locus 1	Ga0417192_01	Bc_ctg1_3905	bshB1	Bc_Ga0417192_01_rn27	N-acetyl-alpha-D-glucosaminyl L-malate deacetylase	CAB14163.2	59.1	
Bacillus cereus UW85	Bacillithiol - Locus 1	Ga0417192_01	Bc_ctg1_3906	bshA	Bc_Ga0417192_01_rn27	N-acetyl-alpha-D-glucosaminyl L-malate synthase	CAB14162.1	61.8	
Bacillus cereus UW85	Bacillithiol - Locus 1	Ga0417192_01	Bc_ctg1_3907	cca	Bc_Ga0417192_01_rn27	tRNA nucleotidyltransferase	CAB14161.1	41.8	
Bacillus cereus UW85	Bacillithiol - Locus 1	Ga0417192_01	Bc_ctg1_3908	birA	Bc_Ga0417192_01_rn27	biotin acetyl-CoA-carboxylase ligase	CAB14160.1	54.9	
Bacillus cereus UW85	Bacillithiol - Locus 1	Ga0417192_01	Bc_ctg1_3910	panB	Bc_Ga0417192_01_rn27	ketopantoate hydroxymethyltransferase	CAB14159.1	67	
Bacillus cereus UW85	Bacillithiol - Locus 1	Ga0417192_01	Bc_ctg1_3911	panC	Bc_Ga0417192_01_rn27	pantothenate synthetase	CAB14158.1	55.9	
Bacillus cereus UW85	Bacillithiol - Locus 1	Ga0417192_01	Bc_ctg1_3912	panD	Bc_Ga0417192_01_rn27	aspartate 1-decarboxylase	CAB14157.1	56.5	
Bacillus cereus UW85	Bacillithiol - Locus 2	Ga0417192_01	Bc_ctg1_356	yojF	NA	uncharacterized protein	O31858.1	53.8	
Bacillus cereus UW85	Bacillithiol - Locus 2	Ga0417192_01	Bc_ctg1_355	bchB2	NA	N-acetyl-alpha-D-glucosaminyl L-malate deacetylase	CAB13838.2	56.1	
Bacillus cereus UW85	Bacillithiol - Locus 3	Ga0417192_01	Bc_ctg1_896	ylbQ	NA	2-dehydropantoate 2-reductase	CAB13384.1	43.6	
Bacillus cereus UW85	Bacillithiol - Locus 3	Ga0417192_01	Bc_ctg1_895	bshC	NA	malate glucosamine cysteine ligase	CAB13385.2	42	
Bacillus cereus UW85	Petrobactin	Ga0417192_01	Bc_ctg1_4289	asbA	Bc_Ga0417192_01_rn31	spermidine-citrate ligase	A0A0F7RJ52.1	91.7	
Bacillus cereus UW85	Petrobactin	Ga0417192_01	Bc_ctg1_4290	asbB	Bc_Ga0417192_01_rn31	citryl-spermidine/3,4-dihydroxybenzoyl-citryl-spermidine:spermidine ligase	Q81RQ8.1	84	
Bacillus cereus UW85	Petrobactin	Ga0417192_01	Bc_ctg1_4291	asbC	Bc_Ga0417192_01_rn31	3,4-dihydroxybenzoic acid-AMP ligase	Q81RQ7.1	92.2	
Bacillus cereus UW85	Petrobactin	Ga0417192_01	Bc_ctg1_4292	asbD	Bc_Ga0417192_01_rn31	acyl carrier protein	A0A0J1I1I3.1	96.7	
Bacillus cereus UW85	Petrobactin	Ga0417192_01	Bc_ctg1_4293	asbE	Bc_Ga0417192_01_rn31	petrobactin synthase	Q81RQ5.1	92.9	
Bacillus cereus UW85	Petrobactin	Ga0417192_01	Bc_ctg1_4294	asbF	Bc_Ga0417192_01_rn31	3-dehydroshikimate dehydratase	Q81RQ4.1	88.9	
Bacillus cereus UW85	Bacillibactin	Ga0417192_01	Bc_ctg1_4639	pagR	Bc_Ga0417192_01_rn32	transcriptional repressor (metal binding)	O31178.1	53.1	
Bacillus cereus UW85	Bacillibactin	Ga0417192_01	Bc_ctg1_4640	ykvS	Bc_Ga0417192_01_rn32	uncharacterized protein	O31684.2	40.4	
Bacillus cereus UW85	Bacillibactin	Ga0417192_01	Bc_ctg1_4641	yhjX	Bc_Ga0417192_01_rn32	MFS transporter	P37662.1	44.8	
Bacillus cereus UW85	Bacillibactin	Ga0417192_01	Bc_ctg1_4642	dhbA	Bc_Ga0417192_01_rn32	2,3-dihydro-2,3-dihydroxybenzoate dehydrogenase	CAB15190.2	73.9	
Bacillus cereus UW85	Bacillibactin	Ga0417192_01	Bc_ctg1_4643	dhbC	Bc_Ga0417192_01_rn32	isochorismate synthase	CAB15189.2	65.8	
Bacillus cereus UW85	Bacillibactin	Ga0417192_01	Bc_ctg1_4644	dhbE	Bc_Ga0417192_01_rn32	2,3-dihydroxybenzoate-AMP ligase	CAB15188.1	74.4	
Bacillus cereus UW85	Bacillibactin	Ga0417192_01	Bc_ctg1_4645	dhbB	Bc_Ga0417192_01_rn32	isochorismatase	CAB15187.1	57.1	
Bacillus cereus UW85	Bacillibactin	Ga0417192_01	Bc_ctg1_4646	dhbF	Bc_Ga0417192_01_rn32	siderophore 2,3-dihydroxybenzoate-glycine-threonine trimeric ester bacillibactin synthetase	CAB15186.3	64.9	
Bacillus cereus UW85	Bacillibactin	Ga0417192_01	Bc_ctg1_4647	ybdZ	Bc_Ga0417192_01_rn32	uncharacterized protein	C0H3Q6.1	56.7	
Bacillus cereus UW85	Bacillibactin	Ga0417192_01	Bc_ctg1_4648	yhcA	Bc_Ga0417192_01_rn32	MFS transporter	P54585.2	31.9	
Bacillus cereus UW85	Bacillibactin	Ga0417192_01	Bc_ctg1_4649	hetI	Bc_Ga0417192_01_rn32	4'-phosphopantetheinyl transferase	P37695.2	32.1	
Bacillus cereus UW85	Bacillibactin	Ga0417192_01	Bc_ctg1_4650		Bc_Ga0417192_01_rn32	hypothetical protein	WP_000959120.1	100	
Bacillus cereus UW85	Nicotinate - Locus 1	Ga0417192_01	Bc_ctg1_1410	nadA	NA	quinolinate synthetase	NP_390663.1	69.9	
Bacillus cereus UW85	Nicotinate - Locus 1	Ga0417192_01	Bc_ctg1_1411	nadC	NA	nicotinate-nucleotide pyrophosphorylase	NP_390664.1	44.6	
Bacillus cereus UW85	Nicotinate - Locus 1	Ga0417192_01	Bc_ctg1_1412	nadB	NA	L-aspartate oxidase	NP_390665.1	57.5	
Bacillus cereus UW85	Nicotinate - Locus 2	Ga0417192_01	Bc_ctg1_3016	pchB	NA	nicotinate phosphoribosyltransferase	NP_391053.1	68.7	
Bacillus cereus UW85	Kurstakin	Ga0417192_01	Bc_ctg1_4789	krsE	Bc_Ga0417192_01_rn33	Major facilitator superfamily MFS_1	EEM74021.1	98.3	
Bacillus cereus UW85	Kurstakin	Ga0417192_01	Bc_ctg1_4790	krsA	Bc_Ga0417192_01_rn33	NRPS	EEM74022.1	93.3	
Bacillus cereus UW85	Kurstakin	Ga0417192_01	Bc_ctg1_4791	krsB	Bc_Ga0417192_01_rn33	NRPS	EEM74023.1	95.7	
Bacillus cereus UW85	Kurstakin	Ga0417192_01	Bc_ctg1_4792	krsC	Bc_Ga0417192_01_rn33	NRPS	EEM74024.1	95.9	
Bacillus cereus UW85	Kurstakin	Ga0417192_01	Bc_ctg1_4793	sfp	Bc_Ga0417192_01_rn33	4'-phosphopantetheinyl transferase	EEM74025.1	96	
Bacillus cereus UW85	Kurstakin	Ga0417192_01	Bc_ctg1_4794	krsD	Bc_Ga0417192_01_rn33	type II thioesterase	EEM74026.1	92.1	
Bacillus cereus UW85	Zwittermicin/Kanosamine	Ga0417192_02	Bc_ctg2_422	zmaA	Bc_Ga0417192_02_rn03				
Bacillus cereus UW85	Zwittermicin/Kanosamine	Ga0417192_02	Bc_ctg2_423	zmaB	Bc_Ga0417192_02_rn03				
Bacillus cereus UW85	Zwittermicin/Kanosamine	Ga0417192_02	Bc_ctg2_424	zmaC	Bc_Ga0417192_02_rn03				
Bacillus cereus UW85	Zwittermicin/Kanosamine	Ga0417192_02	Bc_ctg2_425	zmaD	Bc_Ga0417192_02_rn03				
Bacillus cereus UW85	Zwittermicin/Kanosamine	Ga0417192_02	Bc_ctg2_426	zmaE	Bc_Ga0417192_02_rn03				
Bacillus cereus UW85	Zwittermicin/Kanosamine	Ga0417192_02	Bc_ctg2_427	zmaR	Bc_Ga0417192_02_rn03				
Bacillus cereus UW85	Zwittermicin/Kanosamine	Ga0417192_02	Bc_ctg2_428	zmaF	Bc_Ga0417192_02_rn03				
Bacillus cereus UW85	Zwittermicin/Kanosamine	Ga0417192_02	Bc_ctg2_429	zmaG	Bc_Ga0417192_02_rn03				
Bacillus cereus UW85	Zwittermicin/Kanosamine	Ga0417192_02	Bc_ctg2_430	zmaH	Bc_Ga0417192_02_rn03				
Bacillus cereus UW85	Zwittermicin/Kanosamine	Ga0417192_02	Bc_ctg2_431	zmaI	Bc_Ga0417192_02_rn03				
Bacillus cereus UW85	Zwittermicin/Kanosamine	Ga0417192_02	Bc_ctg2_432	zmaJ	Bc_Ga0417192_02_rn03				
Bacillus cereus UW85	Zwittermicin/Kanosamine	Ga0417192_02	Bc_ctg2_433	zmaK	Bc_Ga0417192_02_rn03				
Bacillus cereus UW85	Zwittermicin/Kanosamine	Ga0417192_02	Bc_ctg2_434	zmaL	Bc_Ga0417192_02_rn03				
Bacillus cereus UW85	Zwittermicin/Kanosamine	Ga0417192_02	Bc_ctg2_435	zmaM	Bc_Ga0417192_02_rn03				
Bacillus cereus UW85	Zwittermicin/Kanosamine	Ga0417192_02	Bc_ctg2_436	zmaN	Bc_Ga0417192_02_rn03				
Bacillus cereus UW85	Zwittermicin/Kanosamine	Ga0417192_02	Bc_ctg2_437	zmaO	Bc_Ga0417192_02_rn03				
Bacillus cereus UW85	Zwittermicin/Kanosamine	Ga0417192_02	Bc_ctg2_438	zmaP	Bc_Ga0417192_02_rn03				
Bacillus cereus UW85	Zwittermicin/Kanosamine	Ga0417192_02	Bc_ctg2_439	zmaQ	Bc_Ga0417192_02_rn03				
Bacillus cereus UW85	Zwittermicin/Kanosamine	Ga0417192_02	Bc_ctg2_440	zmaS	Bc_Ga0417192_02_rn03				
Bacillus cereus UW85	Zwittermicin/Kanosamine	Ga0417192_02	Bc_ctg2_441	kabR	Bc_Ga0417192_02_rn03				
Bacillus cereus UW85	Zwittermicin/Kanosamine	Ga0417192_02	Bc_ctg2_442	kabA	Bc_Ga0417192_02_rn03				
Bacillus cereus UW85	Zwittermicin/Kanosamine	Ga0417192_02	Bc_ctg2_443	kabB	Bc_Ga0417192_02_rn03				
Bacillus cereus UW85	Zwittermicin/Kanosamine	Ga0417192_02	Bc_ctg2_444	kabC	Bc_Ga0417192_02_rn03				
Bacillus cereus UW85	Zwittermicin/Kanosamine	Ga0417192_02	Bc_ctg2_445	kabD	Bc_Ga0417192_02_rn03				
Bacillus cereus UW85	Zwittermicin/Kanosamine	Ga0417192_02	Bc_ctg2_446	zmaT	Bc_Ga0417192_02_rn03				
Bacillus cereus UW85	Zwittermicin/Kanosamine	Ga0417192_02	Bc_ctg2_447	zmaU	Bc_Ga0417192_02_rn03				
Bacillus cereus UW85	Zwittermicin/Kanosamine	Ga0417192_02	Bc_ctg2_448	zmaV	Bc_Ga0417192_02_rn03				
Flavobacterium johnsoniae UW101	Flexirubin	NC_009441.1	Fj_ctg1_1108	Fjoh_1075	Fj_NC_009441.1_rn11				
Flavobacterium johnsoniae UW101	Flexirubin	NC_009441.1	Fj_ctg1_1109	Fjoh_1076	Fj_NC_009441.1_rn11				
Flavobacterium johnsoniae UW101	Flexirubin	NC_009441.1	Fj_ctg1_1110	Fjoh_1077	Fj_NC_009441.1_rn11				
Flavobacterium johnsoniae UW101	Flexirubin	NC_009441.1	Fj_ctg1_1111	Fjoh_1078	Fj_NC_009441.1_rn11				
Flavobacterium johnsoniae UW101	Flexirubin	NC_009441.1	Fj_ctg1_1112	Fjoh_1079	Fj_NC_009441.1_rn11				
Flavobacterium johnsoniae UW101	Flexirubin	NC_009441.1	Fj_ctg1_1113	Fjoh_1080	Fj_NC_009441.1_rn11				
Flavobacterium johnsoniae UW101	Flexirubin	NC_009441.1	Fj_ctg1_1114	Fjoh_1081	Fj_NC_009441.1_rn11				
Flavobacterium johnsoniae UW101	Flexirubin	NC_009441.1	Fj_ctg1_1115	Fjoh_1082	Fj_NC_009441.1_rn11				
Flavobacterium johnsoniae UW101	Flexirubin	NC_009441.1	Fj_ctg1_1116	Fjoh_1083	Fj_NC_009441.1_rn11				
Flavobacterium johnsoniae UW101	Flexirubin	NC_009441.1	Fj_ctg1_1117	Fjoh_1084	Fj_NC_009441.1_rn11				
Flavobacterium johnsoniae UW101	Flexirubin	NC_009441.1	Fj_ctg1_1118	Fjoh_1085	Fj_NC_009441.1_rn11				
Flavobacterium johnsoniae UW101	Flexirubin	NC_009441.1	Fj_ctg1_1119	Fjoh_1086	Fj_NC_009441.1_rn11				
Flavobacterium johnsoniae UW101	Flexirubin	NC_009441.1	Fj_ctg1_1120	Fjoh_1087	Fj_NC_009441.1_rn11				
Flavobacterium johnsoniae UW101	Flexirubin	NC_009441.1	Fj_ctg1_1121	Fjoh_1088	Fj_NC_009441.1_rn11				
Flavobacterium johnsoniae UW101	Flexirubin	NC_009441.1	Fj_ctg1_1122	Fjoh_1089	Fj_NC_009441.1_rn11				
Flavobacterium johnsoniae UW101	Flexirubin	NC_009441.1	Fj_ctg1_1123	Fjoh_1090	Fj_NC_009441.1_rn11				
Flavobacterium johnsoniae UW101	Flexirubin	NC_009441.1	Fj_ctg1_1124	Fjoh_1091	Fj_NC_009441.1_rn11				
Flavobacterium johnsoniae UW101	Flexirubin	NC_009441.1	Fj_ctg1_1125	Fjoh_1092	Fj_NC_009441.1_rn11				
Flavobacterium johnsoniae UW101	Flexirubin	NC_009441.1	Fj_ctg1_1126	Fjoh_1093	Fj_NC_009441.1_rn11				
Flavobacterium johnsoniae UW101	Flexirubin	NC_009441.1	Fj_ctg1_1127	Fjoh_1094	Fj_NC_009441.1_rn11				
Flavobacterium johnsoniae UW101	Flexirubin	NC_009441.1	Fj_ctg1_1128	Fjoh_1095	Fj_NC_009441.1_rn11				
Flavobacterium johnsoniae UW101	Flexirubin	NC_009441.1	Fj_ctg1_1129	Fjoh_1096	Fj_NC_009441.1_rn11				
Flavobacterium johnsoniae UW101	Flexirubin	NC_009441.1	Fj_ctg1_1130	Fjoh_1097	Fj_NC_009441.1_rn11				
Flavobacterium johnsoniae UW101	Flexirubin	NC_009441.1	Fj_ctg1_1131	Fjoh_1098	Fj_NC_009441.1_rn11				
Flavobacterium johnsoniae UW101	Flexirubin	NC_009441.1	Fj_ctg1_1132	Fjoh_1100	Fj_NC_009441.1_rn11				
Flavobacterium johnsoniae UW101	Flexirubin	NC_009441.1	Fj_ctg1_1133	Fjoh_1101	Fj_NC_009441.1_rn11				

Flavobacterium johnsoniae UW101	Flexirubin	NC_009441.1	Fj_ctg1_1134	darB	Fj_NC_009441.1_rn11				
Flavobacterium johnsoniae UW101	Flexirubin	NC_009441.1	Fj_ctg1_1135	darA	Fj_NC_009441.1_rn11				
Flavobacterium johnsoniae UW101	Flexirubin	NC_009441.1	Fj_ctg1_1136	Fjoh_1104	Fj_NC_009441.1_rn11				
Flavobacterium johnsoniae UW101	Flexirubin	NC_009441.1	Fj_ctg1_1137	Fjoh_1105	Fj_NC_009441.1_rn11				
Flavobacterium johnsoniae UW101	Flexirubin	NC_009441.1	Fj_ctg1_1138	Fjoh_1106	Fj_NC_009441.1_rn11				
Flavobacterium johnsoniae UW101	Flexirubin	NC_009441.1	Fj_ctg1_1139	Fjoh_1107	Fj_NC_009441.1_rn11				
Flavobacterium johnsoniae UW101	Flexirubin	NC_009441.1	Fj_ctg1_1140	Fjoh_1108	Fj_NC_009441.1_rn11				
Flavobacterium johnsoniae UW101	Flexirubin	NC_009441.1	Fj_ctg1_1141	Fjoh_1109	Fj_NC_009441.1_rn11				
Flavobacterium johnsoniae UW101	Flexirubin	NC_009441.1	Fj_ctg1_1142	Fjoh_1110	Fj_NC_009441.1_rn11				
Flavobacterium johnsoniae UW101	Fulvivirgamide-like siderophore	NC_009441.1	Fj_ctg1_3250	Fjoh_3169	Fj_NC_009441.1_rn24	L-lysine 4-hydroxylase			l-lys to (4R)-4-hydroxy-L-lys (stero-specif. hydroxyl on the gamma carbon)
Flavobacterium johnsoniae UW101	Fulvivirgamide-like siderophore	NC_009441.1	Fj_ctg1_3251	Fjoh_3170	Fj_NC_009441.1_rn24	NAD(P)H nitroreductase			nitroreductase…will reduce a N
Flavobacterium johnsoniae UW101	Fulvivirgamide-like siderophore	NC_009441.1	Fj_ctg1_3252	fulA	Fj_NC_009441.1_rn24	pyridoxal-dependent decarboxylase	QHG11726.1	50.8	strip the cooh off l-lys… makes cadaverine precursor
Flavobacterium johnsoniae UW101	Fulvivirgamide-like siderophore	NC_009441.1	Fj_ctg1_3253	Fjoh_3172	Fj_NC_009441.1_rn24	Spermidine-citrate ligase, iucA-like siderophore synthesis protein			asbA (petrobactin), rhbC (rhizobactin), iucA (aerobactin) homologs; these all take citrate/citric acid and attach it to an NH2
Flavobacterium johnsoniae UW101	Fulvivirgamide-like siderophore	NC_009441.1	Fj_ctg1_3254	fulB	Fj_NC_009441.1_rn24	monooxygenase	QHG11727.1	44.7	install hydroxyl on one of the NH2 ends (now NHOH)
Flavobacterium johnsoniae UW101	Fulvivirgamide-like siderophore	NC_009441.1	Fj_ctg1_3255	fulD	Fj_NC_009441.1_rn24	ATP-dependent siderophore synthetase	QHG11730.1	42.3	put monomers together
Flavobacterium johnsoniae UW101	Fulvivirgamide-like siderophore	NC_009441.1	Fj_ctg1_3256	Fjoh_3175	Fj_NC_009441.1_rn24	MATE family efflux transporter			transport
Flavobacterium johnsoniae UW101	Fulvivirgamide-like siderophore	NC_009441.1	Fj_ctg1_3257	Fjoh_3176	Fj_NC_009441.1_rn24	acyl carrier protein			acp…load?
Flavobacterium johnsoniae UW101	Fulvivirgamide-like siderophore	NC_009441.1	Fj_ctg1_3258	Fjoh_3177	Fj_NC_009441.1_rn24	Long-chain acyl-CoA synthetase			coA ligase….make acyl tail?
Flavobacterium johnsoniae UW101	Fulvivirgamide-like siderophore	NC_009441.1	Fj_ctg1_3259	fulF	Fj_NC_009441.1_rn24	Acyl transferase	QHG11731.1	42.3	attach acyl tail
Pseudomonas koreensis CI12	N-acetylglutaminylglutamine amide	Ga0417193_01	Pk_ctg1_1352	naggn peptidase	Pk_Ga0417193_01_rn06	NAGGN peptidase	NP_252151.1	78.9	
Pseudomonas koreensis CI12	N-acetylglutaminylglutamine amide	Ga0417193_01	Pk_ctg1_1353	ngg	Pk_Ga0417193_01_rn06	acetyltransferase	NP_252150.1	73.9	
Pseudomonas koreensis CI12	N-acetylglutaminylglutamine amide	Ga0417193_01	Pk_ctg1_1354	asnO	Pk_Ga0417193_01_rn06	glutamine amidotransferase	NP_252149.1	79.5	
Pseudomonas koreensis CI12	Pyoverdine-like siderophore - chromophore NRPS locus	Ga0417193_01	Pk_ctg1_1541		Pk_Ga0417193_01_rn08				
Pseudomonas koreensis CI12	Pyoverdine-like siderophore - chromophore NRPS locus	Ga0417193_01	Pk_ctg1_1542		Pk_Ga0417193_01_rn08				
Pseudomonas koreensis CI12	Pyoverdine-like siderophore - chromophore NRPS locus	Ga0417193_01	Pk_ctg1_1543	pvdZ	Pk_Ga0417193_01_rn08	siderophore biosynthesis protein	CAK14654.1	75.6	
Pseudomonas koreensis CI12	Pyoverdine-like siderophore - chromophore NRPS locus	Ga0417193_01	Pk_ctg1_1544	pvdS	Pk_Ga0417193_01_rn08	ferric regulator	CAK14655.1	87.8	
Pseudomonas koreensis CI12	Pyoverdine-like siderophore - chromophore NRPS locus	Ga0417193_01	Pk_ctg1_1545	pvsA	Pk_Ga0417193_01_rn08	nrps - pyoverdine synthetase A	CAK14656.1	73	
Pseudomonas koreensis CI12	Pyoverdine-like siderophore - chromophore NRPS locus	Ga0417193_01	Pk_ctg1_1546		Pk_Ga0417193_01_rn08				
Pseudomonas koreensis CI12	Pyoverdine-like siderophore - chromophore NRPS locus	Ga0417193_01	Pk_ctg1_1547		Pk_Ga0417193_01_rn08				
Pseudomonas koreensis CI12	Pyoverdine-like siderophore - chromophore NRPS locus	Ga0417193_01	Pk_ctg1_1548		Pk_Ga0417193_01_rn08				
Pseudomonas koreensis CI12	Pyoverdine-like siderophore - chromophore NRPS locus	Ga0417193_01	Pk_ctg1_1549		Pk_Ga0417193_01_rn08				
Pseudomonas koreensis CI12	Pyoverdine-like siderophore - chromophore NRPS locus	Ga0417193_01	Pk_ctg1_1550		Pk_Ga0417193_01_rn08				
Pseudomonas koreensis CI12	Pyoverdine-like siderophore - chromophore NRPS locus	Ga0417193_01	Pk_ctg1_1551		Pk_Ga0417193_01_rn08				
Pseudomonas koreensis CI12	Pyoverdine-like siderophore - chromophore NRPS locus	Ga0417193_01	Pk_ctg1_1552		Pk_Ga0417193_01_rn08				
Pseudomonas koreensis CI12	Pyoverdine-like siderophore - chromophore NRPS locus	Ga0417193_01	Pk_ctg1_1553		Pk_Ga0417193_01_rn08				
Pseudomonas koreensis CI12	Pyoverdine-like siderophore - chromophore NRPS locus	Ga0417193_01	Pk_ctg1_1554	dat	Pk_Ga0417193_01_rn08	diaminobutyrate--2-oxoglutarate aminotransferase	CAK16394.1	84.8	
Pseudomonas koreensis CI12	Pyoverdine-like siderophore - chromophore NRPS locus	Ga0417193_01	Pk_ctg1_1555	mbtH	Pk_Ga0417193_01_rn08	mbtH family protein	WP_064391340.1	100	
Pseudomonas koreensis CI12	Pyoverdine-like siderophore - chromophore NRPS locus	Ga0417193_01	Pk_ctg1_1556		Pk_Ga0417193_01_rn08	conserved hypothetical protein, putative signal peptide	CAK16000.1	54.2	
Pseudomonas koreensis CI12	Pyoverdine-like siderophore - chromophore NRPS locus	Ga0417193_01	Pk_ctg1_1557		Pk_Ga0417193_01_rn08	putative cation ABC transporter, permease protein	CAK15999.1	85.9	
Pseudomonas koreensis CI12	Pyoverdine-like siderophore - chromophore NRPS locus	Ga0417193_01	Pk_ctg1_1558		Pk_Ga0417193_01_rn08	putative cation ABC transporter, ATP-binding protein	CAK15998.1	81.1	
Pseudomonas koreensis CI12	Pyoverdine-like siderophore - chromophore NRPS locus	Ga0417193_01	Pk_ctg1_1559		Pk_Ga0417193_01_rn08	putative cation ABC transporter, periplasmic cation-binding protein	CAK15997.1	83.9	
Pseudomonas koreensis CI12	Pyoverdine-like siderophore - chromophore NRPS locus	Ga0417193_01	Pk_ctg1_1560		Pk_Ga0417193_01_rn08	conserved hypothetical protein	CAK15996.1	53.4	
Pseudomonas koreensis CI12	Pyoverdine-like siderophore - chromophore NRPS locus	Ga0417193_01	Pk_ctg1_1561		Pk_Ga0417193_01_rn08	conserved hypothetical protein, putative signal peptide	CAK15995.1	72.4	
Pseudomonas koreensis CI12	Pyoverdine-like siderophore - chromophore NRPS locus	Ga0417193_01	Pk_ctg1_1562		Pk_Ga0417193_01_rn08	conserved hypothetical protein	CAK15994.1	69	
Pseudomonas koreensis CI12	Pyoverdine-like siderophore - chromophore NRPS locus	Ga0417193_01	Pk_ctg1_1563		Pk_Ga0417193_01_rn08	conserved hypothetical protein, putative membrane protein	CAK15993.1	72.7	
Pseudomonas koreensis CI12	Lokisin	Ga0417193_01	Pk_ctg1_2148		Pk_Ga0417193_01_rn11				
Pseudomonas koreensis CI12	Lokisin	Ga0417193_01	Pk_ctg1_2149		Pk_Ga0417193_01_rn11				
Pseudomonas koreensis CI12	Lokisin	Ga0417193_01	Pk_ctg1_2150		Pk_Ga0417193_01_rn11				
Pseudomonas koreensis CI12	Lokisin	Ga0417193_01	Pk_ctg1_2151		Pk_Ga0417193_01_rn11				
Pseudomonas koreensis CI12	Lokisin	Ga0417193_01	Pk_ctg1_2152		Pk_Ga0417193_01_rn11				
Pseudomonas koreensis CI12	Lokisin	Ga0417193_01	Pk_ctg1_2153		Pk_Ga0417193_01_rn11				
Pseudomonas koreensis CI12	Lokisin	Ga0417193_01	Pk_ctg1_2154		Pk_Ga0417193_01_rn11				
Pseudomonas koreensis CI12	Lokisin	Ga0417193_01	Pk_ctg1_2155		Pk_Ga0417193_01_rn11				
Pseudomonas koreensis CI12	Lokisin	Ga0417193_01	Pk_ctg1_2156		Pk_Ga0417193_01_rn11				
Pseudomonas koreensis CI12	Lokisin	Ga0417193_01	Pk_ctg1_2157		Pk_Ga0417193_01_rn11				
Pseudomonas koreensis CI12	Lokisin	Ga0417193_01	Pk_ctg1_2158		Pk_Ga0417193_01_rn11				
Pseudomonas koreensis CI12	Lokisin	Ga0417193_01	Pk_ctg1_2159		Pk_Ga0417193_01_rn11				
Pseudomonas koreensis CI12	Lokisin	Ga0417193_01	Pk_ctg1_2160		Pk_Ga0417193_01_rn11	ABC transporter substrate-binding protein	WP_116029632.1	99.7	
Pseudomonas koreensis CI12	Lokisin	Ga0417193_01	Pk_ctg1_2161		Pk_Ga0417193_01_rn11	hypothetical protein	WP_123594335.1	99.4	
Pseudomonas koreensis CI12	Lokisin	Ga0417193_01	Pk_ctg1_2162		Pk_Ga0417193_01_rn11	copper chaperone Pcu(A)C	WP_077572416.1	100	
Pseudomonas koreensis CI12	Lokisin	Ga0417193_01	Pk_ctg1_2163		Pk_Ga0417193_01_rn11	SCO family protein	WP_008079652.1	100	
Pseudomonas koreensis CI12	Lokisin	Ga0417193_01	Pk_ctg1_2164	lokT	Pk_Ga0417193_01_rn11	RND efflux	QDF82261.1	86.5	
Pseudomonas koreensis CI12	Lokisin	Ga0417193_01	Pk_ctg1_2165	lokR	Pk_Ga0417193_01_rn11	luxR family regulator	QDF82260.1	88.3	
Pseudomonas koreensis CI12	Lokisin	Ga0417193_01	Pk_ctg1_2166		Pk_Ga0417193_01_rn11	hypothetical protein	WP_182404505.1		
Pseudomonas koreensis CI12	Lokisin	Ga0417193_01	Pk_ctg1_2167	lokA	Pk_Ga0417193_01_rn11	nrps	QDF82254.1	76.9	
Pseudomonas koreensis CI12	Lokisin	Ga0417193_01	Pk_ctg1_2168	lokB	Pk_Ga0417193_01_rn11	nrps	QDF82255.1	82.5	
Pseudomonas koreensis CI12	Lokisin	Ga0417193_01	Pk_ctg1_2169	lokC	Pk_Ga0417193_01_rn11	nrps	QDF82259.1	87.5	
Pseudomonas koreensis CI12	Lokisin	Ga0417193_01	Pk_ctg1_2170	macA	Pk_Ga0417193_01_rn11	macrolide-specific efflux protein	QDF82253.1	92.7	
Pseudomonas koreensis CI12	Lokisin	Ga0417193_01	Pk_ctg1_2171	macB	Pk_Ga0417193_01_rn11	macrolide export ATP-binding/permease protein	QDF82252.1	91.3	
Pseudomonas koreensis CI12	Lokisin	Ga0417193_01	Pk_ctg1_2172		Pk_Ga0417193_01_rn11				
Pseudomonas koreensis CI12	Lokisin	Ga0417193_01	Pk_ctg1_2173		Pk_Ga0417193_01_rn11				
Pseudomonas koreensis CI12	Lokisin	Ga0417193_01	Pk_ctg1_2174		Pk_Ga0417193_01_rn11				
Pseudomonas koreensis CI12	Lokisin	Ga0417193_01	Pk_ctg1_2175		Pk_Ga0417193_01_rn11				
Pseudomonas koreensis CI12	Lokisin	Ga0417193_01	Pk_ctg1_2176		Pk_Ga0417193_01_rn11				
Pseudomonas koreensis CI12	Lokisin	Ga0417193_01	Pk_ctg1_2177		Pk_Ga0417193_01_rn11				
Pseudomonas koreensis CI12	Lokisin	Ga0417193_01	Pk_ctg1_2178		Pk_Ga0417193_01_rn11				
Pseudomonas koreensis CI12	Lokisin	Ga0417193_01	Pk_ctg1_2179		Pk_Ga0417193_01_rn11				
Pseudomonas koreensis CI12	Lokisin	Ga0417193_01	Pk_ctg1_2180		Pk_Ga0417193_01_rn11				
Pseudomonas koreensis CI12	Lokisin	Ga0417193_01	Pk_ctg1_2181		Pk_Ga0417193_01_rn11				
Pseudomonas koreensis CI12	Lokisin	Ga0417193_01	Pk_ctg1_2182		Pk_Ga0417193_01_rn11				
Pseudomonas koreensis CI12	Lokisin	Ga0417193_01	Pk_ctg1_2183		Pk_Ga0417193_01_rn11				
Pseudomonas koreensis CI12	Pyoverdine-like siderophore - peptide backbone locus	Ga0417193_01	Pk_ctg1_3654	pvdA	Pk_Ga0417193_01_rn18				
Pseudomonas koreensis CI12	Pyoverdine-like siderophore - peptide backbone locus	Ga0417193_01	Pk_ctg1_3655		Pk_Ga0417193_01_rn18				
Pseudomonas koreensis CI12	Pyoverdine-like siderophore - peptide backbone locus	Ga0417193_01	Pk_ctg1_3656	RND efflux	Pk_Ga0417193_01_rn18				
Pseudomonas koreensis CI12	Pyoverdine-like siderophore - peptide backbone locus	Ga0417193_01	Pk_ctg1_3657	RND efflux	Pk_Ga0417193_01_rn18				
Pseudomonas koreensis CI12	Pyoverdine-like siderophore - peptide backbone locus	Ga0417193_01	Pk_ctg1_3658	RND efflux	Pk_Ga0417193_01_rn18				
Pseudomonas koreensis CI12	Pyoverdine-like siderophore - peptide backbone locus	Ga0417193_01	Pk_ctg1_3659	pvdP	Pk_Ga0417193_01_rn18				
Pseudomonas koreensis CI12	Pyoverdine-like siderophore - peptide backbone locus	Ga0417193_01	Pk_ctg1_3660		Pk_Ga0417193_01_rn18				
Pseudomonas koreensis CI12	Pyoverdine-like siderophore - peptide backbone locus	Ga0417193_01	Pk_ctg1_3661		Pk_Ga0417193_01_rn18				
Pseudomonas koreensis CI12	Pyoverdine-like siderophore - peptide backbone locus	Ga0417193_01	Pk_ctg1_3662		Pk_Ga0417193_01_rn18				
Pseudomonas koreensis CI12	Pyoverdine-like siderophore - peptide backbone locus	Ga0417193_01	Pk_ctg1_3663	pvdE	Pk_Ga0417193_01_rn18				
Pseudomonas koreensis CI12	Pyoverdine-like siderophore - peptide backbone locus	Ga0417193_01	Pk_ctg1_3664	fpvA	Pk_Ga0417193_01_rn18				
Pseudomonas koreensis CI12	Pyoverdine-like siderophore - peptide backbone locus	Ga0417193_01	Pk_ctg1_3665	lipase	Pk_Ga0417193_01_rn18				
Pseudomonas koreensis CI12	Pyoverdine-like siderophore - peptide backbone locus	Ga0417193_01	Pk_ctg1_3666	backbone NRPS	Pk_Ga0417193_01_rn18				
Pseudomonas koreensis CI12	Pyoverdine-like siderophore - peptide backbone locus	Ga0417193_01	Pk_ctg1_3667	backbone NRPS	Pk_Ga0417193_01_rn18				
Pseudomonas koreensis CI12	Pyoverdine-like siderophore - peptide backbone locus	Ga0417193_01	Pk_ctg1_3668	backbone NRPS	Pk_Ga0417193_01_rn18				
Pseudomonas koreensis CI12	Pyoverdine-like siderophore - peptide backbone locus	Ga0417193_01	Pk_ctg1_3669		Pk_Ga0417193_01_rn18				
Pseudomonas koreensis CI12	Pyoverdine-like siderophore - peptide backbone locus	Ga0417193_01	Pk_ctg1_3670		Pk_Ga0417193_01_rn18				
Pseudomonas koreensis CI12	Pyoverdine-like siderophore - peptide backbone locus	Ga0417193_01	Pk_ctg1_3671		Pk_Ga0417193_01_rn18				
Pseudomonas koreensis CI12	Pyoverdine-like siderophore - peptide backbone locus	Ga0417193_01	Pk_ctg1_3672		Pk_Ga0417193_01_rn18				
Pseudomonas koreensis CI12	Pyoverdine-like siderophore - peptide backbone locus	Ga0417193_01	Pk_ctg1_3673		Pk_Ga0417193_01_rn18				
Pseudomonas koreensis CI12	Pyoverdine-like siderophore - peptide backbone locus	Ga0417193_01	Pk_ctg1_3674		Pk_Ga0417193_01_rn18				

Pseudomonas koreensis CI12	Pyoverdine-like siderophore - peptide backbone locus	Ga0417193_01	Pk_ctg1_3675		Pk_Ga0417193_01_rn18				
Pseudomonas koreensis CI12	Pyoverdine-like siderophore - peptide backbone locus	Ga0417193_01	Pk_ctg1_3676		Pk_Ga0417193_01_rn18				
Pseudomonas koreensis CI12	Pyoverdine-like siderophore - peptide backbone locus	Ga0417193_01	Pk_ctg1_3677		Pk_Ga0417193_01_rn18				
Pseudomonas koreensis CI12	Pyoverdine-like siderophore - peptide backbone locus	Ga0417193_01	Pk_ctg1_3678		Pk_Ga0417193_01_rn18				
Pseudomonas koreensis CI12	Koreenceine	Ga0417193_01	Pk_ctg1_4873	kecA	Pk_Ga0417193_01_rn24				
Pseudomonas koreensis CI12	Koreenceine	Ga0417193_01	Pk_ctg1_4872	kecB	Pk_Ga0417193_01_rn24				
Pseudomonas koreensis CI12	Koreenceine	Ga0417193_01	Pk_ctg1_4871	kecC	Pk_Ga0417193_01_rn24				
Pseudomonas koreensis CI12	Koreenceine	Ga0417193_01	Pk_ctg1_4870	kecD	Pk_Ga0417193_01_rn24				
Pseudomonas koreensis CI12	Koreenceine	Ga0417193_01	Pk_ctg1_4869	kecE	Pk_Ga0417193_01_rn24				
Pseudomonas koreensis CI12	Koreenceine	Ga0417193_01	Pk_ctg1_4868	kecF	Pk_Ga0417193_01_rn24				
Pseudomonas koreensis CI12	Koreenceine	Ga0417193_01	Pk_ctg1_4867	kecG	Pk_Ga0417193_01_rn24				
Pseudomonas koreensis CI12	Koreenceine	Ga0417193_01	Pk_ctg1_4866	kecH	Pk_Ga0417193_01_rn24				
Pseudomonas koreensis CI12	Koreenceine	Ga0417193_01	Pk_ctg1_4865	kecI	Pk_Ga0417193_01_rn24				
Pseudomonas koreensis CI12	Koreenceine	Ga0417193_01	Pk_ctg1_4864	kecJ	Pk_Ga0417193_01_rn24				
Pseudomonas koreensis CI12	Koreenceine	Ga0417193_01	Pk_ctg1_4863	kecK	Pk_Ga0417193_01_rn24				
Pseudomonas koreensis CI12	Fragin-like	Ga0417193_01	Pk_ctg1_5433		Pk_Ga0417193_01_rn27				
Pseudomonas koreensis CI12	Fragin-like	Ga0417193_01	Pk_ctg1_5434		Pk_Ga0417193_01_rn27				
Pseudomonas koreensis CI12	Fragin-like	Ga0417193_01	Pk_ctg1_5435		Pk_Ga0417193_01_rn27				
Pseudomonas koreensis CI12	Fragin-like	Ga0417193_01	Pk_ctg1_5436		Pk_Ga0417193_01_rn27				
Pseudomonas koreensis CI12	Fragin-like	Ga0417193_01	Pk_ctg1_5437	hamA	Pk_Ga0417193_01_rn27				
Pseudomonas koreensis CI12	Fragin-like	Ga0417193_01	Pk_ctg1_5438	hamC	Pk_Ga0417193_01_rn27				
Pseudomonas koreensis CI12	Fragin-like	Ga0417193_01	Pk_ctg1_5439	hamD	Pk_Ga0417193_01_rn27				
Pseudomonas koreensis CI12	Fragin-like	Ga0417193_01	Pk_ctg1_5440	hamE	Pk_Ga0417193_01_rn27				
Pseudomonas koreensis CI12	Fragin-like	Ga0417193_01	Pk_ctg1_5441		Pk_Ga0417193_01_rn27				
Pseudomonas koreensis CI12	Fragin-like	Ga0417193_01	Pk_ctg1_5442		Pk_Ga0417193_01_rn27				
Pseudomonas koreensis CI12	Fragin-like	Ga0417193_01	Pk_ctg1_5443		Pk_Ga0417193_01_rn27				
Pseudomonas koreensis CI12	Fragin-like	Ga0417193_01	Pk_ctg1_5444		Pk_Ga0417193_01_rn27				
Pseudomonas koreensis CI12	Fragin-like	Ga0417193_01	Pk_ctg1_5445		Pk_Ga0417193_01_rn27				
Pseudomonas koreensis CI12	Fragin-like	Ga0417193_01	Pk_ctg1_5446		Pk_Ga0417193_01_rn27				
Pseudomonas koreensis CI12	Fragin-like	Ga0417193_01	Pk_ctg1_5447		Pk_Ga0417193_01_rn27				
Pseudomonas koreensis CI12	Fragin-like	Ga0417193_01	Pk_ctg1_5448		Pk_Ga0417193_01_rn27				
Pseudomonas koreensis CI12	Fragin-like	Ga0417193_01	Pk_ctg1_5449		Pk_Ga0417193_01_rn27				
Pseudomonas koreensis CI12	Fragin-like	Ga0417193_01	Pk_ctg1_5450		Pk_Ga0417193_01_rn27				
Pseudomonas koreensis CI12	Fragin-like	Ga0417193_01	Pk_ctg1_5451		Pk_Ga0417193_01_rn27				

The table following this page is:

Table S3

for manuscript:

Chevrette et al. "Microbiome composition modulates secondary metabolism in a multispecies bacterial community." *Proceedings of the National Academy of Sciences*. 2022.

All supplemental materials for this manuscript can be found at:

https://github.com/chevrm/thor_secmet/tree/main/figures/sup

Please refer to the cover sheet of this PDF for the exact file location on GitHub.

gene	sample	cpm	condition	cog_category	organism	is.q	bgc	gene_kind	smcog
Bc_ctg1_1	B1	18.55699732	Bcer	E	Bcer	FALSE	NA	NA	NA
Bc_ctg1_10	B1	52.19775715	Bcer	K	Bcer	FALSE	NA	NA	NA
Bc_ctg1_100	B1	7.938822381	Bcer	S	Bcer	FALSE	NA	NA	NA
Bc_ctg1_1002	B1	19.54935011	Bcer	S	Bcer	FALSE	Bc_Ga0417192_01_rn09	Other	NA
Bc_ctg1_1003	B1	13.19829221	Bcer	S	Bcer	FALSE	Bc_Ga0417192_01_rn09	Other	NA
Bc_ctg1_1004	B1	90.10563402	Bcer	NA	Bcer	FALSE	Bc_Ga0417192_01_rn09	Other	NA
Bc_ctg1_1005	B1	7.343410702	Bcer	S	Bcer	FALSE	Bc_Ga0417192_01_rn09	Other	NA
Bc_ctg1_1006	B1	31.8545248	Bcer	S	Bcer	FALSE	Bc_Ga0417192_01_rn09	Other	NA
Bc_ctg1_1007	B1	348.9112436	Bcer	I	Bcer	FALSE	Bc_Ga0417192_01_rn09	Ancillary biosynthesis	SMCOG1124
Bc_ctg1_1008	B1	84.3499878	Bcer	F	Bcer	FALSE	Bc_Ga0417192_01_rn09	Other	NA
Bc_ctg1_1009	B1	293.1410164	Bcer	S	Bcer	FALSE	Bc_Ga0417192_01_rn09	Other	NA
Bc_ctg1_101	B1	7.045704863	Bcer	S	Bcer	FALSE	NA	NA	NA
Bc_ctg1_1010	B1	0.09923528	Bcer	NA	Bcer	FALSE	NA	NA	NA
Bc_ctg1_1011	B1	11.61052773	Bcer	NA	Bcer	FALSE	NA	NA	NA
Bc_ctg1_1012	B1	13.79370389	Bcer	NA	Bcer	FALSE	NA	NA	NA
Bc_ctg1_1013	B1	2.083940875	Bcer	K	Bcer	FALSE	NA	NA	NA
Bc_ctg1_1014	B1	5.557175667	Bcer	IQ	Bcer	TRUE	NA	NA	NA
Bc_ctg1_1017	B1	0	Bcer	S	Bcer	FALSE	NA	NA	NA
Bc_ctg1_1018	B1	9.824292696	Bcer	E	Bcer	FALSE	NA	NA	NA
Bc_ctg1_1019	B1	14.78605668	Bcer	F	Bcer	FALSE	NA	NA	NA
Bc_ctg1_102	B1	0.09923528	Bcer	L	Bcer	FALSE	NA	NA	NA
Bc_ctg1_1020	B1	14.09140973	Bcer	S	Bcer	FALSE	NA	NA	NA
Bc_ctg1_1021	B1	52.29699243	Bcer	E	Bcer	FALSE	NA	NA	NA
Bc_ctg1_1022	B1	23.22105546	Bcer	G	Bcer	FALSE	NA	NA	NA
Bc_ctg1_1023	B1	8.732704619	Bcer	E	Bcer	FALSE	NA	NA	NA
Bc_ctg1_1024	B1	20.64093819	Bcer	E	Bcer	FALSE	NA	NA	NA
Bc_ctg1_1025	B1	21.73252627	Bcer	S	Bcer	FALSE	NA	NA	NA
Bc_ctg1_1026	B1	0.992352798	Bcer	NA	Bcer	FALSE	NA	NA	NA
Bc_ctg1_1027	B1	14.48835085	Bcer	S	Bcer	FALSE	NA	NA	NA
Bc_ctg1_1028	B1	98.44139752	Bcer	I	Bcer	FALSE	NA	NA	NA
Bc_ctg1_1029	B1	91.29645738	Bcer	V	Bcer	FALSE	NA	NA	NA
Bc_ctg1_103	B1	0	Bcer	L	Bcer	FALSE	NA	NA	NA
Bc_ctg1_1030	B1	3.175528952	Bcer	T	Bcer	FALSE	NA	NA	NA
Bc_ctg1_1031	B1	40.88493526	Bcer	C	Bcer	FALSE	NA	NA	NA
Bc_ctg1_1032	B1	41.77805278	Bcer	NA	Bcer	FALSE	NA	NA	NA
Bc_ctg1_1033	B1	8.23652822	Bcer	S	Bcer	FALSE	NA	NA	NA
Bc_ctg1_1034	B1	2453.294586	Bcer	G	Bcer	FALSE	NA	NA	NA
Bc_ctg1_1035	B1	334.3236575	Bcer	G	Bcer	FALSE	NA	NA	NA
Bc_ctg1_1036	B1	1315.363633	Bcer	G	Bcer	FALSE	NA	NA	NA
Bc_ctg1_1037	B1	95.06739801	Bcer	K	Bcer	FALSE	NA	NA	NA
Bc_ctg1_1038	B1	223.2793795	Bcer	S	Bcer	FALSE	NA	NA	NA
Bc_ctg1_1039	B1	1184.273829	Bcer	K	Bcer	FALSE	NA	NA	NA
Bc_ctg1_104	B1	4.664058149	Bcer	S	Bcer	FALSE	NA	NA	NA
Bc_ctg1_1040	B1	1109.847369	Bcer	G	Bcer	FALSE	NA	NA	NA
Bc_ctg1_1041	B1	142.601097	Bcer	G	Bcer	FALSE	NA	NA	NA
Bc_ctg1_1042	B1	2.778587833	Bcer	S	Bcer	FALSE	NA	NA	NA
Bc_ctg1_1043	B1	10.12199854	Bcer	D	Bcer	FALSE	NA	NA	NA
Bc_ctg1_1044	B1	124.9372172	Bcer	D	Bcer	FALSE	NA	NA	NA
Bc_ctg1_1046	B1	23.22105546	Bcer	S	Bcer	FALSE	NA	NA	NA
Bc_ctg1_1047	B1	282.6220768	Bcer	K	Bcer	FALSE	NA	NA	NA
Bc_ctg1_1048	B1	1067.57314	Bcer	O	Bcer	FALSE	NA	NA	NA
Bc_ctg1_1049	B1	325.987894	Bcer	S	Bcer	FALSE	NA	NA	NA
Bc_ctg1_105	B1	0.694646958	Bcer	NA	Bcer	FALSE	NA	NA	NA
Bc_ctg1_1050	B1	27.38893721	Bcer	S	Bcer	FALSE	NA	NA	NA
Bc_ctg1_1051	B1	3.870175911	Bcer	EG	Bcer	FALSE	NA	NA	NA
Bc_ctg1_1052	B1	0	Bcer	S	Bcer	FALSE	NA	NA	NA
Bc_ctg1_1053	B1	0	Bcer	S	Bcer	FALSE	NA	NA	NA
Bc_ctg1_1054	B1	0.09923528	Bcer	IQ	Bcer	TRUE	NA	NA	NA
Bc_ctg1_1055	B1	0	Bcer	S	Bcer	FALSE	NA	NA	NA
Bc_ctg1_1057	B1	0	Bcer	S	Bcer	FALSE	NA	NA	NA
Bc_ctg1_1059	B1	0.496176399	Bcer	S	Bcer	FALSE	NA	NA	NA

Bc_ctg1_106	B1	0.19847056	Bcer	K	Bcer	FALSE	NA	NA	NA
Bc_ctg1_1060	B1	60.73199121	Bcer	K	Bcer	FALSE	NA	NA	NA
Bc_ctg1_1061	B1	28.18281945	Bcer	F	Bcer	FALSE	NA	NA	NA
Bc_ctg1_1062	B1	22.42717323	Bcer	T	Bcer	FALSE	NA	NA	NA
Bc_ctg1_1063	B1	0.09923528	Bcer	M	Bcer	FALSE	NA	NA	NA
Bc_ctg1_1064	B1	45.15205229	Bcer	K	Bcer	FALSE	NA	NA	NA
Bc_ctg1_1065	B1	120.0746885	Bcer	V	Bcer	FALSE	NA	NA	NA
Bc_ctg1_1066	B1	147.4636257	Bcer	S	Bcer	FALSE	NA	NA	NA
Bc_ctg1_1067	B1	3.870175911	Bcer	D	Bcer	FALSE	NA	NA	NA
Bc_ctg1_1068	B1	263.9658442	Bcer	K	Bcer	FALSE	NA	NA	NA
Bc_ctg1_1069	B1	1.091588077	Bcer	S	Bcer	FALSE	NA	NA	NA
Bc_ctg1_107	B1	10.3204691	Bcer	GIM	Bcer	FALSE	NA	NA	NA
Bc_ctg1_1070	B1	122.3570999	Bcer	S	Bcer	FALSE	NA	NA	NA
Bc_ctg1_1071	B1	114.7159834	Bcer	P	Bcer	FALSE	NA	NA	NA
Bc_ctg1_1072	B1	2122.543399	Bcer	F	Bcer	FALSE	NA	NA	NA
Bc_ctg1_1073	B1	1461.735671	Bcer	F	Bcer	FALSE	NA	NA	NA
Bc_ctg1_1074	B1	2585.178273	Bcer	G	Bcer	FALSE	NA	NA	NA
Bc_ctg1_1075	B1	104.1970437	Bcer	S	Bcer	FALSE	NA	NA	NA
Bc_ctg1_1076	B1	64.89987296	Bcer	L	Bcer	FALSE	NA	NA	NA
Bc_ctg1_1077	B1	0.793882238	Bcer	S	Bcer	FALSE	NA	NA	NA
Bc_ctg1_1078	B1	578.4424457	Bcer	P	Bcer	FALSE	NA	NA	NA
Bc_ctg1_1079	B1	19.84705595	Bcer	S	Bcer	FALSE	NA	NA	NA
Bc_ctg1_108	B1	53.58705107	Bcer	K	Bcer	FALSE	NA	NA	NA
Bc_ctg1_1080	B1	311.4003079	Bcer	J	Bcer	FALSE	NA	NA	NA
Bc_ctg1_1081	B1	120.3723944	Bcer	L	Bcer	FALSE	NA	NA	NA
Bc_ctg1_1083	B1	77.60198877	Bcer	C	Bcer	FALSE	NA	NA	NA
Bc_ctg1_1084	B1	21.93099683	Bcer	S	Bcer	FALSE	NA	NA	NA
Bc_ctg1_1085	B1	7.442645982	Bcer	K	Bcer	FALSE	NA	NA	NA
Bc_ctg1_1086	B1	0.595411679	Bcer	S	Bcer	FALSE	NA	NA	NA
Bc_ctg1_1087	B1	110.8458075	Bcer	F	Bcer	FALSE	NA	NA	NA
Bc_ctg1_1088	B1	1.786235036	Bcer	S	Bcer	FALSE	NA	NA	NA
Bc_ctg1_1089	B1	18.85470315	Bcer	I	Bcer	FALSE	NA	NA	NA
Bc_ctg1_109	B1	5.060999268	Bcer	IQ	Bcer	TRUE	NA	NA	NA
Bc_ctg1_1090	B1	34.53387736	Bcer	S	Bcer	FALSE	NA	NA	NA
Bc_ctg1_1091	B1	32.44993648	Bcer	K	Bcer	FALSE	NA	NA	NA
Bc_ctg1_1092	B1	4.16788175	Bcer	K	Bcer	FALSE	NA	NA	NA
Bc_ctg1_1093	B1	162.2496824	Bcer	S	Bcer	FALSE	NA	NA	NA
Bc_ctg1_1094	B1	15.48070364	Bcer	S	Bcer	FALSE	NA	NA	NA
Bc_ctg1_1095	B1	42.77040558	Bcer	S	Bcer	FALSE	NA	NA	NA
Bc_ctg1_1096	B1	305.9423675	Bcer	H	Bcer	FALSE	NA	NA	NA
Bc_ctg1_1097	B1	262.477315	Bcer	H	Bcer	FALSE	NA	NA	NA
Bc_ctg1_1098	B1	553.8320963	Bcer	H	Bcer	FALSE	NA	NA	NA
Bc_ctg1_1099	B1	282.3243709	Bcer	H	Bcer	FALSE	NA	NA	NA
Bc_ctg1_11	B1	0.496176399	Bcer	S	Bcer	FALSE	NA	NA	NA
Bc_ctg1_110	B1	2.778587833	Bcer	K	Bcer	FALSE	NA	NA	NA
Bc_ctg1_1100	B1	0	Bcer	NA	Bcer	FALSE	NA	NA	NA
Bc_ctg1_1101	B1	4.366352309	Bcer	H	Bcer	FALSE	NA	NA	NA
Bc_ctg1_1102	B1	1.786235036	Bcer	H	Bcer	FALSE	NA	NA	NA
Bc_ctg1_1103	B1	0.992352798	Bcer	S	Bcer	FALSE	NA	NA	NA
Bc_ctg1_1104	B1	1.786235036	Bcer	H	Bcer	FALSE	NA	NA	NA
Bc_ctg1_1105	B1	0.992352798	Bcer	H	Bcer	FALSE	NA	NA	NA
Bc_ctg1_1106	B1	0.793882238	Bcer	H	Bcer	FALSE	NA	NA	NA
Bc_ctg1_1107	B1	0.09923528	Bcer	NA	Bcer	FALSE	NA	NA	NA
Bc_ctg1_1108	B1	19.05317371	Bcer	C	Bcer	FALSE	NA	NA	NA
Bc_ctg1_1109	B1	162.6466235	Bcer	F	Bcer	FALSE	NA	NA	NA
Bc_ctg1_111	B1	4.16788175	Bcer	S	Bcer	FALSE	NA	NA	NA
Bc_ctg1_1110	B1	21.73252627	Bcer	E	Bcer	FALSE	NA	NA	NA
Bc_ctg1_1111	B1	182.4936795	Bcer	K	Bcer	FALSE	NA	NA	NA
Bc_ctg1_1112	B1	4.465587589	Bcer	NA	Bcer	FALSE	NA	NA	NA
Bc_ctg1_1113	B1	43.66352309	Bcer	S	Bcer	FALSE	NA	NA	NA
Bc_ctg1_1114	B1	11.01511605	Bcer	E	Bcer	FALSE	NA	NA	NA
Bc_ctg1_1115	B1	8.732704619	Bcer	E	Bcer	FALSE	NA	NA	NA

Bc_ctg1_1116	B1	1.389293917	Bcer	E	Bcer	FALSE	NA	NA	NA
Bc_ctg1_1117	B1	1.290058637	Bcer	E	Bcer	FALSE	NA	NA	NA
Bc_ctg1_1118	B1	1.686999756	Bcer	E	Bcer	FALSE	NA	NA	NA
Bc_ctg1_1119	B1	0.396941119	Bcer	NA	Bcer	FALSE	NA	NA	NA
Bc_ctg1_112	B1	21.83176155	Bcer	L	Bcer	FALSE	Bc_Ga0417192_01_rn01	Other	NA
Bc_ctg1_1120	B1	132.3798632	Bcer	S	Bcer	FALSE	NA	NA	NA
Bc_ctg1_1121	B1	53.28934523	Bcer	S	Bcer	FALSE	NA	NA	NA
Bc_ctg1_1122	B1	2.183176155	Bcer	E	Bcer	FALSE	NA	NA	NA
Bc_ctg1_1123	B1	127.4180992	Bcer	E	Bcer	FALSE	NA	NA	NA
Bc_ctg1_1124	B1	75.31957734	Bcer	E	Bcer	FALSE	NA	NA	NA
Bc_ctg1_1125	B1	56.3656389	Bcer	S	Bcer	FALSE	NA	NA	NA
Bc_ctg1_1126	B1	111.8381603	Bcer	NA	Bcer	FALSE	NA	NA	NA
Bc_ctg1_1127	B1	1.885470315	Bcer	NA	Bcer	FALSE	NA	NA	NA
Bc_ctg1_1128	B1	51.40387492	Bcer	P	Bcer	FALSE	NA	NA	NA
Bc_ctg1_1129	B1	7.045704863	Bcer	S	Bcer	FALSE	NA	NA	NA
Bc_ctg1_113	B1	6.946469583	Bcer	E	Bcer	FALSE	Bc_Ga0417192_01_rn01	Other	NA
Bc_ctg1_1130	B1	12.70211581	Bcer	L	Bcer	FALSE	NA	NA	NA
Bc_ctg1_1132	B1	1654.74829	Bcer	E	Bcer	FALSE	NA	NA	NA
Bc_ctg1_1133	B1	150.043743	Bcer	J	Bcer	FALSE	NA	NA	NA
Bc_ctg1_1134	B1	26.29734914	Bcer	NA	Bcer	FALSE	NA	NA	NA
Bc_ctg1_1135	B1	13.19829221	Bcer	S	Bcer	FALSE	NA	NA	NA
Bc_ctg1_1136	B1	41.97652334	Bcer	S	Bcer	FALSE	NA	NA	NA
Bc_ctg1_1137	B1	98.24292696	Bcer	E	Bcer	FALSE	NA	NA	NA
Bc_ctg1_1138	B1	62.12128513	Bcer	E	Bcer	FALSE	NA	NA	NA
Bc_ctg1_1139	B1	55.47252139	Bcer	ET	Bcer	FALSE	NA	NA	NA
Bc_ctg1_114	B1	0.694646958	Bcer	NA	Bcer	FALSE	Bc_Ga0417192_01_rn01	Other	NA
Bc_ctg1_1140	B1	57.8541681	Bcer	S	Bcer	FALSE	NA	NA	NA
Bc_ctg1_1141	B1	30.96140729	Bcer	S	Bcer	FALSE	NA	NA	NA
Bc_ctg1_1142	B1	23.0225849	Bcer	F	Bcer	FALSE	NA	NA	NA
Bc_ctg1_1143	B1	0.793882238	Bcer	S	Bcer	FALSE	NA	NA	NA
Bc_ctg1_1144	B1	273.2939605	Bcer	D	Bcer	FALSE	NA	NA	NA
Bc_ctg1_1145	B1	1476.422492	Bcer	C	Bcer	FALSE	NA	NA	NA
Bc_ctg1_1146	B1	514.0387492	Bcer	C	Bcer	FALSE	NA	NA	NA
Bc_ctg1_1147	B1	550.3588616	Bcer	C	Bcer	FALSE	NA	NA	NA
Bc_ctg1_1148	B1	840.8205254	Bcer	C	Bcer	FALSE	NA	NA	NA
Bc_ctg1_1149	B1	558.8930956	Bcer	C	Bcer	FALSE	NA	NA	NA
Bc_ctg1_1150	B1	1837.936616	Bcer	E	Bcer	FALSE	NA	NA	NA
Bc_ctg1_1151	B1	1074.420374	Bcer	C	Bcer	FALSE	NA	NA	NA
Bc_ctg1_1152	B1	40.6864647	Bcer	KT	Bcer	FALSE	NA	NA	NA
Bc_ctg1_1153	B1	3.96941119	Bcer	S	Bcer	FALSE	NA	NA	NA
Bc_ctg1_1154	B1	38.20558271	Bcer	C	Bcer	FALSE	NA	NA	NA
Bc_ctg1_1155	B1	1.686999756	Bcer	K	Bcer	FALSE	NA	NA	NA
Bc_ctg1_1156	B1	4.366352309	Bcer	NA	Bcer	FALSE	NA	NA	NA
Bc_ctg1_1157	B1	488.0391059	Bcer	KT	Bcer	FALSE	NA	NA	NA
Bc_ctg1_1158	B1	7.641116542	Bcer	M	Bcer	FALSE	NA	NA	NA
Bc_ctg1_1159	B1	85.44157587	Bcer	L	Bcer	FALSE	NA	NA	NA
Bc_ctg1_116	B1	4.564822869	Bcer	S	Bcer	FALSE	Bc_Ga0417192_01_rn01	Other	NA
Bc_ctg1_1160	B1	271.8054313	Bcer	K	Bcer	FALSE	NA	NA	NA
Bc_ctg1_1161	B1	125.5326289	Bcer	J	Bcer	FALSE	NA	NA	NA
Bc_ctg1_1162	B1	341.6670682	Bcer	H	Bcer	FALSE	NA	NA	NA
Bc_ctg1_1163	B1	204.5239116	Bcer	H	Bcer	FALSE	NA	NA	NA
Bc_ctg1_1164	B1	37.01475935	Bcer	L	Bcer	FALSE	NA	NA	NA
Bc_ctg1_1165	B1	402.9944711	Bcer	L	Bcer	FALSE	NA	NA	NA
Bc_ctg1_1166	B1	317.0567188	Bcer	F	Bcer	FALSE	NA	NA	NA
Bc_ctg1_1167	B1	46.83905205	Bcer	K	Bcer	FALSE	NA	NA	NA
Bc_ctg1_1168	B1	1014.085324	Bcer	S	Bcer	FALSE	NA	NA	NA
Bc_ctg1_1169	B1	1644.725527	Bcer	I	Bcer	FALSE	NA	NA	NA
Bc_ctg1_117	B1	2.480881994	Bcer	NA	Bcer	FALSE	Bc_Ga0417192_01_rn01	Other	NA
Bc_ctg1_1170	B1	243.324906	Bcer	I	Bcer	FALSE	NA	NA	NA
Bc_ctg1_1171	B1	0.297705839	Bcer	NA	Bcer	FALSE	NA	NA	NA
Bc_ctg1_1172	B1	0	Bcer	S	Bcer	FALSE	NA	NA	NA
Bc_ctg1_1173	B1	0.595411679	Bcer	S	Bcer	FALSE	NA	NA	NA

Bc_ctg1_1174	B1	0.297705839	Bcer	S	Bcer	FALSE	NA	NA	NA
Bc_ctg1_1175	B1	0.496176399	Bcer	S	Bcer	FALSE	NA	NA	NA
Bc_ctg1_1176	B1	0	Bcer	S	Bcer	FALSE	NA	NA	NA
Bc_ctg1_1177	B1	0	Bcer	S	Bcer	FALSE	NA	NA	NA
Bc_ctg1_1178	B1	0	Bcer	S	Bcer	FALSE	NA	NA	NA
Bc_ctg1_1179	B1	0.793882238	Bcer	S	Bcer	FALSE	NA	NA	NA
Bc_ctg1_118	B1	14.78605668	Bcer	EGP	Bcer	FALSE	Bc_Ga0417192_01_rn01	Transport	SMCOG1106
Bc_ctg1_1180	B1	0	Bcer	S	Bcer	FALSE	NA	NA	NA
Bc_ctg1_1181	B1	3.96941119	Bcer	J	Bcer	FALSE	NA	NA	NA
Bc_ctg1_1182	B1	2.282411435	Bcer	NA	Bcer	FALSE	NA	NA	NA
Bc_ctg1_1183	B1	876.3467556	Bcer	J	Bcer	FALSE	NA	NA	NA
Bc_ctg1_1184	B1	472.8561081	Bcer	E	Bcer	FALSE	NA	NA	NA
Bc_ctg1_1185	B1	29.86981921	Bcer	E	Bcer	FALSE	NA	NA	NA
Bc_ctg1_1186	B1	1.190823357	Bcer	S	Bcer	FALSE	NA	NA	NA
Bc_ctg1_1187	B1	91.89186906	Bcer	S	Bcer	FALSE	NA	NA	NA
Bc_ctg1_1188	B1	58.3503445	Bcer	NA	Bcer	FALSE	NA	NA	NA
Bc_ctg1_1189	B1	27.09123137	Bcer	S	Bcer	FALSE	NA	NA	NA
Bc_ctg1_119	B1	5.358705107	Bcer	K	Bcer	FALSE	Bc_Ga0417192_01_rn01	Other	NA
Bc_ctg1_1190	B1	35.92317127	Bcer	K	Bcer	FALSE	NA	NA	NA
Bc_ctg1_1191	B1	0.694646958	Bcer	L	Bcer	FALSE	NA	NA	NA
Bc_ctg1_1192	B1	2.480881994	Bcer	K	Bcer	FALSE	NA	NA	NA
Bc_ctg1_1193	B1	7.045704863	Bcer	S	Bcer	FALSE	NA	NA	NA
Bc_ctg1_1194	B1	12.80135109	Bcer	NU	Bcer	FALSE	NA	NA	NA
Bc_ctg1_1195	B1	75.81575374	Bcer	H	Bcer	FALSE	NA	NA	NA
Bc_ctg1_1196	B1	71.44940143	Bcer	P	Bcer	FALSE	NA	NA	NA
Bc_ctg1_1197	B1	0.396941119	Bcer	NA	Bcer	FALSE	NA	NA	NA
Bc_ctg1_1198	B1	27.68664305	Bcer	K	Bcer	FALSE	NA	NA	NA
Bc_ctg1_1199	B1	30.26676033	Bcer	K	Bcer	FALSE	NA	NA	NA
Bc_ctg1_120	B1	15.97688004	Bcer	K	Bcer	FALSE	Bc_Ga0417192_01_rn01	Other	NA
Bc_ctg1_1200	B1	1.786235036	Bcer	P	Bcer	FALSE	NA	NA	NA
Bc_ctg1_1201	B1	18.75546787	Bcer	P	Bcer	FALSE	NA	NA	NA
Bc_ctg1_1202	B1	3.671705351	Bcer	NA	Bcer	FALSE	NA	NA	NA
Bc_ctg1_1203	B1	0	Bcer	NA	Bcer	FALSE	NA	NA	NA
Bc_ctg1_1204	B1	3.671705351	Bcer	S	Bcer	FALSE	NA	NA	NA
Bc_ctg1_1205	B1	1.587764476	Bcer	S	Bcer	FALSE	NA	NA	NA
Bc_ctg1_1206	B1	2.679352554	Bcer	S	Bcer	FALSE	NA	NA	NA
Bc_ctg1_1207	B1	7.244175423	Bcer	S	Bcer	FALSE	NA	NA	NA
Bc_ctg1_1208	B1	4.564822869	Bcer	V	Bcer	FALSE	NA	NA	NA
Bc_ctg1_1209	B1	0.297705839	Bcer	K	Bcer	FALSE	NA	NA	NA
Bc_ctg1_121	B1	25.40423162	Bcer	C	Bcer	FALSE	Bc_Ga0417192_01_rn01	Ancillary biosynthesis	SMCOG1007
Bc_ctg1_1210	B1	2.183176155	Bcer	NA	Bcer	FALSE	NA	NA	NA
Bc_ctg1_1211	B1	0.396941119	Bcer	S	Bcer	FALSE	NA	NA	NA
Bc_ctg1_1212	B1	2428.386531	Bcer	E	Bcer	FALSE	NA	NA	NA
Bc_ctg1_1213	B1	2735.618957	Bcer	E	Bcer	FALSE	NA	NA	NA
Bc_ctg1_1214	B1	1224.067176	Bcer	E	Bcer	FALSE	NA	NA	NA
Bc_ctg1_1215	B1	3.274764232	Bcer	L	Bcer	FALSE	NA	NA	NA
Bc_ctg1_1216	B1	0.694646958	Bcer	S	Bcer	FALSE	NA	NA	NA
Bc_ctg1_1218	B1	2.183176155	Bcer	NA	Bcer	FALSE	NA	NA	NA
Bc_ctg1_1219	B1	27.98434889	Bcer	NA	Bcer	FALSE	NA	NA	NA
Bc_ctg1_122	B1	15.6791742	Bcer	S	Bcer	FALSE	Bc_Ga0417192_01_rn01	Core biosynthesis	None
Bc_ctg1_1220	B1	0.595411679	Bcer	S	Bcer	FALSE	NA	NA	NA
Bc_ctg1_1221	B1	7.938822381	Bcer	F	Bcer	FALSE	NA	NA	NA
Bc_ctg1_1222	B1	89.51022234	Bcer	O	Bcer	FALSE	NA	NA	NA
Bc_ctg1_1223	B1	0.793882238	Bcer	S	Bcer	FALSE	Bc_Ga0417192_01_rn10	Other	NA
Bc_ctg1_1224	B1	0	Bcer	U	Bcer	FALSE	Bc_Ga0417192_01_rn10	Other	NA
Bc_ctg1_1225	B1	0.09923528	Bcer	NA	Bcer	FALSE	Bc_Ga0417192_01_rn10	Other	NA
Bc_ctg1_1226	B1	0.893117518	Bcer	NU	Bcer	FALSE	Bc_Ga0417192_01_rn10	Other	NA
Bc_ctg1_1227	B1	0	Bcer	U	Bcer	FALSE	Bc_Ga0417192_01_rn10	Other	NA
Bc_ctg1_1228	B1	2.282411435	Bcer	NU	Bcer	FALSE	Bc_Ga0417192_01_rn10	Other	NA
Bc_ctg1_1229	B1	1.885470315	Bcer	NU	Bcer	FALSE	Bc_Ga0417192_01_rn10	Other	NA
Bc_ctg1_123	B1	23.0225849	Bcer	NT	Bcer	FALSE	Bc_Ga0417192_01_rn01	Other	SMCOG1061
Bc_ctg1_1230	B1	432.1696434	Bcer	K	Bcer	FALSE	Bc_Ga0417192_01_rn10	Other	NA

Bc_ctg1_1231	B1	7.045704863	Bcer	S	Bcer	FALSE	Bc_Ga0417192_01_rn10	Other	NA
Bc_ctg1_1232	B1	92.28881018	Bcer	U	Bcer	FALSE	Bc_Ga0417192_01_rn10	Transport	SMCOG1212
Bc_ctg1_1233	B1	0.893117518	Bcer	NA	Bcer	FALSE	Bc_Ga0417192_01_rn10	Other	NA
Bc_ctg1_1234	B1	1.190823357	Bcer	S	Bcer	FALSE	Bc_Ga0417192_01_rn10	Other	NA
Bc_ctg1_1235	B1	85.73928171	Bcer	NA	Bcer	FALSE	Bc_Ga0417192_01_rn10	Other	NA
Bc_ctg1_1236	B1	38.90022967	Bcer	NA	Bcer	FALSE	Bc_Ga0417192_01_rn10	Other	NA
Bc_ctg1_1237	B1	15.6791742	Bcer	NA	Bcer	FALSE	Bc_Ga0417192_01_rn10	Other	NA
Bc_ctg1_1238	B1	20.64093819	Bcer	S	Bcer	FALSE	Bc_Ga0417192_01_rn10	Other	NA
Bc_ctg1_1239	B1	80.47981189	Bcer	M	Bcer	FALSE	Bc_Ga0417192_01_rn10	Core biosynthesis	None
Bc_ctg1_124	B1	0.09923528	Bcer	NA	Bcer	FALSE	Bc_Ga0417192_01_rn01	Other	NA
Bc_ctg1_1240	B1	39.6941119	Bcer	E	Bcer	FALSE	Bc_Ga0417192_01_rn10	Other	NA
Bc_ctg1_1241	B1	11.90823357	Bcer	E	Bcer	FALSE	Bc_Ga0417192_01_rn10	Other	NA
Bc_ctg1_1242	B1	4.366352309	Bcer	E	Bcer	FALSE	Bc_Ga0417192_01_rn10	Core biosynthesis	SMCOG1168
Bc_ctg1_1243	B1	5.060999268	Bcer	E	Bcer	FALSE	Bc_Ga0417192_01_rn10	Ancillary biosynthesis	SMCOG1168
Bc_ctg1_1244	B1	10.91588077	Bcer	Q	Bcer	TRUE	Bc_Ga0417192_01_rn10	Other	NA
Bc_ctg1_1245	B1	169.6923284	Bcer	K	Bcer	FALSE	Bc_Ga0417192_01_rn10	Regulation	SMCOG1058
Bc_ctg1_1246	B1	102.4108087	Bcer	S	Bcer	FALSE	Bc_Ga0417192_01_rn10	Other	NA
Bc_ctg1_1247	B1	58.94575618	Bcer	S	Bcer	FALSE	Bc_Ga0417192_01_rn10	Ancillary biosynthesis	SMCOG1170
Bc_ctg1_1248	B1	15.77840948	Bcer	S	Bcer	FALSE	Bc_Ga0417192_01_rn10	Other	NA
Bc_ctg1_1249	B1	224.2717323	Bcer	G	Bcer	FALSE	Bc_Ga0417192_01_rn10	Other	SMCOG1108
Bc_ctg1_125	B1	0.09923528	Bcer	L	Bcer	FALSE	Bc_Ga0417192_01_rn01	Other	NA
Bc_ctg1_1250	B1	18.75546787	Bcer	S	Bcer	FALSE	Bc_Ga0417192_01_rn10	Other	NA
Bc_ctg1_1251	B1	60.63275593	Bcer	H	Bcer	FALSE	Bc_Ga0417192_01_rn10	Other	NA
Bc_ctg1_1252	B1	88.51786955	Bcer	J	Bcer	FALSE	Bc_Ga0417192_01_rn10	Other	NA
Bc_ctg1_1253	B1	16.47305644	Bcer	GJM	Bcer	FALSE	Bc_Ga0417192_01_rn10	Ancillary biosynthesis	SMCOG1064
Bc_ctg1_1254	B1	1.786235036	Bcer	P	Bcer	FALSE	NA	NA	NA
Bc_ctg1_1255	B1	3.175528952	Bcer	P	Bcer	FALSE	NA	NA	NA
Bc_ctg1_1256	B1	1.488529196	Bcer	P	Bcer	FALSE	NA	NA	NA
Bc_ctg1_1257	B1	2.083940875	Bcer	P	Bcer	FALSE	NA	NA	NA
Bc_ctg1_1258	B1	1.190823357	Bcer	P	Bcer	FALSE	NA	NA	NA
Bc_ctg1_1259	B1	607.4191474	Bcer	M	Bcer	FALSE	NA	NA	NA
Bc_ctg1_126	B1	6.747999024	Bcer	V	Bcer	FALSE	Bc_Ga0417192_01_rn01	Ancillary biosynthesis	SMCOG1053
Bc_ctg1_1260	B1	4.961763988	Bcer	EGP	Bcer	FALSE	NA	NA	NA
Bc_ctg1_1261	B1	303.1637797	Bcer	P	Bcer	FALSE	NA	NA	NA
Bc_ctg1_1262	B1	211.768087	Bcer	S	Bcer	FALSE	NA	NA	NA
Bc_ctg1_1263	B1	0.694646958	Bcer	M	Bcer	FALSE	NA	NA	NA
Bc_ctg1_1264	B1	337.1022453	Bcer	I	Bcer	FALSE	NA	NA	NA
Bc_ctg1_1265	B1	19.15240899	Bcer	P	Bcer	FALSE	NA	NA	NA
Bc_ctg1_1266	B1	64.80063768	Bcer	P	Bcer	FALSE	NA	NA	NA
Bc_ctg1_1267	B1	63.31210849	Bcer	P	Bcer	FALSE	NA	NA	NA
Bc_ctg1_1268	B1	173.1655632	Bcer	S	Bcer	FALSE	NA	NA	NA
Bc_ctg1_127	B1	7.938822381	Bcer	V	Bcer	FALSE	Bc_Ga0417192_01_rn01	Ancillary biosynthesis	SMCOG1053
Bc_ctg1_1270	B1	68.1746372	Bcer	L	Bcer	FALSE	NA	NA	NA
Bc_ctg1_1271	B1	373.2238872	Bcer	JKL	Bcer	FALSE	NA	NA	NA
Bc_ctg1_1272	B1	0	Bcer	NA	Bcer	FALSE	NA	NA	NA
Bc_ctg1_1273	B1	180.7074444	Bcer	IM	Bcer	FALSE	NA	NA	NA
Bc_ctg1_1274	B1	66.48763744	Bcer	S	Bcer	FALSE	NA	NA	NA
Bc_ctg1_1275	B1	15.38146836	Bcer	S	Bcer	FALSE	NA	NA	NA
Bc_ctg1_1276	B1	22.12946739	Bcer	C	Bcer	FALSE	NA	NA	NA
Bc_ctg1_1277	B1	504.0159859	Bcer	K	Bcer	FALSE	NA	NA	NA
Bc_ctg1_1278	B1	1110.740486	Bcer	L	Bcer	FALSE	NA	NA	NA
Bc_ctg1_1279	B1	413.5134108	Bcer	S	Bcer	FALSE	NA	NA	NA
Bc_ctg1_128	B1	1.389293917	Bcer	S	Bcer	FALSE	Bc_Ga0417192_01_rn01	Other	NA
Bc_ctg1_1280	B1	402.9944711	Bcer	K	Bcer	FALSE	NA	NA	NA
Bc_ctg1_1281	B1	71.94557783	Bcer	L	Bcer	FALSE	NA	NA	NA
Bc_ctg1_1282	B1	0.19847056	Bcer	S	Bcer	FALSE	NA	NA	NA
Bc_ctg1_1283	B1	619.7243221	Bcer	S	Bcer	FALSE	NA	NA	NA
Bc_ctg1_1284	B1	104.6932201	Bcer	F	Bcer	FALSE	NA	NA	NA
Bc_ctg1_1285	B1	7.244175423	Bcer	M	Bcer	FALSE	NA	NA	NA
Bc_ctg1_1286	B1	25.70193746	Bcer	S	Bcer	FALSE	NA	NA	NA
Bc_ctg1_1287	B1	238.1646714	Bcer	S	Bcer	FALSE	NA	NA	NA
Bc_ctg1_1288	B1	270.4161373	Bcer	T	Bcer	FALSE	NA	NA	NA

Bc_ctg1_1289	B1	1.290058637	Bcer	S	Bcer	FALSE	NA	NA	NA
Bc_ctg1_129	B1	0.19847056	Bcer	S	Bcer	FALSE	Bc_Ga0417192_01_rn01	Other	NA
Bc_ctg1_1290	B1	0.09923528	Bcer	S	Bcer	FALSE	NA	NA	NA
Bc_ctg1_1291	B1	2.679352554	Bcer	S	Bcer	FALSE	NA	NA	NA
Bc_ctg1_1292	B1	1267.135287	Bcer	S	Bcer	FALSE	NA	NA	NA
Bc_ctg1_1293	B1	539.6414513	Bcer	J	Bcer	FALSE	NA	NA	NA
Bc_ctg1_1294	B1	260.3933741	Bcer	J	Bcer	FALSE	NA	NA	NA
Bc_ctg1_1295	B1	119.5785121	Bcer	J	Bcer	FALSE	NA	NA	NA
Bc_ctg1_1296	B1	300.5836624	Bcer	J	Bcer	FALSE	NA	NA	NA
Bc_ctg1_1297	B1	126.5249817	Bcer	O	Bcer	FALSE	NA	NA	NA
Bc_ctg1_1298	B1	1851.432615	Bcer	O	Bcer	FALSE	NA	NA	NA
Bc_ctg1_1299	B1	373.8192989	Bcer	O	Bcer	FALSE	NA	NA	NA
Bc_ctg1_13	B1	33.73999512	Bcer	Q	Bcer	TRUE	NA	NA	NA
Bc_ctg1_130	B1	2.083940875	Bcer	K	Bcer	FALSE	Bc_Ga0417192_01_rn01	Regulation	SMCOG1284
Bc_ctg1_1300	B1	252.4545517	Bcer	K	Bcer	FALSE	NA	NA	NA
Bc_ctg1_1301	B1	36.91552407	Bcer	H	Bcer	FALSE	NA	NA	NA
Bc_ctg1_1302	B1	8.23652822	Bcer	K	Bcer	FALSE	NA	NA	NA
Bc_ctg1_1303	B1	487.4436942	Bcer	M	Bcer	FALSE	NA	NA	NA
Bc_ctg1_1304	B1	0.595411679	Bcer	NA	Bcer	FALSE	NA	NA	NA
Bc_ctg1_1305	B1	2.381646714	Bcer	C	Bcer	FALSE	NA	NA	NA
Bc_ctg1_1306	B1	269.4237846	Bcer	J	Bcer	FALSE	NA	NA	NA
Bc_ctg1_1307	B1	53.78552163	Bcer	L	Bcer	FALSE	NA	NA	NA
Bc_ctg1_1308	B1	0.694646958	Bcer	S	Bcer	FALSE	NA	NA	NA
Bc_ctg1_1309	B1	9.725057417	Bcer	S	Bcer	FALSE	NA	NA	NA
Bc_ctg1_1310	B1	54.38093331	Bcer	F	Bcer	FALSE	NA	NA	NA
Bc_ctg1_1311	B1	3.96941119	Bcer	L	Bcer	FALSE	NA	NA	NA
Bc_ctg1_1312	B1	0	Bcer	E	Bcer	FALSE	NA	NA	NA
Bc_ctg1_1313	B1	289.1716052	Bcer	Q	Bcer	TRUE	NA	NA	NA
Bc_ctg1_1314	B1	155.1047423	Bcer	J	Bcer	FALSE	NA	NA	NA
Bc_ctg1_1315	B1	88.02169315	Bcer	H	Bcer	FALSE	NA	NA	NA
Bc_ctg1_1316	B1	104.8916907	Bcer	H	Bcer	FALSE	NA	NA	NA
Bc_ctg1_1317	B1	34.03770096	Bcer	J	Bcer	FALSE	NA	NA	NA
Bc_ctg1_1318	B1	184.1806792	Bcer	E	Bcer	FALSE	NA	NA	NA
Bc_ctg1_1319	B1	193.1118544	Bcer	S	Bcer	FALSE	NA	NA	NA
Bc_ctg1_1320	B1	84.44922308	Bcer	S	Bcer	FALSE	NA	NA	NA
Bc_ctg1_1321	B1	1.091588077	Bcer	NA	Bcer	FALSE	NA	NA	NA
Bc_ctg1_1322	B1	77.40351821	Bcer	I	Bcer	FALSE	NA	NA	NA
Bc_ctg1_1323	B1	1.686999756	Bcer	K	Bcer	FALSE	NA	NA	NA
Bc_ctg1_1324	B1	4.16788175	Bcer	NA	Bcer	FALSE	NA	NA	NA
Bc_ctg1_1325	B1	13.99217445	Bcer	K	Bcer	FALSE	NA	NA	NA
Bc_ctg1_1327	B1	11.31282189	Bcer	C	Bcer	FALSE	NA	NA	NA
Bc_ctg1_1328	B1	712.4100734	Bcer	S	Bcer	FALSE	NA	NA	NA
Bc_ctg1_1329	B1	0	Bcer	NA	Bcer	FALSE	NA	NA	NA
Bc_ctg1_133	B1	0.09923528	Bcer	NA	Bcer	FALSE	NA	NA	NA
Bc_ctg1_1330	B1	1.389293917	Bcer	P	Bcer	FALSE	NA	NA	NA
Bc_ctg1_1331	B1	5.160234548	Bcer	K	Bcer	FALSE	NA	NA	NA
Bc_ctg1_1332	B1	12.30517469	Bcer	I	Bcer	FALSE	NA	NA	NA
Bc_ctg1_1333	B1	37.41170047	Bcer	S	Bcer	FALSE	NA	NA	NA
Bc_ctg1_1334	B1	1.488529196	Bcer	NA	Bcer	FALSE	NA	NA	NA
Bc_ctg1_1335	B1	8.732704619	Bcer	G	Bcer	FALSE	NA	NA	NA
Bc_ctg1_1336	B1	8.732704619	Bcer	NA	Bcer	FALSE	NA	NA	NA
Bc_ctg1_1338	B1	3.572470071	Bcer	U	Bcer	FALSE	NA	NA	NA
Bc_ctg1_1339	B1	9.725057417	Bcer	O	Bcer	FALSE	NA	NA	NA
Bc_ctg1_134	B1	0.09923528	Bcer	NA	Bcer	FALSE	NA	NA	NA
Bc_ctg1_1340	B1	1.091588077	Bcer	E	Bcer	FALSE	NA	NA	NA
Bc_ctg1_1341	B1	0.893117518	Bcer	H	Bcer	FALSE	NA	NA	NA
Bc_ctg1_1342	B1	15.28223308	Bcer	S	Bcer	FALSE	NA	NA	NA
Bc_ctg1_1343	B1	0.19847056	Bcer	S	Bcer	FALSE	NA	NA	NA
Bc_ctg1_1344	B1	12.80135109	Bcer	K	Bcer	FALSE	NA	NA	NA
Bc_ctg1_1345	B1	3.572470071	Bcer	I	Bcer	FALSE	NA	NA	NA
Bc_ctg1_1346	B1	5.954116786	Bcer	K	Bcer	FALSE	NA	NA	NA
Bc_ctg1_1347	B1	2.282411435	Bcer	J	Bcer	FALSE	NA	NA	NA

Bc_ctg1_1348	B1	4.564822869	Bcer	C	Bcer	FALSE	NA	NA	NA
Bc_ctg1_1349	B1	37.31246519	Bcer	HP	Bcer	FALSE	NA	NA	NA
Bc_ctg1_135	B1	2.977058393	Bcer	T	Bcer	FALSE	NA	NA	NA
Bc_ctg1_1350	B1	36.32011239	Bcer	P	Bcer	FALSE	NA	NA	NA
Bc_ctg1_1351	B1	74.22798926	Bcer	P	Bcer	FALSE	NA	NA	NA
Bc_ctg1_1352	B1	0	Bcer	S	Bcer	FALSE	NA	NA	NA
Bc_ctg1_1353	B1	4694.821086	Bcer	C	Bcer	FALSE	NA	NA	NA
Bc_ctg1_1354	B1	570.3051528	Bcer	E	Bcer	FALSE	NA	NA	NA
Bc_ctg1_1355	B1	195.09656	Bcer	E	Bcer	FALSE	NA	NA	NA
Bc_ctg1_1356	B1	315.5681896	Bcer	E	Bcer	FALSE	NA	NA	NA
Bc_ctg1_1357	B1	141.9064501	Bcer	Q	Bcer	TRUE	NA	NA	NA
Bc_ctg1_1358	B1	2.580117274	Bcer	S	Bcer	FALSE	NA	NA	NA
Bc_ctg1_1359	B1	362.9034181	Bcer	S	Bcer	FALSE	NA	NA	NA
Bc_ctg1_136	B1	9.526586857	Bcer	T	Bcer	FALSE	NA	NA	NA
Bc_ctg1_1360	B1	0.297705839	Bcer	M	Bcer	FALSE	NA	NA	NA
Bc_ctg1_1363	B1	161.5550355	Bcer	K	Bcer	FALSE	NA	NA	NA
Bc_ctg1_1364	B1	102.510044	Bcer	F	Bcer	FALSE	NA	NA	NA
Bc_ctg1_1365	B1	160.1657415	Bcer	O	Bcer	FALSE	NA	NA	NA
Bc_ctg1_1366	B1	32.25146592	Bcer	O	Bcer	FALSE	NA	NA	NA
Bc_ctg1_1367	B1	16.37382116	Bcer	S	Bcer	FALSE	NA	NA	NA
Bc_ctg1_1368	B1	197.0812656	Bcer	S	Bcer	FALSE	NA	NA	NA
Bc_ctg1_1369	B1	119.6777474	Bcer	S	Bcer	FALSE	NA	NA	NA
Bc_ctg1_137	B1	0.992352798	Bcer	NA	Bcer	FALSE	NA	NA	NA
Bc_ctg1_1370	B1	433.8566431	Bcer	L	Bcer	FALSE	NA	NA	NA
Bc_ctg1_1371	B1	349.5066553	Bcer	S	Bcer	FALSE	NA	NA	NA
Bc_ctg1_1372	B1	645.922436	Bcer	J	Bcer	FALSE	Bc_Ga0417192_01_rn11	Other	NA
Bc_ctg1_1373	B1	8.038057661	Bcer	S	Bcer	FALSE	Bc_Ga0417192_01_rn11	Other	NA
Bc_ctg1_1374	B1	0.992352798	Bcer	S	Bcer	FALSE	Bc_Ga0417192_01_rn11	Other	NA
Bc_ctg1_1375	B1	0.09923528	Bcer	NA	Bcer	FALSE	Bc_Ga0417192_01_rn11	Other	NA
Bc_ctg1_1377	B1	5.854881506	Bcer	NA	Bcer	FALSE	Bc_Ga0417192_01_rn11	Other	NA
Bc_ctg1_1378	B1	72.64022479	Bcer	L	Bcer	FALSE	Bc_Ga0417192_01_rn11	Other	NA
Bc_ctg1_1379	B1	55.57175667	Bcer	S	Bcer	FALSE	Bc_Ga0417192_01_rn11	Other	SMCOG1272
Bc_ctg1_138	B1	60.93046177	Bcer	S	Bcer	FALSE	NA	NA	NA
Bc_ctg1_1380	B1	397.8342366	Bcer	J	Bcer	FALSE	Bc_Ga0417192_01_rn11	Other	NA
Bc_ctg1_1381	B1	300.2859566	Bcer	E	Bcer	FALSE	Bc_Ga0417192_01_rn11	Core biosynthesis	SMCOG1139
Bc_ctg1_1382	B1	71.05246031	Bcer	K	Bcer	FALSE	Bc_Ga0417192_01_rn11	Other	NA
Bc_ctg1_1383	B1	20.14476179	Bcer	L	Bcer	FALSE	Bc_Ga0417192_01_rn11	Other	NA
Bc_ctg1_1384	B1	31.25911312	Bcer	S	Bcer	FALSE	Bc_Ga0417192_01_rn11	Other	NA
Bc_ctg1_1385	B1	226.7526143	Bcer	H	Bcer	FALSE	Bc_Ga0417192_01_rn11	Ancillary biosynthesis	None
Bc_ctg1_1386	B1	329.6595994	Bcer	J	Bcer	FALSE	Bc_Ga0417192_01_rn11	Other	NA
Bc_ctg1_1387	B1	207.8979111	Bcer	J	Bcer	FALSE	Bc_Ga0417192_01_rn11	Other	SMCOG1162
Bc_ctg1_1388	B1	0.09923528	Bcer	NA	Bcer	FALSE	Bc_Ga0417192_01_rn11	Other	NA
Bc_ctg1_1389	B1	75.0218715	Bcer	J	Bcer	FALSE	Bc_Ga0417192_01_rn11	Other	NA
Bc_ctg1_139	B1	0.793882238	Bcer	S	Bcer	FALSE	NA	NA	NA
Bc_ctg1_1390	B1	543.3131567	Bcer	KT	Bcer	FALSE	Bc_Ga0417192_01_rn11	Other	NA
Bc_ctg1_1391	B1	200.8522062	Bcer	F	Bcer	FALSE	Bc_Ga0417192_01_rn11	Other	NA
Bc_ctg1_1392	B1	297.4081334	Bcer	L	Bcer	FALSE	Bc_Ga0417192_01_rn11	Other	NA
Bc_ctg1_1393	B1	7.442645982	Bcer	P	Bcer	FALSE	Bc_Ga0417192_01_rn11	Other	NA
Bc_ctg1_1394	B1	1015.573853	Bcer	U	Bcer	FALSE	Bc_Ga0417192_01_rn11	Other	NA
Bc_ctg1_1395	B1	19.64858539	Bcer	G	Bcer	FALSE	Bc_Ga0417192_01_rn11	Other	NA
Bc_ctg1_1396	B1	0	Bcer	S	Bcer	FALSE	Bc_Ga0417192_01_rn11	Core biosynthesis	SMCOG1086
Bc_ctg1_1397	B1	3.373999512	Bcer	S	Bcer	FALSE	Bc_Ga0417192_01_rn11	Other	NA
Bc_ctg1_1398	B1	0	Bcer	S	Bcer	FALSE	Bc_Ga0417192_01_rn11	Other	NA
Bc_ctg1_1399	B1	133.372216	Bcer	U	Bcer	FALSE	Bc_Ga0417192_01_rn11	Other	NA
Bc_ctg1_14	B1	0.496176399	Bcer	F	Bcer	FALSE	NA	NA	NA
Bc_ctg1_140	B1	3.473234792	Bcer	S	Bcer	FALSE	NA	NA	NA
Bc_ctg1_1400	B1	193.8065014	Bcer	F	Bcer	FALSE	Bc_Ga0417192_01_rn11	Other	NA
Bc_ctg1_1401	B1	139.1278622	Bcer	J	Bcer	FALSE	Bc_Ga0417192_01_rn11	Other	NA
Bc_ctg1_1402	B1	1.488529196	Bcer	S	Bcer	FALSE	Bc_Ga0417192_01_rn11	Other	NA
Bc_ctg1_1403	B1	61.22816761	Bcer	L	Bcer	FALSE	Bc_Ga0417192_01_rn11	Other	NA
Bc_ctg1_1404	B1	29.57211337	Bcer	L	Bcer	FALSE	Bc_Ga0417192_01_rn11	Other	NA
Bc_ctg1_1405	B1	17.36617396	Bcer	P	Bcer	FALSE	Bc_Ga0417192_01_rn11	Transport	SMCOG1033

Bc_ctg1_1406	B1	0.19847056	Bcer	S	Bcer	FALSE	Bc_Ga0417192_01_rn11	Other	NA
Bc_ctg1_1407	B1	31.3583484	Bcer	NA	Bcer	FALSE	Bc_Ga0417192_01_rn11	Other	NA
Bc_ctg1_1408	B1	1.091588077	Bcer	S	Bcer	FALSE	Bc_Ga0417192_01_rn11	Other	NA
Bc_ctg1_1409	B1	0	Bcer	M	Bcer	FALSE	NA	NA	NA
Bc_ctg1_141	B1	0.19847056	Bcer	S	Bcer	FALSE	NA	NA	NA
Bc_ctg1_1410	B1	3.572470071	Bcer	H	Bcer	FALSE	NA	NA	NA
Bc_ctg1_1411	B1	1.290058637	Bcer	H	Bcer	FALSE	NA	NA	NA
Bc_ctg1_1412	B1	1.885470315	Bcer	H	Bcer	FALSE	NA	NA	NA
Bc_ctg1_1413	B1	12.30517469	Bcer	E	Bcer	FALSE	NA	NA	NA
Bc_ctg1_1414	B1	40.09105302	Bcer	S	Bcer	FALSE	NA	NA	NA
Bc_ctg1_1415	B1	48.82375764	Bcer	S	Bcer	FALSE	NA	NA	NA
Bc_ctg1_1416	B1	86.53316395	Bcer	E	Bcer	FALSE	NA	NA	NA
Bc_ctg1_1417	B1	11.70976301	Bcer	V	Bcer	FALSE	NA	NA	NA
Bc_ctg1_1418	B1	44.95358173	Bcer	V	Bcer	FALSE	NA	NA	NA
Bc_ctg1_1419	B1	9.725057417	Bcer	V	Bcer	FALSE	NA	NA	NA
Bc_ctg1_142	B1	24.31264354	Bcer	T	Bcer	FALSE	NA	NA	NA
Bc_ctg1_1420	B1	30.36599561	Bcer	T	Bcer	FALSE	NA	NA	NA
Bc_ctg1_1421	B1	19.05317371	Bcer	T	Bcer	FALSE	NA	NA	NA
Bc_ctg1_1422	B1	252.0576106	Bcer	S	Bcer	FALSE	NA	NA	NA
Bc_ctg1_1423	B1	123.5479233	Bcer	T	Bcer	FALSE	NA	NA	NA
Bc_ctg1_1424	B1	827.1260568	Bcer	J	Bcer	FALSE	NA	NA	NA
Bc_ctg1_1425	B1	1966.446304	Bcer	J	Bcer	FALSE	NA	NA	NA
Bc_ctg1_1426	B1	444.7725239	Bcer	J	Bcer	FALSE	NA	NA	NA
Bc_ctg1_1427	B1	23.41952602	Bcer	J	Bcer	FALSE	NA	NA	NA
Bc_ctg1_1428	B1	8.13729294	Bcer	S	Bcer	FALSE	NA	NA	NA
Bc_ctg1_1429	B1	3.671705351	Bcer	M	Bcer	FALSE	NA	NA	NA
Bc_ctg1_143	B1	1.488529196	Bcer	V	Bcer	FALSE	NA	NA	NA
Bc_ctg1_1430	B1	439.4138188	Bcer	D	Bcer	FALSE	NA	NA	NA
Bc_ctg1_1431	B1	407.1623529	Bcer	D	Bcer	FALSE	NA	NA	NA
Bc_ctg1_1432	B1	200.2567946	Bcer	M	Bcer	FALSE	NA	NA	NA
Bc_ctg1_1433	B1	499.451163	Bcer	M	Bcer	FALSE	NA	NA	NA
Bc_ctg1_1434	B1	251.2637284	Bcer	D	Bcer	FALSE	NA	NA	NA
Bc_ctg1_1435	B1	17.46540924	Bcer	L	Bcer	FALSE	NA	NA	NA
Bc_ctg1_1437	B1	2.381646714	Bcer	E	Bcer	FALSE	NA	NA	NA
Bc_ctg1_1438	B1	31.15987785	Bcer	D	Bcer	FALSE	NA	NA	NA
Bc_ctg1_1439	B1	0.19847056	Bcer	NA	Bcer	FALSE	NA	NA	NA
Bc_ctg1_144	B1	0.297705839	Bcer	V	Bcer	FALSE	NA	NA	NA
Bc_ctg1_1440	B1	75.0218715	Bcer	H	Bcer	FALSE	NA	NA	NA
Bc_ctg1_1441	B1	561.1755071	Bcer	J	Bcer	FALSE	NA	NA	NA
Bc_ctg1_1442	B1	7.144940143	Bcer	S	Bcer	FALSE	NA	NA	NA
Bc_ctg1_1443	B1	0.496176399	Bcer	M	Bcer	FALSE	NA	NA	NA
Bc_ctg1_1444	B1	348.9112436	Bcer	H	Bcer	FALSE	NA	NA	NA
Bc_ctg1_1445	B1	140.3186856	Bcer	H	Bcer	FALSE	NA	NA	NA
Bc_ctg1_1446	B1	50.60999268	Bcer	H	Bcer	FALSE	NA	NA	NA
Bc_ctg1_1447	B1	87.92245787	Bcer	H	Bcer	FALSE	NA	NA	NA
Bc_ctg1_1448	B1	47.33522845	Bcer	O	Bcer	FALSE	NA	NA	NA
Bc_ctg1_1449	B1	226.9510848	Bcer	H	Bcer	FALSE	NA	NA	NA
Bc_ctg1_145	B1	0.595411679	Bcer	T	Bcer	FALSE	NA	NA	NA
Bc_ctg1_1450	B1	14.48835085	Bcer	K	Bcer	FALSE	NA	NA	NA
Bc_ctg1_1451	B1	44.15969949	Bcer	O	Bcer	FALSE	NA	NA	NA
Bc_ctg1_1452	B1	281.7289592	Bcer	D	Bcer	FALSE	NA	NA	NA
Bc_ctg1_1453	B1	563.5571538	Bcer	O	Bcer	FALSE	NA	NA	NA
Bc_ctg1_1454	B1	30.26676033	Bcer	LO	Bcer	FALSE	NA	NA	NA
Bc_ctg1_1455	B1	3108.545139	Bcer	O	Bcer	FALSE	NA	NA	NA
Bc_ctg1_1456	B1	2481.477406	Bcer	D	Bcer	FALSE	NA	NA	NA
Bc_ctg1_1457	B1	274.2863133	Bcer	O	Bcer	FALSE	NA	NA	NA
Bc_ctg1_1458	B1	6.747999024	Bcer	NA	Bcer	FALSE	NA	NA	NA
Bc_ctg1_146	B1	0.297705839	Bcer	T	Bcer	FALSE	NA	NA	NA
Bc_ctg1_1460	B1	160.5626827	Bcer	S	Bcer	FALSE	NA	NA	NA
Bc_ctg1_1461	B1	0.297705839	Bcer	NA	Bcer	FALSE	NA	NA	NA
Bc_ctg1_1462	B1	1.290058637	Bcer	NA	Bcer	FALSE	NA	NA	NA
Bc_ctg1_1463	B1	108.9603372	Bcer	S	Bcer	FALSE	NA	NA	NA

Bc_ctg1_1464	B1	80.67828245	Bcer	F	Bcer	FALSE	NA	NA	NA
Bc_ctg1_1465	B1	38.99946495	Bcer	J	Bcer	FALSE	NA	NA	NA
Bc_ctg1_1466	B1	0.396941119	Bcer	S	Bcer	FALSE	NA	NA	NA
Bc_ctg1_1467	B1	104.5939849	Bcer	M	Bcer	FALSE	NA	NA	NA
Bc_ctg1_1468	B1	2.183176155	Bcer	EGP	Bcer	FALSE	NA	NA	NA
Bc_ctg1_1469	B1	3.572470071	Bcer	S	Bcer	FALSE	NA	NA	NA
Bc_ctg1_147	B1	1.290058637	Bcer	V	Bcer	FALSE	NA	NA	NA
Bc_ctg1_1470	B1	6.549528464	Bcer	K	Bcer	FALSE	NA	NA	NA
Bc_ctg1_1471	B1	0	Bcer	S	Bcer	FALSE	NA	NA	NA
Bc_ctg1_1472	B1	0.496176399	Bcer	K	Bcer	FALSE	NA	NA	NA
Bc_ctg1_1473	B1	5.457940387	Bcer	F	Bcer	FALSE	NA	NA	NA
Bc_ctg1_1474	B1	24.11417298	Bcer	F	Bcer	FALSE	NA	NA	NA
Bc_ctg1_1475	B1	3.572470071	Bcer	KT	Bcer	FALSE	NA	NA	NA
Bc_ctg1_1476	B1	0.893117518	Bcer	K	Bcer	FALSE	NA	NA	NA
Bc_ctg1_1477	B1	4.664058149	Bcer	EG	Bcer	FALSE	NA	NA	NA
Bc_ctg1_1478	B1	7.541881262	Bcer	G	Bcer	FALSE	NA	NA	NA
Bc_ctg1_1479	B1	116.700689	Bcer	K	Bcer	FALSE	NA	NA	NA
Bc_ctg1_148	B1	3.671705351	Bcer	S	Bcer	FALSE	NA	NA	NA
Bc_ctg1_1480	B1	54.77787443	Bcer	CP	Bcer	FALSE	NA	NA	NA
Bc_ctg1_1481	B1	14.58758612	Bcer	S	Bcer	FALSE	NA	NA	NA
Bc_ctg1_1482	B1	83.8538114	Bcer	M	Bcer	FALSE	NA	NA	NA
Bc_ctg1_1483	B1	255.2331395	Bcer	N	Bcer	FALSE	NA	NA	NA
Bc_ctg1_1484	B1	217.424498	Bcer	N	Bcer	FALSE	NA	NA	NA
Bc_ctg1_1485	B1	23.71723186	Bcer	M	Bcer	FALSE	NA	NA	NA
Bc_ctg1_1486	B1	24.0149377	Bcer	S	Bcer	FALSE	NA	NA	NA
Bc_ctg1_1487	B1	1082.259961	Bcer	C	Bcer	FALSE	NA	NA	NA
Bc_ctg1_1488	B1	2441.882529	Bcer	C	Bcer	FALSE	NA	NA	NA
Bc_ctg1_1489	B1	483.5735183	Bcer	C	Bcer	FALSE	NA	NA	NA
Bc_ctg1_149	B1	34.23617152	Bcer	T	Bcer	FALSE	NA	NA	NA
Bc_ctg1_1490	B1	165.8221525	Bcer	S	Bcer	FALSE	NA	NA	NA
Bc_ctg1_1491	B1	55.07558027	Bcer	L	Bcer	FALSE	NA	NA	NA
Bc_ctg1_1492	B1	179.9135622	Bcer	O	Bcer	FALSE	NA	NA	NA
Bc_ctg1_1493	B1	338.1938334	Bcer	C	Bcer	FALSE	NA	NA	NA
Bc_ctg1_1494	B1	342.9571269	Bcer	C	Bcer	FALSE	NA	NA	NA
Bc_ctg1_1495	B1	301.6752505	Bcer	I	Bcer	FALSE	NA	NA	NA
Bc_ctg1_1496	B1	283.7136648	Bcer	K	Bcer	FALSE	NA	NA	NA
Bc_ctg1_1497	B1	19.05317371	Bcer	IQ	Bcer	TRUE	NA	NA	NA
Bc_ctg1_1498	B1	0.297705839	Bcer	Q	Bcer	TRUE	NA	NA	NA
Bc_ctg1_1499	B1	83.65534084	Bcer	P	Bcer	FALSE	NA	NA	NA
Bc_ctg1_15	B1	0.694646958	Bcer	V	Bcer	FALSE	NA	NA	NA
Bc_ctg1_150	B1	9.625822137	Bcer	C	Bcer	FALSE	NA	NA	NA
Bc_ctg1_1500	B1	26.4958197	Bcer	P	Bcer	FALSE	NA	NA	NA
Bc_ctg1_1501	B1	4.763293429	Bcer	NA	Bcer	FALSE	NA	NA	NA
Bc_ctg1_1502	B1	92.28881018	Bcer	S	Bcer	FALSE	NA	NA	NA
Bc_ctg1_1503	B1	14.38911557	Bcer	NA	Bcer	FALSE	NA	NA	NA
Bc_ctg1_1504	B1	41.57958222	Bcer	S	Bcer	FALSE	NA	NA	NA
Bc_ctg1_1505	B1	44.06046421	Bcer	S	Bcer	FALSE	NA	NA	NA
Bc_ctg1_1506	B1	29.86981921	Bcer	S	Bcer	FALSE	NA	NA	NA
Bc_ctg1_1507	B1	1.885470315	Bcer	NA	Bcer	FALSE	NA	NA	NA
Bc_ctg1_1508	B1	30.36599561	Bcer	V	Bcer	FALSE	NA	NA	NA
Bc_ctg1_1509	B1	84.44922308	Bcer	T	Bcer	FALSE	NA	NA	NA
Bc_ctg1_151	B1	4.06864647	Bcer	EGP	Bcer	FALSE	NA	NA	NA
Bc_ctg1_1510	B1	51.10616908	Bcer	T	Bcer	FALSE	NA	NA	NA
Bc_ctg1_1511	B1	2.282411435	Bcer	NA	Bcer	FALSE	NA	NA	NA
Bc_ctg1_1512	B1	0.992352798	Bcer	S	Bcer	FALSE	NA	NA	NA
Bc_ctg1_1513	B1	79.18975325	Bcer	J	Bcer	FALSE	NA	NA	NA
Bc_ctg1_1514	B1	21.93099683	Bcer	C	Bcer	FALSE	NA	NA	NA
Bc_ctg1_1515	B1	13.19829221	Bcer	S	Bcer	FALSE	NA	NA	NA
Bc_ctg1_1516	B1	12.99982165	Bcer	HP	Bcer	FALSE	NA	NA	NA
Bc_ctg1_1517	B1	8.038057661	Bcer	P	Bcer	FALSE	NA	NA	NA
Bc_ctg1_1518	B1	7.442645982	Bcer	P	Bcer	FALSE	NA	NA	NA
Bc_ctg1_1519	B1	81.07522356	Bcer	M	Bcer	FALSE	NA	NA	NA

Bc_ctg1_1520	B1	37.11399463	Bcer	M	Bcer	FALSE	NA	NA	NA
Bc_ctg1_1521	B1	60.53352065	Bcer	M	Bcer	FALSE	NA	NA	NA
Bc_ctg1_1522	B1	29.67134865	Bcer	E	Bcer	FALSE	NA	NA	NA
Bc_ctg1_1523	B1	9.328116298	Bcer	J	Bcer	FALSE	NA	NA	NA
Bc_ctg1_1525	B1	230.920496	Bcer	L	Bcer	FALSE	NA	NA	NA
Bc_ctg1_1526	B1	90.00639874	Bcer	L	Bcer	FALSE	NA	NA	NA
Bc_ctg1_1527	B1	88.61710483	Bcer	S	Bcer	FALSE	NA	NA	NA
Bc_ctg1_1528	B1	36.02240655	Bcer	D	Bcer	FALSE	NA	NA	NA
Bc_ctg1_1529	B1	18.35852676	Bcer	L	Bcer	FALSE	NA	NA	NA
Bc_ctg1_153	B1	37.31246519	Bcer	EU	Bcer	FALSE	NA	NA	NA
Bc_ctg1_1530	B1	13.39676277	Bcer	NA	Bcer	FALSE	NA	NA	NA
Bc_ctg1_1531	B1	3.870175911	Bcer	NA	Bcer	FALSE	NA	NA	NA
Bc_ctg1_1533	B1	292.7440753	Bcer	J	Bcer	FALSE	NA	NA	NA
Bc_ctg1_1534	B1	853.0264648	Bcer	J	Bcer	FALSE	NA	NA	NA
Bc_ctg1_1535	B1	273.7901369	Bcer	J	Bcer	FALSE	NA	NA	NA
Bc_ctg1_1536	B1	109.6549841	Bcer	J	Bcer	FALSE	NA	NA	NA
Bc_ctg1_1538	B1	15.6791742	Bcer	S	Bcer	FALSE	NA	NA	NA
Bc_ctg1_1539	B1	13.49599805	Bcer	S	Bcer	FALSE	NA	NA	NA
Bc_ctg1_154	B1	1.488529196	Bcer	NA	Bcer	FALSE	NA	NA	NA
Bc_ctg1_1540	B1	9.030410458	Bcer	NA	Bcer	FALSE	NA	NA	NA
Bc_ctg1_1541	B1	10.02276326	Bcer	NA	Bcer	FALSE	NA	NA	NA
Bc_ctg1_1542	B1	191.3256194	Bcer	EGP	Bcer	FALSE	NA	NA	NA
Bc_ctg1_1543	B1	38.60252383	Bcer	V	Bcer	FALSE	NA	NA	NA
Bc_ctg1_1544	B1	13.09905693	Bcer	K	Bcer	FALSE	NA	NA	NA
Bc_ctg1_1545	B1	941.5443344	Bcer	G	Bcer	FALSE	NA	NA	NA
Bc_ctg1_1546	B1	207.50097	Bcer	S	Bcer	FALSE	NA	NA	NA
Bc_ctg1_1547	B1	266.4467262	Bcer	J	Bcer	FALSE	NA	NA	NA
Bc_ctg1_1548	B1	206.3101466	Bcer	J	Bcer	FALSE	NA	NA	NA
Bc_ctg1_1549	B1	649.9910824	Bcer	J	Bcer	FALSE	NA	NA	NA
Bc_ctg1_1550	B1	2251.846968	Bcer	J	Bcer	FALSE	NA	NA	NA
Bc_ctg1_1551	B1	0.694646958	Bcer	S	Bcer	FALSE	NA	NA	NA
Bc_ctg1_1552	B1	1.885470315	Bcer	S	Bcer	FALSE	NA	NA	NA
Bc_ctg1_1553	B1	0.09923528	Bcer	L	Bcer	FALSE	NA	NA	NA
Bc_ctg1_1554	B1	0.595411679	Bcer	L	Bcer	FALSE	NA	NA	NA
Bc_ctg1_1555	B1	84.44922308	Bcer	L	Bcer	FALSE	NA	NA	NA
Bc_ctg1_1556	B1	31.55681896	Bcer	L	Bcer	FALSE	NA	NA	NA
Bc_ctg1_1557	B1	23.5187613	Bcer	K	Bcer	FALSE	NA	NA	NA
Bc_ctg1_1558	B1	220.4015563	Bcer	E	Bcer	FALSE	NA	NA	NA
Bc_ctg1_1559	B1	1406.858561	Bcer	G	Bcer	FALSE	NA	NA	NA
Bc_ctg1_156	B1	29.27440753	Bcer	E	Bcer	FALSE	NA	NA	NA
Bc_ctg1_1560	B1	12.20593941	Bcer	F	Bcer	FALSE	NA	NA	NA
Bc_ctg1_1561	B1	78.39587101	Bcer	P	Bcer	FALSE	NA	NA	NA
Bc_ctg1_1562	B1	166.6160347	Bcer	L	Bcer	FALSE	NA	NA	NA
Bc_ctg1_1563	B1	356.1554191	Bcer	L	Bcer	FALSE	NA	NA	NA
Bc_ctg1_1564	B1	121.5632177	Bcer	T	Bcer	FALSE	NA	NA	NA
Bc_ctg1_1565	B1	80.77751773	Bcer	T	Bcer	FALSE	NA	NA	NA
Bc_ctg1_1566	B1	0	Bcer	NA	Bcer	FALSE	NA	NA	NA
Bc_ctg1_1567	B1	0	Bcer	NA	Bcer	FALSE	NA	NA	NA
Bc_ctg1_1568	B1	44.75511117	Bcer	I	Bcer	FALSE	NA	NA	NA
Bc_ctg1_1569	B1	1373.813213	Bcer	C	Bcer	FALSE	NA	NA	NA
Bc_ctg1_157	B1	17.26693868	Bcer	S	Bcer	FALSE	NA	NA	NA
Bc_ctg1_1570	B1	1850.341026	Bcer	C	Bcer	FALSE	NA	NA	NA
Bc_ctg1_1571	B1	1495.773372	Bcer	C	Bcer	FALSE	NA	NA	NA
Bc_ctg1_1572	B1	5.060999268	Bcer	S	Bcer	FALSE	NA	NA	NA
Bc_ctg1_1573	B1	97.84598584	Bcer	S	Bcer	FALSE	NA	NA	NA
Bc_ctg1_1574	B1	2.877823113	Bcer	S	Bcer	FALSE	NA	NA	NA
Bc_ctg1_1575	B1	1938.660425	Bcer	G	Bcer	FALSE	NA	NA	NA
Bc_ctg1_1576	B1	653.1666114	Bcer	F	Bcer	FALSE	NA	NA	NA
Bc_ctg1_1577	B1	240.844024	Bcer	I	Bcer	FALSE	NA	NA	NA
Bc_ctg1_1578	B1	182.8906206	Bcer	I	Bcer	FALSE	NA	NA	NA
Bc_ctg1_1579	B1	212.0657928	Bcer	K	Bcer	FALSE	NA	NA	NA
Bc_ctg1_158	B1	9.526586857	Bcer	S	Bcer	FALSE	NA	NA	NA

Bc_ctg1_1580	B1	799.1417079	Bcer	C	Bcer	FALSE	NA	NA	NA
Bc_ctg1_1581	B1	181.0051503	Bcer	L	Bcer	FALSE	NA	NA	NA
Bc_ctg1_1582	B1	0	Bcer	S	Bcer	FALSE	NA	NA	NA
Bc_ctg1_1583	B1	0.793882238	Bcer	NA	Bcer	FALSE	NA	NA	NA
Bc_ctg1_1584	B1	164.6313291	Bcer	S	Bcer	FALSE	NA	NA	NA
Bc_ctg1_1585	B1	4.16788175	Bcer	S	Bcer	FALSE	NA	NA	NA
Bc_ctg1_1586	B1	11.61052773	Bcer	S	Bcer	FALSE	NA	NA	NA
Bc_ctg1_1587	B1	118.6853946	Bcer	K	Bcer	FALSE	NA	NA	NA
Bc_ctg1_1588	B1	0	Bcer	NA	Bcer	FALSE	NA	NA	NA
Bc_ctg1_1589	B1	177.3334449	Bcer	S	Bcer	FALSE	NA	NA	NA
Bc_ctg1_159	B1	0.396941119	Bcer	S	Bcer	FALSE	NA	NA	NA
Bc_ctg1_1590	B1	294.4310751	Bcer	E	Bcer	FALSE	NA	NA	NA
Bc_ctg1_1591	B1	7.045704863	Bcer	S	Bcer	FALSE	NA	NA	NA
Bc_ctg1_1592	B1	1.488529196	Bcer	NA	Bcer	FALSE	NA	NA	NA
Bc_ctg1_1593	B1	5.755646226	Bcer	S	Bcer	FALSE	NA	NA	NA
Bc_ctg1_1594	B1	0	Bcer	NA	Bcer	FALSE	NA	NA	NA
Bc_ctg1_1595	B1	1.786235036	Bcer	NA	Bcer	FALSE	NA	NA	NA
Bc_ctg1_1596	B1	5.854881506	Bcer	J	Bcer	FALSE	NA	NA	NA
Bc_ctg1_1597	B1	3.870175911	Bcer	G	Bcer	FALSE	NA	NA	NA
Bc_ctg1_1598	B1	1246.89129	Bcer	E	Bcer	FALSE	NA	NA	NA
Bc_ctg1_1599	B1	55.96869779	Bcer	IQ	Bcer	TRUE	NA	NA	NA
Bc_ctg1_16	B1	0.297705839	Bcer	U	Bcer	FALSE	NA	NA	NA
Bc_ctg1_160	B1	7.641116542	Bcer	Q	Bcer	TRUE	NA	NA	NA
Bc_ctg1_1600	B1	624.5868508	Bcer	T	Bcer	FALSE	NA	NA	NA
Bc_ctg1_1601	B1	18.45776204	Bcer	NA	Bcer	FALSE	NA	NA	NA
Bc_ctg1_1602	B1	0.595411679	Bcer	NA	Bcer	FALSE	NA	NA	NA
Bc_ctg1_1603	B1	126.1280406	Bcer	E	Bcer	FALSE	NA	NA	NA
Bc_ctg1_1604	B1	113.7236306	Bcer	E	Bcer	FALSE	NA	NA	NA
Bc_ctg1_1605	B1	20.93864403	Bcer	NA	Bcer	FALSE	NA	NA	NA
Bc_ctg1_1606	B1	0.297705839	Bcer	NA	Bcer	FALSE	NA	NA	NA
Bc_ctg1_1608	B1	413.5134108	Bcer	S	Bcer	FALSE	NA	NA	NA
Bc_ctg1_1609	B1	97.34980945	Bcer	S	Bcer	FALSE	NA	NA	NA
Bc_ctg1_161	B1	1.091588077	Bcer	E	Bcer	FALSE	NA	NA	NA
Bc_ctg1_1610	B1	0.496176399	Bcer	S	Bcer	FALSE	NA	NA	NA
Bc_ctg1_1611	B1	1.885470315	Bcer	F	Bcer	FALSE	NA	NA	NA
Bc_ctg1_1612	B1	244.0195529	Bcer	F	Bcer	FALSE	NA	NA	NA
Bc_ctg1_1613	B1	19.25164427	Bcer	L	Bcer	FALSE	NA	NA	NA
Bc_ctg1_1614	B1	506.1991621	Bcer	O	Bcer	FALSE	NA	NA	NA
Bc_ctg1_1615	B1	0.793882238	Bcer	S	Bcer	FALSE	NA	NA	NA
Bc_ctg1_1616	B1	0.496176399	Bcer	S	Bcer	FALSE	NA	NA	NA
Bc_ctg1_1617	B1	1302.165341	Bcer	G	Bcer	FALSE	NA	NA	NA
Bc_ctg1_1618	B1	17.1677034	Bcer	S	Bcer	FALSE	NA	NA	NA
Bc_ctg1_1619	B1	71.64787199	Bcer	S	Bcer	FALSE	NA	NA	NA
Bc_ctg1_162	B1	564.2518007	Bcer	L	Bcer	FALSE	NA	NA	NA
Bc_ctg1_1620	B1	3.373999512	Bcer	I	Bcer	FALSE	NA	NA	NA
Bc_ctg1_1621	B1	32.25146592	Bcer	S	Bcer	FALSE	NA	NA	NA
Bc_ctg1_1622	B1	201.9437943	Bcer	H	Bcer	FALSE	NA	NA	NA
Bc_ctg1_1623	B1	86.43392867	Bcer	E	Bcer	FALSE	NA	NA	NA
Bc_ctg1_1624	B1	469.3828733	Bcer	D	Bcer	FALSE	NA	NA	NA
Bc_ctg1_1625	B1	2.282411435	Bcer	K	Bcer	FALSE	NA	NA	NA
Bc_ctg1_1626	B1	0	Bcer	EG	Bcer	FALSE	NA	NA	NA
Bc_ctg1_1627	B1	47.73216957	Bcer	K	Bcer	FALSE	NA	NA	NA
Bc_ctg1_1628	B1	27.48817249	Bcer	T	Bcer	FALSE	NA	NA	NA
Bc_ctg1_1629	B1	9.030410458	Bcer	E	Bcer	FALSE	NA	NA	NA
Bc_ctg1_163	B1	30.86217201	Bcer	V	Bcer	FALSE	NA	NA	NA
Bc_ctg1_1630	B1	1541.719306	Bcer	J	Bcer	FALSE	NA	NA	NA
Bc_ctg1_1631	B1	1.190823357	Bcer	S	Bcer	FALSE	NA	NA	NA
Bc_ctg1_1632	B1	0.19847056	Bcer	NA	Bcer	FALSE	NA	NA	NA
Bc_ctg1_1633	B1	158.1810359	Bcer	J	Bcer	FALSE	NA	NA	NA
Bc_ctg1_1634	B1	3.274764232	Bcer	NA	Bcer	FALSE	NA	NA	NA
Bc_ctg1_1635	B1	25.5034669	Bcer	K	Bcer	FALSE	NA	NA	NA
Bc_ctg1_1636	B1	11.21358661	Bcer	NA	Bcer	FALSE	NA	NA	NA

Bc_ctg1_1637	B1	208.1956169	Bcer	S	Bcer	FALSE	NA	NA	NA
Bc_ctg1_1638	B1	84.8461642	Bcer	K	Bcer	FALSE	NA	NA	NA
Bc_ctg1_1639	B1	303.0645444	Bcer	I	Bcer	FALSE	NA	NA	NA
Bc_ctg1_164	B1	127.5173345	Bcer	V	Bcer	FALSE	NA	NA	NA
Bc_ctg1_1640	B1	64.99910824	Bcer	K	Bcer	FALSE	NA	NA	NA
Bc_ctg1_1641	B1	70.95322503	Bcer	S	Bcer	FALSE	NA	NA	NA
Bc_ctg1_1642	B1	39.99181774	Bcer	BQ	Bcer	TRUE	NA	NA	NA
Bc_ctg1_1643	B1	2.480881994	Bcer	I	Bcer	FALSE	NA	NA	NA
Bc_ctg1_1644	B1	14.19064501	Bcer	NA	Bcer	FALSE	NA	NA	NA
Bc_ctg1_1645	B1	77.40351821	Bcer	T	Bcer	FALSE	NA	NA	NA
Bc_ctg1_1646	B1	67.57922552	Bcer	T	Bcer	FALSE	NA	NA	NA
Bc_ctg1_1647	B1	0.793882238	Bcer	S	Bcer	FALSE	NA	NA	NA
Bc_ctg1_1648	B1	138.0362741	Bcer	S	Bcer	FALSE	NA	NA	NA
Bc_ctg1_1649	B1	128.5096873	Bcer	M	Bcer	FALSE	NA	NA	NA
Bc_ctg1_165	B1	16.671527	Bcer	K	Bcer	FALSE	NA	NA	NA
Bc_ctg1_1650	B1	18.25929148	Bcer	S	Bcer	FALSE	NA	NA	NA
Bc_ctg1_1651	B1	3.671705351	Bcer	S	Bcer	FALSE	NA	NA	NA
Bc_ctg1_1652	B1	23.81646714	Bcer	S	Bcer	FALSE	NA	NA	NA
Bc_ctg1_1653	B1	260.9887858	Bcer	K	Bcer	FALSE	NA	NA	NA
Bc_ctg1_1654	B1	0.793882238	Bcer	E	Bcer	FALSE	NA	NA	NA
Bc_ctg1_1655	B1	0	Bcer	E	Bcer	FALSE	NA	NA	NA
Bc_ctg1_1657	B1	221.7908503	Bcer	S	Bcer	FALSE	NA	NA	NA
Bc_ctg1_1658	B1	69.76240167	Bcer	O	Bcer	FALSE	NA	NA	NA
Bc_ctg1_1659	B1	66.88457856	Bcer	NA	Bcer	FALSE	NA	NA	NA
Bc_ctg1_166	B1	1.786235036	Bcer	G	Bcer	FALSE	NA	NA	NA
Bc_ctg1_1660	B1	606.5260299	Bcer	E	Bcer	FALSE	NA	NA	NA
Bc_ctg1_1661	B1	22.52640851	Bcer	S	Bcer	FALSE	NA	NA	NA
Bc_ctg1_1662	B1	7.740351821	Bcer	K	Bcer	FALSE	NA	NA	NA
Bc_ctg1_1663	B1	4.763293429	Bcer	J	Bcer	FALSE	NA	NA	NA
Bc_ctg1_1664	B1	1.984705595	Bcer	EG	Bcer	FALSE	NA	NA	NA
Bc_ctg1_1665	B1	0.396941119	Bcer	K	Bcer	FALSE	NA	NA	NA
Bc_ctg1_1666	B1	766.7910067	Bcer	M	Bcer	FALSE	NA	NA	NA
Bc_ctg1_1667	B1	465.4134621	Bcer	H	Bcer	FALSE	NA	NA	NA
Bc_ctg1_1668	B1	457.2761691	Bcer	D	Bcer	FALSE	NA	NA	NA
Bc_ctg1_1669	B1	3.175528952	Bcer	M	Bcer	FALSE	Bc_Ga0417192_01_rn12	Other	NA
Bc_ctg1_167	B1	4.961763988	Bcer	S	Bcer	FALSE	NA	NA	NA
Bc_ctg1_1670	B1	0.19847056	Bcer	NA	Bcer	FALSE	Bc_Ga0417192_01_rn12	Other	NA
Bc_ctg1_1671	B1	199.363677	Bcer	J	Bcer	FALSE	Bc_Ga0417192_01_rn12	Other	NA
Bc_ctg1_1672	B1	148.4559785	Bcer	S	Bcer	FALSE	Bc_Ga0417192_01_rn12	Other	NA
Bc_ctg1_1673	B1	59.04499146	Bcer	CO	Bcer	FALSE	Bc_Ga0417192_01_rn12	Other	NA
Bc_ctg1_1674	B1	2.083940875	Bcer	F	Bcer	FALSE	Bc_Ga0417192_01_rn12	Other	NA
Bc_ctg1_1675	B1	229.0350257	Bcer	G	Bcer	FALSE	Bc_Ga0417192_01_rn12	Ancillary biosynthesis	None
Bc_ctg1_1676	B1	12.50364525	Bcer	S	Bcer	FALSE	Bc_Ga0417192_01_rn12	Other	NA
Bc_ctg1_1677	B1	35.82393599	Bcer	S	Bcer	FALSE	Bc_Ga0417192_01_rn12	Other	NA
Bc_ctg1_1678	B1	38.40405327	Bcer	J	Bcer	FALSE	Bc_Ga0417192_01_rn12	Other	NA
Bc_ctg1_1679	B1	0	Bcer	S	Bcer	FALSE	Bc_Ga0417192_01_rn12	Other	NA
Bc_ctg1_168	B1	33.93846568	Bcer	T	Bcer	FALSE	NA	NA	NA
Bc_ctg1_1680	B1	69.66316639	Bcer	M	Bcer	FALSE	Bc_Ga0417192_01_rn12	Ancillary biosynthesis	None
Bc_ctg1_1681	B1	22.32793795	Bcer	G	Bcer	FALSE	Bc_Ga0417192_01_rn12	Core biosynthesis	SMCOG1297
Bc_ctg1_1682	B1	3.373999512	Bcer	J	Bcer	FALSE	Bc_Ga0417192_01_rn12	Other	NA
Bc_ctg1_1683	B1	336.7053042	Bcer	E	Bcer	FALSE	Bc_Ga0417192_01_rn12	Ancillary biosynthesis	SMCOG1247
Bc_ctg1_1684	B1	336.7053042	Bcer	K	Bcer	FALSE	Bc_Ga0417192_01_rn12	Other	NA
Bc_ctg1_1685	B1	46.54134621	Bcer	J	Bcer	FALSE	Bc_Ga0417192_01_rn12	Ancillary biosynthesis	SMCOG1154
Bc_ctg1_1686	B1	272.8970193	Bcer	S	Bcer	FALSE	Bc_Ga0417192_01_rn12	Core biosynthesis	SMCOG1086
Bc_ctg1_1687	B1	61.42663817	Bcer	S	Bcer	FALSE	Bc_Ga0417192_01_rn12	Other	NA
Bc_ctg1_1688	B1	114.8152187	Bcer	P	Bcer	FALSE	Bc_Ga0417192_01_rn12	Transport	SMCOG1005
Bc_ctg1_1689	B1	8.43499878	Bcer	K	Bcer	FALSE	Bc_Ga0417192_01_rn12	Regulation	SMCOG1058
Bc_ctg1_169	B1	8.633469339	Bcer	T	Bcer	FALSE	NA	NA	NA
Bc_ctg1_1690	B1	13.79370389	Bcer	S	Bcer	FALSE	Bc_Ga0417192_01_rn12	Other	NA
Bc_ctg1_1691	B1	0.19847056	Bcer	S	Bcer	FALSE	Bc_Ga0417192_01_rn12	Other	NA
Bc_ctg1_1693	B1	0.09923528	Bcer	S	Bcer	FALSE	Bc_Ga0417192_01_rn12	Other	NA
Bc_ctg1_1694	B1	539.2445102	Bcer	K	Bcer	FALSE	Bc_Ga0417192_01_rn12	Other	NA

Bc_ctg1_1695	B1	2.679352554	Bcer	IQ	Bcer	TRUE	Bc_Ga0417192_01_rn12	Ancillary biosynthesis	SMCOG1001
Bc_ctg1_1696	B1	0.09923528	Bcer	U	Bcer	FALSE	Bc_Ga0417192_01_rn12	Other	NA
Bc_ctg1_1697	B1	0	Bcer	S	Bcer	FALSE	Bc_Ga0417192_01_rn12	Other	NA
Bc_ctg1_1698	B1	3.96941119	Bcer	H	Bcer	FALSE	Bc_Ga0417192_01_rn12	Other	NA
Bc_ctg1_1699	B1	12.90058637	Bcer	H	Bcer	FALSE	Bc_Ga0417192_01_rn12	Other	NA
Bc_ctg1_17	B1	12.70211581	Bcer	M	Bcer	FALSE	NA	NA	NA
Bc_ctg1_170	B1	13.59523333	Bcer	S	Bcer	FALSE	NA	NA	NA
Bc_ctg1_1700	B1	33.44228928	Bcer	H	Bcer	FALSE	Bc_Ga0417192_01_rn12	Other	NA
Bc_ctg1_1701	B1	86.23545811	Bcer	H	Bcer	FALSE	Bc_Ga0417192_01_rn12	Other	NA
Bc_ctg1_1702	B1	17.86235036	Bcer	H	Bcer	FALSE	NA	NA	NA
Bc_ctg1_1703	B1	42.37346446	Bcer	H	Bcer	FALSE	NA	NA	NA
Bc_ctg1_1704	B1	48.12911068	Bcer	H	Bcer	FALSE	NA	NA	NA
Bc_ctg1_1705	B1	4.862528708	Bcer	Q	Bcer	TRUE	NA	NA	NA
Bc_ctg1_1706	B1	7.740351821	Bcer	NA	Bcer	FALSE	NA	NA	NA
Bc_ctg1_1707	B1	2.183176155	Bcer	NA	Bcer	FALSE	NA	NA	NA
Bc_ctg1_1708	B1	4.664058149	Bcer	P	Bcer	FALSE	NA	NA	NA
Bc_ctg1_1709	B1	1.290058637	Bcer	NA	Bcer	FALSE	NA	NA	NA
Bc_ctg1_171	B1	34.3354068	Bcer	NA	Bcer	FALSE	NA	NA	NA
Bc_ctg1_1710	B1	5.954116786	Bcer	E	Bcer	FALSE	NA	NA	NA
Bc_ctg1_1711	B1	0.09923528	Bcer	P	Bcer	FALSE	NA	NA	NA
Bc_ctg1_1712	B1	1.389293917	Bcer	E	Bcer	FALSE	NA	NA	NA
Bc_ctg1_1713	B1	2.083940875	Bcer	S	Bcer	FALSE	NA	NA	NA
Bc_ctg1_1714	B1	3.473234792	Bcer	NA	Bcer	FALSE	NA	NA	NA
Bc_ctg1_1715	B1	106.777161	Bcer	S	Bcer	FALSE	NA	NA	NA
Bc_ctg1_1716	B1	0	Bcer	NA	Bcer	FALSE	NA	NA	NA
Bc_ctg1_1718	B1	0.19847056	Bcer	NA	Bcer	FALSE	NA	NA	NA
Bc_ctg1_1719	B1	534.9773932	Bcer	J	Bcer	FALSE	NA	NA	NA
Bc_ctg1_172	B1	39.6941119	Bcer	V	Bcer	FALSE	NA	NA	NA
Bc_ctg1_1720	B1	8.43499878	Bcer	EGP	Bcer	FALSE	NA	NA	NA
Bc_ctg1_1721	B1	12.40440997	Bcer	P	Bcer	FALSE	NA	NA	NA
Bc_ctg1_1722	B1	12.80135109	Bcer	P	Bcer	FALSE	NA	NA	NA
Bc_ctg1_1723	B1	10.41970437	Bcer	L	Bcer	FALSE	NA	NA	NA
Bc_ctg1_1724	B1	78.49510629	Bcer	V	Bcer	FALSE	NA	NA	NA
Bc_ctg1_1725	B1	9.526586857	Bcer	V	Bcer	FALSE	NA	NA	NA
Bc_ctg1_1726	B1	1.389293917	Bcer	S	Bcer	FALSE	NA	NA	NA
Bc_ctg1_1728	B1	0.396941119	Bcer	S	Bcer	FALSE	NA	NA	NA
Bc_ctg1_1729	B1	15.1829978	Bcer	S	Bcer	FALSE	NA	NA	NA
Bc_ctg1_173	B1	19.74782067	Bcer	S	Bcer	FALSE	NA	NA	NA
Bc_ctg1_1730	B1	13.79370389	Bcer	M	Bcer	FALSE	NA	NA	NA
Bc_ctg1_1731	B1	8.23652822	Bcer	V	Bcer	FALSE	NA	NA	NA
Bc_ctg1_1732	B1	6.450293184	Bcer	NA	Bcer	FALSE	NA	NA	NA
Bc_ctg1_1733	B1	4.664058149	Bcer	M	Bcer	FALSE	NA	NA	NA
Bc_ctg1_1734	B1	9.923527976	Bcer	S	Bcer	FALSE	NA	NA	NA
Bc_ctg1_1735	B1	218.8137919	Bcer	S	Bcer	FALSE	NA	NA	NA
Bc_ctg1_1736	B1	29.96905449	Bcer	I	Bcer	FALSE	NA	NA	NA
Bc_ctg1_1737	B1	24.21340826	Bcer	S	Bcer	FALSE	NA	NA	NA
Bc_ctg1_1738	B1	0.297705839	Bcer	I	Bcer	FALSE	NA	NA	NA
Bc_ctg1_1739	B1	0.19847056	Bcer	M	Bcer	FALSE	NA	NA	NA
Bc_ctg1_174	B1	0.297705839	Bcer	I	Bcer	FALSE	NA	NA	NA
Bc_ctg1_1740	B1	27.38893721	Bcer	H	Bcer	FALSE	NA	NA	NA
Bc_ctg1_1741	B1	22.52640851	Bcer	H	Bcer	FALSE	NA	NA	NA
Bc_ctg1_1742	B1	0.992352798	Bcer	NA	Bcer	FALSE	NA	NA	NA
Bc_ctg1_1743	B1	979.6506818	Bcer	H	Bcer	FALSE	NA	NA	NA
Bc_ctg1_1744	B1	1804.692798	Bcer	C	Bcer	FALSE	NA	NA	NA
Bc_ctg1_1745	B1	0	Bcer	S	Bcer	FALSE	NA	NA	NA
Bc_ctg1_1746	B1	0	Bcer	NA	Bcer	FALSE	NA	NA	NA
Bc_ctg1_1747	B1	9.129645738	Bcer	EG	Bcer	FALSE	NA	NA	NA
Bc_ctg1_1748	B1	4.961763988	Bcer	S	Bcer	FALSE	NA	NA	NA
Bc_ctg1_1749	B1	6.946469583	Bcer	S	Bcer	FALSE	NA	NA	NA
Bc_ctg1_175	B1	235.6837894	Bcer	S	Bcer	FALSE	NA	NA	NA
Bc_ctg1_1750	B1	1.190823357	Bcer	Q	Bcer	TRUE	NA	NA	NA
Bc_ctg1_1751	B1	1.786235036	Bcer	S	Bcer	FALSE	NA	NA	NA

Bc_ctg1_1752	B1	1.686999756	Bcer	S	Bcer	FALSE	NA	NA	NA
Bc_ctg1_1753	B1	0.396941119	Bcer	S	Bcer	FALSE	NA	NA	NA
Bc_ctg1_1754	B1	0	Bcer	NA	Bcer	FALSE	NA	NA	NA
Bc_ctg1_1755	B1	0.496176399	Bcer	NU	Bcer	FALSE	NA	NA	NA
Bc_ctg1_1756	B1	27.98434889	Bcer	T	Bcer	FALSE	NA	NA	NA
Bc_ctg1_1757	B1	3.671705351	Bcer	T	Bcer	FALSE	NA	NA	NA
Bc_ctg1_1758	B1	0.19847056	Bcer	S	Bcer	FALSE	NA	NA	NA
Bc_ctg1_1759	B1	111.5404545	Bcer	M	Bcer	FALSE	NA	NA	NA
Bc_ctg1_176	B1	73.0371659	Bcer	EH	Bcer	FALSE	NA	NA	NA
Bc_ctg1_1760	B1	6.450293184	Bcer	NA	Bcer	FALSE	NA	NA	NA
Bc_ctg1_1761	B1	7.442645982	Bcer	S	Bcer	FALSE	NA	NA	NA
Bc_ctg1_1762	B1	0.595411679	Bcer	P	Bcer	FALSE	NA	NA	NA
Bc_ctg1_1763	B1	2.083940875	Bcer	P	Bcer	FALSE	NA	NA	NA
Bc_ctg1_1764	B1	3.175528952	Bcer	P	Bcer	FALSE	NA	NA	NA
Bc_ctg1_1765	B1	0	Bcer	NA	Bcer	FALSE	NA	NA	NA
Bc_ctg1_1766	B1	15.57993892	Bcer	NA	Bcer	FALSE	NA	NA	NA
Bc_ctg1_1767	B1	7.740351821	Bcer	S	Bcer	FALSE	NA	NA	NA
Bc_ctg1_1768	B1	7.244175423	Bcer	S	Bcer	FALSE	NA	NA	NA
Bc_ctg1_1769	B1	179.0204447	Bcer	L	Bcer	FALSE	NA	NA	NA
Bc_ctg1_177	B1	26.59505498	Bcer	E	Bcer	FALSE	NA	NA	NA
Bc_ctg1_1770	B1	0.19847056	Bcer	NA	Bcer	FALSE	NA	NA	NA
Bc_ctg1_1772	B1	0	Bcer	NA	Bcer	FALSE	NA	NA	NA
Bc_ctg1_1773	B1	0.09923528	Bcer	NA	Bcer	FALSE	NA	NA	NA
Bc_ctg1_1774	B1	235.3860836	Bcer	T	Bcer	FALSE	NA	NA	NA
Bc_ctg1_1775	B1	1.488529196	Bcer	S	Bcer	FALSE	NA	NA	NA
Bc_ctg1_1776	B1	136.0515686	Bcer	P	Bcer	FALSE	NA	NA	NA
Bc_ctg1_1777	B1	356.6515955	Bcer	C	Bcer	FALSE	NA	NA	NA
Bc_ctg1_1778	B1	219.7069094	Bcer	C	Bcer	FALSE	NA	NA	NA
Bc_ctg1_1779	B1	0.09923528	Bcer	S	Bcer	FALSE	NA	NA	NA
Bc_ctg1_178	B1	0.19847056	Bcer	S	Bcer	FALSE	NA	NA	NA
Bc_ctg1_1780	B1	7.244175423	Bcer	S	Bcer	FALSE	NA	NA	NA
Bc_ctg1_1781	B1	0	Bcer	S	Bcer	FALSE	NA	NA	NA
Bc_ctg1_1782	B1	55.47252139	Bcer	S	Bcer	FALSE	NA	NA	NA
Bc_ctg1_1783	B1	15.28223308	Bcer	NA	Bcer	FALSE	NA	NA	NA
Bc_ctg1_1784	B1	186.2646201	Bcer	S	Bcer	FALSE	NA	NA	NA
Bc_ctg1_1785	B1	95.76204497	Bcer	S	Bcer	FALSE	NA	NA	NA
Bc_ctg1_1786	B1	29.37364281	Bcer	S	Bcer	FALSE	NA	NA	NA
Bc_ctg1_1787	B1	463.5279918	Bcer	KT	Bcer	FALSE	NA	NA	NA
Bc_ctg1_1788	B1	17.86235036	Bcer	S	Bcer	FALSE	NA	NA	NA
Bc_ctg1_179	B1	14.78605668	Bcer	G	Bcer	FALSE	NA	NA	NA
Bc_ctg1_1791	B1	0.595411679	Bcer	S	Bcer	FALSE	NA	NA	NA
Bc_ctg1_1792	B1	299.6905449	Bcer	P	Bcer	FALSE	NA	NA	NA
Bc_ctg1_1793	B1	23.32029074	Bcer	P	Bcer	FALSE	NA	NA	NA
Bc_ctg1_1794	B1	12.20593941	Bcer	S	Bcer	FALSE	NA	NA	NA
Bc_ctg1_1795	B1	69.76240167	Bcer	T	Bcer	FALSE	NA	NA	NA
Bc_ctg1_1796	B1	22.72487907	Bcer	T	Bcer	FALSE	NA	NA	NA
Bc_ctg1_1797	B1	25.60270218	Bcer	M	Bcer	FALSE	NA	NA	NA
Bc_ctg1_1798	B1	14.38911557	Bcer	NA	Bcer	FALSE	NA	NA	NA
Bc_ctg1_1799	B1	234.7906719	Bcer	NA	Bcer	FALSE	NA	NA	NA
Bc_ctg1_18	B1	30.26676033	Bcer	K	Bcer	FALSE	NA	NA	NA
Bc_ctg1_180	B1	1.091588077	Bcer	G	Bcer	FALSE	NA	NA	NA
Bc_ctg1_1800	B1	48.5260518	Bcer	NA	Bcer	FALSE	NA	NA	NA
Bc_ctg1_1801	B1	272.1031371	Bcer	F	Bcer	FALSE	NA	NA	NA
Bc_ctg1_1802	B1	123.1509822	Bcer	I	Bcer	FALSE	NA	NA	NA
Bc_ctg1_1803	B1	9.526586857	Bcer	S	Bcer	FALSE	NA	NA	NA
Bc_ctg1_1804	B1	11.80899829	Bcer	NA	Bcer	FALSE	NA	NA	NA
Bc_ctg1_1805	B1	13.39676277	Bcer	S	Bcer	FALSE	NA	NA	NA
Bc_ctg1_1806	B1	0.396941119	Bcer	NA	Bcer	FALSE	NA	NA	NA
Bc_ctg1_1807	B1	1.885470315	Bcer	NA	Bcer	FALSE	NA	NA	NA
Bc_ctg1_1808	B1	4.862528708	Bcer	S	Bcer	FALSE	NA	NA	NA
Bc_ctg1_1809	B1	1.984705595	Bcer	I	Bcer	FALSE	NA	NA	NA
Bc_ctg1_181	B1	1.587764476	Bcer	K	Bcer	FALSE	NA	NA	NA

Bc_ctg1_1810	B1	214.248969	Bcer	V	Bcer	FALSE	NA	NA	NA
Bc_ctg1_1811	B1	36.71705351	Bcer	V	Bcer	FALSE	NA	NA	NA
Bc_ctg1_1812	B1	22.32793795	Bcer	K	Bcer	FALSE	NA	NA	NA
Bc_ctg1_1813	B1	21.93099683	Bcer	S	Bcer	FALSE	NA	NA	NA
Bc_ctg1_1814	B1	12.30517469	Bcer	S	Bcer	FALSE	NA	NA	NA
Bc_ctg1_1815	B1	2.877823113	Bcer	K	Bcer	FALSE	NA	NA	NA
Bc_ctg1_1816	B1	36.71705351	Bcer	K	Bcer	FALSE	NA	NA	NA
Bc_ctg1_1817	B1	67.77769608	Bcer	T	Bcer	FALSE	NA	NA	NA
Bc_ctg1_1818	B1	53.19010995	Bcer	V	Bcer	FALSE	NA	NA	NA
Bc_ctg1_1819	B1	43.66352309	Bcer	V	Bcer	FALSE	NA	NA	NA
Bc_ctg1_182	B1	6.549528464	Bcer	S	Bcer	FALSE	NA	NA	NA
Bc_ctg1_1820	B1	12.80135109	Bcer	V	Bcer	FALSE	NA	NA	NA
Bc_ctg1_1821	B1	52.79316883	Bcer	V	Bcer	FALSE	NA	NA	NA
Bc_ctg1_1823	B1	5.656410946	Bcer	EG	Bcer	FALSE	NA	NA	NA
Bc_ctg1_1824	B1	14.88529196	Bcer	K	Bcer	FALSE	NA	NA	NA
Bc_ctg1_1825	B1	12.40440997	Bcer	G	Bcer	FALSE	NA	NA	NA
Bc_ctg1_1826	B1	11.41205717	Bcer	S	Bcer	FALSE	NA	NA	NA
Bc_ctg1_1827	B1	22.32793795	Bcer	E	Bcer	FALSE	NA	NA	NA
Bc_ctg1_1828	B1	6.747999024	Bcer	S	Bcer	FALSE	NA	NA	NA
Bc_ctg1_1829	B1	3.870175911	Bcer	M	Bcer	FALSE	NA	NA	NA
Bc_ctg1_183	B1	25.70193746	Bcer	C	Bcer	FALSE	NA	NA	NA
Bc_ctg1_1830	B1	22.92334962	Bcer	T	Bcer	FALSE	NA	NA	NA
Bc_ctg1_1831	B1	11.31282189	Bcer	T	Bcer	FALSE	NA	NA	NA
Bc_ctg1_1832	B1	3.373999512	Bcer	H	Bcer	FALSE	NA	NA	NA
Bc_ctg1_1833	B1	6.152587345	Bcer	H	Bcer	FALSE	NA	NA	NA
Bc_ctg1_1834	B1	760.7376547	Bcer	H	Bcer	FALSE	NA	NA	NA
Bc_ctg1_1835	B1	32.54917176	Bcer	S	Bcer	FALSE	NA	NA	NA
Bc_ctg1_1836	B1	97.94522112	Bcer	H	Bcer	FALSE	Bc_Ga0417192_01_rn13	Other	NA
Bc_ctg1_1837	B1	106.8763963	Bcer	HQ	Bcer	TRUE	Bc_Ga0417192_01_rn13	Ancillary biosynthesis	SMCOG1018
Bc_ctg1_1838	B1	18.1600562	Bcer	H	Bcer	FALSE	Bc_Ga0417192_01_rn13	Ancillary biosynthesis	None
Bc_ctg1_1839	B1	0.496176399	Bcer	T	Bcer	FALSE	Bc_Ga0417192_01_rn13	Other	NA
Bc_ctg1_184	B1	0	Bcer	L	Bcer	FALSE	NA	NA	NA
Bc_ctg1_1840	B1	266.744432	Bcer	K	Bcer	FALSE	Bc_Ga0417192_01_rn13	Regulation	SMCOG1255
Bc_ctg1_1841	B1	79.48745909	Bcer	S	Bcer	FALSE	Bc_Ga0417192_01_rn13	Other	NA
Bc_ctg1_1842	B1	1.190823357	Bcer	S	Bcer	FALSE	Bc_Ga0417192_01_rn13	Other	NA
Bc_ctg1_1843	B1	3.671705351	Bcer	K	Bcer	FALSE	Bc_Ga0417192_01_rn13	Regulation	SMCOG1171
Bc_ctg1_1844	B1	0.09923528	Bcer	I	Bcer	FALSE	Bc_Ga0417192_01_rn13	Other	NA
Bc_ctg1_1845	B1	0	Bcer	NA	Bcer	FALSE	Bc_Ga0417192_01_rn13	Other	NA
Bc_ctg1_1846	B1	818.9887639	Bcer	G	Bcer	FALSE	Bc_Ga0417192_01_rn13	Other	NA
Bc_ctg1_1847	B1	340.9724213	Bcer	G	Bcer	FALSE	Bc_Ga0417192_01_rn13	Core biosynthesis	SMCOG1045
Bc_ctg1_1848	B1	353.6745371	Bcer	G	Bcer	FALSE	Bc_Ga0417192_01_rn13	Ancillary biosynthesis	SMCOG1064
Bc_ctg1_1849	B1	286.1945468	Bcer	G	Bcer	FALSE	Bc_Ga0417192_01_rn13	Ancillary biosynthesis	SMCOG1064
Bc_ctg1_185	B1	29.27440753	Bcer	M	Bcer	FALSE	NA	NA	NA
Bc_ctg1_1850	B1	447.650347	Bcer	G	Bcer	FALSE	Bc_Ga0417192_01_rn13	Core biosynthesis	SMCOG1297
Bc_ctg1_1852	B1	5.060999268	Bcer	O	Bcer	FALSE	Bc_Ga0417192_01_rn13	Ancillary biosynthesis	SMCOG1075
Bc_ctg1_1853	B1	7072.399153	Bcer	C	Bcer	FALSE	Bc_Ga0417192_01_rn13	Other	NA
Bc_ctg1_1855	B1	7.938822381	Bcer	S	Bcer	FALSE	Bc_Ga0417192_01_rn13	Other	NA
Bc_ctg1_1856	B1	0.297705839	Bcer	P	Bcer	FALSE	Bc_Ga0417192_01_rn13	Other	NA
Bc_ctg1_1857	B1	0.496176399	Bcer	S	Bcer	FALSE	Bc_Ga0417192_01_rn13	Other	NA
Bc_ctg1_1858	B1	1295.516577	Bcer	G	Bcer	FALSE	Bc_Ga0417192_01_rn13	Other	NA
Bc_ctg1_1859	B1	2.480881994	Bcer	S	Bcer	FALSE	Bc_Ga0417192_01_rn13	Other	NA
Bc_ctg1_186	B1	0.793882238	Bcer	NA	Bcer	FALSE	NA	NA	NA
Bc_ctg1_1860	B1	106.4794552	Bcer	J	Bcer	FALSE	Bc_Ga0417192_01_rn13	Other	NA
Bc_ctg1_1861	B1	90.10563402	Bcer	E	Bcer	FALSE	Bc_Ga0417192_01_rn13	Core biosynthesis	SMCOG1019
Bc_ctg1_1862	B1	3.274764232	Bcer	K	Bcer	FALSE	Bc_Ga0417192_01_rn13	Regulation	SMCOG1120
Bc_ctg1_1863	B1	115.6091009	Bcer	CH	Bcer	FALSE	Bc_Ga0417192_01_rn13	Ancillary biosynthesis	SMCOG1072
Bc_ctg1_1864	B1	0	Bcer	I	Bcer	FALSE	Bc_Ga0417192_01_rn13	Ancillary biosynthesis	SMCOG1262
Bc_ctg1_1865	B1	1.190823357	Bcer	S	Bcer	FALSE	Bc_Ga0417192_01_rn13	Other	NA
Bc_ctg1_1866	B1	47.93064012	Bcer	E	Bcer	FALSE	Bc_Ga0417192_01_rn13	Ancillary biosynthesis	SMCOG1019
Bc_ctg1_1867	B1	10.51893965	Bcer	P	Bcer	FALSE	Bc_Ga0417192_01_rn13	Other	NA
Bc_ctg1_1868	B1	0.09923528	Bcer	NA	Bcer	FALSE	Bc_Ga0417192_01_rn13	Other	NA
Bc_ctg1_1869	B1	79.88440021	Bcer	G	Bcer	FALSE	Bc_Ga0417192_01_rn13	Other	NA

Bc_ctg1_187	B1	97.84598584	Bcer	L	Bcer	FALSE	NA	NA	NA
Bc_ctg1_1870	B1	4.564822869	Bcer	L	Bcer	FALSE	Bc_Ga0417192_01_rn13	Other	NA
Bc_ctg1_1871	B1	44.06046421	Bcer	P	Bcer	FALSE	Bc_Ga0417192_01_rn13	Other	NA
Bc_ctg1_1872	B1	0.396941119	Bcer	NA	Bcer	FALSE	Bc_Ga0417192_01_rn13	Other	NA
Bc_ctg1_1873	B1	7.740351821	Bcer	L	Bcer	FALSE	Bc_Ga0417192_01_rn13	Other	NA
Bc_ctg1_1874	B1	426.3147619	Bcer	J	Bcer	FALSE	Bc_Ga0417192_01_rn13	Other	NA
Bc_ctg1_1875	B1	77.00657709	Bcer	Q	Bcer	TRUE	Bc_Ga0417192_01_rn13	Ancillary biosynthesis	SMCOG1144
Bc_ctg1_1876	B1	0.09923528	Bcer	S	Bcer	FALSE	Bc_Ga0417192_01_rn13	Other	NA
Bc_ctg1_1877	B1	19.64858539	Bcer	S	Bcer	FALSE	Bc_Ga0417192_01_rn13	Other	NA
Bc_ctg1_1878	B1	24.21340826	Bcer	S	Bcer	FALSE	Bc_Ga0417192_01_rn13	Other	NA
Bc_ctg1_1879	B1	272.2023724	Bcer	M	Bcer	FALSE	Bc_Ga0417192_01_rn13	Core biosynthesis	SMCOG1064
Bc_ctg1_188	B1	2.877823113	Bcer	NA	Bcer	FALSE	NA	NA	NA
Bc_ctg1_1880	B1	548.374156	Bcer	G	Bcer	FALSE	Bc_Ga0417192_01_rn13	Other	NA
Bc_ctg1_1881	B1	1.091588077	Bcer	S	Bcer	FALSE	Bc_Ga0417192_01_rn13	Other	NA
Bc_ctg1_1882	B1	53.98399219	Bcer	NA	Bcer	FALSE	Bc_Ga0417192_01_rn13	Other	NA
Bc_ctg1_1883	B1	11.51129245	Bcer	S	Bcer	FALSE	Bc_Ga0417192_01_rn13	Other	NA
Bc_ctg1_1884	B1	2.977058393	Bcer	P	Bcer	FALSE	Bc_Ga0417192_01_rn13	Other	NA
Bc_ctg1_1885	B1	98.63986808	Bcer	H	Bcer	FALSE	Bc_Ga0417192_01_rn13	Ancillary biosynthesis	None
Bc_ctg1_1886	B1	834.3702322	Bcer	E	Bcer	FALSE	Bc_Ga0417192_01_rn13	Other	NA
Bc_ctg1_1887	B1	4.366352309	Bcer	S	Bcer	FALSE	Bc_Ga0417192_01_rn13	Other	NA
Bc_ctg1_1888	B1	21.03787931	Bcer	S	Bcer	FALSE	Bc_Ga0417192_01_rn13	Other	NA
Bc_ctg1_1889	B1	7.740351821	Bcer	F	Bcer	FALSE	Bc_Ga0417192_01_rn13	Other	NA
Bc_ctg1_189	B1	56.26640362	Bcer	M	Bcer	FALSE	NA	NA	NA
Bc_ctg1_1890	B1	23.41952602	Bcer	C	Bcer	FALSE	NA	NA	NA
Bc_ctg1_1891	B1	274.4847838	Bcer	C	Bcer	FALSE	NA	NA	NA
Bc_ctg1_1892	B1	56.3656389	Bcer	M	Bcer	FALSE	NA	NA	NA
Bc_ctg1_1893	B1	14.09140973	Bcer	K	Bcer	FALSE	NA	NA	NA
Bc_ctg1_1894	B1	72.73946007	Bcer	EGP	Bcer	FALSE	NA	NA	NA
Bc_ctg1_1895	B1	0.793882238	Bcer	K	Bcer	FALSE	NA	NA	NA
Bc_ctg1_1896	B1	5.160234548	Bcer	S	Bcer	FALSE	NA	NA	NA
Bc_ctg1_1897	B1	7.343410702	Bcer	S	Bcer	FALSE	NA	NA	NA
Bc_ctg1_1898	B1	0.19847056	Bcer	S	Bcer	FALSE	NA	NA	NA
Bc_ctg1_1899	B1	0	Bcer	NA	Bcer	FALSE	NA	NA	NA
Bc_ctg1_19	B1	5.060999268	Bcer	O	Bcer	FALSE	NA	NA	NA
Bc_ctg1_190	B1	75.5180479	Bcer	M	Bcer	FALSE	NA	NA	NA
Bc_ctg1_1900	B1	8.13729294	Bcer	NA	Bcer	FALSE	NA	NA	NA
Bc_ctg1_1901	B1	9.824292696	Bcer	J	Bcer	FALSE	NA	NA	NA
Bc_ctg1_1902	B1	179.6158564	Bcer	S	Bcer	FALSE	NA	NA	NA
Bc_ctg1_1903	B1	36.22087711	Bcer	E	Bcer	FALSE	NA	NA	NA
Bc_ctg1_1904	B1	10.12199854	Bcer	S	Bcer	FALSE	NA	NA	NA
Bc_ctg1_1905	B1	295.5226631	Bcer	C	Bcer	FALSE	NA	NA	NA
Bc_ctg1_1906	B1	7.839587101	Bcer	V	Bcer	FALSE	NA	NA	NA
Bc_ctg1_1907	B1	2.679352554	Bcer	S	Bcer	FALSE	NA	NA	NA
Bc_ctg1_1908	B1	32.15223064	Bcer	P	Bcer	FALSE	NA	NA	NA
Bc_ctg1_1909	B1	5.259469827	Bcer	NU	Bcer	FALSE	NA	NA	NA
Bc_ctg1_191	B1	0.893117518	Bcer	S	Bcer	FALSE	NA	NA	NA
Bc_ctg1_1910	B1	2.381646714	Bcer	S	Bcer	FALSE	NA	NA	NA
Bc_ctg1_1911	B1	0.893117518	Bcer	NA	Bcer	FALSE	NA	NA	NA
Bc_ctg1_1912	B1	0.694646958	Bcer	S	Bcer	FALSE	NA	NA	NA
Bc_ctg1_1913	B1	1.686999756	Bcer	J	Bcer	FALSE	NA	NA	NA
Bc_ctg1_1914	B1	3.373999512	Bcer	S	Bcer	FALSE	NA	NA	NA
Bc_ctg1_1915	B1	98.5406328	Bcer	O	Bcer	FALSE	NA	NA	NA
Bc_ctg1_1916	B1	24.61034938	Bcer	NA	Bcer	FALSE	NA	NA	NA
Bc_ctg1_1917	B1	10.91588077	Bcer	S	Bcer	FALSE	NA	NA	NA
Bc_ctg1_1918	B1	48.42681652	Bcer	I	Bcer	FALSE	NA	NA	NA
Bc_ctg1_1919	B1	1.389293917	Bcer	J	Bcer	FALSE	NA	NA	NA
Bc_ctg1_192	B1	27.58740777	Bcer	EGP	Bcer	FALSE	NA	NA	NA
Bc_ctg1_1920	B1	6.152587345	Bcer	G	Bcer	FALSE	NA	NA	NA
Bc_ctg1_1921	B1	4.366352309	Bcer	F	Bcer	FALSE	NA	NA	NA
Bc_ctg1_1922	B1	2.183176155	Bcer	J	Bcer	FALSE	NA	NA	NA
Bc_ctg1_1923	B1	3.96941119	Bcer	S	Bcer	FALSE	NA	NA	NA
Bc_ctg1_1924	B1	15.48070364	Bcer	S	Bcer	FALSE	NA	NA	NA

Bc_ctg1_1925	B1	6.450293184	Bcer	NA	Bcer	FALSE	NA	NA	NA
Bc_ctg1_1926	B1	108.2656902	Bcer	G	Bcer	FALSE	NA	NA	NA
Bc_ctg1_1927	B1	15.28223308	Bcer	K	Bcer	FALSE	NA	NA	NA
Bc_ctg1_1928	B1	133.0745102	Bcer	S	Bcer	FALSE	NA	NA	NA
Bc_ctg1_1929	B1	122.2578647	Bcer	G	Bcer	FALSE	NA	NA	NA
Bc_ctg1_193	B1	7.641116542	Bcer	NA	Bcer	FALSE	NA	NA	NA
Bc_ctg1_1930	B1	39.29717079	Bcer	S	Bcer	FALSE	NA	NA	NA
Bc_ctg1_1931	B1	0.09923528	Bcer	S	Bcer	FALSE	NA	NA	NA
Bc_ctg1_1932	B1	17.36617396	Bcer	S	Bcer	FALSE	NA	NA	NA
Bc_ctg1_1933	B1	0.09923528	Bcer	S	Bcer	FALSE	NA	NA	NA
Bc_ctg1_1934	B1	4.26711703	Bcer	K	Bcer	FALSE	NA	NA	NA
Bc_ctg1_1935	B1	72.34251895	Bcer	S	Bcer	FALSE	NA	NA	NA
Bc_ctg1_1936	B1	0.595411679	Bcer	NA	Bcer	FALSE	NA	NA	NA
Bc_ctg1_1937	B1	28.08358417	Bcer	G	Bcer	FALSE	NA	NA	NA
Bc_ctg1_1938	B1	11.61052773	Bcer	S	Bcer	FALSE	NA	NA	NA
Bc_ctg1_1939	B1	668.9450209	Bcer	H	Bcer	FALSE	NA	NA	NA
Bc_ctg1_194	B1	79.68592965	Bcer	I	Bcer	FALSE	NA	NA	NA
Bc_ctg1_1940	B1	0.09923528	Bcer	M	Bcer	FALSE	NA	NA	NA
Bc_ctg1_1941	B1	3.671705351	Bcer	S	Bcer	FALSE	NA	NA	NA
Bc_ctg1_1942	B1	8.038057661	Bcer	S	Bcer	FALSE	NA	NA	NA
Bc_ctg1_1943	B1	57.25875642	Bcer	F	Bcer	FALSE	NA	NA	NA
Bc_ctg1_1944	B1	1738.105925	Bcer	O	Bcer	FALSE	NA	NA	NA
Bc_ctg1_1945	B1	267.6375495	Bcer	C	Bcer	FALSE	NA	NA	NA
Bc_ctg1_1946	B1	905.5219278	Bcer	E	Bcer	FALSE	NA	NA	NA
Bc_ctg1_1947	B1	862.8507575	Bcer	O	Bcer	FALSE	NA	NA	NA
Bc_ctg1_1948	B1	398.7273541	Bcer	O	Bcer	FALSE	NA	NA	NA
Bc_ctg1_1949	B1	15.38146836	Bcer	P	Bcer	FALSE	NA	NA	NA
Bc_ctg1_195	B1	15.6791742	Bcer	K	Bcer	FALSE	NA	NA	NA
Bc_ctg1_1950	B1	122.4563352	Bcer	P	Bcer	FALSE	NA	NA	NA
Bc_ctg1_1951	B1	22.32793795	Bcer	P	Bcer	FALSE	NA	NA	NA
Bc_ctg1_1952	B1	28.57976057	Bcer	P	Bcer	FALSE	NA	NA	NA
Bc_ctg1_1953	B1	0.793882238	Bcer	NA	Bcer	FALSE	NA	NA	NA
Bc_ctg1_1954	B1	60.63275593	Bcer	CO	Bcer	FALSE	NA	NA	NA
Bc_ctg1_1955	B1	4.763293429	Bcer	L	Bcer	FALSE	NA	NA	NA
Bc_ctg1_1956	B1	1339.676277	Bcer	E	Bcer	FALSE	NA	NA	NA
Bc_ctg1_1957	B1	387.3152969	Bcer	P	Bcer	FALSE	NA	NA	NA
Bc_ctg1_1958	B1	2.083940875	Bcer	NA	Bcer	FALSE	NA	NA	NA
Bc_ctg1_1959	B1	20.64093819	Bcer	S	Bcer	FALSE	NA	NA	NA
Bc_ctg1_196	B1	31.95376008	Bcer	EGP	Bcer	FALSE	NA	NA	NA
Bc_ctg1_1960	B1	0.396941119	Bcer	NA	Bcer	FALSE	NA	NA	NA
Bc_ctg1_1961	B1	33.04534816	Bcer	S	Bcer	FALSE	NA	NA	NA
Bc_ctg1_1962	B1	32.3507012	Bcer	NA	Bcer	FALSE	NA	NA	NA
Bc_ctg1_1963	B1	14.88529196	Bcer	I	Bcer	FALSE	NA	NA	NA
Bc_ctg1_1964	B1	2897.273228	Bcer	S	Bcer	FALSE	NA	NA	NA
Bc_ctg1_1965	B1	4988.359043	Bcer	C	Bcer	FALSE	NA	NA	NA
Bc_ctg1_1966	B1	0.19847056	Bcer	M	Bcer	FALSE	NA	NA	NA
Bc_ctg1_1967	B1	0	Bcer	NA	Bcer	FALSE	NA	NA	NA
Bc_ctg1_1968	B1	11.80899829	Bcer	S	Bcer	FALSE	NA	NA	NA
Bc_ctg1_1969	B1	0.09923528	Bcer	NA	Bcer	FALSE	NA	NA	NA
Bc_ctg1_197	B1	124.6395114	Bcer	V	Bcer	FALSE	NA	NA	NA
Bc_ctg1_1970	B1	3.671705351	Bcer	K	Bcer	FALSE	NA	NA	NA
Bc_ctg1_1971	B1	6.847234304	Bcer	S	Bcer	FALSE	NA	NA	NA
Bc_ctg1_1972	B1	907.8043393	Bcer	I	Bcer	FALSE	NA	NA	NA
Bc_ctg1_1973	B1	302.568368	Bcer	I	Bcer	FALSE	NA	NA	NA
Bc_ctg1_1974	B1	915.4454558	Bcer	I	Bcer	FALSE	NA	NA	NA
Bc_ctg1_1975	B1	0.09923528	Bcer	S	Bcer	FALSE	NA	NA	NA
Bc_ctg1_1976	B1	26.69429026	Bcer	M	Bcer	FALSE	NA	NA	NA
Bc_ctg1_1977	B1	1839.028205	Bcer	E	Bcer	FALSE	NA	NA	NA
Bc_ctg1_1978	B1	0.595411679	Bcer	S	Bcer	FALSE	NA	NA	NA
Bc_ctg1_1979	B1	86.13622283	Bcer	S	Bcer	FALSE	NA	NA	NA
Bc_ctg1_1980	B1	142.5018617	Bcer	NT	Bcer	FALSE	NA	NA	NA
Bc_ctg1_1981	B1	55.77022723	Bcer	K	Bcer	FALSE	NA	NA	NA

Bc_ctg1_1983	B1	3.076293673	Bcer	EGP	Bcer	FALSE	NA	NA	NA
Bc_ctg1_1984	B1	282.3243709	Bcer	E	Bcer	FALSE	NA	NA	NA
Bc_ctg1_1985	B1	10.22123382	Bcer	NA	Bcer	FALSE	NA	NA	NA
Bc_ctg1_1986	B1	1.686999756	Bcer	NA	Bcer	FALSE	NA	NA	NA
Bc_ctg1_1987	B1	220.7984975	Bcer	U	Bcer	FALSE	NA	NA	NA
Bc_ctg1_1988	B1	59.14422674	Bcer	NA	Bcer	FALSE	NA	NA	NA
Bc_ctg1_1989	B1	133.7691571	Bcer	S	Bcer	FALSE	NA	NA	NA
Bc_ctg1_199	B1	14.6868214	Bcer	V	Bcer	FALSE	NA	NA	NA
Bc_ctg1_1990	B1	29.57211337	Bcer	J	Bcer	FALSE	NA	NA	NA
Bc_ctg1_1991	B1	44.75511117	Bcer	S	Bcer	FALSE	NA	NA	NA
Bc_ctg1_1992	B1	2.977058393	Bcer	NA	Bcer	FALSE	NA	NA	NA
Bc_ctg1_1993	B1	30.06828977	Bcer	S	Bcer	FALSE	NA	NA	NA
Bc_ctg1_1994	B1	11.80899829	Bcer	S	Bcer	FALSE	NA	NA	NA
Bc_ctg1_1995	B1	0.694646958	Bcer	K	Bcer	FALSE	NA	NA	NA
Bc_ctg1_1996	B1	53.48781579	Bcer	S	Bcer	FALSE	NA	NA	NA
Bc_ctg1_1997	B1	0.694646958	Bcer	S	Bcer	FALSE	NA	NA	NA
Bc_ctg1_1998	B1	0.396941119	Bcer	O	Bcer	FALSE	NA	NA	NA
Bc_ctg1_1999	B1	159.7688004	Bcer	T	Bcer	FALSE	NA	NA	NA
Bc_ctg1_2	B1	3.96941119	Bcer	S	Bcer	FALSE	NA	NA	NA
Bc_ctg1_20	B1	2.778587833	Bcer	S	Bcer	FALSE	NA	NA	NA
Bc_ctg1_200	B1	0	Bcer	NA	Bcer	FALSE	NA	NA	NA
Bc_ctg1_2000	B1	353.6745371	Bcer	T	Bcer	FALSE	NA	NA	NA
Bc_ctg1_2001	B1	7.938822381	Bcer	EG	Bcer	FALSE	NA	NA	NA
Bc_ctg1_2002	B1	4.06864647	Bcer	NA	Bcer	FALSE	NA	NA	NA
Bc_ctg1_2003	B1	4.16788175	Bcer	J	Bcer	FALSE	NA	NA	NA
Bc_ctg1_2004	B1	3.572470071	Bcer	H	Bcer	FALSE	NA	NA	NA
Bc_ctg1_2005	B1	1014.879206	Bcer	NT	Bcer	FALSE	NA	NA	NA
Bc_ctg1_2006	B1	160.9596238	Bcer	NT	Bcer	FALSE	NA	NA	NA
Bc_ctg1_2007	B1	27.68664305	Bcer	S	Bcer	FALSE	NA	NA	NA
Bc_ctg1_2008	B1	10.81664549	Bcer	E	Bcer	FALSE	NA	NA	NA
Bc_ctg1_2009	B1	59.73963842	Bcer	S	Bcer	FALSE	NA	NA	NA
Bc_ctg1_201	B1	0.496176399	Bcer	I	Bcer	FALSE	NA	NA	NA
Bc_ctg1_2010	B1	41.28187638	Bcer	K	Bcer	FALSE	NA	NA	NA
Bc_ctg1_2011	B1	0.09923528	Bcer	S	Bcer	FALSE	NA	NA	NA
Bc_ctg1_2012	B1	0.595411679	Bcer	S	Bcer	FALSE	NA	NA	NA
Bc_ctg1_2013	B1	3.870175911	Bcer	P	Bcer	FALSE	NA	NA	NA
Bc_ctg1_2014	B1	1.786235036	Bcer	P	Bcer	FALSE	NA	NA	NA
Bc_ctg1_2015	B1	77.80045933	Bcer	E	Bcer	FALSE	NA	NA	NA
Bc_ctg1_2016	B1	2422.035473	Bcer	P	Bcer	FALSE	NA	NA	NA
Bc_ctg1_2017	B1	2.183176155	Bcer	NA	Bcer	FALSE	NA	NA	NA
Bc_ctg1_2018	B1	0.793882238	Bcer	NA	Bcer	FALSE	NA	NA	NA
Bc_ctg1_2019	B1	7.641116542	Bcer	NA	Bcer	FALSE	NA	NA	NA
Bc_ctg1_202	B1	2.183176155	Bcer	NA	Bcer	FALSE	NA	NA	NA
Bc_ctg1_2020	B1	84.15151724	Bcer	S	Bcer	FALSE	NA	NA	NA
Bc_ctg1_2021	B1	89.21251651	Bcer	S	Bcer	FALSE	NA	NA	NA
Bc_ctg1_2022	B1	216.3329099	Bcer	P	Bcer	FALSE	NA	NA	NA
Bc_ctg1_2023	B1	5.656410946	Bcer	P	Bcer	FALSE	NA	NA	NA
Bc_ctg1_2024	B1	2.083940875	Bcer	P	Bcer	FALSE	NA	NA	NA
Bc_ctg1_2025	B1	860.3698755	Bcer	F	Bcer	FALSE	NA	NA	NA
Bc_ctg1_2026	B1	4.763293429	Bcer	F	Bcer	FALSE	NA	NA	NA
Bc_ctg1_2027	B1	197.4782067	Bcer	E	Bcer	FALSE	NA	NA	NA
Bc_ctg1_2028	B1	176.5395627	Bcer	E	Bcer	FALSE	NA	NA	NA
Bc_ctg1_2029	B1	6.549528464	Bcer	S	Bcer	FALSE	NA	NA	NA
Bc_ctg1_203	B1	1.290058637	Bcer	S	Bcer	FALSE	NA	NA	NA
Bc_ctg1_2030	B1	22.42717323	Bcer	G	Bcer	FALSE	NA	NA	NA
Bc_ctg1_2031	B1	0.496176399	Bcer	M	Bcer	FALSE	NA	NA	NA
Bc_ctg1_2032	B1	17.76311508	Bcer	S	Bcer	FALSE	NA	NA	NA
Bc_ctg1_2033	B1	13.39676277	Bcer	EGP	Bcer	FALSE	NA	NA	NA
Bc_ctg1_2034	B1	11.90823357	Bcer	K	Bcer	FALSE	NA	NA	NA
Bc_ctg1_2035	B1	211.768087	Bcer	S	Bcer	FALSE	NA	NA	NA
Bc_ctg1_2036	B1	573.7783876	Bcer	C	Bcer	FALSE	NA	NA	NA
Bc_ctg1_2037	B1	84.25075252	Bcer	J	Bcer	FALSE	NA	NA	NA

Bc_ctg1_2038	B1	173.3640337	Bcer	M	Bcer	FALSE	NA	NA	NA
Bc_ctg1_2039	B1	164.929035	Bcer	S	Bcer	FALSE	NA	NA	NA
Bc_ctg1_204	B1	1.488529196	Bcer	S	Bcer	FALSE	NA	NA	NA
Bc_ctg1_2040	B1	50.11381628	Bcer	NT	Bcer	FALSE	NA	NA	NA
Bc_ctg1_2041	B1	20.44246763	Bcer	L	Bcer	FALSE	NA	NA	NA
Bc_ctg1_2042	B1	0.19847056	Bcer	D	Bcer	FALSE	NA	NA	NA
Bc_ctg1_2043	B1	0.595411679	Bcer	D	Bcer	FALSE	NA	NA	NA
Bc_ctg1_2044	B1	3.076293673	Bcer	NA	Bcer	FALSE	NA	NA	NA
Bc_ctg1_2045	B1	1.786235036	Bcer	NA	Bcer	FALSE	NA	NA	NA
Bc_ctg1_2046	B1	2.381646714	Bcer	EGP	Bcer	FALSE	NA	NA	NA
Bc_ctg1_2047	B1	0	Bcer	S	Bcer	FALSE	NA	NA	NA
Bc_ctg1_2048	B1	5.954116786	Bcer	J	Bcer	FALSE	NA	NA	NA
Bc_ctg1_2049	B1	21.73252627	Bcer	S	Bcer	FALSE	NA	NA	NA
Bc_ctg1_205	B1	1.389293917	Bcer	NA	Bcer	FALSE	NA	NA	NA
Bc_ctg1_2050	B1	1.190823357	Bcer	S	Bcer	FALSE	NA	NA	NA
Bc_ctg1_2051	B1	0	Bcer	Q	Bcer	TRUE	NA	NA	NA
Bc_ctg1_2052	B1	4.862528708	Bcer	K	Bcer	FALSE	NA	NA	NA
Bc_ctg1_2053	B1	0.09923528	Bcer	S	Bcer	FALSE	NA	NA	NA
Bc_ctg1_2054	B1	8.534234059	Bcer	K	Bcer	FALSE	NA	NA	NA
Bc_ctg1_2055	B1	2.083940875	Bcer	P	Bcer	FALSE	NA	NA	NA
Bc_ctg1_2056	B1	0.694646958	Bcer	P	Bcer	FALSE	NA	NA	NA
Bc_ctg1_2057	B1	0.19847056	Bcer	F	Bcer	FALSE	NA	NA	NA
Bc_ctg1_2058	B1	1293.631107	Bcer	M	Bcer	FALSE	NA	NA	NA
Bc_ctg1_2059	B1	39.09870023	Bcer	P	Bcer	FALSE	NA	NA	NA
Bc_ctg1_206	B1	0	Bcer	NA	Bcer	FALSE	NA	NA	NA
Bc_ctg1_2060	B1	33.44228928	Bcer	P	Bcer	FALSE	NA	NA	NA
Bc_ctg1_2061	B1	32.74764232	Bcer	P	Bcer	FALSE	NA	NA	NA
Bc_ctg1_2062	B1	69.06775471	Bcer	P	Bcer	FALSE	NA	NA	NA
Bc_ctg1_2063	B1	2.580117274	Bcer	T	Bcer	FALSE	NA	NA	NA
Bc_ctg1_2064	B1	20.34323235	Bcer	S	Bcer	FALSE	NA	NA	NA
Bc_ctg1_2065	B1	26.39658442	Bcer	O	Bcer	FALSE	NA	NA	NA
Bc_ctg1_2066	B1	379.0787687	Bcer	K	Bcer	FALSE	NA	NA	NA
Bc_ctg1_2067	B1	146.2728024	Bcer	S	Bcer	FALSE	NA	NA	NA
Bc_ctg1_2068	B1	9.328116298	Bcer	U	Bcer	FALSE	NA	NA	NA
Bc_ctg1_2069	B1	66.98381384	Bcer	M	Bcer	FALSE	NA	NA	NA
Bc_ctg1_207	B1	125.2349231	Bcer	S	Bcer	FALSE	NA	NA	NA
Bc_ctg1_2070	B1	47.13675789	Bcer	S	Bcer	FALSE	NA	NA	NA
Bc_ctg1_2071	B1	0	Bcer	F	Bcer	FALSE	NA	NA	NA
Bc_ctg1_2072	B1	0	Bcer	F	Bcer	FALSE	NA	NA	NA
Bc_ctg1_2073	B1	6395.118369	Bcer	G	Bcer	FALSE	NA	NA	NA
Bc_ctg1_2074	B1	5898.247323	Bcer	G	Bcer	FALSE	NA	NA	NA
Bc_ctg1_2075	B1	2357.1356	Bcer	G	Bcer	FALSE	NA	NA	NA
Bc_ctg1_2076	B1	3452.296148	Bcer	F	Bcer	FALSE	NA	NA	NA
Bc_ctg1_2077	B1	7484.424035	Bcer	G	Bcer	FALSE	NA	NA	NA
Bc_ctg1_2078	B1	2854.205116	Bcer	K	Bcer	FALSE	NA	NA	NA
Bc_ctg1_2079	B1	48.82375764	Bcer	O	Bcer	FALSE	NA	NA	NA
Bc_ctg1_208	B1	8.43499878	Bcer	S	Bcer	FALSE	NA	NA	NA
Bc_ctg1_2080	B1	388.406885	Bcer	K	Bcer	FALSE	NA	NA	NA
Bc_ctg1_2081	B1	0	Bcer	NA	Bcer	FALSE	NA	NA	NA
Bc_ctg1_2082	B1	3.770940631	Bcer	CP	Bcer	FALSE	NA	NA	NA
Bc_ctg1_2083	B1	1.786235036	Bcer	S	Bcer	FALSE	NA	NA	NA
Bc_ctg1_2084	B1	7.740351821	Bcer	T	Bcer	FALSE	NA	NA	NA
Bc_ctg1_2085	B1	51.20540436	Bcer	K	Bcer	FALSE	NA	NA	NA
Bc_ctg1_2087	B1	0	Bcer	NA	Bcer	FALSE	NA	NA	NA
Bc_ctg1_2088	B1	0	Bcer	S	Bcer	FALSE	NA	NA	NA
Bc_ctg1_2089	B1	0.09923528	Bcer	I	Bcer	FALSE	NA	NA	NA
Bc_ctg1_209	B1	14.19064501	Bcer	P	Bcer	FALSE	NA	NA	NA
Bc_ctg1_2090	B1	0	Bcer	S	Bcer	FALSE	NA	NA	NA
Bc_ctg1_2091	B1	0	Bcer	S	Bcer	FALSE	NA	NA	NA
Bc_ctg1_2092	B1	0.694646958	Bcer	S	Bcer	FALSE	NA	NA	NA
Bc_ctg1_2093	B1	679.0670194	Bcer	OU	Bcer	FALSE	NA	NA	NA
Bc_ctg1_2094	B1	16.07611532	Bcer	G	Bcer	FALSE	NA	NA	NA

Bc_ctg1_2095	B1	160.3642121	Bcer	K	Bcer	FALSE	NA	NA	NA
Bc_ctg1_2096	B1	305.1484853	Bcer	S	Bcer	FALSE	NA	NA	NA
Bc_ctg1_2097	B1	244.5157293	Bcer	S	Bcer	FALSE	NA	NA	NA
Bc_ctg1_2098	B1	84.44922308	Bcer	F	Bcer	FALSE	NA	NA	NA
Bc_ctg1_2099	B1	7.144940143	Bcer	S	Bcer	FALSE	NA	NA	NA
Bc_ctg1_21	B1	1.290058637	Bcer	S	Bcer	FALSE	NA	NA	NA
Bc_ctg1_210	B1	5.457940387	Bcer	P	Bcer	FALSE	NA	NA	NA
Bc_ctg1_2100	B1	259.6987271	Bcer	C	Bcer	FALSE	NA	NA	NA
Bc_ctg1_2101	B1	208.0963817	Bcer	S	Bcer	FALSE	NA	NA	NA
Bc_ctg1_2102	B1	21.53405571	Bcer	S	Bcer	FALSE	NA	NA	NA
Bc_ctg1_2103	B1	21.03787931	Bcer	S	Bcer	FALSE	NA	NA	NA
Bc_ctg1_2104	B1	364.8881237	Bcer	M	Bcer	FALSE	NA	NA	NA
Bc_ctg1_2105	B1	256.820904	Bcer	F	Bcer	FALSE	NA	NA	NA
Bc_ctg1_2106	B1	53.28934523	Bcer	S	Bcer	FALSE	NA	NA	NA
Bc_ctg1_2107	B1	333.6290106	Bcer	L	Bcer	FALSE	NA	NA	NA
Bc_ctg1_2108	B1	174.157916	Bcer	L	Bcer	FALSE	NA	NA	NA
Bc_ctg1_2109	B1	1.389293917	Bcer	S	Bcer	FALSE	NA	NA	NA
Bc_ctg1_211	B1	8.931175179	Bcer	I	Bcer	FALSE	NA	NA	NA
Bc_ctg1_2110	B1	12.00746885	Bcer	NA	Bcer	FALSE	NA	NA	NA
Bc_ctg1_2111	B1	6.450293184	Bcer	K	Bcer	FALSE	NA	NA	NA
Bc_ctg1_2112	B1	9.824292696	Bcer	NA	Bcer	FALSE	NA	NA	NA
Bc_ctg1_2113	B1	10.12199854	Bcer	NA	Bcer	FALSE	NA	NA	NA
Bc_ctg1_2114	B1	4.664058149	Bcer	NA	Bcer	FALSE	NA	NA	NA
Bc_ctg1_2115	B1	0.496176399	Bcer	K	Bcer	FALSE	NA	NA	NA
Bc_ctg1_2116	B1	191.9210311	Bcer	S	Bcer	FALSE	NA	NA	NA
Bc_ctg1_2117	B1	3.473234792	Bcer	K	Bcer	FALSE	NA	NA	NA
Bc_ctg1_2118	B1	4.664058149	Bcer	K	Bcer	FALSE	NA	NA	NA
Bc_ctg1_2119	B1	11.41205717	Bcer	S	Bcer	FALSE	NA	NA	NA
Bc_ctg1_212	B1	349.8043612	Bcer	I	Bcer	FALSE	NA	NA	NA
Bc_ctg1_2120	B1	9.725057417	Bcer	L	Bcer	FALSE	NA	NA	NA
Bc_ctg1_2121	B1	0.893117518	Bcer	S	Bcer	FALSE	NA	NA	NA
Bc_ctg1_2122	B1	11.21358661	Bcer	V	Bcer	FALSE	NA	NA	NA
Bc_ctg1_2123	B1	166.71527	Bcer	O	Bcer	FALSE	NA	NA	NA
Bc_ctg1_2124	B1	113.7236306	Bcer	M	Bcer	FALSE	NA	NA	NA
Bc_ctg1_2125	B1	365.0865942	Bcer	D	Bcer	FALSE	NA	NA	NA
Bc_ctg1_2126	B1	97.34980945	Bcer	D	Bcer	FALSE	NA	NA	NA
Bc_ctg1_2127	B1	72.83869534	Bcer	C	Bcer	FALSE	NA	NA	NA
Bc_ctg1_2128	B1	244.9126705	Bcer	J	Bcer	FALSE	NA	NA	NA
Bc_ctg1_2129	B1	1445.36185	Bcer	U	Bcer	FALSE	NA	NA	NA
Bc_ctg1_213	B1	3.473234792	Bcer	H	Bcer	FALSE	NA	NA	NA
Bc_ctg1_2130	B1	4.862528708	Bcer	NA	Bcer	FALSE	NA	NA	NA
Bc_ctg1_2131	B1	3.274764232	Bcer	V	Bcer	FALSE	NA	NA	NA
Bc_ctg1_2132	B1	0.992352798	Bcer	NA	Bcer	FALSE	NA	NA	NA
Bc_ctg1_2133	B1	0.893117518	Bcer	NA	Bcer	FALSE	NA	NA	NA
Bc_ctg1_2134	B1	0.893117518	Bcer	NA	Bcer	FALSE	NA	NA	NA
Bc_ctg1_2135	B1	0.496176399	Bcer	KT	Bcer	FALSE	Bc_Ga0417192_01_rn14	Other	NA
Bc_ctg1_2136	B1	3.870175911	Bcer	NA	Bcer	FALSE	Bc_Ga0417192_01_rn14	Other	NA
Bc_ctg1_2137	B1	7398.585518	Bcer	J	Bcer	FALSE	Bc_Ga0417192_01_rn14	Other	NA
Bc_ctg1_2138	B1	0.19847056	Bcer	K	Bcer	FALSE	Bc_Ga0417192_01_rn14	Regulation	SMCOG1255
Bc_ctg1_2139	B1	1.091588077	Bcer	S	Bcer	FALSE	Bc_Ga0417192_01_rn14	Other	NA
Bc_ctg1_214	B1	0.09923528	Bcer	S	Bcer	FALSE	NA	NA	NA
Bc_ctg1_2140	B1	2.877823113	Bcer	L	Bcer	FALSE	Bc_Ga0417192_01_rn14	Other	NA
Bc_ctg1_2141	B1	842.3090546	Bcer	M	Bcer	FALSE	Bc_Ga0417192_01_rn14	Other	NA
Bc_ctg1_2142	B1	268.3321965	Bcer	K	Bcer	FALSE	Bc_Ga0417192_01_rn14	Regulation	SMCOG1167
Bc_ctg1_2143	B1	44.95358173	Bcer	S	Bcer	FALSE	Bc_Ga0417192_01_rn14	Other	NA
Bc_ctg1_2144	B1	13.29752749	Bcer	S	Bcer	FALSE	Bc_Ga0417192_01_rn14	Other	NA
Bc_ctg1_2145	B1	63.60981433	Bcer	K	Bcer	FALSE	Bc_Ga0417192_01_rn14	Other	NA
Bc_ctg1_2146	B1	647.8079063	Bcer	M	Bcer	FALSE	Bc_Ga0417192_01_rn14	Core biosynthesis	None
Bc_ctg1_2147	B1	4.664058149	Bcer	M	Bcer	FALSE	Bc_Ga0417192_01_rn14	Ancillary biosynthesis	SMCOG1152
Bc_ctg1_2148	B1	499.7488689	Bcer	M	Bcer	FALSE	Bc_Ga0417192_01_rn14	Core biosynthesis	None
Bc_ctg1_2149	B1	3.671705351	Bcer	G	Bcer	FALSE	Bc_Ga0417192_01_rn14	Ancillary biosynthesis	SMCOG1235
Bc_ctg1_215	B1	33.04534816	Bcer	L	Bcer	FALSE	NA	NA	NA

Bc_ctg1_2150	B1	111.3419839	Bcer	K	Bcer	FALSE	Bc_Ga0417192_01_rn14	Regulation	SMCOG1112
Bc_ctg1_2151	B1	0	Bcer	G	Bcer	FALSE	Bc_Ga0417192_01_rn14	Other	NA
Bc_ctg1_2152	B1	9.228881018	Bcer	P	Bcer	FALSE	Bc_Ga0417192_01_rn14	Other	NA
Bc_ctg1_2153	B1	10.02276326	Bcer	G	Bcer	FALSE	Bc_Ga0417192_01_rn14	Other	NA
Bc_ctg1_2154	B1	225.5617909	Bcer	G	Bcer	FALSE	Bc_Ga0417192_01_rn14	Other	NA
Bc_ctg1_2155	B1	19.15240899	Bcer	G	Bcer	FALSE	Bc_Ga0417192_01_rn14	Other	NA
Bc_ctg1_2156	B1	409.1470585	Bcer	G	Bcer	FALSE	Bc_Ga0417192_01_rn14	Other	NA
Bc_ctg1_2157	B1	68.77004887	Bcer	G	Bcer	FALSE	Bc_Ga0417192_01_rn14	Other	NA
Bc_ctg1_2158	B1	0.396941119	Bcer	E	Bcer	FALSE	NA	NA	NA
Bc_ctg1_2159	B1	0	Bcer	E	Bcer	FALSE	NA	NA	NA
Bc_ctg1_216	B1	1.686999756	Bcer	NA	Bcer	FALSE	NA	NA	NA
Bc_ctg1_2160	B1	0.09923528	Bcer	G	Bcer	FALSE	NA	NA	NA
Bc_ctg1_2161	B1	9.824292696	Bcer	G	Bcer	FALSE	NA	NA	NA
Bc_ctg1_2162	B1	0	Bcer	G	Bcer	FALSE	NA	NA	NA
Bc_ctg1_2163	B1	5.259469827	Bcer	C	Bcer	FALSE	NA	NA	NA
Bc_ctg1_2164	B1	88.41863427	Bcer	I	Bcer	FALSE	NA	NA	NA
Bc_ctg1_2165	B1	31.45758368	Bcer	E	Bcer	FALSE	NA	NA	NA
Bc_ctg1_2166	B1	1.587764476	Bcer	S	Bcer	FALSE	NA	NA	NA
Bc_ctg1_2167	B1	17.56464452	Bcer	K	Bcer	FALSE	NA	NA	NA
Bc_ctg1_2168	B1	17.06846812	Bcer	G	Bcer	FALSE	NA	NA	NA
Bc_ctg1_2169	B1	0.09923528	Bcer	NA	Bcer	FALSE	NA	NA	NA
Bc_ctg1_217	B1	102.4108087	Bcer	I	Bcer	FALSE	NA	NA	NA
Bc_ctg1_2170	B1	0.496176399	Bcer	C	Bcer	FALSE	NA	NA	NA
Bc_ctg1_2171	B1	1.587764476	Bcer	C	Bcer	FALSE	NA	NA	NA
Bc_ctg1_2172	B1	0.09923528	Bcer	P	Bcer	FALSE	NA	NA	NA
Bc_ctg1_2173	B1	78.49510629	Bcer	H	Bcer	FALSE	NA	NA	NA
Bc_ctg1_2174	B1	39.99181774	Bcer	NA	Bcer	FALSE	NA	NA	NA
Bc_ctg1_2175	B1	0	Bcer	L	Bcer	FALSE	NA	NA	NA
Bc_ctg1_2177	B1	115.0136892	Bcer	C	Bcer	FALSE	NA	NA	NA
Bc_ctg1_2178	B1	12.00746885	Bcer	S	Bcer	FALSE	NA	NA	NA
Bc_ctg1_2179	B1	53.98399219	Bcer	S	Bcer	FALSE	NA	NA	NA
Bc_ctg1_218	B1	0.396941119	Bcer	NA	Bcer	FALSE	NA	NA	NA
Bc_ctg1_2180	B1	47.13675789	Bcer	S	Bcer	FALSE	NA	NA	NA
Bc_ctg1_2181	B1	548.1756854	Bcer	M	Bcer	FALSE	NA	NA	NA
Bc_ctg1_2182	B1	46.14440509	Bcer	E	Bcer	FALSE	NA	NA	NA
Bc_ctg1_2184	B1	2597.582683	Bcer	MT	Bcer	FALSE	NA	NA	NA
Bc_ctg1_2185	B1	4.862528708	Bcer	F	Bcer	FALSE	NA	NA	NA
Bc_ctg1_2186	B1	25.60270218	Bcer	T	Bcer	FALSE	NA	NA	NA
Bc_ctg1_2187	B1	37.51093575	Bcer	M	Bcer	FALSE	NA	NA	NA
Bc_ctg1_2188	B1	0.595411679	Bcer	NA	Bcer	FALSE	NA	NA	NA
Bc_ctg1_2189	B1	25.5034669	Bcer	M	Bcer	FALSE	NA	NA	NA
Bc_ctg1_219	B1	33.64075984	Bcer	S	Bcer	FALSE	NA	NA	NA
Bc_ctg1_2190	B1	132.1813926	Bcer	T	Bcer	FALSE	NA	NA	NA
Bc_ctg1_2191	B1	47.53369901	Bcer	S	Bcer	FALSE	NA	NA	NA
Bc_ctg1_2192	B1	4.16788175	Bcer	S	Bcer	FALSE	NA	NA	NA
Bc_ctg1_2193	B1	6.549528464	Bcer	NA	Bcer	FALSE	NA	NA	NA
Bc_ctg1_2194	B1	0	Bcer	S	Bcer	FALSE	NA	NA	NA
Bc_ctg1_2195	B1	69.26622527	Bcer	S	Bcer	FALSE	NA	NA	NA
Bc_ctg1_2196	B1	189.9363255	Bcer	L	Bcer	FALSE	NA	NA	NA
Bc_ctg1_2197	B1	47.53369901	Bcer	S	Bcer	FALSE	NA	NA	NA
Bc_ctg1_2198	B1	0	Bcer	S	Bcer	FALSE	NA	NA	NA
Bc_ctg1_2199	B1	0	Bcer	S	Bcer	FALSE	NA	NA	NA
Bc_ctg1_22	B1	1.488529196	Bcer	S	Bcer	FALSE	NA	NA	NA
Bc_ctg1_220	B1	1.686999756	Bcer	J	Bcer	FALSE	NA	NA	NA
Bc_ctg1_2200	B1	7.938822381	Bcer	S	Bcer	FALSE	NA	NA	NA
Bc_ctg1_2201	B1	0.694646958	Bcer	K	Bcer	FALSE	NA	NA	NA
Bc_ctg1_2202	B1	0	Bcer	K	Bcer	FALSE	NA	NA	NA
Bc_ctg1_2203	B1	69.66316639	Bcer	S	Bcer	FALSE	NA	NA	NA
Bc_ctg1_2204	B1	159.1733887	Bcer	V	Bcer	FALSE	Bc_Ga0417192_01_rn15	Other	NA
Bc_ctg1_2205	B1	33.343054	Bcer	V	Bcer	FALSE	Bc_Ga0417192_01_rn15	Transport	SMCOG1000
Bc_ctg1_2206	B1	32.25146592	Bcer	M	Bcer	FALSE	Bc_Ga0417192_01_rn15	Transport	SMCOG1029
Bc_ctg1_2207	B1	0.496176399	Bcer	K	Bcer	FALSE	Bc_Ga0417192_01_rn15	Other	NA

Bc_ctg1_2208	B1	8.732704619	Bcer	S	Bcer	FALSE	Bc_Ga0417192_01_rn15	Other	NA
Bc_ctg1_2209	B1	21.73252627	Bcer	G	Bcer	FALSE	Bc_Ga0417192_01_rn15	Transport	SMCOG1106
Bc_ctg1_221	B1	0.09923528	Bcer	NA	Bcer	FALSE	NA	NA	NA
Bc_ctg1_2210	B1	22.22870267	Bcer	P	Bcer	FALSE	Bc_Ga0417192_01_rn15	Other	NA
Bc_ctg1_2211	B1	30.86217201	Bcer	S	Bcer	FALSE	Bc_Ga0417192_01_rn15	Other	NA
Bc_ctg1_2212	B1	28.18281945	Bcer	T	Bcer	FALSE	Bc_Ga0417192_01_rn15	Regulation	SMCOG1003
Bc_ctg1_2213	B1	8.831939899	Bcer	K	Bcer	FALSE	Bc_Ga0417192_01_rn15	Regulation	SMCOG1008
Bc_ctg1_2214	B1	24.0149377	Bcer	M	Bcer	FALSE	Bc_Ga0417192_01_rn15	Core biosynthesis	SMCOG1010
Bc_ctg1_2215	B1	63.70904961	Bcer	E	Bcer	FALSE	Bc_Ga0417192_01_rn15	Other	NA
Bc_ctg1_2216	B1	230.4243196	Bcer	K	Bcer	FALSE	Bc_Ga0417192_01_rn15	Other	NA
Bc_ctg1_2217	B1	180.8066797	Bcer	M	Bcer	FALSE	Bc_Ga0417192_01_rn15	Core biosynthesis	SMCOG1010
Bc_ctg1_2218	B1	224.7679087	Bcer	M	Bcer	FALSE	Bc_Ga0417192_01_rn15	Ancillary biosynthesis	SMCOG1152
Bc_ctg1_2219	B1	144.3873321	Bcer	S	Bcer	FALSE	Bc_Ga0417192_01_rn15	Core biosynthesis	None
Bc_ctg1_222	B1	2.877823113	Bcer	K	Bcer	FALSE	NA	NA	NA
Bc_ctg1_2220	B1	147.7613316	Bcer	M	Bcer	FALSE	Bc_Ga0417192_01_rn15	Other	NA
Bc_ctg1_2221	B1	83.05992916	Bcer	M	Bcer	FALSE	Bc_Ga0417192_01_rn15	Other	NA
Bc_ctg1_2222	B1	72.04481311	Bcer	M	Bcer	FALSE	Bc_Ga0417192_01_rn15	Core biosynthesis	None
Bc_ctg1_2223	B1	87.32704619	Bcer	M	Bcer	FALSE	Bc_Ga0417192_01_rn15	Core biosynthesis	SMCOG1045
Bc_ctg1_2224	B1	60.23581482	Bcer	M	Bcer	FALSE	Bc_Ga0417192_01_rn15	Core biosynthesis	SMCOG1045
Bc_ctg1_2225	B1	7.740351821	Bcer	S	Bcer	FALSE	Bc_Ga0417192_01_rn15	Other	NA
Bc_ctg1_2226	B1	18.45776204	Bcer	M	Bcer	FALSE	Bc_Ga0417192_01_rn15	Core biosynthesis	None
Bc_ctg1_2227	B1	95.76204497	Bcer	M	Bcer	FALSE	Bc_Ga0417192_01_rn15	Core biosynthesis	SMCOG1146
Bc_ctg1_2228	B1	47.03752261	Bcer	E	Bcer	FALSE	Bc_Ga0417192_01_rn15	Core biosynthesis	SMCOG1056
Bc_ctg1_2229	B1	26.79352554	Bcer	GM	Bcer	FALSE	Bc_Ga0417192_01_rn15	Core biosynthesis	SMCOG1010
Bc_ctg1_223	B1	3.671705351	Bcer	C	Bcer	FALSE	NA	NA	NA
Bc_ctg1_2230	B1	11.41205717	Bcer	M	Bcer	FALSE	Bc_Ga0417192_01_rn15	Core biosynthesis	SMCOG1064
Bc_ctg1_2231	B1	14.38911557	Bcer	GM	Bcer	FALSE	Bc_Ga0417192_01_rn15	Other	NA
Bc_ctg1_2232	B1	100.2276326	Bcer	D	Bcer	FALSE	Bc_Ga0417192_01_rn15	Ancillary biosynthesis	SMCOG1216
Bc_ctg1_2233	B1	56.96105058	Bcer	M	Bcer	FALSE	Bc_Ga0417192_01_rn15	Ancillary biosynthesis	SMCOG1216
Bc_ctg1_2234	B1	54.38093331	Bcer	D	Bcer	FALSE	Bc_Ga0417192_01_rn15	Ancillary biosynthesis	SMCOG1216
Bc_ctg1_2235	B1	146.7689788	Bcer	I	Bcer	FALSE	Bc_Ga0417192_01_rn15	Ancillary biosynthesis	SMCOG1270
Bc_ctg1_2236	B1	285.9960763	Bcer	D	Bcer	FALSE	Bc_Ga0417192_01_rn15	Other	NA
Bc_ctg1_2237	B1	0.19847056	Bcer	K	Bcer	FALSE	Bc_Ga0417192_01_rn15	Other	NA
Bc_ctg1_2238	B1	1.686999756	Bcer	M	Bcer	FALSE	Bc_Ga0417192_01_rn15	Other	NA
Bc_ctg1_2239	B1	21.53405571	Bcer	V	Bcer	FALSE	Bc_Ga0417192_01_rn15	Other	NA
Bc_ctg1_224	B1	9.427351577	Bcer	S	Bcer	FALSE	NA	NA	NA
Bc_ctg1_2240	B1	2.877823113	Bcer	V	Bcer	FALSE	Bc_Ga0417192_01_rn15	Transport	SMCOG1000
Bc_ctg1_2241	B1	13.89293917	Bcer	KTV	Bcer	FALSE	NA	NA	NA
Bc_ctg1_2242	B1	11.11435133	Bcer	D	Bcer	FALSE	NA	NA	NA
Bc_ctg1_2243	B1	733.1502469	Bcer	M	Bcer	FALSE	NA	NA	NA
Bc_ctg1_2244	B1	162.5473882	Bcer	S	Bcer	FALSE	NA	NA	NA
Bc_ctg1_2245	B1	8.43499878	Bcer	S	Bcer	FALSE	NA	NA	NA
Bc_ctg1_2246	B1	6.053352065	Bcer	C	Bcer	FALSE	NA	NA	NA
Bc_ctg1_2247	B1	4.06864647	Bcer	C	Bcer	FALSE	NA	NA	NA
Bc_ctg1_2248	B1	2.679352554	Bcer	CP	Bcer	FALSE	NA	NA	NA
Bc_ctg1_2249	B1	0	Bcer	C	Bcer	FALSE	NA	NA	NA
Bc_ctg1_225	B1	0.09923528	Bcer	G	Bcer	FALSE	NA	NA	NA
Bc_ctg1_2250	B1	0.893117518	Bcer	C	Bcer	FALSE	NA	NA	NA
Bc_ctg1_2251	B1	0.793882238	Bcer	C	Bcer	FALSE	NA	NA	NA
Bc_ctg1_2252	B1	1.190823357	Bcer	C	Bcer	FALSE	NA	NA	NA
Bc_ctg1_2253	B1	1.190823357	Bcer	C	Bcer	FALSE	NA	NA	NA
Bc_ctg1_2254	B1	0.19847056	Bcer	C	Bcer	FALSE	NA	NA	NA
Bc_ctg1_2255	B1	1.389293917	Bcer	C	Bcer	FALSE	NA	NA	NA
Bc_ctg1_2256	B1	0.595411679	Bcer	C	Bcer	FALSE	NA	NA	NA
Bc_ctg1_2257	B1	13.09905693	Bcer	T	Bcer	FALSE	NA	NA	NA
Bc_ctg1_2258	B1	26.4958197	Bcer	S	Bcer	FALSE	NA	NA	NA
Bc_ctg1_2259	B1	20.54170291	Bcer	S	Bcer	FALSE	NA	NA	NA
Bc_ctg1_226	B1	10.22123382	Bcer	NA	Bcer	FALSE	NA	NA	NA
Bc_ctg1_2260	B1	746.5470096	Bcer	C	Bcer	FALSE	NA	NA	NA
Bc_ctg1_2261	B1	2993.134508	Bcer	C	Bcer	FALSE	NA	NA	NA
Bc_ctg1_2262	B1	1072.534904	Bcer	C	Bcer	FALSE	NA	NA	NA
Bc_ctg1_2263	B1	1549.161952	Bcer	C	Bcer	FALSE	NA	NA	NA

Bc_ctg1_2264	B1	416.1927633	Bcer	C	Bcer	FALSE	NA	NA	NA
Bc_ctg1_2265	B1	392.0785903	Bcer	C	Bcer	FALSE	NA	NA	NA
Bc_ctg1_2266	B1	108.6626313	Bcer	C	Bcer	FALSE	NA	NA	NA
Bc_ctg1_2267	B1	590.7476204	Bcer	C	Bcer	FALSE	NA	NA	NA
Bc_ctg1_2268	B1	186.6615612	Bcer	S	Bcer	FALSE	NA	NA	NA
Bc_ctg1_2269	B1	108.4641608	Bcer	S	Bcer	FALSE	NA	NA	NA
Bc_ctg1_2270	B1	227.6457318	Bcer	F	Bcer	FALSE	NA	NA	NA
Bc_ctg1_2271	B1	1189.831004	Bcer	E	Bcer	FALSE	NA	NA	NA
Bc_ctg1_2272	B1	120.2731591	Bcer	S	Bcer	FALSE	NA	NA	NA
Bc_ctg1_2273	B1	65.89222576	Bcer	G	Bcer	FALSE	NA	NA	NA
Bc_ctg1_2274	B1	20.83940875	Bcer	T	Bcer	FALSE	NA	NA	NA
Bc_ctg1_2275	B1	295.125722	Bcer	G	Bcer	FALSE	NA	NA	NA
Bc_ctg1_2276	B1	0.793882238	Bcer	S	Bcer	FALSE	NA	NA	NA
Bc_ctg1_2277	B1	26.89276082	Bcer	S	Bcer	FALSE	NA	NA	NA
Bc_ctg1_2278	B1	16.27458588	Bcer	NA	Bcer	FALSE	NA	NA	NA
Bc_ctg1_2279	B1	0.297705839	Bcer	NA	Bcer	FALSE	NA	NA	NA
Bc_ctg1_2280	B1	46.24364037	Bcer	P	Bcer	FALSE	NA	NA	NA
Bc_ctg1_2281	B1	46.14440509	Bcer	J	Bcer	FALSE	NA	NA	NA
Bc_ctg1_2282	B1	16.1753506	Bcer	E	Bcer	FALSE	NA	NA	NA
Bc_ctg1_2283	B1	0	Bcer	S	Bcer	FALSE	NA	NA	NA
Bc_ctg1_2284	B1	46.14440509	Bcer	J	Bcer	FALSE	NA	NA	NA
Bc_ctg1_2285	B1	343.9494797	Bcer	J	Bcer	FALSE	NA	NA	NA
Bc_ctg1_2286	B1	53.68628635	Bcer	F	Bcer	FALSE	NA	NA	NA
Bc_ctg1_2287	B1	2448.432058	Bcer	J	Bcer	FALSE	NA	NA	NA
Bc_ctg1_2288	B1	587.2743856	Bcer	K	Bcer	FALSE	NA	NA	NA
Bc_ctg1_2289	B1	338.5907745	Bcer	G	Bcer	FALSE	NA	NA	NA
Bc_ctg1_229	B1	0	Bcer	NA	Bcer	FALSE	NA	NA	NA
Bc_ctg1_2290	B1	531.2064526	Bcer	M	Bcer	FALSE	NA	NA	NA
Bc_ctg1_2291	B1	3140.796604	Bcer	G	Bcer	FALSE	NA	NA	NA
Bc_ctg1_2292	B1	90.30410458	Bcer	T	Bcer	FALSE	NA	NA	NA
Bc_ctg1_2293	B1	12.99982165	Bcer	S	Bcer	FALSE	NA	NA	NA
Bc_ctg1_2294	B1	613.3732642	Bcer	F	Bcer	FALSE	NA	NA	NA
Bc_ctg1_2295	B1	590.251444	Bcer	K	Bcer	FALSE	NA	NA	NA
Bc_ctg1_2296	B1	8.3357635	Bcer	K	Bcer	FALSE	NA	NA	NA
Bc_ctg1_2297	B1	41.1826411	Bcer	I	Bcer	FALSE	NA	NA	NA
Bc_ctg1_2298	B1	33.14458344	Bcer	I	Bcer	FALSE	NA	NA	NA
Bc_ctg1_2299	B1	35.72470071	Bcer	I	Bcer	FALSE	NA	NA	NA
Bc_ctg1_23	B1	3.572470071	Bcer	E	Bcer	FALSE	NA	NA	NA
Bc_ctg1_230	B1	2.083940875	Bcer	NA	Bcer	FALSE	NA	NA	NA
Bc_ctg1_2300	B1	20.83940875	Bcer	I	Bcer	FALSE	NA	NA	NA
Bc_ctg1_2301	B1	253.1491987	Bcer	C	Bcer	FALSE	NA	NA	NA
Bc_ctg1_2302	B1	1.686999756	Bcer	I	Bcer	FALSE	NA	NA	NA
Bc_ctg1_2303	B1	0.893117518	Bcer	L	Bcer	FALSE	NA	NA	NA
Bc_ctg1_2304	B1	28.48052529	Bcer	T	Bcer	FALSE	NA	NA	NA
Bc_ctg1_2305	B1	0.19847056	Bcer	S	Bcer	FALSE	NA	NA	NA
Bc_ctg1_2306	B1	48.22834596	Bcer	K	Bcer	FALSE	NA	NA	NA
Bc_ctg1_2307	B1	582.9080333	Bcer	E	Bcer	FALSE	NA	NA	NA
Bc_ctg1_2308	B1	11.90823357	Bcer	T	Bcer	FALSE	NA	NA	NA
Bc_ctg1_2309	B1	37.21322991	Bcer	T	Bcer	FALSE	NA	NA	NA
Bc_ctg1_231	B1	6.549528464	Bcer	IQ	Bcer	TRUE	NA	NA	NA
Bc_ctg1_2310	B1	5.557175667	Bcer	V	Bcer	FALSE	NA	NA	NA
Bc_ctg1_2311	B1	8.038057661	Bcer	V	Bcer	FALSE	NA	NA	NA
Bc_ctg1_2312	B1	332.1404814	Bcer	E	Bcer	FALSE	NA	NA	NA
Bc_ctg1_2313	B1	424.7269974	Bcer	M	Bcer	FALSE	NA	NA	NA
Bc_ctg1_2314	B1	2.480881994	Bcer	S	Bcer	FALSE	NA	NA	NA
Bc_ctg1_2315	B1	9.328116298	Bcer	NA	Bcer	FALSE	NA	NA	NA
Bc_ctg1_2316	B1	0.19847056	Bcer	NA	Bcer	FALSE	NA	NA	NA
Bc_ctg1_2317	B1	0.992352798	Bcer	NA	Bcer	FALSE	NA	NA	NA
Bc_ctg1_2318	B1	0	Bcer	K	Bcer	FALSE	NA	NA	NA
Bc_ctg1_2319	B1	117.4945712	Bcer	J	Bcer	FALSE	NA	NA	NA
Bc_ctg1_232	B1	43.26658198	Bcer	S	Bcer	FALSE	NA	NA	NA
Bc_ctg1_2320	B1	12.99982165	Bcer	S	Bcer	FALSE	NA	NA	NA

Bc_ctg1_2321	B1	27.98434889	Bcer	V	Bcer	FALSE	NA	NA	NA
Bc_ctg1_2322	B1	0	Bcer	S	Bcer	FALSE	NA	NA	NA
Bc_ctg1_2323	B1	26.89276082	Bcer	G	Bcer	FALSE	NA	NA	NA
Bc_ctg1_2324	B1	252.2560812	Bcer	E	Bcer	FALSE	NA	NA	NA
Bc_ctg1_2325	B1	264.0650794	Bcer	E	Bcer	FALSE	NA	NA	NA
Bc_ctg1_2326	B1	4.862528708	Bcer	EGP	Bcer	FALSE	NA	NA	NA
Bc_ctg1_2327	B1	3.870175911	Bcer	K	Bcer	FALSE	NA	NA	NA
Bc_ctg1_2328	B1	172.1732104	Bcer	C	Bcer	FALSE	NA	NA	NA
Bc_ctg1_2329	B1	5.656410946	Bcer	M	Bcer	FALSE	NA	NA	NA
Bc_ctg1_233	B1	6.549528464	Bcer	V	Bcer	FALSE	NA	NA	NA
Bc_ctg1_2330	B1	97.64751529	Bcer	S	Bcer	FALSE	NA	NA	NA
Bc_ctg1_2331	B1	125.1356878	Bcer	S	Bcer	FALSE	NA	NA	NA
Bc_ctg1_2332	B1	207.0047936	Bcer	G	Bcer	FALSE	NA	NA	NA
Bc_ctg1_2333	B1	381.4604154	Bcer	S	Bcer	FALSE	NA	NA	NA
Bc_ctg1_2334	B1	77.40351821	Bcer	P	Bcer	FALSE	NA	NA	NA
Bc_ctg1_2335	B1	66.09069632	Bcer	HP	Bcer	FALSE	NA	NA	NA
Bc_ctg1_2336	B1	60.73199121	Bcer	P	Bcer	FALSE	NA	NA	NA
Bc_ctg1_2337	B1	34.53387736	Bcer	P	Bcer	FALSE	NA	NA	NA
Bc_ctg1_2338	B1	2.877823113	Bcer	S	Bcer	FALSE	NA	NA	NA
Bc_ctg1_2339	B1	0.595411679	Bcer	S	Bcer	FALSE	NA	NA	NA
Bc_ctg1_234	B1	0.496176399	Bcer	NA	Bcer	FALSE	NA	NA	NA
Bc_ctg1_2340	B1	45.25128757	Bcer	H	Bcer	FALSE	NA	NA	NA
Bc_ctg1_2341	B1	0	Bcer	NA	Bcer	FALSE	NA	NA	NA
Bc_ctg1_2343	B1	288.0800171	Bcer	C	Bcer	FALSE	NA	NA	NA
Bc_ctg1_2344	B1	718.0664844	Bcer	C	Bcer	FALSE	NA	NA	NA
Bc_ctg1_2345	B1	48.5260518	Bcer	M	Bcer	FALSE	NA	NA	NA
Bc_ctg1_2346	B1	0.09923528	Bcer	M	Bcer	FALSE	NA	NA	NA
Bc_ctg1_2347	B1	0.694646958	Bcer	S	Bcer	FALSE	NA	NA	NA
Bc_ctg1_2348	B1	50.31228684	Bcer	S	Bcer	FALSE	NA	NA	NA
Bc_ctg1_2349	B1	1.190823357	Bcer	S	Bcer	FALSE	NA	NA	NA
Bc_ctg1_235	B1	3.076293673	Bcer	NA	Bcer	FALSE	NA	NA	NA
Bc_ctg1_2350	B1	14.48835085	Bcer	P	Bcer	FALSE	NA	NA	NA
Bc_ctg1_2351	B1	29.96905449	Bcer	F	Bcer	FALSE	NA	NA	NA
Bc_ctg1_2352	B1	127.9142756	Bcer	S	Bcer	FALSE	Bc_Ga0417192_01_rn16	Ancillary biosynthesis	SMCOG1179
Bc_ctg1_2353	B1	29.67134865	Bcer	EGP	Bcer	FALSE	Bc_Ga0417192_01_rn16	Other	NA
Bc_ctg1_2354	B1	42.57193502	Bcer	L	Bcer	FALSE	Bc_Ga0417192_01_rn16	Other	NA
Bc_ctg1_2355	B1	48.92299292	Bcer	S	Bcer	FALSE	Bc_Ga0417192_01_rn16	Other	NA
Bc_ctg1_2356	B1	32.54917176	Bcer	V	Bcer	FALSE	Bc_Ga0417192_01_rn16	Transport	SMCOG1000
Bc_ctg1_2357	B1	27.19046665	Bcer	E	Bcer	FALSE	Bc_Ga0417192_01_rn16	Other	NA
Bc_ctg1_2358	B1	5.755646226	Bcer	K	Bcer	FALSE	Bc_Ga0417192_01_rn16	Regulation	SMCOG1078
Bc_ctg1_2359	B1	6.251822625	Bcer	S	Bcer	FALSE	Bc_Ga0417192_01_rn16	Other	NA
Bc_ctg1_236	B1	2.381646714	Bcer	K	Bcer	FALSE	NA	NA	NA
Bc_ctg1_2360	B1	39.6941119	Bcer	E	Bcer	FALSE	Bc_Ga0417192_01_rn16	Other	NA
Bc_ctg1_2361	B1	25.10652578	Bcer	E	Bcer	FALSE	Bc_Ga0417192_01_rn16	Other	NA
Bc_ctg1_2362	B1	57.06028586	Bcer	E	Bcer	FALSE	Bc_Ga0417192_01_rn16	Core biosynthesis	SMCOG1168
Bc_ctg1_2363	B1	17.1677034	Bcer	P	Bcer	FALSE	Bc_Ga0417192_01_rn16	Other	NA
Bc_ctg1_2364	B1	1.885470315	Bcer	S	Bcer	FALSE	Bc_Ga0417192_01_rn16	Ancillary biosynthesis	SMCOG1115
Bc_ctg1_2365	B1	17.1677034	Bcer	T	Bcer	FALSE	Bc_Ga0417192_01_rn16	Regulation	SMCOG1133
Bc_ctg1_2366	B1	0.09923528	Bcer	L	Bcer	FALSE	Bc_Ga0417192_01_rn16	Other	SMCOG1164
Bc_ctg1_2367	B1	0.09923528	Bcer	L	Bcer	FALSE	Bc_Ga0417192_01_rn16	Other	SMCOG1026
Bc_ctg1_2368	B1	4.06864647	Bcer	I	Bcer	FALSE	Bc_Ga0417192_01_rn16	Other	NA
Bc_ctg1_2369	B1	7.839587101	Bcer	K	Bcer	FALSE	Bc_Ga0417192_01_rn16	Regulation	SMCOG1016
Bc_ctg1_237	B1	108.5633961	Bcer	NA	Bcer	FALSE	NA	NA	NA
Bc_ctg1_2370	B1	8.13729294	Bcer	T	Bcer	FALSE	Bc_Ga0417192_01_rn16	Regulation	SMCOG1048
Bc_ctg1_2371	B1	444.8717592	Bcer	H	Bcer	FALSE	Bc_Ga0417192_01_rn16	Other	NA
Bc_ctg1_2372	B1	46.34287565	Bcer	T	Bcer	FALSE	Bc_Ga0417192_01_rn16	Ancillary biosynthesis	SMCOG1025
Bc_ctg1_2373	B1	4.465587589	Bcer	S	Bcer	FALSE	Bc_Ga0417192_01_rn16	Other	SMCOG1211
Bc_ctg1_2374	B1	596.7017372	Bcer	T	Bcer	FALSE	Bc_Ga0417192_01_rn16	Other	SMCOG1185
Bc_ctg1_2375	B1	416.3912339	Bcer	KT	Bcer	FALSE	Bc_Ga0417192_01_rn16	Other	NA
Bc_ctg1_2376	B1	30.86217201	Bcer	G	Bcer	FALSE	Bc_Ga0417192_01_rn16	Transport	SMCOG1169
Bc_ctg1_2377	B1	109.3572783	Bcer	M	Bcer	FALSE	Bc_Ga0417192_01_rn16	Other	NA
Bc_ctg1_2378	B1	154.2116247	Bcer	M	Bcer	FALSE	Bc_Ga0417192_01_rn16	Core biosynthesis	SMCOG1045

Bc_ctg1_2379	B1	340.5754801	Bcer	M	Bcer	FALSE	Bc_Ga0417192_01_rn16	Other	NA
Bc_ctg1_238	B1	69.66316639	Bcer	S	Bcer	FALSE	NA	NA	NA
Bc_ctg1_2380	B1	43.36581726	Bcer	S	Bcer	FALSE	Bc_Ga0417192_01_rn16	Other	NA
Bc_ctg1_2381	B1	103.1054557	Bcer	NT	Bcer	FALSE	Bc_Ga0417192_01_rn16	Other	SMCOG1061
Bc_ctg1_2382	B1	29.17517225	Bcer	M	Bcer	FALSE	Bc_Ga0417192_01_rn16	Other	NA
Bc_ctg1_2383	B1	20.93864403	Bcer	I	Bcer	FALSE	Bc_Ga0417192_01_rn16	Other	NA
Bc_ctg1_2384	B1	0	Bcer	M	Bcer	FALSE	Bc_Ga0417192_01_rn16	Other	NA
Bc_ctg1_2385	B1	36.32011239	Bcer	M	Bcer	FALSE	Bc_Ga0417192_01_rn16	Core biosynthesis	SMCOG1123
Bc_ctg1_2386	B1	78.49510629	Bcer	S	Bcer	FALSE	Bc_Ga0417192_01_rn16	Core biosynthesis	None
Bc_ctg1_2387	B1	569.0150942	Bcer	S	Bcer	FALSE	Bc_Ga0417192_01_rn16	Other	NA
Bc_ctg1_2388	B1	304.2553677	Bcer	M	Bcer	FALSE	Bc_Ga0417192_01_rn16	Core biosynthesis	SMCOG1123
Bc_ctg1_239	B1	43.66352309	Bcer	T	Bcer	FALSE	NA	NA	NA
Bc_ctg1_2390	B1	0	Bcer	NA	Bcer	FALSE	Bc_Ga0417192_01_rn16	Other	NA
Bc_ctg1_2391	B1	3.373999512	Bcer	GM	Bcer	FALSE	Bc_Ga0417192_01_rn16	Ancillary biosynthesis	SMCOG1010
Bc_ctg1_2392	B1	34.3354068	Bcer	K	Bcer	FALSE	Bc_Ga0417192_01_rn16	Regulation	SMCOG1057
Bc_ctg1_2393	B1	184.2799145	Bcer	S	Bcer	FALSE	Bc_Ga0417192_01_rn16	Transport	SMCOG1000
Bc_ctg1_2394	B1	32.54917176	Bcer	K	Bcer	FALSE	Bc_Ga0417192_01_rn16	Regulation	SMCOG1057
Bc_ctg1_2395	B1	150.043743	Bcer	V	Bcer	FALSE	Bc_Ga0417192_01_rn16	Transport	SMCOG1049
Bc_ctg1_2396	B1	62.91516737	Bcer	O	Bcer	FALSE	NA	NA	NA
Bc_ctg1_2397	B1	589.0606207	Bcer	S	Bcer	FALSE	NA	NA	NA
Bc_ctg1_2398	B1	820.2788225	Bcer	M	Bcer	FALSE	NA	NA	NA
Bc_ctg1_2399	B1	235.9814953	Bcer	S	Bcer	FALSE	NA	NA	NA
Bc_ctg1_24	B1	2.282411435	Bcer	E	Bcer	FALSE	NA	NA	NA
Bc_ctg1_240	B1	0.694646958	Bcer	K	Bcer	FALSE	NA	NA	NA
Bc_ctg1_2400	B1	23.5187613	Bcer	T	Bcer	FALSE	NA	NA	NA
Bc_ctg1_2401	B1	39.19793551	Bcer	T	Bcer	FALSE	NA	NA	NA
Bc_ctg1_2402	B1	3.076293673	Bcer	EGP	Bcer	FALSE	Bc_Ga0417192_01_rn17	Transport	SMCOG1169
Bc_ctg1_2403	B1	57.06028586	Bcer	M	Bcer	FALSE	Bc_Ga0417192_01_rn17	Other	NA
Bc_ctg1_2404	B1	291.6524872	Bcer	C	Bcer	FALSE	Bc_Ga0417192_01_rn17	Other	NA
Bc_ctg1_2405	B1	30763.23443	Bcer	P	Bcer	FALSE	Bc_Ga0417192_01_rn17	Other	NA
Bc_ctg1_2406	B1	12.30517469	Bcer	GM	Bcer	FALSE	Bc_Ga0417192_01_rn17	Ancillary biosynthesis	SMCOG1010
Bc_ctg1_2407	B1	20.64093819	Bcer	S	Bcer	FALSE	Bc_Ga0417192_01_rn17	Other	NA
Bc_ctg1_2408	B1	0.297705839	Bcer	NA	Bcer	FALSE	Bc_Ga0417192_01_rn17	Other	NA
Bc_ctg1_2409	B1	391.4831787	Bcer	M	Bcer	FALSE	Bc_Ga0417192_01_rn17	Core biosynthesis	SMCOG1010
Bc_ctg1_241	B1	9.923527976	Bcer	S	Bcer	FALSE	NA	NA	NA
Bc_ctg1_2410	B1	687.0058418	Bcer	S	Bcer	FALSE	Bc_Ga0417192_01_rn17	Other	NA
Bc_ctg1_2411	B1	31.65605424	Bcer	K	Bcer	FALSE	Bc_Ga0417192_01_rn17	Other	NA
Bc_ctg1_2412	B1	73.0371659	Bcer	JKL	Bcer	FALSE	Bc_Ga0417192_01_rn17	Other	SMCOG1122
Bc_ctg1_2413	B1	446.2610531	Bcer	F	Bcer	FALSE	Bc_Ga0417192_01_rn17	Other	NA
Bc_ctg1_2414	B1	35.22852432	Bcer	E	Bcer	FALSE	Bc_Ga0417192_01_rn17	Other	NA
Bc_ctg1_2415	B1	35.03005376	Bcer	C	Bcer	FALSE	Bc_Ga0417192_01_rn17	Ancillary biosynthesis	None
Bc_ctg1_2416	B1	79.78516493	Bcer	T	Bcer	FALSE	Bc_Ga0417192_01_rn17	Regulation	SMCOG1003
Bc_ctg1_2417	B1	19.64858539	Bcer	J	Bcer	FALSE	NA	NA	NA
Bc_ctg1_2418	B1	0.496176399	Bcer	S	Bcer	FALSE	NA	NA	NA
Bc_ctg1_2419	B1	145.2804496	Bcer	O	Bcer	FALSE	NA	NA	NA
Bc_ctg1_242	B1	3.572470071	Bcer	F	Bcer	FALSE	NA	NA	NA
Bc_ctg1_2420	B1	140.6163914	Bcer	S	Bcer	FALSE	NA	NA	NA
Bc_ctg1_2421	B1	136.1508038	Bcer	S	Bcer	FALSE	NA	NA	NA
Bc_ctg1_2422	B1	295.9196042	Bcer	S	Bcer	FALSE	NA	NA	NA
Bc_ctg1_2423	B1	465.4134621	Bcer	T	Bcer	FALSE	NA	NA	NA
Bc_ctg1_2424	B1	150.043743	Bcer	T	Bcer	FALSE	NA	NA	NA
Bc_ctg1_2425	B1	795.8669437	Bcer	F	Bcer	FALSE	NA	NA	NA
Bc_ctg1_2426	B1	665.9679625	Bcer	L	Bcer	FALSE	NA	NA	NA
Bc_ctg1_2427	B1	102.9069851	Bcer	J	Bcer	FALSE	NA	NA	NA
Bc_ctg1_2428	B1	835.6602909	Bcer	T	Bcer	FALSE	NA	NA	NA
Bc_ctg1_2429	B1	25.80117274	Bcer	S	Bcer	FALSE	NA	NA	NA
Bc_ctg1_243	B1	3.770940631	Bcer	S	Bcer	FALSE	NA	NA	NA
Bc_ctg1_2430	B1	260.9887858	Bcer	J	Bcer	FALSE	NA	NA	NA
Bc_ctg1_2431	B1	1577.146301	Bcer	L	Bcer	FALSE	NA	NA	NA
Bc_ctg1_2432	B1	776.4168289	Bcer	J	Bcer	FALSE	NA	NA	NA
Bc_ctg1_2433	B1	352.8806548	Bcer	J	Bcer	FALSE	NA	NA	NA
Bc_ctg1_2434	B1	14.19064501	Bcer	S	Bcer	FALSE	NA	NA	NA

Bc_ctg1_2435	B1	119.4792768	Bcer	M	Bcer	FALSE	NA	NA	NA
Bc_ctg1_2436	B1	0.19847056	Bcer	S	Bcer	FALSE	NA	NA	NA
Bc_ctg1_2437	B1	1735.426572	Bcer	K	Bcer	FALSE	NA	NA	NA
Bc_ctg1_2438	B1	458.0700514	Bcer	D	Bcer	FALSE	NA	NA	NA
Bc_ctg1_2439	B1	343.1555974	Bcer	D	Bcer	FALSE	NA	NA	NA
Bc_ctg1_244	B1	2.183176155	Bcer	E	Bcer	FALSE	NA	NA	NA
Bc_ctg1_2440	B1	130.7920987	Bcer	J	Bcer	FALSE	NA	NA	NA
Bc_ctg1_2441	B1	384.2390032	Bcer	D	Bcer	FALSE	NA	NA	NA
Bc_ctg1_2442	B1	191.1271488	Bcer	S	Bcer	FALSE	NA	NA	NA
Bc_ctg1_2443	B1	359.330948	Bcer	S	Bcer	FALSE	NA	NA	NA
Bc_ctg1_2444	B1	301.873721	Bcer	U	Bcer	FALSE	NA	NA	NA
Bc_ctg1_2445	B1	22.62564379	Bcer	J	Bcer	FALSE	NA	NA	NA
Bc_ctg1_2446	B1	0.297705839	Bcer	J	Bcer	FALSE	NA	NA	NA
Bc_ctg1_2447	B1	358.2393599	Bcer	L	Bcer	FALSE	NA	NA	NA
Bc_ctg1_2448	B1	320.0337772	Bcer	L	Bcer	FALSE	NA	NA	NA
Bc_ctg1_2449	B1	12.90058637	Bcer	S	Bcer	FALSE	NA	NA	NA
Bc_ctg1_245	B1	7.045704863	Bcer	GK	Bcer	FALSE	NA	NA	NA
Bc_ctg1_2450	B1	330.3542463	Bcer	L	Bcer	FALSE	NA	NA	NA
Bc_ctg1_2451	B1	1158.671126	Bcer	L	Bcer	FALSE	NA	NA	NA
Bc_ctg1_2452	B1	2034.124765	Bcer	L	Bcer	FALSE	NA	NA	NA
Bc_ctg1_2454	B1	0.694646958	Bcer	S	Bcer	FALSE	NA	NA	NA
Bc_ctg1_2455	B1	1374.210154	Bcer	F	Bcer	FALSE	NA	NA	NA
Bc_ctg1_2456	B1	341.6670682	Bcer	M	Bcer	FALSE	NA	NA	NA
Bc_ctg1_2457	B1	646.6170829	Bcer	H	Bcer	FALSE	NA	NA	NA
Bc_ctg1_2458	B1	527.4355119	Bcer	H	Bcer	FALSE	NA	NA	NA
Bc_ctg1_2459	B1	603.4497362	Bcer	J	Bcer	FALSE	NA	NA	NA
Bc_ctg1_246	B1	2.580117274	Bcer	G	Bcer	FALSE	NA	NA	NA
Bc_ctg1_2460	B1	304.7515441	Bcer	S	Bcer	FALSE	NA	NA	NA
Bc_ctg1_2461	B1	22.52640851	Bcer	F	Bcer	FALSE	NA	NA	NA
Bc_ctg1_2462	B1	13.09905693	Bcer	F	Bcer	FALSE	NA	NA	NA
Bc_ctg1_2463	B1	0	Bcer	Q	Bcer	TRUE	NA	NA	NA
Bc_ctg1_2464	B1	8.13729294	Bcer	FJ	Bcer	FALSE	NA	NA	NA
Bc_ctg1_2465	B1	511.9548083	Bcer	L	Bcer	FALSE	NA	NA	NA
Bc_ctg1_2466	B1	85.34234059	Bcer	S	Bcer	FALSE	NA	NA	NA
Bc_ctg1_2467	B1	437.0321721	Bcer	L	Bcer	FALSE	NA	NA	NA
Bc_ctg1_2468	B1	112.7312778	Bcer	S	Bcer	FALSE	NA	NA	NA
Bc_ctg1_2469	B1	0.793882238	Bcer	S	Bcer	FALSE	NA	NA	NA
Bc_ctg1_247	B1	1.786235036	Bcer	S	Bcer	FALSE	NA	NA	NA
Bc_ctg1_2471	B1	0	Bcer	S	Bcer	FALSE	NA	NA	NA
Bc_ctg1_2472	B1	69.86163695	Bcer	E	Bcer	FALSE	NA	NA	NA
Bc_ctg1_2473	B1	22.82411435	Bcer	F	Bcer	FALSE	NA	NA	NA
Bc_ctg1_2474	B1	182.69215	Bcer	L	Bcer	FALSE	NA	NA	NA
Bc_ctg1_2475	B1	189.5393843	Bcer	S	Bcer	FALSE	NA	NA	NA
Bc_ctg1_2476	B1	47.73216957	Bcer	L	Bcer	FALSE	NA	NA	NA
Bc_ctg1_2477	B1	12.20593941	Bcer	S	Bcer	FALSE	NA	NA	NA
Bc_ctg1_2478	B1	2.183176155	Bcer	L	Bcer	FALSE	NA	NA	NA
Bc_ctg1_2479	B1	21.23634987	Bcer	H	Bcer	FALSE	NA	NA	NA
Bc_ctg1_248	B1	7.244175423	Bcer	J	Bcer	FALSE	NA	NA	NA
Bc_ctg1_2480	B1	1301.470694	Bcer	K	Bcer	FALSE	NA	NA	NA
Bc_ctg1_2481	B1	842.9044663	Bcer	J	Bcer	FALSE	NA	NA	NA
Bc_ctg1_2482	B1	43.76275837	Bcer	L	Bcer	FALSE	NA	NA	NA
Bc_ctg1_2483	B1	24.11417298	Bcer	J	Bcer	FALSE	NA	NA	NA
Bc_ctg1_2484	B1	111.3419839	Bcer	J	Bcer	FALSE	NA	NA	NA
Bc_ctg1_2485	B1	4.16788175	Bcer	S	Bcer	FALSE	NA	NA	NA
Bc_ctg1_2486	B1	0.09923528	Bcer	S	Bcer	FALSE	NA	NA	NA
Bc_ctg1_2488	B1	87.02934035	Bcer	I	Bcer	FALSE	NA	NA	NA
Bc_ctg1_2489	B1	450.9251112	Bcer	F	Bcer	FALSE	NA	NA	NA
Bc_ctg1_249	B1	34.3354068	Bcer	I	Bcer	FALSE	NA	NA	NA
Bc_ctg1_2490	B1	119.7769827	Bcer	J	Bcer	FALSE	NA	NA	NA
Bc_ctg1_2491	B1	1101.809311	Bcer	D	Bcer	FALSE	NA	NA	NA
Bc_ctg1_2492	B1	1255.72323	Bcer	M	Bcer	FALSE	NA	NA	NA
Bc_ctg1_2493	B1	853.7211118	Bcer	F	Bcer	FALSE	NA	NA	NA

Bc_ctg1_2494	B1	107.6702785	Bcer	J	Bcer	FALSE	NA	NA	NA
Bc_ctg1_2495	B1	7.244175423	Bcer	S	Bcer	FALSE	NA	NA	NA
Bc_ctg1_2496	B1	506.7945737	Bcer	L	Bcer	FALSE	NA	NA	NA
Bc_ctg1_2497	B1	142.7995676	Bcer	K	Bcer	FALSE	NA	NA	NA
Bc_ctg1_2498	B1	141.8072148	Bcer	S	Bcer	FALSE	NA	NA	NA
Bc_ctg1_2499	B1	165.3259761	Bcer	S	Bcer	FALSE	NA	NA	NA
Bc_ctg1_25	B1	1.488529196	Bcer	K	Bcer	FALSE	NA	NA	NA
Bc_ctg1_250	B1	0.09923528	Bcer	L	Bcer	FALSE	NA	NA	NA
Bc_ctg1_2500	B1	26.9919961	Bcer	J	Bcer	FALSE	NA	NA	NA
Bc_ctg1_2501	B1	55.27405083	Bcer	S	Bcer	FALSE	NA	NA	NA
Bc_ctg1_2502	B1	178.2265625	Bcer	S	Bcer	FALSE	NA	NA	NA
Bc_ctg1_2503	B1	114.5175128	Bcer	D	Bcer	FALSE	NA	NA	NA
Bc_ctg1_2504	B1	661.0061985	Bcer	J	Bcer	FALSE	NA	NA	NA
Bc_ctg1_2505	B1	7.244175423	Bcer	KT	Bcer	FALSE	NA	NA	NA
Bc_ctg1_2506	B1	2394.448065	Bcer	D	Bcer	FALSE	NA	NA	NA
Bc_ctg1_2507	B1	1198.563709	Bcer	F	Bcer	FALSE	NA	NA	NA
Bc_ctg1_2508	B1	4609.280274	Bcer	O	Bcer	FALSE	NA	NA	NA
Bc_ctg1_2509	B1	91.49492794	Bcer	F	Bcer	FALSE	NA	NA	NA
Bc_ctg1_251	B1	0.09923528	Bcer	L	Bcer	FALSE	NA	NA	NA
Bc_ctg1_2510	B1	192.9133839	Bcer	O	Bcer	FALSE	NA	NA	NA
Bc_ctg1_2511	B1	716.4787199	Bcer	E	Bcer	FALSE	NA	NA	NA
Bc_ctg1_2512	B1	126.4257464	Bcer	EH	Bcer	FALSE	NA	NA	NA
Bc_ctg1_2513	B1	68.47234304	Bcer	EH	Bcer	FALSE	NA	NA	NA
Bc_ctg1_2514	B1	85.83851699	Bcer	EH	Bcer	FALSE	NA	NA	NA
Bc_ctg1_2515	B1	64.89987296	Bcer	H	Bcer	FALSE	NA	NA	NA
Bc_ctg1_2516	B1	23.41952602	Bcer	H	Bcer	FALSE	NA	NA	NA
Bc_ctg1_2517	B1	32.25146592	Bcer	H	Bcer	FALSE	NA	NA	NA
Bc_ctg1_2518	B1	0.396941119	Bcer	K	Bcer	FALSE	NA	NA	NA
Bc_ctg1_2519	B1	50.90769852	Bcer	J	Bcer	FALSE	NA	NA	NA
Bc_ctg1_252	B1	66.1899316	Bcer	S	Bcer	FALSE	NA	NA	NA
Bc_ctg1_2520	B1	782.867122	Bcer	J	Bcer	FALSE	NA	NA	NA
Bc_ctg1_2523	B1	129.7997459	Bcer	K	Bcer	FALSE	NA	NA	NA
Bc_ctg1_2524	B1	177.2342097	Bcer	S	Bcer	FALSE	NA	NA	NA
Bc_ctg1_2525	B1	465.3142268	Bcer	E	Bcer	FALSE	NA	NA	NA
Bc_ctg1_2526	B1	1610.787061	Bcer	O	Bcer	FALSE	NA	NA	NA
Bc_ctg1_2527	B1	58.94575618	Bcer	O	Bcer	FALSE	NA	NA	NA
Bc_ctg1_2528	B1	72.24328367	Bcer	L	Bcer	FALSE	NA	NA	NA
Bc_ctg1_2529	B1	281.9274298	Bcer	S	Bcer	FALSE	NA	NA	NA
Bc_ctg1_253	B1	24.70958466	Bcer	NA	Bcer	FALSE	NA	NA	NA
Bc_ctg1_2530	B1	225.958732	Bcer	I	Bcer	FALSE	NA	NA	NA
Bc_ctg1_2531	B1	47.03752261	Bcer	I	Bcer	FALSE	NA	NA	NA
Bc_ctg1_2532	B1	1111.732839	Bcer	J	Bcer	FALSE	NA	NA	NA
Bc_ctg1_2533	B1	59.24346202	Bcer	E	Bcer	FALSE	NA	NA	NA
Bc_ctg1_2534	B1	240.4470829	Bcer	J	Bcer	FALSE	NA	NA	NA
Bc_ctg1_2535	B1	26.89276082	Bcer	J	Bcer	FALSE	NA	NA	NA
Bc_ctg1_2536	B1	101.4184559	Bcer	J	Bcer	FALSE	NA	NA	NA
Bc_ctg1_2537	B1	99.92992672	Bcer	S	Bcer	FALSE	NA	NA	NA
Bc_ctg1_2538	B1	375.7047692	Bcer	K	Bcer	FALSE	NA	NA	NA
Bc_ctg1_2539	B1	8.633469339	Bcer	U	Bcer	FALSE	NA	NA	NA
Bc_ctg1_254	B1	2.580117274	Bcer	K	Bcer	FALSE	NA	NA	NA
Bc_ctg1_2540	B1	231.4166724	Bcer	K	Bcer	FALSE	NA	NA	NA
Bc_ctg1_2541	B1	3706.933875	Bcer	J	Bcer	FALSE	NA	NA	NA
Bc_ctg1_2542	B1	1066.580787	Bcer	J	Bcer	FALSE	NA	NA	NA
Bc_ctg1_2543	B1	2248.274498	Bcer	J	Bcer	FALSE	NA	NA	NA
Bc_ctg1_2544	B1	493.397811	Bcer	J	Bcer	FALSE	NA	NA	NA
Bc_ctg1_2545	B1	127.6165698	Bcer	J	Bcer	FALSE	NA	NA	NA
Bc_ctg1_2546	B1	6796.326605	Bcer	K	Bcer	FALSE	NA	NA	NA
Bc_ctg1_2547	B1	7302.029591	Bcer	K	Bcer	FALSE	NA	NA	NA
Bc_ctg1_2548	B1	637.8843783	Bcer	J	Bcer	FALSE	NA	NA	NA
Bc_ctg1_2549	B1	2302.159255	Bcer	J	Bcer	FALSE	NA	NA	NA
Bc_ctg1_255	B1	0.297705839	Bcer	NA	Bcer	FALSE	NA	NA	NA
Bc_ctg1_2550	B1	1295.218871	Bcer	J	Bcer	FALSE	NA	NA	NA

Bc_ctg1_2551	B1	9288.12448	Bcer	J	Bcer	FALSE	NA	NA	NA
Bc_ctg1_2552	B1	9457.320632	Bcer	J	Bcer	FALSE	NA	NA	NA
Bc_ctg1_2553	B1	1638.77141	Bcer	J	Bcer	FALSE	NA	NA	NA
Bc_ctg1_2554	B1	2486.538405	Bcer	J	Bcer	FALSE	NA	NA	NA
Bc_ctg1_2555	B1	2836.640472	Bcer	J	Bcer	FALSE	NA	NA	NA
Bc_ctg1_2556	B1	1019.444029	Bcer	J	Bcer	FALSE	NA	NA	NA
Bc_ctg1_2557	B1	4361.192075	Bcer	J	Bcer	FALSE	NA	NA	NA
Bc_ctg1_2558	B1	1568.314361	Bcer	J	Bcer	FALSE	NA	NA	NA
Bc_ctg1_2559	B1	2305.930196	Bcer	J	Bcer	FALSE	NA	NA	NA
Bc_ctg1_256	B1	1.984705595	Bcer	Q	Bcer	TRUE	NA	NA	NA
Bc_ctg1_2560	B1	2991.149803	Bcer	J	Bcer	FALSE	NA	NA	NA
Bc_ctg1_2561	B1	1399.51515	Bcer	J	Bcer	FALSE	NA	NA	NA
Bc_ctg1_2562	B1	270.5153726	Bcer	J	Bcer	FALSE	NA	NA	NA
Bc_ctg1_2563	B1	728.585424	Bcer	J	Bcer	FALSE	NA	NA	NA
Bc_ctg1_2564	B1	1218.51	Bcer	J	Bcer	FALSE	NA	NA	NA
Bc_ctg1_2565	B1	1513.437252	Bcer	J	Bcer	FALSE	NA	NA	NA
Bc_ctg1_2566	B1	2348.10519	Bcer	J	Bcer	FALSE	NA	NA	NA
Bc_ctg1_2567	B1	342.0640093	Bcer	J	Bcer	FALSE	NA	NA	NA
Bc_ctg1_2568	B1	1663.877936	Bcer	J	Bcer	FALSE	NA	NA	NA
Bc_ctg1_2569	B1	2396.432771	Bcer	J	Bcer	FALSE	NA	NA	NA
Bc_ctg1_257	B1	0.19847056	Bcer	Q	Bcer	TRUE	Bc_Ga0417192_01_rn02	Other	NA
Bc_ctg1_2570	B1	1183.579182	Bcer	J	Bcer	FALSE	NA	NA	NA
Bc_ctg1_2571	B1	2936.074222	Bcer	J	Bcer	FALSE	NA	NA	NA
Bc_ctg1_2572	B1	106.2809846	Bcer	J	Bcer	FALSE	NA	NA	NA
Bc_ctg1_2573	B1	1567.123538	Bcer	J	Bcer	FALSE	NA	NA	NA
Bc_ctg1_2574	B1	6741.251025	Bcer	U	Bcer	FALSE	NA	NA	NA
Bc_ctg1_2575	B1	2093.665932	Bcer	F	Bcer	FALSE	NA	NA	NA
Bc_ctg1_2576	B1	3483.35679	Bcer	E	Bcer	FALSE	NA	NA	NA
Bc_ctg1_2577	B1	385.926003	Bcer	J	Bcer	FALSE	NA	NA	NA
Bc_ctg1_2578	B1	0.595411679	Bcer	J	Bcer	FALSE	NA	NA	NA
Bc_ctg1_2579	B1	2634.299737	Bcer	J	Bcer	FALSE	NA	NA	NA
Bc_ctg1_258	B1	20.64093819	Bcer	S	Bcer	FALSE	Bc_Ga0417192_01_rn02	Other	SMCOG1272
Bc_ctg1_2580	B1	997.6122674	Bcer	J	Bcer	FALSE	NA	NA	NA
Bc_ctg1_2581	B1	4836.826771	Bcer	K	Bcer	FALSE	NA	NA	NA
Bc_ctg1_2582	B1	700.3033693	Bcer	J	Bcer	FALSE	NA	NA	NA
Bc_ctg1_2583	B1	106.5786905	Bcer	P	Bcer	FALSE	NA	NA	NA
Bc_ctg1_2584	B1	53.98399219	Bcer	P	Bcer	FALSE	NA	NA	NA
Bc_ctg1_2585	B1	39.99181774	Bcer	P	Bcer	FALSE	NA	NA	NA
Bc_ctg1_2586	B1	72.44175423	Bcer	J	Bcer	FALSE	NA	NA	NA
Bc_ctg1_2587	B1	1929.630015	Bcer	J	Bcer	FALSE	NA	NA	NA
Bc_ctg1_2588	B1	618.2357929	Bcer	J	Bcer	FALSE	NA	NA	NA
Bc_ctg1_2589	B1	0.19847056	Bcer	S	Bcer	FALSE	NA	NA	NA
Bc_ctg1_259	B1	1.686999756	Bcer	NA	Bcer	FALSE	Bc_Ga0417192_01_rn02	Other	NA
Bc_ctg1_2590	B1	4.26711703	Bcer	M	Bcer	FALSE	NA	NA	NA
Bc_ctg1_2591	B1	341.0716565	Bcer	D	Bcer	FALSE	NA	NA	NA
Bc_ctg1_2592	B1	0	Bcer	S	Bcer	FALSE	NA	NA	NA
Bc_ctg1_2593	B1	48.0298754	Bcer	S	Bcer	FALSE	NA	NA	NA
Bc_ctg1_2594	B1	1.488529196	Bcer	G	Bcer	FALSE	NA	NA	NA
Bc_ctg1_2595	B1	0	Bcer	NA	Bcer	FALSE	NA	NA	NA
Bc_ctg1_2597	B1	0.496176399	Bcer	G	Bcer	FALSE	NA	NA	NA
Bc_ctg1_2598	B1	188.4477963	Bcer	E	Bcer	FALSE	NA	NA	NA
Bc_ctg1_2599	B1	152.524625	Bcer	S	Bcer	FALSE	NA	NA	NA
Bc_ctg1_26	B1	158.2802712	Bcer	U	Bcer	FALSE	NA	NA	NA
Bc_ctg1_260	B1	0.297705839	Bcer	S	Bcer	FALSE	Bc_Ga0417192_01_rn02	Other	NA
Bc_ctg1_2600	B1	258.8056096	Bcer	S	Bcer	FALSE	NA	NA	NA
Bc_ctg1_2601	B1	545.3970976	Bcer	G	Bcer	FALSE	NA	NA	NA
Bc_ctg1_2602	B1	786.042651	Bcer	M	Bcer	FALSE	NA	NA	NA
Bc_ctg1_2603	B1	9.824292696	Bcer	EU	Bcer	FALSE	NA	NA	NA
Bc_ctg1_2604	B1	40.88493526	Bcer	S	Bcer	FALSE	NA	NA	NA
Bc_ctg1_2605	B1	735.1349525	Bcer	S	Bcer	FALSE	NA	NA	NA
Bc_ctg1_2606	B1	60.73199121	Bcer	S	Bcer	FALSE	NA	NA	NA
Bc_ctg1_2607	B1	135.2576863	Bcer	IQ	Bcer	TRUE	NA	NA	NA

Bc_ctg1_2608	B1	3.572470071	Bcer	P	Bcer	FALSE	NA	NA	NA
Bc_ctg1_2609	B1	7.144940143	Bcer	P	Bcer	FALSE	NA	NA	NA
Bc_ctg1_261	B1	28.67899585	Bcer	S	Bcer	FALSE	Bc_Ga0417192_01_rn02	Other	NA
Bc_ctg1_2610	B1	5.457940387	Bcer	P	Bcer	FALSE	NA	NA	NA
Bc_ctg1_2611	B1	0.09923528	Bcer	C	Bcer	FALSE	NA	NA	NA
Bc_ctg1_2612	B1	31.3583484	Bcer	NA	Bcer	FALSE	NA	NA	NA
Bc_ctg1_2613	B1	7.938822381	Bcer	C	Bcer	FALSE	NA	NA	NA
Bc_ctg1_2614	B1	1.786235036	Bcer	G	Bcer	FALSE	NA	NA	NA
Bc_ctg1_2615	B1	59.44193258	Bcer	S	Bcer	FALSE	NA	NA	NA
Bc_ctg1_2616	B1	10.81664549	Bcer	EGP	Bcer	FALSE	NA	NA	NA
Bc_ctg1_2617	B1	2.778587833	Bcer	NA	Bcer	FALSE	NA	NA	NA
Bc_ctg1_2618	B1	28.77823113	Bcer	NA	Bcer	FALSE	NA	NA	NA
Bc_ctg1_2619	B1	0.694646958	Bcer	S	Bcer	FALSE	NA	NA	NA
Bc_ctg1_262	B1	18.75546787	Bcer	S	Bcer	FALSE	Bc_Ga0417192_01_rn02	Other	NA
Bc_ctg1_2620	B1	1.587764476	Bcer	P	Bcer	FALSE	NA	NA	NA
Bc_ctg1_2621	B1	1.488529196	Bcer	EP	Bcer	FALSE	NA	NA	NA
Bc_ctg1_2622	B1	2.580117274	Bcer	P	Bcer	FALSE	NA	NA	NA
Bc_ctg1_2623	B1	3.473234792	Bcer	E	Bcer	FALSE	NA	NA	NA
Bc_ctg1_2624	B1	13.29752749	Bcer	E	Bcer	FALSE	NA	NA	NA
Bc_ctg1_2625	B1	0.595411679	Bcer	S	Bcer	FALSE	NA	NA	NA
Bc_ctg1_2626	B1	0.09923528	Bcer	Q	Bcer	TRUE	NA	NA	NA
Bc_ctg1_2627	B1	159.5703299	Bcer	E	Bcer	FALSE	NA	NA	NA
Bc_ctg1_2628	B1	166.1198583	Bcer	E	Bcer	FALSE	NA	NA	NA
Bc_ctg1_2629	B1	132.5783338	Bcer	S	Bcer	FALSE	NA	NA	NA
Bc_ctg1_263	B1	253.5461398	Bcer	S	Bcer	FALSE	Bc_Ga0417192_01_rn02	Other	SMCOG1273
Bc_ctg1_2630	B1	96.35745665	Bcer	E	Bcer	FALSE	NA	NA	NA
Bc_ctg1_2631	B1	7.343410702	Bcer	H	Bcer	FALSE	NA	NA	NA
Bc_ctg1_2632	B1	80.97598829	Bcer	U	Bcer	FALSE	NA	NA	NA
Bc_ctg1_2633	B1	69.66316639	Bcer	P	Bcer	FALSE	NA	NA	NA
Bc_ctg1_2634	B1	142.0056853	Bcer	P	Bcer	FALSE	NA	NA	NA
Bc_ctg1_2635	B1	83.8538114	Bcer	P	Bcer	FALSE	NA	NA	NA
Bc_ctg1_2638	B1	5.755646226	Bcer	K	Bcer	FALSE	NA	NA	NA
Bc_ctg1_2639	B1	4.366352309	Bcer	EG	Bcer	FALSE	NA	NA	NA
Bc_ctg1_264	B1	62.71669681	Bcer	K	Bcer	FALSE	Bc_Ga0417192_01_rn02	Regulation	SMCOG1171
Bc_ctg1_2642	B1	59.73963842	Bcer	I	Bcer	FALSE	NA	NA	NA
Bc_ctg1_2644	B1	28.08358417	Bcer	K	Bcer	FALSE	NA	NA	NA
Bc_ctg1_2645	B1	0.694646958	Bcer	NA	Bcer	FALSE	NA	NA	NA
Bc_ctg1_2646	B1	55.17481555	Bcer	S	Bcer	FALSE	NA	NA	NA
Bc_ctg1_2647	B1	0.09923528	Bcer	S	Bcer	FALSE	NA	NA	NA
Bc_ctg1_2648	B1	0.09923528	Bcer	NA	Bcer	FALSE	NA	NA	NA
Bc_ctg1_2649	B1	7.839587101	Bcer	L	Bcer	FALSE	NA	NA	NA
Bc_ctg1_265	B1	0.09923528	Bcer	NA	Bcer	FALSE	Bc_Ga0417192_01_rn02	Other	NA
Bc_ctg1_2650	B1	35.22852432	Bcer	S	Bcer	FALSE	NA	NA	NA
Bc_ctg1_2651	B1	43.06811142	Bcer	M	Bcer	FALSE	NA	NA	NA
Bc_ctg1_2652	B1	12.60288053	Bcer	C	Bcer	FALSE	NA	NA	NA
Bc_ctg1_2654	B1	13.09905693	Bcer	S	Bcer	FALSE	NA	NA	NA
Bc_ctg1_2655	B1	0	Bcer	NA	Bcer	FALSE	NA	NA	NA
Bc_ctg1_2656	B1	129.105099	Bcer	E	Bcer	FALSE	NA	NA	NA
Bc_ctg1_2657	B1	7.144940143	Bcer	P	Bcer	FALSE	NA	NA	NA
Bc_ctg1_2658	B1	9.427351577	Bcer	EP	Bcer	FALSE	NA	NA	NA
Bc_ctg1_2659	B1	16.671527	Bcer	P	Bcer	FALSE	NA	NA	NA
Bc_ctg1_266	B1	0	Bcer	JM	Bcer	FALSE	Bc_Ga0417192_01_rn02	Ancillary biosynthesis	SMCOG1064
Bc_ctg1_2660	B1	21.13711459	Bcer	E	Bcer	FALSE	NA	NA	NA
Bc_ctg1_2661	B1	270.3169021	Bcer	S	Bcer	FALSE	NA	NA	NA
Bc_ctg1_2663	B1	0.396941119	Bcer	NA	Bcer	FALSE	NA	NA	NA
Bc_ctg1_2664	B1	0	Bcer	NA	Bcer	FALSE	NA	NA	NA
Bc_ctg1_2665	B1	0	Bcer	NA	Bcer	FALSE	NA	NA	NA
Bc_ctg1_2667	B1	66.28916688	Bcer	E	Bcer	FALSE	NA	NA	NA
Bc_ctg1_2668	B1	31.45758368	Bcer	Q	Bcer	TRUE	NA	NA	NA
Bc_ctg1_2669	B1	69.46469583	Bcer	Q	Bcer	TRUE	NA	NA	NA
Bc_ctg1_267	B1	0	Bcer	GM	Bcer	FALSE	Bc_Ga0417192_01_rn02	Core biosynthesis	SMCOG1010
Bc_ctg1_2670	B1	3.473234792	Bcer	E	Bcer	FALSE	NA	NA	NA

Bc_ctg1_2671	B1	5.656410946	Bcer	EGP	Bcer	FALSE	NA	NA	NA
Bc_ctg1_2672	B1	211.5696165	Bcer	F	Bcer	FALSE	NA	NA	NA
Bc_ctg1_2673	B1	152.8223308	Bcer	M	Bcer	FALSE	NA	NA	NA
Bc_ctg1_2674	B1	1446.155732	Bcer	JKL	Bcer	FALSE	NA	NA	NA
Bc_ctg1_2675	B1	5.557175667	Bcer	L	Bcer	FALSE	NA	NA	NA
Bc_ctg1_2676	B1	0.396941119	Bcer	S	Bcer	FALSE	NA	NA	NA
Bc_ctg1_2677	B1	5.358705107	Bcer	I	Bcer	FALSE	NA	NA	NA
Bc_ctg1_2678	B1	19.74782067	Bcer	M	Bcer	FALSE	NA	NA	NA
Bc_ctg1_2679	B1	16.27458588	Bcer	E	Bcer	FALSE	NA	NA	NA
Bc_ctg1_268	B1	0	Bcer	H	Bcer	FALSE	Bc_Ga0417192_01_rn02	Other	NA
Bc_ctg1_2680	B1	398.330413	Bcer	K	Bcer	FALSE	NA	NA	NA
Bc_ctg1_2681	B1	569.7097411	Bcer	L	Bcer	FALSE	NA	NA	NA
Bc_ctg1_2682	B1	165.1275055	Bcer	K	Bcer	FALSE	NA	NA	NA
Bc_ctg1_2683	B1	3.96941119	Bcer	S	Bcer	FALSE	NA	NA	NA
Bc_ctg1_2685	B1	7.244175423	Bcer	S	Bcer	FALSE	NA	NA	NA
Bc_ctg1_2686	B1	30.66370145	Bcer	O	Bcer	FALSE	NA	NA	NA
Bc_ctg1_2687	B1	15.97688004	Bcer	K	Bcer	FALSE	NA	NA	NA
Bc_ctg1_2688	B1	84.8461642	Bcer	O	Bcer	FALSE	NA	NA	NA
Bc_ctg1_2689	B1	160.8603885	Bcer	S	Bcer	FALSE	NA	NA	NA
Bc_ctg1_269	B1	0	Bcer	GM	Bcer	FALSE	Bc_Ga0417192_01_rn02	Core biosynthesis	SMCOG1010
Bc_ctg1_2690	B1	1006.841148	Bcer	K	Bcer	FALSE	NA	NA	NA
Bc_ctg1_2691	B1	7.144940143	Bcer	S	Bcer	FALSE	NA	NA	NA
Bc_ctg1_2692	B1	124.0440997	Bcer	S	Bcer	FALSE	NA	NA	NA
Bc_ctg1_2693	B1	499.6496336	Bcer	O	Bcer	FALSE	NA	NA	NA
Bc_ctg1_2694	B1	2842.197648	Bcer	O	Bcer	FALSE	NA	NA	NA
Bc_ctg1_2695	B1	47.23599317	Bcer	S	Bcer	FALSE	NA	NA	NA
Bc_ctg1_2696	B1	39.29717079	Bcer	S	Bcer	FALSE	NA	NA	NA
Bc_ctg1_2697	B1	1094.565136	Bcer	F	Bcer	FALSE	NA	NA	NA
Bc_ctg1_2698	B1	476.8255193	Bcer	S	Bcer	FALSE	NA	NA	NA
Bc_ctg1_2699	B1	4.564822869	Bcer	T	Bcer	FALSE	NA	NA	NA
Bc_ctg1_27	B1	75.71651846	Bcer	P	Bcer	FALSE	NA	NA	NA
Bc_ctg1_270	B1	0.893117518	Bcer	NA	Bcer	FALSE	Bc_Ga0417192_01_rn02	Other	NA
Bc_ctg1_2700	B1	21.63329099	Bcer	T	Bcer	FALSE	NA	NA	NA
Bc_ctg1_2702	B1	23.32029074	Bcer	S	Bcer	FALSE	NA	NA	NA
Bc_ctg1_2703	B1	38.20558271	Bcer	Q	Bcer	TRUE	NA	NA	NA
Bc_ctg1_2705	B1	14.09140973	Bcer	S	Bcer	FALSE	NA	NA	NA
Bc_ctg1_2707	B1	4.862528708	Bcer	V	Bcer	FALSE	NA	NA	NA
Bc_ctg1_2708	B1	1.984705595	Bcer	S	Bcer	FALSE	NA	NA	NA
Bc_ctg1_2709	B1	2.282411435	Bcer	V	Bcer	FALSE	NA	NA	NA
Bc_ctg1_271	B1	0	Bcer	S	Bcer	FALSE	Bc_Ga0417192_01_rn02	Other	NA
Bc_ctg1_2710	B1	3.175528952	Bcer	T	Bcer	FALSE	NA	NA	NA
Bc_ctg1_2711	B1	2.480881994	Bcer	T	Bcer	FALSE	NA	NA	NA
Bc_ctg1_2713	B1	0.297705839	Bcer	S	Bcer	FALSE	Bc_Ga0417192_01_rn18	Other	NA
Bc_ctg1_2714	B1	0.595411679	Bcer	Q	Bcer	TRUE	Bc_Ga0417192_01_rn18	Ancillary biosynthesis	SMCOG1089
Bc_ctg1_2715	B1	0.595411679	Bcer	Q	Bcer	TRUE	Bc_Ga0417192_01_rn18	Other	NA
Bc_ctg1_2716	B1	0	Bcer	G	Bcer	FALSE	Bc_Ga0417192_01_rn18	Other	NA
Bc_ctg1_2717	B1	5.060999268	Bcer	M	Bcer	FALSE	Bc_Ga0417192_01_rn18	Ancillary biosynthesis	SMCOG1010
Bc_ctg1_2718	B1	1.290058637	Bcer	M	Bcer	FALSE	Bc_Ga0417192_01_rn18	Other	NA
Bc_ctg1_2719	B1	1.885470315	Bcer	M	Bcer	FALSE	Bc_Ga0417192_01_rn18	Core biosynthesis	SMCOG1045
Bc_ctg1_272	B1	33.93846568	Bcer	EG	Bcer	FALSE	Bc_Ga0417192_01_rn02	Transport	SMCOG1166
Bc_ctg1_2721	B1	42.86964086	Bcer	EG	Bcer	FALSE	Bc_Ga0417192_01_rn18	Transport	SMCOG1166
Bc_ctg1_2722	B1	0.09923528	Bcer	G	Bcer	FALSE	Bc_Ga0417192_01_rn18	Other	NA
Bc_ctg1_2724	B1	216.3329099	Bcer	F	Bcer	FALSE	NA	NA	NA
Bc_ctg1_2725	B1	1392.965622	Bcer	F	Bcer	FALSE	NA	NA	NA
Bc_ctg1_2726	B1	1766.685686	Bcer	F	Bcer	FALSE	NA	NA	NA
Bc_ctg1_2727	B1	310.9041315	Bcer	F	Bcer	FALSE	NA	NA	NA
Bc_ctg1_2728	B1	81.57139996	Bcer	F	Bcer	FALSE	NA	NA	NA
Bc_ctg1_2729	B1	422.5438212	Bcer	F	Bcer	FALSE	NA	NA	NA
Bc_ctg1_273	B1	2.778587833	Bcer	S	Bcer	FALSE	Bc_Ga0417192_01_rn02	Other	SMCOG1286
Bc_ctg1_2730	B1	1507.3839	Bcer	F	Bcer	FALSE	NA	NA	NA
Bc_ctg1_2731	B1	1266.24217	Bcer	F	Bcer	FALSE	NA	NA	NA
Bc_ctg1_2732	B1	366.9720646	Bcer	F	Bcer	FALSE	NA	NA	NA

Bc_ctg1_2733	B1	174.4556218	Bcer	F	Bcer	FALSE	NA	NA	NA
Bc_ctg1_2734	B1	911.1783388	Bcer	F	Bcer	FALSE	NA	NA	NA
Bc_ctg1_2735	B1	388.7045908	Bcer	F	Bcer	FALSE	NA	NA	NA
Bc_ctg1_2736	B1	5.755646226	Bcer	EGP	Bcer	FALSE	NA	NA	NA
Bc_ctg1_2737	B1	8.831939899	Bcer	K	Bcer	FALSE	NA	NA	NA
Bc_ctg1_2738	B1	8.3357635	Bcer	S	Bcer	FALSE	NA	NA	NA
Bc_ctg1_2739	B1	179.3181505	Bcer	O	Bcer	FALSE	NA	NA	NA
Bc_ctg1_274	B1	5.656410946	Bcer	S	Bcer	FALSE	Bc_Ga0417192_01_rn02	Other	NA
Bc_ctg1_2740	B1	0	Bcer	S	Bcer	FALSE	NA	NA	NA
Bc_ctg1_2741	B1	19.54935011	Bcer	I	Bcer	FALSE	NA	NA	NA
Bc_ctg1_2742	B1	187.9516199	Bcer	L	Bcer	FALSE	NA	NA	NA
Bc_ctg1_2743	B1	275.1794308	Bcer	L	Bcer	FALSE	NA	NA	NA
Bc_ctg1_2744	B1	173.3640337	Bcer	S	Bcer	FALSE	NA	NA	NA
Bc_ctg1_2745	B1	17.6638798	Bcer	S	Bcer	FALSE	NA	NA	NA
Bc_ctg1_2746	B1	5931.094201	Bcer	C	Bcer	FALSE	NA	NA	NA
Bc_ctg1_2747	B1	8.931175179	Bcer	Q	Bcer	TRUE	NA	NA	NA
Bc_ctg1_2748	B1	39.59487662	Bcer	P	Bcer	FALSE	NA	NA	NA
Bc_ctg1_2749	B1	8.13729294	Bcer	P	Bcer	FALSE	NA	NA	NA
Bc_ctg1_275	B1	55.67099195	Bcer	G	Bcer	FALSE	Bc_Ga0417192_01_rn02	Other	NA
Bc_ctg1_2750	B1	30.46523089	Bcer	M	Bcer	FALSE	NA	NA	NA
Bc_ctg1_2751	B1	0.19847056	Bcer	S	Bcer	FALSE	NA	NA	NA
Bc_ctg1_2752	B1	7.442645982	Bcer	D	Bcer	FALSE	NA	NA	NA
Bc_ctg1_2753	B1	34.8315832	Bcer	K	Bcer	FALSE	NA	NA	NA
Bc_ctg1_2754	B1	9.625822137	Bcer	S	Bcer	FALSE	NA	NA	NA
Bc_ctg1_2755	B1	170.5854459	Bcer	J	Bcer	FALSE	NA	NA	NA
Bc_ctg1_2756	B1	704.9674274	Bcer	J	Bcer	FALSE	NA	NA	NA
Bc_ctg1_2757	B1	868.6064038	Bcer	J	Bcer	FALSE	NA	NA	NA
Bc_ctg1_2758	B1	187.3562082	Bcer	I	Bcer	FALSE	NA	NA	NA
Bc_ctg1_2759	B1	6.351057905	Bcer	S	Bcer	FALSE	NA	NA	NA
Bc_ctg1_276	B1	21.33558515	Bcer	G	Bcer	FALSE	Bc_Ga0417192_01_rn02	Ancillary biosynthesis	SMCOG1210
Bc_ctg1_2760	B1	0.09923528	Bcer	E	Bcer	FALSE	NA	NA	NA
Bc_ctg1_2761	B1	7.938822381	Bcer	KT	Bcer	FALSE	NA	NA	NA
Bc_ctg1_2762	B1	53.09087467	Bcer	C	Bcer	FALSE	NA	NA	NA
Bc_ctg1_2763	B1	8.931175179	Bcer	P	Bcer	FALSE	NA	NA	NA
Bc_ctg1_2764	B1	362.0103006	Bcer	E	Bcer	FALSE	NA	NA	NA
Bc_ctg1_2765	B1	45.25128757	Bcer	G	Bcer	FALSE	Bc_Ga0417192_01_rn19	Other	NA
Bc_ctg1_2766	B1	2.778587833	Bcer	NA	Bcer	FALSE	Bc_Ga0417192_01_rn19	Other	NA
Bc_ctg1_2767	B1	10.41970437	Bcer	NA	Bcer	FALSE	Bc_Ga0417192_01_rn19	Other	NA
Bc_ctg1_2768	B1	11.61052773	Bcer	F	Bcer	FALSE	Bc_Ga0417192_01_rn19	Other	NA
Bc_ctg1_2769	B1	54.87710971	Bcer	J	Bcer	FALSE	Bc_Ga0417192_01_rn19	Other	NA
Bc_ctg1_277	B1	9.129645738	Bcer	EG	Bcer	FALSE	Bc_Ga0417192_01_rn02	Other	NA
Bc_ctg1_2770	B1	43.46505254	Bcer	J	Bcer	FALSE	Bc_Ga0417192_01_rn19	Other	NA
Bc_ctg1_2771	B1	24.70958466	Bcer	V	Bcer	FALSE	Bc_Ga0417192_01_rn19	Transport	SMCOG1288
Bc_ctg1_2772	B1	79.58669437	Bcer	KLT	Bcer	FALSE	Bc_Ga0417192_01_rn19	Core biosynthesis	SMCOG1030
Bc_ctg1_278	B1	28.87746641	Bcer	G	Bcer	FALSE	Bc_Ga0417192_01_rn02	Other	NA
Bc_ctg1_2785	B1	2.580117274	Bcer	S	Bcer	FALSE	Bc_Ga0417192_01_rn19	Other	NA
Bc_ctg1_2786	B1	0.694646958	Bcer	S	Bcer	FALSE	Bc_Ga0417192_01_rn19	Other	NA
Bc_ctg1_2787	B1	9.427351577	Bcer	J	Bcer	FALSE	Bc_Ga0417192_01_rn19	Other	NA
Bc_ctg1_2788	B1	14.38911557	Bcer	M	Bcer	FALSE	Bc_Ga0417192_01_rn19	Ancillary biosynthesis	SMCOG1268
Bc_ctg1_2789	B1	4.961763988	Bcer	KT	Bcer	FALSE	Bc_Ga0417192_01_rn19	Other	NA
Bc_ctg1_279	B1	33.93846568	Bcer	G	Bcer	FALSE	Bc_Ga0417192_01_rn02	Ancillary biosynthesis	SMCOG1100
Bc_ctg1_2790	B1	2.480881994	Bcer	C	Bcer	FALSE	Bc_Ga0417192_01_rn19	Other	NA
Bc_ctg1_2791	B1	51.20540436	Bcer	S	Bcer	FALSE	Bc_Ga0417192_01_rn19	Other	NA
Bc_ctg1_2792	B1	11.61052773	Bcer	M	Bcer	FALSE	NA	NA	NA
Bc_ctg1_2795	B1	6739.861731	Bcer	O	Bcer	FALSE	NA	NA	NA
Bc_ctg1_2796	B1	2810.839299	Bcer	O	Bcer	FALSE	NA	NA	NA
Bc_ctg1_2797	B1	137.7385683	Bcer	F	Bcer	FALSE	NA	NA	NA
Bc_ctg1_2798	B1	158.7764476	Bcer	E	Bcer	FALSE	NA	NA	NA
Bc_ctg1_2799	B1	139.8225092	Bcer	G	Bcer	FALSE	NA	NA	NA
Bc_ctg1_28	B1	13.79370389	Bcer	K	Bcer	FALSE	NA	NA	NA
Bc_ctg1_280	B1	65.39604936	Bcer	G	Bcer	FALSE	NA	NA	NA
Bc_ctg1_2800	B1	0	Bcer	NA	Bcer	FALSE	NA	NA	NA

Bc_ctg1_2801	B1	23.81646714	Bcer	P	Bcer	FALSE	NA	NA	NA
Bc_ctg1_2802	B1	8.534234059	Bcer	P	Bcer	FALSE	NA	NA	NA
Bc_ctg1_2803	B1	236.576907	Bcer	P	Bcer	FALSE	NA	NA	NA
Bc_ctg1_2804	B1	286.2937821	Bcer	C	Bcer	FALSE	NA	NA	NA
Bc_ctg1_2805	B1	204.6231469	Bcer	NA	Bcer	FALSE	NA	NA	NA
Bc_ctg1_2806	B1	1.984705595	Bcer	G	Bcer	FALSE	NA	NA	NA
Bc_ctg1_2807	B1	0.396941119	Bcer	NA	Bcer	FALSE	NA	NA	NA
Bc_ctg1_2808	B1	0	Bcer	NA	Bcer	FALSE	NA	NA	NA
Bc_ctg1_2809	B1	1.190823357	Bcer	NA	Bcer	FALSE	NA	NA	NA
Bc_ctg1_281	B1	40.58722942	Bcer	G	Bcer	FALSE	NA	NA	NA
Bc_ctg1_2810	B1	6.053352065	Bcer	E	Bcer	FALSE	NA	NA	NA
Bc_ctg1_2811	B1	33.04534816	Bcer	T	Bcer	FALSE	NA	NA	NA
Bc_ctg1_2812	B1	9.328116298	Bcer	S	Bcer	FALSE	NA	NA	NA
Bc_ctg1_2813	B1	15.48070364	Bcer	S	Bcer	FALSE	NA	NA	NA
Bc_ctg1_2814	B1	0.496176399	Bcer	F	Bcer	FALSE	NA	NA	NA
Bc_ctg1_2815	B1	61.22816761	Bcer	S	Bcer	FALSE	NA	NA	NA
Bc_ctg1_2816	B1	2.480881994	Bcer	EGP	Bcer	FALSE	NA	NA	NA
Bc_ctg1_2817	B1	0.595411679	Bcer	CE	Bcer	FALSE	NA	NA	NA
Bc_ctg1_2818	B1	824.0497631	Bcer	I	Bcer	FALSE	NA	NA	NA
Bc_ctg1_2819	B1	1.885470315	Bcer	P	Bcer	FALSE	NA	NA	NA
Bc_ctg1_282	B1	0	Bcer	G	Bcer	FALSE	NA	NA	NA
Bc_ctg1_2820	B1	1.587764476	Bcer	ET	Bcer	FALSE	NA	NA	NA
Bc_ctg1_2821	B1	0.793882238	Bcer	E	Bcer	FALSE	NA	NA	NA
Bc_ctg1_2822	B1	63.90752017	Bcer	NT	Bcer	FALSE	NA	NA	NA
Bc_ctg1_2823	B1	61.42663817	Bcer	K	Bcer	FALSE	NA	NA	NA
Bc_ctg1_2824	B1	3961.373133	Bcer	E	Bcer	FALSE	NA	NA	NA
Bc_ctg1_2825	B1	1537.352954	Bcer	E	Bcer	FALSE	NA	NA	NA
Bc_ctg1_2826	B1	1973.789714	Bcer	E	Bcer	FALSE	NA	NA	NA
Bc_ctg1_2827	B1	941.7428049	Bcer	E	Bcer	FALSE	Bc_Ga0417192_01_rn20	Other	NA
Bc_ctg1_2828	B1	351.1936551	Bcer	K	Bcer	FALSE	Bc_Ga0417192_01_rn20	Regulation	SMCOG1133
Bc_ctg1_2829	B1	33.24381872	Bcer	NA	Bcer	FALSE	Bc_Ga0417192_01_rn20	Other	NA
Bc_ctg1_283	B1	35.03005376	Bcer	C	Bcer	FALSE	NA	NA	NA
Bc_ctg1_2830	B1	0	Bcer	C	Bcer	FALSE	Bc_Ga0417192_01_rn20	Other	NA
Bc_ctg1_2831	B1	279.3473125	Bcer	G	Bcer	FALSE	Bc_Ga0417192_01_rn20	Ancillary biosynthesis	SMCOG1257
Bc_ctg1_2832	B1	311.0033668	Bcer	G	Bcer	FALSE	Bc_Ga0417192_01_rn20	Ancillary biosynthesis	SMCOG1145
Bc_ctg1_2833	B1	183.1883264	Bcer	L	Bcer	FALSE	Bc_Ga0417192_01_rn20	Other	NA
Bc_ctg1_2834	B1	229.3327315	Bcer	S	Bcer	FALSE	Bc_Ga0417192_01_rn20	Other	NA
Bc_ctg1_2835	B1	165.3259761	Bcer	L	Bcer	FALSE	Bc_Ga0417192_01_rn20	Other	NA
Bc_ctg1_2836	B1	19.15240899	Bcer	H	Bcer	FALSE	Bc_Ga0417192_01_rn20	Other	NA
Bc_ctg1_2837	B1	11.31282189	Bcer	H	Bcer	FALSE	Bc_Ga0417192_01_rn20	Ancillary biosynthesis	SMCOG1280
Bc_ctg1_2838	B1	6.351057905	Bcer	S	Bcer	FALSE	Bc_Ga0417192_01_rn20	Other	NA
Bc_ctg1_2839	B1	70.95322503	Bcer	NT	Bcer	FALSE	Bc_Ga0417192_01_rn20	Other	SMCOG1061
Bc_ctg1_284	B1	12.80135109	Bcer	E	Bcer	FALSE	NA	NA	NA
Bc_ctg1_2840	B1	20.64093819	Bcer	Q	Bcer	TRUE	Bc_Ga0417192_01_rn20	Core biosynthesis	SMCOG1002
Bc_ctg1_2841	B1	21.13711459	Bcer	S	Bcer	FALSE	Bc_Ga0417192_01_rn20	Other	NA
Bc_ctg1_2842	B1	69.66316639	Bcer	P	Bcer	FALSE	Bc_Ga0417192_01_rn20	Transport	SMCOG1044
Bc_ctg1_2843	B1	54.38093331	Bcer	P	Bcer	FALSE	Bc_Ga0417192_01_rn20	Other	NA
Bc_ctg1_2844	B1	69.16698999	Bcer	P	Bcer	FALSE	Bc_Ga0417192_01_rn20	Transport	SMCOG1000
Bc_ctg1_2845	B1	4.366352309	Bcer	S	Bcer	FALSE	Bc_Ga0417192_01_rn20	Transport	SMCOG1000
Bc_ctg1_2846	B1	0.793882238	Bcer	G	Bcer	FALSE	Bc_Ga0417192_01_rn20	Other	NA
Bc_ctg1_2847	B1	7.343410702	Bcer	S	Bcer	FALSE	Bc_Ga0417192_01_rn20	Other	NA
Bc_ctg1_2848	B1	17.26693868	Bcer	CO	Bcer	FALSE	Bc_Ga0417192_01_rn20	Other	NA
Bc_ctg1_2849	B1	3.274764232	Bcer	NA	Bcer	FALSE	Bc_Ga0417192_01_rn20	Other	NA
Bc_ctg1_285	B1	11.01511605	Bcer	S	Bcer	FALSE	NA	NA	NA
Bc_ctg1_2852	B1	12.70211581	Bcer	K	Bcer	FALSE	Bc_Ga0417192_01_rn20	Regulation	SMCOG1057
Bc_ctg1_2853	B1	1.885470315	Bcer	G	Bcer	FALSE	Bc_Ga0417192_01_rn20	Transport	SMCOG1020
Bc_ctg1_2854	B1	2.480881994	Bcer	K	Bcer	FALSE	Bc_Ga0417192_01_rn20	Other	NA
Bc_ctg1_2855	B1	32.74764232	Bcer	S	Bcer	FALSE	Bc_Ga0417192_01_rn20	Transport	SMCOG1106
Bc_ctg1_2856	B1	13.89293917	Bcer	S	Bcer	FALSE	Bc_Ga0417192_01_rn20	Other	NA
Bc_ctg1_2857	B1	9.725057417	Bcer	S	Bcer	FALSE	Bc_Ga0417192_01_rn20	Other	NA
Bc_ctg1_2858	B1	182.2952089	Bcer	J	Bcer	FALSE	Bc_Ga0417192_01_rn20	Other	SMCOG1162
Bc_ctg1_2859	B1	0	Bcer	NU	Bcer	FALSE	Bc_Ga0417192_01_rn20	Other	NA

Bc_ctg1_286	B1	0.297705839	Bcer	S	Bcer	FALSE	NA	NA	NA
Bc_ctg1_2860	B1	2.679352554	Bcer	GK	Bcer	FALSE	Bc_Ga0417192_01_rn20	Other	SMCOG1108
Bc_ctg1_2861	B1	751.9057148	Bcer	T	Bcer	FALSE	Bc_Ga0417192_01_rn20	Other	SMCOG1296
Bc_ctg1_2862	B1	862.9499928	Bcer	T	Bcer	FALSE	Bc_Ga0417192_01_rn20	Other	SMCOG1296
Bc_ctg1_2863	B1	179.7150916	Bcer	T	Bcer	FALSE	NA	NA	NA
Bc_ctg1_2864	B1	384.2390032	Bcer	P	Bcer	FALSE	NA	NA	NA
Bc_ctg1_2865	B1	183.1883264	Bcer	O	Bcer	FALSE	NA	NA	NA
Bc_ctg1_2866	B1	256.9201393	Bcer	P	Bcer	FALSE	NA	NA	NA
Bc_ctg1_2867	B1	621.4113219	Bcer	P	Bcer	FALSE	NA	NA	NA
Bc_ctg1_2868	B1	30.76293673	Bcer	S	Bcer	FALSE	NA	NA	NA
Bc_ctg1_2869	B1	32.74764232	Bcer	T	Bcer	FALSE	NA	NA	NA
Bc_ctg1_287	B1	0.893117518	Bcer	S	Bcer	FALSE	NA	NA	NA
Bc_ctg1_2870	B1	103.204691	Bcer	NA	Bcer	FALSE	NA	NA	NA
Bc_ctg1_2871	B1	155.2039775	Bcer	S	Bcer	FALSE	NA	NA	NA
Bc_ctg1_2872	B1	41.87728806	Bcer	P	Bcer	FALSE	NA	NA	NA
Bc_ctg1_2873	B1	1.091588077	Bcer	EG	Bcer	FALSE	NA	NA	NA
Bc_ctg1_2874	B1	1.587764476	Bcer	EG	Bcer	FALSE	NA	NA	NA
Bc_ctg1_2875	B1	43.96122893	Bcer	S	Bcer	FALSE	NA	NA	NA
Bc_ctg1_2876	B1	11.21358661	Bcer	U	Bcer	FALSE	NA	NA	NA
Bc_ctg1_2877	B1	1071.34408	Bcer	S	Bcer	FALSE	NA	NA	NA
Bc_ctg1_2878	B1	21.63329099	Bcer	S	Bcer	FALSE	NA	NA	NA
Bc_ctg1_2879	B1	2.679352554	Bcer	S	Bcer	FALSE	NA	NA	NA
Bc_ctg1_288	B1	1.389293917	Bcer	S	Bcer	FALSE	NA	NA	NA
Bc_ctg1_2880	B1	0	Bcer	S	Bcer	FALSE	NA	NA	NA
Bc_ctg1_2881	B1	0.09923528	Bcer	S	Bcer	FALSE	NA	NA	NA
Bc_ctg1_2882	B1	0.694646958	Bcer	P	Bcer	FALSE	NA	NA	NA
Bc_ctg1_2883	B1	4.16788175	Bcer	S	Bcer	FALSE	NA	NA	NA
Bc_ctg1_2884	B1	93.08269242	Bcer	S	Bcer	FALSE	NA	NA	NA
Bc_ctg1_2885	B1	170.7839165	Bcer	NA	Bcer	FALSE	NA	NA	NA
Bc_ctg1_2886	B1	519.1989837	Bcer	C	Bcer	FALSE	NA	NA	NA
Bc_ctg1_2887	B1	15.28223308	Bcer	G	Bcer	FALSE	NA	NA	NA
Bc_ctg1_2888	B1	51.60234548	Bcer	L	Bcer	FALSE	NA	NA	NA
Bc_ctg1_2889	B1	117.6930418	Bcer	J	Bcer	FALSE	NA	NA	NA
Bc_ctg1_2890	B1	23.61799658	Bcer	J	Bcer	FALSE	NA	NA	NA
Bc_ctg1_2891	B1	43.86199365	Bcer	NA	Bcer	FALSE	NA	NA	NA
Bc_ctg1_2892	B1	16.37382116	Bcer	KT	Bcer	FALSE	NA	NA	NA
Bc_ctg1_2893	B1	0	Bcer	I	Bcer	FALSE	NA	NA	NA
Bc_ctg1_2894	B1	2.480881994	Bcer	E	Bcer	FALSE	Bc_Ga0417192_01_rn21	Ancillary biosynthesis	SMCOG1038
Bc_ctg1_2895	B1	6.251822625	Bcer	E	Bcer	FALSE	Bc_Ga0417192_01_rn21	Ancillary biosynthesis	SMCOG1247
Bc_ctg1_2896	B1	0	Bcer	K	Bcer	FALSE	Bc_Ga0417192_01_rn21	Other	NA
Bc_ctg1_2897	B1	0.595411679	Bcer	P	Bcer	FALSE	Bc_Ga0417192_01_rn21	Other	NA
Bc_ctg1_2898	B1	0.396941119	Bcer	S	Bcer	FALSE	Bc_Ga0417192_01_rn21	Other	NA
Bc_ctg1_2899	B1	14.78605668	Bcer	E	Bcer	FALSE	Bc_Ga0417192_01_rn21	Other	NA
Bc_ctg1_29	B1	8.13729294	Bcer	S	Bcer	FALSE	NA	NA	NA
Bc_ctg1_290	B1	402.0021183	Bcer	E	Bcer	FALSE	NA	NA	NA
Bc_ctg1_2900	B1	168.501505	Bcer	G	Bcer	FALSE	Bc_Ga0417192_01_rn21	Ancillary biosynthesis	SMCOG1145
Bc_ctg1_2901	B1	180.905915	Bcer	V	Bcer	FALSE	Bc_Ga0417192_01_rn21	Ancillary biosynthesis	SMCOG1053
Bc_ctg1_2902	B1	0.595411679	Bcer	NA	Bcer	FALSE	Bc_Ga0417192_01_rn21	Other	NA
Bc_ctg1_2903	B1	0.09923528	Bcer	NA	Bcer	FALSE	Bc_Ga0417192_01_rn21	Other	NA
Bc_ctg1_2904	B1	0.19847056	Bcer	M	Bcer	FALSE	Bc_Ga0417192_01_rn21	Core biosynthesis	SMCOG1123
Bc_ctg1_2905	B1	0	Bcer	M	Bcer	FALSE	Bc_Ga0417192_01_rn21	Ancillary biosynthesis	SMCOG1152
Bc_ctg1_2906	B1	1.587764476	Bcer	M	Bcer	FALSE	Bc_Ga0417192_01_rn21	Core biosynthesis	SMCOG1010
Bc_ctg1_2907	B1	10.12199854	Bcer	M	Bcer	FALSE	Bc_Ga0417192_01_rn21	Core biosynthesis	SMCOG1123
Bc_ctg1_2908	B1	0	Bcer	NA	Bcer	FALSE	Bc_Ga0417192_01_rn21	Other	NA
Bc_ctg1_2909	B1	31087.93227	Bcer	C	Bcer	FALSE	Bc_Ga0417192_01_rn21	Other	NA
Bc_ctg1_291	B1	10.61817493	Bcer	S	Bcer	FALSE	NA	NA	NA
Bc_ctg1_2910	B1	8511.608416	Bcer	C	Bcer	FALSE	Bc_Ga0417192_01_rn21	Ancillary biosynthesis	None
Bc_ctg1_2911	B1	209.6841461	Bcer	M	Bcer	FALSE	Bc_Ga0417192_01_rn21	Core biosynthesis	None
Bc_ctg1_2913	B1	40.28952358	Bcer	J	Bcer	FALSE	Bc_Ga0417192_01_rn21	Other	NA
Bc_ctg1_2914	B1	12.00746885	Bcer	Q	Bcer	TRUE	Bc_Ga0417192_01_rn21	Other	NA
Bc_ctg1_2915	B1	22.82411435	Bcer	S	Bcer	FALSE	Bc_Ga0417192_01_rn21	Ancillary biosynthesis	SMCOG1010
Bc_ctg1_2916	B1	101.9146323	Bcer	S	Bcer	FALSE	Bc_Ga0417192_01_rn21	Other	NA

Bc_ctg1_2917	B1	25.80117274	Bcer	S	Bcer	FALSE	Bc_Ga0417192_01_rn21	Other	NA
Bc_ctg1_2919	B1	0	Bcer	NA	Bcer	FALSE	Bc_Ga0417192_01_rn21	Other	NA
Bc_ctg1_292	B1	8.3357635	Bcer	K	Bcer	FALSE	NA	NA	NA
Bc_ctg1_2920	B1	4.564822869	Bcer	S	Bcer	FALSE	Bc_Ga0417192_01_rn21	Other	NA
Bc_ctg1_2921	B1	60.43428537	Bcer	S	Bcer	FALSE	Bc_Ga0417192_01_rn21	Other	NA
Bc_ctg1_2922	B1	14.09140973	Bcer	L	Bcer	FALSE	Bc_Ga0417192_01_rn21	Other	NA
Bc_ctg1_2924	B1	0.992352798	Bcer	S	Bcer	FALSE	Bc_Ga0417192_01_rn21	Other	NA
Bc_ctg1_2925	B1	0.297705839	Bcer	S	Bcer	FALSE	Bc_Ga0417192_01_rn21	Other	NA
Bc_ctg1_2926	B1	133.4714513	Bcer	J	Bcer	FALSE	Bc_Ga0417192_01_rn21	Other	NA
Bc_ctg1_2927	B1	23.12182018	Bcer	V	Bcer	FALSE	NA	NA	NA
Bc_ctg1_2928	B1	96.65516249	Bcer	S	Bcer	FALSE	NA	NA	NA
Bc_ctg1_2929	B1	81.17445884	Bcer	E	Bcer	FALSE	NA	NA	NA
Bc_ctg1_293	B1	0	Bcer	NA	Bcer	FALSE	NA	NA	NA
Bc_ctg1_2930	B1	274.4847838	Bcer	H	Bcer	FALSE	NA	NA	NA
Bc_ctg1_2931	B1	8.23652822	Bcer	S	Bcer	FALSE	NA	NA	NA
Bc_ctg1_2932	B1	4.06864647	Bcer	S	Bcer	FALSE	NA	NA	NA
Bc_ctg1_2933	B1	9.328116298	Bcer	S	Bcer	FALSE	NA	NA	NA
Bc_ctg1_2934	B1	6.450293184	Bcer	P	Bcer	FALSE	NA	NA	NA
Bc_ctg1_2935	B1	36.02240655	Bcer	O	Bcer	FALSE	NA	NA	NA
Bc_ctg1_2936	B1	916.7355144	Bcer	P	Bcer	FALSE	NA	NA	NA
Bc_ctg1_2937	B1	22.62564379	Bcer	K	Bcer	FALSE	NA	NA	NA
Bc_ctg1_2938	B1	10.22123382	Bcer	S	Bcer	FALSE	NA	NA	NA
Bc_ctg1_294	B1	20.54170291	Bcer	K	Bcer	FALSE	NA	NA	NA
Bc_ctg1_2940	B1	0.992352798	Bcer	NA	Bcer	FALSE	NA	NA	NA
Bc_ctg1_2942	B1	296.6142512	Bcer	K	Bcer	FALSE	NA	NA	NA
Bc_ctg1_2943	B1	0.595411679	Bcer	F	Bcer	FALSE	NA	NA	NA
Bc_ctg1_2944	B1	54.38093331	Bcer	M	Bcer	FALSE	NA	NA	NA
Bc_ctg1_2945	B1	110.2503958	Bcer	O	Bcer	FALSE	NA	NA	NA
Bc_ctg1_2946	B1	20.44246763	Bcer	C	Bcer	FALSE	NA	NA	NA
Bc_ctg1_2947	B1	16.671527	Bcer	S	Bcer	FALSE	Bc_Ga0417192_01_rn22	Other	NA
Bc_ctg1_2948	B1	279.5457831	Bcer	J	Bcer	FALSE	Bc_Ga0417192_01_rn22	Other	NA
Bc_ctg1_2949	B1	74.92263622	Bcer	T	Bcer	FALSE	Bc_Ga0417192_01_rn22	Ancillary biosynthesis	SMCOG1025
Bc_ctg1_295	B1	12.70211581	Bcer	K	Bcer	FALSE	NA	NA	NA
Bc_ctg1_2951	B1	18.35852676	Bcer	T	Bcer	FALSE	Bc_Ga0417192_01_rn22	Other	NA
Bc_ctg1_2952	B1	8.931175179	Bcer	S	Bcer	FALSE	Bc_Ga0417192_01_rn22	Other	NA
Bc_ctg1_2953	B1	551.946626	Bcer	E	Bcer	FALSE	Bc_Ga0417192_01_rn22	Transport	SMCOG1202
Bc_ctg1_2954	B1	48.92299292	Bcer	NA	Bcer	FALSE	Bc_Ga0417192_01_rn22	Other	NA
Bc_ctg1_2955	B1	117.0976301	Bcer	H	Bcer	FALSE	Bc_Ga0417192_01_rn22	Ancillary biosynthesis	SMCOG1012
Bc_ctg1_2956	B1	109.3572783	Bcer	T	Bcer	FALSE	Bc_Ga0417192_01_rn22	Regulation	SMCOG1048
Bc_ctg1_2957	B1	67.38075496	Bcer	KT	Bcer	FALSE	Bc_Ga0417192_01_rn22	Regulation	SMCOG1016
Bc_ctg1_2958	B1	2590.338508	Bcer	V	Bcer	FALSE	Bc_Ga0417192_01_rn22	Transport	SMCOG1000
Bc_ctg1_2959	B1	3753.475222	Bcer	V	Bcer	FALSE	Bc_Ga0417192_01_rn22	Other	NA
Bc_ctg1_296	B1	23.22105546	Bcer	S	Bcer	FALSE	NA	NA	NA
Bc_ctg1_2960	B1	2720.237489	Bcer	V	Bcer	FALSE	Bc_Ga0417192_01_rn22	Other	NA
Bc_ctg1_2961	B1	13.19829221	Bcer	NA	Bcer	FALSE	Bc_Ga0417192_01_rn22	Other	NA
Bc_ctg1_2962	B1	519.298219	Bcer	Q	Bcer	TRUE	Bc_Ga0417192_01_rn22	Ancillary biosynthesis	SMCOG1004
Bc_ctg1_2963	B1	2321.807841	Bcer	Q	Bcer	TRUE	Bc_Ga0417192_01_rn22	Core biosynthesis	SMCOG1002
Bc_ctg1_2964	B1	3030.248503	Bcer	Q	Bcer	TRUE	Bc_Ga0417192_01_rn22	Core biosynthesis	SMCOG1209
Bc_ctg1_2965	B1	289.7670169	Bcer	I	Bcer	FALSE	Bc_Ga0417192_01_rn22	Core biosynthesis	SMCOG1021
Bc_ctg1_2966	B1	1570.398302	Bcer	Q	Bcer	TRUE	Bc_Ga0417192_01_rn22	Core biosynthesis	SMCOG1022
Bc_ctg1_2967	B1	1066.481552	Bcer	Q	Bcer	TRUE	Bc_Ga0417192_01_rn22	Core biosynthesis	SMCOG1022
Bc_ctg1_2968	B1	213.9512632	Bcer	Q	Bcer	TRUE	Bc_Ga0417192_01_rn22	Ancillary biosynthesis	SMCOG1222
Bc_ctg1_2969	B1	1477.216375	Bcer	Q	Bcer	TRUE	Bc_Ga0417192_01_rn22	Core biosynthesis	SMCOG1022
Bc_ctg1_297	B1	0	Bcer	K	Bcer	FALSE	NA	NA	NA
Bc_ctg1_2970	B1	346.7280675	Bcer	Q	Bcer	TRUE	Bc_Ga0417192_01_rn22	Ancillary biosynthesis	SMCOG1042
Bc_ctg1_2971	B1	12.20593941	Bcer	M	Bcer	FALSE	Bc_Ga0417192_01_rn22	Other	NA
Bc_ctg1_2972	B1	2.877823113	Bcer	K	Bcer	FALSE	Bc_Ga0417192_01_rn22	Other	NA
Bc_ctg1_2973	B1	45.35052285	Bcer	M	Bcer	FALSE	Bc_Ga0417192_01_rn22	Other	NA
Bc_ctg1_2974	B1	279.6450184	Bcer	S	Bcer	FALSE	Bc_Ga0417192_01_rn22	Other	NA
Bc_ctg1_2975	B1	62.31975569	Bcer	OU	Bcer	FALSE	Bc_Ga0417192_01_rn22	Other	NA
Bc_ctg1_2976	B1	91.6933985	Bcer	S	Bcer	FALSE	Bc_Ga0417192_01_rn22	Other	NA
Bc_ctg1_2977	B1	150.1429783	Bcer	NT	Bcer	FALSE	Bc_Ga0417192_01_rn22	Other	SMCOG1061

Bc_ctg1_2978	B1	16.57229172	Bcer	T	Bcer	FALSE	Bc_Ga0417192_01_rn22	Other	NA
Bc_ctg1_2979	B1	9.923527976	Bcer	KT	Bcer	FALSE	Bc_Ga0417192_01_rn22	Regulation	SMCOG1008
Bc_ctg1_298	B1	3.770940631	Bcer	C	Bcer	FALSE	NA	NA	NA
Bc_ctg1_2980	B1	7.740351821	Bcer	C	Bcer	FALSE	Bc_Ga0417192_01_rn22	Other	NA
Bc_ctg1_2981	B1	0	Bcer	I	Bcer	FALSE	Bc_Ga0417192_01_rn22	Other	NA
Bc_ctg1_2982	B1	2.083940875	Bcer	C	Bcer	FALSE	Bc_Ga0417192_01_rn22	Other	NA
Bc_ctg1_2983	B1	57.65569754	Bcer	S	Bcer	FALSE	NA	NA	NA
Bc_ctg1_2984	B1	0	Bcer	S	Bcer	FALSE	NA	NA	NA
Bc_ctg1_2985	B1	0.19847056	Bcer	K	Bcer	FALSE	NA	NA	NA
Bc_ctg1_2986	B1	0.396941119	Bcer	P	Bcer	FALSE	NA	NA	NA
Bc_ctg1_2987	B1	0.496176399	Bcer	G	Bcer	FALSE	NA	NA	NA
Bc_ctg1_2988	B1	0.793882238	Bcer	P	Bcer	FALSE	NA	NA	NA
Bc_ctg1_2989	B1	9.824292696	Bcer	G	Bcer	FALSE	NA	NA	NA
Bc_ctg1_299	B1	0.496176399	Bcer	S	Bcer	FALSE	NA	NA	NA
Bc_ctg1_2990	B1	4.564822869	Bcer	T	Bcer	FALSE	NA	NA	NA
Bc_ctg1_2991	B1	3.870175911	Bcer	T	Bcer	FALSE	NA	NA	NA
Bc_ctg1_2992	B1	14.48835085	Bcer	T	Bcer	FALSE	NA	NA	NA
Bc_ctg1_2993	B1	0.694646958	Bcer	NA	Bcer	FALSE	NA	NA	NA
Bc_ctg1_2994	B1	1.190823357	Bcer	M	Bcer	FALSE	NA	NA	NA
Bc_ctg1_2995	B1	38.20558271	Bcer	NT	Bcer	FALSE	NA	NA	NA
Bc_ctg1_2996	B1	7.045704863	Bcer	T	Bcer	FALSE	NA	NA	NA
Bc_ctg1_2997	B1	12.60288053	Bcer	T	Bcer	FALSE	NA	NA	NA
Bc_ctg1_2998	B1	1.488529196	Bcer	C	Bcer	FALSE	NA	NA	NA
Bc_ctg1_2999	B1	2.381646714	Bcer	C	Bcer	FALSE	NA	NA	NA
Bc_ctg1_3	B1	19.64858539	Bcer	L	Bcer	FALSE	NA	NA	NA
Bc_ctg1_30	B1	52.29699243	Bcer	I	Bcer	FALSE	NA	NA	NA
Bc_ctg1_300	B1	6.450293184	Bcer	O	Bcer	FALSE	NA	NA	NA
Bc_ctg1_3000	B1	0.297705839	Bcer	NA	Bcer	FALSE	NA	NA	NA
Bc_ctg1_3001	B1	0.19847056	Bcer	EG	Bcer	FALSE	NA	NA	NA
Bc_ctg1_3002	B1	8.13729294	Bcer	K	Bcer	FALSE	NA	NA	NA
Bc_ctg1_3003	B1	42.96887614	Bcer	K	Bcer	FALSE	NA	NA	NA
Bc_ctg1_3004	B1	26.9919961	Bcer	T	Bcer	FALSE	NA	NA	NA
Bc_ctg1_3005	B1	27.98434889	Bcer	KT	Bcer	FALSE	NA	NA	NA
Bc_ctg1_3006	B1	0	Bcer	NA	Bcer	FALSE	NA	NA	NA
Bc_ctg1_3007	B1	4.366352309	Bcer	M	Bcer	FALSE	NA	NA	NA
Bc_ctg1_3008	B1	27.78587833	Bcer	C	Bcer	FALSE	NA	NA	NA
Bc_ctg1_3009	B1	878.0337553	Bcer	C	Bcer	FALSE	NA	NA	NA
Bc_ctg1_301	B1	26.9919961	Bcer	E	Bcer	FALSE	Bc_Ga0417192_01_rn03	Other	NA
Bc_ctg1_3010	B1	179.7150916	Bcer	S	Bcer	FALSE	NA	NA	NA
Bc_ctg1_3011	B1	65.6937552	Bcer	C	Bcer	FALSE	NA	NA	NA
Bc_ctg1_3012	B1	1167.106125	Bcer	E	Bcer	FALSE	NA	NA	NA
Bc_ctg1_3013	B1	542.5192745	Bcer	E	Bcer	FALSE	NA	NA	NA
Bc_ctg1_3014	B1	69.96087223	Bcer	K	Bcer	FALSE	NA	NA	NA
Bc_ctg1_3015	B1	1269.417699	Bcer	P	Bcer	FALSE	NA	NA	NA
Bc_ctg1_3016	B1	105.9832788	Bcer	H	Bcer	FALSE	NA	NA	NA
Bc_ctg1_3017	B1	572.0913878	Bcer	K	Bcer	FALSE	NA	NA	NA
Bc_ctg1_3019	B1	15.1829978	Bcer	E	Bcer	FALSE	NA	NA	NA
Bc_ctg1_302	B1	35.82393599	Bcer	S	Bcer	FALSE	Bc_Ga0417192_01_rn03	Other	NA
Bc_ctg1_3020	B1	11.61052773	Bcer	S	Bcer	FALSE	NA	NA	NA
Bc_ctg1_3021	B1	28.38129001	Bcer	NA	Bcer	FALSE	NA	NA	NA
Bc_ctg1_3022	B1	6.351057905	Bcer	NA	Bcer	FALSE	NA	NA	NA
Bc_ctg1_3023	B1	18.6562326	Bcer	V	Bcer	FALSE	NA	NA	NA
Bc_ctg1_3024	B1	22.52640851	Bcer	K	Bcer	FALSE	NA	NA	NA
Bc_ctg1_3025	B1	0.19847056	Bcer	F	Bcer	FALSE	NA	NA	NA
Bc_ctg1_3026	B1	2.282411435	Bcer	E	Bcer	FALSE	NA	NA	NA
Bc_ctg1_3027	B1	15.97688004	Bcer	S	Bcer	FALSE	NA	NA	NA
Bc_ctg1_3028	B1	192.9133839	Bcer	E	Bcer	FALSE	NA	NA	NA
Bc_ctg1_3029	B1	2347.112837	Bcer	C	Bcer	FALSE	NA	NA	NA
Bc_ctg1_303	B1	36.32011239	Bcer	S	Bcer	FALSE	Bc_Ga0417192_01_rn03	Ancillary biosynthesis	None
Bc_ctg1_3030	B1	312.9880724	Bcer	K	Bcer	FALSE	Bc_Ga0417192_01_rn23	Regulation	SMCOG1058
Bc_ctg1_3031	B1	0	Bcer	S	Bcer	FALSE	Bc_Ga0417192_01_rn23	Other	NA
Bc_ctg1_3032	B1	4983.198808	Bcer	E	Bcer	FALSE	Bc_Ga0417192_01_rn23	Transport	SMCOG1202

Bc_ctg1_3033	B1	298.301251	Bcer	P	Bcer	FALSE	Bc_Ga0417192_01_rn23	Transport	SMCOG1033
Bc_ctg1_3034	B1	102.9069851	Bcer	P	Bcer	FALSE	Bc_Ga0417192_01_rn23	Transport	SMCOG1011
Bc_ctg1_3035	B1	70.95322503	Bcer	P	Bcer	FALSE	Bc_Ga0417192_01_rn23	Transport	SMCOG1011
Bc_ctg1_3036	B1	128.5096873	Bcer	HP	Bcer	FALSE	Bc_Ga0417192_01_rn23	Transport	SMCOG1000
Bc_ctg1_3037	B1	20.34323235	Bcer	Q	Bcer	TRUE	Bc_Ga0417192_01_rn23	Ancillary biosynthesis	SMCOG1248
Bc_ctg1_3038	B1	1372.324684	Bcer	H	Bcer	FALSE	Bc_Ga0417192_01_rn23	Ancillary biosynthesis	SMCOG1109
Bc_ctg1_3039	B1	1219.303882	Bcer	GM	Bcer	FALSE	Bc_Ga0417192_01_rn23	Core biosynthesis	SMCOG1010
Bc_ctg1_304	B1	3.076293673	Bcer	S	Bcer	FALSE	Bc_Ga0417192_01_rn03	Other	NA
Bc_ctg1_3040	B1	7.343410702	Bcer	F	Bcer	FALSE	Bc_Ga0417192_01_rn23	Other	NA
Bc_ctg1_3041	B1	1.389293917	Bcer	F	Bcer	FALSE	Bc_Ga0417192_01_rn23	Other	NA
Bc_ctg1_3042	B1	89.21251651	Bcer	P	Bcer	FALSE	Bc_Ga0417192_01_rn23	Other	NA
Bc_ctg1_3043	B1	23.5187613	Bcer	S	Bcer	FALSE	Bc_Ga0417192_01_rn23	Other	NA
Bc_ctg1_3044	B1	45.64822869	Bcer	I	Bcer	FALSE	Bc_Ga0417192_01_rn23	Other	NA
Bc_ctg1_3046	B1	40.88493526	Bcer	T	Bcer	FALSE	Bc_Ga0417192_01_rn23	Ancillary biosynthesis	SMCOG1025
Bc_ctg1_3047	B1	21.13711459	Bcer	E	Bcer	FALSE	Bc_Ga0417192_01_rn23	Ancillary biosynthesis	SMCOG1038
Bc_ctg1_3048	B1	43.36581726	Bcer	K	Bcer	FALSE	Bc_Ga0417192_01_rn23	Regulation	SMCOG1136
Bc_ctg1_3049	B1	3418.556152	Bcer	G	Bcer	FALSE	Bc_Ga0417192_01_rn23	Other	NA
Bc_ctg1_3050	B1	3676.270174	Bcer	G	Bcer	FALSE	Bc_Ga0417192_01_rn23	Core biosynthesis	SMCOG1257
Bc_ctg1_3051	B1	1.885470315	Bcer	S	Bcer	FALSE	Bc_Ga0417192_01_rn23	Other	NA
Bc_ctg1_3052	B1	0.297705839	Bcer	E	Bcer	FALSE	Bc_Ga0417192_01_rn23	Other	NA
Bc_ctg1_3053	B1	0	Bcer	EG	Bcer	FALSE	Bc_Ga0417192_01_rn23	Other	NA
Bc_ctg1_3054	B1	97.54828001	Bcer	S	Bcer	FALSE	Bc_Ga0417192_01_rn23	Ancillary biosynthesis	None
Bc_ctg1_3055	B1	3.671705351	Bcer	E	Bcer	FALSE	Bc_Ga0417192_01_rn23	Ancillary biosynthesis	SMCOG1153
Bc_ctg1_3056	B1	0	Bcer	E	Bcer	FALSE	Bc_Ga0417192_01_rn23	Transport	SMCOG1000
Bc_ctg1_3057	B1	0.893117518	Bcer	ET	Bcer	FALSE	Bc_Ga0417192_01_rn23	Ancillary biosynthesis	SMCOG1091
Bc_ctg1_3058	B1	0.09923528	Bcer	P	Bcer	FALSE	Bc_Ga0417192_01_rn23	Transport	SMCOG1096
Bc_ctg1_3059	B1	0.19847056	Bcer	P	Bcer	FALSE	Bc_Ga0417192_01_rn23	Transport	SMCOG1096
Bc_ctg1_306	B1	4.465587589	Bcer	S	Bcer	FALSE	Bc_Ga0417192_01_rn03	Other	NA
Bc_ctg1_3060	B1	123.5479233	Bcer	E	Bcer	FALSE	NA	NA	NA
Bc_ctg1_3061	B1	53.09087467	Bcer	O	Bcer	FALSE	NA	NA	NA
Bc_ctg1_3062	B1	4.06864647	Bcer	FG	Bcer	FALSE	NA	NA	NA
Bc_ctg1_3063	B1	1.290058637	Bcer	K	Bcer	FALSE	NA	NA	NA
Bc_ctg1_3064	B1	5.259469827	Bcer	NA	Bcer	FALSE	NA	NA	NA
Bc_ctg1_3065	B1	2.679352554	Bcer	K	Bcer	FALSE	NA	NA	NA
Bc_ctg1_3066	B1	0.893117518	Bcer	H	Bcer	FALSE	NA	NA	NA
Bc_ctg1_3067	B1	149.4483313	Bcer	T	Bcer	FALSE	NA	NA	NA
Bc_ctg1_3068	B1	99.5329856	Bcer	KT	Bcer	FALSE	NA	NA	NA
Bc_ctg1_3069	B1	2605.918447	Bcer	NA	Bcer	FALSE	NA	NA	NA
Bc_ctg1_3070	B1	36.02240655	Bcer	P	Bcer	FALSE	NA	NA	NA
Bc_ctg1_3071	B1	26.09887858	Bcer	T	Bcer	FALSE	NA	NA	NA
Bc_ctg1_3073	B1	108.2656902	Bcer	E	Bcer	FALSE	NA	NA	NA
Bc_ctg1_3074	B1	23.22105546	Bcer	EP	Bcer	FALSE	NA	NA	NA
Bc_ctg1_3075	B1	24.31264354	Bcer	P	Bcer	FALSE	NA	NA	NA
Bc_ctg1_3076	B1	17.6638798	Bcer	P	Bcer	FALSE	NA	NA	NA
Bc_ctg1_3077	B1	14.98452724	Bcer	P	Bcer	FALSE	NA	NA	NA
Bc_ctg1_3078	B1	20.44246763	Bcer	G	Bcer	FALSE	NA	NA	NA
Bc_ctg1_3079	B1	40.88493526	Bcer	K	Bcer	FALSE	NA	NA	NA
Bc_ctg1_308	B1	19.84705595	Bcer	IQ	Bcer	TRUE	Bc_Ga0417192_01_rn03	Ancillary biosynthesis	SMCOG1002
Bc_ctg1_3080	B1	417.0858808	Bcer	P	Bcer	FALSE	NA	NA	NA
Bc_ctg1_3081	B1	73.13640118	Bcer	K	Bcer	FALSE	NA	NA	NA
Bc_ctg1_3082	B1	59.64040314	Bcer	H	Bcer	FALSE	NA	NA	NA
Bc_ctg1_3083	B1	11.01511605	Bcer	G	Bcer	FALSE	NA	NA	NA
Bc_ctg1_3084	B1	112.6320425	Bcer	G	Bcer	FALSE	NA	NA	NA
Bc_ctg1_3085	B1	200.752971	Bcer	G	Bcer	FALSE	NA	NA	NA
Bc_ctg1_3086	B1	411.0325288	Bcer	G	Bcer	FALSE	NA	NA	NA
Bc_ctg1_3087	B1	627.861615	Bcer	G	Bcer	FALSE	NA	NA	NA
Bc_ctg1_3088	B1	677.9754313	Bcer	V	Bcer	FALSE	NA	NA	NA
Bc_ctg1_3089	B1	0	Bcer	NA	Bcer	FALSE	NA	NA	NA
Bc_ctg1_309	B1	16.47305644	Bcer	S	Bcer	FALSE	Bc_Ga0417192_01_rn03	Other	NA
Bc_ctg1_3090	B1	0.496176399	Bcer	NA	Bcer	FALSE	NA	NA	NA
Bc_ctg1_3091	B1	45.44975813	Bcer	EGP	Bcer	FALSE	NA	NA	NA
Bc_ctg1_3092	B1	4090.279761	Bcer	E	Bcer	FALSE	NA	NA	NA

Bc_ctg1_3093	B1	3.473234792	Bcer	P	Bcer	FALSE	NA	NA	NA
Bc_ctg1_3094	B1	696.5324286	Bcer	M	Bcer	FALSE	NA	NA	NA
Bc_ctg1_3095	B1	83.15916444	Bcer	S	Bcer	FALSE	NA	NA	NA
Bc_ctg1_3096	B1	2.083940875	Bcer	E	Bcer	FALSE	NA	NA	NA
Bc_ctg1_3097	B1	6.747999024	Bcer	C	Bcer	FALSE	NA	NA	NA
Bc_ctg1_3098	B1	6.251822625	Bcer	V	Bcer	FALSE	NA	NA	NA
Bc_ctg1_3099	B1	12.30517469	Bcer	NA	Bcer	FALSE	NA	NA	NA
Bc_ctg1_31	B1	22.72487907	Bcer	S	Bcer	FALSE	NA	NA	NA
Bc_ctg1_310	B1	6.053352065	Bcer	K	Bcer	FALSE	Bc_Ga0417192_01_rn03	Regulation	SMCOG1167
Bc_ctg1_3100	B1	0.694646958	Bcer	NA	Bcer	FALSE	NA	NA	NA
Bc_ctg1_3101	B1	10.71741021	Bcer	V	Bcer	FALSE	NA	NA	NA
Bc_ctg1_3102	B1	130.196687	Bcer	NT	Bcer	FALSE	NA	NA	NA
Bc_ctg1_3103	B1	292.64484	Bcer	S	Bcer	FALSE	NA	NA	NA
Bc_ctg1_3104	B1	1.290058637	Bcer	K	Bcer	FALSE	NA	NA	NA
Bc_ctg1_3105	B1	3.373999512	Bcer	NA	Bcer	FALSE	NA	NA	NA
Bc_ctg1_3106	B1	9.427351577	Bcer	M	Bcer	FALSE	NA	NA	NA
Bc_ctg1_3107	B1	3.076293673	Bcer	L	Bcer	FALSE	NA	NA	NA
Bc_ctg1_3108	B1	183.0890912	Bcer	E	Bcer	FALSE	NA	NA	NA
Bc_ctg1_3109	B1	3.870175911	Bcer	E	Bcer	FALSE	NA	NA	NA
Bc_ctg1_311	B1	0	Bcer	NA	Bcer	FALSE	Bc_Ga0417192_01_rn03	Other	NA
Bc_ctg1_3110	B1	79.28898853	Bcer	S	Bcer	FALSE	NA	NA	NA
Bc_ctg1_3111	B1	102.1131029	Bcer	G	Bcer	FALSE	NA	NA	NA
Bc_ctg1_3112	B1	12.99982165	Bcer	E	Bcer	FALSE	NA	NA	NA
Bc_ctg1_3113	B1	47.33522845	Bcer	S	Bcer	FALSE	NA	NA	NA
Bc_ctg1_3114	B1	0	Bcer	K	Bcer	FALSE	NA	NA	NA
Bc_ctg1_3115	B1	2.679352554	Bcer	NA	Bcer	FALSE	NA	NA	NA
Bc_ctg1_3116	B1	10.12199854	Bcer	K	Bcer	FALSE	NA	NA	NA
Bc_ctg1_3117	B1	2.381646714	Bcer	S	Bcer	FALSE	NA	NA	NA
Bc_ctg1_3118	B1	18.1600562	Bcer	P	Bcer	FALSE	NA	NA	NA
Bc_ctg1_3119	B1	78.09816517	Bcer	C	Bcer	FALSE	NA	NA	NA
Bc_ctg1_312	B1	1.488529196	Bcer	K	Bcer	FALSE	Bc_Ga0417192_01_rn03	Regulation	SMCOG1058
Bc_ctg1_3120	B1	420.6583509	Bcer	C	Bcer	FALSE	NA	NA	NA
Bc_ctg1_3121	B1	1553.726775	Bcer	C	Bcer	FALSE	NA	NA	NA
Bc_ctg1_3122	B1	767.1879478	Bcer	C	Bcer	FALSE	NA	NA	NA
Bc_ctg1_3123	B1	30.46523089	Bcer	E	Bcer	FALSE	NA	NA	NA
Bc_ctg1_3124	B1	19.54935011	Bcer	F	Bcer	FALSE	NA	NA	NA
Bc_ctg1_3125	B1	0.893117518	Bcer	E	Bcer	FALSE	NA	NA	NA
Bc_ctg1_3126	B1	0	Bcer	S	Bcer	FALSE	NA	NA	NA
Bc_ctg1_3127	B1	5.954116786	Bcer	E	Bcer	FALSE	NA	NA	NA
Bc_ctg1_3128	B1	0	Bcer	EG	Bcer	FALSE	NA	NA	NA
Bc_ctg1_3129	B1	0.09923528	Bcer	E	Bcer	FALSE	NA	NA	NA
Bc_ctg1_3130	B1	0.297705839	Bcer	S	Bcer	FALSE	NA	NA	NA
Bc_ctg1_3131	B1	2080.368405	Bcer	P	Bcer	FALSE	NA	NA	NA
Bc_ctg1_3132	B1	1600.565827	Bcer	P	Bcer	FALSE	NA	NA	NA
Bc_ctg1_3133	B1	51.0069338	Bcer	P	Bcer	FALSE	NA	NA	NA
Bc_ctg1_3134	B1	2.480881994	Bcer	P	Bcer	FALSE	NA	NA	NA
Bc_ctg1_3135	B1	1.290058637	Bcer	P	Bcer	FALSE	NA	NA	NA
Bc_ctg1_3136	B1	5.954116786	Bcer	P	Bcer	FALSE	NA	NA	NA
Bc_ctg1_3137	B1	0.694646958	Bcer	S	Bcer	FALSE	NA	NA	NA
Bc_ctg1_3138	B1	12.20593941	Bcer	V	Bcer	FALSE	NA	NA	NA
Bc_ctg1_314	B1	0.09923528	Bcer	L	Bcer	FALSE	Bc_Ga0417192_01_rn03	Other	SMCOG1026
Bc_ctg1_3140	B1	13.39676277	Bcer	T	Bcer	FALSE	NA	NA	NA
Bc_ctg1_3141	B1	133.4714513	Bcer	S	Bcer	FALSE	NA	NA	NA
Bc_ctg1_3142	B1	65.1975788	Bcer	NA	Bcer	FALSE	NA	NA	NA
Bc_ctg1_3143	B1	372.2315344	Bcer	MT	Bcer	FALSE	NA	NA	NA
Bc_ctg1_3144	B1	0.297705839	Bcer	K	Bcer	FALSE	NA	NA	NA
Bc_ctg1_3145	B1	1.587764476	Bcer	P	Bcer	FALSE	NA	NA	NA
Bc_ctg1_3146	B1	0.793882238	Bcer	P	Bcer	FALSE	NA	NA	NA
Bc_ctg1_3147	B1	1.190823357	Bcer	P	Bcer	FALSE	NA	NA	NA
Bc_ctg1_3148	B1	0.595411679	Bcer	H	Bcer	FALSE	NA	NA	NA
Bc_ctg1_3149	B1	0.893117518	Bcer	E	Bcer	FALSE	NA	NA	NA
Bc_ctg1_315	B1	1.984705595	Bcer	M	Bcer	FALSE	Bc_Ga0417192_01_rn03	Core biosynthesis	SMCOG1123

Bc_ctg1_3150	B1	0.19847056	Bcer	H	Bcer	FALSE	NA	NA	NA
Bc_ctg1_3151	B1	2.877823113	Bcer	H	Bcer	FALSE	NA	NA	NA
Bc_ctg1_3152	B1	4.16788175	Bcer	H	Bcer	FALSE	NA	NA	NA
Bc_ctg1_3153	B1	2.679352554	Bcer	H	Bcer	FALSE	NA	NA	NA
Bc_ctg1_3154	B1	10.02276326	Bcer	NA	Bcer	FALSE	NA	NA	NA
Bc_ctg1_3155	B1	8.3357635	Bcer	NA	Bcer	FALSE	NA	NA	NA
Bc_ctg1_3157	B1	425.0247032	Bcer	P	Bcer	FALSE	NA	NA	NA
Bc_ctg1_3158	B1	529.5194528	Bcer	P	Bcer	FALSE	NA	NA	NA
Bc_ctg1_3159	B1	83.65534084	Bcer	P	Bcer	FALSE	NA	NA	NA
Bc_ctg1_316	B1	0.297705839	Bcer	S	Bcer	FALSE	Bc_Ga0417192_01_rn03	Other	NA
Bc_ctg1_3160	B1	9.526586857	Bcer	T	Bcer	FALSE	NA	NA	NA
Bc_ctg1_3161	B1	0	Bcer	EGP	Bcer	FALSE	NA	NA	NA
Bc_ctg1_3162	B1	5.160234548	Bcer	K	Bcer	FALSE	NA	NA	NA
Bc_ctg1_3163	B1	55.07558027	Bcer	S	Bcer	FALSE	NA	NA	NA
Bc_ctg1_3164	B1	2.183176155	Bcer	NA	Bcer	FALSE	NA	NA	NA
Bc_ctg1_3165	B1	0.396941119	Bcer	V	Bcer	FALSE	NA	NA	NA
Bc_ctg1_3166	B1	2.282411435	Bcer	V	Bcer	FALSE	NA	NA	NA
Bc_ctg1_3167	B1	1.290058637	Bcer	V	Bcer	FALSE	NA	NA	NA
Bc_ctg1_3168	B1	0.297705839	Bcer	NA	Bcer	FALSE	NA	NA	NA
Bc_ctg1_3169	B1	9.625822137	Bcer	S	Bcer	FALSE	NA	NA	NA
Bc_ctg1_317	B1	0.09923528	Bcer	K	Bcer	FALSE	Bc_Ga0417192_01_rn03	Other	NA
Bc_ctg1_3170	B1	0	Bcer	IQ	Bcer	TRUE	NA	NA	NA
Bc_ctg1_3171	B1	1.290058637	Bcer	S	Bcer	FALSE	NA	NA	NA
Bc_ctg1_3172	B1	0	Bcer	NA	Bcer	FALSE	NA	NA	NA
Bc_ctg1_3173	B1	11.70976301	Bcer	S	Bcer	FALSE	NA	NA	NA
Bc_ctg1_3174	B1	31.55681896	Bcer	CO	Bcer	FALSE	NA	NA	NA
Bc_ctg1_3175	B1	19.25164427	Bcer	C	Bcer	FALSE	NA	NA	NA
Bc_ctg1_3176	B1	1.885470315	Bcer	S	Bcer	FALSE	NA	NA	NA
Bc_ctg1_3177	B1	28.18281945	Bcer	S	Bcer	FALSE	NA	NA	NA
Bc_ctg1_3178	B1	0.19847056	Bcer	E	Bcer	FALSE	NA	NA	NA
Bc_ctg1_3179	B1	0.09923528	Bcer	S	Bcer	FALSE	NA	NA	NA
Bc_ctg1_318	B1	18.45776204	Bcer	M	Bcer	FALSE	Bc_Ga0417192_01_rn03	Core biosynthesis	None
Bc_ctg1_3180	B1	0.595411679	Bcer	P	Bcer	FALSE	NA	NA	NA
Bc_ctg1_3181	B1	0.595411679	Bcer	NA	Bcer	FALSE	NA	NA	NA
Bc_ctg1_3182	B1	25.5034669	Bcer	C	Bcer	FALSE	NA	NA	NA
Bc_ctg1_3183	B1	0.09923528	Bcer	S	Bcer	FALSE	NA	NA	NA
Bc_ctg1_3184	B1	11.11435133	Bcer	S	Bcer	FALSE	NA	NA	NA
Bc_ctg1_3185	B1	9.228881018	Bcer	M	Bcer	FALSE	NA	NA	NA
Bc_ctg1_3186	B1	2.580117274	Bcer	NA	Bcer	FALSE	NA	NA	NA
Bc_ctg1_3187	B1	7.938822381	Bcer	P	Bcer	FALSE	NA	NA	NA
Bc_ctg1_3188	B1	33.64075984	Bcer	S	Bcer	FALSE	NA	NA	NA
Bc_ctg1_3189	B1	0.694646958	Bcer	OP	Bcer	FALSE	NA	NA	NA
Bc_ctg1_319	B1	10.81664549	Bcer	E	Bcer	FALSE	Bc_Ga0417192_01_rn03	Transport	SMCOG1116
Bc_ctg1_3190	B1	0.793882238	Bcer	P	Bcer	FALSE	NA	NA	NA
Bc_ctg1_3191	B1	0	Bcer	O	Bcer	FALSE	NA	NA	NA
Bc_ctg1_3192	B1	0	Bcer	P	Bcer	FALSE	NA	NA	NA
Bc_ctg1_3193	B1	0	Bcer	P	Bcer	FALSE	NA	NA	NA
Bc_ctg1_3194	B1	0	Bcer	O	Bcer	FALSE	NA	NA	NA
Bc_ctg1_3195	B1	17.36617396	Bcer	S	Bcer	FALSE	NA	NA	NA
Bc_ctg1_3196	B1	191.3256194	Bcer	NA	Bcer	FALSE	NA	NA	NA
Bc_ctg1_3197	B1	472.6576375	Bcer	K	Bcer	FALSE	NA	NA	NA
Bc_ctg1_3198	B1	50.11381628	Bcer	C	Bcer	FALSE	NA	NA	NA
Bc_ctg1_32	B1	29.57211337	Bcer	J	Bcer	FALSE	NA	NA	NA
Bc_ctg1_320	B1	36.51858295	Bcer	S	Bcer	FALSE	Bc_Ga0417192_01_rn03	Other	NA
Bc_ctg1_3201	B1	7.740351821	Bcer	P	Bcer	FALSE	NA	NA	NA
Bc_ctg1_3202	B1	12.70211581	Bcer	EGP	Bcer	FALSE	NA	NA	NA
Bc_ctg1_3203	B1	189.2416785	Bcer	S	Bcer	FALSE	NA	NA	NA
Bc_ctg1_3204	B1	26.09887858	Bcer	K	Bcer	FALSE	NA	NA	NA
Bc_ctg1_3205	B1	5.358705107	Bcer	G	Bcer	FALSE	NA	NA	NA
Bc_ctg1_3206	B1	14.19064501	Bcer	G	Bcer	FALSE	NA	NA	NA
Bc_ctg1_3207	B1	42.1749939	Bcer	G	Bcer	FALSE	NA	NA	NA
Bc_ctg1_3208	B1	5.656410946	Bcer	NA	Bcer	FALSE	NA	NA	NA

Bc_ctg1_3209	B1	24.70958466	Bcer	S	Bcer	FALSE	NA	NA	NA
Bc_ctg1_321	B1	0.595411679	Bcer	NA	Bcer	FALSE	Bc_Ga0417192_01_rn03	Other	NA
Bc_ctg1_3210	B1	1336.004571	Bcer	M	Bcer	FALSE	NA	NA	NA
Bc_ctg1_3211	B1	78.19740045	Bcer	V	Bcer	FALSE	NA	NA	NA
Bc_ctg1_3212	B1	45.15205229	Bcer	V	Bcer	FALSE	NA	NA	NA
Bc_ctg1_3213	B1	44.55664061	Bcer	M	Bcer	FALSE	NA	NA	NA
Bc_ctg1_3214	B1	9.923527976	Bcer	S	Bcer	FALSE	NA	NA	NA
Bc_ctg1_3215	B1	0	Bcer	EP	Bcer	FALSE	NA	NA	NA
Bc_ctg1_3216	B1	1.786235036	Bcer	EP	Bcer	FALSE	NA	NA	NA
Bc_ctg1_3217	B1	34.43464208	Bcer	E	Bcer	FALSE	NA	NA	NA
Bc_ctg1_3218	B1	0	Bcer	P	Bcer	FALSE	NA	NA	NA
Bc_ctg1_322	B1	0.396941119	Bcer	NA	Bcer	FALSE	Bc_Ga0417192_01_rn03	Other	NA
Bc_ctg1_3220	B1	0	Bcer	S	Bcer	FALSE	NA	NA	NA
Bc_ctg1_3221	B1	0.09923528	Bcer	E	Bcer	FALSE	NA	NA	NA
Bc_ctg1_3222	B1	1.786235036	Bcer	S	Bcer	FALSE	NA	NA	NA
Bc_ctg1_3223	B1	0.595411679	Bcer	L	Bcer	FALSE	NA	NA	NA
Bc_ctg1_3224	B1	0.396941119	Bcer	S	Bcer	FALSE	NA	NA	NA
Bc_ctg1_3225	B1	1.984705595	Bcer	S	Bcer	FALSE	NA	NA	NA
Bc_ctg1_3226	B1	0.694646958	Bcer	S	Bcer	FALSE	NA	NA	NA
Bc_ctg1_3227	B1	1.091588077	Bcer	S	Bcer	FALSE	NA	NA	NA
Bc_ctg1_3228	B1	0.396941119	Bcer	NA	Bcer	FALSE	NA	NA	NA
Bc_ctg1_3229	B1	4.06864647	Bcer	S	Bcer	FALSE	NA	NA	NA
Bc_ctg1_323	B1	10.81664549	Bcer	K	Bcer	FALSE	Bc_Ga0417192_01_rn03	Other	NA
Bc_ctg1_3230	B1	8.3357635	Bcer	NA	Bcer	FALSE	NA	NA	NA
Bc_ctg1_3231	B1	8.3357635	Bcer	NA	Bcer	FALSE	NA	NA	NA
Bc_ctg1_3232	B1	0.893117518	Bcer	E	Bcer	FALSE	NA	NA	NA
Bc_ctg1_3233	B1	0.595411679	Bcer	P	Bcer	FALSE	NA	NA	NA
Bc_ctg1_3234	B1	7.839587101	Bcer	NA	Bcer	FALSE	NA	NA	NA
Bc_ctg1_3235	B1	57.65569754	Bcer	K	Bcer	FALSE	NA	NA	NA
Bc_ctg1_3236	B1	25.9996433	Bcer	G	Bcer	FALSE	NA	NA	NA
Bc_ctg1_3237	B1	231.5159077	Bcer	G	Bcer	FALSE	NA	NA	NA
Bc_ctg1_3238	B1	168.6007403	Bcer	S	Bcer	FALSE	NA	NA	NA
Bc_ctg1_3239	B1	0	Bcer	S	Bcer	FALSE	NA	NA	NA
Bc_ctg1_324	B1	0.19847056	Bcer	K	Bcer	FALSE	Bc_Ga0417192_01_rn03	Other	NA
Bc_ctg1_3241	B1	0	Bcer	NA	Bcer	FALSE	NA	NA	NA
Bc_ctg1_3242	B1	9.228881018	Bcer	EG	Bcer	FALSE	NA	NA	NA
Bc_ctg1_3243	B1	3.175528952	Bcer	K	Bcer	FALSE	NA	NA	NA
Bc_ctg1_3244	B1	0.297705839	Bcer	E	Bcer	FALSE	NA	NA	NA
Bc_ctg1_3245	B1	1.290058637	Bcer	S	Bcer	FALSE	NA	NA	NA
Bc_ctg1_3246	B1	0.09923528	Bcer	K	Bcer	FALSE	NA	NA	NA
Bc_ctg1_3247	B1	2.877823113	Bcer	P	Bcer	FALSE	NA	NA	NA
Bc_ctg1_3248	B1	2.282411435	Bcer	P	Bcer	FALSE	NA	NA	NA
Bc_ctg1_3249	B1	34.03770096	Bcer	K	Bcer	FALSE	NA	NA	NA
Bc_ctg1_325	B1	2.083940875	Bcer	V	Bcer	FALSE	Bc_Ga0417192_01_rn03	Transport	SMCOG1065
Bc_ctg1_3250	B1	30.36599561	Bcer	EGP	Bcer	FALSE	NA	NA	NA
Bc_ctg1_3251	B1	0.496176399	Bcer	NA	Bcer	FALSE	NA	NA	NA
Bc_ctg1_3252	B1	12.99982165	Bcer	T	Bcer	FALSE	NA	NA	NA
Bc_ctg1_3253	B1	29.37364281	Bcer	T	Bcer	FALSE	NA	NA	NA
Bc_ctg1_3254	B1	0.19847056	Bcer	M	Bcer	FALSE	NA	NA	NA
Bc_ctg1_3255	B1	0.793882238	Bcer	V	Bcer	FALSE	NA	NA	NA
Bc_ctg1_3256	B1	1.587764476	Bcer	V	Bcer	FALSE	NA	NA	NA
Bc_ctg1_3257	B1	0.09923528	Bcer	V	Bcer	FALSE	NA	NA	NA
Bc_ctg1_3258	B1	0.694646958	Bcer	V	Bcer	FALSE	NA	NA	NA
Bc_ctg1_3259	B1	4.16788175	Bcer	S	Bcer	FALSE	NA	NA	NA
Bc_ctg1_326	B1	0.992352798	Bcer	V	Bcer	FALSE	Bc_Ga0417192_01_rn03	Transport	SMCOG1000
Bc_ctg1_3260	B1	3.373999512	Bcer	K	Bcer	FALSE	NA	NA	NA
Bc_ctg1_3261	B1	0.297705839	Bcer	S	Bcer	FALSE	NA	NA	NA
Bc_ctg1_3262	B1	0.893117518	Bcer	S	Bcer	FALSE	NA	NA	NA
Bc_ctg1_3263	B1	2.083940875	Bcer	S	Bcer	FALSE	NA	NA	NA
Bc_ctg1_3264	B1	9.427351577	Bcer	S	Bcer	FALSE	NA	NA	NA
Bc_ctg1_3265	B1	35.3277596	Bcer	E	Bcer	FALSE	NA	NA	NA
Bc_ctg1_3266	B1	0.09923528	Bcer	NA	Bcer	FALSE	NA	NA	NA

Bc_ctg1_3267	B1	4.366352309	Bcer	S	Bcer	FALSE	NA	NA	NA
Bc_ctg1_3268	B1	27.38893721	Bcer	P	Bcer	FALSE	NA	NA	NA
Bc_ctg1_3269	B1	58.8465209	Bcer	U	Bcer	FALSE	NA	NA	NA
Bc_ctg1_327	B1	7.938822381	Bcer	S	Bcer	FALSE	Bc_Ga0417192_01_rn03	Other	NA
Bc_ctg1_3270	B1	33.73999512	Bcer	E	Bcer	FALSE	NA	NA	NA
Bc_ctg1_3271	B1	7.244175423	Bcer	S	Bcer	FALSE	NA	NA	NA
Bc_ctg1_3272	B1	357.544713	Bcer	C	Bcer	FALSE	NA	NA	NA
Bc_ctg1_3273	B1	13.79370389	Bcer	V	Bcer	FALSE	NA	NA	NA
Bc_ctg1_3274	B1	26.9919961	Bcer	V	Bcer	FALSE	NA	NA	NA
Bc_ctg1_3275	B1	177.7303861	Bcer	ET	Bcer	FALSE	NA	NA	NA
Bc_ctg1_3276	B1	94.47198633	Bcer	P	Bcer	FALSE	NA	NA	NA
Bc_ctg1_3277	B1	142.2041559	Bcer	E	Bcer	FALSE	NA	NA	NA
Bc_ctg1_3278	B1	0	Bcer	S	Bcer	FALSE	NA	NA	NA
Bc_ctg1_3279	B1	0	Bcer	NA	Bcer	FALSE	NA	NA	NA
Bc_ctg1_328	B1	26.9919961	Bcer	K	Bcer	FALSE	Bc_Ga0417192_01_rn03	Other	NA
Bc_ctg1_3280	B1	0.396941119	Bcer	HJ	Bcer	FALSE	NA	NA	NA
Bc_ctg1_3281	B1	0.09923528	Bcer	HJ	Bcer	FALSE	NA	NA	NA
Bc_ctg1_3282	B1	8.43499878	Bcer	S	Bcer	FALSE	NA	NA	NA
Bc_ctg1_3283	B1	275.1794308	Bcer	S	Bcer	FALSE	NA	NA	NA
Bc_ctg1_3284	B1	9.725057417	Bcer	NA	Bcer	FALSE	NA	NA	NA
Bc_ctg1_3285	B1	6.351057905	Bcer	S	Bcer	FALSE	NA	NA	NA
Bc_ctg1_3286	B1	66.28916688	Bcer	L	Bcer	FALSE	NA	NA	NA
Bc_ctg1_3287	B1	240.0501417	Bcer	EH	Bcer	FALSE	NA	NA	NA
Bc_ctg1_3288	B1	99.5329856	Bcer	Q	Bcer	TRUE	NA	NA	NA
Bc_ctg1_3289	B1	1.786235036	Bcer	L	Bcer	FALSE	NA	NA	NA
Bc_ctg1_329	B1	304.5530736	Bcer	C	Bcer	FALSE	Bc_Ga0417192_01_rn03	Other	NA
Bc_ctg1_3290	B1	1.091588077	Bcer	S	Bcer	FALSE	NA	NA	NA
Bc_ctg1_3291	B1	63.70904961	Bcer	M	Bcer	FALSE	NA	NA	NA
Bc_ctg1_3292	B1	205.3177938	Bcer	M	Bcer	FALSE	NA	NA	NA
Bc_ctg1_3293	B1	220.2030858	Bcer	H	Bcer	FALSE	NA	NA	NA
Bc_ctg1_3294	B1	6.847234304	Bcer	IQ	Bcer	TRUE	NA	NA	NA
Bc_ctg1_3295	B1	0.09923528	Bcer	NA	Bcer	FALSE	NA	NA	NA
Bc_ctg1_3296	B1	74.22798926	Bcer	NA	Bcer	FALSE	NA	NA	NA
Bc_ctg1_3297	B1	52.09852187	Bcer	P	Bcer	FALSE	NA	NA	NA
Bc_ctg1_3298	B1	99.43375032	Bcer	S	Bcer	FALSE	NA	NA	NA
Bc_ctg1_3299	B1	34.63311264	Bcer	S	Bcer	FALSE	NA	NA	NA
Bc_ctg1_3300	B1	150.2422136	Bcer	G	Bcer	FALSE	NA	NA	NA
Bc_ctg1_3301	B1	55.47252139	Bcer	M	Bcer	FALSE	NA	NA	NA
Bc_ctg1_3302	B1	71.64787199	Bcer	I	Bcer	FALSE	NA	NA	NA
Bc_ctg1_3303	B1	3.274764232	Bcer	NA	Bcer	FALSE	NA	NA	NA
Bc_ctg1_3304	B1	3.274764232	Bcer	I	Bcer	FALSE	NA	NA	NA
Bc_ctg1_3305	B1	0.793882238	Bcer	NA	Bcer	FALSE	NA	NA	NA
Bc_ctg1_3306	B1	102.9069851	Bcer	G	Bcer	FALSE	NA	NA	NA
Bc_ctg1_3307	B1	111.0442781	Bcer	M	Bcer	FALSE	NA	NA	NA
Bc_ctg1_3308	B1	21.33558515	Bcer	L	Bcer	FALSE	NA	NA	NA
Bc_ctg1_331	B1	0.992352798	Bcer	S	Bcer	FALSE	NA	NA	NA
Bc_ctg1_3310	B1	0.496176399	Bcer	E	Bcer	FALSE	NA	NA	NA
Bc_ctg1_3311	B1	3.770940631	Bcer	E	Bcer	FALSE	NA	NA	NA
Bc_ctg1_3312	B1	14.6868214	Bcer	NA	Bcer	FALSE	NA	NA	NA
Bc_ctg1_3313	B1	77.99892989	Bcer	E	Bcer	FALSE	NA	NA	NA
Bc_ctg1_3314	B1	10.61817493	Bcer	P	Bcer	FALSE	NA	NA	NA
Bc_ctg1_3315	B1	8.931175179	Bcer	EP	Bcer	FALSE	NA	NA	NA
Bc_ctg1_3316	B1	19.45011483	Bcer	P	Bcer	FALSE	NA	NA	NA
Bc_ctg1_3317	B1	29.96905449	Bcer	E	Bcer	FALSE	NA	NA	NA
Bc_ctg1_3318	B1	0	Bcer	O	Bcer	FALSE	NA	NA	NA
Bc_ctg1_3319	B1	0.694646958	Bcer	S	Bcer	FALSE	NA	NA	NA
Bc_ctg1_332	B1	4.763293429	Bcer	M	Bcer	FALSE	NA	NA	NA
Bc_ctg1_3320	B1	0	Bcer	S	Bcer	FALSE	NA	NA	NA
Bc_ctg1_3321	B1	2.083940875	Bcer	S	Bcer	FALSE	NA	NA	NA
Bc_ctg1_3322	B1	1.587764476	Bcer	NA	Bcer	FALSE	NA	NA	NA
Bc_ctg1_3324	B1	0	Bcer	NA	Bcer	FALSE	NA	NA	NA
Bc_ctg1_3325	B1	1.290058637	Bcer	V	Bcer	FALSE	NA	NA	NA

Bc_ctg1_3326	B1	0	Bcer	EGP	Bcer	FALSE	NA	NA	NA
Bc_ctg1_3327	B1	4.862528708	Bcer	T	Bcer	FALSE	NA	NA	NA
Bc_ctg1_3328	B1	0.595411679	Bcer	T	Bcer	FALSE	NA	NA	NA
Bc_ctg1_3329	B1	0.595411679	Bcer	S	Bcer	FALSE	NA	NA	NA
Bc_ctg1_333	B1	0	Bcer	S	Bcer	FALSE	NA	NA	NA
Bc_ctg1_3330	B1	0	Bcer	NA	Bcer	FALSE	NA	NA	NA
Bc_ctg1_3331	B1	6.946469583	Bcer	L	Bcer	FALSE	NA	NA	NA
Bc_ctg1_3332	B1	6.648763744	Bcer	L	Bcer	FALSE	NA	NA	NA
Bc_ctg1_3333	B1	20.24399707	Bcer	S	Bcer	FALSE	NA	NA	NA
Bc_ctg1_3334	B1	19.84705595	Bcer	K	Bcer	FALSE	NA	NA	NA
Bc_ctg1_3335	B1	1.290058637	Bcer	S	Bcer	FALSE	NA	NA	NA
Bc_ctg1_3336	B1	1.389293917	Bcer	EG	Bcer	FALSE	NA	NA	NA
Bc_ctg1_3337	B1	16.27458588	Bcer	KT	Bcer	FALSE	NA	NA	NA
Bc_ctg1_3338	B1	0	Bcer	S	Bcer	FALSE	NA	NA	NA
Bc_ctg1_3339	B1	0.694646958	Bcer	S	Bcer	FALSE	NA	NA	NA
Bc_ctg1_334	B1	15.48070364	Bcer	S	Bcer	FALSE	NA	NA	NA
Bc_ctg1_3340	B1	0.09923528	Bcer	NA	Bcer	FALSE	NA	NA	NA
Bc_ctg1_3341	B1	12.90058637	Bcer	K	Bcer	FALSE	NA	NA	NA
Bc_ctg1_3342	B1	36.41934767	Bcer	EGP	Bcer	FALSE	NA	NA	NA
Bc_ctg1_3343	B1	8.732704619	Bcer	NA	Bcer	FALSE	NA	NA	NA
Bc_ctg1_3345	B1	17.06846812	Bcer	NA	Bcer	FALSE	NA	NA	NA
Bc_ctg1_3346	B1	6.152587345	Bcer	M	Bcer	FALSE	NA	NA	NA
Bc_ctg1_3347	B1	1.984705595	Bcer	NA	Bcer	FALSE	NA	NA	NA
Bc_ctg1_3348	B1	5.457940387	Bcer	G	Bcer	FALSE	NA	NA	NA
Bc_ctg1_335	B1	0.09923528	Bcer	NA	Bcer	FALSE	NA	NA	NA
Bc_ctg1_3350	B1	1.190823357	Bcer	NA	Bcer	FALSE	NA	NA	NA
Bc_ctg1_3351	B1	7.144940143	Bcer	S	Bcer	FALSE	NA	NA	NA
Bc_ctg1_3353	B1	22.03023211	Bcer	S	Bcer	FALSE	NA	NA	NA
Bc_ctg1_3354	B1	2.679352554	Bcer	T	Bcer	FALSE	NA	NA	NA
Bc_ctg1_3356	B1	3.671705351	Bcer	K	Bcer	FALSE	NA	NA	NA
Bc_ctg1_3357	B1	3.076293673	Bcer	I	Bcer	FALSE	NA	NA	NA
Bc_ctg1_3358	B1	2.679352554	Bcer	S	Bcer	FALSE	NA	NA	NA
Bc_ctg1_3359	B1	0.09923528	Bcer	EGP	Bcer	FALSE	NA	NA	NA
Bc_ctg1_336	B1	0.19847056	Bcer	NA	Bcer	FALSE	NA	NA	NA
Bc_ctg1_3360	B1	24.41187882	Bcer	K	Bcer	FALSE	NA	NA	NA
Bc_ctg1_3362	B1	0.396941119	Bcer	V	Bcer	FALSE	NA	NA	NA
Bc_ctg1_3363	B1	10.3204691	Bcer	G	Bcer	FALSE	NA	NA	NA
Bc_ctg1_3364	B1	5.160234548	Bcer	K	Bcer	FALSE	NA	NA	NA
Bc_ctg1_3365	B1	148.4559785	Bcer	G	Bcer	FALSE	NA	NA	NA
Bc_ctg1_3366	B1	12.40440997	Bcer	I	Bcer	FALSE	NA	NA	NA
Bc_ctg1_3367	B1	17.36617396	Bcer	K	Bcer	FALSE	NA	NA	NA
Bc_ctg1_3368	B1	0.297705839	Bcer	NA	Bcer	FALSE	NA	NA	NA
Bc_ctg1_337	B1	9.824292696	Bcer	V	Bcer	FALSE	NA	NA	NA
Bc_ctg1_3371	B1	5.060999268	Bcer	NA	Bcer	FALSE	NA	NA	NA
Bc_ctg1_3372	B1	0.09923528	Bcer	NA	Bcer	FALSE	NA	NA	NA
Bc_ctg1_3373	B1	18.75546787	Bcer	K	Bcer	FALSE	NA	NA	NA
Bc_ctg1_3374	B1	0.09923528	Bcer	NA	Bcer	FALSE	NA	NA	NA
Bc_ctg1_3375	B1	0.992352798	Bcer	NA	Bcer	FALSE	NA	NA	NA
Bc_ctg1_3376	B1	12.80135109	Bcer	S	Bcer	FALSE	NA	NA	NA
Bc_ctg1_3377	B1	0.09923528	Bcer	NA	Bcer	FALSE	NA	NA	NA
Bc_ctg1_3378	B1	18.85470315	Bcer	D	Bcer	FALSE	NA	NA	NA
Bc_ctg1_3379	B1	0.595411679	Bcer	NA	Bcer	FALSE	NA	NA	NA
Bc_ctg1_338	B1	1.786235036	Bcer	NA	Bcer	FALSE	NA	NA	NA
Bc_ctg1_3380	B1	0.893117518	Bcer	K	Bcer	FALSE	NA	NA	NA
Bc_ctg1_3381	B1	1.587764476	Bcer	S	Bcer	FALSE	NA	NA	NA
Bc_ctg1_3382	B1	0.694646958	Bcer	S	Bcer	FALSE	NA	NA	NA
Bc_ctg1_3383	B1	0.297705839	Bcer	NA	Bcer	FALSE	NA	NA	NA
Bc_ctg1_3384	B1	0.297705839	Bcer	NA	Bcer	FALSE	NA	NA	NA
Bc_ctg1_3385	B1	0.595411679	Bcer	S	Bcer	FALSE	NA	NA	NA
Bc_ctg1_3386	B1	0.19847056	Bcer	K	Bcer	FALSE	NA	NA	NA
Bc_ctg1_3387	B1	0.793882238	Bcer	S	Bcer	FALSE	NA	NA	NA
Bc_ctg1_3388	B1	1.786235036	Bcer	NA	Bcer	FALSE	NA	NA	NA

Bc_ctg1_3389	B1	18.06082092	Bcer	T	Bcer	FALSE	NA	NA	NA
Bc_ctg1_339	B1	10.12199854	Bcer	P	Bcer	FALSE	NA	NA	NA
Bc_ctg1_3390	B1	36.02240655	Bcer	T	Bcer	FALSE	NA	NA	NA
Bc_ctg1_3391	B1	48.92299292	Bcer	K	Bcer	FALSE	NA	NA	NA
Bc_ctg1_3392	B1	77.70122405	Bcer	S	Bcer	FALSE	NA	NA	NA
Bc_ctg1_3393	B1	43.56428782	Bcer	T	Bcer	FALSE	NA	NA	NA
Bc_ctg1_3394	B1	41.48034694	Bcer	NT	Bcer	FALSE	NA	NA	NA
Bc_ctg1_3395	B1	88.02169315	Bcer	T	Bcer	FALSE	NA	NA	NA
Bc_ctg1_3396	B1	0.793882238	Bcer	NA	Bcer	FALSE	NA	NA	NA
Bc_ctg1_3398	B1	1.786235036	Bcer	S	Bcer	FALSE	NA	NA	NA
Bc_ctg1_3399	B1	3.373999512	Bcer	NA	Bcer	FALSE	NA	NA	NA
Bc_ctg1_34	B1	0.595411679	Bcer	S	Bcer	FALSE	NA	NA	NA
Bc_ctg1_340	B1	4.16788175	Bcer	E	Bcer	FALSE	NA	NA	NA
Bc_ctg1_3400	B1	431.1772906	Bcer	S	Bcer	FALSE	NA	NA	NA
Bc_ctg1_3402	B1	3.96941119	Bcer	NA	Bcer	FALSE	NA	NA	NA
Bc_ctg1_3403	B1	1.885470315	Bcer	P	Bcer	FALSE	NA	NA	NA
Bc_ctg1_3404	B1	15.77840948	Bcer	K	Bcer	FALSE	NA	NA	NA
Bc_ctg1_3405	B1	8.931175179	Bcer	C	Bcer	FALSE	NA	NA	NA
Bc_ctg1_3406	B1	26.59505498	Bcer	M	Bcer	FALSE	NA	NA	NA
Bc_ctg1_3407	B1	0.793882238	Bcer	S	Bcer	FALSE	NA	NA	NA
Bc_ctg1_3408	B1	29.57211337	Bcer	NA	Bcer	FALSE	NA	NA	NA
Bc_ctg1_3409	B1	18.45776204	Bcer	S	Bcer	FALSE	NA	NA	NA
Bc_ctg1_341	B1	2.977058393	Bcer	K	Bcer	FALSE	NA	NA	NA
Bc_ctg1_3410	B1	12.20593941	Bcer	L	Bcer	FALSE	NA	NA	NA
Bc_ctg1_3411	B1	20.34323235	Bcer	L	Bcer	FALSE	NA	NA	NA
Bc_ctg1_3412	B1	310.5071904	Bcer	L	Bcer	FALSE	NA	NA	NA
Bc_ctg1_3413	B1	46.64058149	Bcer	S	Bcer	FALSE	NA	NA	NA
Bc_ctg1_3414	B1	61.72434401	Bcer	E	Bcer	FALSE	NA	NA	NA
Bc_ctg1_3415	B1	76.21269486	Bcer	E	Bcer	FALSE	NA	NA	NA
Bc_ctg1_3416	B1	89.41098706	Bcer	E	Bcer	FALSE	NA	NA	NA
Bc_ctg1_3417	B1	3.373999512	Bcer	K	Bcer	FALSE	NA	NA	NA
Bc_ctg1_3418	B1	0.19847056	Bcer	NA	Bcer	FALSE	NA	NA	NA
Bc_ctg1_3419	B1	2.480881994	Bcer	S	Bcer	FALSE	NA	NA	NA
Bc_ctg1_342	B1	1.190823357	Bcer	S	Bcer	FALSE	NA	NA	NA
Bc_ctg1_3420	B1	0.595411679	Bcer	S	Bcer	FALSE	NA	NA	NA
Bc_ctg1_3421	B1	2.183176155	Bcer	S	Bcer	FALSE	NA	NA	NA
Bc_ctg1_3423	B1	1.290058637	Bcer	G	Bcer	FALSE	NA	NA	NA
Bc_ctg1_3424	B1	0.694646958	Bcer	K	Bcer	FALSE	NA	NA	NA
Bc_ctg1_3425	B1	90.60181042	Bcer	L	Bcer	FALSE	NA	NA	NA
Bc_ctg1_3426	B1	77.60198877	Bcer	K	Bcer	FALSE	NA	NA	NA
Bc_ctg1_3427	B1	26.59505498	Bcer	G	Bcer	FALSE	NA	NA	NA
Bc_ctg1_3428	B1	157.3871537	Bcer	C	Bcer	FALSE	NA	NA	NA
Bc_ctg1_3429	B1	194.6003836	Bcer	C	Bcer	FALSE	NA	NA	NA
Bc_ctg1_343	B1	1.488529196	Bcer	S	Bcer	FALSE	NA	NA	NA
Bc_ctg1_3430	B1	16.671527	Bcer	K	Bcer	FALSE	NA	NA	NA
Bc_ctg1_3431	B1	26.79352554	Bcer	V	Bcer	FALSE	NA	NA	NA
Bc_ctg1_3432	B1	2.679352554	Bcer	NA	Bcer	FALSE	NA	NA	NA
Bc_ctg1_3433	B1	19.74782067	Bcer	L	Bcer	FALSE	NA	NA	NA
Bc_ctg1_3434	B1	0.595411679	Bcer	K	Bcer	FALSE	NA	NA	NA
Bc_ctg1_3435	B1	0.496176399	Bcer	K	Bcer	FALSE	NA	NA	NA
Bc_ctg1_3436	B1	10.12199854	Bcer	D	Bcer	FALSE	NA	NA	NA
Bc_ctg1_3437	B1	16.671527	Bcer	L	Bcer	FALSE	NA	NA	NA
Bc_ctg1_3438	B1	4.862528708	Bcer	NA	Bcer	FALSE	NA	NA	NA
Bc_ctg1_3439	B1	969.7271538	Bcer	M	Bcer	FALSE	NA	NA	NA
Bc_ctg1_344	B1	1.091588077	Bcer	S	Bcer	FALSE	NA	NA	NA
Bc_ctg1_3440	B1	0.893117518	Bcer	NA	Bcer	FALSE	NA	NA	NA
Bc_ctg1_3441	B1	13.59523333	Bcer	S	Bcer	FALSE	NA	NA	NA
Bc_ctg1_3442	B1	178.6235036	Bcer	K	Bcer	FALSE	NA	NA	NA
Bc_ctg1_3443	B1	175.1502688	Bcer	S	Bcer	FALSE	NA	NA	NA
Bc_ctg1_3444	B1	74.72416566	Bcer	FG	Bcer	FALSE	NA	NA	NA
Bc_ctg1_3445	B1	48.72452236	Bcer	V	Bcer	FALSE	NA	NA	NA
Bc_ctg1_3446	B1	51.70158076	Bcer	U	Bcer	FALSE	NA	NA	NA

Bc_ctg1_3447	B1	39.19793551	Bcer	P	Bcer	FALSE	NA	NA	NA
Bc_ctg1_3448	B1	4.961763988	Bcer	NA	Bcer	FALSE	NA	NA	NA
Bc_ctg1_3449	B1	5.656410946	Bcer	NA	Bcer	FALSE	NA	NA	NA
Bc_ctg1_345	B1	17.46540924	Bcer	S	Bcer	FALSE	Bc_Ga0417192_01_rn04	Other	NA
Bc_ctg1_3450	B1	13.49599805	Bcer	NA	Bcer	FALSE	NA	NA	NA
Bc_ctg1_3451	B1	8.931175179	Bcer	IU	Bcer	FALSE	NA	NA	NA
Bc_ctg1_3452	B1	2.282411435	Bcer	NA	Bcer	FALSE	NA	NA	NA
Bc_ctg1_3453	B1	7.641116542	Bcer	K	Bcer	FALSE	NA	NA	NA
Bc_ctg1_3454	B1	0	Bcer	NA	Bcer	FALSE	NA	NA	NA
Bc_ctg1_3455	B1	12.10670413	Bcer	S	Bcer	FALSE	NA	NA	NA
Bc_ctg1_3456	B1	73.43410702	Bcer	S	Bcer	FALSE	NA	NA	NA
Bc_ctg1_3457	B1	46.24364037	Bcer	S	Bcer	FALSE	NA	NA	NA
Bc_ctg1_3458	B1	3.770940631	Bcer	NA	Bcer	FALSE	NA	NA	NA
Bc_ctg1_3459	B1	0.19847056	Bcer	S	Bcer	FALSE	NA	NA	NA
Bc_ctg1_346	B1	17.76311508	Bcer	C	Bcer	FALSE	Bc_Ga0417192_01_rn04	Ancillary biosynthesis	SMCOG1028
Bc_ctg1_3460	B1	6.251822625	Bcer	NA	Bcer	FALSE	NA	NA	NA
Bc_ctg1_3461	B1	0.793882238	Bcer	K	Bcer	FALSE	NA	NA	NA
Bc_ctg1_3462	B1	12.00746885	Bcer	M	Bcer	FALSE	NA	NA	NA
Bc_ctg1_3463	B1	104.5939849	Bcer	E	Bcer	FALSE	NA	NA	NA
Bc_ctg1_3464	B1	87.12857563	Bcer	IQ	Bcer	TRUE	NA	NA	NA
Bc_ctg1_3465	B1	40.1902883	Bcer	EG	Bcer	FALSE	NA	NA	NA
Bc_ctg1_3466	B1	3.076293673	Bcer	S	Bcer	FALSE	NA	NA	NA
Bc_ctg1_3467	B1	3.572470071	Bcer	S	Bcer	FALSE	NA	NA	NA
Bc_ctg1_3468	B1	114.0213364	Bcer	S	Bcer	FALSE	NA	NA	NA
Bc_ctg1_3469	B1	4.16788175	Bcer	M	Bcer	FALSE	NA	NA	NA
Bc_ctg1_347	B1	45.35052285	Bcer	E	Bcer	FALSE	Bc_Ga0417192_01_rn04	Ancillary biosynthesis	SMCOG1038
Bc_ctg1_3470	B1	93.77733937	Bcer	H	Bcer	FALSE	NA	NA	NA
Bc_ctg1_3471	B1	83.05992916	Bcer	H	Bcer	FALSE	NA	NA	NA
Bc_ctg1_3472	B1	222.7832031	Bcer	H	Bcer	FALSE	NA	NA	NA
Bc_ctg1_3473	B1	97.34980945	Bcer	S	Bcer	FALSE	NA	NA	NA
Bc_ctg1_3474	B1	11.01511605	Bcer	S	Bcer	FALSE	NA	NA	NA
Bc_ctg1_3475	B1	0.893117518	Bcer	NA	Bcer	FALSE	NA	NA	NA
Bc_ctg1_3476	B1	86.43392867	Bcer	NA	Bcer	FALSE	NA	NA	NA
Bc_ctg1_3477	B1	1.290058637	Bcer	K	Bcer	FALSE	NA	NA	NA
Bc_ctg1_3478	B1	8.633469339	Bcer	KT	Bcer	FALSE	NA	NA	NA
Bc_ctg1_3479	B1	127.0211581	Bcer	K	Bcer	FALSE	NA	NA	NA
Bc_ctg1_348	B1	48.62528708	Bcer	EGP	Bcer	FALSE	Bc_Ga0417192_01_rn04	Transport	SMCOG1005
Bc_ctg1_3480	B1	11.11435133	Bcer	S	Bcer	FALSE	NA	NA	NA
Bc_ctg1_3481	B1	3.572470071	Bcer	NA	Bcer	FALSE	NA	NA	NA
Bc_ctg1_3482	B1	12.60288053	Bcer	K	Bcer	FALSE	NA	NA	NA
Bc_ctg1_3483	B1	6.747999024	Bcer	S	Bcer	FALSE	NA	NA	NA
Bc_ctg1_3484	B1	2.977058393	Bcer	M	Bcer	FALSE	NA	NA	NA
Bc_ctg1_3485	B1	638.0828489	Bcer	K	Bcer	FALSE	NA	NA	NA
Bc_ctg1_3486	B1	0	Bcer	S	Bcer	FALSE	NA	NA	NA
Bc_ctg1_3487	B1	147.9598021	Bcer	S	Bcer	FALSE	NA	NA	NA
Bc_ctg1_3488	B1	348.1173614	Bcer	H	Bcer	FALSE	NA	NA	NA
Bc_ctg1_3489	B1	1.091588077	Bcer	NA	Bcer	FALSE	NA	NA	NA
Bc_ctg1_349	B1	0.09923528	Bcer	NA	Bcer	FALSE	Bc_Ga0417192_01_rn04	Other	NA
Bc_ctg1_3490	B1	21.43482043	Bcer	IQ	Bcer	TRUE	NA	NA	NA
Bc_ctg1_3491	B1	0	Bcer	M	Bcer	FALSE	NA	NA	NA
Bc_ctg1_3492	B1	0.595411679	Bcer	P	Bcer	FALSE	NA	NA	NA
Bc_ctg1_3493	B1	2.183176155	Bcer	O	Bcer	FALSE	NA	NA	NA
Bc_ctg1_3494	B1	1.885470315	Bcer	P	Bcer	FALSE	NA	NA	NA
Bc_ctg1_3495	B1	4.862528708	Bcer	P	Bcer	FALSE	NA	NA	NA
Bc_ctg1_3496	B1	524.0615124	Bcer	S	Bcer	FALSE	NA	NA	NA
Bc_ctg1_3497	B1	46.54134621	Bcer	S	Bcer	FALSE	NA	NA	NA
Bc_ctg1_3498	B1	1.786235036	Bcer	S	Bcer	FALSE	NA	NA	NA
Bc_ctg1_3499	B1	10.02276326	Bcer	S	Bcer	FALSE	NA	NA	NA
Bc_ctg1_35	B1	4.26711703	Bcer	S	Bcer	FALSE	NA	NA	NA
Bc_ctg1_350	B1	0.09923528	Bcer	S	Bcer	FALSE	Bc_Ga0417192_01_rn04	Other	NA
Bc_ctg1_3500	B1	5.854881506	Bcer	K	Bcer	FALSE	NA	NA	NA
Bc_ctg1_3501	B1	9.228881018	Bcer	U	Bcer	FALSE	NA	NA	NA

Bc_ctg1_3502	B1	5.755646226	Bcer	S	Bcer	FALSE	NA	NA	NA
Bc_ctg1_3503	B1	1.091588077	Bcer	K	Bcer	FALSE	NA	NA	NA
Bc_ctg1_3504	B1	57.8541681	Bcer	K	Bcer	FALSE	NA	NA	NA
Bc_ctg1_3505	B1	1.389293917	Bcer	E	Bcer	FALSE	NA	NA	NA
Bc_ctg1_3506	B1	15.48070364	Bcer	T	Bcer	FALSE	NA	NA	NA
Bc_ctg1_3507	B1	7.442645982	Bcer	T	Bcer	FALSE	NA	NA	NA
Bc_ctg1_3508	B1	37.80864159	Bcer	S	Bcer	FALSE	NA	NA	NA
Bc_ctg1_3509	B1	6.549528464	Bcer	NA	Bcer	FALSE	NA	NA	NA
Bc_ctg1_351	B1	0.09923528	Bcer	NA	Bcer	FALSE	Bc_Ga0417192_01_rn04	Other	NA
Bc_ctg1_3510	B1	2.679352554	Bcer	NA	Bcer	FALSE	NA	NA	NA
Bc_ctg1_3511	B1	13.19829221	Bcer	O	Bcer	FALSE	NA	NA	NA
Bc_ctg1_3512	B1	36.61781823	Bcer	NT	Bcer	FALSE	NA	NA	NA
Bc_ctg1_3513	B1	37.11399463	Bcer	D	Bcer	FALSE	NA	NA	NA
Bc_ctg1_3515	B1	3.175528952	Bcer	D	Bcer	FALSE	NA	NA	NA
Bc_ctg1_3516	B1	4.961763988	Bcer	C	Bcer	FALSE	NA	NA	NA
Bc_ctg1_3517	B1	9.824292696	Bcer	C	Bcer	FALSE	NA	NA	NA
Bc_ctg1_3518	B1	6.946469583	Bcer	S	Bcer	FALSE	NA	NA	NA
Bc_ctg1_3519	B1	1.885470315	Bcer	NA	Bcer	FALSE	NA	NA	NA
Bc_ctg1_352	B1	0	Bcer	NA	Bcer	FALSE	Bc_Ga0417192_01_rn04	Other	NA
Bc_ctg1_3520	B1	83.8538114	Bcer	K	Bcer	FALSE	NA	NA	NA
Bc_ctg1_3521	B1	0	Bcer	NA	Bcer	FALSE	NA	NA	NA
Bc_ctg1_3522	B1	5.755646226	Bcer	NA	Bcer	FALSE	NA	NA	NA
Bc_ctg1_3523	B1	23.0225849	Bcer	K	Bcer	FALSE	NA	NA	NA
Bc_ctg1_3524	B1	17.46540924	Bcer	S	Bcer	FALSE	NA	NA	NA
Bc_ctg1_3525	B1	21.43482043	Bcer	U	Bcer	FALSE	NA	NA	NA
Bc_ctg1_3526	B1	103.204691	Bcer	L	Bcer	FALSE	NA	NA	NA
Bc_ctg1_3527	B1	144.1888615	Bcer	L	Bcer	FALSE	NA	NA	NA
Bc_ctg1_3528	B1	2.083940875	Bcer	K	Bcer	FALSE	NA	NA	NA
Bc_ctg1_3529	B1	0.396941119	Bcer	S	Bcer	FALSE	NA	NA	NA
Bc_ctg1_353	B1	4.06864647	Bcer	NA	Bcer	FALSE	Bc_Ga0417192_01_rn04	Other	NA
Bc_ctg1_3530	B1	0.496176399	Bcer	S	Bcer	FALSE	NA	NA	NA
Bc_ctg1_3531	B1	0	Bcer	S	Bcer	FALSE	NA	NA	NA
Bc_ctg1_3532	B1	1.686999756	Bcer	S	Bcer	FALSE	NA	NA	NA
Bc_ctg1_3533	B1	0.19847056	Bcer	S	Bcer	FALSE	NA	NA	NA
Bc_ctg1_3534	B1	0.595411679	Bcer	S	Bcer	FALSE	NA	NA	NA
Bc_ctg1_3535	B1	0.595411679	Bcer	NA	Bcer	FALSE	NA	NA	NA
Bc_ctg1_3536	B1	23.61799658	Bcer	Q	Bcer	TRUE	NA	NA	NA
Bc_ctg1_3537	B1	6.053352065	Bcer	NA	Bcer	FALSE	NA	NA	NA
Bc_ctg1_3538	B1	1698.213343	Bcer	E	Bcer	FALSE	NA	NA	NA
Bc_ctg1_3539	B1	58.64805034	Bcer	S	Bcer	FALSE	NA	NA	NA
Bc_ctg1_354	B1	20.54170291	Bcer	IQ	Bcer	TRUE	Bc_Ga0417192_01_rn04	Ancillary biosynthesis	SMCOG1001
Bc_ctg1_3540	B1	0.09923528	Bcer	S	Bcer	FALSE	NA	NA	NA
Bc_ctg1_3541	B1	5.557175667	Bcer	E	Bcer	FALSE	NA	NA	NA
Bc_ctg1_3542	B1	253.8438456	Bcer	H	Bcer	FALSE	NA	NA	NA
Bc_ctg1_3543	B1	2988.569685	Bcer	P	Bcer	FALSE	NA	NA	NA
Bc_ctg1_3544	B1	3.076293673	Bcer	P	Bcer	FALSE	NA	NA	NA
Bc_ctg1_3545	B1	13.59523333	Bcer	G	Bcer	FALSE	NA	NA	NA
Bc_ctg1_3546	B1	38.80099439	Bcer	K	Bcer	FALSE	NA	NA	NA
Bc_ctg1_3547	B1	215.9359688	Bcer	S	Bcer	FALSE	NA	NA	NA
Bc_ctg1_3548	B1	64.10599073	Bcer	S	Bcer	FALSE	NA	NA	NA
Bc_ctg1_3549	B1	75.22034206	Bcer	M	Bcer	FALSE	Bc_Ga0417192_01_rn24	Other	NA
Bc_ctg1_355	B1	109.5557489	Bcer	S	Bcer	FALSE	Bc_Ga0417192_01_rn04	Core biosynthesis	None
Bc_ctg1_3551	B1	4.06864647	Bcer	S	Bcer	FALSE	Bc_Ga0417192_01_rn24	Other	NA
Bc_ctg1_3552	B1	6.053352065	Bcer	S	Bcer	FALSE	Bc_Ga0417192_01_rn24	Other	NA
Bc_ctg1_3554	B1	84.8461642	Bcer	S	Bcer	FALSE	Bc_Ga0417192_01_rn24	Ancillary biosynthesis	SMCOG1179
Bc_ctg1_3555	B1	3.175528952	Bcer	S	Bcer	FALSE	Bc_Ga0417192_01_rn24	Other	NA
Bc_ctg1_3556	B1	281.2327828	Bcer	O	Bcer	FALSE	Bc_Ga0417192_01_rn24	Other	SMCOG1223
Bc_ctg1_3557	B1	176.7380333	Bcer	S	Bcer	FALSE	Bc_Ga0417192_01_rn24	Other	NA
Bc_ctg1_3558	B1	0.496176399	Bcer	I	Bcer	FALSE	Bc_Ga0417192_01_rn24	Other	NA
Bc_ctg1_3559	B1	0.496176399	Bcer	GM	Bcer	FALSE	Bc_Ga0417192_01_rn24	Other	NA
Bc_ctg1_356	B1	45.54899341	Bcer	S	Bcer	FALSE	Bc_Ga0417192_01_rn04	Other	NA
Bc_ctg1_3560	B1	0.19847056	Bcer	S	Bcer	FALSE	Bc_Ga0417192_01_rn24	Other	NA

Bc_ctg1_3561	B1	77.70122405	Bcer	I	Bcer	FALSE	Bc_Ga0417192_01_rn24	Core biosynthesis	SMCOG1084
Bc_ctg1_3562	B1	315.1712485	Bcer	I	Bcer	FALSE	Bc_Ga0417192_01_rn24	Core biosynthesis	SMCOG1022
Bc_ctg1_3563	B1	1.686999756	Bcer	O	Bcer	FALSE	Bc_Ga0417192_01_rn24	Other	NA
Bc_ctg1_3564	B1	3.373999512	Bcer	S	Bcer	FALSE	Bc_Ga0417192_01_rn24	Other	NA
Bc_ctg1_3565	B1	135.6546274	Bcer	J	Bcer	FALSE	Bc_Ga0417192_01_rn24	Other	NA
Bc_ctg1_3566	B1	12.40440997	Bcer	S	Bcer	FALSE	Bc_Ga0417192_01_rn24	Other	NA
Bc_ctg1_3567	B1	500.840457	Bcer	E	Bcer	FALSE	Bc_Ga0417192_01_rn24	Transport	SMCOG1068
Bc_ctg1_3568	B1	64.20522601	Bcer	E	Bcer	FALSE	Bc_Ga0417192_01_rn24	Other	NA
Bc_ctg1_3569	B1	369.0560054	Bcer	P	Bcer	FALSE	Bc_Ga0417192_01_rn24	Transport	SMCOG1118
Bc_ctg1_357	B1	51.80081604	Bcer	P	Bcer	FALSE	Bc_Ga0417192_01_rn04	Other	NA
Bc_ctg1_3570	B1	395.9487662	Bcer	EP	Bcer	FALSE	Bc_Ga0417192_01_rn24	Transport	SMCOG1085
Bc_ctg1_3571	B1	683.4333717	Bcer	P	Bcer	FALSE	Bc_Ga0417192_01_rn24	Transport	SMCOG1000
Bc_ctg1_3572	B1	675.4945493	Bcer	E	Bcer	FALSE	Bc_Ga0417192_01_rn24	Transport	SMCOG1000
Bc_ctg1_3573	B1	0.09923528	Bcer	L	Bcer	FALSE	NA	NA	NA
Bc_ctg1_3574	B1	0	Bcer	L	Bcer	FALSE	NA	NA	NA
Bc_ctg1_3575	B1	11.01511605	Bcer	V	Bcer	FALSE	NA	NA	NA
Bc_ctg1_3576	B1	703.3796629	Bcer	E	Bcer	FALSE	NA	NA	NA
Bc_ctg1_3577	B1	6.152587345	Bcer	NA	Bcer	FALSE	NA	NA	NA
Bc_ctg1_3578	B1	2329.349722	Bcer	K	Bcer	FALSE	NA	NA	NA
Bc_ctg1_3579	B1	0.297705839	Bcer	P	Bcer	FALSE	NA	NA	NA
Bc_ctg1_358	B1	2.083940875	Bcer	NA	Bcer	FALSE	Bc_Ga0417192_01_rn04	Other	NA
Bc_ctg1_3580	B1	174.8525629	Bcer	NOT	Bcer	FALSE	NA	NA	NA
Bc_ctg1_3581	B1	61.62510873	Bcer	I	Bcer	FALSE	NA	NA	NA
Bc_ctg1_3582	B1	4.366352309	Bcer	S	Bcer	FALSE	NA	NA	NA
Bc_ctg1_3583	B1	752.699597	Bcer	E	Bcer	FALSE	NA	NA	NA
Bc_ctg1_3584	B1	31.75528952	Bcer	Q	Bcer	TRUE	Bc_Ga0417192_01_rn25	Other	NA
Bc_ctg1_3585	B1	9.526586857	Bcer	S	Bcer	FALSE	Bc_Ga0417192_01_rn25	Other	NA
Bc_ctg1_3586	B1	20.14476179	Bcer	S	Bcer	FALSE	Bc_Ga0417192_01_rn25	Other	NA
Bc_ctg1_3587	B1	40.6864647	Bcer	S	Bcer	FALSE	Bc_Ga0417192_01_rn25	Other	NA
Bc_ctg1_3588	B1	262.6757855	Bcer	S	Bcer	FALSE	Bc_Ga0417192_01_rn25	Other	NA
Bc_ctg1_3589	B1	225.958732	Bcer	G	Bcer	FALSE	Bc_Ga0417192_01_rn25	Other	NA
Bc_ctg1_359	B1	2.083940875	Bcer	J	Bcer	FALSE	Bc_Ga0417192_01_rn04	Ancillary biosynthesis	SMCOG1111
Bc_ctg1_3590	B1	177.8296213	Bcer	J	Bcer	FALSE	Bc_Ga0417192_01_rn25	Ancillary biosynthesis	SMCOG1279
Bc_ctg1_3591	B1	0.396941119	Bcer	UW	Bcer	FALSE	Bc_Ga0417192_01_rn25	Other	NA
Bc_ctg1_3592	B1	2.977058393	Bcer	T	Bcer	FALSE	Bc_Ga0417192_01_rn25	Other	NA
Bc_ctg1_3593	B1	0.595411679	Bcer	D	Bcer	FALSE	Bc_Ga0417192_01_rn25	Other	NA
Bc_ctg1_3594	B1	0.396941119	Bcer	M	Bcer	FALSE	Bc_Ga0417192_01_rn25	Core biosynthesis	None
Bc_ctg1_3595	B1	0	Bcer	H	Bcer	FALSE	Bc_Ga0417192_01_rn25	Other	NA
Bc_ctg1_3596	B1	0	Bcer	S	Bcer	FALSE	Bc_Ga0417192_01_rn25	Other	NA
Bc_ctg1_3597	B1	0.496176399	Bcer	K	Bcer	FALSE	Bc_Ga0417192_01_rn25	Other	NA
Bc_ctg1_3598	B1	0.992352798	Bcer	M	Bcer	FALSE	Bc_Ga0417192_01_rn25	Core biosynthesis	None
Bc_ctg1_3599	B1	0.19847056	Bcer	H	Bcer	FALSE	Bc_Ga0417192_01_rn25	Other	NA
Bc_ctg1_36	B1	1.885470315	Bcer	K	Bcer	FALSE	NA	NA	NA
Bc_ctg1_360	B1	30.66370145	Bcer	P	Bcer	FALSE	Bc_Ga0417192_01_rn04	Transport	SMCOG1033
Bc_ctg1_3600	B1	1.091588077	Bcer	S	Bcer	FALSE	Bc_Ga0417192_01_rn25	Other	NA
Bc_ctg1_3601	B1	1.686999756	Bcer	S	Bcer	FALSE	Bc_Ga0417192_01_rn25	Other	NA
Bc_ctg1_3602	B1	0.09923528	Bcer	M	Bcer	FALSE	Bc_Ga0417192_01_rn25	Ancillary biosynthesis	SMCOG1064
Bc_ctg1_3603	B1	3.175528952	Bcer	M	Bcer	FALSE	Bc_Ga0417192_01_rn25	Core biosynthesis	None
Bc_ctg1_3604	B1	4.664058149	Bcer	M	Bcer	FALSE	Bc_Ga0417192_01_rn25	Core biosynthesis	SMCOG1010
Bc_ctg1_3605	B1	3.175528952	Bcer	M	Bcer	FALSE	Bc_Ga0417192_01_rn25	Core biosynthesis	SMCOG1010
Bc_ctg1_3606	B1	290.0647227	Bcer	I	Bcer	FALSE	Bc_Ga0417192_01_rn25	Ancillary biosynthesis	SMCOG1001
Bc_ctg1_3607	B1	0	Bcer	S	Bcer	FALSE	Bc_Ga0417192_01_rn25	Other	NA
Bc_ctg1_3608	B1	0.09923528	Bcer	S	Bcer	FALSE	Bc_Ga0417192_01_rn25	Other	NA
Bc_ctg1_3609	B1	0.09923528	Bcer	S	Bcer	FALSE	Bc_Ga0417192_01_rn25	Other	NA
Bc_ctg1_361	B1	9.328116298	Bcer	P	Bcer	FALSE	Bc_Ga0417192_01_rn04	Transport	SMCOG1011
Bc_ctg1_3610	B1	0	Bcer	NA	Bcer	FALSE	Bc_Ga0417192_01_rn25	Other	NA
Bc_ctg1_3611	B1	5.755646226	Bcer	S	Bcer	FALSE	Bc_Ga0417192_01_rn25	Other	NA
Bc_ctg1_3612	B1	132.9752749	Bcer	L	Bcer	FALSE	Bc_Ga0417192_01_rn25	Other	NA
Bc_ctg1_3613	B1	305.6446617	Bcer	S	Bcer	FALSE	Bc_Ga0417192_01_rn25	Other	NA
Bc_ctg1_3614	B1	327.2779527	Bcer	J	Bcer	FALSE	Bc_Ga0417192_01_rn25	Other	NA
Bc_ctg1_3615	B1	39.6941119	Bcer	P	Bcer	FALSE	Bc_Ga0417192_01_rn25	Ancillary biosynthesis	SMCOG1098
Bc_ctg1_3616	B1	106.6779257	Bcer	EG	Bcer	FALSE	Bc_Ga0417192_01_rn25	Transport	SMCOG1166

Bc_ctg1_3617	B1	0.297705839	Bcer	S	Bcer	FALSE	Bc_Ga0417192_01_rn25	Other	NA
Bc_ctg1_3618	B1	2.083940875	Bcer	N	Bcer	FALSE	NA	NA	NA
Bc_ctg1_3619	B1	48.92299292	Bcer	E	Bcer	FALSE	NA	NA	NA
Bc_ctg1_362	B1	8.931175179	Bcer	P	Bcer	FALSE	Bc_Ga0417192_01_rn04	Transport	SMCOG1011
Bc_ctg1_3620	B1	7.938822381	Bcer	EH	Bcer	FALSE	NA	NA	NA
Bc_ctg1_3621	B1	3.274764232	Bcer	EH	Bcer	FALSE	NA	NA	NA
Bc_ctg1_3622	B1	7.740351821	Bcer	E	Bcer	FALSE	NA	NA	NA
Bc_ctg1_3623	B1	10.81664549	Bcer	E	Bcer	FALSE	NA	NA	NA
Bc_ctg1_3624	B1	6.747999024	Bcer	E	Bcer	FALSE	NA	NA	NA
Bc_ctg1_3625	B1	17.26693868	Bcer	E	Bcer	FALSE	NA	NA	NA
Bc_ctg1_3626	B1	13.89293917	Bcer	E	Bcer	FALSE	Bc_Ga0417192_01_rn26	Other	NA
Bc_ctg1_3627	B1	3.175528952	Bcer	NA	Bcer	FALSE	Bc_Ga0417192_01_rn26	Other	NA
Bc_ctg1_3628	B1	5.755646226	Bcer	C	Bcer	FALSE	Bc_Ga0417192_01_rn26	Other	NA
Bc_ctg1_3629	B1	35.52623015	Bcer	S	Bcer	FALSE	Bc_Ga0417192_01_rn26	Other	NA
Bc_ctg1_363	B1	3.076293673	Bcer	S	Bcer	FALSE	Bc_Ga0417192_01_rn04	Other	NA
Bc_ctg1_3630	B1	16.27458588	Bcer	NA	Bcer	FALSE	Bc_Ga0417192_01_rn26	Other	NA
Bc_ctg1_3631	B1	0.496176399	Bcer	M	Bcer	FALSE	Bc_Ga0417192_01_rn26	Other	NA
Bc_ctg1_3632	B1	1.984705595	Bcer	S	Bcer	FALSE	Bc_Ga0417192_01_rn26	Other	NA
Bc_ctg1_3633	B1	0.496176399	Bcer	NA	Bcer	FALSE	Bc_Ga0417192_01_rn26	Other	NA
Bc_ctg1_3634	B1	12.40440997	Bcer	P	Bcer	FALSE	Bc_Ga0417192_01_rn26	Ancillary biosynthesis	SMCOG1175
Bc_ctg1_3635	B1	2.778587833	Bcer	S	Bcer	FALSE	Bc_Ga0417192_01_rn26	Other	NA
Bc_ctg1_3636	B1	7.541881262	Bcer	NA	Bcer	FALSE	Bc_Ga0417192_01_rn26	Other	NA
Bc_ctg1_3637	B1	1.290058637	Bcer	C	Bcer	FALSE	Bc_Ga0417192_01_rn26	Core biosynthesis	None
Bc_ctg1_3638	B1	0.793882238	Bcer	S	Bcer	FALSE	Bc_Ga0417192_01_rn26	Core biosynthesis	None
Bc_ctg1_3639	B1	0.992352798	Bcer	H	Bcer	FALSE	Bc_Ga0417192_01_rn26	Core biosynthesis	None
Bc_ctg1_364	B1	12.20593941	Bcer	S	Bcer	FALSE	Bc_Ga0417192_01_rn04	Other	NA
Bc_ctg1_3640	B1	786.5388274	Bcer	C	Bcer	FALSE	Bc_Ga0417192_01_rn26	Other	NA
Bc_ctg1_3641	B1	1535.665954	Bcer	C	Bcer	FALSE	Bc_Ga0417192_01_rn26	Other	NA
Bc_ctg1_3642	B1	0.396941119	Bcer	NA	Bcer	FALSE	Bc_Ga0417192_01_rn26	Other	NA
Bc_ctg1_3643	B1	0.992352798	Bcer	NA	Bcer	FALSE	Bc_Ga0417192_01_rn26	Other	NA
Bc_ctg1_3644	B1	0.992352798	Bcer	NA	Bcer	FALSE	Bc_Ga0417192_01_rn26	Other	NA
Bc_ctg1_3645	B1	4.862528708	Bcer	NA	Bcer	FALSE	Bc_Ga0417192_01_rn26	Other	NA
Bc_ctg1_3646	B1	159.272624	Bcer	NA	Bcer	FALSE	Bc_Ga0417192_01_rn26	Other	NA
Bc_ctg1_3647	B1	0.19847056	Bcer	NA	Bcer	FALSE	NA	NA	NA
Bc_ctg1_3648	B1	2.480881994	Bcer	NA	Bcer	FALSE	NA	NA	NA
Bc_ctg1_3649	B1	58.94575618	Bcer	M	Bcer	FALSE	NA	NA	NA
Bc_ctg1_365	B1	52.59469827	Bcer	S	Bcer	FALSE	Bc_Ga0417192_01_rn04	Other	NA
Bc_ctg1_3650	B1	0.694646958	Bcer	U	Bcer	FALSE	NA	NA	NA
Bc_ctg1_3651	B1	1.290058637	Bcer	S	Bcer	FALSE	NA	NA	NA
Bc_ctg1_3652	B1	0.496176399	Bcer	M	Bcer	FALSE	NA	NA	NA
Bc_ctg1_3653	B1	4.465587589	Bcer	S	Bcer	FALSE	NA	NA	NA
Bc_ctg1_3654	B1	73.0371659	Bcer	K	Bcer	FALSE	NA	NA	NA
Bc_ctg1_3655	B1	0	Bcer	NA	Bcer	FALSE	NA	NA	NA
Bc_ctg1_3656	B1	10.71741021	Bcer	V	Bcer	FALSE	NA	NA	NA
Bc_ctg1_3657	B1	50.60999268	Bcer	C	Bcer	FALSE	NA	NA	NA
Bc_ctg1_3658	B1	50.80846324	Bcer	P	Bcer	FALSE	NA	NA	NA
Bc_ctg1_3659	B1	36.12164183	Bcer	E	Bcer	FALSE	NA	NA	NA
Bc_ctg1_366	B1	9.923527976	Bcer	S	Bcer	FALSE	Bc_Ga0417192_01_rn04	Other	NA
Bc_ctg1_3660	B1	32.05299536	Bcer	E	Bcer	FALSE	NA	NA	NA
Bc_ctg1_3661	B1	73.0371659	Bcer	P	Bcer	FALSE	NA	NA	NA
Bc_ctg1_3662	B1	13.09905693	Bcer	I	Bcer	FALSE	NA	NA	NA
Bc_ctg1_3663	B1	166.020623	Bcer	S	Bcer	FALSE	NA	NA	NA
Bc_ctg1_3664	B1	0.496176399	Bcer	S	Bcer	FALSE	NA	NA	NA
Bc_ctg1_3665	B1	32.64840704	Bcer	J	Bcer	FALSE	NA	NA	NA
Bc_ctg1_3666	B1	81.76987052	Bcer	S	Bcer	FALSE	NA	NA	NA
Bc_ctg1_3667	B1	0.496176399	Bcer	J	Bcer	FALSE	NA	NA	NA
Bc_ctg1_3668	B1	39.79334718	Bcer	C	Bcer	FALSE	NA	NA	NA
Bc_ctg1_3669	B1	50.11381628	Bcer	C	Bcer	FALSE	NA	NA	NA
Bc_ctg1_367	B1	0.09923528	Bcer	S	Bcer	FALSE	Bc_Ga0417192_01_rn04	Other	NA
Bc_ctg1_3670	B1	3.770940631	Bcer	KT	Bcer	FALSE	NA	NA	NA
Bc_ctg1_3671	B1	17.46540924	Bcer	S	Bcer	FALSE	NA	NA	NA
Bc_ctg1_3672	B1	10.71741021	Bcer	T	Bcer	FALSE	NA	NA	NA

Bc_ctg1_3673	B1	52.19775715	Bcer	T	Bcer	FALSE	NA	NA	NA
Bc_ctg1_3674	B1	526.9393355	Bcer	K	Bcer	FALSE	NA	NA	NA
Bc_ctg1_3675	B1	175.3487393	Bcer	C	Bcer	FALSE	NA	NA	NA
Bc_ctg1_3676	B1	573.2822112	Bcer	C	Bcer	FALSE	NA	NA	NA
Bc_ctg1_3677	B1	262.3780797	Bcer	S	Bcer	FALSE	NA	NA	NA
Bc_ctg1_3678	B1	35.12928904	Bcer	NOU	Bcer	FALSE	NA	NA	NA
Bc_ctg1_3679	B1	8.13729294	Bcer	S	Bcer	FALSE	NA	NA	NA
Bc_ctg1_368	B1	0.297705839	Bcer	S	Bcer	FALSE	Bc_Ga0417192_01_rn04	Other	NA
Bc_ctg1_3680	B1	519.3974543	Bcer	P	Bcer	FALSE	NA	NA	NA
Bc_ctg1_3681	B1	8.13729294	Bcer	P	Bcer	FALSE	NA	NA	NA
Bc_ctg1_3682	B1	0.793882238	Bcer	P	Bcer	FALSE	NA	NA	NA
Bc_ctg1_3683	B1	0.793882238	Bcer	S	Bcer	FALSE	NA	NA	NA
Bc_ctg1_3684	B1	214.943616	Bcer	I	Bcer	FALSE	NA	NA	NA
Bc_ctg1_3685	B1	599.5795603	Bcer	S	Bcer	FALSE	NA	NA	NA
Bc_ctg1_3686	B1	209.8826167	Bcer	K	Bcer	FALSE	NA	NA	NA
Bc_ctg1_3687	B1	54.38093331	Bcer	S	Bcer	FALSE	NA	NA	NA
Bc_ctg1_3688	B1	95.66280969	Bcer	IQ	Bcer	TRUE	NA	NA	NA
Bc_ctg1_3689	B1	107.5710433	Bcer	I	Bcer	FALSE	NA	NA	NA
Bc_ctg1_369	B1	2.977058393	Bcer	K	Bcer	FALSE	Bc_Ga0417192_01_rn04	Regulation	SMCOG1014
Bc_ctg1_3690	B1	35.92317127	Bcer	E	Bcer	FALSE	NA	NA	NA
Bc_ctg1_3691	B1	92.48728074	Bcer	P	Bcer	FALSE	NA	NA	NA
Bc_ctg1_3694	B1	0.09923528	Bcer	P	Bcer	FALSE	NA	NA	NA
Bc_ctg1_3695	B1	1.091588077	Bcer	P	Bcer	FALSE	NA	NA	NA
Bc_ctg1_3696	B1	1.488529196	Bcer	P	Bcer	FALSE	NA	NA	NA
Bc_ctg1_3697	B1	0.19847056	Bcer	S	Bcer	FALSE	NA	NA	NA
Bc_ctg1_3698	B1	0.793882238	Bcer	E	Bcer	FALSE	NA	NA	NA
Bc_ctg1_3699	B1	2.877823113	Bcer	E	Bcer	FALSE	NA	NA	NA
Bc_ctg1_37	B1	0.19847056	Bcer	S	Bcer	FALSE	NA	NA	NA
Bc_ctg1_370	B1	1.984705595	Bcer	C	Bcer	FALSE	Bc_Ga0417192_01_rn04	Ancillary biosynthesis	SMCOG1028
Bc_ctg1_3700	B1	1.885470315	Bcer	K	Bcer	FALSE	NA	NA	NA
Bc_ctg1_3702	B1	232.9052016	Bcer	M	Bcer	FALSE	NA	NA	NA
Bc_ctg1_3703	B1	0.992352798	Bcer	E	Bcer	FALSE	NA	NA	NA
Bc_ctg1_3704	B1	3.473234792	Bcer	S	Bcer	FALSE	NA	NA	NA
Bc_ctg1_3705	B1	1.190823357	Bcer	S	Bcer	FALSE	NA	NA	NA
Bc_ctg1_3706	B1	0.694646958	Bcer	S	Bcer	FALSE	NA	NA	NA
Bc_ctg1_3707	B1	28.08358417	Bcer	T	Bcer	FALSE	NA	NA	NA
Bc_ctg1_3708	B1	26.89276082	Bcer	K	Bcer	FALSE	NA	NA	NA
Bc_ctg1_3709	B1	992.6505035	Bcer	E	Bcer	FALSE	NA	NA	NA
Bc_ctg1_371	B1	8.23652822	Bcer	G	Bcer	FALSE	Bc_Ga0417192_01_rn04	Ancillary biosynthesis	SMCOG1101
Bc_ctg1_3710	B1	71.74710727	Bcer	S	Bcer	FALSE	NA	NA	NA
Bc_ctg1_3711	B1	0	Bcer	NA	Bcer	FALSE	NA	NA	NA
Bc_ctg1_3712	B1	94.37275105	Bcer	T	Bcer	FALSE	NA	NA	NA
Bc_ctg1_3713	B1	56.96105058	Bcer	F	Bcer	FALSE	NA	NA	NA
Bc_ctg1_3714	B1	51.99928659	Bcer	H	Bcer	FALSE	NA	NA	NA
Bc_ctg1_3715	B1	43.66352309	Bcer	H	Bcer	FALSE	NA	NA	NA
Bc_ctg1_3716	B1	51.20540436	Bcer	S	Bcer	FALSE	NA	NA	NA
Bc_ctg1_3717	B1	6.549528464	Bcer	S	Bcer	FALSE	NA	NA	NA
Bc_ctg1_3718	B1	0.19847056	Bcer	NA	Bcer	FALSE	NA	NA	NA
Bc_ctg1_3719	B1	35.42699487	Bcer	G	Bcer	FALSE	NA	NA	NA
Bc_ctg1_372	B1	1.587764476	Bcer	S	Bcer	FALSE	Bc_Ga0417192_01_rn04	Other	NA
Bc_ctg1_3720	B1	102.3115734	Bcer	K	Bcer	FALSE	NA	NA	NA
Bc_ctg1_3721	B1	6.450293184	Bcer	NA	Bcer	FALSE	NA	NA	NA
Bc_ctg1_3722	B1	18.45776204	Bcer	NA	Bcer	FALSE	NA	NA	NA
Bc_ctg1_3723	B1	87.82322259	Bcer	F	Bcer	FALSE	NA	NA	NA
Bc_ctg1_3724	B1	890.7358711	Bcer	F	Bcer	FALSE	NA	NA	NA
Bc_ctg1_3725	B1	187.2569729	Bcer	F	Bcer	FALSE	NA	NA	NA
Bc_ctg1_3726	B1	383.8420621	Bcer	L	Bcer	FALSE	NA	NA	NA
Bc_ctg1_3727	B1	138.33398	Bcer	F	Bcer	FALSE	NA	NA	NA
Bc_ctg1_3728	B1	9.328116298	Bcer	K	Bcer	FALSE	NA	NA	NA
Bc_ctg1_3729	B1	19.05317371	Bcer	V	Bcer	FALSE	NA	NA	NA
Bc_ctg1_373	B1	5.755646226	Bcer	L	Bcer	FALSE	Bc_Ga0417192_01_rn04	Other	NA
Bc_ctg1_3730	B1	11.51129245	Bcer	NA	Bcer	FALSE	NA	NA	NA

Bc_ctg1_3731	B1	46.44211093	Bcer	V	Bcer	FALSE	NA	NA	NA
Bc_ctg1_3732	B1	83.05992916	Bcer	S	Bcer	FALSE	NA	NA	NA
Bc_ctg1_3733	B1	38.00711215	Bcer	S	Bcer	FALSE	NA	NA	NA
Bc_ctg1_3734	B1	0	Bcer	NA	Bcer	FALSE	NA	NA	NA
Bc_ctg1_3735	B1	4.564822869	Bcer	K	Bcer	FALSE	NA	NA	NA
Bc_ctg1_3736	B1	1.488529196	Bcer	S	Bcer	FALSE	NA	NA	NA
Bc_ctg1_3737	B1	2.877823113	Bcer	Q	Bcer	TRUE	NA	NA	NA
Bc_ctg1_3738	B1	41.77805278	Bcer	S	Bcer	FALSE	NA	NA	NA
Bc_ctg1_3739	B1	6.450293184	Bcer	V	Bcer	FALSE	NA	NA	NA
Bc_ctg1_374	B1	0.396941119	Bcer	NA	Bcer	FALSE	Bc_Ga0417192_01_rn04	Other	NA
Bc_ctg1_3740	B1	9.328116298	Bcer	S	Bcer	FALSE	NA	NA	NA
Bc_ctg1_3741	B1	215.6382629	Bcer	M	Bcer	FALSE	NA	NA	NA
Bc_ctg1_3742	B1	105.9832788	Bcer	IQ	Bcer	TRUE	NA	NA	NA
Bc_ctg1_3743	B1	544.3055095	Bcer	M	Bcer	FALSE	NA	NA	NA
Bc_ctg1_3744	B1	685.4180773	Bcer	Q	Bcer	TRUE	NA	NA	NA
Bc_ctg1_3745	B1	2.381646714	Bcer	NA	Bcer	FALSE	NA	NA	NA
Bc_ctg1_3746	B1	0.496176399	Bcer	E	Bcer	FALSE	NA	NA	NA
Bc_ctg1_3747	B1	0.496176399	Bcer	NA	Bcer	FALSE	NA	NA	NA
Bc_ctg1_3748	B1	4.862528708	Bcer	C	Bcer	FALSE	NA	NA	NA
Bc_ctg1_3749	B1	16.1753506	Bcer	NA	Bcer	FALSE	NA	NA	NA
Bc_ctg1_375	B1	4.564822869	Bcer	G	Bcer	FALSE	Bc_Ga0417192_01_rn04	Core biosynthesis	SMCOG1257
Bc_ctg1_3750	B1	0.19847056	Bcer	NA	Bcer	FALSE	NA	NA	NA
Bc_ctg1_3751	B1	4.664058149	Bcer	S	Bcer	FALSE	NA	NA	NA
Bc_ctg1_3752	B1	1.587764476	Bcer	NA	Bcer	FALSE	NA	NA	NA
Bc_ctg1_3754	B1	1.190823357	Bcer	NA	Bcer	FALSE	NA	NA	NA
Bc_ctg1_3755	B1	6.152587345	Bcer	V	Bcer	FALSE	NA	NA	NA
Bc_ctg1_3756	B1	194.9973247	Bcer	V	Bcer	FALSE	NA	NA	NA
Bc_ctg1_3757	B1	67.97616664	Bcer	CO	Bcer	FALSE	NA	NA	NA
Bc_ctg1_3758	B1	6.946469583	Bcer	S	Bcer	FALSE	NA	NA	NA
Bc_ctg1_3759	B1	29.37364281	Bcer	K	Bcer	FALSE	NA	NA	NA
Bc_ctg1_376	B1	15.1829978	Bcer	Q	Bcer	TRUE	Bc_Ga0417192_01_rn04	Other	NA
Bc_ctg1_3761	B1	7.839587101	Bcer	C	Bcer	FALSE	NA	NA	NA
Bc_ctg1_3762	B1	304.0568972	Bcer	P	Bcer	FALSE	NA	NA	NA
Bc_ctg1_3763	B1	0.297705839	Bcer	NA	Bcer	FALSE	NA	NA	NA
Bc_ctg1_3764	B1	0.297705839	Bcer	S	Bcer	FALSE	NA	NA	NA
Bc_ctg1_3766	B1	0.595411679	Bcer	S	Bcer	FALSE	NA	NA	NA
Bc_ctg1_3767	B1	2.877823113	Bcer	S	Bcer	FALSE	NA	NA	NA
Bc_ctg1_3768	B1	7.343410702	Bcer	E	Bcer	FALSE	NA	NA	NA
Bc_ctg1_3769	B1	7.938822381	Bcer	E	Bcer	FALSE	NA	NA	NA
Bc_ctg1_377	B1	134.2653335	Bcer	E	Bcer	FALSE	Bc_Ga0417192_01_rn04	Other	NA
Bc_ctg1_3770	B1	1.091588077	Bcer	E	Bcer	FALSE	NA	NA	NA
Bc_ctg1_3771	B1	1.984705595	Bcer	EH	Bcer	FALSE	NA	NA	NA
Bc_ctg1_3772	B1	5.457940387	Bcer	E	Bcer	FALSE	NA	NA	NA
Bc_ctg1_3773	B1	0.893117518	Bcer	CE	Bcer	FALSE	NA	NA	NA
Bc_ctg1_3774	B1	2.083940875	Bcer	E	Bcer	FALSE	NA	NA	NA
Bc_ctg1_3775	B1	1.488529196	Bcer	E	Bcer	FALSE	NA	NA	NA
Bc_ctg1_3776	B1	8.534234059	Bcer	E	Bcer	FALSE	NA	NA	NA
Bc_ctg1_3777	B1	6.747999024	Bcer	E	Bcer	FALSE	NA	NA	NA
Bc_ctg1_3778	B1	3.870175911	Bcer	E	Bcer	FALSE	NA	NA	NA
Bc_ctg1_3779	B1	1.290058637	Bcer	E	Bcer	FALSE	NA	NA	NA
Bc_ctg1_378	B1	0.297705839	Bcer	E	Bcer	FALSE	Bc_Ga0417192_01_rn04	Other	NA
Bc_ctg1_3780	B1	0.595411679	Bcer	E	Bcer	FALSE	NA	NA	NA
Bc_ctg1_3781	B1	1.290058637	Bcer	E	Bcer	FALSE	NA	NA	NA
Bc_ctg1_3782	B1	2.679352554	Bcer	E	Bcer	FALSE	NA	NA	NA
Bc_ctg1_3783	B1	0	Bcer	E	Bcer	FALSE	NA	NA	NA
Bc_ctg1_3784	B1	1.190823357	Bcer	E	Bcer	FALSE	NA	NA	NA
Bc_ctg1_3785	B1	9.228881018	Bcer	E	Bcer	FALSE	NA	NA	NA
Bc_ctg1_3786	B1	0.793882238	Bcer	NA	Bcer	FALSE	NA	NA	NA
Bc_ctg1_3787	B1	101.6169265	Bcer	CH	Bcer	FALSE	NA	NA	NA
Bc_ctg1_3788	B1	71.64787199	Bcer	I	Bcer	FALSE	NA	NA	NA
Bc_ctg1_3789	B1	176.9365038	Bcer	M	Bcer	FALSE	NA	NA	NA
Bc_ctg1_379	B1	3.96941119	Bcer	K	Bcer	FALSE	Bc_Ga0417192_01_rn04	Regulation	SMCOG1171

Bc_ctg1_3790	B1	0.694646958	Bcer	S	Bcer	FALSE	NA	NA	NA
Bc_ctg1_3791	B1	54.67863915	Bcer	E	Bcer	FALSE	NA	NA	NA
Bc_ctg1_3792	B1	0	Bcer	S	Bcer	FALSE	NA	NA	NA
Bc_ctg1_3793	B1	15.97688004	Bcer	EH	Bcer	FALSE	NA	NA	NA
Bc_ctg1_3794	B1	48.82375764	Bcer	P	Bcer	FALSE	NA	NA	NA
Bc_ctg1_3795	B1	29.86981921	Bcer	P	Bcer	FALSE	NA	NA	NA
Bc_ctg1_3796	B1	128.410452	Bcer	P	Bcer	FALSE	NA	NA	NA
Bc_ctg1_3797	B1	15.38146836	Bcer	S	Bcer	FALSE	NA	NA	NA
Bc_ctg1_3798	B1	7.343410702	Bcer	H	Bcer	FALSE	NA	NA	NA
Bc_ctg1_3799	B1	11.90823357	Bcer	S	Bcer	FALSE	NA	NA	NA
Bc_ctg1_38	B1	0.09923528	Bcer	S	Bcer	FALSE	NA	NA	NA
Bc_ctg1_380	B1	2.580117274	Bcer	E	Bcer	FALSE	Bc_Ga0417192_01_rn04	Other	NA
Bc_ctg1_3800	B1	9.328116298	Bcer	H	Bcer	FALSE	NA	NA	NA
Bc_ctg1_3801	B1	19.25164427	Bcer	S	Bcer	FALSE	NA	NA	NA
Bc_ctg1_3802	B1	0.396941119	Bcer	NA	Bcer	FALSE	NA	NA	NA
Bc_ctg1_3803	B1	253.6453751	Bcer	D	Bcer	FALSE	NA	NA	NA
Bc_ctg1_3804	B1	751.5087736	Bcer	C	Bcer	FALSE	NA	NA	NA
Bc_ctg1_3805	B1	2.381646714	Bcer	S	Bcer	FALSE	NA	NA	NA
Bc_ctg1_3806	B1	49.71687516	Bcer	C	Bcer	FALSE	NA	NA	NA
Bc_ctg1_3807	B1	99.43375032	Bcer	NA	Bcer	FALSE	NA	NA	NA
Bc_ctg1_3808	B1	440.207701	Bcer	KT	Bcer	FALSE	NA	NA	NA
Bc_ctg1_3809	B1	26.29734914	Bcer	S	Bcer	FALSE	NA	NA	NA
Bc_ctg1_381	B1	0.19847056	Bcer	S	Bcer	FALSE	Bc_Ga0417192_01_rn04	Other	NA
Bc_ctg1_3810	B1	37.70940631	Bcer	T	Bcer	FALSE	NA	NA	NA
Bc_ctg1_3811	B1	41.28187638	Bcer	KT	Bcer	FALSE	NA	NA	NA
Bc_ctg1_3812	B1	23.12182018	Bcer	NA	Bcer	FALSE	NA	NA	NA
Bc_ctg1_3813	B1	10.41970437	Bcer	E	Bcer	FALSE	NA	NA	NA
Bc_ctg1_3814	B1	83.8538114	Bcer	NA	Bcer	FALSE	NA	NA	NA
Bc_ctg1_3815	B1	20.54170291	Bcer	Q	Bcer	TRUE	NA	NA	NA
Bc_ctg1_3816	B1	13.19829221	Bcer	NA	Bcer	FALSE	NA	NA	NA
Bc_ctg1_3818	B1	7.641116542	Bcer	S	Bcer	FALSE	NA	NA	NA
Bc_ctg1_3819	B1	4.16788175	Bcer	NA	Bcer	FALSE	NA	NA	NA
Bc_ctg1_382	B1	0	Bcer	E	Bcer	FALSE	Bc_Ga0417192_01_rn04	Other	NA
Bc_ctg1_3820	B1	470.8714025	Bcer	C	Bcer	FALSE	NA	NA	NA
Bc_ctg1_3821	B1	10.02276326	Bcer	T	Bcer	FALSE	NA	NA	NA
Bc_ctg1_3822	B1	0.992352798	Bcer	NA	Bcer	FALSE	NA	NA	NA
Bc_ctg1_3823	B1	169.2953873	Bcer	S	Bcer	FALSE	NA	NA	NA
Bc_ctg1_3824	B1	567.9235061	Bcer	P	Bcer	FALSE	NA	NA	NA
Bc_ctg1_3825	B1	366.9720646	Bcer	P	Bcer	FALSE	NA	NA	NA
Bc_ctg1_3826	B1	27.19046665	Bcer	M	Bcer	FALSE	NA	NA	NA
Bc_ctg1_3827	B1	0.297705839	Bcer	NA	Bcer	FALSE	NA	NA	NA
Bc_ctg1_3828	B1	0	Bcer	NA	Bcer	FALSE	NA	NA	NA
Bc_ctg1_3829	B1	42.96887614	Bcer	T	Bcer	FALSE	NA	NA	NA
Bc_ctg1_383	B1	108.6626313	Bcer	V	Bcer	FALSE	Bc_Ga0417192_01_rn04	Ancillary biosynthesis	SMCOG1053
Bc_ctg1_3830	B1	7.244175423	Bcer	S	Bcer	FALSE	NA	NA	NA
Bc_ctg1_3831	B1	27.48817249	Bcer	S	Bcer	FALSE	NA	NA	NA
Bc_ctg1_3832	B1	1293.333401	Bcer	L	Bcer	FALSE	NA	NA	NA
Bc_ctg1_3833	B1	197.7759126	Bcer	T	Bcer	FALSE	NA	NA	NA
Bc_ctg1_3834	B1	412.0248816	Bcer	F	Bcer	FALSE	NA	NA	NA
Bc_ctg1_3835	B1	32.3507012	Bcer	S	Bcer	FALSE	NA	NA	NA
Bc_ctg1_3836	B1	177.5319155	Bcer	S	Bcer	FALSE	NA	NA	NA
Bc_ctg1_3837	B1	4.16788175	Bcer	NA	Bcer	FALSE	NA	NA	NA
Bc_ctg1_3838	B1	11.01511605	Bcer	S	Bcer	FALSE	NA	NA	NA
Bc_ctg1_3839	B1	0.595411679	Bcer	P	Bcer	FALSE	NA	NA	NA
Bc_ctg1_384	B1	2.480881994	Bcer	NA	Bcer	FALSE	NA	NA	NA
Bc_ctg1_3840	B1	0.793882238	Bcer	M	Bcer	FALSE	NA	NA	NA
Bc_ctg1_3841	B1	1.488529196	Bcer	S	Bcer	FALSE	NA	NA	NA
Bc_ctg1_3842	B1	0.694646958	Bcer	S	Bcer	FALSE	NA	NA	NA
Bc_ctg1_3843	B1	113.4259248	Bcer	J	Bcer	FALSE	NA	NA	NA
Bc_ctg1_3844	B1	42.86964086	Bcer	CO	Bcer	FALSE	NA	NA	NA
Bc_ctg1_3845	B1	38.70175911	Bcer	O	Bcer	FALSE	NA	NA	NA
Bc_ctg1_3846	B1	71.94557783	Bcer	O	Bcer	FALSE	NA	NA	NA

Bc_ctg1_3847	B1	169.7915637	Bcer	T	Bcer	FALSE	NA	NA	NA
Bc_ctg1_3848	B1	350.3005376	Bcer	T	Bcer	FALSE	NA	NA	NA
Bc_ctg1_3849	B1	3.870175911	Bcer	S	Bcer	FALSE	NA	NA	NA
Bc_ctg1_385	B1	2.877823113	Bcer	G	Bcer	FALSE	NA	NA	NA
Bc_ctg1_3850	B1	2.083940875	Bcer	M	Bcer	FALSE	NA	NA	NA
Bc_ctg1_3851	B1	108.0672197	Bcer	M	Bcer	FALSE	NA	NA	NA
Bc_ctg1_3852	B1	119.9754532	Bcer	U	Bcer	FALSE	NA	NA	NA
Bc_ctg1_3853	B1	182.3944442	Bcer	C	Bcer	FALSE	NA	NA	NA
Bc_ctg1_3854	B1	36.61781823	Bcer	S	Bcer	FALSE	NA	NA	NA
Bc_ctg1_3855	B1	48.72452236	Bcer	L	Bcer	FALSE	NA	NA	NA
Bc_ctg1_3856	B1	53.68628635	Bcer	S	Bcer	FALSE	NA	NA	NA
Bc_ctg1_3857	B1	48.72452236	Bcer	M	Bcer	FALSE	NA	NA	NA
Bc_ctg1_3858	B1	131.9829221	Bcer	S	Bcer	FALSE	NA	NA	NA
Bc_ctg1_3859	B1	204.325441	Bcer	K	Bcer	FALSE	NA	NA	NA
Bc_ctg1_386	B1	9.427351577	Bcer	K	Bcer	FALSE	NA	NA	NA
Bc_ctg1_3860	B1	163.1427999	Bcer	NOT	Bcer	FALSE	NA	NA	NA
Bc_ctg1_3861	B1	2055.361114	Bcer	E	Bcer	FALSE	NA	NA	NA
Bc_ctg1_3862	B1	0	Bcer	NA	Bcer	FALSE	NA	NA	NA
Bc_ctg1_3863	B1	3.076293673	Bcer	S	Bcer	FALSE	NA	NA	NA
Bc_ctg1_3864	B1	6.747999024	Bcer	M	Bcer	FALSE	NA	NA	NA
Bc_ctg1_3865	B1	16.07611532	Bcer	O	Bcer	FALSE	NA	NA	NA
Bc_ctg1_3866	B1	123.448688	Bcer	EJ	Bcer	FALSE	NA	NA	NA
Bc_ctg1_3867	B1	0	Bcer	S	Bcer	FALSE	NA	NA	NA
Bc_ctg1_3868	B1	71.84634255	Bcer	F	Bcer	FALSE	NA	NA	NA
Bc_ctg1_3869	B1	1331.83669	Bcer	J	Bcer	FALSE	NA	NA	NA
Bc_ctg1_387	B1	5.854881506	Bcer	S	Bcer	FALSE	NA	NA	NA
Bc_ctg1_3870	B1	43.66352309	Bcer	C	Bcer	FALSE	NA	NA	NA
Bc_ctg1_3872	B1	0	Bcer	NA	Bcer	FALSE	NA	NA	NA
Bc_ctg1_3873	B1	0	Bcer	S	Bcer	FALSE	NA	NA	NA
Bc_ctg1_3875	B1	414.7042341	Bcer	S	Bcer	FALSE	NA	NA	NA
Bc_ctg1_3876	B1	295.7211337	Bcer	I	Bcer	FALSE	NA	NA	NA
Bc_ctg1_3877	B1	0.992352798	Bcer	S	Bcer	FALSE	NA	NA	NA
Bc_ctg1_3878	B1	1.786235036	Bcer	S	Bcer	FALSE	NA	NA	NA
Bc_ctg1_3879	B1	8.831939899	Bcer	NA	Bcer	FALSE	NA	NA	NA
Bc_ctg1_388	B1	63.60981433	Bcer	C	Bcer	FALSE	NA	NA	NA
Bc_ctg1_3880	B1	3.770940631	Bcer	S	Bcer	FALSE	NA	NA	NA
Bc_ctg1_3881	B1	1323.103985	Bcer	L	Bcer	FALSE	NA	NA	NA
Bc_ctg1_3882	B1	278.7519008	Bcer	H	Bcer	FALSE	NA	NA	NA
Bc_ctg1_3883	B1	64.99910824	Bcer	H	Bcer	FALSE	NA	NA	NA
Bc_ctg1_3884	B1	64.30446129	Bcer	H	Bcer	FALSE	NA	NA	NA
Bc_ctg1_3885	B1	101.815397	Bcer	H	Bcer	FALSE	NA	NA	NA
Bc_ctg1_3886	B1	1392.965622	Bcer	F	Bcer	FALSE	NA	NA	NA
Bc_ctg1_3887	B1	61.62510873	Bcer	E	Bcer	FALSE	NA	NA	NA
Bc_ctg1_3888	B1	121.9601588	Bcer	E	Bcer	FALSE	NA	NA	NA
Bc_ctg1_3889	B1	366.6743587	Bcer	E	Bcer	FALSE	NA	NA	NA
Bc_ctg1_389	B1	8.3357635	Bcer	NA	Bcer	FALSE	NA	NA	NA
Bc_ctg1_3890	B1	116.700689	Bcer	S	Bcer	FALSE	NA	NA	NA
Bc_ctg1_3891	B1	152.3261544	Bcer	S	Bcer	FALSE	Bc_Ga0417192_01_rn27	Other	NA
Bc_ctg1_3892	B1	15.97688004	Bcer	S	Bcer	FALSE	Bc_Ga0417192_01_rn27	Other	NA
Bc_ctg1_3893	B1	15.77840948	Bcer	C	Bcer	FALSE	Bc_Ga0417192_01_rn27	Other	NA
Bc_ctg1_3894	B1	26.9919961	Bcer	C	Bcer	FALSE	Bc_Ga0417192_01_rn27	Other	NA
Bc_ctg1_3895	B1	13.99217445	Bcer	C	Bcer	FALSE	Bc_Ga0417192_01_rn27	Other	NA
Bc_ctg1_3896	B1	22.22870267	Bcer	S	Bcer	FALSE	Bc_Ga0417192_01_rn27	Other	NA
Bc_ctg1_3897	B1	21.23634987	Bcer	S	Bcer	FALSE	Bc_Ga0417192_01_rn27	Other	NA
Bc_ctg1_3898	B1	3.770940631	Bcer	S	Bcer	FALSE	Bc_Ga0417192_01_rn27	Other	NA
Bc_ctg1_3899	B1	286.591488	Bcer	S	Bcer	FALSE	Bc_Ga0417192_01_rn27	Other	NA
Bc_ctg1_39	B1	2.977058393	Bcer	J	Bcer	FALSE	NA	NA	NA
Bc_ctg1_390	B1	5.457940387	Bcer	NA	Bcer	FALSE	NA	NA	NA
Bc_ctg1_3900	B1	0.793882238	Bcer	L	Bcer	FALSE	Bc_Ga0417192_01_rn27	Other	NA
Bc_ctg1_3901	B1	14.78605668	Bcer	S	Bcer	FALSE	Bc_Ga0417192_01_rn27	Other	NA
Bc_ctg1_3902	B1	6.648763744	Bcer	S	Bcer	FALSE	Bc_Ga0417192_01_rn27	Other	NA
Bc_ctg1_3903	B1	107.2733374	Bcer	E	Bcer	FALSE	Bc_Ga0417192_01_rn27	Other	NA

Bc_ctg1_3904	B1	57.65569754	Bcer	G	Bcer	FALSE	Bc_Ga0417192_01_rn27	Other	NA
Bc_ctg1_3905	B1	76.21269486	Bcer	S	Bcer	FALSE	Bc_Ga0417192_01_rn27	Core biosynthesis	None
Bc_ctg1_3906	B1	122.1586294	Bcer	M	Bcer	FALSE	Bc_Ga0417192_01_rn27	Core biosynthesis	SMCOG1045
Bc_ctg1_3907	B1	188.8447374	Bcer	J	Bcer	FALSE	Bc_Ga0417192_01_rn27	Other	NA
Bc_ctg1_3908	B1	126.624217	Bcer	K	Bcer	FALSE	Bc_Ga0417192_01_rn27	Other	NA
Bc_ctg1_3909	B1	0	Bcer	C	Bcer	FALSE	Bc_Ga0417192_01_rn27	Other	NA
Bc_ctg1_391	B1	4.465587589	Bcer	S	Bcer	FALSE	NA	NA	NA
Bc_ctg1_3910	B1	69.46469583	Bcer	H	Bcer	FALSE	Bc_Ga0417192_01_rn27	Other	NA
Bc_ctg1_3911	B1	124.3418055	Bcer	H	Bcer	FALSE	Bc_Ga0417192_01_rn27	Other	NA
Bc_ctg1_3912	B1	16.96923284	Bcer	H	Bcer	FALSE	Bc_Ga0417192_01_rn27	Other	NA
Bc_ctg1_3913	B1	301.4767799	Bcer	L	Bcer	FALSE	Bc_Ga0417192_01_rn27	Other	SMCOG1194
Bc_ctg1_3914	B1	38.00711215	Bcer	S	Bcer	FALSE	Bc_Ga0417192_01_rn27	Other	NA
Bc_ctg1_3915	B1	99.5329856	Bcer	S	Bcer	FALSE	Bc_Ga0417192_01_rn27	Other	NA
Bc_ctg1_3916	B1	892.9190473	Bcer	E	Bcer	FALSE	Bc_Ga0417192_01_rn27	Core biosynthesis	SMCOG1019
Bc_ctg1_3917	B1	53.38858051	Bcer	L	Bcer	FALSE	Bc_Ga0417192_01_rn27	Other	NA
Bc_ctg1_3918	B1	56.26640362	Bcer	L	Bcer	FALSE	Bc_Ga0417192_01_rn27	Other	NA
Bc_ctg1_3919	B1	23.12182018	Bcer	NA	Bcer	FALSE	Bc_Ga0417192_01_rn27	Other	NA
Bc_ctg1_392	B1	39.89258246	Bcer	NA	Bcer	FALSE	NA	NA	NA
Bc_ctg1_3920	B1	256.3247276	Bcer	M	Bcer	FALSE	Bc_Ga0417192_01_rn27	Other	NA
Bc_ctg1_3921	B1	19.94629123	Bcer	L	Bcer	FALSE	Bc_Ga0417192_01_rn27	Other	NA
Bc_ctg1_3922	B1	2.083940875	Bcer	S	Bcer	FALSE	Bc_Ga0417192_01_rn27	Other	NA
Bc_ctg1_3924	B1	2.977058393	Bcer	NA	Bcer	FALSE	Bc_Ga0417192_01_rn27	Other	NA
Bc_ctg1_3925	B1	3.870175911	Bcer	S	Bcer	FALSE	Bc_Ga0417192_01_rn27	Other	NA
Bc_ctg1_3926	B1	12.60288053	Bcer	S	Bcer	FALSE	Bc_Ga0417192_01_rn27	Other	NA
Bc_ctg1_3927	B1	0.297705839	Bcer	NA	Bcer	FALSE	Bc_Ga0417192_01_rn27	Other	NA
Bc_ctg1_3928	B1	0.297705839	Bcer	S	Bcer	FALSE	Bc_Ga0417192_01_rn27	Other	NA
Bc_ctg1_3929	B1	5.557175667	Bcer	S	Bcer	FALSE	Bc_Ga0417192_01_rn27	Other	NA
Bc_ctg1_393	B1	59.73963842	Bcer	S	Bcer	FALSE	NA	NA	NA
Bc_ctg1_3930	B1	239.2562595	Bcer	D	Bcer	FALSE	NA	NA	NA
Bc_ctg1_3931	B1	112.433572	Bcer	L	Bcer	FALSE	NA	NA	NA
Bc_ctg1_3932	B1	10.3204691	Bcer	S	Bcer	FALSE	NA	NA	NA
Bc_ctg1_3933	B1	87.62475203	Bcer	KL	Bcer	FALSE	NA	NA	NA
Bc_ctg1_3934	B1	533.9850404	Bcer	E	Bcer	FALSE	NA	NA	NA
Bc_ctg1_3935	B1	0.297705839	Bcer	L	Bcer	FALSE	NA	NA	NA
Bc_ctg1_3937	B1	0.595411679	Bcer	J	Bcer	FALSE	NA	NA	NA
Bc_ctg1_3938	B1	11.80899829	Bcer	E	Bcer	FALSE	NA	NA	NA
Bc_ctg1_3939	B1	74.22798926	Bcer	F	Bcer	FALSE	NA	NA	NA
Bc_ctg1_394	B1	41.57958222	Bcer	NA	Bcer	FALSE	NA	NA	NA
Bc_ctg1_3940	B1	599.480325	Bcer	F	Bcer	FALSE	NA	NA	NA
Bc_ctg1_3941	B1	0	Bcer	NA	Bcer	FALSE	NA	NA	NA
Bc_ctg1_3943	B1	4.862528708	Bcer	P	Bcer	FALSE	NA	NA	NA
Bc_ctg1_3945	B1	220.5007916	Bcer	S	Bcer	FALSE	NA	NA	NA
Bc_ctg1_3946	B1	0.09923528	Bcer	S	Bcer	FALSE	NA	NA	NA
Bc_ctg1_3947	B1	34.93081848	Bcer	K	Bcer	FALSE	NA	NA	NA
Bc_ctg1_3948	B1	38.90022967	Bcer	S	Bcer	FALSE	Bc_Ga0417192_01_rn28	Other	NA
Bc_ctg1_3949	B1	171.5777987	Bcer	P	Bcer	FALSE	Bc_Ga0417192_01_rn28	Other	NA
Bc_ctg1_395	B1	3.473234792	Bcer	CH	Bcer	FALSE	NA	NA	NA
Bc_ctg1_3950	B1	7.144940143	Bcer	NA	Bcer	FALSE	Bc_Ga0417192_01_rn28	Other	NA
Bc_ctg1_3951	B1	10.3204691	Bcer	NA	Bcer	FALSE	Bc_Ga0417192_01_rn28	Other	NA
Bc_ctg1_3952	B1	36.12164183	Bcer	E	Bcer	FALSE	Bc_Ga0417192_01_rn28	Other	NA
Bc_ctg1_3953	B1	7.442645982	Bcer	S	Bcer	FALSE	Bc_Ga0417192_01_rn28	Other	NA
Bc_ctg1_3954	B1	286.6907232	Bcer	P	Bcer	FALSE	Bc_Ga0417192_01_rn28	Other	NA
Bc_ctg1_3955	B1	81.47216468	Bcer	L	Bcer	FALSE	Bc_Ga0417192_01_rn28	Other	NA
Bc_ctg1_3956	B1	0.09923528	Bcer	NA	Bcer	FALSE	Bc_Ga0417192_01_rn28	Other	NA
Bc_ctg1_3957	B1	2.282411435	Bcer	I	Bcer	FALSE	Bc_Ga0417192_01_rn28	Ancillary biosynthesis	SMCOG1088
Bc_ctg1_3958	B1	1.587764476	Bcer	M	Bcer	FALSE	Bc_Ga0417192_01_rn28	Ancillary biosynthesis	SMCOG1216
Bc_ctg1_3959	B1	1.488529196	Bcer	D	Bcer	FALSE	Bc_Ga0417192_01_rn28	Other	NA
Bc_ctg1_396	B1	0.297705839	Bcer	K	Bcer	FALSE	NA	NA	NA
Bc_ctg1_3960	B1	2.183176155	Bcer	M	Bcer	FALSE	Bc_Ga0417192_01_rn28	Core biosynthesis	SMCOG1146
Bc_ctg1_3961	B1	5.557175667	Bcer	S	Bcer	FALSE	Bc_Ga0417192_01_rn28	Core biosynthesis	None
Bc_ctg1_3962	B1	3.671705351	Bcer	M	Bcer	FALSE	Bc_Ga0417192_01_rn28	Core biosynthesis	None
Bc_ctg1_3963	B1	2.679352554	Bcer	M	Bcer	FALSE	Bc_Ga0417192_01_rn28	Core biosynthesis	None

Bc_ctg1_3964	B1	1.190823357	Bcer	M	Bcer	FALSE	Bc_Ga0417192_01_rn28	Core biosynthesis	SMCOG1045
Bc_ctg1_3965	B1	0.595411679	Bcer	M	Bcer	FALSE	Bc_Ga0417192_01_rn28	Other	NA
Bc_ctg1_3966	B1	4.465587589	Bcer	M	Bcer	FALSE	NA	NA	NA
Bc_ctg1_3967	B1	1.290058637	Bcer	K	Bcer	FALSE	NA	NA	NA
Bc_ctg1_3968	B1	28.67899585	Bcer	Q	Bcer	TRUE	NA	NA	NA
Bc_ctg1_3969	B1	9.228881018	Bcer	NA	Bcer	FALSE	NA	NA	NA
Bc_ctg1_397	B1	5.656410946	Bcer	C	Bcer	FALSE	NA	NA	NA
Bc_ctg1_3970	B1	12.70211581	Bcer	EG	Bcer	FALSE	NA	NA	NA
Bc_ctg1_3971	B1	3.076293673	Bcer	L	Bcer	FALSE	NA	NA	NA
Bc_ctg1_3972	B1	55.96869779	Bcer	G	Bcer	FALSE	NA	NA	NA
Bc_ctg1_3973	B1	4.465587589	Bcer	S	Bcer	FALSE	NA	NA	NA
Bc_ctg1_3974	B1	6.152587345	Bcer	L	Bcer	FALSE	NA	NA	NA
Bc_ctg1_3975	B1	2.183176155	Bcer	S	Bcer	FALSE	NA	NA	NA
Bc_ctg1_3976	B1	1.786235036	Bcer	S	Bcer	FALSE	NA	NA	NA
Bc_ctg1_3977	B1	12.90058637	Bcer	K	Bcer	FALSE	NA	NA	NA
Bc_ctg1_3978	B1	0.893117518	Bcer	S	Bcer	FALSE	NA	NA	NA
Bc_ctg1_3979	B1	27.88511361	Bcer	E	Bcer	FALSE	NA	NA	NA
Bc_ctg1_398	B1	93.08269242	Bcer	S	Bcer	FALSE	NA	NA	NA
Bc_ctg1_3980	B1	5.160234548	Bcer	NA	Bcer	FALSE	NA	NA	NA
Bc_ctg1_3981	B1	5.854881506	Bcer	I	Bcer	FALSE	NA	NA	NA
Bc_ctg1_3982	B1	0	Bcer	S	Bcer	FALSE	NA	NA	NA
Bc_ctg1_3983	B1	2.282411435	Bcer	S	Bcer	FALSE	NA	NA	NA
Bc_ctg1_3984	B1	61.02969705	Bcer	E	Bcer	FALSE	NA	NA	NA
Bc_ctg1_3985	B1	0	Bcer	S	Bcer	FALSE	NA	NA	NA
Bc_ctg1_3986	B1	44.35817005	Bcer	P	Bcer	FALSE	NA	NA	NA
Bc_ctg1_3987	B1	15.08376252	Bcer	KT	Bcer	FALSE	NA	NA	NA
Bc_ctg1_3988	B1	50.5107574	Bcer	V	Bcer	FALSE	NA	NA	NA
Bc_ctg1_3989	B1	0.19847056	Bcer	S	Bcer	FALSE	NA	NA	NA
Bc_ctg1_399	B1	9.725057417	Bcer	S	Bcer	FALSE	NA	NA	NA
Bc_ctg1_3990	B1	22.22870267	Bcer	S	Bcer	FALSE	NA	NA	NA
Bc_ctg1_3991	B1	2.381646714	Bcer	S	Bcer	FALSE	NA	NA	NA
Bc_ctg1_3992	B1	36.02240655	Bcer	C	Bcer	FALSE	NA	NA	NA
Bc_ctg1_3993	B1	12.00746885	Bcer	K	Bcer	FALSE	NA	NA	NA
Bc_ctg1_3994	B1	8.3357635	Bcer	NA	Bcer	FALSE	NA	NA	NA
Bc_ctg1_3995	B1	6.450293184	Bcer	S	Bcer	FALSE	NA	NA	NA
Bc_ctg1_3996	B1	2.083940875	Bcer	G	Bcer	FALSE	NA	NA	NA
Bc_ctg1_3997	B1	1.290058637	Bcer	K	Bcer	FALSE	NA	NA	NA
Bc_ctg1_3998	B1	1.984705595	Bcer	EGP	Bcer	FALSE	NA	NA	NA
Bc_ctg1_3999	B1	0.297705839	Bcer	E	Bcer	FALSE	NA	NA	NA
Bc_ctg1_4	B1	0.595411679	Bcer	F	Bcer	FALSE	NA	NA	NA
Bc_ctg1_40	B1	0	Bcer	L	Bcer	FALSE	NA	NA	NA
Bc_ctg1_400	B1	9.427351577	Bcer	NA	Bcer	FALSE	NA	NA	NA
Bc_ctg1_4001	B1	0.694646958	Bcer	NA	Bcer	FALSE	NA	NA	NA
Bc_ctg1_4002	B1	5.160234548	Bcer	S	Bcer	FALSE	NA	NA	NA
Bc_ctg1_4003	B1	56.96105058	Bcer	S	Bcer	FALSE	NA	NA	NA
Bc_ctg1_4004	B1	48.32758124	Bcer	N	Bcer	FALSE	NA	NA	NA
Bc_ctg1_4005	B1	65.99146104	Bcer	N	Bcer	FALSE	NA	NA	NA
Bc_ctg1_4006	B1	98.44139752	Bcer	T	Bcer	FALSE	NA	NA	NA
Bc_ctg1_4007	B1	657.0367873	Bcer	NT	Bcer	FALSE	NA	NA	NA
Bc_ctg1_4008	B1	152.7230956	Bcer	N	Bcer	FALSE	NA	NA	NA
Bc_ctg1_4009	B1	38.20558271	Bcer	NA	Bcer	FALSE	NA	NA	NA
Bc_ctg1_401	B1	12.00746885	Bcer	K	Bcer	FALSE	NA	NA	NA
Bc_ctg1_4010	B1	95.86128025	Bcer	NA	Bcer	FALSE	NA	NA	NA
Bc_ctg1_4011	B1	35.42699487	Bcer	NT	Bcer	FALSE	NA	NA	NA
Bc_ctg1_4012	B1	26.39658442	Bcer	NA	Bcer	FALSE	NA	NA	NA
Bc_ctg1_4013	B1	49.81611044	Bcer	S	Bcer	FALSE	NA	NA	NA
Bc_ctg1_4014	B1	42.86964086	Bcer	NA	Bcer	FALSE	NA	NA	NA
Bc_ctg1_4015	B1	407.2615881	Bcer	N	Bcer	FALSE	NA	NA	NA
Bc_ctg1_4016	B1	195.9896775	Bcer	N	Bcer	FALSE	NA	NA	NA
Bc_ctg1_4017	B1	553.0382141	Bcer	N	Bcer	FALSE	NA	NA	NA
Bc_ctg1_4018	B1	194.7988542	Bcer	N	Bcer	FALSE	NA	NA	NA
Bc_ctg1_4019	B1	97.34980945	Bcer	NA	Bcer	FALSE	NA	NA	NA

Bc_ctg1_402	B1	0.793882238	Bcer	S	Bcer	FALSE	NA	NA	NA
Bc_ctg1_4020	B1	81.8691058	Bcer	N	Bcer	FALSE	NA	NA	NA
Bc_ctg1_4021	B1	52.79316883	Bcer	N	Bcer	FALSE	NA	NA	NA
Bc_ctg1_4022	B1	30.06828977	Bcer	N	Bcer	FALSE	NA	NA	NA
Bc_ctg1_4023	B1	233.7983191	Bcer	N	Bcer	FALSE	NA	NA	NA
Bc_ctg1_4024	B1	222.6839678	Bcer	N	Bcer	FALSE	NA	NA	NA
Bc_ctg1_4025	B1	36.91552407	Bcer	N	Bcer	FALSE	NA	NA	NA
Bc_ctg1_4026	B1	183.5852676	Bcer	NU	Bcer	FALSE	NA	NA	NA
Bc_ctg1_4027	B1	113.8228659	Bcer	NA	Bcer	FALSE	NA	NA	NA
Bc_ctg1_4028	B1	229.4319668	Bcer	N	Bcer	FALSE	NA	NA	NA
Bc_ctg1_4029	B1	160.1657415	Bcer	N	Bcer	FALSE	NA	NA	NA
Bc_ctg1_403	B1	1.488529196	Bcer	S	Bcer	FALSE	NA	NA	NA
Bc_ctg1_4030	B1	425.6201149	Bcer	N	Bcer	FALSE	NA	NA	NA
Bc_ctg1_4031	B1	82.66298804	Bcer	S	Bcer	FALSE	NA	NA	NA
Bc_ctg1_4032	B1	182.2952089	Bcer	NA	Bcer	FALSE	NA	NA	NA
Bc_ctg1_4033	B1	627.3654387	Bcer	T	Bcer	FALSE	NA	NA	NA
Bc_ctg1_4034	B1	46.04516981	Bcer	K	Bcer	FALSE	NA	NA	NA
Bc_ctg1_4035	B1	39.29717079	Bcer	N	Bcer	FALSE	NA	NA	NA
Bc_ctg1_4036	B1	14834.4835	Bcer	N	Bcer	FALSE	NA	NA	NA
Bc_ctg1_4037	B1	67.57922552	Bcer	M	Bcer	FALSE	NA	NA	NA
Bc_ctg1_4038	B1	3.572470071	Bcer	N	Bcer	FALSE	NA	NA	NA
Bc_ctg1_4039	B1	118.2884535	Bcer	N	Bcer	FALSE	NA	NA	NA
Bc_ctg1_404	B1	1.984705595	Bcer	C	Bcer	FALSE	NA	NA	NA
Bc_ctg1_4040	B1	52.59469827	Bcer	N	Bcer	FALSE	NA	NA	NA
Bc_ctg1_4041	B1	21.83176155	Bcer	N	Bcer	FALSE	NA	NA	NA
Bc_ctg1_4042	B1	56.66334474	Bcer	N	Bcer	FALSE	NA	NA	NA
Bc_ctg1_4043	B1	7.938822381	Bcer	N	Bcer	FALSE	NA	NA	NA
Bc_ctg1_4044	B1	52.59469827	Bcer	N	Bcer	FALSE	NA	NA	NA
Bc_ctg1_4045	B1	104.3955143	Bcer	N	Bcer	FALSE	NA	NA	NA
Bc_ctg1_4046	B1	164.6313291	Bcer	N	Bcer	FALSE	NA	NA	NA
Bc_ctg1_4047	B1	250.3706108	Bcer	N	Bcer	FALSE	NA	NA	NA
Bc_ctg1_4048	B1	206.6078525	Bcer	N	Bcer	FALSE	NA	NA	NA
Bc_ctg1_4049	B1	65.99146104	Bcer	C	Bcer	FALSE	NA	NA	NA
Bc_ctg1_405	B1	7.839587101	Bcer	S	Bcer	FALSE	NA	NA	NA
Bc_ctg1_4050	B1	3.076293673	Bcer	S	Bcer	FALSE	NA	NA	NA
Bc_ctg1_4051	B1	12.60288053	Bcer	K	Bcer	FALSE	NA	NA	NA
Bc_ctg1_4052	B1	8.23652822	Bcer	E	Bcer	FALSE	NA	NA	NA
Bc_ctg1_4053	B1	3.373999512	Bcer	S	Bcer	FALSE	NA	NA	NA
Bc_ctg1_4054	B1	1.290058637	Bcer	NA	Bcer	FALSE	NA	NA	NA
Bc_ctg1_4055	B1	1.786235036	Bcer	NA	Bcer	FALSE	NA	NA	NA
Bc_ctg1_4056	B1	3.870175911	Bcer	Q	Bcer	TRUE	NA	NA	NA
Bc_ctg1_4057	B1	0.496176399	Bcer	S	Bcer	FALSE	NA	NA	NA
Bc_ctg1_4058	B1	0.694646958	Bcer	S	Bcer	FALSE	NA	NA	NA
Bc_ctg1_4059	B1	17.46540924	Bcer	K	Bcer	FALSE	NA	NA	NA
Bc_ctg1_406	B1	26.9919961	Bcer	J	Bcer	FALSE	NA	NA	NA
Bc_ctg1_4060	B1	0.496176399	Bcer	EGP	Bcer	FALSE	NA	NA	NA
Bc_ctg1_4061	B1	0.396941119	Bcer	S	Bcer	FALSE	NA	NA	NA
Bc_ctg1_4062	B1	1.091588077	Bcer	K	Bcer	FALSE	NA	NA	NA
Bc_ctg1_4063	B1	0	Bcer	P	Bcer	FALSE	NA	NA	NA
Bc_ctg1_4064	B1	0	Bcer	P	Bcer	FALSE	NA	NA	NA
Bc_ctg1_4065	B1	0.496176399	Bcer	P	Bcer	FALSE	NA	NA	NA
Bc_ctg1_4066	B1	0.694646958	Bcer	J	Bcer	FALSE	NA	NA	NA
Bc_ctg1_4067	B1	0.893117518	Bcer	NA	Bcer	FALSE	NA	NA	NA
Bc_ctg1_4068	B1	163.4405058	Bcer	V	Bcer	FALSE	NA	NA	NA
Bc_ctg1_4069	B1	60.73199121	Bcer	S	Bcer	FALSE	NA	NA	NA
Bc_ctg1_407	B1	0	Bcer	NA	Bcer	FALSE	NA	NA	NA
Bc_ctg1_4070	B1	11.41205717	Bcer	NA	Bcer	FALSE	NA	NA	NA
Bc_ctg1_4071	B1	1.290058637	Bcer	V	Bcer	FALSE	NA	NA	NA
Bc_ctg1_4072	B1	0.09923528	Bcer	G	Bcer	FALSE	NA	NA	NA
Bc_ctg1_4073	B1	1571.688361	Bcer	NA	Bcer	FALSE	NA	NA	NA
Bc_ctg1_4074	B1	0.396941119	Bcer	S	Bcer	FALSE	NA	NA	NA
Bc_ctg1_4075	B1	0.496176399	Bcer	NA	Bcer	FALSE	NA	NA	NA

Bc_ctg1_4076	B1	1.091588077	Bcer	K	Bcer	FALSE	NA	NA	NA
Bc_ctg1_4077	B1	20.83940875	Bcer	T	Bcer	FALSE	NA	NA	NA
Bc_ctg1_4078	B1	24.61034938	Bcer	E	Bcer	FALSE	NA	NA	NA
Bc_ctg1_4079	B1	0	Bcer	K	Bcer	FALSE	NA	NA	NA
Bc_ctg1_408	B1	0.396941119	Bcer	I	Bcer	FALSE	NA	NA	NA
Bc_ctg1_4080	B1	0.893117518	Bcer	S	Bcer	FALSE	NA	NA	NA
Bc_ctg1_4081	B1	0.19847056	Bcer	S	Bcer	FALSE	NA	NA	NA
Bc_ctg1_4082	B1	68.07540192	Bcer	P	Bcer	FALSE	NA	NA	NA
Bc_ctg1_4083	B1	2.381646714	Bcer	S	Bcer	FALSE	NA	NA	NA
Bc_ctg1_4084	B1	3.076293673	Bcer	K	Bcer	FALSE	NA	NA	NA
Bc_ctg1_4085	B1	4.961763988	Bcer	EG	Bcer	FALSE	NA	NA	NA
Bc_ctg1_4086	B1	0.19847056	Bcer	NA	Bcer	FALSE	NA	NA	NA
Bc_ctg1_4087	B1	1.587764476	Bcer	S	Bcer	FALSE	NA	NA	NA
Bc_ctg1_4088	B1	0	Bcer	S	Bcer	FALSE	NA	NA	NA
Bc_ctg1_4089	B1	0.694646958	Bcer	S	Bcer	FALSE	NA	NA	NA
Bc_ctg1_409	B1	0.496176399	Bcer	NA	Bcer	FALSE	NA	NA	NA
Bc_ctg1_4090	B1	0.396941119	Bcer	M	Bcer	FALSE	NA	NA	NA
Bc_ctg1_4091	B1	73.23563646	Bcer	C	Bcer	FALSE	NA	NA	NA
Bc_ctg1_4092	B1	3.96941119	Bcer	K	Bcer	FALSE	NA	NA	NA
Bc_ctg1_4093	B1	0.694646958	Bcer	IQ	Bcer	TRUE	NA	NA	NA
Bc_ctg1_4094	B1	643.8384951	Bcer	C	Bcer	FALSE	NA	NA	NA
Bc_ctg1_4095	B1	24.70958466	Bcer	S	Bcer	FALSE	NA	NA	NA
Bc_ctg1_4096	B1	11.31282189	Bcer	K	Bcer	FALSE	NA	NA	NA
Bc_ctg1_4097	B1	20.93864403	Bcer	V	Bcer	FALSE	NA	NA	NA
Bc_ctg1_4098	B1	26.29734914	Bcer	E	Bcer	FALSE	NA	NA	NA
Bc_ctg1_4099	B1	1.885470315	Bcer	NA	Bcer	FALSE	NA	NA	NA
Bc_ctg1_41	B1	0	Bcer	L	Bcer	FALSE	NA	NA	NA
Bc_ctg1_410	B1	7.045704863	Bcer	P	Bcer	FALSE	NA	NA	NA
Bc_ctg1_4100	B1	11.61052773	Bcer	K	Bcer	FALSE	NA	NA	NA
Bc_ctg1_4101	B1	10.71741021	Bcer	O	Bcer	FALSE	NA	NA	NA
Bc_ctg1_4102	B1	0.893117518	Bcer	O	Bcer	FALSE	NA	NA	NA
Bc_ctg1_4103	B1	0.992352798	Bcer	CO	Bcer	FALSE	NA	NA	NA
Bc_ctg1_4104	B1	3.870175911	Bcer	M	Bcer	FALSE	NA	NA	NA
Bc_ctg1_4105	B1	0	Bcer	NA	Bcer	FALSE	NA	NA	NA
Bc_ctg1_4106	B1	41.6788175	Bcer	K	Bcer	FALSE	NA	NA	NA
Bc_ctg1_4107	B1	10.91588077	Bcer	K	Bcer	FALSE	NA	NA	NA
Bc_ctg1_4108	B1	10.22123382	Bcer	E	Bcer	FALSE	NA	NA	NA
Bc_ctg1_4109	B1	64.20522601	Bcer	S	Bcer	FALSE	NA	NA	NA
Bc_ctg1_411	B1	4.366352309	Bcer	S	Bcer	FALSE	NA	NA	NA
Bc_ctg1_4110	B1	7.938822381	Bcer	NA	Bcer	FALSE	NA	NA	NA
Bc_ctg1_4111	B1	5.060999268	Bcer	NA	Bcer	FALSE	NA	NA	NA
Bc_ctg1_4112	B1	1.290058637	Bcer	NA	Bcer	FALSE	NA	NA	NA
Bc_ctg1_4113	B1	1.587764476	Bcer	K	Bcer	FALSE	NA	NA	NA
Bc_ctg1_4114	B1	3.870175911	Bcer	K	Bcer	FALSE	NA	NA	NA
Bc_ctg1_4115	B1	7.144940143	Bcer	T	Bcer	FALSE	NA	NA	NA
Bc_ctg1_4116	B1	45.15205229	Bcer	V	Bcer	FALSE	NA	NA	NA
Bc_ctg1_4117	B1	48.42681652	Bcer	V	Bcer	FALSE	NA	NA	NA
Bc_ctg1_4118	B1	89.60945762	Bcer	V	Bcer	FALSE	NA	NA	NA
Bc_ctg1_4119	B1	0	Bcer	S	Bcer	FALSE	NA	NA	NA
Bc_ctg1_412	B1	0.09923528	Bcer	NA	Bcer	FALSE	NA	NA	NA
Bc_ctg1_4120	B1	2.580117274	Bcer	I	Bcer	FALSE	NA	NA	NA
Bc_ctg1_4121	B1	25.60270218	Bcer	F	Bcer	FALSE	NA	NA	NA
Bc_ctg1_4122	B1	210.2795578	Bcer	C	Bcer	FALSE	NA	NA	NA
Bc_ctg1_4123	B1	265.3551381	Bcer	E	Bcer	FALSE	NA	NA	NA
Bc_ctg1_4124	B1	266.049785	Bcer	C	Bcer	FALSE	NA	NA	NA
Bc_ctg1_4125	B1	18.85470315	Bcer	T	Bcer	FALSE	NA	NA	NA
Bc_ctg1_4126	B1	12.70211581	Bcer	T	Bcer	FALSE	NA	NA	NA
Bc_ctg1_4127	B1	68.07540192	Bcer	L	Bcer	FALSE	NA	NA	NA
Bc_ctg1_4128	B1	0.09923528	Bcer	NA	Bcer	FALSE	NA	NA	NA
Bc_ctg1_413	B1	1.389293917	Bcer	U	Bcer	FALSE	NA	NA	NA
Bc_ctg1_4130	B1	76.70887126	Bcer	F	Bcer	FALSE	NA	NA	NA
Bc_ctg1_4131	B1	0.694646958	Bcer	K	Bcer	FALSE	NA	NA	NA

Bc_ctg1_4132	B1	3.870175911	Bcer	E	Bcer	FALSE	NA	NA	NA
Bc_ctg1_4133	B1	11.41205717	Bcer	E	Bcer	FALSE	NA	NA	NA
Bc_ctg1_4134	B1	9.625822137	Bcer	S	Bcer	FALSE	NA	NA	NA
Bc_ctg1_4135	B1	3.076293673	Bcer	S	Bcer	FALSE	Bc_Ga0417192_01_rn29	Other	NA
Bc_ctg1_4136	B1	15.38146836	Bcer	S	Bcer	FALSE	Bc_Ga0417192_01_rn29	Other	NA
Bc_ctg1_4137	B1	0	Bcer	NA	Bcer	FALSE	Bc_Ga0417192_01_rn29	Other	NA
Bc_ctg1_4138	B1	29.86981921	Bcer	S	Bcer	FALSE	Bc_Ga0417192_01_rn29	Other	NA
Bc_ctg1_4139	B1	22.03023211	Bcer	K	Bcer	FALSE	Bc_Ga0417192_01_rn29	Regulation	SMCOG1278
Bc_ctg1_414	B1	16.47305644	Bcer	S	Bcer	FALSE	NA	NA	NA
Bc_ctg1_4140	B1	134.4638041	Bcer	EGP	Bcer	FALSE	Bc_Ga0417192_01_rn29	Transport	SMCOG1005
Bc_ctg1_4141	B1	8.931175179	Bcer	S	Bcer	FALSE	Bc_Ga0417192_01_rn29	Other	NA
Bc_ctg1_4142	B1	9.824292696	Bcer	EGP	Bcer	FALSE	Bc_Ga0417192_01_rn29	Transport	SMCOG1202
Bc_ctg1_4143	B1	4.366352309	Bcer	I	Bcer	FALSE	Bc_Ga0417192_01_rn29	Core biosynthesis	SMCOG1084
Bc_ctg1_4144	B1	52.19775715	Bcer	S	Bcer	FALSE	Bc_Ga0417192_01_rn29	Other	NA
Bc_ctg1_4145	B1	6.351057905	Bcer	P	Bcer	FALSE	Bc_Ga0417192_01_rn29	Transport	SMCOG1106
Bc_ctg1_4146	B1	53.19010995	Bcer	E	Bcer	FALSE	Bc_Ga0417192_01_rn29	Ancillary biosynthesis	SMCOG1081
Bc_ctg1_4147	B1	6.351057905	Bcer	S	Bcer	FALSE	Bc_Ga0417192_01_rn29	Other	NA
Bc_ctg1_4148	B1	16.671527	Bcer	S	Bcer	FALSE	Bc_Ga0417192_01_rn29	Other	NA
Bc_ctg1_4149	B1	11.31282189	Bcer	NA	Bcer	FALSE	Bc_Ga0417192_01_rn29	Other	NA
Bc_ctg1_415	B1	33.44228928	Bcer	S	Bcer	FALSE	NA	NA	NA
Bc_ctg1_4150	B1	5.358705107	Bcer	S	Bcer	FALSE	Bc_Ga0417192_01_rn29	Other	NA
Bc_ctg1_4151	B1	7.045704863	Bcer	G	Bcer	FALSE	Bc_Ga0417192_01_rn29	Ancillary biosynthesis	SMCOG1235
Bc_ctg1_4152	B1	8.831939899	Bcer	S	Bcer	FALSE	Bc_Ga0417192_01_rn29	Other	NA
Bc_ctg1_4153	B1	3.076293673	Bcer	S	Bcer	FALSE	NA	NA	NA
Bc_ctg1_4154	B1	77.00657709	Bcer	S	Bcer	FALSE	NA	NA	NA
Bc_ctg1_4155	B1	0.396941119	Bcer	DZ	Bcer	FALSE	NA	NA	NA
Bc_ctg1_4156	B1	0.297705839	Bcer	NA	Bcer	FALSE	NA	NA	NA
Bc_ctg1_4158	B1	31.8545248	Bcer	O	Bcer	FALSE	NA	NA	NA
Bc_ctg1_4159	B1	0.19847056	Bcer	IQ	Bcer	TRUE	NA	NA	NA
Bc_ctg1_416	B1	0.694646958	Bcer	P	Bcer	FALSE	Bc_Ga0417192_01_rn05	Other	NA
Bc_ctg1_4160	B1	0.396941119	Bcer	E	Bcer	FALSE	NA	NA	NA
Bc_ctg1_4161	B1	0.793882238	Bcer	E	Bcer	FALSE	NA	NA	NA
Bc_ctg1_4162	B1	0	Bcer	E	Bcer	FALSE	NA	NA	NA
Bc_ctg1_4163	B1	0.19847056	Bcer	EH	Bcer	FALSE	NA	NA	NA
Bc_ctg1_4164	B1	3.870175911	Bcer	E	Bcer	FALSE	NA	NA	NA
Bc_ctg1_4165	B1	6.946469583	Bcer	E	Bcer	FALSE	NA	NA	NA
Bc_ctg1_4166	B1	0.09923528	Bcer	NA	Bcer	FALSE	NA	NA	NA
Bc_ctg1_4167	B1	2.877823113	Bcer	M	Bcer	FALSE	NA	NA	NA
Bc_ctg1_4168	B1	0.09923528	Bcer	S	Bcer	FALSE	NA	NA	NA
Bc_ctg1_4169	B1	0.694646958	Bcer	K	Bcer	FALSE	NA	NA	NA
Bc_ctg1_417	B1	0.396941119	Bcer	E	Bcer	FALSE	Bc_Ga0417192_01_rn05	Other	NA
Bc_ctg1_4170	B1	12.10670413	Bcer	EGP	Bcer	FALSE	NA	NA	NA
Bc_ctg1_4171	B1	13.49599805	Bcer	S	Bcer	FALSE	NA	NA	NA
Bc_ctg1_4172	B1	63.01440265	Bcer	I	Bcer	FALSE	NA	NA	NA
Bc_ctg1_4173	B1	36.41934767	Bcer	K	Bcer	FALSE	NA	NA	NA
Bc_ctg1_4174	B1	240.2486123	Bcer	C	Bcer	FALSE	NA	NA	NA
Bc_ctg1_4175	B1	2.183176155	Bcer	K	Bcer	FALSE	NA	NA	NA
Bc_ctg1_4176	B1	0.595411679	Bcer	S	Bcer	FALSE	NA	NA	NA
Bc_ctg1_4177	B1	57.45722698	Bcer	F	Bcer	FALSE	NA	NA	NA
Bc_ctg1_4178	B1	2.480881994	Bcer	EGP	Bcer	FALSE	NA	NA	NA
Bc_ctg1_4179	B1	8.23652822	Bcer	K	Bcer	FALSE	NA	NA	NA
Bc_ctg1_418	B1	3.473234792	Bcer	S	Bcer	FALSE	Bc_Ga0417192_01_rn05	Other	NA
Bc_ctg1_4180	B1	58.25110922	Bcer	E	Bcer	FALSE	NA	NA	NA
Bc_ctg1_4181	B1	4.664058149	Bcer	J	Bcer	FALSE	NA	NA	NA
Bc_ctg1_4182	B1	2.977058393	Bcer	T	Bcer	FALSE	NA	NA	NA
Bc_ctg1_4183	B1	1.488529196	Bcer	T	Bcer	FALSE	NA	NA	NA
Bc_ctg1_4184	B1	2.580117274	Bcer	M	Bcer	FALSE	NA	NA	NA
Bc_ctg1_4185	B1	150.5399194	Bcer	P	Bcer	FALSE	NA	NA	NA
Bc_ctg1_4186	B1	58.25110922	Bcer	S	Bcer	FALSE	NA	NA	NA
Bc_ctg1_4187	B1	0.496176399	Bcer	P	Bcer	FALSE	NA	NA	NA
Bc_ctg1_4188	B1	154.1123895	Bcer	H	Bcer	FALSE	NA	NA	NA
Bc_ctg1_419	B1	0.992352798	Bcer	S	Bcer	FALSE	Bc_Ga0417192_01_rn05	Other	NA

Bc_ctg1_4190	B1	115.5098656	Bcer	E	Bcer	FALSE	NA	NA	NA
Bc_ctg1_4191	B1	1848.15785	Bcer	O	Bcer	FALSE	NA	NA	NA
Bc_ctg1_4192	B1	2135.741691	Bcer	E	Bcer	FALSE	NA	NA	NA
Bc_ctg1_4193	B1	443.38323	Bcer	E	Bcer	FALSE	NA	NA	NA
Bc_ctg1_4194	B1	16.86999756	Bcer	O	Bcer	FALSE	NA	NA	NA
Bc_ctg1_4195	B1	5.854881506	Bcer	E	Bcer	FALSE	NA	NA	NA
Bc_ctg1_4196	B1	6.450293184	Bcer	S	Bcer	FALSE	NA	NA	NA
Bc_ctg1_4197	B1	3.572470071	Bcer	K	Bcer	FALSE	NA	NA	NA
Bc_ctg1_4198	B1	6.747999024	Bcer	K	Bcer	FALSE	NA	NA	NA
Bc_ctg1_4199	B1	0	Bcer	S	Bcer	FALSE	NA	NA	NA
Bc_ctg1_42	B1	16.37382116	Bcer	S	Bcer	FALSE	NA	NA	NA
Bc_ctg1_420	B1	0	Bcer	JM	Bcer	FALSE	Bc_Ga0417192_01_rn05	Ancillary biosynthesis	SMCOG1064
Bc_ctg1_4200	B1	0	Bcer	S	Bcer	FALSE	NA	NA	NA
Bc_ctg1_4201	B1	15.08376252	Bcer	K	Bcer	FALSE	NA	NA	NA
Bc_ctg1_4202	B1	35.62546543	Bcer	K	Bcer	FALSE	NA	NA	NA
Bc_ctg1_4203	B1	85.83851699	Bcer	F	Bcer	FALSE	NA	NA	NA
Bc_ctg1_4204	B1	61.82357929	Bcer	F	Bcer	FALSE	NA	NA	NA
Bc_ctg1_4205	B1	54.77787443	Bcer	F	Bcer	FALSE	NA	NA	NA
Bc_ctg1_4206	B1	10.81664549	Bcer	F	Bcer	FALSE	NA	NA	NA
Bc_ctg1_4207	B1	10.22123382	Bcer	O	Bcer	FALSE	NA	NA	NA
Bc_ctg1_4208	B1	9.328116298	Bcer	S	Bcer	FALSE	NA	NA	NA
Bc_ctg1_4209	B1	7.641116542	Bcer	NA	Bcer	FALSE	NA	NA	NA
Bc_ctg1_421	B1	0.595411679	Bcer	M	Bcer	FALSE	Bc_Ga0417192_01_rn05	Other	NA
Bc_ctg1_4210	B1	310.2094845	Bcer	O	Bcer	FALSE	NA	NA	NA
Bc_ctg1_4211	B1	317.0567188	Bcer	S	Bcer	FALSE	NA	NA	NA
Bc_ctg1_4212	B1	148.6544491	Bcer	E	Bcer	FALSE	NA	NA	NA
Bc_ctg1_4213	B1	424.0323504	Bcer	Q	Bcer	TRUE	NA	NA	NA
Bc_ctg1_4214	B1	0.19847056	Bcer	O	Bcer	FALSE	NA	NA	NA
Bc_ctg1_4215	B1	17.46540924	Bcer	MT	Bcer	FALSE	NA	NA	NA
Bc_ctg1_4216	B1	0	Bcer	NA	Bcer	FALSE	NA	NA	NA
Bc_ctg1_4217	B1	38.90022967	Bcer	L	Bcer	FALSE	NA	NA	NA
Bc_ctg1_4218	B1	3.274764232	Bcer	S	Bcer	FALSE	NA	NA	NA
Bc_ctg1_4219	B1	26.69429026	Bcer	P	Bcer	FALSE	NA	NA	NA
Bc_ctg1_422	B1	0	Bcer	GM	Bcer	FALSE	Bc_Ga0417192_01_rn05	Core biosynthesis	SMCOG1010
Bc_ctg1_4220	B1	8.534234059	Bcer	I	Bcer	FALSE	NA	NA	NA
Bc_ctg1_4221	B1	282.2251356	Bcer	E	Bcer	FALSE	NA	NA	NA
Bc_ctg1_4222	B1	6.351057905	Bcer	E	Bcer	FALSE	NA	NA	NA
Bc_ctg1_4223	B1	4.763293429	Bcer	P	Bcer	FALSE	NA	NA	NA
Bc_ctg1_4224	B1	0.992352798	Bcer	S	Bcer	FALSE	NA	NA	NA
Bc_ctg1_4225	B1	9.625822137	Bcer	EG	Bcer	FALSE	NA	NA	NA
Bc_ctg1_4226	B1	6.450293184	Bcer	K	Bcer	FALSE	NA	NA	NA
Bc_ctg1_4227	B1	2.679352554	Bcer	K	Bcer	FALSE	NA	NA	NA
Bc_ctg1_4228	B1	1.786235036	Bcer	Q	Bcer	TRUE	NA	NA	NA
Bc_ctg1_4229	B1	4.664058149	Bcer	S	Bcer	FALSE	NA	NA	NA
Bc_ctg1_423	B1	0.992352798	Bcer	GM	Bcer	FALSE	Bc_Ga0417192_01_rn05	Core biosynthesis	SMCOG1010
Bc_ctg1_4231	B1	1.984705595	Bcer	J	Bcer	FALSE	NA	NA	NA
Bc_ctg1_4232	B1	9.129645738	Bcer	S	Bcer	FALSE	NA	NA	NA
Bc_ctg1_4233	B1	38.90022967	Bcer	C	Bcer	FALSE	NA	NA	NA
Bc_ctg1_4234	B1	0.496176399	Bcer	E	Bcer	FALSE	NA	NA	NA
Bc_ctg1_4235	B1	17.56464452	Bcer	S	Bcer	FALSE	NA	NA	NA
Bc_ctg1_4236	B1	4.465587589	Bcer	E	Bcer	FALSE	NA	NA	NA
Bc_ctg1_4237	B1	1.686999756	Bcer	E	Bcer	FALSE	NA	NA	NA
Bc_ctg1_4238	B1	0.19847056	Bcer	E	Bcer	FALSE	NA	NA	NA
Bc_ctg1_4239	B1	1.290058637	Bcer	E	Bcer	FALSE	NA	NA	NA
Bc_ctg1_424	B1	3.274764232	Bcer	K	Bcer	FALSE	Bc_Ga0417192_01_rn05	Other	NA
Bc_ctg1_4240	B1	3.572470071	Bcer	E	Bcer	FALSE	NA	NA	NA
Bc_ctg1_4241	B1	11.01511605	Bcer	K	Bcer	FALSE	NA	NA	NA
Bc_ctg1_4242	B1	1.091588077	Bcer	S	Bcer	FALSE	NA	NA	NA
Bc_ctg1_4243	B1	0.396941119	Bcer	S	Bcer	FALSE	Bc_Ga0417192_01_rn30	Other	NA
Bc_ctg1_4244	B1	0	Bcer	S	Bcer	FALSE	Bc_Ga0417192_01_rn30	Other	NA
Bc_ctg1_4245	B1	76.90734182	Bcer	K	Bcer	FALSE	Bc_Ga0417192_01_rn30	Regulation	SMCOG1135
Bc_ctg1_4246	B1	36.41934767	Bcer	C	Bcer	FALSE	Bc_Ga0417192_01_rn30	Other	SMCOG1251

Bc_ctg1_4247	B1	24.70958466	Bcer	C	Bcer	FALSE	Bc_Ga0417192_01_rn30	Other	NA
Bc_ctg1_4248	B1	17.6638798	Bcer	C	Bcer	FALSE	Bc_Ga0417192_01_rn30	Other	NA
Bc_ctg1_4249	B1	18.06082092	Bcer	V	Bcer	FALSE	Bc_Ga0417192_01_rn30	Transport	SMCOG1288
Bc_ctg1_425	B1	0.09923528	Bcer	NA	Bcer	FALSE	Bc_Ga0417192_01_rn05	Other	NA
Bc_ctg1_4250	B1	21.43482043	Bcer	V	Bcer	FALSE	Bc_Ga0417192_01_rn30	Transport	SMCOG1288
Bc_ctg1_4251	B1	0.19847056	Bcer	E	Bcer	FALSE	Bc_Ga0417192_01_rn30	Other	NA
Bc_ctg1_4252	B1	4.26711703	Bcer	MQ	Bcer	TRUE	Bc_Ga0417192_01_rn30	Core biosynthesis	None
Bc_ctg1_4253	B1	2.877823113	Bcer	E	Bcer	FALSE	Bc_Ga0417192_01_rn30	Core biosynthesis	SMCOG1056
Bc_ctg1_4254	B1	1.389293917	Bcer	S	Bcer	FALSE	Bc_Ga0417192_01_rn30	Ancillary biosynthesis	SMCOG1156
Bc_ctg1_4256	B1	3.671705351	Bcer	NA	Bcer	FALSE	Bc_Ga0417192_01_rn30	Other	NA
Bc_ctg1_4257	B1	38.00711215	Bcer	P	Bcer	FALSE	Bc_Ga0417192_01_rn30	Other	NA
Bc_ctg1_4258	B1	57.15952114	Bcer	EG	Bcer	FALSE	Bc_Ga0417192_01_rn30	Other	NA
Bc_ctg1_4259	B1	30.76293673	Bcer	V	Bcer	FALSE	Bc_Ga0417192_01_rn30	Other	NA
Bc_ctg1_426	B1	46.93828733	Bcer	NT	Bcer	FALSE	Bc_Ga0417192_01_rn05	Other	SMCOG1061
Bc_ctg1_4260	B1	30.46523089	Bcer	M	Bcer	FALSE	Bc_Ga0417192_01_rn30	Other	NA
Bc_ctg1_4261	B1	652.5711997	Bcer	S	Bcer	FALSE	Bc_Ga0417192_01_rn30	Ancillary biosynthesis	None
Bc_ctg1_4262	B1	0.595411679	Bcer	NA	Bcer	FALSE	Bc_Ga0417192_01_rn30	Other	NA
Bc_ctg1_4263	B1	2180.496802	Bcer	M	Bcer	FALSE	Bc_Ga0417192_01_rn30	Other	NA
Bc_ctg1_4264	B1	17.26693868	Bcer	S	Bcer	FALSE	Bc_Ga0417192_01_rn30	Ancillary biosynthesis	SMCOG1036
Bc_ctg1_4265	B1	18.95393843	Bcer	V	Bcer	FALSE	NA	NA	NA
Bc_ctg1_4266	B1	11.31282189	Bcer	T	Bcer	FALSE	NA	NA	NA
Bc_ctg1_4267	B1	11.01511605	Bcer	T	Bcer	FALSE	NA	NA	NA
Bc_ctg1_4268	B1	1.091588077	Bcer	NA	Bcer	FALSE	NA	NA	NA
Bc_ctg1_4269	B1	128.9066284	Bcer	S	Bcer	FALSE	NA	NA	NA
Bc_ctg1_427	B1	0.793882238	Bcer	NA	Bcer	FALSE	Bc_Ga0417192_01_rn05	Other	NA
Bc_ctg1_4270	B1	29.07593697	Bcer	S	Bcer	FALSE	NA	NA	NA
Bc_ctg1_4271	B1	63.60981433	Bcer	G	Bcer	FALSE	NA	NA	NA
Bc_ctg1_4272	B1	0.19847056	Bcer	G	Bcer	FALSE	NA	NA	NA
Bc_ctg1_4273	B1	6.053352065	Bcer	S	Bcer	FALSE	NA	NA	NA
Bc_ctg1_4274	B1	47.53369901	Bcer	I	Bcer	FALSE	NA	NA	NA
Bc_ctg1_4275	B1	3.076293673	Bcer	S	Bcer	FALSE	NA	NA	NA
Bc_ctg1_4276	B1	60.13657954	Bcer	E	Bcer	FALSE	NA	NA	NA
Bc_ctg1_4277	B1	80.18210605	Bcer	E	Bcer	FALSE	NA	NA	NA
Bc_ctg1_4278	B1	81.96834108	Bcer	E	Bcer	FALSE	NA	NA	NA
Bc_ctg1_4279	B1	0.09923528	Bcer	S	Bcer	FALSE	NA	NA	NA
Bc_ctg1_428	B1	1.091588077	Bcer	NA	Bcer	FALSE	Bc_Ga0417192_01_rn05	Other	NA
Bc_ctg1_4280	B1	1.488529196	Bcer	S	Bcer	FALSE	NA	NA	NA
Bc_ctg1_4281	B1	157.2879184	Bcer	K	Bcer	FALSE	NA	NA	NA
Bc_ctg1_4282	B1	9.328116298	Bcer	S	Bcer	FALSE	NA	NA	NA
Bc_ctg1_4283	B1	4.664058149	Bcer	T	Bcer	FALSE	Bc_Ga0417192_01_rn31	Other	NA
Bc_ctg1_4284	B1	11.51129245	Bcer	T	Bcer	FALSE	Bc_Ga0417192_01_rn31	Regulation	SMCOG1003
Bc_ctg1_4285	B1	156.791742	Bcer	G	Bcer	FALSE	Bc_Ga0417192_01_rn31	Ancillary biosynthesis	SMCOG1235
Bc_ctg1_4286	B1	135.158451	Bcer	L	Bcer	FALSE	Bc_Ga0417192_01_rn31	Other	NA
Bc_ctg1_4287	B1	17.06846812	Bcer	S	Bcer	FALSE	Bc_Ga0417192_01_rn31	Other	NA
Bc_ctg1_4288	B1	8.931175179	Bcer	M	Bcer	FALSE	Bc_Ga0417192_01_rn31	Other	NA
Bc_ctg1_4289	B1	46.04516981	Bcer	Q	Bcer	TRUE	Bc_Ga0417192_01_rn31	Core biosynthesis	None
Bc_ctg1_429	B1	0	Bcer	NA	Bcer	FALSE	Bc_Ga0417192_01_rn05	Other	NA
Bc_ctg1_4290	B1	41.77805278	Bcer	Q	Bcer	TRUE	Bc_Ga0417192_01_rn31	Core biosynthesis	None
Bc_ctg1_4291	B1	15.08376252	Bcer	IQ	Bcer	TRUE	Bc_Ga0417192_01_rn31	Ancillary biosynthesis	SMCOG1002
Bc_ctg1_4292	B1	0.992352798	Bcer	IQ	Bcer	TRUE	Bc_Ga0417192_01_rn31	Other	NA
Bc_ctg1_4293	B1	21.83176155	Bcer	IQ	Bcer	TRUE	Bc_Ga0417192_01_rn31	Other	NA
Bc_ctg1_4294	B1	13.89293917	Bcer	G	Bcer	FALSE	Bc_Ga0417192_01_rn31	Other	NA
Bc_ctg1_4295	B1	0	Bcer	S	Bcer	FALSE	Bc_Ga0417192_01_rn31	Other	NA
Bc_ctg1_4296	B1	0.297705839	Bcer	EGP	Bcer	FALSE	Bc_Ga0417192_01_rn31	Other	NA
Bc_ctg1_4297	B1	3.96941119	Bcer	S	Bcer	FALSE	NA	NA	NA
Bc_ctg1_4298	B1	52.89240411	Bcer	K	Bcer	FALSE	NA	NA	NA
Bc_ctg1_4299	B1	0.297705839	Bcer	S	Bcer	FALSE	NA	NA	NA
Bc_ctg1_43	B1	8.633469339	Bcer	K	Bcer	FALSE	NA	NA	NA
Bc_ctg1_430	B1	0.19847056	Bcer	Q	Bcer	TRUE	Bc_Ga0417192_01_rn05	Other	NA
Bc_ctg1_4300	B1	0.893117518	Bcer	S	Bcer	FALSE	NA	NA	NA
Bc_ctg1_4302	B1	44.15969949	Bcer	S	Bcer	FALSE	NA	NA	NA
Bc_ctg1_4303	B1	326.3848351	Bcer	D	Bcer	FALSE	NA	NA	NA

Bc_ctg1_4304	B1	5.259469827	Bcer	S	Bcer	FALSE	NA	NA	NA
Bc_ctg1_4305	B1	21.83176155	Bcer	F	Bcer	FALSE	NA	NA	NA
Bc_ctg1_4306	B1	97.15133889	Bcer	H	Bcer	FALSE	NA	NA	NA
Bc_ctg1_4307	B1	13.99217445	Bcer	V	Bcer	FALSE	NA	NA	NA
Bc_ctg1_4308	B1	25.5034669	Bcer	K	Bcer	FALSE	NA	NA	NA
Bc_ctg1_4309	B1	30.06828977	Bcer	O	Bcer	FALSE	NA	NA	NA
Bc_ctg1_431	B1	4.26711703	Bcer	S	Bcer	FALSE	NA	NA	NA
Bc_ctg1_4310	B1	8.038057661	Bcer	K	Bcer	FALSE	NA	NA	NA
Bc_ctg1_4311	B1	1.389293917	Bcer	C	Bcer	FALSE	NA	NA	NA
Bc_ctg1_4312	B1	1.786235036	Bcer	EGP	Bcer	FALSE	NA	NA	NA
Bc_ctg1_4313	B1	28.97670169	Bcer	NA	Bcer	FALSE	NA	NA	NA
Bc_ctg1_4314	B1	27.48817249	Bcer	S	Bcer	FALSE	NA	NA	NA
Bc_ctg1_4315	B1	3.96941119	Bcer	S	Bcer	FALSE	NA	NA	NA
Bc_ctg1_4316	B1	53.68628635	Bcer	S	Bcer	FALSE	NA	NA	NA
Bc_ctg1_4317	B1	43.36581726	Bcer	NT	Bcer	FALSE	NA	NA	NA
Bc_ctg1_4318	B1	24.90805522	Bcer	NA	Bcer	FALSE	NA	NA	NA
Bc_ctg1_4319	B1	11.80899829	Bcer	S	Bcer	FALSE	NA	NA	NA
Bc_ctg1_432	B1	2.977058393	Bcer	O	Bcer	FALSE	NA	NA	NA
Bc_ctg1_4320	B1	0.09923528	Bcer	S	Bcer	FALSE	NA	NA	NA
Bc_ctg1_4321	B1	202.2415002	Bcer	P	Bcer	FALSE	NA	NA	NA
Bc_ctg1_4322	B1	268.530667	Bcer	S	Bcer	FALSE	NA	NA	NA
Bc_ctg1_4323	B1	1.686999756	Bcer	S	Bcer	FALSE	NA	NA	NA
Bc_ctg1_4326	B1	39.59487662	Bcer	C	Bcer	FALSE	NA	NA	NA
Bc_ctg1_4328	B1	2.877823113	Bcer	E	Bcer	FALSE	NA	NA	NA
Bc_ctg1_4329	B1	12.70211581	Bcer	J	Bcer	FALSE	NA	NA	NA
Bc_ctg1_433	B1	10.61817493	Bcer	G	Bcer	FALSE	NA	NA	NA
Bc_ctg1_4330	B1	256.6224335	Bcer	S	Bcer	FALSE	NA	NA	NA
Bc_ctg1_4331	B1	44.55664061	Bcer	S	Bcer	FALSE	NA	NA	NA
Bc_ctg1_4332	B1	4.961763988	Bcer	K	Bcer	FALSE	NA	NA	NA
Bc_ctg1_4333	B1	66.58687272	Bcer	P	Bcer	FALSE	NA	NA	NA
Bc_ctg1_4335	B1	42.37346446	Bcer	GM	Bcer	FALSE	NA	NA	NA
Bc_ctg1_4336	B1	13.69446861	Bcer	C	Bcer	FALSE	NA	NA	NA
Bc_ctg1_4337	B1	4.465587589	Bcer	L	Bcer	FALSE	NA	NA	NA
Bc_ctg1_4338	B1	29.27440753	Bcer	NU	Bcer	FALSE	NA	NA	NA
Bc_ctg1_4339	B1	3.870175911	Bcer	E	Bcer	FALSE	NA	NA	NA
Bc_ctg1_4340	B1	12.99982165	Bcer	H	Bcer	FALSE	NA	NA	NA
Bc_ctg1_4341	B1	3.373999512	Bcer	S	Bcer	FALSE	NA	NA	NA
Bc_ctg1_4342	B1	7.144940143	Bcer	G	Bcer	FALSE	NA	NA	NA
Bc_ctg1_4343	B1	0.496176399	Bcer	NA	Bcer	FALSE	NA	NA	NA
Bc_ctg1_4344	B1	7.541881262	Bcer	M	Bcer	FALSE	NA	NA	NA
Bc_ctg1_4345	B1	15.97688004	Bcer	F	Bcer	FALSE	NA	NA	NA
Bc_ctg1_4346	B1	39.6941119	Bcer	S	Bcer	FALSE	NA	NA	NA
Bc_ctg1_4347	B1	6.549528464	Bcer	S	Bcer	FALSE	NA	NA	NA
Bc_ctg1_4348	B1	91.99110434	Bcer	P	Bcer	FALSE	NA	NA	NA
Bc_ctg1_4349	B1	32.94611288	Bcer	S	Bcer	FALSE	NA	NA	NA
Bc_ctg1_435	B1	8.732704619	Bcer	T	Bcer	FALSE	NA	NA	NA
Bc_ctg1_4350	B1	46.34287565	Bcer	S	Bcer	FALSE	NA	NA	NA
Bc_ctg1_4351	B1	3.870175911	Bcer	I	Bcer	FALSE	NA	NA	NA
Bc_ctg1_4352	B1	0.09923528	Bcer	I	Bcer	FALSE	NA	NA	NA
Bc_ctg1_4353	B1	0.09923528	Bcer	M	Bcer	FALSE	NA	NA	NA
Bc_ctg1_4355	B1	2.183176155	Bcer	GM	Bcer	FALSE	NA	NA	NA
Bc_ctg1_4356	B1	10.91588077	Bcer	S	Bcer	FALSE	NA	NA	NA
Bc_ctg1_4357	B1	62.71669681	Bcer	S	Bcer	FALSE	NA	NA	NA
Bc_ctg1_4358	B1	0.19847056	Bcer	NA	Bcer	FALSE	NA	NA	NA
Bc_ctg1_4359	B1	3.671705351	Bcer	K	Bcer	FALSE	NA	NA	NA
Bc_ctg1_436	B1	14.6868214	Bcer	S	Bcer	FALSE	NA	NA	NA
Bc_ctg1_4360	B1	1.190823357	Bcer	S	Bcer	FALSE	NA	NA	NA
Bc_ctg1_4361	B1	365.1858295	Bcer	S	Bcer	FALSE	NA	NA	NA
Bc_ctg1_4362	B1	0	Bcer	L	Bcer	FALSE	NA	NA	NA
Bc_ctg1_4364	B1	82.26604692	Bcer	E	Bcer	FALSE	NA	NA	NA
Bc_ctg1_4365	B1	0	Bcer	NA	Bcer	FALSE	NA	NA	NA
Bc_ctg1_4367	B1	13.09905693	Bcer	J	Bcer	FALSE	NA	NA	NA

Bc_ctg1_4368	B1	6.648763744	Bcer	S	Bcer	FALSE	NA	NA	NA
Bc_ctg1_437	B1	12.00746885	Bcer	K	Bcer	FALSE	NA	NA	NA
Bc_ctg1_4370	B1	10.51893965	Bcer	M	Bcer	FALSE	NA	NA	NA
Bc_ctg1_4371	B1	3.373999512	Bcer	J	Bcer	FALSE	NA	NA	NA
Bc_ctg1_4372	B1	11.21358661	Bcer	M	Bcer	FALSE	NA	NA	NA
Bc_ctg1_4373	B1	3.076293673	Bcer	F	Bcer	FALSE	NA	NA	NA
Bc_ctg1_4374	B1	24.11417298	Bcer	J	Bcer	FALSE	NA	NA	NA
Bc_ctg1_4375	B1	5.060999268	Bcer	G	Bcer	FALSE	NA	NA	NA
Bc_ctg1_4376	B1	128.0135109	Bcer	OU	Bcer	FALSE	NA	NA	NA
Bc_ctg1_4377	B1	375.9032397	Bcer	O	Bcer	FALSE	NA	NA	NA
Bc_ctg1_4378	B1	1.587764476	Bcer	M	Bcer	FALSE	NA	NA	NA
Bc_ctg1_4379	B1	18.6562326	Bcer	G	Bcer	FALSE	NA	NA	NA
Bc_ctg1_438	B1	11.01511605	Bcer	S	Bcer	FALSE	NA	NA	NA
Bc_ctg1_4380	B1	9.725057417	Bcer	S	Bcer	FALSE	NA	NA	NA
Bc_ctg1_4381	B1	9.228881018	Bcer	K	Bcer	FALSE	NA	NA	NA
Bc_ctg1_4382	B1	5.557175667	Bcer	EGP	Bcer	FALSE	NA	NA	NA
Bc_ctg1_4383	B1	0.396941119	Bcer	S	Bcer	FALSE	NA	NA	NA
Bc_ctg1_4384	B1	89.80792818	Bcer	E	Bcer	FALSE	NA	NA	NA
Bc_ctg1_4385	B1	8.633469339	Bcer	S	Bcer	FALSE	NA	NA	NA
Bc_ctg1_4386	B1	15.1829978	Bcer	K	Bcer	FALSE	NA	NA	NA
Bc_ctg1_4387	B1	10.12199854	Bcer	S	Bcer	FALSE	NA	NA	NA
Bc_ctg1_4388	B1	149.8452724	Bcer	CG	Bcer	FALSE	NA	NA	NA
Bc_ctg1_4389	B1	33.24381872	Bcer	S	Bcer	FALSE	NA	NA	NA
Bc_ctg1_439	B1	2.381646714	Bcer	M	Bcer	FALSE	NA	NA	NA
Bc_ctg1_4390	B1	12.50364525	Bcer	K	Bcer	FALSE	NA	NA	NA
Bc_ctg1_4391	B1	2.877823113	Bcer	K	Bcer	FALSE	NA	NA	NA
Bc_ctg1_4392	B1	17.06846812	Bcer	T	Bcer	FALSE	NA	NA	NA
Bc_ctg1_4393	B1	8.038057661	Bcer	S	Bcer	FALSE	NA	NA	NA
Bc_ctg1_4394	B1	12.60288053	Bcer	J	Bcer	FALSE	NA	NA	NA
Bc_ctg1_4395	B1	27.28970193	Bcer	J	Bcer	FALSE	NA	NA	NA
Bc_ctg1_4396	B1	8.831939899	Bcer	M	Bcer	FALSE	NA	NA	NA
Bc_ctg1_4397	B1	4.06864647	Bcer	S	Bcer	FALSE	NA	NA	NA
Bc_ctg1_4398	B1	1.091588077	Bcer	K	Bcer	FALSE	NA	NA	NA
Bc_ctg1_4399	B1	0.19847056	Bcer	NA	Bcer	FALSE	NA	NA	NA
Bc_ctg1_44	B1	42.37346446	Bcer	GT	Bcer	FALSE	NA	NA	NA
Bc_ctg1_4400	B1	1.091588077	Bcer	S	Bcer	FALSE	NA	NA	NA
Bc_ctg1_4401	B1	1.686999756	Bcer	NA	Bcer	FALSE	NA	NA	NA
Bc_ctg1_4402	B1	0	Bcer	NA	Bcer	FALSE	NA	NA	NA
Bc_ctg1_4403	B1	1.587764476	Bcer	NA	Bcer	FALSE	NA	NA	NA
Bc_ctg1_4404	B1	110.7465722	Bcer	NA	Bcer	FALSE	NA	NA	NA
Bc_ctg1_4405	B1	6.747999024	Bcer	NA	Bcer	FALSE	NA	NA	NA
Bc_ctg1_4406	B1	0.297705839	Bcer	NA	Bcer	FALSE	NA	NA	NA
Bc_ctg1_4407	B1	0.496176399	Bcer	NA	Bcer	FALSE	NA	NA	NA
Bc_ctg1_4408	B1	0	Bcer	NA	Bcer	FALSE	NA	NA	NA
Bc_ctg1_441	B1	7.740351821	Bcer	M	Bcer	FALSE	NA	NA	NA
Bc_ctg1_4411	B1	0	Bcer	NA	Bcer	FALSE	NA	NA	NA
Bc_ctg1_4414	B1	0.09923528	Bcer	NA	Bcer	FALSE	NA	NA	NA
Bc_ctg1_4416	B1	0	Bcer	NA	Bcer	FALSE	NA	NA	NA
Bc_ctg1_4418	B1	0	Bcer	NA	Bcer	FALSE	NA	NA	NA
Bc_ctg1_4419	B1	0.09923528	Bcer	D	Bcer	FALSE	NA	NA	NA
Bc_ctg1_442	B1	34.73234792	Bcer	NA	Bcer	FALSE	NA	NA	NA
Bc_ctg1_4420	B1	0	Bcer	NA	Bcer	FALSE	NA	NA	NA
Bc_ctg1_4422	B1	0.893117518	Bcer	NA	Bcer	FALSE	NA	NA	NA
Bc_ctg1_4423	B1	0.396941119	Bcer	NA	Bcer	FALSE	NA	NA	NA
Bc_ctg1_4424	B1	0.496176399	Bcer	NA	Bcer	FALSE	NA	NA	NA
Bc_ctg1_4425	B1	0.793882238	Bcer	NA	Bcer	FALSE	NA	NA	NA
Bc_ctg1_4426	B1	0.09923528	Bcer	S	Bcer	FALSE	NA	NA	NA
Bc_ctg1_4427	B1	0.396941119	Bcer	L	Bcer	FALSE	NA	NA	NA
Bc_ctg1_4428	B1	8.3357635	Bcer	S	Bcer	FALSE	NA	NA	NA
Bc_ctg1_4429	B1	0.09923528	Bcer	S	Bcer	FALSE	NA	NA	NA
Bc_ctg1_443	B1	17.46540924	Bcer	M	Bcer	FALSE	NA	NA	NA
Bc_ctg1_4430	B1	398.6281188	Bcer	S	Bcer	FALSE	NA	NA	NA

Bc_ctg1_4431	B1	17.26693868	Bcer	NA	Bcer	FALSE	NA	NA	NA
Bc_ctg1_4433	B1	0	Bcer	NA	Bcer	FALSE	NA	NA	NA
Bc_ctg1_4434	B1	8.43499878	Bcer	Q	Bcer	TRUE	NA	NA	NA
Bc_ctg1_4435	B1	1347.019687	Bcer	F	Bcer	FALSE	NA	NA	NA
Bc_ctg1_4436	B1	31.55681896	Bcer	NA	Bcer	FALSE	NA	NA	NA
Bc_ctg1_4437	B1	73.43410702	Bcer	L	Bcer	FALSE	NA	NA	NA
Bc_ctg1_4438	B1	5.557175667	Bcer	G	Bcer	FALSE	NA	NA	NA
Bc_ctg1_4439	B1	10.61817493	Bcer	E	Bcer	FALSE	NA	NA	NA
Bc_ctg1_444	B1	6.351057905	Bcer	NA	Bcer	FALSE	NA	NA	NA
Bc_ctg1_4440	B1	34.63311264	Bcer	NA	Bcer	FALSE	NA	NA	NA
Bc_ctg1_4441	B1	0.09923528	Bcer	C	Bcer	FALSE	NA	NA	NA
Bc_ctg1_4442	B1	0.595411679	Bcer	K	Bcer	FALSE	NA	NA	NA
Bc_ctg1_4443	B1	6.648763744	Bcer	S	Bcer	FALSE	NA	NA	NA
Bc_ctg1_4444	B1	0	Bcer	NA	Bcer	FALSE	NA	NA	NA
Bc_ctg1_4445	B1	84.54845836	Bcer	S	Bcer	FALSE	NA	NA	NA
Bc_ctg1_4446	B1	11.11435133	Bcer	S	Bcer	FALSE	NA	NA	NA
Bc_ctg1_4447	B1	23.12182018	Bcer	O	Bcer	FALSE	NA	NA	NA
Bc_ctg1_4448	B1	2.183176155	Bcer	E	Bcer	FALSE	NA	NA	NA
Bc_ctg1_4449	B1	1.290058637	Bcer	S	Bcer	FALSE	NA	NA	NA
Bc_ctg1_445	B1	769.7680651	Bcer	NA	Bcer	FALSE	NA	NA	NA
Bc_ctg1_4450	B1	0.496176399	Bcer	S	Bcer	FALSE	NA	NA	NA
Bc_ctg1_4451	B1	246.5996702	Bcer	C	Bcer	FALSE	NA	NA	NA
Bc_ctg1_4452	B1	97.34980945	Bcer	C	Bcer	FALSE	NA	NA	NA
Bc_ctg1_4453	B1	41.38111166	Bcer	C	Bcer	FALSE	NA	NA	NA
Bc_ctg1_4454	B1	63.21287321	Bcer	C	Bcer	FALSE	NA	NA	NA
Bc_ctg1_4455	B1	36.81628879	Bcer	K	Bcer	FALSE	NA	NA	NA
Bc_ctg1_4456	B1	52.89240411	Bcer	H	Bcer	FALSE	NA	NA	NA
Bc_ctg1_4457	B1	50.11381628	Bcer	H	Bcer	FALSE	NA	NA	NA
Bc_ctg1_4458	B1	72.34251895	Bcer	H	Bcer	FALSE	NA	NA	NA
Bc_ctg1_4459	B1	16.07611532	Bcer	H	Bcer	FALSE	NA	NA	NA
Bc_ctg1_446	B1	11.11435133	Bcer	T	Bcer	FALSE	NA	NA	NA
Bc_ctg1_4460	B1	1.290058637	Bcer	H	Bcer	FALSE	NA	NA	NA
Bc_ctg1_4461	B1	63.51057905	Bcer	P	Bcer	FALSE	NA	NA	NA
Bc_ctg1_4462	B1	2.282411435	Bcer	S	Bcer	FALSE	NA	NA	NA
Bc_ctg1_4463	B1	1.686999756	Bcer	NA	Bcer	FALSE	NA	NA	NA
Bc_ctg1_4464	B1	3.373999512	Bcer	H	Bcer	FALSE	NA	NA	NA
Bc_ctg1_4465	B1	20.24399707	Bcer	S	Bcer	FALSE	NA	NA	NA
Bc_ctg1_4466	B1	49.5184046	Bcer	H	Bcer	FALSE	NA	NA	NA
Bc_ctg1_4467	B1	38.20558271	Bcer	P	Bcer	FALSE	NA	NA	NA
Bc_ctg1_4468	B1	323.6062473	Bcer	C	Bcer	FALSE	NA	NA	NA
Bc_ctg1_4469	B1	7.839587101	Bcer	D	Bcer	FALSE	NA	NA	NA
Bc_ctg1_447	B1	0.893117518	Bcer	S	Bcer	FALSE	NA	NA	NA
Bc_ctg1_4470	B1	0.694646958	Bcer	M	Bcer	FALSE	NA	NA	NA
Bc_ctg1_4472	B1	0.09923528	Bcer	NA	Bcer	FALSE	NA	NA	NA
Bc_ctg1_4473	B1	16.37382116	Bcer	Q	Bcer	TRUE	NA	NA	NA
Bc_ctg1_4474	B1	6.450293184	Bcer	S	Bcer	FALSE	NA	NA	NA
Bc_ctg1_4475	B1	0.09923528	Bcer	NA	Bcer	FALSE	NA	NA	NA
Bc_ctg1_4476	B1	12.99982165	Bcer	S	Bcer	FALSE	NA	NA	NA
Bc_ctg1_4477	B1	0.396941119	Bcer	NA	Bcer	FALSE	NA	NA	NA
Bc_ctg1_448	B1	0.09923528	Bcer	S	Bcer	FALSE	NA	NA	NA
Bc_ctg1_4480	B1	24.90805522	Bcer	S	Bcer	FALSE	NA	NA	NA
Bc_ctg1_4481	B1	148.3567432	Bcer	S	Bcer	FALSE	NA	NA	NA
Bc_ctg1_4482	B1	224.7679087	Bcer	S	Bcer	FALSE	NA	NA	NA
Bc_ctg1_4483	B1	1.786235036	Bcer	S	Bcer	FALSE	NA	NA	NA
Bc_ctg1_4484	B1	1.389293917	Bcer	L	Bcer	FALSE	NA	NA	NA
Bc_ctg1_4485	B1	0	Bcer	NA	Bcer	FALSE	NA	NA	NA
Bc_ctg1_4486	B1	69.06775471	Bcer	C	Bcer	FALSE	NA	NA	NA
Bc_ctg1_4487	B1	14.58758612	Bcer	S	Bcer	FALSE	NA	NA	NA
Bc_ctg1_4488	B1	171.8755045	Bcer	S	Bcer	FALSE	NA	NA	NA
Bc_ctg1_4489	B1	20.64093819	Bcer	J	Bcer	FALSE	NA	NA	NA
Bc_ctg1_449	B1	0.992352798	Bcer	S	Bcer	FALSE	NA	NA	NA
Bc_ctg1_4490	B1	29.77058393	Bcer	J	Bcer	FALSE	NA	NA	NA

Bc_ctg1_4491	B1	1.488529196	Bcer	Q	Bcer	TRUE	NA	NA	NA
Bc_ctg1_4492	B1	45.84669925	Bcer	H	Bcer	FALSE	NA	NA	NA
Bc_ctg1_4493	B1	201.844559	Bcer	J	Bcer	FALSE	NA	NA	NA
Bc_ctg1_4494	B1	4.16788175	Bcer	S	Bcer	FALSE	NA	NA	NA
Bc_ctg1_4495	B1	3.671705351	Bcer	K	Bcer	FALSE	NA	NA	NA
Bc_ctg1_4496	B1	2.778587833	Bcer	E	Bcer	FALSE	NA	NA	NA
Bc_ctg1_4498	B1	14.78605668	Bcer	J	Bcer	FALSE	NA	NA	NA
Bc_ctg1_4499	B1	17.36617396	Bcer	S	Bcer	FALSE	NA	NA	NA
Bc_ctg1_45	B1	1.389293917	Bcer	E	Bcer	FALSE	NA	NA	NA
Bc_ctg1_450	B1	30.36599561	Bcer	S	Bcer	FALSE	NA	NA	NA
Bc_ctg1_4500	B1	15.77840948	Bcer	T	Bcer	FALSE	NA	NA	NA
Bc_ctg1_4501	B1	3.770940631	Bcer	NA	Bcer	FALSE	NA	NA	NA
Bc_ctg1_4502	B1	207.6994405	Bcer	D	Bcer	FALSE	NA	NA	NA
Bc_ctg1_4503	B1	87.12857563	Bcer	S	Bcer	FALSE	NA	NA	NA
Bc_ctg1_4504	B1	126.4257464	Bcer	KLT	Bcer	FALSE	NA	NA	NA
Bc_ctg1_4505	B1	174.4556218	Bcer	NU	Bcer	FALSE	NA	NA	NA
Bc_ctg1_4506	B1	3.96941119	Bcer	S	Bcer	FALSE	NA	NA	NA
Bc_ctg1_4507	B1	54.57940387	Bcer	MS	Bcer	FALSE	NA	NA	NA
Bc_ctg1_4508	B1	48.92299292	Bcer	S	Bcer	FALSE	NA	NA	NA
Bc_ctg1_4509	B1	10.41970437	Bcer	S	Bcer	FALSE	NA	NA	NA
Bc_ctg1_451	B1	48.5260518	Bcer	V	Bcer	FALSE	NA	NA	NA
Bc_ctg1_4510	B1	12.00746885	Bcer	I	Bcer	FALSE	NA	NA	NA
Bc_ctg1_4511	B1	60.73199121	Bcer	S	Bcer	FALSE	NA	NA	NA
Bc_ctg1_4512	B1	45.05281701	Bcer	U	Bcer	FALSE	NA	NA	NA
Bc_ctg1_4513	B1	3.076293673	Bcer	K	Bcer	FALSE	NA	NA	NA
Bc_ctg1_4514	B1	15.6791742	Bcer	EG	Bcer	FALSE	NA	NA	NA
Bc_ctg1_4515	B1	12.10670413	Bcer	C	Bcer	FALSE	NA	NA	NA
Bc_ctg1_4516	B1	4.564822869	Bcer	M	Bcer	FALSE	NA	NA	NA
Bc_ctg1_4517	B1	123.1509822	Bcer	S	Bcer	FALSE	NA	NA	NA
Bc_ctg1_4518	B1	30.06828977	Bcer	K	Bcer	FALSE	NA	NA	NA
Bc_ctg1_4519	B1	6.053352065	Bcer	S	Bcer	FALSE	NA	NA	NA
Bc_ctg1_452	B1	2.480881994	Bcer	NA	Bcer	FALSE	NA	NA	NA
Bc_ctg1_4520	B1	1.488529196	Bcer	K	Bcer	FALSE	NA	NA	NA
Bc_ctg1_4521	B1	47.83140484	Bcer	E	Bcer	FALSE	NA	NA	NA
Bc_ctg1_4522	B1	15.97688004	Bcer	K	Bcer	FALSE	NA	NA	NA
Bc_ctg1_4523	B1	13.29752749	Bcer	V	Bcer	FALSE	NA	NA	NA
Bc_ctg1_4524	B1	11.21358661	Bcer	U	Bcer	FALSE	NA	NA	NA
Bc_ctg1_4525	B1	296.6142512	Bcer	S	Bcer	FALSE	NA	NA	NA
Bc_ctg1_4526	B1	4.06864647	Bcer	S	Bcer	FALSE	NA	NA	NA
Bc_ctg1_4527	B1	0.396941119	Bcer	E	Bcer	FALSE	NA	NA	NA
Bc_ctg1_4528	B1	7.144940143	Bcer	S	Bcer	FALSE	NA	NA	NA
Bc_ctg1_4529	B1	61.42663817	Bcer	V	Bcer	FALSE	NA	NA	NA
Bc_ctg1_453	B1	66.38840216	Bcer	S	Bcer	FALSE	NA	NA	NA
Bc_ctg1_4530	B1	77.30428293	Bcer	F	Bcer	FALSE	NA	NA	NA
Bc_ctg1_4531	B1	16.47305644	Bcer	H	Bcer	FALSE	NA	NA	NA
Bc_ctg1_4532	B1	335.0183045	Bcer	M	Bcer	FALSE	NA	NA	NA
Bc_ctg1_4533	B1	84.8461642	Bcer	I	Bcer	FALSE	NA	NA	NA
Bc_ctg1_4534	B1	58.44957978	Bcer	I	Bcer	FALSE	NA	NA	NA
Bc_ctg1_4535	B1	85.44157587	Bcer	S	Bcer	FALSE	NA	NA	NA
Bc_ctg1_4536	B1	10.22123382	Bcer	U	Bcer	FALSE	NA	NA	NA
Bc_ctg1_4537	B1	105.6855729	Bcer	U	Bcer	FALSE	NA	NA	NA
Bc_ctg1_4538	B1	5.358705107	Bcer	S	Bcer	FALSE	NA	NA	NA
Bc_ctg1_4539	B1	188.8447374	Bcer	S	Bcer	FALSE	NA	NA	NA
Bc_ctg1_454	B1	2.381646714	Bcer	NA	Bcer	FALSE	NA	NA	NA
Bc_ctg1_4540	B1	17.86235036	Bcer	S	Bcer	FALSE	NA	NA	NA
Bc_ctg1_4541	B1	55.47252139	Bcer	S	Bcer	FALSE	NA	NA	NA
Bc_ctg1_4542	B1	0	Bcer	O	Bcer	FALSE	NA	NA	NA
Bc_ctg1_4543	B1	800.4317666	Bcer	E	Bcer	FALSE	NA	NA	NA
Bc_ctg1_4544	B1	1.091588077	Bcer	K	Bcer	FALSE	NA	NA	NA
Bc_ctg1_4545	B1	7.740351821	Bcer	NA	Bcer	FALSE	NA	NA	NA
Bc_ctg1_4546	B1	0.09923528	Bcer	M	Bcer	FALSE	NA	NA	NA
Bc_ctg1_4547	B1	151.6315075	Bcer	P	Bcer	FALSE	NA	NA	NA

Bc_ctg1_4548	B1	38.99946495	Bcer	E	Bcer	FALSE	NA	NA	NA	
Bc_ctg1_4549	B1	103.0062204	Bcer	C	Bcer	FALSE	NA	NA	NA	
Bc_ctg1_455	B1	0.09923528	Bcer	NA	Bcer	FALSE	NA	NA	NA	
Bc_ctg1_4550	B1	104.8916907	Bcer	NA	Bcer	FALSE	NA	NA	NA	
Bc_ctg1_4551	B1	39.29717079	Bcer	Q	Bcer	TRUE	NA	NA	NA	
Bc_ctg1_4552	B1	0	Bcer	F	Bcer	FALSE	NA	NA	NA	
Bc_ctg1_4553	B1	33.14458344	Bcer	M	Bcer	FALSE	NA	NA	NA	
Bc_ctg1_4554	B1	9.625822137	Bcer	T	Bcer	FALSE	NA	NA	NA	
Bc_ctg1_4555	B1	4.862528708	Bcer	K	Bcer	FALSE	NA	NA	NA	
Bc_ctg1_4556	B1	51.80081604	Bcer	K	Bcer	FALSE	NA	NA	NA	
Bc_ctg1_4557	B1	45.44975813	Bcer	S	Bcer	FALSE	NA	NA	NA	
Bc_ctg1_4558	B1	4204.102627	Bcer	C	Bcer	FALSE	NA	NA	NA	
Bc_ctg1_4559	B1	2.877823113	Bcer	EP	Bcer	FALSE	NA	NA	NA	
Bc_ctg1_456	B1	2.778587833	Bcer	L	Bcer	FALSE	NA	NA	NA	
Bc_ctg1_4560	B1	1.587764476	Bcer	EP	Bcer	FALSE	NA	NA	NA	
Bc_ctg1_4562	B1	104.5939849	Bcer	G	Bcer	FALSE	NA	NA	NA	
Bc_ctg1_4563	B1	81.57139996	Bcer	EG	Bcer	FALSE	NA	NA	NA	
Bc_ctg1_4564	B1	63.11363793	Bcer	G	Bcer	FALSE	NA	NA	NA	
Bc_ctg1_4565	B1	2.381646714	Bcer	S	Bcer	FALSE	NA	NA	NA	
Bc_ctg1_4566	B1	0.595411679	Bcer	M	Bcer	FALSE	NA	NA	NA	
Bc_ctg1_4567	B1	1.290058637	Bcer	O	Bcer	FALSE	NA	NA	NA	
Bc_ctg1_4568	B1	10.22123382	Bcer	S	Bcer	FALSE	NA	NA	NA	
Bc_ctg1_4569	B1	53.98399219	Bcer	EGP	Bcer	FALSE	NA	NA	NA	
Bc_ctg1_457	B1	494.6878696	Bcer	K	Bcer	FALSE	NA	NA	NA	
Bc_ctg1_4570	B1	21.43482043	Bcer	S	Bcer	FALSE	NA	NA	NA	
Bc_ctg1_4571	B1	10.61817493	Bcer	E	Bcer	FALSE	NA	NA	NA	
Bc_ctg1_4572	B1	16.96923284	Bcer	EM	Bcer	FALSE	NA	NA	NA	
Bc_ctg1_4573	B1	46.83905205	Bcer	E	Bcer	FALSE	NA	NA	NA	
Bc_ctg1_4574	B1	36.12164183	Bcer	NA	Bcer	FALSE	NA	NA	NA	
Bc_ctg1_4575	B1	2.183176155	Bcer	V	Bcer	FALSE	NA	NA	NA	
Bc_ctg1_4576	B1	0.893117518	Bcer	NA	Bcer	FALSE	NA	NA	NA	
Bc_ctg1_4577	B1	72.24328367	Bcer	NA	Bcer	FALSE	NA	NA	NA	
Bc_ctg1_4578	B1	0.19847056	Bcer	K	Bcer	FALSE	NA	NA	NA	
Bc_ctg1_4579	B1	33.04534816	Bcer	S	Bcer	FALSE	NA	NA	NA	
Bc_ctg1_458	B1	134.066863	Bcer	NA	Bcer	FALSE	NA	NA	NA	
Bc_ctg1_4580	B1	55.37328611	Bcer	C	Bcer	FALSE	NA	NA	NA	
Bc_ctg1_4581	B1	107.7695138	Bcer	S	Bcer	FALSE	NA	NA	NA	
Bc_ctg1_4582	B1	141.4102737	Bcer	T	Bcer	FALSE	NA	NA	NA	
Bc_ctg1_4583	B1	0.694646958	Bcer	O	Bcer	FALSE	NA	NA	NA	
Bc_ctg1_4584	B1	2.282411435	Bcer	H	Bcer	FALSE	NA	NA	NA	
Bc_ctg1_4585	B1	1.389293917	Bcer	I	Bcer	FALSE	NA	NA	NA	
Bc_ctg1_4586	B1	1.389293917	Bcer	I	Bcer	FALSE	NA	NA	NA	
Bc_ctg1_4587	B1	1.885470315	Bcer	E	Bcer	FALSE	NA	NA	NA	
Bc_ctg1_4588	B1	21.13711459	Bcer	K	Bcer	FALSE	NA	NA	NA	
Bc_ctg1_4589	B1	30.46523089	Bcer	KT	Bcer	FALSE	NA	NA	NA	
Bc_ctg1_459	B1	18.1600562	Bcer	C	Bcer	FALSE	NA	NA	NA	
Bc_ctg1_4590	B1	7.343410702	Bcer	E	Bcer	FALSE	NA	NA	NA	
Bc_ctg1_4591	B1	1.190823357	Bcer	S	Bcer	FALSE	NA	NA	NA	
Bc_ctg1_4592	B1	6.152587345	Bcer	S	Bcer	FALSE	NA	NA	NA	
Bc_ctg1_4593	B1	0	Bcer	NA	Bcer	FALSE	NA	NA	NA	
Bc_ctg1_4596	B1	9.725057417	Bcer	NA	Bcer	FALSE	NA	NA	NA	
Bc_ctg1_4597	B1	46.64058149	Bcer	KLT	Bcer	FALSE	NA	NA	NA	
Bc_ctg1_4598	B1	57.15952114	Bcer	S	Bcer	FALSE	NA	NA	NA	
Bc_ctg1_4599	B1	15.48070364	Bcer	I	Bcer	FALSE	NA	NA	NA	
Bc_ctg1_46	B1	2.183176155	Bcer	S	Bcer	FALSE	NA	NA	NA	
Bc_ctg1_460	B1	6.648763744	Bcer	S	Bcer	FALSE	NA	NA	NA	
Bc_ctg1_4600	B1	1.686999756	Bcer	P	Bcer	FALSE	NA	NA	NA	
Bc_ctg1_4602	B1	128.0135109	Bcer	CO	Bcer	FALSE	NA	NA	NA	
Bc_ctg1_4603	B1	32.25146592	Bcer	J	Bcer	FALSE	NA	NA	NA	
Bc_ctg1_4604	B1	0.09923528	Bcer	NA	Bcer	FALSE	NA	NA	NA	
Bc_ctg1_4605	B1	0.09923528	Bcer	NA	Bcer	FALSE	NA	NA	NA	
Bc_ctg1_4606	B1	0.893117518	Bcer	NA	Bcer	FALSE	NA	NA	NA	

Bc_ctg1_4607	B1	0.09923528	Bcer	NA	Bcer	FALSE	NA	NA	NA
Bc_ctg1_4608	B1	0.297705839	Bcer	NA	Bcer	FALSE	NA	NA	NA
Bc_ctg1_4609	B1	0	Bcer	NA	Bcer	FALSE	NA	NA	NA
Bc_ctg1_461	B1	0.09923528	Bcer	F	Bcer	FALSE	NA	NA	NA
Bc_ctg1_4610	B1	15.48070364	Bcer	M	Bcer	FALSE	NA	NA	NA
Bc_ctg1_4611	B1	59.04499146	Bcer	M	Bcer	FALSE	NA	NA	NA
Bc_ctg1_4612	B1	0.09923528	Bcer	NA	Bcer	FALSE	NA	NA	NA
Bc_ctg1_4613	B1	0.297705839	Bcer	NA	Bcer	FALSE	NA	NA	NA
Bc_ctg1_4614	B1	0.793882238	Bcer	S	Bcer	FALSE	NA	NA	NA
Bc_ctg1_4616	B1	32.44993648	Bcer	NA	Bcer	FALSE	NA	NA	NA
Bc_ctg1_4617	B1	0.297705839	Bcer	NA	Bcer	FALSE	NA	NA	NA
Bc_ctg1_4618	B1	15.6791742	Bcer	IQ	Bcer	TRUE	NA	NA	NA
Bc_ctg1_4619	B1	0.396941119	Bcer	NA	Bcer	FALSE	NA	NA	NA
Bc_ctg1_462	B1	0.496176399	Bcer	S	Bcer	FALSE	NA	NA	NA
Bc_ctg1_4620	B1	1.984705595	Bcer	S	Bcer	FALSE	NA	NA	NA
Bc_ctg1_4621	B1	0	Bcer	S	Bcer	FALSE	NA	NA	NA
Bc_ctg1_4622	B1	539.5422161	Bcer	M	Bcer	FALSE	NA	NA	NA
Bc_ctg1_4623	B1	0	Bcer	NA	Bcer	FALSE	NA	NA	NA
Bc_ctg1_4624	B1	3.870175911	Bcer	EGP	Bcer	FALSE	NA	NA	NA
Bc_ctg1_4626	B1	2.778587833	Bcer	C	Bcer	FALSE	NA	NA	NA
Bc_ctg1_4627	B1	10.71741021	Bcer	S	Bcer	FALSE	Bc_Ga0417192_01_rn32	Other	NA
Bc_ctg1_4628	B1	3.373999512	Bcer	G	Bcer	FALSE	Bc_Ga0417192_01_rn32	Ancillary biosynthesis	SMCOG1231
Bc_ctg1_4629	B1	83.45687028	Bcer	C	Bcer	FALSE	Bc_Ga0417192_01_rn32	Ancillary biosynthesis	SMCOG1006
Bc_ctg1_463	B1	4.366352309	Bcer	S	Bcer	FALSE	NA	NA	NA
Bc_ctg1_4630	B1	39.39640607	Bcer	I	Bcer	FALSE	Bc_Ga0417192_01_rn32	Ancillary biosynthesis	SMCOG1100
Bc_ctg1_4631	B1	123.8456291	Bcer	C	Bcer	FALSE	Bc_Ga0417192_01_rn32	Ancillary biosynthesis	SMCOG1017
Bc_ctg1_4632	B1	6.847234304	Bcer	E	Bcer	FALSE	Bc_Ga0417192_01_rn32	Transport	SMCOG1116
Bc_ctg1_4633	B1	181.600562	Bcer	I	Bcer	FALSE	Bc_Ga0417192_01_rn32	Ancillary biosynthesis	SMCOG1023
Bc_ctg1_4634	B1	3.175528952	Bcer	NA	Bcer	FALSE	Bc_Ga0417192_01_rn32	Other	NA
Bc_ctg1_4635	B1	2.778587833	Bcer	S	Bcer	FALSE	Bc_Ga0417192_01_rn32	Other	SMCOG1300
Bc_ctg1_4636	B1	39.49564134	Bcer	L	Bcer	FALSE	Bc_Ga0417192_01_rn32	Other	SMCOG1230
Bc_ctg1_4637	B1	122.6548058	Bcer	L	Bcer	FALSE	Bc_Ga0417192_01_rn32	Other	SMCOG1218
Bc_ctg1_4639	B1	53.09087467	Bcer	K	Bcer	FALSE	Bc_Ga0417192_01_rn32	Regulation	SMCOG1058
Bc_ctg1_464	B1	7.740351821	Bcer	M	Bcer	FALSE	NA	NA	NA
Bc_ctg1_4640	B1	0.297705839	Bcer	NA	Bcer	FALSE	Bc_Ga0417192_01_rn32	Other	NA
Bc_ctg1_4641	B1	768.7757123	Bcer	P	Bcer	FALSE	Bc_Ga0417192_01_rn32	Transport	SMCOG1137
Bc_ctg1_4642	B1	54.77787443	Bcer	IQ	Bcer	TRUE	Bc_Ga0417192_01_rn32	Ancillary biosynthesis	SMCOG1001
Bc_ctg1_4643	B1	86.53316395	Bcer	HQ	Bcer	TRUE	Bc_Ga0417192_01_rn32	Ancillary biosynthesis	SMCOG1018
Bc_ctg1_4644	B1	155.6009187	Bcer	Q	Bcer	TRUE	Bc_Ga0417192_01_rn32	Core biosynthesis	SMCOG1002
Bc_ctg1_4645	B1	56.96105058	Bcer	Q	Bcer	TRUE	Bc_Ga0417192_01_rn32	Ancillary biosynthesis	SMCOG1027
Bc_ctg1_4646	B1	791.004415	Bcer	Q	Bcer	TRUE	Bc_Ga0417192_01_rn32	Core biosynthesis	SMCOG1127
Bc_ctg1_4647	B1	13.19829221	Bcer	S	Bcer	FALSE	Bc_Ga0417192_01_rn32	Ancillary biosynthesis	SMCOG1009
Bc_ctg1_4648	B1	63.51057905	Bcer	P	Bcer	FALSE	Bc_Ga0417192_01_rn32	Transport	SMCOG1005
Bc_ctg1_4649	B1	13.99217445	Bcer	H	Bcer	FALSE	Bc_Ga0417192_01_rn32	Ancillary biosynthesis	SMCOG1012
Bc_ctg1_465	B1	68.96851943	Bcer	IQ	Bcer	TRUE	Bc_Ga0417192_01_rn06	Other	NA
Bc_ctg1_4650	B1	39.79334718	Bcer	H	Bcer	FALSE	Bc_Ga0417192_01_rn32	Ancillary biosynthesis	SMCOG1259
Bc_ctg1_4651	B1	20.74017347	Bcer	L	Bcer	FALSE	Bc_Ga0417192_01_rn32	Other	SMCOG1132
Bc_ctg1_4652	B1	27.98434889	Bcer	S	Bcer	FALSE	Bc_Ga0417192_01_rn32	Other	SMCOG1267
Bc_ctg1_4653	B1	50.11381628	Bcer	S	Bcer	FALSE	Bc_Ga0417192_01_rn32	Other	SMCOG1250
Bc_ctg1_4654	B1	0	Bcer	O	Bcer	FALSE	Bc_Ga0417192_01_rn32	Ancillary biosynthesis	SMCOG1075
Bc_ctg1_4655	B1	22.72487907	Bcer	E	Bcer	FALSE	Bc_Ga0417192_01_rn32	Ancillary biosynthesis	SMCOG1153
Bc_ctg1_4656	B1	4.961763988	Bcer	NA	Bcer	FALSE	Bc_Ga0417192_01_rn32	Other	SMCOG1289
Bc_ctg1_4657	B1	0.496176399	Bcer	NA	Bcer	FALSE	Bc_Ga0417192_01_rn32	Other	NA
Bc_ctg1_4658	B1	2.183176155	Bcer	G	Bcer	FALSE	Bc_Ga0417192_01_rn32	Ancillary biosynthesis	SMCOG1258
Bc_ctg1_4659	B1	40.88493526	Bcer	J	Bcer	FALSE	Bc_Ga0417192_01_rn32	Ancillary biosynthesis	SMCOG1154
Bc_ctg1_466	B1	33.343054	Bcer	S	Bcer	FALSE	Bc_Ga0417192_01_rn06	Other	NA
Bc_ctg1_4660	B1	58.74728562	Bcer	J	Bcer	FALSE	Bc_Ga0417192_01_rn32	Other	SMCOG1162
Bc_ctg1_4661	B1	2.381646714	Bcer	V	Bcer	FALSE	Bc_Ga0417192_01_rn32	Transport	SMCOG1000
Bc_ctg1_4662	B1	19.35087955	Bcer	V	Bcer	FALSE	Bc_Ga0417192_01_rn32	Transport	SMCOG1243
Bc_ctg1_4663	B1	8.13729294	Bcer	G	Bcer	FALSE	Bc_Ga0417192_01_rn32	Transport	SMCOG1020
Bc_ctg1_4664	B1	4.862528708	Bcer	P	Bcer	FALSE	Bc_Ga0417192_01_rn32	Transport	SMCOG1118
Bc_ctg1_4665	B1	22.32793795	Bcer	P	Bcer	FALSE	Bc_Ga0417192_01_rn32	Transport	SMCOG1085

Bc_ctg1_4666	B1	2.480881994	Bcer	EH	Bcer	FALSE	NA	NA	NA
Bc_ctg1_4667	B1	0.19847056	Bcer	P	Bcer	FALSE	NA	NA	NA
Bc_ctg1_4668	B1	21.53405571	Bcer	S	Bcer	FALSE	NA	NA	NA
Bc_ctg1_4669	B1	9.625822137	Bcer	F	Bcer	FALSE	NA	NA	NA
Bc_ctg1_467	B1	1.984705595	Bcer	E	Bcer	FALSE	Bc_Ga0417192_01_rn06	Ancillary biosynthesis	SMCOG1063
Bc_ctg1_4670	B1	6.847234304	Bcer	S	Bcer	FALSE	NA	NA	NA
Bc_ctg1_4671	B1	6.053352065	Bcer	NA	Bcer	FALSE	NA	NA	NA
Bc_ctg1_4673	B1	0.19847056	Bcer	K	Bcer	FALSE	NA	NA	NA
Bc_ctg1_4674	B1	0	Bcer	S	Bcer	FALSE	NA	NA	NA
Bc_ctg1_4675	B1	2.580117274	Bcer	H	Bcer	FALSE	NA	NA	NA
Bc_ctg1_4676	B1	2.282411435	Bcer	K	Bcer	FALSE	NA	NA	NA
Bc_ctg1_4677	B1	1.091588077	Bcer	K	Bcer	FALSE	NA	NA	NA
Bc_ctg1_4678	B1	4.465587589	Bcer	S	Bcer	FALSE	NA	NA	NA
Bc_ctg1_4679	B1	12.80135109	Bcer	K	Bcer	FALSE	NA	NA	NA
Bc_ctg1_468	B1	87.52551675	Bcer	V	Bcer	FALSE	Bc_Ga0417192_01_rn06	Other	NA
Bc_ctg1_4680	B1	22.22870267	Bcer	S	Bcer	FALSE	NA	NA	NA
Bc_ctg1_4682	B1	10.41970437	Bcer	V	Bcer	FALSE	NA	NA	NA
Bc_ctg1_4683	B1	1.587764476	Bcer	K	Bcer	FALSE	NA	NA	NA
Bc_ctg1_4684	B1	5.755646226	Bcer	K	Bcer	FALSE	NA	NA	NA
Bc_ctg1_4685	B1	10.12199854	Bcer	J	Bcer	FALSE	NA	NA	NA
Bc_ctg1_4686	B1	3.274764232	Bcer	EG	Bcer	FALSE	NA	NA	NA
Bc_ctg1_4687	B1	3.870175911	Bcer	K	Bcer	FALSE	NA	NA	NA
Bc_ctg1_4688	B1	12.40440997	Bcer	NA	Bcer	FALSE	NA	NA	NA
Bc_ctg1_4689	B1	0	Bcer	NA	Bcer	FALSE	NA	NA	NA
Bc_ctg1_469	B1	5.954116786	Bcer	F	Bcer	FALSE	Bc_Ga0417192_01_rn06	Other	NA
Bc_ctg1_4690	B1	92.98345714	Bcer	H	Bcer	FALSE	NA	NA	NA
Bc_ctg1_4691	B1	1.290058637	Bcer	NA	Bcer	FALSE	NA	NA	NA
Bc_ctg1_4692	B1	146.2728024	Bcer	J	Bcer	FALSE	NA	NA	NA
Bc_ctg1_4693	B1	32.74764232	Bcer	S	Bcer	FALSE	NA	NA	NA
Bc_ctg1_4694	B1	33.343054	Bcer	K	Bcer	FALSE	NA	NA	NA
Bc_ctg1_4695	B1	3.076293673	Bcer	S	Bcer	FALSE	NA	NA	NA
Bc_ctg1_4696	B1	5.557175667	Bcer	F	Bcer	FALSE	NA	NA	NA
Bc_ctg1_4697	B1	5.358705107	Bcer	NA	Bcer	FALSE	NA	NA	NA
Bc_ctg1_4698	B1	6.251822625	Bcer	K	Bcer	FALSE	NA	NA	NA
Bc_ctg1_4699	B1	5.457940387	Bcer	E	Bcer	FALSE	NA	NA	NA
Bc_ctg1_47	B1	0.496176399	Bcer	K	Bcer	FALSE	NA	NA	NA
Bc_ctg1_470	B1	7.442645982	Bcer	H	Bcer	FALSE	Bc_Ga0417192_01_rn06	Other	NA
Bc_ctg1_4700	B1	1.984705595	Bcer	NA	Bcer	FALSE	NA	NA	NA
Bc_ctg1_4701	B1	7.938822381	Bcer	S	Bcer	FALSE	NA	NA	NA
Bc_ctg1_4702	B1	15.48070364	Bcer	K	Bcer	FALSE	NA	NA	NA
Bc_ctg1_4703	B1	10.02276326	Bcer	K	Bcer	FALSE	NA	NA	NA
Bc_ctg1_4704	B1	0.793882238	Bcer	S	Bcer	FALSE	NA	NA	NA
Bc_ctg1_4705	B1	59.04499146	Bcer	S	Bcer	FALSE	NA	NA	NA
Bc_ctg1_4706	B1	44.15969949	Bcer	S	Bcer	FALSE	NA	NA	NA
Bc_ctg1_4707	B1	53.68628635	Bcer	V	Bcer	FALSE	NA	NA	NA
Bc_ctg1_4708	B1	79.18975325	Bcer	V	Bcer	FALSE	NA	NA	NA
Bc_ctg1_4709	B1	0.893117518	Bcer	NA	Bcer	FALSE	NA	NA	NA
Bc_ctg1_471	B1	0.893117518	Bcer	S	Bcer	FALSE	Bc_Ga0417192_01_rn06	Other	NA
Bc_ctg1_4710	B1	0.396941119	Bcer	S	Bcer	FALSE	NA	NA	NA
Bc_ctg1_4711	B1	10.61817493	Bcer	NA	Bcer	FALSE	NA	NA	NA
Bc_ctg1_4712	B1	4.06864647	Bcer	NA	Bcer	FALSE	NA	NA	NA
Bc_ctg1_4713	B1	6.053352065	Bcer	E	Bcer	FALSE	NA	NA	NA
Bc_ctg1_4714	B1	6.351057905	Bcer	K	Bcer	FALSE	NA	NA	NA
Bc_ctg1_4715	B1	0.09923528	Bcer	NA	Bcer	FALSE	NA	NA	NA
Bc_ctg1_4716	B1	2.183176155	Bcer	M	Bcer	FALSE	NA	NA	NA
Bc_ctg1_4717	B1	1.488529196	Bcer	M	Bcer	FALSE	NA	NA	NA
Bc_ctg1_4719	B1	0	Bcer	G	Bcer	FALSE	NA	NA	NA
Bc_ctg1_472	B1	53.68628635	Bcer	C	Bcer	FALSE	Bc_Ga0417192_01_rn06	Ancillary biosynthesis	SMCOG1017
Bc_ctg1_4720	B1	0	Bcer	NA	Bcer	FALSE	NA	NA	NA
Bc_ctg1_4721	B1	1.686999756	Bcer	H	Bcer	FALSE	NA	NA	NA
Bc_ctg1_4722	B1	4.465587589	Bcer	K	Bcer	FALSE	NA	NA	NA
Bc_ctg1_4724	B1	25.0072905	Bcer	S	Bcer	FALSE	NA	NA	NA

Bc_ctg1_4725	B1	152.3261544	Bcer	P	Bcer	FALSE	NA	NA	NA
Bc_ctg1_4726	B1	390.887767	Bcer	S	Bcer	FALSE	NA	NA	NA
Bc_ctg1_4727	B1	0.19847056	Bcer	T	Bcer	FALSE	NA	NA	NA
Bc_ctg1_4728	B1	0	Bcer	G	Bcer	FALSE	NA	NA	NA
Bc_ctg1_4729	B1	0.19847056	Bcer	G	Bcer	FALSE	NA	NA	NA
Bc_ctg1_473	B1	34.8315832	Bcer	IQ	Bcer	TRUE	Bc_Ga0417192_01_rn06	Ancillary biosynthesis	SMCOG1001
Bc_ctg1_4730	B1	0	Bcer	NA	Bcer	FALSE	NA	NA	NA
Bc_ctg1_4731	B1	1.389293917	Bcer	O	Bcer	FALSE	NA	NA	NA
Bc_ctg1_4732	B1	0.694646958	Bcer	G	Bcer	FALSE	NA	NA	NA
Bc_ctg1_4733	B1	6.351057905	Bcer	I	Bcer	FALSE	NA	NA	NA
Bc_ctg1_4734	B1	14.28988029	Bcer	E	Bcer	FALSE	NA	NA	NA
Bc_ctg1_4735	B1	5.160234548	Bcer	E	Bcer	FALSE	NA	NA	NA
Bc_ctg1_4736	B1	1.984705595	Bcer	K	Bcer	FALSE	NA	NA	NA
Bc_ctg1_4737	B1	0.694646958	Bcer	EGP	Bcer	FALSE	NA	NA	NA
Bc_ctg1_4738	B1	4.465587589	Bcer	S	Bcer	FALSE	NA	NA	NA
Bc_ctg1_4739	B1	5.656410946	Bcer	S	Bcer	FALSE	NA	NA	NA
Bc_ctg1_4741	B1	157.8833301	Bcer	L	Bcer	FALSE	NA	NA	NA
Bc_ctg1_4742	B1	4.664058149	Bcer	NA	Bcer	FALSE	NA	NA	NA
Bc_ctg1_4743	B1	8.23652822	Bcer	K	Bcer	FALSE	NA	NA	NA
Bc_ctg1_4744	B1	6.946469583	Bcer	EGP	Bcer	FALSE	NA	NA	NA
Bc_ctg1_4746	B1	0	Bcer	NA	Bcer	FALSE	NA	NA	NA
Bc_ctg1_4747	B1	0.694646958	Bcer	S	Bcer	FALSE	NA	NA	NA
Bc_ctg1_4748	B1	0.19847056	Bcer	NA	Bcer	FALSE	NA	NA	NA
Bc_ctg1_4749	B1	0.396941119	Bcer	NA	Bcer	FALSE	NA	NA	NA
Bc_ctg1_475	B1	3.274764232	Bcer	I	Bcer	FALSE	Bc_Ga0417192_01_rn06	Core biosynthesis	None
Bc_ctg1_4750	B1	0.595411679	Bcer	NA	Bcer	FALSE	NA	NA	NA
Bc_ctg1_4751	B1	4.06864647	Bcer	NA	Bcer	FALSE	NA	NA	NA
Bc_ctg1_4754	B1	0.496176399	Bcer	E	Bcer	FALSE	NA	NA	NA
Bc_ctg1_4755	B1	2.282411435	Bcer	N	Bcer	FALSE	NA	NA	NA
Bc_ctg1_4756	B1	0.09923528	Bcer	NA	Bcer	FALSE	NA	NA	NA
Bc_ctg1_4758	B1	0.19847056	Bcer	NA	Bcer	FALSE	NA	NA	NA
Bc_ctg1_4759	B1	4.366352309	Bcer	NA	Bcer	FALSE	NA	NA	NA
Bc_ctg1_476	B1	25.0072905	Bcer	S	Bcer	FALSE	Bc_Ga0417192_01_rn06	Other	NA
Bc_ctg1_4760	B1	0.396941119	Bcer	S	Bcer	FALSE	NA	NA	NA
Bc_ctg1_4761	B1	0	Bcer	NA	Bcer	FALSE	NA	NA	NA
Bc_ctg1_4762	B1	1.091588077	Bcer	NA	Bcer	FALSE	NA	NA	NA
Bc_ctg1_4763	B1	3.274764232	Bcer	NA	Bcer	FALSE	NA	NA	NA
Bc_ctg1_4765	B1	2.778587833	Bcer	Q	Bcer	TRUE	NA	NA	NA
Bc_ctg1_4766	B1	3.373999512	Bcer	S	Bcer	FALSE	NA	NA	NA
Bc_ctg1_4767	B1	14.28988029	Bcer	Q	Bcer	TRUE	NA	NA	NA
Bc_ctg1_4768	B1	0.595411679	Bcer	GH	Bcer	FALSE	NA	NA	NA
Bc_ctg1_4769	B1	264.4620206	Bcer	K	Bcer	FALSE	NA	NA	NA
Bc_ctg1_477	B1	55.77022723	Bcer	S	Bcer	FALSE	Bc_Ga0417192_01_rn06	Transport	SMCOG1166
Bc_ctg1_4770	B1	217.1267921	Bcer	G	Bcer	FALSE	Bc_Ga0417192_01_rn33	Ancillary biosynthesis	SMCOG1101
Bc_ctg1_4771	B1	147.6620963	Bcer	S	Bcer	FALSE	Bc_Ga0417192_01_rn33	Other	NA
Bc_ctg1_4773	B1	2.977058393	Bcer	NA	Bcer	FALSE	Bc_Ga0417192_01_rn33	Other	NA
Bc_ctg1_4774	B1	1.488529196	Bcer	S	Bcer	FALSE	Bc_Ga0417192_01_rn33	Other	NA
Bc_ctg1_4775	B1	1089.504136	Bcer	E	Bcer	FALSE	Bc_Ga0417192_01_rn33	Ancillary biosynthesis	SMCOG1247
Bc_ctg1_4776	B1	0.09923528	Bcer	S	Bcer	FALSE	Bc_Ga0417192_01_rn33	Other	NA
Bc_ctg1_4777	B1	1.488529196	Bcer	NA	Bcer	FALSE	Bc_Ga0417192_01_rn33	Other	NA
Bc_ctg1_4778	B1	7.442645982	Bcer	E	Bcer	FALSE	Bc_Ga0417192_01_rn33	Other	NA
Bc_ctg1_4779	B1	0.992352798	Bcer	KT	Bcer	FALSE	Bc_Ga0417192_01_rn33	Other	NA
Bc_ctg1_478	B1	0	Bcer	S	Bcer	FALSE	Bc_Ga0417192_01_rn06	Other	NA
Bc_ctg1_4781	B1	4.763293429	Bcer	K	Bcer	FALSE	Bc_Ga0417192_01_rn33	Other	NA
Bc_ctg1_4782	B1	0.893117518	Bcer	K	Bcer	FALSE	Bc_Ga0417192_01_rn33	Other	NA
Bc_ctg1_4783	B1	3.373999512	Bcer	NA	Bcer	FALSE	Bc_Ga0417192_01_rn33	Other	NA
Bc_ctg1_4784	B1	1.389293917	Bcer	S	Bcer	FALSE	Bc_Ga0417192_01_rn33	Other	NA
Bc_ctg1_4785	B1	13.59523333	Bcer	S	Bcer	FALSE	Bc_Ga0417192_01_rn33	Ancillary biosynthesis	SMCOG1156
Bc_ctg1_4786	B1	0.496176399	Bcer	M	Bcer	FALSE	Bc_Ga0417192_01_rn33	Other	NA
Bc_ctg1_4787	B1	2.381646714	Bcer	I	Bcer	FALSE	Bc_Ga0417192_01_rn33	Ancillary biosynthesis	SMCOG1088
Bc_ctg1_4788	B1	17.26693868	Bcer	E	Bcer	FALSE	Bc_Ga0417192_01_rn33	Other	NA
Bc_ctg1_4789	B1	1.488529196	Bcer	EGP	Bcer	FALSE	Bc_Ga0417192_01_rn33	Transport	SMCOG1020

Bc_ctg1_479	B1	7.442645982	Bcer	NA	Bcer	FALSE	Bc_Ga0417192_01_rn06	Other	NA
Bc_ctg1_4790	B1	3.870175911	Bcer	Q	Bcer	TRUE	Bc_Ga0417192_01_rn33	Core biosynthesis	SMCOG1002
Bc_ctg1_4791	B1	8.43499878	Bcer	Q	Bcer	TRUE	Bc_Ga0417192_01_rn33	Core biosynthesis	SMCOG1127
Bc_ctg1_4792	B1	49.0222282	Bcer	Q	Bcer	TRUE	Bc_Ga0417192_01_rn33	Core biosynthesis	SMCOG1127
Bc_ctg1_4793	B1	1.984705595	Bcer	H	Bcer	FALSE	Bc_Ga0417192_01_rn33	Ancillary biosynthesis	SMCOG1012
Bc_ctg1_4794	B1	5.954116786	Bcer	Q	Bcer	TRUE	Bc_Ga0417192_01_rn33	Ancillary biosynthesis	SMCOG1004
Bc_ctg1_4795	B1	3.96941119	Bcer	P	Bcer	FALSE	Bc_Ga0417192_01_rn33	Other	NA
Bc_ctg1_4796	B1	2.282411435	Bcer	E	Bcer	FALSE	Bc_Ga0417192_01_rn33	Ancillary biosynthesis	SMCOG1153
Bc_ctg1_4798	B1	1.885470315	Bcer	E	Bcer	FALSE	Bc_Ga0417192_01_rn33	Other	NA
Bc_ctg1_4799	B1	23.32029074	Bcer	T	Bcer	FALSE	Bc_Ga0417192_01_rn33	Other	NA
Bc_ctg1_48	B1	1.686999756	Bcer	IQ	Bcer	TRUE	NA	NA	NA
Bc_ctg1_480	B1	2.580117274	Bcer	NA	Bcer	FALSE	Bc_Ga0417192_01_rn06	Other	NA
Bc_ctg1_4800	B1	15.48070364	Bcer	T	Bcer	FALSE	Bc_Ga0417192_01_rn33	Regulation	SMCOG1008
Bc_ctg1_4801	B1	0.496176399	Bcer	EG	Bcer	FALSE	Bc_Ga0417192_01_rn33	Other	NA
Bc_ctg1_4802	B1	0.19847056	Bcer	S	Bcer	FALSE	Bc_Ga0417192_01_rn33	Ancillary biosynthesis	SMCOG1038
Bc_ctg1_4803	B1	0.694646958	Bcer	S	Bcer	FALSE	Bc_Ga0417192_01_rn33	Other	NA
Bc_ctg1_4804	B1	0.09923528	Bcer	E	Bcer	FALSE	Bc_Ga0417192_01_rn33	Other	NA
Bc_ctg1_4805	B1	1.290058637	Bcer	E	Bcer	FALSE	Bc_Ga0417192_01_rn33	Ancillary biosynthesis	SMCOG1038
Bc_ctg1_4806	B1	3.770940631	Bcer	L	Bcer	FALSE	Bc_Ga0417192_01_rn33	Other	NA
Bc_ctg1_4807	B1	7.641116542	Bcer	K	Bcer	FALSE	NA	NA	NA
Bc_ctg1_4808	B1	883.6901663	Bcer	S	Bcer	FALSE	NA	NA	NA
Bc_ctg1_4809	B1	607.1214416	Bcer	E	Bcer	FALSE	NA	NA	NA
Bc_ctg1_481	B1	35.92317127	Bcer	D	Bcer	FALSE	Bc_Ga0417192_01_rn06	Other	SMCOG1157
Bc_ctg1_4810	B1	996.4214441	Bcer	E	Bcer	FALSE	NA	NA	NA
Bc_ctg1_4811	B1	97.25057417	Bcer	E	Bcer	FALSE	NA	NA	NA
Bc_ctg1_4812	B1	72.73946007	Bcer	T	Bcer	FALSE	NA	NA	NA
Bc_ctg1_4813	B1	0.09923528	Bcer	P	Bcer	FALSE	NA	NA	NA
Bc_ctg1_4814	B1	1.190823357	Bcer	E	Bcer	FALSE	NA	NA	NA
Bc_ctg1_4815	B1	0.992352798	Bcer	S	Bcer	FALSE	NA	NA	NA
Bc_ctg1_4817	B1	3.870175911	Bcer	M	Bcer	FALSE	NA	NA	NA
Bc_ctg1_4818	B1	49.71687516	Bcer	J	Bcer	FALSE	NA	NA	NA
Bc_ctg1_4819	B1	0.297705839	Bcer	NA	Bcer	FALSE	NA	NA	NA
Bc_ctg1_482	B1	1.190823357	Bcer	H	Bcer	FALSE	Bc_Ga0417192_01_rn06	Other	NA
Bc_ctg1_4820	B1	31.45758368	Bcer	M	Bcer	FALSE	NA	NA	NA
Bc_ctg1_4821	B1	3.175528952	Bcer	K	Bcer	FALSE	NA	NA	NA
Bc_ctg1_4822	B1	1.091588077	Bcer	NA	Bcer	FALSE	NA	NA	NA
Bc_ctg1_4823	B1	1.786235036	Bcer	G	Bcer	FALSE	NA	NA	NA
Bc_ctg1_4824	B1	3.671705351	Bcer	K	Bcer	FALSE	NA	NA	NA
Bc_ctg1_4825	B1	6.946469583	Bcer	E	Bcer	FALSE	NA	NA	NA
Bc_ctg1_4826	B1	24.11417298	Bcer	T	Bcer	FALSE	NA	NA	NA
Bc_ctg1_4827	B1	7.144940143	Bcer	T	Bcer	FALSE	NA	NA	NA
Bc_ctg1_4828	B1	1.686999756	Bcer	O	Bcer	FALSE	NA	NA	NA
Bc_ctg1_4829	B1	1.587764476	Bcer	S	Bcer	FALSE	NA	NA	NA
Bc_ctg1_483	B1	1.686999756	Bcer	H	Bcer	FALSE	Bc_Ga0417192_01_rn06	Other	NA
Bc_ctg1_4830	B1	7.343410702	Bcer	S	Bcer	FALSE	NA	NA	NA
Bc_ctg1_4834	B1	7.938822381	Bcer	KT	Bcer	FALSE	NA	NA	NA
Bc_ctg1_4835	B1	7.641116542	Bcer	NA	Bcer	FALSE	NA	NA	NA
Bc_ctg1_4836	B1	1.190823357	Bcer	NA	Bcer	FALSE	NA	NA	NA
Bc_ctg1_4837	B1	61.02969705	Bcer	NA	Bcer	FALSE	NA	NA	NA
Bc_ctg1_4838	B1	0	Bcer	NA	Bcer	FALSE	NA	NA	NA
Bc_ctg1_4839	B1	2.778587833	Bcer	NA	Bcer	FALSE	NA	NA	NA
Bc_ctg1_484	B1	19.64858539	Bcer	H	Bcer	FALSE	Bc_Ga0417192_01_rn06	Other	NA
Bc_ctg1_4840	B1	0.793882238	Bcer	S	Bcer	FALSE	NA	NA	NA
Bc_ctg1_4841	B1	0.09923528	Bcer	NA	Bcer	FALSE	NA	NA	NA
Bc_ctg1_4842	B1	22.22870267	Bcer	E	Bcer	FALSE	NA	NA	NA
Bc_ctg1_4843	B1	17.46540924	Bcer	E	Bcer	FALSE	NA	NA	NA
Bc_ctg1_4844	B1	36.61781823	Bcer	E	Bcer	FALSE	NA	NA	NA
Bc_ctg1_4845	B1	0	Bcer	NA	Bcer	FALSE	NA	NA	NA
Bc_ctg1_4847	B1	0	Bcer	S	Bcer	FALSE	NA	NA	NA
Bc_ctg1_4848	B1	7.144940143	Bcer	L	Bcer	FALSE	NA	NA	NA
Bc_ctg1_4849	B1	1.091588077	Bcer	S	Bcer	FALSE	NA	NA	NA
Bc_ctg1_485	B1	7.541881262	Bcer	H	Bcer	FALSE	Bc_Ga0417192_01_rn06	Ancillary biosynthesis	None

Bc_ctg1_4850	B1	7.343410702	Bcer	P	Bcer	FALSE	NA	NA	NA
Bc_ctg1_4851	B1	5.060999268	Bcer	D	Bcer	FALSE	NA	NA	NA
Bc_ctg1_4852	B1	45.84669925	Bcer	P	Bcer	FALSE	NA	NA	NA
Bc_ctg1_4853	B1	57.65569754	Bcer	G	Bcer	FALSE	NA	NA	NA
Bc_ctg1_4855	B1	1.488529196	Bcer	M	Bcer	FALSE	NA	NA	NA
Bc_ctg1_4856	B1	0.595411679	Bcer	K	Bcer	FALSE	NA	NA	NA
Bc_ctg1_4857	B1	0.595411679	Bcer	NA	Bcer	FALSE	NA	NA	NA
Bc_ctg1_4858	B1	34.13693624	Bcer	M	Bcer	FALSE	NA	NA	NA
Bc_ctg1_4859	B1	0.19847056	Bcer	M	Bcer	FALSE	NA	NA	NA
Bc_ctg1_486	B1	5.457940387	Bcer	P	Bcer	FALSE	Bc_Ga0417192_01_rn06	Other	NA
Bc_ctg1_4860	B1	0.396941119	Bcer	V	Bcer	FALSE	NA	NA	NA
Bc_ctg1_4861	B1	1.488529196	Bcer	J	Bcer	FALSE	NA	NA	NA
Bc_ctg1_4862	B1	6.847234304	Bcer	S	Bcer	FALSE	Bc_Ga0417192_01_rn34	Other	NA
Bc_ctg1_4863	B1	0.297705839	Bcer	NA	Bcer	FALSE	Bc_Ga0417192_01_rn34	Other	NA
Bc_ctg1_4864	B1	2.480881994	Bcer	V	Bcer	FALSE	Bc_Ga0417192_01_rn34	Transport	SMCOG1000
Bc_ctg1_4865	B1	12.99982165	Bcer	V	Bcer	FALSE	Bc_Ga0417192_01_rn34	Transport	SMCOG1065
Bc_ctg1_4866	B1	9.427351577	Bcer	K	Bcer	FALSE	Bc_Ga0417192_01_rn34	Regulation	SMCOG1057
Bc_ctg1_4869	B1	0	Bcer	NA	Bcer	FALSE	Bc_Ga0417192_01_rn34	Other	NA
Bc_ctg1_487	B1	68.47234304	Bcer	H	Bcer	FALSE	Bc_Ga0417192_01_rn06	Ancillary biosynthesis	None
Bc_ctg1_4870	B1	4.862528708	Bcer	S	Bcer	FALSE	Bc_Ga0417192_01_rn34	Other	NA
Bc_ctg1_4871	B1	8.23652822	Bcer	I	Bcer	FALSE	Bc_Ga0417192_01_rn34	Ancillary biosynthesis	SMCOG1006
Bc_ctg1_4872	B1	7.740351821	Bcer	I	Bcer	FALSE	Bc_Ga0417192_01_rn34	Ancillary biosynthesis	SMCOG1090
Bc_ctg1_4873	B1	0	Bcer	I	Bcer	FALSE	Bc_Ga0417192_01_rn34	Other	NA
Bc_ctg1_4874	B1	7.938822381	Bcer	E	Bcer	FALSE	Bc_Ga0417192_01_rn34	Core biosynthesis	SMCOG1271
Bc_ctg1_4875	B1	4.465587589	Bcer	I	Bcer	FALSE	Bc_Ga0417192_01_rn34	Ancillary biosynthesis	SMCOG1023
Bc_ctg1_4876	B1	10.71741021	Bcer	I	Bcer	FALSE	Bc_Ga0417192_01_rn34	Ancillary biosynthesis	SMCOG1059
Bc_ctg1_4877	B1	14.38911557	Bcer	I	Bcer	FALSE	Bc_Ga0417192_01_rn34	Core biosynthesis	SMCOG1002
Bc_ctg1_4878	B1	0	Bcer	NA	Bcer	FALSE	Bc_Ga0417192_01_rn34	Other	NA
Bc_ctg1_4879	B1	0.09923528	Bcer	NA	Bcer	FALSE	Bc_Ga0417192_01_rn34	Other	NA
Bc_ctg1_488	B1	38.60252383	Bcer	C	Bcer	FALSE	Bc_Ga0417192_01_rn06	Other	NA
Bc_ctg1_4880	B1	1.786235036	Bcer	J	Bcer	FALSE	Bc_Ga0417192_01_rn34	Ancillary biosynthesis	SMCOG1111
Bc_ctg1_4881	B1	0.09923528	Bcer	NA	Bcer	FALSE	Bc_Ga0417192_01_rn34	Other	NA
Bc_ctg1_4884	B1	7.442645982	Bcer	M	Bcer	FALSE	Bc_Ga0417192_01_rn34	Other	NA
Bc_ctg1_4885	B1	16.1753506	Bcer	T	Bcer	FALSE	Bc_Ga0417192_01_rn34	Regulation	SMCOG1003
Bc_ctg1_4886	B1	13.09905693	Bcer	K	Bcer	FALSE	Bc_Ga0417192_01_rn34	Regulation	SMCOG1008
Bc_ctg1_4887	B1	2.977058393	Bcer	H	Bcer	FALSE	Bc_Ga0417192_01_rn34	Other	NA
Bc_ctg1_4888	B1	2.580117274	Bcer	H	Bcer	FALSE	Bc_Ga0417192_01_rn34	Other	NA
Bc_ctg1_4889	B1	8.23652822	Bcer	S	Bcer	FALSE	Bc_Ga0417192_01_rn34	Other	NA
Bc_ctg1_489	B1	12.70211581	Bcer	S	Bcer	FALSE	Bc_Ga0417192_01_rn06	Other	NA
Bc_ctg1_4890	B1	18.35852676	Bcer	F	Bcer	FALSE	Bc_Ga0417192_01_rn34	Other	NA
Bc_ctg1_4891	B1	35.42699487	Bcer	K	Bcer	FALSE	Bc_Ga0417192_01_rn34	Other	NA
Bc_ctg1_4892	B1	36.32011239	Bcer	NA	Bcer	FALSE	NA	NA	NA
Bc_ctg1_4893	B1	23.32029074	Bcer	E	Bcer	FALSE	NA	NA	NA
Bc_ctg1_4894	B1	0.694646958	Bcer	K	Bcer	FALSE	NA	NA	NA
Bc_ctg1_4895	B1	0.297705839	Bcer	M	Bcer	FALSE	NA	NA	NA
Bc_ctg1_4896	B1	0	Bcer	M	Bcer	FALSE	NA	NA	NA
Bc_ctg1_4897	B1	0.297705839	Bcer	M	Bcer	FALSE	NA	NA	NA
Bc_ctg1_4898	B1	8.23652822	Bcer	S	Bcer	FALSE	NA	NA	NA
Bc_ctg1_4899	B1	12.10670413	Bcer	S	Bcer	FALSE	NA	NA	NA
Bc_ctg1_49	B1	0.297705839	Bcer	S	Bcer	FALSE	NA	NA	NA
Bc_ctg1_490	B1	19.05317371	Bcer	S	Bcer	FALSE	NA	NA	NA
Bc_ctg1_4900	B1	97.64751529	Bcer	L	Bcer	FALSE	NA	NA	NA
Bc_ctg1_4901	B1	33.64075984	Bcer	K	Bcer	FALSE	NA	NA	NA
Bc_ctg1_4902	B1	2.282411435	Bcer	IQ	Bcer	TRUE	NA	NA	NA
Bc_ctg1_4903	B1	1.488529196	Bcer	K	Bcer	FALSE	NA	NA	NA
Bc_ctg1_4904	B1	12.90058637	Bcer	Q	Bcer	TRUE	NA	NA	NA
Bc_ctg1_4906	B1	1.786235036	Bcer	E	Bcer	FALSE	NA	NA	NA
Bc_ctg1_4907	B1	4.862528708	Bcer	M	Bcer	FALSE	NA	NA	NA
Bc_ctg1_4908	B1	4.06864647	Bcer	S	Bcer	FALSE	NA	NA	NA
Bc_ctg1_4909	B1	4.26711703	Bcer	K	Bcer	FALSE	Bc_Ga0417192_01_rn35	Other	NA
Bc_ctg1_491	B1	232.0120841	Bcer	C	Bcer	FALSE	NA	NA	NA
Bc_ctg1_4910	B1	56.46487418	Bcer	E	Bcer	FALSE	Bc_Ga0417192_01_rn35	Other	NA

Bc_ctg1_4911	B1	0.496176399	Bcer	S	Bcer	FALSE	Bc_Ga0417192_01_rn35	Other	NA
Bc_ctg1_4912	B1	5.259469827	Bcer	K	Bcer	FALSE	Bc_Ga0417192_01_rn35	Ancillary biosynthesis	SMCOG1019
Bc_ctg1_4913	B1	1.587764476	Bcer	NA	Bcer	FALSE	Bc_Ga0417192_01_rn35	Other	NA
Bc_ctg1_4914	B1	0.793882238	Bcer	Q	Bcer	TRUE	Bc_Ga0417192_01_rn35	Other	NA
Bc_ctg1_4915	B1	0.09923528	Bcer	S	Bcer	FALSE	Bc_Ga0417192_01_rn35	Other	NA
Bc_ctg1_4916	B1	0	Bcer	NA	Bcer	FALSE	Bc_Ga0417192_01_rn35	Other	NA
Bc_ctg1_4917	B1	3.373999512	Bcer	E	Bcer	FALSE	Bc_Ga0417192_01_rn35	Ancillary biosynthesis	SMCOG1040
Bc_ctg1_4918	B1	2.977058393	Bcer	GM	Bcer	FALSE	Bc_Ga0417192_01_rn35	Core biosynthesis	SMCOG1010
Bc_ctg1_4919	B1	1.488529196	Bcer	S	Bcer	FALSE	Bc_Ga0417192_01_rn35	Other	NA
Bc_ctg1_492	B1	0.396941119	Bcer	EG	Bcer	FALSE	NA	NA	NA
Bc_ctg1_4920	B1	7.740351821	Bcer	Q	Bcer	TRUE	Bc_Ga0417192_01_rn35	Ancillary biosynthesis	SMCOG1248
Bc_ctg1_4921	B1	24.61034938	Bcer	S	Bcer	FALSE	Bc_Ga0417192_01_rn35	Other	NA
Bc_ctg1_4922	B1	6.351057905	Bcer	Q	Bcer	TRUE	Bc_Ga0417192_01_rn35	Other	NA
Bc_ctg1_4923	B1	7.641116542	Bcer	G	Bcer	FALSE	Bc_Ga0417192_01_rn35	Other	NA
Bc_ctg1_4924	B1	0	Bcer	NA	Bcer	FALSE	Bc_Ga0417192_01_rn35	Other	NA
Bc_ctg1_4925	B1	4.664058149	Bcer	J	Bcer	FALSE	Bc_Ga0417192_01_rn35	Other	NA
Bc_ctg1_4926	B1	0	Bcer	M	Bcer	FALSE	Bc_Ga0417192_01_rn35	Other	NA
Bc_ctg1_4927	B1	12.20593941	Bcer	C	Bcer	FALSE	Bc_Ga0417192_01_rn35	Other	NA
Bc_ctg1_4928	B1	10.61817493	Bcer	T	Bcer	FALSE	Bc_Ga0417192_01_rn35	Regulation	SMCOG1008
Bc_ctg1_4929	B1	24.70958466	Bcer	T	Bcer	FALSE	Bc_Ga0417192_01_rn35	Regulation	SMCOG1003
Bc_ctg1_493	B1	0.396941119	Bcer	E	Bcer	FALSE	NA	NA	NA
Bc_ctg1_4930	B1	0	Bcer	NA	Bcer	FALSE	Bc_Ga0417192_01_rn35	Other	NA
Bc_ctg1_4931	B1	6.946469583	Bcer	NA	Bcer	FALSE	NA	NA	NA
Bc_ctg1_4932	B1	19.45011483	Bcer	S	Bcer	FALSE	NA	NA	NA
Bc_ctg1_4933	B1	8.23652822	Bcer	J	Bcer	FALSE	NA	NA	NA
Bc_ctg1_4934	B1	0	Bcer	NA	Bcer	FALSE	NA	NA	NA
Bc_ctg1_4935	B1	2.083940875	Bcer	V	Bcer	FALSE	NA	NA	NA
Bc_ctg1_4936	B1	10.02276326	Bcer	V	Bcer	FALSE	NA	NA	NA
Bc_ctg1_4937	B1	3.572470071	Bcer	S	Bcer	FALSE	NA	NA	NA
Bc_ctg1_4938	B1	306.5377792	Bcer	NA	Bcer	FALSE	NA	NA	NA
Bc_ctg1_4939	B1	0.595411679	Bcer	D	Bcer	FALSE	NA	NA	NA
Bc_ctg1_494	B1	0.19847056	Bcer	S	Bcer	FALSE	NA	NA	NA
Bc_ctg1_4940	B1	3.96941119	Bcer	E	Bcer	FALSE	NA	NA	NA
Bc_ctg1_4941	B1	13.69446861	Bcer	K	Bcer	FALSE	Bc_Ga0417192_01_rn36	Ancillary biosynthesis	SMCOG1019
Bc_ctg1_4942	B1	12.20593941	Bcer	F	Bcer	FALSE	Bc_Ga0417192_01_rn36	Ancillary biosynthesis	None
Bc_ctg1_4943	B1	1.786235036	Bcer	K	Bcer	FALSE	Bc_Ga0417192_01_rn36	Other	NA
Bc_ctg1_4944	B1	147.0666846	Bcer	S	Bcer	FALSE	Bc_Ga0417192_01_rn36	Other	NA
Bc_ctg1_4945	B1	0.496176399	Bcer	NA	Bcer	FALSE	Bc_Ga0417192_01_rn36	Core biosynthesis	None
Bc_ctg1_4946	B1	7.641116542	Bcer	NA	Bcer	FALSE	Bc_Ga0417192_01_rn36	Other	NA
Bc_ctg1_4947	B1	0.297705839	Bcer	S	Bcer	FALSE	Bc_Ga0417192_01_rn36	Other	NA
Bc_ctg1_4948	B1	21.63329099	Bcer	L	Bcer	FALSE	Bc_Ga0417192_01_rn36	Other	SMCOG1077
Bc_ctg1_4949	B1	2.282411435	Bcer	NA	Bcer	FALSE	Bc_Ga0417192_01_rn36	Other	NA
Bc_ctg1_495	B1	117.0976301	Bcer	E	Bcer	FALSE	NA	NA	NA
Bc_ctg1_4950	B1	4.564822869	Bcer	NA	Bcer	FALSE	NA	NA	NA
Bc_ctg1_4951	B1	0	Bcer	NA	Bcer	FALSE	NA	NA	NA
Bc_ctg1_4953	B1	12.99982165	Bcer	K	Bcer	FALSE	NA	NA	NA
Bc_ctg1_4954	B1	0	Bcer	K	Bcer	FALSE	NA	NA	NA
Bc_ctg1_4955	B1	0.19847056	Bcer	NA	Bcer	FALSE	NA	NA	NA
Bc_ctg1_4956	B1	0	Bcer	NA	Bcer	FALSE	NA	NA	NA
Bc_ctg1_4957	B1	1.190823357	Bcer	L	Bcer	FALSE	NA	NA	NA
Bc_ctg1_4958	B1	0	Bcer	K	Bcer	FALSE	NA	NA	NA
Bc_ctg1_4959	B1	0.09923528	Bcer	NA	Bcer	FALSE	NA	NA	NA
Bc_ctg1_4961	B1	0	Bcer	NA	Bcer	FALSE	NA	NA	NA
Bc_ctg1_4962	B1	0.396941119	Bcer	S	Bcer	FALSE	NA	NA	NA
Bc_ctg1_4963	B1	9.725057417	Bcer	NA	Bcer	FALSE	NA	NA	NA
Bc_ctg1_4964	B1	0	Bcer	NA	Bcer	FALSE	NA	NA	NA
Bc_ctg1_4965	B1	2.282411435	Bcer	K	Bcer	FALSE	NA	NA	NA
Bc_ctg1_4966	B1	6.747999024	Bcer	NA	Bcer	FALSE	NA	NA	NA
Bc_ctg1_4967	B1	0	Bcer	NA	Bcer	FALSE	NA	NA	NA
Bc_ctg1_497	B1	5.755646226	Bcer	L	Bcer	FALSE	NA	NA	NA
Bc_ctg1_4970	B1	2.778587833	Bcer	S	Bcer	FALSE	NA	NA	NA
Bc_ctg1_4971	B1	6.351057905	Bcer	K	Bcer	FALSE	NA	NA	NA

Bc_ctg1_4972	B1	292.4463695	Bcer	L	Bcer	FALSE	NA	NA	NA
Bc_ctg1_4973	B1	96.65516249	Bcer	NA	Bcer	FALSE	NA	NA	NA
Bc_ctg1_4974	B1	0.09923528	Bcer	NA	Bcer	FALSE	NA	NA	NA
Bc_ctg1_4976	B1	0	Bcer	NA	Bcer	FALSE	NA	NA	NA
Bc_ctg1_4977	B1	0	Bcer	S	Bcer	FALSE	NA	NA	NA
Bc_ctg1_4978	B1	0	Bcer	S	Bcer	FALSE	NA	NA	NA
Bc_ctg1_4979	B1	0.595411679	Bcer	S	Bcer	FALSE	NA	NA	NA
Bc_ctg1_498	B1	0.19847056	Bcer	S	Bcer	FALSE	NA	NA	NA
Bc_ctg1_4980	B1	0	Bcer	OU	Bcer	FALSE	NA	NA	NA
Bc_ctg1_4981	B1	0.793882238	Bcer	S	Bcer	FALSE	NA	NA	NA
Bc_ctg1_4982	B1	0	Bcer	NA	Bcer	FALSE	NA	NA	NA
Bc_ctg1_4983	B1	0	Bcer	NA	Bcer	FALSE	NA	NA	NA
Bc_ctg1_4984	B1	0	Bcer	NA	Bcer	FALSE	NA	NA	NA
Bc_ctg1_4985	B1	0	Bcer	NA	Bcer	FALSE	NA	NA	NA
Bc_ctg1_4986	B1	0	Bcer	NA	Bcer	FALSE	NA	NA	NA
Bc_ctg1_4987	B1	0	Bcer	NA	Bcer	FALSE	NA	NA	NA
Bc_ctg1_4988	B1	0	Bcer	E	Bcer	FALSE	NA	NA	NA
Bc_ctg1_4989	B1	0.496176399	Bcer	S	Bcer	FALSE	NA	NA	NA
Bc_ctg1_499	B1	5.358705107	Bcer	E	Bcer	FALSE	NA	NA	NA
Bc_ctg1_4990	B1	0.19847056	Bcer	M	Bcer	FALSE	NA	NA	NA
Bc_ctg1_4993	B1	0	Bcer	S	Bcer	FALSE	NA	NA	NA
Bc_ctg1_4994	B1	0.694646958	Bcer	Q	Bcer	TRUE	NA	NA	NA
Bc_ctg1_4995	B1	4.564822869	Bcer	NA	Bcer	FALSE	NA	NA	NA
Bc_ctg1_4996	B1	40.38875886	Bcer	T	Bcer	FALSE	NA	NA	NA
Bc_ctg1_4997	B1	2.083940875	Bcer	S	Bcer	FALSE	NA	NA	NA
Bc_ctg1_4998	B1	69.16698999	Bcer	S	Bcer	FALSE	NA	NA	NA
Bc_ctg1_4999	B1	7.244175423	Bcer	S	Bcer	FALSE	NA	NA	NA
Bc_ctg1_50	B1	0.694646958	Bcer	S	Bcer	FALSE	NA	NA	NA
Bc_ctg1_5000	B1	3.473234792	Bcer	NA	Bcer	FALSE	NA	NA	NA
Bc_ctg1_5001	B1	0.09923528	Bcer	NA	Bcer	FALSE	NA	NA	NA
Bc_ctg1_5002	B1	17.6638798	Bcer	S	Bcer	FALSE	NA	NA	NA
Bc_ctg1_5003	B1	0.19847056	Bcer	NA	Bcer	FALSE	NA	NA	NA
Bc_ctg1_5004	B1	262.0803738	Bcer	C	Bcer	FALSE	NA	NA	NA
Bc_ctg1_5005	B1	20.93864403	Bcer	EGP	Bcer	FALSE	NA	NA	NA
Bc_ctg1_5006	B1	11.51129245	Bcer	S	Bcer	FALSE	NA	NA	NA
Bc_ctg1_5007	B1	0	Bcer	S	Bcer	FALSE	NA	NA	NA
Bc_ctg1_5008	B1	1.587764476	Bcer	K	Bcer	FALSE	NA	NA	NA
Bc_ctg1_5009	B1	5.854881506	Bcer	Q	Bcer	TRUE	NA	NA	NA
Bc_ctg1_501	B1	0	Bcer	NA	Bcer	FALSE	NA	NA	NA
Bc_ctg1_5010	B1	1.290058637	Bcer	C	Bcer	FALSE	NA	NA	NA
Bc_ctg1_5011	B1	37.31246519	Bcer	S	Bcer	FALSE	NA	NA	NA
Bc_ctg1_5012	B1	49.0222282	Bcer	T	Bcer	FALSE	NA	NA	NA
Bc_ctg1_5013	B1	94.07504521	Bcer	M	Bcer	FALSE	NA	NA	NA
Bc_ctg1_5014	B1	4.564822869	Bcer	U	Bcer	FALSE	NA	NA	NA
Bc_ctg1_5015	B1	4.366352309	Bcer	CG	Bcer	FALSE	NA	NA	NA
Bc_ctg1_5016	B1	2.282411435	Bcer	M	Bcer	FALSE	NA	NA	NA
Bc_ctg1_5017	B1	0.992352798	Bcer	S	Bcer	FALSE	NA	NA	NA
Bc_ctg1_5018	B1	2.778587833	Bcer	P	Bcer	FALSE	NA	NA	NA
Bc_ctg1_5019	B1	2.282411435	Bcer	P	Bcer	FALSE	NA	NA	NA
Bc_ctg1_502	B1	0.396941119	Bcer	Q	Bcer	TRUE	NA	NA	NA
Bc_ctg1_5021	B1	37.90787687	Bcer	T	Bcer	FALSE	NA	NA	NA
Bc_ctg1_5023	B1	6.053352065	Bcer	NA	Bcer	FALSE	NA	NA	NA
Bc_ctg1_5024	B1	0.09923528	Bcer	NA	Bcer	FALSE	NA	NA	NA
Bc_ctg1_5025	B1	0.297705839	Bcer	NA	Bcer	FALSE	NA	NA	NA
Bc_ctg1_5026	B1	0	Bcer	NA	Bcer	FALSE	NA	NA	NA
Bc_ctg1_5028	B1	0.19847056	Bcer	F	Bcer	FALSE	NA	NA	NA
Bc_ctg1_503	B1	5.259469827	Bcer	E	Bcer	FALSE	NA	NA	NA
Bc_ctg1_5033	B1	2.381646714	Bcer	D	Bcer	FALSE	NA	NA	NA
Bc_ctg1_5034	B1	0.19847056	Bcer	NA	Bcer	FALSE	NA	NA	NA
Bc_ctg1_5035	B1	0.893117518	Bcer	NA	Bcer	FALSE	NA	NA	NA
Bc_ctg1_5036	B1	17.06846812	Bcer	F	Bcer	FALSE	NA	NA	NA
Bc_ctg1_5037	B1	0.19847056	Bcer	NA	Bcer	FALSE	NA	NA	NA

Bc_ctg1_5038	B1	15.38146836	Bcer	KLT	Bcer	FALSE	NA	NA	NA
Bc_ctg1_5039	B1	1.885470315	Bcer	S	Bcer	FALSE	NA	NA	NA
Bc_ctg1_504	B1	0.694646958	Bcer	L	Bcer	FALSE	NA	NA	NA
Bc_ctg1_5040	B1	7.839587101	Bcer	C	Bcer	FALSE	NA	NA	NA
Bc_ctg1_5041	B1	125.5326289	Bcer	I	Bcer	FALSE	NA	NA	NA
Bc_ctg1_5042	B1	13.49599805	Bcer	M	Bcer	FALSE	NA	NA	NA
Bc_ctg1_5043	B1	2.679352554	Bcer	EGP	Bcer	FALSE	NA	NA	NA
Bc_ctg1_5044	B1	4.564822869	Bcer	V	Bcer	FALSE	NA	NA	NA
Bc_ctg1_5045	B1	3.175528952	Bcer	V	Bcer	FALSE	NA	NA	NA
Bc_ctg1_5046	B1	1.686999756	Bcer	S	Bcer	FALSE	NA	NA	NA
Bc_ctg1_5047	B1	0.793882238	Bcer	S	Bcer	FALSE	NA	NA	NA
Bc_ctg1_5048	B1	49.81611044	Bcer	S	Bcer	FALSE	NA	NA	NA
Bc_ctg1_5049	B1	6.946469583	Bcer	K	Bcer	FALSE	NA	NA	NA
Bc_ctg1_505	B1	0	Bcer	L	Bcer	FALSE	NA	NA	NA
Bc_ctg1_5050	B1	6.648763744	Bcer	NA	Bcer	FALSE	NA	NA	NA
Bc_ctg1_5051	B1	65.49528464	Bcer	S	Bcer	FALSE	NA	NA	NA
Bc_ctg1_5052	B1	64.60216712	Bcer	KQ	Bcer	TRUE	NA	NA	NA
Bc_ctg1_5053	B1	1.389293917	Bcer	EGP	Bcer	FALSE	NA	NA	NA
Bc_ctg1_5054	B1	2.381646714	Bcer	K	Bcer	FALSE	NA	NA	NA
Bc_ctg1_5055	B1	1.885470315	Bcer	M	Bcer	FALSE	NA	NA	NA
Bc_ctg1_5056	B1	10.02276326	Bcer	NA	Bcer	FALSE	NA	NA	NA
Bc_ctg1_5058	B1	5.954116786	Bcer	K	Bcer	FALSE	NA	NA	NA
Bc_ctg1_5059	B1	9.030410458	Bcer	C	Bcer	FALSE	NA	NA	NA
Bc_ctg1_506	B1	4.366352309	Bcer	E	Bcer	FALSE	NA	NA	NA
Bc_ctg1_5060	B1	2.381646714	Bcer	S	Bcer	FALSE	NA	NA	NA
Bc_ctg1_5061	B1	0	Bcer	NA	Bcer	FALSE	NA	NA	NA
Bc_ctg1_5062	B1	7.938822381	Bcer	FG	Bcer	FALSE	NA	NA	NA
Bc_ctg1_5063	B1	7.541881262	Bcer	K	Bcer	FALSE	NA	NA	NA
Bc_ctg1_5064	B1	0.297705839	Bcer	EG	Bcer	FALSE	Bc_Ga0417192_01_rn37	Other	NA
Bc_ctg1_5065	B1	1.686999756	Bcer	G	Bcer	FALSE	Bc_Ga0417192_01_rn37	Other	NA
Bc_ctg1_5066	B1	0	Bcer	F	Bcer	FALSE	Bc_Ga0417192_01_rn37	Other	NA
Bc_ctg1_5067	B1	15.48070364	Bcer	K	Bcer	FALSE	Bc_Ga0417192_01_rn37	Other	NA
Bc_ctg1_5068	B1	1.587764476	Bcer	S	Bcer	FALSE	Bc_Ga0417192_01_rn37	Other	NA
Bc_ctg1_5069	B1	10.71741021	Bcer	F	Bcer	FALSE	Bc_Ga0417192_01_rn37	Other	NA
Bc_ctg1_507	B1	988.9787981	Bcer	E	Bcer	FALSE	NA	NA	NA
Bc_ctg1_5070	B1	0	Bcer	NA	Bcer	FALSE	Bc_Ga0417192_01_rn37	Core biosynthesis	None
Bc_ctg1_5071	B1	1.488529196	Bcer	V	Bcer	FALSE	Bc_Ga0417192_01_rn37	Other	NA
Bc_ctg1_5072	B1	5.457940387	Bcer	V	Bcer	FALSE	Bc_Ga0417192_01_rn37	Other	NA
Bc_ctg1_5073	B1	3.870175911	Bcer	V	Bcer	FALSE	Bc_Ga0417192_01_rn37	Other	NA
Bc_ctg1_5075	B1	2.083940875	Bcer	P	Bcer	FALSE	Bc_Ga0417192_01_rn37	Transport	SMCOG1137
Bc_ctg1_5076	B1	0.893117518	Bcer	P	Bcer	FALSE	Bc_Ga0417192_01_rn37	Other	NA
Bc_ctg1_5077	B1	1.587764476	Bcer	K	Bcer	FALSE	Bc_Ga0417192_01_rn37	Other	NA
Bc_ctg1_5078	B1	0.793882238	Bcer	F	Bcer	FALSE	Bc_Ga0417192_01_rn37	Other	NA
Bc_ctg1_5079	B1	6.847234304	Bcer	L	Bcer	FALSE	Bc_Ga0417192_01_rn37	Other	NA
Bc_ctg1_508	B1	3.870175911	Bcer	K	Bcer	FALSE	NA	NA	NA
Bc_ctg1_5080	B1	11.01511605	Bcer	M	Bcer	FALSE	NA	NA	NA
Bc_ctg1_5081	B1	12.30517469	Bcer	S	Bcer	FALSE	NA	NA	NA
Bc_ctg1_5082	B1	24.5111141	Bcer	S	Bcer	FALSE	NA	NA	NA
Bc_ctg1_5083	B1	0.595411679	Bcer	L	Bcer	FALSE	NA	NA	NA
Bc_ctg1_5084	B1	7.839587101	Bcer	J	Bcer	FALSE	NA	NA	NA
Bc_ctg1_5085	B1	42.1749939	Bcer	S	Bcer	FALSE	NA	NA	NA
Bc_ctg1_5086	B1	18.45776204	Bcer	P	Bcer	FALSE	NA	NA	NA
Bc_ctg1_5087	B1	12.20593941	Bcer	NA	Bcer	FALSE	NA	NA	NA
Bc_ctg1_5088	B1	3.473234792	Bcer	S	Bcer	FALSE	NA	NA	NA
Bc_ctg1_5089	B1	5.656410946	Bcer	S	Bcer	FALSE	NA	NA	NA
Bc_ctg1_509	B1	3.671705351	Bcer	NA	Bcer	FALSE	NA	NA	NA
Bc_ctg1_5090	B1	56.56410946	Bcer	S	Bcer	FALSE	NA	NA	NA
Bc_ctg1_5091	B1	11.31282189	Bcer	K	Bcer	FALSE	NA	NA	NA
Bc_ctg1_5092	B1	8.038057661	Bcer	S	Bcer	FALSE	NA	NA	NA
Bc_ctg1_5093	B1	13.39676277	Bcer	J	Bcer	FALSE	NA	NA	NA
Bc_ctg1_5094	B1	17.56464452	Bcer	CH	Bcer	FALSE	NA	NA	NA
Bc_ctg1_5095	B1	0	Bcer	V	Bcer	FALSE	NA	NA	NA

Bc_ctg1_5096	B1	0.09923528	Bcer	NA	Bcer	FALSE	NA	NA	NA
Bc_ctg1_5097	B1	98.83833864	Bcer	L	Bcer	FALSE	NA	NA	NA
Bc_ctg1_5098	B1	10.81664549	Bcer	K	Bcer	FALSE	NA	NA	NA
Bc_ctg1_5099	B1	0.19847056	Bcer	NA	Bcer	FALSE	NA	NA	NA
Bc_ctg1_51	B1	0.496176399	Bcer	S	Bcer	FALSE	NA	NA	NA
Bc_ctg1_510	B1	0.893117518	Bcer	K	Bcer	FALSE	NA	NA	NA
Bc_ctg1_5100	B1	3.274764232	Bcer	G	Bcer	FALSE	NA	NA	NA
Bc_ctg1_5101	B1	8.3357635	Bcer	NA	Bcer	FALSE	NA	NA	NA
Bc_ctg1_5102	B1	0.396941119	Bcer	NA	Bcer	FALSE	NA	NA	NA
Bc_ctg1_5103	B1	3.373999512	Bcer	V	Bcer	FALSE	Bc_Ga0417192_01_rn38	Other	NA
Bc_ctg1_5104	B1	2.083940875	Bcer	CG	Bcer	FALSE	Bc_Ga0417192_01_rn38	Ancillary biosynthesis	SMCOG1062
Bc_ctg1_5105	B1	5.160234548	Bcer	KLT	Bcer	FALSE	Bc_Ga0417192_01_rn38	Other	NA
Bc_ctg1_5106	B1	1.290058637	Bcer	L	Bcer	FALSE	Bc_Ga0417192_01_rn38	Other	NA
Bc_ctg1_5107	B1	38.80099439	Bcer	E	Bcer	FALSE	Bc_Ga0417192_01_rn38	Ancillary biosynthesis	SMCOG1103
Bc_ctg1_5108	B1	11.80899829	Bcer	S	Bcer	FALSE	Bc_Ga0417192_01_rn38	Other	NA
Bc_ctg1_5109	B1	0.09923528	Bcer	NA	Bcer	FALSE	Bc_Ga0417192_01_rn38	Other	NA
Bc_ctg1_511	B1	28.77823113	Bcer	S	Bcer	FALSE	NA	NA	NA
Bc_ctg1_5110	B1	3.175528952	Bcer	Q	Bcer	TRUE	Bc_Ga0417192_01_rn38	Other	NA
Bc_ctg1_5111	B1	3.572470071	Bcer	S	Bcer	FALSE	Bc_Ga0417192_01_rn38	Other	NA
Bc_ctg1_5112	B1	2.877823113	Bcer	K	Bcer	FALSE	Bc_Ga0417192_01_rn38	Other	NA
Bc_ctg1_5113	B1	7.343410702	Bcer	S	Bcer	FALSE	Bc_Ga0417192_01_rn38	Ancillary biosynthesis	SMCOG1115
Bc_ctg1_5114	B1	6.847234304	Bcer	T	Bcer	FALSE	Bc_Ga0417192_01_rn38	Regulation	SMCOG1008
Bc_ctg1_5115	B1	25.70193746	Bcer	T	Bcer	FALSE	Bc_Ga0417192_01_rn38	Regulation	SMCOG1003
Bc_ctg1_5116	B1	0	Bcer	S	Bcer	FALSE	Bc_Ga0417192_01_rn38	Other	NA
Bc_ctg1_5117	B1	1.290058637	Bcer	NA	Bcer	FALSE	Bc_Ga0417192_01_rn38	Other	NA
Bc_ctg1_5118	B1	2.083940875	Bcer	V	Bcer	FALSE	Bc_Ga0417192_01_rn38	Transport	SMCOG1000
Bc_ctg1_5119	B1	1.686999756	Bcer	V	Bcer	FALSE	Bc_Ga0417192_01_rn38	Other	NA
Bc_ctg1_512	B1	20.74017347	Bcer	V	Bcer	FALSE	NA	NA	NA
Bc_ctg1_5120	B1	4.961763988	Bcer	S	Bcer	FALSE	Bc_Ga0417192_01_rn38	Other	NA
Bc_ctg1_5121	B1	17.76311508	Bcer	I	Bcer	FALSE	Bc_Ga0417192_01_rn38	Ancillary biosynthesis	SMCOG1066
Bc_ctg1_5122	B1	3.96941119	Bcer	E	Bcer	FALSE	Bc_Ga0417192_01_rn38	Ancillary biosynthesis	SMCOG1180
Bc_ctg1_5123	B1	12.40440997	Bcer	Q	Bcer	TRUE	Bc_Ga0417192_01_rn38	Core biosynthesis	SMCOG1002
Bc_ctg1_5124	B1	0.992352798	Bcer	C	Bcer	FALSE	Bc_Ga0417192_01_rn38	Ancillary biosynthesis	None
Bc_ctg1_5125	B1	18.95393843	Bcer	G	Bcer	FALSE	Bc_Ga0417192_01_rn38	Core biosynthesis	SMCOG1297
Bc_ctg1_5126	B1	1753.785099	Bcer	E	Bcer	FALSE	Bc_Ga0417192_01_rn38	Other	NA
Bc_ctg1_5127	B1	29.27440753	Bcer	F	Bcer	FALSE	Bc_Ga0417192_01_rn38	Ancillary biosynthesis	SMCOG1283
Bc_ctg1_5128	B1	10.12199854	Bcer	S	Bcer	FALSE	Bc_Ga0417192_01_rn38	Other	NA
Bc_ctg1_5129	B1	2.282411435	Bcer	L	Bcer	FALSE	Bc_Ga0417192_01_rn38	Other	NA
Bc_ctg1_513	B1	20.93864403	Bcer	K	Bcer	FALSE	NA	NA	NA
Bc_ctg1_5130	B1	0.19847056	Bcer	E	Bcer	FALSE	Bc_Ga0417192_01_rn38	Other	NA
Bc_ctg1_5131	B1	148.4559785	Bcer	U	Bcer	FALSE	Bc_Ga0417192_01_rn38	Other	NA
Bc_ctg1_5132	B1	1.488529196	Bcer	EG	Bcer	FALSE	Bc_Ga0417192_01_rn38	Transport	SMCOG1166
Bc_ctg1_5133	B1	2.282411435	Bcer	K	Bcer	FALSE	Bc_Ga0417192_01_rn38	Ancillary biosynthesis	SMCOG1019
Bc_ctg1_5134	B1	8.23652822	Bcer	C	Bcer	FALSE	Bc_Ga0417192_01_rn38	Ancillary biosynthesis	SMCOG1028
Bc_ctg1_5135	B1	0.992352798	Bcer	I	Bcer	FALSE	Bc_Ga0417192_01_rn38	Ancillary biosynthesis	SMCOG1036
Bc_ctg1_5136	B1	0	Bcer	NA	Bcer	FALSE	Bc_Ga0417192_01_rn38	Other	NA
Bc_ctg1_5137	B1	0.396941119	Bcer	NU	Bcer	FALSE	Bc_Ga0417192_01_rn38	Other	NA
Bc_ctg1_5138	B1	3.870175911	Bcer	NA	Bcer	FALSE	Bc_Ga0417192_01_rn38	Other	NA
Bc_ctg1_5139	B1	7.938822381	Bcer	S	Bcer	FALSE	Bc_Ga0417192_01_rn38	Other	NA
Bc_ctg1_514	B1	8.3357635	Bcer	K	Bcer	FALSE	NA	NA	NA
Bc_ctg1_5140	B1	11.31282189	Bcer	S	Bcer	FALSE	NA	NA	NA
Bc_ctg1_5141	B1	16.57229172	Bcer	J	Bcer	FALSE	NA	NA	NA
Bc_ctg1_5142	B1	4.564822869	Bcer	NA	Bcer	FALSE	NA	NA	NA
Bc_ctg1_5143	B1	0	Bcer	S	Bcer	FALSE	NA	NA	NA
Bc_ctg1_5144	B1	0.19847056	Bcer	H	Bcer	FALSE	NA	NA	NA
Bc_ctg1_5145	B1	0.793882238	Bcer	M	Bcer	FALSE	NA	NA	NA
Bc_ctg1_5147	B1	2.381646714	Bcer	C	Bcer	FALSE	NA	NA	NA
Bc_ctg1_5148	B1	8.831939899	Bcer	V	Bcer	FALSE	NA	NA	NA
Bc_ctg1_5149	B1	38.30481799	Bcer	E	Bcer	FALSE	NA	NA	NA
Bc_ctg1_515	B1	1.488529196	Bcer	S	Bcer	FALSE	NA	NA	NA
Bc_ctg1_5150	B1	20.34323235	Bcer	S	Bcer	FALSE	NA	NA	NA
Bc_ctg1_5151	B1	37.01475935	Bcer	E	Bcer	FALSE	NA	NA	NA

Bc_ctg1_5152	B1	9.228881018	Bcer	K	Bcer	FALSE	NA	NA	NA
Bc_ctg1_5153	B1	0	Bcer	F	Bcer	FALSE	NA	NA	NA
Bc_ctg1_5154	B1	0.19847056	Bcer	Q	Bcer	TRUE	NA	NA	NA
Bc_ctg1_5155	B1	0.793882238	Bcer	Q	Bcer	TRUE	NA	NA	NA
Bc_ctg1_5156	B1	3.175528952	Bcer	G	Bcer	FALSE	NA	NA	NA
Bc_ctg1_5157	B1	0	Bcer	S	Bcer	FALSE	NA	NA	NA
Bc_ctg1_5158	B1	23.0225849	Bcer	KQ	Bcer	TRUE	NA	NA	NA
Bc_ctg1_5159	B1	0.09923528	Bcer	NA	Bcer	FALSE	NA	NA	NA
Bc_ctg1_516	B1	23.91570242	Bcer	S	Bcer	FALSE	NA	NA	NA
Bc_ctg1_5160	B1	0.694646958	Bcer	F	Bcer	FALSE	NA	NA	NA
Bc_ctg1_5161	B1	3.175528952	Bcer	K	Bcer	FALSE	NA	NA	NA
Bc_ctg1_5162	B1	7.541881262	Bcer	K	Bcer	FALSE	NA	NA	NA
Bc_ctg1_5163	B1	2.977058393	Bcer	NA	Bcer	FALSE	NA	NA	NA
Bc_ctg1_5164	B1	0.396941119	Bcer	Q	Bcer	TRUE	NA	NA	NA
Bc_ctg1_5165	B1	16.57229172	Bcer	K	Bcer	FALSE	NA	NA	NA
Bc_ctg1_5166	B1	4.26711703	Bcer	S	Bcer	FALSE	NA	NA	NA
Bc_ctg1_5167	B1	0.496176399	Bcer	NA	Bcer	FALSE	NA	NA	NA
Bc_ctg1_5168	B1	0.496176399	Bcer	C	Bcer	FALSE	NA	NA	NA
Bc_ctg1_5169	B1	0.09923528	Bcer	C	Bcer	FALSE	NA	NA	NA
Bc_ctg1_517	B1	326.4840704	Bcer	L	Bcer	FALSE	NA	NA	NA
Bc_ctg1_5170	B1	0.09923528	Bcer	C	Bcer	FALSE	NA	NA	NA
Bc_ctg1_5171	B1	0.396941119	Bcer	C	Bcer	FALSE	NA	NA	NA
Bc_ctg1_5173	B1	16.07611532	Bcer	S	Bcer	FALSE	NA	NA	NA
Bc_ctg1_5174	B1	6.351057905	Bcer	S	Bcer	FALSE	NA	NA	NA
Bc_ctg1_5175	B1	5.358705107	Bcer	S	Bcer	FALSE	NA	NA	NA
Bc_ctg1_5176	B1	0.396941119	Bcer	S	Bcer	FALSE	NA	NA	NA
Bc_ctg1_5177	B1	1.885470315	Bcer	L	Bcer	FALSE	NA	NA	NA
Bc_ctg1_5178	B1	0.297705839	Bcer	S	Bcer	FALSE	NA	NA	NA
Bc_ctg1_5179	B1	1.389293917	Bcer	T	Bcer	FALSE	NA	NA	NA
Bc_ctg1_518	B1	157.5856243	Bcer	L	Bcer	FALSE	NA	NA	NA
Bc_ctg1_5180	B1	1.885470315	Bcer	FG	Bcer	FALSE	NA	NA	NA
Bc_ctg1_5182	B1	0.595411679	Bcer	P	Bcer	FALSE	NA	NA	NA
Bc_ctg1_5183	B1	0.09923528	Bcer	E	Bcer	FALSE	NA	NA	NA
Bc_ctg1_5184	B1	0.09923528	Bcer	E	Bcer	FALSE	NA	NA	NA
Bc_ctg1_5185	B1	3.572470071	Bcer	OU	Bcer	FALSE	NA	NA	NA
Bc_ctg1_5186	B1	7.244175423	Bcer	K	Bcer	FALSE	NA	NA	NA
Bc_ctg1_5187	B1	0	Bcer	C	Bcer	FALSE	NA	NA	NA
Bc_ctg1_5188	B1	0.396941119	Bcer	G	Bcer	FALSE	NA	NA	NA
Bc_ctg1_5189	B1	19.45011483	Bcer	S	Bcer	FALSE	NA	NA	NA
Bc_ctg1_519	B1	51.70158076	Bcer	S	Bcer	FALSE	NA	NA	NA
Bc_ctg1_5190	B1	484.7643416	Bcer	S	Bcer	FALSE	NA	NA	NA
Bc_ctg1_5191	B1	8.23652822	Bcer	CG	Bcer	FALSE	NA	NA	NA
Bc_ctg1_5192	B1	2.381646714	Bcer	S	Bcer	FALSE	NA	NA	NA
Bc_ctg1_5193	B1	1.488529196	Bcer	F	Bcer	FALSE	NA	NA	NA
Bc_ctg1_5194	B1	4.06864647	Bcer	T	Bcer	FALSE	NA	NA	NA
Bc_ctg1_5195	B1	10.3204691	Bcer	Q	Bcer	TRUE	NA	NA	NA
Bc_ctg1_5196	B1	2.679352554	Bcer	S	Bcer	FALSE	NA	NA	NA
Bc_ctg1_5197	B1	4.465587589	Bcer	K	Bcer	FALSE	NA	NA	NA
Bc_ctg1_5198	B1	5.854881506	Bcer	K	Bcer	FALSE	NA	NA	NA
Bc_ctg1_5199	B1	2.877823113	Bcer	NA	Bcer	FALSE	NA	NA	NA
Bc_ctg1_520	B1	472.8561081	Bcer	O	Bcer	FALSE	NA	NA	NA
Bc_ctg1_5200	B1	10.3204691	Bcer	M	Bcer	FALSE	NA	NA	NA
Bc_ctg1_5201	B1	2.183176155	Bcer	C	Bcer	FALSE	NA	NA	NA
Bc_ctg1_5202	B1	0.297705839	Bcer	K	Bcer	FALSE	NA	NA	NA
Bc_ctg1_5203	B1	5.854881506	Bcer	S	Bcer	FALSE	NA	NA	NA
Bc_ctg1_5204	B1	8.23652822	Bcer	NA	Bcer	FALSE	NA	NA	NA
Bc_ctg1_5205	B1	25.60270218	Bcer	L	Bcer	FALSE	NA	NA	NA
Bc_ctg1_5206	B1	0.09923528	Bcer	S	Bcer	FALSE	NA	NA	NA
Bc_ctg1_5207	B1	11.80899829	Bcer	S	Bcer	FALSE	NA	NA	NA
Bc_ctg1_5208	B1	67.38075496	Bcer	L	Bcer	FALSE	NA	NA	NA
Bc_ctg1_521	B1	42.07575862	Bcer	T	Bcer	FALSE	NA	NA	NA
Bc_ctg1_5210	B1	0	Bcer	M	Bcer	FALSE	NA	NA	NA

Bc_ctg1_5211	B1	2.282411435	Bcer	NA	Bcer	FALSE	NA	NA	NA
Bc_ctg1_5212	B1	3.274764232	Bcer	NA	Bcer	FALSE	NA	NA	NA
Bc_ctg1_5213	B1	0	Bcer	NA	Bcer	FALSE	NA	NA	NA
Bc_ctg1_5214	B1	1.984705595	Bcer	S	Bcer	FALSE	NA	NA	NA
Bc_ctg1_5215	B1	0.396941119	Bcer	S	Bcer	FALSE	NA	NA	NA
Bc_ctg1_5216	B1	4.16788175	Bcer	E	Bcer	FALSE	NA	NA	NA
Bc_ctg1_5217	B1	420.7575862	Bcer	C	Bcer	FALSE	NA	NA	NA
Bc_ctg1_5218	B1	24.21340826	Bcer	S	Bcer	FALSE	NA	NA	NA
Bc_ctg1_5219	B1	16.07611532	Bcer	K	Bcer	FALSE	NA	NA	NA
Bc_ctg1_522	B1	3.96941119	Bcer	C	Bcer	FALSE	NA	NA	NA
Bc_ctg1_5220	B1	10.41970437	Bcer	H	Bcer	FALSE	NA	NA	NA
Bc_ctg1_5221	B1	0.19847056	Bcer	S	Bcer	FALSE	NA	NA	NA
Bc_ctg1_5222	B1	1.290058637	Bcer	E	Bcer	FALSE	NA	NA	NA
Bc_ctg1_5223	B1	2.083940875	Bcer	C	Bcer	FALSE	NA	NA	NA
Bc_ctg1_5224	B1	1.091588077	Bcer	E	Bcer	FALSE	NA	NA	NA
Bc_ctg1_5225	B1	0.496176399	Bcer	E	Bcer	FALSE	NA	NA	NA
Bc_ctg1_5226	B1	0.09923528	Bcer	E	Bcer	FALSE	NA	NA	NA
Bc_ctg1_5227	B1	0.992352798	Bcer	E	Bcer	FALSE	NA	NA	NA
Bc_ctg1_5228	B1	9.228881018	Bcer	KT	Bcer	FALSE	NA	NA	NA
Bc_ctg1_5229	B1	0.396941119	Bcer	C	Bcer	FALSE	NA	NA	NA
Bc_ctg1_523	B1	71.94557783	Bcer	F	Bcer	FALSE	NA	NA	NA
Bc_ctg1_5230	B1	1.786235036	Bcer	C	Bcer	FALSE	NA	NA	NA
Bc_ctg1_5231	B1	4.862528708	Bcer	C	Bcer	FALSE	NA	NA	NA
Bc_ctg1_5232	B1	11.31282189	Bcer	S	Bcer	FALSE	NA	NA	NA
Bc_ctg1_5233	B1	57.95340338	Bcer	S	Bcer	FALSE	NA	NA	NA
Bc_ctg1_5234	B1	56.96105058	Bcer	NA	Bcer	FALSE	NA	NA	NA
Bc_ctg1_5236	B1	74.92263622	Bcer	S	Bcer	FALSE	NA	NA	NA
Bc_ctg1_5238	B1	14.19064501	Bcer	S	Bcer	FALSE	NA	NA	NA
Bc_ctg1_5239	B1	0	Bcer	NA	Bcer	FALSE	NA	NA	NA
Bc_ctg1_524	B1	79.48745909	Bcer	I	Bcer	FALSE	NA	NA	NA
Bc_ctg1_5240	B1	46.14440509	Bcer	M	Bcer	FALSE	NA	NA	NA
Bc_ctg1_5241	B1	15.38146836	Bcer	E	Bcer	FALSE	NA	NA	NA
Bc_ctg1_5242	B1	8.633469339	Bcer	M	Bcer	FALSE	NA	NA	NA
Bc_ctg1_5243	B1	4.06864647	Bcer	M	Bcer	FALSE	NA	NA	NA
Bc_ctg1_5244	B1	8.038057661	Bcer	S	Bcer	FALSE	NA	NA	NA
Bc_ctg1_5245	B1	1.190823357	Bcer	D	Bcer	FALSE	NA	NA	NA
Bc_ctg1_5246	B1	2.183176155	Bcer	C	Bcer	FALSE	NA	NA	NA
Bc_ctg1_5247	B1	10.12199854	Bcer	K	Bcer	FALSE	NA	NA	NA
Bc_ctg1_5248	B1	1.984705595	Bcer	S	Bcer	FALSE	NA	NA	NA
Bc_ctg1_5249	B1	1.389293917	Bcer	NA	Bcer	FALSE	NA	NA	NA
Bc_ctg1_525	B1	51.70158076	Bcer	S	Bcer	FALSE	NA	NA	NA
Bc_ctg1_5250	B1	0.893117518	Bcer	NA	Bcer	FALSE	NA	NA	NA
Bc_ctg1_5251	B1	20.44246763	Bcer	Q	Bcer	TRUE	NA	NA	NA
Bc_ctg1_5252	B1	0.595411679	Bcer	S	Bcer	FALSE	NA	NA	NA
Bc_ctg1_5253	B1	45.64822869	Bcer	M	Bcer	FALSE	NA	NA	NA
Bc_ctg1_5254	B1	8.23652822	Bcer	T	Bcer	FALSE	NA	NA	NA
Bc_ctg1_5255	B1	1.786235036	Bcer	T	Bcer	FALSE	NA	NA	NA
Bc_ctg1_5256	B1	0	Bcer	NA	Bcer	FALSE	NA	NA	NA
Bc_ctg1_5257	B1	0.992352798	Bcer	K	Bcer	FALSE	NA	NA	NA
Bc_ctg1_5258	B1	17.86235036	Bcer	S	Bcer	FALSE	NA	NA	NA
Bc_ctg1_5259	B1	14.28988029	Bcer	F	Bcer	FALSE	NA	NA	NA
Bc_ctg1_526	B1	98.24292696	Bcer	S	Bcer	FALSE	NA	NA	NA
Bc_ctg1_5260	B1	5.954116786	Bcer	F	Bcer	FALSE	NA	NA	NA
Bc_ctg1_5261	B1	14.28988029	Bcer	S	Bcer	FALSE	NA	NA	NA
Bc_ctg1_5262	B1	10.61817493	Bcer	M	Bcer	FALSE	NA	NA	NA
Bc_ctg1_5263	B1	32.8468776	Bcer	E	Bcer	FALSE	NA	NA	NA
Bc_ctg1_5264	B1	40.58722942	Bcer	S	Bcer	FALSE	NA	NA	NA
Bc_ctg1_5265	B1	0.992352798	Bcer	S	Bcer	FALSE	NA	NA	NA
Bc_ctg1_5267	B1	0	Bcer	S	Bcer	FALSE	NA	NA	NA
Bc_ctg1_5268	B1	18.25929148	Bcer	K	Bcer	FALSE	NA	NA	NA
Bc_ctg1_5269	B1	15.48070364	Bcer	K	Bcer	FALSE	NA	NA	NA
Bc_ctg1_527	B1	2.282411435	Bcer	M	Bcer	FALSE	NA	NA	NA

Bc_ctg1_5270	B1	0.793882238	Bcer	NA	Bcer	FALSE	NA	NA	NA
Bc_ctg1_5271	B1	137.2423919	Bcer	NA	Bcer	FALSE	NA	NA	NA
Bc_ctg1_5272	B1	230.4243196	Bcer	EGP	Bcer	FALSE	NA	NA	NA
Bc_ctg1_5273	B1	111.0442781	Bcer	M	Bcer	FALSE	NA	NA	NA
Bc_ctg1_5274	B1	168.2037992	Bcer	M	Bcer	FALSE	NA	NA	NA
Bc_ctg1_5275	B1	506.7945737	Bcer	K	Bcer	FALSE	NA	NA	NA
Bc_ctg1_5276	B1	8.534234059	Bcer	S	Bcer	FALSE	NA	NA	NA
Bc_ctg1_5277	B1	0.992352798	Bcer	EGP	Bcer	FALSE	NA	NA	NA
Bc_ctg1_5278	B1	14.48835085	Bcer	G	Bcer	FALSE	NA	NA	NA
Bc_ctg1_5279	B1	10.51893965	Bcer	NA	Bcer	FALSE	NA	NA	NA
Bc_ctg1_5280	B1	0.19847056	Bcer	NA	Bcer	FALSE	NA	NA	NA
Bc_ctg1_5281	B1	52.59469827	Bcer	NA	Bcer	FALSE	NA	NA	NA
Bc_ctg1_5282	B1	0	Bcer	S	Bcer	FALSE	NA	NA	NA
Bc_ctg1_5283	B1	0.992352798	Bcer	F	Bcer	FALSE	NA	NA	NA
Bc_ctg1_5284	B1	0.09923528	Bcer	S	Bcer	FALSE	NA	NA	NA
Bc_ctg1_5285	B1	36.91552407	Bcer	Q	Bcer	TRUE	NA	NA	NA
Bc_ctg1_5286	B1	0	Bcer	NA	Bcer	FALSE	NA	NA	NA
Bc_ctg1_5289	B1	0.297705839	Bcer	NA	Bcer	FALSE	NA	NA	NA
Bc_ctg1_5290	B1	21.53405571	Bcer	EGP	Bcer	FALSE	NA	NA	NA
Bc_ctg1_5291	B1	0.496176399	Bcer	NA	Bcer	FALSE	NA	NA	NA
Bc_ctg1_5292	B1	11.90823357	Bcer	NA	Bcer	FALSE	NA	NA	NA
Bc_ctg1_5293	B1	199.5621476	Bcer	E	Bcer	FALSE	NA	NA	NA
Bc_ctg1_5294	B1	10.71741021	Bcer	I	Bcer	FALSE	NA	NA	NA
Bc_ctg1_5295	B1	22.22870267	Bcer	H	Bcer	FALSE	NA	NA	NA
Bc_ctg1_5296	B1	2.778587833	Bcer	J	Bcer	FALSE	NA	NA	NA
Bc_ctg1_5297	B1	6.351057905	Bcer	O	Bcer	FALSE	NA	NA	NA
Bc_ctg1_5298	B1	3.473234792	Bcer	NA	Bcer	FALSE	NA	NA	NA
Bc_ctg1_5299	B1	3.473234792	Bcer	V	Bcer	FALSE	NA	NA	NA
Bc_ctg1_53	B1	0.496176399	Bcer	E	Bcer	FALSE	NA	NA	NA
Bc_ctg1_5300	B1	0.694646958	Bcer	K	Bcer	FALSE	NA	NA	NA
Bc_ctg1_5301	B1	0	Bcer	NA	Bcer	FALSE	NA	NA	NA
Bc_ctg1_5302	B1	0	Bcer	S	Bcer	FALSE	NA	NA	NA
Bc_ctg1_5304	B1	0	Bcer	NA	Bcer	FALSE	NA	NA	NA
Bc_ctg1_5305	B1	0	Bcer	NA	Bcer	FALSE	NA	NA	NA
Bc_ctg1_5307	B1	0	Bcer	S	Bcer	FALSE	NA	NA	NA
Bc_ctg1_5309	B1	47.33522845	Bcer	S	Bcer	FALSE	NA	NA	NA
Bc_ctg1_5310	B1	58.8465209	Bcer	S	Bcer	FALSE	NA	NA	NA
Bc_ctg1_5311	B1	4.26711703	Bcer	C	Bcer	FALSE	NA	NA	NA
Bc_ctg1_5312	B1	4.26711703	Bcer	P	Bcer	FALSE	NA	NA	NA
Bc_ctg1_5313	B1	2.977058393	Bcer	P	Bcer	FALSE	NA	NA	NA
Bc_ctg1_5314	B1	1.686999756	Bcer	P	Bcer	FALSE	NA	NA	NA
Bc_ctg1_5315	B1	7.244175423	Bcer	S	Bcer	FALSE	NA	NA	NA
Bc_ctg1_5317	B1	23.12182018	Bcer	NA	Bcer	FALSE	NA	NA	NA
Bc_ctg1_5318	B1	5.755646226	Bcer	S	Bcer	FALSE	NA	NA	NA
Bc_ctg1_5319	B1	6.251822625	Bcer	H	Bcer	FALSE	NA	NA	NA
Bc_ctg1_532	B1	16.27458588	Bcer	L	Bcer	FALSE	NA	NA	NA
Bc_ctg1_5320	B1	19.74782067	Bcer	V	Bcer	FALSE	NA	NA	NA
Bc_ctg1_5321	B1	0.09923528	Bcer	K	Bcer	FALSE	NA	NA	NA
Bc_ctg1_5322	B1	24.5111141	Bcer	EG	Bcer	FALSE	NA	NA	NA
Bc_ctg1_5323	B1	0.595411679	Bcer	NA	Bcer	FALSE	NA	NA	NA
Bc_ctg1_5324	B1	7.938822381	Bcer	K	Bcer	FALSE	NA	NA	NA
Bc_ctg1_5325	B1	7.144940143	Bcer	J	Bcer	FALSE	NA	NA	NA
Bc_ctg1_5326	B1	3.671705351	Bcer	S	Bcer	FALSE	NA	NA	NA
Bc_ctg1_5327	B1	9.526586857	Bcer	L	Bcer	FALSE	NA	NA	NA
Bc_ctg1_5328	B1	0.19847056	Bcer	E	Bcer	FALSE	NA	NA	NA
Bc_ctg1_5329	B1	0	Bcer	E	Bcer	FALSE	NA	NA	NA
Bc_ctg1_533	B1	1.587764476	Bcer	CO	Bcer	FALSE	NA	NA	NA
Bc_ctg1_5330	B1	0.793882238	Bcer	E	Bcer	FALSE	NA	NA	NA
Bc_ctg1_5331	B1	0.793882238	Bcer	S	Bcer	FALSE	NA	NA	NA
Bc_ctg1_5332	B1	21.33558515	Bcer	S	Bcer	FALSE	NA	NA	NA
Bc_ctg1_5333	B1	130.9905693	Bcer	G	Bcer	FALSE	NA	NA	NA
Bc_ctg1_5334	B1	0.496176399	Bcer	S	Bcer	FALSE	NA	NA	NA

Bc_ctg1_5335	B1	2.480881994	Bcer	P	Bcer	FALSE	NA	NA	NA
Bc_ctg1_5336	B1	1127.610484	Bcer	M	Bcer	FALSE	NA	NA	NA
Bc_ctg1_5337	B1	620.2204985	Bcer	S	Bcer	FALSE	NA	NA	NA
Bc_ctg1_5338	B1	17.1677034	Bcer	S	Bcer	FALSE	NA	NA	NA
Bc_ctg1_5339	B1	66.38840216	Bcer	K	Bcer	FALSE	NA	NA	NA
Bc_ctg1_534	B1	1.190823357	Bcer	NA	Bcer	FALSE	NA	NA	NA
Bc_ctg1_5340	B1	58.74728562	Bcer	S	Bcer	FALSE	NA	NA	NA
Bc_ctg1_5341	B1	144.6850379	Bcer	E	Bcer	FALSE	NA	NA	NA
Bc_ctg1_5342	B1	56.16716834	Bcer	E	Bcer	FALSE	NA	NA	NA
Bc_ctg1_5343	B1	70.25857807	Bcer	E	Bcer	FALSE	NA	NA	NA
Bc_ctg1_5344	B1	30.36599561	Bcer	E	Bcer	FALSE	NA	NA	NA
Bc_ctg1_5345	B1	165.2267408	Bcer	E	Bcer	FALSE	NA	NA	NA
Bc_ctg1_5346	B1	95.56357441	Bcer	K	Bcer	FALSE	NA	NA	NA
Bc_ctg1_5347	B1	0.297705839	Bcer	S	Bcer	FALSE	NA	NA	NA
Bc_ctg1_5348	B1	10.41970437	Bcer	Q	Bcer	TRUE	NA	NA	NA
Bc_ctg1_5349	B1	4.961763988	Bcer	J	Bcer	FALSE	NA	NA	NA
Bc_ctg1_535	B1	0.19847056	Bcer	V	Bcer	FALSE	NA	NA	NA
Bc_ctg1_5350	B1	5.557175667	Bcer	S	Bcer	FALSE	NA	NA	NA
Bc_ctg1_5351	B1	0.19847056	Bcer	S	Bcer	FALSE	NA	NA	NA
Bc_ctg1_5352	B1	2.679352554	Bcer	NA	Bcer	FALSE	NA	NA	NA
Bc_ctg1_5353	B1	2.381646714	Bcer	S	Bcer	FALSE	NA	NA	NA
Bc_ctg1_5354	B1	33.73999512	Bcer	M	Bcer	FALSE	NA	NA	NA
Bc_ctg1_5355	B1	0.09923528	Bcer	S	Bcer	FALSE	NA	NA	NA
Bc_ctg1_5356	B1	4.06864647	Bcer	E	Bcer	FALSE	NA	NA	NA
Bc_ctg1_5357	B1	2.679352554	Bcer	C	Bcer	FALSE	NA	NA	NA
Bc_ctg1_5358	B1	1.290058637	Bcer	K	Bcer	FALSE	NA	NA	NA
Bc_ctg1_5359	B1	4.366352309	Bcer	NA	Bcer	FALSE	NA	NA	NA
Bc_ctg1_536	B1	4580.402808	Bcer	C	Bcer	FALSE	NA	NA	NA
Bc_ctg1_5360	B1	8.43499878	Bcer	NA	Bcer	FALSE	NA	NA	NA
Bc_ctg1_5361	B1	51.60234548	Bcer	S	Bcer	FALSE	NA	NA	NA
Bc_ctg1_5362	B1	622.7013805	Bcer	S	Bcer	FALSE	NA	NA	NA
Bc_ctg1_5363	B1	0.694646958	Bcer	G	Bcer	FALSE	NA	NA	NA
Bc_ctg1_5364	B1	1.091588077	Bcer	G	Bcer	FALSE	NA	NA	NA
Bc_ctg1_5365	B1	0.396941119	Bcer	G	Bcer	FALSE	NA	NA	NA
Bc_ctg1_5366	B1	0	Bcer	G	Bcer	FALSE	NA	NA	NA
Bc_ctg1_5367	B1	0	Bcer	K	Bcer	FALSE	NA	NA	NA
Bc_ctg1_5368	B1	0	Bcer	G	Bcer	FALSE	NA	NA	NA
Bc_ctg1_5369	B1	0	Bcer	G	Bcer	FALSE	NA	NA	NA
Bc_ctg1_537	B1	0	Bcer	S	Bcer	FALSE	NA	NA	NA
Bc_ctg1_5370	B1	0	Bcer	G	Bcer	FALSE	NA	NA	NA
Bc_ctg1_5371	B1	3.373999512	Bcer	E	Bcer	FALSE	NA	NA	NA
Bc_ctg1_5372	B1	4.564822869	Bcer	S	Bcer	FALSE	NA	NA	NA
Bc_ctg1_5373	B1	8.534234059	Bcer	EGP	Bcer	FALSE	NA	NA	NA
Bc_ctg1_5374	B1	6.747999024	Bcer	K	Bcer	FALSE	NA	NA	NA
Bc_ctg1_5375	B1	0.595411679	Bcer	F	Bcer	FALSE	NA	NA	NA
Bc_ctg1_5376	B1	24.41187882	Bcer	E	Bcer	FALSE	NA	NA	NA
Bc_ctg1_5377	B1	11.61052773	Bcer	E	Bcer	FALSE	NA	NA	NA
Bc_ctg1_5378	B1	6.549528464	Bcer	E	Bcer	FALSE	NA	NA	NA
Bc_ctg1_5379	B1	15.97688004	Bcer	E	Bcer	FALSE	NA	NA	NA
Bc_ctg1_538	B1	3.274764232	Bcer	G	Bcer	FALSE	NA	NA	NA
Bc_ctg1_5380	B1	7.442645982	Bcer	K	Bcer	FALSE	NA	NA	NA
Bc_ctg1_5381	B1	1.290058637	Bcer	E	Bcer	FALSE	NA	NA	NA
Bc_ctg1_5382	B1	5.954116786	Bcer	E	Bcer	FALSE	NA	NA	NA
Bc_ctg1_5383	B1	0	Bcer	E	Bcer	FALSE	NA	NA	NA
Bc_ctg1_5384	B1	198.1728537	Bcer	I	Bcer	FALSE	NA	NA	NA
Bc_ctg1_5385	B1	13.49599805	Bcer	V	Bcer	FALSE	NA	NA	NA
Bc_ctg1_5386	B1	1.389293917	Bcer	V	Bcer	FALSE	NA	NA	NA
Bc_ctg1_5387	B1	0.297705839	Bcer	P	Bcer	FALSE	NA	NA	NA
Bc_ctg1_5388	B1	6.351057905	Bcer	S	Bcer	FALSE	NA	NA	NA
Bc_ctg1_5389	B1	1.686999756	Bcer	K	Bcer	FALSE	NA	NA	NA
Bc_ctg1_539	B1	0.793882238	Bcer	NA	Bcer	FALSE	NA	NA	NA
Bc_ctg1_5390	B1	13.59523333	Bcer	L	Bcer	FALSE	NA	NA	NA

Bc_ctg1_5391	B1	2.679352554	Bcer	NA	Bcer	FALSE	NA	NA	NA
Bc_ctg1_5392	B1	21.33558515	Bcer	S	Bcer	FALSE	NA	NA	NA
Bc_ctg1_5393	B1	41.28187638	Bcer	F	Bcer	FALSE	NA	NA	NA
Bc_ctg1_5394	B1	25.10652578	Bcer	J	Bcer	FALSE	NA	NA	NA
Bc_ctg1_5395	B1	22.52640851	Bcer	E	Bcer	FALSE	NA	NA	NA
Bc_ctg1_5397	B1	0.09923528	Bcer	NA	Bcer	FALSE	NA	NA	NA
Bc_ctg1_5398	B1	11.61052773	Bcer	S	Bcer	FALSE	NA	NA	NA
Bc_ctg1_5399	B1	4.763293429	Bcer	K	Bcer	FALSE	NA	NA	NA
Bc_ctg1_54	B1	0.793882238	Bcer	P	Bcer	FALSE	NA	NA	NA
Bc_ctg1_5400	B1	0.694646958	Bcer	S	Bcer	FALSE	NA	NA	NA
Bc_ctg1_5401	B1	0.992352798	Bcer	S	Bcer	FALSE	NA	NA	NA
Bc_ctg1_5402	B1	0.793882238	Bcer	NA	Bcer	FALSE	NA	NA	NA
Bc_ctg1_5403	B1	535.275099	Bcer	EGP	Bcer	FALSE	NA	NA	NA
Bc_ctg1_5404	B1	2.183176155	Bcer	E	Bcer	FALSE	NA	NA	NA
Bc_ctg1_5405	B1	1.290058637	Bcer	C	Bcer	FALSE	NA	NA	NA
Bc_ctg1_5406	B1	0.893117518	Bcer	S	Bcer	FALSE	NA	NA	NA
Bc_ctg1_5407	B1	0	Bcer	NA	Bcer	FALSE	NA	NA	NA
Bc_ctg1_5408	B1	5.755646226	Bcer	F	Bcer	FALSE	NA	NA	NA
Bc_ctg1_5409	B1	27.48817249	Bcer	V	Bcer	FALSE	NA	NA	NA
Bc_ctg1_541	B1	0.09923528	Bcer	O	Bcer	FALSE	NA	NA	NA
Bc_ctg1_5410	B1	0.595411679	Bcer	NA	Bcer	FALSE	NA	NA	NA
Bc_ctg1_5411	B1	5.557175667	Bcer	S	Bcer	FALSE	NA	NA	NA
Bc_ctg1_5412	B1	14.58758612	Bcer	C	Bcer	FALSE	NA	NA	NA
Bc_ctg1_5413	B1	7.541881262	Bcer	K	Bcer	FALSE	NA	NA	NA
Bc_ctg1_5414	B1	42.1749939	Bcer	J	Bcer	FALSE	NA	NA	NA
Bc_ctg1_5415	B1	0.09923528	Bcer	F	Bcer	FALSE	NA	NA	NA
Bc_ctg1_5416	B1	3.274764232	Bcer	S	Bcer	FALSE	NA	NA	NA
Bc_ctg1_5417	B1	12.10670413	Bcer	S	Bcer	FALSE	NA	NA	NA
Bc_ctg1_5418	B1	22.42717323	Bcer	V	Bcer	FALSE	NA	NA	NA
Bc_ctg1_5419	B1	6.152587345	Bcer	K	Bcer	FALSE	NA	NA	NA
Bc_ctg1_542	B1	30.96140729	Bcer	EGP	Bcer	FALSE	NA	NA	NA
Bc_ctg1_5421	B1	6.549528464	Bcer	GM	Bcer	FALSE	NA	NA	NA
Bc_ctg1_5422	B1	5.954116786	Bcer	V	Bcer	FALSE	NA	NA	NA
Bc_ctg1_5423	B1	5.656410946	Bcer	K	Bcer	FALSE	NA	NA	NA
Bc_ctg1_5424	B1	7.740351821	Bcer	K	Bcer	FALSE	NA	NA	NA
Bc_ctg1_5425	B1	3.473234792	Bcer	NA	Bcer	FALSE	NA	NA	NA
Bc_ctg1_5426	B1	1.091588077	Bcer	L	Bcer	FALSE	NA	NA	NA
Bc_ctg1_5427	B1	0.09923528	Bcer	EG	Bcer	FALSE	NA	NA	NA
Bc_ctg1_5428	B1	15.57993892	Bcer	K	Bcer	FALSE	NA	NA	NA
Bc_ctg1_5429	B1	16.671527	Bcer	S	Bcer	FALSE	NA	NA	NA
Bc_ctg1_543	B1	35.72470071	Bcer	NA	Bcer	FALSE	NA	NA	NA
Bc_ctg1_5430	B1	2.083940875	Bcer	S	Bcer	FALSE	NA	NA	NA
Bc_ctg1_5431	B1	16.07611532	Bcer	S	Bcer	FALSE	NA	NA	NA
Bc_ctg1_5432	B1	13.79370389	Bcer	F	Bcer	FALSE	NA	NA	NA
Bc_ctg1_5433	B1	9.526586857	Bcer	T	Bcer	FALSE	NA	NA	NA
Bc_ctg1_5434	B1	7.839587101	Bcer	S	Bcer	FALSE	NA	NA	NA
Bc_ctg1_5435	B1	9.030410458	Bcer	E	Bcer	FALSE	NA	NA	NA
Bc_ctg1_5436	B1	2.282411435	Bcer	S	Bcer	FALSE	NA	NA	NA
Bc_ctg1_5437	B1	0.09923528	Bcer	J	Bcer	FALSE	NA	NA	NA
Bc_ctg1_544	B1	34.8315832	Bcer	J	Bcer	FALSE	NA	NA	NA
Bc_ctg1_545	B1	2.778587833	Bcer	F	Bcer	FALSE	NA	NA	NA
Bc_ctg1_546	B1	3.770940631	Bcer	NA	Bcer	FALSE	NA	NA	NA
Bc_ctg1_547	B1	0.793882238	Bcer	I	Bcer	FALSE	NA	NA	NA
Bc_ctg1_548	B1	1.290058637	Bcer	IQ	Bcer	TRUE	NA	NA	NA
Bc_ctg1_549	B1	3.870175911	Bcer	S	Bcer	FALSE	NA	NA	NA
Bc_ctg1_55	B1	1.389293917	Bcer	S	Bcer	FALSE	NA	NA	NA
Bc_ctg1_550	B1	6.351057905	Bcer	IQ	Bcer	TRUE	NA	NA	NA
Bc_ctg1_551	B1	0.893117518	Bcer	S	Bcer	FALSE	NA	NA	NA
Bc_ctg1_552	B1	6.251822625	Bcer	V	Bcer	FALSE	NA	NA	NA
Bc_ctg1_553	B1	4.465587589	Bcer	K	Bcer	FALSE	NA	NA	NA
Bc_ctg1_554	B1	3.671705351	Bcer	NA	Bcer	FALSE	NA	NA	NA
Bc_ctg1_555	B1	2.580117274	Bcer	K	Bcer	FALSE	NA	NA	NA

Bc_ctg1_556	B1	6.251822625	Bcer	NA	Bcer	FALSE	NA	NA	NA	
Bc_ctg1_557	B1	32.8468776	Bcer	S	Bcer	FALSE	NA	NA	NA	
Bc_ctg1_558	B1	6.251822625	Bcer	V	Bcer	FALSE	NA	NA	NA	
Bc_ctg1_559	B1	8.23652822	Bcer	T	Bcer	FALSE	NA	NA	NA	
Bc_ctg1_56	B1	0	Bcer	L	Bcer	FALSE	NA	NA	NA	
Bc_ctg1_560	B1	20.83940875	Bcer	S	Bcer	FALSE	NA	NA	NA	
Bc_ctg1_561	B1	1.488529196	Bcer	E	Bcer	FALSE	NA	NA	NA	
Bc_ctg1_562	B1	32.74764232	Bcer	T	Bcer	FALSE	NA	NA	NA	
Bc_ctg1_563	B1	16.77076228	Bcer	S	Bcer	FALSE	NA	NA	NA	
Bc_ctg1_564	B1	13.29752749	Bcer	E	Bcer	FALSE	NA	NA	NA	
Bc_ctg1_565	B1	3.274764232	Bcer	E	Bcer	FALSE	NA	NA	NA	
Bc_ctg1_566	B1	1.587764476	Bcer	O	Bcer	FALSE	NA	NA	NA	
Bc_ctg1_567	B1	0.09923528	Bcer	E	Bcer	FALSE	NA	NA	NA	
Bc_ctg1_568	B1	57.25875642	Bcer	C	Bcer	FALSE	NA	NA	NA	
Bc_ctg1_569	B1	239.3554948	Bcer	K	Bcer	FALSE	NA	NA	NA	
Bc_ctg1_57	B1	14.98452724	Bcer	L	Bcer	FALSE	NA	NA	NA	
Bc_ctg1_570	B1	30.56446617	Bcer	E	Bcer	FALSE	NA	NA	NA	
Bc_ctg1_571	B1	24.0149377	Bcer	Q	Bcer	TRUE	NA	NA	NA	
Bc_ctg1_572	B1	28.87746641	Bcer	E	Bcer	FALSE	NA	NA	NA	
Bc_ctg1_573	B1	26.4958197	Bcer	E	Bcer	FALSE	NA	NA	NA	
Bc_ctg1_574	B1	686.7081359	Bcer	K	Bcer	FALSE	NA	NA	NA	
Bc_ctg1_575	B1	5.755646226	Bcer	Q	Bcer	TRUE	NA	NA	NA	
Bc_ctg1_576	B1	2.679352554	Bcer	S	Bcer	FALSE	NA	NA	NA	
Bc_ctg1_577	B1	0.396941119	Bcer	F	Bcer	FALSE	NA	NA	NA	
Bc_ctg1_578	B1	24.5111141	Bcer	NA	Bcer	FALSE	NA	NA	NA	
Bc_ctg1_579	B1	8.534234059	Bcer	S	Bcer	FALSE	NA	NA	NA	
Bc_ctg1_58	B1	11.51129245	Bcer	L	Bcer	FALSE	NA	NA	NA	
Bc_ctg1_580	B1	211.5696165	Bcer	O	Bcer	FALSE	NA	NA	NA	
Bc_ctg1_581	B1	61.72434401	Bcer	O	Bcer	FALSE	NA	NA	NA	
Bc_ctg1_582	B1	0	Bcer	NA	Bcer	FALSE	NA	NA	NA	
Bc_ctg1_583	B1	28.38129001	Bcer	EG	Bcer	FALSE	NA	NA	NA	
Bc_ctg1_584	B1	20.83940875	Bcer	E	Bcer	FALSE	NA	NA	NA	
Bc_ctg1_585	B1	24.80881994	Bcer	S	Bcer	FALSE	NA	NA	NA	
Bc_ctg1_586	B1	13.19829221	Bcer	V	Bcer	FALSE	NA	NA	NA	
Bc_ctg1_588	B1	7.938822381	Bcer	V	Bcer	FALSE	NA	NA	NA	
Bc_ctg1_589	B1	1.190823357	Bcer	NA	Bcer	FALSE	NA	NA	NA	
Bc_ctg1_59	B1	2.778587833	Bcer	NA	Bcer	FALSE	NA	NA	NA	
Bc_ctg1_590	B1	36.71705351	Bcer	K	Bcer	FALSE	NA	NA	NA	
Bc_ctg1_591	B1	5.954116786	Bcer	S	Bcer	FALSE	NA	NA	NA	
Bc_ctg1_592	B1	27.38893721	Bcer	M	Bcer	FALSE	NA	NA	NA	
Bc_ctg1_593	B1	1.091588077	Bcer	F	Bcer	FALSE	NA	NA	NA	
Bc_ctg1_594	B1	0.297705839	Bcer	S	Bcer	FALSE	NA	NA	NA	
Bc_ctg1_595	B1	17.46540924	Bcer	S	Bcer	FALSE	NA	NA	NA	
Bc_ctg1_596	B1	0	Bcer	NA	Bcer	FALSE	NA	NA	NA	
Bc_ctg1_597	B1	12.90058637	Bcer	NA	Bcer	FALSE	NA	NA	NA	
Bc_ctg1_598	B1	6.946469583	Bcer	S	Bcer	FALSE	NA	NA	NA	
Bc_ctg1_599	B1	0.793882238	Bcer	M	Bcer	FALSE	NA	NA	NA	
Bc_ctg1_6	B1	5.358705107	Bcer	NA	Bcer	FALSE	NA	NA	NA	
Bc_ctg1_60	B1	13.29752749	Bcer	E	Bcer	FALSE	NA	NA	NA	
Bc_ctg1_600	B1	22.03023211	Bcer	V	Bcer	FALSE	NA	NA	NA	
Bc_ctg1_601	B1	9.625822137	Bcer	V	Bcer	FALSE	NA	NA	NA	
Bc_ctg1_602	B1	142.1049206	Bcer	S	Bcer	FALSE	NA	NA	NA	
Bc_ctg1_603	B1	41.1826411	Bcer	S	Bcer	FALSE	NA	NA	NA	
Bc_ctg1_604	B1	2376.68495	Bcer	G	Bcer	FALSE	NA	NA	NA	
Bc_ctg1_605	B1	3.373999512	Bcer	P	Bcer	FALSE	NA	NA	NA	
Bc_ctg1_606	B1	2.083940875	Bcer	P	Bcer	FALSE	NA	NA	NA	
Bc_ctg1_607	B1	4.465587589	Bcer	P	Bcer	FALSE	NA	NA	NA	
Bc_ctg1_608	B1	2.877823113	Bcer	P	Bcer	FALSE	NA	NA	NA	
Bc_ctg1_609	B1	15.77840948	Bcer	F	Bcer	FALSE	NA	NA	NA	
Bc_ctg1_61	B1	20.74017347	Bcer	K	Bcer	FALSE	NA	NA	NA	
Bc_ctg1_610	B1	10.61817493	Bcer	S	Bcer	FALSE	NA	NA	NA	
Bc_ctg1_611	B1	20.04552651	Bcer	L	Bcer	FALSE	NA	NA	NA	

Bc_ctg1_612	B1	363.6973003	Bcer	K	Bcer	FALSE	NA	NA	NA
Bc_ctg1_613	B1	8.3357635	Bcer	S	Bcer	FALSE	NA	NA	NA
Bc_ctg1_614	B1	10.3204691	Bcer	S	Bcer	FALSE	NA	NA	NA
Bc_ctg1_615	B1	0	Bcer	K	Bcer	FALSE	NA	NA	NA
Bc_ctg1_616	B1	3.274764232	Bcer	S	Bcer	FALSE	NA	NA	NA
Bc_ctg1_617	B1	28.28205473	Bcer	S	Bcer	FALSE	NA	NA	NA
Bc_ctg1_618	B1	41.48034694	Bcer	M	Bcer	FALSE	NA	NA	NA
Bc_ctg1_619	B1	339.6823626	Bcer	M	Bcer	FALSE	NA	NA	NA
Bc_ctg1_62	B1	1.984705595	Bcer	L	Bcer	FALSE	NA	NA	NA
Bc_ctg1_620	B1	2.381646714	Bcer	S	Bcer	FALSE	NA	NA	NA
Bc_ctg1_621	B1	6.847234304	Bcer	L	Bcer	FALSE	NA	NA	NA
Bc_ctg1_622	B1	0.09923528	Bcer	NA	Bcer	FALSE	NA	NA	NA
Bc_ctg1_623	B1	4.862528708	Bcer	NA	Bcer	FALSE	NA	NA	NA
Bc_ctg1_624	B1	0.09923528	Bcer	NA	Bcer	FALSE	NA	NA	NA
Bc_ctg1_625	B1	63.01440265	Bcer	K	Bcer	FALSE	NA	NA	NA
Bc_ctg1_626	B1	2063.299937	Bcer	E	Bcer	FALSE	NA	NA	NA
Bc_ctg1_627	B1	236.0807306	Bcer	K	Bcer	FALSE	NA	NA	NA
Bc_ctg1_628	B1	53.68628635	Bcer	P	Bcer	FALSE	NA	NA	NA
Bc_ctg1_629	B1	65.6937552	Bcer	S	Bcer	FALSE	NA	NA	NA
Bc_ctg1_63	B1	20.44246763	Bcer	V	Bcer	FALSE	NA	NA	NA
Bc_ctg1_630	B1	19.45011483	Bcer	S	Bcer	FALSE	NA	NA	NA
Bc_ctg1_631	B1	0	Bcer	NA	Bcer	FALSE	NA	NA	NA
Bc_ctg1_632	B1	1.885470315	Bcer	O	Bcer	FALSE	NA	NA	NA
Bc_ctg1_633	B1	74.5256951	Bcer	L	Bcer	FALSE	NA	NA	NA
Bc_ctg1_634	B1	1.190823357	Bcer	Q	Bcer	TRUE	NA	NA	NA
Bc_ctg1_635	B1	370.0483582	Bcer	J	Bcer	FALSE	NA	NA	NA
Bc_ctg1_636	B1	133.7691571	Bcer	J	Bcer	FALSE	NA	NA	NA
Bc_ctg1_637	B1	0.09923528	Bcer	NA	Bcer	FALSE	NA	NA	NA
Bc_ctg1_638	B1	68.77004887	Bcer	T	Bcer	FALSE	NA	NA	NA
Bc_ctg1_639	B1	3794.161686	Bcer	GT	Bcer	FALSE	NA	NA	NA
Bc_ctg1_64	B1	16.57229172	Bcer	S	Bcer	FALSE	NA	NA	NA
Bc_ctg1_640	B1	1021.627205	Bcer	G	Bcer	FALSE	NA	NA	NA
Bc_ctg1_641	B1	2405.264711	Bcer	K	Bcer	FALSE	NA	NA	NA
Bc_ctg1_642	B1	180.7074444	Bcer	C	Bcer	FALSE	NA	NA	NA
Bc_ctg1_643	B1	145.9750965	Bcer	S	Bcer	FALSE	NA	NA	NA
Bc_ctg1_645	B1	99.63222088	Bcer	NA	Bcer	FALSE	NA	NA	NA
Bc_ctg1_646	B1	0.694646958	Bcer	E	Bcer	FALSE	NA	NA	NA
Bc_ctg1_647	B1	0	Bcer	G	Bcer	FALSE	NA	NA	NA
Bc_ctg1_648	B1	1.885470315	Bcer	S	Bcer	FALSE	NA	NA	NA
Bc_ctg1_649	B1	2.877823113	Bcer	P	Bcer	FALSE	NA	NA	NA
Bc_ctg1_65	B1	58.44957978	Bcer	F	Bcer	FALSE	NA	NA	NA
Bc_ctg1_650	B1	350.0028317	Bcer	L	Bcer	FALSE	NA	NA	NA
Bc_ctg1_651	B1	5.557175667	Bcer	M	Bcer	FALSE	NA	NA	NA
Bc_ctg1_652	B1	414.4065283	Bcer	P	Bcer	FALSE	NA	NA	NA
Bc_ctg1_653	B1	92.98345714	Bcer	P	Bcer	FALSE	NA	NA	NA
Bc_ctg1_654	B1	61.82357929	Bcer	S	Bcer	FALSE	NA	NA	NA
Bc_ctg1_655	B1	0.09923528	Bcer	NA	Bcer	FALSE	NA	NA	NA
Bc_ctg1_656	B1	80.38057661	Bcer	S	Bcer	FALSE	NA	NA	NA
Bc_ctg1_657	B1	43.66352309	Bcer	HP	Bcer	FALSE	NA	NA	NA
Bc_ctg1_658	B1	55.96869779	Bcer	P	Bcer	FALSE	NA	NA	NA
Bc_ctg1_659	B1	97.44904473	Bcer	P	Bcer	FALSE	NA	NA	NA
Bc_ctg1_66	B1	8.831939899	Bcer	I	Bcer	FALSE	NA	NA	NA
Bc_ctg1_660	B1	336.0106573	Bcer	P	Bcer	FALSE	NA	NA	NA
Bc_ctg1_661	B1	16.47305644	Bcer	L	Bcer	FALSE	NA	NA	NA
Bc_ctg1_662	B1	8.831939899	Bcer	K	Bcer	FALSE	Bc_Ga0417192_01_rn07	Regulation	SMCOG1287
Bc_ctg1_663	B1	7.045704863	Bcer	L	Bcer	FALSE	Bc_Ga0417192_01_rn07	Other	NA
Bc_ctg1_664	B1	29.17517225	Bcer	L	Bcer	FALSE	Bc_Ga0417192_01_rn07	Other	NA
Bc_ctg1_665	B1	286.8891938	Bcer	E	Bcer	FALSE	Bc_Ga0417192_01_rn07	Other	NA
Bc_ctg1_666	B1	0.992352798	Bcer	S	Bcer	FALSE	Bc_Ga0417192_01_rn07	Other	NA
Bc_ctg1_667	B1	25.20576106	Bcer	NA	Bcer	FALSE	Bc_Ga0417192_01_rn07	Other	NA
Bc_ctg1_668	B1	12.70211581	Bcer	G	Bcer	FALSE	Bc_Ga0417192_01_rn07	Ancillary biosynthesis	SMCOG1101
Bc_ctg1_669	B1	23.61799658	Bcer	S	Bcer	FALSE	Bc_Ga0417192_01_rn07	Ancillary biosynthesis	SMCOG1262

Bc_ctg1_67	B1	1.290058637	Bcer	Q	Bcer	TRUE	NA	NA	NA
Bc_ctg1_670	B1	183.2875617	Bcer	T	Bcer	FALSE	Bc_Ga0417192_01_rn07	Ancillary biosynthesis	SMCOG1025
Bc_ctg1_671	B1	0.793882238	Bcer	M	Bcer	FALSE	Bc_Ga0417192_01_rn07	Core biosynthesis	None
Bc_ctg1_672	B1	0	Bcer	NA	Bcer	FALSE	Bc_Ga0417192_01_rn07	Other	NA
Bc_ctg1_673	B1	0	Bcer	GM	Bcer	FALSE	Bc_Ga0417192_01_rn07	Core biosynthesis	SMCOG1010
Bc_ctg1_674	B1	0.396941119	Bcer	M	Bcer	FALSE	Bc_Ga0417192_01_rn07	Core biosynthesis	SMCOG1010
Bc_ctg1_675	B1	0	Bcer	S	Bcer	FALSE	Bc_Ga0417192_01_rn07	Other	NA
Bc_ctg1_676	B1	1.984705595	Bcer	S	Bcer	FALSE	Bc_Ga0417192_01_rn07	Other	NA
Bc_ctg1_677	B1	1.488529196	Bcer	M	Bcer	FALSE	Bc_Ga0417192_01_rn07	Other	NA
Bc_ctg1_678	B1	2.282411435	Bcer	S	Bcer	FALSE	Bc_Ga0417192_01_rn07	Other	NA
Bc_ctg1_679	B1	11.11435133	Bcer	K	Bcer	FALSE	Bc_Ga0417192_01_rn07	Ancillary biosynthesis	SMCOG1019
Bc_ctg1_68	B1	1.290058637	Bcer	Q	Bcer	TRUE	NA	NA	NA
Bc_ctg1_680	B1	0.793882238	Bcer	NA	Bcer	FALSE	Bc_Ga0417192_01_rn07	Other	NA
Bc_ctg1_681	B1	27.78587833	Bcer	K	Bcer	FALSE	Bc_Ga0417192_01_rn07	Regulation	SMCOG1136
Bc_ctg1_682	B1	69.46469583	Bcer	G	Bcer	FALSE	Bc_Ga0417192_01_rn07	Other	NA
Bc_ctg1_683	B1	57.15952114	Bcer	U	Bcer	FALSE	Bc_Ga0417192_01_rn07	Other	NA
Bc_ctg1_684	B1	47.83140484	Bcer	O	Bcer	FALSE	NA	NA	NA
Bc_ctg1_685	B1	386.8191205	Bcer	M	Bcer	FALSE	NA	NA	NA
Bc_ctg1_686	B1	8.43499878	Bcer	S	Bcer	FALSE	NA	NA	NA
Bc_ctg1_687	B1	20.34323235	Bcer	M	Bcer	FALSE	NA	NA	NA
Bc_ctg1_688	B1	17.46540924	Bcer	S	Bcer	FALSE	NA	NA	NA
Bc_ctg1_69	B1	0.595411679	Bcer	NA	Bcer	FALSE	NA	NA	NA
Bc_ctg1_690	B1	134.1660982	Bcer	L	Bcer	FALSE	NA	NA	NA
Bc_ctg1_691	B1	152.9215661	Bcer	L	Bcer	FALSE	NA	NA	NA
Bc_ctg1_692	B1	1.389293917	Bcer	S	Bcer	FALSE	NA	NA	NA
Bc_ctg1_693	B1	116.1052773	Bcer	S	Bcer	FALSE	NA	NA	NA
Bc_ctg1_694	B1	393.9640607	Bcer	J	Bcer	FALSE	NA	NA	NA
Bc_ctg1_695	B1	864.3392867	Bcer	C	Bcer	FALSE	NA	NA	NA
Bc_ctg1_696	B1	1571.886831	Bcer	C	Bcer	FALSE	NA	NA	NA
Bc_ctg1_697	B1	14.78605668	Bcer	E	Bcer	FALSE	NA	NA	NA
Bc_ctg1_698	B1	36.12164183	Bcer	S	Bcer	FALSE	NA	NA	NA
Bc_ctg1_699	B1	97.94522112	Bcer	S	Bcer	FALSE	NA	NA	NA
Bc_ctg1_7	B1	0.595411679	Bcer	S	Bcer	FALSE	NA	NA	NA
Bc_ctg1_70	B1	3.770940631	Bcer	S	Bcer	FALSE	NA	NA	NA
Bc_ctg1_700	B1	2554.713042	Bcer	S	Bcer	FALSE	NA	NA	NA
Bc_ctg1_701	B1	360.7202419	Bcer	L	Bcer	FALSE	NA	NA	NA
Bc_ctg1_702	B1	297.0111923	Bcer	S	Bcer	FALSE	NA	NA	NA
Bc_ctg1_703	B1	112.3343367	Bcer	I	Bcer	FALSE	NA	NA	NA
Bc_ctg1_704	B1	209.4856756	Bcer	S	Bcer	FALSE	NA	NA	NA
Bc_ctg1_705	B1	179.2189152	Bcer	S	Bcer	FALSE	NA	NA	NA
Bc_ctg1_706	B1	1.488529196	Bcer	S	Bcer	FALSE	NA	NA	NA
Bc_ctg1_707	B1	24.0149377	Bcer	IQ	Bcer	TRUE	NA	NA	NA
Bc_ctg1_708	B1	256.7216687	Bcer	S	Bcer	FALSE	NA	NA	NA
Bc_ctg1_709	B1	156.9902126	Bcer	S	Bcer	FALSE	NA	NA	NA
Bc_ctg1_71	B1	4.664058149	Bcer	K	Bcer	FALSE	NA	NA	NA
Bc_ctg1_710	B1	315.6674249	Bcer	S	Bcer	FALSE	NA	NA	NA
Bc_ctg1_711	B1	279.3473125	Bcer	S	Bcer	FALSE	NA	NA	NA
Bc_ctg1_712	B1	346.4303616	Bcer	S	Bcer	FALSE	NA	NA	NA
Bc_ctg1_713	B1	1307.821752	Bcer	S	Bcer	FALSE	NA	NA	NA
Bc_ctg1_714	B1	412.4218227	Bcer	K	Bcer	FALSE	NA	NA	NA
Bc_ctg1_715	B1	107.471808	Bcer	NA	Bcer	FALSE	NA	NA	NA
Bc_ctg1_716	B1	109.3572783	Bcer	D	Bcer	FALSE	NA	NA	NA
Bc_ctg1_717	B1	0.893117518	Bcer	S	Bcer	FALSE	NA	NA	NA
Bc_ctg1_718	B1	4.16788175	Bcer	OU	Bcer	FALSE	NA	NA	NA
Bc_ctg1_719	B1	1094.763606	Bcer	S	Bcer	FALSE	NA	NA	NA
Bc_ctg1_72	B1	0.297705839	Bcer	K	Bcer	FALSE	NA	NA	NA
Bc_ctg1_720	B1	815.6147644	Bcer	E	Bcer	FALSE	NA	NA	NA
Bc_ctg1_721	B1	1113.916015	Bcer	E	Bcer	FALSE	NA	NA	NA
Bc_ctg1_722	B1	1207.197178	Bcer	E	Bcer	FALSE	NA	NA	NA
Bc_ctg1_723	B1	2.679352554	Bcer	H	Bcer	FALSE	NA	NA	NA
Bc_ctg1_724	B1	16.47305644	Bcer	NA	Bcer	FALSE	NA	NA	NA
Bc_ctg1_726	B1	55.07558027	Bcer	T	Bcer	FALSE	NA	NA	NA

Bc_ctg1_727	B1	66.1899316	Bcer	NA	Bcer	FALSE	NA	NA	NA
Bc_ctg1_728	B1	1.488529196	Bcer	NA	Bcer	FALSE	NA	NA	NA
Bc_ctg1_729	B1	0.19847056	Bcer	L	Bcer	FALSE	NA	NA	NA
Bc_ctg1_73	B1	3.770940631	Bcer	P	Bcer	FALSE	NA	NA	NA
Bc_ctg1_731	B1	0.396941119	Bcer	M	Bcer	FALSE	NA	NA	NA
Bc_ctg1_732	B1	0	Bcer	S	Bcer	FALSE	NA	NA	NA
Bc_ctg1_733	B1	110.0519253	Bcer	S	Bcer	FALSE	NA	NA	NA
Bc_ctg1_734	B1	0.19847056	Bcer	NA	Bcer	FALSE	NA	NA	NA
Bc_ctg1_735	B1	1.488529196	Bcer	U	Bcer	FALSE	NA	NA	NA
Bc_ctg1_736	B1	0.396941119	Bcer	S	Bcer	FALSE	NA	NA	NA
Bc_ctg1_737	B1	1.488529196	Bcer	D	Bcer	FALSE	NA	NA	NA
Bc_ctg1_739	B1	0	Bcer	S	Bcer	FALSE	NA	NA	NA
Bc_ctg1_74	B1	6.450293184	Bcer	S	Bcer	FALSE	NA	NA	NA
Bc_ctg1_740	B1	0	Bcer	S	Bcer	FALSE	NA	NA	NA
Bc_ctg1_741	B1	0	Bcer	NA	Bcer	FALSE	NA	NA	NA
Bc_ctg1_742	B1	0.09923528	Bcer	S	Bcer	FALSE	NA	NA	NA
Bc_ctg1_743	B1	0	Bcer	S	Bcer	FALSE	NA	NA	NA
Bc_ctg1_745	B1	0.396941119	Bcer	S	Bcer	FALSE	NA	NA	NA
Bc_ctg1_746	B1	0.396941119	Bcer	OU	Bcer	FALSE	NA	NA	NA
Bc_ctg1_747	B1	0.793882238	Bcer	S	Bcer	FALSE	NA	NA	NA
Bc_ctg1_748	B1	0.893117518	Bcer	S	Bcer	FALSE	NA	NA	NA
Bc_ctg1_749	B1	0.992352798	Bcer	S	Bcer	FALSE	NA	NA	NA
Bc_ctg1_75	B1	0.595411679	Bcer	NA	Bcer	FALSE	NA	NA	NA
Bc_ctg1_750	B1	1.190823357	Bcer	V	Bcer	FALSE	NA	NA	NA
Bc_ctg1_751	B1	0.496176399	Bcer	NA	Bcer	FALSE	NA	NA	NA
Bc_ctg1_752	B1	1648.297997	Bcer	NA	Bcer	FALSE	NA	NA	NA
Bc_ctg1_753	B1	0.09923528	Bcer	NA	Bcer	FALSE	NA	NA	NA
Bc_ctg1_754	B1	0	Bcer	NA	Bcer	FALSE	NA	NA	NA
Bc_ctg1_755	B1	0.396941119	Bcer	L	Bcer	FALSE	NA	NA	NA
Bc_ctg1_756	B1	0	Bcer	S	Bcer	FALSE	NA	NA	NA
Bc_ctg1_757	B1	84.64769364	Bcer	S	Bcer	FALSE	NA	NA	NA
Bc_ctg1_758	B1	87.62475203	Bcer	S	Bcer	FALSE	NA	NA	NA
Bc_ctg1_759	B1	0.09923528	Bcer	NA	Bcer	FALSE	NA	NA	NA
Bc_ctg1_76	B1	0.09923528	Bcer	S	Bcer	FALSE	NA	NA	NA
Bc_ctg1_760	B1	0.09923528	Bcer	NA	Bcer	FALSE	NA	NA	NA
Bc_ctg1_761	B1	0.09923528	Bcer	NA	Bcer	FALSE	NA	NA	NA
Bc_ctg1_762	B1	0.694646958	Bcer	L	Bcer	FALSE	NA	NA	NA
Bc_ctg1_763	B1	1.488529196	Bcer	L	Bcer	FALSE	NA	NA	NA
Bc_ctg1_764	B1	0.297705839	Bcer	NA	Bcer	FALSE	NA	NA	NA
Bc_ctg1_765	B1	0.396941119	Bcer	NA	Bcer	FALSE	NA	NA	NA
Bc_ctg1_766	B1	0.09923528	Bcer	NA	Bcer	FALSE	NA	NA	NA
Bc_ctg1_767	B1	0.19847056	Bcer	NA	Bcer	FALSE	NA	NA	NA
Bc_ctg1_768	B1	0.09923528	Bcer	NA	Bcer	FALSE	NA	NA	NA
Bc_ctg1_769	B1	0.19847056	Bcer	K	Bcer	FALSE	NA	NA	NA
Bc_ctg1_77	B1	46.83905205	Bcer	G	Bcer	FALSE	NA	NA	NA
Bc_ctg1_770	B1	23.12182018	Bcer	K	Bcer	FALSE	NA	NA	NA
Bc_ctg1_771	B1	0.19847056	Bcer	E	Bcer	FALSE	NA	NA	NA
Bc_ctg1_772	B1	0.893117518	Bcer	NA	Bcer	FALSE	NA	NA	NA
Bc_ctg1_773	B1	2.778587833	Bcer	NA	Bcer	FALSE	NA	NA	NA
Bc_ctg1_774	B1	7.740351821	Bcer	L	Bcer	FALSE	NA	NA	NA
Bc_ctg1_775	B1	0	Bcer	H	Bcer	FALSE	NA	NA	NA
Bc_ctg1_776	B1	0.09923528	Bcer	E	Bcer	FALSE	NA	NA	NA
Bc_ctg1_778	B1	322.5146592	Bcer	S	Bcer	FALSE	NA	NA	NA
Bc_ctg1_779	B1	25.80117274	Bcer	G	Bcer	FALSE	NA	NA	NA
Bc_ctg1_78	B1	0	Bcer	S	Bcer	FALSE	NA	NA	NA
Bc_ctg1_780	B1	1486.048314	Bcer	J	Bcer	FALSE	NA	NA	NA
Bc_ctg1_781	B1	761.8292427	Bcer	J	Bcer	FALSE	NA	NA	NA
Bc_ctg1_782	B1	224.7679087	Bcer	H	Bcer	FALSE	NA	NA	NA
Bc_ctg1_783	B1	85.04463476	Bcer	J	Bcer	FALSE	NA	NA	NA
Bc_ctg1_784	B1	365.2850648	Bcer	J	Bcer	FALSE	NA	NA	NA
Bc_ctg1_785	B1	168.4022698	Bcer	S	Bcer	FALSE	NA	NA	NA
Bc_ctg1_786	B1	2111.627518	Bcer	J	Bcer	FALSE	NA	NA	NA

Bc_ctg1_787	B1	188.9439727	Bcer	J	Bcer	FALSE	NA	NA	NA
Bc_ctg1_788	B1	52.79316883	Bcer	K	Bcer	FALSE	NA	NA	NA
Bc_ctg1_789	B1	589.0606207	Bcer	K	Bcer	FALSE	NA	NA	NA
Bc_ctg1_79	B1	6.450293184	Bcer	I	Bcer	FALSE	NA	NA	NA
Bc_ctg1_790	B1	379.178004	Bcer	S	Bcer	FALSE	NA	NA	NA
Bc_ctg1_791	B1	321.6215417	Bcer	L	Bcer	FALSE	NA	NA	NA
Bc_ctg1_792	B1	407.4600587	Bcer	J	Bcer	FALSE	NA	NA	NA
Bc_ctg1_793	B1	352.0867726	Bcer	M	Bcer	FALSE	NA	NA	NA
Bc_ctg1_794	B1	249.378258	Bcer	I	Bcer	FALSE	NA	NA	NA
Bc_ctg1_795	B1	119.2808063	Bcer	S	Bcer	FALSE	NA	NA	NA
Bc_ctg1_796	B1	101.3192206	Bcer	I	Bcer	FALSE	NA	NA	NA
Bc_ctg1_797	B1	449.6350526	Bcer	J	Bcer	FALSE	NA	NA	NA
Bc_ctg1_798	B1	371.2391816	Bcer	F	Bcer	FALSE	NA	NA	NA
Bc_ctg1_799	B1	1405.171561	Bcer	J	Bcer	FALSE	NA	NA	NA
Bc_ctg1_8	B1	1.587764476	Bcer	NA	Bcer	FALSE	NA	NA	NA
Bc_ctg1_80	B1	8.931175179	Bcer	T	Bcer	FALSE	NA	NA	NA
Bc_ctg1_800	B1	4434.526947	Bcer	J	Bcer	FALSE	NA	NA	NA
Bc_ctg1_801	B1	468.6882263	Bcer	K	Bcer	FALSE	NA	NA	NA
Bc_ctg1_802	B1	494.4893991	Bcer	O	Bcer	FALSE	NA	NA	NA
Bc_ctg1_803	B1	36.41934767	Bcer	O	Bcer	FALSE	NA	NA	NA
Bc_ctg1_804	B1	121.0670413	Bcer	L	Bcer	FALSE	NA	NA	NA
Bc_ctg1_805	B1	272.1031371	Bcer	J	Bcer	FALSE	NA	NA	NA
Bc_ctg1_806	B1	1950.469424	Bcer	L	Bcer	FALSE	NA	NA	NA
Bc_ctg1_807	B1	1.488529196	Bcer	LU	Bcer	FALSE	NA	NA	NA
Bc_ctg1_808	B1	1070.947139	Bcer	C	Bcer	FALSE	NA	NA	NA
Bc_ctg1_809	B1	1377.981095	Bcer	C	Bcer	FALSE	NA	NA	NA
Bc_ctg1_81	B1	29.17517225	Bcer	L	Bcer	FALSE	NA	NA	NA
Bc_ctg1_810	B1	9.328116298	Bcer	L	Bcer	FALSE	NA	NA	NA
Bc_ctg1_811	B1	150.5399194	Bcer	S	Bcer	FALSE	NA	NA	NA
Bc_ctg1_812	B1	98.44139752	Bcer	U	Bcer	FALSE	NA	NA	NA
Bc_ctg1_813	B1	2919.898872	Bcer	J	Bcer	FALSE	NA	NA	NA
Bc_ctg1_814	B1	53.58705107	Bcer	J	Bcer	FALSE	NA	NA	NA
Bc_ctg1_815	B1	28.08358417	Bcer	J	Bcer	FALSE	NA	NA	NA
Bc_ctg1_816	B1	201.0506768	Bcer	S	Bcer	FALSE	NA	NA	NA
Bc_ctg1_817	B1	765.7986539	Bcer	J	Bcer	FALSE	NA	NA	NA
Bc_ctg1_818	B1	1116.297662	Bcer	U	Bcer	FALSE	NA	NA	NA
Bc_ctg1_819	B1	102.9069851	Bcer	S	Bcer	FALSE	NA	NA	NA
Bc_ctg1_82	B1	19.64858539	Bcer	K	Bcer	FALSE	NA	NA	NA
Bc_ctg1_820	B1	123.2502175	Bcer	U	Bcer	FALSE	NA	NA	NA
Bc_ctg1_821	B1	156.791742	Bcer	D	Bcer	FALSE	NA	NA	NA
Bc_ctg1_822	B1	288.5761935	Bcer	J	Bcer	FALSE	NA	NA	NA
Bc_ctg1_823	B1	147.1659199	Bcer	IQ	Bcer	TRUE	NA	NA	NA
Bc_ctg1_824	B1	230.4243196	Bcer	IQ	Bcer	TRUE	NA	NA	NA
Bc_ctg1_825	B1	125.92957	Bcer	I	Bcer	FALSE	NA	NA	NA
Bc_ctg1_826	B1	80.47981189	Bcer	I	Bcer	FALSE	NA	NA	NA
Bc_ctg1_827	B1	46.64058149	Bcer	K	Bcer	FALSE	NA	NA	NA
Bc_ctg1_828	B1	83.25839972	Bcer	L	Bcer	FALSE	NA	NA	NA
Bc_ctg1_829	B1	1194.495062	Bcer	S	Bcer	FALSE	NA	NA	NA
Bc_ctg1_83	B1	1.091588077	Bcer	EGP	Bcer	FALSE	NA	NA	NA
Bc_ctg1_830	B1	235.7830247	Bcer	S	Bcer	FALSE	NA	NA	NA
Bc_ctg1_831	B1	29.17517225	Bcer	J	Bcer	FALSE	NA	NA	NA
Bc_ctg1_832	B1	22.42717323	Bcer	H	Bcer	FALSE	NA	NA	NA
Bc_ctg1_833	B1	224.9663792	Bcer	G	Bcer	FALSE	NA	NA	NA
Bc_ctg1_834	B1	114.6167481	Bcer	S	Bcer	FALSE	NA	NA	NA
Bc_ctg1_835	B1	653.8612583	Bcer	KLT	Bcer	FALSE	NA	NA	NA
Bc_ctg1_836	B1	138.1355094	Bcer	T	Bcer	FALSE	NA	NA	NA
Bc_ctg1_837	B1	416.6889397	Bcer	J	Bcer	FALSE	NA	NA	NA
Bc_ctg1_838	B1	165.6236819	Bcer	J	Bcer	FALSE	NA	NA	NA
Bc_ctg1_839	B1	106.4794552	Bcer	J	Bcer	FALSE	NA	NA	NA
Bc_ctg1_84	B1	2.877823113	Bcer	E	Bcer	FALSE	NA	NA	NA
Bc_ctg1_840	B1	25.70193746	Bcer	J	Bcer	FALSE	NA	NA	NA
Bc_ctg1_841	B1	79.68592965	Bcer	L	Bcer	FALSE	NA	NA	NA

Bc_ctg1_842	B1	35.22852432	Bcer	H	Bcer	FALSE	NA	NA	NA
Bc_ctg1_843	B1	40.09105302	Bcer	K	Bcer	FALSE	NA	NA	NA
Bc_ctg1_844	B1	105.5863377	Bcer	F	Bcer	FALSE	NA	NA	NA
Bc_ctg1_845	B1	37.51093575	Bcer	S	Bcer	FALSE	NA	NA	NA
Bc_ctg1_846	B1	132.9752749	Bcer	S	Bcer	FALSE	NA	NA	NA
Bc_ctg1_847	B1	17.76311508	Bcer	P	Bcer	FALSE	NA	NA	NA
Bc_ctg1_848	B1	89.31175179	Bcer	K	Bcer	FALSE	NA	NA	NA
Bc_ctg1_849	B1	12.60288053	Bcer	S	Bcer	FALSE	NA	NA	NA
Bc_ctg1_85	B1	0.297705839	Bcer	I	Bcer	FALSE	NA	NA	NA
Bc_ctg1_850	B1	0.793882238	Bcer	E	Bcer	FALSE	NA	NA	NA
Bc_ctg1_851	B1	0.793882238	Bcer	S	Bcer	FALSE	NA	NA	NA
Bc_ctg1_852	B1	2.977058393	Bcer	S	Bcer	FALSE	NA	NA	NA
Bc_ctg1_853	B1	0.496176399	Bcer	S	Bcer	FALSE	NA	NA	NA
Bc_ctg1_855	B1	0.297705839	Bcer	C	Bcer	FALSE	NA	NA	NA
Bc_ctg1_856	B1	20.83940875	Bcer	S	Bcer	FALSE	NA	NA	NA
Bc_ctg1_857	B1	39.49564134	Bcer	S	Bcer	FALSE	NA	NA	NA
Bc_ctg1_858	B1	178.1273272	Bcer	F	Bcer	FALSE	NA	NA	NA
Bc_ctg1_859	B1	192.1195016	Bcer	F	Bcer	FALSE	NA	NA	NA
Bc_ctg1_86	B1	0.496176399	Bcer	P	Bcer	FALSE	NA	NA	NA
Bc_ctg1_860	B1	312.0949548	Bcer	F	Bcer	FALSE	NA	NA	NA
Bc_ctg1_861	B1	187.0585024	Bcer	C	Bcer	FALSE	NA	NA	NA
Bc_ctg1_862	B1	1211.067354	Bcer	F	Bcer	FALSE	NA	NA	NA
Bc_ctg1_863	B1	273.7901369	Bcer	F	Bcer	FALSE	NA	NA	NA
Bc_ctg1_864	B1	101.815397	Bcer	F	Bcer	FALSE	NA	NA	NA
Bc_ctg1_865	B1	55.67099195	Bcer	F	Bcer	FALSE	NA	NA	NA
Bc_ctg1_866	B1	106.2809846	Bcer	F	Bcer	FALSE	NA	NA	NA
Bc_ctg1_867	B1	155.005507	Bcer	F	Bcer	FALSE	NA	NA	NA
Bc_ctg1_868	B1	274.9809602	Bcer	J	Bcer	FALSE	NA	NA	NA
Bc_ctg1_869	B1	122.4563352	Bcer	MU	Bcer	FALSE	NA	NA	NA
Bc_ctg1_87	B1	2.778587833	Bcer	S	Bcer	FALSE	NA	NA	NA
Bc_ctg1_870	B1	0	Bcer	T	Bcer	FALSE	NA	NA	NA
Bc_ctg1_871	B1	928.4452774	Bcer	J	Bcer	FALSE	NA	NA	NA
Bc_ctg1_872	B1	449.1388762	Bcer	D	Bcer	FALSE	NA	NA	NA
Bc_ctg1_873	B1	665.8687272	Bcer	S	Bcer	FALSE	NA	NA	NA
Bc_ctg1_874	B1	63.80828489	Bcer	S	Bcer	FALSE	NA	NA	NA
Bc_ctg1_875	B1	183.386797	Bcer	D	Bcer	FALSE	NA	NA	NA
Bc_ctg1_876	B1	310.4079551	Bcer	S	Bcer	FALSE	Bc_Ga0417192_01_rn08	Other	NA
Bc_ctg1_877	B1	370.8422405	Bcer	S	Bcer	FALSE	Bc_Ga0417192_01_rn08	Other	NA
Bc_ctg1_878	B1	0	Bcer	S	Bcer	FALSE	Bc_Ga0417192_01_rn08	Other	NA
Bc_ctg1_879	B1	0.19847056	Bcer	K	Bcer	FALSE	Bc_Ga0417192_01_rn08	Other	NA
Bc_ctg1_88	B1	0.19847056	Bcer	CP	Bcer	FALSE	NA	NA	NA
Bc_ctg1_880	B1	1.686999756	Bcer	K	Bcer	FALSE	Bc_Ga0417192_01_rn08	Other	NA
Bc_ctg1_881	B1	2.778587833	Bcer	M	Bcer	FALSE	Bc_Ga0417192_01_rn08	Other	NA
Bc_ctg1_882	B1	1126.121955	Bcer	D	Bcer	FALSE	Bc_Ga0417192_01_rn08	Other	NA
Bc_ctg1_883	B1	971.3149183	Bcer	D	Bcer	FALSE	Bc_Ga0417192_01_rn08	Other	NA
Bc_ctg1_884	B1	19.35087955	Bcer	D	Bcer	FALSE	Bc_Ga0417192_01_rn08	Other	NA
Bc_ctg1_885	B1	75.61728318	Bcer	M	Bcer	FALSE	Bc_Ga0417192_01_rn08	Other	NA
Bc_ctg1_886	B1	35.82393599	Bcer	M	Bcer	FALSE	Bc_Ga0417192_01_rn08	Core biosynthesis	SMCOG1062
Bc_ctg1_887	B1	50.41152212	Bcer	D	Bcer	FALSE	Bc_Ga0417192_01_rn08	Other	NA
Bc_ctg1_888	B1	473.6499903	Bcer	M	Bcer	FALSE	Bc_Ga0417192_01_rn08	Other	NA
Bc_ctg1_889	B1	199.363677	Bcer	M	Bcer	FALSE	Bc_Ga0417192_01_rn08	Core biosynthesis	None
Bc_ctg1_89	B1	0	Bcer	S	Bcer	FALSE	NA	NA	NA
Bc_ctg1_890	B1	151.829978	Bcer	M	Bcer	FALSE	Bc_Ga0417192_01_rn08	Other	NA
Bc_ctg1_891	B1	276.9656658	Bcer	M	Bcer	FALSE	Bc_Ga0417192_01_rn08	Other	NA
Bc_ctg1_892	B1	430.3834083	Bcer	M	Bcer	FALSE	Bc_Ga0417192_01_rn08	Other	NA
Bc_ctg1_893	B1	304.0568972	Bcer	D	Bcer	FALSE	Bc_Ga0417192_01_rn08	Other	NA
Bc_ctg1_894	B1	372.2315344	Bcer	J	Bcer	FALSE	Bc_Ga0417192_01_rn08	Other	NA
Bc_ctg1_895	B1	167.6083875	Bcer	S	Bcer	FALSE	Bc_Ga0417192_01_rn08	Other	NA
Bc_ctg1_896	B1	24.11417298	Bcer	H	Bcer	FALSE	NA	NA	NA
Bc_ctg1_897	B1	241.2409651	Bcer	K	Bcer	FALSE	NA	NA	NA
Bc_ctg1_898	B1	10.71741021	Bcer	S	Bcer	FALSE	NA	NA	NA
Bc_ctg1_899	B1	88.51786955	Bcer	J	Bcer	FALSE	NA	NA	NA

Bc_ctg1_9	B1	0	Bcer	NA	Bcer	FALSE	NA	NA	NA
Bc_ctg1_90	B1	0.19847056	Bcer	EGP	Bcer	FALSE	NA	NA	NA
Bc_ctg1_900	B1	821.2711753	Bcer	S	Bcer	FALSE	NA	NA	NA
Bc_ctg1_901	B1	68.1746372	Bcer	S	Bcer	FALSE	NA	NA	NA
Bc_ctg1_902	B1	19.35087955	Bcer	T	Bcer	FALSE	NA	NA	NA
Bc_ctg1_903	B1	15.38146836	Bcer	S	Bcer	FALSE	NA	NA	NA
Bc_ctg1_904	B1	0.396941119	Bcer	S	Bcer	FALSE	NA	NA	NA
Bc_ctg1_905	B1	60.73199121	Bcer	H	Bcer	FALSE	NA	NA	NA
Bc_ctg1_906	B1	31.45758368	Bcer	L	Bcer	FALSE	NA	NA	NA
Bc_ctg1_907	B1	90.30410458	Bcer	S	Bcer	FALSE	NA	NA	NA
Bc_ctg1_908	B1	75.71651846	Bcer	S	Bcer	FALSE	NA	NA	NA
Bc_ctg1_909	B1	85.24310531	Bcer	S	Bcer	FALSE	NA	NA	NA
Bc_ctg1_91	B1	2.381646714	Bcer	K	Bcer	FALSE	NA	NA	NA
Bc_ctg1_910	B1	32.44993648	Bcer	G	Bcer	FALSE	NA	NA	NA
Bc_ctg1_911	B1	0	Bcer	S	Bcer	FALSE	NA	NA	NA
Bc_ctg1_912	B1	0.09923528	Bcer	S	Bcer	FALSE	NA	NA	NA
Bc_ctg1_913	B1	72.64022479	Bcer	S	Bcer	FALSE	NA	NA	NA
Bc_ctg1_914	B1	4.961763988	Bcer	S	Bcer	FALSE	NA	NA	NA
Bc_ctg1_915	B1	8.931175179	Bcer	S	Bcer	FALSE	NA	NA	NA
Bc_ctg1_916	B1	32.8468776	Bcer	S	Bcer	FALSE	NA	NA	NA
Bc_ctg1_917	B1	22.82411435	Bcer	C	Bcer	FALSE	NA	NA	NA
Bc_ctg1_918	B1	64.40369657	Bcer	C	Bcer	FALSE	NA	NA	NA
Bc_ctg1_919	B1	191.1271488	Bcer	C	Bcer	FALSE	NA	NA	NA
Bc_ctg1_92	B1	0.396941119	Bcer	S	Bcer	FALSE	NA	NA	NA
Bc_ctg1_920	B1	173.9594454	Bcer	C	Bcer	FALSE	NA	NA	NA
Bc_ctg1_921	B1	284.8052529	Bcer	O	Bcer	FALSE	NA	NA	NA
Bc_ctg1_922	B1	207.50097	Bcer	O	Bcer	FALSE	NA	NA	NA
Bc_ctg1_923	B1	0.19847056	Bcer	L	Bcer	FALSE	NA	NA	NA
Bc_ctg1_924	B1	1.389293917	Bcer	L	Bcer	FALSE	NA	NA	NA
Bc_ctg1_925	B1	2924.959871	Bcer	C	Bcer	FALSE	NA	NA	NA
Bc_ctg1_926	B1	143.295744	Bcer	D	Bcer	FALSE	NA	NA	NA
Bc_ctg1_927	B1	250.2713756	Bcer	S	Bcer	FALSE	NA	NA	NA
Bc_ctg1_928	B1	152.3261544	Bcer	S	Bcer	FALSE	NA	NA	NA
Bc_ctg1_929	B1	249.7751992	Bcer	T	Bcer	FALSE	NA	NA	NA
Bc_ctg1_93	B1	20.34323235	Bcer	G	Bcer	FALSE	NA	NA	NA
Bc_ctg1_930	B1	238.5616125	Bcer	S	Bcer	FALSE	NA	NA	NA
Bc_ctg1_931	B1	15.48070364	Bcer	S	Bcer	FALSE	NA	NA	NA
Bc_ctg1_932	B1	1.389293917	Bcer	NA	Bcer	FALSE	NA	NA	NA
Bc_ctg1_933	B1	4.06864647	Bcer	S	Bcer	FALSE	NA	NA	NA
Bc_ctg1_934	B1	2347.906719	Bcer	T	Bcer	FALSE	NA	NA	NA
Bc_ctg1_935	B1	68.37310776	Bcer	NA	Bcer	FALSE	NA	NA	NA
Bc_ctg1_936	B1	37.51093575	Bcer	G	Bcer	FALSE	NA	NA	NA
Bc_ctg1_937	B1	0.694646958	Bcer	NA	Bcer	FALSE	NA	NA	NA
Bc_ctg1_938	B1	52.69393355	Bcer	S	Bcer	FALSE	NA	NA	NA
Bc_ctg1_939	B1	4.465587589	Bcer	S	Bcer	FALSE	NA	NA	NA
Bc_ctg1_94	B1	5.954116786	Bcer	K	Bcer	FALSE	NA	NA	NA
Bc_ctg1_940	B1	443.9786417	Bcer	E	Bcer	FALSE	NA	NA	NA
Bc_ctg1_941	B1	2.679352554	Bcer	H	Bcer	FALSE	NA	NA	NA
Bc_ctg1_942	B1	0.09923528	Bcer	S	Bcer	FALSE	NA	NA	NA
Bc_ctg1_943	B1	0.694646958	Bcer	S	Bcer	FALSE	NA	NA	NA
Bc_ctg1_944	B1	0.19847056	Bcer	S	Bcer	FALSE	NA	NA	NA
Bc_ctg1_945	B1	7.938822381	Bcer	S	Bcer	FALSE	NA	NA	NA
Bc_ctg1_946	B1	4.564822869	Bcer	S	Bcer	FALSE	NA	NA	NA
Bc_ctg1_947	B1	0.297705839	Bcer	NA	Bcer	FALSE	NA	NA	NA
Bc_ctg1_948	B1	2070.444877	Bcer	C	Bcer	FALSE	NA	NA	NA
Bc_ctg1_949	B1	2445.85194	Bcer	C	Bcer	FALSE	NA	NA	NA
Bc_ctg1_95	B1	47.63293429	Bcer	H	Bcer	FALSE	NA	NA	NA
Bc_ctg1_950	B1	1013.986089	Bcer	C	Bcer	FALSE	NA	NA	NA
Bc_ctg1_951	B1	1951.263306	Bcer	C	Bcer	FALSE	NA	NA	NA
Bc_ctg1_953	B1	102.9069851	Bcer	J	Bcer	FALSE	NA	NA	NA
Bc_ctg1_954	B1	84.15151724	Bcer	S	Bcer	FALSE	NA	NA	NA
Bc_ctg1_955	B1	38.10634743	Bcer	S	Bcer	FALSE	NA	NA	NA

Bc_ctg1_956	B1	1383.637506	Bcer	S	Bcer	FALSE	NA	NA	NA
Bc_ctg1_957	B1	87.12857563	Bcer	P	Bcer	FALSE	NA	NA	NA
Bc_ctg1_958	B1	63.90752017	Bcer	C	Bcer	FALSE	NA	NA	NA
Bc_ctg1_959	B1	161.4558002	Bcer	E	Bcer	FALSE	NA	NA	NA
Bc_ctg1_96	B1	1.190823357	Bcer	T	Bcer	FALSE	NA	NA	NA
Bc_ctg1_960	B1	461.3448156	Bcer	E	Bcer	FALSE	NA	NA	NA
Bc_ctg1_961	B1	105.7848082	Bcer	K	Bcer	FALSE	NA	NA	NA
Bc_ctg1_962	B1	83.05992916	Bcer	S	Bcer	FALSE	NA	NA	NA
Bc_ctg1_963	B1	3.870175911	Bcer	S	Bcer	FALSE	NA	NA	NA
Bc_ctg1_964	B1	0.297705839	Bcer	S	Bcer	FALSE	NA	NA	NA
Bc_ctg1_966	B1	23.22105546	Bcer	S	Bcer	FALSE	NA	NA	NA
Bc_ctg1_967	B1	3.870175911	Bcer	O	Bcer	FALSE	NA	NA	NA
Bc_ctg1_968	B1	11.21358661	Bcer	S	Bcer	FALSE	NA	NA	NA
Bc_ctg1_969	B1	120.2731591	Bcer	T	Bcer	FALSE	NA	NA	NA
Bc_ctg1_97	B1	1.290058637	Bcer	P	Bcer	FALSE	NA	NA	NA
Bc_ctg1_970	B1	96.75439777	Bcer	IQ	Bcer	TRUE	NA	NA	NA
Bc_ctg1_971	B1	1.488529196	Bcer	S	Bcer	FALSE	NA	NA	NA
Bc_ctg1_972	B1	0	Bcer	NA	Bcer	FALSE	NA	NA	NA
Bc_ctg1_973	B1	232.706731	Bcer	P	Bcer	FALSE	NA	NA	NA
Bc_ctg1_974	B1	46.14440509	Bcer	FP	Bcer	FALSE	NA	NA	NA
Bc_ctg1_975	B1	67.47999024	Bcer	S	Bcer	FALSE	NA	NA	NA
Bc_ctg1_976	B1	62.31975569	Bcer	L	Bcer	FALSE	NA	NA	NA
Bc_ctg1_977	B1	0.297705839	Bcer	T	Bcer	FALSE	NA	NA	NA
Bc_ctg1_978	B1	0	Bcer	S	Bcer	FALSE	Bc_Ga0417192_01_rn09	Other	NA
Bc_ctg1_979	B1	64.50293184	Bcer	NA	Bcer	FALSE	Bc_Ga0417192_01_rn09	Other	NA
Bc_ctg1_98	B1	1.190823357	Bcer	E	Bcer	FALSE	NA	NA	NA
Bc_ctg1_980	B1	55.96869779	Bcer	P	Bcer	FALSE	Bc_Ga0417192_01_rn09	Transport	SMCOG1005
Bc_ctg1_981	B1	16.47305644	Bcer	K	Bcer	FALSE	Bc_Ga0417192_01_rn09	Regulation	SMCOG1278
Bc_ctg1_982	B1	2130.283751	Bcer	E	Bcer	FALSE	Bc_Ga0417192_01_rn09	Other	NA
Bc_ctg1_983	B1	7.244175423	Bcer	S	Bcer	FALSE	Bc_Ga0417192_01_rn09	Other	NA
Bc_ctg1_984	B1	3.274764232	Bcer	M	Bcer	FALSE	Bc_Ga0417192_01_rn09	Other	NA
Bc_ctg1_985	B1	0.297705839	Bcer	S	Bcer	FALSE	Bc_Ga0417192_01_rn09	Other	NA
Bc_ctg1_986	B1	61.62510873	Bcer	T	Bcer	FALSE	Bc_Ga0417192_01_rn09	Regulation	SMCOG1003
Bc_ctg1_987	B1	64.00675545	Bcer	NA	Bcer	FALSE	Bc_Ga0417192_01_rn09	Other	NA
Bc_ctg1_988	B1	82.06757636	Bcer	E	Bcer	FALSE	Bc_Ga0417192_01_rn09	Core biosynthesis	SMCOG1019
Bc_ctg1_989	B1	80.08287077	Bcer	K	Bcer	FALSE	Bc_Ga0417192_01_rn09	Regulation	SMCOG1078
Bc_ctg1_99	B1	0.396941119	Bcer	K	Bcer	FALSE	NA	NA	NA
Bc_ctg1_990	B1	296.4157806	Bcer	P	Bcer	FALSE	Bc_Ga0417192_01_rn09	Transport	SMCOG1069
Bc_ctg1_991	B1	514.6341608	Bcer	P	Bcer	FALSE	Bc_Ga0417192_01_rn09	Transport	SMCOG1067
Bc_ctg1_992	B1	474.7415784	Bcer	G	Bcer	FALSE	Bc_Ga0417192_01_rn09	Ancillary biosynthesis	SMCOG1143
Bc_ctg1_993	B1	33.44228928	Bcer	G	Bcer	FALSE	Bc_Ga0417192_01_rn09	Core biosynthesis	SMCOG1257
Bc_ctg1_994	B1	0.09923528	Bcer	L	Bcer	FALSE	Bc_Ga0417192_01_rn09	Other	SMCOG1026
Bc_ctg1_996	B1	212.7604398	Bcer	G	Bcer	FALSE	Bc_Ga0417192_01_rn09	Core biosynthesis	SMCOG1257
Bc_ctg1_997	B1	352.7814196	Bcer	P	Bcer	FALSE	Bc_Ga0417192_01_rn09	Transport	SMCOG1000
Bc_ctg1_998	B1	255.4316101	Bcer	S	Bcer	FALSE	Bc_Ga0417192_01_rn09	Ancillary biosynthesis	SMCOG1001
Bc_ctg2_1	B1	0.793882238	Bcer	M	Bcer	FALSE	Bc_Ga0417192_02_rn01	Core biosynthesis	None
Bc_ctg2_10	B1	1.290058637	Bcer	S	Bcer	FALSE	Bc_Ga0417192_02_rn01	Other	NA
Bc_ctg2_100	B1	19.25164427	Bcer	K	Bcer	FALSE	Bc_Ga0417192_02_rn02	Ancillary biosynthesis	SMCOG1019
Bc_ctg2_101	B1	15.1829978	Bcer	NA	Bcer	FALSE	Bc_Ga0417192_02_rn02	Other	NA
Bc_ctg2_102	B1	128.410452	Bcer	E	Bcer	FALSE	Bc_Ga0417192_02_rn02	Transport	SMCOG1202
Bc_ctg2_103	B1	0.595411679	Bcer	M	Bcer	FALSE	Bc_Ga0417192_02_rn02	Ancillary biosynthesis	SMCOG1064
Bc_ctg2_104	B1	0.595411679	Bcer	M	Bcer	FALSE	Bc_Ga0417192_02_rn02	Ancillary biosynthesis	SMCOG1152
Bc_ctg2_105	B1	0.595411679	Bcer	M	Bcer	FALSE	Bc_Ga0417192_02_rn02	Core biosynthesis	SMCOG1010
Bc_ctg2_106	B1	0.496176399	Bcer	M	Bcer	FALSE	Bc_Ga0417192_02_rn02	Core biosynthesis	SMCOG1045
Bc_ctg2_107	B1	0	Bcer	NA	Bcer	FALSE	Bc_Ga0417192_02_rn02	Other	NA
Bc_ctg2_108	B1	0.297705839	Bcer	NA	Bcer	FALSE	Bc_Ga0417192_02_rn02	Other	NA
Bc_ctg2_109	B1	1.190823357	Bcer	S	Bcer	FALSE	Bc_Ga0417192_02_rn02	Other	NA
Bc_ctg2_11	B1	0.19847056	Bcer	NA	Bcer	FALSE	Bc_Ga0417192_02_rn01	Other	NA
Bc_ctg2_110	B1	0.793882238	Bcer	M	Bcer	FALSE	Bc_Ga0417192_02_rn02	Core biosynthesis	SMCOG1045
Bc_ctg2_111	B1	0.19847056	Bcer	S	Bcer	FALSE	Bc_Ga0417192_02_rn02	Ancillary biosynthesis	None
Bc_ctg2_116	B1	2.480881994	Bcer	NA	Bcer	FALSE	Bc_Ga0417192_02_rn02	Other	NA
Bc_ctg2_118	B1	2.778587833	Bcer	K	Bcer	FALSE	Bc_Ga0417192_02_rn02	Other	NA

Bc_ctg2_119	B1	14.78605668	Bcer	E	Bcer	FALSE	Bc_Ga0417192_02_rn02	Ancillary biosynthesis	SMCOG1158
Bc_ctg2_12	B1	0	Bcer	NA	Bcer	FALSE	Bc_Ga0417192_02_rn01	Other	NA
Bc_ctg2_120	B1	6.549528464	Bcer	E	Bcer	FALSE	Bc_Ga0417192_02_rn02	Ancillary biosynthesis	SMCOG1081
Bc_ctg2_121	B1	11.11435133	Bcer	K	Bcer	FALSE	Bc_Ga0417192_02_rn02	Other	NA
Bc_ctg2_122	B1	0.297705839	Bcer	NA	Bcer	FALSE	Bc_Ga0417192_02_rn02	Other	NA
Bc_ctg2_123	B1	2.282411435	Bcer	Q	Bcer	TRUE	Bc_Ga0417192_02_rn02	Ancillary biosynthesis	SMCOG1004
Bc_ctg2_124	B1	26.59505498	Bcer	Q	Bcer	TRUE	Bc_Ga0417192_02_rn02	Core biosynthesis	SMCOG1127
Bc_ctg2_125	B1	9.625822137	Bcer	Q	Bcer	TRUE	Bc_Ga0417192_02_rn02	Core biosynthesis	SMCOG1127
Bc_ctg2_126	B1	16.57229172	Bcer	IQ	Bcer	TRUE	Bc_Ga0417192_02_rn02	Core biosynthesis	SMCOG1127
Bc_ctg2_127	B1	1.290058637	Bcer	S	Bcer	FALSE	Bc_Ga0417192_02_rn02	Other	NA
Bc_ctg2_128	B1	63.31210849	Bcer	EGP	Bcer	FALSE	Bc_Ga0417192_02_rn02	Transport	SMCOG1202
Bc_ctg2_129	B1	96.55592721	Bcer	K	Bcer	FALSE	Bc_Ga0417192_02_rn02	Other	NA
Bc_ctg2_130	B1	27.38893721	Bcer	EG	Bcer	FALSE	Bc_Ga0417192_02_rn02	Transport	SMCOG1166
Bc_ctg2_131	B1	5.358705107	Bcer	K	Bcer	FALSE	Bc_Ga0417192_02_rn02	Regulation	SMCOG1071
Bc_ctg2_132	B1	12.20593941	Bcer	EG	Bcer	FALSE	Bc_Ga0417192_02_rn02	Transport	SMCOG1166
Bc_ctg2_133	B1	1.190823357	Bcer	NA	Bcer	FALSE	Bc_Ga0417192_02_rn02	Other	NA
Bc_ctg2_134	B1	5.954116786	Bcer	C	Bcer	FALSE	Bc_Ga0417192_02_rn02	Ancillary biosynthesis	None
Bc_ctg2_135	B1	0.19847056	Bcer	K	Bcer	FALSE	Bc_Ga0417192_02_rn02	Other	NA
Bc_ctg2_136	B1	0.496176399	Bcer	K	Bcer	FALSE	Bc_Ga0417192_02_rn02	Other	NA
Bc_ctg2_137	B1	11.80899829	Bcer	K	Bcer	FALSE	Bc_Ga0417192_02_rn02	Other	NA
Bc_ctg2_139	B1	0.09923528	Bcer	NA	Bcer	FALSE	Bc_Ga0417192_02_rn02	Other	NA
Bc_ctg2_14	B1	37.21322991	Bcer	S	Bcer	FALSE	Bc_Ga0417192_02_rn01	Other	NA
Bc_ctg2_140	B1	0	Bcer	L	Bcer	FALSE	Bc_Ga0417192_02_rn02	Other	SMCOG1132
Bc_ctg2_141	B1	1.885470315	Bcer	L	Bcer	FALSE	Bc_Ga0417192_02_rn02	Other	NA
Bc_ctg2_142	B1	0.09923528	Bcer	L	Bcer	FALSE	Bc_Ga0417192_02_rn02	Other	NA
Bc_ctg2_143	B1	13.29752749	Bcer	V	Bcer	FALSE	Bc_Ga0417192_02_rn02	Ancillary biosynthesis	SMCOG1053
Bc_ctg2_144	B1	0.09923528	Bcer	NA	Bcer	FALSE	Bc_Ga0417192_02_rn02	Other	NA
Bc_ctg2_145	B1	3.96941119	Bcer	L	Bcer	FALSE	NA	NA	NA
Bc_ctg2_146	B1	0.793882238	Bcer	S	Bcer	FALSE	NA	NA	NA
Bc_ctg2_147	B1	2.977058393	Bcer	NA	Bcer	FALSE	NA	NA	NA
Bc_ctg2_148	B1	39.29717079	Bcer	NA	Bcer	FALSE	NA	NA	NA
Bc_ctg2_149	B1	1.786235036	Bcer	CO	Bcer	FALSE	NA	NA	NA
Bc_ctg2_15	B1	51.90005132	Bcer	P	Bcer	FALSE	Bc_Ga0417192_02_rn01	Ancillary biosynthesis	SMCOG1170
Bc_ctg2_150	B1	0	Bcer	P	Bcer	FALSE	NA	NA	NA
Bc_ctg2_152	B1	0	Bcer	NA	Bcer	FALSE	NA	NA	NA
Bc_ctg2_153	B1	5.755646226	Bcer	L	Bcer	FALSE	NA	NA	NA
Bc_ctg2_154	B1	3.473234792	Bcer	NA	Bcer	FALSE	NA	NA	NA
Bc_ctg2_156	B1	0.19847056	Bcer	NA	Bcer	FALSE	NA	NA	NA
Bc_ctg2_157	B1	0.09923528	Bcer	NA	Bcer	FALSE	NA	NA	NA
Bc_ctg2_158	B1	0	Bcer	L	Bcer	FALSE	NA	NA	NA
Bc_ctg2_159	B1	0.694646958	Bcer	NA	Bcer	FALSE	NA	NA	NA
Bc_ctg2_16	B1	12.80135109	Bcer	NA	Bcer	FALSE	Bc_Ga0417192_02_rn01	Other	NA
Bc_ctg2_160	B1	0.09923528	Bcer	S	Bcer	FALSE	NA	NA	NA
Bc_ctg2_161	B1	0.297705839	Bcer	L	Bcer	FALSE	NA	NA	NA
Bc_ctg2_162	B1	0	Bcer	S	Bcer	FALSE	NA	NA	NA
Bc_ctg2_166	B1	0	Bcer	NA	Bcer	FALSE	NA	NA	NA
Bc_ctg2_167	B1	0	Bcer	S	Bcer	FALSE	NA	NA	NA
Bc_ctg2_169	B1	16.86999756	Bcer	G	Bcer	FALSE	NA	NA	NA
Bc_ctg2_17	B1	0.992352798	Bcer	V	Bcer	FALSE	NA	NA	NA
Bc_ctg2_170	B1	1.686999756	Bcer	K	Bcer	FALSE	NA	NA	NA
Bc_ctg2_171	B1	5.457940387	Bcer	NA	Bcer	FALSE	NA	NA	NA
Bc_ctg2_172	B1	3.274764232	Bcer	O	Bcer	FALSE	NA	NA	NA
Bc_ctg2_173	B1	8.038057661	Bcer	K	Bcer	FALSE	NA	NA	NA
Bc_ctg2_174	B1	22.92334962	Bcer	V	Bcer	FALSE	NA	NA	NA
Bc_ctg2_176	B1	4.862528708	Bcer	NA	Bcer	FALSE	NA	NA	NA
Bc_ctg2_177	B1	0.595411679	Bcer	NA	Bcer	FALSE	NA	NA	NA
Bc_ctg2_178	B1	10.91588077	Bcer	V	Bcer	FALSE	NA	NA	NA
Bc_ctg2_179	B1	1.389293917	Bcer	H	Bcer	FALSE	NA	NA	NA
Bc_ctg2_18	B1	12.40440997	Bcer	V	Bcer	FALSE	NA	NA	NA
Bc_ctg2_180	B1	0.694646958	Bcer	NA	Bcer	FALSE	NA	NA	NA
Bc_ctg2_181	B1	0.595411679	Bcer	S	Bcer	FALSE	NA	NA	NA
Bc_ctg2_182	B1	0.297705839	Bcer	NA	Bcer	FALSE	NA	NA	NA

Bc_ctg2_184	B1	0	Bcer	NA	Bcer	FALSE	NA	NA	NA
Bc_ctg2_185	B1	0.396941119	Bcer	L	Bcer	FALSE	NA	NA	NA
Bc_ctg2_186	B1	0.595411679	Bcer	L	Bcer	FALSE	NA	NA	NA
Bc_ctg2_187	B1	0.396941119	Bcer	S	Bcer	FALSE	NA	NA	NA
Bc_ctg2_188	B1	1.587764476	Bcer	NA	Bcer	FALSE	NA	NA	NA
Bc_ctg2_189	B1	3.572470071	Bcer	L	Bcer	FALSE	NA	NA	NA
Bc_ctg2_19	B1	9.526586857	Bcer	T	Bcer	FALSE	NA	NA	NA
Bc_ctg2_190	B1	1.091588077	Bcer	NA	Bcer	FALSE	NA	NA	NA
Bc_ctg2_191	B1	14.38911557	Bcer	NA	Bcer	FALSE	NA	NA	NA
Bc_ctg2_192	B1	105.3878671	Bcer	S	Bcer	FALSE	NA	NA	NA
Bc_ctg2_193	B1	4.862528708	Bcer	S	Bcer	FALSE	NA	NA	NA
Bc_ctg2_194	B1	0	Bcer	CO	Bcer	FALSE	NA	NA	NA
Bc_ctg2_195	B1	0.595411679	Bcer	P	Bcer	FALSE	NA	NA	NA
Bc_ctg2_196	B1	1.290058637	Bcer	NA	Bcer	FALSE	NA	NA	NA
Bc_ctg2_197	B1	1.984705595	Bcer	V	Bcer	FALSE	NA	NA	NA
Bc_ctg2_198	B1	0.793882238	Bcer	S	Bcer	FALSE	NA	NA	NA
Bc_ctg2_199	B1	0.396941119	Bcer	NA	Bcer	FALSE	NA	NA	NA
Bc_ctg2_2	B1	1.290058637	Bcer	M	Bcer	FALSE	Bc_Ga0417192_02_rn01	Core biosynthesis	None
Bc_ctg2_20	B1	17.46540924	Bcer	T	Bcer	FALSE	NA	NA	NA
Bc_ctg2_200	B1	1.786235036	Bcer	NA	Bcer	FALSE	NA	NA	NA
Bc_ctg2_201	B1	2.977058393	Bcer	M	Bcer	FALSE	NA	NA	NA
Bc_ctg2_202	B1	1.587764476	Bcer	NA	Bcer	FALSE	NA	NA	NA
Bc_ctg2_203	B1	0.09923528	Bcer	U	Bcer	FALSE	NA	NA	NA
Bc_ctg2_204	B1	0	Bcer	NA	Bcer	FALSE	NA	NA	NA
Bc_ctg2_205	B1	0	Bcer	NA	Bcer	FALSE	NA	NA	NA
Bc_ctg2_206	B1	0.09923528	Bcer	NA	Bcer	FALSE	NA	NA	NA
Bc_ctg2_207	B1	0.09923528	Bcer	NA	Bcer	FALSE	NA	NA	NA
Bc_ctg2_208	B1	0.19847056	Bcer	NA	Bcer	FALSE	NA	NA	NA
Bc_ctg2_209	B1	0	Bcer	NA	Bcer	FALSE	NA	NA	NA
Bc_ctg2_21	B1	14.58758612	Bcer	T	Bcer	FALSE	NA	NA	NA
Bc_ctg2_210	B1	0	Bcer	L	Bcer	FALSE	NA	NA	NA
Bc_ctg2_211	B1	0.396941119	Bcer	NA	Bcer	FALSE	NA	NA	NA
Bc_ctg2_212	B1	0.496176399	Bcer	S	Bcer	FALSE	NA	NA	NA
Bc_ctg2_213	B1	0.09923528	Bcer	NA	Bcer	FALSE	NA	NA	NA
Bc_ctg2_214	B1	37.70940631	Bcer	S	Bcer	FALSE	NA	NA	NA
Bc_ctg2_215	B1	5.656410946	Bcer	M	Bcer	FALSE	NA	NA	NA
Bc_ctg2_216	B1	0.496176399	Bcer	S	Bcer	FALSE	NA	NA	NA
Bc_ctg2_217	B1	3.96941119	Bcer	S	Bcer	FALSE	NA	NA	NA
Bc_ctg2_218	B1	0	Bcer	S	Bcer	FALSE	NA	NA	NA
Bc_ctg2_22	B1	3.572470071	Bcer	T	Bcer	FALSE	NA	NA	NA
Bc_ctg2_220	B1	0.19847056	Bcer	NA	Bcer	FALSE	NA	NA	NA
Bc_ctg2_221	B1	0.992352798	Bcer	NA	Bcer	FALSE	NA	NA	NA
Bc_ctg2_223	B1	0.793882238	Bcer	NA	Bcer	FALSE	NA	NA	NA
Bc_ctg2_224	B1	0.595411679	Bcer	NA	Bcer	FALSE	NA	NA	NA
Bc_ctg2_225	B1	1.389293917	Bcer	NA	Bcer	FALSE	NA	NA	NA
Bc_ctg2_226	B1	0.19847056	Bcer	NA	Bcer	FALSE	NA	NA	NA
Bc_ctg2_227	B1	0.09923528	Bcer	NA	Bcer	FALSE	NA	NA	NA
Bc_ctg2_228	B1	0.09923528	Bcer	NA	Bcer	FALSE	NA	NA	NA
Bc_ctg2_229	B1	0.297705839	Bcer	NA	Bcer	FALSE	NA	NA	NA
Bc_ctg2_23	B1	3.473234792	Bcer	S	Bcer	FALSE	NA	NA	NA
Bc_ctg2_230	B1	0.19847056	Bcer	NA	Bcer	FALSE	NA	NA	NA
Bc_ctg2_231	B1	1.686999756	Bcer	NA	Bcer	FALSE	NA	NA	NA
Bc_ctg2_232	B1	1.290058637	Bcer	U	Bcer	FALSE	NA	NA	NA
Bc_ctg2_233	B1	0.595411679	Bcer	D	Bcer	FALSE	NA	NA	NA
Bc_ctg2_234	B1	0.19847056	Bcer	U	Bcer	FALSE	NA	NA	NA
Bc_ctg2_235	B1	0	Bcer	NA	Bcer	FALSE	NA	NA	NA
Bc_ctg2_236	B1	2.580117274	Bcer	M	Bcer	FALSE	NA	NA	NA
Bc_ctg2_237	B1	41.08340582	Bcer	GM	Bcer	FALSE	NA	NA	NA
Bc_ctg2_238	B1	0.19847056	Bcer	NA	Bcer	FALSE	NA	NA	NA
Bc_ctg2_239	B1	0.496176399	Bcer	NA	Bcer	FALSE	NA	NA	NA
Bc_ctg2_24	B1	5.854881506	Bcer	V	Bcer	FALSE	NA	NA	NA
Bc_ctg2_240	B1	0.694646958	Bcer	D	Bcer	FALSE	NA	NA	NA

Bc_ctg2_241	B1	1.091588077	Bcer	NA	Bcer	FALSE	NA	NA	NA
Bc_ctg2_242	B1	0.09923528	Bcer	NA	Bcer	FALSE	NA	NA	NA
Bc_ctg2_243	B1	108.9603372	Bcer	NA	Bcer	FALSE	NA	NA	NA
Bc_ctg2_244	B1	164.6313291	Bcer	D	Bcer	FALSE	NA	NA	NA
Bc_ctg2_245	B1	20.34323235	Bcer	NA	Bcer	FALSE	NA	NA	NA
Bc_ctg2_246	B1	9.129645738	Bcer	L	Bcer	FALSE	NA	NA	NA
Bc_ctg2_247	B1	18.6562326	Bcer	NA	Bcer	FALSE	NA	NA	NA
Bc_ctg2_248	B1	0.297705839	Bcer	NA	Bcer	FALSE	NA	NA	NA
Bc_ctg2_249	B1	16.1753506	Bcer	U	Bcer	FALSE	NA	NA	NA
Bc_ctg2_25	B1	34.3354068	Bcer	V	Bcer	FALSE	NA	NA	NA
Bc_ctg2_250	B1	1.389293917	Bcer	J	Bcer	FALSE	NA	NA	NA
Bc_ctg2_251	B1	6.152587345	Bcer	NA	Bcer	FALSE	NA	NA	NA
Bc_ctg2_252	B1	21.13711459	Bcer	NA	Bcer	FALSE	NA	NA	NA
Bc_ctg2_253	B1	0	Bcer	NA	Bcer	FALSE	NA	NA	NA
Bc_ctg2_254	B1	1.389293917	Bcer	NA	Bcer	FALSE	NA	NA	NA
Bc_ctg2_255	B1	40.38875886	Bcer	S	Bcer	FALSE	NA	NA	NA
Bc_ctg2_256	B1	2.282411435	Bcer	NA	Bcer	FALSE	NA	NA	NA
Bc_ctg2_257	B1	0	Bcer	NA	Bcer	FALSE	NA	NA	NA
Bc_ctg2_259	B1	0	Bcer	NA	Bcer	FALSE	NA	NA	NA
Bc_ctg2_26	B1	13.59523333	Bcer	NA	Bcer	FALSE	NA	NA	NA
Bc_ctg2_260	B1	9.824292696	Bcer	NA	Bcer	FALSE	NA	NA	NA
Bc_ctg2_261	B1	4.763293429	Bcer	NA	Bcer	FALSE	NA	NA	NA
Bc_ctg2_262	B1	51.40387492	Bcer	K	Bcer	FALSE	NA	NA	NA
Bc_ctg2_263	B1	15.6791742	Bcer	S	Bcer	FALSE	NA	NA	NA
Bc_ctg2_264	B1	306.3393086	Bcer	S	Bcer	FALSE	NA	NA	NA
Bc_ctg2_265	B1	514.7333961	Bcer	E	Bcer	FALSE	NA	NA	NA
Bc_ctg2_266	B1	784.4548865	Bcer	E	Bcer	FALSE	NA	NA	NA
Bc_ctg2_267	B1	0	Bcer	NA	Bcer	FALSE	NA	NA	NA
Bc_ctg2_268	B1	0	Bcer	L	Bcer	FALSE	NA	NA	NA
Bc_ctg2_269	B1	0.19847056	Bcer	NA	Bcer	FALSE	NA	NA	NA
Bc_ctg2_27	B1	1.885470315	Bcer	NA	Bcer	FALSE	NA	NA	NA
Bc_ctg2_270	B1	70.75475447	Bcer	S	Bcer	FALSE	NA	NA	NA
Bc_ctg2_271	B1	32.54917176	Bcer	S	Bcer	FALSE	NA	NA	NA
Bc_ctg2_272	B1	5.160234548	Bcer	EH	Bcer	FALSE	NA	NA	NA
Bc_ctg2_273	B1	7.740351821	Bcer	NA	Bcer	FALSE	NA	NA	NA
Bc_ctg2_275	B1	20.24399707	Bcer	S	Bcer	FALSE	NA	NA	NA
Bc_ctg2_276	B1	4.664058149	Bcer	L	Bcer	FALSE	NA	NA	NA
Bc_ctg2_278	B1	30.36599561	Bcer	NA	Bcer	FALSE	NA	NA	NA
Bc_ctg2_279	B1	124.9372172	Bcer	K	Bcer	FALSE	NA	NA	NA
Bc_ctg2_28	B1	2.877823113	Bcer	EGP	Bcer	FALSE	NA	NA	NA
Bc_ctg2_280	B1	1.190823357	Bcer	S	Bcer	FALSE	NA	NA	NA
Bc_ctg2_281	B1	7.938822381	Bcer	O	Bcer	FALSE	NA	NA	NA
Bc_ctg2_282	B1	1.091588077	Bcer	S	Bcer	FALSE	NA	NA	NA
Bc_ctg2_283	B1	46.64058149	Bcer	S	Bcer	FALSE	NA	NA	NA
Bc_ctg2_284	B1	80.28134133	Bcer	S	Bcer	FALSE	NA	NA	NA
Bc_ctg2_285	B1	2.480881994	Bcer	NA	Bcer	FALSE	NA	NA	NA
Bc_ctg2_286	B1	3.96941119	Bcer	S	Bcer	FALSE	NA	NA	NA
Bc_ctg2_287	B1	11.21358661	Bcer	NT	Bcer	FALSE	NA	NA	NA
Bc_ctg2_288	B1	0.19847056	Bcer	S	Bcer	FALSE	NA	NA	NA
Bc_ctg2_289	B1	191.1271488	Bcer	NA	Bcer	FALSE	NA	NA	NA
Bc_ctg2_29	B1	2.183176155	Bcer	M	Bcer	FALSE	NA	NA	NA
Bc_ctg2_290	B1	0	Bcer	NA	Bcer	FALSE	NA	NA	NA
Bc_ctg2_291	B1	18.6562326	Bcer	K	Bcer	FALSE	NA	NA	NA
Bc_ctg2_292	B1	0.396941119	Bcer	S	Bcer	FALSE	NA	NA	NA
Bc_ctg2_293	B1	2.977058393	Bcer	O	Bcer	FALSE	NA	NA	NA
Bc_ctg2_294	B1	1.190823357	Bcer	S	Bcer	FALSE	NA	NA	NA
Bc_ctg2_295	B1	4.664058149	Bcer	S	Bcer	FALSE	NA	NA	NA
Bc_ctg2_296	B1	0.992352798	Bcer	NA	Bcer	FALSE	NA	NA	NA
Bc_ctg2_297	B1	4.564822869	Bcer	V	Bcer	FALSE	NA	NA	NA
Bc_ctg2_298	B1	4.564822869	Bcer	NA	Bcer	FALSE	NA	NA	NA
Bc_ctg2_299	B1	3.076293673	Bcer	S	Bcer	FALSE	NA	NA	NA
Bc_ctg2_3	B1	0	Bcer	M	Bcer	FALSE	Bc_Ga0417192_02_rn01	Core biosynthesis	SMCOG1010

Bc_ctg2_30	B1	3.175528952	Bcer	S	Bcer	FALSE	NA	NA	NA
Bc_ctg2_300	B1	21.43482043	Bcer	L	Bcer	FALSE	NA	NA	NA
Bc_ctg2_303	B1	2.183176155	Bcer	O	Bcer	FALSE	NA	NA	NA
Bc_ctg2_304	B1	0	Bcer	NA	Bcer	FALSE	NA	NA	NA
Bc_ctg2_305	B1	1.587764476	Bcer	DZ	Bcer	FALSE	NA	NA	NA
Bc_ctg2_307	B1	0.297705839	Bcer	O	Bcer	FALSE	NA	NA	NA
Bc_ctg2_309	B1	0	Bcer	NA	Bcer	FALSE	NA	NA	NA
Bc_ctg2_31	B1	2.083940875	Bcer	NA	Bcer	FALSE	NA	NA	NA
Bc_ctg2_310	B1	2.778587833	Bcer	GM	Bcer	FALSE	NA	NA	NA
Bc_ctg2_311	B1	4.16788175	Bcer	E	Bcer	FALSE	NA	NA	NA
Bc_ctg2_312	B1	7.740351821	Bcer	K	Bcer	FALSE	NA	NA	NA
Bc_ctg2_313	B1	0.09923528	Bcer	K	Bcer	FALSE	NA	NA	NA
Bc_ctg2_314	B1	1.786235036	Bcer	L	Bcer	FALSE	NA	NA	NA
Bc_ctg2_315	B1	0.496176399	Bcer	S	Bcer	FALSE	NA	NA	NA
Bc_ctg2_316	B1	529.7179234	Bcer	M	Bcer	FALSE	NA	NA	NA
Bc_ctg2_317	B1	54.38093331	Bcer	M	Bcer	FALSE	NA	NA	NA
Bc_ctg2_318	B1	85.14387004	Bcer	S	Bcer	FALSE	NA	NA	NA
Bc_ctg2_319	B1	4.26711703	Bcer	S	Bcer	FALSE	NA	NA	NA
Bc_ctg2_32	B1	0.694646958	Bcer	C	Bcer	FALSE	NA	NA	NA
Bc_ctg2_320	B1	106.6779257	Bcer	S	Bcer	FALSE	NA	NA	NA
Bc_ctg2_322	B1	0	Bcer	NA	Bcer	FALSE	NA	NA	NA
Bc_ctg2_323	B1	19.94629123	Bcer	NA	Bcer	FALSE	NA	NA	NA
Bc_ctg2_324	B1	0	Bcer	NA	Bcer	FALSE	NA	NA	NA
Bc_ctg2_325	B1	0.19847056	Bcer	NA	Bcer	FALSE	NA	NA	NA
Bc_ctg2_326	B1	0	Bcer	L	Bcer	FALSE	NA	NA	NA
Bc_ctg2_327	B1	1.091588077	Bcer	NA	Bcer	FALSE	NA	NA	NA
Bc_ctg2_328	B1	0.992352798	Bcer	H	Bcer	FALSE	NA	NA	NA
Bc_ctg2_329	B1	0.297705839	Bcer	NA	Bcer	FALSE	NA	NA	NA
Bc_ctg2_33	B1	5.060999268	Bcer	IQ	Bcer	TRUE	NA	NA	NA
Bc_ctg2_330	B1	0.396941119	Bcer	T	Bcer	FALSE	NA	NA	NA
Bc_ctg2_331	B1	9.030410458	Bcer	T	Bcer	FALSE	NA	NA	NA
Bc_ctg2_332	B1	29.86981921	Bcer	M	Bcer	FALSE	NA	NA	NA
Bc_ctg2_333	B1	12.99982165	Bcer	K	Bcer	FALSE	NA	NA	NA
Bc_ctg2_334	B1	0.19847056	Bcer	E	Bcer	FALSE	NA	NA	NA
Bc_ctg2_335	B1	1.190823357	Bcer	NA	Bcer	FALSE	NA	NA	NA
Bc_ctg2_336	B1	8.038057661	Bcer	G	Bcer	FALSE	NA	NA	NA
Bc_ctg2_337	B1	8.633469339	Bcer	K	Bcer	FALSE	NA	NA	NA
Bc_ctg2_338	B1	8.43499878	Bcer	E	Bcer	FALSE	NA	NA	NA
Bc_ctg2_339	B1	1.091588077	Bcer	K	Bcer	FALSE	NA	NA	NA
Bc_ctg2_34	B1	0	Bcer	S	Bcer	FALSE	NA	NA	NA
Bc_ctg2_340	B1	0.694646958	Bcer	S	Bcer	FALSE	NA	NA	NA
Bc_ctg2_341	B1	0	Bcer	K	Bcer	FALSE	NA	NA	NA
Bc_ctg2_342	B1	2.282411435	Bcer	E	Bcer	FALSE	NA	NA	NA
Bc_ctg2_343	B1	0.19847056	Bcer	K	Bcer	FALSE	NA	NA	NA
Bc_ctg2_344	B1	13.49599805	Bcer	S	Bcer	FALSE	NA	NA	NA
Bc_ctg2_345	B1	74.42645982	Bcer	K	Bcer	FALSE	NA	NA	NA
Bc_ctg2_346	B1	2.083940875	Bcer	L	Bcer	FALSE	NA	NA	NA
Bc_ctg2_347	B1	3.870175911	Bcer	L	Bcer	FALSE	NA	NA	NA
Bc_ctg2_348	B1	3.96941119	Bcer	NA	Bcer	FALSE	NA	NA	NA
Bc_ctg2_349	B1	13.39676277	Bcer	L	Bcer	FALSE	NA	NA	NA
Bc_ctg2_35	B1	0.19847056	Bcer	NA	Bcer	FALSE	NA	NA	NA
Bc_ctg2_350	B1	7.144940143	Bcer	L	Bcer	FALSE	NA	NA	NA
Bc_ctg2_351	B1	0.694646958	Bcer	K	Bcer	FALSE	NA	NA	NA
Bc_ctg2_352	B1	3.770940631	Bcer	NA	Bcer	FALSE	NA	NA	NA
Bc_ctg2_353	B1	25.40423162	Bcer	NA	Bcer	FALSE	NA	NA	NA
Bc_ctg2_354	B1	12.30517469	Bcer	S	Bcer	FALSE	NA	NA	NA
Bc_ctg2_355	B1	5.557175667	Bcer	NA	Bcer	FALSE	NA	NA	NA
Bc_ctg2_356	B1	3.870175911	Bcer	NA	Bcer	FALSE	NA	NA	NA
Bc_ctg2_359	B1	3.572470071	Bcer	CH	Bcer	FALSE	NA	NA	NA
Bc_ctg2_36	B1	0	Bcer	NA	Bcer	FALSE	NA	NA	NA
Bc_ctg2_360	B1	10.71741021	Bcer	L	Bcer	FALSE	NA	NA	NA
Bc_ctg2_361	B1	1.389293917	Bcer	NA	Bcer	FALSE	NA	NA	NA

Bc_ctg2_362	B1	6.053352065	Bcer	KT	Bcer	FALSE	NA	NA	NA
Bc_ctg2_363	B1	30.46523089	Bcer	K	Bcer	FALSE	NA	NA	NA
Bc_ctg2_364	B1	8.534234059	Bcer	P	Bcer	FALSE	NA	NA	NA
Bc_ctg2_365	B1	3.175528952	Bcer	NA	Bcer	FALSE	NA	NA	NA
Bc_ctg2_366	B1	17.36617396	Bcer	S	Bcer	FALSE	NA	NA	NA
Bc_ctg2_367	B1	7.839587101	Bcer	L	Bcer	FALSE	NA	NA	NA
Bc_ctg2_368	B1	14.78605668	Bcer	S	Bcer	FALSE	NA	NA	NA
Bc_ctg2_369	B1	0.992352798	Bcer	NA	Bcer	FALSE	NA	NA	NA
Bc_ctg2_37	B1	0.992352798	Bcer	NA	Bcer	FALSE	NA	NA	NA
Bc_ctg2_370	B1	0.694646958	Bcer	P	Bcer	FALSE	NA	NA	NA
Bc_ctg2_371	B1	0.992352798	Bcer	NA	Bcer	FALSE	NA	NA	NA
Bc_ctg2_372	B1	18.45776204	Bcer	S	Bcer	FALSE	NA	NA	NA
Bc_ctg2_373	B1	24.21340826	Bcer	NA	Bcer	FALSE	NA	NA	NA
Bc_ctg2_374	B1	0	Bcer	NA	Bcer	FALSE	NA	NA	NA
Bc_ctg2_375	B1	3.572470071	Bcer	NA	Bcer	FALSE	NA	NA	NA
Bc_ctg2_376	B1	58.54881506	Bcer	LO	Bcer	FALSE	NA	NA	NA
Bc_ctg2_377	B1	26.79352554	Bcer	L	Bcer	FALSE	NA	NA	NA
Bc_ctg2_378	B1	50.70922796	Bcer	V	Bcer	FALSE	NA	NA	NA
Bc_ctg2_379	B1	39.19793551	Bcer	V	Bcer	FALSE	NA	NA	NA
Bc_ctg2_38	B1	7.144940143	Bcer	NA	Bcer	FALSE	NA	NA	NA
Bc_ctg2_380	B1	14.09140973	Bcer	NA	Bcer	FALSE	NA	NA	NA
Bc_ctg2_381	B1	60.63275593	Bcer	NA	Bcer	FALSE	NA	NA	NA
Bc_ctg2_382	B1	1.091588077	Bcer	NA	Bcer	FALSE	NA	NA	NA
Bc_ctg2_383	B1	0.297705839	Bcer	NA	Bcer	FALSE	NA	NA	NA
Bc_ctg2_384	B1	165.8221525	Bcer	D	Bcer	FALSE	NA	NA	NA
Bc_ctg2_385	B1	35.82393599	Bcer	NA	Bcer	FALSE	NA	NA	NA
Bc_ctg2_386	B1	219.0122624	Bcer	NA	Bcer	FALSE	NA	NA	NA
Bc_ctg2_387	B1	3.274764232	Bcer	NA	Bcer	FALSE	NA	NA	NA
Bc_ctg2_388	B1	5.259469827	Bcer	NA	Bcer	FALSE	NA	NA	NA
Bc_ctg2_389	B1	44.75511117	Bcer	NA	Bcer	FALSE	Bc_Ga0417192_02_rn03	Other	NA
Bc_ctg2_39	B1	32.54917176	Bcer	NA	Bcer	FALSE	NA	NA	NA
Bc_ctg2_390	B1	73.0371659	Bcer	NA	Bcer	FALSE	Bc_Ga0417192_02_rn03	Other	NA
Bc_ctg2_391	B1	176.1426216	Bcer	NA	Bcer	FALSE	Bc_Ga0417192_02_rn03	Other	NA
Bc_ctg2_392	B1	124.5402761	Bcer	T	Bcer	FALSE	Bc_Ga0417192_02_rn03	Other	NA
Bc_ctg2_393	B1	49.41916932	Bcer	NA	Bcer	FALSE	Bc_Ga0417192_02_rn03	Other	NA
Bc_ctg2_394	B1	76.5104007	Bcer	L	Bcer	FALSE	Bc_Ga0417192_02_rn03	Other	SMCOG1077
Bc_ctg2_395	B1	13.59523333	Bcer	K	Bcer	FALSE	Bc_Ga0417192_02_rn03	Other	SMCOG1148
Bc_ctg2_396	B1	0.09923528	Bcer	K	Bcer	FALSE	Bc_Ga0417192_02_rn03	Other	NA
Bc_ctg2_397	B1	0.496176399	Bcer	NA	Bcer	FALSE	Bc_Ga0417192_02_rn03	Other	NA
Bc_ctg2_398	B1	37.31246519	Bcer	EGP	Bcer	FALSE	Bc_Ga0417192_02_rn03	Transport	SMCOG1005
Bc_ctg2_399	B1	23.81646714	Bcer	UW	Bcer	FALSE	Bc_Ga0417192_02_rn03	Other	NA
Bc_ctg2_4	B1	0.694646958	Bcer	M	Bcer	FALSE	Bc_Ga0417192_02_rn01	Core biosynthesis	SMCOG1010
Bc_ctg2_40	B1	27.38893721	Bcer	L	Bcer	FALSE	NA	NA	NA
Bc_ctg2_400	B1	0.19847056	Bcer	NA	Bcer	FALSE	Bc_Ga0417192_02_rn03	Other	NA
Bc_ctg2_401	B1	1.786235036	Bcer	S	Bcer	FALSE	Bc_Ga0417192_02_rn03	Other	NA
Bc_ctg2_402	B1	0.595411679	Bcer	NA	Bcer	FALSE	Bc_Ga0417192_02_rn03	Other	NA
Bc_ctg2_403	B1	71.15169559	Bcer	NA	Bcer	FALSE	Bc_Ga0417192_02_rn03	Other	NA
Bc_ctg2_404	B1	1.587764476	Bcer	CO	Bcer	FALSE	Bc_Ga0417192_02_rn03	Other	NA
Bc_ctg2_405	B1	14.88529196	Bcer	NA	Bcer	FALSE	Bc_Ga0417192_02_rn03	Other	NA
Bc_ctg2_406	B1	23.61799658	Bcer	E	Bcer	FALSE	Bc_Ga0417192_02_rn03	Ancillary biosynthesis	SMCOG1180
Bc_ctg2_407	B1	0.496176399	Bcer	C	Bcer	FALSE	Bc_Ga0417192_02_rn03	Ancillary biosynthesis	SMCOG1039
Bc_ctg2_408	B1	181.7990325	Bcer	Q	Bcer	TRUE	Bc_Ga0417192_02_rn03	Core biosynthesis	SMCOG1127
Bc_ctg2_409	B1	0	Bcer	NA	Bcer	FALSE	Bc_Ga0417192_02_rn03	Other	NA
Bc_ctg2_41	B1	0.19847056	Bcer	NA	Bcer	FALSE	NA	NA	NA
Bc_ctg2_410	B1	3.274764232	Bcer	NA	Bcer	FALSE	Bc_Ga0417192_02_rn03	Other	NA
Bc_ctg2_411	B1	16.47305644	Bcer	NA	Bcer	FALSE	Bc_Ga0417192_02_rn03	Other	NA
Bc_ctg2_412	B1	46.64058149	Bcer	NA	Bcer	FALSE	Bc_Ga0417192_02_rn03	Other	NA
Bc_ctg2_413	B1	5.457940387	Bcer	NA	Bcer	FALSE	Bc_Ga0417192_02_rn03	Other	NA
Bc_ctg2_414	B1	0.19847056	Bcer	L	Bcer	FALSE	Bc_Ga0417192_02_rn03	Other	NA
Bc_ctg2_415	B1	1.786235036	Bcer	L	Bcer	FALSE	Bc_Ga0417192_02_rn03	Other	NA
Bc_ctg2_416	B1	4.16788175	Bcer	G	Bcer	FALSE	Bc_Ga0417192_02_rn03	Ancillary biosynthesis	SMCOG1101
Bc_ctg2_417	B1	1.190823357	Bcer	NA	Bcer	FALSE	Bc_Ga0417192_02_rn03	Other	NA

Bc_ctg2_418	B1	6.549528464	Bcer	NA	Bcer	FALSE	Bc_Ga0417192_02_rn03	Other	NA
Bc_ctg2_419	B1	6.053352065	Bcer	ST	Bcer	FALSE	Bc_Ga0417192_02_rn03	Other	NA
Bc_ctg2_42	B1	0.09923528	Bcer	NA	Bcer	FALSE	NA	NA	NA
Bc_ctg2_420	B1	20.14476179	Bcer	M	Bcer	FALSE	Bc_Ga0417192_02_rn03	Other	NA
Bc_ctg2_421	B1	0.595411679	Bcer	S	Bcer	FALSE	Bc_Ga0417192_02_rn03	Other	NA
Bc_ctg2_422	B1	8.13729294	Bcer	Q	Bcer	TRUE	Bc_Ga0417192_02_rn03	Core biosynthesis	SMCOG1022
Bc_ctg2_423	B1	25.80117274	Bcer	Q	Bcer	TRUE	Bc_Ga0417192_02_rn03	Core biosynthesis	SMCOG1127
Bc_ctg2_424	B1	12.40440997	Bcer	Q	Bcer	TRUE	Bc_Ga0417192_02_rn03	Core biosynthesis	SMCOG1127
Bc_ctg2_425	B1	0.19847056	Bcer	IQ	Bcer	TRUE	Bc_Ga0417192_02_rn03	Ancillary biosynthesis	SMCOG1147
Bc_ctg2_426	B1	8.831939899	Bcer	I	Bcer	FALSE	Bc_Ga0417192_02_rn03	Ancillary biosynthesis	SMCOG1006
Bc_ctg2_427	B1	12.50364525	Bcer	J	Bcer	FALSE	Bc_Ga0417192_02_rn03	Other	NA
Bc_ctg2_428	B1	12.80135109	Bcer	I	Bcer	FALSE	Bc_Ga0417192_02_rn03	Ancillary biosynthesis	SMCOG1021
Bc_ctg2_429	B1	5.854881506	Bcer	I	Bcer	FALSE	Bc_Ga0417192_02_rn03	Ancillary biosynthesis	SMCOG1095
Bc_ctg2_430	B1	0.396941119	Bcer	IQ	Bcer	TRUE	Bc_Ga0417192_02_rn03	Ancillary biosynthesis	SMCOG1147
Bc_ctg2_431	B1	4.16788175	Bcer	I	Bcer	FALSE	Bc_Ga0417192_02_rn03	Ancillary biosynthesis	SMCOG1006
Bc_ctg2_432	B1	2.977058393	Bcer	Q	Bcer	TRUE	Bc_Ga0417192_02_rn03	Core biosynthesis	SMCOG1002
Bc_ctg2_433	B1	43.26658198	Bcer	Q	Bcer	TRUE	Bc_Ga0417192_02_rn03	Core biosynthesis	SMCOG1127
Bc_ctg2_434	B1	5.457940387	Bcer	C	Bcer	FALSE	Bc_Ga0417192_02_rn03	Ancillary biosynthesis	SMCOG1083
Bc_ctg2_435	B1	14.98452724	Bcer	V	Bcer	FALSE	Bc_Ga0417192_02_rn03	Ancillary biosynthesis	SMCOG1053
Bc_ctg2_436	B1	3.770940631	Bcer	Q	Bcer	TRUE	Bc_Ga0417192_02_rn03	Ancillary biosynthesis	SMCOG1256
Bc_ctg2_437	B1	11.70976301	Bcer	Q	Bcer	TRUE	Bc_Ga0417192_02_rn03	Core biosynthesis	SMCOG1002
Bc_ctg2_438	B1	2.282411435	Bcer	Q	Bcer	TRUE	Bc_Ga0417192_02_rn03	Ancillary biosynthesis	SMCOG1004
Bc_ctg2_439	B1	34.8315832	Bcer	Q	Bcer	TRUE	Bc_Ga0417192_02_rn03	Core biosynthesis	SMCOG1127
Bc_ctg2_440	B1	2.381646714	Bcer	H	Bcer	FALSE	Bc_Ga0417192_02_rn03	Ancillary biosynthesis	SMCOG1012
Bc_ctg2_441	B1	15.77840948	Bcer	K	Bcer	FALSE	Bc_Ga0417192_02_rn03	Regulation	SMCOG1078
Bc_ctg2_442	B1	8.23652822	Bcer	E	Bcer	FALSE	Bc_Ga0417192_02_rn03	Ancillary biosynthesis	SMCOG1056
Bc_ctg2_443	B1	3.671705351	Bcer	S	Bcer	FALSE	Bc_Ga0417192_02_rn03	Ancillary biosynthesis	SMCOG1179
Bc_ctg2_444	B1	9.725057417	Bcer	S	Bcer	FALSE	Bc_Ga0417192_02_rn03	Ancillary biosynthesis	SMCOG1079
Bc_ctg2_445	B1	1.587764476	Bcer	G	Bcer	FALSE	Bc_Ga0417192_02_rn03	Other	NA
Bc_ctg2_446	B1	2.778587833	Bcer	E	Bcer	FALSE	Bc_Ga0417192_02_rn03	Ancillary biosynthesis	SMCOG1114
Bc_ctg2_447	B1	1.389293917	Bcer	E	Bcer	FALSE	Bc_Ga0417192_02_rn03	Ancillary biosynthesis	SMCOG1081
Bc_ctg2_448	B1	4.862528708	Bcer	E	Bcer	FALSE	Bc_Ga0417192_02_rn03	Ancillary biosynthesis	SMCOG1158
Bc_ctg2_449	B1	2.480881994	Bcer	K	Bcer	FALSE	Bc_Ga0417192_02_rn03	Other	NA
Bc_ctg2_45	B1	0	Bcer	NA	Bcer	FALSE	NA	NA	NA
Bc_ctg2_450	B1	0.893117518	Bcer	NA	Bcer	FALSE	Bc_Ga0417192_02_rn03	Other	NA
Bc_ctg2_451	B1	46.24364037	Bcer	NA	Bcer	FALSE	Bc_Ga0417192_02_rn03	Other	NA
Bc_ctg2_453	B1	0.297705839	Bcer	NA	Bcer	FALSE	Bc_Ga0417192_02_rn03	Other	NA
Bc_ctg2_454	B1	0	Bcer	L	Bcer	FALSE	Bc_Ga0417192_02_rn03	Other	NA
Bc_ctg2_455	B1	0.19847056	Bcer	NA	Bcer	FALSE	Bc_Ga0417192_02_rn03	Other	NA
Bc_ctg2_456	B1	0.694646958	Bcer	NA	Bcer	FALSE	Bc_Ga0417192_02_rn03	Other	NA
Bc_ctg2_457	B1	17.06846812	Bcer	NA	Bcer	FALSE	Bc_Ga0417192_02_rn03	Other	NA
Bc_ctg2_458	B1	26.59505498	Bcer	S	Bcer	FALSE	Bc_Ga0417192_02_rn03	Other	NA
Bc_ctg2_459	B1	0	Bcer	E	Bcer	FALSE	Bc_Ga0417192_02_rn03	Other	NA
Bc_ctg2_46	B1	0	Bcer	NA	Bcer	FALSE	NA	NA	NA
Bc_ctg2_460	B1	0	Bcer	G	Bcer	FALSE	Bc_Ga0417192_02_rn03	Ancillary biosynthesis	SMCOG1101
Bc_ctg2_462	B1	0	Bcer	A	Bcer	FALSE	Bc_Ga0417192_02_rn03	Other	NA
Bc_ctg2_464	B1	0	Bcer	NA	Bcer	FALSE	NA	NA	NA
Bc_ctg2_465	B1	2.580117274	Bcer	NA	Bcer	FALSE	NA	NA	NA
Bc_ctg2_466	B1	5.755646226	Bcer	NA	Bcer	FALSE	NA	NA	NA
Bc_ctg2_467	B1	3.473234792	Bcer	NA	Bcer	FALSE	NA	NA	NA
Bc_ctg2_468	B1	0	Bcer	NA	Bcer	FALSE	NA	NA	NA
Bc_ctg2_469	B1	0.793882238	Bcer	L	Bcer	FALSE	NA	NA	NA
Bc_ctg2_47	B1	4.664058149	Bcer	V	Bcer	FALSE	NA	NA	NA
Bc_ctg2_471	B1	8.534234059	Bcer	NA	Bcer	FALSE	NA	NA	NA
Bc_ctg2_472	B1	1.885470315	Bcer	S	Bcer	FALSE	NA	NA	NA
Bc_ctg2_473	B1	23.91570242	Bcer	S	Bcer	FALSE	NA	NA	NA
Bc_ctg2_474	B1	14.58758612	Bcer	NA	Bcer	FALSE	NA	NA	NA
Bc_ctg2_475	B1	0	Bcer	L	Bcer	FALSE	NA	NA	NA
Bc_ctg2_476	B1	4.16788175	Bcer	L	Bcer	FALSE	NA	NA	NA
Bc_ctg2_477	B1	0	Bcer	NA	Bcer	FALSE	NA	NA	NA
Bc_ctg2_478	B1	0	Bcer	L	Bcer	FALSE	NA	NA	NA
Bc_ctg2_479	B1	1.091588077	Bcer	L	Bcer	FALSE	NA	NA	NA

Bc_ctg2_48	B1	8.931175179	Bcer	M	Bcer	FALSE	NA	NA	NA
Bc_ctg2_480	B1	1.686999756	Bcer	L	Bcer	FALSE	NA	NA	NA
Bc_ctg2_481	B1	0.793882238	Bcer	L	Bcer	FALSE	NA	NA	NA
Bc_ctg2_482	B1	0.297705839	Bcer	NA	Bcer	FALSE	NA	NA	NA
Bc_ctg2_483	B1	0.595411679	Bcer	NA	Bcer	FALSE	NA	NA	NA
Bc_ctg2_484	B1	0.396941119	Bcer	NA	Bcer	FALSE	NA	NA	NA
Bc_ctg2_485	B1	0.496176399	Bcer	O	Bcer	FALSE	NA	NA	NA
Bc_ctg2_486	B1	0.793882238	Bcer	K	Bcer	FALSE	NA	NA	NA
Bc_ctg2_487	B1	30.26676033	Bcer	S	Bcer	FALSE	NA	NA	NA
Bc_ctg2_488	B1	0.297705839	Bcer	NA	Bcer	FALSE	NA	NA	NA
Bc_ctg2_489	B1	0	Bcer	NA	Bcer	FALSE	NA	NA	NA
Bc_ctg2_49	B1	0.496176399	Bcer	E	Bcer	FALSE	NA	NA	NA
Bc_ctg2_490	B1	2.778587833	Bcer	S	Bcer	FALSE	NA	NA	NA
Bc_ctg2_491	B1	30.06828977	Bcer	NA	Bcer	FALSE	Bc_Ga0417192_02_rn04	Other	NA
Bc_ctg2_492	B1	3.870175911	Bcer	NA	Bcer	FALSE	Bc_Ga0417192_02_rn04	Other	NA
Bc_ctg2_493	B1	1.984705595	Bcer	NA	Bcer	FALSE	Bc_Ga0417192_02_rn04	Other	NA
Bc_ctg2_494	B1	6.152587345	Bcer	NA	Bcer	FALSE	Bc_Ga0417192_02_rn04	Other	NA
Bc_ctg2_495	B1	65.29681408	Bcer	L	Bcer	FALSE	Bc_Ga0417192_02_rn04	Other	NA
Bc_ctg2_496	B1	152.1276839	Bcer	L	Bcer	FALSE	Bc_Ga0417192_02_rn04	Other	NA
Bc_ctg2_497	B1	102.1131029	Bcer	K	Bcer	FALSE	Bc_Ga0417192_02_rn04	Other	NA
Bc_ctg2_498	B1	45.64822869	Bcer	EGP	Bcer	FALSE	Bc_Ga0417192_02_rn04	Transport	SMCOG1020
Bc_ctg2_499	B1	42.47269974	Bcer	KLT	Bcer	FALSE	Bc_Ga0417192_02_rn04	Core biosynthesis	SMCOG1070
Bc_ctg2_5	B1	0.297705839	Bcer	E	Bcer	FALSE	Bc_Ga0417192_02_rn01	Core biosynthesis	SMCOG1056
Bc_ctg2_50	B1	0.19847056	Bcer	NA	Bcer	FALSE	NA	NA	NA
Bc_ctg2_500	B1	4.862528708	Bcer	J	Bcer	FALSE	Bc_Ga0417192_02_rn04	Other	NA
Bc_ctg2_504	B1	0.793882238	Bcer	NA	Bcer	FALSE	Bc_Ga0417192_02_rn04	Other	NA
Bc_ctg2_505	B1	107.8687491	Bcer	S	Bcer	FALSE	Bc_Ga0417192_02_rn04	Other	NA
Bc_ctg2_506	B1	25.60270218	Bcer	L	Bcer	FALSE	Bc_Ga0417192_02_rn04	Other	SMCOG1077
Bc_ctg2_507	B1	1.587764476	Bcer	K	Bcer	FALSE	Bc_Ga0417192_02_rn04	Regulation	SMCOG1058
Bc_ctg2_508	B1	23.12182018	Bcer	L	Bcer	FALSE	Bc_Ga0417192_02_rn04	Other	NA
Bc_ctg2_509	B1	2.679352554	Bcer	NA	Bcer	FALSE	Bc_Ga0417192_02_rn04	Other	NA
Bc_ctg2_51	B1	2.580117274	Bcer	L	Bcer	FALSE	NA	NA	NA
Bc_ctg2_510	B1	8.3357635	Bcer	S	Bcer	FALSE	Bc_Ga0417192_02_rn04	Other	NA
Bc_ctg2_511	B1	1.587764476	Bcer	NA	Bcer	FALSE	NA	NA	NA
Bc_ctg2_512	B1	1.686999756	Bcer	NA	Bcer	FALSE	NA	NA	NA
Bc_ctg2_513	B1	1.290058637	Bcer	M	Bcer	FALSE	NA	NA	NA
Bc_ctg2_52	B1	1.389293917	Bcer	M	Bcer	FALSE	NA	NA	NA
Bc_ctg2_53	B1	3.274764232	Bcer	M	Bcer	FALSE	NA	NA	NA
Bc_ctg2_54	B1	0.09923528	Bcer	M	Bcer	FALSE	NA	NA	NA
Bc_ctg2_55	B1	1.587764476	Bcer	NA	Bcer	FALSE	NA	NA	NA
Bc_ctg2_56	B1	0.09923528	Bcer	V	Bcer	FALSE	NA	NA	NA
Bc_ctg2_57	B1	7.045704863	Bcer	CH	Bcer	FALSE	NA	NA	NA
Bc_ctg2_58	B1	4.465587589	Bcer	E	Bcer	FALSE	NA	NA	NA
Bc_ctg2_59	B1	2.183176155	Bcer	S	Bcer	FALSE	NA	NA	NA
Bc_ctg2_6	B1	0.09923528	Bcer	M	Bcer	FALSE	Bc_Ga0417192_02_rn01	Other	NA
Bc_ctg2_60	B1	2.183176155	Bcer	K	Bcer	FALSE	NA	NA	NA
Bc_ctg2_61	B1	18.85470315	Bcer	D	Bcer	FALSE	NA	NA	NA
Bc_ctg2_62	B1	1.190823357	Bcer	NA	Bcer	FALSE	NA	NA	NA
Bc_ctg2_63	B1	2.480881994	Bcer	K	Bcer	FALSE	NA	NA	NA
Bc_ctg2_64	B1	3.671705351	Bcer	E	Bcer	FALSE	NA	NA	NA
Bc_ctg2_65	B1	2.977058393	Bcer	L	Bcer	FALSE	NA	NA	NA
Bc_ctg2_66	B1	20.64093819	Bcer	NA	Bcer	FALSE	NA	NA	NA
Bc_ctg2_67	B1	0.496176399	Bcer	NA	Bcer	FALSE	NA	NA	NA
Bc_ctg2_68	B1	0.09923528	Bcer	NA	Bcer	FALSE	NA	NA	NA
Bc_ctg2_69	B1	0	Bcer	NA	Bcer	FALSE	NA	NA	NA
Bc_ctg2_7	B1	0.19847056	Bcer	M	Bcer	FALSE	Bc_Ga0417192_02_rn01	Core biosynthesis	None
Bc_ctg2_70	B1	0.09923528	Bcer	M	Bcer	FALSE	NA	NA	NA
Bc_ctg2_71	B1	0.793882238	Bcer	NA	Bcer	FALSE	NA	NA	NA
Bc_ctg2_72	B1	13.09905693	Bcer	NA	Bcer	FALSE	NA	NA	NA
Bc_ctg2_73	B1	20.24399707	Bcer	S	Bcer	FALSE	NA	NA	NA
Bc_ctg2_74	B1	30.16752505	Bcer	S	Bcer	FALSE	NA	NA	NA
Bc_ctg2_75	B1	2.580117274	Bcer	NA	Bcer	FALSE	NA	NA	NA

Bc_ctg2_76	B1	2.679352554	Bcer	NA	Bcer	FALSE	NA	NA	NA
Bc_ctg2_77	B1	15.77840948	Bcer	KT	Bcer	FALSE	NA	NA	NA
Bc_ctg2_78	B1	3.671705351	Bcer	K	Bcer	FALSE	NA	NA	NA
Bc_ctg2_79	B1	1.686999756	Bcer	NA	Bcer	FALSE	NA	NA	NA
Bc_ctg2_8	B1	0.297705839	Bcer	J	Bcer	FALSE	Bc_Ga0417192_02_rn01	Other	NA
Bc_ctg2_80	B1	11.61052773	Bcer	K	Bcer	FALSE	NA	NA	NA
Bc_ctg2_81	B1	25.80117274	Bcer	M	Bcer	FALSE	NA	NA	NA
Bc_ctg2_82	B1	85.44157587	Bcer	S	Bcer	FALSE	NA	NA	NA
Bc_ctg2_83	B1	1.091588077	Bcer	K	Bcer	FALSE	NA	NA	NA
Bc_ctg2_84	B1	0.694646958	Bcer	E	Bcer	FALSE	NA	NA	NA
Bc_ctg2_86	B1	1.190823357	Bcer	S	Bcer	FALSE	NA	NA	NA
Bc_ctg2_87	B1	0.297705839	Bcer	K	Bcer	FALSE	NA	NA	NA
Bc_ctg2_88	B1	148.3567432	Bcer	M	Bcer	FALSE	NA	NA	NA
Bc_ctg2_89	B1	6.946469583	Bcer	S	Bcer	FALSE	NA	NA	NA
Bc_ctg2_9	B1	2.083940875	Bcer	M	Bcer	FALSE	Bc_Ga0417192_02_rn01	Other	NA
Bc_ctg2_90	B1	5.557175667	Bcer	NA	Bcer	FALSE	NA	NA	NA
Bc_ctg2_91	B1	3.770940631	Bcer	V	Bcer	FALSE	NA	NA	NA
Bc_ctg2_93	B1	4.366352309	Bcer	I	Bcer	FALSE	NA	NA	NA
Bc_ctg2_94	B1	1.488529196	Bcer	NA	Bcer	FALSE	NA	NA	NA
Bc_ctg2_95	B1	825.7367629	Bcer	S	Bcer	FALSE	NA	NA	NA
Bc_ctg2_96	B1	4.564822869	Bcer	IQ	Bcer	TRUE	NA	NA	NA
Bc_ctg2_98	B1	0	Bcer	NA	Bcer	FALSE	Bc_Ga0417192_02_rn02	Other	NA
Bc_ctg2_99	B1	5.656410946	Bcer	EG	Bcer	FALSE	Bc_Ga0417192_02_rn02	Transport	SMCOG1166
Bc_ctg3_1	B1	16.57229172	Bcer	P	Bcer	FALSE	NA	NA	NA
Bc_ctg3_10	B1	15.87764476	Bcer	EGP	Bcer	FALSE	Bc_Ga0417192_03_rn01	Transport	SMCOG1106
Bc_ctg3_100	B1	2.083940875	Bcer	V	Bcer	FALSE	NA	NA	NA
Bc_ctg3_101	B1	0	Bcer	S	Bcer	FALSE	NA	NA	NA
Bc_ctg3_102	B1	0.694646958	Bcer	K	Bcer	FALSE	NA	NA	NA
Bc_ctg3_103	B1	4.664058149	Bcer	K	Bcer	FALSE	NA	NA	NA
Bc_ctg3_104	B1	0.19847056	Bcer	L	Bcer	FALSE	NA	NA	NA
Bc_ctg3_105	B1	0.396941119	Bcer	L	Bcer	FALSE	NA	NA	NA
Bc_ctg3_106	B1	0.19847056	Bcer	S	Bcer	FALSE	NA	NA	NA
Bc_ctg3_107	B1	12.60288053	Bcer	V	Bcer	FALSE	NA	NA	NA
Bc_ctg3_108	B1	1.587764476	Bcer	V	Bcer	FALSE	NA	NA	NA
Bc_ctg3_109	B1	0.793882238	Bcer	V	Bcer	FALSE	NA	NA	NA
Bc_ctg3_11	B1	0.396941119	Bcer	NA	Bcer	FALSE	Bc_Ga0417192_03_rn01	Other	NA
Bc_ctg3_110	B1	5.060999268	Bcer	S	Bcer	FALSE	NA	NA	NA
Bc_ctg3_111	B1	1.686999756	Bcer	K	Bcer	FALSE	NA	NA	NA
Bc_ctg3_112	B1	149.349096	Bcer	M	Bcer	FALSE	NA	NA	NA
Bc_ctg3_113	B1	22.03023211	Bcer	M	Bcer	FALSE	NA	NA	NA
Bc_ctg3_114	B1	11.80899829	Bcer	S	Bcer	FALSE	NA	NA	NA
Bc_ctg3_115	B1	431.0780553	Bcer	G	Bcer	FALSE	NA	NA	NA
Bc_ctg3_116	B1	136.9446861	Bcer	G	Bcer	FALSE	NA	NA	NA
Bc_ctg3_117	B1	303.1637797	Bcer	G	Bcer	FALSE	NA	NA	NA
Bc_ctg3_118	B1	13.79370389	Bcer	U	Bcer	FALSE	NA	NA	NA
Bc_ctg3_119	B1	1.587764476	Bcer	G	Bcer	FALSE	NA	NA	NA
Bc_ctg3_12	B1	2.778587833	Bcer	Q	Bcer	TRUE	Bc_Ga0417192_03_rn01	Ancillary biosynthesis	SMCOG1027
Bc_ctg3_120	B1	18.25929148	Bcer	S	Bcer	FALSE	NA	NA	NA
Bc_ctg3_121	B1	10.51893965	Bcer	G	Bcer	FALSE	NA	NA	NA
Bc_ctg3_122	B1	1079.878314	Bcer	C	Bcer	FALSE	NA	NA	NA
Bc_ctg3_123	B1	93.67810409	Bcer	G	Bcer	FALSE	NA	NA	NA
Bc_ctg3_124	B1	6.946469583	Bcer	NA	Bcer	FALSE	NA	NA	NA
Bc_ctg3_126	B1	5.160234548	Bcer	S	Bcer	FALSE	NA	NA	NA
Bc_ctg3_128	B1	4.763293429	Bcer	K	Bcer	FALSE	NA	NA	NA
Bc_ctg3_129	B1	7.641116542	Bcer	V	Bcer	FALSE	NA	NA	NA
Bc_ctg3_13	B1	2.381646714	Bcer	G	Bcer	FALSE	Bc_Ga0417192_03_rn01	Ancillary biosynthesis	SMCOG1235
Bc_ctg3_130	B1	3.274764232	Bcer	S	Bcer	FALSE	NA	NA	NA
Bc_ctg3_131	B1	10.81664549	Bcer	NA	Bcer	FALSE	NA	NA	NA
Bc_ctg3_132	B1	13.09905693	Bcer	U	Bcer	FALSE	NA	NA	NA
Bc_ctg3_133	B1	22.03023211	Bcer	NA	Bcer	FALSE	NA	NA	NA
Bc_ctg3_134	B1	29.27440753	Bcer	L	Bcer	FALSE	NA	NA	NA
Bc_ctg3_135	B1	1.686999756	Bcer	V	Bcer	FALSE	NA	NA	NA

Bc_ctg3_136	B1	0.992352798	Bcer	V	Bcer	FALSE	NA	NA	NA
Bc_ctg3_137	B1	0.793882238	Bcer	O	Bcer	FALSE	NA	NA	NA
Bc_ctg3_139	B1	16.57229172	Bcer	T	Bcer	FALSE	NA	NA	NA
Bc_ctg3_14	B1	14.6868214	Bcer	EGP	Bcer	FALSE	Bc_Ga0417192_03_rn01	Transport	SMCOG1020
Bc_ctg3_140	B1	51.80081604	Bcer	T	Bcer	FALSE	NA	NA	NA
Bc_ctg3_141	B1	0	Bcer	NA	Bcer	FALSE	NA	NA	NA
Bc_ctg3_142	B1	12.30517469	Bcer	S	Bcer	FALSE	NA	NA	NA
Bc_ctg3_143	B1	124.143335	Bcer	S	Bcer	FALSE	NA	NA	NA
Bc_ctg3_144	B1	50.90769852	Bcer	G	Bcer	FALSE	NA	NA	NA
Bc_ctg3_145	B1	21.93099683	Bcer	S	Bcer	FALSE	NA	NA	NA
Bc_ctg3_146	B1	190.829443	Bcer	Q	Bcer	TRUE	NA	NA	NA
Bc_ctg3_147	B1	112.0366309	Bcer	H	Bcer	FALSE	NA	NA	NA
Bc_ctg3_148	B1	318.2475422	Bcer	F	Bcer	FALSE	Bc_Ga0417192_03_rn02	Other	NA
Bc_ctg3_149	B1	497.3672222	Bcer	J	Bcer	FALSE	Bc_Ga0417192_03_rn02	Other	NA
Bc_ctg3_15	B1	0	Bcer	L	Bcer	FALSE	Bc_Ga0417192_03_rn01	Other	NA
Bc_ctg3_150	B1	81.27369412	Bcer	NA	Bcer	FALSE	Bc_Ga0417192_03_rn02	Ancillary biosynthesis	None
Bc_ctg3_151	B1	612.579382	Bcer	NA	Bcer	FALSE	Bc_Ga0417192_03_rn02	Other	NA
Bc_ctg3_152	B1	83.65534084	Bcer	NA	Bcer	FALSE	Bc_Ga0417192_03_rn02	Other	NA
Bc_ctg3_153	B1	201.2491474	Bcer	T	Bcer	FALSE	Bc_Ga0417192_03_rn02	Other	SMCOG1226
Bc_ctg3_154	B1	184.081444	Bcer	NA	Bcer	FALSE	Bc_Ga0417192_03_rn02	Other	NA
Bc_ctg3_155	B1	368.0636526	Bcer	EGP	Bcer	FALSE	Bc_Ga0417192_03_rn02	Transport	SMCOG1020
Bc_ctg3_156	B1	7.641116542	Bcer	NA	Bcer	FALSE	Bc_Ga0417192_03_rn02	Other	NA
Bc_ctg3_158	B1	12.40440997	Bcer	S	Bcer	FALSE	Bc_Ga0417192_03_rn02	Other	NA
Bc_ctg3_159	B1	47.43446373	Bcer	C	Bcer	FALSE	Bc_Ga0417192_03_rn02	Ancillary biosynthesis	None
Bc_ctg3_16	B1	0.09923528	Bcer	NA	Bcer	FALSE	Bc_Ga0417192_03_rn01	Other	NA
Bc_ctg3_160	B1	0.496176399	Bcer	S	Bcer	FALSE	Bc_Ga0417192_03_rn02	Core biosynthesis	None
Bc_ctg3_161	B1	32.25146592	Bcer	OS	Bcer	FALSE	Bc_Ga0417192_03_rn02	Ancillary biosynthesis	None
Bc_ctg3_162	B1	17.1677034	Bcer	O	Bcer	FALSE	Bc_Ga0417192_03_rn02	Other	NA
Bc_ctg3_163	B1	16.57229172	Bcer	E	Bcer	FALSE	Bc_Ga0417192_03_rn02	Ancillary biosynthesis	SMCOG1038
Bc_ctg3_164	B1	320.4307183	Bcer	E	Bcer	FALSE	Bc_Ga0417192_03_rn02	Ancillary biosynthesis	SMCOG1081
Bc_ctg3_165	B1	299.392839	Bcer	E	Bcer	FALSE	Bc_Ga0417192_03_rn02	Other	NA
Bc_ctg3_166	B1	4.16788175	Bcer	E	Bcer	FALSE	NA	NA	NA
Bc_ctg3_167	B1	6.053352065	Bcer	S	Bcer	FALSE	NA	NA	NA
Bc_ctg3_168	B1	4.664058149	Bcer	C	Bcer	FALSE	NA	NA	NA
Bc_ctg3_169	B1	1.488529196	Bcer	P	Bcer	FALSE	NA	NA	NA
Bc_ctg3_17	B1	0	Bcer	K	Bcer	FALSE	Bc_Ga0417192_03_rn01	Other	NA
Bc_ctg3_170	B1	51.99928659	Bcer	C	Bcer	FALSE	NA	NA	NA
Bc_ctg3_171	B1	4.16788175	Bcer	L	Bcer	FALSE	NA	NA	NA
Bc_ctg3_172	B1	11.41205717	Bcer	L	Bcer	FALSE	NA	NA	NA
Bc_ctg3_173	B1	2.977058393	Bcer	NU	Bcer	FALSE	NA	NA	NA
Bc_ctg3_174	B1	23.32029074	Bcer	L	Bcer	FALSE	NA	NA	NA
Bc_ctg3_175	B1	23.81646714	Bcer	L	Bcer	FALSE	NA	NA	NA
Bc_ctg3_176	B1	26.39658442	Bcer	S	Bcer	FALSE	NA	NA	NA
Bc_ctg3_177	B1	13.19829221	Bcer	F	Bcer	FALSE	NA	NA	NA
Bc_ctg3_178	B1	6.450293184	Bcer	O	Bcer	FALSE	NA	NA	NA
Bc_ctg3_179	B1	11.90823357	Bcer	C	Bcer	FALSE	NA	NA	NA
Bc_ctg3_18	B1	2.083940875	Bcer	L	Bcer	FALSE	Bc_Ga0417192_03_rn01	Other	NA
Bc_ctg3_180	B1	1.290058637	Bcer	NA	Bcer	FALSE	NA	NA	NA
Bc_ctg3_181	B1	3278.535173	Bcer	C	Bcer	FALSE	NA	NA	NA
Bc_ctg3_182	B1	51.20540436	Bcer	NA	Bcer	FALSE	NA	NA	NA
Bc_ctg3_183	B1	36.22087711	Bcer	NA	Bcer	FALSE	NA	NA	NA
Bc_ctg3_184	B1	81.17445884	Bcer	K	Bcer	FALSE	NA	NA	NA
Bc_ctg3_185	B1	40.38875886	Bcer	NA	Bcer	FALSE	NA	NA	NA
Bc_ctg3_186	B1	292.5456047	Bcer	NA	Bcer	FALSE	NA	NA	NA
Bc_ctg3_187	B1	55.47252139	Bcer	K	Bcer	FALSE	NA	NA	NA
Bc_ctg3_188	B1	10.81664549	Bcer	NA	Bcer	FALSE	NA	NA	NA
Bc_ctg3_189	B1	0.496176399	Bcer	K	Bcer	FALSE	NA	NA	NA
Bc_ctg3_19	B1	0	Bcer	NA	Bcer	FALSE	Bc_Ga0417192_03_rn01	Other	NA
Bc_ctg3_190	B1	3.373999512	Bcer	NA	Bcer	FALSE	NA	NA	NA
Bc_ctg3_191	B1	5.755646226	Bcer	S	Bcer	FALSE	NA	NA	NA
Bc_ctg3_192	B1	1.686999756	Bcer	NA	Bcer	FALSE	NA	NA	NA
Bc_ctg3_193	B1	3.572470071	Bcer	NA	Bcer	FALSE	NA	NA	NA

Bc_ctg3_194	B1	6.648763744	Bcer	NA	Bcer	FALSE	NA	NA	NA
Bc_ctg3_195	B1	179.3181505	Bcer	T	Bcer	FALSE	NA	NA	NA
Bc_ctg3_196	B1	119.5785121	Bcer	L	Bcer	FALSE	NA	NA	NA
Bc_ctg3_197	B1	19.35087955	Bcer	NA	Bcer	FALSE	NA	NA	NA
Bc_ctg3_198	B1	2.679352554	Bcer	S	Bcer	FALSE	NA	NA	NA
Bc_ctg3_199	B1	4.26711703	Bcer	K	Bcer	FALSE	NA	NA	NA
Bc_ctg3_2	B1	95.96051553	Bcer	P	Bcer	FALSE	NA	NA	NA
Bc_ctg3_20	B1	0	Bcer	NA	Bcer	FALSE	Bc_Ga0417192_03_rn01	Other	NA
Bc_ctg3_200	B1	15.97688004	Bcer	NA	Bcer	FALSE	NA	NA	NA
Bc_ctg3_201	B1	23.5187613	Bcer	NA	Bcer	FALSE	NA	NA	NA
Bc_ctg3_202	B1	63.80828489	Bcer	P	Bcer	FALSE	NA	NA	NA
Bc_ctg3_203	B1	15.77840948	Bcer	NA	Bcer	FALSE	NA	NA	NA
Bc_ctg3_204	B1	6.946469583	Bcer	K	Bcer	FALSE	NA	NA	NA
Bc_ctg3_206	B1	0.09923528	Bcer	NA	Bcer	FALSE	NA	NA	NA
Bc_ctg3_207	B1	3.175528952	Bcer	Q	Bcer	TRUE	NA	NA	NA
Bc_ctg3_208	B1	37.51093575	Bcer	S	Bcer	FALSE	NA	NA	NA
Bc_ctg3_209	B1	2.580117274	Bcer	NA	Bcer	FALSE	NA	NA	NA
Bc_ctg3_21	B1	3.274764232	Bcer	M	Bcer	FALSE	Bc_Ga0417192_03_rn01	Core biosynthesis	SMCOG1010
Bc_ctg3_212	B1	5.358705107	Bcer	S	Bcer	FALSE	NA	NA	NA
Bc_ctg3_213	B1	1.885470315	Bcer	NA	Bcer	FALSE	NA	NA	NA
Bc_ctg3_214	B1	4.06864647	Bcer	E	Bcer	FALSE	NA	NA	NA
Bc_ctg3_215	B1	5.060999268	Bcer	E	Bcer	FALSE	NA	NA	NA
Bc_ctg3_216	B1	0.396941119	Bcer	L	Bcer	FALSE	NA	NA	NA
Bc_ctg3_217	B1	2.083940875	Bcer	K	Bcer	FALSE	NA	NA	NA
Bc_ctg3_218	B1	2.282411435	Bcer	S	Bcer	FALSE	NA	NA	NA
Bc_ctg3_219	B1	0.893117518	Bcer	S	Bcer	FALSE	NA	NA	NA
Bc_ctg3_22	B1	3.274764232	Bcer	M	Bcer	FALSE	Bc_Ga0417192_03_rn01	Core biosynthesis	SMCOG1010
Bc_ctg3_220	B1	3.274764232	Bcer	M	Bcer	FALSE	NA	NA	NA
Bc_ctg3_221	B1	0	Bcer	L	Bcer	FALSE	NA	NA	NA
Bc_ctg3_222	B1	0.595411679	Bcer	L	Bcer	FALSE	NA	NA	NA
Bc_ctg3_223	B1	12.00746885	Bcer	NA	Bcer	FALSE	NA	NA	NA
Bc_ctg3_224	B1	7.839587101	Bcer	C	Bcer	FALSE	NA	NA	NA
Bc_ctg3_225	B1	14.38911557	Bcer	K	Bcer	FALSE	NA	NA	NA
Bc_ctg3_226	B1	124.5402761	Bcer	GKT	Bcer	FALSE	NA	NA	NA
Bc_ctg3_227	B1	1037.70332	Bcer	G	Bcer	FALSE	NA	NA	NA
Bc_ctg3_228	B1	607.2206769	Bcer	G	Bcer	FALSE	NA	NA	NA
Bc_ctg3_229	B1	0.595411679	Bcer	NA	Bcer	FALSE	NA	NA	NA
Bc_ctg3_23	B1	1.290058637	Bcer	M	Bcer	FALSE	Bc_Ga0417192_03_rn01	Core biosynthesis	None
Bc_ctg3_230	B1	27.28970193	Bcer	P	Bcer	FALSE	NA	NA	NA
Bc_ctg3_24	B1	3.770940631	Bcer	M	Bcer	FALSE	Bc_Ga0417192_03_rn01	Ancillary biosynthesis	SMCOG1064
Bc_ctg3_25	B1	7.045704863	Bcer	I	Bcer	FALSE	Bc_Ga0417192_03_rn01	Core biosynthesis	SMCOG1022
Bc_ctg3_26	B1	2.679352554	Bcer	E	Bcer	FALSE	Bc_Ga0417192_03_rn01	Other	NA
Bc_ctg3_27	B1	2.083940875	Bcer	NA	Bcer	FALSE	Bc_Ga0417192_03_rn01	Other	NA
Bc_ctg3_28	B1	6.152587345	Bcer	K	Bcer	FALSE	Bc_Ga0417192_03_rn01	Other	NA
Bc_ctg3_29	B1	0.595411679	Bcer	I	Bcer	FALSE	Bc_Ga0417192_03_rn01	Ancillary biosynthesis	SMCOG1270
Bc_ctg3_3	B1	19.64858539	Bcer	E	Bcer	FALSE	NA	NA	NA
Bc_ctg3_30	B1	0.694646958	Bcer	IQ	Bcer	TRUE	Bc_Ga0417192_03_rn01	Ancillary biosynthesis	None
Bc_ctg3_31	B1	0	Bcer	IQ	Bcer	TRUE	Bc_Ga0417192_03_rn01	Ancillary biosynthesis	SMCOG1001
Bc_ctg3_32	B1	1.190823357	Bcer	I	Bcer	FALSE	Bc_Ga0417192_03_rn01	Ancillary biosynthesis	SMCOG1021
Bc_ctg3_33	B1	0.297705839	Bcer	S	Bcer	FALSE	Bc_Ga0417192_03_rn01	Other	NA
Bc_ctg3_34	B1	0.595411679	Bcer	V	Bcer	FALSE	Bc_Ga0417192_03_rn01	Transport	SMCOG1065
Bc_ctg3_35	B1	0.09923528	Bcer	V	Bcer	FALSE	Bc_Ga0417192_03_rn01	Transport	SMCOG1000
Bc_ctg3_36	B1	1.686999756	Bcer	S	Bcer	FALSE	Bc_Ga0417192_03_rn01	Other	NA
Bc_ctg3_37	B1	48.5260518	Bcer	S	Bcer	FALSE	Bc_Ga0417192_03_rn01	Other	NA
Bc_ctg3_38	B1	14.58758612	Bcer	NA	Bcer	FALSE	Bc_Ga0417192_03_rn01	Other	NA
Bc_ctg3_39	B1	8.931175179	Bcer	L	Bcer	FALSE	Bc_Ga0417192_03_rn01	Other	SMCOG1077
Bc_ctg3_4	B1	17.1677034	Bcer	H	Bcer	FALSE	NA	NA	NA
Bc_ctg3_40	B1	0	Bcer	NA	Bcer	FALSE	Bc_Ga0417192_03_rn01	Other	NA
Bc_ctg3_41	B1	5.160234548	Bcer	E	Bcer	FALSE	Bc_Ga0417192_03_rn01	Other	NA
Bc_ctg3_42	B1	8.633469339	Bcer	E	Bcer	FALSE	Bc_Ga0417192_03_rn01	Ancillary biosynthesis	SMCOG1153
Bc_ctg3_43	B1	5.557175667	Bcer	E	Bcer	FALSE	Bc_Ga0417192_03_rn01	Other	NA
Bc_ctg3_44	B1	0.297705839	Bcer	P	Bcer	FALSE	NA	NA	NA

Bc_ctg3_45	B1	0.595411679	Bcer	E	Bcer	FALSE	NA	NA	NA
Bc_ctg3_46	B1	2.183176155	Bcer	S	Bcer	FALSE	NA	NA	NA
Bc_ctg3_47	B1	0.595411679	Bcer	NA	Bcer	FALSE	NA	NA	NA
Bc_ctg3_48	B1	0.09923528	Bcer	NA	Bcer	FALSE	NA	NA	NA
Bc_ctg3_49	B1	1.786235036	Bcer	NA	Bcer	FALSE	NA	NA	NA
Bc_ctg3_5	B1	10.91588077	Bcer	K	Bcer	FALSE	NA	NA	NA
Bc_ctg3_50	B1	3.175528952	Bcer	V	Bcer	FALSE	NA	NA	NA
Bc_ctg3_51	B1	3.671705351	Bcer	V	Bcer	FALSE	NA	NA	NA
Bc_ctg3_52	B1	33.24381872	Bcer	K	Bcer	FALSE	NA	NA	NA
Bc_ctg3_53	B1	28.28205473	Bcer	S	Bcer	FALSE	NA	NA	NA
Bc_ctg3_54	B1	0	Bcer	NA	Bcer	FALSE	NA	NA	NA
Bc_ctg3_55	B1	0.09923528	Bcer	NA	Bcer	FALSE	NA	NA	NA
Bc_ctg3_56	B1	11.01511605	Bcer	NA	Bcer	FALSE	NA	NA	NA
Bc_ctg3_57	B1	1.290058637	Bcer	S	Bcer	FALSE	NA	NA	NA
Bc_ctg3_58	B1	2.083940875	Bcer	NA	Bcer	FALSE	NA	NA	NA
Bc_ctg3_59	B1	0.19847056	Bcer	NA	Bcer	FALSE	NA	NA	NA
Bc_ctg3_6	B1	1.290058637	Bcer	NA	Bcer	FALSE	NA	NA	NA
Bc_ctg3_60	B1	118.6853946	Bcer	K	Bcer	FALSE	NA	NA	NA
Bc_ctg3_61	B1	3.671705351	Bcer	S	Bcer	FALSE	NA	NA	NA
Bc_ctg3_62	B1	5.259469827	Bcer	NA	Bcer	FALSE	NA	NA	NA
Bc_ctg3_63	B1	1.091588077	Bcer	L	Bcer	FALSE	NA	NA	NA
Bc_ctg3_64	B1	36.91552407	Bcer	K	Bcer	FALSE	NA	NA	NA
Bc_ctg3_65	B1	2.679352554	Bcer	E	Bcer	FALSE	NA	NA	NA
Bc_ctg3_66	B1	1.686999756	Bcer	I	Bcer	FALSE	NA	NA	NA
Bc_ctg3_67	B1	0.297705839	Bcer	S	Bcer	FALSE	NA	NA	NA
Bc_ctg3_68	B1	1.389293917	Bcer	H	Bcer	FALSE	NA	NA	NA
Bc_ctg3_69	B1	0.793882238	Bcer	H	Bcer	FALSE	NA	NA	NA
Bc_ctg3_7	B1	18.25929148	Bcer	QT	Bcer	TRUE	Bc_Ga0417192_03_rn01	Other	NA
Bc_ctg3_70	B1	0	Bcer	L	Bcer	FALSE	NA	NA	NA
Bc_ctg3_71	B1	113.2274542	Bcer	I	Bcer	FALSE	NA	NA	NA
Bc_ctg3_72	B1	1.290058637	Bcer	L	Bcer	FALSE	NA	NA	NA
Bc_ctg3_73	B1	40.48799414	Bcer	K	Bcer	FALSE	NA	NA	NA
Bc_ctg3_74	B1	113.8228659	Bcer	S	Bcer	FALSE	NA	NA	NA
Bc_ctg3_75	B1	69.46469583	Bcer	K	Bcer	FALSE	NA	NA	NA
Bc_ctg3_76	B1	0	Bcer	L	Bcer	FALSE	NA	NA	NA
Bc_ctg3_77	B1	0	Bcer	L	Bcer	FALSE	NA	NA	NA
Bc_ctg3_78	B1	26.19811386	Bcer	D	Bcer	FALSE	NA	NA	NA
Bc_ctg3_79	B1	5.457940387	Bcer	M	Bcer	FALSE	NA	NA	NA
Bc_ctg3_8	B1	5.557175667	Bcer	EGP	Bcer	FALSE	Bc_Ga0417192_03_rn01	Transport	SMCOG1106
Bc_ctg3_80	B1	31.8545248	Bcer	I	Bcer	FALSE	NA	NA	NA
Bc_ctg3_81	B1	75.41881262	Bcer	M	Bcer	FALSE	NA	NA	NA
Bc_ctg3_82	B1	0.396941119	Bcer	NA	Bcer	FALSE	NA	NA	NA
Bc_ctg3_83	B1	0.496176399	Bcer	K	Bcer	FALSE	NA	NA	NA
Bc_ctg3_84	B1	16.57229172	Bcer	O	Bcer	FALSE	NA	NA	NA
Bc_ctg3_85	B1	0.595411679	Bcer	O	Bcer	FALSE	NA	NA	NA
Bc_ctg3_86	B1	0.297705839	Bcer	NA	Bcer	FALSE	NA	NA	NA
Bc_ctg3_87	B1	1.488529196	Bcer	NA	Bcer	FALSE	NA	NA	NA
Bc_ctg3_88	B1	4.366352309	Bcer	P	Bcer	FALSE	NA	NA	NA
Bc_ctg3_89	B1	4.664058149	Bcer	NA	Bcer	FALSE	NA	NA	NA
Bc_ctg3_9	B1	5.954116786	Bcer	E	Bcer	FALSE	Bc_Ga0417192_03_rn01	Ancillary biosynthesis	SMCOG1244
Bc_ctg3_90	B1	0.09923528	Bcer	NA	Bcer	FALSE	NA	NA	NA
Bc_ctg3_91	B1	119.4792768	Bcer	C	Bcer	FALSE	NA	NA	NA
Bc_ctg3_92	B1	26.89276082	Bcer	NA	Bcer	FALSE	NA	NA	NA
Bc_ctg3_93	B1	6.549528464	Bcer	NA	Bcer	FALSE	NA	NA	NA
Bc_ctg3_94	B1	2.580117274	Bcer	S	Bcer	FALSE	NA	NA	NA
Bc_ctg3_95	B1	8.931175179	Bcer	NA	Bcer	FALSE	NA	NA	NA
Bc_ctg3_96	B1	22.72487907	Bcer	I	Bcer	FALSE	NA	NA	NA
Bc_ctg3_97	B1	37.90787687	Bcer	T	Bcer	FALSE	NA	NA	NA
Bc_ctg3_98	B1	8.633469339	Bcer	T	Bcer	FALSE	NA	NA	NA
Bc_ctg3_99	B1	12.30517469	Bcer	V	Bcer	FALSE	NA	NA	NA
Bc_ctg1_1	B2	26.34145961	Bcer	E	Bcer	FALSE	NA	NA	NA
Bc_ctg1_10	B2	50.46837172	Bcer	K	Bcer	FALSE	NA	NA	NA

Bc_ctg1_100	B2	7.342973253	Bcer	S	Bcer	FALSE	NA	NA	NA
Bc_ctg1_1002	B2	13.63695033	Bcer	S	Bcer	FALSE	Bc_Ga0417192_01_rn09	Other	NA
Bc_ctg1_1003	B2	14.21972598	Bcer	S	Bcer	FALSE	Bc_Ga0417192_01_rn09	Other	NA
Bc_ctg1_1004	B2	60.49211299	Bcer	NA	Bcer	FALSE	Bc_Ga0417192_01_rn09	Other	NA
Bc_ctg1_1005	B2	7.109862991	Bcer	S	Bcer	FALSE	Bc_Ga0417192_01_rn09	Other	NA
Bc_ctg1_1006	B2	26.92423526	Bcer	S	Bcer	FALSE	Bc_Ga0417192_01_rn09	Other	NA
Bc_ctg1_1007	B2	527.9947434	Bcer	I	Bcer	FALSE	Bc_Ga0417192_01_rn09	Ancillary biosynthesis	SMCOG1124
Bc_ctg1_1008	B2	81.23892631	Bcer	F	Bcer	FALSE	Bc_Ga0417192_01_rn09	Other	NA
Bc_ctg1_1009	B2	164.9255104	Bcer	S	Bcer	FALSE	Bc_Ga0417192_01_rn09	Other	NA
Bc_ctg1_101	B2	8.15885917	Bcer	S	Bcer	FALSE	NA	NA	NA
Bc_ctg1_1010	B2	0	Bcer	NA	Bcer	FALSE	NA	NA	NA
Bc_ctg1_1011	B2	9.557520742	Bcer	NA	Bcer	FALSE	NA	NA	NA
Bc_ctg1_1012	B2	10.48996179	Bcer	NA	Bcer	FALSE	NA	NA	NA
Bc_ctg1_1013	B2	1.16555131	Bcer	K	Bcer	FALSE	NA	NA	NA
Bc_ctg1_1014	B2	4.895315502	Bcer	IQ	Bcer	TRUE	NA	NA	NA
Bc_ctg1_1017	B2	0	Bcer	S	Bcer	FALSE	NA	NA	NA
Bc_ctg1_1018	B2	12.35484389	Bcer	E	Bcer	FALSE	NA	NA	NA
Bc_ctg1_1019	B2	16.90049399	Bcer	F	Bcer	FALSE	NA	NA	NA
Bc_ctg1_102	B2	0	Bcer	L	Bcer	FALSE	NA	NA	NA
Bc_ctg1_1020	B2	11.42240284	Bcer	S	Bcer	FALSE	NA	NA	NA
Bc_ctg1_1021	B2	48.37037936	Bcer	E	Bcer	FALSE	NA	NA	NA
Bc_ctg1_1022	B2	18.88193122	Bcer	G	Bcer	FALSE	NA	NA	NA
Bc_ctg1_1023	B2	6.643642467	Bcer	E	Bcer	FALSE	NA	NA	NA
Bc_ctg1_1024	B2	15.38527729	Bcer	E	Bcer	FALSE	NA	NA	NA
Bc_ctg1_1025	B2	12.70450928	Bcer	S	Bcer	FALSE	NA	NA	NA
Bc_ctg1_1026	B2	1.16555131	Bcer	NA	Bcer	FALSE	NA	NA	NA
Bc_ctg1_1027	B2	11.18929258	Bcer	S	Bcer	FALSE	NA	NA	NA
Bc_ctg1_1028	B2	96.74075873	Bcer	I	Bcer	FALSE	NA	NA	NA
Bc_ctg1_1029	B2	98.48908569	Bcer	V	Bcer	FALSE	NA	NA	NA
Bc_ctg1_103	B2	0	Bcer	L	Bcer	FALSE	NA	NA	NA
Bc_ctg1_1030	B2	1.981437227	Bcer	T	Bcer	FALSE	NA	NA	NA
Bc_ctg1_1031	B2	40.32807533	Bcer	C	Bcer	FALSE	NA	NA	NA
Bc_ctg1_1032	B2	37.64730731	Bcer	NA	Bcer	FALSE	NA	NA	NA
Bc_ctg1_1033	B2	7.809193777	Bcer	S	Bcer	FALSE	NA	NA	NA
Bc_ctg1_1034	B2	2643.237261	Bcer	G	Bcer	FALSE	NA	NA	NA
Bc_ctg1_1035	B2	372.5101987	Bcer	G	Bcer	FALSE	NA	NA	NA
Bc_ctg1_1036	B2	1351.922964	Bcer	G	Bcer	FALSE	NA	NA	NA
Bc_ctg1_1037	B2	88.11567903	Bcer	K	Bcer	FALSE	NA	NA	NA
Bc_ctg1_1038	B2	194.7636239	Bcer	S	Bcer	FALSE	NA	NA	NA
Bc_ctg1_1039	B2	1016.943518	Bcer	K	Bcer	FALSE	NA	NA	NA
Bc_ctg1_104	B2	2.214547489	Bcer	S	Bcer	FALSE	NA	NA	NA
Bc_ctg1_1040	B2	1171.029401	Bcer	G	Bcer	FALSE	NA	NA	NA
Bc_ctg1_1041	B2	135.0873968	Bcer	G	Bcer	FALSE	NA	NA	NA
Bc_ctg1_1042	B2	3.263543668	Bcer	S	Bcer	FALSE	NA	NA	NA
Bc_ctg1_1043	B2	10.37340666	Bcer	D	Bcer	FALSE	NA	NA	NA
Bc_ctg1_1044	B2	90.09711626	Bcer	D	Bcer	FALSE	NA	NA	NA
Bc_ctg1_1046	B2	19.34815175	Bcer	S	Bcer	FALSE	NA	NA	NA
Bc_ctg1_1047	B2	172.618149	Bcer	K	Bcer	FALSE	NA	NA	NA
Bc_ctg1_1048	B2	1147.60182	Bcer	O	Bcer	FALSE	NA	NA	NA
Bc_ctg1_1049	B2	254.3232958	Bcer	S	Bcer	FALSE	NA	NA	NA
Bc_ctg1_105	B2	1.748326965	Bcer	NA	Bcer	FALSE	NA	NA	NA
Bc_ctg1_1050	B2	17.01704913	Bcer	S	Bcer	FALSE	NA	NA	NA
Bc_ctg1_1051	B2	4.312539847	Bcer	EG	Bcer	FALSE	NA	NA	NA
Bc_ctg1_1052	B2	0.116555131	Bcer	S	Bcer	FALSE	NA	NA	NA
Bc_ctg1_1053	B2	0.116555131	Bcer	S	Bcer	FALSE	NA	NA	NA
Bc_ctg1_1054	B2	0.116555131	Bcer	IQ	Bcer	TRUE	NA	NA	NA
Bc_ctg1_1055	B2	0	Bcer	S	Bcer	FALSE	NA	NA	NA
Bc_ctg1_1057	B2	0	Bcer	S	Bcer	FALSE	NA	NA	NA
Bc_ctg1_1059	B2	1.631771834	Bcer	S	Bcer	FALSE	NA	NA	NA
Bc_ctg1_106	B2	0.349665393	Bcer	K	Bcer	FALSE	NA	NA	NA
Bc_ctg1_1060	B2	44.05783952	Bcer	K	Bcer	FALSE	NA	NA	NA
Bc_ctg1_1061	B2	23.19447107	Bcer	F	Bcer	FALSE	NA	NA	NA

Bc_ctg1_1062	B2	19.23159661	Bcer	T	Bcer	FALSE	NA	NA	NA
Bc_ctg1_1063	B2	0.233110262	Bcer	M	Bcer	FALSE	NA	NA	NA
Bc_ctg1_1064	B2	49.6524858	Bcer	K	Bcer	FALSE	NA	NA	NA
Bc_ctg1_1065	B2	119.8186747	Bcer	V	Bcer	FALSE	NA	NA	NA
Bc_ctg1_1066	B2	165.8579514	Bcer	S	Bcer	FALSE	NA	NA	NA
Bc_ctg1_1067	B2	2.33110262	Bcer	D	Bcer	FALSE	NA	NA	NA
Bc_ctg1_1068	B2	136.8357238	Bcer	K	Bcer	FALSE	NA	NA	NA
Bc_ctg1_1069	B2	0	Bcer	S	Bcer	FALSE	NA	NA	NA
Bc_ctg1_107	B2	7.459528384	Bcer	GIM	Bcer	FALSE	NA	NA	NA
Bc_ctg1_1070	B2	132.8728493	Bcer	S	Bcer	FALSE	NA	NA	NA
Bc_ctg1_1071	B2	92.77788427	Bcer	P	Bcer	FALSE	NA	NA	NA
Bc_ctg1_1072	B2	2602.79263	Bcer	F	Bcer	FALSE	NA	NA	NA
Bc_ctg1_1073	B2	2080.625643	Bcer	F	Bcer	FALSE	NA	NA	NA
Bc_ctg1_1074	B2	2539.036974	Bcer	G	Bcer	FALSE	NA	NA	NA
Bc_ctg1_1075	B2	85.08524563	Bcer	S	Bcer	FALSE	NA	NA	NA
Bc_ctg1_1076	B2	61.19144377	Bcer	L	Bcer	FALSE	NA	NA	NA
Bc_ctg1_1077	B2	0.233110262	Bcer	S	Bcer	FALSE	NA	NA	NA
Bc_ctg1_1078	B2	549.2077773	Bcer	P	Bcer	FALSE	NA	NA	NA
Bc_ctg1_1079	B2	13.28728493	Bcer	S	Bcer	FALSE	NA	NA	NA
Bc_ctg1_108	B2	37.53075218	Bcer	K	Bcer	FALSE	NA	NA	NA
Bc_ctg1_1080	B2	222.7368553	Bcer	J	Bcer	FALSE	NA	NA	NA
Bc_ctg1_1081	B2	70.28274399	Bcer	L	Bcer	FALSE	NA	NA	NA
Bc_ctg1_1083	B2	96.2745382	Bcer	C	Bcer	FALSE	NA	NA	NA
Bc_ctg1_1084	B2	19.46470688	Bcer	S	Bcer	FALSE	NA	NA	NA
Bc_ctg1_1085	B2	6.99330786	Bcer	K	Bcer	FALSE	NA	NA	NA
Bc_ctg1_1086	B2	0	Bcer	S	Bcer	FALSE	NA	NA	NA
Bc_ctg1_1087	B2	160.4964154	Bcer	F	Bcer	FALSE	NA	NA	NA
Bc_ctg1_1088	B2	1.631771834	Bcer	S	Bcer	FALSE	NA	NA	NA
Bc_ctg1_1089	B2	16.08460808	Bcer	I	Bcer	FALSE	NA	NA	NA
Bc_ctg1_109	B2	3.613209061	Bcer	IQ	Bcer	TRUE	NA	NA	NA
Bc_ctg1_1090	B2	49.06971015	Bcer	S	Bcer	FALSE	NA	NA	NA
Bc_ctg1_1091	B2	31.70299563	Bcer	K	Bcer	FALSE	NA	NA	NA
Bc_ctg1_1092	B2	6.410532205	Bcer	K	Bcer	FALSE	NA	NA	NA
Bc_ctg1_1093	B2	149.8898985	Bcer	S	Bcer	FALSE	NA	NA	NA
Bc_ctg1_1094	B2	12.58795415	Bcer	S	Bcer	FALSE	NA	NA	NA
Bc_ctg1_1095	B2	33.80098799	Bcer	S	Bcer	FALSE	NA	NA	NA
Bc_ctg1_1096	B2	210.9647871	Bcer	H	Bcer	FALSE	NA	NA	NA
Bc_ctg1_1097	B2	146.2766894	Bcer	H	Bcer	FALSE	NA	NA	NA
Bc_ctg1_1098	B2	400.2503198	Bcer	H	Bcer	FALSE	NA	NA	NA
Bc_ctg1_1099	B2	165.9745065	Bcer	H	Bcer	FALSE	NA	NA	NA
Bc_ctg1_11	B2	0.466220524	Bcer	S	Bcer	FALSE	NA	NA	NA
Bc_ctg1_110	B2	0.815885917	Bcer	K	Bcer	FALSE	NA	NA	NA
Bc_ctg1_1100	B2	0	Bcer	NA	Bcer	FALSE	NA	NA	NA
Bc_ctg1_1101	B2	2.447657751	Bcer	H	Bcer	FALSE	NA	NA	NA
Bc_ctg1_1102	B2	1.631771834	Bcer	H	Bcer	FALSE	NA	NA	NA
Bc_ctg1_1103	B2	0.932441048	Bcer	S	Bcer	FALSE	NA	NA	NA
Bc_ctg1_1104	B2	1.282106441	Bcer	H	Bcer	FALSE	NA	NA	NA
Bc_ctg1_1105	B2	2.097992358	Bcer	H	Bcer	FALSE	NA	NA	NA
Bc_ctg1_1106	B2	2.447657751	Bcer	H	Bcer	FALSE	NA	NA	NA
Bc_ctg1_1107	B2	0	Bcer	NA	Bcer	FALSE	NA	NA	NA
Bc_ctg1_1108	B2	17.36671452	Bcer	C	Bcer	FALSE	NA	NA	NA
Bc_ctg1_1109	B2	234.042703	Bcer	F	Bcer	FALSE	NA	NA	NA
Bc_ctg1_111	B2	6.99330786	Bcer	S	Bcer	FALSE	NA	NA	NA
Bc_ctg1_1110	B2	18.4157107	Bcer	E	Bcer	FALSE	NA	NA	NA
Bc_ctg1_1111	B2	139.9827123	Bcer	K	Bcer	FALSE	NA	NA	NA
Bc_ctg1_1112	B2	6.760197598	Bcer	NA	Bcer	FALSE	NA	NA	NA
Bc_ctg1_1113	B2	35.54931495	Bcer	S	Bcer	FALSE	NA	NA	NA
Bc_ctg1_1114	B2	9.557520742	Bcer	E	Bcer	FALSE	NA	NA	NA
Bc_ctg1_1115	B2	5.594646288	Bcer	E	Bcer	FALSE	NA	NA	NA
Bc_ctg1_1116	B2	2.680768013	Bcer	E	Bcer	FALSE	NA	NA	NA
Bc_ctg1_1117	B2	3.49665393	Bcer	E	Bcer	FALSE	NA	NA	NA
Bc_ctg1_1118	B2	2.913878275	Bcer	E	Bcer	FALSE	NA	NA	NA

Bc_ctg1_1119	B2	0.932441048	Bcer	NA	Bcer	FALSE	NA	NA	NA
Bc_ctg1_112	B2	28.55600709	Bcer	L	Bcer	FALSE	Bc_Ga0417192_01_rn01	Other	NA
Bc_ctg1_1120	B2	120.4014503	Bcer	S	Bcer	FALSE	NA	NA	NA
Bc_ctg1_1121	B2	61.07488864	Bcer	S	Bcer	FALSE	NA	NA	NA
Bc_ctg1_1122	B2	3.263543668	Bcer	E	Bcer	FALSE	NA	NA	NA
Bc_ctg1_1123	B2	136.6026135	Bcer	E	Bcer	FALSE	NA	NA	NA
Bc_ctg1_1124	B2	68.65097216	Bcer	E	Bcer	FALSE	NA	NA	NA
Bc_ctg1_1125	B2	51.28425764	Bcer	S	Bcer	FALSE	NA	NA	NA
Bc_ctg1_1126	B2	101.2864088	Bcer	NA	Bcer	FALSE	NA	NA	NA
Bc_ctg1_1127	B2	2.097992358	Bcer	NA	Bcer	FALSE	NA	NA	NA
Bc_ctg1_1128	B2	35.89898035	Bcer	P	Bcer	FALSE	NA	NA	NA
Bc_ctg1_1129	B2	4.079429585	Bcer	S	Bcer	FALSE	NA	NA	NA
Bc_ctg1_113	B2	7.576083515	Bcer	E	Bcer	FALSE	Bc_Ga0417192_01_rn01	Other	NA
Bc_ctg1_1130	B2	19.58126201	Bcer	L	Bcer	FALSE	NA	NA	NA
Bc_ctg1_1132	B2	1941.109152	Bcer	E	Bcer	FALSE	NA	NA	NA
Bc_ctg1_1133	B2	96.04142794	Bcer	J	Bcer	FALSE	NA	NA	NA
Bc_ctg1_1134	B2	11.6555131	Bcer	NA	Bcer	FALSE	NA	NA	NA
Bc_ctg1_1135	B2	14.80250164	Bcer	S	Bcer	FALSE	NA	NA	NA
Bc_ctg1_1136	B2	32.86854694	Bcer	S	Bcer	FALSE	NA	NA	NA
Bc_ctg1_1137	B2	55.01402183	Bcer	E	Bcer	FALSE	NA	NA	NA
Bc_ctg1_1138	B2	36.481756	Bcer	E	Bcer	FALSE	NA	NA	NA
Bc_ctg1_1139	B2	26.22490447	Bcer	ET	Bcer	FALSE	NA	NA	NA
Bc_ctg1_114	B2	1.981437227	Bcer	NA	Bcer	FALSE	Bc_Ga0417192_01_rn01	Other	NA
Bc_ctg1_1140	B2	74.59528384	Bcer	S	Bcer	FALSE	NA	NA	NA
Bc_ctg1_1141	B2	24.24346725	Bcer	S	Bcer	FALSE	NA	NA	NA
Bc_ctg1_1142	B2	20.39714792	Bcer	F	Bcer	FALSE	NA	NA	NA
Bc_ctg1_1143	B2	0.815885917	Bcer	S	Bcer	FALSE	NA	NA	NA
Bc_ctg1_1144	B2	274.4873335	Bcer	D	Bcer	FALSE	NA	NA	NA
Bc_ctg1_1145	B2	1768.840668	Bcer	C	Bcer	FALSE	NA	NA	NA
Bc_ctg1_1146	B2	672.1734405	Bcer	C	Bcer	FALSE	NA	NA	NA
Bc_ctg1_1147	B2	795.4887691	Bcer	C	Bcer	FALSE	NA	NA	NA
Bc_ctg1_1148	B2	1053.541829	Bcer	C	Bcer	FALSE	NA	NA	NA
Bc_ctg1_1149	B2	702.4777745	Bcer	C	Bcer	FALSE	NA	NA	NA
Bc_ctg1_1150	B2	2260.703321	Bcer	E	Bcer	FALSE	NA	NA	NA
Bc_ctg1_1151	B2	1325.115284	Bcer	C	Bcer	FALSE	NA	NA	NA
Bc_ctg1_1152	B2	35.31620469	Bcer	KT	Bcer	FALSE	NA	NA	NA
Bc_ctg1_1153	B2	2.913878275	Bcer	S	Bcer	FALSE	NA	NA	NA
Bc_ctg1_1154	B2	42.30951255	Bcer	C	Bcer	FALSE	NA	NA	NA
Bc_ctg1_1155	B2	0.349665393	Bcer	K	Bcer	FALSE	NA	NA	NA
Bc_ctg1_1156	B2	3.146988537	Bcer	NA	Bcer	FALSE	NA	NA	NA
Bc_ctg1_1157	B2	490.1143258	Bcer	KT	Bcer	FALSE	NA	NA	NA
Bc_ctg1_1158	B2	11.30584771	Bcer	M	Bcer	FALSE	NA	NA	NA
Bc_ctg1_1159	B2	92.31166375	Bcer	L	Bcer	FALSE	NA	NA	NA
Bc_ctg1_116	B2	3.49665393	Bcer	S	Bcer	FALSE	Bc_Ga0417192_01_rn01	Other	NA
Bc_ctg1_1160	B2	238.1221326	Bcer	K	Bcer	FALSE	NA	NA	NA
Bc_ctg1_1161	B2	113.7578079	Bcer	J	Bcer	FALSE	NA	NA	NA
Bc_ctg1_1162	B2	457.1292238	Bcer	H	Bcer	FALSE	NA	NA	NA
Bc_ctg1_1163	B2	244.64922	Bcer	H	Bcer	FALSE	NA	NA	NA
Bc_ctg1_1164	B2	45.45650109	Bcer	L	Bcer	FALSE	NA	NA	NA
Bc_ctg1_1165	B2	452.2339083	Bcer	L	Bcer	FALSE	NA	NA	NA
Bc_ctg1_1166	B2	280.5482003	Bcer	F	Bcer	FALSE	NA	NA	NA
Bc_ctg1_1167	B2	32.98510207	Bcer	K	Bcer	FALSE	NA	NA	NA
Bc_ctg1_1168	B2	870.6668285	Bcer	S	Bcer	FALSE	NA	NA	NA
Bc_ctg1_1169	B2	1934.115844	Bcer	I	Bcer	FALSE	NA	NA	NA
Bc_ctg1_117	B2	2.214547489	Bcer	NA	Bcer	FALSE	Bc_Ga0417192_01_rn01	Other	NA
Bc_ctg1_1170	B2	267.9602462	Bcer	I	Bcer	FALSE	NA	NA	NA
Bc_ctg1_1171	B2	0	Bcer	NA	Bcer	FALSE	NA	NA	NA
Bc_ctg1_1172	B2	0.582775655	Bcer	S	Bcer	FALSE	NA	NA	NA
Bc_ctg1_1173	B2	0.932441048	Bcer	S	Bcer	FALSE	NA	NA	NA
Bc_ctg1_1174	B2	1.748326965	Bcer	S	Bcer	FALSE	NA	NA	NA
Bc_ctg1_1175	B2	1.16555131	Bcer	S	Bcer	FALSE	NA	NA	NA
Bc_ctg1_1176	B2	0.116555131	Bcer	S	Bcer	FALSE	NA	NA	NA

Bc_ctg1_1177	B2	0	Bcer	S	Bcer	FALSE	NA	NA	NA
Bc_ctg1_1178	B2	0.582775655	Bcer	S	Bcer	FALSE	NA	NA	NA
Bc_ctg1_1179	B2	1.16555131	Bcer	S	Bcer	FALSE	NA	NA	NA
Bc_ctg1_118	B2	15.50183242	Bcer	EGP	Bcer	FALSE	Bc_Ga0417192_01_rn01	Transport	SMCOG1106
Bc_ctg1_1180	B2	0	Bcer	S	Bcer	FALSE	NA	NA	NA
Bc_ctg1_1181	B2	3.49665393	Bcer	J	Bcer	FALSE	NA	NA	NA
Bc_ctg1_1182	B2	1.282106441	Bcer	NA	Bcer	FALSE	NA	NA	NA
Bc_ctg1_1183	B2	864.4894066	Bcer	J	Bcer	FALSE	NA	NA	NA
Bc_ctg1_1184	B2	507.5975955	Bcer	E	Bcer	FALSE	NA	NA	NA
Bc_ctg1_1185	B2	31.46988537	Bcer	E	Bcer	FALSE	NA	NA	NA
Bc_ctg1_1186	B2	0.233110262	Bcer	S	Bcer	FALSE	NA	NA	NA
Bc_ctg1_1187	B2	87.41634825	Bcer	S	Bcer	FALSE	NA	NA	NA
Bc_ctg1_1188	B2	44.05783952	Bcer	NA	Bcer	FALSE	NA	NA	NA
Bc_ctg1_1189	B2	27.62356605	Bcer	S	Bcer	FALSE	NA	NA	NA
Bc_ctg1_119	B2	7.342973253	Bcer	K	Bcer	FALSE	Bc_Ga0417192_01_rn01	Other	NA
Bc_ctg1_1190	B2	28.43945196	Bcer	K	Bcer	FALSE	NA	NA	NA
Bc_ctg1_1191	B2	0	Bcer	L	Bcer	FALSE	NA	NA	NA
Bc_ctg1_1192	B2	4.66220524	Bcer	K	Bcer	FALSE	NA	NA	NA
Bc_ctg1_1193	B2	8.508524563	Bcer	S	Bcer	FALSE	NA	NA	NA
Bc_ctg1_1194	B2	23.19447107	Bcer	NU	Bcer	FALSE	NA	NA	NA
Bc_ctg1_1195	B2	55.94646288	Bcer	H	Bcer	FALSE	NA	NA	NA
Bc_ctg1_1196	B2	90.56333678	Bcer	P	Bcer	FALSE	NA	NA	NA
Bc_ctg1_1197	B2	0.932441048	Bcer	NA	Bcer	FALSE	NA	NA	NA
Bc_ctg1_1198	B2	30.18777893	Bcer	K	Bcer	FALSE	NA	NA	NA
Bc_ctg1_1199	B2	23.42758133	Bcer	K	Bcer	FALSE	NA	NA	NA
Bc_ctg1_120	B2	24.70968777	Bcer	K	Bcer	FALSE	Bc_Ga0417192_01_rn01	Other	NA
Bc_ctg1_1200	B2	2.447657751	Bcer	P	Bcer	FALSE	NA	NA	NA
Bc_ctg1_1201	B2	21.32958897	Bcer	P	Bcer	FALSE	NA	NA	NA
Bc_ctg1_1202	B2	5.711201419	Bcer	NA	Bcer	FALSE	NA	NA	NA
Bc_ctg1_1203	B2	0	Bcer	NA	Bcer	FALSE	NA	NA	NA
Bc_ctg1_1204	B2	1.631771834	Bcer	S	Bcer	FALSE	NA	NA	NA
Bc_ctg1_1205	B2	0.582775655	Bcer	S	Bcer	FALSE	NA	NA	NA
Bc_ctg1_1206	B2	2.214547489	Bcer	S	Bcer	FALSE	NA	NA	NA
Bc_ctg1_1207	B2	6.760197598	Bcer	S	Bcer	FALSE	NA	NA	NA
Bc_ctg1_1208	B2	4.778760371	Bcer	V	Bcer	FALSE	NA	NA	NA
Bc_ctg1_1209	B2	0	Bcer	K	Bcer	FALSE	NA	NA	NA
Bc_ctg1_121	B2	25.05935316	Bcer	C	Bcer	FALSE	Bc_Ga0417192_01_rn01	Ancillary biosynthesis	SMCOG1007
Bc_ctg1_1210	B2	2.447657751	Bcer	NA	Bcer	FALSE	NA	NA	NA
Bc_ctg1_1211	B2	0.815885917	Bcer	S	Bcer	FALSE	NA	NA	NA
Bc_ctg1_1212	B2	2971.92273	Bcer	E	Bcer	FALSE	NA	NA	NA
Bc_ctg1_1213	B2	2877.746184	Bcer	E	Bcer	FALSE	NA	NA	NA
Bc_ctg1_1214	B2	1231.404959	Bcer	E	Bcer	FALSE	NA	NA	NA
Bc_ctg1_1215	B2	2.447657751	Bcer	L	Bcer	FALSE	NA	NA	NA
Bc_ctg1_1216	B2	1.048996179	Bcer	S	Bcer	FALSE	NA	NA	NA
Bc_ctg1_1218	B2	0.699330786	Bcer	NA	Bcer	FALSE	NA	NA	NA
Bc_ctg1_1219	B2	18.99848635	Bcer	NA	Bcer	FALSE	NA	NA	NA
Bc_ctg1_122	B2	12.00517849	Bcer	S	Bcer	FALSE	Bc_Ga0417192_01_rn01	Core biosynthesis	None
Bc_ctg1_1220	B2	0.233110262	Bcer	S	Bcer	FALSE	NA	NA	NA
Bc_ctg1_1221	B2	7.925748908	Bcer	F	Bcer	FALSE	NA	NA	NA
Bc_ctg1_1222	B2	81.00581604	Bcer	O	Bcer	FALSE	NA	NA	NA
Bc_ctg1_1223	B2	0.116555131	Bcer	S	Bcer	FALSE	Bc_Ga0417192_01_rn10	Other	NA
Bc_ctg1_1224	B2	0	Bcer	U	Bcer	FALSE	Bc_Ga0417192_01_rn10	Other	NA
Bc_ctg1_1225	B2	0	Bcer	NA	Bcer	FALSE	Bc_Ga0417192_01_rn10	Other	NA
Bc_ctg1_1226	B2	1.048996179	Bcer	NU	Bcer	FALSE	Bc_Ga0417192_01_rn10	Other	NA
Bc_ctg1_1227	B2	0.116555131	Bcer	U	Bcer	FALSE	Bc_Ga0417192_01_rn10	Other	NA
Bc_ctg1_1228	B2	0.932441048	Bcer	NU	Bcer	FALSE	Bc_Ga0417192_01_rn10	Other	NA
Bc_ctg1_1229	B2	2.913878275	Bcer	NU	Bcer	FALSE	Bc_Ga0417192_01_rn10	Other	NA
Bc_ctg1_123	B2	18.53226583	Bcer	NT	Bcer	FALSE	Bc_Ga0417192_01_rn01	Other	SMCOG1061
Bc_ctg1_1230	B2	554.8024235	Bcer	K	Bcer	FALSE	Bc_Ga0417192_01_rn10	Other	NA
Bc_ctg1_1231	B2	5.361536026	Bcer	S	Bcer	FALSE	Bc_Ga0417192_01_rn10	Other	NA
Bc_ctg1_1232	B2	89.39778547	Bcer	U	Bcer	FALSE	Bc_Ga0417192_01_rn10	Transport	SMCOG1212
Bc_ctg1_1233	B2	0.116555131	Bcer	NA	Bcer	FALSE	Bc_Ga0417192_01_rn10	Other	NA

Bc_ctg1_1234	B2	1.048996179	Bcer	S	Bcer	FALSE	Bc_Ga0417192_01_rn10	Other	NA
Bc_ctg1_1235	B2	46.50549727	Bcer	NA	Bcer	FALSE	Bc_Ga0417192_01_rn10	Other	NA
Bc_ctg1_1236	B2	32.40232642	Bcer	NA	Bcer	FALSE	Bc_Ga0417192_01_rn10	Other	NA
Bc_ctg1_1237	B2	13.5203952	Bcer	NA	Bcer	FALSE	Bc_Ga0417192_01_rn10	Other	NA
Bc_ctg1_1238	B2	18.29915557	Bcer	S	Bcer	FALSE	Bc_Ga0417192_01_rn10	Other	NA
Bc_ctg1_1239	B2	86.13424181	Bcer	M	Bcer	FALSE	Bc_Ga0417192_01_rn10	Core biosynthesis	None
Bc_ctg1_124	B2	0	Bcer	NA	Bcer	FALSE	Bc_Ga0417192_01_rn01	Other	NA
Bc_ctg1_1240	B2	50.23526146	Bcer	E	Bcer	FALSE	Bc_Ga0417192_01_rn10	Other	NA
Bc_ctg1_1241	B2	17.71637991	Bcer	E	Bcer	FALSE	Bc_Ga0417192_01_rn10	Other	NA
Bc_ctg1_1242	B2	6.760197598	Bcer	E	Bcer	FALSE	Bc_Ga0417192_01_rn10	Core biosynthesis	SMCOG1168
Bc_ctg1_1243	B2	6.99330786	Bcer	E	Bcer	FALSE	Bc_Ga0417192_01_rn10	Ancillary biosynthesis	SMCOG1168
Bc_ctg1_1244	B2	10.83962718	Bcer	Q	Bcer	TRUE	Bc_Ga0417192_01_rn10	Other	NA
Bc_ctg1_1245	B2	105.3658384	Bcer	K	Bcer	FALSE	Bc_Ga0417192_01_rn10	Regulation	SMCOG1058
Bc_ctg1_1246	B2	91.14611244	Bcer	S	Bcer	FALSE	Bc_Ga0417192_01_rn10	Other	NA
Bc_ctg1_1247	B2	43.82472925	Bcer	S	Bcer	FALSE	Bc_Ga0417192_01_rn10	Ancillary biosynthesis	SMCOG1170
Bc_ctg1_1248	B2	22.14547489	Bcer	S	Bcer	FALSE	Bc_Ga0417192_01_rn10	Other	NA
Bc_ctg1_1249	B2	190.8007494	Bcer	G	Bcer	FALSE	Bc_Ga0417192_01_rn10	Other	SMCOG1108
Bc_ctg1_125	B2	0.466220524	Bcer	L	Bcer	FALSE	Bc_Ga0417192_01_rn01	Other	NA
Bc_ctg1_1250	B2	15.38527729	Bcer	S	Bcer	FALSE	Bc_Ga0417192_01_rn10	Other	NA
Bc_ctg1_1251	B2	58.97689628	Bcer	H	Bcer	FALSE	Bc_Ga0417192_01_rn10	Other	NA
Bc_ctg1_1252	B2	44.52406004	Bcer	J	Bcer	FALSE	Bc_Ga0417192_01_rn10	Other	NA
Bc_ctg1_1253	B2	14.80250164	Bcer	GJM	Bcer	FALSE	Bc_Ga0417192_01_rn10	Ancillary biosynthesis	SMCOG1064
Bc_ctg1_1254	B2	2.214547489	Bcer	P	Bcer	FALSE	NA	NA	NA
Bc_ctg1_1255	B2	2.797323144	Bcer	P	Bcer	FALSE	NA	NA	NA
Bc_ctg1_1256	B2	1.748326965	Bcer	P	Bcer	FALSE	NA	NA	NA
Bc_ctg1_1257	B2	1.048996179	Bcer	P	Bcer	FALSE	NA	NA	NA
Bc_ctg1_1258	B2	2.214547489	Bcer	P	Bcer	FALSE	NA	NA	NA
Bc_ctg1_1259	B2	678.1177521	Bcer	M	Bcer	FALSE	NA	NA	NA
Bc_ctg1_126	B2	13.98661572	Bcer	V	Bcer	FALSE	Bc_Ga0417192_01_rn01	Ancillary biosynthesis	SMCOG1053
Bc_ctg1_1260	B2	5.244980895	Bcer	EGP	Bcer	FALSE	NA	NA	NA
Bc_ctg1_1261	B2	320.5266102	Bcer	P	Bcer	FALSE	NA	NA	NA
Bc_ctg1_1262	B2	155.2514345	Bcer	S	Bcer	FALSE	NA	NA	NA
Bc_ctg1_1263	B2	1.16555131	Bcer	M	Bcer	FALSE	NA	NA	NA
Bc_ctg1_1264	B2	409.8078406	Bcer	I	Bcer	FALSE	NA	NA	NA
Bc_ctg1_1265	B2	17.94949017	Bcer	P	Bcer	FALSE	NA	NA	NA
Bc_ctg1_1266	B2	55.82990775	Bcer	P	Bcer	FALSE	NA	NA	NA
Bc_ctg1_1267	B2	50.35181659	Bcer	P	Bcer	FALSE	NA	NA	NA
Bc_ctg1_1268	B2	167.0235027	Bcer	S	Bcer	FALSE	NA	NA	NA
Bc_ctg1_127	B2	11.42240284	Bcer	V	Bcer	FALSE	Bc_Ga0417192_01_rn01	Ancillary biosynthesis	SMCOG1053
Bc_ctg1_1270	B2	72.963512	Bcer	L	Bcer	FALSE	NA	NA	NA
Bc_ctg1_1271	B2	376.5896283	Bcer	JKL	Bcer	FALSE	NA	NA	NA
Bc_ctg1_1272	B2	0	Bcer	NA	Bcer	FALSE	NA	NA	NA
Bc_ctg1_1273	B2	228.0983914	Bcer	IM	Bcer	FALSE	NA	NA	NA
Bc_ctg1_1274	B2	69.23374781	Bcer	S	Bcer	FALSE	NA	NA	NA
Bc_ctg1_1275	B2	14.56939137	Bcer	S	Bcer	FALSE	NA	NA	NA
Bc_ctg1_1276	B2	15.0356119	Bcer	C	Bcer	FALSE	NA	NA	NA
Bc_ctg1_1277	B2	691.4050371	Bcer	K	Bcer	FALSE	NA	NA	NA
Bc_ctg1_1278	B2	1206.695271	Bcer	L	Bcer	FALSE	NA	NA	NA
Bc_ctg1_1279	B2	560.3970698	Bcer	S	Bcer	FALSE	NA	NA	NA
Bc_ctg1_128	B2	0.699330786	Bcer	S	Bcer	FALSE	Bc_Ga0417192_01_rn01	Other	NA
Bc_ctg1_1280	B2	425.7758935	Bcer	K	Bcer	FALSE	NA	NA	NA
Bc_ctg1_1281	B2	80.30648526	Bcer	L	Bcer	FALSE	NA	NA	NA
Bc_ctg1_1282	B2	0.233110262	Bcer	S	Bcer	FALSE	NA	NA	NA
Bc_ctg1_1283	B2	612.8468788	Bcer	S	Bcer	FALSE	NA	NA	NA
Bc_ctg1_1284	B2	114.3405835	Bcer	F	Bcer	FALSE	NA	NA	NA
Bc_ctg1_1285	B2	6.876752729	Bcer	M	Bcer	FALSE	NA	NA	NA
Bc_ctg1_1286	B2	24.24346725	Bcer	S	Bcer	FALSE	NA	NA	NA
Bc_ctg1_1287	B2	237.539357	Bcer	S	Bcer	FALSE	NA	NA	NA
Bc_ctg1_1288	B2	273.7880027	Bcer	T	Bcer	FALSE	NA	NA	NA
Bc_ctg1_1289	B2	0.349665393	Bcer	S	Bcer	FALSE	NA	NA	NA
Bc_ctg1_129	B2	0	Bcer	S	Bcer	FALSE	Bc_Ga0417192_01_rn01	Other	NA
Bc_ctg1_1290	B2	0	Bcer	S	Bcer	FALSE	NA	NA	NA

Bc_ctg1_1291	B2	1.631771834	Bcer	S	Bcer	FALSE	NA	NA	NA
Bc_ctg1_1292	B2	1090.14014	Bcer	S	Bcer	FALSE	NA	NA	NA
Bc_ctg1_1293	B2	420.7640229	Bcer	J	Bcer	FALSE	NA	NA	NA
Bc_ctg1_1294	B2	352.2296059	Bcer	J	Bcer	FALSE	NA	NA	NA
Bc_ctg1_1295	B2	131.2410775	Bcer	J	Bcer	FALSE	NA	NA	NA
Bc_ctg1_1296	B2	318.7782833	Bcer	J	Bcer	FALSE	NA	NA	NA
Bc_ctg1_1297	B2	197.560947	Bcer	O	Bcer	FALSE	NA	NA	NA
Bc_ctg1_1298	B2	2387.165638	Bcer	O	Bcer	FALSE	NA	NA	NA
Bc_ctg1_1299	B2	452.3504634	Bcer	O	Bcer	FALSE	NA	NA	NA
Bc_ctg1_13	B2	28.08978657	Bcer	Q	Bcer	TRUE	NA	NA	NA
Bc_ctg1_130	B2	2.797323144	Bcer	K	Bcer	FALSE	Bc_Ga0417192_01_rn01	Regulation	SMCOG1284
Bc_ctg1_1300	B2	254.5564061	Bcer	K	Bcer	FALSE	NA	NA	NA
Bc_ctg1_1301	B2	41.49362663	Bcer	H	Bcer	FALSE	NA	NA	NA
Bc_ctg1_1302	B2	7.925748908	Bcer	K	Bcer	FALSE	NA	NA	NA
Bc_ctg1_1303	B2	477.1767063	Bcer	M	Bcer	FALSE	NA	NA	NA
Bc_ctg1_1304	B2	0.233110262	Bcer	NA	Bcer	FALSE	NA	NA	NA
Bc_ctg1_1305	B2	3.613209061	Bcer	C	Bcer	FALSE	NA	NA	NA
Bc_ctg1_1306	B2	277.9839874	Bcer	J	Bcer	FALSE	NA	NA	NA
Bc_ctg1_1307	B2	48.83659989	Bcer	L	Bcer	FALSE	NA	NA	NA
Bc_ctg1_1308	B2	0.116555131	Bcer	S	Bcer	FALSE	NA	NA	NA
Bc_ctg1_1309	B2	8.625079694	Bcer	S	Bcer	FALSE	NA	NA	NA
Bc_ctg1_1310	B2	36.481756	Bcer	F	Bcer	FALSE	NA	NA	NA
Bc_ctg1_1311	B2	3.49665393	Bcer	L	Bcer	FALSE	NA	NA	NA
Bc_ctg1_1312	B2	0	Bcer	E	Bcer	FALSE	NA	NA	NA
Bc_ctg1_1313	B2	237.3062467	Bcer	Q	Bcer	TRUE	NA	NA	NA
Bc_ctg1_1314	B2	179.3783466	Bcer	J	Bcer	FALSE	NA	NA	NA
Bc_ctg1_1315	B2	121.2173362	Bcer	H	Bcer	FALSE	NA	NA	NA
Bc_ctg1_1316	B2	134.6211763	Bcer	H	Bcer	FALSE	NA	NA	NA
Bc_ctg1_1317	B2	43.2419536	Bcer	J	Bcer	FALSE	NA	NA	NA
Bc_ctg1_1318	B2	213.0627795	Bcer	E	Bcer	FALSE	NA	NA	NA
Bc_ctg1_1319	B2	248.0293188	Bcer	S	Bcer	FALSE	NA	NA	NA
Bc_ctg1_1320	B2	90.67989192	Bcer	S	Bcer	FALSE	NA	NA	NA
Bc_ctg1_1321	B2	0.116555131	Bcer	NA	Bcer	FALSE	NA	NA	NA
Bc_ctg1_1322	B2	61.89077456	Bcer	I	Bcer	FALSE	NA	NA	NA
Bc_ctg1_1323	B2	2.33110262	Bcer	K	Bcer	FALSE	NA	NA	NA
Bc_ctg1_1324	B2	3.49665393	Bcer	NA	Bcer	FALSE	NA	NA	NA
Bc_ctg1_1325	B2	11.30584771	Bcer	K	Bcer	FALSE	NA	NA	NA
Bc_ctg1_1327	B2	7.576083515	Bcer	C	Bcer	FALSE	NA	NA	NA
Bc_ctg1_1328	B2	461.4417636	Bcer	S	Bcer	FALSE	NA	NA	NA
Bc_ctg1_1329	B2	0	Bcer	NA	Bcer	FALSE	NA	NA	NA
Bc_ctg1_133	B2	0.349665393	Bcer	NA	Bcer	FALSE	NA	NA	NA
Bc_ctg1_1330	B2	0.699330786	Bcer	P	Bcer	FALSE	NA	NA	NA
Bc_ctg1_1331	B2	7.459528384	Bcer	K	Bcer	FALSE	NA	NA	NA
Bc_ctg1_1332	B2	10.02374127	Bcer	I	Bcer	FALSE	NA	NA	NA
Bc_ctg1_1333	B2	18.53226583	Bcer	S	Bcer	FALSE	NA	NA	NA
Bc_ctg1_1334	B2	0.116555131	Bcer	NA	Bcer	FALSE	NA	NA	NA
Bc_ctg1_1335	B2	3.846319323	Bcer	G	Bcer	FALSE	NA	NA	NA
Bc_ctg1_1336	B2	8.974745087	Bcer	NA	Bcer	FALSE	NA	NA	NA
Bc_ctg1_1338	B2	3.729764192	Bcer	U	Bcer	FALSE	NA	NA	NA
Bc_ctg1_1339	B2	13.98661572	Bcer	O	Bcer	FALSE	NA	NA	NA
Bc_ctg1_134	B2	0.116555131	Bcer	NA	Bcer	FALSE	NA	NA	NA
Bc_ctg1_1340	B2	2.680768013	Bcer	E	Bcer	FALSE	NA	NA	NA
Bc_ctg1_1341	B2	0.699330786	Bcer	H	Bcer	FALSE	NA	NA	NA
Bc_ctg1_1342	B2	16.90049399	Bcer	S	Bcer	FALSE	NA	NA	NA
Bc_ctg1_1343	B2	0.582775655	Bcer	S	Bcer	FALSE	NA	NA	NA
Bc_ctg1_1344	B2	7.342973253	Bcer	K	Bcer	FALSE	NA	NA	NA
Bc_ctg1_1345	B2	1.16555131	Bcer	I	Bcer	FALSE	NA	NA	NA
Bc_ctg1_1346	B2	6.410532205	Bcer	K	Bcer	FALSE	NA	NA	NA
Bc_ctg1_1347	B2	1.631771834	Bcer	J	Bcer	FALSE	NA	NA	NA
Bc_ctg1_1348	B2	2.913878275	Bcer	C	Bcer	FALSE	NA	NA	NA
Bc_ctg1_1349	B2	22.72825054	Bcer	HP	Bcer	FALSE	NA	NA	NA
Bc_ctg1_135	B2	2.447657751	Bcer	T	Bcer	FALSE	NA	NA	NA

Bc_ctg1_1350	B2	29.95466867	Bcer	P	Bcer	FALSE	NA	NA	NA
Bc_ctg1_1351	B2	34.26720851	Bcer	P	Bcer	FALSE	NA	NA	NA
Bc_ctg1_1352	B2	0.349665393	Bcer	S	Bcer	FALSE	NA	NA	NA
Bc_ctg1_1353	B2	7918.289379	Bcer	C	Bcer	FALSE	NA	NA	NA
Bc_ctg1_1354	B2	431.487095	Bcer	E	Bcer	FALSE	NA	NA	NA
Bc_ctg1_1355	B2	203.0390382	Bcer	E	Bcer	FALSE	NA	NA	NA
Bc_ctg1_1356	B2	331.4827926	Bcer	E	Bcer	FALSE	NA	NA	NA
Bc_ctg1_1357	B2	104.4333974	Bcer	Q	Bcer	TRUE	NA	NA	NA
Bc_ctg1_1358	B2	0.932441048	Bcer	S	Bcer	FALSE	NA	NA	NA
Bc_ctg1_1359	B2	323.5570436	Bcer	S	Bcer	FALSE	NA	NA	NA
Bc_ctg1_136	B2	13.98661572	Bcer	T	Bcer	FALSE	NA	NA	NA
Bc_ctg1_1360	B2	1.282106441	Bcer	M	Bcer	FALSE	NA	NA	NA
Bc_ctg1_1363	B2	215.277327	Bcer	K	Bcer	FALSE	NA	NA	NA
Bc_ctg1_1364	B2	115.3895797	Bcer	F	Bcer	FALSE	NA	NA	NA
Bc_ctg1_1365	B2	243.6002238	Bcer	O	Bcer	FALSE	NA	NA	NA
Bc_ctg1_1366	B2	36.94797653	Bcer	O	Bcer	FALSE	NA	NA	NA
Bc_ctg1_1367	B2	18.29915557	Bcer	S	Bcer	FALSE	NA	NA	NA
Bc_ctg1_1368	B2	170.1704913	Bcer	S	Bcer	FALSE	NA	NA	NA
Bc_ctg1_1369	B2	111.8929258	Bcer	S	Bcer	FALSE	NA	NA	NA
Bc_ctg1_137	B2	0.349665393	Bcer	NA	Bcer	FALSE	NA	NA	NA
Bc_ctg1_1370	B2	454.4484558	Bcer	L	Bcer	FALSE	NA	NA	NA
Bc_ctg1_1371	B2	416.9177036	Bcer	S	Bcer	FALSE	NA	NA	NA
Bc_ctg1_1372	B2	823.928221	Bcer	J	Bcer	FALSE	Bc_Ga0417192_01_rn11	Other	NA
Bc_ctg1_1373	B2	4.195984716	Bcer	S	Bcer	FALSE	Bc_Ga0417192_01_rn11	Other	NA
Bc_ctg1_1374	B2	0.932441048	Bcer	S	Bcer	FALSE	Bc_Ga0417192_01_rn11	Other	NA
Bc_ctg1_1375	B2	0	Bcer	NA	Bcer	FALSE	Bc_Ga0417192_01_rn11	Other	NA
Bc_ctg1_1377	B2	6.99330786	Bcer	NA	Bcer	FALSE	Bc_Ga0417192_01_rn11	Other	NA
Bc_ctg1_1378	B2	107.8134962	Bcer	L	Bcer	FALSE	Bc_Ga0417192_01_rn11	Other	NA
Bc_ctg1_1379	B2	58.04445524	Bcer	S	Bcer	FALSE	Bc_Ga0417192_01_rn11	Other	SMCOG1272
Bc_ctg1_138	B2	70.51585425	Bcer	S	Bcer	FALSE	NA	NA	NA
Bc_ctg1_1380	B2	439.4128439	Bcer	J	Bcer	FALSE	Bc_Ga0417192_01_rn11	Other	NA
Bc_ctg1_1381	B2	406.8939623	Bcer	E	Bcer	FALSE	Bc_Ga0417192_01_rn11	Core biosynthesis	SMCOG1139
Bc_ctg1_1382	B2	101.8691845	Bcer	K	Bcer	FALSE	Bc_Ga0417192_01_rn11	Other	NA
Bc_ctg1_1383	B2	32.98510207	Bcer	L	Bcer	FALSE	Bc_Ga0417192_01_rn11	Other	NA
Bc_ctg1_1384	B2	27.62356605	Bcer	S	Bcer	FALSE	Bc_Ga0417192_01_rn11	Other	NA
Bc_ctg1_1385	B2	203.0390382	Bcer	H	Bcer	FALSE	Bc_Ga0417192_01_rn11	Ancillary biosynthesis	None
Bc_ctg1_1386	B2	458.1782199	Bcer	J	Bcer	FALSE	Bc_Ga0417192_01_rn11	Other	NA
Bc_ctg1_1387	B2	245.6982161	Bcer	J	Bcer	FALSE	Bc_Ga0417192_01_rn11	Other	SMCOG1162
Bc_ctg1_1388	B2	0.116555131	Bcer	NA	Bcer	FALSE	Bc_Ga0417192_01_rn11	Other	NA
Bc_ctg1_1389	B2	68.18475163	Bcer	J	Bcer	FALSE	Bc_Ga0417192_01_rn11	Other	NA
Bc_ctg1_139	B2	1.282106441	Bcer	S	Bcer	FALSE	NA	NA	NA
Bc_ctg1_1390	B2	597.8112669	Bcer	KT	Bcer	FALSE	Bc_Ga0417192_01_rn11	Other	NA
Bc_ctg1_1391	B2	189.7517533	Bcer	F	Bcer	FALSE	Bc_Ga0417192_01_rn11	Other	NA
Bc_ctg1_1392	B2	342.3224197	Bcer	L	Bcer	FALSE	Bc_Ga0417192_01_rn11	Other	NA
Bc_ctg1_1393	B2	7.692638646	Bcer	P	Bcer	FALSE	Bc_Ga0417192_01_rn11	Other	NA
Bc_ctg1_1394	B2	1050.045175	Bcer	U	Bcer	FALSE	Bc_Ga0417192_01_rn11	Other	NA
Bc_ctg1_1395	B2	16.20116321	Bcer	G	Bcer	FALSE	Bc_Ga0417192_01_rn11	Other	NA
Bc_ctg1_1396	B2	0	Bcer	S	Bcer	FALSE	Bc_Ga0417192_01_rn11	Core biosynthesis	SMCOG1086
Bc_ctg1_1397	B2	3.030433406	Bcer	S	Bcer	FALSE	Bc_Ga0417192_01_rn11	Other	NA
Bc_ctg1_1398	B2	0.116555131	Bcer	S	Bcer	FALSE	Bc_Ga0417192_01_rn11	Other	NA
Bc_ctg1_1399	B2	98.95530622	Bcer	U	Bcer	FALSE	Bc_Ga0417192_01_rn11	Other	NA
Bc_ctg1_14	B2	0.815885917	Bcer	F	Bcer	FALSE	NA	NA	NA
Bc_ctg1_140	B2	2.447657751	Bcer	S	Bcer	FALSE	NA	NA	NA
Bc_ctg1_1400	B2	234.3923684	Bcer	F	Bcer	FALSE	Bc_Ga0417192_01_rn11	Other	NA
Bc_ctg1_1401	B2	167.9559438	Bcer	J	Bcer	FALSE	Bc_Ga0417192_01_rn11	Other	NA
Bc_ctg1_1402	B2	1.16555131	Bcer	S	Bcer	FALSE	Bc_Ga0417192_01_rn11	Other	NA
Bc_ctg1_1403	B2	80.77270578	Bcer	L	Bcer	FALSE	Bc_Ga0417192_01_rn11	Other	NA
Bc_ctg1_1404	B2	28.67256223	Bcer	L	Bcer	FALSE	Bc_Ga0417192_01_rn11	Other	NA
Bc_ctg1_1405	B2	13.05417467	Bcer	P	Bcer	FALSE	Bc_Ga0417192_01_rn11	Transport	SMCOG1033
Bc_ctg1_1406	B2	0.233110262	Bcer	S	Bcer	FALSE	Bc_Ga0417192_01_rn11	Other	NA
Bc_ctg1_1407	B2	17.71637991	Bcer	NA	Bcer	FALSE	Bc_Ga0417192_01_rn11	Other	NA
Bc_ctg1_1408	B2	0.582775655	Bcer	S	Bcer	FALSE	Bc_Ga0417192_01_rn11	Other	NA

Bc_ctg1_1409	B2	0	Bcer	M	Bcer	FALSE	NA	NA	NA
Bc_ctg1_141	B2	1.398661572	Bcer	S	Bcer	FALSE	NA	NA	NA
Bc_ctg1_1410	B2	4.429094978	Bcer	H	Bcer	FALSE	NA	NA	NA
Bc_ctg1_1411	B2	2.214547489	Bcer	H	Bcer	FALSE	NA	NA	NA
Bc_ctg1_1412	B2	3.146988537	Bcer	H	Bcer	FALSE	NA	NA	NA
Bc_ctg1_1413	B2	10.95618231	Bcer	E	Bcer	FALSE	NA	NA	NA
Bc_ctg1_1414	B2	34.9665393	Bcer	S	Bcer	FALSE	NA	NA	NA
Bc_ctg1_1415	B2	63.98876692	Bcer	S	Bcer	FALSE	NA	NA	NA
Bc_ctg1_1416	B2	93.12754967	Bcer	E	Bcer	FALSE	NA	NA	NA
Bc_ctg1_1417	B2	13.05417467	Bcer	V	Bcer	FALSE	NA	NA	NA
Bc_ctg1_1418	B2	49.88559607	Bcer	V	Bcer	FALSE	NA	NA	NA
Bc_ctg1_1419	B2	16.31771834	Bcer	V	Bcer	FALSE	NA	NA	NA
Bc_ctg1_142	B2	29.7215584	Bcer	T	Bcer	FALSE	NA	NA	NA
Bc_ctg1_1420	B2	26.92423526	Bcer	T	Bcer	FALSE	NA	NA	NA
Bc_ctg1_1421	B2	20.63025819	Bcer	T	Bcer	FALSE	NA	NA	NA
Bc_ctg1_1422	B2	412.838274	Bcer	S	Bcer	FALSE	NA	NA	NA
Bc_ctg1_1423	B2	177.2803542	Bcer	T	Bcer	FALSE	NA	NA	NA
Bc_ctg1_1424	B2	1123.591463	Bcer	J	Bcer	FALSE	NA	NA	NA
Bc_ctg1_1425	B2	2073.982001	Bcer	J	Bcer	FALSE	NA	NA	NA
Bc_ctg1_1426	B2	383.8160464	Bcer	J	Bcer	FALSE	NA	NA	NA
Bc_ctg1_1427	B2	23.19447107	Bcer	J	Bcer	FALSE	NA	NA	NA
Bc_ctg1_1428	B2	6.060866812	Bcer	S	Bcer	FALSE	NA	NA	NA
Bc_ctg1_1429	B2	2.913878275	Bcer	M	Bcer	FALSE	NA	NA	NA
Bc_ctg1_143	B2	1.398661572	Bcer	V	Bcer	FALSE	NA	NA	NA
Bc_ctg1_1430	B2	578.34656	Bcer	D	Bcer	FALSE	NA	NA	NA
Bc_ctg1_1431	B2	451.6511326	Bcer	D	Bcer	FALSE	NA	NA	NA
Bc_ctg1_1432	B2	221.1050835	Bcer	M	Bcer	FALSE	NA	NA	NA
Bc_ctg1_1433	B2	538.8343706	Bcer	M	Bcer	FALSE	NA	NA	NA
Bc_ctg1_1434	B2	279.2660939	Bcer	D	Bcer	FALSE	NA	NA	NA
Bc_ctg1_1435	B2	31.23677511	Bcer	L	Bcer	FALSE	NA	NA	NA
Bc_ctg1_1437	B2	1.748326965	Bcer	E	Bcer	FALSE	NA	NA	NA
Bc_ctg1_1438	B2	22.37858515	Bcer	D	Bcer	FALSE	NA	NA	NA
Bc_ctg1_1439	B2	1.048996179	Bcer	NA	Bcer	FALSE	NA	NA	NA
Bc_ctg1_144	B2	0.116555131	Bcer	V	Bcer	FALSE	NA	NA	NA
Bc_ctg1_1440	B2	65.38742849	Bcer	H	Bcer	FALSE	NA	NA	NA
Bc_ctg1_1441	B2	732.4324432	Bcer	J	Bcer	FALSE	NA	NA	NA
Bc_ctg1_1442	B2	5.128425764	Bcer	S	Bcer	FALSE	NA	NA	NA
Bc_ctg1_1443	B2	0.699330786	Bcer	M	Bcer	FALSE	NA	NA	NA
Bc_ctg1_1444	B2	356.4255906	Bcer	H	Bcer	FALSE	NA	NA	NA
Bc_ctg1_1445	B2	136.6026135	Bcer	H	Bcer	FALSE	NA	NA	NA
Bc_ctg1_1446	B2	49.30282041	Bcer	H	Bcer	FALSE	NA	NA	NA
Bc_ctg1_1447	B2	114.806804	Bcer	H	Bcer	FALSE	NA	NA	NA
Bc_ctg1_1448	B2	38.23008297	Bcer	O	Bcer	FALSE	NA	NA	NA
Bc_ctg1_1449	B2	177.1637991	Bcer	H	Bcer	FALSE	NA	NA	NA
Bc_ctg1_145	B2	0.116555131	Bcer	T	Bcer	FALSE	NA	NA	NA
Bc_ctg1_1450	B2	9.907186135	Bcer	K	Bcer	FALSE	NA	NA	NA
Bc_ctg1_1451	B2	50.58492685	Bcer	O	Bcer	FALSE	NA	NA	NA
Bc_ctg1_1452	B2	250.1273111	Bcer	D	Bcer	FALSE	NA	NA	NA
Bc_ctg1_1453	B2	640.8201102	Bcer	O	Bcer	FALSE	NA	NA	NA
Bc_ctg1_1454	B2	48.83659989	Bcer	LO	Bcer	FALSE	NA	NA	NA
Bc_ctg1_1455	B2	3080.901778	Bcer	O	Bcer	FALSE	NA	NA	NA
Bc_ctg1_1456	B2	2750.934202	Bcer	D	Bcer	FALSE	NA	NA	NA
Bc_ctg1_1457	B2	255.2557369	Bcer	O	Bcer	FALSE	NA	NA	NA
Bc_ctg1_1458	B2	10.60651692	Bcer	NA	Bcer	FALSE	NA	NA	NA
Bc_ctg1_146	B2	0.233110262	Bcer	T	Bcer	FALSE	NA	NA	NA
Bc_ctg1_1460	B2	98.72219595	Bcer	S	Bcer	FALSE	NA	NA	NA
Bc_ctg1_1461	B2	0.116555131	Bcer	NA	Bcer	FALSE	NA	NA	NA
Bc_ctg1_1462	B2	0.582775655	Bcer	NA	Bcer	FALSE	NA	NA	NA
Bc_ctg1_1463	B2	100.3539678	Bcer	S	Bcer	FALSE	NA	NA	NA
Bc_ctg1_1464	B2	89.864006	Bcer	F	Bcer	FALSE	NA	NA	NA
Bc_ctg1_1465	B2	43.47506386	Bcer	J	Bcer	FALSE	NA	NA	NA
Bc_ctg1_1466	B2	2.097992358	Bcer	S	Bcer	FALSE	NA	NA	NA

Bc_ctg1_1467	B2	69.58341321	Bcer	M	Bcer	FALSE	NA	NA	NA
Bc_ctg1_1468	B2	1.398661572	Bcer	EGP	Bcer	FALSE	NA	NA	NA
Bc_ctg1_1469	B2	5.128425764	Bcer	S	Bcer	FALSE	NA	NA	NA
Bc_ctg1_147	B2	1.631771834	Bcer	V	Bcer	FALSE	NA	NA	NA
Bc_ctg1_1470	B2	3.729764192	Bcer	K	Bcer	FALSE	NA	NA	NA
Bc_ctg1_1471	B2	0	Bcer	S	Bcer	FALSE	NA	NA	NA
Bc_ctg1_1472	B2	1.864882096	Bcer	K	Bcer	FALSE	NA	NA	NA
Bc_ctg1_1473	B2	6.293977074	Bcer	F	Bcer	FALSE	NA	NA	NA
Bc_ctg1_1474	B2	27.27390065	Bcer	F	Bcer	FALSE	NA	NA	NA
Bc_ctg1_1475	B2	0.466220524	Bcer	KT	Bcer	FALSE	NA	NA	NA
Bc_ctg1_1476	B2	0.932441048	Bcer	K	Bcer	FALSE	NA	NA	NA
Bc_ctg1_1477	B2	3.729764192	Bcer	EG	Bcer	FALSE	NA	NA	NA
Bc_ctg1_1478	B2	9.440965611	Bcer	G	Bcer	FALSE	NA	NA	NA
Bc_ctg1_1479	B2	96.15798307	Bcer	K	Bcer	FALSE	NA	NA	NA
Bc_ctg1_148	B2	3.030433406	Bcer	S	Bcer	FALSE	NA	NA	NA
Bc_ctg1_1480	B2	57.81134497	Bcer	CP	Bcer	FALSE	NA	NA	NA
Bc_ctg1_1481	B2	17.13360426	Bcer	S	Bcer	FALSE	NA	NA	NA
Bc_ctg1_1482	B2	75.87739028	Bcer	M	Bcer	FALSE	NA	NA	NA
Bc_ctg1_1483	B2	270.8741244	Bcer	N	Bcer	FALSE	NA	NA	NA
Bc_ctg1_1484	B2	232.8771517	Bcer	N	Bcer	FALSE	NA	NA	NA
Bc_ctg1_1485	B2	24.8262429	Bcer	M	Bcer	FALSE	NA	NA	NA
Bc_ctg1_1486	B2	31.81955076	Bcer	S	Bcer	FALSE	NA	NA	NA
Bc_ctg1_1487	B2	1369.406234	Bcer	C	Bcer	FALSE	NA	NA	NA
Bc_ctg1_1488	B2	2976.701491	Bcer	C	Bcer	FALSE	NA	NA	NA
Bc_ctg1_1489	B2	556.08453	Bcer	C	Bcer	FALSE	NA	NA	NA
Bc_ctg1_149	B2	39.51218941	Bcer	T	Bcer	FALSE	NA	NA	NA
Bc_ctg1_1490	B2	126.4623171	Bcer	S	Bcer	FALSE	NA	NA	NA
Bc_ctg1_1491	B2	68.88408242	Bcer	L	Bcer	FALSE	NA	NA	NA
Bc_ctg1_1492	B2	114.4571386	Bcer	O	Bcer	FALSE	NA	NA	NA
Bc_ctg1_1493	B2	479.0415884	Bcer	C	Bcer	FALSE	NA	NA	NA
Bc_ctg1_1494	B2	435.9161899	Bcer	C	Bcer	FALSE	NA	NA	NA
Bc_ctg1_1495	B2	354.3275982	Bcer	I	Bcer	FALSE	NA	NA	NA
Bc_ctg1_1496	B2	288.5905043	Bcer	K	Bcer	FALSE	NA	NA	NA
Bc_ctg1_1497	B2	28.67256223	Bcer	IQ	Bcer	TRUE	NA	NA	NA
Bc_ctg1_1498	B2	0.116555131	Bcer	Q	Bcer	TRUE	NA	NA	NA
Bc_ctg1_1499	B2	57.11201419	Bcer	P	Bcer	FALSE	NA	NA	NA
Bc_ctg1_15	B2	0.699330786	Bcer	V	Bcer	FALSE	NA	NA	NA
Bc_ctg1_150	B2	11.88862336	Bcer	C	Bcer	FALSE	NA	NA	NA
Bc_ctg1_1500	B2	31.81955076	Bcer	P	Bcer	FALSE	NA	NA	NA
Bc_ctg1_1501	B2	3.613209061	Bcer	NA	Bcer	FALSE	NA	NA	NA
Bc_ctg1_1502	B2	72.14762609	Bcer	S	Bcer	FALSE	NA	NA	NA
Bc_ctg1_1503	B2	3.962874454	Bcer	NA	Bcer	FALSE	NA	NA	NA
Bc_ctg1_1504	B2	39.39563428	Bcer	S	Bcer	FALSE	NA	NA	NA
Bc_ctg1_1505	B2	25.87523908	Bcer	S	Bcer	FALSE	NA	NA	NA
Bc_ctg1_1506	B2	23.3110262	Bcer	S	Bcer	FALSE	NA	NA	NA
Bc_ctg1_1507	B2	1.515216703	Bcer	NA	Bcer	FALSE	NA	NA	NA
Bc_ctg1_1508	B2	24.70968777	Bcer	V	Bcer	FALSE	NA	NA	NA
Bc_ctg1_1509	B2	59.32656168	Bcer	T	Bcer	FALSE	NA	NA	NA
Bc_ctg1_151	B2	4.778760371	Bcer	EGP	Bcer	FALSE	NA	NA	NA
Bc_ctg1_1510	B2	43.59161899	Bcer	T	Bcer	FALSE	NA	NA	NA
Bc_ctg1_1511	B2	0.815885917	Bcer	NA	Bcer	FALSE	NA	NA	NA
Bc_ctg1_1512	B2	0.116555131	Bcer	S	Bcer	FALSE	NA	NA	NA
Bc_ctg1_1513	B2	54.43124618	Bcer	J	Bcer	FALSE	NA	NA	NA
Bc_ctg1_1514	B2	11.30584771	Bcer	C	Bcer	FALSE	NA	NA	NA
Bc_ctg1_1515	B2	5.594646288	Bcer	S	Bcer	FALSE	NA	NA	NA
Bc_ctg1_1516	B2	6.99330786	Bcer	HP	Bcer	FALSE	NA	NA	NA
Bc_ctg1_1517	B2	3.846319323	Bcer	P	Bcer	FALSE	NA	NA	NA
Bc_ctg1_1518	B2	4.429094978	Bcer	P	Bcer	FALSE	NA	NA	NA
Bc_ctg1_1519	B2	28.78911736	Bcer	M	Bcer	FALSE	NA	NA	NA
Bc_ctg1_1520	B2	11.30584771	Bcer	M	Bcer	FALSE	NA	NA	NA
Bc_ctg1_1521	B2	19.34815175	Bcer	M	Bcer	FALSE	NA	NA	NA
Bc_ctg1_1522	B2	46.73860753	Bcer	E	Bcer	FALSE	NA	NA	NA

Bc_ctg1_1523	B2	10.25685153	Bcer	J	Bcer	FALSE	NA	NA	NA
Bc_ctg1_1525	B2	300.3625726	Bcer	L	Bcer	FALSE	NA	NA	NA
Bc_ctg1_1526	B2	104.2002871	Bcer	L	Bcer	FALSE	NA	NA	NA
Bc_ctg1_1527	B2	75.99394541	Bcer	S	Bcer	FALSE	NA	NA	NA
Bc_ctg1_1528	B2	32.05266102	Bcer	D	Bcer	FALSE	NA	NA	NA
Bc_ctg1_1529	B2	19.58126201	Bcer	L	Bcer	FALSE	NA	NA	NA
Bc_ctg1_153	B2	36.481756	Bcer	EU	Bcer	FALSE	NA	NA	NA
Bc_ctg1_1530	B2	5.594646288	Bcer	NA	Bcer	FALSE	NA	NA	NA
Bc_ctg1_1531	B2	2.680768013	Bcer	NA	Bcer	FALSE	NA	NA	NA
Bc_ctg1_1533	B2	389.2941375	Bcer	J	Bcer	FALSE	NA	NA	NA
Bc_ctg1_1534	B2	1142.123729	Bcer	J	Bcer	FALSE	NA	NA	NA
Bc_ctg1_1535	B2	361.4374612	Bcer	J	Bcer	FALSE	NA	NA	NA
Bc_ctg1_1536	B2	94.52621124	Bcer	J	Bcer	FALSE	NA	NA	NA
Bc_ctg1_1538	B2	17.83293504	Bcer	S	Bcer	FALSE	NA	NA	NA
Bc_ctg1_1539	B2	14.80250164	Bcer	S	Bcer	FALSE	NA	NA	NA
Bc_ctg1_154	B2	1.282106441	Bcer	NA	Bcer	FALSE	NA	NA	NA
Bc_ctg1_1540	B2	6.99330786	Bcer	NA	Bcer	FALSE	NA	NA	NA
Bc_ctg1_1541	B2	11.18929258	Bcer	NA	Bcer	FALSE	NA	NA	NA
Bc_ctg1_1542	B2	206.3025819	Bcer	EGP	Bcer	FALSE	NA	NA	NA
Bc_ctg1_1543	B2	50.81803711	Bcer	V	Bcer	FALSE	NA	NA	NA
Bc_ctg1_1544	B2	14.45283624	Bcer	K	Bcer	FALSE	NA	NA	NA
Bc_ctg1_1545	B2	1062.166909	Bcer	G	Bcer	FALSE	NA	NA	NA
Bc_ctg1_1546	B2	140.4489329	Bcer	S	Bcer	FALSE	NA	NA	NA
Bc_ctg1_1547	B2	426.7083346	Bcer	J	Bcer	FALSE	NA	NA	NA
Bc_ctg1_1548	B2	259.4517216	Bcer	J	Bcer	FALSE	NA	NA	NA
Bc_ctg1_1549	B2	848.7544639	Bcer	J	Bcer	FALSE	NA	NA	NA
Bc_ctg1_1550	B2	2500.34067	Bcer	J	Bcer	FALSE	NA	NA	NA
Bc_ctg1_1551	B2	0.932441048	Bcer	S	Bcer	FALSE	NA	NA	NA
Bc_ctg1_1552	B2	1.515216703	Bcer	S	Bcer	FALSE	NA	NA	NA
Bc_ctg1_1553	B2	0.349665393	Bcer	L	Bcer	FALSE	NA	NA	NA
Bc_ctg1_1554	B2	0.699330786	Bcer	L	Bcer	FALSE	NA	NA	NA
Bc_ctg1_1555	B2	67.13575545	Bcer	L	Bcer	FALSE	NA	NA	NA
Bc_ctg1_1556	B2	49.53593067	Bcer	L	Bcer	FALSE	NA	NA	NA
Bc_ctg1_1557	B2	23.54413646	Bcer	K	Bcer	FALSE	NA	NA	NA
Bc_ctg1_1558	B2	232.4109312	Bcer	E	Bcer	FALSE	NA	NA	NA
Bc_ctg1_1559	B2	1123.125242	Bcer	G	Bcer	FALSE	NA	NA	NA
Bc_ctg1_156	B2	35.89898035	Bcer	E	Bcer	FALSE	NA	NA	NA
Bc_ctg1_1560	B2	11.88862336	Bcer	F	Bcer	FALSE	NA	NA	NA
Bc_ctg1_1561	B2	83.45347379	Bcer	P	Bcer	FALSE	NA	NA	NA
Bc_ctg1_1562	B2	203.738369	Bcer	L	Bcer	FALSE	NA	NA	NA
Bc_ctg1_1563	B2	389.5272478	Bcer	L	Bcer	FALSE	NA	NA	NA
Bc_ctg1_1564	B2	120.6345606	Bcer	T	Bcer	FALSE	NA	NA	NA
Bc_ctg1_1565	B2	52.33325382	Bcer	T	Bcer	FALSE	NA	NA	NA
Bc_ctg1_1566	B2	0.233110262	Bcer	NA	Bcer	FALSE	NA	NA	NA
Bc_ctg1_1567	B2	0	Bcer	NA	Bcer	FALSE	NA	NA	NA
Bc_ctg1_1568	B2	41.61018177	Bcer	I	Bcer	FALSE	NA	NA	NA
Bc_ctg1_1569	B2	1958.359311	Bcer	C	Bcer	FALSE	NA	NA	NA
Bc_ctg1_157	B2	16.90049399	Bcer	S	Bcer	FALSE	NA	NA	NA
Bc_ctg1_1570	B2	2800.470132	Bcer	C	Bcer	FALSE	NA	NA	NA
Bc_ctg1_1571	B2	1978.756459	Bcer	C	Bcer	FALSE	NA	NA	NA
Bc_ctg1_1572	B2	4.429094978	Bcer	S	Bcer	FALSE	NA	NA	NA
Bc_ctg1_1573	B2	113.1750322	Bcer	S	Bcer	FALSE	NA	NA	NA
Bc_ctg1_1574	B2	1.748326965	Bcer	S	Bcer	FALSE	NA	NA	NA
Bc_ctg1_1575	B2	2487.752716	Bcer	G	Bcer	FALSE	NA	NA	NA
Bc_ctg1_1576	B2	773.5764044	Bcer	F	Bcer	FALSE	NA	NA	NA
Bc_ctg1_1577	B2	323.6735988	Bcer	I	Bcer	FALSE	NA	NA	NA
Bc_ctg1_1578	B2	303.8592265	Bcer	I	Bcer	FALSE	NA	NA	NA
Bc_ctg1_1579	B2	329.0351348	Bcer	K	Bcer	FALSE	NA	NA	NA
Bc_ctg1_158	B2	5.944311681	Bcer	S	Bcer	FALSE	NA	NA	NA
Bc_ctg1_1580	B2	1140.958177	Bcer	C	Bcer	FALSE	NA	NA	NA
Bc_ctg1_1581	B2	199.8920497	Bcer	L	Bcer	FALSE	NA	NA	NA
Bc_ctg1_1582	B2	0.466220524	Bcer	S	Bcer	FALSE	NA	NA	NA

Bc_ctg1_1583	B2	1.048996179	Bcer	NA	Bcer	FALSE	NA	NA	NA
Bc_ctg1_1584	B2	148.1415715	Bcer	S	Bcer	FALSE	NA	NA	NA
Bc_ctg1_1585	B2	3.380098799	Bcer	S	Bcer	FALSE	NA	NA	NA
Bc_ctg1_1586	B2	7.809193777	Bcer	S	Bcer	FALSE	NA	NA	NA
Bc_ctg1_1587	B2	125.1802107	Bcer	K	Bcer	FALSE	NA	NA	NA
Bc_ctg1_1588	B2	0.233110262	Bcer	NA	Bcer	FALSE	NA	NA	NA
Bc_ctg1_1589	B2	127.1616479	Bcer	S	Bcer	FALSE	NA	NA	NA
Bc_ctg1_159	B2	0.699330786	Bcer	S	Bcer	FALSE	NA	NA	NA
Bc_ctg1_1590	B2	300.712238	Bcer	E	Bcer	FALSE	NA	NA	NA
Bc_ctg1_1591	B2	3.962874454	Bcer	S	Bcer	FALSE	NA	NA	NA
Bc_ctg1_1592	B2	1.631771834	Bcer	NA	Bcer	FALSE	NA	NA	NA
Bc_ctg1_1593	B2	6.643642467	Bcer	S	Bcer	FALSE	NA	NA	NA
Bc_ctg1_1594	B2	0	Bcer	NA	Bcer	FALSE	NA	NA	NA
Bc_ctg1_1595	B2	3.146988537	Bcer	NA	Bcer	FALSE	NA	NA	NA
Bc_ctg1_1596	B2	5.244980895	Bcer	J	Bcer	FALSE	NA	NA	NA
Bc_ctg1_1597	B2	3.846319323	Bcer	G	Bcer	FALSE	NA	NA	NA
Bc_ctg1_1598	B2	1343.997216	Bcer	E	Bcer	FALSE	NA	NA	NA
Bc_ctg1_1599	B2	49.76904094	Bcer	IQ	Bcer	TRUE	NA	NA	NA
Bc_ctg1_16	B2	0.815885917	Bcer	U	Bcer	FALSE	NA	NA	NA
Bc_ctg1_160	B2	8.15885917	Bcer	Q	Bcer	TRUE	NA	NA	NA
Bc_ctg1_1600	B2	640.0042243	Bcer	T	Bcer	FALSE	NA	NA	NA
Bc_ctg1_1601	B2	18.88193122	Bcer	NA	Bcer	FALSE	NA	NA	NA
Bc_ctg1_1602	B2	0.233110262	Bcer	NA	Bcer	FALSE	NA	NA	NA
Bc_ctg1_1603	B2	109.3287129	Bcer	E	Bcer	FALSE	NA	NA	NA
Bc_ctg1_1604	B2	102.2188499	Bcer	E	Bcer	FALSE	NA	NA	NA
Bc_ctg1_1605	B2	12.58795415	Bcer	NA	Bcer	FALSE	NA	NA	NA
Bc_ctg1_1606	B2	0.582775655	Bcer	NA	Bcer	FALSE	NA	NA	NA
Bc_ctg1_1608	B2	327.403363	Bcer	S	Bcer	FALSE	NA	NA	NA
Bc_ctg1_1609	B2	77.27605185	Bcer	S	Bcer	FALSE	NA	NA	NA
Bc_ctg1_161	B2	0.932441048	Bcer	E	Bcer	FALSE	NA	NA	NA
Bc_ctg1_1610	B2	0.233110262	Bcer	S	Bcer	FALSE	NA	NA	NA
Bc_ctg1_1611	B2	1.282106441	Bcer	F	Bcer	FALSE	NA	NA	NA
Bc_ctg1_1612	B2	290.6884967	Bcer	F	Bcer	FALSE	NA	NA	NA
Bc_ctg1_1613	B2	16.43427347	Bcer	L	Bcer	FALSE	NA	NA	NA
Bc_ctg1_1614	B2	456.3133379	Bcer	O	Bcer	FALSE	NA	NA	NA
Bc_ctg1_1615	B2	1.048996179	Bcer	S	Bcer	FALSE	NA	NA	NA
Bc_ctg1_1616	B2	0.116555131	Bcer	S	Bcer	FALSE	NA	NA	NA
Bc_ctg1_1617	B2	1170.563181	Bcer	G	Bcer	FALSE	NA	NA	NA
Bc_ctg1_1618	B2	14.45283624	Bcer	S	Bcer	FALSE	NA	NA	NA
Bc_ctg1_1619	B2	49.06971015	Bcer	S	Bcer	FALSE	NA	NA	NA
Bc_ctg1_162	B2	486.734227	Bcer	L	Bcer	FALSE	NA	NA	NA
Bc_ctg1_1620	B2	3.846319323	Bcer	I	Bcer	FALSE	NA	NA	NA
Bc_ctg1_1621	B2	25.40901856	Bcer	S	Bcer	FALSE	NA	NA	NA
Bc_ctg1_1622	B2	264.1139268	Bcer	H	Bcer	FALSE	NA	NA	NA
Bc_ctg1_1623	B2	114.2240284	Bcer	E	Bcer	FALSE	NA	NA	NA
Bc_ctg1_1624	B2	448.7372543	Bcer	D	Bcer	FALSE	NA	NA	NA
Bc_ctg1_1625	B2	2.214547489	Bcer	K	Bcer	FALSE	NA	NA	NA
Bc_ctg1_1626	B2	0.349665393	Bcer	EG	Bcer	FALSE	NA	NA	NA
Bc_ctg1_1627	B2	26.92423526	Bcer	K	Bcer	FALSE	NA	NA	NA
Bc_ctg1_1628	B2	23.42758133	Bcer	T	Bcer	FALSE	NA	NA	NA
Bc_ctg1_1629	B2	6.177421943	Bcer	E	Bcer	FALSE	NA	NA	NA
Bc_ctg1_163	B2	24.59313264	Bcer	V	Bcer	FALSE	NA	NA	NA
Bc_ctg1_1630	B2	2590.088121	Bcer	J	Bcer	FALSE	NA	NA	NA
Bc_ctg1_1631	B2	1.398661572	Bcer	S	Bcer	FALSE	NA	NA	NA
Bc_ctg1_1632	B2	0	Bcer	NA	Bcer	FALSE	NA	NA	NA
Bc_ctg1_1633	B2	172.3850387	Bcer	J	Bcer	FALSE	NA	NA	NA
Bc_ctg1_1634	B2	3.030433406	Bcer	NA	Bcer	FALSE	NA	NA	NA
Bc_ctg1_1635	B2	29.48844814	Bcer	K	Bcer	FALSE	NA	NA	NA
Bc_ctg1_1636	B2	12.47139902	Bcer	NA	Bcer	FALSE	NA	NA	NA
Bc_ctg1_1637	B2	243.0174481	Bcer	S	Bcer	FALSE	NA	NA	NA
Bc_ctg1_1638	B2	111.7763706	Bcer	K	Bcer	FALSE	NA	NA	NA
Bc_ctg1_1639	B2	407.476738	Bcer	I	Bcer	FALSE	NA	NA	NA

Bc_ctg1_164	B2	79.95681986	Bcer	V	Bcer	FALSE	NA	NA	NA
Bc_ctg1_1640	B2	87.29979312	Bcer	K	Bcer	FALSE	NA	NA	NA
Bc_ctg1_1641	B2	84.73558023	Bcer	S	Bcer	FALSE	NA	NA	NA
Bc_ctg1_1642	B2	60.14244759	Bcer	BQ	Bcer	TRUE	NA	NA	NA
Bc_ctg1_1643	B2	3.729764192	Bcer	I	Bcer	FALSE	NA	NA	NA
Bc_ctg1_1644	B2	10.60651692	Bcer	NA	Bcer	FALSE	NA	NA	NA
Bc_ctg1_1645	B2	64.92120797	Bcer	T	Bcer	FALSE	NA	NA	NA
Bc_ctg1_1646	B2	46.6220524	Bcer	T	Bcer	FALSE	NA	NA	NA
Bc_ctg1_1647	B2	0.116555131	Bcer	S	Bcer	FALSE	NA	NA	NA
Bc_ctg1_1648	B2	119.9352298	Bcer	S	Bcer	FALSE	NA	NA	NA
Bc_ctg1_1649	B2	84.50246997	Bcer	M	Bcer	FALSE	NA	NA	NA
Bc_ctg1_165	B2	17.83293504	Bcer	K	Bcer	FALSE	NA	NA	NA
Bc_ctg1_1650	B2	11.53895797	Bcer	S	Bcer	FALSE	NA	NA	NA
Bc_ctg1_1651	B2	3.613209061	Bcer	S	Bcer	FALSE	NA	NA	NA
Bc_ctg1_1652	B2	14.91905677	Bcer	S	Bcer	FALSE	NA	NA	NA
Bc_ctg1_1653	B2	246.1644367	Bcer	K	Bcer	FALSE	NA	NA	NA
Bc_ctg1_1654	B2	1.16555131	Bcer	E	Bcer	FALSE	NA	NA	NA
Bc_ctg1_1655	B2	0.582775655	Bcer	E	Bcer	FALSE	NA	NA	NA
Bc_ctg1_1657	B2	139.8661572	Bcer	S	Bcer	FALSE	NA	NA	NA
Bc_ctg1_1658	B2	65.27087336	Bcer	O	Bcer	FALSE	NA	NA	NA
Bc_ctg1_1659	B2	69.9330786	Bcer	NA	Bcer	FALSE	NA	NA	NA
Bc_ctg1_166	B2	3.146988537	Bcer	G	Bcer	FALSE	NA	NA	NA
Bc_ctg1_1660	B2	852.367673	Bcer	E	Bcer	FALSE	NA	NA	NA
Bc_ctg1_1661	B2	16.43427347	Bcer	S	Bcer	FALSE	NA	NA	NA
Bc_ctg1_1662	B2	8.042304039	Bcer	K	Bcer	FALSE	NA	NA	NA
Bc_ctg1_1663	B2	4.312539847	Bcer	J	Bcer	FALSE	NA	NA	NA
Bc_ctg1_1664	B2	3.146988537	Bcer	EG	Bcer	FALSE	NA	NA	NA
Bc_ctg1_1665	B2	0	Bcer	K	Bcer	FALSE	NA	NA	NA
Bc_ctg1_1666	B2	830.105643	Bcer	M	Bcer	FALSE	NA	NA	NA
Bc_ctg1_1667	B2	531.8410627	Bcer	H	Bcer	FALSE	NA	NA	NA
Bc_ctg1_1668	B2	591.8669552	Bcer	D	Bcer	FALSE	NA	NA	NA
Bc_ctg1_1669	B2	3.146988537	Bcer	M	Bcer	FALSE	Bc_Ga0417192_01_rn12	Other	NA
Bc_ctg1_167	B2	4.312539847	Bcer	S	Bcer	FALSE	NA	NA	NA
Bc_ctg1_1670	B2	0	Bcer	NA	Bcer	FALSE	Bc_Ga0417192_01_rn12	Other	NA
Bc_ctg1_1671	B2	239.4042391	Bcer	J	Bcer	FALSE	Bc_Ga0417192_01_rn12	Other	NA
Bc_ctg1_1672	B2	144.178697	Bcer	S	Bcer	FALSE	Bc_Ga0417192_01_rn12	Other	NA
Bc_ctg1_1673	B2	43.2419536	Bcer	CO	Bcer	FALSE	Bc_Ga0417192_01_rn12	Other	NA
Bc_ctg1_1674	B2	2.680768013	Bcer	F	Bcer	FALSE	Bc_Ga0417192_01_rn12	Other	NA
Bc_ctg1_1675	B2	276.0025502	Bcer	G	Bcer	FALSE	Bc_Ga0417192_01_rn12	Ancillary biosynthesis	None
Bc_ctg1_1676	B2	9.790631004	Bcer	S	Bcer	FALSE	Bc_Ga0417192_01_rn12	Other	NA
Bc_ctg1_1677	B2	31.70299563	Bcer	S	Bcer	FALSE	Bc_Ga0417192_01_rn12	Other	NA
Bc_ctg1_1678	B2	34.61687391	Bcer	J	Bcer	FALSE	Bc_Ga0417192_01_rn12	Other	NA
Bc_ctg1_1679	B2	0	Bcer	S	Bcer	FALSE	Bc_Ga0417192_01_rn12	Other	NA
Bc_ctg1_168	B2	28.78911736	Bcer	T	Bcer	FALSE	NA	NA	NA
Bc_ctg1_1680	B2	80.073375	Bcer	M	Bcer	FALSE	Bc_Ga0417192_01_rn12	Ancillary biosynthesis	None
Bc_ctg1_1681	B2	26.22490447	Bcer	G	Bcer	FALSE	Bc_Ga0417192_01_rn12	Core biosynthesis	SMCOG1297
Bc_ctg1_1682	B2	3.846319323	Bcer	J	Bcer	FALSE	Bc_Ga0417192_01_rn12	Other	NA
Bc_ctg1_1683	B2	275.5363297	Bcer	E	Bcer	FALSE	Bc_Ga0417192_01_rn12	Ancillary biosynthesis	SMCOG1247
Bc_ctg1_1684	B2	245.9313264	Bcer	K	Bcer	FALSE	Bc_Ga0417192_01_rn12	Other	NA
Bc_ctg1_1685	B2	43.00884334	Bcer	J	Bcer	FALSE	Bc_Ga0417192_01_rn12	Ancillary biosynthesis	SMCOG1154
Bc_ctg1_1686	B2	277.634322	Bcer	S	Bcer	FALSE	Bc_Ga0417192_01_rn12	Core biosynthesis	SMCOG1086
Bc_ctg1_1687	B2	72.84695687	Bcer	S	Bcer	FALSE	Bc_Ga0417192_01_rn12	Other	NA
Bc_ctg1_1688	B2	112.3591463	Bcer	P	Bcer	FALSE	Bc_Ga0417192_01_rn12	Transport	SMCOG1005
Bc_ctg1_1689	B2	6.410532205	Bcer	K	Bcer	FALSE	Bc_Ga0417192_01_rn12	Regulation	SMCOG1058
Bc_ctg1_169	B2	14.21972598	Bcer	T	Bcer	FALSE	NA	NA	NA
Bc_ctg1_1690	B2	13.28728493	Bcer	S	Bcer	FALSE	Bc_Ga0417192_01_rn12	Other	NA
Bc_ctg1_1691	B2	0	Bcer	S	Bcer	FALSE	Bc_Ga0417192_01_rn12	Other	NA
Bc_ctg1_1693	B2	0	Bcer	S	Bcer	FALSE	Bc_Ga0417192_01_rn12	Other	NA
Bc_ctg1_1694	B2	640.7035551	Bcer	K	Bcer	FALSE	Bc_Ga0417192_01_rn12	Other	NA
Bc_ctg1_1695	B2	2.214547489	Bcer	IQ	Bcer	TRUE	Bc_Ga0417192_01_rn12	Ancillary biosynthesis	SMCOG1001
Bc_ctg1_1696	B2	0	Bcer	U	Bcer	FALSE	Bc_Ga0417192_01_rn12	Other	NA
Bc_ctg1_1697	B2	0	Bcer	S	Bcer	FALSE	Bc_Ga0417192_01_rn12	Other	NA

Bc_ctg1_1698	B2	4.312539847	Bcer	H	Bcer	FALSE	Bc_Ga0417192_01_rn12	Other	NA
Bc_ctg1_1699	B2	13.28728493	Bcer	H	Bcer	FALSE	Bc_Ga0417192_01_rn12	Other	NA
Bc_ctg1_17	B2	15.26872216	Bcer	M	Bcer	FALSE	NA	NA	NA
Bc_ctg1_170	B2	14.10317085	Bcer	S	Bcer	FALSE	NA	NA	NA
Bc_ctg1_1700	B2	45.45650109	Bcer	H	Bcer	FALSE	Bc_Ga0417192_01_rn12	Other	NA
Bc_ctg1_1701	B2	109.0956026	Bcer	H	Bcer	FALSE	Bc_Ga0417192_01_rn12	Other	NA
Bc_ctg1_1702	B2	13.5203952	Bcer	H	Bcer	FALSE	NA	NA	NA
Bc_ctg1_1703	B2	46.97171779	Bcer	H	Bcer	FALSE	NA	NA	NA
Bc_ctg1_1704	B2	54.08158078	Bcer	H	Bcer	FALSE	NA	NA	NA
Bc_ctg1_1705	B2	6.410532205	Bcer	Q	Bcer	TRUE	NA	NA	NA
Bc_ctg1_1706	B2	6.876752729	Bcer	NA	Bcer	FALSE	NA	NA	NA
Bc_ctg1_1707	B2	0.815885917	Bcer	NA	Bcer	FALSE	NA	NA	NA
Bc_ctg1_1708	B2	3.146988537	Bcer	P	Bcer	FALSE	NA	NA	NA
Bc_ctg1_1709	B2	0.466220524	Bcer	NA	Bcer	FALSE	NA	NA	NA
Bc_ctg1_171	B2	26.45801474	Bcer	NA	Bcer	FALSE	NA	NA	NA
Bc_ctg1_1710	B2	4.195984716	Bcer	E	Bcer	FALSE	NA	NA	NA
Bc_ctg1_1711	B2	0.466220524	Bcer	P	Bcer	FALSE	NA	NA	NA
Bc_ctg1_1712	B2	0.582775655	Bcer	E	Bcer	FALSE	NA	NA	NA
Bc_ctg1_1713	B2	1.981437227	Bcer	S	Bcer	FALSE	NA	NA	NA
Bc_ctg1_1714	B2	2.214547489	Bcer	NA	Bcer	FALSE	NA	NA	NA
Bc_ctg1_1715	B2	76.11050054	Bcer	S	Bcer	FALSE	NA	NA	NA
Bc_ctg1_1716	B2	0.116555131	Bcer	NA	Bcer	FALSE	NA	NA	NA
Bc_ctg1_1718	B2	0.116555131	Bcer	NA	Bcer	FALSE	NA	NA	NA
Bc_ctg1_1719	B2	739.5423062	Bcer	J	Bcer	FALSE	NA	NA	NA
Bc_ctg1_172	B2	26.45801474	Bcer	V	Bcer	FALSE	NA	NA	NA
Bc_ctg1_1720	B2	12.93761954	Bcer	EGP	Bcer	FALSE	NA	NA	NA
Bc_ctg1_1721	B2	19.46470688	Bcer	P	Bcer	FALSE	NA	NA	NA
Bc_ctg1_1722	B2	10.83962718	Bcer	P	Bcer	FALSE	NA	NA	NA
Bc_ctg1_1723	B2	11.07273744	Bcer	L	Bcer	FALSE	NA	NA	NA
Bc_ctg1_1724	B2	69.23374781	Bcer	V	Bcer	FALSE	NA	NA	NA
Bc_ctg1_1725	B2	11.6555131	Bcer	V	Bcer	FALSE	NA	NA	NA
Bc_ctg1_1726	B2	1.048996179	Bcer	S	Bcer	FALSE	NA	NA	NA
Bc_ctg1_1728	B2	0.233110262	Bcer	S	Bcer	FALSE	NA	NA	NA
Bc_ctg1_1729	B2	24.12691212	Bcer	S	Bcer	FALSE	NA	NA	NA
Bc_ctg1_173	B2	7.692638646	Bcer	S	Bcer	FALSE	NA	NA	NA
Bc_ctg1_1730	B2	21.56269923	Bcer	M	Bcer	FALSE	NA	NA	NA
Bc_ctg1_1731	B2	7.809193777	Bcer	V	Bcer	FALSE	NA	NA	NA
Bc_ctg1_1732	B2	4.778760371	Bcer	NA	Bcer	FALSE	NA	NA	NA
Bc_ctg1_1733	B2	5.82775655	Bcer	M	Bcer	FALSE	NA	NA	NA
Bc_ctg1_1734	B2	16.08460808	Bcer	S	Bcer	FALSE	NA	NA	NA
Bc_ctg1_1735	B2	193.4815175	Bcer	S	Bcer	FALSE	NA	NA	NA
Bc_ctg1_1736	B2	26.10834934	Bcer	I	Bcer	FALSE	NA	NA	NA
Bc_ctg1_1737	B2	30.42088919	Bcer	S	Bcer	FALSE	NA	NA	NA
Bc_ctg1_1738	B2	0.582775655	Bcer	I	Bcer	FALSE	NA	NA	NA
Bc_ctg1_1739	B2	0	Bcer	M	Bcer	FALSE	NA	NA	NA
Bc_ctg1_174	B2	0	Bcer	I	Bcer	FALSE	NA	NA	NA
Bc_ctg1_1740	B2	20.86336845	Bcer	H	Bcer	FALSE	NA	NA	NA
Bc_ctg1_1741	B2	20.63025819	Bcer	H	Bcer	FALSE	NA	NA	NA
Bc_ctg1_1742	B2	0.932441048	Bcer	NA	Bcer	FALSE	NA	NA	NA
Bc_ctg1_1743	B2	1470.342978	Bcer	H	Bcer	FALSE	NA	NA	NA
Bc_ctg1_1744	B2	2732.168826	Bcer	C	Bcer	FALSE	NA	NA	NA
Bc_ctg1_1745	B2	0	Bcer	S	Bcer	FALSE	NA	NA	NA
Bc_ctg1_1746	B2	0	Bcer	NA	Bcer	FALSE	NA	NA	NA
Bc_ctg1_1747	B2	6.643642467	Bcer	EG	Bcer	FALSE	NA	NA	NA
Bc_ctg1_1748	B2	4.195984716	Bcer	S	Bcer	FALSE	NA	NA	NA
Bc_ctg1_1749	B2	9.091300218	Bcer	S	Bcer	FALSE	NA	NA	NA
Bc_ctg1_175	B2	255.7219574	Bcer	S	Bcer	FALSE	NA	NA	NA
Bc_ctg1_1750	B2	1.981437227	Bcer	Q	Bcer	TRUE	NA	NA	NA
Bc_ctg1_1751	B2	1.282106441	Bcer	S	Bcer	FALSE	NA	NA	NA
Bc_ctg1_1752	B2	0.582775655	Bcer	S	Bcer	FALSE	NA	NA	NA
Bc_ctg1_1753	B2	0.233110262	Bcer	S	Bcer	FALSE	NA	NA	NA
Bc_ctg1_1754	B2	0.116555131	Bcer	NA	Bcer	FALSE	NA	NA	NA

Bc_ctg1_1755	B2	0.116555131	Bcer	NU	Bcer	FALSE	NA	NA	NA
Bc_ctg1_1756	B2	23.42758133	Bcer	T	Bcer	FALSE	NA	NA	NA
Bc_ctg1_1757	B2	5.82775655	Bcer	T	Bcer	FALSE	NA	NA	NA
Bc_ctg1_1758	B2	0.349665393	Bcer	S	Bcer	FALSE	NA	NA	NA
Bc_ctg1_1759	B2	97.32353438	Bcer	M	Bcer	FALSE	NA	NA	NA
Bc_ctg1_176	B2	95.57520742	Bcer	EH	Bcer	FALSE	NA	NA	NA
Bc_ctg1_1760	B2	4.778760371	Bcer	NA	Bcer	FALSE	NA	NA	NA
Bc_ctg1_1761	B2	3.962874454	Bcer	S	Bcer	FALSE	NA	NA	NA
Bc_ctg1_1762	B2	2.447657751	Bcer	P	Bcer	FALSE	NA	NA	NA
Bc_ctg1_1763	B2	2.447657751	Bcer	P	Bcer	FALSE	NA	NA	NA
Bc_ctg1_1764	B2	4.429094978	Bcer	P	Bcer	FALSE	NA	NA	NA
Bc_ctg1_1765	B2	0	Bcer	NA	Bcer	FALSE	NA	NA	NA
Bc_ctg1_1766	B2	15.85149782	Bcer	NA	Bcer	FALSE	NA	NA	NA
Bc_ctg1_1767	B2	8.508524563	Bcer	S	Bcer	FALSE	NA	NA	NA
Bc_ctg1_1768	B2	6.060866812	Bcer	S	Bcer	FALSE	NA	NA	NA
Bc_ctg1_1769	B2	144.2952522	Bcer	L	Bcer	FALSE	NA	NA	NA
Bc_ctg1_177	B2	48.83659989	Bcer	E	Bcer	FALSE	NA	NA	NA
Bc_ctg1_1770	B2	0.932441048	Bcer	NA	Bcer	FALSE	NA	NA	NA
Bc_ctg1_1772	B2	0.466220524	Bcer	NA	Bcer	FALSE	NA	NA	NA
Bc_ctg1_1773	B2	0	Bcer	NA	Bcer	FALSE	NA	NA	NA
Bc_ctg1_1774	B2	192.6656315	Bcer	T	Bcer	FALSE	NA	NA	NA
Bc_ctg1_1775	B2	0.466220524	Bcer	S	Bcer	FALSE	NA	NA	NA
Bc_ctg1_1776	B2	118.6531234	Bcer	P	Bcer	FALSE	NA	NA	NA
Bc_ctg1_1777	B2	475.5449345	Bcer	C	Bcer	FALSE	NA	NA	NA
Bc_ctg1_1778	B2	301.4115688	Bcer	C	Bcer	FALSE	NA	NA	NA
Bc_ctg1_1779	B2	0	Bcer	S	Bcer	FALSE	NA	NA	NA
Bc_ctg1_178	B2	0	Bcer	S	Bcer	FALSE	NA	NA	NA
Bc_ctg1_1780	B2	9.440965611	Bcer	S	Bcer	FALSE	NA	NA	NA
Bc_ctg1_1781	B2	0	Bcer	S	Bcer	FALSE	NA	NA	NA
Bc_ctg1_1782	B2	47.67104858	Bcer	S	Bcer	FALSE	NA	NA	NA
Bc_ctg1_1783	B2	16.43427347	Bcer	NA	Bcer	FALSE	NA	NA	NA
Bc_ctg1_1784	B2	245.2319956	Bcer	S	Bcer	FALSE	NA	NA	NA
Bc_ctg1_1785	B2	101.2864088	Bcer	S	Bcer	FALSE	NA	NA	NA
Bc_ctg1_1786	B2	34.03409825	Bcer	S	Bcer	FALSE	NA	NA	NA
Bc_ctg1_1787	B2	451.1849121	Bcer	KT	Bcer	FALSE	NA	NA	NA
Bc_ctg1_1788	B2	5.82775655	Bcer	S	Bcer	FALSE	NA	NA	NA
Bc_ctg1_179	B2	12.12173362	Bcer	G	Bcer	FALSE	NA	NA	NA
Bc_ctg1_1791	B2	1.16555131	Bcer	S	Bcer	FALSE	NA	NA	NA
Bc_ctg1_1792	B2	230.7791594	Bcer	P	Bcer	FALSE	NA	NA	NA
Bc_ctg1_1793	B2	12.12173362	Bcer	P	Bcer	FALSE	NA	NA	NA
Bc_ctg1_1794	B2	15.50183242	Bcer	S	Bcer	FALSE	NA	NA	NA
Bc_ctg1_1795	B2	65.38742849	Bcer	T	Bcer	FALSE	NA	NA	NA
Bc_ctg1_1796	B2	22.72825054	Bcer	T	Bcer	FALSE	NA	NA	NA
Bc_ctg1_1797	B2	30.77055458	Bcer	M	Bcer	FALSE	NA	NA	NA
Bc_ctg1_1798	B2	12.70450928	Bcer	NA	Bcer	FALSE	NA	NA	NA
Bc_ctg1_1799	B2	168.0724989	Bcer	NA	Bcer	FALSE	NA	NA	NA
Bc_ctg1_18	B2	27.50701092	Bcer	K	Bcer	FALSE	NA	NA	NA
Bc_ctg1_180	B2	0.815885917	Bcer	G	Bcer	FALSE	NA	NA	NA
Bc_ctg1_1800	B2	17.83293504	Bcer	NA	Bcer	FALSE	NA	NA	NA
Bc_ctg1_1801	B2	215.0442167	Bcer	F	Bcer	FALSE	NA	NA	NA
Bc_ctg1_1802	B2	131.2410775	Bcer	I	Bcer	FALSE	NA	NA	NA
Bc_ctg1_1803	B2	10.83962718	Bcer	S	Bcer	FALSE	NA	NA	NA
Bc_ctg1_1804	B2	8.858189956	Bcer	NA	Bcer	FALSE	NA	NA	NA
Bc_ctg1_1805	B2	10.1402964	Bcer	S	Bcer	FALSE	NA	NA	NA
Bc_ctg1_1806	B2	0.116555131	Bcer	NA	Bcer	FALSE	NA	NA	NA
Bc_ctg1_1807	B2	0.582775655	Bcer	NA	Bcer	FALSE	NA	NA	NA
Bc_ctg1_1808	B2	1.16555131	Bcer	S	Bcer	FALSE	NA	NA	NA
Bc_ctg1_1809	B2	1.16555131	Bcer	I	Bcer	FALSE	NA	NA	NA
Bc_ctg1_181	B2	1.748326965	Bcer	K	Bcer	FALSE	NA	NA	NA
Bc_ctg1_1810	B2	173.0843695	Bcer	V	Bcer	FALSE	NA	NA	NA
Bc_ctg1_1811	B2	46.38894214	Bcer	V	Bcer	FALSE	NA	NA	NA
Bc_ctg1_1812	B2	24.59313264	Bcer	K	Bcer	FALSE	NA	NA	NA

Bc_ctg1_1813	B2	19.9309274	Bcer	S	Bcer	FALSE	NA	NA	NA
Bc_ctg1_1814	B2	11.6555131	Bcer	S	Bcer	FALSE	NA	NA	NA
Bc_ctg1_1815	B2	3.380098799	Bcer	K	Bcer	FALSE	NA	NA	NA
Bc_ctg1_1816	B2	49.76904094	Bcer	K	Bcer	FALSE	NA	NA	NA
Bc_ctg1_1817	B2	79.7237096	Bcer	T	Bcer	FALSE	NA	NA	NA
Bc_ctg1_1818	B2	51.5173679	Bcer	V	Bcer	FALSE	NA	NA	NA
Bc_ctg1_1819	B2	46.50549727	Bcer	V	Bcer	FALSE	NA	NA	NA
Bc_ctg1_182	B2	5.711201419	Bcer	S	Bcer	FALSE	NA	NA	NA
Bc_ctg1_1820	B2	15.96805295	Bcer	V	Bcer	FALSE	NA	NA	NA
Bc_ctg1_1821	B2	51.5173679	Bcer	V	Bcer	FALSE	NA	NA	NA
Bc_ctg1_1823	B2	7.809193777	Bcer	EG	Bcer	FALSE	NA	NA	NA
Bc_ctg1_1824	B2	16.5508286	Bcer	K	Bcer	FALSE	NA	NA	NA
Bc_ctg1_1825	B2	13.1707298	Bcer	G	Bcer	FALSE	NA	NA	NA
Bc_ctg1_1826	B2	18.53226583	Bcer	S	Bcer	FALSE	NA	NA	NA
Bc_ctg1_1827	B2	19.9309274	Bcer	E	Bcer	FALSE	NA	NA	NA
Bc_ctg1_1828	B2	10.95618231	Bcer	S	Bcer	FALSE	NA	NA	NA
Bc_ctg1_1829	B2	4.079429585	Bcer	M	Bcer	FALSE	NA	NA	NA
Bc_ctg1_183	B2	29.60500327	Bcer	C	Bcer	FALSE	NA	NA	NA
Bc_ctg1_1830	B2	20.28059279	Bcer	T	Bcer	FALSE	NA	NA	NA
Bc_ctg1_1831	B2	10.83962718	Bcer	T	Bcer	FALSE	NA	NA	NA
Bc_ctg1_1832	B2	3.962874454	Bcer	H	Bcer	FALSE	NA	NA	NA
Bc_ctg1_1833	B2	8.275414301	Bcer	H	Bcer	FALSE	NA	NA	NA
Bc_ctg1_1834	B2	875.5621441	Bcer	H	Bcer	FALSE	NA	NA	NA
Bc_ctg1_1835	B2	45.1068357	Bcer	S	Bcer	FALSE	NA	NA	NA
Bc_ctg1_1836	B2	129.02653	Bcer	H	Bcer	FALSE	Bc_Ga0417192_01_rn13	Other	NA
Bc_ctg1_1837	B2	110.0280437	Bcer	HQ	Bcer	TRUE	Bc_Ga0417192_01_rn13	Ancillary biosynthesis	SMCOG1018
Bc_ctg1_1838	B2	17.48326965	Bcer	H	Bcer	FALSE	Bc_Ga0417192_01_rn13	Ancillary biosynthesis	None
Bc_ctg1_1839	B2	0.699330786	Bcer	T	Bcer	FALSE	Bc_Ga0417192_01_rn13	Other	NA
Bc_ctg1_184	B2	0.116555131	Bcer	L	Bcer	FALSE	NA	NA	NA
Bc_ctg1_1840	B2	221.4547489	Bcer	K	Bcer	FALSE	Bc_Ga0417192_01_rn13	Regulation	SMCOG1255
Bc_ctg1_1841	B2	93.82688045	Bcer	S	Bcer	FALSE	Bc_Ga0417192_01_rn13	Other	NA
Bc_ctg1_1842	B2	1.398661572	Bcer	S	Bcer	FALSE	Bc_Ga0417192_01_rn13	Other	NA
Bc_ctg1_1843	B2	1.748326965	Bcer	K	Bcer	FALSE	Bc_Ga0417192_01_rn13	Regulation	SMCOG1171
Bc_ctg1_1844	B2	0	Bcer	I	Bcer	FALSE	Bc_Ga0417192_01_rn13	Other	NA
Bc_ctg1_1845	B2	0	Bcer	NA	Bcer	FALSE	Bc_Ga0417192_01_rn13	Other	NA
Bc_ctg1_1846	B2	779.2876058	Bcer	G	Bcer	FALSE	Bc_Ga0417192_01_rn13	Other	NA
Bc_ctg1_1847	B2	321.1093859	Bcer	G	Bcer	FALSE	Bc_Ga0417192_01_rn13	Core biosynthesis	SMCOG1045
Bc_ctg1_1848	B2	306.6565497	Bcer	G	Bcer	FALSE	Bc_Ga0417192_01_rn13	Ancillary biosynthesis	SMCOG1064
Bc_ctg1_1849	B2	252.691524	Bcer	G	Bcer	FALSE	Bc_Ga0417192_01_rn13	Ancillary biosynthesis	SMCOG1064
Bc_ctg1_185	B2	36.8314214	Bcer	M	Bcer	FALSE	NA	NA	NA
Bc_ctg1_1850	B2	416.6845933	Bcer	G	Bcer	FALSE	Bc_Ga0417192_01_rn13	Core biosynthesis	SMCOG1297
Bc_ctg1_1852	B2	3.380098799	Bcer	O	Bcer	FALSE	Bc_Ga0417192_01_rn13	Ancillary biosynthesis	SMCOG1075
Bc_ctg1_1853	B2	8945.023528	Bcer	C	Bcer	FALSE	Bc_Ga0417192_01_rn13	Other	NA
Bc_ctg1_1855	B2	8.974745087	Bcer	S	Bcer	FALSE	Bc_Ga0417192_01_rn13	Other	NA
Bc_ctg1_1856	B2	0.932441048	Bcer	P	Bcer	FALSE	Bc_Ga0417192_01_rn13	Other	NA
Bc_ctg1_1857	B2	0.582775655	Bcer	S	Bcer	FALSE	Bc_Ga0417192_01_rn13	Other	NA
Bc_ctg1_1858	B2	1398.078796	Bcer	G	Bcer	FALSE	Bc_Ga0417192_01_rn13	Other	NA
Bc_ctg1_1859	B2	3.146988537	Bcer	S	Bcer	FALSE	Bc_Ga0417192_01_rn13	Other	NA
Bc_ctg1_186	B2	0.349665393	Bcer	NA	Bcer	FALSE	NA	NA	NA
Bc_ctg1_1860	B2	64.80465283	Bcer	J	Bcer	FALSE	Bc_Ga0417192_01_rn13	Other	NA
Bc_ctg1_1861	B2	110.0280437	Bcer	E	Bcer	FALSE	Bc_Ga0417192_01_rn13	Core biosynthesis	SMCOG1019
Bc_ctg1_1862	B2	3.146988537	Bcer	K	Bcer	FALSE	Bc_Ga0417192_01_rn13	Regulation	SMCOG1120
Bc_ctg1_1863	B2	123.5484389	Bcer	CH	Bcer	FALSE	Bc_Ga0417192_01_rn13	Ancillary biosynthesis	SMCOG1072
Bc_ctg1_1864	B2	0	Bcer	I	Bcer	FALSE	Bc_Ga0417192_01_rn13	Ancillary biosynthesis	SMCOG1262
Bc_ctg1_1865	B2	1.864882096	Bcer	S	Bcer	FALSE	Bc_Ga0417192_01_rn13	Other	NA
Bc_ctg1_1866	B2	55.48024235	Bcer	E	Bcer	FALSE	Bc_Ga0417192_01_rn13	Ancillary biosynthesis	SMCOG1019
Bc_ctg1_1867	B2	8.858189956	Bcer	P	Bcer	FALSE	Bc_Ga0417192_01_rn13	Other	NA
Bc_ctg1_1868	B2	0	Bcer	NA	Bcer	FALSE	Bc_Ga0417192_01_rn13	Other	NA
Bc_ctg1_1869	B2	53.38225	Bcer	G	Bcer	FALSE	Bc_Ga0417192_01_rn13	Other	NA
Bc_ctg1_187	B2	96.15798307	Bcer	L	Bcer	FALSE	NA	NA	NA
Bc_ctg1_1870	B2	4.195984716	Bcer	L	Bcer	FALSE	Bc_Ga0417192_01_rn13	Other	NA
Bc_ctg1_1871	B2	46.50549727	Bcer	P	Bcer	FALSE	Bc_Ga0417192_01_rn13	Other	NA

Bc_ctg1_1872	B2	0.932441048	Bcer	NA	Bcer	FALSE	Bc_Ga0417192_01_rn13	Other	NA
Bc_ctg1_1873	B2	6.177421943	Bcer	L	Bcer	FALSE	Bc_Ga0417192_01_rn13	Other	NA
Bc_ctg1_1874	B2	554.2196479	Bcer	J	Bcer	FALSE	Bc_Ga0417192_01_rn13	Other	NA
Bc_ctg1_1875	B2	40.67774072	Bcer	Q	Bcer	TRUE	Bc_Ga0417192_01_rn13	Ancillary biosynthesis	SMCOG1144
Bc_ctg1_1876	B2	0	Bcer	S	Bcer	FALSE	Bc_Ga0417192_01_rn13	Other	NA
Bc_ctg1_1877	B2	14.91905677	Bcer	S	Bcer	FALSE	Bc_Ga0417192_01_rn13	Other	NA
Bc_ctg1_1878	B2	15.15216703	Bcer	S	Bcer	FALSE	Bc_Ga0417192_01_rn13	Other	NA
Bc_ctg1_1879	B2	253.7405202	Bcer	M	Bcer	FALSE	Bc_Ga0417192_01_rn13	Core biosynthesis	SMCOG1064
Bc_ctg1_188	B2	1.282106441	Bcer	NA	Bcer	FALSE	NA	NA	NA
Bc_ctg1_1880	B2	664.1311364	Bcer	G	Bcer	FALSE	Bc_Ga0417192_01_rn13	Other	NA
Bc_ctg1_1881	B2	1.16555131	Bcer	S	Bcer	FALSE	Bc_Ga0417192_01_rn13	Other	NA
Bc_ctg1_1882	B2	52.33325382	Bcer	NA	Bcer	FALSE	Bc_Ga0417192_01_rn13	Other	NA
Bc_ctg1_1883	B2	19.9309274	Bcer	S	Bcer	FALSE	Bc_Ga0417192_01_rn13	Other	NA
Bc_ctg1_1884	B2	5.711201419	Bcer	P	Bcer	FALSE	Bc_Ga0417192_01_rn13	Other	NA
Bc_ctg1_1885	B2	98.37253056	Bcer	H	Bcer	FALSE	Bc_Ga0417192_01_rn13	Ancillary biosynthesis	None
Bc_ctg1_1886	B2	1365.326805	Bcer	E	Bcer	FALSE	Bc_Ga0417192_01_rn13	Other	NA
Bc_ctg1_1887	B2	5.244980895	Bcer	S	Bcer	FALSE	Bc_Ga0417192_01_rn13	Other	NA
Bc_ctg1_1888	B2	18.64882096	Bcer	S	Bcer	FALSE	Bc_Ga0417192_01_rn13	Other	NA
Bc_ctg1_1889	B2	6.876752729	Bcer	F	Bcer	FALSE	Bc_Ga0417192_01_rn13	Other	NA
Bc_ctg1_189	B2	66.5529798	Bcer	M	Bcer	FALSE	NA	NA	NA
Bc_ctg1_1890	B2	23.77724672	Bcer	C	Bcer	FALSE	NA	NA	NA
Bc_ctg1_1891	B2	375.7737423	Bcer	C	Bcer	FALSE	NA	NA	NA
Bc_ctg1_1892	B2	70.86551965	Bcer	M	Bcer	FALSE	NA	NA	NA
Bc_ctg1_1893	B2	26.22490447	Bcer	K	Bcer	FALSE	NA	NA	NA
Bc_ctg1_1894	B2	66.90264519	Bcer	EGP	Bcer	FALSE	NA	NA	NA
Bc_ctg1_1895	B2	1.864882096	Bcer	K	Bcer	FALSE	NA	NA	NA
Bc_ctg1_1896	B2	3.380098799	Bcer	S	Bcer	FALSE	NA	NA	NA
Bc_ctg1_1897	B2	7.459528384	Bcer	S	Bcer	FALSE	NA	NA	NA
Bc_ctg1_1898	B2	0.815885917	Bcer	S	Bcer	FALSE	NA	NA	NA
Bc_ctg1_1899	B2	0	Bcer	NA	Bcer	FALSE	NA	NA	NA
Bc_ctg1_19	B2	5.244980895	Bcer	O	Bcer	FALSE	NA	NA	NA
Bc_ctg1_190	B2	83.22036353	Bcer	M	Bcer	FALSE	NA	NA	NA
Bc_ctg1_1900	B2	6.293977074	Bcer	NA	Bcer	FALSE	NA	NA	NA
Bc_ctg1_1901	B2	4.66220524	Bcer	J	Bcer	FALSE	NA	NA	NA
Bc_ctg1_1902	B2	135.3205071	Bcer	S	Bcer	FALSE	NA	NA	NA
Bc_ctg1_1903	B2	40.79429585	Bcer	E	Bcer	FALSE	NA	NA	NA
Bc_ctg1_1904	B2	9.32441048	Bcer	S	Bcer	FALSE	NA	NA	NA
Bc_ctg1_1905	B2	444.3081594	Bcer	C	Bcer	FALSE	NA	NA	NA
Bc_ctg1_1906	B2	9.091300218	Bcer	V	Bcer	FALSE	NA	NA	NA
Bc_ctg1_1907	B2	5.82775655	Bcer	S	Bcer	FALSE	NA	NA	NA
Bc_ctg1_1908	B2	15.50183242	Bcer	P	Bcer	FALSE	NA	NA	NA
Bc_ctg1_1909	B2	3.263543668	Bcer	NU	Bcer	FALSE	NA	NA	NA
Bc_ctg1_191	B2	1.515216703	Bcer	S	Bcer	FALSE	NA	NA	NA
Bc_ctg1_1910	B2	2.680768013	Bcer	S	Bcer	FALSE	NA	NA	NA
Bc_ctg1_1911	B2	2.33110262	Bcer	NA	Bcer	FALSE	NA	NA	NA
Bc_ctg1_1912	B2	1.048996179	Bcer	S	Bcer	FALSE	NA	NA	NA
Bc_ctg1_1913	B2	1.631771834	Bcer	J	Bcer	FALSE	NA	NA	NA
Bc_ctg1_1914	B2	2.564212882	Bcer	S	Bcer	FALSE	NA	NA	NA
Bc_ctg1_1915	B2	86.13424181	Bcer	O	Bcer	FALSE	NA	NA	NA
Bc_ctg1_1916	B2	17.01704913	Bcer	NA	Bcer	FALSE	NA	NA	NA
Bc_ctg1_1917	B2	10.48996179	Bcer	S	Bcer	FALSE	NA	NA	NA
Bc_ctg1_1918	B2	42.77573308	Bcer	I	Bcer	FALSE	NA	NA	NA
Bc_ctg1_1919	B2	3.263543668	Bcer	J	Bcer	FALSE	NA	NA	NA
Bc_ctg1_192	B2	47.55449345	Bcer	EGP	Bcer	FALSE	NA	NA	NA
Bc_ctg1_1920	B2	5.478091157	Bcer	G	Bcer	FALSE	NA	NA	NA
Bc_ctg1_1921	B2	5.478091157	Bcer	F	Bcer	FALSE	NA	NA	NA
Bc_ctg1_1922	B2	3.030433406	Bcer	J	Bcer	FALSE	NA	NA	NA
Bc_ctg1_1923	B2	3.962874454	Bcer	S	Bcer	FALSE	NA	NA	NA
Bc_ctg1_1924	B2	12.93761954	Bcer	S	Bcer	FALSE	NA	NA	NA
Bc_ctg1_1925	B2	3.613209061	Bcer	NA	Bcer	FALSE	NA	NA	NA
Bc_ctg1_1926	B2	110.4942642	Bcer	G	Bcer	FALSE	NA	NA	NA
Bc_ctg1_1927	B2	18.76537609	Bcer	K	Bcer	FALSE	NA	NA	NA

Bc_ctg1_1928	B2	77.39260698	Bcer	S	Bcer	FALSE	NA	NA	NA
Bc_ctg1_1929	B2	141.2648188	Bcer	G	Bcer	FALSE	NA	NA	NA
Bc_ctg1_193	B2	6.99330786	Bcer	NA	Bcer	FALSE	NA	NA	NA
Bc_ctg1_1930	B2	47.32138318	Bcer	S	Bcer	FALSE	NA	NA	NA
Bc_ctg1_1931	B2	1.631771834	Bcer	S	Bcer	FALSE	NA	NA	NA
Bc_ctg1_1932	B2	12.58795415	Bcer	S	Bcer	FALSE	NA	NA	NA
Bc_ctg1_1933	B2	0	Bcer	S	Bcer	FALSE	NA	NA	NA
Bc_ctg1_1934	B2	4.778760371	Bcer	K	Bcer	FALSE	NA	NA	NA
Bc_ctg1_1935	B2	55.59679749	Bcer	S	Bcer	FALSE	NA	NA	NA
Bc_ctg1_1936	B2	0	Bcer	NA	Bcer	FALSE	NA	NA	NA
Bc_ctg1_1937	B2	31.5864405	Bcer	G	Bcer	FALSE	NA	NA	NA
Bc_ctg1_1938	B2	16.43427347	Bcer	S	Bcer	FALSE	NA	NA	NA
Bc_ctg1_1939	B2	736.0456522	Bcer	H	Bcer	FALSE	NA	NA	NA
Bc_ctg1_194	B2	79.95681986	Bcer	I	Bcer	FALSE	NA	NA	NA
Bc_ctg1_1940	B2	0.116555131	Bcer	M	Bcer	FALSE	NA	NA	NA
Bc_ctg1_1941	B2	4.079429585	Bcer	S	Bcer	FALSE	NA	NA	NA
Bc_ctg1_1942	B2	9.207855349	Bcer	S	Bcer	FALSE	NA	NA	NA
Bc_ctg1_1943	B2	56.4126834	Bcer	F	Bcer	FALSE	NA	NA	NA
Bc_ctg1_1944	B2	1946.470688	Bcer	O	Bcer	FALSE	NA	NA	NA
Bc_ctg1_1945	B2	303.0433406	Bcer	C	Bcer	FALSE	NA	NA	NA
Bc_ctg1_1946	B2	1088.158703	Bcer	E	Bcer	FALSE	NA	NA	NA
Bc_ctg1_1947	B2	1105.991638	Bcer	O	Bcer	FALSE	NA	NA	NA
Bc_ctg1_1948	B2	572.985024	Bcer	O	Bcer	FALSE	NA	NA	NA
Bc_ctg1_1949	B2	14.56939137	Bcer	P	Bcer	FALSE	NA	NA	NA
Bc_ctg1_195	B2	14.45283624	Bcer	K	Bcer	FALSE	NA	NA	NA
Bc_ctg1_1950	B2	99.88774726	Bcer	P	Bcer	FALSE	NA	NA	NA
Bc_ctg1_1951	B2	13.75350546	Bcer	P	Bcer	FALSE	NA	NA	NA
Bc_ctg1_1952	B2	32.86854694	Bcer	P	Bcer	FALSE	NA	NA	NA
Bc_ctg1_1953	B2	0.932441048	Bcer	NA	Bcer	FALSE	NA	NA	NA
Bc_ctg1_1954	B2	48.25382423	Bcer	CO	Bcer	FALSE	NA	NA	NA
Bc_ctg1_1955	B2	4.895315502	Bcer	L	Bcer	FALSE	NA	NA	NA
Bc_ctg1_1956	B2	1417.193838	Bcer	E	Bcer	FALSE	NA	NA	NA
Bc_ctg1_1957	B2	289.7560557	Bcer	P	Bcer	FALSE	NA	NA	NA
Bc_ctg1_1958	B2	0.699330786	Bcer	NA	Bcer	FALSE	NA	NA	NA
Bc_ctg1_1959	B2	20.63025819	Bcer	S	Bcer	FALSE	NA	NA	NA
Bc_ctg1_196	B2	39.04596888	Bcer	EGP	Bcer	FALSE	NA	NA	NA
Bc_ctg1_1960	B2	0.233110262	Bcer	NA	Bcer	FALSE	NA	NA	NA
Bc_ctg1_1961	B2	30.77055458	Bcer	S	Bcer	FALSE	NA	NA	NA
Bc_ctg1_1962	B2	15.61838755	Bcer	NA	Bcer	FALSE	NA	NA	NA
Bc_ctg1_1963	B2	13.28728493	Bcer	I	Bcer	FALSE	NA	NA	NA
Bc_ctg1_1964	B2	4691.693688	Bcer	S	Bcer	FALSE	NA	NA	NA
Bc_ctg1_1965	B2	6608.675928	Bcer	C	Bcer	FALSE	NA	NA	NA
Bc_ctg1_1966	B2	0	Bcer	M	Bcer	FALSE	NA	NA	NA
Bc_ctg1_1967	B2	0	Bcer	NA	Bcer	FALSE	NA	NA	NA
Bc_ctg1_1968	B2	8.391969432	Bcer	S	Bcer	FALSE	NA	NA	NA
Bc_ctg1_1969	B2	0.116555131	Bcer	NA	Bcer	FALSE	NA	NA	NA
Bc_ctg1_197	B2	179.3783466	Bcer	V	Bcer	FALSE	NA	NA	NA
Bc_ctg1_1970	B2	6.643642467	Bcer	K	Bcer	FALSE	NA	NA	NA
Bc_ctg1_1971	B2	8.974745087	Bcer	S	Bcer	FALSE	NA	NA	NA
Bc_ctg1_1972	B2	1220.215666	Bcer	I	Bcer	FALSE	NA	NA	NA
Bc_ctg1_1973	B2	628.9314869	Bcer	I	Bcer	FALSE	NA	NA	NA
Bc_ctg1_1974	B2	1369.98901	Bcer	I	Bcer	FALSE	NA	NA	NA
Bc_ctg1_1975	B2	0	Bcer	S	Bcer	FALSE	NA	NA	NA
Bc_ctg1_1976	B2	28.78911736	Bcer	M	Bcer	FALSE	NA	NA	NA
Bc_ctg1_1977	B2	1910.688262	Bcer	E	Bcer	FALSE	NA	NA	NA
Bc_ctg1_1978	B2	1.16555131	Bcer	S	Bcer	FALSE	NA	NA	NA
Bc_ctg1_1979	B2	69.69996834	Bcer	S	Bcer	FALSE	NA	NA	NA
Bc_ctg1_1980	B2	182.40878	Bcer	NT	Bcer	FALSE	NA	NA	NA
Bc_ctg1_1981	B2	42.89228821	Bcer	K	Bcer	FALSE	NA	NA	NA
Bc_ctg1_1983	B2	7.692638646	Bcer	EGP	Bcer	FALSE	NA	NA	NA
Bc_ctg1_1984	B2	293.1361545	Bcer	E	Bcer	FALSE	NA	NA	NA
Bc_ctg1_1985	B2	9.907186135	Bcer	NA	Bcer	FALSE	NA	NA	NA

Bc_ctg1_1986	B2	2.33110262	Bcer	NA	Bcer	FALSE	NA	NA	NA
Bc_ctg1_1987	B2	263.6477063	Bcer	U	Bcer	FALSE	NA	NA	NA
Bc_ctg1_1988	B2	38.23008297	Bcer	NA	Bcer	FALSE	NA	NA	NA
Bc_ctg1_1989	B2	111.8929258	Bcer	S	Bcer	FALSE	NA	NA	NA
Bc_ctg1_199	B2	25.87523908	Bcer	V	Bcer	FALSE	NA	NA	NA
Bc_ctg1_1990	B2	18.99848635	Bcer	J	Bcer	FALSE	NA	NA	NA
Bc_ctg1_1991	B2	61.77421943	Bcer	S	Bcer	FALSE	NA	NA	NA
Bc_ctg1_1992	B2	1.864882096	Bcer	NA	Bcer	FALSE	NA	NA	NA
Bc_ctg1_1993	B2	32.05266102	Bcer	S	Bcer	FALSE	NA	NA	NA
Bc_ctg1_1994	B2	15.96805295	Bcer	S	Bcer	FALSE	NA	NA	NA
Bc_ctg1_1995	B2	0	Bcer	K	Bcer	FALSE	NA	NA	NA
Bc_ctg1_1996	B2	37.53075218	Bcer	S	Bcer	FALSE	NA	NA	NA
Bc_ctg1_1997	B2	0.932441048	Bcer	S	Bcer	FALSE	NA	NA	NA
Bc_ctg1_1998	B2	2.214547489	Bcer	O	Bcer	FALSE	NA	NA	NA
Bc_ctg1_1999	B2	141.2648188	Bcer	T	Bcer	FALSE	NA	NA	NA
Bc_ctg1_2	B2	3.729764192	Bcer	S	Bcer	FALSE	NA	NA	NA
Bc_ctg1_20	B2	6.060866812	Bcer	S	Bcer	FALSE	NA	NA	NA
Bc_ctg1_200	B2	0	Bcer	NA	Bcer	FALSE	NA	NA	NA
Bc_ctg1_2000	B2	414.586601	Bcer	T	Bcer	FALSE	NA	NA	NA
Bc_ctg1_2001	B2	11.77206823	Bcer	EG	Bcer	FALSE	NA	NA	NA
Bc_ctg1_2002	B2	5.478091157	Bcer	NA	Bcer	FALSE	NA	NA	NA
Bc_ctg1_2003	B2	5.594646288	Bcer	J	Bcer	FALSE	NA	NA	NA
Bc_ctg1_2004	B2	3.030433406	Bcer	H	Bcer	FALSE	NA	NA	NA
Bc_ctg1_2005	B2	1441.32075	Bcer	NT	Bcer	FALSE	NA	NA	NA
Bc_ctg1_2006	B2	202.3397074	Bcer	NT	Bcer	FALSE	NA	NA	NA
Bc_ctg1_2007	B2	15.96805295	Bcer	S	Bcer	FALSE	NA	NA	NA
Bc_ctg1_2008	B2	10.60651692	Bcer	E	Bcer	FALSE	NA	NA	NA
Bc_ctg1_2009	B2	54.66435644	Bcer	S	Bcer	FALSE	NA	NA	NA
Bc_ctg1_201	B2	0.349665393	Bcer	I	Bcer	FALSE	NA	NA	NA
Bc_ctg1_2010	B2	32.16921616	Bcer	K	Bcer	FALSE	NA	NA	NA
Bc_ctg1_2011	B2	0	Bcer	S	Bcer	FALSE	NA	NA	NA
Bc_ctg1_2012	B2	0.116555131	Bcer	S	Bcer	FALSE	NA	NA	NA
Bc_ctg1_2013	B2	3.146988537	Bcer	P	Bcer	FALSE	NA	NA	NA
Bc_ctg1_2014	B2	3.146988537	Bcer	P	Bcer	FALSE	NA	NA	NA
Bc_ctg1_2015	B2	79.7237096	Bcer	E	Bcer	FALSE	NA	NA	NA
Bc_ctg1_2016	B2	1742.382653	Bcer	P	Bcer	FALSE	NA	NA	NA
Bc_ctg1_2017	B2	4.079429585	Bcer	NA	Bcer	FALSE	NA	NA	NA
Bc_ctg1_2018	B2	1.16555131	Bcer	NA	Bcer	FALSE	NA	NA	NA
Bc_ctg1_2019	B2	5.594646288	Bcer	NA	Bcer	FALSE	NA	NA	NA
Bc_ctg1_202	B2	1.398661572	Bcer	NA	Bcer	FALSE	NA	NA	NA
Bc_ctg1_2020	B2	92.31166375	Bcer	S	Bcer	FALSE	NA	NA	NA
Bc_ctg1_2021	B2	70.86551965	Bcer	S	Bcer	FALSE	NA	NA	NA
Bc_ctg1_2022	B2	211.5475628	Bcer	P	Bcer	FALSE	NA	NA	NA
Bc_ctg1_2023	B2	9.440965611	Bcer	P	Bcer	FALSE	NA	NA	NA
Bc_ctg1_2024	B2	2.797323144	Bcer	P	Bcer	FALSE	NA	NA	NA
Bc_ctg1_2025	B2	743.1555152	Bcer	F	Bcer	FALSE	NA	NA	NA
Bc_ctg1_2026	B2	4.895315502	Bcer	F	Bcer	FALSE	NA	NA	NA
Bc_ctg1_2027	B2	184.8564378	Bcer	E	Bcer	FALSE	NA	NA	NA
Bc_ctg1_2028	B2	174.7161414	Bcer	E	Bcer	FALSE	NA	NA	NA
Bc_ctg1_2029	B2	6.060866812	Bcer	S	Bcer	FALSE	NA	NA	NA
Bc_ctg1_203	B2	1.748326965	Bcer	S	Bcer	FALSE	NA	NA	NA
Bc_ctg1_2030	B2	17.25015939	Bcer	G	Bcer	FALSE	NA	NA	NA
Bc_ctg1_2031	B2	0	Bcer	M	Bcer	FALSE	NA	NA	NA
Bc_ctg1_2032	B2	11.6555131	Bcer	S	Bcer	FALSE	NA	NA	NA
Bc_ctg1_2033	B2	8.741634825	Bcer	EGP	Bcer	FALSE	NA	NA	NA
Bc_ctg1_2034	B2	7.692638646	Bcer	K	Bcer	FALSE	NA	NA	NA
Bc_ctg1_2035	B2	267.7271359	Bcer	S	Bcer	FALSE	NA	NA	NA
Bc_ctg1_2036	B2	964.1440436	Bcer	C	Bcer	FALSE	NA	NA	NA
Bc_ctg1_2037	B2	65.0377631	Bcer	J	Bcer	FALSE	NA	NA	NA
Bc_ctg1_2038	B2	153.2699973	Bcer	M	Bcer	FALSE	NA	NA	NA
Bc_ctg1_2039	B2	131.8238532	Bcer	S	Bcer	FALSE	NA	NA	NA
Bc_ctg1_204	B2	1.16555131	Bcer	S	Bcer	FALSE	NA	NA	NA

Bc_ctg1_2040	B2	72.14762609	Bcer	NT	Bcer	FALSE	NA	NA	NA
Bc_ctg1_2041	B2	32.28577129	Bcer	L	Bcer	FALSE	NA	NA	NA
Bc_ctg1_2042	B2	0.932441048	Bcer	D	Bcer	FALSE	NA	NA	NA
Bc_ctg1_2043	B2	0.582775655	Bcer	D	Bcer	FALSE	NA	NA	NA
Bc_ctg1_2044	B2	1.981437227	Bcer	NA	Bcer	FALSE	NA	NA	NA
Bc_ctg1_2045	B2	1.631771834	Bcer	NA	Bcer	FALSE	NA	NA	NA
Bc_ctg1_2046	B2	1.631771834	Bcer	EGP	Bcer	FALSE	NA	NA	NA
Bc_ctg1_2047	B2	0.116555131	Bcer	S	Bcer	FALSE	NA	NA	NA
Bc_ctg1_2048	B2	3.613209061	Bcer	J	Bcer	FALSE	NA	NA	NA
Bc_ctg1_2049	B2	13.1707298	Bcer	S	Bcer	FALSE	NA	NA	NA
Bc_ctg1_205	B2	2.564212882	Bcer	NA	Bcer	FALSE	NA	NA	NA
Bc_ctg1_2050	B2	1.048996179	Bcer	S	Bcer	FALSE	NA	NA	NA
Bc_ctg1_2051	B2	0	Bcer	Q	Bcer	TRUE	NA	NA	NA
Bc_ctg1_2052	B2	4.545650109	Bcer	K	Bcer	FALSE	NA	NA	NA
Bc_ctg1_2053	B2	0	Bcer	S	Bcer	FALSE	NA	NA	NA
Bc_ctg1_2054	B2	8.042304039	Bcer	K	Bcer	FALSE	NA	NA	NA
Bc_ctg1_2055	B2	1.631771834	Bcer	P	Bcer	FALSE	NA	NA	NA
Bc_ctg1_2056	B2	1.515216703	Bcer	P	Bcer	FALSE	NA	NA	NA
Bc_ctg1_2057	B2	1.16555131	Bcer	F	Bcer	FALSE	NA	NA	NA
Bc_ctg1_2058	B2	1628.04207	Bcer	M	Bcer	FALSE	NA	NA	NA
Bc_ctg1_2059	B2	27.04079039	Bcer	P	Bcer	FALSE	NA	NA	NA
Bc_ctg1_206	B2	0	Bcer	NA	Bcer	FALSE	NA	NA	NA
Bc_ctg1_2060	B2	25.64212882	Bcer	P	Bcer	FALSE	NA	NA	NA
Bc_ctg1_2061	B2	28.08978657	Bcer	P	Bcer	FALSE	NA	NA	NA
Bc_ctg1_2062	B2	42.30951255	Bcer	P	Bcer	FALSE	NA	NA	NA
Bc_ctg1_2063	B2	3.962874454	Bcer	T	Bcer	FALSE	NA	NA	NA
Bc_ctg1_2064	B2	14.56939137	Bcer	S	Bcer	FALSE	NA	NA	NA
Bc_ctg1_2065	B2	27.50701092	Bcer	O	Bcer	FALSE	NA	NA	NA
Bc_ctg1_2066	B2	490.6971015	Bcer	K	Bcer	FALSE	NA	NA	NA
Bc_ctg1_2067	B2	137.0688341	Bcer	S	Bcer	FALSE	NA	NA	NA
Bc_ctg1_2068	B2	15.50183242	Bcer	U	Bcer	FALSE	NA	NA	NA
Bc_ctg1_2069	B2	72.61384661	Bcer	M	Bcer	FALSE	NA	NA	NA
Bc_ctg1_207	B2	64.22187718	Bcer	S	Bcer	FALSE	NA	NA	NA
Bc_ctg1_2070	B2	34.84998417	Bcer	S	Bcer	FALSE	NA	NA	NA
Bc_ctg1_2071	B2	0	Bcer	F	Bcer	FALSE	NA	NA	NA
Bc_ctg1_2072	B2	0.116555131	Bcer	F	Bcer	FALSE	NA	NA	NA
Bc_ctg1_2073	B2	9373.130525	Bcer	G	Bcer	FALSE	NA	NA	NA
Bc_ctg1_2074	B2	10261.16407	Bcer	G	Bcer	FALSE	NA	NA	NA
Bc_ctg1_2075	B2	4373.49818	Bcer	G	Bcer	FALSE	NA	NA	NA
Bc_ctg1_2076	B2	6648.537782	Bcer	F	Bcer	FALSE	NA	NA	NA
Bc_ctg1_2077	B2	12036.76493	Bcer	G	Bcer	FALSE	NA	NA	NA
Bc_ctg1_2078	B2	4467.325061	Bcer	K	Bcer	FALSE	NA	NA	NA
Bc_ctg1_2079	B2	40.79429585	Bcer	O	Bcer	FALSE	NA	NA	NA
Bc_ctg1_208	B2	8.974745087	Bcer	S	Bcer	FALSE	NA	NA	NA
Bc_ctg1_2080	B2	443.7253837	Bcer	K	Bcer	FALSE	NA	NA	NA
Bc_ctg1_2081	B2	0.116555131	Bcer	NA	Bcer	FALSE	NA	NA	NA
Bc_ctg1_2082	B2	3.729764192	Bcer	CP	Bcer	FALSE	NA	NA	NA
Bc_ctg1_2083	B2	2.447657751	Bcer	S	Bcer	FALSE	NA	NA	NA
Bc_ctg1_2084	B2	7.925748908	Bcer	T	Bcer	FALSE	NA	NA	NA
Bc_ctg1_2085	B2	27.04079039	Bcer	K	Bcer	FALSE	NA	NA	NA
Bc_ctg1_2087	B2	0	Bcer	NA	Bcer	FALSE	NA	NA	NA
Bc_ctg1_2088	B2	0	Bcer	S	Bcer	FALSE	NA	NA	NA
Bc_ctg1_2089	B2	0.116555131	Bcer	I	Bcer	FALSE	NA	NA	NA
Bc_ctg1_209	B2	20.39714792	Bcer	P	Bcer	FALSE	NA	NA	NA
Bc_ctg1_2090	B2	0	Bcer	S	Bcer	FALSE	NA	NA	NA
Bc_ctg1_2091	B2	0	Bcer	S	Bcer	FALSE	NA	NA	NA
Bc_ctg1_2092	B2	0	Bcer	S	Bcer	FALSE	NA	NA	NA
Bc_ctg1_2093	B2	756.2096899	Bcer	OU	Bcer	FALSE	NA	NA	NA
Bc_ctg1_2094	B2	11.88862336	Bcer	G	Bcer	FALSE	NA	NA	NA
Bc_ctg1_2095	B2	171.9188182	Bcer	K	Bcer	FALSE	NA	NA	NA
Bc_ctg1_2096	B2	339.6416517	Bcer	S	Bcer	FALSE	NA	NA	NA
Bc_ctg1_2097	B2	267.2609154	Bcer	S	Bcer	FALSE	NA	NA	NA

Bc_ctg1_2098	B2	77.74227238	Bcer	F	Bcer	FALSE	NA	NA	NA
Bc_ctg1_2099	B2	5.594646288	Bcer	S	Bcer	FALSE	NA	NA	NA
Bc_ctg1_21	B2	0.582775655	Bcer	S	Bcer	FALSE	NA	NA	NA
Bc_ctg1_210	B2	8.15885917	Bcer	P	Bcer	FALSE	NA	NA	NA
Bc_ctg1_2100	B2	321.1093859	Bcer	C	Bcer	FALSE	NA	NA	NA
Bc_ctg1_2101	B2	173.6671452	Bcer	S	Bcer	FALSE	NA	NA	NA
Bc_ctg1_2102	B2	20.04748253	Bcer	S	Bcer	FALSE	NA	NA	NA
Bc_ctg1_2103	B2	21.91236463	Bcer	S	Bcer	FALSE	NA	NA	NA
Bc_ctg1_2104	B2	412.2554983	Bcer	M	Bcer	FALSE	NA	NA	NA
Bc_ctg1_2105	B2	354.6772636	Bcer	F	Bcer	FALSE	NA	NA	NA
Bc_ctg1_2106	B2	38.46319323	Bcer	S	Bcer	FALSE	NA	NA	NA
Bc_ctg1_2107	B2	470.8827292	Bcer	L	Bcer	FALSE	NA	NA	NA
Bc_ctg1_2108	B2	205.6032511	Bcer	L	Bcer	FALSE	NA	NA	NA
Bc_ctg1_2109	B2	3.729764192	Bcer	S	Bcer	FALSE	NA	NA	NA
Bc_ctg1_211	B2	12.93761954	Bcer	I	Bcer	FALSE	NA	NA	NA
Bc_ctg1_2110	B2	9.32441048	Bcer	NA	Bcer	FALSE	NA	NA	NA
Bc_ctg1_2111	B2	5.361536026	Bcer	K	Bcer	FALSE	NA	NA	NA
Bc_ctg1_2112	B2	5.944311681	Bcer	NA	Bcer	FALSE	NA	NA	NA
Bc_ctg1_2113	B2	8.15885917	Bcer	NA	Bcer	FALSE	NA	NA	NA
Bc_ctg1_2114	B2	4.545650109	Bcer	NA	Bcer	FALSE	NA	NA	NA
Bc_ctg1_2115	B2	0.233110262	Bcer	K	Bcer	FALSE	NA	NA	NA
Bc_ctg1_2116	B2	293.9520404	Bcer	S	Bcer	FALSE	NA	NA	NA
Bc_ctg1_2117	B2	7.459528384	Bcer	K	Bcer	FALSE	NA	NA	NA
Bc_ctg1_2118	B2	7.692638646	Bcer	K	Bcer	FALSE	NA	NA	NA
Bc_ctg1_2119	B2	8.858189956	Bcer	S	Bcer	FALSE	NA	NA	NA
Bc_ctg1_212	B2	357.4745868	Bcer	I	Bcer	FALSE	NA	NA	NA
Bc_ctg1_2120	B2	6.177421943	Bcer	L	Bcer	FALSE	NA	NA	NA
Bc_ctg1_2121	B2	0.349665393	Bcer	S	Bcer	FALSE	NA	NA	NA
Bc_ctg1_2122	B2	12.93761954	Bcer	V	Bcer	FALSE	NA	NA	NA
Bc_ctg1_2123	B2	148.9574574	Bcer	O	Bcer	FALSE	NA	NA	NA
Bc_ctg1_2124	B2	137.0688341	Bcer	M	Bcer	FALSE	NA	NA	NA
Bc_ctg1_2125	B2	301.0619034	Bcer	D	Bcer	FALSE	NA	NA	NA
Bc_ctg1_2126	B2	77.85882751	Bcer	D	Bcer	FALSE	NA	NA	NA
Bc_ctg1_2127	B2	63.172881	Bcer	C	Bcer	FALSE	NA	NA	NA
Bc_ctg1_2128	B2	257.9365049	Bcer	J	Bcer	FALSE	NA	NA	NA
Bc_ctg1_2129	B2	1524.890779	Bcer	U	Bcer	FALSE	NA	NA	NA
Bc_ctg1_213	B2	2.797323144	Bcer	H	Bcer	FALSE	NA	NA	NA
Bc_ctg1_2130	B2	1.748326965	Bcer	NA	Bcer	FALSE	NA	NA	NA
Bc_ctg1_2131	B2	2.33110262	Bcer	V	Bcer	FALSE	NA	NA	NA
Bc_ctg1_2132	B2	0.349665393	Bcer	NA	Bcer	FALSE	NA	NA	NA
Bc_ctg1_2133	B2	1.16555131	Bcer	NA	Bcer	FALSE	NA	NA	NA
Bc_ctg1_2134	B2	1.981437227	Bcer	NA	Bcer	FALSE	NA	NA	NA
Bc_ctg1_2135	B2	0.932441048	Bcer	KT	Bcer	FALSE	Bc_Ga0417192_01_rn14	Other	NA
Bc_ctg1_2136	B2	2.214547489	Bcer	NA	Bcer	FALSE	Bc_Ga0417192_01_rn14	Other	NA
Bc_ctg1_2137	B2	4498.794946	Bcer	J	Bcer	FALSE	Bc_Ga0417192_01_rn14	Other	NA
Bc_ctg1_2138	B2	0	Bcer	K	Bcer	FALSE	Bc_Ga0417192_01_rn14	Regulation	SMCOG1255
Bc_ctg1_2139	B2	0.932441048	Bcer	S	Bcer	FALSE	Bc_Ga0417192_01_rn14	Other	NA
Bc_ctg1_214	B2	0	Bcer	S	Bcer	FALSE	NA	NA	NA
Bc_ctg1_2140	B2	3.030433406	Bcer	L	Bcer	FALSE	Bc_Ga0417192_01_rn14	Other	NA
Bc_ctg1_2141	B2	894.3275201	Bcer	M	Bcer	FALSE	Bc_Ga0417192_01_rn14	Other	NA
Bc_ctg1_2142	B2	206.5356921	Bcer	K	Bcer	FALSE	Bc_Ga0417192_01_rn14	Regulation	SMCOG1167
Bc_ctg1_2143	B2	38.81285862	Bcer	S	Bcer	FALSE	Bc_Ga0417192_01_rn14	Other	NA
Bc_ctg1_2144	B2	8.974745087	Bcer	S	Bcer	FALSE	Bc_Ga0417192_01_rn14	Other	NA
Bc_ctg1_2145	B2	78.67471342	Bcer	K	Bcer	FALSE	Bc_Ga0417192_01_rn14	Other	NA
Bc_ctg1_2146	B2	808.0767232	Bcer	M	Bcer	FALSE	Bc_Ga0417192_01_rn14	Core biosynthesis	None
Bc_ctg1_2147	B2	4.895315502	Bcer	M	Bcer	FALSE	Bc_Ga0417192_01_rn14	Ancillary biosynthesis	SMCOG1152
Bc_ctg1_2148	B2	514.8240136	Bcer	M	Bcer	FALSE	Bc_Ga0417192_01_rn14	Core biosynthesis	None
Bc_ctg1_2149	B2	4.895315502	Bcer	G	Bcer	FALSE	Bc_Ga0417192_01_rn14	Ancillary biosynthesis	SMCOG1235
Bc_ctg1_215	B2	22.72825054	Bcer	L	Bcer	FALSE	NA	NA	NA
Bc_ctg1_2150	B2	96.74075873	Bcer	K	Bcer	FALSE	Bc_Ga0417192_01_rn14	Regulation	SMCOG1112
Bc_ctg1_2151	B2	0	Bcer	G	Bcer	FALSE	Bc_Ga0417192_01_rn14	Other	NA
Bc_ctg1_2152	B2	4.66220524	Bcer	P	Bcer	FALSE	Bc_Ga0417192_01_rn14	Other	NA

Bc_ctg1_2153	B2	11.6555131	Bcer	G	Bcer	FALSE	Bc_Ga0417192_01_rn14	Other	NA
Bc_ctg1_2154	B2	221.4547489	Bcer	G	Bcer	FALSE	Bc_Ga0417192_01_rn14	Other	NA
Bc_ctg1_2155	B2	17.48326965	Bcer	G	Bcer	FALSE	Bc_Ga0417192_01_rn14	Other	NA
Bc_ctg1_2156	B2	350.4812789	Bcer	G	Bcer	FALSE	Bc_Ga0417192_01_rn14	Other	NA
Bc_ctg1_2157	B2	56.7623488	Bcer	G	Bcer	FALSE	Bc_Ga0417192_01_rn14	Other	NA
Bc_ctg1_2158	B2	0.116555131	Bcer	E	Bcer	FALSE	NA	NA	NA
Bc_ctg1_2159	B2	0	Bcer	E	Bcer	FALSE	NA	NA	NA
Bc_ctg1_216	B2	1.748326965	Bcer	NA	Bcer	FALSE	NA	NA	NA
Bc_ctg1_2160	B2	0.582775655	Bcer	G	Bcer	FALSE	NA	NA	NA
Bc_ctg1_2161	B2	7.925748908	Bcer	G	Bcer	FALSE	NA	NA	NA
Bc_ctg1_2162	B2	0	Bcer	G	Bcer	FALSE	NA	NA	NA
Bc_ctg1_2163	B2	8.042304039	Bcer	C	Bcer	FALSE	NA	NA	NA
Bc_ctg1_2164	B2	58.51067576	Bcer	I	Bcer	FALSE	NA	NA	NA
Bc_ctg1_2165	B2	30.77055458	Bcer	E	Bcer	FALSE	NA	NA	NA
Bc_ctg1_2166	B2	3.263543668	Bcer	S	Bcer	FALSE	NA	NA	NA
Bc_ctg1_2167	B2	20.16403766	Bcer	K	Bcer	FALSE	NA	NA	NA
Bc_ctg1_2168	B2	10.72307205	Bcer	G	Bcer	FALSE	NA	NA	NA
Bc_ctg1_2169	B2	0	Bcer	NA	Bcer	FALSE	NA	NA	NA
Bc_ctg1_217	B2	96.85731386	Bcer	I	Bcer	FALSE	NA	NA	NA
Bc_ctg1_2170	B2	1.16555131	Bcer	C	Bcer	FALSE	NA	NA	NA
Bc_ctg1_2171	B2	2.214547489	Bcer	C	Bcer	FALSE	NA	NA	NA
Bc_ctg1_2172	B2	0.233110262	Bcer	P	Bcer	FALSE	NA	NA	NA
Bc_ctg1_2173	B2	120.2848952	Bcer	H	Bcer	FALSE	NA	NA	NA
Bc_ctg1_2174	B2	21.56269923	Bcer	NA	Bcer	FALSE	NA	NA	NA
Bc_ctg1_2175	B2	0.116555131	Bcer	L	Bcer	FALSE	NA	NA	NA
Bc_ctg1_2177	B2	111.5432604	Bcer	C	Bcer	FALSE	NA	NA	NA
Bc_ctg1_2178	B2	11.6555131	Bcer	S	Bcer	FALSE	NA	NA	NA
Bc_ctg1_2179	B2	52.79947434	Bcer	S	Bcer	FALSE	NA	NA	NA
Bc_ctg1_218	B2	0.116555131	Bcer	NA	Bcer	FALSE	NA	NA	NA
Bc_ctg1_2180	B2	50.70148198	Bcer	S	Bcer	FALSE	NA	NA	NA
Bc_ctg1_2181	B2	593.6152822	Bcer	M	Bcer	FALSE	NA	NA	NA
Bc_ctg1_2182	B2	38.11352784	Bcer	E	Bcer	FALSE	NA	NA	NA
Bc_ctg1_2184	B2	3683.14214	Bcer	MT	Bcer	FALSE	NA	NA	NA
Bc_ctg1_2185	B2	16.20116321	Bcer	F	Bcer	FALSE	NA	NA	NA
Bc_ctg1_2186	B2	24.24346725	Bcer	T	Bcer	FALSE	NA	NA	NA
Bc_ctg1_2187	B2	54.54780131	Bcer	M	Bcer	FALSE	NA	NA	NA
Bc_ctg1_2188	B2	0.233110262	Bcer	NA	Bcer	FALSE	NA	NA	NA
Bc_ctg1_2189	B2	38.23008297	Bcer	M	Bcer	FALSE	NA	NA	NA
Bc_ctg1_219	B2	25.1759083	Bcer	S	Bcer	FALSE	NA	NA	NA
Bc_ctg1_2190	B2	160.0301949	Bcer	T	Bcer	FALSE	NA	NA	NA
Bc_ctg1_2191	B2	36.24864574	Bcer	S	Bcer	FALSE	NA	NA	NA
Bc_ctg1_2192	B2	7.925748908	Bcer	S	Bcer	FALSE	NA	NA	NA
Bc_ctg1_2193	B2	4.079429585	Bcer	NA	Bcer	FALSE	NA	NA	NA
Bc_ctg1_2194	B2	0.815885917	Bcer	S	Bcer	FALSE	NA	NA	NA
Bc_ctg1_2195	B2	89.63089574	Bcer	S	Bcer	FALSE	NA	NA	NA
Bc_ctg1_2196	B2	245.6982161	Bcer	L	Bcer	FALSE	NA	NA	NA
Bc_ctg1_2197	B2	29.83811354	Bcer	S	Bcer	FALSE	NA	NA	NA
Bc_ctg1_2198	B2	0	Bcer	S	Bcer	FALSE	NA	NA	NA
Bc_ctg1_2199	B2	0	Bcer	S	Bcer	FALSE	NA	NA	NA
Bc_ctg1_22	B2	2.913878275	Bcer	S	Bcer	FALSE	NA	NA	NA
Bc_ctg1_220	B2	2.447657751	Bcer	J	Bcer	FALSE	NA	NA	NA
Bc_ctg1_2200	B2	1.981437227	Bcer	S	Bcer	FALSE	NA	NA	NA
Bc_ctg1_2201	B2	1.981437227	Bcer	K	Bcer	FALSE	NA	NA	NA
Bc_ctg1_2202	B2	0.582775655	Bcer	K	Bcer	FALSE	NA	NA	NA
Bc_ctg1_2203	B2	72.73040174	Bcer	S	Bcer	FALSE	NA	NA	NA
Bc_ctg1_2204	B2	182.40878	Bcer	V	Bcer	FALSE	Bc_Ga0417192_01_rn15	Other	NA
Bc_ctg1_2205	B2	56.17957314	Bcer	V	Bcer	FALSE	Bc_Ga0417192_01_rn15	Transport	SMCOG1000
Bc_ctg1_2206	B2	66.78609006	Bcer	M	Bcer	FALSE	Bc_Ga0417192_01_rn15	Transport	SMCOG1029
Bc_ctg1_2207	B2	0.582775655	Bcer	K	Bcer	FALSE	Bc_Ga0417192_01_rn15	Other	NA
Bc_ctg1_2208	B2	6.410532205	Bcer	S	Bcer	FALSE	Bc_Ga0417192_01_rn15	Other	NA
Bc_ctg1_2209	B2	15.96805295	Bcer	G	Bcer	FALSE	Bc_Ga0417192_01_rn15	Transport	SMCOG1106
Bc_ctg1_221	B2	0.466220524	Bcer	NA	Bcer	FALSE	NA	NA	NA

Bc_ctg1_2210	B2	18.88193122	Bcer	P	Bcer	FALSE	Bc_Ga0417192_01_rn15	Other	NA
Bc_ctg1_2211	B2	23.66069159	Bcer	S	Bcer	FALSE	Bc_Ga0417192_01_rn15	Other	NA
Bc_ctg1_2212	B2	33.91754312	Bcer	T	Bcer	FALSE	Bc_Ga0417192_01_rn15	Regulation	SMCOG1003
Bc_ctg1_2213	B2	7.576083515	Bcer	K	Bcer	FALSE	Bc_Ga0417192_01_rn15	Regulation	SMCOG1008
Bc_ctg1_2214	B2	33.33476747	Bcer	M	Bcer	FALSE	Bc_Ga0417192_01_rn15	Core biosynthesis	SMCOG1010
Bc_ctg1_2215	B2	56.4126834	Bcer	E	Bcer	FALSE	Bc_Ga0417192_01_rn15	Other	NA
Bc_ctg1_2216	B2	232.1778209	Bcer	K	Bcer	FALSE	Bc_Ga0417192_01_rn15	Other	NA
Bc_ctg1_2217	B2	223.4361861	Bcer	M	Bcer	FALSE	Bc_Ga0417192_01_rn15	Core biosynthesis	SMCOG1010
Bc_ctg1_2218	B2	257.120619	Bcer	M	Bcer	FALSE	Bc_Ga0417192_01_rn15	Ancillary biosynthesis	SMCOG1152
Bc_ctg1_2219	B2	144.2952522	Bcer	S	Bcer	FALSE	Bc_Ga0417192_01_rn15	Core biosynthesis	None
Bc_ctg1_222	B2	0.466220524	Bcer	K	Bcer	FALSE	NA	NA	NA
Bc_ctg1_2220	B2	183.8074416	Bcer	M	Bcer	FALSE	Bc_Ga0417192_01_rn15	Other	NA
Bc_ctg1_2221	B2	74.94494923	Bcer	M	Bcer	FALSE	Bc_Ga0417192_01_rn15	Other	NA
Bc_ctg1_2222	B2	55.71335262	Bcer	M	Bcer	FALSE	Bc_Ga0417192_01_rn15	Core biosynthesis	None
Bc_ctg1_2223	B2	71.4482953	Bcer	M	Bcer	FALSE	Bc_Ga0417192_01_rn15	Core biosynthesis	SMCOG1045
Bc_ctg1_2224	B2	66.5529798	Bcer	M	Bcer	FALSE	Bc_Ga0417192_01_rn15	Core biosynthesis	SMCOG1045
Bc_ctg1_2225	B2	13.5203952	Bcer	S	Bcer	FALSE	Bc_Ga0417192_01_rn15	Other	NA
Bc_ctg1_2226	B2	16.31771834	Bcer	M	Bcer	FALSE	Bc_Ga0417192_01_rn15	Core biosynthesis	None
Bc_ctg1_2227	B2	70.04963373	Bcer	M	Bcer	FALSE	Bc_Ga0417192_01_rn15	Core biosynthesis	SMCOG1146
Bc_ctg1_2228	B2	50.11870633	Bcer	E	Bcer	FALSE	Bc_Ga0417192_01_rn15	Core biosynthesis	SMCOG1056
Bc_ctg1_2229	B2	33.4513226	Bcer	GM	Bcer	FALSE	Bc_Ga0417192_01_rn15	Core biosynthesis	SMCOG1010
Bc_ctg1_223	B2	1.515216703	Bcer	C	Bcer	FALSE	NA	NA	NA
Bc_ctg1_2230	B2	9.32441048	Bcer	M	Bcer	FALSE	Bc_Ga0417192_01_rn15	Core biosynthesis	SMCOG1064
Bc_ctg1_2231	B2	7.459528384	Bcer	GM	Bcer	FALSE	Bc_Ga0417192_01_rn15	Other	NA
Bc_ctg1_2232	B2	113.2915873	Bcer	D	Bcer	FALSE	Bc_Ga0417192_01_rn15	Ancillary biosynthesis	SMCOG1216
Bc_ctg1_2233	B2	52.10014356	Bcer	M	Bcer	FALSE	Bc_Ga0417192_01_rn15	Ancillary biosynthesis	SMCOG1216
Bc_ctg1_2234	B2	62.00732969	Bcer	D	Bcer	FALSE	Bc_Ga0417192_01_rn15	Ancillary biosynthesis	SMCOG1216
Bc_ctg1_2235	B2	146.9760202	Bcer	I	Bcer	FALSE	Bc_Ga0417192_01_rn15	Ancillary biosynthesis	SMCOG1270
Bc_ctg1_2236	B2	254.0901856	Bcer	D	Bcer	FALSE	Bc_Ga0417192_01_rn15	Other	NA
Bc_ctg1_2237	B2	0.699330786	Bcer	K	Bcer	FALSE	Bc_Ga0417192_01_rn15	Other	NA
Bc_ctg1_2238	B2	1.981437227	Bcer	M	Bcer	FALSE	Bc_Ga0417192_01_rn15	Other	NA
Bc_ctg1_2239	B2	9.790631004	Bcer	V	Bcer	FALSE	Bc_Ga0417192_01_rn15	Other	NA
Bc_ctg1_224	B2	7.459528384	Bcer	S	Bcer	FALSE	NA	NA	NA
Bc_ctg1_2240	B2	3.380098799	Bcer	V	Bcer	FALSE	Bc_Ga0417192_01_rn15	Transport	SMCOG1000
Bc_ctg1_2241	B2	12.23828875	Bcer	KTV	Bcer	FALSE	NA	NA	NA
Bc_ctg1_2242	B2	17.71637991	Bcer	D	Bcer	FALSE	NA	NA	NA
Bc_ctg1_2243	B2	928.4781735	Bcer	M	Bcer	FALSE	NA	NA	NA
Bc_ctg1_2244	B2	164.8089552	Bcer	S	Bcer	FALSE	NA	NA	NA
Bc_ctg1_2245	B2	11.88862336	Bcer	S	Bcer	FALSE	NA	NA	NA
Bc_ctg1_2246	B2	7.226418122	Bcer	C	Bcer	FALSE	NA	NA	NA
Bc_ctg1_2247	B2	5.594646288	Bcer	C	Bcer	FALSE	NA	NA	NA
Bc_ctg1_2248	B2	6.876752729	Bcer	CP	Bcer	FALSE	NA	NA	NA
Bc_ctg1_2249	B2	0.349665393	Bcer	C	Bcer	FALSE	NA	NA	NA
Bc_ctg1_225	B2	0.233110262	Bcer	G	Bcer	FALSE	NA	NA	NA
Bc_ctg1_2250	B2	1.048996179	Bcer	C	Bcer	FALSE	NA	NA	NA
Bc_ctg1_2251	B2	1.048996179	Bcer	C	Bcer	FALSE	NA	NA	NA
Bc_ctg1_2252	B2	2.097992358	Bcer	C	Bcer	FALSE	NA	NA	NA
Bc_ctg1_2253	B2	2.797323144	Bcer	C	Bcer	FALSE	NA	NA	NA
Bc_ctg1_2254	B2	0.466220524	Bcer	C	Bcer	FALSE	NA	NA	NA
Bc_ctg1_2255	B2	0.582775655	Bcer	C	Bcer	FALSE	NA	NA	NA
Bc_ctg1_2256	B2	0.582775655	Bcer	C	Bcer	FALSE	NA	NA	NA
Bc_ctg1_2257	B2	13.28728493	Bcer	T	Bcer	FALSE	NA	NA	NA
Bc_ctg1_2258	B2	17.36671452	Bcer	S	Bcer	FALSE	NA	NA	NA
Bc_ctg1_2259	B2	11.42240284	Bcer	S	Bcer	FALSE	NA	NA	NA
Bc_ctg1_226	B2	8.974745087	Bcer	NA	Bcer	FALSE	NA	NA	NA
Bc_ctg1_2260	B2	820.1984568	Bcer	C	Bcer	FALSE	NA	NA	NA
Bc_ctg1_2261	B2	4038.868399	Bcer	C	Bcer	FALSE	NA	NA	NA
Bc_ctg1_2262	B2	1280.125004	Bcer	C	Bcer	FALSE	NA	NA	NA
Bc_ctg1_2263	B2	2450.804739	Bcer	C	Bcer	FALSE	NA	NA	NA
Bc_ctg1_2264	B2	465.8708586	Bcer	C	Bcer	FALSE	NA	NA	NA
Bc_ctg1_2265	B2	407.0105174	Bcer	C	Bcer	FALSE	NA	NA	NA
Bc_ctg1_2266	B2	134.0384006	Bcer	C	Bcer	FALSE	NA	NA	NA

Bc_ctg1_2267	B2	711.3359645	Bcer	C	Bcer	FALSE	NA	NA	NA
Bc_ctg1_2268	B2	206.7688024	Bcer	S	Bcer	FALSE	NA	NA	NA
Bc_ctg1_2269	B2	70.51585425	Bcer	S	Bcer	FALSE	NA	NA	NA
Bc_ctg1_2270	B2	219.1236463	Bcer	F	Bcer	FALSE	NA	NA	NA
Bc_ctg1_2271	B2	1590.394762	Bcer	E	Bcer	FALSE	NA	NA	NA
Bc_ctg1_2272	B2	143.3628111	Bcer	S	Bcer	FALSE	NA	NA	NA
Bc_ctg1_2273	B2	72.61384661	Bcer	G	Bcer	FALSE	NA	NA	NA
Bc_ctg1_2274	B2	24.8262429	Bcer	T	Bcer	FALSE	NA	NA	NA
Bc_ctg1_2275	B2	213.4124449	Bcer	G	Bcer	FALSE	NA	NA	NA
Bc_ctg1_2276	B2	0.466220524	Bcer	S	Bcer	FALSE	NA	NA	NA
Bc_ctg1_2277	B2	34.03409825	Bcer	S	Bcer	FALSE	NA	NA	NA
Bc_ctg1_2278	B2	15.38527729	Bcer	NA	Bcer	FALSE	NA	NA	NA
Bc_ctg1_2279	B2	0.466220524	Bcer	NA	Bcer	FALSE	NA	NA	NA
Bc_ctg1_2280	B2	55.36368722	Bcer	P	Bcer	FALSE	NA	NA	NA
Bc_ctg1_2281	B2	51.63392303	Bcer	J	Bcer	FALSE	NA	NA	NA
Bc_ctg1_2282	B2	7.692638646	Bcer	E	Bcer	FALSE	NA	NA	NA
Bc_ctg1_2283	B2	0	Bcer	S	Bcer	FALSE	NA	NA	NA
Bc_ctg1_2284	B2	56.29612827	Bcer	J	Bcer	FALSE	NA	NA	NA
Bc_ctg1_2285	B2	447.1054825	Bcer	J	Bcer	FALSE	NA	NA	NA
Bc_ctg1_2286	B2	74.36217358	Bcer	F	Bcer	FALSE	NA	NA	NA
Bc_ctg1_2287	B2	1702.870464	Bcer	J	Bcer	FALSE	NA	NA	NA
Bc_ctg1_2288	B2	681.9640715	Bcer	K	Bcer	FALSE	NA	NA	NA
Bc_ctg1_2289	B2	453.1663493	Bcer	G	Bcer	FALSE	NA	NA	NA
Bc_ctg1_229	B2	0	Bcer	NA	Bcer	FALSE	NA	NA	NA
Bc_ctg1_2290	B2	718.2127172	Bcer	M	Bcer	FALSE	NA	NA	NA
Bc_ctg1_2291	B2	4641.458427	Bcer	G	Bcer	FALSE	NA	NA	NA
Bc_ctg1_2292	B2	82.28792248	Bcer	T	Bcer	FALSE	NA	NA	NA
Bc_ctg1_2293	B2	18.99848635	Bcer	S	Bcer	FALSE	NA	NA	NA
Bc_ctg1_2294	B2	780.4531572	Bcer	F	Bcer	FALSE	NA	NA	NA
Bc_ctg1_2295	B2	573.3346894	Bcer	K	Bcer	FALSE	NA	NA	NA
Bc_ctg1_2296	B2	9.557520742	Bcer	K	Bcer	FALSE	NA	NA	NA
Bc_ctg1_2297	B2	61.89077456	Bcer	I	Bcer	FALSE	NA	NA	NA
Bc_ctg1_2298	B2	61.89077456	Bcer	I	Bcer	FALSE	NA	NA	NA
Bc_ctg1_2299	B2	55.59679749	Bcer	I	Bcer	FALSE	NA	NA	NA
Bc_ctg1_23	B2	4.778760371	Bcer	E	Bcer	FALSE	NA	NA	NA
Bc_ctg1_230	B2	1.048996179	Bcer	NA	Bcer	FALSE	NA	NA	NA
Bc_ctg1_2300	B2	44.52406004	Bcer	I	Bcer	FALSE	NA	NA	NA
Bc_ctg1_2301	B2	346.6349596	Bcer	C	Bcer	FALSE	NA	NA	NA
Bc_ctg1_2302	B2	1.16555131	Bcer	I	Bcer	FALSE	NA	NA	NA
Bc_ctg1_2303	B2	1.981437227	Bcer	L	Bcer	FALSE	NA	NA	NA
Bc_ctg1_2304	B2	20.63025819	Bcer	T	Bcer	FALSE	NA	NA	NA
Bc_ctg1_2305	B2	0.116555131	Bcer	S	Bcer	FALSE	NA	NA	NA
Bc_ctg1_2306	B2	35.19964956	Bcer	K	Bcer	FALSE	NA	NA	NA
Bc_ctg1_2307	B2	539.5337014	Bcer	E	Bcer	FALSE	NA	NA	NA
Bc_ctg1_2308	B2	13.75350546	Bcer	T	Bcer	FALSE	NA	NA	NA
Bc_ctg1_2309	B2	38.92941375	Bcer	T	Bcer	FALSE	NA	NA	NA
Bc_ctg1_231	B2	13.05417467	Bcer	IQ	Bcer	TRUE	NA	NA	NA
Bc_ctg1_2310	B2	3.613209061	Bcer	V	Bcer	FALSE	NA	NA	NA
Bc_ctg1_2311	B2	4.66220524	Bcer	V	Bcer	FALSE	NA	NA	NA
Bc_ctg1_2312	B2	413.8872702	Bcer	E	Bcer	FALSE	NA	NA	NA
Bc_ctg1_2313	B2	585.9226435	Bcer	M	Bcer	FALSE	NA	NA	NA
Bc_ctg1_2314	B2	1.282106441	Bcer	S	Bcer	FALSE	NA	NA	NA
Bc_ctg1_2315	B2	10.60651692	Bcer	NA	Bcer	FALSE	NA	NA	NA
Bc_ctg1_2316	B2	0.116555131	Bcer	NA	Bcer	FALSE	NA	NA	NA
Bc_ctg1_2317	B2	0.349665393	Bcer	NA	Bcer	FALSE	NA	NA	NA
Bc_ctg1_2318	B2	0.466220524	Bcer	K	Bcer	FALSE	NA	NA	NA
Bc_ctg1_2319	B2	155.6010999	Bcer	J	Bcer	FALSE	NA	NA	NA
Bc_ctg1_232	B2	43.82472925	Bcer	S	Bcer	FALSE	NA	NA	NA
Bc_ctg1_2320	B2	13.40384006	Bcer	S	Bcer	FALSE	NA	NA	NA
Bc_ctg1_2321	B2	29.48844814	Bcer	V	Bcer	FALSE	NA	NA	NA
Bc_ctg1_2322	B2	0.815885917	Bcer	S	Bcer	FALSE	NA	NA	NA
Bc_ctg1_2323	B2	26.10834934	Bcer	G	Bcer	FALSE	NA	NA	NA

Bc_ctg1_2324	B2	290.8050518	Bcer	E	Bcer	FALSE	NA	NA	NA
Bc_ctg1_2325	B2	224.6017374	Bcer	E	Bcer	FALSE	NA	NA	NA
Bc_ctg1_2326	B2	5.82775655	Bcer	EGP	Bcer	FALSE	NA	NA	NA
Bc_ctg1_2327	B2	3.146988537	Bcer	K	Bcer	FALSE	NA	NA	NA
Bc_ctg1_2328	B2	149.7733433	Bcer	C	Bcer	FALSE	NA	NA	NA
Bc_ctg1_2329	B2	9.091300218	Bcer	M	Bcer	FALSE	NA	NA	NA
Bc_ctg1_233	B2	10.02374127	Bcer	V	Bcer	FALSE	NA	NA	NA
Bc_ctg1_2330	B2	96.15798307	Bcer	S	Bcer	FALSE	NA	NA	NA
Bc_ctg1_2331	B2	117.3710169	Bcer	S	Bcer	FALSE	NA	NA	NA
Bc_ctg1_2332	B2	168.0724989	Bcer	G	Bcer	FALSE	NA	NA	NA
Bc_ctg1_2333	B2	382.6504951	Bcer	S	Bcer	FALSE	NA	NA	NA
Bc_ctg1_2334	B2	58.62723089	Bcer	P	Bcer	FALSE	NA	NA	NA
Bc_ctg1_2335	B2	61.42455404	Bcer	HP	Bcer	FALSE	NA	NA	NA
Bc_ctg1_2336	B2	48.25382423	Bcer	P	Bcer	FALSE	NA	NA	NA
Bc_ctg1_2337	B2	39.39563428	Bcer	P	Bcer	FALSE	NA	NA	NA
Bc_ctg1_2338	B2	3.030433406	Bcer	S	Bcer	FALSE	NA	NA	NA
Bc_ctg1_2339	B2	0	Bcer	S	Bcer	FALSE	NA	NA	NA
Bc_ctg1_234	B2	0.699330786	Bcer	NA	Bcer	FALSE	NA	NA	NA
Bc_ctg1_2340	B2	58.51067576	Bcer	H	Bcer	FALSE	NA	NA	NA
Bc_ctg1_2341	B2	0	Bcer	NA	Bcer	FALSE	NA	NA	NA
Bc_ctg1_2343	B2	296.6328084	Bcer	C	Bcer	FALSE	NA	NA	NA
Bc_ctg1_2344	B2	726.7212418	Bcer	C	Bcer	FALSE	NA	NA	NA
Bc_ctg1_2345	B2	58.97689628	Bcer	M	Bcer	FALSE	NA	NA	NA
Bc_ctg1_2346	B2	0.116555131	Bcer	M	Bcer	FALSE	NA	NA	NA
Bc_ctg1_2347	B2	0.932441048	Bcer	S	Bcer	FALSE	NA	NA	NA
Bc_ctg1_2348	B2	46.27238701	Bcer	S	Bcer	FALSE	NA	NA	NA
Bc_ctg1_2349	B2	1.398661572	Bcer	S	Bcer	FALSE	NA	NA	NA
Bc_ctg1_235	B2	1.748326965	Bcer	NA	Bcer	FALSE	NA	NA	NA
Bc_ctg1_2350	B2	18.99848635	Bcer	P	Bcer	FALSE	NA	NA	NA
Bc_ctg1_2351	B2	33.68443286	Bcer	F	Bcer	FALSE	NA	NA	NA
Bc_ctg1_2352	B2	98.83875109	Bcer	S	Bcer	FALSE	Bc_Ga0417192_01_rn16	Ancillary biosynthesis	SMCOG1179
Bc_ctg1_2353	B2	18.18260044	Bcer	EGP	Bcer	FALSE	Bc_Ga0417192_01_rn16	Other	NA
Bc_ctg1_2354	B2	41.84329203	Bcer	L	Bcer	FALSE	Bc_Ga0417192_01_rn16	Other	NA
Bc_ctg1_2355	B2	46.97171779	Bcer	S	Bcer	FALSE	Bc_Ga0417192_01_rn16	Other	NA
Bc_ctg1_2356	B2	36.24864574	Bcer	V	Bcer	FALSE	Bc_Ga0417192_01_rn16	Transport	SMCOG1000
Bc_ctg1_2357	B2	25.1759083	Bcer	E	Bcer	FALSE	Bc_Ga0417192_01_rn16	Other	NA
Bc_ctg1_2358	B2	9.32441048	Bcer	K	Bcer	FALSE	Bc_Ga0417192_01_rn16	Regulation	SMCOG1078
Bc_ctg1_2359	B2	1.515216703	Bcer	S	Bcer	FALSE	Bc_Ga0417192_01_rn16	Other	NA
Bc_ctg1_236	B2	0.466220524	Bcer	K	Bcer	FALSE	NA	NA	NA
Bc_ctg1_2360	B2	37.88041757	Bcer	E	Bcer	FALSE	Bc_Ga0417192_01_rn16	Other	NA
Bc_ctg1_2361	B2	27.85667631	Bcer	E	Bcer	FALSE	Bc_Ga0417192_01_rn16	Other	NA
Bc_ctg1_2362	B2	53.26569487	Bcer	E	Bcer	FALSE	Bc_Ga0417192_01_rn16	Core biosynthesis	SMCOG1168
Bc_ctg1_2363	B2	18.4157107	Bcer	P	Bcer	FALSE	Bc_Ga0417192_01_rn16	Other	NA
Bc_ctg1_2364	B2	1.398661572	Bcer	S	Bcer	FALSE	Bc_Ga0417192_01_rn16	Ancillary biosynthesis	SMCOG1115
Bc_ctg1_2365	B2	16.78393886	Bcer	T	Bcer	FALSE	Bc_Ga0417192_01_rn16	Regulation	SMCOG1133
Bc_ctg1_2366	B2	0	Bcer	L	Bcer	FALSE	Bc_Ga0417192_01_rn16	Other	SMCOG1164
Bc_ctg1_2367	B2	0	Bcer	L	Bcer	FALSE	Bc_Ga0417192_01_rn16	Other	SMCOG1026
Bc_ctg1_2368	B2	6.293977074	Bcer	I	Bcer	FALSE	Bc_Ga0417192_01_rn16	Other	NA
Bc_ctg1_2369	B2	5.478091157	Bcer	K	Bcer	FALSE	Bc_Ga0417192_01_rn16	Regulation	SMCOG1016
Bc_ctg1_237	B2	119.8186747	Bcer	NA	Bcer	FALSE	NA	NA	NA
Bc_ctg1_2370	B2	8.15885917	Bcer	T	Bcer	FALSE	Bc_Ga0417192_01_rn16	Regulation	SMCOG1048
Bc_ctg1_2371	B2	495.1261965	Bcer	H	Bcer	FALSE	Bc_Ga0417192_01_rn16	Other	NA
Bc_ctg1_2372	B2	35.78242522	Bcer	T	Bcer	FALSE	Bc_Ga0417192_01_rn16	Ancillary biosynthesis	SMCOG1025
Bc_ctg1_2373	B2	3.962874454	Bcer	S	Bcer	FALSE	Bc_Ga0417192_01_rn16	Other	SMCOG1211
Bc_ctg1_2374	B2	851.4352319	Bcer	T	Bcer	FALSE	Bc_Ga0417192_01_rn16	Other	SMCOG1185
Bc_ctg1_2375	B2	343.1383057	Bcer	KT	Bcer	FALSE	Bc_Ga0417192_01_rn16	Other	NA
Bc_ctg1_2376	B2	36.94797653	Bcer	G	Bcer	FALSE	Bc_Ga0417192_01_rn16	Transport	SMCOG1169
Bc_ctg1_2377	B2	145.4608035	Bcer	M	Bcer	FALSE	Bc_Ga0417192_01_rn16	Other	NA
Bc_ctg1_2378	B2	175.6485824	Bcer	M	Bcer	FALSE	Bc_Ga0417192_01_rn16	Core biosynthesis	SMCOG1045
Bc_ctg1_2379	B2	267.843691	Bcer	M	Bcer	FALSE	Bc_Ga0417192_01_rn16	Other	NA
Bc_ctg1_238	B2	50.23526146	Bcer	S	Bcer	FALSE	NA	NA	NA
Bc_ctg1_2380	B2	33.21821233	Bcer	S	Bcer	FALSE	Bc_Ga0417192_01_rn16	Other	NA

Bc_ctg1_2381	B2	110.0280437	Bcer	NT	Bcer	FALSE	Bc_Ga0417192_01_rn16	Other	SMCOG1061
Bc_ctg1_2382	B2	23.3110262	Bcer	M	Bcer	FALSE	Bc_Ga0417192_01_rn16	Other	NA
Bc_ctg1_2383	B2	21.21303384	Bcer	I	Bcer	FALSE	Bc_Ga0417192_01_rn16	Other	NA
Bc_ctg1_2384	B2	0.582775655	Bcer	M	Bcer	FALSE	Bc_Ga0417192_01_rn16	Other	NA
Bc_ctg1_2385	B2	31.23677511	Bcer	M	Bcer	FALSE	Bc_Ga0417192_01_rn16	Core biosynthesis	SMCOG1123
Bc_ctg1_2386	B2	97.78975491	Bcer	S	Bcer	FALSE	Bc_Ga0417192_01_rn16	Core biosynthesis	None
Bc_ctg1_2387	B2	487.8997784	Bcer	S	Bcer	FALSE	Bc_Ga0417192_01_rn16	Other	NA
Bc_ctg1_2388	B2	319.0113935	Bcer	M	Bcer	FALSE	Bc_Ga0417192_01_rn16	Core biosynthesis	SMCOG1123
Bc_ctg1_239	B2	40.09496506	Bcer	T	Bcer	FALSE	NA	NA	NA
Bc_ctg1_2390	B2	0	Bcer	NA	Bcer	FALSE	Bc_Ga0417192_01_rn16	Other	NA
Bc_ctg1_2391	B2	3.49665393	Bcer	GM	Bcer	FALSE	Bc_Ga0417192_01_rn16	Ancillary biosynthesis	SMCOG1010
Bc_ctg1_2392	B2	18.88193122	Bcer	K	Bcer	FALSE	Bc_Ga0417192_01_rn16	Regulation	SMCOG1057
Bc_ctg1_2393	B2	158.6315333	Bcer	S	Bcer	FALSE	Bc_Ga0417192_01_rn16	Transport	SMCOG1000
Bc_ctg1_2394	B2	20.51370306	Bcer	K	Bcer	FALSE	Bc_Ga0417192_01_rn16	Regulation	SMCOG1057
Bc_ctg1_2395	B2	201.8734869	Bcer	V	Bcer	FALSE	Bc_Ga0417192_01_rn16	Transport	SMCOG1049
Bc_ctg1_2396	B2	60.49211299	Bcer	O	Bcer	FALSE	NA	NA	NA
Bc_ctg1_2397	B2	402.2317571	Bcer	S	Bcer	FALSE	NA	NA	NA
Bc_ctg1_2398	B2	546.293899	Bcer	M	Bcer	FALSE	NA	NA	NA
Bc_ctg1_2399	B2	177.2803542	Bcer	S	Bcer	FALSE	NA	NA	NA
Bc_ctg1_24	B2	6.177421943	Bcer	E	Bcer	FALSE	NA	NA	NA
Bc_ctg1_240	B2	1.748326965	Bcer	K	Bcer	FALSE	NA	NA	NA
Bc_ctg1_2400	B2	26.34145961	Bcer	T	Bcer	FALSE	NA	NA	NA
Bc_ctg1_2401	B2	49.06971015	Bcer	T	Bcer	FALSE	NA	NA	NA
Bc_ctg1_2402	B2	2.097992358	Bcer	EGP	Bcer	FALSE	Bc_Ga0417192_01_rn17	Transport	SMCOG1169
Bc_ctg1_2403	B2	81.00581604	Bcer	M	Bcer	FALSE	Bc_Ga0417192_01_rn17	Other	NA
Bc_ctg1_2404	B2	331.3662374	Bcer	C	Bcer	FALSE	Bc_Ga0417192_01_rn17	Other	NA
Bc_ctg1_2405	B2	25915.80027	Bcer	P	Bcer	FALSE	Bc_Ga0417192_01_rn17	Other	NA
Bc_ctg1_2406	B2	14.80250164	Bcer	GM	Bcer	FALSE	Bc_Ga0417192_01_rn17	Ancillary biosynthesis	SMCOG1010
Bc_ctg1_2407	B2	13.40384006	Bcer	S	Bcer	FALSE	Bc_Ga0417192_01_rn17	Other	NA
Bc_ctg1_2408	B2	0.466220524	Bcer	NA	Bcer	FALSE	Bc_Ga0417192_01_rn17	Other	NA
Bc_ctg1_2409	B2	368.314214	Bcer	M	Bcer	FALSE	Bc_Ga0417192_01_rn17	Core biosynthesis	SMCOG1010
Bc_ctg1_241	B2	9.674075873	Bcer	S	Bcer	FALSE	NA	NA	NA
Bc_ctg1_2410	B2	275.6528848	Bcer	S	Bcer	FALSE	Bc_Ga0417192_01_rn17	Other	NA
Bc_ctg1_2411	B2	14.91905677	Bcer	K	Bcer	FALSE	Bc_Ga0417192_01_rn17	Other	NA
Bc_ctg1_2412	B2	69.58341321	Bcer	JKL	Bcer	FALSE	Bc_Ga0417192_01_rn17	Other	SMCOG1122
Bc_ctg1_2413	B2	357.9408073	Bcer	F	Bcer	FALSE	Bc_Ga0417192_01_rn17	Other	NA
Bc_ctg1_2414	B2	47.08827292	Bcer	E	Bcer	FALSE	Bc_Ga0417192_01_rn17	Other	NA
Bc_ctg1_2415	B2	32.51888155	Bcer	C	Bcer	FALSE	Bc_Ga0417192_01_rn17	Ancillary biosynthesis	None
Bc_ctg1_2416	B2	92.54477401	Bcer	T	Bcer	FALSE	Bc_Ga0417192_01_rn17	Regulation	SMCOG1003
Bc_ctg1_2417	B2	21.4461441	Bcer	J	Bcer	FALSE	NA	NA	NA
Bc_ctg1_2418	B2	0.582775655	Bcer	S	Bcer	FALSE	NA	NA	NA
Bc_ctg1_2419	B2	231.8281556	Bcer	O	Bcer	FALSE	NA	NA	NA
Bc_ctg1_242	B2	6.527087336	Bcer	F	Bcer	FALSE	NA	NA	NA
Bc_ctg1_2420	B2	149.3071228	Bcer	S	Bcer	FALSE	NA	NA	NA
Bc_ctg1_2421	B2	128.6768646	Bcer	S	Bcer	FALSE	NA	NA	NA
Bc_ctg1_2422	B2	293.1361545	Bcer	S	Bcer	FALSE	NA	NA	NA
Bc_ctg1_2423	B2	520.6517702	Bcer	T	Bcer	FALSE	NA	NA	NA
Bc_ctg1_2424	B2	127.1616479	Bcer	T	Bcer	FALSE	NA	NA	NA
Bc_ctg1_2425	B2	842.9267074	Bcer	F	Bcer	FALSE	NA	NA	NA
Bc_ctg1_2426	B2	819.8487914	Bcer	L	Bcer	FALSE	NA	NA	NA
Bc_ctg1_2427	B2	123.3153286	Bcer	J	Bcer	FALSE	NA	NA	NA
Bc_ctg1_2428	B2	870.3171632	Bcer	T	Bcer	FALSE	NA	NA	NA
Bc_ctg1_2429	B2	19.69781714	Bcer	S	Bcer	FALSE	NA	NA	NA
Bc_ctg1_243	B2	2.564212882	Bcer	S	Bcer	FALSE	NA	NA	NA
Bc_ctg1_2430	B2	346.6349596	Bcer	J	Bcer	FALSE	NA	NA	NA
Bc_ctg1_2431	B2	1620.349431	Bcer	L	Bcer	FALSE	NA	NA	NA
Bc_ctg1_2432	B2	689.4235998	Bcer	J	Bcer	FALSE	NA	NA	NA
Bc_ctg1_2433	B2	364.9341152	Bcer	J	Bcer	FALSE	NA	NA	NA
Bc_ctg1_2434	B2	6.760197598	Bcer	S	Bcer	FALSE	NA	NA	NA
Bc_ctg1_2435	B2	96.74075873	Bcer	M	Bcer	FALSE	NA	NA	NA
Bc_ctg1_2436	B2	0.815885917	Bcer	S	Bcer	FALSE	NA	NA	NA
Bc_ctg1_2437	B2	1462.883449	Bcer	K	Bcer	FALSE	NA	NA	NA

Bc_ctg1_2438	B2	503.2850556	Bcer	D	Bcer	FALSE	NA	NA	NA
Bc_ctg1_2439	B2	336.4946632	Bcer	D	Bcer	FALSE	NA	NA	NA
Bc_ctg1_244	B2	5.128425764	Bcer	E	Bcer	FALSE	NA	NA	NA
Bc_ctg1_2440	B2	145.4608035	Bcer	J	Bcer	FALSE	NA	NA	NA
Bc_ctg1_2441	B2	488.482554	Bcer	D	Bcer	FALSE	NA	NA	NA
Bc_ctg1_2442	B2	221.571304	Bcer	S	Bcer	FALSE	NA	NA	NA
Bc_ctg1_2443	B2	318.3120628	Bcer	S	Bcer	FALSE	NA	NA	NA
Bc_ctg1_2444	B2	326.9371424	Bcer	U	Bcer	FALSE	NA	NA	NA
Bc_ctg1_2445	B2	30.77055458	Bcer	J	Bcer	FALSE	NA	NA	NA
Bc_ctg1_2446	B2	0.582775655	Bcer	J	Bcer	FALSE	NA	NA	NA
Bc_ctg1_2447	B2	350.2481686	Bcer	L	Bcer	FALSE	NA	NA	NA
Bc_ctg1_2448	B2	253.9736304	Bcer	L	Bcer	FALSE	NA	NA	NA
Bc_ctg1_2449	B2	9.440965611	Bcer	S	Bcer	FALSE	NA	NA	NA
Bc_ctg1_245	B2	6.876752729	Bcer	GK	Bcer	FALSE	NA	NA	NA
Bc_ctg1_2450	B2	394.5391184	Bcer	L	Bcer	FALSE	NA	NA	NA
Bc_ctg1_2451	B2	1259.37819	Bcer	L	Bcer	FALSE	NA	NA	NA
Bc_ctg1_2452	B2	2328.888072	Bcer	L	Bcer	FALSE	NA	NA	NA
Bc_ctg1_2454	B2	0.582775655	Bcer	S	Bcer	FALSE	NA	NA	NA
Bc_ctg1_2455	B2	1156.69312	Bcer	F	Bcer	FALSE	NA	NA	NA
Bc_ctg1_2456	B2	301.1784585	Bcer	M	Bcer	FALSE	NA	NA	NA
Bc_ctg1_2457	B2	853.300114	Bcer	H	Bcer	FALSE	NA	NA	NA
Bc_ctg1_2458	B2	555.9679749	Bcer	H	Bcer	FALSE	NA	NA	NA
Bc_ctg1_2459	B2	804.9297347	Bcer	J	Bcer	FALSE	NA	NA	NA
Bc_ctg1_246	B2	4.429094978	Bcer	G	Bcer	FALSE	NA	NA	NA
Bc_ctg1_2460	B2	246.2809918	Bcer	S	Bcer	FALSE	NA	NA	NA
Bc_ctg1_2461	B2	29.95466867	Bcer	F	Bcer	FALSE	NA	NA	NA
Bc_ctg1_2462	B2	18.76537609	Bcer	F	Bcer	FALSE	NA	NA	NA
Bc_ctg1_2463	B2	0.233110262	Bcer	Q	Bcer	TRUE	NA	NA	NA
Bc_ctg1_2464	B2	6.760197598	Bcer	FJ	Bcer	FALSE	NA	NA	NA
Bc_ctg1_2465	B2	642.4518821	Bcer	L	Bcer	FALSE	NA	NA	NA
Bc_ctg1_2466	B2	121.2173362	Bcer	S	Bcer	FALSE	NA	NA	NA
Bc_ctg1_2467	B2	474.4959383	Bcer	L	Bcer	FALSE	NA	NA	NA
Bc_ctg1_2468	B2	118.7696785	Bcer	S	Bcer	FALSE	NA	NA	NA
Bc_ctg1_2469	B2	0.466220524	Bcer	S	Bcer	FALSE	NA	NA	NA
Bc_ctg1_247	B2	1.631771834	Bcer	S	Bcer	FALSE	NA	NA	NA
Bc_ctg1_2471	B2	0	Bcer	S	Bcer	FALSE	NA	NA	NA
Bc_ctg1_2472	B2	69.81652347	Bcer	E	Bcer	FALSE	NA	NA	NA
Bc_ctg1_2473	B2	24.59313264	Bcer	F	Bcer	FALSE	NA	NA	NA
Bc_ctg1_2474	B2	161.7785218	Bcer	L	Bcer	FALSE	NA	NA	NA
Bc_ctg1_2475	B2	242.6677827	Bcer	S	Bcer	FALSE	NA	NA	NA
Bc_ctg1_2476	B2	49.30282041	Bcer	L	Bcer	FALSE	NA	NA	NA
Bc_ctg1_2477	B2	15.50183242	Bcer	S	Bcer	FALSE	NA	NA	NA
Bc_ctg1_2478	B2	3.263543668	Bcer	L	Bcer	FALSE	NA	NA	NA
Bc_ctg1_2479	B2	17.94949017	Bcer	H	Bcer	FALSE	NA	NA	NA
Bc_ctg1_248	B2	4.778760371	Bcer	J	Bcer	FALSE	NA	NA	NA
Bc_ctg1_2480	B2	1020.323617	Bcer	K	Bcer	FALSE	NA	NA	NA
Bc_ctg1_2481	B2	850.3862358	Bcer	J	Bcer	FALSE	NA	NA	NA
Bc_ctg1_2482	B2	43.82472925	Bcer	L	Bcer	FALSE	NA	NA	NA
Bc_ctg1_2483	B2	27.39045578	Bcer	J	Bcer	FALSE	NA	NA	NA
Bc_ctg1_2484	B2	107.2307205	Bcer	J	Bcer	FALSE	NA	NA	NA
Bc_ctg1_2485	B2	7.342973253	Bcer	S	Bcer	FALSE	NA	NA	NA
Bc_ctg1_2486	B2	0.233110262	Bcer	S	Bcer	FALSE	NA	NA	NA
Bc_ctg1_2488	B2	104.5499525	Bcer	I	Bcer	FALSE	NA	NA	NA
Bc_ctg1_2489	B2	427.7573308	Bcer	F	Bcer	FALSE	NA	NA	NA
Bc_ctg1_249	B2	44.17439465	Bcer	I	Bcer	FALSE	NA	NA	NA
Bc_ctg1_2490	B2	98.48908569	Bcer	J	Bcer	FALSE	NA	NA	NA
Bc_ctg1_2491	B2	674.5045431	Bcer	D	Bcer	FALSE	NA	NA	NA
Bc_ctg1_2492	B2	1231.288404	Bcer	M	Bcer	FALSE	NA	NA	NA
Bc_ctg1_2493	B2	1045.14986	Bcer	F	Bcer	FALSE	NA	NA	NA
Bc_ctg1_2494	B2	73.89595305	Bcer	J	Bcer	FALSE	NA	NA	NA
Bc_ctg1_2495	B2	6.410532205	Bcer	S	Bcer	FALSE	NA	NA	NA
Bc_ctg1_2496	B2	546.6435644	Bcer	L	Bcer	FALSE	NA	NA	NA

Bc_ctg1_2497	B2	126.9285377	Bcer	K	Bcer	FALSE	NA	NA	NA
Bc_ctg1_2498	B2	143.8290317	Bcer	S	Bcer	FALSE	NA	NA	NA
Bc_ctg1_2499	B2	187.8868712	Bcer	S	Bcer	FALSE	NA	NA	NA
Bc_ctg1_25	B2	1.748326965	Bcer	K	Bcer	FALSE	NA	NA	NA
Bc_ctg1_250	B2	0.116555131	Bcer	L	Bcer	FALSE	NA	NA	NA
Bc_ctg1_2500	B2	30.42088919	Bcer	J	Bcer	FALSE	NA	NA	NA
Bc_ctg1_2501	B2	47.20482805	Bcer	S	Bcer	FALSE	NA	NA	NA
Bc_ctg1_2502	B2	163.4102937	Bcer	S	Bcer	FALSE	NA	NA	NA
Bc_ctg1_2503	B2	112.3591463	Bcer	D	Bcer	FALSE	NA	NA	NA
Bc_ctg1_2504	B2	756.4428002	Bcer	J	Bcer	FALSE	NA	NA	NA
Bc_ctg1_2505	B2	7.809193777	Bcer	KT	Bcer	FALSE	NA	NA	NA
Bc_ctg1_2506	B2	2286.462005	Bcer	D	Bcer	FALSE	NA	NA	NA
Bc_ctg1_2507	B2	1161.821546	Bcer	F	Bcer	FALSE	NA	NA	NA
Bc_ctg1_2508	B2	6943.538819	Bcer	O	Bcer	FALSE	NA	NA	NA
Bc_ctg1_2509	B2	104.6665076	Bcer	F	Bcer	FALSE	NA	NA	NA
Bc_ctg1_251	B2	0.233110262	Bcer	L	Bcer	FALSE	NA	NA	NA
Bc_ctg1_2510	B2	215.0442167	Bcer	O	Bcer	FALSE	NA	NA	NA
Bc_ctg1_2511	B2	644.5498744	Bcer	E	Bcer	FALSE	NA	NA	NA
Bc_ctg1_2512	B2	153.2699973	Bcer	EH	Bcer	FALSE	NA	NA	NA
Bc_ctg1_2513	B2	92.42821888	Bcer	EH	Bcer	FALSE	NA	NA	NA
Bc_ctg1_2514	B2	85.66802128	Bcer	EH	Bcer	FALSE	NA	NA	NA
Bc_ctg1_2515	B2	91.84544323	Bcer	H	Bcer	FALSE	NA	NA	NA
Bc_ctg1_2516	B2	23.66069159	Bcer	H	Bcer	FALSE	NA	NA	NA
Bc_ctg1_2517	B2	29.02222762	Bcer	H	Bcer	FALSE	NA	NA	NA
Bc_ctg1_2518	B2	0.466220524	Bcer	K	Bcer	FALSE	NA	NA	NA
Bc_ctg1_2519	B2	69.81652347	Bcer	J	Bcer	FALSE	NA	NA	NA
Bc_ctg1_252	B2	51.75047816	Bcer	S	Bcer	FALSE	NA	NA	NA
Bc_ctg1_2520	B2	759.5897887	Bcer	J	Bcer	FALSE	NA	NA	NA
Bc_ctg1_2523	B2	104.4333974	Bcer	K	Bcer	FALSE	NA	NA	NA
Bc_ctg1_2524	B2	195.4629547	Bcer	S	Bcer	FALSE	NA	NA	NA
Bc_ctg1_2525	B2	550.6064388	Bcer	E	Bcer	FALSE	NA	NA	NA
Bc_ctg1_2526	B2	1753.571946	Bcer	O	Bcer	FALSE	NA	NA	NA
Bc_ctg1_2527	B2	74.71183897	Bcer	O	Bcer	FALSE	NA	NA	NA
Bc_ctg1_2528	B2	73.54628766	Bcer	L	Bcer	FALSE	NA	NA	NA
Bc_ctg1_2529	B2	288.3573941	Bcer	S	Bcer	FALSE	NA	NA	NA
Bc_ctg1_253	B2	17.59982478	Bcer	NA	Bcer	FALSE	NA	NA	NA
Bc_ctg1_2530	B2	285.5600709	Bcer	I	Bcer	FALSE	NA	NA	NA
Bc_ctg1_2531	B2	51.28425764	Bcer	I	Bcer	FALSE	NA	NA	NA
Bc_ctg1_2532	B2	1047.131297	Bcer	J	Bcer	FALSE	NA	NA	NA
Bc_ctg1_2533	B2	65.50398362	Bcer	E	Bcer	FALSE	NA	NA	NA
Bc_ctg1_2534	B2	303.5095611	Bcer	J	Bcer	FALSE	NA	NA	NA
Bc_ctg1_2535	B2	29.95466867	Bcer	J	Bcer	FALSE	NA	NA	NA
Bc_ctg1_2536	B2	142.3138149	Bcer	J	Bcer	FALSE	NA	NA	NA
Bc_ctg1_2537	B2	114.6902489	Bcer	S	Bcer	FALSE	NA	NA	NA
Bc_ctg1_2538	B2	269.8251283	Bcer	K	Bcer	FALSE	NA	NA	NA
Bc_ctg1_2539	B2	6.643642467	Bcer	U	Bcer	FALSE	NA	NA	NA
Bc_ctg1_254	B2	3.263543668	Bcer	K	Bcer	FALSE	NA	NA	NA
Bc_ctg1_2540	B2	248.6120944	Bcer	K	Bcer	FALSE	NA	NA	NA
Bc_ctg1_2541	B2	5961.445285	Bcer	J	Bcer	FALSE	NA	NA	NA
Bc_ctg1_2542	B2	1849.846484	Bcer	J	Bcer	FALSE	NA	NA	NA
Bc_ctg1_2543	B2	2999.313186	Bcer	J	Bcer	FALSE	NA	NA	NA
Bc_ctg1_2544	B2	898.8731703	Bcer	J	Bcer	FALSE	NA	NA	NA
Bc_ctg1_2545	B2	236.2572505	Bcer	J	Bcer	FALSE	NA	NA	NA
Bc_ctg1_2546	B2	10912.12447	Bcer	K	Bcer	FALSE	NA	NA	NA
Bc_ctg1_2547	B2	11219.83002	Bcer	K	Bcer	FALSE	NA	NA	NA
Bc_ctg1_2548	B2	653.7577298	Bcer	J	Bcer	FALSE	NA	NA	NA
Bc_ctg1_2549	B2	3011.318364	Bcer	J	Bcer	FALSE	NA	NA	NA
Bc_ctg1_255	B2	0	Bcer	NA	Bcer	FALSE	NA	NA	NA
Bc_ctg1_2550	B2	1843.435952	Bcer	J	Bcer	FALSE	NA	NA	NA
Bc_ctg1_2551	B2	14510.99725	Bcer	J	Bcer	FALSE	NA	NA	NA
Bc_ctg1_2552	B2	15140.97774	Bcer	J	Bcer	FALSE	NA	NA	NA
Bc_ctg1_2553	B2	2024.329515	Bcer	J	Bcer	FALSE	NA	NA	NA

Bc_ctg1_2554	B2	4121.739097	Bcer	J	Bcer	FALSE	NA	NA	NA
Bc_ctg1_2555	B2	4205.658792	Bcer	J	Bcer	FALSE	NA	NA	NA
Bc_ctg1_2556	B2	1197.603971	Bcer	J	Bcer	FALSE	NA	NA	NA
Bc_ctg1_2557	B2	7086.66852	Bcer	J	Bcer	FALSE	NA	NA	NA
Bc_ctg1_2558	B2	2582.395482	Bcer	J	Bcer	FALSE	NA	NA	NA
Bc_ctg1_2559	B2	3357.836769	Bcer	J	Bcer	FALSE	NA	NA	NA
Bc_ctg1_256	B2	1.16555131	Bcer	Q	Bcer	TRUE	NA	NA	NA
Bc_ctg1_2560	B2	4866.526385	Bcer	J	Bcer	FALSE	NA	NA	NA
Bc_ctg1_2561	B2	2533.675438	Bcer	J	Bcer	FALSE	NA	NA	NA
Bc_ctg1_2562	B2	476.011155	Bcer	J	Bcer	FALSE	NA	NA	NA
Bc_ctg1_2563	B2	1324.532509	Bcer	J	Bcer	FALSE	NA	NA	NA
Bc_ctg1_2564	B2	2237.50885	Bcer	J	Bcer	FALSE	NA	NA	NA
Bc_ctg1_2565	B2	2408.611782	Bcer	J	Bcer	FALSE	NA	NA	NA
Bc_ctg1_2566	B2	4384.104697	Bcer	J	Bcer	FALSE	NA	NA	NA
Bc_ctg1_2567	B2	632.1950305	Bcer	J	Bcer	FALSE	NA	NA	NA
Bc_ctg1_2568	B2	2958.752	Bcer	J	Bcer	FALSE	NA	NA	NA
Bc_ctg1_2569	B2	4146.09912	Bcer	J	Bcer	FALSE	NA	NA	NA
Bc_ctg1_257	B2	0	Bcer	Q	Bcer	TRUE	Bc_Ga0417192_01_rn02	Other	NA
Bc_ctg1_2570	B2	2041.463119	Bcer	J	Bcer	FALSE	NA	NA	NA
Bc_ctg1_2571	B2	4766.172417	Bcer	J	Bcer	FALSE	NA	NA	NA
Bc_ctg1_2572	B2	200.9410458	Bcer	J	Bcer	FALSE	NA	NA	NA
Bc_ctg1_2573	B2	2770.398909	Bcer	J	Bcer	FALSE	NA	NA	NA
Bc_ctg1_2574	B2	10221.30221	Bcer	U	Bcer	FALSE	NA	NA	NA
Bc_ctg1_2575	B2	3064.81717	Bcer	F	Bcer	FALSE	NA	NA	NA
Bc_ctg1_2576	B2	5619.589086	Bcer	E	Bcer	FALSE	NA	NA	NA
Bc_ctg1_2577	B2	552.5878761	Bcer	J	Bcer	FALSE	NA	NA	NA
Bc_ctg1_2578	B2	0.233110262	Bcer	J	Bcer	FALSE	NA	NA	NA
Bc_ctg1_2579	B2	4084.674566	Bcer	J	Bcer	FALSE	NA	NA	NA
Bc_ctg1_258	B2	12.70450928	Bcer	S	Bcer	FALSE	Bc_Ga0417192_01_rn02	Other	SMCOG1272
Bc_ctg1_2580	B2	1950.433562	Bcer	J	Bcer	FALSE	NA	NA	NA
Bc_ctg1_2581	B2	7698.582957	Bcer	K	Bcer	FALSE	NA	NA	NA
Bc_ctg1_2582	B2	1304.834692	Bcer	J	Bcer	FALSE	NA	NA	NA
Bc_ctg1_2583	B2	131.2410775	Bcer	P	Bcer	FALSE	NA	NA	NA
Bc_ctg1_2584	B2	75.06150436	Bcer	P	Bcer	FALSE	NA	NA	NA
Bc_ctg1_2585	B2	48.83659989	Bcer	P	Bcer	FALSE	NA	NA	NA
Bc_ctg1_2586	B2	97.78975491	Bcer	J	Bcer	FALSE	NA	NA	NA
Bc_ctg1_2587	B2	2742.309122	Bcer	J	Bcer	FALSE	NA	NA	NA
Bc_ctg1_2588	B2	1069.276772	Bcer	J	Bcer	FALSE	NA	NA	NA
Bc_ctg1_2589	B2	1.631771834	Bcer	S	Bcer	FALSE	NA	NA	NA
Bc_ctg1_259	B2	2.564212882	Bcer	NA	Bcer	FALSE	Bc_Ga0417192_01_rn02	Other	NA
Bc_ctg1_2590	B2	1.864882096	Bcer	M	Bcer	FALSE	NA	NA	NA
Bc_ctg1_2591	B2	452.2339083	Bcer	D	Bcer	FALSE	NA	NA	NA
Bc_ctg1_2592	B2	0	Bcer	S	Bcer	FALSE	NA	NA	NA
Bc_ctg1_2593	B2	59.90933733	Bcer	S	Bcer	FALSE	NA	NA	NA
Bc_ctg1_2594	B2	1.515216703	Bcer	G	Bcer	FALSE	NA	NA	NA
Bc_ctg1_2595	B2	0	Bcer	NA	Bcer	FALSE	NA	NA	NA
Bc_ctg1_2597	B2	1.515216703	Bcer	G	Bcer	FALSE	NA	NA	NA
Bc_ctg1_2598	B2	215.1607718	Bcer	E	Bcer	FALSE	NA	NA	NA
Bc_ctg1_2599	B2	138.2343854	Bcer	S	Bcer	FALSE	NA	NA	NA
Bc_ctg1_26	B2	149.5402331	Bcer	U	Bcer	FALSE	NA	NA	NA
Bc_ctg1_260	B2	0.466220524	Bcer	S	Bcer	FALSE	Bc_Ga0417192_01_rn02	Other	NA
Bc_ctg1_2600	B2	340.6906479	Bcer	S	Bcer	FALSE	NA	NA	NA
Bc_ctg1_2601	B2	636.7406806	Bcer	G	Bcer	FALSE	NA	NA	NA
Bc_ctg1_2602	B2	908.3141359	Bcer	M	Bcer	FALSE	NA	NA	NA
Bc_ctg1_2603	B2	6.060866812	Bcer	EU	Bcer	FALSE	NA	NA	NA
Bc_ctg1_2604	B2	24.8262429	Bcer	S	Bcer	FALSE	NA	NA	NA
Bc_ctg1_2605	B2	669.4926724	Bcer	S	Bcer	FALSE	NA	NA	NA
Bc_ctg1_2606	B2	57.92790011	Bcer	S	Bcer	FALSE	NA	NA	NA
Bc_ctg1_2607	B2	146.3932445	Bcer	IQ	Bcer	TRUE	NA	NA	NA
Bc_ctg1_2608	B2	3.146988537	Bcer	P	Bcer	FALSE	NA	NA	NA
Bc_ctg1_2609	B2	8.275414301	Bcer	P	Bcer	FALSE	NA	NA	NA
Bc_ctg1_261	B2	13.05417467	Bcer	S	Bcer	FALSE	Bc_Ga0417192_01_rn02	Other	NA

Bc_ctg1_2610	B2	5.244980895	Bcer	P	Bcer	FALSE	NA	NA	NA
Bc_ctg1_2611	B2	0.233110262	Bcer	C	Bcer	FALSE	NA	NA	NA
Bc_ctg1_2612	B2	20.63025819	Bcer	NA	Bcer	FALSE	NA	NA	NA
Bc_ctg1_2613	B2	9.440965611	Bcer	C	Bcer	FALSE	NA	NA	NA
Bc_ctg1_2614	B2	3.729764192	Bcer	G	Bcer	FALSE	NA	NA	NA
Bc_ctg1_2615	B2	66.66953493	Bcer	S	Bcer	FALSE	NA	NA	NA
Bc_ctg1_2616	B2	16.66738373	Bcer	EGP	Bcer	FALSE	NA	NA	NA
Bc_ctg1_2617	B2	3.846319323	Bcer	NA	Bcer	FALSE	NA	NA	NA
Bc_ctg1_2618	B2	18.99848635	Bcer	NA	Bcer	FALSE	NA	NA	NA
Bc_ctg1_2619	B2	0	Bcer	S	Bcer	FALSE	NA	NA	NA
Bc_ctg1_262	B2	20.51370306	Bcer	S	Bcer	FALSE	Bc_Ga0417192_01_rn02	Other	NA
Bc_ctg1_2620	B2	0.582775655	Bcer	P	Bcer	FALSE	NA	NA	NA
Bc_ctg1_2621	B2	0.815885917	Bcer	EP	Bcer	FALSE	NA	NA	NA
Bc_ctg1_2622	B2	2.447657751	Bcer	P	Bcer	FALSE	NA	NA	NA
Bc_ctg1_2623	B2	1.864882096	Bcer	E	Bcer	FALSE	NA	NA	NA
Bc_ctg1_2624	B2	8.275414301	Bcer	E	Bcer	FALSE	NA	NA	NA
Bc_ctg1_2625	B2	0.116555131	Bcer	S	Bcer	FALSE	NA	NA	NA
Bc_ctg1_2626	B2	0	Bcer	Q	Bcer	TRUE	NA	NA	NA
Bc_ctg1_2627	B2	177.6300196	Bcer	E	Bcer	FALSE	NA	NA	NA
Bc_ctg1_2628	B2	169.2380502	Bcer	E	Bcer	FALSE	NA	NA	NA
Bc_ctg1_2629	B2	129.02653	Bcer	S	Bcer	FALSE	NA	NA	NA
Bc_ctg1_263	B2	258.9855011	Bcer	S	Bcer	FALSE	Bc_Ga0417192_01_rn02	Other	SMCOG1273
Bc_ctg1_2630	B2	108.7459372	Bcer	E	Bcer	FALSE	NA	NA	NA
Bc_ctg1_2631	B2	8.508524563	Bcer	H	Bcer	FALSE	NA	NA	NA
Bc_ctg1_2632	B2	61.3079989	Bcer	U	Bcer	FALSE	NA	NA	NA
Bc_ctg1_2633	B2	78.90782369	Bcer	P	Bcer	FALSE	NA	NA	NA
Bc_ctg1_2634	B2	122.6159978	Bcer	P	Bcer	FALSE	NA	NA	NA
Bc_ctg1_2635	B2	55.71335262	Bcer	P	Bcer	FALSE	NA	NA	NA
Bc_ctg1_2638	B2	7.342973253	Bcer	K	Bcer	FALSE	NA	NA	NA
Bc_ctg1_2639	B2	2.564212882	Bcer	EG	Bcer	FALSE	NA	NA	NA
Bc_ctg1_264	B2	71.91451583	Bcer	K	Bcer	FALSE	Bc_Ga0417192_01_rn02	Regulation	SMCOG1171
Bc_ctg1_2642	B2	44.17439465	Bcer	I	Bcer	FALSE	NA	NA	NA
Bc_ctg1_2644	B2	15.26872216	Bcer	K	Bcer	FALSE	NA	NA	NA
Bc_ctg1_2645	B2	1.048996179	Bcer	NA	Bcer	FALSE	NA	NA	NA
Bc_ctg1_2646	B2	50.23526146	Bcer	S	Bcer	FALSE	NA	NA	NA
Bc_ctg1_2647	B2	0.116555131	Bcer	S	Bcer	FALSE	NA	NA	NA
Bc_ctg1_2648	B2	0	Bcer	NA	Bcer	FALSE	NA	NA	NA
Bc_ctg1_2649	B2	5.711201419	Bcer	L	Bcer	FALSE	NA	NA	NA
Bc_ctg1_265	B2	0	Bcer	NA	Bcer	FALSE	Bc_Ga0417192_01_rn02	Other	NA
Bc_ctg1_2650	B2	36.01553548	Bcer	S	Bcer	FALSE	NA	NA	NA
Bc_ctg1_2651	B2	54.54780131	Bcer	M	Bcer	FALSE	NA	NA	NA
Bc_ctg1_2652	B2	9.32441048	Bcer	C	Bcer	FALSE	NA	NA	NA
Bc_ctg1_2654	B2	20.04748253	Bcer	S	Bcer	FALSE	NA	NA	NA
Bc_ctg1_2655	B2	0	Bcer	NA	Bcer	FALSE	NA	NA	NA
Bc_ctg1_2656	B2	109.5618231	Bcer	E	Bcer	FALSE	NA	NA	NA
Bc_ctg1_2657	B2	9.091300218	Bcer	P	Bcer	FALSE	NA	NA	NA
Bc_ctg1_2658	B2	11.18929258	Bcer	EP	Bcer	FALSE	NA	NA	NA
Bc_ctg1_2659	B2	20.74681332	Bcer	P	Bcer	FALSE	NA	NA	NA
Bc_ctg1_266	B2	0.582775655	Bcer	JM	Bcer	FALSE	Bc_Ga0417192_01_rn02	Ancillary biosynthesis	SMCOG1064
Bc_ctg1_2660	B2	24.12691212	Bcer	E	Bcer	FALSE	NA	NA	NA
Bc_ctg1_2661	B2	264.8132576	Bcer	S	Bcer	FALSE	NA	NA	NA
Bc_ctg1_2663	B2	0	Bcer	NA	Bcer	FALSE	NA	NA	NA
Bc_ctg1_2664	B2	0	Bcer	NA	Bcer	FALSE	NA	NA	NA
Bc_ctg1_2665	B2	0	Bcer	NA	Bcer	FALSE	NA	NA	NA
Bc_ctg1_2667	B2	41.02740611	Bcer	E	Bcer	FALSE	NA	NA	NA
Bc_ctg1_2668	B2	18.88193122	Bcer	Q	Bcer	TRUE	NA	NA	NA
Bc_ctg1_2669	B2	54.43124618	Bcer	Q	Bcer	TRUE	NA	NA	NA
Bc_ctg1_267	B2	0	Bcer	GM	Bcer	FALSE	Bc_Ga0417192_01_rn02	Core biosynthesis	SMCOG1010
Bc_ctg1_2670	B2	5.478091157	Bcer	E	Bcer	FALSE	NA	NA	NA
Bc_ctg1_2671	B2	5.82775655	Bcer	EGP	Bcer	FALSE	NA	NA	NA
Bc_ctg1_2672	B2	188.9358673	Bcer	F	Bcer	FALSE	NA	NA	NA
Bc_ctg1_2673	B2	193.8311828	Bcer	M	Bcer	FALSE	NA	NA	NA

Bc_ctg1_2674	B2	1783.060394	Bcer	JKL	Bcer	FALSE	NA	NA	NA
Bc_ctg1_2675	B2	5.478091157	Bcer	L	Bcer	FALSE	NA	NA	NA
Bc_ctg1_2676	B2	0.116555131	Bcer	S	Bcer	FALSE	NA	NA	NA
Bc_ctg1_2677	B2	2.797323144	Bcer	I	Bcer	FALSE	NA	NA	NA
Bc_ctg1_2678	B2	21.4461441	Bcer	M	Bcer	FALSE	NA	NA	NA
Bc_ctg1_2679	B2	15.38527729	Bcer	E	Bcer	FALSE	NA	NA	NA
Bc_ctg1_268	B2	0	Bcer	H	Bcer	FALSE	Bc_Ga0417192_01_rn02	Other	NA
Bc_ctg1_2680	B2	482.3051321	Bcer	K	Bcer	FALSE	NA	NA	NA
Bc_ctg1_2681	B2	608.7674492	Bcer	L	Bcer	FALSE	NA	NA	NA
Bc_ctg1_2682	B2	158.1653128	Bcer	K	Bcer	FALSE	NA	NA	NA
Bc_ctg1_2683	B2	2.214547489	Bcer	S	Bcer	FALSE	NA	NA	NA
Bc_ctg1_2685	B2	10.72307205	Bcer	S	Bcer	FALSE	NA	NA	NA
Bc_ctg1_2686	B2	36.481756	Bcer	O	Bcer	FALSE	NA	NA	NA
Bc_ctg1_2687	B2	8.391969432	Bcer	K	Bcer	FALSE	NA	NA	NA
Bc_ctg1_2688	B2	80.30648526	Bcer	O	Bcer	FALSE	NA	NA	NA
Bc_ctg1_2689	B2	218.1912052	Bcer	S	Bcer	FALSE	NA	NA	NA
Bc_ctg1_269	B2	0	Bcer	GM	Bcer	FALSE	Bc_Ga0417192_01_rn02	Core biosynthesis	SMCOG1010
Bc_ctg1_2690	B2	748.5170513	Bcer	K	Bcer	FALSE	NA	NA	NA
Bc_ctg1_2691	B2	6.293977074	Bcer	S	Bcer	FALSE	NA	NA	NA
Bc_ctg1_2692	B2	109.6783783	Bcer	S	Bcer	FALSE	NA	NA	NA
Bc_ctg1_2693	B2	564.9427199	Bcer	O	Bcer	FALSE	NA	NA	NA
Bc_ctg1_2694	B2	3441.057132	Bcer	O	Bcer	FALSE	NA	NA	NA
Bc_ctg1_2695	B2	53.49880513	Bcer	S	Bcer	FALSE	NA	NA	NA
Bc_ctg1_2696	B2	39.97840993	Bcer	S	Bcer	FALSE	NA	NA	NA
Bc_ctg1_2697	B2	1247.839232	Bcer	F	Bcer	FALSE	NA	NA	NA
Bc_ctg1_2698	B2	429.1559923	Bcer	S	Bcer	FALSE	NA	NA	NA
Bc_ctg1_2699	B2	6.177421943	Bcer	T	Bcer	FALSE	NA	NA	NA
Bc_ctg1_27	B2	76.6932762	Bcer	P	Bcer	FALSE	NA	NA	NA
Bc_ctg1_270	B2	1.398661572	Bcer	NA	Bcer	FALSE	Bc_Ga0417192_01_rn02	Other	NA
Bc_ctg1_2700	B2	31.46988537	Bcer	T	Bcer	FALSE	NA	NA	NA
Bc_ctg1_2702	B2	17.01704913	Bcer	S	Bcer	FALSE	NA	NA	NA
Bc_ctg1_2703	B2	25.87523908	Bcer	Q	Bcer	TRUE	NA	NA	NA
Bc_ctg1_2705	B2	9.091300218	Bcer	S	Bcer	FALSE	NA	NA	NA
Bc_ctg1_2707	B2	7.342973253	Bcer	V	Bcer	FALSE	NA	NA	NA
Bc_ctg1_2708	B2	3.846319323	Bcer	S	Bcer	FALSE	NA	NA	NA
Bc_ctg1_2709	B2	4.079429585	Bcer	V	Bcer	FALSE	NA	NA	NA
Bc_ctg1_271	B2	0	Bcer	S	Bcer	FALSE	Bc_Ga0417192_01_rn02	Other	NA
Bc_ctg1_2710	B2	3.146988537	Bcer	T	Bcer	FALSE	NA	NA	NA
Bc_ctg1_2711	B2	2.797323144	Bcer	T	Bcer	FALSE	NA	NA	NA
Bc_ctg1_2713	B2	0	Bcer	S	Bcer	FALSE	Bc_Ga0417192_01_rn18	Other	NA
Bc_ctg1_2714	B2	0.582775655	Bcer	Q	Bcer	TRUE	Bc_Ga0417192_01_rn18	Ancillary biosynthesis	SMCOG1089
Bc_ctg1_2715	B2	1.515216703	Bcer	Q	Bcer	TRUE	Bc_Ga0417192_01_rn18	Other	NA
Bc_ctg1_2716	B2	0.233110262	Bcer	G	Bcer	FALSE	Bc_Ga0417192_01_rn18	Other	NA
Bc_ctg1_2717	B2	2.680768013	Bcer	M	Bcer	FALSE	Bc_Ga0417192_01_rn18	Ancillary biosynthesis	SMCOG1010
Bc_ctg1_2718	B2	0.233110262	Bcer	M	Bcer	FALSE	Bc_Ga0417192_01_rn18	Other	NA
Bc_ctg1_2719	B2	0.932441048	Bcer	M	Bcer	FALSE	Bc_Ga0417192_01_rn18	Core biosynthesis	SMCOG1045
Bc_ctg1_272	B2	43.35850873	Bcer	EG	Bcer	FALSE	Bc_Ga0417192_01_rn02	Transport	SMCOG1166
Bc_ctg1_2721	B2	27.15734552	Bcer	EG	Bcer	FALSE	Bc_Ga0417192_01_rn18	Transport	SMCOG1166
Bc_ctg1_2722	B2	0.233110262	Bcer	G	Bcer	FALSE	Bc_Ga0417192_01_rn18	Other	NA
Bc_ctg1_2724	B2	220.1726425	Bcer	F	Bcer	FALSE	NA	NA	NA
Bc_ctg1_2725	B2	1191.776214	Bcer	F	Bcer	FALSE	NA	NA	NA
Bc_ctg1_2726	B2	1778.631299	Bcer	F	Bcer	FALSE	NA	NA	NA
Bc_ctg1_2727	B2	235.5579197	Bcer	F	Bcer	FALSE	NA	NA	NA
Bc_ctg1_2728	B2	84.9686905	Bcer	F	Bcer	FALSE	NA	NA	NA
Bc_ctg1_2729	B2	421.5799088	Bcer	F	Bcer	FALSE	NA	NA	NA
Bc_ctg1_273	B2	6.527087336	Bcer	S	Bcer	FALSE	Bc_Ga0417192_01_rn02	Other	SMCOG1286
Bc_ctg1_2730	B2	1748.093855	Bcer	F	Bcer	FALSE	NA	NA	NA
Bc_ctg1_2731	B2	1258.91197	Bcer	F	Bcer	FALSE	NA	NA	NA
Bc_ctg1_2732	B2	497.9235196	Bcer	F	Bcer	FALSE	NA	NA	NA
Bc_ctg1_2733	B2	207.4681332	Bcer	F	Bcer	FALSE	NA	NA	NA
Bc_ctg1_2734	B2	1150.399143	Bcer	F	Bcer	FALSE	NA	NA	NA
Bc_ctg1_2735	B2	467.8522958	Bcer	F	Bcer	FALSE	NA	NA	NA

Bc_ctg1_2736	B2	3.613209061	Bcer	EGP	Bcer	FALSE	NA	NA	NA
Bc_ctg1_2737	B2	4.895315502	Bcer	K	Bcer	FALSE	NA	NA	NA
Bc_ctg1_2738	B2	8.391969432	Bcer	S	Bcer	FALSE	NA	NA	NA
Bc_ctg1_2739	B2	188.586202	Bcer	O	Bcer	FALSE	NA	NA	NA
Bc_ctg1_274	B2	4.66220524	Bcer	S	Bcer	FALSE	Bc_Ga0417192_01_rn02	Other	NA
Bc_ctg1_2740	B2	0	Bcer	S	Bcer	FALSE	NA	NA	NA
Bc_ctg1_2741	B2	20.74681332	Bcer	I	Bcer	FALSE	NA	NA	NA
Bc_ctg1_2742	B2	197.2112816	Bcer	L	Bcer	FALSE	NA	NA	NA
Bc_ctg1_2743	B2	343.7210813	Bcer	L	Bcer	FALSE	NA	NA	NA
Bc_ctg1_2744	B2	203.3887036	Bcer	S	Bcer	FALSE	NA	NA	NA
Bc_ctg1_2745	B2	15.0356119	Bcer	S	Bcer	FALSE	NA	NA	NA
Bc_ctg1_2746	B2	7866.655456	Bcer	C	Bcer	FALSE	NA	NA	NA
Bc_ctg1_2747	B2	6.643642467	Bcer	Q	Bcer	TRUE	NA	NA	NA
Bc_ctg1_2748	B2	36.01553548	Bcer	P	Bcer	FALSE	NA	NA	NA
Bc_ctg1_2749	B2	11.53895797	Bcer	P	Bcer	FALSE	NA	NA	NA
Bc_ctg1_275	B2	48.95315502	Bcer	G	Bcer	FALSE	Bc_Ga0417192_01_rn02	Other	NA
Bc_ctg1_2750	B2	26.10834934	Bcer	M	Bcer	FALSE	NA	NA	NA
Bc_ctg1_2751	B2	0	Bcer	S	Bcer	FALSE	NA	NA	NA
Bc_ctg1_2752	B2	9.32441048	Bcer	D	Bcer	FALSE	NA	NA	NA
Bc_ctg1_2753	B2	20.97992358	Bcer	K	Bcer	FALSE	NA	NA	NA
Bc_ctg1_2754	B2	4.895315502	Bcer	S	Bcer	FALSE	NA	NA	NA
Bc_ctg1_2755	B2	168.4221643	Bcer	J	Bcer	FALSE	NA	NA	NA
Bc_ctg1_2756	B2	951.6726446	Bcer	J	Bcer	FALSE	NA	NA	NA
Bc_ctg1_2757	B2	1159.140778	Bcer	J	Bcer	FALSE	NA	NA	NA
Bc_ctg1_2758	B2	169.937381	Bcer	I	Bcer	FALSE	NA	NA	NA
Bc_ctg1_2759	B2	4.66220524	Bcer	S	Bcer	FALSE	NA	NA	NA
Bc_ctg1_276	B2	21.67925437	Bcer	G	Bcer	FALSE	Bc_Ga0417192_01_rn02	Ancillary biosynthesis	SMCOG1210
Bc_ctg1_2760	B2	0.233110262	Bcer	E	Bcer	FALSE	NA	NA	NA
Bc_ctg1_2761	B2	9.674075873	Bcer	KT	Bcer	FALSE	NA	NA	NA
Bc_ctg1_2762	B2	46.73860753	Bcer	C	Bcer	FALSE	NA	NA	NA
Bc_ctg1_2763	B2	6.99330786	Bcer	P	Bcer	FALSE	NA	NA	NA
Bc_ctg1_2764	B2	372.2770884	Bcer	E	Bcer	FALSE	NA	NA	NA
Bc_ctg1_2765	B2	41.61018177	Bcer	G	Bcer	FALSE	Bc_Ga0417192_01_rn19	Other	NA
Bc_ctg1_2766	B2	1.398661572	Bcer	NA	Bcer	FALSE	Bc_Ga0417192_01_rn19	Other	NA
Bc_ctg1_2767	B2	6.177421943	Bcer	NA	Bcer	FALSE	Bc_Ga0417192_01_rn19	Other	NA
Bc_ctg1_2768	B2	12.47139902	Bcer	F	Bcer	FALSE	Bc_Ga0417192_01_rn19	Other	NA
Bc_ctg1_2769	B2	65.97020414	Bcer	J	Bcer	FALSE	Bc_Ga0417192_01_rn19	Other	NA
Bc_ctg1_277	B2	12.35484389	Bcer	EG	Bcer	FALSE	Bc_Ga0417192_01_rn02	Other	NA
Bc_ctg1_2770	B2	61.19144377	Bcer	J	Bcer	FALSE	Bc_Ga0417192_01_rn19	Other	NA
Bc_ctg1_2771	B2	34.84998417	Bcer	V	Bcer	FALSE	Bc_Ga0417192_01_rn19	Transport	SMCOG1288
Bc_ctg1_2772	B2	79.60715447	Bcer	KLT	Bcer	FALSE	Bc_Ga0417192_01_rn19	Core biosynthesis	SMCOG1030
Bc_ctg1_278	B2	19.69781714	Bcer	G	Bcer	FALSE	Bc_Ga0417192_01_rn02	Other	NA
Bc_ctg1_2785	B2	2.33110262	Bcer	S	Bcer	FALSE	Bc_Ga0417192_01_rn19	Other	NA
Bc_ctg1_2786	B2	0.932441048	Bcer	S	Bcer	FALSE	Bc_Ga0417192_01_rn19	Other	NA
Bc_ctg1_2787	B2	17.36671452	Bcer	J	Bcer	FALSE	Bc_Ga0417192_01_rn19	Other	NA
Bc_ctg1_2788	B2	22.37858515	Bcer	M	Bcer	FALSE	Bc_Ga0417192_01_rn19	Ancillary biosynthesis	SMCOG1268
Bc_ctg1_2789	B2	5.944311681	Bcer	KT	Bcer	FALSE	Bc_Ga0417192_01_rn19	Other	NA
Bc_ctg1_279	B2	29.7215584	Bcer	G	Bcer	FALSE	Bc_Ga0417192_01_rn02	Ancillary biosynthesis	SMCOG1100
Bc_ctg1_2790	B2	3.729764192	Bcer	C	Bcer	FALSE	Bc_Ga0417192_01_rn19	Other	NA
Bc_ctg1_2791	B2	51.86703329	Bcer	S	Bcer	FALSE	Bc_Ga0417192_01_rn19	Other	NA
Bc_ctg1_2792	B2	5.944311681	Bcer	M	Bcer	FALSE	NA	NA	NA
Bc_ctg1_2795	B2	8063.633628	Bcer	O	Bcer	FALSE	NA	NA	NA
Bc_ctg1_2796	B2	3953.433488	Bcer	O	Bcer	FALSE	NA	NA	NA
Bc_ctg1_2797	B2	120.1683401	Bcer	F	Bcer	FALSE	NA	NA	NA
Bc_ctg1_2798	B2	150.5892292	Bcer	E	Bcer	FALSE	NA	NA	NA
Bc_ctg1_2799	B2	115.3895797	Bcer	G	Bcer	FALSE	NA	NA	NA
Bc_ctg1_28	B2	13.1707298	Bcer	K	Bcer	FALSE	NA	NA	NA
Bc_ctg1_280	B2	64.10532205	Bcer	G	Bcer	FALSE	NA	NA	NA
Bc_ctg1_2800	B2	0.233110262	Bcer	NA	Bcer	FALSE	NA	NA	NA
Bc_ctg1_2801	B2	19.81437227	Bcer	P	Bcer	FALSE	NA	NA	NA
Bc_ctg1_2802	B2	8.741634825	Bcer	P	Bcer	FALSE	NA	NA	NA
Bc_ctg1_2803	B2	148.7243472	Bcer	P	Bcer	FALSE	NA	NA	NA

Bc_ctg1_2804	B2	202.6893728	Bcer	C	Bcer	FALSE	NA	NA	NA
Bc_ctg1_2805	B2	134.8542866	Bcer	NA	Bcer	FALSE	NA	NA	NA
Bc_ctg1_2806	B2	1.048996179	Bcer	G	Bcer	FALSE	NA	NA	NA
Bc_ctg1_2807	B2	0.116555131	Bcer	NA	Bcer	FALSE	NA	NA	NA
Bc_ctg1_2808	B2	0.233110262	Bcer	NA	Bcer	FALSE	NA	NA	NA
Bc_ctg1_2809	B2	0.932441048	Bcer	NA	Bcer	FALSE	NA	NA	NA
Bc_ctg1_281	B2	41.84329203	Bcer	G	Bcer	FALSE	NA	NA	NA
Bc_ctg1_2810	B2	2.447657751	Bcer	E	Bcer	FALSE	NA	NA	NA
Bc_ctg1_2811	B2	26.34145961	Bcer	T	Bcer	FALSE	NA	NA	NA
Bc_ctg1_2812	B2	5.244980895	Bcer	S	Bcer	FALSE	NA	NA	NA
Bc_ctg1_2813	B2	15.26872216	Bcer	S	Bcer	FALSE	NA	NA	NA
Bc_ctg1_2814	B2	1.515216703	Bcer	F	Bcer	FALSE	NA	NA	NA
Bc_ctg1_2815	B2	37.18108679	Bcer	S	Bcer	FALSE	NA	NA	NA
Bc_ctg1_2816	B2	2.680768013	Bcer	EGP	Bcer	FALSE	NA	NA	NA
Bc_ctg1_2817	B2	1.16555131	Bcer	CE	Bcer	FALSE	NA	NA	NA
Bc_ctg1_2818	B2	701.8949989	Bcer	I	Bcer	FALSE	NA	NA	NA
Bc_ctg1_2819	B2	0.699330786	Bcer	P	Bcer	FALSE	NA	NA	NA
Bc_ctg1_282	B2	0	Bcer	G	Bcer	FALSE	NA	NA	NA
Bc_ctg1_2820	B2	1.048996179	Bcer	ET	Bcer	FALSE	NA	NA	NA
Bc_ctg1_2821	B2	0.932441048	Bcer	E	Bcer	FALSE	NA	NA	NA
Bc_ctg1_2822	B2	71.56485043	Bcer	NT	Bcer	FALSE	NA	NA	NA
Bc_ctg1_2823	B2	26.691125	Bcer	K	Bcer	FALSE	NA	NA	NA
Bc_ctg1_2824	B2	3539.546218	Bcer	E	Bcer	FALSE	NA	NA	NA
Bc_ctg1_2825	B2	1443.185632	Bcer	E	Bcer	FALSE	NA	NA	NA
Bc_ctg1_2826	B2	1806.954196	Bcer	E	Bcer	FALSE	NA	NA	NA
Bc_ctg1_2827	B2	975.6830016	Bcer	E	Bcer	FALSE	Bc_Ga0417192_01_rn20	Other	NA
Bc_ctg1_2828	B2	203.8549241	Bcer	K	Bcer	FALSE	Bc_Ga0417192_01_rn20	Regulation	SMCOG1133
Bc_ctg1_2829	B2	21.56269923	Bcer	NA	Bcer	FALSE	Bc_Ga0417192_01_rn20	Other	NA
Bc_ctg1_283	B2	55.13057696	Bcer	C	Bcer	FALSE	NA	NA	NA
Bc_ctg1_2830	B2	0	Bcer	C	Bcer	FALSE	Bc_Ga0417192_01_rn20	Other	NA
Bc_ctg1_2831	B2	230.5460491	Bcer	G	Bcer	FALSE	Bc_Ga0417192_01_rn20	Ancillary biosynthesis	SMCOG1257
Bc_ctg1_2832	B2	269.4754629	Bcer	G	Bcer	FALSE	Bc_Ga0417192_01_rn20	Ancillary biosynthesis	SMCOG1145
Bc_ctg1_2833	B2	107.1141654	Bcer	L	Bcer	FALSE	Bc_Ga0417192_01_rn20	Other	NA
Bc_ctg1_2834	B2	205.7198062	Bcer	S	Bcer	FALSE	Bc_Ga0417192_01_rn20	Other	NA
Bc_ctg1_2835	B2	225.7672887	Bcer	L	Bcer	FALSE	Bc_Ga0417192_01_rn20	Other	NA
Bc_ctg1_2836	B2	21.56269923	Bcer	H	Bcer	FALSE	Bc_Ga0417192_01_rn20	Other	NA
Bc_ctg1_2837	B2	8.974745087	Bcer	H	Bcer	FALSE	Bc_Ga0417192_01_rn20	Ancillary biosynthesis	SMCOG1280
Bc_ctg1_2838	B2	7.109862991	Bcer	S	Bcer	FALSE	Bc_Ga0417192_01_rn20	Other	NA
Bc_ctg1_2839	B2	66.43642467	Bcer	NT	Bcer	FALSE	Bc_Ga0417192_01_rn20	Other	SMCOG1061
Bc_ctg1_284	B2	17.13360426	Bcer	E	Bcer	FALSE	NA	NA	NA
Bc_ctg1_2840	B2	23.66069159	Bcer	Q	Bcer	TRUE	Bc_Ga0417192_01_rn20	Core biosynthesis	SMCOG1002
Bc_ctg1_2841	B2	23.07791594	Bcer	S	Bcer	FALSE	Bc_Ga0417192_01_rn20	Other	NA
Bc_ctg1_2842	B2	81.12237117	Bcer	P	Bcer	FALSE	Bc_Ga0417192_01_rn20	Transport	SMCOG1044
Bc_ctg1_2843	B2	68.4178619	Bcer	P	Bcer	FALSE	Bc_Ga0417192_01_rn20	Other	NA
Bc_ctg1_2844	B2	90.91300218	Bcer	P	Bcer	FALSE	Bc_Ga0417192_01_rn20	Transport	SMCOG1000
Bc_ctg1_2845	B2	4.895315502	Bcer	S	Bcer	FALSE	Bc_Ga0417192_01_rn20	Transport	SMCOG1000
Bc_ctg1_2846	B2	1.515216703	Bcer	G	Bcer	FALSE	Bc_Ga0417192_01_rn20	Other	NA
Bc_ctg1_2847	B2	2.680768013	Bcer	S	Bcer	FALSE	Bc_Ga0417192_01_rn20	Other	NA
Bc_ctg1_2848	B2	11.07273744	Bcer	CO	Bcer	FALSE	Bc_Ga0417192_01_rn20	Other	NA
Bc_ctg1_2849	B2	3.380098799	Bcer	NA	Bcer	FALSE	Bc_Ga0417192_01_rn20	Other	NA
Bc_ctg1_285	B2	9.207855349	Bcer	S	Bcer	FALSE	NA	NA	NA
Bc_ctg1_2852	B2	10.95618231	Bcer	K	Bcer	FALSE	Bc_Ga0417192_01_rn20	Regulation	SMCOG1057
Bc_ctg1_2853	B2	0.932441048	Bcer	G	Bcer	FALSE	Bc_Ga0417192_01_rn20	Transport	SMCOG1020
Bc_ctg1_2854	B2	1.048996179	Bcer	K	Bcer	FALSE	Bc_Ga0417192_01_rn20	Other	NA
Bc_ctg1_2855	B2	28.55600709	Bcer	S	Bcer	FALSE	Bc_Ga0417192_01_rn20	Transport	SMCOG1106
Bc_ctg1_2856	B2	24.12691212	Bcer	S	Bcer	FALSE	Bc_Ga0417192_01_rn20	Other	NA
Bc_ctg1_2857	B2	8.741634825	Bcer	S	Bcer	FALSE	Bc_Ga0417192_01_rn20	Other	NA
Bc_ctg1_2858	B2	171.9188182	Bcer	J	Bcer	FALSE	Bc_Ga0417192_01_rn20	Other	SMCOG1162
Bc_ctg1_2859	B2	0.233110262	Bcer	NU	Bcer	FALSE	Bc_Ga0417192_01_rn20	Other	NA
Bc_ctg1_286	B2	0.466220524	Bcer	S	Bcer	FALSE	NA	NA	NA
Bc_ctg1_2860	B2	1.048996179	Bcer	GK	Bcer	FALSE	Bc_Ga0417192_01_rn20	Other	SMCOG1108
Bc_ctg1_2861	B2	567.623488	Bcer	T	Bcer	FALSE	Bc_Ga0417192_01_rn20	Other	SMCOG1296

Bc_ctg1_2862	B2	796.4212101	Bcer	T	Bcer	FALSE	Bc_Ga0417192_01_rn20	Other	SMCOG1296
Bc_ctg1_2863	B2	153.7362178	Bcer	T	Bcer	FALSE	NA	NA	NA
Bc_ctg1_2864	B2	343.487971	Bcer	P	Bcer	FALSE	NA	NA	NA
Bc_ctg1_2865	B2	243.7167789	Bcer	O	Bcer	FALSE	NA	NA	NA
Bc_ctg1_2866	B2	210.848232	Bcer	P	Bcer	FALSE	NA	NA	NA
Bc_ctg1_2867	B2	809.5919399	Bcer	P	Bcer	FALSE	NA	NA	NA
Bc_ctg1_2868	B2	12.58795415	Bcer	S	Bcer	FALSE	NA	NA	NA
Bc_ctg1_2869	B2	25.99179421	Bcer	T	Bcer	FALSE	NA	NA	NA
Bc_ctg1_287	B2	0.582775655	Bcer	S	Bcer	FALSE	NA	NA	NA
Bc_ctg1_2870	B2	66.20331441	Bcer	NA	Bcer	FALSE	NA	NA	NA
Bc_ctg1_2871	B2	121.1007811	Bcer	S	Bcer	FALSE	NA	NA	NA
Bc_ctg1_2872	B2	68.4178619	Bcer	P	Bcer	FALSE	NA	NA	NA
Bc_ctg1_2873	B2	0.815885917	Bcer	EG	Bcer	FALSE	NA	NA	NA
Bc_ctg1_2874	B2	1.864882096	Bcer	EG	Bcer	FALSE	NA	NA	NA
Bc_ctg1_2875	B2	31.23677511	Bcer	S	Bcer	FALSE	NA	NA	NA
Bc_ctg1_2876	B2	5.82775655	Bcer	U	Bcer	FALSE	NA	NA	NA
Bc_ctg1_2877	B2	983.8418607	Bcer	S	Bcer	FALSE	NA	NA	NA
Bc_ctg1_2878	B2	17.59982478	Bcer	S	Bcer	FALSE	NA	NA	NA
Bc_ctg1_2879	B2	2.214547489	Bcer	S	Bcer	FALSE	NA	NA	NA
Bc_ctg1_288	B2	1.048996179	Bcer	S	Bcer	FALSE	NA	NA	NA
Bc_ctg1_2880	B2	0	Bcer	S	Bcer	FALSE	NA	NA	NA
Bc_ctg1_2881	B2	0	Bcer	S	Bcer	FALSE	NA	NA	NA
Bc_ctg1_2882	B2	0.466220524	Bcer	P	Bcer	FALSE	NA	NA	NA
Bc_ctg1_2883	B2	3.729764192	Bcer	S	Bcer	FALSE	NA	NA	NA
Bc_ctg1_2884	B2	97.32353438	Bcer	S	Bcer	FALSE	NA	NA	NA
Bc_ctg1_2885	B2	76.57672107	Bcer	NA	Bcer	FALSE	NA	NA	NA
Bc_ctg1_2886	B2	961.2301653	Bcer	C	Bcer	FALSE	NA	NA	NA
Bc_ctg1_2887	B2	13.28728493	Bcer	G	Bcer	FALSE	NA	NA	NA
Bc_ctg1_2888	B2	45.68961135	Bcer	L	Bcer	FALSE	NA	NA	NA
Bc_ctg1_2889	B2	144.6449176	Bcer	J	Bcer	FALSE	NA	NA	NA
Bc_ctg1_2890	B2	22.72825054	Bcer	J	Bcer	FALSE	NA	NA	NA
Bc_ctg1_2891	B2	32.75199181	Bcer	NA	Bcer	FALSE	NA	NA	NA
Bc_ctg1_2892	B2	20.16403766	Bcer	KT	Bcer	FALSE	NA	NA	NA
Bc_ctg1_2893	B2	0	Bcer	I	Bcer	FALSE	NA	NA	NA
Bc_ctg1_2894	B2	2.913878275	Bcer	E	Bcer	FALSE	Bc_Ga0417192_01_rn21	Ancillary biosynthesis	SMCOG1038
Bc_ctg1_2895	B2	5.82775655	Bcer	E	Bcer	FALSE	Bc_Ga0417192_01_rn21	Ancillary biosynthesis	SMCOG1247
Bc_ctg1_2896	B2	0	Bcer	K	Bcer	FALSE	Bc_Ga0417192_01_rn21	Other	NA
Bc_ctg1_2897	B2	0	Bcer	P	Bcer	FALSE	Bc_Ga0417192_01_rn21	Other	NA
Bc_ctg1_2898	B2	0.116555131	Bcer	S	Bcer	FALSE	Bc_Ga0417192_01_rn21	Other	NA
Bc_ctg1_2899	B2	8.741634825	Bcer	E	Bcer	FALSE	Bc_Ga0417192_01_rn21	Other	NA
Bc_ctg1_29	B2	5.594646288	Bcer	S	Bcer	FALSE	NA	NA	NA
Bc_ctg1_290	B2	404.6794148	Bcer	E	Bcer	FALSE	NA	NA	NA
Bc_ctg1_2900	B2	135.4370622	Bcer	G	Bcer	FALSE	Bc_Ga0417192_01_rn21	Ancillary biosynthesis	SMCOG1145
Bc_ctg1_2901	B2	220.8719732	Bcer	V	Bcer	FALSE	Bc_Ga0417192_01_rn21	Ancillary biosynthesis	SMCOG1053
Bc_ctg1_2902	B2	1.048996179	Bcer	NA	Bcer	FALSE	Bc_Ga0417192_01_rn21	Other	NA
Bc_ctg1_2903	B2	0.116555131	Bcer	NA	Bcer	FALSE	Bc_Ga0417192_01_rn21	Other	NA
Bc_ctg1_2904	B2	0.582775655	Bcer	M	Bcer	FALSE	Bc_Ga0417192_01_rn21	Core biosynthesis	SMCOG1123
Bc_ctg1_2905	B2	1.16555131	Bcer	M	Bcer	FALSE	Bc_Ga0417192_01_rn21	Ancillary biosynthesis	SMCOG1152
Bc_ctg1_2906	B2	2.214547489	Bcer	M	Bcer	FALSE	Bc_Ga0417192_01_rn21	Core biosynthesis	SMCOG1010
Bc_ctg1_2907	B2	8.741634825	Bcer	M	Bcer	FALSE	Bc_Ga0417192_01_rn21	Core biosynthesis	SMCOG1123
Bc_ctg1_2908	B2	0	Bcer	NA	Bcer	FALSE	Bc_Ga0417192_01_rn21	Other	NA
Bc_ctg1_2909	B2	34784.59674	Bcer	C	Bcer	FALSE	Bc_Ga0417192_01_rn21	Other	NA
Bc_ctg1_291	B2	8.275414301	Bcer	S	Bcer	FALSE	NA	NA	NA
Bc_ctg1_2910	B2	9944.716887	Bcer	C	Bcer	FALSE	Bc_Ga0417192_01_rn21	Ancillary biosynthesis	None
Bc_ctg1_2911	B2	199.6589394	Bcer	M	Bcer	FALSE	Bc_Ga0417192_01_rn21	Core biosynthesis	None
Bc_ctg1_2913	B2	32.05266102	Bcer	J	Bcer	FALSE	Bc_Ga0417192_01_rn21	Other	NA
Bc_ctg1_2914	B2	19.11504148	Bcer	Q	Bcer	TRUE	Bc_Ga0417192_01_rn21	Other	NA
Bc_ctg1_2915	B2	21.91236463	Bcer	S	Bcer	FALSE	Bc_Ga0417192_01_rn21	Ancillary biosynthesis	SMCOG1010
Bc_ctg1_2916	B2	80.30648526	Bcer	S	Bcer	FALSE	Bc_Ga0417192_01_rn21	Other	NA
Bc_ctg1_2917	B2	30.0712238	Bcer	S	Bcer	FALSE	Bc_Ga0417192_01_rn21	Other	NA
Bc_ctg1_2919	B2	0	Bcer	NA	Bcer	FALSE	Bc_Ga0417192_01_rn21	Other	NA
Bc_ctg1_292	B2	7.692638646	Bcer	K	Bcer	FALSE	NA	NA	NA

Bc_ctg1_2920	B2	5.594646288	Bcer	S	Bcer	FALSE	Bc_Ga0417192_01_rn21	Other	NA
Bc_ctg1_2921	B2	52.56636408	Bcer	S	Bcer	FALSE	Bc_Ga0417192_01_rn21	Other	NA
Bc_ctg1_2922	B2	15.0356119	Bcer	L	Bcer	FALSE	Bc_Ga0417192_01_rn21	Other	NA
Bc_ctg1_2924	B2	0.932441048	Bcer	S	Bcer	FALSE	Bc_Ga0417192_01_rn21	Other	NA
Bc_ctg1_2925	B2	0.233110262	Bcer	S	Bcer	FALSE	Bc_Ga0417192_01_rn21	Other	NA
Bc_ctg1_2926	B2	119.0027887	Bcer	J	Bcer	FALSE	Bc_Ga0417192_01_rn21	Other	NA
Bc_ctg1_2927	B2	28.78911736	Bcer	V	Bcer	FALSE	NA	NA	NA
Bc_ctg1_2928	B2	74.8283941	Bcer	S	Bcer	FALSE	NA	NA	NA
Bc_ctg1_2929	B2	158.7480884	Bcer	E	Bcer	FALSE	NA	NA	NA
Bc_ctg1_293	B2	0	Bcer	NA	Bcer	FALSE	NA	NA	NA
Bc_ctg1_2930	B2	369.5963204	Bcer	H	Bcer	FALSE	NA	NA	NA
Bc_ctg1_2931	B2	10.02374127	Bcer	S	Bcer	FALSE	NA	NA	NA
Bc_ctg1_2932	B2	4.778760371	Bcer	S	Bcer	FALSE	NA	NA	NA
Bc_ctg1_2933	B2	11.07273744	Bcer	S	Bcer	FALSE	NA	NA	NA
Bc_ctg1_2934	B2	7.459528384	Bcer	P	Bcer	FALSE	NA	NA	NA
Bc_ctg1_2935	B2	25.99179421	Bcer	O	Bcer	FALSE	NA	NA	NA
Bc_ctg1_2936	B2	651.4266271	Bcer	P	Bcer	FALSE	NA	NA	NA
Bc_ctg1_2937	B2	18.0660453	Bcer	K	Bcer	FALSE	NA	NA	NA
Bc_ctg1_2938	B2	9.907186135	Bcer	S	Bcer	FALSE	NA	NA	NA
Bc_ctg1_294	B2	15.26872216	Bcer	K	Bcer	FALSE	NA	NA	NA
Bc_ctg1_2940	B2	0	Bcer	NA	Bcer	FALSE	NA	NA	NA
Bc_ctg1_2942	B2	222.1540797	Bcer	K	Bcer	FALSE	NA	NA	NA
Bc_ctg1_2943	B2	0.116555131	Bcer	F	Bcer	FALSE	NA	NA	NA
Bc_ctg1_2944	B2	37.88041757	Bcer	M	Bcer	FALSE	NA	NA	NA
Bc_ctg1_2945	B2	73.42973253	Bcer	O	Bcer	FALSE	NA	NA	NA
Bc_ctg1_2946	B2	19.11504148	Bcer	C	Bcer	FALSE	NA	NA	NA
Bc_ctg1_2947	B2	20.86336845	Bcer	S	Bcer	FALSE	Bc_Ga0417192_01_rn22	Other	NA
Bc_ctg1_2948	B2	155.717655	Bcer	J	Bcer	FALSE	Bc_Ga0417192_01_rn22	Other	NA
Bc_ctg1_2949	B2	44.29094978	Bcer	T	Bcer	FALSE	Bc_Ga0417192_01_rn22	Ancillary biosynthesis	SMCOG1025
Bc_ctg1_295	B2	19.9309274	Bcer	K	Bcer	FALSE	NA	NA	NA
Bc_ctg1_2951	B2	11.6555131	Bcer	T	Bcer	FALSE	Bc_Ga0417192_01_rn22	Other	NA
Bc_ctg1_2952	B2	8.508524563	Bcer	S	Bcer	FALSE	Bc_Ga0417192_01_rn22	Other	NA
Bc_ctg1_2953	B2	415.519042	Bcer	E	Bcer	FALSE	Bc_Ga0417192_01_rn22	Transport	SMCOG1202
Bc_ctg1_2954	B2	31.12021998	Bcer	NA	Bcer	FALSE	Bc_Ga0417192_01_rn22	Other	NA
Bc_ctg1_2955	B2	83.57002892	Bcer	H	Bcer	FALSE	Bc_Ga0417192_01_rn22	Ancillary biosynthesis	SMCOG1012
Bc_ctg1_2956	B2	117.6041272	Bcer	T	Bcer	FALSE	Bc_Ga0417192_01_rn22	Regulation	SMCOG1048
Bc_ctg1_2957	B2	56.64579366	Bcer	KT	Bcer	FALSE	Bc_Ga0417192_01_rn22	Regulation	SMCOG1016
Bc_ctg1_2958	B2	2298.000963	Bcer	V	Bcer	FALSE	Bc_Ga0417192_01_rn22	Transport	SMCOG1000
Bc_ctg1_2959	B2	3319.839796	Bcer	V	Bcer	FALSE	Bc_Ga0417192_01_rn22	Other	NA
Bc_ctg1_296	B2	26.34145961	Bcer	S	Bcer	FALSE	NA	NA	NA
Bc_ctg1_2960	B2	2252.078241	Bcer	V	Bcer	FALSE	Bc_Ga0417192_01_rn22	Other	NA
Bc_ctg1_2961	B2	14.91905677	Bcer	NA	Bcer	FALSE	Bc_Ga0417192_01_rn22	Other	NA
Bc_ctg1_2962	B2	481.7223564	Bcer	Q	Bcer	TRUE	Bc_Ga0417192_01_rn22	Ancillary biosynthesis	SMCOG1004
Bc_ctg1_2963	B2	2599.762197	Bcer	Q	Bcer	TRUE	Bc_Ga0417192_01_rn22	Core biosynthesis	SMCOG1002
Bc_ctg1_2964	B2	3055.609314	Bcer	Q	Bcer	TRUE	Bc_Ga0417192_01_rn22	Core biosynthesis	SMCOG1209
Bc_ctg1_2965	B2	305.9572189	Bcer	I	Bcer	FALSE	Bc_Ga0417192_01_rn22	Core biosynthesis	SMCOG1021
Bc_ctg1_2966	B2	1496.567882	Bcer	Q	Bcer	TRUE	Bc_Ga0417192_01_rn22	Core biosynthesis	SMCOG1022
Bc_ctg1_2967	B2	1139.442961	Bcer	Q	Bcer	TRUE	Bc_Ga0417192_01_rn22	Core biosynthesis	SMCOG1022
Bc_ctg1_2968	B2	263.4145961	Bcer	Q	Bcer	TRUE	Bc_Ga0417192_01_rn22	Ancillary biosynthesis	SMCOG1222
Bc_ctg1_2969	B2	1550.882573	Bcer	Q	Bcer	TRUE	Bc_Ga0417192_01_rn22	Core biosynthesis	SMCOG1022
Bc_ctg1_297	B2	0	Bcer	K	Bcer	FALSE	NA	NA	NA
Bc_ctg1_2970	B2	352.4627161	Bcer	Q	Bcer	TRUE	Bc_Ga0417192_01_rn22	Ancillary biosynthesis	SMCOG1042
Bc_ctg1_2971	B2	6.876752729	Bcer	M	Bcer	FALSE	Bc_Ga0417192_01_rn22	Other	NA
Bc_ctg1_2972	B2	0.932441048	Bcer	K	Bcer	FALSE	Bc_Ga0417192_01_rn22	Other	NA
Bc_ctg1_2973	B2	46.15583187	Bcer	M	Bcer	FALSE	Bc_Ga0417192_01_rn22	Other	NA
Bc_ctg1_2974	B2	274.1376681	Bcer	S	Bcer	FALSE	Bc_Ga0417192_01_rn22	Other	NA
Bc_ctg1_2975	B2	53.73191539	Bcer	OU	Bcer	FALSE	Bc_Ga0417192_01_rn22	Other	NA
Bc_ctg1_2976	B2	166.3241719	Bcer	S	Bcer	FALSE	Bc_Ga0417192_01_rn22	Other	NA
Bc_ctg1_2977	B2	152.104446	Bcer	NT	Bcer	FALSE	Bc_Ga0417192_01_rn22	Other	SMCOG1061
Bc_ctg1_2978	B2	18.29915557	Bcer	T	Bcer	FALSE	Bc_Ga0417192_01_rn22	Other	NA
Bc_ctg1_2979	B2	11.6555131	Bcer	KT	Bcer	FALSE	Bc_Ga0417192_01_rn22	Regulation	SMCOG1008
Bc_ctg1_298	B2	4.195984716	Bcer	C	Bcer	FALSE	NA	NA	NA

Bc_ctg1_2980	B2	10.95618231	Bcer	C	Bcer	FALSE	Bc_Ga0417192_01_rn22	Other	NA
Bc_ctg1_2981	B2	0	Bcer	I	Bcer	FALSE	Bc_Ga0417192_01_rn22	Other	NA
Bc_ctg1_2982	B2	1.864882096	Bcer	C	Bcer	FALSE	Bc_Ga0417192_01_rn22	Other	NA
Bc_ctg1_2983	B2	62.93977074	Bcer	S	Bcer	FALSE	NA	NA	NA
Bc_ctg1_2984	B2	0	Bcer	S	Bcer	FALSE	NA	NA	NA
Bc_ctg1_2985	B2	0.466220524	Bcer	K	Bcer	FALSE	NA	NA	NA
Bc_ctg1_2986	B2	0.699330786	Bcer	P	Bcer	FALSE	NA	NA	NA
Bc_ctg1_2987	B2	0	Bcer	G	Bcer	FALSE	NA	NA	NA
Bc_ctg1_2988	B2	0.349665393	Bcer	P	Bcer	FALSE	NA	NA	NA
Bc_ctg1_2989	B2	12.35484389	Bcer	G	Bcer	FALSE	NA	NA	NA
Bc_ctg1_299	B2	0.815885917	Bcer	S	Bcer	FALSE	NA	NA	NA
Bc_ctg1_2990	B2	4.66220524	Bcer	T	Bcer	FALSE	NA	NA	NA
Bc_ctg1_2991	B2	2.447657751	Bcer	T	Bcer	FALSE	NA	NA	NA
Bc_ctg1_2992	B2	12.70450928	Bcer	T	Bcer	FALSE	NA	NA	NA
Bc_ctg1_2993	B2	1.282106441	Bcer	NA	Bcer	FALSE	NA	NA	NA
Bc_ctg1_2994	B2	2.447657751	Bcer	M	Bcer	FALSE	NA	NA	NA
Bc_ctg1_2995	B2	51.05114738	Bcer	NT	Bcer	FALSE	NA	NA	NA
Bc_ctg1_2996	B2	11.30584771	Bcer	T	Bcer	FALSE	NA	NA	NA
Bc_ctg1_2997	B2	15.73494268	Bcer	T	Bcer	FALSE	NA	NA	NA
Bc_ctg1_2998	B2	0.699330786	Bcer	C	Bcer	FALSE	NA	NA	NA
Bc_ctg1_2999	B2	2.447657751	Bcer	C	Bcer	FALSE	NA	NA	NA
Bc_ctg1_3	B2	16.43427347	Bcer	L	Bcer	FALSE	NA	NA	NA
Bc_ctg1_30	B2	79.02437882	Bcer	I	Bcer	FALSE	NA	NA	NA
Bc_ctg1_300	B2	7.692638646	Bcer	O	Bcer	FALSE	NA	NA	NA
Bc_ctg1_3000	B2	0	Bcer	NA	Bcer	FALSE	NA	NA	NA
Bc_ctg1_3001	B2	0.116555131	Bcer	EG	Bcer	FALSE	NA	NA	NA
Bc_ctg1_3002	B2	4.66220524	Bcer	K	Bcer	FALSE	NA	NA	NA
Bc_ctg1_3003	B2	29.83811354	Bcer	K	Bcer	FALSE	NA	NA	NA
Bc_ctg1_3004	B2	22.61169541	Bcer	T	Bcer	FALSE	NA	NA	NA
Bc_ctg1_3005	B2	28.08978657	Bcer	KT	Bcer	FALSE	NA	NA	NA
Bc_ctg1_3006	B2	0	Bcer	NA	Bcer	FALSE	NA	NA	NA
Bc_ctg1_3007	B2	5.244980895	Bcer	M	Bcer	FALSE	NA	NA	NA
Bc_ctg1_3008	B2	33.80098799	Bcer	C	Bcer	FALSE	NA	NA	NA
Bc_ctg1_3009	B2	1200.517849	Bcer	C	Bcer	FALSE	NA	NA	NA
Bc_ctg1_301	B2	20.97992358	Bcer	E	Bcer	FALSE	Bc_Ga0417192_01_rn03	Other	NA
Bc_ctg1_3010	B2	220.2891976	Bcer	S	Bcer	FALSE	NA	NA	NA
Bc_ctg1_3011	B2	81.35548144	Bcer	C	Bcer	FALSE	NA	NA	NA
Bc_ctg1_3012	B2	1682.007095	Bcer	E	Bcer	FALSE	NA	NA	NA
Bc_ctg1_3013	B2	581.9597691	Bcer	E	Bcer	FALSE	NA	NA	NA
Bc_ctg1_3014	B2	97.67319978	Bcer	K	Bcer	FALSE	NA	NA	NA
Bc_ctg1_3015	B2	1691.564616	Bcer	P	Bcer	FALSE	NA	NA	NA
Bc_ctg1_3016	B2	126.4623171	Bcer	H	Bcer	FALSE	NA	NA	NA
Bc_ctg1_3017	B2	371.3446474	Bcer	K	Bcer	FALSE	NA	NA	NA
Bc_ctg1_3019	B2	21.32958897	Bcer	E	Bcer	FALSE	NA	NA	NA
Bc_ctg1_302	B2	36.24864574	Bcer	S	Bcer	FALSE	Bc_Ga0417192_01_rn03	Other	NA
Bc_ctg1_3020	B2	11.30584771	Bcer	S	Bcer	FALSE	NA	NA	NA
Bc_ctg1_3021	B2	17.01704913	Bcer	NA	Bcer	FALSE	NA	NA	NA
Bc_ctg1_3022	B2	2.447657751	Bcer	NA	Bcer	FALSE	NA	NA	NA
Bc_ctg1_3023	B2	10.02374127	Bcer	V	Bcer	FALSE	NA	NA	NA
Bc_ctg1_3024	B2	19.69781714	Bcer	K	Bcer	FALSE	NA	NA	NA
Bc_ctg1_3025	B2	0.233110262	Bcer	F	Bcer	FALSE	NA	NA	NA
Bc_ctg1_3026	B2	2.097992358	Bcer	E	Bcer	FALSE	NA	NA	NA
Bc_ctg1_3027	B2	12.58795415	Bcer	S	Bcer	FALSE	NA	NA	NA
Bc_ctg1_3028	B2	166.3241719	Bcer	E	Bcer	FALSE	NA	NA	NA
Bc_ctg1_3029	B2	3478.70444	Bcer	C	Bcer	FALSE	NA	NA	NA
Bc_ctg1_303	B2	34.9665393	Bcer	S	Bcer	FALSE	Bc_Ga0417192_01_rn03	Ancillary biosynthesis	None
Bc_ctg1_3030	B2	224.6017374	Bcer	K	Bcer	FALSE	Bc_Ga0417192_01_rn23	Regulation	SMCOG1058
Bc_ctg1_3031	B2	0	Bcer	S	Bcer	FALSE	Bc_Ga0417192_01_rn23	Other	NA
Bc_ctg1_3032	B2	5255.937077	Bcer	E	Bcer	FALSE	Bc_Ga0417192_01_rn23	Transport	SMCOG1202
Bc_ctg1_3033	B2	215.5104372	Bcer	P	Bcer	FALSE	Bc_Ga0417192_01_rn23	Transport	SMCOG1033
Bc_ctg1_3034	B2	101.1698537	Bcer	P	Bcer	FALSE	Bc_Ga0417192_01_rn23	Transport	SMCOG1011
Bc_ctg1_3035	B2	78.55815829	Bcer	P	Bcer	FALSE	Bc_Ga0417192_01_rn23	Transport	SMCOG1011

Bc_ctg1_3036	B2	134.388066	Bcer	HP	Bcer	FALSE	Bc_Ga0417192_01_rn23	Transport	SMCOG1000
Bc_ctg1_3037	B2	23.66069159	Bcer	Q	Bcer	TRUE	Bc_Ga0417192_01_rn23	Ancillary biosynthesis	SMCOG1248
Bc_ctg1_3038	B2	1802.758211	Bcer	H	Bcer	FALSE	Bc_Ga0417192_01_rn23	Ancillary biosynthesis	SMCOG1109
Bc_ctg1_3039	B2	1635.385043	Bcer	GM	Bcer	FALSE	Bc_Ga0417192_01_rn23	Core biosynthesis	SMCOG1010
Bc_ctg1_304	B2	5.128425764	Bcer	S	Bcer	FALSE	Bc_Ga0417192_01_rn03	Other	NA
Bc_ctg1_3040	B2	7.459528384	Bcer	F	Bcer	FALSE	Bc_Ga0417192_01_rn23	Other	NA
Bc_ctg1_3041	B2	0.815885917	Bcer	F	Bcer	FALSE	Bc_Ga0417192_01_rn23	Other	NA
Bc_ctg1_3042	B2	105.7155038	Bcer	P	Bcer	FALSE	Bc_Ga0417192_01_rn23	Other	NA
Bc_ctg1_3043	B2	34.15065338	Bcer	S	Bcer	FALSE	Bc_Ga0417192_01_rn23	Other	NA
Bc_ctg1_3044	B2	46.50549727	Bcer	I	Bcer	FALSE	Bc_Ga0417192_01_rn23	Other	NA
Bc_ctg1_3046	B2	45.33994596	Bcer	T	Bcer	FALSE	Bc_Ga0417192_01_rn23	Ancillary biosynthesis	SMCOG1025
Bc_ctg1_3047	B2	19.58126201	Bcer	E	Bcer	FALSE	Bc_Ga0417192_01_rn23	Ancillary biosynthesis	SMCOG1038
Bc_ctg1_3048	B2	25.40901856	Bcer	K	Bcer	FALSE	Bc_Ga0417192_01_rn23	Regulation	SMCOG1136
Bc_ctg1_3049	B2	2816.08852	Bcer	G	Bcer	FALSE	Bc_Ga0417192_01_rn23	Other	NA
Bc_ctg1_3050	B2	2916.675598	Bcer	G	Bcer	FALSE	Bc_Ga0417192_01_rn23	Core biosynthesis	SMCOG1257
Bc_ctg1_3051	B2	0.699330786	Bcer	S	Bcer	FALSE	Bc_Ga0417192_01_rn23	Other	NA
Bc_ctg1_3052	B2	0.233110262	Bcer	E	Bcer	FALSE	Bc_Ga0417192_01_rn23	Other	NA
Bc_ctg1_3053	B2	0	Bcer	EG	Bcer	FALSE	Bc_Ga0417192_01_rn23	Other	NA
Bc_ctg1_3054	B2	61.89077456	Bcer	S	Bcer	FALSE	Bc_Ga0417192_01_rn23	Ancillary biosynthesis	None
Bc_ctg1_3055	B2	6.876752729	Bcer	E	Bcer	FALSE	Bc_Ga0417192_01_rn23	Ancillary biosynthesis	SMCOG1153
Bc_ctg1_3056	B2	0	Bcer	E	Bcer	FALSE	Bc_Ga0417192_01_rn23	Transport	SMCOG1000
Bc_ctg1_3057	B2	0.349665393	Bcer	ET	Bcer	FALSE	Bc_Ga0417192_01_rn23	Ancillary biosynthesis	SMCOG1091
Bc_ctg1_3058	B2	0.349665393	Bcer	P	Bcer	FALSE	Bc_Ga0417192_01_rn23	Transport	SMCOG1096
Bc_ctg1_3059	B2	0.582775655	Bcer	P	Bcer	FALSE	Bc_Ga0417192_01_rn23	Transport	SMCOG1096
Bc_ctg1_306	B2	7.226418122	Bcer	S	Bcer	FALSE	Bc_Ga0417192_01_rn03	Other	NA
Bc_ctg1_3060	B2	106.4148346	Bcer	E	Bcer	FALSE	NA	NA	NA
Bc_ctg1_3061	B2	35.31620469	Bcer	O	Bcer	FALSE	NA	NA	NA
Bc_ctg1_3062	B2	4.312539847	Bcer	FG	Bcer	FALSE	NA	NA	NA
Bc_ctg1_3063	B2	2.680768013	Bcer	K	Bcer	FALSE	NA	NA	NA
Bc_ctg1_3064	B2	9.207855349	Bcer	NA	Bcer	FALSE	NA	NA	NA
Bc_ctg1_3065	B2	2.797323144	Bcer	K	Bcer	FALSE	NA	NA	NA
Bc_ctg1_3066	B2	0.116555131	Bcer	H	Bcer	FALSE	NA	NA	NA
Bc_ctg1_3067	B2	83.57002892	Bcer	T	Bcer	FALSE	NA	NA	NA
Bc_ctg1_3068	B2	32.63543668	Bcer	KT	Bcer	FALSE	NA	NA	NA
Bc_ctg1_3069	B2	1048.296848	Bcer	NA	Bcer	FALSE	NA	NA	NA
Bc_ctg1_3070	B2	14.45283624	Bcer	P	Bcer	FALSE	NA	NA	NA
Bc_ctg1_3071	B2	14.45283624	Bcer	T	Bcer	FALSE	NA	NA	NA
Bc_ctg1_3073	B2	153.969328	Bcer	E	Bcer	FALSE	NA	NA	NA
Bc_ctg1_3074	B2	48.48693449	Bcer	EP	Bcer	FALSE	NA	NA	NA
Bc_ctg1_3075	B2	38.23008297	Bcer	P	Bcer	FALSE	NA	NA	NA
Bc_ctg1_3076	B2	31.35333024	Bcer	P	Bcer	FALSE	NA	NA	NA
Bc_ctg1_3077	B2	39.62874454	Bcer	P	Bcer	FALSE	NA	NA	NA
Bc_ctg1_3078	B2	28.67256223	Bcer	G	Bcer	FALSE	NA	NA	NA
Bc_ctg1_3079	B2	48.95315502	Bcer	K	Bcer	FALSE	NA	NA	NA
Bc_ctg1_308	B2	18.64882096	Bcer	IQ	Bcer	TRUE	Bc_Ga0417192_01_rn03	Ancillary biosynthesis	SMCOG1002
Bc_ctg1_3080	B2	436.2658553	Bcer	P	Bcer	FALSE	NA	NA	NA
Bc_ctg1_3081	B2	54.54780131	Bcer	K	Bcer	FALSE	NA	NA	NA
Bc_ctg1_3082	B2	69.00063755	Bcer	H	Bcer	FALSE	NA	NA	NA
Bc_ctg1_3083	B2	12.00517849	Bcer	G	Bcer	FALSE	NA	NA	NA
Bc_ctg1_3084	B2	108.6293821	Bcer	G	Bcer	FALSE	NA	NA	NA
Bc_ctg1_3085	B2	189.2855327	Bcer	G	Bcer	FALSE	NA	NA	NA
Bc_ctg1_3086	B2	424.1441217	Bcer	G	Bcer	FALSE	NA	NA	NA
Bc_ctg1_3087	B2	490.1143258	Bcer	G	Bcer	FALSE	NA	NA	NA
Bc_ctg1_3088	B2	917.0557707	Bcer	V	Bcer	FALSE	NA	NA	NA
Bc_ctg1_3089	B2	0	Bcer	NA	Bcer	FALSE	NA	NA	NA
Bc_ctg1_309	B2	8.15885917	Bcer	S	Bcer	FALSE	Bc_Ga0417192_01_rn03	Other	NA
Bc_ctg1_3090	B2	0	Bcer	NA	Bcer	FALSE	NA	NA	NA
Bc_ctg1_3091	B2	42.07640229	Bcer	EGP	Bcer	FALSE	NA	NA	NA
Bc_ctg1_3092	B2	3890.610273	Bcer	E	Bcer	FALSE	NA	NA	NA
Bc_ctg1_3093	B2	4.895315502	Bcer	P	Bcer	FALSE	NA	NA	NA
Bc_ctg1_3094	B2	637.9062319	Bcer	M	Bcer	FALSE	NA	NA	NA
Bc_ctg1_3095	B2	111.6598155	Bcer	S	Bcer	FALSE	NA	NA	NA

Bc_ctg1_3096	B2	3.030433406	Bcer	E	Bcer	FALSE	NA	NA	NA
Bc_ctg1_3097	B2	4.079429585	Bcer	C	Bcer	FALSE	NA	NA	NA
Bc_ctg1_3098	B2	3.846319323	Bcer	V	Bcer	FALSE	NA	NA	NA
Bc_ctg1_3099	B2	10.72307205	Bcer	NA	Bcer	FALSE	NA	NA	NA
Bc_ctg1_31	B2	20.63025819	Bcer	S	Bcer	FALSE	NA	NA	NA
Bc_ctg1_310	B2	2.564212882	Bcer	K	Bcer	FALSE	Bc_Ga0417192_01_rn03	Regulation	SMCOG1167
Bc_ctg1_3100	B2	0.349665393	Bcer	NA	Bcer	FALSE	NA	NA	NA
Bc_ctg1_3101	B2	9.091300218	Bcer	V	Bcer	FALSE	NA	NA	NA
Bc_ctg1_3102	B2	121.8001119	Bcer	NT	Bcer	FALSE	NA	NA	NA
Bc_ctg1_3103	B2	322.0418269	Bcer	S	Bcer	FALSE	NA	NA	NA
Bc_ctg1_3104	B2	0.466220524	Bcer	K	Bcer	FALSE	NA	NA	NA
Bc_ctg1_3105	B2	3.030433406	Bcer	NA	Bcer	FALSE	NA	NA	NA
Bc_ctg1_3106	B2	10.83962718	Bcer	M	Bcer	FALSE	NA	NA	NA
Bc_ctg1_3107	B2	1.16555131	Bcer	L	Bcer	FALSE	NA	NA	NA
Bc_ctg1_3108	B2	190.2179738	Bcer	E	Bcer	FALSE	NA	NA	NA
Bc_ctg1_3109	B2	1.864882096	Bcer	E	Bcer	FALSE	NA	NA	NA
Bc_ctg1_311	B2	0	Bcer	NA	Bcer	FALSE	Bc_Ga0417192_01_rn03	Other	NA
Bc_ctg1_3110	B2	68.18475163	Bcer	S	Bcer	FALSE	NA	NA	NA
Bc_ctg1_3111	B2	126.8119825	Bcer	G	Bcer	FALSE	NA	NA	NA
Bc_ctg1_3112	B2	21.67925437	Bcer	E	Bcer	FALSE	NA	NA	NA
Bc_ctg1_3113	B2	35.43275982	Bcer	S	Bcer	FALSE	NA	NA	NA
Bc_ctg1_3114	B2	0	Bcer	K	Bcer	FALSE	NA	NA	NA
Bc_ctg1_3115	B2	3.030433406	Bcer	NA	Bcer	FALSE	NA	NA	NA
Bc_ctg1_3116	B2	15.73494268	Bcer	K	Bcer	FALSE	NA	NA	NA
Bc_ctg1_3117	B2	3.030433406	Bcer	S	Bcer	FALSE	NA	NA	NA
Bc_ctg1_3118	B2	13.05417467	Bcer	P	Bcer	FALSE	NA	NA	NA
Bc_ctg1_3119	B2	74.24561845	Bcer	C	Bcer	FALSE	NA	NA	NA
Bc_ctg1_312	B2	0.815885917	Bcer	K	Bcer	FALSE	Bc_Ga0417192_01_rn03	Regulation	SMCOG1058
Bc_ctg1_3120	B2	519.602774	Bcer	C	Bcer	FALSE	NA	NA	NA
Bc_ctg1_3121	B2	2289.026218	Bcer	C	Bcer	FALSE	NA	NA	NA
Bc_ctg1_3122	B2	1003.889343	Bcer	C	Bcer	FALSE	NA	NA	NA
Bc_ctg1_3123	B2	45.33994596	Bcer	E	Bcer	FALSE	NA	NA	NA
Bc_ctg1_3124	B2	16.5508286	Bcer	F	Bcer	FALSE	NA	NA	NA
Bc_ctg1_3125	B2	0.349665393	Bcer	E	Bcer	FALSE	NA	NA	NA
Bc_ctg1_3126	B2	0	Bcer	S	Bcer	FALSE	NA	NA	NA
Bc_ctg1_3127	B2	6.760197598	Bcer	E	Bcer	FALSE	NA	NA	NA
Bc_ctg1_3128	B2	0	Bcer	EG	Bcer	FALSE	NA	NA	NA
Bc_ctg1_3129	B2	0	Bcer	E	Bcer	FALSE	NA	NA	NA
Bc_ctg1_3130	B2	0.116555131	Bcer	S	Bcer	FALSE	NA	NA	NA
Bc_ctg1_3131	B2	2560.366563	Bcer	P	Bcer	FALSE	NA	NA	NA
Bc_ctg1_3132	B2	1460.086126	Bcer	P	Bcer	FALSE	NA	NA	NA
Bc_ctg1_3133	B2	74.36217358	Bcer	P	Bcer	FALSE	NA	NA	NA
Bc_ctg1_3134	B2	2.680768013	Bcer	P	Bcer	FALSE	NA	NA	NA
Bc_ctg1_3135	B2	2.680768013	Bcer	P	Bcer	FALSE	NA	NA	NA
Bc_ctg1_3136	B2	4.312539847	Bcer	P	Bcer	FALSE	NA	NA	NA
Bc_ctg1_3137	B2	1.048996179	Bcer	S	Bcer	FALSE	NA	NA	NA
Bc_ctg1_3138	B2	17.94949017	Bcer	V	Bcer	FALSE	NA	NA	NA
Bc_ctg1_314	B2	0.116555131	Bcer	L	Bcer	FALSE	Bc_Ga0417192_01_rn03	Other	SMCOG1026
Bc_ctg1_3140	B2	7.109862991	Bcer	T	Bcer	FALSE	NA	NA	NA
Bc_ctg1_3141	B2	100.1208575	Bcer	S	Bcer	FALSE	NA	NA	NA
Bc_ctg1_3142	B2	64.45498744	Bcer	NA	Bcer	FALSE	NA	NA	NA
Bc_ctg1_3143	B2	459.3437713	Bcer	MT	Bcer	FALSE	NA	NA	NA
Bc_ctg1_3144	B2	1.16555131	Bcer	K	Bcer	FALSE	NA	NA	NA
Bc_ctg1_3145	B2	0.582775655	Bcer	P	Bcer	FALSE	NA	NA	NA
Bc_ctg1_3146	B2	0	Bcer	P	Bcer	FALSE	NA	NA	NA
Bc_ctg1_3147	B2	1.515216703	Bcer	P	Bcer	FALSE	NA	NA	NA
Bc_ctg1_3148	B2	1.981437227	Bcer	H	Bcer	FALSE	NA	NA	NA
Bc_ctg1_3149	B2	0.932441048	Bcer	E	Bcer	FALSE	NA	NA	NA
Bc_ctg1_315	B2	3.49665393	Bcer	M	Bcer	FALSE	Bc_Ga0417192_01_rn03	Core biosynthesis	SMCOG1123
Bc_ctg1_3150	B2	0	Bcer	H	Bcer	FALSE	NA	NA	NA
Bc_ctg1_3151	B2	1.515216703	Bcer	H	Bcer	FALSE	NA	NA	NA
Bc_ctg1_3152	B2	4.429094978	Bcer	H	Bcer	FALSE	NA	NA	NA

Bc_ctg1_3153	B2	1.864882096	Bcer	H	Bcer	FALSE	NA	NA	NA
Bc_ctg1_3154	B2	3.49665393	Bcer	NA	Bcer	FALSE	NA	NA	NA
Bc_ctg1_3155	B2	4.429094978	Bcer	NA	Bcer	FALSE	NA	NA	NA
Bc_ctg1_3157	B2	208.8667947	Bcer	P	Bcer	FALSE	NA	NA	NA
Bc_ctg1_3158	B2	249.3114252	Bcer	P	Bcer	FALSE	NA	NA	NA
Bc_ctg1_3159	B2	52.79947434	Bcer	P	Bcer	FALSE	NA	NA	NA
Bc_ctg1_316	B2	0	Bcer	S	Bcer	FALSE	Bc_Ga0417192_01_rn03	Other	NA
Bc_ctg1_3160	B2	4.312539847	Bcer	T	Bcer	FALSE	NA	NA	NA
Bc_ctg1_3161	B2	0	Bcer	EGP	Bcer	FALSE	NA	NA	NA
Bc_ctg1_3162	B2	3.962874454	Bcer	K	Bcer	FALSE	NA	NA	NA
Bc_ctg1_3163	B2	37.88041757	Bcer	S	Bcer	FALSE	NA	NA	NA
Bc_ctg1_3164	B2	1.282106441	Bcer	NA	Bcer	FALSE	NA	NA	NA
Bc_ctg1_3165	B2	0.582775655	Bcer	V	Bcer	FALSE	NA	NA	NA
Bc_ctg1_3166	B2	2.913878275	Bcer	V	Bcer	FALSE	NA	NA	NA
Bc_ctg1_3167	B2	1.048996179	Bcer	V	Bcer	FALSE	NA	NA	NA
Bc_ctg1_3168	B2	0.349665393	Bcer	NA	Bcer	FALSE	NA	NA	NA
Bc_ctg1_3169	B2	8.391969432	Bcer	S	Bcer	FALSE	NA	NA	NA
Bc_ctg1_317	B2	0	Bcer	K	Bcer	FALSE	Bc_Ga0417192_01_rn03	Other	NA
Bc_ctg1_3170	B2	0.116555131	Bcer	IQ	Bcer	TRUE	NA	NA	NA
Bc_ctg1_3171	B2	0.582775655	Bcer	S	Bcer	FALSE	NA	NA	NA
Bc_ctg1_3172	B2	0	Bcer	NA	Bcer	FALSE	NA	NA	NA
Bc_ctg1_3173	B2	9.32441048	Bcer	S	Bcer	FALSE	NA	NA	NA
Bc_ctg1_3174	B2	33.80098799	Bcer	CO	Bcer	FALSE	NA	NA	NA
Bc_ctg1_3175	B2	26.57456987	Bcer	C	Bcer	FALSE	NA	NA	NA
Bc_ctg1_3176	B2	1.048996179	Bcer	S	Bcer	FALSE	NA	NA	NA
Bc_ctg1_3177	B2	36.8314214	Bcer	S	Bcer	FALSE	NA	NA	NA
Bc_ctg1_3178	B2	0.932441048	Bcer	E	Bcer	FALSE	NA	NA	NA
Bc_ctg1_3179	B2	0.815885917	Bcer	S	Bcer	FALSE	NA	NA	NA
Bc_ctg1_318	B2	16.08460808	Bcer	M	Bcer	FALSE	Bc_Ga0417192_01_rn03	Core biosynthesis	None
Bc_ctg1_3180	B2	0	Bcer	P	Bcer	FALSE	NA	NA	NA
Bc_ctg1_3181	B2	0.116555131	Bcer	NA	Bcer	FALSE	NA	NA	NA
Bc_ctg1_3182	B2	24.94279803	Bcer	C	Bcer	FALSE	NA	NA	NA
Bc_ctg1_3183	B2	0.815885917	Bcer	S	Bcer	FALSE	NA	NA	NA
Bc_ctg1_3184	B2	10.37340666	Bcer	S	Bcer	FALSE	NA	NA	NA
Bc_ctg1_3185	B2	6.760197598	Bcer	M	Bcer	FALSE	NA	NA	NA
Bc_ctg1_3186	B2	1.981437227	Bcer	NA	Bcer	FALSE	NA	NA	NA
Bc_ctg1_3187	B2	7.109862991	Bcer	P	Bcer	FALSE	NA	NA	NA
Bc_ctg1_3188	B2	18.99848635	Bcer	S	Bcer	FALSE	NA	NA	NA
Bc_ctg1_3189	B2	0.466220524	Bcer	OP	Bcer	FALSE	NA	NA	NA
Bc_ctg1_319	B2	9.674075873	Bcer	E	Bcer	FALSE	Bc_Ga0417192_01_rn03	Transport	SMCOG1116
Bc_ctg1_3190	B2	1.16555131	Bcer	P	Bcer	FALSE	NA	NA	NA
Bc_ctg1_3191	B2	0	Bcer	O	Bcer	FALSE	NA	NA	NA
Bc_ctg1_3192	B2	0	Bcer	P	Bcer	FALSE	NA	NA	NA
Bc_ctg1_3193	B2	0.466220524	Bcer	P	Bcer	FALSE	NA	NA	NA
Bc_ctg1_3194	B2	0	Bcer	O	Bcer	FALSE	NA	NA	NA
Bc_ctg1_3195	B2	17.25015939	Bcer	S	Bcer	FALSE	NA	NA	NA
Bc_ctg1_3196	B2	147.675351	Bcer	NA	Bcer	FALSE	NA	NA	NA
Bc_ctg1_3197	B2	426.0090038	Bcer	K	Bcer	FALSE	NA	NA	NA
Bc_ctg1_3198	B2	55.94646288	Bcer	C	Bcer	FALSE	NA	NA	NA
Bc_ctg1_32	B2	31.00366485	Bcer	J	Bcer	FALSE	NA	NA	NA
Bc_ctg1_320	B2	35.78242522	Bcer	S	Bcer	FALSE	Bc_Ga0417192_01_rn03	Other	NA
Bc_ctg1_3201	B2	8.042304039	Bcer	P	Bcer	FALSE	NA	NA	NA
Bc_ctg1_3202	B2	21.21303384	Bcer	EGP	Bcer	FALSE	NA	NA	NA
Bc_ctg1_3203	B2	115.0399143	Bcer	S	Bcer	FALSE	NA	NA	NA
Bc_ctg1_3204	B2	17.13360426	Bcer	K	Bcer	FALSE	NA	NA	NA
Bc_ctg1_3205	B2	4.895315502	Bcer	G	Bcer	FALSE	NA	NA	NA
Bc_ctg1_3206	B2	7.692638646	Bcer	G	Bcer	FALSE	NA	NA	NA
Bc_ctg1_3207	B2	32.16921616	Bcer	G	Bcer	FALSE	NA	NA	NA
Bc_ctg1_3208	B2	4.778760371	Bcer	NA	Bcer	FALSE	NA	NA	NA
Bc_ctg1_3209	B2	24.70968777	Bcer	S	Bcer	FALSE	NA	NA	NA
Bc_ctg1_321	B2	1.16555131	Bcer	NA	Bcer	FALSE	Bc_Ga0417192_01_rn03	Other	NA
Bc_ctg1_3210	B2	1712.54454	Bcer	M	Bcer	FALSE	NA	NA	NA

Bc_ctg1_3211	B2	108.512827	Bcer	V	Bcer	FALSE	NA	NA	NA
Bc_ctg1_3212	B2	47.55449345	Bcer	V	Bcer	FALSE	NA	NA	NA
Bc_ctg1_3213	B2	48.83659989	Bcer	M	Bcer	FALSE	NA	NA	NA
Bc_ctg1_3214	B2	4.778760371	Bcer	S	Bcer	FALSE	NA	NA	NA
Bc_ctg1_3215	B2	0.233110262	Bcer	EP	Bcer	FALSE	NA	NA	NA
Bc_ctg1_3216	B2	2.913878275	Bcer	EP	Bcer	FALSE	NA	NA	NA
Bc_ctg1_3217	B2	30.53744432	Bcer	E	Bcer	FALSE	NA	NA	NA
Bc_ctg1_3218	B2	0.233110262	Bcer	P	Bcer	FALSE	NA	NA	NA
Bc_ctg1_322	B2	0.699330786	Bcer	NA	Bcer	FALSE	Bc_Ga0417192_01_rn03	Other	NA
Bc_ctg1_3220	B2	0	Bcer	S	Bcer	FALSE	NA	NA	NA
Bc_ctg1_3221	B2	0	Bcer	E	Bcer	FALSE	NA	NA	NA
Bc_ctg1_3222	B2	0.699330786	Bcer	S	Bcer	FALSE	NA	NA	NA
Bc_ctg1_3223	B2	0.466220524	Bcer	L	Bcer	FALSE	NA	NA	NA
Bc_ctg1_3224	B2	0.466220524	Bcer	S	Bcer	FALSE	NA	NA	NA
Bc_ctg1_3225	B2	3.030433406	Bcer	S	Bcer	FALSE	NA	NA	NA
Bc_ctg1_3226	B2	1.748326965	Bcer	S	Bcer	FALSE	NA	NA	NA
Bc_ctg1_3227	B2	1.515216703	Bcer	S	Bcer	FALSE	NA	NA	NA
Bc_ctg1_3228	B2	0.116555131	Bcer	NA	Bcer	FALSE	NA	NA	NA
Bc_ctg1_3229	B2	6.760197598	Bcer	S	Bcer	FALSE	NA	NA	NA
Bc_ctg1_323	B2	7.925748908	Bcer	K	Bcer	FALSE	Bc_Ga0417192_01_rn03	Other	NA
Bc_ctg1_3230	B2	7.925748908	Bcer	NA	Bcer	FALSE	NA	NA	NA
Bc_ctg1_3231	B2	8.042304039	Bcer	NA	Bcer	FALSE	NA	NA	NA
Bc_ctg1_3232	B2	1.864882096	Bcer	E	Bcer	FALSE	NA	NA	NA
Bc_ctg1_3233	B2	0.699330786	Bcer	P	Bcer	FALSE	NA	NA	NA
Bc_ctg1_3234	B2	4.079429585	Bcer	NA	Bcer	FALSE	NA	NA	NA
Bc_ctg1_3235	B2	39.62874454	Bcer	K	Bcer	FALSE	NA	NA	NA
Bc_ctg1_3236	B2	44.52406004	Bcer	G	Bcer	FALSE	NA	NA	NA
Bc_ctg1_3237	B2	455.1477865	Bcer	G	Bcer	FALSE	NA	NA	NA
Bc_ctg1_3238	B2	293.2527096	Bcer	S	Bcer	FALSE	NA	NA	NA
Bc_ctg1_3239	B2	0	Bcer	S	Bcer	FALSE	NA	NA	NA
Bc_ctg1_324	B2	0.349665393	Bcer	K	Bcer	FALSE	Bc_Ga0417192_01_rn03	Other	NA
Bc_ctg1_3241	B2	0	Bcer	NA	Bcer	FALSE	NA	NA	NA
Bc_ctg1_3242	B2	13.98661572	Bcer	EG	Bcer	FALSE	NA	NA	NA
Bc_ctg1_3243	B2	0.466220524	Bcer	K	Bcer	FALSE	NA	NA	NA
Bc_ctg1_3244	B2	0	Bcer	E	Bcer	FALSE	NA	NA	NA
Bc_ctg1_3245	B2	0.466220524	Bcer	S	Bcer	FALSE	NA	NA	NA
Bc_ctg1_3246	B2	0.116555131	Bcer	K	Bcer	FALSE	NA	NA	NA
Bc_ctg1_3247	B2	2.564212882	Bcer	P	Bcer	FALSE	NA	NA	NA
Bc_ctg1_3248	B2	1.515216703	Bcer	P	Bcer	FALSE	NA	NA	NA
Bc_ctg1_3249	B2	34.38376364	Bcer	K	Bcer	FALSE	NA	NA	NA
Bc_ctg1_325	B2	4.66220524	Bcer	V	Bcer	FALSE	Bc_Ga0417192_01_rn03	Transport	SMCOG1065
Bc_ctg1_3250	B2	36.36520087	Bcer	EGP	Bcer	FALSE	NA	NA	NA
Bc_ctg1_3251	B2	0.233110262	Bcer	NA	Bcer	FALSE	NA	NA	NA
Bc_ctg1_3252	B2	9.091300218	Bcer	T	Bcer	FALSE	NA	NA	NA
Bc_ctg1_3253	B2	20.63025819	Bcer	T	Bcer	FALSE	NA	NA	NA
Bc_ctg1_3254	B2	1.748326965	Bcer	M	Bcer	FALSE	NA	NA	NA
Bc_ctg1_3255	B2	0.233110262	Bcer	V	Bcer	FALSE	NA	NA	NA
Bc_ctg1_3256	B2	0.699330786	Bcer	V	Bcer	FALSE	NA	NA	NA
Bc_ctg1_3257	B2	0.116555131	Bcer	V	Bcer	FALSE	NA	NA	NA
Bc_ctg1_3258	B2	0.699330786	Bcer	V	Bcer	FALSE	NA	NA	NA
Bc_ctg1_3259	B2	3.49665393	Bcer	S	Bcer	FALSE	NA	NA	NA
Bc_ctg1_326	B2	3.613209061	Bcer	V	Bcer	FALSE	Bc_Ga0417192_01_rn03	Transport	SMCOG1000
Bc_ctg1_3260	B2	2.097992358	Bcer	K	Bcer	FALSE	NA	NA	NA
Bc_ctg1_3261	B2	0.233110262	Bcer	S	Bcer	FALSE	NA	NA	NA
Bc_ctg1_3262	B2	0.699330786	Bcer	S	Bcer	FALSE	NA	NA	NA
Bc_ctg1_3263	B2	1.398661572	Bcer	S	Bcer	FALSE	NA	NA	NA
Bc_ctg1_3264	B2	12.00517849	Bcer	S	Bcer	FALSE	NA	NA	NA
Bc_ctg1_3265	B2	32.51888155	Bcer	E	Bcer	FALSE	NA	NA	NA
Bc_ctg1_3266	B2	0	Bcer	NA	Bcer	FALSE	NA	NA	NA
Bc_ctg1_3267	B2	6.177421943	Bcer	S	Bcer	FALSE	NA	NA	NA
Bc_ctg1_3268	B2	32.05266102	Bcer	P	Bcer	FALSE	NA	NA	NA
Bc_ctg1_3269	B2	52.33325382	Bcer	U	Bcer	FALSE	NA	NA	NA

Bc_ctg1_327	B2	7.342973253	Bcer	S	Bcer	FALSE	Bc_Ga0417192_01_rn03	Other	NA
Bc_ctg1_3270	B2	32.86854694	Bcer	E	Bcer	FALSE	NA	NA	NA
Bc_ctg1_3271	B2	6.527087336	Bcer	S	Bcer	FALSE	NA	NA	NA
Bc_ctg1_3272	B2	480.3236948	Bcer	C	Bcer	FALSE	NA	NA	NA
Bc_ctg1_3273	B2	19.11504148	Bcer	V	Bcer	FALSE	NA	NA	NA
Bc_ctg1_3274	B2	19.58126201	Bcer	V	Bcer	FALSE	NA	NA	NA
Bc_ctg1_3275	B2	151.28856	Bcer	ET	Bcer	FALSE	NA	NA	NA
Bc_ctg1_3276	B2	99.30497161	Bcer	P	Bcer	FALSE	NA	NA	NA
Bc_ctg1_3277	B2	149.1905677	Bcer	E	Bcer	FALSE	NA	NA	NA
Bc_ctg1_3278	B2	0	Bcer	S	Bcer	FALSE	NA	NA	NA
Bc_ctg1_3279	B2	0	Bcer	NA	Bcer	FALSE	NA	NA	NA
Bc_ctg1_328	B2	23.07791594	Bcer	K	Bcer	FALSE	Bc_Ga0417192_01_rn03	Other	NA
Bc_ctg1_3280	B2	0.116555131	Bcer	HJ	Bcer	FALSE	NA	NA	NA
Bc_ctg1_3281	B2	0.116555131	Bcer	HJ	Bcer	FALSE	NA	NA	NA
Bc_ctg1_3282	B2	9.790631004	Bcer	S	Bcer	FALSE	NA	NA	NA
Bc_ctg1_3283	B2	184.6233275	Bcer	S	Bcer	FALSE	NA	NA	NA
Bc_ctg1_3284	B2	3.729764192	Bcer	NA	Bcer	FALSE	NA	NA	NA
Bc_ctg1_3285	B2	4.079429585	Bcer	S	Bcer	FALSE	NA	NA	NA
Bc_ctg1_3286	B2	102.1022948	Bcer	L	Bcer	FALSE	NA	NA	NA
Bc_ctg1_3287	B2	232.0612658	Bcer	EH	Bcer	FALSE	NA	NA	NA
Bc_ctg1_3288	B2	82.63758788	Bcer	Q	Bcer	TRUE	NA	NA	NA
Bc_ctg1_3289	B2	2.447657751	Bcer	L	Bcer	FALSE	NA	NA	NA
Bc_ctg1_329	B2	503.634721	Bcer	C	Bcer	FALSE	Bc_Ga0417192_01_rn03	Other	NA
Bc_ctg1_3290	B2	0.466220524	Bcer	S	Bcer	FALSE	NA	NA	NA
Bc_ctg1_3291	B2	65.85364901	Bcer	M	Bcer	FALSE	NA	NA	NA
Bc_ctg1_3292	B2	233.9261479	Bcer	M	Bcer	FALSE	NA	NA	NA
Bc_ctg1_3293	B2	205.4866959	Bcer	H	Bcer	FALSE	NA	NA	NA
Bc_ctg1_3294	B2	6.760197598	Bcer	IQ	Bcer	TRUE	NA	NA	NA
Bc_ctg1_3295	B2	0.116555131	Bcer	NA	Bcer	FALSE	NA	NA	NA
Bc_ctg1_3296	B2	50.70148198	Bcer	NA	Bcer	FALSE	NA	NA	NA
Bc_ctg1_3297	B2	59.32656168	Bcer	P	Bcer	FALSE	NA	NA	NA
Bc_ctg1_3298	B2	78.09193777	Bcer	S	Bcer	FALSE	NA	NA	NA
Bc_ctg1_3299	B2	41.95984716	Bcer	S	Bcer	FALSE	NA	NA	NA
Bc_ctg1_3300	B2	142.1972598	Bcer	G	Bcer	FALSE	NA	NA	NA
Bc_ctg1_3301	B2	52.91602947	Bcer	M	Bcer	FALSE	NA	NA	NA
Bc_ctg1_3302	B2	51.5173679	Bcer	I	Bcer	FALSE	NA	NA	NA
Bc_ctg1_3303	B2	2.913878275	Bcer	NA	Bcer	FALSE	NA	NA	NA
Bc_ctg1_3304	B2	1.16555131	Bcer	I	Bcer	FALSE	NA	NA	NA
Bc_ctg1_3305	B2	0.233110262	Bcer	NA	Bcer	FALSE	NA	NA	NA
Bc_ctg1_3306	B2	100.5870781	Bcer	G	Bcer	FALSE	NA	NA	NA
Bc_ctg1_3307	B2	80.18993013	Bcer	M	Bcer	FALSE	NA	NA	NA
Bc_ctg1_3308	B2	30.65399945	Bcer	L	Bcer	FALSE	NA	NA	NA
Bc_ctg1_331	B2	1.398661572	Bcer	S	Bcer	FALSE	NA	NA	NA
Bc_ctg1_3310	B2	0.116555131	Bcer	E	Bcer	FALSE	NA	NA	NA
Bc_ctg1_3311	B2	4.429094978	Bcer	E	Bcer	FALSE	NA	NA	NA
Bc_ctg1_3312	B2	7.809193777	Bcer	NA	Bcer	FALSE	NA	NA	NA
Bc_ctg1_3313	B2	74.47872871	Bcer	E	Bcer	FALSE	NA	NA	NA
Bc_ctg1_3314	B2	12.00517849	Bcer	P	Bcer	FALSE	NA	NA	NA
Bc_ctg1_3315	B2	15.26872216	Bcer	EP	Bcer	FALSE	NA	NA	NA
Bc_ctg1_3316	B2	29.83811354	Bcer	P	Bcer	FALSE	NA	NA	NA
Bc_ctg1_3317	B2	40.91085098	Bcer	E	Bcer	FALSE	NA	NA	NA
Bc_ctg1_3318	B2	0.116555131	Bcer	O	Bcer	FALSE	NA	NA	NA
Bc_ctg1_3319	B2	0.466220524	Bcer	S	Bcer	FALSE	NA	NA	NA
Bc_ctg1_332	B2	3.030433406	Bcer	M	Bcer	FALSE	NA	NA	NA
Bc_ctg1_3320	B2	0.466220524	Bcer	S	Bcer	FALSE	NA	NA	NA
Bc_ctg1_3321	B2	0.582775655	Bcer	S	Bcer	FALSE	NA	NA	NA
Bc_ctg1_3322	B2	0.699330786	Bcer	NA	Bcer	FALSE	NA	NA	NA
Bc_ctg1_3324	B2	0	Bcer	NA	Bcer	FALSE	NA	NA	NA
Bc_ctg1_3325	B2	1.282106441	Bcer	V	Bcer	FALSE	NA	NA	NA
Bc_ctg1_3326	B2	0.116555131	Bcer	EGP	Bcer	FALSE	NA	NA	NA
Bc_ctg1_3327	B2	4.195984716	Bcer	T	Bcer	FALSE	NA	NA	NA
Bc_ctg1_3328	B2	1.048996179	Bcer	T	Bcer	FALSE	NA	NA	NA

Bc_ctg1_3329	B2	0.582775655	Bcer	S	Bcer	FALSE	NA	NA	NA
Bc_ctg1_333	B2	0	Bcer	S	Bcer	FALSE	NA	NA	NA
Bc_ctg1_3330	B2	0	Bcer	NA	Bcer	FALSE	NA	NA	NA
Bc_ctg1_3331	B2	9.557520742	Bcer	L	Bcer	FALSE	NA	NA	NA
Bc_ctg1_3332	B2	9.557520742	Bcer	L	Bcer	FALSE	NA	NA	NA
Bc_ctg1_3333	B2	13.75350546	Bcer	S	Bcer	FALSE	NA	NA	NA
Bc_ctg1_3334	B2	23.42758133	Bcer	K	Bcer	FALSE	NA	NA	NA
Bc_ctg1_3335	B2	1.864882096	Bcer	S	Bcer	FALSE	NA	NA	NA
Bc_ctg1_3336	B2	1.515216703	Bcer	EG	Bcer	FALSE	NA	NA	NA
Bc_ctg1_3337	B2	16.66738373	Bcer	KT	Bcer	FALSE	NA	NA	NA
Bc_ctg1_3338	B2	0	Bcer	S	Bcer	FALSE	NA	NA	NA
Bc_ctg1_3339	B2	0.349665393	Bcer	S	Bcer	FALSE	NA	NA	NA
Bc_ctg1_334	B2	16.5508286	Bcer	S	Bcer	FALSE	NA	NA	NA
Bc_ctg1_3340	B2	0.116555131	Bcer	NA	Bcer	FALSE	NA	NA	NA
Bc_ctg1_3341	B2	11.88862336	Bcer	K	Bcer	FALSE	NA	NA	NA
Bc_ctg1_3342	B2	54.8974667	Bcer	EGP	Bcer	FALSE	NA	NA	NA
Bc_ctg1_3343	B2	8.275414301	Bcer	NA	Bcer	FALSE	NA	NA	NA
Bc_ctg1_3345	B2	20.74681332	Bcer	NA	Bcer	FALSE	NA	NA	NA
Bc_ctg1_3346	B2	7.925748908	Bcer	M	Bcer	FALSE	NA	NA	NA
Bc_ctg1_3347	B2	1.282106441	Bcer	NA	Bcer	FALSE	NA	NA	NA
Bc_ctg1_3348	B2	5.361536026	Bcer	G	Bcer	FALSE	NA	NA	NA
Bc_ctg1_335	B2	0.116555131	Bcer	NA	Bcer	FALSE	NA	NA	NA
Bc_ctg1_3350	B2	1.631771834	Bcer	NA	Bcer	FALSE	NA	NA	NA
Bc_ctg1_3351	B2	5.011870633	Bcer	S	Bcer	FALSE	NA	NA	NA
Bc_ctg1_3353	B2	25.52557369	Bcer	S	Bcer	FALSE	NA	NA	NA
Bc_ctg1_3354	B2	4.312539847	Bcer	T	Bcer	FALSE	NA	NA	NA
Bc_ctg1_3356	B2	1.981437227	Bcer	K	Bcer	FALSE	NA	NA	NA
Bc_ctg1_3357	B2	4.778760371	Bcer	I	Bcer	FALSE	NA	NA	NA
Bc_ctg1_3358	B2	2.447657751	Bcer	S	Bcer	FALSE	NA	NA	NA
Bc_ctg1_3359	B2	0.116555131	Bcer	EGP	Bcer	FALSE	NA	NA	NA
Bc_ctg1_336	B2	0.233110262	Bcer	NA	Bcer	FALSE	NA	NA	NA
Bc_ctg1_3360	B2	17.25015939	Bcer	K	Bcer	FALSE	NA	NA	NA
Bc_ctg1_3362	B2	0.466220524	Bcer	V	Bcer	FALSE	NA	NA	NA
Bc_ctg1_3363	B2	12.00517849	Bcer	G	Bcer	FALSE	NA	NA	NA
Bc_ctg1_3364	B2	3.846319323	Bcer	K	Bcer	FALSE	NA	NA	NA
Bc_ctg1_3365	B2	202.3397074	Bcer	G	Bcer	FALSE	NA	NA	NA
Bc_ctg1_3366	B2	6.643642467	Bcer	I	Bcer	FALSE	NA	NA	NA
Bc_ctg1_3367	B2	8.974745087	Bcer	K	Bcer	FALSE	NA	NA	NA
Bc_ctg1_3368	B2	0.582775655	Bcer	NA	Bcer	FALSE	NA	NA	NA
Bc_ctg1_337	B2	9.557520742	Bcer	V	Bcer	FALSE	NA	NA	NA
Bc_ctg1_3371	B2	3.380098799	Bcer	NA	Bcer	FALSE	NA	NA	NA
Bc_ctg1_3372	B2	0.466220524	Bcer	NA	Bcer	FALSE	NA	NA	NA
Bc_ctg1_3373	B2	7.692638646	Bcer	K	Bcer	FALSE	NA	NA	NA
Bc_ctg1_3374	B2	0.233110262	Bcer	NA	Bcer	FALSE	NA	NA	NA
Bc_ctg1_3375	B2	0	Bcer	NA	Bcer	FALSE	NA	NA	NA
Bc_ctg1_3376	B2	15.50183242	Bcer	S	Bcer	FALSE	NA	NA	NA
Bc_ctg1_3377	B2	0.116555131	Bcer	NA	Bcer	FALSE	NA	NA	NA
Bc_ctg1_3378	B2	12.70450928	Bcer	D	Bcer	FALSE	NA	NA	NA
Bc_ctg1_3379	B2	0.582775655	Bcer	NA	Bcer	FALSE	NA	NA	NA
Bc_ctg1_338	B2	1.282106441	Bcer	NA	Bcer	FALSE	NA	NA	NA
Bc_ctg1_3380	B2	0	Bcer	K	Bcer	FALSE	NA	NA	NA
Bc_ctg1_3381	B2	1.398661572	Bcer	S	Bcer	FALSE	NA	NA	NA
Bc_ctg1_3382	B2	0.349665393	Bcer	S	Bcer	FALSE	NA	NA	NA
Bc_ctg1_3383	B2	0	Bcer	NA	Bcer	FALSE	NA	NA	NA
Bc_ctg1_3384	B2	0.349665393	Bcer	NA	Bcer	FALSE	NA	NA	NA
Bc_ctg1_3385	B2	0.233110262	Bcer	S	Bcer	FALSE	NA	NA	NA
Bc_ctg1_3386	B2	0.116555131	Bcer	K	Bcer	FALSE	NA	NA	NA
Bc_ctg1_3387	B2	0.233110262	Bcer	S	Bcer	FALSE	NA	NA	NA
Bc_ctg1_3388	B2	0.233110262	Bcer	NA	Bcer	FALSE	NA	NA	NA
Bc_ctg1_3389	B2	9.091300218	Bcer	T	Bcer	FALSE	NA	NA	NA
Bc_ctg1_339	B2	10.48996179	Bcer	P	Bcer	FALSE	NA	NA	NA
Bc_ctg1_3390	B2	22.84480568	Bcer	T	Bcer	FALSE	NA	NA	NA

Bc_ctg1_3391	B2	29.60500327	Bcer	K	Bcer	FALSE	NA	NA	NA	
Bc_ctg1_3392	B2	48.48693449	Bcer	S	Bcer	FALSE	NA	NA	NA	
Bc_ctg1_3393	B2	33.91754312	Bcer	T	Bcer	FALSE	NA	NA	NA	
Bc_ctg1_3394	B2	33.56787773	Bcer	NT	Bcer	FALSE	NA	NA	NA	
Bc_ctg1_3395	B2	79.7237096	Bcer	T	Bcer	FALSE	NA	NA	NA	
Bc_ctg1_3396	B2	0.582775655	Bcer	NA	Bcer	FALSE	NA	NA	NA	
Bc_ctg1_3398	B2	1.981437227	Bcer	S	Bcer	FALSE	NA	NA	NA	
Bc_ctg1_3399	B2	0.699330786	Bcer	NA	Bcer	FALSE	NA	NA	NA	
Bc_ctg1_34	B2	1.048996179	Bcer	S	Bcer	FALSE	NA	NA	NA	
Bc_ctg1_340	B2	2.33110262	Bcer	E	Bcer	FALSE	NA	NA	NA	
Bc_ctg1_3400	B2	225.5341785	Bcer	S	Bcer	FALSE	NA	NA	NA	
Bc_ctg1_3402	B2	3.146988537	Bcer	NA	Bcer	FALSE	NA	NA	NA	
Bc_ctg1_3403	B2	1.631771834	Bcer	P	Bcer	FALSE	NA	NA	NA	
Bc_ctg1_3404	B2	18.18260044	Bcer	K	Bcer	FALSE	NA	NA	NA	
Bc_ctg1_3405	B2	14.80250164	Bcer	C	Bcer	FALSE	NA	NA	NA	
Bc_ctg1_3406	B2	30.88710971	Bcer	M	Bcer	FALSE	NA	NA	NA	
Bc_ctg1_3407	B2	0.582775655	Bcer	S	Bcer	FALSE	NA	NA	NA	
Bc_ctg1_3408	B2	26.57456987	Bcer	NA	Bcer	FALSE	NA	NA	NA	
Bc_ctg1_3409	B2	16.43427347	Bcer	S	Bcer	FALSE	NA	NA	NA	
Bc_ctg1_341	B2	1.631771834	Bcer	K	Bcer	FALSE	NA	NA	NA	
Bc_ctg1_3410	B2	11.53895797	Bcer	L	Bcer	FALSE	NA	NA	NA	
Bc_ctg1_3411	B2	31.00366485	Bcer	L	Bcer	FALSE	NA	NA	NA	
Bc_ctg1_3412	B2	271.9231206	Bcer	L	Bcer	FALSE	NA	NA	NA	
Bc_ctg1_3413	B2	36.481756	Bcer	S	Bcer	FALSE	NA	NA	NA	
Bc_ctg1_3414	B2	46.85516266	Bcer	E	Bcer	FALSE	NA	NA	NA	
Bc_ctg1_3415	B2	76.22705567	Bcer	E	Bcer	FALSE	NA	NA	NA	
Bc_ctg1_3416	B2	124.9471004	Bcer	E	Bcer	FALSE	NA	NA	NA	
Bc_ctg1_3417	B2	2.913878275	Bcer	K	Bcer	FALSE	NA	NA	NA	
Bc_ctg1_3418	B2	0	Bcer	NA	Bcer	FALSE	NA	NA	NA	
Bc_ctg1_3419	B2	1.748326965	Bcer	S	Bcer	FALSE	NA	NA	NA	
Bc_ctg1_342	B2	0.815885917	Bcer	S	Bcer	FALSE	NA	NA	NA	
Bc_ctg1_3420	B2	0.582775655	Bcer	S	Bcer	FALSE	NA	NA	NA	
Bc_ctg1_3421	B2	1.398661572	Bcer	S	Bcer	FALSE	NA	NA	NA	
Bc_ctg1_3423	B2	1.981437227	Bcer	G	Bcer	FALSE	NA	NA	NA	
Bc_ctg1_3424	B2	1.048996179	Bcer	K	Bcer	FALSE	NA	NA	NA	
Bc_ctg1_3425	B2	127.7444236	Bcer	L	Bcer	FALSE	NA	NA	NA	
Bc_ctg1_3426	B2	62.93977074	Bcer	K	Bcer	FALSE	NA	NA	NA	
Bc_ctg1_3427	B2	23.42758133	Bcer	G	Bcer	FALSE	NA	NA	NA	
Bc_ctg1_3428	B2	157.3494268	Bcer	C	Bcer	FALSE	NA	NA	NA	
Bc_ctg1_3429	B2	232.6440415	Bcer	C	Bcer	FALSE	NA	NA	NA	
Bc_ctg1_343	B2	1.864882096	Bcer	S	Bcer	FALSE	NA	NA	NA	
Bc_ctg1_3430	B2	9.32441048	Bcer	K	Bcer	FALSE	NA	NA	NA	
Bc_ctg1_3431	B2	24.24346725	Bcer	V	Bcer	FALSE	NA	NA	NA	
Bc_ctg1_3432	B2	1.282106441	Bcer	NA	Bcer	FALSE	NA	NA	NA	
Bc_ctg1_3433	B2	13.63695033	Bcer	L	Bcer	FALSE	NA	NA	NA	
Bc_ctg1_3434	B2	1.048996179	Bcer	K	Bcer	FALSE	NA	NA	NA	
Bc_ctg1_3435	B2	0	Bcer	K	Bcer	FALSE	NA	NA	NA	
Bc_ctg1_3436	B2	13.05417467	Bcer	D	Bcer	FALSE	NA	NA	NA	
Bc_ctg1_3437	B2	26.45801474	Bcer	L	Bcer	FALSE	NA	NA	NA	
Bc_ctg1_3438	B2	2.447657751	Bcer	NA	Bcer	FALSE	NA	NA	NA	
Bc_ctg1_3439	B2	720.1941544	Bcer	M	Bcer	FALSE	NA	NA	NA	
Bc_ctg1_344	B2	1.16555131	Bcer	S	Bcer	FALSE	NA	NA	NA	
Bc_ctg1_3440	B2	0.582775655	Bcer	NA	Bcer	FALSE	NA	NA	NA	
Bc_ctg1_3441	B2	10.60651692	Bcer	S	Bcer	FALSE	NA	NA	NA	
Bc_ctg1_3442	B2	157.465982	Bcer	K	Bcer	FALSE	NA	NA	NA	
Bc_ctg1_3443	B2	132.5231839	Bcer	S	Bcer	FALSE	NA	NA	NA	
Bc_ctg1_3444	B2	72.38073635	Bcer	FG	Bcer	FALSE	NA	NA	NA	
Bc_ctg1_3445	B2	38.46319323	Bcer	V	Bcer	FALSE	NA	NA	NA	
Bc_ctg1_3446	B2	56.99545906	Bcer	U	Bcer	FALSE	NA	NA	NA	
Bc_ctg1_3447	B2	50.0021512	Bcer	P	Bcer	FALSE	NA	NA	NA	
Bc_ctg1_3448	B2	5.244980895	Bcer	NA	Bcer	FALSE	NA	NA	NA	
Bc_ctg1_3449	B2	5.011870633	Bcer	NA	Bcer	FALSE	NA	NA	NA	

Bc_ctg1_345	B2	17.48326965	Bcer	S	Bcer	FALSE	Bc_Ga0417192_01_rn04	Other	NA
Bc_ctg1_3450	B2	9.207855349	Bcer	NA	Bcer	FALSE	NA	NA	NA
Bc_ctg1_3451	B2	6.177421943	Bcer	IU	Bcer	FALSE	NA	NA	NA
Bc_ctg1_3452	B2	1.398661572	Bcer	NA	Bcer	FALSE	NA	NA	NA
Bc_ctg1_3453	B2	3.846319323	Bcer	K	Bcer	FALSE	NA	NA	NA
Bc_ctg1_3454	B2	0.233110262	Bcer	NA	Bcer	FALSE	NA	NA	NA
Bc_ctg1_3455	B2	15.73494268	Bcer	S	Bcer	FALSE	NA	NA	NA
Bc_ctg1_3456	B2	78.44160316	Bcer	S	Bcer	FALSE	NA	NA	NA
Bc_ctg1_3457	B2	46.6220524	Bcer	S	Bcer	FALSE	NA	NA	NA
Bc_ctg1_3458	B2	3.030433406	Bcer	NA	Bcer	FALSE	NA	NA	NA
Bc_ctg1_3459	B2	0.582775655	Bcer	S	Bcer	FALSE	NA	NA	NA
Bc_ctg1_346	B2	24.47657751	Bcer	C	Bcer	FALSE	Bc_Ga0417192_01_rn04	Ancillary biosynthesis	SMCOG1028
Bc_ctg1_3460	B2	4.195984716	Bcer	NA	Bcer	FALSE	NA	NA	NA
Bc_ctg1_3461	B2	1.282106441	Bcer	K	Bcer	FALSE	NA	NA	NA
Bc_ctg1_3462	B2	6.99330786	Bcer	M	Bcer	FALSE	NA	NA	NA
Bc_ctg1_3463	B2	103.0347358	Bcer	E	Bcer	FALSE	NA	NA	NA
Bc_ctg1_3464	B2	101.1698537	Bcer	IQ	Bcer	TRUE	NA	NA	NA
Bc_ctg1_3465	B2	46.50549727	Bcer	EG	Bcer	FALSE	NA	NA	NA
Bc_ctg1_3466	B2	4.429094978	Bcer	S	Bcer	FALSE	NA	NA	NA
Bc_ctg1_3467	B2	7.459528384	Bcer	S	Bcer	FALSE	NA	NA	NA
Bc_ctg1_3468	B2	133.2225147	Bcer	S	Bcer	FALSE	NA	NA	NA
Bc_ctg1_3469	B2	4.312539847	Bcer	M	Bcer	FALSE	NA	NA	NA
Bc_ctg1_347	B2	67.83508624	Bcer	E	Bcer	FALSE	Bc_Ga0417192_01_rn04	Ancillary biosynthesis	SMCOG1038
Bc_ctg1_3470	B2	99.88774726	Bcer	H	Bcer	FALSE	NA	NA	NA
Bc_ctg1_3471	B2	112.0094809	Bcer	H	Bcer	FALSE	NA	NA	NA
Bc_ctg1_3472	B2	263.9973717	Bcer	H	Bcer	FALSE	NA	NA	NA
Bc_ctg1_3473	B2	93.94343558	Bcer	S	Bcer	FALSE	NA	NA	NA
Bc_ctg1_3474	B2	10.83962718	Bcer	S	Bcer	FALSE	NA	NA	NA
Bc_ctg1_3475	B2	0.349665393	Bcer	NA	Bcer	FALSE	NA	NA	NA
Bc_ctg1_3476	B2	117.8372374	Bcer	NA	Bcer	FALSE	NA	NA	NA
Bc_ctg1_3477	B2	0.932441048	Bcer	K	Bcer	FALSE	NA	NA	NA
Bc_ctg1_3478	B2	6.527087336	Bcer	KT	Bcer	FALSE	NA	NA	NA
Bc_ctg1_3479	B2	54.78091157	Bcer	K	Bcer	FALSE	NA	NA	NA
Bc_ctg1_348	B2	53.61536026	Bcer	EGP	Bcer	FALSE	Bc_Ga0417192_01_rn04	Transport	SMCOG1005
Bc_ctg1_3480	B2	9.32441048	Bcer	S	Bcer	FALSE	NA	NA	NA
Bc_ctg1_3481	B2	0.932441048	Bcer	NA	Bcer	FALSE	NA	NA	NA
Bc_ctg1_3482	B2	3.846319323	Bcer	K	Bcer	FALSE	NA	NA	NA
Bc_ctg1_3483	B2	3.613209061	Bcer	S	Bcer	FALSE	NA	NA	NA
Bc_ctg1_3484	B2	1.048996179	Bcer	M	Bcer	FALSE	NA	NA	NA
Bc_ctg1_3485	B2	590.7014039	Bcer	K	Bcer	FALSE	NA	NA	NA
Bc_ctg1_3486	B2	0	Bcer	S	Bcer	FALSE	NA	NA	NA
Bc_ctg1_3487	B2	154.785214	Bcer	S	Bcer	FALSE	NA	NA	NA
Bc_ctg1_3488	B2	385.3312631	Bcer	H	Bcer	FALSE	NA	NA	NA
Bc_ctg1_3489	B2	0.233110262	Bcer	NA	Bcer	FALSE	NA	NA	NA
Bc_ctg1_349	B2	0.582775655	Bcer	NA	Bcer	FALSE	Bc_Ga0417192_01_rn04	Other	NA
Bc_ctg1_3490	B2	29.83811354	Bcer	IQ	Bcer	TRUE	NA	NA	NA
Bc_ctg1_3491	B2	0	Bcer	M	Bcer	FALSE	NA	NA	NA
Bc_ctg1_3492	B2	2.913878275	Bcer	P	Bcer	FALSE	NA	NA	NA
Bc_ctg1_3493	B2	1.748326965	Bcer	O	Bcer	FALSE	NA	NA	NA
Bc_ctg1_3494	B2	1.864882096	Bcer	P	Bcer	FALSE	NA	NA	NA
Bc_ctg1_3495	B2	2.680768013	Bcer	P	Bcer	FALSE	NA	NA	NA
Bc_ctg1_3496	B2	858.4285398	Bcer	S	Bcer	FALSE	NA	NA	NA
Bc_ctg1_3497	B2	48.02071397	Bcer	S	Bcer	FALSE	NA	NA	NA
Bc_ctg1_3498	B2	2.214547489	Bcer	S	Bcer	FALSE	NA	NA	NA
Bc_ctg1_3499	B2	5.478091157	Bcer	S	Bcer	FALSE	NA	NA	NA
Bc_ctg1_35	B2	4.429094978	Bcer	S	Bcer	FALSE	NA	NA	NA
Bc_ctg1_350	B2	0	Bcer	S	Bcer	FALSE	Bc_Ga0417192_01_rn04	Other	NA
Bc_ctg1_3500	B2	6.643642467	Bcer	K	Bcer	FALSE	NA	NA	NA
Bc_ctg1_3501	B2	4.66220524	Bcer	U	Bcer	FALSE	NA	NA	NA
Bc_ctg1_3502	B2	5.361536026	Bcer	S	Bcer	FALSE	NA	NA	NA
Bc_ctg1_3503	B2	1.981437227	Bcer	K	Bcer	FALSE	NA	NA	NA
Bc_ctg1_3504	B2	57.92790011	Bcer	K	Bcer	FALSE	NA	NA	NA

Bc_ctg1_3505	B2	1.981437227	Bcer	E	Bcer	FALSE	NA	NA	NA
Bc_ctg1_3506	B2	18.0660453	Bcer	T	Bcer	FALSE	NA	NA	NA
Bc_ctg1_3507	B2	6.643642467	Bcer	T	Bcer	FALSE	NA	NA	NA
Bc_ctg1_3508	B2	30.77055458	Bcer	S	Bcer	FALSE	NA	NA	NA
Bc_ctg1_3509	B2	6.410532205	Bcer	NA	Bcer	FALSE	NA	NA	NA
Bc_ctg1_351	B2	0.466220524	Bcer	NA	Bcer	FALSE	Bc_Ga0417192_01_rn04	Other	NA
Bc_ctg1_3510	B2	3.030433406	Bcer	NA	Bcer	FALSE	NA	NA	NA
Bc_ctg1_3511	B2	9.440965611	Bcer	O	Bcer	FALSE	NA	NA	NA
Bc_ctg1_3512	B2	36.13209061	Bcer	NT	Bcer	FALSE	NA	NA	NA
Bc_ctg1_3513	B2	26.22490447	Bcer	D	Bcer	FALSE	NA	NA	NA
Bc_ctg1_3515	B2	2.097992358	Bcer	D	Bcer	FALSE	NA	NA	NA
Bc_ctg1_3516	B2	6.527087336	Bcer	C	Bcer	FALSE	NA	NA	NA
Bc_ctg1_3517	B2	14.33628111	Bcer	C	Bcer	FALSE	NA	NA	NA
Bc_ctg1_3518	B2	6.99330786	Bcer	S	Bcer	FALSE	NA	NA	NA
Bc_ctg1_3519	B2	1.515216703	Bcer	NA	Bcer	FALSE	NA	NA	NA
Bc_ctg1_352	B2	0	Bcer	NA	Bcer	FALSE	Bc_Ga0417192_01_rn04	Other	NA
Bc_ctg1_3520	B2	53.49880513	Bcer	K	Bcer	FALSE	NA	NA	NA
Bc_ctg1_3521	B2	0	Bcer	NA	Bcer	FALSE	NA	NA	NA
Bc_ctg1_3522	B2	1.864882096	Bcer	NA	Bcer	FALSE	NA	NA	NA
Bc_ctg1_3523	B2	11.18929258	Bcer	K	Bcer	FALSE	NA	NA	NA
Bc_ctg1_3524	B2	16.78393886	Bcer	S	Bcer	FALSE	NA	NA	NA
Bc_ctg1_3525	B2	16.20116321	Bcer	U	Bcer	FALSE	NA	NA	NA
Bc_ctg1_3526	B2	124.597435	Bcer	L	Bcer	FALSE	NA	NA	NA
Bc_ctg1_3527	B2	232.1778209	Bcer	L	Bcer	FALSE	NA	NA	NA
Bc_ctg1_3528	B2	0.932441048	Bcer	K	Bcer	FALSE	NA	NA	NA
Bc_ctg1_3529	B2	0.349665393	Bcer	S	Bcer	FALSE	NA	NA	NA
Bc_ctg1_353	B2	2.214547489	Bcer	NA	Bcer	FALSE	Bc_Ga0417192_01_rn04	Other	NA
Bc_ctg1_3530	B2	0.349665393	Bcer	S	Bcer	FALSE	NA	NA	NA
Bc_ctg1_3531	B2	0	Bcer	S	Bcer	FALSE	NA	NA	NA
Bc_ctg1_3532	B2	1.631771834	Bcer	S	Bcer	FALSE	NA	NA	NA
Bc_ctg1_3533	B2	0	Bcer	S	Bcer	FALSE	NA	NA	NA
Bc_ctg1_3534	B2	0	Bcer	S	Bcer	FALSE	NA	NA	NA
Bc_ctg1_3535	B2	0.932441048	Bcer	NA	Bcer	FALSE	NA	NA	NA
Bc_ctg1_3536	B2	32.05266102	Bcer	Q	Bcer	TRUE	NA	NA	NA
Bc_ctg1_3537	B2	6.760197598	Bcer	NA	Bcer	FALSE	NA	NA	NA
Bc_ctg1_3538	B2	2420.38385	Bcer	E	Bcer	FALSE	NA	NA	NA
Bc_ctg1_3539	B2	40.67774072	Bcer	S	Bcer	FALSE	NA	NA	NA
Bc_ctg1_354	B2	20.74681332	Bcer	IQ	Bcer	TRUE	Bc_Ga0417192_01_rn04	Ancillary biosynthesis	SMCOG1001
Bc_ctg1_3540	B2	0.582775655	Bcer	S	Bcer	FALSE	NA	NA	NA
Bc_ctg1_3541	B2	7.109862991	Bcer	E	Bcer	FALSE	NA	NA	NA
Bc_ctg1_3542	B2	219.1236463	Bcer	H	Bcer	FALSE	NA	NA	NA
Bc_ctg1_3543	B2	2695.570515	Bcer	P	Bcer	FALSE	NA	NA	NA
Bc_ctg1_3544	B2	1.16555131	Bcer	P	Bcer	FALSE	NA	NA	NA
Bc_ctg1_3545	B2	14.10317085	Bcer	G	Bcer	FALSE	NA	NA	NA
Bc_ctg1_3546	B2	24.36002238	Bcer	K	Bcer	FALSE	NA	NA	NA
Bc_ctg1_3547	B2	144.178697	Bcer	S	Bcer	FALSE	NA	NA	NA
Bc_ctg1_3548	B2	61.07488864	Bcer	S	Bcer	FALSE	NA	NA	NA
Bc_ctg1_3549	B2	66.08675928	Bcer	M	Bcer	FALSE	Bc_Ga0417192_01_rn24	Other	NA
Bc_ctg1_355	B2	107.5803859	Bcer	S	Bcer	FALSE	Bc_Ga0417192_01_rn04	Core biosynthesis	None
Bc_ctg1_3551	B2	3.380098799	Bcer	S	Bcer	FALSE	Bc_Ga0417192_01_rn24	Other	NA
Bc_ctg1_3552	B2	13.98661572	Bcer	S	Bcer	FALSE	Bc_Ga0417192_01_rn24	Other	NA
Bc_ctg1_3554	B2	99.07186135	Bcer	S	Bcer	FALSE	Bc_Ga0417192_01_rn24	Ancillary biosynthesis	SMCOG1179
Bc_ctg1_3555	B2	1.748326965	Bcer	S	Bcer	FALSE	Bc_Ga0417192_01_rn24	Other	NA
Bc_ctg1_3556	B2	416.5680382	Bcer	O	Bcer	FALSE	Bc_Ga0417192_01_rn24	Other	SMCOG1223
Bc_ctg1_3557	B2	102.8016255	Bcer	S	Bcer	FALSE	Bc_Ga0417192_01_rn24	Other	NA
Bc_ctg1_3558	B2	0.349665393	Bcer	I	Bcer	FALSE	Bc_Ga0417192_01_rn24	Other	NA
Bc_ctg1_3559	B2	0.349665393	Bcer	GM	Bcer	FALSE	Bc_Ga0417192_01_rn24	Other	NA
Bc_ctg1_356	B2	34.50031878	Bcer	S	Bcer	FALSE	Bc_Ga0417192_01_rn04	Other	NA
Bc_ctg1_3560	B2	0.466220524	Bcer	S	Bcer	FALSE	Bc_Ga0417192_01_rn24	Other	NA
Bc_ctg1_3561	B2	100.8201883	Bcer	I	Bcer	FALSE	Bc_Ga0417192_01_rn24	Core biosynthesis	SMCOG1084
Bc_ctg1_3562	B2	414.1203804	Bcer	I	Bcer	FALSE	Bc_Ga0417192_01_rn24	Core biosynthesis	SMCOG1022
Bc_ctg1_3563	B2	1.748326965	Bcer	O	Bcer	FALSE	Bc_Ga0417192_01_rn24	Other	NA

Bc_ctg1_3564	B2	4.195984716	Bcer	S	Bcer	FALSE	Bc_Ga0417192_01_rn24	Other	NA
Bc_ctg1_3565	B2	137.7681648	Bcer	J	Bcer	FALSE	Bc_Ga0417192_01_rn24	Other	NA
Bc_ctg1_3566	B2	8.508524563	Bcer	S	Bcer	FALSE	Bc_Ga0417192_01_rn24	Other	NA
Bc_ctg1_3567	B2	684.8779497	Bcer	E	Bcer	FALSE	Bc_Ga0417192_01_rn24	Transport	SMCOG1068
Bc_ctg1_3568	B2	70.28274399	Bcer	E	Bcer	FALSE	Bc_Ga0417192_01_rn24	Other	NA
Bc_ctg1_3569	B2	557.3666364	Bcer	P	Bcer	FALSE	Bc_Ga0417192_01_rn24	Transport	SMCOG1118
Bc_ctg1_357	B2	46.27238701	Bcer	P	Bcer	FALSE	Bc_Ga0417192_01_rn04	Other	NA
Bc_ctg1_3570	B2	621.8216239	Bcer	EP	Bcer	FALSE	Bc_Ga0417192_01_rn24	Transport	SMCOG1085
Bc_ctg1_3571	B2	1211.590587	Bcer	P	Bcer	FALSE	Bc_Ga0417192_01_rn24	Transport	SMCOG1000
Bc_ctg1_3572	B2	859.3609808	Bcer	E	Bcer	FALSE	Bc_Ga0417192_01_rn24	Transport	SMCOG1000
Bc_ctg1_3573	B2	0	Bcer	L	Bcer	FALSE	NA	NA	NA
Bc_ctg1_3574	B2	0	Bcer	L	Bcer	FALSE	NA	NA	NA
Bc_ctg1_3575	B2	9.557520742	Bcer	V	Bcer	FALSE	NA	NA	NA
Bc_ctg1_3576	B2	808.1932783	Bcer	E	Bcer	FALSE	NA	NA	NA
Bc_ctg1_3577	B2	3.263543668	Bcer	NA	Bcer	FALSE	NA	NA	NA
Bc_ctg1_3578	B2	1334.439695	Bcer	K	Bcer	FALSE	NA	NA	NA
Bc_ctg1_3579	B2	0	Bcer	P	Bcer	FALSE	NA	NA	NA
Bc_ctg1_358	B2	0.815885917	Bcer	NA	Bcer	FALSE	Bc_Ga0417192_01_rn04	Other	NA
Bc_ctg1_3580	B2	133.1059596	Bcer	NOT	Bcer	FALSE	NA	NA	NA
Bc_ctg1_3581	B2	51.5173679	Bcer	I	Bcer	FALSE	NA	NA	NA
Bc_ctg1_3582	B2	2.680768013	Bcer	S	Bcer	FALSE	NA	NA	NA
Bc_ctg1_3583	B2	589.5358526	Bcer	E	Bcer	FALSE	NA	NA	NA
Bc_ctg1_3584	B2	38.69630349	Bcer	Q	Bcer	TRUE	Bc_Ga0417192_01_rn25	Other	NA
Bc_ctg1_3585	B2	13.1707298	Bcer	S	Bcer	FALSE	Bc_Ga0417192_01_rn25	Other	NA
Bc_ctg1_3586	B2	11.77206823	Bcer	S	Bcer	FALSE	Bc_Ga0417192_01_rn25	Other	NA
Bc_ctg1_3587	B2	34.03409825	Bcer	S	Bcer	FALSE	Bc_Ga0417192_01_rn25	Other	NA
Bc_ctg1_3588	B2	240.4532352	Bcer	S	Bcer	FALSE	Bc_Ga0417192_01_rn25	Other	NA
Bc_ctg1_3589	B2	265.162923	Bcer	G	Bcer	FALSE	Bc_Ga0417192_01_rn25	Other	NA
Bc_ctg1_359	B2	1.981437227	Bcer	J	Bcer	FALSE	Bc_Ga0417192_01_rn04	Ancillary biosynthesis	SMCOG1111
Bc_ctg1_3590	B2	231.7116004	Bcer	J	Bcer	FALSE	Bc_Ga0417192_01_rn25	Ancillary biosynthesis	SMCOG1279
Bc_ctg1_3591	B2	1.16555131	Bcer	UW	Bcer	FALSE	Bc_Ga0417192_01_rn25	Other	NA
Bc_ctg1_3592	B2	4.079429585	Bcer	T	Bcer	FALSE	Bc_Ga0417192_01_rn25	Other	NA
Bc_ctg1_3593	B2	0.466220524	Bcer	D	Bcer	FALSE	Bc_Ga0417192_01_rn25	Other	NA
Bc_ctg1_3594	B2	0.233110262	Bcer	M	Bcer	FALSE	Bc_Ga0417192_01_rn25	Core biosynthesis	None
Bc_ctg1_3595	B2	0.466220524	Bcer	H	Bcer	FALSE	Bc_Ga0417192_01_rn25	Other	NA
Bc_ctg1_3596	B2	0	Bcer	S	Bcer	FALSE	Bc_Ga0417192_01_rn25	Other	NA
Bc_ctg1_3597	B2	0.699330786	Bcer	K	Bcer	FALSE	Bc_Ga0417192_01_rn25	Other	NA
Bc_ctg1_3598	B2	2.33110262	Bcer	M	Bcer	FALSE	Bc_Ga0417192_01_rn25	Core biosynthesis	None
Bc_ctg1_3599	B2	0.932441048	Bcer	H	Bcer	FALSE	Bc_Ga0417192_01_rn25	Other	NA
Bc_ctg1_36	B2	1.398661572	Bcer	K	Bcer	FALSE	NA	NA	NA
Bc_ctg1_360	B2	13.1707298	Bcer	P	Bcer	FALSE	Bc_Ga0417192_01_rn04	Transport	SMCOG1033
Bc_ctg1_3600	B2	1.631771834	Bcer	S	Bcer	FALSE	Bc_Ga0417192_01_rn25	Other	NA
Bc_ctg1_3601	B2	1.631771834	Bcer	S	Bcer	FALSE	Bc_Ga0417192_01_rn25	Other	NA
Bc_ctg1_3602	B2	0.932441048	Bcer	M	Bcer	FALSE	Bc_Ga0417192_01_rn25	Ancillary biosynthesis	SMCOG1064
Bc_ctg1_3603	B2	1.864882096	Bcer	M	Bcer	FALSE	Bc_Ga0417192_01_rn25	Core biosynthesis	None
Bc_ctg1_3604	B2	5.944311681	Bcer	M	Bcer	FALSE	Bc_Ga0417192_01_rn25	Core biosynthesis	SMCOG1010
Bc_ctg1_3605	B2	4.195984716	Bcer	M	Bcer	FALSE	Bc_Ga0417192_01_rn25	Core biosynthesis	SMCOG1010
Bc_ctg1_3606	B2	350.597834	Bcer	I	Bcer	FALSE	Bc_Ga0417192_01_rn25	Ancillary biosynthesis	SMCOG1001
Bc_ctg1_3607	B2	0.466220524	Bcer	S	Bcer	FALSE	Bc_Ga0417192_01_rn25	Other	NA
Bc_ctg1_3608	B2	0	Bcer	S	Bcer	FALSE	Bc_Ga0417192_01_rn25	Other	NA
Bc_ctg1_3609	B2	0.116555131	Bcer	S	Bcer	FALSE	Bc_Ga0417192_01_rn25	Other	NA
Bc_ctg1_361	B2	7.226418122	Bcer	P	Bcer	FALSE	Bc_Ga0417192_01_rn04	Transport	SMCOG1011
Bc_ctg1_3610	B2	0.349665393	Bcer	NA	Bcer	FALSE	Bc_Ga0417192_01_rn25	Other	NA
Bc_ctg1_3611	B2	8.508524563	Bcer	S	Bcer	FALSE	Bc_Ga0417192_01_rn25	Other	NA
Bc_ctg1_3612	B2	135.203952	Bcer	L	Bcer	FALSE	Bc_Ga0417192_01_rn25	Other	NA
Bc_ctg1_3613	B2	319.4776141	Bcer	S	Bcer	FALSE	Bc_Ga0417192_01_rn25	Other	NA
Bc_ctg1_3614	B2	343.7210813	Bcer	J	Bcer	FALSE	Bc_Ga0417192_01_rn25	Other	NA
Bc_ctg1_3615	B2	51.40081277	Bcer	P	Bcer	FALSE	Bc_Ga0417192_01_rn25	Ancillary biosynthesis	SMCOG1098
Bc_ctg1_3616	B2	95.69176255	Bcer	EG	Bcer	FALSE	Bc_Ga0417192_01_rn25	Transport	SMCOG1166
Bc_ctg1_3617	B2	0.349665393	Bcer	S	Bcer	FALSE	Bc_Ga0417192_01_rn25	Other	NA
Bc_ctg1_3618	B2	1.631771834	Bcer	N	Bcer	FALSE	NA	NA	NA
Bc_ctg1_3619	B2	56.17957314	Bcer	E	Bcer	FALSE	NA	NA	NA

Bc_ctg1_362	B2	9.091300218	Bcer	P	Bcer	FALSE	Bc_Ga0417192_01_rn04	Transport	SMCOG1011
Bc_ctg1_3620	B2	4.545650109	Bcer	EH	Bcer	FALSE	NA	NA	NA
Bc_ctg1_3621	B2	2.097992358	Bcer	EH	Bcer	FALSE	NA	NA	NA
Bc_ctg1_3622	B2	5.478091157	Bcer	E	Bcer	FALSE	NA	NA	NA
Bc_ctg1_3623	B2	1.398661572	Bcer	E	Bcer	FALSE	NA	NA	NA
Bc_ctg1_3624	B2	1.631771834	Bcer	E	Bcer	FALSE	NA	NA	NA
Bc_ctg1_3625	B2	5.594646288	Bcer	E	Bcer	FALSE	NA	NA	NA
Bc_ctg1_3626	B2	2.33110262	Bcer	E	Bcer	FALSE	Bc_Ga0417192_01_rn26	Other	NA
Bc_ctg1_3627	B2	1.515216703	Bcer	NA	Bcer	FALSE	Bc_Ga0417192_01_rn26	Other	NA
Bc_ctg1_3628	B2	4.195984716	Bcer	C	Bcer	FALSE	Bc_Ga0417192_01_rn26	Other	NA
Bc_ctg1_3629	B2	17.59982478	Bcer	S	Bcer	FALSE	Bc_Ga0417192_01_rn26	Other	NA
Bc_ctg1_363	B2	2.564212882	Bcer	S	Bcer	FALSE	Bc_Ga0417192_01_rn04	Other	NA
Bc_ctg1_3630	B2	12.82106441	Bcer	NA	Bcer	FALSE	Bc_Ga0417192_01_rn26	Other	NA
Bc_ctg1_3631	B2	0.815885917	Bcer	M	Bcer	FALSE	Bc_Ga0417192_01_rn26	Other	NA
Bc_ctg1_3632	B2	1.864882096	Bcer	S	Bcer	FALSE	Bc_Ga0417192_01_rn26	Other	NA
Bc_ctg1_3633	B2	0	Bcer	NA	Bcer	FALSE	Bc_Ga0417192_01_rn26	Other	NA
Bc_ctg1_3634	B2	13.87006059	Bcer	P	Bcer	FALSE	Bc_Ga0417192_01_rn26	Ancillary biosynthesis	SMCOG1175
Bc_ctg1_3635	B2	1.515216703	Bcer	S	Bcer	FALSE	Bc_Ga0417192_01_rn26	Other	NA
Bc_ctg1_3636	B2	5.361536026	Bcer	NA	Bcer	FALSE	Bc_Ga0417192_01_rn26	Other	NA
Bc_ctg1_3637	B2	1.864882096	Bcer	C	Bcer	FALSE	Bc_Ga0417192_01_rn26	Core biosynthesis	None
Bc_ctg1_3638	B2	0.932441048	Bcer	S	Bcer	FALSE	Bc_Ga0417192_01_rn26	Core biosynthesis	None
Bc_ctg1_3639	B2	1.864882096	Bcer	H	Bcer	FALSE	Bc_Ga0417192_01_rn26	Core biosynthesis	None
Bc_ctg1_364	B2	14.33628111	Bcer	S	Bcer	FALSE	Bc_Ga0417192_01_rn04	Other	NA
Bc_ctg1_3640	B2	1225.460647	Bcer	C	Bcer	FALSE	Bc_Ga0417192_01_rn26	Other	NA
Bc_ctg1_3641	B2	2147.994509	Bcer	C	Bcer	FALSE	Bc_Ga0417192_01_rn26	Other	NA
Bc_ctg1_3642	B2	0.932441048	Bcer	NA	Bcer	FALSE	Bc_Ga0417192_01_rn26	Other	NA
Bc_ctg1_3643	B2	0.466220524	Bcer	NA	Bcer	FALSE	Bc_Ga0417192_01_rn26	Other	NA
Bc_ctg1_3644	B2	2.33110262	Bcer	NA	Bcer	FALSE	Bc_Ga0417192_01_rn26	Other	NA
Bc_ctg1_3645	B2	5.711201419	Bcer	NA	Bcer	FALSE	Bc_Ga0417192_01_rn26	Other	NA
Bc_ctg1_3646	B2	130.8914121	Bcer	NA	Bcer	FALSE	Bc_Ga0417192_01_rn26	Other	NA
Bc_ctg1_3647	B2	0.233110262	Bcer	NA	Bcer	FALSE	NA	NA	NA
Bc_ctg1_3648	B2	3.729764192	Bcer	NA	Bcer	FALSE	NA	NA	NA
Bc_ctg1_3649	B2	65.0377631	Bcer	M	Bcer	FALSE	NA	NA	NA
Bc_ctg1_365	B2	52.21669869	Bcer	S	Bcer	FALSE	Bc_Ga0417192_01_rn04	Other	NA
Bc_ctg1_3650	B2	0.815885917	Bcer	U	Bcer	FALSE	NA	NA	NA
Bc_ctg1_3651	B2	0.582775655	Bcer	S	Bcer	FALSE	NA	NA	NA
Bc_ctg1_3652	B2	0.699330786	Bcer	M	Bcer	FALSE	NA	NA	NA
Bc_ctg1_3653	B2	3.846319323	Bcer	S	Bcer	FALSE	NA	NA	NA
Bc_ctg1_3654	B2	68.65097216	Bcer	K	Bcer	FALSE	NA	NA	NA
Bc_ctg1_3655	B2	0	Bcer	NA	Bcer	FALSE	NA	NA	NA
Bc_ctg1_3656	B2	11.07273744	Bcer	V	Bcer	FALSE	NA	NA	NA
Bc_ctg1_3657	B2	59.90933733	Bcer	C	Bcer	FALSE	NA	NA	NA
Bc_ctg1_3658	B2	50.81803711	Bcer	P	Bcer	FALSE	NA	NA	NA
Bc_ctg1_3659	B2	39.16252402	Bcer	E	Bcer	FALSE	NA	NA	NA
Bc_ctg1_366	B2	6.410532205	Bcer	S	Bcer	FALSE	Bc_Ga0417192_01_rn04	Other	NA
Bc_ctg1_3660	B2	32.40232642	Bcer	E	Bcer	FALSE	NA	NA	NA
Bc_ctg1_3661	B2	83.80313919	Bcer	P	Bcer	FALSE	NA	NA	NA
Bc_ctg1_3662	B2	11.07273744	Bcer	I	Bcer	FALSE	NA	NA	NA
Bc_ctg1_3663	B2	144.994583	Bcer	S	Bcer	FALSE	NA	NA	NA
Bc_ctg1_3664	B2	0.116555131	Bcer	S	Bcer	FALSE	NA	NA	NA
Bc_ctg1_3665	B2	35.43275982	Bcer	J	Bcer	FALSE	NA	NA	NA
Bc_ctg1_3666	B2	53.61536026	Bcer	S	Bcer	FALSE	NA	NA	NA
Bc_ctg1_3667	B2	1.864882096	Bcer	J	Bcer	FALSE	NA	NA	NA
Bc_ctg1_3668	B2	72.14762609	Bcer	C	Bcer	FALSE	NA	NA	NA
Bc_ctg1_3669	B2	87.41634825	Bcer	C	Bcer	FALSE	NA	NA	NA
Bc_ctg1_367	B2	0.233110262	Bcer	S	Bcer	FALSE	Bc_Ga0417192_01_rn04	Other	NA
Bc_ctg1_3670	B2	2.33110262	Bcer	KT	Bcer	FALSE	NA	NA	NA
Bc_ctg1_3671	B2	14.45283624	Bcer	S	Bcer	FALSE	NA	NA	NA
Bc_ctg1_3672	B2	6.177421943	Bcer	T	Bcer	FALSE	NA	NA	NA
Bc_ctg1_3673	B2	43.00884334	Bcer	T	Bcer	FALSE	NA	NA	NA
Bc_ctg1_3674	B2	532.1907281	Bcer	K	Bcer	FALSE	NA	NA	NA
Bc_ctg1_3675	B2	203.6218139	Bcer	C	Bcer	FALSE	NA	NA	NA

Bc_ctg1_3676	B2	732.8986637	Bcer	C	Bcer	FALSE	NA	NA	NA
Bc_ctg1_3677	B2	297.6818046	Bcer	S	Bcer	FALSE	NA	NA	NA
Bc_ctg1_3678	B2	32.98510207	Bcer	NOU	Bcer	FALSE	NA	NA	NA
Bc_ctg1_3679	B2	8.508524563	Bcer	S	Bcer	FALSE	NA	NA	NA
Bc_ctg1_368	B2	0	Bcer	S	Bcer	FALSE	Bc_Ga0417192_01_rn04	Other	NA
Bc_ctg1_3680	B2	503.9843864	Bcer	P	Bcer	FALSE	NA	NA	NA
Bc_ctg1_3681	B2	13.1707298	Bcer	P	Bcer	FALSE	NA	NA	NA
Bc_ctg1_3682	B2	0.815885917	Bcer	P	Bcer	FALSE	NA	NA	NA
Bc_ctg1_3683	B2	0.699330786	Bcer	S	Bcer	FALSE	NA	NA	NA
Bc_ctg1_3684	B2	324.3729296	Bcer	I	Bcer	FALSE	NA	NA	NA
Bc_ctg1_3685	B2	719.0286031	Bcer	S	Bcer	FALSE	NA	NA	NA
Bc_ctg1_3686	B2	267.9602462	Bcer	K	Bcer	FALSE	NA	NA	NA
Bc_ctg1_3687	B2	94.64276637	Bcer	S	Bcer	FALSE	NA	NA	NA
Bc_ctg1_3688	B2	141.7310393	Bcer	IQ	Bcer	TRUE	NA	NA	NA
Bc_ctg1_3689	B2	140.0992675	Bcer	I	Bcer	FALSE	NA	NA	NA
Bc_ctg1_369	B2	3.613209061	Bcer	K	Bcer	FALSE	Bc_Ga0417192_01_rn04	Regulation	SMCOG1014
Bc_ctg1_3690	B2	23.54413646	Bcer	E	Bcer	FALSE	NA	NA	NA
Bc_ctg1_3691	B2	84.85213537	Bcer	P	Bcer	FALSE	NA	NA	NA
Bc_ctg1_3694	B2	0.116555131	Bcer	P	Bcer	FALSE	NA	NA	NA
Bc_ctg1_3695	B2	1.048996179	Bcer	P	Bcer	FALSE	NA	NA	NA
Bc_ctg1_3696	B2	1.282106441	Bcer	P	Bcer	FALSE	NA	NA	NA
Bc_ctg1_3697	B2	1.048996179	Bcer	S	Bcer	FALSE	NA	NA	NA
Bc_ctg1_3698	B2	0.233110262	Bcer	E	Bcer	FALSE	NA	NA	NA
Bc_ctg1_3699	B2	3.729764192	Bcer	E	Bcer	FALSE	NA	NA	NA
Bc_ctg1_37	B2	0.582775655	Bcer	S	Bcer	FALSE	NA	NA	NA
Bc_ctg1_370	B2	1.16555131	Bcer	C	Bcer	FALSE	Bc_Ga0417192_01_rn04	Ancillary biosynthesis	SMCOG1028
Bc_ctg1_3700	B2	3.030433406	Bcer	K	Bcer	FALSE	NA	NA	NA
Bc_ctg1_3702	B2	174.4830311	Bcer	M	Bcer	FALSE	NA	NA	NA
Bc_ctg1_3703	B2	0.699330786	Bcer	E	Bcer	FALSE	NA	NA	NA
Bc_ctg1_3704	B2	3.263543668	Bcer	S	Bcer	FALSE	NA	NA	NA
Bc_ctg1_3705	B2	1.398661572	Bcer	S	Bcer	FALSE	NA	NA	NA
Bc_ctg1_3706	B2	1.048996179	Bcer	S	Bcer	FALSE	NA	NA	NA
Bc_ctg1_3707	B2	32.28577129	Bcer	T	Bcer	FALSE	NA	NA	NA
Bc_ctg1_3708	B2	15.73494268	Bcer	K	Bcer	FALSE	NA	NA	NA
Bc_ctg1_3709	B2	745.2535076	Bcer	E	Bcer	FALSE	NA	NA	NA
Bc_ctg1_371	B2	7.226418122	Bcer	G	Bcer	FALSE	Bc_Ga0417192_01_rn04	Ancillary biosynthesis	SMCOG1101
Bc_ctg1_3710	B2	57.11201419	Bcer	S	Bcer	FALSE	NA	NA	NA
Bc_ctg1_3711	B2	0	Bcer	NA	Bcer	FALSE	NA	NA	NA
Bc_ctg1_3712	B2	92.42821888	Bcer	T	Bcer	FALSE	NA	NA	NA
Bc_ctg1_3713	B2	56.06301801	Bcer	F	Bcer	FALSE	NA	NA	NA
Bc_ctg1_3714	B2	62.93977074	Bcer	H	Bcer	FALSE	NA	NA	NA
Bc_ctg1_3715	B2	51.75047816	Bcer	H	Bcer	FALSE	NA	NA	NA
Bc_ctg1_3716	B2	49.53593067	Bcer	S	Bcer	FALSE	NA	NA	NA
Bc_ctg1_3717	B2	3.146988537	Bcer	S	Bcer	FALSE	NA	NA	NA
Bc_ctg1_3718	B2	0.349665393	Bcer	NA	Bcer	FALSE	NA	NA	NA
Bc_ctg1_3719	B2	39.16252402	Bcer	G	Bcer	FALSE	NA	NA	NA
Bc_ctg1_372	B2	1.515216703	Bcer	S	Bcer	FALSE	Bc_Ga0417192_01_rn04	Other	NA
Bc_ctg1_3720	B2	93.47721506	Bcer	K	Bcer	FALSE	NA	NA	NA
Bc_ctg1_3721	B2	3.613209061	Bcer	NA	Bcer	FALSE	NA	NA	NA
Bc_ctg1_3722	B2	17.71637991	Bcer	NA	Bcer	FALSE	NA	NA	NA
Bc_ctg1_3723	B2	62.00732969	Bcer	F	Bcer	FALSE	NA	NA	NA
Bc_ctg1_3724	B2	909.5962423	Bcer	F	Bcer	FALSE	NA	NA	NA
Bc_ctg1_3725	B2	156.0673204	Bcer	F	Bcer	FALSE	NA	NA	NA
Bc_ctg1_3726	B2	322.1583821	Bcer	L	Bcer	FALSE	NA	NA	NA
Bc_ctg1_3727	B2	119.5855644	Bcer	F	Bcer	FALSE	NA	NA	NA
Bc_ctg1_3728	B2	14.21972598	Bcer	K	Bcer	FALSE	NA	NA	NA
Bc_ctg1_3729	B2	27.97323144	Bcer	V	Bcer	FALSE	NA	NA	NA
Bc_ctg1_373	B2	3.962874454	Bcer	L	Bcer	FALSE	Bc_Ga0417192_01_rn04	Other	NA
Bc_ctg1_3730	B2	16.20116321	Bcer	NA	Bcer	FALSE	NA	NA	NA
Bc_ctg1_3731	B2	74.36217358	Bcer	V	Bcer	FALSE	NA	NA	NA
Bc_ctg1_3732	B2	90.21367139	Bcer	S	Bcer	FALSE	NA	NA	NA
Bc_ctg1_3733	B2	32.86854694	Bcer	S	Bcer	FALSE	NA	NA	NA

Bc_ctg1_3734	B2	0.116555131	Bcer	NA	Bcer	FALSE	NA	NA	NA
Bc_ctg1_3735	B2	4.66220524	Bcer	K	Bcer	FALSE	NA	NA	NA
Bc_ctg1_3736	B2	2.097992358	Bcer	S	Bcer	FALSE	NA	NA	NA
Bc_ctg1_3737	B2	3.030433406	Bcer	Q	Bcer	TRUE	NA	NA	NA
Bc_ctg1_3738	B2	34.61687391	Bcer	S	Bcer	FALSE	NA	NA	NA
Bc_ctg1_3739	B2	5.244980895	Bcer	V	Bcer	FALSE	NA	NA	NA
Bc_ctg1_374	B2	0.116555131	Bcer	NA	Bcer	FALSE	Bc_Ga0417192_01_rn04	Other	NA
Bc_ctg1_3740	B2	11.42240284	Bcer	S	Bcer	FALSE	NA	NA	NA
Bc_ctg1_3741	B2	263.9973717	Bcer	M	Bcer	FALSE	NA	NA	NA
Bc_ctg1_3742	B2	92.77788427	Bcer	IQ	Bcer	TRUE	NA	NA	NA
Bc_ctg1_3743	B2	543.1469104	Bcer	M	Bcer	FALSE	NA	NA	NA
Bc_ctg1_3744	B2	824.2778864	Bcer	Q	Bcer	TRUE	NA	NA	NA
Bc_ctg1_3745	B2	2.33110262	Bcer	NA	Bcer	FALSE	NA	NA	NA
Bc_ctg1_3746	B2	0.582775655	Bcer	E	Bcer	FALSE	NA	NA	NA
Bc_ctg1_3747	B2	0.349665393	Bcer	NA	Bcer	FALSE	NA	NA	NA
Bc_ctg1_3748	B2	5.82775655	Bcer	C	Bcer	FALSE	NA	NA	NA
Bc_ctg1_3749	B2	10.95618231	Bcer	NA	Bcer	FALSE	NA	NA	NA
Bc_ctg1_375	B2	7.576083515	Bcer	G	Bcer	FALSE	Bc_Ga0417192_01_rn04	Core biosynthesis	SMCOG1257
Bc_ctg1_3750	B2	0	Bcer	NA	Bcer	FALSE	NA	NA	NA
Bc_ctg1_3751	B2	3.030433406	Bcer	S	Bcer	FALSE	NA	NA	NA
Bc_ctg1_3752	B2	1.282106441	Bcer	NA	Bcer	FALSE	NA	NA	NA
Bc_ctg1_3754	B2	0.466220524	Bcer	NA	Bcer	FALSE	NA	NA	NA
Bc_ctg1_3755	B2	9.091300218	Bcer	V	Bcer	FALSE	NA	NA	NA
Bc_ctg1_3756	B2	217.7249847	Bcer	V	Bcer	FALSE	NA	NA	NA
Bc_ctg1_3757	B2	66.5529798	Bcer	CO	Bcer	FALSE	NA	NA	NA
Bc_ctg1_3758	B2	6.760197598	Bcer	S	Bcer	FALSE	NA	NA	NA
Bc_ctg1_3759	B2	16.78393886	Bcer	K	Bcer	FALSE	NA	NA	NA
Bc_ctg1_376	B2	8.858189956	Bcer	Q	Bcer	TRUE	Bc_Ga0417192_01_rn04	Other	NA
Bc_ctg1_3761	B2	9.907186135	Bcer	C	Bcer	FALSE	NA	NA	NA
Bc_ctg1_3762	B2	294.8844814	Bcer	P	Bcer	FALSE	NA	NA	NA
Bc_ctg1_3763	B2	1.398661572	Bcer	NA	Bcer	FALSE	NA	NA	NA
Bc_ctg1_3764	B2	0.466220524	Bcer	S	Bcer	FALSE	NA	NA	NA
Bc_ctg1_3766	B2	0.582775655	Bcer	S	Bcer	FALSE	NA	NA	NA
Bc_ctg1_3767	B2	2.214547489	Bcer	S	Bcer	FALSE	NA	NA	NA
Bc_ctg1_3768	B2	12.47139902	Bcer	E	Bcer	FALSE	NA	NA	NA
Bc_ctg1_3769	B2	11.88862336	Bcer	E	Bcer	FALSE	NA	NA	NA
Bc_ctg1_377	B2	118.6531234	Bcer	E	Bcer	FALSE	Bc_Ga0417192_01_rn04	Other	NA
Bc_ctg1_3770	B2	0.466220524	Bcer	E	Bcer	FALSE	NA	NA	NA
Bc_ctg1_3771	B2	3.846319323	Bcer	EH	Bcer	FALSE	NA	NA	NA
Bc_ctg1_3772	B2	6.99330786	Bcer	E	Bcer	FALSE	NA	NA	NA
Bc_ctg1_3773	B2	1.515216703	Bcer	CE	Bcer	FALSE	NA	NA	NA
Bc_ctg1_3774	B2	1.981437227	Bcer	E	Bcer	FALSE	NA	NA	NA
Bc_ctg1_3775	B2	0.466220524	Bcer	E	Bcer	FALSE	NA	NA	NA
Bc_ctg1_3776	B2	6.99330786	Bcer	E	Bcer	FALSE	NA	NA	NA
Bc_ctg1_3777	B2	4.66220524	Bcer	E	Bcer	FALSE	NA	NA	NA
Bc_ctg1_3778	B2	10.37340666	Bcer	E	Bcer	FALSE	NA	NA	NA
Bc_ctg1_3779	B2	0.582775655	Bcer	E	Bcer	FALSE	NA	NA	NA
Bc_ctg1_378	B2	0	Bcer	E	Bcer	FALSE	Bc_Ga0417192_01_rn04	Other	NA
Bc_ctg1_3780	B2	1.981437227	Bcer	E	Bcer	FALSE	NA	NA	NA
Bc_ctg1_3781	B2	2.214547489	Bcer	E	Bcer	FALSE	NA	NA	NA
Bc_ctg1_3782	B2	1.981437227	Bcer	E	Bcer	FALSE	NA	NA	NA
Bc_ctg1_3783	B2	0.699330786	Bcer	E	Bcer	FALSE	NA	NA	NA
Bc_ctg1_3784	B2	1.515216703	Bcer	E	Bcer	FALSE	NA	NA	NA
Bc_ctg1_3785	B2	11.07273744	Bcer	E	Bcer	FALSE	NA	NA	NA
Bc_ctg1_3786	B2	0.815885917	Bcer	NA	Bcer	FALSE	NA	NA	NA
Bc_ctg1_3787	B2	108.6293821	Bcer	CH	Bcer	FALSE	NA	NA	NA
Bc_ctg1_3788	B2	88.3487893	Bcer	I	Bcer	FALSE	NA	NA	NA
Bc_ctg1_3789	B2	155.3679896	Bcer	M	Bcer	FALSE	NA	NA	NA
Bc_ctg1_379	B2	2.797323144	Bcer	K	Bcer	FALSE	Bc_Ga0417192_01_rn04	Regulation	SMCOG1171
Bc_ctg1_3790	B2	0.466220524	Bcer	S	Bcer	FALSE	NA	NA	NA
Bc_ctg1_3791	B2	86.83357259	Bcer	E	Bcer	FALSE	NA	NA	NA
Bc_ctg1_3792	B2	0.116555131	Bcer	S	Bcer	FALSE	NA	NA	NA

Bc_ctg1_3793	B2	18.4157107	Bcer	EH	Bcer	FALSE	NA	NA	NA
Bc_ctg1_3794	B2	56.64579366	Bcer	P	Bcer	FALSE	NA	NA	NA
Bc_ctg1_3795	B2	28.32289683	Bcer	P	Bcer	FALSE	NA	NA	NA
Bc_ctg1_3796	B2	123.5484389	Bcer	P	Bcer	FALSE	NA	NA	NA
Bc_ctg1_3797	B2	12.12173362	Bcer	S	Bcer	FALSE	NA	NA	NA
Bc_ctg1_3798	B2	6.527087336	Bcer	H	Bcer	FALSE	NA	NA	NA
Bc_ctg1_3799	B2	17.94949017	Bcer	S	Bcer	FALSE	NA	NA	NA
Bc_ctg1_38	B2	0	Bcer	S	Bcer	FALSE	NA	NA	NA
Bc_ctg1_380	B2	1.048996179	Bcer	E	Bcer	FALSE	Bc_Ga0417192_01_rn04	Other	NA
Bc_ctg1_3800	B2	13.87006059	Bcer	H	Bcer	FALSE	NA	NA	NA
Bc_ctg1_3801	B2	20.74681332	Bcer	S	Bcer	FALSE	NA	NA	NA
Bc_ctg1_3802	B2	0	Bcer	NA	Bcer	FALSE	NA	NA	NA
Bc_ctg1_3803	B2	288.4739492	Bcer	D	Bcer	FALSE	NA	NA	NA
Bc_ctg1_3804	B2	705.3916528	Bcer	C	Bcer	FALSE	NA	NA	NA
Bc_ctg1_3805	B2	4.66220524	Bcer	S	Bcer	FALSE	NA	NA	NA
Bc_ctg1_3806	B2	35.31620469	Bcer	C	Bcer	FALSE	NA	NA	NA
Bc_ctg1_3807	B2	62.47355021	Bcer	NA	Bcer	FALSE	NA	NA	NA
Bc_ctg1_3808	B2	214.1117756	Bcer	KT	Bcer	FALSE	NA	NA	NA
Bc_ctg1_3809	B2	19.23159661	Bcer	S	Bcer	FALSE	NA	NA	NA
Bc_ctg1_381	B2	0	Bcer	S	Bcer	FALSE	Bc_Ga0417192_01_rn04	Other	NA
Bc_ctg1_3810	B2	36.71486626	Bcer	T	Bcer	FALSE	NA	NA	NA
Bc_ctg1_3811	B2	40.44463046	Bcer	KT	Bcer	FALSE	NA	NA	NA
Bc_ctg1_3812	B2	14.33628111	Bcer	NA	Bcer	FALSE	NA	NA	NA
Bc_ctg1_3813	B2	13.05417467	Bcer	E	Bcer	FALSE	NA	NA	NA
Bc_ctg1_3814	B2	50.46837172	Bcer	NA	Bcer	FALSE	NA	NA	NA
Bc_ctg1_3815	B2	11.88862336	Bcer	Q	Bcer	TRUE	NA	NA	NA
Bc_ctg1_3816	B2	14.80250164	Bcer	NA	Bcer	FALSE	NA	NA	NA
Bc_ctg1_3818	B2	12.12173362	Bcer	S	Bcer	FALSE	NA	NA	NA
Bc_ctg1_3819	B2	1.515216703	Bcer	NA	Bcer	FALSE	NA	NA	NA
Bc_ctg1_382	B2	1.282106441	Bcer	E	Bcer	FALSE	Bc_Ga0417192_01_rn04	Other	NA
Bc_ctg1_3820	B2	441.277726	Bcer	C	Bcer	FALSE	NA	NA	NA
Bc_ctg1_3821	B2	15.73494268	Bcer	T	Bcer	FALSE	NA	NA	NA
Bc_ctg1_3822	B2	1.398661572	Bcer	NA	Bcer	FALSE	NA	NA	NA
Bc_ctg1_3823	B2	126.1126517	Bcer	S	Bcer	FALSE	NA	NA	NA
Bc_ctg1_3824	B2	566.3413815	Bcer	P	Bcer	FALSE	NA	NA	NA
Bc_ctg1_3825	B2	279.2660939	Bcer	P	Bcer	FALSE	NA	NA	NA
Bc_ctg1_3826	B2	21.21303384	Bcer	M	Bcer	FALSE	NA	NA	NA
Bc_ctg1_3827	B2	0	Bcer	NA	Bcer	FALSE	NA	NA	NA
Bc_ctg1_3828	B2	0	Bcer	NA	Bcer	FALSE	NA	NA	NA
Bc_ctg1_3829	B2	33.68443286	Bcer	T	Bcer	FALSE	NA	NA	NA
Bc_ctg1_383	B2	79.95681986	Bcer	V	Bcer	FALSE	Bc_Ga0417192_01_rn04	Ancillary biosynthesis	SMCOG1053
Bc_ctg1_3830	B2	8.508524563	Bcer	S	Bcer	FALSE	NA	NA	NA
Bc_ctg1_3831	B2	17.36671452	Bcer	S	Bcer	FALSE	NA	NA	NA
Bc_ctg1_3832	B2	665.6463531	Bcer	L	Bcer	FALSE	NA	NA	NA
Bc_ctg1_3833	B2	211.5475628	Bcer	T	Bcer	FALSE	NA	NA	NA
Bc_ctg1_3834	B2	310.3863138	Bcer	F	Bcer	FALSE	NA	NA	NA
Bc_ctg1_3835	B2	18.64882096	Bcer	S	Bcer	FALSE	NA	NA	NA
Bc_ctg1_3836	B2	225.5341785	Bcer	S	Bcer	FALSE	NA	NA	NA
Bc_ctg1_3837	B2	4.429094978	Bcer	NA	Bcer	FALSE	NA	NA	NA
Bc_ctg1_3838	B2	8.974745087	Bcer	S	Bcer	FALSE	NA	NA	NA
Bc_ctg1_3839	B2	0.349665393	Bcer	P	Bcer	FALSE	NA	NA	NA
Bc_ctg1_384	B2	0.582775655	Bcer	NA	Bcer	FALSE	NA	NA	NA
Bc_ctg1_3840	B2	0.116555131	Bcer	M	Bcer	FALSE	NA	NA	NA
Bc_ctg1_3841	B2	0.349665393	Bcer	S	Bcer	FALSE	NA	NA	NA
Bc_ctg1_3842	B2	1.048996179	Bcer	S	Bcer	FALSE	NA	NA	NA
Bc_ctg1_3843	B2	145.4608035	Bcer	J	Bcer	FALSE	NA	NA	NA
Bc_ctg1_3844	B2	32.40232642	Bcer	CO	Bcer	FALSE	NA	NA	NA
Bc_ctg1_3845	B2	53.96502565	Bcer	O	Bcer	FALSE	NA	NA	NA
Bc_ctg1_3846	B2	77.97538264	Bcer	O	Bcer	FALSE	NA	NA	NA
Bc_ctg1_3847	B2	175.7651375	Bcer	T	Bcer	FALSE	NA	NA	NA
Bc_ctg1_3848	B2	363.7685638	Bcer	T	Bcer	FALSE	NA	NA	NA
Bc_ctg1_3849	B2	4.079429585	Bcer	S	Bcer	FALSE	NA	NA	NA

Bc_ctg1_385	B2	2.097992358	Bcer	G	Bcer	FALSE	NA	NA	NA
Bc_ctg1_3850	B2	1.16555131	Bcer	M	Bcer	FALSE	NA	NA	NA
Bc_ctg1_3851	B2	102.5685153	Bcer	M	Bcer	FALSE	NA	NA	NA
Bc_ctg1_3852	B2	98.1394203	Bcer	U	Bcer	FALSE	NA	NA	NA
Bc_ctg1_3853	B2	112.0094809	Bcer	C	Bcer	FALSE	NA	NA	NA
Bc_ctg1_3854	B2	26.22490447	Bcer	S	Bcer	FALSE	NA	NA	NA
Bc_ctg1_3855	B2	57.22856932	Bcer	L	Bcer	FALSE	NA	NA	NA
Bc_ctg1_3856	B2	47.55449345	Bcer	S	Bcer	FALSE	NA	NA	NA
Bc_ctg1_3857	B2	37.53075218	Bcer	M	Bcer	FALSE	NA	NA	NA
Bc_ctg1_3858	B2	121.916667	Bcer	S	Bcer	FALSE	NA	NA	NA
Bc_ctg1_3859	B2	174.2499208	Bcer	K	Bcer	FALSE	NA	NA	NA
Bc_ctg1_386	B2	7.925748908	Bcer	K	Bcer	FALSE	NA	NA	NA
Bc_ctg1_3860	B2	160.0301949	Bcer	NOT	Bcer	FALSE	NA	NA	NA
Bc_ctg1_3861	B2	1977.474352	Bcer	E	Bcer	FALSE	NA	NA	NA
Bc_ctg1_3862	B2	0.116555131	Bcer	NA	Bcer	FALSE	NA	NA	NA
Bc_ctg1_3863	B2	2.33110262	Bcer	S	Bcer	FALSE	NA	NA	NA
Bc_ctg1_3864	B2	3.613209061	Bcer	M	Bcer	FALSE	NA	NA	NA
Bc_ctg1_3865	B2	14.45283624	Bcer	O	Bcer	FALSE	NA	NA	NA
Bc_ctg1_3866	B2	162.2447423	Bcer	EJ	Bcer	FALSE	NA	NA	NA
Bc_ctg1_3867	B2	0	Bcer	S	Bcer	FALSE	NA	NA	NA
Bc_ctg1_3868	B2	78.2084929	Bcer	F	Bcer	FALSE	NA	NA	NA
Bc_ctg1_3869	B2	1502.512194	Bcer	J	Bcer	FALSE	NA	NA	NA
Bc_ctg1_387	B2	1.981437227	Bcer	S	Bcer	FALSE	NA	NA	NA
Bc_ctg1_3870	B2	41.14396124	Bcer	C	Bcer	FALSE	NA	NA	NA
Bc_ctg1_3872	B2	0	Bcer	NA	Bcer	FALSE	NA	NA	NA
Bc_ctg1_3873	B2	0.116555131	Bcer	S	Bcer	FALSE	NA	NA	NA
Bc_ctg1_3875	B2	516.8054508	Bcer	S	Bcer	FALSE	NA	NA	NA
Bc_ctg1_3876	B2	456.429893	Bcer	I	Bcer	FALSE	NA	NA	NA
Bc_ctg1_3877	B2	0.815885917	Bcer	S	Bcer	FALSE	NA	NA	NA
Bc_ctg1_3878	B2	1.048996179	Bcer	S	Bcer	FALSE	NA	NA	NA
Bc_ctg1_3879	B2	9.907186135	Bcer	NA	Bcer	FALSE	NA	NA	NA
Bc_ctg1_388	B2	65.27087336	Bcer	C	Bcer	FALSE	NA	NA	NA
Bc_ctg1_3880	B2	8.391969432	Bcer	S	Bcer	FALSE	NA	NA	NA
Bc_ctg1_3881	B2	879.7581288	Bcer	L	Bcer	FALSE	NA	NA	NA
Bc_ctg1_3882	B2	259.3351665	Bcer	H	Bcer	FALSE	NA	NA	NA
Bc_ctg1_3883	B2	68.18475163	Bcer	H	Bcer	FALSE	NA	NA	NA
Bc_ctg1_3884	B2	73.66284279	Bcer	H	Bcer	FALSE	NA	NA	NA
Bc_ctg1_3885	B2	115.2730246	Bcer	H	Bcer	FALSE	NA	NA	NA
Bc_ctg1_3886	B2	1539.576725	Bcer	F	Bcer	FALSE	NA	NA	NA
Bc_ctg1_3887	B2	77.74227238	Bcer	E	Bcer	FALSE	NA	NA	NA
Bc_ctg1_3888	B2	154.6686588	Bcer	E	Bcer	FALSE	NA	NA	NA
Bc_ctg1_3889	B2	330.6669066	Bcer	E	Bcer	FALSE	NA	NA	NA
Bc_ctg1_389	B2	11.42240284	Bcer	NA	Bcer	FALSE	NA	NA	NA
Bc_ctg1_3890	B2	146.0435791	Bcer	S	Bcer	FALSE	NA	NA	NA
Bc_ctg1_3891	B2	107.1141654	Bcer	S	Bcer	FALSE	Bc_Ga0417192_01_rn27	Other	NA
Bc_ctg1_3892	B2	13.63695033	Bcer	S	Bcer	FALSE	Bc_Ga0417192_01_rn27	Other	NA
Bc_ctg1_3893	B2	16.90049399	Bcer	C	Bcer	FALSE	Bc_Ga0417192_01_rn27	Other	NA
Bc_ctg1_3894	B2	46.50549727	Bcer	C	Bcer	FALSE	Bc_Ga0417192_01_rn27	Other	NA
Bc_ctg1_3895	B2	20.16403766	Bcer	C	Bcer	FALSE	Bc_Ga0417192_01_rn27	Other	NA
Bc_ctg1_3896	B2	19.11504148	Bcer	S	Bcer	FALSE	Bc_Ga0417192_01_rn27	Other	NA
Bc_ctg1_3897	B2	14.80250164	Bcer	S	Bcer	FALSE	Bc_Ga0417192_01_rn27	Other	NA
Bc_ctg1_3898	B2	2.33110262	Bcer	S	Bcer	FALSE	Bc_Ga0417192_01_rn27	Other	NA
Bc_ctg1_3899	B2	304.6751124	Bcer	S	Bcer	FALSE	Bc_Ga0417192_01_rn27	Other	NA
Bc_ctg1_39	B2	2.097992358	Bcer	J	Bcer	FALSE	NA	NA	NA
Bc_ctg1_390	B2	4.895315502	Bcer	NA	Bcer	FALSE	NA	NA	NA
Bc_ctg1_3900	B2	0.699330786	Bcer	L	Bcer	FALSE	Bc_Ga0417192_01_rn27	Other	NA
Bc_ctg1_3901	B2	21.21303384	Bcer	S	Bcer	FALSE	Bc_Ga0417192_01_rn27	Other	NA
Bc_ctg1_3902	B2	3.613209061	Bcer	S	Bcer	FALSE	Bc_Ga0417192_01_rn27	Other	NA
Bc_ctg1_3903	B2	121.8001119	Bcer	E	Bcer	FALSE	Bc_Ga0417192_01_rn27	Other	NA
Bc_ctg1_3904	B2	63.98876692	Bcer	G	Bcer	FALSE	Bc_Ga0417192_01_rn27	Other	NA
Bc_ctg1_3905	B2	88.11567903	Bcer	S	Bcer	FALSE	Bc_Ga0417192_01_rn27	Core biosynthesis	None
Bc_ctg1_3906	B2	161.3123013	Bcer	M	Bcer	FALSE	Bc_Ga0417192_01_rn27	Core biosynthesis	SMCOG1045

Bc_ctg1_3907	B2	208.8667947	Bcer	J	Bcer	FALSE	Bc_Ga0417192_01_rn27	Other	NA
Bc_ctg1_3908	B2	165.1586206	Bcer	K	Bcer	FALSE	Bc_Ga0417192_01_rn27	Other	NA
Bc_ctg1_3909	B2	0	Bcer	C	Bcer	FALSE	Bc_Ga0417192_01_rn27	Other	NA
Bc_ctg1_391	B2	2.33110262	Bcer	S	Bcer	FALSE	NA	NA	NA
Bc_ctg1_3910	B2	63.98876692	Bcer	H	Bcer	FALSE	Bc_Ga0417192_01_rn27	Other	NA
Bc_ctg1_3911	B2	126.1126517	Bcer	H	Bcer	FALSE	Bc_Ga0417192_01_rn27	Other	NA
Bc_ctg1_3912	B2	12.12173362	Bcer	H	Bcer	FALSE	Bc_Ga0417192_01_rn27	Other	NA
Bc_ctg1_3913	B2	338.126435	Bcer	L	Bcer	FALSE	Bc_Ga0417192_01_rn27	Other	SMCOG1194
Bc_ctg1_3914	B2	40.79429585	Bcer	S	Bcer	FALSE	Bc_Ga0417192_01_rn27	Other	NA
Bc_ctg1_3915	B2	91.72888809	Bcer	S	Bcer	FALSE	Bc_Ga0417192_01_rn27	Other	NA
Bc_ctg1_3916	B2	1006.570111	Bcer	E	Bcer	FALSE	Bc_Ga0417192_01_rn27	Core biosynthesis	SMCOG1019
Bc_ctg1_3917	B2	56.17957314	Bcer	L	Bcer	FALSE	Bc_Ga0417192_01_rn27	Other	NA
Bc_ctg1_3918	B2	57.46167958	Bcer	L	Bcer	FALSE	Bc_Ga0417192_01_rn27	Other	NA
Bc_ctg1_3919	B2	20.63025819	Bcer	NA	Bcer	FALSE	Bc_Ga0417192_01_rn27	Other	NA
Bc_ctg1_392	B2	33.91754312	Bcer	NA	Bcer	FALSE	NA	NA	NA
Bc_ctg1_3920	B2	434.8671938	Bcer	M	Bcer	FALSE	Bc_Ga0417192_01_rn27	Other	NA
Bc_ctg1_3921	B2	25.75868395	Bcer	L	Bcer	FALSE	Bc_Ga0417192_01_rn27	Other	NA
Bc_ctg1_3922	B2	2.913878275	Bcer	S	Bcer	FALSE	Bc_Ga0417192_01_rn27	Other	NA
Bc_ctg1_3924	B2	2.447657751	Bcer	NA	Bcer	FALSE	Bc_Ga0417192_01_rn27	Other	NA
Bc_ctg1_3925	B2	2.797323144	Bcer	S	Bcer	FALSE	Bc_Ga0417192_01_rn27	Other	NA
Bc_ctg1_3926	B2	9.32441048	Bcer	S	Bcer	FALSE	Bc_Ga0417192_01_rn27	Other	NA
Bc_ctg1_3927	B2	0.466220524	Bcer	NA	Bcer	FALSE	Bc_Ga0417192_01_rn27	Other	NA
Bc_ctg1_3928	B2	1.048996179	Bcer	S	Bcer	FALSE	Bc_Ga0417192_01_rn27	Other	NA
Bc_ctg1_3929	B2	5.244980895	Bcer	S	Bcer	FALSE	Bc_Ga0417192_01_rn27	Other	NA
Bc_ctg1_393	B2	70.51585425	Bcer	S	Bcer	FALSE	NA	NA	NA
Bc_ctg1_3930	B2	321.8087167	Bcer	D	Bcer	FALSE	NA	NA	NA
Bc_ctg1_3931	B2	136.0198379	Bcer	L	Bcer	FALSE	NA	NA	NA
Bc_ctg1_3932	B2	9.207855349	Bcer	S	Bcer	FALSE	NA	NA	NA
Bc_ctg1_3933	B2	121.4504465	Bcer	KL	Bcer	FALSE	NA	NA	NA
Bc_ctg1_3934	B2	448.2710338	Bcer	E	Bcer	FALSE	NA	NA	NA
Bc_ctg1_3935	B2	0	Bcer	L	Bcer	FALSE	NA	NA	NA
Bc_ctg1_3937	B2	0.815885917	Bcer	J	Bcer	FALSE	NA	NA	NA
Bc_ctg1_3938	B2	18.76537609	Bcer	E	Bcer	FALSE	NA	NA	NA
Bc_ctg1_3939	B2	108.9790475	Bcer	F	Bcer	FALSE	NA	NA	NA
Bc_ctg1_394	B2	40.67774072	Bcer	NA	Bcer	FALSE	NA	NA	NA
Bc_ctg1_3940	B2	682.8965125	Bcer	F	Bcer	FALSE	NA	NA	NA
Bc_ctg1_3941	B2	0	Bcer	NA	Bcer	FALSE	NA	NA	NA
Bc_ctg1_3943	B2	5.244980895	Bcer	P	Bcer	FALSE	NA	NA	NA
Bc_ctg1_3945	B2	252.9246343	Bcer	S	Bcer	FALSE	NA	NA	NA
Bc_ctg1_3946	B2	0	Bcer	S	Bcer	FALSE	NA	NA	NA
Bc_ctg1_3947	B2	39.51218941	Bcer	K	Bcer	FALSE	NA	NA	NA
Bc_ctg1_3948	B2	39.74529967	Bcer	S	Bcer	FALSE	Bc_Ga0417192_01_rn28	Other	NA
Bc_ctg1_3949	B2	195.6960649	Bcer	P	Bcer	FALSE	Bc_Ga0417192_01_rn28	Other	NA
Bc_ctg1_395	B2	2.797323144	Bcer	CH	Bcer	FALSE	NA	NA	NA
Bc_ctg1_3950	B2	7.809193777	Bcer	NA	Bcer	FALSE	Bc_Ga0417192_01_rn28	Other	NA
Bc_ctg1_3951	B2	8.042304039	Bcer	NA	Bcer	FALSE	Bc_Ga0417192_01_rn28	Other	NA
Bc_ctg1_3952	B2	40.56118559	Bcer	E	Bcer	FALSE	Bc_Ga0417192_01_rn28	Other	NA
Bc_ctg1_3953	B2	2.797323144	Bcer	S	Bcer	FALSE	Bc_Ga0417192_01_rn28	Other	NA
Bc_ctg1_3954	B2	237.8890224	Bcer	P	Bcer	FALSE	Bc_Ga0417192_01_rn28	Other	NA
Bc_ctg1_3955	B2	94.64276637	Bcer	L	Bcer	FALSE	Bc_Ga0417192_01_rn28	Other	NA
Bc_ctg1_3956	B2	0	Bcer	NA	Bcer	FALSE	Bc_Ga0417192_01_rn28	Other	NA
Bc_ctg1_3957	B2	1.282106441	Bcer	I	Bcer	FALSE	Bc_Ga0417192_01_rn28	Ancillary biosynthesis	SMCOG1088
Bc_ctg1_3958	B2	1.282106441	Bcer	M	Bcer	FALSE	Bc_Ga0417192_01_rn28	Ancillary biosynthesis	SMCOG1216
Bc_ctg1_3959	B2	0.815885917	Bcer	D	Bcer	FALSE	Bc_Ga0417192_01_rn28	Other	NA
Bc_ctg1_396	B2	0.233110262	Bcer	K	Bcer	FALSE	NA	NA	NA
Bc_ctg1_3960	B2	1.16555131	Bcer	M	Bcer	FALSE	Bc_Ga0417192_01_rn28	Core biosynthesis	SMCOG1146
Bc_ctg1_3961	B2	2.214547489	Bcer	S	Bcer	FALSE	Bc_Ga0417192_01_rn28	Core biosynthesis	None
Bc_ctg1_3962	B2	1.864882096	Bcer	M	Bcer	FALSE	Bc_Ga0417192_01_rn28	Core biosynthesis	None
Bc_ctg1_3963	B2	3.962874454	Bcer	M	Bcer	FALSE	Bc_Ga0417192_01_rn28	Core biosynthesis	None
Bc_ctg1_3964	B2	0	Bcer	M	Bcer	FALSE	Bc_Ga0417192_01_rn28	Core biosynthesis	SMCOG1045
Bc_ctg1_3965	B2	0.233110262	Bcer	M	Bcer	FALSE	Bc_Ga0417192_01_rn28	Other	NA
Bc_ctg1_3966	B2	5.128425764	Bcer	M	Bcer	FALSE	NA	NA	NA

Bc_ctg1_3967	B2	1.748326965	Bcer	K	Bcer	FALSE	NA	NA	NA
Bc_ctg1_3968	B2	20.04748253	Bcer	Q	Bcer	TRUE	NA	NA	NA
Bc_ctg1_3969	B2	6.527087336	Bcer	NA	Bcer	FALSE	NA	NA	NA
Bc_ctg1_397	B2	7.226418122	Bcer	C	Bcer	FALSE	NA	NA	NA
Bc_ctg1_3970	B2	18.29915557	Bcer	EG	Bcer	FALSE	NA	NA	NA
Bc_ctg1_3971	B2	4.778760371	Bcer	L	Bcer	FALSE	NA	NA	NA
Bc_ctg1_3972	B2	69.23374781	Bcer	G	Bcer	FALSE	NA	NA	NA
Bc_ctg1_3973	B2	2.214547489	Bcer	S	Bcer	FALSE	NA	NA	NA
Bc_ctg1_3974	B2	6.643642467	Bcer	L	Bcer	FALSE	NA	NA	NA
Bc_ctg1_3975	B2	1.282106441	Bcer	S	Bcer	FALSE	NA	NA	NA
Bc_ctg1_3976	B2	0.815885917	Bcer	S	Bcer	FALSE	NA	NA	NA
Bc_ctg1_3977	B2	12.00517849	Bcer	K	Bcer	FALSE	NA	NA	NA
Bc_ctg1_3978	B2	0.466220524	Bcer	S	Bcer	FALSE	NA	NA	NA
Bc_ctg1_3979	B2	15.61838755	Bcer	E	Bcer	FALSE	NA	NA	NA
Bc_ctg1_398	B2	127.0450928	Bcer	S	Bcer	FALSE	NA	NA	NA
Bc_ctg1_3980	B2	2.564212882	Bcer	NA	Bcer	FALSE	NA	NA	NA
Bc_ctg1_3981	B2	2.680768013	Bcer	I	Bcer	FALSE	NA	NA	NA
Bc_ctg1_3982	B2	0.233110262	Bcer	S	Bcer	FALSE	NA	NA	NA
Bc_ctg1_3983	B2	0.932441048	Bcer	S	Bcer	FALSE	NA	NA	NA
Bc_ctg1_3984	B2	51.86703329	Bcer	E	Bcer	FALSE	NA	NA	NA
Bc_ctg1_3985	B2	0	Bcer	S	Bcer	FALSE	NA	NA	NA
Bc_ctg1_3986	B2	41.26051637	Bcer	P	Bcer	FALSE	NA	NA	NA
Bc_ctg1_3987	B2	9.091300218	Bcer	KT	Bcer	FALSE	NA	NA	NA
Bc_ctg1_3988	B2	48.25382423	Bcer	V	Bcer	FALSE	NA	NA	NA
Bc_ctg1_3989	B2	0.116555131	Bcer	S	Bcer	FALSE	NA	NA	NA
Bc_ctg1_399	B2	8.042304039	Bcer	S	Bcer	FALSE	NA	NA	NA
Bc_ctg1_3990	B2	17.36671452	Bcer	S	Bcer	FALSE	NA	NA	NA
Bc_ctg1_3991	B2	0.932441048	Bcer	S	Bcer	FALSE	NA	NA	NA
Bc_ctg1_3992	B2	30.65399945	Bcer	C	Bcer	FALSE	NA	NA	NA
Bc_ctg1_3993	B2	5.711201419	Bcer	K	Bcer	FALSE	NA	NA	NA
Bc_ctg1_3994	B2	2.680768013	Bcer	NA	Bcer	FALSE	NA	NA	NA
Bc_ctg1_3995	B2	5.361536026	Bcer	S	Bcer	FALSE	NA	NA	NA
Bc_ctg1_3996	B2	1.981437227	Bcer	G	Bcer	FALSE	NA	NA	NA
Bc_ctg1_3997	B2	0.815885917	Bcer	K	Bcer	FALSE	NA	NA	NA
Bc_ctg1_3998	B2	0.582775655	Bcer	EGP	Bcer	FALSE	NA	NA	NA
Bc_ctg1_3999	B2	0.815885917	Bcer	E	Bcer	FALSE	NA	NA	NA
Bc_ctg1_4	B2	1.048996179	Bcer	F	Bcer	FALSE	NA	NA	NA
Bc_ctg1_40	B2	0	Bcer	L	Bcer	FALSE	NA	NA	NA
Bc_ctg1_400	B2	8.275414301	Bcer	NA	Bcer	FALSE	NA	NA	NA
Bc_ctg1_4001	B2	0.349665393	Bcer	NA	Bcer	FALSE	NA	NA	NA
Bc_ctg1_4002	B2	4.195984716	Bcer	S	Bcer	FALSE	NA	NA	NA
Bc_ctg1_4003	B2	49.30282041	Bcer	S	Bcer	FALSE	NA	NA	NA
Bc_ctg1_4004	B2	56.99545906	Bcer	N	Bcer	FALSE	NA	NA	NA
Bc_ctg1_4005	B2	82.75414301	Bcer	N	Bcer	FALSE	NA	NA	NA
Bc_ctg1_4006	B2	77.85882751	Bcer	T	Bcer	FALSE	NA	NA	NA
Bc_ctg1_4007	B2	753.8785873	Bcer	NT	Bcer	FALSE	NA	NA	NA
Bc_ctg1_4008	B2	189.0524225	Bcer	N	Bcer	FALSE	NA	NA	NA
Bc_ctg1_4009	B2	57.57823471	Bcer	NA	Bcer	FALSE	NA	NA	NA
Bc_ctg1_401	B2	6.293977074	Bcer	K	Bcer	FALSE	NA	NA	NA
Bc_ctg1_4010	B2	116.2054656	Bcer	NA	Bcer	FALSE	NA	NA	NA
Bc_ctg1_4011	B2	44.7571703	Bcer	NT	Bcer	FALSE	NA	NA	NA
Bc_ctg1_4012	B2	32.63543668	Bcer	NA	Bcer	FALSE	NA	NA	NA
Bc_ctg1_4013	B2	50.81803711	Bcer	S	Bcer	FALSE	NA	NA	NA
Bc_ctg1_4014	B2	45.80616648	Bcer	NA	Bcer	FALSE	NA	NA	NA
Bc_ctg1_4015	B2	429.9718782	Bcer	N	Bcer	FALSE	NA	NA	NA
Bc_ctg1_4016	B2	271.5734552	Bcer	N	Bcer	FALSE	NA	NA	NA
Bc_ctg1_4017	B2	549.2077773	Bcer	N	Bcer	FALSE	NA	NA	NA
Bc_ctg1_4018	B2	192.7821867	Bcer	N	Bcer	FALSE	NA	NA	NA
Bc_ctg1_4019	B2	133.2225147	Bcer	NA	Bcer	FALSE	NA	NA	NA
Bc_ctg1_402	B2	0.582775655	Bcer	S	Bcer	FALSE	NA	NA	NA
Bc_ctg1_4020	B2	72.03107096	Bcer	N	Bcer	FALSE	NA	NA	NA
Bc_ctg1_4021	B2	76.80983133	Bcer	N	Bcer	FALSE	NA	NA	NA

Bc_ctg1_4022	B2	34.26720851	Bcer	N	Bcer	FALSE	NA	NA	NA
Bc_ctg1_4023	B2	360.2719099	Bcer	N	Bcer	FALSE	NA	NA	NA
Bc_ctg1_4024	B2	299.5466867	Bcer	N	Bcer	FALSE	NA	NA	NA
Bc_ctg1_4025	B2	65.0377631	Bcer	N	Bcer	FALSE	NA	NA	NA
Bc_ctg1_4026	B2	211.780673	Bcer	NU	Bcer	FALSE	NA	NA	NA
Bc_ctg1_4027	B2	114.9233592	Bcer	NA	Bcer	FALSE	NA	NA	NA
Bc_ctg1_4028	B2	255.372292	Bcer	N	Bcer	FALSE	NA	NA	NA
Bc_ctg1_4029	B2	204.67081	Bcer	N	Bcer	FALSE	NA	NA	NA
Bc_ctg1_403	B2	1.631771834	Bcer	S	Bcer	FALSE	NA	NA	NA
Bc_ctg1_4030	B2	539.0674809	Bcer	N	Bcer	FALSE	NA	NA	NA
Bc_ctg1_4031	B2	113.5246976	Bcer	S	Bcer	FALSE	NA	NA	NA
Bc_ctg1_4032	B2	261.549714	Bcer	NA	Bcer	FALSE	NA	NA	NA
Bc_ctg1_4033	B2	658.5364901	Bcer	T	Bcer	FALSE	NA	NA	NA
Bc_ctg1_4034	B2	44.52406004	Bcer	K	Bcer	FALSE	NA	NA	NA
Bc_ctg1_4035	B2	59.32656168	Bcer	N	Bcer	FALSE	NA	NA	NA
Bc_ctg1_4036	B2	16536.49232	Bcer	N	Bcer	FALSE	NA	NA	NA
Bc_ctg1_4037	B2	82.17136735	Bcer	M	Bcer	FALSE	NA	NA	NA
Bc_ctg1_4038	B2	3.030433406	Bcer	N	Bcer	FALSE	NA	NA	NA
Bc_ctg1_4039	B2	129.2596403	Bcer	N	Bcer	FALSE	NA	NA	NA
Bc_ctg1_404	B2	4.079429585	Bcer	C	Bcer	FALSE	NA	NA	NA
Bc_ctg1_4040	B2	60.60866812	Bcer	N	Bcer	FALSE	NA	NA	NA
Bc_ctg1_4041	B2	23.89380185	Bcer	N	Bcer	FALSE	NA	NA	NA
Bc_ctg1_4042	B2	77.85882751	Bcer	N	Bcer	FALSE	NA	NA	NA
Bc_ctg1_4043	B2	12.58795415	Bcer	N	Bcer	FALSE	NA	NA	NA
Bc_ctg1_4044	B2	74.59528384	Bcer	N	Bcer	FALSE	NA	NA	NA
Bc_ctg1_4045	B2	135.4370622	Bcer	N	Bcer	FALSE	NA	NA	NA
Bc_ctg1_4046	B2	244.4161097	Bcer	N	Bcer	FALSE	NA	NA	NA
Bc_ctg1_4047	B2	290.4553864	Bcer	N	Bcer	FALSE	NA	NA	NA
Bc_ctg1_4048	B2	211.8972282	Bcer	N	Bcer	FALSE	NA	NA	NA
Bc_ctg1_4049	B2	40.79429585	Bcer	C	Bcer	FALSE	NA	NA	NA
Bc_ctg1_405	B2	5.711201419	Bcer	S	Bcer	FALSE	NA	NA	NA
Bc_ctg1_4050	B2	4.429094978	Bcer	S	Bcer	FALSE	NA	NA	NA
Bc_ctg1_4051	B2	14.10317085	Bcer	K	Bcer	FALSE	NA	NA	NA
Bc_ctg1_4052	B2	14.56939137	Bcer	E	Bcer	FALSE	NA	NA	NA
Bc_ctg1_4053	B2	4.195984716	Bcer	S	Bcer	FALSE	NA	NA	NA
Bc_ctg1_4054	B2	0.466220524	Bcer	NA	Bcer	FALSE	NA	NA	NA
Bc_ctg1_4055	B2	1.398661572	Bcer	NA	Bcer	FALSE	NA	NA	NA
Bc_ctg1_4056	B2	1.864882096	Bcer	Q	Bcer	TRUE	NA	NA	NA
Bc_ctg1_4057	B2	0.349665393	Bcer	S	Bcer	FALSE	NA	NA	NA
Bc_ctg1_4058	B2	0.349665393	Bcer	S	Bcer	FALSE	NA	NA	NA
Bc_ctg1_4059	B2	18.99848635	Bcer	K	Bcer	FALSE	NA	NA	NA
Bc_ctg1_406	B2	18.88193122	Bcer	J	Bcer	FALSE	NA	NA	NA
Bc_ctg1_4060	B2	1.048996179	Bcer	EGP	Bcer	FALSE	NA	NA	NA
Bc_ctg1_4061	B2	0.233110262	Bcer	S	Bcer	FALSE	NA	NA	NA
Bc_ctg1_4062	B2	0.932441048	Bcer	K	Bcer	FALSE	NA	NA	NA
Bc_ctg1_4063	B2	0	Bcer	P	Bcer	FALSE	NA	NA	NA
Bc_ctg1_4064	B2	0.349665393	Bcer	P	Bcer	FALSE	NA	NA	NA
Bc_ctg1_4065	B2	1.515216703	Bcer	P	Bcer	FALSE	NA	NA	NA
Bc_ctg1_4066	B2	1.16555131	Bcer	J	Bcer	FALSE	NA	NA	NA
Bc_ctg1_4067	B2	0.116555131	Bcer	NA	Bcer	FALSE	NA	NA	NA
Bc_ctg1_4068	B2	229.9632735	Bcer	V	Bcer	FALSE	NA	NA	NA
Bc_ctg1_4069	B2	41.02740611	Bcer	S	Bcer	FALSE	NA	NA	NA
Bc_ctg1_407	B2	0.116555131	Bcer	NA	Bcer	FALSE	NA	NA	NA
Bc_ctg1_4070	B2	13.40384006	Bcer	NA	Bcer	FALSE	NA	NA	NA
Bc_ctg1_4071	B2	1.631771834	Bcer	V	Bcer	FALSE	NA	NA	NA
Bc_ctg1_4072	B2	0.466220524	Bcer	G	Bcer	FALSE	NA	NA	NA
Bc_ctg1_4073	B2	1135.130421	Bcer	NA	Bcer	FALSE	NA	NA	NA
Bc_ctg1_4074	B2	1.864882096	Bcer	S	Bcer	FALSE	NA	NA	NA
Bc_ctg1_4075	B2	0.815885917	Bcer	NA	Bcer	FALSE	NA	NA	NA
Bc_ctg1_4076	B2	0.582775655	Bcer	K	Bcer	FALSE	NA	NA	NA
Bc_ctg1_4077	B2	12.58795415	Bcer	T	Bcer	FALSE	NA	NA	NA
Bc_ctg1_4078	B2	26.22490447	Bcer	E	Bcer	FALSE	NA	NA	NA

Bc_ctg1_4079	B2	0.582775655	Bcer	K	Bcer	FALSE	NA	NA	NA
Bc_ctg1_408	B2	0.233110262	Bcer	I	Bcer	FALSE	NA	NA	NA
Bc_ctg1_4080	B2	0.116555131	Bcer	S	Bcer	FALSE	NA	NA	NA
Bc_ctg1_4081	B2	0.582775655	Bcer	S	Bcer	FALSE	NA	NA	NA
Bc_ctg1_4082	B2	46.73860753	Bcer	P	Bcer	FALSE	NA	NA	NA
Bc_ctg1_4083	B2	2.214547489	Bcer	S	Bcer	FALSE	NA	NA	NA
Bc_ctg1_4084	B2	2.797323144	Bcer	K	Bcer	FALSE	NA	NA	NA
Bc_ctg1_4085	B2	3.729764192	Bcer	EG	Bcer	FALSE	NA	NA	NA
Bc_ctg1_4086	B2	0.116555131	Bcer	NA	Bcer	FALSE	NA	NA	NA
Bc_ctg1_4087	B2	0.582775655	Bcer	S	Bcer	FALSE	NA	NA	NA
Bc_ctg1_4088	B2	0.233110262	Bcer	S	Bcer	FALSE	NA	NA	NA
Bc_ctg1_4089	B2	0.233110262	Bcer	S	Bcer	FALSE	NA	NA	NA
Bc_ctg1_409	B2	1.048996179	Bcer	NA	Bcer	FALSE	NA	NA	NA
Bc_ctg1_4090	B2	0.349665393	Bcer	M	Bcer	FALSE	NA	NA	NA
Bc_ctg1_4091	B2	71.79796069	Bcer	C	Bcer	FALSE	NA	NA	NA
Bc_ctg1_4092	B2	2.680768013	Bcer	K	Bcer	FALSE	NA	NA	NA
Bc_ctg1_4093	B2	0.815885917	Bcer	IQ	Bcer	TRUE	NA	NA	NA
Bc_ctg1_4094	B2	437.4314066	Bcer	C	Bcer	FALSE	NA	NA	NA
Bc_ctg1_4095	B2	16.78393886	Bcer	S	Bcer	FALSE	NA	NA	NA
Bc_ctg1_4096	B2	16.5508286	Bcer	K	Bcer	FALSE	NA	NA	NA
Bc_ctg1_4097	B2	27.62356605	Bcer	V	Bcer	FALSE	NA	NA	NA
Bc_ctg1_4098	B2	9.32441048	Bcer	E	Bcer	FALSE	NA	NA	NA
Bc_ctg1_4099	B2	0.932441048	Bcer	NA	Bcer	FALSE	NA	NA	NA
Bc_ctg1_41	B2	0.116555131	Bcer	L	Bcer	FALSE	NA	NA	NA
Bc_ctg1_410	B2	7.692638646	Bcer	P	Bcer	FALSE	NA	NA	NA
Bc_ctg1_4100	B2	13.28728493	Bcer	K	Bcer	FALSE	NA	NA	NA
Bc_ctg1_4101	B2	11.07273744	Bcer	O	Bcer	FALSE	NA	NA	NA
Bc_ctg1_4102	B2	0.349665393	Bcer	O	Bcer	FALSE	NA	NA	NA
Bc_ctg1_4103	B2	1.515216703	Bcer	CO	Bcer	FALSE	NA	NA	NA
Bc_ctg1_4104	B2	3.030433406	Bcer	M	Bcer	FALSE	NA	NA	NA
Bc_ctg1_4105	B2	0	Bcer	NA	Bcer	FALSE	NA	NA	NA
Bc_ctg1_4106	B2	32.86854694	Bcer	K	Bcer	FALSE	NA	NA	NA
Bc_ctg1_4107	B2	12.58795415	Bcer	K	Bcer	FALSE	NA	NA	NA
Bc_ctg1_4108	B2	12.47139902	Bcer	E	Bcer	FALSE	NA	NA	NA
Bc_ctg1_4109	B2	48.95315502	Bcer	S	Bcer	FALSE	NA	NA	NA
Bc_ctg1_411	B2	3.263543668	Bcer	S	Bcer	FALSE	NA	NA	NA
Bc_ctg1_4110	B2	11.30584771	Bcer	NA	Bcer	FALSE	NA	NA	NA
Bc_ctg1_4111	B2	5.711201419	Bcer	NA	Bcer	FALSE	NA	NA	NA
Bc_ctg1_4112	B2	1.748326965	Bcer	NA	Bcer	FALSE	NA	NA	NA
Bc_ctg1_4113	B2	1.398661572	Bcer	K	Bcer	FALSE	NA	NA	NA
Bc_ctg1_4114	B2	1.864882096	Bcer	K	Bcer	FALSE	NA	NA	NA
Bc_ctg1_4115	B2	5.478091157	Bcer	T	Bcer	FALSE	NA	NA	NA
Bc_ctg1_4116	B2	53.49880513	Bcer	V	Bcer	FALSE	NA	NA	NA
Bc_ctg1_4117	B2	55.13057696	Bcer	V	Bcer	FALSE	NA	NA	NA
Bc_ctg1_4118	B2	98.37253056	Bcer	V	Bcer	FALSE	NA	NA	NA
Bc_ctg1_4119	B2	0	Bcer	S	Bcer	FALSE	NA	NA	NA
Bc_ctg1_412	B2	0.582775655	Bcer	NA	Bcer	FALSE	NA	NA	NA
Bc_ctg1_4120	B2	1.864882096	Bcer	I	Bcer	FALSE	NA	NA	NA
Bc_ctg1_4121	B2	25.40901856	Bcer	F	Bcer	FALSE	NA	NA	NA
Bc_ctg1_4122	B2	224.252072	Bcer	C	Bcer	FALSE	NA	NA	NA
Bc_ctg1_4123	B2	312.4843062	Bcer	E	Bcer	FALSE	NA	NA	NA
Bc_ctg1_4124	B2	382.5339399	Bcer	C	Bcer	FALSE	NA	NA	NA
Bc_ctg1_4125	B2	18.99848635	Bcer	T	Bcer	FALSE	NA	NA	NA
Bc_ctg1_4126	B2	14.80250164	Bcer	T	Bcer	FALSE	NA	NA	NA
Bc_ctg1_4127	B2	79.37404421	Bcer	L	Bcer	FALSE	NA	NA	NA
Bc_ctg1_4128	B2	0	Bcer	NA	Bcer	FALSE	NA	NA	NA
Bc_ctg1_413	B2	1.048996179	Bcer	U	Bcer	FALSE	NA	NA	NA
Bc_ctg1_4130	B2	74.36217358	Bcer	F	Bcer	FALSE	NA	NA	NA
Bc_ctg1_4131	B2	0.815885917	Bcer	K	Bcer	FALSE	NA	NA	NA
Bc_ctg1_4132	B2	8.858189956	Bcer	E	Bcer	FALSE	NA	NA	NA
Bc_ctg1_4133	B2	14.68594651	Bcer	E	Bcer	FALSE	NA	NA	NA
Bc_ctg1_4134	B2	7.692638646	Bcer	S	Bcer	FALSE	NA	NA	NA

Bc_ctg1_4135	B2	3.729764192	Bcer	S	Bcer	FALSE	Bc_Ga0417192_01_rn29	Other	NA
Bc_ctg1_4136	B2	14.45283624	Bcer	S	Bcer	FALSE	Bc_Ga0417192_01_rn29	Other	NA
Bc_ctg1_4137	B2	0.466220524	Bcer	NA	Bcer	FALSE	Bc_Ga0417192_01_rn29	Other	NA
Bc_ctg1_4138	B2	37.06453166	Bcer	S	Bcer	FALSE	Bc_Ga0417192_01_rn29	Other	NA
Bc_ctg1_4139	B2	10.83962718	Bcer	K	Bcer	FALSE	Bc_Ga0417192_01_rn29	Regulation	SMCOG1278
Bc_ctg1_414	B2	16.78393886	Bcer	S	Bcer	FALSE	NA	NA	NA
Bc_ctg1_4140	B2	103.5009563	Bcer	EGP	Bcer	FALSE	Bc_Ga0417192_01_rn29	Transport	SMCOG1005
Bc_ctg1_4141	B2	7.925748908	Bcer	S	Bcer	FALSE	Bc_Ga0417192_01_rn29	Other	NA
Bc_ctg1_4142	B2	11.07273744	Bcer	EGP	Bcer	FALSE	Bc_Ga0417192_01_rn29	Transport	SMCOG1202
Bc_ctg1_4143	B2	5.011870633	Bcer	I	Bcer	FALSE	Bc_Ga0417192_01_rn29	Core biosynthesis	SMCOG1084
Bc_ctg1_4144	B2	75.17805949	Bcer	S	Bcer	FALSE	Bc_Ga0417192_01_rn29	Other	NA
Bc_ctg1_4145	B2	15.85149782	Bcer	P	Bcer	FALSE	Bc_Ga0417192_01_rn29	Transport	SMCOG1106
Bc_ctg1_4146	B2	63.05632587	Bcer	E	Bcer	FALSE	Bc_Ga0417192_01_rn29	Ancillary biosynthesis	SMCOG1081
Bc_ctg1_4147	B2	8.275414301	Bcer	S	Bcer	FALSE	Bc_Ga0417192_01_rn29	Other	NA
Bc_ctg1_4148	B2	18.4157107	Bcer	S	Bcer	FALSE	Bc_Ga0417192_01_rn29	Other	NA
Bc_ctg1_4149	B2	15.50183242	Bcer	NA	Bcer	FALSE	Bc_Ga0417192_01_rn29	Other	NA
Bc_ctg1_415	B2	19.58126201	Bcer	S	Bcer	FALSE	NA	NA	NA
Bc_ctg1_4150	B2	6.99330786	Bcer	S	Bcer	FALSE	Bc_Ga0417192_01_rn29	Other	NA
Bc_ctg1_4151	B2	4.66220524	Bcer	G	Bcer	FALSE	Bc_Ga0417192_01_rn29	Ancillary biosynthesis	SMCOG1235
Bc_ctg1_4152	B2	2.564212882	Bcer	S	Bcer	FALSE	Bc_Ga0417192_01_rn29	Other	NA
Bc_ctg1_4153	B2	1.748326965	Bcer	S	Bcer	FALSE	NA	NA	NA
Bc_ctg1_4154	B2	36.36520087	Bcer	S	Bcer	FALSE	NA	NA	NA
Bc_ctg1_4155	B2	1.048996179	Bcer	DZ	Bcer	FALSE	NA	NA	NA
Bc_ctg1_4156	B2	0	Bcer	NA	Bcer	FALSE	NA	NA	NA
Bc_ctg1_4158	B2	32.63543668	Bcer	O	Bcer	FALSE	NA	NA	NA
Bc_ctg1_4159	B2	0.233110262	Bcer	IQ	Bcer	TRUE	NA	NA	NA
Bc_ctg1_416	B2	1.631771834	Bcer	P	Bcer	FALSE	Bc_Ga0417192_01_rn05	Other	NA
Bc_ctg1_4160	B2	0.116555131	Bcer	E	Bcer	FALSE	NA	NA	NA
Bc_ctg1_4161	B2	1.398661572	Bcer	E	Bcer	FALSE	NA	NA	NA
Bc_ctg1_4162	B2	0.116555131	Bcer	E	Bcer	FALSE	NA	NA	NA
Bc_ctg1_4163	B2	0.116555131	Bcer	EH	Bcer	FALSE	NA	NA	NA
Bc_ctg1_4164	B2	4.545650109	Bcer	E	Bcer	FALSE	NA	NA	NA
Bc_ctg1_4165	B2	9.440965611	Bcer	E	Bcer	FALSE	NA	NA	NA
Bc_ctg1_4166	B2	0	Bcer	NA	Bcer	FALSE	NA	NA	NA
Bc_ctg1_4167	B2	1.864882096	Bcer	M	Bcer	FALSE	NA	NA	NA
Bc_ctg1_4168	B2	0.116555131	Bcer	S	Bcer	FALSE	NA	NA	NA
Bc_ctg1_4169	B2	0.116555131	Bcer	K	Bcer	FALSE	NA	NA	NA
Bc_ctg1_417	B2	0.466220524	Bcer	E	Bcer	FALSE	Bc_Ga0417192_01_rn05	Other	NA
Bc_ctg1_4170	B2	16.08460808	Bcer	EGP	Bcer	FALSE	NA	NA	NA
Bc_ctg1_4171	B2	9.32441048	Bcer	S	Bcer	FALSE	NA	NA	NA
Bc_ctg1_4172	B2	39.97840993	Bcer	I	Bcer	FALSE	NA	NA	NA
Bc_ctg1_4173	B2	26.45801474	Bcer	K	Bcer	FALSE	NA	NA	NA
Bc_ctg1_4174	B2	294.7679263	Bcer	C	Bcer	FALSE	NA	NA	NA
Bc_ctg1_4175	B2	1.864882096	Bcer	K	Bcer	FALSE	NA	NA	NA
Bc_ctg1_4176	B2	0	Bcer	S	Bcer	FALSE	NA	NA	NA
Bc_ctg1_4177	B2	104.6665076	Bcer	F	Bcer	FALSE	NA	NA	NA
Bc_ctg1_4178	B2	2.447657751	Bcer	EGP	Bcer	FALSE	NA	NA	NA
Bc_ctg1_4179	B2	4.895315502	Bcer	K	Bcer	FALSE	NA	NA	NA
Bc_ctg1_418	B2	3.380098799	Bcer	S	Bcer	FALSE	Bc_Ga0417192_01_rn05	Other	NA
Bc_ctg1_4180	B2	48.1372691	Bcer	E	Bcer	FALSE	NA	NA	NA
Bc_ctg1_4181	B2	4.312539847	Bcer	J	Bcer	FALSE	NA	NA	NA
Bc_ctg1_4182	B2	1.748326965	Bcer	T	Bcer	FALSE	NA	NA	NA
Bc_ctg1_4183	B2	2.680768013	Bcer	T	Bcer	FALSE	NA	NA	NA
Bc_ctg1_4184	B2	2.33110262	Bcer	M	Bcer	FALSE	NA	NA	NA
Bc_ctg1_4185	B2	240.3366801	Bcer	P	Bcer	FALSE	NA	NA	NA
Bc_ctg1_4186	B2	56.06301801	Bcer	S	Bcer	FALSE	NA	NA	NA
Bc_ctg1_4187	B2	0	Bcer	P	Bcer	FALSE	NA	NA	NA
Bc_ctg1_4188	B2	128.094089	Bcer	H	Bcer	FALSE	NA	NA	NA
Bc_ctg1_419	B2	0.582775655	Bcer	S	Bcer	FALSE	Bc_Ga0417192_01_rn05	Other	NA
Bc_ctg1_4190	B2	102.5685153	Bcer	E	Bcer	FALSE	NA	NA	NA
Bc_ctg1_4191	B2	2056.032511	Bcer	O	Bcer	FALSE	NA	NA	NA
Bc_ctg1_4192	B2	2670.511161	Bcer	E	Bcer	FALSE	NA	NA	NA

Bc_ctg1_4193	B2	388.0120311	Bcer	E	Bcer	FALSE	NA	NA	NA
Bc_ctg1_4194	B2	11.77206823	Bcer	O	Bcer	FALSE	NA	NA	NA
Bc_ctg1_4195	B2	8.741634825	Bcer	E	Bcer	FALSE	NA	NA	NA
Bc_ctg1_4196	B2	13.75350546	Bcer	S	Bcer	FALSE	NA	NA	NA
Bc_ctg1_4197	B2	7.576083515	Bcer	K	Bcer	FALSE	NA	NA	NA
Bc_ctg1_4198	B2	12.93761954	Bcer	K	Bcer	FALSE	NA	NA	NA
Bc_ctg1_4199	B2	0	Bcer	S	Bcer	FALSE	NA	NA	NA
Bc_ctg1_42	B2	117.0213515	Bcer	S	Bcer	FALSE	NA	NA	NA
Bc_ctg1_420	B2	0	Bcer	JM	Bcer	FALSE	Bc_Ga0417192_01_rn05	Ancillary biosynthesis	SMCOG1064
Bc_ctg1_4200	B2	0	Bcer	S	Bcer	FALSE	NA	NA	NA
Bc_ctg1_4201	B2	10.25685153	Bcer	K	Bcer	FALSE	NA	NA	NA
Bc_ctg1_4202	B2	28.90567249	Bcer	K	Bcer	FALSE	NA	NA	NA
Bc_ctg1_4203	B2	77.39260698	Bcer	F	Bcer	FALSE	NA	NA	NA
Bc_ctg1_4204	B2	38.11352784	Bcer	F	Bcer	FALSE	NA	NA	NA
Bc_ctg1_4205	B2	53.14913973	Bcer	F	Bcer	FALSE	NA	NA	NA
Bc_ctg1_4206	B2	10.95618231	Bcer	F	Bcer	FALSE	NA	NA	NA
Bc_ctg1_4207	B2	3.49665393	Bcer	O	Bcer	FALSE	NA	NA	NA
Bc_ctg1_4208	B2	7.809193777	Bcer	S	Bcer	FALSE	NA	NA	NA
Bc_ctg1_4209	B2	6.99330786	Bcer	NA	Bcer	FALSE	NA	NA	NA
Bc_ctg1_421	B2	0	Bcer	M	Bcer	FALSE	Bc_Ga0417192_01_rn05	Other	NA
Bc_ctg1_4210	B2	80.42304039	Bcer	O	Bcer	FALSE	NA	NA	NA
Bc_ctg1_4211	B2	114.1074732	Bcer	S	Bcer	FALSE	NA	NA	NA
Bc_ctg1_4212	B2	83.33691866	Bcer	E	Bcer	FALSE	NA	NA	NA
Bc_ctg1_4213	B2	150.2395639	Bcer	Q	Bcer	TRUE	NA	NA	NA
Bc_ctg1_4214	B2	0	Bcer	O	Bcer	FALSE	NA	NA	NA
Bc_ctg1_4215	B2	12.35484389	Bcer	MT	Bcer	FALSE	NA	NA	NA
Bc_ctg1_4216	B2	0	Bcer	NA	Bcer	FALSE	NA	NA	NA
Bc_ctg1_4217	B2	49.30282041	Bcer	L	Bcer	FALSE	NA	NA	NA
Bc_ctg1_4218	B2	4.778760371	Bcer	S	Bcer	FALSE	NA	NA	NA
Bc_ctg1_4219	B2	29.95466867	Bcer	P	Bcer	FALSE	NA	NA	NA
Bc_ctg1_422	B2	0	Bcer	GM	Bcer	FALSE	Bc_Ga0417192_01_rn05	Core biosynthesis	SMCOG1010
Bc_ctg1_4220	B2	5.594646288	Bcer	I	Bcer	FALSE	NA	NA	NA
Bc_ctg1_4221	B2	293.4858199	Bcer	E	Bcer	FALSE	NA	NA	NA
Bc_ctg1_4222	B2	6.760197598	Bcer	E	Bcer	FALSE	NA	NA	NA
Bc_ctg1_4223	B2	6.060866812	Bcer	P	Bcer	FALSE	NA	NA	NA
Bc_ctg1_4224	B2	2.33110262	Bcer	S	Bcer	FALSE	NA	NA	NA
Bc_ctg1_4225	B2	9.440965611	Bcer	EG	Bcer	FALSE	NA	NA	NA
Bc_ctg1_4226	B2	3.49665393	Bcer	K	Bcer	FALSE	NA	NA	NA
Bc_ctg1_4227	B2	1.16555131	Bcer	K	Bcer	FALSE	NA	NA	NA
Bc_ctg1_4228	B2	1.515216703	Bcer	Q	Bcer	TRUE	NA	NA	NA
Bc_ctg1_4229	B2	1.981437227	Bcer	S	Bcer	FALSE	NA	NA	NA
Bc_ctg1_423	B2	1.048996179	Bcer	GM	Bcer	FALSE	Bc_Ga0417192_01_rn05	Core biosynthesis	SMCOG1010
Bc_ctg1_4231	B2	0.815885917	Bcer	J	Bcer	FALSE	NA	NA	NA
Bc_ctg1_4232	B2	7.342973253	Bcer	S	Bcer	FALSE	NA	NA	NA
Bc_ctg1_4233	B2	36.59831113	Bcer	C	Bcer	FALSE	NA	NA	NA
Bc_ctg1_4234	B2	0.116555131	Bcer	E	Bcer	FALSE	NA	NA	NA
Bc_ctg1_4235	B2	11.07273744	Bcer	S	Bcer	FALSE	NA	NA	NA
Bc_ctg1_4236	B2	3.49665393	Bcer	E	Bcer	FALSE	NA	NA	NA
Bc_ctg1_4237	B2	0.699330786	Bcer	E	Bcer	FALSE	NA	NA	NA
Bc_ctg1_4238	B2	0.233110262	Bcer	E	Bcer	FALSE	NA	NA	NA
Bc_ctg1_4239	B2	1.16555131	Bcer	E	Bcer	FALSE	NA	NA	NA
Bc_ctg1_424	B2	3.49665393	Bcer	K	Bcer	FALSE	Bc_Ga0417192_01_rn05	Other	NA
Bc_ctg1_4240	B2	3.729764192	Bcer	E	Bcer	FALSE	NA	NA	NA
Bc_ctg1_4241	B2	6.527087336	Bcer	K	Bcer	FALSE	NA	NA	NA
Bc_ctg1_4242	B2	0.582775655	Bcer	S	Bcer	FALSE	NA	NA	NA
Bc_ctg1_4243	B2	0.466220524	Bcer	S	Bcer	FALSE	Bc_Ga0417192_01_rn30	Other	NA
Bc_ctg1_4244	B2	0.116555131	Bcer	S	Bcer	FALSE	Bc_Ga0417192_01_rn30	Other	NA
Bc_ctg1_4245	B2	54.54780131	Bcer	K	Bcer	FALSE	Bc_Ga0417192_01_rn30	Regulation	SMCOG1135
Bc_ctg1_4246	B2	50.23526146	Bcer	C	Bcer	FALSE	Bc_Ga0417192_01_rn30	Other	SMCOG1251
Bc_ctg1_4247	B2	19.46470688	Bcer	C	Bcer	FALSE	Bc_Ga0417192_01_rn30	Other	NA
Bc_ctg1_4248	B2	18.18260044	Bcer	C	Bcer	FALSE	Bc_Ga0417192_01_rn30	Other	NA
Bc_ctg1_4249	B2	20.51370306	Bcer	V	Bcer	FALSE	Bc_Ga0417192_01_rn30	Transport	SMCOG1288

Bc_ctg1_425	B2	0	Bcer	NA	Bcer	FALSE	Bc_Ga0417192_01_rn05	Other	NA
Bc_ctg1_4250	B2	24.36002238	Bcer	V	Bcer	FALSE	Bc_Ga0417192_01_rn30	Transport	SMCOG1288
Bc_ctg1_4251	B2	0.466220524	Bcer	E	Bcer	FALSE	Bc_Ga0417192_01_rn30	Other	NA
Bc_ctg1_4252	B2	4.66220524	Bcer	MQ	Bcer	TRUE	Bc_Ga0417192_01_rn30	Core biosynthesis	None
Bc_ctg1_4253	B2	3.263543668	Bcer	E	Bcer	FALSE	Bc_Ga0417192_01_rn30	Core biosynthesis	SMCOG1056
Bc_ctg1_4254	B2	1.748326965	Bcer	S	Bcer	FALSE	Bc_Ga0417192_01_rn30	Ancillary biosynthesis	SMCOG1156
Bc_ctg1_4256	B2	3.49665393	Bcer	NA	Bcer	FALSE	Bc_Ga0417192_01_rn30	Other	NA
Bc_ctg1_4257	B2	30.30433406	Bcer	P	Bcer	FALSE	Bc_Ga0417192_01_rn30	Other	NA
Bc_ctg1_4258	B2	38.92941375	Bcer	EG	Bcer	FALSE	Bc_Ga0417192_01_rn30	Other	NA
Bc_ctg1_4259	B2	44.87372543	Bcer	V	Bcer	FALSE	Bc_Ga0417192_01_rn30	Other	NA
Bc_ctg1_426	B2	58.2775655	Bcer	NT	Bcer	FALSE	Bc_Ga0417192_01_rn05	Other	SMCOG1061
Bc_ctg1_4260	B2	40.79429585	Bcer	M	Bcer	FALSE	Bc_Ga0417192_01_rn30	Other	NA
Bc_ctg1_4261	B2	498.8559607	Bcer	S	Bcer	FALSE	Bc_Ga0417192_01_rn30	Ancillary biosynthesis	None
Bc_ctg1_4262	B2	1.631771834	Bcer	NA	Bcer	FALSE	Bc_Ga0417192_01_rn30	Other	NA
Bc_ctg1_4263	B2	2625.520881	Bcer	M	Bcer	FALSE	Bc_Ga0417192_01_rn30	Other	NA
Bc_ctg1_4264	B2	19.9309274	Bcer	S	Bcer	FALSE	Bc_Ga0417192_01_rn30	Ancillary biosynthesis	SMCOG1036
Bc_ctg1_4265	B2	22.37858515	Bcer	V	Bcer	FALSE	NA	NA	NA
Bc_ctg1_4266	B2	12.47139902	Bcer	T	Bcer	FALSE	NA	NA	NA
Bc_ctg1_4267	B2	10.60651692	Bcer	T	Bcer	FALSE	NA	NA	NA
Bc_ctg1_4268	B2	0.582775655	Bcer	NA	Bcer	FALSE	NA	NA	NA
Bc_ctg1_4269	B2	108.6293821	Bcer	S	Bcer	FALSE	NA	NA	NA
Bc_ctg1_427	B2	1.282106441	Bcer	NA	Bcer	FALSE	Bc_Ga0417192_01_rn05	Other	NA
Bc_ctg1_4270	B2	30.88710971	Bcer	S	Bcer	FALSE	NA	NA	NA
Bc_ctg1_4271	B2	67.13575545	Bcer	G	Bcer	FALSE	NA	NA	NA
Bc_ctg1_4272	B2	0.116555131	Bcer	G	Bcer	FALSE	NA	NA	NA
Bc_ctg1_4273	B2	4.895315502	Bcer	S	Bcer	FALSE	NA	NA	NA
Bc_ctg1_4274	B2	37.06453166	Bcer	I	Bcer	FALSE	NA	NA	NA
Bc_ctg1_4275	B2	4.778760371	Bcer	S	Bcer	FALSE	NA	NA	NA
Bc_ctg1_4276	B2	85.31835589	Bcer	E	Bcer	FALSE	NA	NA	NA
Bc_ctg1_4277	B2	102.335405	Bcer	E	Bcer	FALSE	NA	NA	NA
Bc_ctg1_4278	B2	122.4994427	Bcer	E	Bcer	FALSE	NA	NA	NA
Bc_ctg1_4279	B2	0.932441048	Bcer	S	Bcer	FALSE	NA	NA	NA
Bc_ctg1_428	B2	0.932441048	Bcer	NA	Bcer	FALSE	Bc_Ga0417192_01_rn05	Other	NA
Bc_ctg1_4280	B2	4.545650109	Bcer	S	Bcer	FALSE	NA	NA	NA
Bc_ctg1_4281	B2	112.126036	Bcer	K	Bcer	FALSE	NA	NA	NA
Bc_ctg1_4282	B2	10.02374127	Bcer	S	Bcer	FALSE	NA	NA	NA
Bc_ctg1_4283	B2	6.177421943	Bcer	T	Bcer	FALSE	Bc_Ga0417192_01_rn31	Other	NA
Bc_ctg1_4284	B2	12.93761954	Bcer	T	Bcer	FALSE	Bc_Ga0417192_01_rn31	Regulation	SMCOG1003
Bc_ctg1_4285	B2	209.5661255	Bcer	G	Bcer	FALSE	Bc_Ga0417192_01_rn31	Ancillary biosynthesis	SMCOG1235
Bc_ctg1_4286	B2	98.95530622	Bcer	L	Bcer	FALSE	Bc_Ga0417192_01_rn31	Other	NA
Bc_ctg1_4287	B2	15.73494268	Bcer	S	Bcer	FALSE	Bc_Ga0417192_01_rn31	Other	NA
Bc_ctg1_4288	B2	3.263543668	Bcer	M	Bcer	FALSE	Bc_Ga0417192_01_rn31	Other	NA
Bc_ctg1_4289	B2	33.68443286	Bcer	Q	Bcer	TRUE	Bc_Ga0417192_01_rn31	Core biosynthesis	None
Bc_ctg1_429	B2	0	Bcer	NA	Bcer	FALSE	Bc_Ga0417192_01_rn05	Other	NA
Bc_ctg1_4290	B2	30.30433406	Bcer	Q	Bcer	TRUE	Bc_Ga0417192_01_rn31	Core biosynthesis	None
Bc_ctg1_4291	B2	15.0356119	Bcer	IQ	Bcer	TRUE	Bc_Ga0417192_01_rn31	Ancillary biosynthesis	SMCOG1002
Bc_ctg1_4292	B2	1.981437227	Bcer	IQ	Bcer	TRUE	Bc_Ga0417192_01_rn31	Other	NA
Bc_ctg1_4293	B2	15.38527729	Bcer	IQ	Bcer	TRUE	Bc_Ga0417192_01_rn31	Other	NA
Bc_ctg1_4294	B2	14.80250164	Bcer	G	Bcer	FALSE	Bc_Ga0417192_01_rn31	Other	NA
Bc_ctg1_4295	B2	0	Bcer	S	Bcer	FALSE	Bc_Ga0417192_01_rn31	Other	NA
Bc_ctg1_4296	B2	1.398661572	Bcer	EGP	Bcer	FALSE	Bc_Ga0417192_01_rn31	Other	NA
Bc_ctg1_4297	B2	2.680768013	Bcer	S	Bcer	FALSE	NA	NA	NA
Bc_ctg1_4298	B2	43.59161899	Bcer	K	Bcer	FALSE	NA	NA	NA
Bc_ctg1_4299	B2	0.349665393	Bcer	S	Bcer	FALSE	NA	NA	NA
Bc_ctg1_43	B2	15.0356119	Bcer	K	Bcer	FALSE	NA	NA	NA
Bc_ctg1_430	B2	0.582775655	Bcer	Q	Bcer	TRUE	Bc_Ga0417192_01_rn05	Other	NA
Bc_ctg1_4300	B2	1.398661572	Bcer	S	Bcer	FALSE	NA	NA	NA
Bc_ctg1_4302	B2	31.23677511	Bcer	S	Bcer	FALSE	NA	NA	NA
Bc_ctg1_4303	B2	367.1486626	Bcer	D	Bcer	FALSE	NA	NA	NA
Bc_ctg1_4304	B2	2.447657751	Bcer	S	Bcer	FALSE	NA	NA	NA
Bc_ctg1_4305	B2	14.21972598	Bcer	F	Bcer	FALSE	NA	NA	NA
Bc_ctg1_4306	B2	92.8944394	Bcer	H	Bcer	FALSE	NA	NA	NA

Bc_ctg1_4307	B2	17.94949017	Bcer	V	Bcer	FALSE	NA	NA	NA
Bc_ctg1_4308	B2	25.40901856	Bcer	K	Bcer	FALSE	NA	NA	NA
Bc_ctg1_4309	B2	29.7215584	Bcer	O	Bcer	FALSE	NA	NA	NA
Bc_ctg1_431	B2	8.508524563	Bcer	S	Bcer	FALSE	NA	NA	NA
Bc_ctg1_4310	B2	6.643642467	Bcer	K	Bcer	FALSE	NA	NA	NA
Bc_ctg1_4311	B2	2.564212882	Bcer	C	Bcer	FALSE	NA	NA	NA
Bc_ctg1_4312	B2	1.631771834	Bcer	EGP	Bcer	FALSE	NA	NA	NA
Bc_ctg1_4313	B2	21.91236463	Bcer	NA	Bcer	FALSE	NA	NA	NA
Bc_ctg1_4314	B2	18.88193122	Bcer	S	Bcer	FALSE	NA	NA	NA
Bc_ctg1_4315	B2	2.680768013	Bcer	S	Bcer	FALSE	NA	NA	NA
Bc_ctg1_4316	B2	53.26569487	Bcer	S	Bcer	FALSE	NA	NA	NA
Bc_ctg1_4317	B2	61.3079989	Bcer	NT	Bcer	FALSE	NA	NA	NA
Bc_ctg1_4318	B2	11.53895797	Bcer	NA	Bcer	FALSE	NA	NA	NA
Bc_ctg1_4319	B2	7.576083515	Bcer	S	Bcer	FALSE	NA	NA	NA
Bc_ctg1_432	B2	4.895315502	Bcer	O	Bcer	FALSE	NA	NA	NA
Bc_ctg1_4320	B2	0	Bcer	S	Bcer	FALSE	NA	NA	NA
Bc_ctg1_4321	B2	171.3360426	Bcer	P	Bcer	FALSE	NA	NA	NA
Bc_ctg1_4322	B2	168.0724989	Bcer	S	Bcer	FALSE	NA	NA	NA
Bc_ctg1_4323	B2	2.097992358	Bcer	S	Bcer	FALSE	NA	NA	NA
Bc_ctg1_4326	B2	51.86703329	Bcer	C	Bcer	FALSE	NA	NA	NA
Bc_ctg1_4328	B2	2.680768013	Bcer	E	Bcer	FALSE	NA	NA	NA
Bc_ctg1_4329	B2	9.907186135	Bcer	J	Bcer	FALSE	NA	NA	NA
Bc_ctg1_433	B2	11.53895797	Bcer	G	Bcer	FALSE	NA	NA	NA
Bc_ctg1_4330	B2	272.0396757	Bcer	S	Bcer	FALSE	NA	NA	NA
Bc_ctg1_4331	B2	31.81955076	Bcer	S	Bcer	FALSE	NA	NA	NA
Bc_ctg1_4332	B2	1.981437227	Bcer	K	Bcer	FALSE	NA	NA	NA
Bc_ctg1_4333	B2	60.84177838	Bcer	P	Bcer	FALSE	NA	NA	NA
Bc_ctg1_4335	B2	28.78911736	Bcer	GM	Bcer	FALSE	NA	NA	NA
Bc_ctg1_4336	B2	14.80250164	Bcer	C	Bcer	FALSE	NA	NA	NA
Bc_ctg1_4337	B2	5.594646288	Bcer	L	Bcer	FALSE	NA	NA	NA
Bc_ctg1_4338	B2	33.33476747	Bcer	NU	Bcer	FALSE	NA	NA	NA
Bc_ctg1_4339	B2	5.82775655	Bcer	E	Bcer	FALSE	NA	NA	NA
Bc_ctg1_4340	B2	10.1402964	Bcer	H	Bcer	FALSE	NA	NA	NA
Bc_ctg1_4341	B2	5.128425764	Bcer	S	Bcer	FALSE	NA	NA	NA
Bc_ctg1_4342	B2	9.790631004	Bcer	G	Bcer	FALSE	NA	NA	NA
Bc_ctg1_4343	B2	1.398661572	Bcer	NA	Bcer	FALSE	NA	NA	NA
Bc_ctg1_4344	B2	8.858189956	Bcer	M	Bcer	FALSE	NA	NA	NA
Bc_ctg1_4345	B2	16.5508286	Bcer	F	Bcer	FALSE	NA	NA	NA
Bc_ctg1_4346	B2	48.72004476	Bcer	S	Bcer	FALSE	NA	NA	NA
Bc_ctg1_4347	B2	9.091300218	Bcer	S	Bcer	FALSE	NA	NA	NA
Bc_ctg1_4348	B2	66.20331441	Bcer	P	Bcer	FALSE	NA	NA	NA
Bc_ctg1_4349	B2	31.93610589	Bcer	S	Bcer	FALSE	NA	NA	NA
Bc_ctg1_435	B2	7.692638646	Bcer	T	Bcer	FALSE	NA	NA	NA
Bc_ctg1_4350	B2	43.00884334	Bcer	S	Bcer	FALSE	NA	NA	NA
Bc_ctg1_4351	B2	4.195984716	Bcer	I	Bcer	FALSE	NA	NA	NA
Bc_ctg1_4352	B2	0	Bcer	I	Bcer	FALSE	NA	NA	NA
Bc_ctg1_4353	B2	0.116555131	Bcer	M	Bcer	FALSE	NA	NA	NA
Bc_ctg1_4355	B2	1.981437227	Bcer	GM	Bcer	FALSE	NA	NA	NA
Bc_ctg1_4356	B2	14.68594651	Bcer	S	Bcer	FALSE	NA	NA	NA
Bc_ctg1_4357	B2	52.56636408	Bcer	S	Bcer	FALSE	NA	NA	NA
Bc_ctg1_4358	B2	0.233110262	Bcer	NA	Bcer	FALSE	NA	NA	NA
Bc_ctg1_4359	B2	2.680768013	Bcer	K	Bcer	FALSE	NA	NA	NA
Bc_ctg1_436	B2	13.5203952	Bcer	S	Bcer	FALSE	NA	NA	NA
Bc_ctg1_4360	B2	1.282106441	Bcer	S	Bcer	FALSE	NA	NA	NA
Bc_ctg1_4361	B2	232.4109312	Bcer	S	Bcer	FALSE	NA	NA	NA
Bc_ctg1_4362	B2	0	Bcer	L	Bcer	FALSE	NA	NA	NA
Bc_ctg1_4364	B2	84.73558023	Bcer	E	Bcer	FALSE	NA	NA	NA
Bc_ctg1_4365	B2	0	Bcer	NA	Bcer	FALSE	NA	NA	NA
Bc_ctg1_4367	B2	5.244980895	Bcer	J	Bcer	FALSE	NA	NA	NA
Bc_ctg1_4368	B2	5.711201419	Bcer	S	Bcer	FALSE	NA	NA	NA
Bc_ctg1_437	B2	7.342973253	Bcer	K	Bcer	FALSE	NA	NA	NA
Bc_ctg1_4370	B2	13.75350546	Bcer	M	Bcer	FALSE	NA	NA	NA

Bc_ctg1_4371	B2	1.748326965	Bcer	J	Bcer	FALSE	NA	NA	NA
Bc_ctg1_4372	B2	6.760197598	Bcer	M	Bcer	FALSE	NA	NA	NA
Bc_ctg1_4373	B2	3.146988537	Bcer	F	Bcer	FALSE	NA	NA	NA
Bc_ctg1_4374	B2	23.3110262	Bcer	J	Bcer	FALSE	NA	NA	NA
Bc_ctg1_4375	B2	8.275414301	Bcer	G	Bcer	FALSE	NA	NA	NA
Bc_ctg1_4376	B2	122.8491081	Bcer	OU	Bcer	FALSE	NA	NA	NA
Bc_ctg1_4377	B2	383.2332707	Bcer	O	Bcer	FALSE	NA	NA	NA
Bc_ctg1_4378	B2	1.16555131	Bcer	M	Bcer	FALSE	NA	NA	NA
Bc_ctg1_4379	B2	15.38527729	Bcer	G	Bcer	FALSE	NA	NA	NA
Bc_ctg1_438	B2	9.32441048	Bcer	S	Bcer	FALSE	NA	NA	NA
Bc_ctg1_4380	B2	9.557520742	Bcer	S	Bcer	FALSE	NA	NA	NA
Bc_ctg1_4381	B2	8.858189956	Bcer	K	Bcer	FALSE	NA	NA	NA
Bc_ctg1_4382	B2	3.613209061	Bcer	EGP	Bcer	FALSE	NA	NA	NA
Bc_ctg1_4383	B2	0.233110262	Bcer	S	Bcer	FALSE	NA	NA	NA
Bc_ctg1_4384	B2	110.4942642	Bcer	E	Bcer	FALSE	NA	NA	NA
Bc_ctg1_4385	B2	9.790631004	Bcer	S	Bcer	FALSE	NA	NA	NA
Bc_ctg1_4386	B2	16.20116321	Bcer	K	Bcer	FALSE	NA	NA	NA
Bc_ctg1_4387	B2	18.0660453	Bcer	S	Bcer	FALSE	NA	NA	NA
Bc_ctg1_4388	B2	99.77119213	Bcer	CG	Bcer	FALSE	NA	NA	NA
Bc_ctg1_4389	B2	34.84998417	Bcer	S	Bcer	FALSE	NA	NA	NA
Bc_ctg1_439	B2	2.33110262	Bcer	M	Bcer	FALSE	NA	NA	NA
Bc_ctg1_4390	B2	16.5508286	Bcer	K	Bcer	FALSE	NA	NA	NA
Bc_ctg1_4391	B2	2.214547489	Bcer	K	Bcer	FALSE	NA	NA	NA
Bc_ctg1_4392	B2	9.907186135	Bcer	T	Bcer	FALSE	NA	NA	NA
Bc_ctg1_4393	B2	6.060866812	Bcer	S	Bcer	FALSE	NA	NA	NA
Bc_ctg1_4394	B2	12.35484389	Bcer	J	Bcer	FALSE	NA	NA	NA
Bc_ctg1_4395	B2	22.26203002	Bcer	J	Bcer	FALSE	NA	NA	NA
Bc_ctg1_4396	B2	10.37340666	Bcer	M	Bcer	FALSE	NA	NA	NA
Bc_ctg1_4397	B2	1.515216703	Bcer	S	Bcer	FALSE	NA	NA	NA
Bc_ctg1_4398	B2	0.699330786	Bcer	K	Bcer	FALSE	NA	NA	NA
Bc_ctg1_4399	B2	0	Bcer	NA	Bcer	FALSE	NA	NA	NA
Bc_ctg1_44	B2	46.50549727	Bcer	GT	Bcer	FALSE	NA	NA	NA
Bc_ctg1_4400	B2	1.515216703	Bcer	S	Bcer	FALSE	NA	NA	NA
Bc_ctg1_4401	B2	2.447657751	Bcer	NA	Bcer	FALSE	NA	NA	NA
Bc_ctg1_4402	B2	0	Bcer	NA	Bcer	FALSE	NA	NA	NA
Bc_ctg1_4403	B2	0.815885917	Bcer	NA	Bcer	FALSE	NA	NA	NA
Bc_ctg1_4404	B2	104.5499525	Bcer	NA	Bcer	FALSE	NA	NA	NA
Bc_ctg1_4405	B2	11.88862336	Bcer	NA	Bcer	FALSE	NA	NA	NA
Bc_ctg1_4406	B2	0.349665393	Bcer	NA	Bcer	FALSE	NA	NA	NA
Bc_ctg1_4407	B2	2.913878275	Bcer	NA	Bcer	FALSE	NA	NA	NA
Bc_ctg1_4408	B2	0.349665393	Bcer	NA	Bcer	FALSE	NA	NA	NA
Bc_ctg1_441	B2	8.042304039	Bcer	M	Bcer	FALSE	NA	NA	NA
Bc_ctg1_4411	B2	0	Bcer	NA	Bcer	FALSE	NA	NA	NA
Bc_ctg1_4414	B2	0.116555131	Bcer	NA	Bcer	FALSE	NA	NA	NA
Bc_ctg1_4416	B2	0.116555131	Bcer	NA	Bcer	FALSE	NA	NA	NA
Bc_ctg1_4418	B2	0	Bcer	NA	Bcer	FALSE	NA	NA	NA
Bc_ctg1_4419	B2	0.349665393	Bcer	D	Bcer	FALSE	NA	NA	NA
Bc_ctg1_442	B2	15.50183242	Bcer	NA	Bcer	FALSE	NA	NA	NA
Bc_ctg1_4420	B2	0	Bcer	NA	Bcer	FALSE	NA	NA	NA
Bc_ctg1_4422	B2	1.515216703	Bcer	NA	Bcer	FALSE	NA	NA	NA
Bc_ctg1_4423	B2	0	Bcer	NA	Bcer	FALSE	NA	NA	NA
Bc_ctg1_4424	B2	0.699330786	Bcer	NA	Bcer	FALSE	NA	NA	NA
Bc_ctg1_4425	B2	1.748326965	Bcer	NA	Bcer	FALSE	NA	NA	NA
Bc_ctg1_4426	B2	0	Bcer	S	Bcer	FALSE	NA	NA	NA
Bc_ctg1_4427	B2	0	Bcer	L	Bcer	FALSE	NA	NA	NA
Bc_ctg1_4428	B2	11.18929258	Bcer	S	Bcer	FALSE	NA	NA	NA
Bc_ctg1_4429	B2	0.349665393	Bcer	S	Bcer	FALSE	NA	NA	NA
Bc_ctg1_443	B2	14.45283624	Bcer	M	Bcer	FALSE	NA	NA	NA
Bc_ctg1_4430	B2	258.9855011	Bcer	S	Bcer	FALSE	NA	NA	NA
Bc_ctg1_4431	B2	15.96805295	Bcer	NA	Bcer	FALSE	NA	NA	NA
Bc_ctg1_4433	B2	0	Bcer	NA	Bcer	FALSE	NA	NA	NA
Bc_ctg1_4434	B2	9.091300218	Bcer	Q	Bcer	TRUE	NA	NA	NA

Bc_ctg1_4435	B2	1483.979928	Bcer	F	Bcer	FALSE	NA	NA	NA
Bc_ctg1_4436	B2	35.66587009	Bcer	NA	Bcer	FALSE	NA	NA	NA
Bc_ctg1_4437	B2	97.67319978	Bcer	L	Bcer	FALSE	NA	NA	NA
Bc_ctg1_4438	B2	3.962874454	Bcer	G	Bcer	FALSE	NA	NA	NA
Bc_ctg1_4439	B2	6.760197598	Bcer	E	Bcer	FALSE	NA	NA	NA
Bc_ctg1_444	B2	2.913878275	Bcer	NA	Bcer	FALSE	NA	NA	NA
Bc_ctg1_4440	B2	30.30433406	Bcer	NA	Bcer	FALSE	NA	NA	NA
Bc_ctg1_4441	B2	1.981437227	Bcer	C	Bcer	FALSE	NA	NA	NA
Bc_ctg1_4442	B2	0.582775655	Bcer	K	Bcer	FALSE	NA	NA	NA
Bc_ctg1_4443	B2	4.895315502	Bcer	S	Bcer	FALSE	NA	NA	NA
Bc_ctg1_4444	B2	0	Bcer	NA	Bcer	FALSE	NA	NA	NA
Bc_ctg1_4445	B2	114.2240284	Bcer	S	Bcer	FALSE	NA	NA	NA
Bc_ctg1_4446	B2	4.66220524	Bcer	S	Bcer	FALSE	NA	NA	NA
Bc_ctg1_4447	B2	21.21303384	Bcer	O	Bcer	FALSE	NA	NA	NA
Bc_ctg1_4448	B2	2.564212882	Bcer	E	Bcer	FALSE	NA	NA	NA
Bc_ctg1_4449	B2	0.349665393	Bcer	S	Bcer	FALSE	NA	NA	NA
Bc_ctg1_445	B2	484.6362347	Bcer	NA	Bcer	FALSE	NA	NA	NA
Bc_ctg1_4450	B2	0.699330786	Bcer	S	Bcer	FALSE	NA	NA	NA
Bc_ctg1_4451	B2	438.7135131	Bcer	C	Bcer	FALSE	NA	NA	NA
Bc_ctg1_4452	B2	198.6099432	Bcer	C	Bcer	FALSE	NA	NA	NA
Bc_ctg1_4453	B2	68.4178619	Bcer	C	Bcer	FALSE	NA	NA	NA
Bc_ctg1_4454	B2	109.5618231	Bcer	C	Bcer	FALSE	NA	NA	NA
Bc_ctg1_4455	B2	29.95466867	Bcer	K	Bcer	FALSE	NA	NA	NA
Bc_ctg1_4456	B2	52.68291921	Bcer	H	Bcer	FALSE	NA	NA	NA
Bc_ctg1_4457	B2	58.51067576	Bcer	H	Bcer	FALSE	NA	NA	NA
Bc_ctg1_4458	B2	90.44678165	Bcer	H	Bcer	FALSE	NA	NA	NA
Bc_ctg1_4459	B2	18.88193122	Bcer	H	Bcer	FALSE	NA	NA	NA
Bc_ctg1_446	B2	17.01704913	Bcer	T	Bcer	FALSE	NA	NA	NA
Bc_ctg1_4460	B2	1.748326965	Bcer	H	Bcer	FALSE	NA	NA	NA
Bc_ctg1_4461	B2	99.07186135	Bcer	P	Bcer	FALSE	NA	NA	NA
Bc_ctg1_4462	B2	1.398661572	Bcer	S	Bcer	FALSE	NA	NA	NA
Bc_ctg1_4463	B2	1.981437227	Bcer	NA	Bcer	FALSE	NA	NA	NA
Bc_ctg1_4464	B2	17.59982478	Bcer	H	Bcer	FALSE	NA	NA	NA
Bc_ctg1_4465	B2	35.08309443	Bcer	S	Bcer	FALSE	NA	NA	NA
Bc_ctg1_4466	B2	89.28123034	Bcer	H	Bcer	FALSE	NA	NA	NA
Bc_ctg1_4467	B2	60.49211299	Bcer	P	Bcer	FALSE	NA	NA	NA
Bc_ctg1_4468	B2	571.236697	Bcer	C	Bcer	FALSE	NA	NA	NA
Bc_ctg1_4469	B2	6.060866812	Bcer	D	Bcer	FALSE	NA	NA	NA
Bc_ctg1_447	B2	1.981437227	Bcer	S	Bcer	FALSE	NA	NA	NA
Bc_ctg1_4470	B2	0.815885917	Bcer	M	Bcer	FALSE	NA	NA	NA
Bc_ctg1_4472	B2	0	Bcer	NA	Bcer	FALSE	NA	NA	NA
Bc_ctg1_4473	B2	9.907186135	Bcer	Q	Bcer	TRUE	NA	NA	NA
Bc_ctg1_4474	B2	6.177421943	Bcer	S	Bcer	FALSE	NA	NA	NA
Bc_ctg1_4475	B2	0	Bcer	NA	Bcer	FALSE	NA	NA	NA
Bc_ctg1_4476	B2	11.42240284	Bcer	S	Bcer	FALSE	NA	NA	NA
Bc_ctg1_4477	B2	0	Bcer	NA	Bcer	FALSE	NA	NA	NA
Bc_ctg1_448	B2	0.116555131	Bcer	S	Bcer	FALSE	NA	NA	NA
Bc_ctg1_4480	B2	31.81955076	Bcer	S	Bcer	FALSE	NA	NA	NA
Bc_ctg1_4481	B2	217.4918744	Bcer	S	Bcer	FALSE	NA	NA	NA
Bc_ctg1_4482	B2	288.0077287	Bcer	S	Bcer	FALSE	NA	NA	NA
Bc_ctg1_4483	B2	1.515216703	Bcer	S	Bcer	FALSE	NA	NA	NA
Bc_ctg1_4484	B2	0.116555131	Bcer	L	Bcer	FALSE	NA	NA	NA
Bc_ctg1_4485	B2	0	Bcer	NA	Bcer	FALSE	NA	NA	NA
Bc_ctg1_4486	B2	85.43491102	Bcer	C	Bcer	FALSE	NA	NA	NA
Bc_ctg1_4487	B2	17.01704913	Bcer	S	Bcer	FALSE	NA	NA	NA
Bc_ctg1_4488	B2	193.7146277	Bcer	S	Bcer	FALSE	NA	NA	NA
Bc_ctg1_4489	B2	19.81437227	Bcer	J	Bcer	FALSE	NA	NA	NA
Bc_ctg1_449	B2	2.680768013	Bcer	S	Bcer	FALSE	NA	NA	NA
Bc_ctg1_4490	B2	36.94797653	Bcer	J	Bcer	FALSE	NA	NA	NA
Bc_ctg1_4491	B2	1.048996179	Bcer	Q	Bcer	TRUE	NA	NA	NA
Bc_ctg1_4492	B2	43.59161899	Bcer	H	Bcer	FALSE	NA	NA	NA
Bc_ctg1_4493	B2	224.1355169	Bcer	J	Bcer	FALSE	NA	NA	NA

Bc_ctg1_4494	B2	7.226418122	Bcer	S	Bcer	FALSE	NA	NA	NA
Bc_ctg1_4495	B2	3.49665393	Bcer	K	Bcer	FALSE	NA	NA	NA
Bc_ctg1_4496	B2	6.643642467	Bcer	E	Bcer	FALSE	NA	NA	NA
Bc_ctg1_4498	B2	16.66738373	Bcer	J	Bcer	FALSE	NA	NA	NA
Bc_ctg1_4499	B2	17.59982478	Bcer	S	Bcer	FALSE	NA	NA	NA
Bc_ctg1_45	B2	1.282106441	Bcer	E	Bcer	FALSE	NA	NA	NA
Bc_ctg1_450	B2	30.88710971	Bcer	S	Bcer	FALSE	NA	NA	NA
Bc_ctg1_4500	B2	19.23159661	Bcer	T	Bcer	FALSE	NA	NA	NA
Bc_ctg1_4501	B2	6.643642467	Bcer	NA	Bcer	FALSE	NA	NA	NA
Bc_ctg1_4502	B2	283.9282991	Bcer	D	Bcer	FALSE	NA	NA	NA
Bc_ctg1_4503	B2	107.696941	Bcer	S	Bcer	FALSE	NA	NA	NA
Bc_ctg1_4504	B2	153.3865524	Bcer	KLT	Bcer	FALSE	NA	NA	NA
Bc_ctg1_4505	B2	210.3820114	Bcer	NU	Bcer	FALSE	NA	NA	NA
Bc_ctg1_4506	B2	6.060866812	Bcer	S	Bcer	FALSE	NA	NA	NA
Bc_ctg1_4507	B2	69.69996834	Bcer	MS	Bcer	FALSE	NA	NA	NA
Bc_ctg1_4508	B2	35.89898035	Bcer	S	Bcer	FALSE	NA	NA	NA
Bc_ctg1_4509	B2	12.23828875	Bcer	S	Bcer	FALSE	NA	NA	NA
Bc_ctg1_451	B2	46.27238701	Bcer	V	Bcer	FALSE	NA	NA	NA
Bc_ctg1_4510	B2	10.1402964	Bcer	I	Bcer	FALSE	NA	NA	NA
Bc_ctg1_4511	B2	54.8974667	Bcer	S	Bcer	FALSE	NA	NA	NA
Bc_ctg1_4512	B2	52.33325382	Bcer	U	Bcer	FALSE	NA	NA	NA
Bc_ctg1_4513	B2	2.214547489	Bcer	K	Bcer	FALSE	NA	NA	NA
Bc_ctg1_4514	B2	9.907186135	Bcer	EG	Bcer	FALSE	NA	NA	NA
Bc_ctg1_4515	B2	15.0356119	Bcer	C	Bcer	FALSE	NA	NA	NA
Bc_ctg1_4516	B2	7.109862991	Bcer	M	Bcer	FALSE	NA	NA	NA
Bc_ctg1_4517	B2	96.6242036	Bcer	S	Bcer	FALSE	NA	NA	NA
Bc_ctg1_4518	B2	27.74012118	Bcer	K	Bcer	FALSE	NA	NA	NA
Bc_ctg1_4519	B2	4.312539847	Bcer	S	Bcer	FALSE	NA	NA	NA
Bc_ctg1_452	B2	2.33110262	Bcer	NA	Bcer	FALSE	NA	NA	NA
Bc_ctg1_4520	B2	1.16555131	Bcer	K	Bcer	FALSE	NA	NA	NA
Bc_ctg1_4521	B2	37.18108679	Bcer	E	Bcer	FALSE	NA	NA	NA
Bc_ctg1_4522	B2	13.75350546	Bcer	K	Bcer	FALSE	NA	NA	NA
Bc_ctg1_4523	B2	7.226418122	Bcer	V	Bcer	FALSE	NA	NA	NA
Bc_ctg1_4524	B2	9.207855349	Bcer	U	Bcer	FALSE	NA	NA	NA
Bc_ctg1_4525	B2	280.4316452	Bcer	S	Bcer	FALSE	NA	NA	NA
Bc_ctg1_4526	B2	4.429094978	Bcer	S	Bcer	FALSE	NA	NA	NA
Bc_ctg1_4527	B2	1.515216703	Bcer	E	Bcer	FALSE	NA	NA	NA
Bc_ctg1_4528	B2	8.741634825	Bcer	S	Bcer	FALSE	NA	NA	NA
Bc_ctg1_4529	B2	63.28943613	Bcer	V	Bcer	FALSE	NA	NA	NA
Bc_ctg1_453	B2	83.57002892	Bcer	S	Bcer	FALSE	NA	NA	NA
Bc_ctg1_4530	B2	84.6190251	Bcer	F	Bcer	FALSE	NA	NA	NA
Bc_ctg1_4531	B2	23.07791594	Bcer	H	Bcer	FALSE	NA	NA	NA
Bc_ctg1_4532	B2	427.7573308	Bcer	M	Bcer	FALSE	NA	NA	NA
Bc_ctg1_4533	B2	96.15798307	Bcer	I	Bcer	FALSE	NA	NA	NA
Bc_ctg1_4534	B2	46.73860753	Bcer	I	Bcer	FALSE	NA	NA	NA
Bc_ctg1_4535	B2	101.8691845	Bcer	S	Bcer	FALSE	NA	NA	NA
Bc_ctg1_4536	B2	9.32441048	Bcer	U	Bcer	FALSE	NA	NA	NA
Bc_ctg1_4537	B2	95.92487281	Bcer	U	Bcer	FALSE	NA	NA	NA
Bc_ctg1_4538	B2	4.545650109	Bcer	S	Bcer	FALSE	NA	NA	NA
Bc_ctg1_4539	B2	146.8594651	Bcer	S	Bcer	FALSE	NA	NA	NA
Bc_ctg1_454	B2	2.33110262	Bcer	NA	Bcer	FALSE	NA	NA	NA
Bc_ctg1_4540	B2	18.4157107	Bcer	S	Bcer	FALSE	NA	NA	NA
Bc_ctg1_4541	B2	32.75199181	Bcer	S	Bcer	FALSE	NA	NA	NA
Bc_ctg1_4542	B2	0	Bcer	O	Bcer	FALSE	NA	NA	NA
Bc_ctg1_4543	B2	920.6689797	Bcer	E	Bcer	FALSE	NA	NA	NA
Bc_ctg1_4544	B2	1.981437227	Bcer	K	Bcer	FALSE	NA	NA	NA
Bc_ctg1_4545	B2	6.060866812	Bcer	NA	Bcer	FALSE	NA	NA	NA
Bc_ctg1_4546	B2	0	Bcer	M	Bcer	FALSE	NA	NA	NA
Bc_ctg1_4547	B2	116.0889105	Bcer	P	Bcer	FALSE	NA	NA	NA
Bc_ctg1_4548	B2	35.54931495	Bcer	E	Bcer	FALSE	NA	NA	NA
Bc_ctg1_4549	B2	77.97538264	Bcer	C	Bcer	FALSE	NA	NA	NA
Bc_ctg1_455	B2	0.116555131	Bcer	NA	Bcer	FALSE	NA	NA	NA

Bc_ctg1_4550	B2	72.26418122	Bcer	NA	Bcer	FALSE	NA	NA	NA
Bc_ctg1_4551	B2	25.75868395	Bcer	Q	Bcer	TRUE	NA	NA	NA
Bc_ctg1_4552	B2	0	Bcer	F	Bcer	FALSE	NA	NA	NA
Bc_ctg1_4553	B2	22.37858515	Bcer	M	Bcer	FALSE	NA	NA	NA
Bc_ctg1_4554	B2	12.70450928	Bcer	T	Bcer	FALSE	NA	NA	NA
Bc_ctg1_4555	B2	7.342973253	Bcer	K	Bcer	FALSE	NA	NA	NA
Bc_ctg1_4556	B2	67.83508624	Bcer	K	Bcer	FALSE	NA	NA	NA
Bc_ctg1_4557	B2	53.96502565	Bcer	S	Bcer	FALSE	NA	NA	NA
Bc_ctg1_4558	B2	5375.988862	Bcer	C	Bcer	FALSE	NA	NA	NA
Bc_ctg1_4559	B2	2.447657751	Bcer	EP	Bcer	FALSE	NA	NA	NA
Bc_ctg1_456	B2	3.613209061	Bcer	L	Bcer	FALSE	NA	NA	NA
Bc_ctg1_4560	B2	0.932441048	Bcer	EP	Bcer	FALSE	NA	NA	NA
Bc_ctg1_4562	B2	136.8357238	Bcer	G	Bcer	FALSE	NA	NA	NA
Bc_ctg1_4563	B2	78.55815829	Bcer	EG	Bcer	FALSE	NA	NA	NA
Bc_ctg1_4564	B2	72.61384661	Bcer	G	Bcer	FALSE	NA	NA	NA
Bc_ctg1_4565	B2	2.33110262	Bcer	S	Bcer	FALSE	NA	NA	NA
Bc_ctg1_4566	B2	0.233110262	Bcer	M	Bcer	FALSE	NA	NA	NA
Bc_ctg1_4567	B2	2.097992358	Bcer	O	Bcer	FALSE	NA	NA	NA
Bc_ctg1_4568	B2	14.21972598	Bcer	S	Bcer	FALSE	NA	NA	NA
Bc_ctg1_4569	B2	51.16770251	Bcer	EGP	Bcer	FALSE	NA	NA	NA
Bc_ctg1_457	B2	430.2049885	Bcer	K	Bcer	FALSE	NA	NA	NA
Bc_ctg1_4570	B2	24.8262429	Bcer	S	Bcer	FALSE	NA	NA	NA
Bc_ctg1_4571	B2	9.790631004	Bcer	E	Bcer	FALSE	NA	NA	NA
Bc_ctg1_4572	B2	21.21303384	Bcer	EM	Bcer	FALSE	NA	NA	NA
Bc_ctg1_4573	B2	42.30951255	Bcer	E	Bcer	FALSE	NA	NA	NA
Bc_ctg1_4574	B2	18.29915557	Bcer	NA	Bcer	FALSE	NA	NA	NA
Bc_ctg1_4575	B2	2.564212882	Bcer	V	Bcer	FALSE	NA	NA	NA
Bc_ctg1_4576	B2	0.699330786	Bcer	NA	Bcer	FALSE	NA	NA	NA
Bc_ctg1_4577	B2	47.08827292	Bcer	NA	Bcer	FALSE	NA	NA	NA
Bc_ctg1_4578	B2	1.398661572	Bcer	K	Bcer	FALSE	NA	NA	NA
Bc_ctg1_4579	B2	37.99697271	Bcer	S	Bcer	FALSE	NA	NA	NA
Bc_ctg1_458	B2	68.65097216	Bcer	NA	Bcer	FALSE	NA	NA	NA
Bc_ctg1_4580	B2	68.4178619	Bcer	C	Bcer	FALSE	NA	NA	NA
Bc_ctg1_4581	B2	98.72219595	Bcer	S	Bcer	FALSE	NA	NA	NA
Bc_ctg1_4582	B2	120.2848952	Bcer	T	Bcer	FALSE	NA	NA	NA
Bc_ctg1_4583	B2	0.233110262	Bcer	O	Bcer	FALSE	NA	NA	NA
Bc_ctg1_4584	B2	4.429094978	Bcer	H	Bcer	FALSE	NA	NA	NA
Bc_ctg1_4585	B2	2.33110262	Bcer	I	Bcer	FALSE	NA	NA	NA
Bc_ctg1_4586	B2	3.030433406	Bcer	I	Bcer	FALSE	NA	NA	NA
Bc_ctg1_4587	B2	3.030433406	Bcer	E	Bcer	FALSE	NA	NA	NA
Bc_ctg1_4588	B2	14.21972598	Bcer	K	Bcer	FALSE	NA	NA	NA
Bc_ctg1_4589	B2	32.05266102	Bcer	KT	Bcer	FALSE	NA	NA	NA
Bc_ctg1_459	B2	8.275414301	Bcer	C	Bcer	FALSE	NA	NA	NA
Bc_ctg1_4590	B2	13.63695033	Bcer	E	Bcer	FALSE	NA	NA	NA
Bc_ctg1_4591	B2	0.582775655	Bcer	S	Bcer	FALSE	NA	NA	NA
Bc_ctg1_4592	B2	1.864882096	Bcer	S	Bcer	FALSE	NA	NA	NA
Bc_ctg1_4593	B2	0	Bcer	NA	Bcer	FALSE	NA	NA	NA
Bc_ctg1_4596	B2	6.643642467	Bcer	NA	Bcer	FALSE	NA	NA	NA
Bc_ctg1_4597	B2	39.27907915	Bcer	KLT	Bcer	FALSE	NA	NA	NA
Bc_ctg1_4598	B2	57.57823471	Bcer	S	Bcer	FALSE	NA	NA	NA
Bc_ctg1_4599	B2	19.46470688	Bcer	I	Bcer	FALSE	NA	NA	NA
Bc_ctg1_46	B2	1.282106441	Bcer	S	Bcer	FALSE	NA	NA	NA
Bc_ctg1_460	B2	9.907186135	Bcer	S	Bcer	FALSE	NA	NA	NA
Bc_ctg1_4600	B2	1.631771834	Bcer	P	Bcer	FALSE	NA	NA	NA
Bc_ctg1_4602	B2	71.4482953	Bcer	CO	Bcer	FALSE	NA	NA	NA
Bc_ctg1_4603	B2	15.38527729	Bcer	J	Bcer	FALSE	NA	NA	NA
Bc_ctg1_4604	B2	0	Bcer	NA	Bcer	FALSE	NA	NA	NA
Bc_ctg1_4605	B2	0	Bcer	NA	Bcer	FALSE	NA	NA	NA
Bc_ctg1_4606	B2	0.582775655	Bcer	NA	Bcer	FALSE	NA	NA	NA
Bc_ctg1_4607	B2	0.699330786	Bcer	NA	Bcer	FALSE	NA	NA	NA
Bc_ctg1_4608	B2	1.515216703	Bcer	NA	Bcer	FALSE	NA	NA	NA
Bc_ctg1_4609	B2	0.116555131	Bcer	NA	Bcer	FALSE	NA	NA	NA

Bc_ctg1_461	B2	0.932441048	Bcer	F	Bcer	FALSE	NA	NA	NA
Bc_ctg1_4610	B2	11.6555131	Bcer	M	Bcer	FALSE	NA	NA	NA
Bc_ctg1_4611	B2	47.67104858	Bcer	M	Bcer	FALSE	NA	NA	NA
Bc_ctg1_4612	B2	0	Bcer	NA	Bcer	FALSE	NA	NA	NA
Bc_ctg1_4613	B2	0.349665393	Bcer	NA	Bcer	FALSE	NA	NA	NA
Bc_ctg1_4614	B2	0.582775655	Bcer	S	Bcer	FALSE	NA	NA	NA
Bc_ctg1_4616	B2	18.53226583	Bcer	NA	Bcer	FALSE	NA	NA	NA
Bc_ctg1_4617	B2	0.466220524	Bcer	NA	Bcer	FALSE	NA	NA	NA
Bc_ctg1_4618	B2	15.96805295	Bcer	IQ	Bcer	TRUE	NA	NA	NA
Bc_ctg1_4619	B2	0.582775655	Bcer	NA	Bcer	FALSE	NA	NA	NA
Bc_ctg1_462	B2	0	Bcer	S	Bcer	FALSE	NA	NA	NA
Bc_ctg1_4620	B2	1.515216703	Bcer	S	Bcer	FALSE	NA	NA	NA
Bc_ctg1_4621	B2	0	Bcer	S	Bcer	FALSE	NA	NA	NA
Bc_ctg1_4622	B2	699.9135616	Bcer	M	Bcer	FALSE	NA	NA	NA
Bc_ctg1_4623	B2	0	Bcer	NA	Bcer	FALSE	NA	NA	NA
Bc_ctg1_4624	B2	4.429094978	Bcer	EGP	Bcer	FALSE	NA	NA	NA
Bc_ctg1_4626	B2	3.846319323	Bcer	C	Bcer	FALSE	NA	NA	NA
Bc_ctg1_4627	B2	13.28728493	Bcer	S	Bcer	FALSE	Bc_Ga0417192_01_rn32	Other	NA
Bc_ctg1_4628	B2	7.925748908	Bcer	G	Bcer	FALSE	Bc_Ga0417192_01_rn32	Ancillary biosynthesis	SMCOG1231
Bc_ctg1_4629	B2	88.11567903	Bcer	C	Bcer	FALSE	Bc_Ga0417192_01_rn32	Ancillary biosynthesis	SMCOG1006
Bc_ctg1_463	B2	6.177421943	Bcer	S	Bcer	FALSE	NA	NA	NA
Bc_ctg1_4630	B2	50.0021512	Bcer	I	Bcer	FALSE	Bc_Ga0417192_01_rn32	Ancillary biosynthesis	SMCOG1100
Bc_ctg1_4631	B2	107.2307205	Bcer	C	Bcer	FALSE	Bc_Ga0417192_01_rn32	Ancillary biosynthesis	SMCOG1017
Bc_ctg1_4632	B2	3.846319323	Bcer	E	Bcer	FALSE	Bc_Ga0417192_01_rn32	Transport	SMCOG1116
Bc_ctg1_4633	B2	203.1555933	Bcer	I	Bcer	FALSE	Bc_Ga0417192_01_rn32	Ancillary biosynthesis	SMCOG1023
Bc_ctg1_4634	B2	2.33110262	Bcer	NA	Bcer	FALSE	Bc_Ga0417192_01_rn32	Other	NA
Bc_ctg1_4635	B2	2.097992358	Bcer	S	Bcer	FALSE	Bc_Ga0417192_01_rn32	Other	SMCOG1300
Bc_ctg1_4636	B2	37.99697271	Bcer	L	Bcer	FALSE	Bc_Ga0417192_01_rn32	Other	SMCOG1230
Bc_ctg1_4637	B2	118.6531234	Bcer	L	Bcer	FALSE	Bc_Ga0417192_01_rn32	Other	SMCOG1218
Bc_ctg1_4639	B2	31.81955076	Bcer	K	Bcer	FALSE	Bc_Ga0417192_01_rn32	Regulation	SMCOG1058
Bc_ctg1_464	B2	18.18260044	Bcer	M	Bcer	FALSE	NA	NA	NA
Bc_ctg1_4640	B2	0	Bcer	NA	Bcer	FALSE	Bc_Ga0417192_01_rn32	Other	NA
Bc_ctg1_4641	B2	577.414119	Bcer	P	Bcer	FALSE	Bc_Ga0417192_01_rn32	Transport	SMCOG1137
Bc_ctg1_4642	B2	28.90567249	Bcer	IQ	Bcer	TRUE	Bc_Ga0417192_01_rn32	Ancillary biosynthesis	SMCOG1001
Bc_ctg1_4643	B2	61.89077456	Bcer	HQ	Bcer	TRUE	Bc_Ga0417192_01_rn32	Ancillary biosynthesis	SMCOG1018
Bc_ctg1_4644	B2	93.94343558	Bcer	Q	Bcer	TRUE	Bc_Ga0417192_01_rn32	Core biosynthesis	SMCOG1002
Bc_ctg1_4645	B2	35.43275982	Bcer	Q	Bcer	TRUE	Bc_Ga0417192_01_rn32	Ancillary biosynthesis	SMCOG1027
Bc_ctg1_4646	B2	395.5881146	Bcer	Q	Bcer	TRUE	Bc_Ga0417192_01_rn32	Core biosynthesis	SMCOG1127
Bc_ctg1_4647	B2	3.729764192	Bcer	S	Bcer	FALSE	Bc_Ga0417192_01_rn32	Ancillary biosynthesis	SMCOG1009
Bc_ctg1_4648	B2	25.52557369	Bcer	P	Bcer	FALSE	Bc_Ga0417192_01_rn32	Transport	SMCOG1005
Bc_ctg1_4649	B2	13.40384006	Bcer	H	Bcer	FALSE	Bc_Ga0417192_01_rn32	Ancillary biosynthesis	SMCOG1012
Bc_ctg1_465	B2	64.6880977	Bcer	IQ	Bcer	TRUE	Bc_Ga0417192_01_rn06	Other	NA
Bc_ctg1_4650	B2	19.81437227	Bcer	H	Bcer	FALSE	Bc_Ga0417192_01_rn32	Ancillary biosynthesis	SMCOG1259
Bc_ctg1_4651	B2	17.48326965	Bcer	L	Bcer	FALSE	Bc_Ga0417192_01_rn32	Other	SMCOG1132
Bc_ctg1_4652	B2	19.34815175	Bcer	S	Bcer	FALSE	Bc_Ga0417192_01_rn32	Other	SMCOG1267
Bc_ctg1_4653	B2	26.57456987	Bcer	S	Bcer	FALSE	Bc_Ga0417192_01_rn32	Other	SMCOG1250
Bc_ctg1_4654	B2	0.349665393	Bcer	O	Bcer	FALSE	Bc_Ga0417192_01_rn32	Ancillary biosynthesis	SMCOG1075
Bc_ctg1_4655	B2	14.45283624	Bcer	E	Bcer	FALSE	Bc_Ga0417192_01_rn32	Ancillary biosynthesis	SMCOG1153
Bc_ctg1_4656	B2	6.99330786	Bcer	NA	Bcer	FALSE	Bc_Ga0417192_01_rn32	Other	SMCOG1289
Bc_ctg1_4657	B2	0	Bcer	NA	Bcer	FALSE	Bc_Ga0417192_01_rn32	Other	NA
Bc_ctg1_4658	B2	1.16555131	Bcer	G	Bcer	FALSE	Bc_Ga0417192_01_rn32	Ancillary biosynthesis	SMCOG1258
Bc_ctg1_4659	B2	42.42606768	Bcer	J	Bcer	FALSE	Bc_Ga0417192_01_rn32	Ancillary biosynthesis	SMCOG1154
Bc_ctg1_466	B2	17.25015939	Bcer	S	Bcer	FALSE	Bc_Ga0417192_01_rn06	Other	NA
Bc_ctg1_4660	B2	80.42304039	Bcer	J	Bcer	FALSE	Bc_Ga0417192_01_rn32	Other	SMCOG1162
Bc_ctg1_4661	B2	5.478091157	Bcer	V	Bcer	FALSE	Bc_Ga0417192_01_rn32	Transport	SMCOG1000
Bc_ctg1_4662	B2	28.2063417	Bcer	V	Bcer	FALSE	Bc_Ga0417192_01_rn32	Transport	SMCOG1243
Bc_ctg1_4663	B2	6.760197598	Bcer	G	Bcer	FALSE	Bc_Ga0417192_01_rn32	Transport	SMCOG1020
Bc_ctg1_4664	B2	4.66220524	Bcer	P	Bcer	FALSE	Bc_Ga0417192_01_rn32	Transport	SMCOG1118
Bc_ctg1_4665	B2	23.42758133	Bcer	P	Bcer	FALSE	Bc_Ga0417192_01_rn32	Transport	SMCOG1085
Bc_ctg1_4666	B2	3.380098799	Bcer	EH	Bcer	FALSE	NA	NA	NA
Bc_ctg1_4667	B2	0.699330786	Bcer	P	Bcer	FALSE	NA	NA	NA
Bc_ctg1_4668	B2	22.72825054	Bcer	S	Bcer	FALSE	NA	NA	NA

Bc_ctg1_4669	B2	16.31771834	Bcer	F	Bcer	FALSE	NA	NA	NA
Bc_ctg1_467	B2	1.631771834	Bcer	E	Bcer	FALSE	Bc_Ga0417192_01_rn06	Ancillary biosynthesis	SMCOG1063
Bc_ctg1_4670	B2	5.128425764	Bcer	S	Bcer	FALSE	NA	NA	NA
Bc_ctg1_4671	B2	5.711201419	Bcer	NA	Bcer	FALSE	NA	NA	NA
Bc_ctg1_4673	B2	0.116555131	Bcer	K	Bcer	FALSE	NA	NA	NA
Bc_ctg1_4674	B2	0.116555131	Bcer	S	Bcer	FALSE	NA	NA	NA
Bc_ctg1_4675	B2	3.380098799	Bcer	H	Bcer	FALSE	NA	NA	NA
Bc_ctg1_4676	B2	1.282106441	Bcer	K	Bcer	FALSE	NA	NA	NA
Bc_ctg1_4677	B2	2.214547489	Bcer	K	Bcer	FALSE	NA	NA	NA
Bc_ctg1_4678	B2	4.312539847	Bcer	S	Bcer	FALSE	NA	NA	NA
Bc_ctg1_4679	B2	12.12173362	Bcer	K	Bcer	FALSE	NA	NA	NA
Bc_ctg1_468	B2	74.24561845	Bcer	V	Bcer	FALSE	Bc_Ga0417192_01_rn06	Other	NA
Bc_ctg1_4680	B2	12.58795415	Bcer	S	Bcer	FALSE	NA	NA	NA
Bc_ctg1_4682	B2	9.091300218	Bcer	V	Bcer	FALSE	NA	NA	NA
Bc_ctg1_4683	B2	1.282106441	Bcer	K	Bcer	FALSE	NA	NA	NA
Bc_ctg1_4684	B2	3.49665393	Bcer	K	Bcer	FALSE	NA	NA	NA
Bc_ctg1_4685	B2	8.508524563	Bcer	J	Bcer	FALSE	NA	NA	NA
Bc_ctg1_4686	B2	2.33110262	Bcer	EG	Bcer	FALSE	NA	NA	NA
Bc_ctg1_4687	B2	2.097992358	Bcer	K	Bcer	FALSE	NA	NA	NA
Bc_ctg1_4688	B2	10.83962718	Bcer	NA	Bcer	FALSE	NA	NA	NA
Bc_ctg1_4689	B2	0	Bcer	NA	Bcer	FALSE	NA	NA	NA
Bc_ctg1_469	B2	4.66220524	Bcer	F	Bcer	FALSE	Bc_Ga0417192_01_rn06	Other	NA
Bc_ctg1_4690	B2	81.35548144	Bcer	H	Bcer	FALSE	NA	NA	NA
Bc_ctg1_4691	B2	1.048996179	Bcer	NA	Bcer	FALSE	NA	NA	NA
Bc_ctg1_4692	B2	100.1208575	Bcer	J	Bcer	FALSE	NA	NA	NA
Bc_ctg1_4693	B2	20.97992358	Bcer	S	Bcer	FALSE	NA	NA	NA
Bc_ctg1_4694	B2	35.43275982	Bcer	K	Bcer	FALSE	NA	NA	NA
Bc_ctg1_4695	B2	1.16555131	Bcer	S	Bcer	FALSE	NA	NA	NA
Bc_ctg1_4696	B2	6.99330786	Bcer	F	Bcer	FALSE	NA	NA	NA
Bc_ctg1_4697	B2	5.011870633	Bcer	NA	Bcer	FALSE	NA	NA	NA
Bc_ctg1_4698	B2	2.680768013	Bcer	K	Bcer	FALSE	NA	NA	NA
Bc_ctg1_4699	B2	6.876752729	Bcer	E	Bcer	FALSE	NA	NA	NA
Bc_ctg1_47	B2	0.582775655	Bcer	K	Bcer	FALSE	NA	NA	NA
Bc_ctg1_470	B2	10.1402964	Bcer	H	Bcer	FALSE	Bc_Ga0417192_01_rn06	Other	NA
Bc_ctg1_4700	B2	1.398661572	Bcer	NA	Bcer	FALSE	NA	NA	NA
Bc_ctg1_4701	B2	5.944311681	Bcer	S	Bcer	FALSE	NA	NA	NA
Bc_ctg1_4702	B2	14.10317085	Bcer	K	Bcer	FALSE	NA	NA	NA
Bc_ctg1_4703	B2	8.508524563	Bcer	K	Bcer	FALSE	NA	NA	NA
Bc_ctg1_4704	B2	0	Bcer	S	Bcer	FALSE	NA	NA	NA
Bc_ctg1_4705	B2	53.49880513	Bcer	S	Bcer	FALSE	NA	NA	NA
Bc_ctg1_4706	B2	37.53075218	Bcer	S	Bcer	FALSE	NA	NA	NA
Bc_ctg1_4707	B2	63.172881	Bcer	V	Bcer	FALSE	NA	NA	NA
Bc_ctg1_4708	B2	72.73040174	Bcer	V	Bcer	FALSE	NA	NA	NA
Bc_ctg1_4709	B2	1.282106441	Bcer	NA	Bcer	FALSE	NA	NA	NA
Bc_ctg1_471	B2	1.048996179	Bcer	S	Bcer	FALSE	Bc_Ga0417192_01_rn06	Other	NA
Bc_ctg1_4710	B2	0.349665393	Bcer	S	Bcer	FALSE	NA	NA	NA
Bc_ctg1_4711	B2	10.48996179	Bcer	NA	Bcer	FALSE	NA	NA	NA
Bc_ctg1_4712	B2	6.876752729	Bcer	NA	Bcer	FALSE	NA	NA	NA
Bc_ctg1_4713	B2	9.790631004	Bcer	E	Bcer	FALSE	NA	NA	NA
Bc_ctg1_4714	B2	7.109862991	Bcer	K	Bcer	FALSE	NA	NA	NA
Bc_ctg1_4715	B2	0.233110262	Bcer	NA	Bcer	FALSE	NA	NA	NA
Bc_ctg1_4716	B2	4.429094978	Bcer	M	Bcer	FALSE	NA	NA	NA
Bc_ctg1_4717	B2	0.699330786	Bcer	M	Bcer	FALSE	NA	NA	NA
Bc_ctg1_4719	B2	0	Bcer	G	Bcer	FALSE	NA	NA	NA
Bc_ctg1_472	B2	66.5529798	Bcer	C	Bcer	FALSE	Bc_Ga0417192_01_rn06	Ancillary biosynthesis	SMCOG1017
Bc_ctg1_4720	B2	0	Bcer	NA	Bcer	FALSE	NA	NA	NA
Bc_ctg1_4721	B2	2.913878275	Bcer	H	Bcer	FALSE	NA	NA	NA
Bc_ctg1_4722	B2	3.613209061	Bcer	K	Bcer	FALSE	NA	NA	NA
Bc_ctg1_4724	B2	22.49514028	Bcer	S	Bcer	FALSE	NA	NA	NA
Bc_ctg1_4725	B2	151.9878908	Bcer	P	Bcer	FALSE	NA	NA	NA
Bc_ctg1_4726	B2	309.5704279	Bcer	S	Bcer	FALSE	NA	NA	NA
Bc_ctg1_4727	B2	1.16555131	Bcer	T	Bcer	FALSE	NA	NA	NA

Bc_ctg1_4728	B2	0	Bcer	G	Bcer	FALSE	NA	NA	NA
Bc_ctg1_4729	B2	1.048996179	Bcer	G	Bcer	FALSE	NA	NA	NA
Bc_ctg1_473	B2	37.99697271	Bcer	IQ	Bcer	TRUE	Bc_Ga0417192_01_rn06	Ancillary biosynthesis	SMCOG1001
Bc_ctg1_4730	B2	0	Bcer	NA	Bcer	FALSE	NA	NA	NA
Bc_ctg1_4731	B2	0	Bcer	O	Bcer	FALSE	NA	NA	NA
Bc_ctg1_4732	B2	1.16555131	Bcer	G	Bcer	FALSE	NA	NA	NA
Bc_ctg1_4733	B2	3.962874454	Bcer	I	Bcer	FALSE	NA	NA	NA
Bc_ctg1_4734	B2	12.93761954	Bcer	E	Bcer	FALSE	NA	NA	NA
Bc_ctg1_4735	B2	7.692638646	Bcer	E	Bcer	FALSE	NA	NA	NA
Bc_ctg1_4736	B2	1.282106441	Bcer	K	Bcer	FALSE	NA	NA	NA
Bc_ctg1_4737	B2	0.116555131	Bcer	EGP	Bcer	FALSE	NA	NA	NA
Bc_ctg1_4738	B2	2.913878275	Bcer	S	Bcer	FALSE	NA	NA	NA
Bc_ctg1_4739	B2	3.49665393	Bcer	S	Bcer	FALSE	NA	NA	NA
Bc_ctg1_4741	B2	159.0977538	Bcer	L	Bcer	FALSE	NA	NA	NA
Bc_ctg1_4742	B2	3.380098799	Bcer	NA	Bcer	FALSE	NA	NA	NA
Bc_ctg1_4743	B2	6.527087336	Bcer	K	Bcer	FALSE	NA	NA	NA
Bc_ctg1_4744	B2	5.128425764	Bcer	EGP	Bcer	FALSE	NA	NA	NA
Bc_ctg1_4746	B2	0.116555131	Bcer	NA	Bcer	FALSE	NA	NA	NA
Bc_ctg1_4747	B2	0.466220524	Bcer	S	Bcer	FALSE	NA	NA	NA
Bc_ctg1_4748	B2	0	Bcer	NA	Bcer	FALSE	NA	NA	NA
Bc_ctg1_4749	B2	0.116555131	Bcer	NA	Bcer	FALSE	NA	NA	NA
Bc_ctg1_475	B2	5.244980895	Bcer	I	Bcer	FALSE	Bc_Ga0417192_01_rn06	Core biosynthesis	None
Bc_ctg1_4750	B2	0	Bcer	NA	Bcer	FALSE	NA	NA	NA
Bc_ctg1_4751	B2	1.282106441	Bcer	NA	Bcer	FALSE	NA	NA	NA
Bc_ctg1_4754	B2	0	Bcer	E	Bcer	FALSE	NA	NA	NA
Bc_ctg1_4755	B2	0.932441048	Bcer	N	Bcer	FALSE	NA	NA	NA
Bc_ctg1_4756	B2	0	Bcer	NA	Bcer	FALSE	NA	NA	NA
Bc_ctg1_4758	B2	0.815885917	Bcer	NA	Bcer	FALSE	NA	NA	NA
Bc_ctg1_4759	B2	4.545650109	Bcer	NA	Bcer	FALSE	NA	NA	NA
Bc_ctg1_476	B2	18.64882096	Bcer	S	Bcer	FALSE	Bc_Ga0417192_01_rn06	Other	NA
Bc_ctg1_4760	B2	0	Bcer	S	Bcer	FALSE	NA	NA	NA
Bc_ctg1_4761	B2	0.466220524	Bcer	NA	Bcer	FALSE	NA	NA	NA
Bc_ctg1_4762	B2	0.582775655	Bcer	NA	Bcer	FALSE	NA	NA	NA
Bc_ctg1_4763	B2	2.680768013	Bcer	NA	Bcer	FALSE	NA	NA	NA
Bc_ctg1_4765	B2	2.680768013	Bcer	Q	Bcer	TRUE	NA	NA	NA
Bc_ctg1_4766	B2	3.962874454	Bcer	S	Bcer	FALSE	NA	NA	NA
Bc_ctg1_4767	B2	8.741634825	Bcer	Q	Bcer	TRUE	NA	NA	NA
Bc_ctg1_4768	B2	1.048996179	Bcer	GH	Bcer	FALSE	NA	NA	NA
Bc_ctg1_4769	B2	259.3351665	Bcer	K	Bcer	FALSE	NA	NA	NA
Bc_ctg1_477	B2	44.64061517	Bcer	S	Bcer	FALSE	Bc_Ga0417192_01_rn06	Transport	SMCOG1166
Bc_ctg1_4770	B2	236.8400262	Bcer	G	Bcer	FALSE	Bc_Ga0417192_01_rn33	Ancillary biosynthesis	SMCOG1101
Bc_ctg1_4771	B2	163.7599591	Bcer	S	Bcer	FALSE	Bc_Ga0417192_01_rn33	Other	NA
Bc_ctg1_4773	B2	1.16555131	Bcer	NA	Bcer	FALSE	Bc_Ga0417192_01_rn33	Other	NA
Bc_ctg1_4774	B2	0.233110262	Bcer	S	Bcer	FALSE	Bc_Ga0417192_01_rn33	Other	NA
Bc_ctg1_4775	B2	980.8114273	Bcer	E	Bcer	FALSE	Bc_Ga0417192_01_rn33	Ancillary biosynthesis	SMCOG1247
Bc_ctg1_4776	B2	0.233110262	Bcer	S	Bcer	FALSE	Bc_Ga0417192_01_rn33	Other	NA
Bc_ctg1_4777	B2	0.699330786	Bcer	NA	Bcer	FALSE	Bc_Ga0417192_01_rn33	Other	NA
Bc_ctg1_4778	B2	7.576083515	Bcer	E	Bcer	FALSE	Bc_Ga0417192_01_rn33	Other	NA
Bc_ctg1_4779	B2	0.466220524	Bcer	KT	Bcer	FALSE	Bc_Ga0417192_01_rn33	Other	NA
Bc_ctg1_478	B2	0.116555131	Bcer	S	Bcer	FALSE	Bc_Ga0417192_01_rn06	Other	NA
Bc_ctg1_4781	B2	1.864882096	Bcer	K	Bcer	FALSE	Bc_Ga0417192_01_rn33	Other	NA
Bc_ctg1_4782	B2	1.282106441	Bcer	K	Bcer	FALSE	Bc_Ga0417192_01_rn33	Other	NA
Bc_ctg1_4783	B2	4.66220524	Bcer	NA	Bcer	FALSE	Bc_Ga0417192_01_rn33	Other	NA
Bc_ctg1_4784	B2	0.582775655	Bcer	S	Bcer	FALSE	Bc_Ga0417192_01_rn33	Other	NA
Bc_ctg1_4785	B2	13.98661572	Bcer	S	Bcer	FALSE	Bc_Ga0417192_01_rn33	Ancillary biosynthesis	SMCOG1156
Bc_ctg1_4786	B2	2.214547489	Bcer	M	Bcer	FALSE	Bc_Ga0417192_01_rn33	Other	NA
Bc_ctg1_4787	B2	2.797323144	Bcer	I	Bcer	FALSE	Bc_Ga0417192_01_rn33	Ancillary biosynthesis	SMCOG1088
Bc_ctg1_4788	B2	18.64882096	Bcer	E	Bcer	FALSE	Bc_Ga0417192_01_rn33	Other	NA
Bc_ctg1_4789	B2	2.33110262	Bcer	EGP	Bcer	FALSE	Bc_Ga0417192_01_rn33	Transport	SMCOG1020
Bc_ctg1_479	B2	4.079429585	Bcer	NA	Bcer	FALSE	Bc_Ga0417192_01_rn06	Other	NA
Bc_ctg1_4790	B2	10.83962718	Bcer	Q	Bcer	TRUE	Bc_Ga0417192_01_rn33	Core biosynthesis	SMCOG1002
Bc_ctg1_4791	B2	15.38527729	Bcer	Q	Bcer	TRUE	Bc_Ga0417192_01_rn33	Core biosynthesis	SMCOG1127

Bc_ctg1_4792	B2	75.06150436	Bcer	Q	Bcer	TRUE	Bc_Ga0417192_01_rn33	Core biosynthesis	SMCOG1127
Bc_ctg1_4793	B2	2.214547489	Bcer	H	Bcer	FALSE	Bc_Ga0417192_01_rn33	Ancillary biosynthesis	SMCOG1012
Bc_ctg1_4794	B2	1.748326965	Bcer	Q	Bcer	TRUE	Bc_Ga0417192_01_rn33	Ancillary biosynthesis	SMCOG1004
Bc_ctg1_4795	B2	3.613209061	Bcer	P	Bcer	FALSE	Bc_Ga0417192_01_rn33	Other	NA
Bc_ctg1_4796	B2	2.097992358	Bcer	E	Bcer	FALSE	Bc_Ga0417192_01_rn33	Ancillary biosynthesis	SMCOG1153
Bc_ctg1_4798	B2	2.214547489	Bcer	E	Bcer	FALSE	Bc_Ga0417192_01_rn33	Other	NA
Bc_ctg1_4799	B2	23.3110262	Bcer	T	Bcer	FALSE	Bc_Ga0417192_01_rn33	Other	NA
Bc_ctg1_48	B2	1.398661572	Bcer	IQ	Bcer	TRUE	NA	NA	NA
Bc_ctg1_480	B2	2.214547489	Bcer	NA	Bcer	FALSE	Bc_Ga0417192_01_rn06	Other	NA
Bc_ctg1_4800	B2	18.64882096	Bcer	T	Bcer	FALSE	Bc_Ga0417192_01_rn33	Regulation	SMCOG1008
Bc_ctg1_4801	B2	0.233110262	Bcer	EG	Bcer	FALSE	Bc_Ga0417192_01_rn33	Other	NA
Bc_ctg1_4802	B2	0.233110262	Bcer	S	Bcer	FALSE	Bc_Ga0417192_01_rn33	Ancillary biosynthesis	SMCOG1038
Bc_ctg1_4803	B2	0.233110262	Bcer	S	Bcer	FALSE	Bc_Ga0417192_01_rn33	Other	NA
Bc_ctg1_4804	B2	0.233110262	Bcer	E	Bcer	FALSE	Bc_Ga0417192_01_rn33	Other	NA
Bc_ctg1_4805	B2	0.815885917	Bcer	E	Bcer	FALSE	Bc_Ga0417192_01_rn33	Ancillary biosynthesis	SMCOG1038
Bc_ctg1_4806	B2	3.729764192	Bcer	L	Bcer	FALSE	Bc_Ga0417192_01_rn33	Other	NA
Bc_ctg1_4807	B2	9.091300218	Bcer	K	Bcer	FALSE	NA	NA	NA
Bc_ctg1_4808	B2	1024.869267	Bcer	S	Bcer	FALSE	NA	NA	NA
Bc_ctg1_4809	B2	911.2280141	Bcer	E	Bcer	FALSE	NA	NA	NA
Bc_ctg1_481	B2	24.47657751	Bcer	D	Bcer	FALSE	Bc_Ga0417192_01_rn06	Other	SMCOG1157
Bc_ctg1_4810	B2	1165.900975	Bcer	E	Bcer	FALSE	NA	NA	NA
Bc_ctg1_4811	B2	98.25597543	Bcer	E	Bcer	FALSE	NA	NA	NA
Bc_ctg1_4812	B2	63.52254639	Bcer	T	Bcer	FALSE	NA	NA	NA
Bc_ctg1_4813	B2	0	Bcer	P	Bcer	FALSE	NA	NA	NA
Bc_ctg1_4814	B2	0.233110262	Bcer	E	Bcer	FALSE	NA	NA	NA
Bc_ctg1_4815	B2	0.116555131	Bcer	S	Bcer	FALSE	NA	NA	NA
Bc_ctg1_4817	B2	2.564212882	Bcer	M	Bcer	FALSE	NA	NA	NA
Bc_ctg1_4818	B2	38.81285862	Bcer	J	Bcer	FALSE	NA	NA	NA
Bc_ctg1_4819	B2	0.233110262	Bcer	NA	Bcer	FALSE	NA	NA	NA
Bc_ctg1_482	B2	0.233110262	Bcer	H	Bcer	FALSE	Bc_Ga0417192_01_rn06	Other	NA
Bc_ctg1_4820	B2	32.51888155	Bcer	M	Bcer	FALSE	NA	NA	NA
Bc_ctg1_4821	B2	4.079429585	Bcer	K	Bcer	FALSE	NA	NA	NA
Bc_ctg1_4822	B2	0.699330786	Bcer	NA	Bcer	FALSE	NA	NA	NA
Bc_ctg1_4823	B2	3.729764192	Bcer	G	Bcer	FALSE	NA	NA	NA
Bc_ctg1_4824	B2	4.66220524	Bcer	K	Bcer	FALSE	NA	NA	NA
Bc_ctg1_4825	B2	6.876752729	Bcer	E	Bcer	FALSE	NA	NA	NA
Bc_ctg1_4826	B2	18.64882096	Bcer	T	Bcer	FALSE	NA	NA	NA
Bc_ctg1_4827	B2	5.361536026	Bcer	T	Bcer	FALSE	NA	NA	NA
Bc_ctg1_4828	B2	1.864882096	Bcer	O	Bcer	FALSE	NA	NA	NA
Bc_ctg1_4829	B2	1.048996179	Bcer	S	Bcer	FALSE	NA	NA	NA
Bc_ctg1_483	B2	2.913878275	Bcer	H	Bcer	FALSE	Bc_Ga0417192_01_rn06	Other	NA
Bc_ctg1_4830	B2	4.195984716	Bcer	S	Bcer	FALSE	NA	NA	NA
Bc_ctg1_4834	B2	7.692638646	Bcer	KT	Bcer	FALSE	NA	NA	NA
Bc_ctg1_4835	B2	6.643642467	Bcer	NA	Bcer	FALSE	NA	NA	NA
Bc_ctg1_4836	B2	0.699330786	Bcer	NA	Bcer	FALSE	NA	NA	NA
Bc_ctg1_4837	B2	101.0532986	Bcer	NA	Bcer	FALSE	NA	NA	NA
Bc_ctg1_4838	B2	0.233110262	Bcer	NA	Bcer	FALSE	NA	NA	NA
Bc_ctg1_4839	B2	3.030433406	Bcer	NA	Bcer	FALSE	NA	NA	NA
Bc_ctg1_484	B2	14.91905677	Bcer	H	Bcer	FALSE	Bc_Ga0417192_01_rn06	Other	NA
Bc_ctg1_4840	B2	1.864882096	Bcer	S	Bcer	FALSE	NA	NA	NA
Bc_ctg1_4841	B2	0	Bcer	NA	Bcer	FALSE	NA	NA	NA
Bc_ctg1_4842	B2	16.31771834	Bcer	E	Bcer	FALSE	NA	NA	NA
Bc_ctg1_4843	B2	15.61838755	Bcer	E	Bcer	FALSE	NA	NA	NA
Bc_ctg1_4844	B2	27.15734552	Bcer	E	Bcer	FALSE	NA	NA	NA
Bc_ctg1_4845	B2	0	Bcer	NA	Bcer	FALSE	NA	NA	NA
Bc_ctg1_4847	B2	0	Bcer	S	Bcer	FALSE	NA	NA	NA
Bc_ctg1_4848	B2	9.790631004	Bcer	L	Bcer	FALSE	NA	NA	NA
Bc_ctg1_4849	B2	1.282106441	Bcer	S	Bcer	FALSE	NA	NA	NA
Bc_ctg1_485	B2	7.692638646	Bcer	H	Bcer	FALSE	Bc_Ga0417192_01_rn06	Ancillary biosynthesis	None
Bc_ctg1_4850	B2	10.1402964	Bcer	P	Bcer	FALSE	NA	NA	NA
Bc_ctg1_4851	B2	4.312539847	Bcer	D	Bcer	FALSE	NA	NA	NA
Bc_ctg1_4852	B2	43.82472925	Bcer	P	Bcer	FALSE	NA	NA	NA

Bc_ctg1_4853	B2	65.27087336	Bcer	G	Bcer	FALSE	NA	NA	NA
Bc_ctg1_4855	B2	1.398661572	Bcer	M	Bcer	FALSE	NA	NA	NA
Bc_ctg1_4856	B2	0.349665393	Bcer	K	Bcer	FALSE	NA	NA	NA
Bc_ctg1_4857	B2	0.349665393	Bcer	NA	Bcer	FALSE	NA	NA	NA
Bc_ctg1_4858	B2	51.16770251	Bcer	M	Bcer	FALSE	NA	NA	NA
Bc_ctg1_4859	B2	1.282106441	Bcer	M	Bcer	FALSE	NA	NA	NA
Bc_ctg1_486	B2	4.66220524	Bcer	P	Bcer	FALSE	Bc_Ga0417192_01_rn06	Other	NA
Bc_ctg1_4860	B2	0.233110262	Bcer	V	Bcer	FALSE	NA	NA	NA
Bc_ctg1_4861	B2	0.116555131	Bcer	J	Bcer	FALSE	NA	NA	NA
Bc_ctg1_4862	B2	5.011870633	Bcer	S	Bcer	FALSE	Bc_Ga0417192_01_rn34	Other	NA
Bc_ctg1_4863	B2	0	Bcer	NA	Bcer	FALSE	Bc_Ga0417192_01_rn34	Other	NA
Bc_ctg1_4864	B2	4.66220524	Bcer	V	Bcer	FALSE	Bc_Ga0417192_01_rn34	Transport	SMCOG1000
Bc_ctg1_4865	B2	17.48326965	Bcer	V	Bcer	FALSE	Bc_Ga0417192_01_rn34	Transport	SMCOG1065
Bc_ctg1_4866	B2	11.53895797	Bcer	K	Bcer	FALSE	Bc_Ga0417192_01_rn34	Regulation	SMCOG1057
Bc_ctg1_4869	B2	0	Bcer	NA	Bcer	FALSE	Bc_Ga0417192_01_rn34	Other	NA
Bc_ctg1_487	B2	53.96502565	Bcer	H	Bcer	FALSE	Bc_Ga0417192_01_rn06	Ancillary biosynthesis	None
Bc_ctg1_4870	B2	9.674075873	Bcer	S	Bcer	FALSE	Bc_Ga0417192_01_rn34	Other	NA
Bc_ctg1_4871	B2	12.23828875	Bcer	I	Bcer	FALSE	Bc_Ga0417192_01_rn34	Ancillary biosynthesis	SMCOG1006
Bc_ctg1_4872	B2	11.07273744	Bcer	I	Bcer	FALSE	Bc_Ga0417192_01_rn34	Ancillary biosynthesis	SMCOG1090
Bc_ctg1_4873	B2	0	Bcer	I	Bcer	FALSE	Bc_Ga0417192_01_rn34	Other	NA
Bc_ctg1_4874	B2	6.99330786	Bcer	E	Bcer	FALSE	Bc_Ga0417192_01_rn34	Core biosynthesis	SMCOG1271
Bc_ctg1_4875	B2	5.128425764	Bcer	I	Bcer	FALSE	Bc_Ga0417192_01_rn34	Ancillary biosynthesis	SMCOG1023
Bc_ctg1_4876	B2	13.28728493	Bcer	I	Bcer	FALSE	Bc_Ga0417192_01_rn34	Ancillary biosynthesis	SMCOG1059
Bc_ctg1_4877	B2	17.36671452	Bcer	I	Bcer	FALSE	Bc_Ga0417192_01_rn34	Core biosynthesis	SMCOG1002
Bc_ctg1_4878	B2	0.116555131	Bcer	NA	Bcer	FALSE	Bc_Ga0417192_01_rn34	Other	NA
Bc_ctg1_4879	B2	0.116555131	Bcer	NA	Bcer	FALSE	Bc_Ga0417192_01_rn34	Other	NA
Bc_ctg1_488	B2	38.23008297	Bcer	C	Bcer	FALSE	Bc_Ga0417192_01_rn06	Other	NA
Bc_ctg1_4880	B2	0.815885917	Bcer	J	Bcer	FALSE	Bc_Ga0417192_01_rn34	Ancillary biosynthesis	SMCOG1111
Bc_ctg1_4881	B2	0.116555131	Bcer	NA	Bcer	FALSE	Bc_Ga0417192_01_rn34	Other	NA
Bc_ctg1_4884	B2	7.576083515	Bcer	M	Bcer	FALSE	Bc_Ga0417192_01_rn34	Other	NA
Bc_ctg1_4885	B2	17.36671452	Bcer	T	Bcer	FALSE	Bc_Ga0417192_01_rn34	Regulation	SMCOG1003
Bc_ctg1_4886	B2	5.711201419	Bcer	K	Bcer	FALSE	Bc_Ga0417192_01_rn34	Regulation	SMCOG1008
Bc_ctg1_4887	B2	1.515216703	Bcer	H	Bcer	FALSE	Bc_Ga0417192_01_rn34	Other	NA
Bc_ctg1_4888	B2	1.515216703	Bcer	H	Bcer	FALSE	Bc_Ga0417192_01_rn34	Other	NA
Bc_ctg1_4889	B2	5.478091157	Bcer	S	Bcer	FALSE	Bc_Ga0417192_01_rn34	Other	NA
Bc_ctg1_489	B2	10.48996179	Bcer	S	Bcer	FALSE	Bc_Ga0417192_01_rn06	Other	NA
Bc_ctg1_4890	B2	15.38527729	Bcer	F	Bcer	FALSE	Bc_Ga0417192_01_rn34	Other	NA
Bc_ctg1_4891	B2	25.99179421	Bcer	K	Bcer	FALSE	Bc_Ga0417192_01_rn34	Other	NA
Bc_ctg1_4892	B2	27.27390065	Bcer	NA	Bcer	FALSE	NA	NA	NA
Bc_ctg1_4893	B2	20.16403766	Bcer	E	Bcer	FALSE	NA	NA	NA
Bc_ctg1_4894	B2	0.699330786	Bcer	K	Bcer	FALSE	NA	NA	NA
Bc_ctg1_4895	B2	0.466220524	Bcer	M	Bcer	FALSE	NA	NA	NA
Bc_ctg1_4896	B2	0	Bcer	M	Bcer	FALSE	NA	NA	NA
Bc_ctg1_4897	B2	0.699330786	Bcer	M	Bcer	FALSE	NA	NA	NA
Bc_ctg1_4898	B2	7.342973253	Bcer	S	Bcer	FALSE	NA	NA	NA
Bc_ctg1_4899	B2	8.508524563	Bcer	S	Bcer	FALSE	NA	NA	NA
Bc_ctg1_49	B2	0.699330786	Bcer	S	Bcer	FALSE	NA	NA	NA
Bc_ctg1_490	B2	13.87006059	Bcer	S	Bcer	FALSE	NA	NA	NA
Bc_ctg1_4900	B2	99.07186135	Bcer	L	Bcer	FALSE	NA	NA	NA
Bc_ctg1_4901	B2	32.05266102	Bcer	K	Bcer	FALSE	NA	NA	NA
Bc_ctg1_4902	B2	1.048996179	Bcer	IQ	Bcer	TRUE	NA	NA	NA
Bc_ctg1_4903	B2	0.466220524	Bcer	K	Bcer	FALSE	NA	NA	NA
Bc_ctg1_4904	B2	9.32441048	Bcer	Q	Bcer	TRUE	NA	NA	NA
Bc_ctg1_4906	B2	2.214547489	Bcer	E	Bcer	FALSE	NA	NA	NA
Bc_ctg1_4907	B2	2.680768013	Bcer	M	Bcer	FALSE	NA	NA	NA
Bc_ctg1_4908	B2	3.846319323	Bcer	S	Bcer	FALSE	NA	NA	NA
Bc_ctg1_4909	B2	2.797323144	Bcer	K	Bcer	FALSE	Bc_Ga0417192_01_rn35	Other	NA
Bc_ctg1_491	B2	227.2825054	Bcer	C	Bcer	FALSE	NA	NA	NA
Bc_ctg1_4910	B2	42.30951255	Bcer	E	Bcer	FALSE	Bc_Ga0417192_01_rn35	Other	NA
Bc_ctg1_4911	B2	0.233110262	Bcer	S	Bcer	FALSE	Bc_Ga0417192_01_rn35	Other	NA
Bc_ctg1_4912	B2	5.944311681	Bcer	K	Bcer	FALSE	Bc_Ga0417192_01_rn35	Ancillary biosynthesis	SMCOG1019
Bc_ctg1_4913	B2	0.699330786	Bcer	NA	Bcer	FALSE	Bc_Ga0417192_01_rn35	Other	NA

Bc_ctg1_4914	B2	1.631771834	Bcer	Q	Bcer	TRUE	Bc_Ga0417192_01_rn35	Other	NA
Bc_ctg1_4915	B2	0	Bcer	S	Bcer	FALSE	Bc_Ga0417192_01_rn35	Other	NA
Bc_ctg1_4916	B2	0.349665393	Bcer	NA	Bcer	FALSE	Bc_Ga0417192_01_rn35	Other	NA
Bc_ctg1_4917	B2	3.49665393	Bcer	E	Bcer	FALSE	Bc_Ga0417192_01_rn35	Ancillary biosynthesis	SMCOG1040
Bc_ctg1_4918	B2	5.478091157	Bcer	GM	Bcer	FALSE	Bc_Ga0417192_01_rn35	Core biosynthesis	SMCOG1010
Bc_ctg1_4919	B2	0.932441048	Bcer	S	Bcer	FALSE	Bc_Ga0417192_01_rn35	Other	NA
Bc_ctg1_492	B2	0	Bcer	EG	Bcer	FALSE	NA	NA	NA
Bc_ctg1_4920	B2	3.030433406	Bcer	Q	Bcer	TRUE	Bc_Ga0417192_01_rn35	Ancillary biosynthesis	SMCOG1248
Bc_ctg1_4921	B2	22.61169541	Bcer	S	Bcer	FALSE	Bc_Ga0417192_01_rn35	Other	NA
Bc_ctg1_4922	B2	7.226418122	Bcer	Q	Bcer	TRUE	Bc_Ga0417192_01_rn35	Other	NA
Bc_ctg1_4923	B2	4.895315502	Bcer	G	Bcer	FALSE	Bc_Ga0417192_01_rn35	Other	NA
Bc_ctg1_4924	B2	0	Bcer	NA	Bcer	FALSE	Bc_Ga0417192_01_rn35	Other	NA
Bc_ctg1_4925	B2	4.66220524	Bcer	J	Bcer	FALSE	Bc_Ga0417192_01_rn35	Other	NA
Bc_ctg1_4926	B2	0	Bcer	M	Bcer	FALSE	Bc_Ga0417192_01_rn35	Other	NA
Bc_ctg1_4927	B2	12.35484389	Bcer	C	Bcer	FALSE	Bc_Ga0417192_01_rn35	Other	NA
Bc_ctg1_4928	B2	7.692638646	Bcer	T	Bcer	FALSE	Bc_Ga0417192_01_rn35	Regulation	SMCOG1008
Bc_ctg1_4929	B2	21.21303384	Bcer	T	Bcer	FALSE	Bc_Ga0417192_01_rn35	Regulation	SMCOG1003
Bc_ctg1_493	B2	0.233110262	Bcer	E	Bcer	FALSE	NA	NA	NA
Bc_ctg1_4930	B2	0	Bcer	NA	Bcer	FALSE	Bc_Ga0417192_01_rn35	Other	NA
Bc_ctg1_4931	B2	2.797323144	Bcer	NA	Bcer	FALSE	NA	NA	NA
Bc_ctg1_4932	B2	25.05935316	Bcer	S	Bcer	FALSE	NA	NA	NA
Bc_ctg1_4933	B2	9.674075873	Bcer	J	Bcer	FALSE	NA	NA	NA
Bc_ctg1_4934	B2	0	Bcer	NA	Bcer	FALSE	NA	NA	NA
Bc_ctg1_4935	B2	1.631771834	Bcer	V	Bcer	FALSE	NA	NA	NA
Bc_ctg1_4936	B2	6.527087336	Bcer	V	Bcer	FALSE	NA	NA	NA
Bc_ctg1_4937	B2	1.398661572	Bcer	S	Bcer	FALSE	NA	NA	NA
Bc_ctg1_4938	B2	229.1473875	Bcer	NA	Bcer	FALSE	NA	NA	NA
Bc_ctg1_4939	B2	0.466220524	Bcer	D	Bcer	FALSE	NA	NA	NA
Bc_ctg1_494	B2	0.233110262	Bcer	S	Bcer	FALSE	NA	NA	NA
Bc_ctg1_4940	B2	2.913878275	Bcer	E	Bcer	FALSE	NA	NA	NA
Bc_ctg1_4941	B2	15.73494268	Bcer	K	Bcer	FALSE	Bc_Ga0417192_01_rn36	Ancillary biosynthesis	SMCOG1019
Bc_ctg1_4942	B2	10.95618231	Bcer	F	Bcer	FALSE	Bc_Ga0417192_01_rn36	Ancillary biosynthesis	None
Bc_ctg1_4943	B2	1.515216703	Bcer	K	Bcer	FALSE	Bc_Ga0417192_01_rn36	Other	NA
Bc_ctg1_4944	B2	97.78975491	Bcer	S	Bcer	FALSE	Bc_Ga0417192_01_rn36	Other	NA
Bc_ctg1_4945	B2	1.048996179	Bcer	NA	Bcer	FALSE	Bc_Ga0417192_01_rn36	Core biosynthesis	None
Bc_ctg1_4946	B2	6.643642467	Bcer	NA	Bcer	FALSE	Bc_Ga0417192_01_rn36	Other	NA
Bc_ctg1_4947	B2	0.349665393	Bcer	S	Bcer	FALSE	Bc_Ga0417192_01_rn36	Other	NA
Bc_ctg1_4948	B2	15.96805295	Bcer	L	Bcer	FALSE	Bc_Ga0417192_01_rn36	Other	SMCOG1077
Bc_ctg1_4949	B2	1.864882096	Bcer	NA	Bcer	FALSE	Bc_Ga0417192_01_rn36	Other	NA
Bc_ctg1_495	B2	141.2648188	Bcer	E	Bcer	FALSE	NA	NA	NA
Bc_ctg1_4950	B2	3.030433406	Bcer	NA	Bcer	FALSE	NA	NA	NA
Bc_ctg1_4951	B2	0.116555131	Bcer	NA	Bcer	FALSE	NA	NA	NA
Bc_ctg1_4953	B2	12.23828875	Bcer	K	Bcer	FALSE	NA	NA	NA
Bc_ctg1_4954	B2	0	Bcer	K	Bcer	FALSE	NA	NA	NA
Bc_ctg1_4955	B2	0.116555131	Bcer	NA	Bcer	FALSE	NA	NA	NA
Bc_ctg1_4956	B2	0	Bcer	NA	Bcer	FALSE	NA	NA	NA
Bc_ctg1_4957	B2	1.748326965	Bcer	L	Bcer	FALSE	NA	NA	NA
Bc_ctg1_4958	B2	0	Bcer	K	Bcer	FALSE	NA	NA	NA
Bc_ctg1_4959	B2	0.349665393	Bcer	NA	Bcer	FALSE	NA	NA	NA
Bc_ctg1_4961	B2	0.116555131	Bcer	NA	Bcer	FALSE	NA	NA	NA
Bc_ctg1_4962	B2	0.815885917	Bcer	S	Bcer	FALSE	NA	NA	NA
Bc_ctg1_4963	B2	5.711201419	Bcer	NA	Bcer	FALSE	NA	NA	NA
Bc_ctg1_4964	B2	0	Bcer	NA	Bcer	FALSE	NA	NA	NA
Bc_ctg1_4965	B2	4.079429585	Bcer	K	Bcer	FALSE	NA	NA	NA
Bc_ctg1_4966	B2	3.380098799	Bcer	NA	Bcer	FALSE	NA	NA	NA
Bc_ctg1_4967	B2	0.233110262	Bcer	NA	Bcer	FALSE	NA	NA	NA
Bc_ctg1_497	B2	5.361536026	Bcer	L	Bcer	FALSE	NA	NA	NA
Bc_ctg1_4970	B2	2.447657751	Bcer	S	Bcer	FALSE	NA	NA	NA
Bc_ctg1_4971	B2	4.66220524	Bcer	K	Bcer	FALSE	NA	NA	NA
Bc_ctg1_4972	B2	272.0396757	Bcer	L	Bcer	FALSE	NA	NA	NA
Bc_ctg1_4973	B2	80.073375	Bcer	NA	Bcer	FALSE	NA	NA	NA
Bc_ctg1_4974	B2	0.116555131	Bcer	NA	Bcer	FALSE	NA	NA	NA

Bc_ctg1_4976	B2	0.116555131	Bcer	NA	Bcer	FALSE	NA	NA	NA
Bc_ctg1_4977	B2	0	Bcer	S	Bcer	FALSE	NA	NA	NA
Bc_ctg1_4978	B2	0.932441048	Bcer	S	Bcer	FALSE	NA	NA	NA
Bc_ctg1_4979	B2	0.349665393	Bcer	S	Bcer	FALSE	NA	NA	NA
Bc_ctg1_498	B2	1.282106441	Bcer	S	Bcer	FALSE	NA	NA	NA
Bc_ctg1_4980	B2	0	Bcer	OU	Bcer	FALSE	NA	NA	NA
Bc_ctg1_4981	B2	0.699330786	Bcer	S	Bcer	FALSE	NA	NA	NA
Bc_ctg1_4982	B2	0.116555131	Bcer	NA	Bcer	FALSE	NA	NA	NA
Bc_ctg1_4983	B2	0	Bcer	NA	Bcer	FALSE	NA	NA	NA
Bc_ctg1_4984	B2	0	Bcer	NA	Bcer	FALSE	NA	NA	NA
Bc_ctg1_4985	B2	0	Bcer	NA	Bcer	FALSE	NA	NA	NA
Bc_ctg1_4986	B2	0.116555131	Bcer	NA	Bcer	FALSE	NA	NA	NA
Bc_ctg1_4987	B2	0	Bcer	NA	Bcer	FALSE	NA	NA	NA
Bc_ctg1_4988	B2	0	Bcer	E	Bcer	FALSE	NA	NA	NA
Bc_ctg1_4989	B2	0.116555131	Bcer	S	Bcer	FALSE	NA	NA	NA
Bc_ctg1_499	B2	4.778760371	Bcer	E	Bcer	FALSE	NA	NA	NA
Bc_ctg1_4990	B2	0.349665393	Bcer	M	Bcer	FALSE	NA	NA	NA
Bc_ctg1_4993	B2	0	Bcer	S	Bcer	FALSE	NA	NA	NA
Bc_ctg1_4994	B2	0.466220524	Bcer	Q	Bcer	TRUE	NA	NA	NA
Bc_ctg1_4995	B2	3.729764192	Bcer	NA	Bcer	FALSE	NA	NA	NA
Bc_ctg1_4996	B2	53.26569487	Bcer	T	Bcer	FALSE	NA	NA	NA
Bc_ctg1_4997	B2	1.981437227	Bcer	S	Bcer	FALSE	NA	NA	NA
Bc_ctg1_4998	B2	37.53075218	Bcer	S	Bcer	FALSE	NA	NA	NA
Bc_ctg1_4999	B2	5.82775655	Bcer	S	Bcer	FALSE	NA	NA	NA
Bc_ctg1_50	B2	0.699330786	Bcer	S	Bcer	FALSE	NA	NA	NA
Bc_ctg1_5000	B2	3.030433406	Bcer	NA	Bcer	FALSE	NA	NA	NA
Bc_ctg1_5001	B2	0.116555131	Bcer	NA	Bcer	FALSE	NA	NA	NA
Bc_ctg1_5002	B2	11.30584771	Bcer	S	Bcer	FALSE	NA	NA	NA
Bc_ctg1_5003	B2	0.466220524	Bcer	NA	Bcer	FALSE	NA	NA	NA
Bc_ctg1_5004	B2	307.5889907	Bcer	C	Bcer	FALSE	NA	NA	NA
Bc_ctg1_5005	B2	21.56269923	Bcer	EGP	Bcer	FALSE	NA	NA	NA
Bc_ctg1_5006	B2	9.091300218	Bcer	S	Bcer	FALSE	NA	NA	NA
Bc_ctg1_5007	B2	0	Bcer	S	Bcer	FALSE	NA	NA	NA
Bc_ctg1_5008	B2	3.962874454	Bcer	K	Bcer	FALSE	NA	NA	NA
Bc_ctg1_5009	B2	5.128425764	Bcer	Q	Bcer	TRUE	NA	NA	NA
Bc_ctg1_501	B2	0	Bcer	NA	Bcer	FALSE	NA	NA	NA
Bc_ctg1_5010	B2	0.466220524	Bcer	C	Bcer	FALSE	NA	NA	NA
Bc_ctg1_5011	B2	38.46319323	Bcer	S	Bcer	FALSE	NA	NA	NA
Bc_ctg1_5012	B2	40.44463046	Bcer	T	Bcer	FALSE	NA	NA	NA
Bc_ctg1_5013	B2	100.8201883	Bcer	M	Bcer	FALSE	NA	NA	NA
Bc_ctg1_5014	B2	3.146988537	Bcer	U	Bcer	FALSE	NA	NA	NA
Bc_ctg1_5015	B2	2.447657751	Bcer	CG	Bcer	FALSE	NA	NA	NA
Bc_ctg1_5016	B2	1.398661572	Bcer	M	Bcer	FALSE	NA	NA	NA
Bc_ctg1_5017	B2	1.398661572	Bcer	S	Bcer	FALSE	NA	NA	NA
Bc_ctg1_5018	B2	3.263543668	Bcer	P	Bcer	FALSE	NA	NA	NA
Bc_ctg1_5019	B2	1.16555131	Bcer	P	Bcer	FALSE	NA	NA	NA
Bc_ctg1_502	B2	0.116555131	Bcer	Q	Bcer	TRUE	NA	NA	NA
Bc_ctg1_5021	B2	37.88041757	Bcer	T	Bcer	FALSE	NA	NA	NA
Bc_ctg1_5023	B2	3.380098799	Bcer	NA	Bcer	FALSE	NA	NA	NA
Bc_ctg1_5024	B2	0	Bcer	NA	Bcer	FALSE	NA	NA	NA
Bc_ctg1_5025	B2	0.932441048	Bcer	NA	Bcer	FALSE	NA	NA	NA
Bc_ctg1_5026	B2	0	Bcer	NA	Bcer	FALSE	NA	NA	NA
Bc_ctg1_5028	B2	0	Bcer	F	Bcer	FALSE	NA	NA	NA
Bc_ctg1_503	B2	3.846319323	Bcer	E	Bcer	FALSE	NA	NA	NA
Bc_ctg1_5033	B2	1.282106441	Bcer	D	Bcer	FALSE	NA	NA	NA
Bc_ctg1_5034	B2	0	Bcer	NA	Bcer	FALSE	NA	NA	NA
Bc_ctg1_5035	B2	0.349665393	Bcer	NA	Bcer	FALSE	NA	NA	NA
Bc_ctg1_5036	B2	13.28728493	Bcer	F	Bcer	FALSE	NA	NA	NA
Bc_ctg1_5037	B2	0.349665393	Bcer	NA	Bcer	FALSE	NA	NA	NA
Bc_ctg1_5038	B2	10.37340666	Bcer	KLT	Bcer	FALSE	NA	NA	NA
Bc_ctg1_5039	B2	0.815885917	Bcer	S	Bcer	FALSE	NA	NA	NA
Bc_ctg1_504	B2	0.116555131	Bcer	L	Bcer	FALSE	NA	NA	NA

Bc_ctg1_5040	B2	8.974745087	Bcer	C	Bcer	FALSE	NA	NA	NA
Bc_ctg1_5041	B2	115.7392451	Bcer	I	Bcer	FALSE	NA	NA	NA
Bc_ctg1_5042	B2	14.45283624	Bcer	M	Bcer	FALSE	NA	NA	NA
Bc_ctg1_5043	B2	3.729764192	Bcer	EGP	Bcer	FALSE	NA	NA	NA
Bc_ctg1_5044	B2	6.527087336	Bcer	V	Bcer	FALSE	NA	NA	NA
Bc_ctg1_5045	B2	3.030433406	Bcer	V	Bcer	FALSE	NA	NA	NA
Bc_ctg1_5046	B2	1.048996179	Bcer	S	Bcer	FALSE	NA	NA	NA
Bc_ctg1_5047	B2	1.048996179	Bcer	S	Bcer	FALSE	NA	NA	NA
Bc_ctg1_5048	B2	55.01402183	Bcer	S	Bcer	FALSE	NA	NA	NA
Bc_ctg1_5049	B2	6.643642467	Bcer	K	Bcer	FALSE	NA	NA	NA
Bc_ctg1_505	B2	0	Bcer	L	Bcer	FALSE	NA	NA	NA
Bc_ctg1_5050	B2	8.508524563	Bcer	NA	Bcer	FALSE	NA	NA	NA
Bc_ctg1_5051	B2	71.21518504	Bcer	S	Bcer	FALSE	NA	NA	NA
Bc_ctg1_5052	B2	34.26720851	Bcer	KQ	Bcer	TRUE	NA	NA	NA
Bc_ctg1_5053	B2	1.515216703	Bcer	EGP	Bcer	FALSE	NA	NA	NA
Bc_ctg1_5054	B2	2.33110262	Bcer	K	Bcer	FALSE	NA	NA	NA
Bc_ctg1_5055	B2	1.864882096	Bcer	M	Bcer	FALSE	NA	NA	NA
Bc_ctg1_5056	B2	9.674075873	Bcer	NA	Bcer	FALSE	NA	NA	NA
Bc_ctg1_5058	B2	4.195984716	Bcer	K	Bcer	FALSE	NA	NA	NA
Bc_ctg1_5059	B2	11.42240284	Bcer	C	Bcer	FALSE	NA	NA	NA
Bc_ctg1_506	B2	6.99330786	Bcer	E	Bcer	FALSE	NA	NA	NA
Bc_ctg1_5060	B2	4.66220524	Bcer	S	Bcer	FALSE	NA	NA	NA
Bc_ctg1_5061	B2	0	Bcer	NA	Bcer	FALSE	NA	NA	NA
Bc_ctg1_5062	B2	9.674075873	Bcer	FG	Bcer	FALSE	NA	NA	NA
Bc_ctg1_5063	B2	6.99330786	Bcer	K	Bcer	FALSE	NA	NA	NA
Bc_ctg1_5064	B2	0	Bcer	EG	Bcer	FALSE	Bc_Ga0417192_01_rn37	Other	NA
Bc_ctg1_5065	B2	2.214547489	Bcer	G	Bcer	FALSE	Bc_Ga0417192_01_rn37	Other	NA
Bc_ctg1_5066	B2	0.815885917	Bcer	F	Bcer	FALSE	Bc_Ga0417192_01_rn37	Other	NA
Bc_ctg1_5067	B2	8.974745087	Bcer	K	Bcer	FALSE	Bc_Ga0417192_01_rn37	Other	NA
Bc_ctg1_5068	B2	1.631771834	Bcer	S	Bcer	FALSE	Bc_Ga0417192_01_rn37	Other	NA
Bc_ctg1_5069	B2	9.790631004	Bcer	F	Bcer	FALSE	Bc_Ga0417192_01_rn37	Other	NA
Bc_ctg1_507	B2	1442.253191	Bcer	E	Bcer	FALSE	NA	NA	NA
Bc_ctg1_5070	B2	0	Bcer	NA	Bcer	FALSE	Bc_Ga0417192_01_rn37	Core biosynthesis	None
Bc_ctg1_5071	B2	1.864882096	Bcer	V	Bcer	FALSE	Bc_Ga0417192_01_rn37	Other	NA
Bc_ctg1_5072	B2	5.128425764	Bcer	V	Bcer	FALSE	Bc_Ga0417192_01_rn37	Other	NA
Bc_ctg1_5073	B2	1.981437227	Bcer	V	Bcer	FALSE	Bc_Ga0417192_01_rn37	Other	NA
Bc_ctg1_5075	B2	3.846319323	Bcer	P	Bcer	FALSE	Bc_Ga0417192_01_rn37	Transport	SMCOG1137
Bc_ctg1_5076	B2	0.466220524	Bcer	P	Bcer	FALSE	Bc_Ga0417192_01_rn37	Other	NA
Bc_ctg1_5077	B2	1.515216703	Bcer	K	Bcer	FALSE	Bc_Ga0417192_01_rn37	Other	NA
Bc_ctg1_5078	B2	0.116555131	Bcer	F	Bcer	FALSE	Bc_Ga0417192_01_rn37	Other	NA
Bc_ctg1_5079	B2	11.07273744	Bcer	L	Bcer	FALSE	Bc_Ga0417192_01_rn37	Other	NA
Bc_ctg1_508	B2	3.613209061	Bcer	K	Bcer	FALSE	NA	NA	NA
Bc_ctg1_5080	B2	9.440965611	Bcer	M	Bcer	FALSE	NA	NA	NA
Bc_ctg1_5081	B2	12.82106441	Bcer	S	Bcer	FALSE	NA	NA	NA
Bc_ctg1_5082	B2	20.97992358	Bcer	S	Bcer	FALSE	NA	NA	NA
Bc_ctg1_5083	B2	0.466220524	Bcer	L	Bcer	FALSE	NA	NA	NA
Bc_ctg1_5084	B2	6.760197598	Bcer	J	Bcer	FALSE	NA	NA	NA
Bc_ctg1_5085	B2	32.16921616	Bcer	S	Bcer	FALSE	NA	NA	NA
Bc_ctg1_5086	B2	16.20116321	Bcer	P	Bcer	FALSE	NA	NA	NA
Bc_ctg1_5087	B2	4.545650109	Bcer	NA	Bcer	FALSE	NA	NA	NA
Bc_ctg1_5088	B2	2.913878275	Bcer	S	Bcer	FALSE	NA	NA	NA
Bc_ctg1_5089	B2	5.944311681	Bcer	S	Bcer	FALSE	NA	NA	NA
Bc_ctg1_509	B2	3.49665393	Bcer	NA	Bcer	FALSE	NA	NA	NA
Bc_ctg1_5090	B2	33.56787773	Bcer	S	Bcer	FALSE	NA	NA	NA
Bc_ctg1_5091	B2	7.226418122	Bcer	K	Bcer	FALSE	NA	NA	NA
Bc_ctg1_5092	B2	11.88862336	Bcer	S	Bcer	FALSE	NA	NA	NA
Bc_ctg1_5093	B2	14.56939137	Bcer	J	Bcer	FALSE	NA	NA	NA
Bc_ctg1_5094	B2	18.76537609	Bcer	CH	Bcer	FALSE	NA	NA	NA
Bc_ctg1_5095	B2	0.116555131	Bcer	V	Bcer	FALSE	NA	NA	NA
Bc_ctg1_5096	B2	0	Bcer	NA	Bcer	FALSE	NA	NA	NA
Bc_ctg1_5097	B2	60.14244759	Bcer	L	Bcer	FALSE	NA	NA	NA
Bc_ctg1_5098	B2	7.925748908	Bcer	K	Bcer	FALSE	NA	NA	NA

Bc_ctg1_5099	B2	0.699330786	Bcer	NA	Bcer	FALSE	NA	NA	NA
Bc_ctg1_51	B2	0.582775655	Bcer	S	Bcer	FALSE	NA	NA	NA
Bc_ctg1_510	B2	0	Bcer	K	Bcer	FALSE	NA	NA	NA
Bc_ctg1_5100	B2	1.16555131	Bcer	G	Bcer	FALSE	NA	NA	NA
Bc_ctg1_5101	B2	7.809193777	Bcer	NA	Bcer	FALSE	NA	NA	NA
Bc_ctg1_5102	B2	0.349665393	Bcer	NA	Bcer	FALSE	NA	NA	NA
Bc_ctg1_5103	B2	6.060866812	Bcer	V	Bcer	FALSE	Bc_Ga0417192_01_rn38	Other	NA
Bc_ctg1_5104	B2	2.214547489	Bcer	CG	Bcer	FALSE	Bc_Ga0417192_01_rn38	Ancillary biosynthesis	SMCOG1062
Bc_ctg1_5105	B2	6.293977074	Bcer	KLT	Bcer	FALSE	Bc_Ga0417192_01_rn38	Other	NA
Bc_ctg1_5106	B2	3.030433406	Bcer	L	Bcer	FALSE	Bc_Ga0417192_01_rn38	Other	NA
Bc_ctg1_5107	B2	38.69630349	Bcer	E	Bcer	FALSE	Bc_Ga0417192_01_rn38	Ancillary biosynthesis	SMCOG1103
Bc_ctg1_5108	B2	8.974745087	Bcer	S	Bcer	FALSE	Bc_Ga0417192_01_rn38	Other	NA
Bc_ctg1_5109	B2	0.233110262	Bcer	NA	Bcer	FALSE	Bc_Ga0417192_01_rn38	Other	NA
Bc_ctg1_511	B2	31.70299563	Bcer	S	Bcer	FALSE	NA	NA	NA
Bc_ctg1_5110	B2	5.594646288	Bcer	Q	Bcer	TRUE	Bc_Ga0417192_01_rn38	Other	NA
Bc_ctg1_5111	B2	4.545650109	Bcer	S	Bcer	FALSE	Bc_Ga0417192_01_rn38	Other	NA
Bc_ctg1_5112	B2	2.797323144	Bcer	K	Bcer	FALSE	Bc_Ga0417192_01_rn38	Other	NA
Bc_ctg1_5113	B2	7.692638646	Bcer	S	Bcer	FALSE	Bc_Ga0417192_01_rn38	Ancillary biosynthesis	SMCOG1115
Bc_ctg1_5114	B2	2.447657751	Bcer	T	Bcer	FALSE	Bc_Ga0417192_01_rn38	Regulation	SMCOG1008
Bc_ctg1_5115	B2	11.42240284	Bcer	T	Bcer	FALSE	Bc_Ga0417192_01_rn38	Regulation	SMCOG1003
Bc_ctg1_5116	B2	0.116555131	Bcer	S	Bcer	FALSE	Bc_Ga0417192_01_rn38	Other	NA
Bc_ctg1_5117	B2	0.349665393	Bcer	NA	Bcer	FALSE	Bc_Ga0417192_01_rn38	Other	NA
Bc_ctg1_5118	B2	3.962874454	Bcer	V	Bcer	FALSE	Bc_Ga0417192_01_rn38	Transport	SMCOG1000
Bc_ctg1_5119	B2	1.16555131	Bcer	V	Bcer	FALSE	Bc_Ga0417192_01_rn38	Other	NA
Bc_ctg1_512	B2	28.67256223	Bcer	V	Bcer	FALSE	NA	NA	NA
Bc_ctg1_5120	B2	5.944311681	Bcer	S	Bcer	FALSE	Bc_Ga0417192_01_rn38	Other	NA
Bc_ctg1_5121	B2	22.26203002	Bcer	I	Bcer	FALSE	Bc_Ga0417192_01_rn38	Ancillary biosynthesis	SMCOG1066
Bc_ctg1_5122	B2	5.011870633	Bcer	E	Bcer	FALSE	Bc_Ga0417192_01_rn38	Ancillary biosynthesis	SMCOG1180
Bc_ctg1_5123	B2	14.91905677	Bcer	Q	Bcer	TRUE	Bc_Ga0417192_01_rn38	Core biosynthesis	SMCOG1002
Bc_ctg1_5124	B2	0.699330786	Bcer	C	Bcer	FALSE	Bc_Ga0417192_01_rn38	Ancillary biosynthesis	None
Bc_ctg1_5125	B2	28.32289683	Bcer	G	Bcer	FALSE	Bc_Ga0417192_01_rn38	Core biosynthesis	SMCOG1297
Bc_ctg1_5126	B2	2487.636161	Bcer	E	Bcer	FALSE	Bc_Ga0417192_01_rn38	Other	NA
Bc_ctg1_5127	B2	37.64730731	Bcer	F	Bcer	FALSE	Bc_Ga0417192_01_rn38	Ancillary biosynthesis	SMCOG1283
Bc_ctg1_5128	B2	6.060866812	Bcer	S	Bcer	FALSE	Bc_Ga0417192_01_rn38	Other	NA
Bc_ctg1_5129	B2	1.631771834	Bcer	L	Bcer	FALSE	Bc_Ga0417192_01_rn38	Other	NA
Bc_ctg1_513	B2	13.28728493	Bcer	K	Bcer	FALSE	NA	NA	NA
Bc_ctg1_5130	B2	0	Bcer	E	Bcer	FALSE	Bc_Ga0417192_01_rn38	Other	NA
Bc_ctg1_5131	B2	202.4562625	Bcer	U	Bcer	FALSE	Bc_Ga0417192_01_rn38	Other	NA
Bc_ctg1_5132	B2	0.932441048	Bcer	EG	Bcer	FALSE	Bc_Ga0417192_01_rn38	Transport	SMCOG1166
Bc_ctg1_5133	B2	3.030433406	Bcer	K	Bcer	FALSE	Bc_Ga0417192_01_rn38	Ancillary biosynthesis	SMCOG1019
Bc_ctg1_5134	B2	5.594646288	Bcer	C	Bcer	FALSE	Bc_Ga0417192_01_rn38	Ancillary biosynthesis	SMCOG1028
Bc_ctg1_5135	B2	0.349665393	Bcer	I	Bcer	FALSE	Bc_Ga0417192_01_rn38	Ancillary biosynthesis	SMCOG1036
Bc_ctg1_5136	B2	0	Bcer	NA	Bcer	FALSE	Bc_Ga0417192_01_rn38	Other	NA
Bc_ctg1_5137	B2	0.116555131	Bcer	NU	Bcer	FALSE	Bc_Ga0417192_01_rn38	Other	NA
Bc_ctg1_5138	B2	4.079429585	Bcer	NA	Bcer	FALSE	Bc_Ga0417192_01_rn38	Other	NA
Bc_ctg1_5139	B2	6.293977074	Bcer	S	Bcer	FALSE	Bc_Ga0417192_01_rn38	Other	NA
Bc_ctg1_514	B2	5.478091157	Bcer	K	Bcer	FALSE	NA	NA	NA
Bc_ctg1_5140	B2	8.508524563	Bcer	S	Bcer	FALSE	NA	NA	NA
Bc_ctg1_5141	B2	14.33628111	Bcer	J	Bcer	FALSE	NA	NA	NA
Bc_ctg1_5142	B2	1.748326965	Bcer	NA	Bcer	FALSE	NA	NA	NA
Bc_ctg1_5143	B2	0.233110262	Bcer	S	Bcer	FALSE	NA	NA	NA
Bc_ctg1_5144	B2	0.699330786	Bcer	H	Bcer	FALSE	NA	NA	NA
Bc_ctg1_5145	B2	0	Bcer	M	Bcer	FALSE	NA	NA	NA
Bc_ctg1_5147	B2	5.944311681	Bcer	C	Bcer	FALSE	NA	NA	NA
Bc_ctg1_5148	B2	5.244980895	Bcer	V	Bcer	FALSE	NA	NA	NA
Bc_ctg1_5149	B2	39.27907915	Bcer	E	Bcer	FALSE	NA	NA	NA
Bc_ctg1_515	B2	2.447657751	Bcer	S	Bcer	FALSE	NA	NA	NA
Bc_ctg1_5150	B2	24.70968777	Bcer	S	Bcer	FALSE	NA	NA	NA
Bc_ctg1_5151	B2	46.6220524	Bcer	E	Bcer	FALSE	NA	NA	NA
Bc_ctg1_5152	B2	7.925748908	Bcer	K	Bcer	FALSE	NA	NA	NA
Bc_ctg1_5153	B2	0.466220524	Bcer	F	Bcer	FALSE	NA	NA	NA
Bc_ctg1_5154	B2	0.233110262	Bcer	Q	Bcer	TRUE	NA	NA	NA

Bc_ctg1_5155	B2	0.815885917	Bcer	Q	Bcer	TRUE	NA	NA	NA
Bc_ctg1_5156	B2	3.613209061	Bcer	G	Bcer	FALSE	NA	NA	NA
Bc_ctg1_5157	B2	0.233110262	Bcer	S	Bcer	FALSE	NA	NA	NA
Bc_ctg1_5158	B2	24.01035699	Bcer	KQ	Bcer	TRUE	NA	NA	NA
Bc_ctg1_5159	B2	0.233110262	Bcer	NA	Bcer	FALSE	NA	NA	NA
Bc_ctg1_516	B2	22.02891976	Bcer	S	Bcer	FALSE	NA	NA	NA
Bc_ctg1_5160	B2	0.582775655	Bcer	F	Bcer	FALSE	NA	NA	NA
Bc_ctg1_5161	B2	1.282106441	Bcer	K	Bcer	FALSE	NA	NA	NA
Bc_ctg1_5162	B2	5.82775655	Bcer	K	Bcer	FALSE	NA	NA	NA
Bc_ctg1_5163	B2	1.748326965	Bcer	NA	Bcer	FALSE	NA	NA	NA
Bc_ctg1_5164	B2	1.16555131	Bcer	Q	Bcer	TRUE	NA	NA	NA
Bc_ctg1_5165	B2	11.53895797	Bcer	K	Bcer	FALSE	NA	NA	NA
Bc_ctg1_5166	B2	5.711201419	Bcer	S	Bcer	FALSE	NA	NA	NA
Bc_ctg1_5167	B2	0.466220524	Bcer	NA	Bcer	FALSE	NA	NA	NA
Bc_ctg1_5168	B2	0.699330786	Bcer	C	Bcer	FALSE	NA	NA	NA
Bc_ctg1_5169	B2	0.116555131	Bcer	C	Bcer	FALSE	NA	NA	NA
Bc_ctg1_517	B2	372.1605333	Bcer	L	Bcer	FALSE	NA	NA	NA
Bc_ctg1_5170	B2	0.116555131	Bcer	C	Bcer	FALSE	NA	NA	NA
Bc_ctg1_5171	B2	0.233110262	Bcer	C	Bcer	FALSE	NA	NA	NA
Bc_ctg1_5173	B2	11.30584771	Bcer	S	Bcer	FALSE	NA	NA	NA
Bc_ctg1_5174	B2	5.944311681	Bcer	S	Bcer	FALSE	NA	NA	NA
Bc_ctg1_5175	B2	4.079429585	Bcer	S	Bcer	FALSE	NA	NA	NA
Bc_ctg1_5176	B2	0	Bcer	S	Bcer	FALSE	NA	NA	NA
Bc_ctg1_5177	B2	1.864882096	Bcer	L	Bcer	FALSE	NA	NA	NA
Bc_ctg1_5178	B2	1.282106441	Bcer	S	Bcer	FALSE	NA	NA	NA
Bc_ctg1_5179	B2	3.263543668	Bcer	T	Bcer	FALSE	NA	NA	NA
Bc_ctg1_518	B2	177.2803542	Bcer	L	Bcer	FALSE	NA	NA	NA
Bc_ctg1_5180	B2	2.33110262	Bcer	FG	Bcer	FALSE	NA	NA	NA
Bc_ctg1_5182	B2	0.466220524	Bcer	P	Bcer	FALSE	NA	NA	NA
Bc_ctg1_5183	B2	0.116555131	Bcer	E	Bcer	FALSE	NA	NA	NA
Bc_ctg1_5184	B2	0	Bcer	E	Bcer	FALSE	NA	NA	NA
Bc_ctg1_5185	B2	3.263543668	Bcer	OU	Bcer	FALSE	NA	NA	NA
Bc_ctg1_5186	B2	6.410532205	Bcer	K	Bcer	FALSE	NA	NA	NA
Bc_ctg1_5187	B2	0	Bcer	C	Bcer	FALSE	NA	NA	NA
Bc_ctg1_5188	B2	0	Bcer	G	Bcer	FALSE	NA	NA	NA
Bc_ctg1_5189	B2	15.73494268	Bcer	S	Bcer	FALSE	NA	NA	NA
Bc_ctg1_519	B2	50.11870633	Bcer	S	Bcer	FALSE	NA	NA	NA
Bc_ctg1_5190	B2	568.0897085	Bcer	S	Bcer	FALSE	NA	NA	NA
Bc_ctg1_5191	B2	12.35484389	Bcer	CG	Bcer	FALSE	NA	NA	NA
Bc_ctg1_5192	B2	1.864882096	Bcer	S	Bcer	FALSE	NA	NA	NA
Bc_ctg1_5193	B2	2.797323144	Bcer	F	Bcer	FALSE	NA	NA	NA
Bc_ctg1_5194	B2	5.594646288	Bcer	T	Bcer	FALSE	NA	NA	NA
Bc_ctg1_5195	B2	10.37340666	Bcer	Q	Bcer	TRUE	NA	NA	NA
Bc_ctg1_5196	B2	3.613209061	Bcer	S	Bcer	FALSE	NA	NA	NA
Bc_ctg1_5197	B2	4.545650109	Bcer	K	Bcer	FALSE	NA	NA	NA
Bc_ctg1_5198	B2	5.711201419	Bcer	K	Bcer	FALSE	NA	NA	NA
Bc_ctg1_5199	B2	3.030433406	Bcer	NA	Bcer	FALSE	NA	NA	NA
Bc_ctg1_520	B2	482.7713526	Bcer	O	Bcer	FALSE	NA	NA	NA
Bc_ctg1_5200	B2	9.207855349	Bcer	M	Bcer	FALSE	NA	NA	NA
Bc_ctg1_5201	B2	2.680768013	Bcer	C	Bcer	FALSE	NA	NA	NA
Bc_ctg1_5202	B2	0	Bcer	K	Bcer	FALSE	NA	NA	NA
Bc_ctg1_5203	B2	2.913878275	Bcer	S	Bcer	FALSE	NA	NA	NA
Bc_ctg1_5204	B2	3.49665393	Bcer	NA	Bcer	FALSE	NA	NA	NA
Bc_ctg1_5205	B2	26.10834934	Bcer	L	Bcer	FALSE	NA	NA	NA
Bc_ctg1_5206	B2	0	Bcer	S	Bcer	FALSE	NA	NA	NA
Bc_ctg1_5207	B2	12.58795415	Bcer	S	Bcer	FALSE	NA	NA	NA
Bc_ctg1_5208	B2	72.26418122	Bcer	L	Bcer	FALSE	NA	NA	NA
Bc_ctg1_521	B2	53.49880513	Bcer	T	Bcer	FALSE	NA	NA	NA
Bc_ctg1_5210	B2	0.233110262	Bcer	M	Bcer	FALSE	NA	NA	NA
Bc_ctg1_5211	B2	0.932441048	Bcer	NA	Bcer	FALSE	NA	NA	NA
Bc_ctg1_5212	B2	2.214547489	Bcer	NA	Bcer	FALSE	NA	NA	NA
Bc_ctg1_5213	B2	0	Bcer	NA	Bcer	FALSE	NA	NA	NA

Bc_ctg1_5214	B2	1.631771834	Bcer	S	Bcer	FALSE	NA	NA	NA
Bc_ctg1_5215	B2	0.233110262	Bcer	S	Bcer	FALSE	NA	NA	NA
Bc_ctg1_5216	B2	2.33110262	Bcer	E	Bcer	FALSE	NA	NA	NA
Bc_ctg1_5217	B2	406.7774072	Bcer	C	Bcer	FALSE	NA	NA	NA
Bc_ctg1_5218	B2	26.92423526	Bcer	S	Bcer	FALSE	NA	NA	NA
Bc_ctg1_5219	B2	16.90049399	Bcer	K	Bcer	FALSE	NA	NA	NA
Bc_ctg1_522	B2	2.447657751	Bcer	C	Bcer	FALSE	NA	NA	NA
Bc_ctg1_5220	B2	7.692638646	Bcer	H	Bcer	FALSE	NA	NA	NA
Bc_ctg1_5221	B2	0	Bcer	S	Bcer	FALSE	NA	NA	NA
Bc_ctg1_5222	B2	0.932441048	Bcer	E	Bcer	FALSE	NA	NA	NA
Bc_ctg1_5223	B2	1.981437227	Bcer	C	Bcer	FALSE	NA	NA	NA
Bc_ctg1_5224	B2	0.582775655	Bcer	E	Bcer	FALSE	NA	NA	NA
Bc_ctg1_5225	B2	0	Bcer	E	Bcer	FALSE	NA	NA	NA
Bc_ctg1_5226	B2	0.116555131	Bcer	E	Bcer	FALSE	NA	NA	NA
Bc_ctg1_5227	B2	0.116555131	Bcer	E	Bcer	FALSE	NA	NA	NA
Bc_ctg1_5228	B2	14.56939137	Bcer	KT	Bcer	FALSE	NA	NA	NA
Bc_ctg1_5229	B2	0.582775655	Bcer	C	Bcer	FALSE	NA	NA	NA
Bc_ctg1_523	B2	57.81134497	Bcer	F	Bcer	FALSE	NA	NA	NA
Bc_ctg1_5230	B2	1.398661572	Bcer	C	Bcer	FALSE	NA	NA	NA
Bc_ctg1_5231	B2	9.907186135	Bcer	C	Bcer	FALSE	NA	NA	NA
Bc_ctg1_5232	B2	16.31771834	Bcer	S	Bcer	FALSE	NA	NA	NA
Bc_ctg1_5233	B2	39.27907915	Bcer	S	Bcer	FALSE	NA	NA	NA
Bc_ctg1_5234	B2	45.1068357	Bcer	NA	Bcer	FALSE	NA	NA	NA
Bc_ctg1_5236	B2	63.75565666	Bcer	S	Bcer	FALSE	NA	NA	NA
Bc_ctg1_5238	B2	18.4157107	Bcer	S	Bcer	FALSE	NA	NA	NA
Bc_ctg1_5239	B2	0	Bcer	NA	Bcer	FALSE	NA	NA	NA
Bc_ctg1_524	B2	87.76601364	Bcer	I	Bcer	FALSE	NA	NA	NA
Bc_ctg1_5240	B2	33.91754312	Bcer	M	Bcer	FALSE	NA	NA	NA
Bc_ctg1_5241	B2	16.78393886	Bcer	E	Bcer	FALSE	NA	NA	NA
Bc_ctg1_5242	B2	13.5203952	Bcer	M	Bcer	FALSE	NA	NA	NA
Bc_ctg1_5243	B2	4.895315502	Bcer	M	Bcer	FALSE	NA	NA	NA
Bc_ctg1_5244	B2	5.361536026	Bcer	S	Bcer	FALSE	NA	NA	NA
Bc_ctg1_5245	B2	0.233110262	Bcer	D	Bcer	FALSE	NA	NA	NA
Bc_ctg1_5246	B2	0.815885917	Bcer	C	Bcer	FALSE	NA	NA	NA
Bc_ctg1_5247	B2	7.576083515	Bcer	K	Bcer	FALSE	NA	NA	NA
Bc_ctg1_5248	B2	1.282106441	Bcer	S	Bcer	FALSE	NA	NA	NA
Bc_ctg1_5249	B2	0.582775655	Bcer	NA	Bcer	FALSE	NA	NA	NA
Bc_ctg1_525	B2	40.09496506	Bcer	S	Bcer	FALSE	NA	NA	NA
Bc_ctg1_5250	B2	1.048996179	Bcer	NA	Bcer	FALSE	NA	NA	NA
Bc_ctg1_5251	B2	20.51370306	Bcer	Q	Bcer	TRUE	NA	NA	NA
Bc_ctg1_5252	B2	0.349665393	Bcer	S	Bcer	FALSE	NA	NA	NA
Bc_ctg1_5253	B2	29.7215584	Bcer	M	Bcer	FALSE	NA	NA	NA
Bc_ctg1_5254	B2	8.275414301	Bcer	T	Bcer	FALSE	NA	NA	NA
Bc_ctg1_5255	B2	1.048996179	Bcer	T	Bcer	FALSE	NA	NA	NA
Bc_ctg1_5256	B2	0	Bcer	NA	Bcer	FALSE	NA	NA	NA
Bc_ctg1_5257	B2	1.398661572	Bcer	K	Bcer	FALSE	NA	NA	NA
Bc_ctg1_5258	B2	16.31771834	Bcer	S	Bcer	FALSE	NA	NA	NA
Bc_ctg1_5259	B2	16.08460808	Bcer	F	Bcer	FALSE	NA	NA	NA
Bc_ctg1_526	B2	71.91451583	Bcer	S	Bcer	FALSE	NA	NA	NA
Bc_ctg1_5260	B2	4.895315502	Bcer	F	Bcer	FALSE	NA	NA	NA
Bc_ctg1_5261	B2	18.29915557	Bcer	S	Bcer	FALSE	NA	NA	NA
Bc_ctg1_5262	B2	10.1402964	Bcer	M	Bcer	FALSE	NA	NA	NA
Bc_ctg1_5263	B2	34.84998417	Bcer	E	Bcer	FALSE	NA	NA	NA
Bc_ctg1_5264	B2	40.44463046	Bcer	S	Bcer	FALSE	NA	NA	NA
Bc_ctg1_5265	B2	1.398661572	Bcer	S	Bcer	FALSE	NA	NA	NA
Bc_ctg1_5267	B2	0	Bcer	S	Bcer	FALSE	NA	NA	NA
Bc_ctg1_5268	B2	14.21972598	Bcer	K	Bcer	FALSE	NA	NA	NA
Bc_ctg1_5269	B2	8.15885917	Bcer	K	Bcer	FALSE	NA	NA	NA
Bc_ctg1_527	B2	1.864882096	Bcer	M	Bcer	FALSE	NA	NA	NA
Bc_ctg1_5270	B2	0	Bcer	NA	Bcer	FALSE	NA	NA	NA
Bc_ctg1_5271	B2	99.07186135	Bcer	NA	Bcer	FALSE	NA	NA	NA
Bc_ctg1_5272	B2	133.455625	Bcer	EGP	Bcer	FALSE	NA	NA	NA

Bc_ctg1_5273	B2	60.37555786	Bcer	M	Bcer	FALSE	NA	NA	NA
Bc_ctg1_5274	B2	102.1022948	Bcer	M	Bcer	FALSE	NA	NA	NA
Bc_ctg1_5275	B2	187.770316	Bcer	K	Bcer	FALSE	NA	NA	NA
Bc_ctg1_5276	B2	9.091300218	Bcer	S	Bcer	FALSE	NA	NA	NA
Bc_ctg1_5277	B2	0.699330786	Bcer	EGP	Bcer	FALSE	NA	NA	NA
Bc_ctg1_5278	B2	6.060866812	Bcer	G	Bcer	FALSE	NA	NA	NA
Bc_ctg1_5279	B2	5.82775655	Bcer	NA	Bcer	FALSE	NA	NA	NA
Bc_ctg1_5280	B2	0.233110262	Bcer	NA	Bcer	FALSE	NA	NA	NA
Bc_ctg1_5281	B2	50.70148198	Bcer	NA	Bcer	FALSE	NA	NA	NA
Bc_ctg1_5282	B2	0.349665393	Bcer	S	Bcer	FALSE	NA	NA	NA
Bc_ctg1_5283	B2	0.233110262	Bcer	F	Bcer	FALSE	NA	NA	NA
Bc_ctg1_5284	B2	0.116555131	Bcer	S	Bcer	FALSE	NA	NA	NA
Bc_ctg1_5285	B2	24.24346725	Bcer	Q	Bcer	TRUE	NA	NA	NA
Bc_ctg1_5286	B2	0.233110262	Bcer	NA	Bcer	FALSE	NA	NA	NA
Bc_ctg1_5289	B2	0.116555131	Bcer	NA	Bcer	FALSE	NA	NA	NA
Bc_ctg1_5290	B2	26.691125	Bcer	EGP	Bcer	FALSE	NA	NA	NA
Bc_ctg1_5291	B2	0.349665393	Bcer	NA	Bcer	FALSE	NA	NA	NA
Bc_ctg1_5292	B2	7.226418122	Bcer	NA	Bcer	FALSE	NA	NA	NA
Bc_ctg1_5293	B2	227.5156157	Bcer	E	Bcer	FALSE	NA	NA	NA
Bc_ctg1_5294	B2	12.47139902	Bcer	I	Bcer	FALSE	NA	NA	NA
Bc_ctg1_5295	B2	29.60500327	Bcer	H	Bcer	FALSE	NA	NA	NA
Bc_ctg1_5296	B2	1.864882096	Bcer	J	Bcer	FALSE	NA	NA	NA
Bc_ctg1_5297	B2	3.146988537	Bcer	O	Bcer	FALSE	NA	NA	NA
Bc_ctg1_5298	B2	2.797323144	Bcer	NA	Bcer	FALSE	NA	NA	NA
Bc_ctg1_5299	B2	1.398661572	Bcer	V	Bcer	FALSE	NA	NA	NA
Bc_ctg1_53	B2	1.16555131	Bcer	E	Bcer	FALSE	NA	NA	NA
Bc_ctg1_5300	B2	0.233110262	Bcer	K	Bcer	FALSE	NA	NA	NA
Bc_ctg1_5301	B2	0.815885917	Bcer	NA	Bcer	FALSE	NA	NA	NA
Bc_ctg1_5302	B2	0	Bcer	S	Bcer	FALSE	NA	NA	NA
Bc_ctg1_5304	B2	0	Bcer	NA	Bcer	FALSE	NA	NA	NA
Bc_ctg1_5305	B2	0	Bcer	NA	Bcer	FALSE	NA	NA	NA
Bc_ctg1_5307	B2	0	Bcer	S	Bcer	FALSE	NA	NA	NA
Bc_ctg1_5309	B2	57.92790011	Bcer	S	Bcer	FALSE	NA	NA	NA
Bc_ctg1_5310	B2	61.3079989	Bcer	S	Bcer	FALSE	NA	NA	NA
Bc_ctg1_5311	B2	4.778760371	Bcer	C	Bcer	FALSE	NA	NA	NA
Bc_ctg1_5312	B2	7.576083515	Bcer	P	Bcer	FALSE	NA	NA	NA
Bc_ctg1_5313	B2	3.49665393	Bcer	P	Bcer	FALSE	NA	NA	NA
Bc_ctg1_5314	B2	2.447657751	Bcer	P	Bcer	FALSE	NA	NA	NA
Bc_ctg1_5315	B2	6.760197598	Bcer	S	Bcer	FALSE	NA	NA	NA
Bc_ctg1_5317	B2	22.96136081	Bcer	NA	Bcer	FALSE	NA	NA	NA
Bc_ctg1_5318	B2	10.02374127	Bcer	S	Bcer	FALSE	NA	NA	NA
Bc_ctg1_5319	B2	10.02374127	Bcer	H	Bcer	FALSE	NA	NA	NA
Bc_ctg1_532	B2	9.790631004	Bcer	L	Bcer	FALSE	NA	NA	NA
Bc_ctg1_5320	B2	24.12691212	Bcer	V	Bcer	FALSE	NA	NA	NA
Bc_ctg1_5321	B2	0.116555131	Bcer	K	Bcer	FALSE	NA	NA	NA
Bc_ctg1_5322	B2	23.07791594	Bcer	EG	Bcer	FALSE	NA	NA	NA
Bc_ctg1_5323	B2	0.233110262	Bcer	NA	Bcer	FALSE	NA	NA	NA
Bc_ctg1_5324	B2	6.527087336	Bcer	K	Bcer	FALSE	NA	NA	NA
Bc_ctg1_5325	B2	2.913878275	Bcer	J	Bcer	FALSE	NA	NA	NA
Bc_ctg1_5326	B2	2.913878275	Bcer	S	Bcer	FALSE	NA	NA	NA
Bc_ctg1_5327	B2	8.275414301	Bcer	L	Bcer	FALSE	NA	NA	NA
Bc_ctg1_5328	B2	0	Bcer	E	Bcer	FALSE	NA	NA	NA
Bc_ctg1_5329	B2	0	Bcer	E	Bcer	FALSE	NA	NA	NA
Bc_ctg1_533	B2	0.349665393	Bcer	CO	Bcer	FALSE	NA	NA	NA
Bc_ctg1_5330	B2	0.815885917	Bcer	E	Bcer	FALSE	NA	NA	NA
Bc_ctg1_5331	B2	0.349665393	Bcer	S	Bcer	FALSE	NA	NA	NA
Bc_ctg1_5332	B2	25.29246343	Bcer	S	Bcer	FALSE	NA	NA	NA
Bc_ctg1_5333	B2	141.6144842	Bcer	G	Bcer	FALSE	NA	NA	NA
Bc_ctg1_5334	B2	0.815885917	Bcer	S	Bcer	FALSE	NA	NA	NA
Bc_ctg1_5335	B2	0.815885917	Bcer	P	Bcer	FALSE	NA	NA	NA
Bc_ctg1_5336	B2	1262.525179	Bcer	M	Bcer	FALSE	NA	NA	NA
Bc_ctg1_5337	B2	773.5764044	Bcer	S	Bcer	FALSE	NA	NA	NA

Bc_ctg1_5338	B2	10.48996179	Bcer	S	Bcer	FALSE	NA	NA	NA
Bc_ctg1_5339	B2	59.21000655	Bcer	K	Bcer	FALSE	NA	NA	NA
Bc_ctg1_534	B2	1.048996179	Bcer	NA	Bcer	FALSE	NA	NA	NA
Bc_ctg1_5340	B2	54.78091157	Bcer	S	Bcer	FALSE	NA	NA	NA
Bc_ctg1_5341	B2	192.5490764	Bcer	E	Bcer	FALSE	NA	NA	NA
Bc_ctg1_5342	B2	51.63392303	Bcer	E	Bcer	FALSE	NA	NA	NA
Bc_ctg1_5343	B2	84.03624945	Bcer	E	Bcer	FALSE	NA	NA	NA
Bc_ctg1_5344	B2	39.16252402	Bcer	E	Bcer	FALSE	NA	NA	NA
Bc_ctg1_5345	B2	170.6367118	Bcer	E	Bcer	FALSE	NA	NA	NA
Bc_ctg1_5346	B2	105.8320589	Bcer	K	Bcer	FALSE	NA	NA	NA
Bc_ctg1_5347	B2	0	Bcer	S	Bcer	FALSE	NA	NA	NA
Bc_ctg1_5348	B2	15.15216703	Bcer	Q	Bcer	TRUE	NA	NA	NA
Bc_ctg1_5349	B2	6.060866812	Bcer	J	Bcer	FALSE	NA	NA	NA
Bc_ctg1_535	B2	0.233110262	Bcer	V	Bcer	FALSE	NA	NA	NA
Bc_ctg1_5350	B2	5.82775655	Bcer	S	Bcer	FALSE	NA	NA	NA
Bc_ctg1_5351	B2	0.116555131	Bcer	S	Bcer	FALSE	NA	NA	NA
Bc_ctg1_5352	B2	2.797323144	Bcer	NA	Bcer	FALSE	NA	NA	NA
Bc_ctg1_5353	B2	0.582775655	Bcer	S	Bcer	FALSE	NA	NA	NA
Bc_ctg1_5354	B2	31.93610589	Bcer	M	Bcer	FALSE	NA	NA	NA
Bc_ctg1_5355	B2	0	Bcer	S	Bcer	FALSE	NA	NA	NA
Bc_ctg1_5356	B2	4.545650109	Bcer	E	Bcer	FALSE	NA	NA	NA
Bc_ctg1_5357	B2	3.846319323	Bcer	C	Bcer	FALSE	NA	NA	NA
Bc_ctg1_5358	B2	0.815885917	Bcer	K	Bcer	FALSE	NA	NA	NA
Bc_ctg1_5359	B2	2.680768013	Bcer	NA	Bcer	FALSE	NA	NA	NA
Bc_ctg1_536	B2	7476.661988	Bcer	C	Bcer	FALSE	NA	NA	NA
Bc_ctg1_5360	B2	7.809193777	Bcer	NA	Bcer	FALSE	NA	NA	NA
Bc_ctg1_5361	B2	61.54110917	Bcer	S	Bcer	FALSE	NA	NA	NA
Bc_ctg1_5362	B2	953.6540818	Bcer	S	Bcer	FALSE	NA	NA	NA
Bc_ctg1_5363	B2	0.815885917	Bcer	G	Bcer	FALSE	NA	NA	NA
Bc_ctg1_5364	B2	0.466220524	Bcer	G	Bcer	FALSE	NA	NA	NA
Bc_ctg1_5365	B2	0.116555131	Bcer	G	Bcer	FALSE	NA	NA	NA
Bc_ctg1_5366	B2	0	Bcer	G	Bcer	FALSE	NA	NA	NA
Bc_ctg1_5367	B2	0	Bcer	K	Bcer	FALSE	NA	NA	NA
Bc_ctg1_5368	B2	0.349665393	Bcer	G	Bcer	FALSE	NA	NA	NA
Bc_ctg1_5369	B2	0	Bcer	G	Bcer	FALSE	NA	NA	NA
Bc_ctg1_537	B2	0	Bcer	S	Bcer	FALSE	NA	NA	NA
Bc_ctg1_5370	B2	0.116555131	Bcer	G	Bcer	FALSE	NA	NA	NA
Bc_ctg1_5371	B2	2.097992358	Bcer	E	Bcer	FALSE	NA	NA	NA
Bc_ctg1_5372	B2	3.146988537	Bcer	S	Bcer	FALSE	NA	NA	NA
Bc_ctg1_5373	B2	9.557520742	Bcer	EGP	Bcer	FALSE	NA	NA	NA
Bc_ctg1_5374	B2	6.527087336	Bcer	K	Bcer	FALSE	NA	NA	NA
Bc_ctg1_5375	B2	1.048996179	Bcer	F	Bcer	FALSE	NA	NA	NA
Bc_ctg1_5376	B2	27.04079039	Bcer	E	Bcer	FALSE	NA	NA	NA
Bc_ctg1_5377	B2	17.25015939	Bcer	E	Bcer	FALSE	NA	NA	NA
Bc_ctg1_5378	B2	3.962874454	Bcer	E	Bcer	FALSE	NA	NA	NA
Bc_ctg1_5379	B2	18.76537609	Bcer	E	Bcer	FALSE	NA	NA	NA
Bc_ctg1_538	B2	3.962874454	Bcer	G	Bcer	FALSE	NA	NA	NA
Bc_ctg1_5380	B2	7.342973253	Bcer	K	Bcer	FALSE	NA	NA	NA
Bc_ctg1_5381	B2	1.048996179	Bcer	E	Bcer	FALSE	NA	NA	NA
Bc_ctg1_5382	B2	5.361536026	Bcer	E	Bcer	FALSE	NA	NA	NA
Bc_ctg1_5383	B2	0	Bcer	E	Bcer	FALSE	NA	NA	NA
Bc_ctg1_5384	B2	255.372292	Bcer	I	Bcer	FALSE	NA	NA	NA
Bc_ctg1_5385	B2	21.91236463	Bcer	V	Bcer	FALSE	NA	NA	NA
Bc_ctg1_5386	B2	2.564212882	Bcer	V	Bcer	FALSE	NA	NA	NA
Bc_ctg1_5387	B2	0.932441048	Bcer	P	Bcer	FALSE	NA	NA	NA
Bc_ctg1_5388	B2	4.429094978	Bcer	S	Bcer	FALSE	NA	NA	NA
Bc_ctg1_5389	B2	0.932441048	Bcer	K	Bcer	FALSE	NA	NA	NA
Bc_ctg1_539	B2	1.282106441	Bcer	NA	Bcer	FALSE	NA	NA	NA
Bc_ctg1_5390	B2	14.33628111	Bcer	L	Bcer	FALSE	NA	NA	NA
Bc_ctg1_5391	B2	1.515216703	Bcer	NA	Bcer	FALSE	NA	NA	NA
Bc_ctg1_5392	B2	15.61838755	Bcer	S	Bcer	FALSE	NA	NA	NA
Bc_ctg1_5393	B2	25.40901856	Bcer	F	Bcer	FALSE	NA	NA	NA

Bc_ctg1_5394	B2	15.50183242	Bcer	J	Bcer	FALSE	NA	NA	NA
Bc_ctg1_5395	B2	15.85149782	Bcer	E	Bcer	FALSE	NA	NA	NA
Bc_ctg1_5397	B2	0	Bcer	NA	Bcer	FALSE	NA	NA	NA
Bc_ctg1_5398	B2	6.876752729	Bcer	S	Bcer	FALSE	NA	NA	NA
Bc_ctg1_5399	B2	3.263543668	Bcer	K	Bcer	FALSE	NA	NA	NA
Bc_ctg1_54	B2	0.699330786	Bcer	P	Bcer	FALSE	NA	NA	NA
Bc_ctg1_5400	B2	0.699330786	Bcer	S	Bcer	FALSE	NA	NA	NA
Bc_ctg1_5401	B2	0.699330786	Bcer	S	Bcer	FALSE	NA	NA	NA
Bc_ctg1_5402	B2	1.631771834	Bcer	NA	Bcer	FALSE	NA	NA	NA
Bc_ctg1_5403	B2	698.8645655	Bcer	EGP	Bcer	FALSE	NA	NA	NA
Bc_ctg1_5404	B2	1.748326965	Bcer	E	Bcer	FALSE	NA	NA	NA
Bc_ctg1_5405	B2	1.981437227	Bcer	C	Bcer	FALSE	NA	NA	NA
Bc_ctg1_5406	B2	0.349665393	Bcer	S	Bcer	FALSE	NA	NA	NA
Bc_ctg1_5407	B2	0.116555131	Bcer	NA	Bcer	FALSE	NA	NA	NA
Bc_ctg1_5408	B2	11.42240284	Bcer	F	Bcer	FALSE	NA	NA	NA
Bc_ctg1_5409	B2	26.92423526	Bcer	V	Bcer	FALSE	NA	NA	NA
Bc_ctg1_541	B2	0.349665393	Bcer	O	Bcer	FALSE	NA	NA	NA
Bc_ctg1_5410	B2	0.582775655	Bcer	NA	Bcer	FALSE	NA	NA	NA
Bc_ctg1_5411	B2	7.576083515	Bcer	S	Bcer	FALSE	NA	NA	NA
Bc_ctg1_5412	B2	21.09647871	Bcer	C	Bcer	FALSE	NA	NA	NA
Bc_ctg1_5413	B2	5.711201419	Bcer	K	Bcer	FALSE	NA	NA	NA
Bc_ctg1_5414	B2	49.30282041	Bcer	J	Bcer	FALSE	NA	NA	NA
Bc_ctg1_5415	B2	0	Bcer	F	Bcer	FALSE	NA	NA	NA
Bc_ctg1_5416	B2	2.214547489	Bcer	S	Bcer	FALSE	NA	NA	NA
Bc_ctg1_5417	B2	13.05417467	Bcer	S	Bcer	FALSE	NA	NA	NA
Bc_ctg1_5418	B2	13.5203952	Bcer	V	Bcer	FALSE	NA	NA	NA
Bc_ctg1_5419	B2	5.128425764	Bcer	K	Bcer	FALSE	NA	NA	NA
Bc_ctg1_542	B2	36.01553548	Bcer	EGP	Bcer	FALSE	NA	NA	NA
Bc_ctg1_5421	B2	9.907186135	Bcer	GM	Bcer	FALSE	NA	NA	NA
Bc_ctg1_5422	B2	4.545650109	Bcer	V	Bcer	FALSE	NA	NA	NA
Bc_ctg1_5423	B2	7.925748908	Bcer	K	Bcer	FALSE	NA	NA	NA
Bc_ctg1_5424	B2	4.895315502	Bcer	K	Bcer	FALSE	NA	NA	NA
Bc_ctg1_5425	B2	1.864882096	Bcer	NA	Bcer	FALSE	NA	NA	NA
Bc_ctg1_5426	B2	0.116555131	Bcer	L	Bcer	FALSE	NA	NA	NA
Bc_ctg1_5427	B2	0	Bcer	EG	Bcer	FALSE	NA	NA	NA
Bc_ctg1_5428	B2	18.0660453	Bcer	K	Bcer	FALSE	NA	NA	NA
Bc_ctg1_5429	B2	17.36671452	Bcer	S	Bcer	FALSE	NA	NA	NA
Bc_ctg1_543	B2	22.02891976	Bcer	NA	Bcer	FALSE	NA	NA	NA
Bc_ctg1_5430	B2	1.631771834	Bcer	S	Bcer	FALSE	NA	NA	NA
Bc_ctg1_5431	B2	18.29915557	Bcer	S	Bcer	FALSE	NA	NA	NA
Bc_ctg1_5432	B2	9.907186135	Bcer	F	Bcer	FALSE	NA	NA	NA
Bc_ctg1_5433	B2	11.30584771	Bcer	T	Bcer	FALSE	NA	NA	NA
Bc_ctg1_5434	B2	8.042304039	Bcer	S	Bcer	FALSE	NA	NA	NA
Bc_ctg1_5435	B2	9.091300218	Bcer	E	Bcer	FALSE	NA	NA	NA
Bc_ctg1_5436	B2	3.729764192	Bcer	S	Bcer	FALSE	NA	NA	NA
Bc_ctg1_5437	B2	0.116555131	Bcer	J	Bcer	FALSE	NA	NA	NA
Bc_ctg1_544	B2	36.59831113	Bcer	J	Bcer	FALSE	NA	NA	NA
Bc_ctg1_545	B2	1.981437227	Bcer	F	Bcer	FALSE	NA	NA	NA
Bc_ctg1_546	B2	4.429094978	Bcer	NA	Bcer	FALSE	NA	NA	NA
Bc_ctg1_547	B2	4.312539847	Bcer	I	Bcer	FALSE	NA	NA	NA
Bc_ctg1_548	B2	3.030433406	Bcer	IQ	Bcer	TRUE	NA	NA	NA
Bc_ctg1_549	B2	3.729764192	Bcer	S	Bcer	FALSE	NA	NA	NA
Bc_ctg1_55	B2	1.282106441	Bcer	S	Bcer	FALSE	NA	NA	NA
Bc_ctg1_550	B2	9.32441048	Bcer	IQ	Bcer	TRUE	NA	NA	NA
Bc_ctg1_551	B2	1.631771834	Bcer	S	Bcer	FALSE	NA	NA	NA
Bc_ctg1_552	B2	2.913878275	Bcer	V	Bcer	FALSE	NA	NA	NA
Bc_ctg1_553	B2	7.226418122	Bcer	K	Bcer	FALSE	NA	NA	NA
Bc_ctg1_554	B2	1.398661572	Bcer	NA	Bcer	FALSE	NA	NA	NA
Bc_ctg1_555	B2	1.282106441	Bcer	K	Bcer	FALSE	NA	NA	NA
Bc_ctg1_556	B2	1.282106441	Bcer	NA	Bcer	FALSE	NA	NA	NA
Bc_ctg1_557	B2	14.45283624	Bcer	S	Bcer	FALSE	NA	NA	NA
Bc_ctg1_558	B2	7.925748908	Bcer	V	Bcer	FALSE	NA	NA	NA

Bc_ctg1_559	B2	9.790631004	Bcer	T	Bcer	FALSE	NA	NA	NA
Bc_ctg1_56	B2	0	Bcer	L	Bcer	FALSE	NA	NA	NA
Bc_ctg1_560	B2	23.54413646	Bcer	S	Bcer	FALSE	NA	NA	NA
Bc_ctg1_561	B2	1.631771834	Bcer	E	Bcer	FALSE	NA	NA	NA
Bc_ctg1_562	B2	32.16921616	Bcer	T	Bcer	FALSE	NA	NA	NA
Bc_ctg1_563	B2	13.63695033	Bcer	S	Bcer	FALSE	NA	NA	NA
Bc_ctg1_564	B2	10.60651692	Bcer	E	Bcer	FALSE	NA	NA	NA
Bc_ctg1_565	B2	1.748326965	Bcer	E	Bcer	FALSE	NA	NA	NA
Bc_ctg1_566	B2	1.398661572	Bcer	O	Bcer	FALSE	NA	NA	NA
Bc_ctg1_567	B2	0.116555131	Bcer	E	Bcer	FALSE	NA	NA	NA
Bc_ctg1_568	B2	51.98358842	Bcer	C	Bcer	FALSE	NA	NA	NA
Bc_ctg1_569	B2	221.2216386	Bcer	K	Bcer	FALSE	NA	NA	NA
Bc_ctg1_57	B2	16.66738373	Bcer	L	Bcer	FALSE	NA	NA	NA
Bc_ctg1_570	B2	57.11201419	Bcer	E	Bcer	FALSE	NA	NA	NA
Bc_ctg1_571	B2	63.172881	Bcer	Q	Bcer	TRUE	NA	NA	NA
Bc_ctg1_572	B2	82.98725327	Bcer	E	Bcer	FALSE	NA	NA	NA
Bc_ctg1_573	B2	64.6880977	Bcer	E	Bcer	FALSE	NA	NA	NA
Bc_ctg1_574	B2	747.7011653	Bcer	K	Bcer	FALSE	NA	NA	NA
Bc_ctg1_575	B2	3.962874454	Bcer	Q	Bcer	TRUE	NA	NA	NA
Bc_ctg1_576	B2	2.797323144	Bcer	S	Bcer	FALSE	NA	NA	NA
Bc_ctg1_577	B2	0.699330786	Bcer	F	Bcer	FALSE	NA	NA	NA
Bc_ctg1_578	B2	25.40901856	Bcer	NA	Bcer	FALSE	NA	NA	NA
Bc_ctg1_579	B2	9.907186135	Bcer	S	Bcer	FALSE	NA	NA	NA
Bc_ctg1_58	B2	20.86336845	Bcer	L	Bcer	FALSE	NA	NA	NA
Bc_ctg1_580	B2	151.6382254	Bcer	O	Bcer	FALSE	NA	NA	NA
Bc_ctg1_581	B2	50.0021512	Bcer	O	Bcer	FALSE	NA	NA	NA
Bc_ctg1_582	B2	0	Bcer	NA	Bcer	FALSE	NA	NA	NA
Bc_ctg1_583	B2	34.9665393	Bcer	EG	Bcer	FALSE	NA	NA	NA
Bc_ctg1_584	B2	26.22490447	Bcer	E	Bcer	FALSE	NA	NA	NA
Bc_ctg1_585	B2	22.37858515	Bcer	S	Bcer	FALSE	NA	NA	NA
Bc_ctg1_586	B2	15.38527729	Bcer	V	Bcer	FALSE	NA	NA	NA
Bc_ctg1_588	B2	4.429094978	Bcer	V	Bcer	FALSE	NA	NA	NA
Bc_ctg1_589	B2	1.282106441	Bcer	NA	Bcer	FALSE	NA	NA	NA
Bc_ctg1_59	B2	2.680768013	Bcer	NA	Bcer	FALSE	NA	NA	NA
Bc_ctg1_590	B2	44.64061517	Bcer	K	Bcer	FALSE	NA	NA	NA
Bc_ctg1_591	B2	4.429094978	Bcer	S	Bcer	FALSE	NA	NA	NA
Bc_ctg1_592	B2	30.53744432	Bcer	M	Bcer	FALSE	NA	NA	NA
Bc_ctg1_593	B2	1.398661572	Bcer	F	Bcer	FALSE	NA	NA	NA
Bc_ctg1_594	B2	0.233110262	Bcer	S	Bcer	FALSE	NA	NA	NA
Bc_ctg1_595	B2	12.93761954	Bcer	S	Bcer	FALSE	NA	NA	NA
Bc_ctg1_596	B2	0.116555131	Bcer	NA	Bcer	FALSE	NA	NA	NA
Bc_ctg1_597	B2	10.83962718	Bcer	NA	Bcer	FALSE	NA	NA	NA
Bc_ctg1_598	B2	7.342973253	Bcer	S	Bcer	FALSE	NA	NA	NA
Bc_ctg1_599	B2	0.699330786	Bcer	M	Bcer	FALSE	NA	NA	NA
Bc_ctg1_6	B2	3.030433406	Bcer	NA	Bcer	FALSE	NA	NA	NA
Bc_ctg1_60	B2	15.15216703	Bcer	E	Bcer	FALSE	NA	NA	NA
Bc_ctg1_600	B2	31.5864405	Bcer	V	Bcer	FALSE	NA	NA	NA
Bc_ctg1_601	B2	12.12173362	Bcer	V	Bcer	FALSE	NA	NA	NA
Bc_ctg1_602	B2	94.7593215	Bcer	S	Bcer	FALSE	NA	NA	NA
Bc_ctg1_603	B2	32.40232642	Bcer	S	Bcer	FALSE	NA	NA	NA
Bc_ctg1_604	B2	2687.06199	Bcer	G	Bcer	FALSE	NA	NA	NA
Bc_ctg1_605	B2	5.244980895	Bcer	P	Bcer	FALSE	NA	NA	NA
Bc_ctg1_606	B2	1.048996179	Bcer	P	Bcer	FALSE	NA	NA	NA
Bc_ctg1_607	B2	3.146988537	Bcer	P	Bcer	FALSE	NA	NA	NA
Bc_ctg1_608	B2	1.748326965	Bcer	P	Bcer	FALSE	NA	NA	NA
Bc_ctg1_609	B2	17.59982478	Bcer	F	Bcer	FALSE	NA	NA	NA
Bc_ctg1_61	B2	18.76537609	Bcer	K	Bcer	FALSE	NA	NA	NA
Bc_ctg1_610	B2	9.674075873	Bcer	S	Bcer	FALSE	NA	NA	NA
Bc_ctg1_611	B2	11.77206823	Bcer	L	Bcer	FALSE	NA	NA	NA
Bc_ctg1_612	B2	415.1693766	Bcer	K	Bcer	FALSE	NA	NA	NA
Bc_ctg1_613	B2	10.1402964	Bcer	S	Bcer	FALSE	NA	NA	NA
Bc_ctg1_614	B2	6.410532205	Bcer	S	Bcer	FALSE	NA	NA	NA

Bc_ctg1_615	B2	0	Bcer	K	Bcer	FALSE	NA	NA	NA
Bc_ctg1_616	B2	3.613209061	Bcer	S	Bcer	FALSE	NA	NA	NA
Bc_ctg1_617	B2	36.24864574	Bcer	S	Bcer	FALSE	NA	NA	NA
Bc_ctg1_618	B2	40.09496506	Bcer	M	Bcer	FALSE	NA	NA	NA
Bc_ctg1_619	B2	306.5399945	Bcer	M	Bcer	FALSE	NA	NA	NA
Bc_ctg1_62	B2	0.932441048	Bcer	L	Bcer	FALSE	NA	NA	NA
Bc_ctg1_620	B2	0.582775655	Bcer	S	Bcer	FALSE	NA	NA	NA
Bc_ctg1_621	B2	2.797323144	Bcer	L	Bcer	FALSE	NA	NA	NA
Bc_ctg1_622	B2	0.233110262	Bcer	NA	Bcer	FALSE	NA	NA	NA
Bc_ctg1_623	B2	2.913878275	Bcer	NA	Bcer	FALSE	NA	NA	NA
Bc_ctg1_624	B2	0.116555131	Bcer	NA	Bcer	FALSE	NA	NA	NA
Bc_ctg1_625	B2	69.69996834	Bcer	K	Bcer	FALSE	NA	NA	NA
Bc_ctg1_626	B2	2502.322107	Bcer	E	Bcer	FALSE	NA	NA	NA
Bc_ctg1_627	B2	215.277327	Bcer	K	Bcer	FALSE	NA	NA	NA
Bc_ctg1_628	B2	82.52103275	Bcer	P	Bcer	FALSE	NA	NA	NA
Bc_ctg1_629	B2	76.22705567	Bcer	S	Bcer	FALSE	NA	NA	NA
Bc_ctg1_63	B2	23.42758133	Bcer	V	Bcer	FALSE	NA	NA	NA
Bc_ctg1_630	B2	17.83293504	Bcer	S	Bcer	FALSE	NA	NA	NA
Bc_ctg1_631	B2	0	Bcer	NA	Bcer	FALSE	NA	NA	NA
Bc_ctg1_632	B2	2.564212882	Bcer	O	Bcer	FALSE	NA	NA	NA
Bc_ctg1_633	B2	94.7593215	Bcer	L	Bcer	FALSE	NA	NA	NA
Bc_ctg1_634	B2	2.447657751	Bcer	Q	Bcer	TRUE	NA	NA	NA
Bc_ctg1_635	B2	270.8741244	Bcer	J	Bcer	FALSE	NA	NA	NA
Bc_ctg1_636	B2	111.193595	Bcer	J	Bcer	FALSE	NA	NA	NA
Bc_ctg1_637	B2	0	Bcer	NA	Bcer	FALSE	NA	NA	NA
Bc_ctg1_638	B2	100.5870781	Bcer	T	Bcer	FALSE	NA	NA	NA
Bc_ctg1_639	B2	3929.073466	Bcer	GT	Bcer	FALSE	NA	NA	NA
Bc_ctg1_64	B2	12.58795415	Bcer	S	Bcer	FALSE	NA	NA	NA
Bc_ctg1_640	B2	1005.17145	Bcer	G	Bcer	FALSE	NA	NA	NA
Bc_ctg1_641	B2	1896.468536	Bcer	K	Bcer	FALSE	NA	NA	NA
Bc_ctg1_642	B2	163.7599591	Bcer	C	Bcer	FALSE	NA	NA	NA
Bc_ctg1_643	B2	111.0770398	Bcer	S	Bcer	FALSE	NA	NA	NA
Bc_ctg1_645	B2	77.74227238	Bcer	NA	Bcer	FALSE	NA	NA	NA
Bc_ctg1_646	B2	1.864882096	Bcer	E	Bcer	FALSE	NA	NA	NA
Bc_ctg1_647	B2	0.233110262	Bcer	G	Bcer	FALSE	NA	NA	NA
Bc_ctg1_648	B2	1.398661572	Bcer	S	Bcer	FALSE	NA	NA	NA
Bc_ctg1_649	B2	3.146988537	Bcer	P	Bcer	FALSE	NA	NA	NA
Bc_ctg1_65	B2	60.02589246	Bcer	F	Bcer	FALSE	NA	NA	NA
Bc_ctg1_650	B2	372.1605333	Bcer	L	Bcer	FALSE	NA	NA	NA
Bc_ctg1_651	B2	6.060866812	Bcer	M	Bcer	FALSE	NA	NA	NA
Bc_ctg1_652	B2	195.3463996	Bcer	P	Bcer	FALSE	NA	NA	NA
Bc_ctg1_653	B2	44.17439465	Bcer	P	Bcer	FALSE	NA	NA	NA
Bc_ctg1_654	B2	22.02891976	Bcer	S	Bcer	FALSE	NA	NA	NA
Bc_ctg1_655	B2	0.116555131	Bcer	NA	Bcer	FALSE	NA	NA	NA
Bc_ctg1_656	B2	52.56636408	Bcer	S	Bcer	FALSE	NA	NA	NA
Bc_ctg1_657	B2	28.78911736	Bcer	HP	Bcer	FALSE	NA	NA	NA
Bc_ctg1_658	B2	39.51218941	Bcer	P	Bcer	FALSE	NA	NA	NA
Bc_ctg1_659	B2	63.05632587	Bcer	P	Bcer	FALSE	NA	NA	NA
Bc_ctg1_66	B2	8.275414301	Bcer	I	Bcer	FALSE	NA	NA	NA
Bc_ctg1_660	B2	233.3433723	Bcer	P	Bcer	FALSE	NA	NA	NA
Bc_ctg1_661	B2	14.33628111	Bcer	L	Bcer	FALSE	NA	NA	NA
Bc_ctg1_662	B2	10.02374127	Bcer	K	Bcer	FALSE	Bc_Ga0417192_01_rn07	Regulation	SMCOG1287
Bc_ctg1_663	B2	5.478091157	Bcer	L	Bcer	FALSE	Bc_Ga0417192_01_rn07	Other	NA
Bc_ctg1_664	B2	19.81437227	Bcer	L	Bcer	FALSE	Bc_Ga0417192_01_rn07	Other	NA
Bc_ctg1_665	B2	329.3848002	Bcer	E	Bcer	FALSE	Bc_Ga0417192_01_rn07	Other	NA
Bc_ctg1_666	B2	1.631771834	Bcer	S	Bcer	FALSE	Bc_Ga0417192_01_rn07	Other	NA
Bc_ctg1_667	B2	10.83962718	Bcer	NA	Bcer	FALSE	Bc_Ga0417192_01_rn07	Other	NA
Bc_ctg1_668	B2	8.15885917	Bcer	G	Bcer	FALSE	Bc_Ga0417192_01_rn07	Ancillary biosynthesis	SMCOG1101
Bc_ctg1_669	B2	12.00517849	Bcer	S	Bcer	FALSE	Bc_Ga0417192_01_rn07	Ancillary biosynthesis	SMCOG1262
Bc_ctg1_67	B2	1.16555131	Bcer	Q	Bcer	TRUE	NA	NA	NA
Bc_ctg1_670	B2	174.366476	Bcer	T	Bcer	FALSE	Bc_Ga0417192_01_rn07	Ancillary biosynthesis	SMCOG1025
Bc_ctg1_671	B2	0.699330786	Bcer	M	Bcer	FALSE	Bc_Ga0417192_01_rn07	Core biosynthesis	None

Bc_ctg1_672	B2	0	Bcer	NA	Bcer	FALSE	Bc_Ga0417192_01_rn07	Other	NA
Bc_ctg1_673	B2	0	Bcer	GM	Bcer	FALSE	Bc_Ga0417192_01_rn07	Core biosynthesis	SMCOG1010
Bc_ctg1_674	B2	0	Bcer	M	Bcer	FALSE	Bc_Ga0417192_01_rn07	Core biosynthesis	SMCOG1010
Bc_ctg1_675	B2	0.233110262	Bcer	S	Bcer	FALSE	Bc_Ga0417192_01_rn07	Other	NA
Bc_ctg1_676	B2	1.515216703	Bcer	S	Bcer	FALSE	Bc_Ga0417192_01_rn07	Other	NA
Bc_ctg1_677	B2	3.030433406	Bcer	M	Bcer	FALSE	Bc_Ga0417192_01_rn07	Other	NA
Bc_ctg1_678	B2	1.282106441	Bcer	S	Bcer	FALSE	Bc_Ga0417192_01_rn07	Other	NA
Bc_ctg1_679	B2	10.25685153	Bcer	K	Bcer	FALSE	Bc_Ga0417192_01_rn07	Ancillary biosynthesis	SMCOG1019
Bc_ctg1_68	B2	1.16555131	Bcer	Q	Bcer	TRUE	NA	NA	NA
Bc_ctg1_680	B2	0.233110262	Bcer	NA	Bcer	FALSE	Bc_Ga0417192_01_rn07	Other	NA
Bc_ctg1_681	B2	21.91236463	Bcer	K	Bcer	FALSE	Bc_Ga0417192_01_rn07	Regulation	SMCOG1136
Bc_ctg1_682	B2	46.85516266	Bcer	G	Bcer	FALSE	Bc_Ga0417192_01_rn07	Other	NA
Bc_ctg1_683	B2	49.41937554	Bcer	U	Bcer	FALSE	Bc_Ga0417192_01_rn07	Other	NA
Bc_ctg1_684	B2	57.22856932	Bcer	O	Bcer	FALSE	NA	NA	NA
Bc_ctg1_685	B2	585.2233127	Bcer	M	Bcer	FALSE	NA	NA	NA
Bc_ctg1_686	B2	10.83962718	Bcer	S	Bcer	FALSE	NA	NA	NA
Bc_ctg1_687	B2	12.70450928	Bcer	M	Bcer	FALSE	NA	NA	NA
Bc_ctg1_688	B2	9.790631004	Bcer	S	Bcer	FALSE	NA	NA	NA
Bc_ctg1_69	B2	0.466220524	Bcer	NA	Bcer	FALSE	NA	NA	NA
Bc_ctg1_690	B2	169.937381	Bcer	L	Bcer	FALSE	NA	NA	NA
Bc_ctg1_691	B2	169.2380502	Bcer	L	Bcer	FALSE	NA	NA	NA
Bc_ctg1_692	B2	2.33110262	Bcer	S	Bcer	FALSE	NA	NA	NA
Bc_ctg1_693	B2	98.60564082	Bcer	S	Bcer	FALSE	NA	NA	NA
Bc_ctg1_694	B2	508.063816	Bcer	J	Bcer	FALSE	NA	NA	NA
Bc_ctg1_695	B2	872.9979312	Bcer	C	Bcer	FALSE	NA	NA	NA
Bc_ctg1_696	B2	1909.639266	Bcer	C	Bcer	FALSE	NA	NA	NA
Bc_ctg1_697	B2	17.71637991	Bcer	E	Bcer	FALSE	NA	NA	NA
Bc_ctg1_698	B2	46.38894214	Bcer	S	Bcer	FALSE	NA	NA	NA
Bc_ctg1_699	B2	135.9032827	Bcer	S	Bcer	FALSE	NA	NA	NA
Bc_ctg1_7	B2	0.466220524	Bcer	S	Bcer	FALSE	NA	NA	NA
Bc_ctg1_70	B2	5.361536026	Bcer	S	Bcer	FALSE	NA	NA	NA
Bc_ctg1_700	B2	3495.022158	Bcer	S	Bcer	FALSE	NA	NA	NA
Bc_ctg1_701	B2	542.2144694	Bcer	L	Bcer	FALSE	NA	NA	NA
Bc_ctg1_702	B2	366.9155524	Bcer	S	Bcer	FALSE	NA	NA	NA
Bc_ctg1_703	B2	123.664994	Bcer	I	Bcer	FALSE	NA	NA	NA
Bc_ctg1_704	B2	364.1182292	Bcer	S	Bcer	FALSE	NA	NA	NA
Bc_ctg1_705	B2	225.7672887	Bcer	S	Bcer	FALSE	NA	NA	NA
Bc_ctg1_706	B2	1.16555131	Bcer	S	Bcer	FALSE	NA	NA	NA
Bc_ctg1_707	B2	22.96136081	Bcer	IQ	Bcer	TRUE	NA	NA	NA
Bc_ctg1_708	B2	214.5779962	Bcer	S	Bcer	FALSE	NA	NA	NA
Bc_ctg1_709	B2	134.8542866	Bcer	S	Bcer	FALSE	NA	NA	NA
Bc_ctg1_71	B2	4.429094978	Bcer	K	Bcer	FALSE	NA	NA	NA
Bc_ctg1_710	B2	398.2688826	Bcer	S	Bcer	FALSE	NA	NA	NA
Bc_ctg1_711	B2	336.6112183	Bcer	S	Bcer	FALSE	NA	NA	NA
Bc_ctg1_712	B2	438.9466233	Bcer	S	Bcer	FALSE	NA	NA	NA
Bc_ctg1_713	B2	1302.853254	Bcer	S	Bcer	FALSE	NA	NA	NA
Bc_ctg1_714	B2	391.7417953	Bcer	K	Bcer	FALSE	NA	NA	NA
Bc_ctg1_715	B2	70.74896452	Bcer	NA	Bcer	FALSE	NA	NA	NA
Bc_ctg1_716	B2	161.3123013	Bcer	D	Bcer	FALSE	NA	NA	NA
Bc_ctg1_717	B2	0.932441048	Bcer	S	Bcer	FALSE	NA	NA	NA
Bc_ctg1_718	B2	3.962874454	Bcer	OU	Bcer	FALSE	NA	NA	NA
Bc_ctg1_719	B2	1099.697661	Bcer	S	Bcer	FALSE	NA	NA	NA
Bc_ctg1_72	B2	0	Bcer	K	Bcer	FALSE	NA	NA	NA
Bc_ctg1_720	B2	876.0283646	Bcer	E	Bcer	FALSE	NA	NA	NA
Bc_ctg1_721	B2	1442.835967	Bcer	E	Bcer	FALSE	NA	NA	NA
Bc_ctg1_722	B2	1259.611301	Bcer	E	Bcer	FALSE	NA	NA	NA
Bc_ctg1_723	B2	2.680768013	Bcer	H	Bcer	FALSE	NA	NA	NA
Bc_ctg1_724	B2	14.10317085	Bcer	NA	Bcer	FALSE	NA	NA	NA
Bc_ctg1_726	B2	82.87069814	Bcer	T	Bcer	FALSE	NA	NA	NA
Bc_ctg1_727	B2	53.84847052	Bcer	NA	Bcer	FALSE	NA	NA	NA
Bc_ctg1_728	B2	1.048996179	Bcer	NA	Bcer	FALSE	NA	NA	NA
Bc_ctg1_729	B2	0.116555131	Bcer	L	Bcer	FALSE	NA	NA	NA

Bc_ctg1_73	B2	1.398661572	Bcer	P	Bcer	FALSE	NA	NA	NA
Bc_ctg1_731	B2	0.116555131	Bcer	M	Bcer	FALSE	NA	NA	NA
Bc_ctg1_732	B2	0	Bcer	S	Bcer	FALSE	NA	NA	NA
Bc_ctg1_733	B2	117.8372374	Bcer	S	Bcer	FALSE	NA	NA	NA
Bc_ctg1_734	B2	0	Bcer	NA	Bcer	FALSE	NA	NA	NA
Bc_ctg1_735	B2	0.233110262	Bcer	U	Bcer	FALSE	NA	NA	NA
Bc_ctg1_736	B2	0.466220524	Bcer	S	Bcer	FALSE	NA	NA	NA
Bc_ctg1_737	B2	2.564212882	Bcer	D	Bcer	FALSE	NA	NA	NA
Bc_ctg1_739	B2	0	Bcer	S	Bcer	FALSE	NA	NA	NA
Bc_ctg1_74	B2	4.895315502	Bcer	S	Bcer	FALSE	NA	NA	NA
Bc_ctg1_740	B2	0	Bcer	S	Bcer	FALSE	NA	NA	NA
Bc_ctg1_741	B2	0.116555131	Bcer	NA	Bcer	FALSE	NA	NA	NA
Bc_ctg1_742	B2	0	Bcer	S	Bcer	FALSE	NA	NA	NA
Bc_ctg1_743	B2	0	Bcer	S	Bcer	FALSE	NA	NA	NA
Bc_ctg1_745	B2	0.349665393	Bcer	S	Bcer	FALSE	NA	NA	NA
Bc_ctg1_746	B2	1.048996179	Bcer	OU	Bcer	FALSE	NA	NA	NA
Bc_ctg1_747	B2	0.815885917	Bcer	S	Bcer	FALSE	NA	NA	NA
Bc_ctg1_748	B2	0.466220524	Bcer	S	Bcer	FALSE	NA	NA	NA
Bc_ctg1_749	B2	0.349665393	Bcer	S	Bcer	FALSE	NA	NA	NA
Bc_ctg1_75	B2	0	Bcer	NA	Bcer	FALSE	NA	NA	NA
Bc_ctg1_750	B2	0.815885917	Bcer	V	Bcer	FALSE	NA	NA	NA
Bc_ctg1_751	B2	1.048996179	Bcer	NA	Bcer	FALSE	NA	NA	NA
Bc_ctg1_752	B2	1957.543425	Bcer	NA	Bcer	FALSE	NA	NA	NA
Bc_ctg1_753	B2	0	Bcer	NA	Bcer	FALSE	NA	NA	NA
Bc_ctg1_754	B2	0.116555131	Bcer	NA	Bcer	FALSE	NA	NA	NA
Bc_ctg1_755	B2	0	Bcer	L	Bcer	FALSE	NA	NA	NA
Bc_ctg1_756	B2	0.116555131	Bcer	S	Bcer	FALSE	NA	NA	NA
Bc_ctg1_757	B2	68.4178619	Bcer	S	Bcer	FALSE	NA	NA	NA
Bc_ctg1_758	B2	69.23374781	Bcer	S	Bcer	FALSE	NA	NA	NA
Bc_ctg1_759	B2	0	Bcer	NA	Bcer	FALSE	NA	NA	NA
Bc_ctg1_76	B2	0	Bcer	S	Bcer	FALSE	NA	NA	NA
Bc_ctg1_760	B2	0.932441048	Bcer	NA	Bcer	FALSE	NA	NA	NA
Bc_ctg1_761	B2	0.116555131	Bcer	NA	Bcer	FALSE	NA	NA	NA
Bc_ctg1_762	B2	2.447657751	Bcer	L	Bcer	FALSE	NA	NA	NA
Bc_ctg1_763	B2	1.282106441	Bcer	L	Bcer	FALSE	NA	NA	NA
Bc_ctg1_764	B2	0.116555131	Bcer	NA	Bcer	FALSE	NA	NA	NA
Bc_ctg1_765	B2	0.466220524	Bcer	NA	Bcer	FALSE	NA	NA	NA
Bc_ctg1_766	B2	0.466220524	Bcer	NA	Bcer	FALSE	NA	NA	NA
Bc_ctg1_767	B2	2.447657751	Bcer	NA	Bcer	FALSE	NA	NA	NA
Bc_ctg1_768	B2	0.699330786	Bcer	NA	Bcer	FALSE	NA	NA	NA
Bc_ctg1_769	B2	0	Bcer	K	Bcer	FALSE	NA	NA	NA
Bc_ctg1_77	B2	46.73860753	Bcer	G	Bcer	FALSE	NA	NA	NA
Bc_ctg1_770	B2	15.15216703	Bcer	K	Bcer	FALSE	NA	NA	NA
Bc_ctg1_771	B2	1.048996179	Bcer	E	Bcer	FALSE	NA	NA	NA
Bc_ctg1_772	B2	0.466220524	Bcer	NA	Bcer	FALSE	NA	NA	NA
Bc_ctg1_773	B2	0.466220524	Bcer	NA	Bcer	FALSE	NA	NA	NA
Bc_ctg1_774	B2	8.391969432	Bcer	L	Bcer	FALSE	NA	NA	NA
Bc_ctg1_775	B2	0.116555131	Bcer	H	Bcer	FALSE	NA	NA	NA
Bc_ctg1_776	B2	0	Bcer	E	Bcer	FALSE	NA	NA	NA
Bc_ctg1_778	B2	266.3284743	Bcer	S	Bcer	FALSE	NA	NA	NA
Bc_ctg1_779	B2	28.67256223	Bcer	G	Bcer	FALSE	NA	NA	NA
Bc_ctg1_78	B2	0.116555131	Bcer	S	Bcer	FALSE	NA	NA	NA
Bc_ctg1_780	B2	1572.445272	Bcer	J	Bcer	FALSE	NA	NA	NA
Bc_ctg1_781	B2	569.9545906	Bcer	J	Bcer	FALSE	NA	NA	NA
Bc_ctg1_782	B2	238.7049083	Bcer	H	Bcer	FALSE	NA	NA	NA
Bc_ctg1_783	B2	92.77788427	Bcer	J	Bcer	FALSE	NA	NA	NA
Bc_ctg1_784	B2	356.775256	Bcer	J	Bcer	FALSE	NA	NA	NA
Bc_ctg1_785	B2	153.3865524	Bcer	S	Bcer	FALSE	NA	NA	NA
Bc_ctg1_786	B2	2786.833182	Bcer	J	Bcer	FALSE	NA	NA	NA
Bc_ctg1_787	B2	253.0411894	Bcer	J	Bcer	FALSE	NA	NA	NA
Bc_ctg1_788	B2	76.57672107	Bcer	K	Bcer	FALSE	NA	NA	NA
Bc_ctg1_789	B2	871.1330491	Bcer	K	Bcer	FALSE	NA	NA	NA

Bc_ctg1_79	B2	9.207855349	Bcer	I	Bcer	FALSE	NA	NA	NA
Bc_ctg1_790	B2	544.0793515	Bcer	S	Bcer	FALSE	NA	NA	NA
Bc_ctg1_791	B2	460.9755431	Bcer	L	Bcer	FALSE	NA	NA	NA
Bc_ctg1_792	B2	469.6006228	Bcer	J	Bcer	FALSE	NA	NA	NA
Bc_ctg1_793	B2	405.6118559	Bcer	M	Bcer	FALSE	NA	NA	NA
Bc_ctg1_794	B2	324.9557052	Bcer	I	Bcer	FALSE	NA	NA	NA
Bc_ctg1_795	B2	142.7800355	Bcer	S	Bcer	FALSE	NA	NA	NA
Bc_ctg1_796	B2	152.2210011	Bcer	I	Bcer	FALSE	NA	NA	NA
Bc_ctg1_797	B2	453.6325698	Bcer	J	Bcer	FALSE	NA	NA	NA
Bc_ctg1_798	B2	518.7868881	Bcer	F	Bcer	FALSE	NA	NA	NA
Bc_ctg1_799	B2	2010.459455	Bcer	J	Bcer	FALSE	NA	NA	NA
Bc_ctg1_8	B2	2.33110262	Bcer	NA	Bcer	FALSE	NA	NA	NA
Bc_ctg1_80	B2	8.858189956	Bcer	T	Bcer	FALSE	NA	NA	NA
Bc_ctg1_800	B2	4500.077053	Bcer	J	Bcer	FALSE	NA	NA	NA
Bc_ctg1_801	B2	610.0495556	Bcer	K	Bcer	FALSE	NA	NA	NA
Bc_ctg1_802	B2	626.4838291	Bcer	O	Bcer	FALSE	NA	NA	NA
Bc_ctg1_803	B2	68.76752729	Bcer	O	Bcer	FALSE	NA	NA	NA
Bc_ctg1_804	B2	156.5335409	Bcer	L	Bcer	FALSE	NA	NA	NA
Bc_ctg1_805	B2	350.9474994	Bcer	J	Bcer	FALSE	NA	NA	NA
Bc_ctg1_806	B2	2353.36465	Bcer	L	Bcer	FALSE	NA	NA	NA
Bc_ctg1_807	B2	1.16555131	Bcer	LU	Bcer	FALSE	NA	NA	NA
Bc_ctg1_808	B2	1389.337161	Bcer	C	Bcer	FALSE	NA	NA	NA
Bc_ctg1_809	B2	1713.360426	Bcer	C	Bcer	FALSE	NA	NA	NA
Bc_ctg1_81	B2	37.41419705	Bcer	L	Bcer	FALSE	NA	NA	NA
Bc_ctg1_810	B2	22.26203002	Bcer	L	Bcer	FALSE	NA	NA	NA
Bc_ctg1_811	B2	152.5706665	Bcer	S	Bcer	FALSE	NA	NA	NA
Bc_ctg1_812	B2	112.5922565	Bcer	U	Bcer	FALSE	NA	NA	NA
Bc_ctg1_813	B2	2868.188664	Bcer	J	Bcer	FALSE	NA	NA	NA
Bc_ctg1_814	B2	83.33691866	Bcer	J	Bcer	FALSE	NA	NA	NA
Bc_ctg1_815	B2	44.99028056	Bcer	J	Bcer	FALSE	NA	NA	NA
Bc_ctg1_816	B2	167.4897232	Bcer	S	Bcer	FALSE	NA	NA	NA
Bc_ctg1_817	B2	764.7182145	Bcer	J	Bcer	FALSE	NA	NA	NA
Bc_ctg1_818	B2	1689.233514	Bcer	U	Bcer	FALSE	NA	NA	NA
Bc_ctg1_819	B2	121.6835568	Bcer	S	Bcer	FALSE	NA	NA	NA
Bc_ctg1_82	B2	17.01704913	Bcer	K	Bcer	FALSE	NA	NA	NA
Bc_ctg1_820	B2	154.3189934	Bcer	U	Bcer	FALSE	NA	NA	NA
Bc_ctg1_821	B2	207.2350229	Bcer	D	Bcer	FALSE	NA	NA	NA
Bc_ctg1_822	B2	305.8406637	Bcer	J	Bcer	FALSE	NA	NA	NA
Bc_ctg1_823	B2	113.5246976	Bcer	IQ	Bcer	TRUE	NA	NA	NA
Bc_ctg1_824	B2	239.7539045	Bcer	IQ	Bcer	TRUE	NA	NA	NA
Bc_ctg1_825	B2	182.1756697	Bcer	I	Bcer	FALSE	NA	NA	NA
Bc_ctg1_826	B2	117.9537926	Bcer	I	Bcer	FALSE	NA	NA	NA
Bc_ctg1_827	B2	47.08827292	Bcer	K	Bcer	FALSE	NA	NA	NA
Bc_ctg1_828	B2	111.7763706	Bcer	L	Bcer	FALSE	NA	NA	NA
Bc_ctg1_829	B2	1307.74857	Bcer	S	Bcer	FALSE	NA	NA	NA
Bc_ctg1_83	B2	0.116555131	Bcer	EGP	Bcer	FALSE	NA	NA	NA
Bc_ctg1_830	B2	195.5795098	Bcer	S	Bcer	FALSE	NA	NA	NA
Bc_ctg1_831	B2	23.66069159	Bcer	J	Bcer	FALSE	NA	NA	NA
Bc_ctg1_832	B2	16.5508286	Bcer	H	Bcer	FALSE	NA	NA	NA
Bc_ctg1_833	B2	261.7828242	Bcer	G	Bcer	FALSE	NA	NA	NA
Bc_ctg1_834	B2	144.4118073	Bcer	S	Bcer	FALSE	NA	NA	NA
Bc_ctg1_835	B2	743.0389601	Bcer	KLT	Bcer	FALSE	NA	NA	NA
Bc_ctg1_836	B2	147.675351	Bcer	T	Bcer	FALSE	NA	NA	NA
Bc_ctg1_837	B2	417.0342587	Bcer	J	Bcer	FALSE	NA	NA	NA
Bc_ctg1_838	B2	271.2237898	Bcer	J	Bcer	FALSE	NA	NA	NA
Bc_ctg1_839	B2	133.3390699	Bcer	J	Bcer	FALSE	NA	NA	NA
Bc_ctg1_84	B2	1.515216703	Bcer	E	Bcer	FALSE	NA	NA	NA
Bc_ctg1_840	B2	33.91754312	Bcer	J	Bcer	FALSE	NA	NA	NA
Bc_ctg1_841	B2	129.1430851	Bcer	L	Bcer	FALSE	NA	NA	NA
Bc_ctg1_842	B2	50.11870633	Bcer	H	Bcer	FALSE	NA	NA	NA
Bc_ctg1_843	B2	55.82990775	Bcer	K	Bcer	FALSE	NA	NA	NA
Bc_ctg1_844	B2	142.1972598	Bcer	F	Bcer	FALSE	NA	NA	NA

Bc_ctg1_845	B2	49.30282041	Bcer	S	Bcer	FALSE	NA	NA	NA
Bc_ctg1_846	B2	158.8646435	Bcer	S	Bcer	FALSE	NA	NA	NA
Bc_ctg1_847	B2	16.31771834	Bcer	P	Bcer	FALSE	NA	NA	NA
Bc_ctg1_848	B2	108.3962718	Bcer	K	Bcer	FALSE	NA	NA	NA
Bc_ctg1_849	B2	8.625079694	Bcer	S	Bcer	FALSE	NA	NA	NA
Bc_ctg1_85	B2	0.116555131	Bcer	I	Bcer	FALSE	NA	NA	NA
Bc_ctg1_850	B2	1.631771834	Bcer	E	Bcer	FALSE	NA	NA	NA
Bc_ctg1_851	B2	0.116555131	Bcer	S	Bcer	FALSE	NA	NA	NA
Bc_ctg1_852	B2	0.582775655	Bcer	S	Bcer	FALSE	NA	NA	NA
Bc_ctg1_853	B2	0.116555131	Bcer	S	Bcer	FALSE	NA	NA	NA
Bc_ctg1_855	B2	0.116555131	Bcer	C	Bcer	FALSE	NA	NA	NA
Bc_ctg1_856	B2	15.38527729	Bcer	S	Bcer	FALSE	NA	NA	NA
Bc_ctg1_857	B2	19.81437227	Bcer	S	Bcer	FALSE	NA	NA	NA
Bc_ctg1_858	B2	243.3671135	Bcer	F	Bcer	FALSE	NA	NA	NA
Bc_ctg1_859	B2	247.9127636	Bcer	F	Bcer	FALSE	NA	NA	NA
Bc_ctg1_86	B2	0.815885917	Bcer	P	Bcer	FALSE	NA	NA	NA
Bc_ctg1_860	B2	480.44025	Bcer	F	Bcer	FALSE	NA	NA	NA
Bc_ctg1_861	B2	266.3284743	Bcer	C	Bcer	FALSE	NA	NA	NA
Bc_ctg1_862	B2	1770.588995	Bcer	F	Bcer	FALSE	NA	NA	NA
Bc_ctg1_863	B2	347.6839558	Bcer	F	Bcer	FALSE	NA	NA	NA
Bc_ctg1_864	B2	207.8177986	Bcer	F	Bcer	FALSE	NA	NA	NA
Bc_ctg1_865	B2	77.74227238	Bcer	F	Bcer	FALSE	NA	NA	NA
Bc_ctg1_866	B2	118.1869028	Bcer	F	Bcer	FALSE	NA	NA	NA
Bc_ctg1_867	B2	105.9486141	Bcer	F	Bcer	FALSE	NA	NA	NA
Bc_ctg1_868	B2	369.0135447	Bcer	J	Bcer	FALSE	NA	NA	NA
Bc_ctg1_869	B2	103.9671768	Bcer	MU	Bcer	FALSE	NA	NA	NA
Bc_ctg1_87	B2	2.447657751	Bcer	S	Bcer	FALSE	NA	NA	NA
Bc_ctg1_870	B2	0	Bcer	T	Bcer	FALSE	NA	NA	NA
Bc_ctg1_871	B2	1174.4095	Bcer	J	Bcer	FALSE	NA	NA	NA
Bc_ctg1_872	B2	501.7698389	Bcer	D	Bcer	FALSE	NA	NA	NA
Bc_ctg1_873	B2	700.3797822	Bcer	S	Bcer	FALSE	NA	NA	NA
Bc_ctg1_874	B2	87.53290338	Bcer	S	Bcer	FALSE	NA	NA	NA
Bc_ctg1_875	B2	200.8244907	Bcer	D	Bcer	FALSE	NA	NA	NA
Bc_ctg1_876	B2	334.3966708	Bcer	S	Bcer	FALSE	Bc_Ga0417192_01_rn08	Other	NA
Bc_ctg1_877	B2	383.3498258	Bcer	S	Bcer	FALSE	Bc_Ga0417192_01_rn08	Other	NA
Bc_ctg1_878	B2	0	Bcer	S	Bcer	FALSE	Bc_Ga0417192_01_rn08	Other	NA
Bc_ctg1_879	B2	0.116555131	Bcer	K	Bcer	FALSE	Bc_Ga0417192_01_rn08	Other	NA
Bc_ctg1_88	B2	0.233110262	Bcer	CP	Bcer	FALSE	NA	NA	NA
Bc_ctg1_880	B2	1.398661572	Bcer	K	Bcer	FALSE	Bc_Ga0417192_01_rn08	Other	NA
Bc_ctg1_881	B2	3.380098799	Bcer	M	Bcer	FALSE	Bc_Ga0417192_01_rn08	Other	NA
Bc_ctg1_882	B2	1369.522789	Bcer	D	Bcer	FALSE	Bc_Ga0417192_01_rn08	Other	NA
Bc_ctg1_883	B2	1255.298761	Bcer	D	Bcer	FALSE	Bc_Ga0417192_01_rn08	Other	NA
Bc_ctg1_884	B2	18.88193122	Bcer	D	Bcer	FALSE	Bc_Ga0417192_01_rn08	Other	NA
Bc_ctg1_885	B2	80.073375	Bcer	M	Bcer	FALSE	Bc_Ga0417192_01_rn08	Other	NA
Bc_ctg1_886	B2	48.1372691	Bcer	M	Bcer	FALSE	Bc_Ga0417192_01_rn08	Core biosynthesis	SMCOG1062
Bc_ctg1_887	B2	54.8974667	Bcer	D	Bcer	FALSE	Bc_Ga0417192_01_rn08	Other	NA
Bc_ctg1_888	B2	456.5464481	Bcer	M	Bcer	FALSE	Bc_Ga0417192_01_rn08	Other	NA
Bc_ctg1_889	B2	219.1236463	Bcer	M	Bcer	FALSE	Bc_Ga0417192_01_rn08	Core biosynthesis	None
Bc_ctg1_89	B2	0.466220524	Bcer	S	Bcer	FALSE	NA	NA	NA
Bc_ctg1_890	B2	171.4525977	Bcer	M	Bcer	FALSE	Bc_Ga0417192_01_rn08	Other	NA
Bc_ctg1_891	B2	360.2719099	Bcer	M	Bcer	FALSE	Bc_Ga0417192_01_rn08	Other	NA
Bc_ctg1_892	B2	609.5833351	Bcer	M	Bcer	FALSE	Bc_Ga0417192_01_rn08	Other	NA
Bc_ctg1_893	B2	351.6468302	Bcer	D	Bcer	FALSE	Bc_Ga0417192_01_rn08	Other	NA
Bc_ctg1_894	B2	432.8857565	Bcer	J	Bcer	FALSE	Bc_Ga0417192_01_rn08	Other	NA
Bc_ctg1_895	B2	204.7873652	Bcer	S	Bcer	FALSE	Bc_Ga0417192_01_rn08	Other	NA
Bc_ctg1_896	B2	16.78393886	Bcer	H	Bcer	FALSE	NA	NA	NA
Bc_ctg1_897	B2	305.9572189	Bcer	K	Bcer	FALSE	NA	NA	NA
Bc_ctg1_898	B2	12.35484389	Bcer	S	Bcer	FALSE	NA	NA	NA
Bc_ctg1_899	B2	63.98876692	Bcer	J	Bcer	FALSE	NA	NA	NA
Bc_ctg1_9	B2	0.233110262	Bcer	NA	Bcer	FALSE	NA	NA	NA
Bc_ctg1_90	B2	0	Bcer	EGP	Bcer	FALSE	NA	NA	NA
Bc_ctg1_900	B2	757.142131	Bcer	S	Bcer	FALSE	NA	NA	NA

Bc_ctg1_901	B2	90.33022652	Bcer	S	Bcer	FALSE	NA	NA	NA
Bc_ctg1_902	B2	25.75868395	Bcer	T	Bcer	FALSE	NA	NA	NA
Bc_ctg1_903	B2	20.39714792	Bcer	S	Bcer	FALSE	NA	NA	NA
Bc_ctg1_904	B2	0.932441048	Bcer	S	Bcer	FALSE	NA	NA	NA
Bc_ctg1_905	B2	52.21669869	Bcer	H	Bcer	FALSE	NA	NA	NA
Bc_ctg1_906	B2	28.08978657	Bcer	L	Bcer	FALSE	NA	NA	NA
Bc_ctg1_907	B2	91.3792227	Bcer	S	Bcer	FALSE	NA	NA	NA
Bc_ctg1_908	B2	65.38742849	Bcer	S	Bcer	FALSE	NA	NA	NA
Bc_ctg1_909	B2	95.92487281	Bcer	S	Bcer	FALSE	NA	NA	NA
Bc_ctg1_91	B2	3.380098799	Bcer	K	Bcer	FALSE	NA	NA	NA
Bc_ctg1_910	B2	38.57974836	Bcer	G	Bcer	FALSE	NA	NA	NA
Bc_ctg1_911	B2	0.349665393	Bcer	S	Bcer	FALSE	NA	NA	NA
Bc_ctg1_912	B2	0.116555131	Bcer	S	Bcer	FALSE	NA	NA	NA
Bc_ctg1_913	B2	92.77788427	Bcer	S	Bcer	FALSE	NA	NA	NA
Bc_ctg1_914	B2	6.760197598	Bcer	S	Bcer	FALSE	NA	NA	NA
Bc_ctg1_915	B2	12.00517849	Bcer	S	Bcer	FALSE	NA	NA	NA
Bc_ctg1_916	B2	41.7267369	Bcer	S	Bcer	FALSE	NA	NA	NA
Bc_ctg1_917	B2	31.5864405	Bcer	C	Bcer	FALSE	NA	NA	NA
Bc_ctg1_918	B2	90.33022652	Bcer	C	Bcer	FALSE	NA	NA	NA
Bc_ctg1_919	B2	343.7210813	Bcer	C	Bcer	FALSE	NA	NA	NA
Bc_ctg1_92	B2	0	Bcer	S	Bcer	FALSE	NA	NA	NA
Bc_ctg1_920	B2	214.461441	Bcer	C	Bcer	FALSE	NA	NA	NA
Bc_ctg1_921	B2	222.9699656	Bcer	O	Bcer	FALSE	NA	NA	NA
Bc_ctg1_922	B2	191.26697	Bcer	O	Bcer	FALSE	NA	NA	NA
Bc_ctg1_923	B2	0.349665393	Bcer	L	Bcer	FALSE	NA	NA	NA
Bc_ctg1_924	B2	2.097992358	Bcer	L	Bcer	FALSE	NA	NA	NA
Bc_ctg1_925	B2	3242.563744	Bcer	C	Bcer	FALSE	NA	NA	NA
Bc_ctg1_926	B2	109.6783783	Bcer	D	Bcer	FALSE	NA	NA	NA
Bc_ctg1_927	B2	226.5831747	Bcer	S	Bcer	FALSE	NA	NA	NA
Bc_ctg1_928	B2	135.0873968	Bcer	S	Bcer	FALSE	NA	NA	NA
Bc_ctg1_929	B2	277.0515464	Bcer	T	Bcer	FALSE	NA	NA	NA
Bc_ctg1_93	B2	12.47139902	Bcer	G	Bcer	FALSE	NA	NA	NA
Bc_ctg1_930	B2	177.6300196	Bcer	S	Bcer	FALSE	NA	NA	NA
Bc_ctg1_931	B2	14.91905677	Bcer	S	Bcer	FALSE	NA	NA	NA
Bc_ctg1_932	B2	1.748326965	Bcer	NA	Bcer	FALSE	NA	NA	NA
Bc_ctg1_933	B2	2.564212882	Bcer	S	Bcer	FALSE	NA	NA	NA
Bc_ctg1_934	B2	2956.187787	Bcer	T	Bcer	FALSE	NA	NA	NA
Bc_ctg1_935	B2	46.27238701	Bcer	NA	Bcer	FALSE	NA	NA	NA
Bc_ctg1_936	B2	52.79947434	Bcer	G	Bcer	FALSE	NA	NA	NA
Bc_ctg1_937	B2	0.466220524	Bcer	NA	Bcer	FALSE	NA	NA	NA
Bc_ctg1_938	B2	42.54262281	Bcer	S	Bcer	FALSE	NA	NA	NA
Bc_ctg1_939	B2	4.66220524	Bcer	S	Bcer	FALSE	NA	NA	NA
Bc_ctg1_94	B2	3.962874454	Bcer	K	Bcer	FALSE	NA	NA	NA
Bc_ctg1_940	B2	391.8583504	Bcer	E	Bcer	FALSE	NA	NA	NA
Bc_ctg1_941	B2	4.195984716	Bcer	H	Bcer	FALSE	NA	NA	NA
Bc_ctg1_942	B2	0.233110262	Bcer	S	Bcer	FALSE	NA	NA	NA
Bc_ctg1_943	B2	0.815885917	Bcer	S	Bcer	FALSE	NA	NA	NA
Bc_ctg1_944	B2	0.116555131	Bcer	S	Bcer	FALSE	NA	NA	NA
Bc_ctg1_945	B2	4.312539847	Bcer	S	Bcer	FALSE	NA	NA	NA
Bc_ctg1_946	B2	4.545650109	Bcer	S	Bcer	FALSE	NA	NA	NA
Bc_ctg1_947	B2	0	Bcer	NA	Bcer	FALSE	NA	NA	NA
Bc_ctg1_948	B2	3378.700137	Bcer	C	Bcer	FALSE	NA	NA	NA
Bc_ctg1_949	B2	4165.214161	Bcer	C	Bcer	FALSE	NA	NA	NA
Bc_ctg1_95	B2	34.9665393	Bcer	H	Bcer	FALSE	NA	NA	NA
Bc_ctg1_950	B2	1829.799002	Bcer	C	Bcer	FALSE	NA	NA	NA
Bc_ctg1_951	B2	3233.588999	Bcer	C	Bcer	FALSE	NA	NA	NA
Bc_ctg1_953	B2	193.1318521	Bcer	J	Bcer	FALSE	NA	NA	NA
Bc_ctg1_954	B2	53.38225	Bcer	S	Bcer	FALSE	NA	NA	NA
Bc_ctg1_955	B2	32.40232642	Bcer	S	Bcer	FALSE	NA	NA	NA
Bc_ctg1_956	B2	1640.630024	Bcer	S	Bcer	FALSE	NA	NA	NA
Bc_ctg1_957	B2	84.26935971	Bcer	P	Bcer	FALSE	NA	NA	NA
Bc_ctg1_958	B2	58.74378602	Bcer	C	Bcer	FALSE	NA	NA	NA

Bc_ctg1_959	B2	127.278203	Bcer	E	Bcer	FALSE	NA	NA	NA
Bc_ctg1_96	B2	1.282106441	Bcer	T	Bcer	FALSE	NA	NA	NA
Bc_ctg1_960	B2	389.8769132	Bcer	E	Bcer	FALSE	NA	NA	NA
Bc_ctg1_961	B2	100.3539678	Bcer	K	Bcer	FALSE	NA	NA	NA
Bc_ctg1_962	B2	49.30282041	Bcer	S	Bcer	FALSE	NA	NA	NA
Bc_ctg1_963	B2	1.398661572	Bcer	S	Bcer	FALSE	NA	NA	NA
Bc_ctg1_964	B2	0.349665393	Bcer	S	Bcer	FALSE	NA	NA	NA
Bc_ctg1_966	B2	20.39714792	Bcer	S	Bcer	FALSE	NA	NA	NA
Bc_ctg1_967	B2	2.797323144	Bcer	O	Bcer	FALSE	NA	NA	NA
Bc_ctg1_968	B2	5.82775655	Bcer	S	Bcer	FALSE	NA	NA	NA
Bc_ctg1_969	B2	74.71183897	Bcer	T	Bcer	FALSE	NA	NA	NA
Bc_ctg1_97	B2	0.699330786	Bcer	P	Bcer	FALSE	NA	NA	NA
Bc_ctg1_970	B2	103.7340666	Bcer	IQ	Bcer	TRUE	NA	NA	NA
Bc_ctg1_971	B2	0.466220524	Bcer	S	Bcer	FALSE	NA	NA	NA
Bc_ctg1_972	B2	0	Bcer	NA	Bcer	FALSE	NA	NA	NA
Bc_ctg1_973	B2	186.9544301	Bcer	P	Bcer	FALSE	NA	NA	NA
Bc_ctg1_974	B2	35.31620469	Bcer	FP	Bcer	FALSE	NA	NA	NA
Bc_ctg1_975	B2	52.33325382	Bcer	S	Bcer	FALSE	NA	NA	NA
Bc_ctg1_976	B2	35.43275982	Bcer	L	Bcer	FALSE	NA	NA	NA
Bc_ctg1_977	B2	0.116555131	Bcer	T	Bcer	FALSE	NA	NA	NA
Bc_ctg1_978	B2	0.233110262	Bcer	S	Bcer	FALSE	Bc_Ga0417192_01_rn09	Other	NA
Bc_ctg1_979	B2	30.30433406	Bcer	NA	Bcer	FALSE	Bc_Ga0417192_01_rn09	Other	NA
Bc_ctg1_98	B2	0.116555131	Bcer	E	Bcer	FALSE	NA	NA	NA
Bc_ctg1_980	B2	66.43642467	Bcer	P	Bcer	FALSE	Bc_Ga0417192_01_rn09	Transport	SMCOG1005
Bc_ctg1_981	B2	16.78393886	Bcer	K	Bcer	FALSE	Bc_Ga0417192_01_rn09	Regulation	SMCOG1278
Bc_ctg1_982	B2	2424.23017	Bcer	E	Bcer	FALSE	Bc_Ga0417192_01_rn09	Other	NA
Bc_ctg1_983	B2	6.410532205	Bcer	S	Bcer	FALSE	Bc_Ga0417192_01_rn09	Other	NA
Bc_ctg1_984	B2	2.797323144	Bcer	M	Bcer	FALSE	Bc_Ga0417192_01_rn09	Other	NA
Bc_ctg1_985	B2	0.233110262	Bcer	S	Bcer	FALSE	Bc_Ga0417192_01_rn09	Other	NA
Bc_ctg1_986	B2	66.31986954	Bcer	T	Bcer	FALSE	Bc_Ga0417192_01_rn09	Regulation	SMCOG1003
Bc_ctg1_987	B2	20.28059279	Bcer	NA	Bcer	FALSE	Bc_Ga0417192_01_rn09	Other	NA
Bc_ctg1_988	B2	76.92638646	Bcer	E	Bcer	FALSE	Bc_Ga0417192_01_rn09	Core biosynthesis	SMCOG1019
Bc_ctg1_989	B2	88.11567903	Bcer	K	Bcer	FALSE	Bc_Ga0417192_01_rn09	Regulation	SMCOG1078
Bc_ctg1_99	B2	0.349665393	Bcer	K	Bcer	FALSE	NA	NA	NA
Bc_ctg1_990	B2	329.7344656	Bcer	P	Bcer	FALSE	Bc_Ga0417192_01_rn09	Transport	SMCOG1069
Bc_ctg1_991	B2	600.3754798	Bcer	P	Bcer	FALSE	Bc_Ga0417192_01_rn09	Transport	SMCOG1067
Bc_ctg1_992	B2	599.9092592	Bcer	G	Bcer	FALSE	Bc_Ga0417192_01_rn09	Ancillary biosynthesis	SMCOG1143
Bc_ctg1_993	B2	36.01553548	Bcer	G	Bcer	FALSE	Bc_Ga0417192_01_rn09	Core biosynthesis	SMCOG1257
Bc_ctg1_994	B2	0	Bcer	L	Bcer	FALSE	Bc_Ga0417192_01_rn09	Other	SMCOG1026
Bc_ctg1_996	B2	177.047244	Bcer	G	Bcer	FALSE	Bc_Ga0417192_01_rn09	Core biosynthesis	SMCOG1257
Bc_ctg1_997	B2	357.1249214	Bcer	P	Bcer	FALSE	Bc_Ga0417192_01_rn09	Transport	SMCOG1000
Bc_ctg1_998	B2	194.7636239	Bcer	S	Bcer	FALSE	Bc_Ga0417192_01_rn09	Ancillary biosynthesis	SMCOG1001
Bc_ctg2_1	B2	0.582775655	Bcer	M	Bcer	FALSE	Bc_Ga0417192_02_rn01	Core biosynthesis	None
Bc_ctg2_10	B2	1.515216703	Bcer	S	Bcer	FALSE	Bc_Ga0417192_02_rn01	Other	NA
Bc_ctg2_100	B2	15.15216703	Bcer	K	Bcer	FALSE	Bc_Ga0417192_02_rn02	Ancillary biosynthesis	SMCOG1019
Bc_ctg2_101	B2	21.09647871	Bcer	NA	Bcer	FALSE	Bc_Ga0417192_02_rn02	Other	NA
Bc_ctg2_102	B2	173.5505901	Bcer	E	Bcer	FALSE	Bc_Ga0417192_02_rn02	Transport	SMCOG1202
Bc_ctg2_103	B2	1.048996179	Bcer	M	Bcer	FALSE	Bc_Ga0417192_02_rn02	Ancillary biosynthesis	SMCOG1064
Bc_ctg2_104	B2	0.116555131	Bcer	M	Bcer	FALSE	Bc_Ga0417192_02_rn02	Ancillary biosynthesis	SMCOG1152
Bc_ctg2_105	B2	0.349665393	Bcer	M	Bcer	FALSE	Bc_Ga0417192_02_rn02	Core biosynthesis	SMCOG1010
Bc_ctg2_106	B2	0.349665393	Bcer	M	Bcer	FALSE	Bc_Ga0417192_02_rn02	Core biosynthesis	SMCOG1045
Bc_ctg2_107	B2	0	Bcer	NA	Bcer	FALSE	Bc_Ga0417192_02_rn02	Other	NA
Bc_ctg2_108	B2	0.349665393	Bcer	NA	Bcer	FALSE	Bc_Ga0417192_02_rn02	Other	NA
Bc_ctg2_109	B2	0.349665393	Bcer	S	Bcer	FALSE	Bc_Ga0417192_02_rn02	Other	NA
Bc_ctg2_11	B2	0	Bcer	NA	Bcer	FALSE	Bc_Ga0417192_02_rn01	Other	NA
Bc_ctg2_110	B2	0.466220524	Bcer	M	Bcer	FALSE	Bc_Ga0417192_02_rn02	Core biosynthesis	SMCOG1045
Bc_ctg2_111	B2	0	Bcer	S	Bcer	FALSE	Bc_Ga0417192_02_rn02	Ancillary biosynthesis	None
Bc_ctg2_116	B2	2.33110262	Bcer	NA	Bcer	FALSE	Bc_Ga0417192_02_rn02	Other	NA
Bc_ctg2_118	B2	3.962874454	Bcer	K	Bcer	FALSE	Bc_Ga0417192_02_rn02	Other	NA
Bc_ctg2_119	B2	15.96805295	Bcer	E	Bcer	FALSE	Bc_Ga0417192_02_rn02	Ancillary biosynthesis	SMCOG1158
Bc_ctg2_12	B2	0	Bcer	NA	Bcer	FALSE	Bc_Ga0417192_02_rn01	Other	NA
Bc_ctg2_120	B2	7.342973253	Bcer	E	Bcer	FALSE	Bc_Ga0417192_02_rn02	Ancillary biosynthesis	SMCOG1081

Bc_ctg2_121	B2	9.32441048	Bcer	K	Bcer	FALSE	Bc_Ga0417192_02_rn02	Other	NA
Bc_ctg2_122	B2	0.233110262	Bcer	NA	Bcer	FALSE	Bc_Ga0417192_02_rn02	Other	NA
Bc_ctg2_123	B2	2.33110262	Bcer	Q	Bcer	TRUE	Bc_Ga0417192_02_rn02	Ancillary biosynthesis	SMCOG1004
Bc_ctg2_124	B2	29.83811354	Bcer	Q	Bcer	TRUE	Bc_Ga0417192_02_rn02	Core biosynthesis	SMCOG1127
Bc_ctg2_125	B2	4.545650109	Bcer	Q	Bcer	TRUE	Bc_Ga0417192_02_rn02	Core biosynthesis	SMCOG1127
Bc_ctg2_126	B2	19.11504148	Bcer	IQ	Bcer	TRUE	Bc_Ga0417192_02_rn02	Core biosynthesis	SMCOG1127
Bc_ctg2_127	B2	1.515216703	Bcer	S	Bcer	FALSE	Bc_Ga0417192_02_rn02	Other	NA
Bc_ctg2_128	B2	63.28943613	Bcer	EGP	Bcer	FALSE	Bc_Ga0417192_02_rn02	Transport	SMCOG1202
Bc_ctg2_129	B2	79.02437882	Bcer	K	Bcer	FALSE	Bc_Ga0417192_02_rn02	Other	NA
Bc_ctg2_130	B2	20.39714792	Bcer	EG	Bcer	FALSE	Bc_Ga0417192_02_rn02	Transport	SMCOG1166
Bc_ctg2_131	B2	5.944311681	Bcer	K	Bcer	FALSE	Bc_Ga0417192_02_rn02	Regulation	SMCOG1071
Bc_ctg2_132	B2	9.207855349	Bcer	EG	Bcer	FALSE	Bc_Ga0417192_02_rn02	Transport	SMCOG1166
Bc_ctg2_133	B2	0.932441048	Bcer	NA	Bcer	FALSE	Bc_Ga0417192_02_rn02	Other	NA
Bc_ctg2_134	B2	4.895315502	Bcer	C	Bcer	FALSE	Bc_Ga0417192_02_rn02	Ancillary biosynthesis	None
Bc_ctg2_135	B2	0.932441048	Bcer	K	Bcer	FALSE	Bc_Ga0417192_02_rn02	Other	NA
Bc_ctg2_136	B2	1.398661572	Bcer	K	Bcer	FALSE	Bc_Ga0417192_02_rn02	Other	NA
Bc_ctg2_137	B2	10.1402964	Bcer	K	Bcer	FALSE	Bc_Ga0417192_02_rn02	Other	NA
Bc_ctg2_139	B2	0.233110262	Bcer	NA	Bcer	FALSE	Bc_Ga0417192_02_rn02	Other	NA
Bc_ctg2_14	B2	31.81955076	Bcer	S	Bcer	FALSE	Bc_Ga0417192_02_rn01	Other	NA
Bc_ctg2_140	B2	0	Bcer	L	Bcer	FALSE	Bc_Ga0417192_02_rn02	Other	SMCOG1132
Bc_ctg2_141	B2	0.699330786	Bcer	L	Bcer	FALSE	Bc_Ga0417192_02_rn02	Other	NA
Bc_ctg2_142	B2	0	Bcer	L	Bcer	FALSE	Bc_Ga0417192_02_rn02	Other	NA
Bc_ctg2_143	B2	12.58795415	Bcer	V	Bcer	FALSE	Bc_Ga0417192_02_rn02	Ancillary biosynthesis	SMCOG1053
Bc_ctg2_144	B2	0.116555131	Bcer	NA	Bcer	FALSE	Bc_Ga0417192_02_rn02	Other	NA
Bc_ctg2_145	B2	4.079429585	Bcer	L	Bcer	FALSE	NA	NA	NA
Bc_ctg2_146	B2	0	Bcer	S	Bcer	FALSE	NA	NA	NA
Bc_ctg2_147	B2	4.195984716	Bcer	NA	Bcer	FALSE	NA	NA	NA
Bc_ctg2_148	B2	30.53744432	Bcer	NA	Bcer	FALSE	NA	NA	NA
Bc_ctg2_149	B2	1.282106441	Bcer	CO	Bcer	FALSE	NA	NA	NA
Bc_ctg2_15	B2	65.38742849	Bcer	P	Bcer	FALSE	Bc_Ga0417192_02_rn01	Ancillary biosynthesis	SMCOG1170
Bc_ctg2_150	B2	0	Bcer	P	Bcer	FALSE	NA	NA	NA
Bc_ctg2_152	B2	0	Bcer	NA	Bcer	FALSE	NA	NA	NA
Bc_ctg2_153	B2	3.380098799	Bcer	L	Bcer	FALSE	NA	NA	NA
Bc_ctg2_154	B2	1.048996179	Bcer	NA	Bcer	FALSE	NA	NA	NA
Bc_ctg2_156	B2	0.699330786	Bcer	NA	Bcer	FALSE	NA	NA	NA
Bc_ctg2_157	B2	0	Bcer	NA	Bcer	FALSE	NA	NA	NA
Bc_ctg2_158	B2	0.116555131	Bcer	L	Bcer	FALSE	NA	NA	NA
Bc_ctg2_159	B2	0.466220524	Bcer	NA	Bcer	FALSE	NA	NA	NA
Bc_ctg2_16	B2	9.207855349	Bcer	NA	Bcer	FALSE	Bc_Ga0417192_02_rn01	Other	NA
Bc_ctg2_160	B2	0.466220524	Bcer	S	Bcer	FALSE	NA	NA	NA
Bc_ctg2_161	B2	0.233110262	Bcer	L	Bcer	FALSE	NA	NA	NA
Bc_ctg2_162	B2	0	Bcer	S	Bcer	FALSE	NA	NA	NA
Bc_ctg2_166	B2	0	Bcer	NA	Bcer	FALSE	NA	NA	NA
Bc_ctg2_167	B2	0	Bcer	S	Bcer	FALSE	NA	NA	NA
Bc_ctg2_169	B2	40.67774072	Bcer	G	Bcer	FALSE	NA	NA	NA
Bc_ctg2_17	B2	0.466220524	Bcer	V	Bcer	FALSE	NA	NA	NA
Bc_ctg2_170	B2	0.699330786	Bcer	K	Bcer	FALSE	NA	NA	NA
Bc_ctg2_171	B2	1.864882096	Bcer	NA	Bcer	FALSE	NA	NA	NA
Bc_ctg2_172	B2	0.932441048	Bcer	O	Bcer	FALSE	NA	NA	NA
Bc_ctg2_173	B2	5.011870633	Bcer	K	Bcer	FALSE	NA	NA	NA
Bc_ctg2_174	B2	33.21821233	Bcer	V	Bcer	FALSE	NA	NA	NA
Bc_ctg2_176	B2	3.030433406	Bcer	NA	Bcer	FALSE	NA	NA	NA
Bc_ctg2_177	B2	0	Bcer	NA	Bcer	FALSE	NA	NA	NA
Bc_ctg2_178	B2	10.1402964	Bcer	V	Bcer	FALSE	NA	NA	NA
Bc_ctg2_179	B2	0.699330786	Bcer	H	Bcer	FALSE	NA	NA	NA
Bc_ctg2_18	B2	13.28728493	Bcer	V	Bcer	FALSE	NA	NA	NA
Bc_ctg2_180	B2	0.932441048	Bcer	NA	Bcer	FALSE	NA	NA	NA
Bc_ctg2_181	B2	1.048996179	Bcer	S	Bcer	FALSE	NA	NA	NA
Bc_ctg2_182	B2	0.116555131	Bcer	NA	Bcer	FALSE	NA	NA	NA
Bc_ctg2_184	B2	0	Bcer	NA	Bcer	FALSE	NA	NA	NA
Bc_ctg2_185	B2	0.233110262	Bcer	L	Bcer	FALSE	NA	NA	NA
Bc_ctg2_186	B2	0.233110262	Bcer	L	Bcer	FALSE	NA	NA	NA

Bc_ctg2_187	B2	0.233110262	Bcer	S	Bcer	FALSE	NA	NA	NA
Bc_ctg2_188	B2	1.398661572	Bcer	NA	Bcer	FALSE	NA	NA	NA
Bc_ctg2_189	B2	3.146988537	Bcer	L	Bcer	FALSE	NA	NA	NA
Bc_ctg2_19	B2	6.760197598	Bcer	T	Bcer	FALSE	NA	NA	NA
Bc_ctg2_190	B2	1.282106441	Bcer	NA	Bcer	FALSE	NA	NA	NA
Bc_ctg2_191	B2	15.61838755	Bcer	NA	Bcer	FALSE	NA	NA	NA
Bc_ctg2_192	B2	60.37555786	Bcer	S	Bcer	FALSE	NA	NA	NA
Bc_ctg2_193	B2	1.631771834	Bcer	S	Bcer	FALSE	NA	NA	NA
Bc_ctg2_194	B2	0.116555131	Bcer	CO	Bcer	FALSE	NA	NA	NA
Bc_ctg2_195	B2	0.233110262	Bcer	P	Bcer	FALSE	NA	NA	NA
Bc_ctg2_196	B2	1.631771834	Bcer	NA	Bcer	FALSE	NA	NA	NA
Bc_ctg2_197	B2	1.282106441	Bcer	V	Bcer	FALSE	NA	NA	NA
Bc_ctg2_198	B2	0.349665393	Bcer	S	Bcer	FALSE	NA	NA	NA
Bc_ctg2_199	B2	0.349665393	Bcer	NA	Bcer	FALSE	NA	NA	NA
Bc_ctg2_2	B2	0.466220524	Bcer	M	Bcer	FALSE	Bc_Ga0417192_02_rn01	Core biosynthesis	None
Bc_ctg2_20	B2	13.05417467	Bcer	T	Bcer	FALSE	NA	NA	NA
Bc_ctg2_200	B2	2.214547489	Bcer	NA	Bcer	FALSE	NA	NA	NA
Bc_ctg2_201	B2	3.146988537	Bcer	M	Bcer	FALSE	NA	NA	NA
Bc_ctg2_202	B2	2.564212882	Bcer	NA	Bcer	FALSE	NA	NA	NA
Bc_ctg2_203	B2	0.349665393	Bcer	U	Bcer	FALSE	NA	NA	NA
Bc_ctg2_204	B2	0.116555131	Bcer	NA	Bcer	FALSE	NA	NA	NA
Bc_ctg2_205	B2	0	Bcer	NA	Bcer	FALSE	NA	NA	NA
Bc_ctg2_206	B2	0.699330786	Bcer	NA	Bcer	FALSE	NA	NA	NA
Bc_ctg2_207	B2	0	Bcer	NA	Bcer	FALSE	NA	NA	NA
Bc_ctg2_208	B2	0.233110262	Bcer	NA	Bcer	FALSE	NA	NA	NA
Bc_ctg2_209	B2	0	Bcer	NA	Bcer	FALSE	NA	NA	NA
Bc_ctg2_21	B2	5.711201419	Bcer	T	Bcer	FALSE	NA	NA	NA
Bc_ctg2_210	B2	0	Bcer	L	Bcer	FALSE	NA	NA	NA
Bc_ctg2_211	B2	0.233110262	Bcer	NA	Bcer	FALSE	NA	NA	NA
Bc_ctg2_212	B2	0.815885917	Bcer	S	Bcer	FALSE	NA	NA	NA
Bc_ctg2_213	B2	0	Bcer	NA	Bcer	FALSE	NA	NA	NA
Bc_ctg2_214	B2	40.91085098	Bcer	S	Bcer	FALSE	NA	NA	NA
Bc_ctg2_215	B2	3.613209061	Bcer	M	Bcer	FALSE	NA	NA	NA
Bc_ctg2_216	B2	0.233110262	Bcer	S	Bcer	FALSE	NA	NA	NA
Bc_ctg2_217	B2	4.079429585	Bcer	S	Bcer	FALSE	NA	NA	NA
Bc_ctg2_218	B2	0	Bcer	S	Bcer	FALSE	NA	NA	NA
Bc_ctg2_22	B2	4.079429585	Bcer	T	Bcer	FALSE	NA	NA	NA
Bc_ctg2_220	B2	0.233110262	Bcer	NA	Bcer	FALSE	NA	NA	NA
Bc_ctg2_221	B2	0.582775655	Bcer	NA	Bcer	FALSE	NA	NA	NA
Bc_ctg2_223	B2	0.815885917	Bcer	NA	Bcer	FALSE	NA	NA	NA
Bc_ctg2_224	B2	1.398661572	Bcer	NA	Bcer	FALSE	NA	NA	NA
Bc_ctg2_225	B2	0.699330786	Bcer	NA	Bcer	FALSE	NA	NA	NA
Bc_ctg2_226	B2	0.699330786	Bcer	NA	Bcer	FALSE	NA	NA	NA
Bc_ctg2_227	B2	0	Bcer	NA	Bcer	FALSE	NA	NA	NA
Bc_ctg2_228	B2	0.233110262	Bcer	NA	Bcer	FALSE	NA	NA	NA
Bc_ctg2_229	B2	0.116555131	Bcer	NA	Bcer	FALSE	NA	NA	NA
Bc_ctg2_23	B2	1.16555131	Bcer	S	Bcer	FALSE	NA	NA	NA
Bc_ctg2_230	B2	0.699330786	Bcer	NA	Bcer	FALSE	NA	NA	NA
Bc_ctg2_231	B2	0.932441048	Bcer	NA	Bcer	FALSE	NA	NA	NA
Bc_ctg2_232	B2	1.048996179	Bcer	U	Bcer	FALSE	NA	NA	NA
Bc_ctg2_233	B2	0.582775655	Bcer	D	Bcer	FALSE	NA	NA	NA
Bc_ctg2_234	B2	0	Bcer	U	Bcer	FALSE	NA	NA	NA
Bc_ctg2_235	B2	0	Bcer	NA	Bcer	FALSE	NA	NA	NA
Bc_ctg2_236	B2	2.097992358	Bcer	M	Bcer	FALSE	NA	NA	NA
Bc_ctg2_237	B2	42.19295742	Bcer	GM	Bcer	FALSE	NA	NA	NA
Bc_ctg2_238	B2	0.582775655	Bcer	NA	Bcer	FALSE	NA	NA	NA
Bc_ctg2_239	B2	0.349665393	Bcer	NA	Bcer	FALSE	NA	NA	NA
Bc_ctg2_24	B2	6.177421943	Bcer	V	Bcer	FALSE	NA	NA	NA
Bc_ctg2_240	B2	2.214547489	Bcer	D	Bcer	FALSE	NA	NA	NA
Bc_ctg2_241	B2	0.932441048	Bcer	NA	Bcer	FALSE	NA	NA	NA
Bc_ctg2_242	B2	0	Bcer	NA	Bcer	FALSE	NA	NA	NA
Bc_ctg2_243	B2	73.19662227	Bcer	NA	Bcer	FALSE	NA	NA	NA

Bc_ctg2_244	B2	143.1297009	Bcer	D	Bcer	FALSE	NA	NA	NA
Bc_ctg2_245	B2	23.89380185	Bcer	NA	Bcer	FALSE	NA	NA	NA
Bc_ctg2_246	B2	11.88862336	Bcer	L	Bcer	FALSE	NA	NA	NA
Bc_ctg2_247	B2	13.75350546	Bcer	NA	Bcer	FALSE	NA	NA	NA
Bc_ctg2_248	B2	0.466220524	Bcer	NA	Bcer	FALSE	NA	NA	NA
Bc_ctg2_249	B2	14.80250164	Bcer	U	Bcer	FALSE	NA	NA	NA
Bc_ctg2_25	B2	33.33476747	Bcer	V	Bcer	FALSE	NA	NA	NA
Bc_ctg2_250	B2	2.214547489	Bcer	J	Bcer	FALSE	NA	NA	NA
Bc_ctg2_251	B2	3.613209061	Bcer	NA	Bcer	FALSE	NA	NA	NA
Bc_ctg2_252	B2	11.30584771	Bcer	NA	Bcer	FALSE	NA	NA	NA
Bc_ctg2_253	B2	0.116555131	Bcer	NA	Bcer	FALSE	NA	NA	NA
Bc_ctg2_254	B2	1.16555131	Bcer	NA	Bcer	FALSE	NA	NA	NA
Bc_ctg2_255	B2	43.82472925	Bcer	S	Bcer	FALSE	NA	NA	NA
Bc_ctg2_256	B2	0.815885917	Bcer	NA	Bcer	FALSE	NA	NA	NA
Bc_ctg2_257	B2	0	Bcer	NA	Bcer	FALSE	NA	NA	NA
Bc_ctg2_259	B2	0	Bcer	NA	Bcer	FALSE	NA	NA	NA
Bc_ctg2_26	B2	12.00517849	Bcer	NA	Bcer	FALSE	NA	NA	NA
Bc_ctg2_260	B2	7.459528384	Bcer	NA	Bcer	FALSE	NA	NA	NA
Bc_ctg2_261	B2	7.226418122	Bcer	NA	Bcer	FALSE	NA	NA	NA
Bc_ctg2_262	B2	53.26569487	Bcer	K	Bcer	FALSE	NA	NA	NA
Bc_ctg2_263	B2	14.80250164	Bcer	S	Bcer	FALSE	NA	NA	NA
Bc_ctg2_264	B2	389.1775824	Bcer	S	Bcer	FALSE	NA	NA	NA
Bc_ctg2_265	B2	668.3271211	Bcer	E	Bcer	FALSE	NA	NA	NA
Bc_ctg2_266	B2	700.6128924	Bcer	E	Bcer	FALSE	NA	NA	NA
Bc_ctg2_267	B2	0.116555131	Bcer	NA	Bcer	FALSE	NA	NA	NA
Bc_ctg2_268	B2	0.233110262	Bcer	L	Bcer	FALSE	NA	NA	NA
Bc_ctg2_269	B2	0	Bcer	NA	Bcer	FALSE	NA	NA	NA
Bc_ctg2_27	B2	0.582775655	Bcer	NA	Bcer	FALSE	NA	NA	NA
Bc_ctg2_270	B2	48.72004476	Bcer	S	Bcer	FALSE	NA	NA	NA
Bc_ctg2_271	B2	25.29246343	Bcer	S	Bcer	FALSE	NA	NA	NA
Bc_ctg2_272	B2	7.459528384	Bcer	EH	Bcer	FALSE	NA	NA	NA
Bc_ctg2_273	B2	4.312539847	Bcer	NA	Bcer	FALSE	NA	NA	NA
Bc_ctg2_275	B2	21.56269923	Bcer	S	Bcer	FALSE	NA	NA	NA
Bc_ctg2_276	B2	5.244980895	Bcer	L	Bcer	FALSE	NA	NA	NA
Bc_ctg2_278	B2	29.48844814	Bcer	NA	Bcer	FALSE	NA	NA	NA
Bc_ctg2_279	B2	94.99243176	Bcer	K	Bcer	FALSE	NA	NA	NA
Bc_ctg2_28	B2	4.195984716	Bcer	EGP	Bcer	FALSE	NA	NA	NA
Bc_ctg2_280	B2	1.282106441	Bcer	S	Bcer	FALSE	NA	NA	NA
Bc_ctg2_281	B2	12.00517849	Bcer	O	Bcer	FALSE	NA	NA	NA
Bc_ctg2_282	B2	0.815885917	Bcer	S	Bcer	FALSE	NA	NA	NA
Bc_ctg2_283	B2	26.92423526	Bcer	S	Bcer	FALSE	NA	NA	NA
Bc_ctg2_284	B2	46.38894214	Bcer	S	Bcer	FALSE	NA	NA	NA
Bc_ctg2_285	B2	1.16555131	Bcer	NA	Bcer	FALSE	NA	NA	NA
Bc_ctg2_286	B2	2.680768013	Bcer	S	Bcer	FALSE	NA	NA	NA
Bc_ctg2_287	B2	9.440965611	Bcer	NT	Bcer	FALSE	NA	NA	NA
Bc_ctg2_288	B2	0.699330786	Bcer	S	Bcer	FALSE	NA	NA	NA
Bc_ctg2_289	B2	72.84695687	Bcer	NA	Bcer	FALSE	NA	NA	NA
Bc_ctg2_29	B2	0.349665393	Bcer	M	Bcer	FALSE	NA	NA	NA
Bc_ctg2_290	B2	0	Bcer	NA	Bcer	FALSE	NA	NA	NA
Bc_ctg2_291	B2	13.5203952	Bcer	K	Bcer	FALSE	NA	NA	NA
Bc_ctg2_292	B2	1.282106441	Bcer	S	Bcer	FALSE	NA	NA	NA
Bc_ctg2_293	B2	3.263543668	Bcer	O	Bcer	FALSE	NA	NA	NA
Bc_ctg2_294	B2	1.048996179	Bcer	S	Bcer	FALSE	NA	NA	NA
Bc_ctg2_295	B2	2.33110262	Bcer	S	Bcer	FALSE	NA	NA	NA
Bc_ctg2_296	B2	0.233110262	Bcer	NA	Bcer	FALSE	NA	NA	NA
Bc_ctg2_297	B2	4.312539847	Bcer	V	Bcer	FALSE	NA	NA	NA
Bc_ctg2_298	B2	3.146988537	Bcer	NA	Bcer	FALSE	NA	NA	NA
Bc_ctg2_299	B2	0.815885917	Bcer	S	Bcer	FALSE	NA	NA	NA
Bc_ctg2_3	B2	0.116555131	Bcer	M	Bcer	FALSE	Bc_Ga0417192_02_rn01	Core biosynthesis	SMCOG1010
Bc_ctg2_30	B2	2.797323144	Bcer	S	Bcer	FALSE	NA	NA	NA
Bc_ctg2_300	B2	16.43427347	Bcer	L	Bcer	FALSE	NA	NA	NA
Bc_ctg2_303	B2	3.146988537	Bcer	O	Bcer	FALSE	NA	NA	NA

Bc_ctg2_304	B2	0	Bcer	NA	Bcer	FALSE	NA	NA	NA	
Bc_ctg2_305	B2	1.048996179	Bcer	DZ	Bcer	FALSE	NA	NA	NA	
Bc_ctg2_307	B2	0.466220524	Bcer	O	Bcer	FALSE	NA	NA	NA	
Bc_ctg2_309	B2	0	Bcer	NA	Bcer	FALSE	NA	NA	NA	
Bc_ctg2_31	B2	2.214547489	Bcer	NA	Bcer	FALSE	NA	NA	NA	
Bc_ctg2_310	B2	1.981437227	Bcer	GM	Bcer	FALSE	NA	NA	NA	
Bc_ctg2_311	B2	4.778760371	Bcer	E	Bcer	FALSE	NA	NA	NA	
Bc_ctg2_312	B2	7.109862991	Bcer	K	Bcer	FALSE	NA	NA	NA	
Bc_ctg2_313	B2	0	Bcer	K	Bcer	FALSE	NA	NA	NA	
Bc_ctg2_314	B2	1.981437227	Bcer	L	Bcer	FALSE	NA	NA	NA	
Bc_ctg2_315	B2	0.582775655	Bcer	S	Bcer	FALSE	NA	NA	NA	
Bc_ctg2_316	B2	530.325846	Bcer	M	Bcer	FALSE	NA	NA	NA	
Bc_ctg2_317	B2	55.48024235	Bcer	M	Bcer	FALSE	NA	NA	NA	
Bc_ctg2_318	B2	77.85882751	Bcer	S	Bcer	FALSE	NA	NA	NA	
Bc_ctg2_319	B2	3.030433406	Bcer	S	Bcer	FALSE	NA	NA	NA	
Bc_ctg2_32	B2	0.116555131	Bcer	C	Bcer	FALSE	NA	NA	NA	
Bc_ctg2_320	B2	93.2441048	Bcer	S	Bcer	FALSE	NA	NA	NA	
Bc_ctg2_322	B2	0	Bcer	NA	Bcer	FALSE	NA	NA	NA	
Bc_ctg2_323	B2	17.13360426	Bcer	NA	Bcer	FALSE	NA	NA	NA	
Bc_ctg2_324	B2	0	Bcer	NA	Bcer	FALSE	NA	NA	NA	
Bc_ctg2_325	B2	0	Bcer	NA	Bcer	FALSE	NA	NA	NA	
Bc_ctg2_326	B2	0	Bcer	L	Bcer	FALSE	NA	NA	NA	
Bc_ctg2_327	B2	0.116555131	Bcer	NA	Bcer	FALSE	NA	NA	NA	
Bc_ctg2_328	B2	0.699330786	Bcer	H	Bcer	FALSE	NA	NA	NA	
Bc_ctg2_329	B2	0.349665393	Bcer	NA	Bcer	FALSE	NA	NA	NA	
Bc_ctg2_33	B2	2.913878275	Bcer	IQ	Bcer	TRUE	NA	NA	NA	
Bc_ctg2_330	B2	0	Bcer	T	Bcer	FALSE	NA	NA	NA	
Bc_ctg2_331	B2	3.146988537	Bcer	T	Bcer	FALSE	NA	NA	NA	
Bc_ctg2_332	B2	36.36520087	Bcer	M	Bcer	FALSE	NA	NA	NA	
Bc_ctg2_333	B2	8.042304039	Bcer	K	Bcer	FALSE	NA	NA	NA	
Bc_ctg2_334	B2	0	Bcer	E	Bcer	FALSE	NA	NA	NA	
Bc_ctg2_335	B2	1.631771834	Bcer	NA	Bcer	FALSE	NA	NA	NA	
Bc_ctg2_336	B2	10.37340666	Bcer	G	Bcer	FALSE	NA	NA	NA	
Bc_ctg2_337	B2	4.429094978	Bcer	K	Bcer	FALSE	NA	NA	NA	
Bc_ctg2_338	B2	6.99330786	Bcer	E	Bcer	FALSE	NA	NA	NA	
Bc_ctg2_339	B2	0.233110262	Bcer	K	Bcer	FALSE	NA	NA	NA	
Bc_ctg2_34	B2	0.582775655	Bcer	S	Bcer	FALSE	NA	NA	NA	
Bc_ctg2_340	B2	1.048996179	Bcer	S	Bcer	FALSE	NA	NA	NA	
Bc_ctg2_341	B2	0	Bcer	K	Bcer	FALSE	NA	NA	NA	
Bc_ctg2_342	B2	2.097992358	Bcer	E	Bcer	FALSE	NA	NA	NA	
Bc_ctg2_343	B2	0.815885917	Bcer	K	Bcer	FALSE	NA	NA	NA	
Bc_ctg2_344	B2	8.625079694	Bcer	S	Bcer	FALSE	NA	NA	NA	
Bc_ctg2_345	B2	30.18777893	Bcer	K	Bcer	FALSE	NA	NA	NA	
Bc_ctg2_346	B2	1.515216703	Bcer	L	Bcer	FALSE	NA	NA	NA	
Bc_ctg2_347	B2	6.527087336	Bcer	L	Bcer	FALSE	NA	NA	NA	
Bc_ctg2_348	B2	2.33110262	Bcer	NA	Bcer	FALSE	NA	NA	NA	
Bc_ctg2_349	B2	16.90049399	Bcer	L	Bcer	FALSE	NA	NA	NA	
Bc_ctg2_35	B2	0.116555131	Bcer	NA	Bcer	FALSE	NA	NA	NA	
Bc_ctg2_350	B2	6.410532205	Bcer	L	Bcer	FALSE	NA	NA	NA	
Bc_ctg2_351	B2	0.582775655	Bcer	K	Bcer	FALSE	NA	NA	NA	
Bc_ctg2_352	B2	3.263543668	Bcer	NA	Bcer	FALSE	NA	NA	NA	
Bc_ctg2_353	B2	16.90049399	Bcer	NA	Bcer	FALSE	NA	NA	NA	
Bc_ctg2_354	B2	10.72307205	Bcer	S	Bcer	FALSE	NA	NA	NA	
Bc_ctg2_355	B2	11.18929258	Bcer	NA	Bcer	FALSE	NA	NA	NA	
Bc_ctg2_356	B2	1.398661572	Bcer	NA	Bcer	FALSE	NA	NA	NA	
Bc_ctg2_359	B2	4.429094978	Bcer	CH	Bcer	FALSE	NA	NA	NA	
Bc_ctg2_36	B2	0	Bcer	NA	Bcer	FALSE	NA	NA	NA	
Bc_ctg2_360	B2	10.83962718	Bcer	L	Bcer	FALSE	NA	NA	NA	
Bc_ctg2_361	B2	2.097992358	Bcer	NA	Bcer	FALSE	NA	NA	NA	
Bc_ctg2_362	B2	7.109862991	Bcer	KT	Bcer	FALSE	NA	NA	NA	
Bc_ctg2_363	B2	21.4461441	Bcer	K	Bcer	FALSE	NA	NA	NA	
Bc_ctg2_364	B2	9.207855349	Bcer	P	Bcer	FALSE	NA	NA	NA	

Bc_ctg2_365	B2	1.398661572	Bcer	NA	Bcer	FALSE	NA	NA	NA
Bc_ctg2_366	B2	12.00517849	Bcer	S	Bcer	FALSE	NA	NA	NA
Bc_ctg2_367	B2	6.293977074	Bcer	L	Bcer	FALSE	NA	NA	NA
Bc_ctg2_368	B2	15.96805295	Bcer	S	Bcer	FALSE	NA	NA	NA
Bc_ctg2_369	B2	0.466220524	Bcer	NA	Bcer	FALSE	NA	NA	NA
Bc_ctg2_37	B2	0.582775655	Bcer	NA	Bcer	FALSE	NA	NA	NA
Bc_ctg2_370	B2	1.981437227	Bcer	P	Bcer	FALSE	NA	NA	NA
Bc_ctg2_371	B2	0.699330786	Bcer	NA	Bcer	FALSE	NA	NA	NA
Bc_ctg2_372	B2	19.34815175	Bcer	S	Bcer	FALSE	NA	NA	NA
Bc_ctg2_373	B2	9.907186135	Bcer	NA	Bcer	FALSE	NA	NA	NA
Bc_ctg2_374	B2	0	Bcer	NA	Bcer	FALSE	NA	NA	NA
Bc_ctg2_375	B2	3.380098799	Bcer	NA	Bcer	FALSE	NA	NA	NA
Bc_ctg2_376	B2	67.36886572	Bcer	LO	Bcer	FALSE	NA	NA	NA
Bc_ctg2_377	B2	30.18777893	Bcer	L	Bcer	FALSE	NA	NA	NA
Bc_ctg2_378	B2	43.12539847	Bcer	V	Bcer	FALSE	NA	NA	NA
Bc_ctg2_379	B2	26.92423526	Bcer	V	Bcer	FALSE	NA	NA	NA
Bc_ctg2_38	B2	5.594646288	Bcer	NA	Bcer	FALSE	NA	NA	NA
Bc_ctg2_380	B2	10.72307205	Bcer	NA	Bcer	FALSE	NA	NA	NA
Bc_ctg2_381	B2	36.36520087	Bcer	NA	Bcer	FALSE	NA	NA	NA
Bc_ctg2_382	B2	1.282106441	Bcer	NA	Bcer	FALSE	NA	NA	NA
Bc_ctg2_383	B2	0.233110262	Bcer	NA	Bcer	FALSE	NA	NA	NA
Bc_ctg2_384	B2	141.6144842	Bcer	D	Bcer	FALSE	NA	NA	NA
Bc_ctg2_385	B2	25.05935316	Bcer	NA	Bcer	FALSE	NA	NA	NA
Bc_ctg2_386	B2	149.5402331	Bcer	NA	Bcer	FALSE	NA	NA	NA
Bc_ctg2_387	B2	2.33110262	Bcer	NA	Bcer	FALSE	NA	NA	NA
Bc_ctg2_388	B2	5.594646288	Bcer	NA	Bcer	FALSE	NA	NA	NA
Bc_ctg2_389	B2	28.55600709	Bcer	NA	Bcer	FALSE	Bc_Ga0417192_02_rn03	Other	NA
Bc_ctg2_39	B2	39.27907915	Bcer	NA	Bcer	FALSE	NA	NA	NA
Bc_ctg2_390	B2	57.69478984	Bcer	NA	Bcer	FALSE	Bc_Ga0417192_02_rn03	Other	NA
Bc_ctg2_391	B2	181.8260044	Bcer	NA	Bcer	FALSE	Bc_Ga0417192_02_rn03	Other	NA
Bc_ctg2_392	B2	116.3220207	Bcer	T	Bcer	FALSE	Bc_Ga0417192_02_rn03	Other	NA
Bc_ctg2_393	B2	43.94128439	Bcer	NA	Bcer	FALSE	Bc_Ga0417192_02_rn03	Other	NA
Bc_ctg2_394	B2	113.4081425	Bcer	L	Bcer	FALSE	Bc_Ga0417192_02_rn03	Other	SMCOG1077
Bc_ctg2_395	B2	9.674075873	Bcer	K	Bcer	FALSE	Bc_Ga0417192_02_rn03	Other	SMCOG1148
Bc_ctg2_396	B2	0.233110262	Bcer	K	Bcer	FALSE	Bc_Ga0417192_02_rn03	Other	NA
Bc_ctg2_397	B2	0.466220524	Bcer	NA	Bcer	FALSE	Bc_Ga0417192_02_rn03	Other	NA
Bc_ctg2_398	B2	32.86854694	Bcer	EGP	Bcer	FALSE	Bc_Ga0417192_02_rn03	Transport	SMCOG1005
Bc_ctg2_399	B2	26.10834934	Bcer	UW	Bcer	FALSE	Bc_Ga0417192_02_rn03	Other	NA
Bc_ctg2_4	B2	1.515216703	Bcer	M	Bcer	FALSE	Bc_Ga0417192_02_rn01	Core biosynthesis	SMCOG1010
Bc_ctg2_40	B2	37.18108679	Bcer	L	Bcer	FALSE	NA	NA	NA
Bc_ctg2_400	B2	0	Bcer	NA	Bcer	FALSE	Bc_Ga0417192_02_rn03	Other	NA
Bc_ctg2_401	B2	2.097992358	Bcer	S	Bcer	FALSE	Bc_Ga0417192_02_rn03	Other	NA
Bc_ctg2_402	B2	0.815885917	Bcer	NA	Bcer	FALSE	Bc_Ga0417192_02_rn03	Other	NA
Bc_ctg2_403	B2	46.27238701	Bcer	NA	Bcer	FALSE	Bc_Ga0417192_02_rn03	Other	NA
Bc_ctg2_404	B2	1.16555131	Bcer	CO	Bcer	FALSE	Bc_Ga0417192_02_rn03	Other	NA
Bc_ctg2_405	B2	14.21972598	Bcer	NA	Bcer	FALSE	Bc_Ga0417192_02_rn03	Other	NA
Bc_ctg2_406	B2	25.64212882	Bcer	E	Bcer	FALSE	Bc_Ga0417192_02_rn03	Ancillary biosynthesis	SMCOG1180
Bc_ctg2_407	B2	0.233110262	Bcer	C	Bcer	FALSE	Bc_Ga0417192_02_rn03	Ancillary biosynthesis	SMCOG1039
Bc_ctg2_408	B2	182.7584454	Bcer	Q	Bcer	TRUE	Bc_Ga0417192_02_rn03	Core biosynthesis	SMCOG1127
Bc_ctg2_409	B2	0.233110262	Bcer	NA	Bcer	FALSE	Bc_Ga0417192_02_rn03	Other	NA
Bc_ctg2_41	B2	0	Bcer	NA	Bcer	FALSE	NA	NA	NA
Bc_ctg2_410	B2	4.195984716	Bcer	NA	Bcer	FALSE	Bc_Ga0417192_02_rn03	Other	NA
Bc_ctg2_411	B2	13.40384006	Bcer	NA	Bcer	FALSE	Bc_Ga0417192_02_rn03	Other	NA
Bc_ctg2_412	B2	27.04079039	Bcer	NA	Bcer	FALSE	Bc_Ga0417192_02_rn03	Other	NA
Bc_ctg2_413	B2	7.109862991	Bcer	NA	Bcer	FALSE	Bc_Ga0417192_02_rn03	Other	NA
Bc_ctg2_414	B2	0.349665393	Bcer	L	Bcer	FALSE	Bc_Ga0417192_02_rn03	Other	NA
Bc_ctg2_415	B2	1.048996179	Bcer	L	Bcer	FALSE	Bc_Ga0417192_02_rn03	Other	NA
Bc_ctg2_416	B2	4.195984716	Bcer	G	Bcer	FALSE	Bc_Ga0417192_02_rn03	Ancillary biosynthesis	SMCOG1101
Bc_ctg2_417	B2	0.466220524	Bcer	NA	Bcer	FALSE	Bc_Ga0417192_02_rn03	Other	NA
Bc_ctg2_418	B2	5.244980895	Bcer	NA	Bcer	FALSE	Bc_Ga0417192_02_rn03	Other	NA
Bc_ctg2_419	B2	3.613209061	Bcer	ST	Bcer	FALSE	Bc_Ga0417192_02_rn03	Other	NA
Bc_ctg2_42	B2	0	Bcer	NA	Bcer	FALSE	NA	NA	NA

Bc_ctg2_420	B2	19.9309274	Bcer	M	Bcer	FALSE	Bc_Ga0417192_02_rn03	Other	NA
Bc_ctg2_421	B2	0.815885917	Bcer	S	Bcer	FALSE	Bc_Ga0417192_02_rn03	Other	NA
Bc_ctg2_422	B2	12.93761954	Bcer	Q	Bcer	TRUE	Bc_Ga0417192_02_rn03	Core biosynthesis	SMCOG1022
Bc_ctg2_423	B2	22.14547489	Bcer	Q	Bcer	TRUE	Bc_Ga0417192_02_rn03	Core biosynthesis	SMCOG1127
Bc_ctg2_424	B2	13.28728493	Bcer	Q	Bcer	TRUE	Bc_Ga0417192_02_rn03	Core biosynthesis	SMCOG1127
Bc_ctg2_425	B2	0.582775655	Bcer	IQ	Bcer	TRUE	Bc_Ga0417192_02_rn03	Ancillary biosynthesis	SMCOG1147
Bc_ctg2_426	B2	13.75350546	Bcer	I	Bcer	FALSE	Bc_Ga0417192_02_rn03	Ancillary biosynthesis	SMCOG1006
Bc_ctg2_427	B2	16.78393886	Bcer	J	Bcer	FALSE	Bc_Ga0417192_02_rn03	Other	NA
Bc_ctg2_428	B2	13.87006059	Bcer	I	Bcer	FALSE	Bc_Ga0417192_02_rn03	Ancillary biosynthesis	SMCOG1021
Bc_ctg2_429	B2	7.342973253	Bcer	I	Bcer	FALSE	Bc_Ga0417192_02_rn03	Ancillary biosynthesis	SMCOG1095
Bc_ctg2_430	B2	0.349665393	Bcer	IQ	Bcer	TRUE	Bc_Ga0417192_02_rn03	Ancillary biosynthesis	SMCOG1147
Bc_ctg2_431	B2	3.613209061	Bcer	I	Bcer	FALSE	Bc_Ga0417192_02_rn03	Ancillary biosynthesis	SMCOG1006
Bc_ctg2_432	B2	5.82775655	Bcer	Q	Bcer	TRUE	Bc_Ga0417192_02_rn03	Core biosynthesis	SMCOG1002
Bc_ctg2_433	B2	50.35181659	Bcer	Q	Bcer	TRUE	Bc_Ga0417192_02_rn03	Core biosynthesis	SMCOG1127
Bc_ctg2_434	B2	9.091300218	Bcer	C	Bcer	FALSE	Bc_Ga0417192_02_rn03	Ancillary biosynthesis	SMCOG1083
Bc_ctg2_435	B2	16.43427347	Bcer	V	Bcer	FALSE	Bc_Ga0417192_02_rn03	Ancillary biosynthesis	SMCOG1053
Bc_ctg2_436	B2	3.146988537	Bcer	Q	Bcer	TRUE	Bc_Ga0417192_02_rn03	Ancillary biosynthesis	SMCOG1256
Bc_ctg2_437	B2	14.45283624	Bcer	Q	Bcer	TRUE	Bc_Ga0417192_02_rn03	Core biosynthesis	SMCOG1002
Bc_ctg2_438	B2	3.613209061	Bcer	Q	Bcer	TRUE	Bc_Ga0417192_02_rn03	Ancillary biosynthesis	SMCOG1004
Bc_ctg2_439	B2	33.4513226	Bcer	Q	Bcer	TRUE	Bc_Ga0417192_02_rn03	Core biosynthesis	SMCOG1127
Bc_ctg2_440	B2	2.447657751	Bcer	H	Bcer	FALSE	Bc_Ga0417192_02_rn03	Ancillary biosynthesis	SMCOG1012
Bc_ctg2_441	B2	17.36671452	Bcer	K	Bcer	FALSE	Bc_Ga0417192_02_rn03	Regulation	SMCOG1078
Bc_ctg2_442	B2	6.760197598	Bcer	E	Bcer	FALSE	Bc_Ga0417192_02_rn03	Ancillary biosynthesis	SMCOG1056
Bc_ctg2_443	B2	4.778760371	Bcer	S	Bcer	FALSE	Bc_Ga0417192_02_rn03	Ancillary biosynthesis	SMCOG1179
Bc_ctg2_444	B2	6.527087336	Bcer	S	Bcer	FALSE	Bc_Ga0417192_02_rn03	Ancillary biosynthesis	SMCOG1079
Bc_ctg2_445	B2	1.981437227	Bcer	G	Bcer	FALSE	Bc_Ga0417192_02_rn03	Other	NA
Bc_ctg2_446	B2	3.030433406	Bcer	E	Bcer	FALSE	Bc_Ga0417192_02_rn03	Ancillary biosynthesis	SMCOG1114
Bc_ctg2_447	B2	1.864882096	Bcer	E	Bcer	FALSE	Bc_Ga0417192_02_rn03	Ancillary biosynthesis	SMCOG1081
Bc_ctg2_448	B2	3.729764192	Bcer	E	Bcer	FALSE	Bc_Ga0417192_02_rn03	Ancillary biosynthesis	SMCOG1158
Bc_ctg2_449	B2	3.846319323	Bcer	K	Bcer	FALSE	Bc_Ga0417192_02_rn03	Other	NA
Bc_ctg2_45	B2	0	Bcer	NA	Bcer	FALSE	NA	NA	NA
Bc_ctg2_450	B2	0.932441048	Bcer	NA	Bcer	FALSE	Bc_Ga0417192_02_rn03	Other	NA
Bc_ctg2_451	B2	36.36520087	Bcer	NA	Bcer	FALSE	Bc_Ga0417192_02_rn03	Other	NA
Bc_ctg2_453	B2	0.466220524	Bcer	NA	Bcer	FALSE	Bc_Ga0417192_02_rn03	Other	NA
Bc_ctg2_454	B2	0	Bcer	L	Bcer	FALSE	Bc_Ga0417192_02_rn03	Other	NA
Bc_ctg2_455	B2	0	Bcer	NA	Bcer	FALSE	Bc_Ga0417192_02_rn03	Other	NA
Bc_ctg2_456	B2	0.582775655	Bcer	NA	Bcer	FALSE	Bc_Ga0417192_02_rn03	Other	NA
Bc_ctg2_457	B2	7.459528384	Bcer	NA	Bcer	FALSE	Bc_Ga0417192_02_rn03	Other	NA
Bc_ctg2_458	B2	25.75868395	Bcer	S	Bcer	FALSE	Bc_Ga0417192_02_rn03	Other	NA
Bc_ctg2_459	B2	0	Bcer	E	Bcer	FALSE	Bc_Ga0417192_02_rn03	Other	NA
Bc_ctg2_46	B2	0	Bcer	NA	Bcer	FALSE	NA	NA	NA
Bc_ctg2_460	B2	0	Bcer	G	Bcer	FALSE	Bc_Ga0417192_02_rn03	Ancillary biosynthesis	SMCOG1101
Bc_ctg2_462	B2	0.349665393	Bcer	A	Bcer	FALSE	Bc_Ga0417192_02_rn03	Other	NA
Bc_ctg2_464	B2	0	Bcer	NA	Bcer	FALSE	NA	NA	NA
Bc_ctg2_465	B2	3.49665393	Bcer	NA	Bcer	FALSE	NA	NA	NA
Bc_ctg2_466	B2	3.030433406	Bcer	NA	Bcer	FALSE	NA	NA	NA
Bc_ctg2_467	B2	4.895315502	Bcer	NA	Bcer	FALSE	NA	NA	NA
Bc_ctg2_468	B2	0	Bcer	NA	Bcer	FALSE	NA	NA	NA
Bc_ctg2_469	B2	0.699330786	Bcer	L	Bcer	FALSE	NA	NA	NA
Bc_ctg2_47	B2	4.079429585	Bcer	V	Bcer	FALSE	NA	NA	NA
Bc_ctg2_471	B2	8.741634825	Bcer	NA	Bcer	FALSE	NA	NA	NA
Bc_ctg2_472	B2	3.380098799	Bcer	S	Bcer	FALSE	NA	NA	NA
Bc_ctg2_473	B2	29.7215584	Bcer	S	Bcer	FALSE	NA	NA	NA
Bc_ctg2_474	B2	8.15885917	Bcer	NA	Bcer	FALSE	NA	NA	NA
Bc_ctg2_475	B2	0	Bcer	L	Bcer	FALSE	NA	NA	NA
Bc_ctg2_476	B2	6.760197598	Bcer	L	Bcer	FALSE	NA	NA	NA
Bc_ctg2_477	B2	0	Bcer	NA	Bcer	FALSE	NA	NA	NA
Bc_ctg2_478	B2	0	Bcer	L	Bcer	FALSE	NA	NA	NA
Bc_ctg2_479	B2	0.233110262	Bcer	L	Bcer	FALSE	NA	NA	NA
Bc_ctg2_48	B2	7.342973253	Bcer	M	Bcer	FALSE	NA	NA	NA
Bc_ctg2_480	B2	0.699330786	Bcer	L	Bcer	FALSE	NA	NA	NA
Bc_ctg2_481	B2	0.582775655	Bcer	L	Bcer	FALSE	NA	NA	NA

Bc_ctg2_482	B2	0.466220524	Bcer	NA	Bcer	FALSE	NA	NA	NA
Bc_ctg2_483	B2	1.16555131	Bcer	NA	Bcer	FALSE	NA	NA	NA
Bc_ctg2_484	B2	1.16555131	Bcer	NA	Bcer	FALSE	NA	NA	NA
Bc_ctg2_485	B2	1.282106441	Bcer	O	Bcer	FALSE	NA	NA	NA
Bc_ctg2_486	B2	0.699330786	Bcer	K	Bcer	FALSE	NA	NA	NA
Bc_ctg2_487	B2	35.78242522	Bcer	S	Bcer	FALSE	NA	NA	NA
Bc_ctg2_488	B2	0.233110262	Bcer	NA	Bcer	FALSE	NA	NA	NA
Bc_ctg2_489	B2	0	Bcer	NA	Bcer	FALSE	NA	NA	NA
Bc_ctg2_49	B2	1.282106441	Bcer	E	Bcer	FALSE	NA	NA	NA
Bc_ctg2_490	B2	2.680768013	Bcer	S	Bcer	FALSE	NA	NA	NA
Bc_ctg2_491	B2	36.8314214	Bcer	NA	Bcer	FALSE	Bc_Ga0417192_02_rn04	Other	NA
Bc_ctg2_492	B2	4.545650109	Bcer	NA	Bcer	FALSE	Bc_Ga0417192_02_rn04	Other	NA
Bc_ctg2_493	B2	5.478091157	Bcer	NA	Bcer	FALSE	Bc_Ga0417192_02_rn04	Other	NA
Bc_ctg2_494	B2	11.42240284	Bcer	NA	Bcer	FALSE	Bc_Ga0417192_02_rn04	Other	NA
Bc_ctg2_495	B2	64.22187718	Bcer	L	Bcer	FALSE	Bc_Ga0417192_02_rn04	Other	NA
Bc_ctg2_496	B2	117.7206823	Bcer	L	Bcer	FALSE	Bc_Ga0417192_02_rn04	Other	NA
Bc_ctg2_497	B2	69.9330786	Bcer	K	Bcer	FALSE	Bc_Ga0417192_02_rn04	Other	NA
Bc_ctg2_498	B2	25.75868395	Bcer	EGP	Bcer	FALSE	Bc_Ga0417192_02_rn04	Transport	SMCOG1020
Bc_ctg2_499	B2	24.59313264	Bcer	KLT	Bcer	FALSE	Bc_Ga0417192_02_rn04	Core biosynthesis	SMCOG1070
Bc_ctg2_5	B2	1.282106441	Bcer	E	Bcer	FALSE	Bc_Ga0417192_02_rn01	Core biosynthesis	SMCOG1056
Bc_ctg2_50	B2	0	Bcer	NA	Bcer	FALSE	NA	NA	NA
Bc_ctg2_500	B2	3.146988537	Bcer	J	Bcer	FALSE	Bc_Ga0417192_02_rn04	Other	NA
Bc_ctg2_504	B2	0	Bcer	NA	Bcer	FALSE	Bc_Ga0417192_02_rn04	Other	NA
Bc_ctg2_505	B2	72.84695687	Bcer	S	Bcer	FALSE	Bc_Ga0417192_02_rn04	Other	NA
Bc_ctg2_506	B2	22.72825054	Bcer	L	Bcer	FALSE	Bc_Ga0417192_02_rn04	Other	SMCOG1077
Bc_ctg2_507	B2	0.815885917	Bcer	K	Bcer	FALSE	Bc_Ga0417192_02_rn04	Regulation	SMCOG1058
Bc_ctg2_508	B2	16.66738373	Bcer	L	Bcer	FALSE	Bc_Ga0417192_02_rn04	Other	NA
Bc_ctg2_509	B2	1.398661572	Bcer	NA	Bcer	FALSE	Bc_Ga0417192_02_rn04	Other	NA
Bc_ctg2_51	B2	3.263543668	Bcer	L	Bcer	FALSE	NA	NA	NA
Bc_ctg2_510	B2	4.66220524	Bcer	S	Bcer	FALSE	Bc_Ga0417192_02_rn04	Other	NA
Bc_ctg2_511	B2	0.233110262	Bcer	NA	Bcer	FALSE	NA	NA	NA
Bc_ctg2_512	B2	1.631771834	Bcer	NA	Bcer	FALSE	NA	NA	NA
Bc_ctg2_513	B2	1.398661572	Bcer	M	Bcer	FALSE	NA	NA	NA
Bc_ctg2_52	B2	1.748326965	Bcer	M	Bcer	FALSE	NA	NA	NA
Bc_ctg2_53	B2	2.214547489	Bcer	M	Bcer	FALSE	NA	NA	NA
Bc_ctg2_54	B2	0.349665393	Bcer	M	Bcer	FALSE	NA	NA	NA
Bc_ctg2_55	B2	1.515216703	Bcer	NA	Bcer	FALSE	NA	NA	NA
Bc_ctg2_56	B2	0.233110262	Bcer	V	Bcer	FALSE	NA	NA	NA
Bc_ctg2_57	B2	6.060866812	Bcer	CH	Bcer	FALSE	NA	NA	NA
Bc_ctg2_58	B2	3.846319323	Bcer	E	Bcer	FALSE	NA	NA	NA
Bc_ctg2_59	B2	3.729764192	Bcer	S	Bcer	FALSE	NA	NA	NA
Bc_ctg2_6	B2	0.349665393	Bcer	M	Bcer	FALSE	Bc_Ga0417192_02_rn01	Other	NA
Bc_ctg2_60	B2	1.282106441	Bcer	K	Bcer	FALSE	NA	NA	NA
Bc_ctg2_61	B2	11.42240284	Bcer	D	Bcer	FALSE	NA	NA	NA
Bc_ctg2_62	B2	0.815885917	Bcer	NA	Bcer	FALSE	NA	NA	NA
Bc_ctg2_63	B2	3.49665393	Bcer	K	Bcer	FALSE	NA	NA	NA
Bc_ctg2_64	B2	2.797323144	Bcer	E	Bcer	FALSE	NA	NA	NA
Bc_ctg2_65	B2	3.263543668	Bcer	L	Bcer	FALSE	NA	NA	NA
Bc_ctg2_66	B2	25.64212882	Bcer	NA	Bcer	FALSE	NA	NA	NA
Bc_ctg2_67	B2	0.582775655	Bcer	NA	Bcer	FALSE	NA	NA	NA
Bc_ctg2_68	B2	0	Bcer	NA	Bcer	FALSE	NA	NA	NA
Bc_ctg2_69	B2	0	Bcer	NA	Bcer	FALSE	NA	NA	NA
Bc_ctg2_7	B2	0.233110262	Bcer	M	Bcer	FALSE	Bc_Ga0417192_02_rn01	Core biosynthesis	None
Bc_ctg2_70	B2	0	Bcer	M	Bcer	FALSE	NA	NA	NA
Bc_ctg2_71	B2	1.048996179	Bcer	NA	Bcer	FALSE	NA	NA	NA
Bc_ctg2_72	B2	6.410532205	Bcer	NA	Bcer	FALSE	NA	NA	NA
Bc_ctg2_73	B2	21.7958095	Bcer	S	Bcer	FALSE	NA	NA	NA
Bc_ctg2_74	B2	44.64061517	Bcer	S	Bcer	FALSE	NA	NA	NA
Bc_ctg2_75	B2	1.748326965	Bcer	NA	Bcer	FALSE	NA	NA	NA
Bc_ctg2_76	B2	2.913878275	Bcer	NA	Bcer	FALSE	NA	NA	NA
Bc_ctg2_77	B2	17.25015939	Bcer	KT	Bcer	FALSE	NA	NA	NA
Bc_ctg2_78	B2	2.564212882	Bcer	K	Bcer	FALSE	NA	NA	NA

Bc_ctg2_79	B2	1.048996179	Bcer	NA	Bcer	FALSE	NA	NA	NA
Bc_ctg2_8	B2	0	Bcer	J	Bcer	FALSE	Bc_Ga0417192_02_rn01	Other	NA
Bc_ctg2_80	B2	7.226418122	Bcer	K	Bcer	FALSE	NA	NA	NA
Bc_ctg2_81	B2	39.97840993	Bcer	M	Bcer	FALSE	NA	NA	NA
Bc_ctg2_82	B2	56.29612827	Bcer	S	Bcer	FALSE	NA	NA	NA
Bc_ctg2_83	B2	0.466220524	Bcer	K	Bcer	FALSE	NA	NA	NA
Bc_ctg2_84	B2	1.048996179	Bcer	E	Bcer	FALSE	NA	NA	NA
Bc_ctg2_86	B2	2.797323144	Bcer	S	Bcer	FALSE	NA	NA	NA
Bc_ctg2_87	B2	0.466220524	Bcer	K	Bcer	FALSE	NA	NA	NA
Bc_ctg2_88	B2	205.7198062	Bcer	M	Bcer	FALSE	NA	NA	NA
Bc_ctg2_89	B2	7.226418122	Bcer	S	Bcer	FALSE	NA	NA	NA
Bc_ctg2_9	B2	0.815885917	Bcer	M	Bcer	FALSE	Bc_Ga0417192_02_rn01	Other	NA
Bc_ctg2_90	B2	3.49665393	Bcer	NA	Bcer	FALSE	NA	NA	NA
Bc_ctg2_91	B2	7.226418122	Bcer	V	Bcer	FALSE	NA	NA	NA
Bc_ctg2_93	B2	4.66220524	Bcer	I	Bcer	FALSE	NA	NA	NA
Bc_ctg2_94	B2	0.699330786	Bcer	NA	Bcer	FALSE	NA	NA	NA
Bc_ctg2_95	B2	833.2526315	Bcer	S	Bcer	FALSE	NA	NA	NA
Bc_ctg2_96	B2	5.594646288	Bcer	IQ	Bcer	TRUE	NA	NA	NA
Bc_ctg2_98	B2	0.116555131	Bcer	NA	Bcer	FALSE	Bc_Ga0417192_02_rn02	Other	NA
Bc_ctg2_99	B2	4.778760371	Bcer	EG	Bcer	FALSE	Bc_Ga0417192_02_rn02	Transport	SMCOG1166
Bc_ctg3_1	B2	15.15216703	Bcer	P	Bcer	FALSE	NA	NA	NA
Bc_ctg3_10	B2	12.82106441	Bcer	EGP	Bcer	FALSE	Bc_Ga0417192_03_rn01	Transport	SMCOG1106
Bc_ctg3_100	B2	0.699330786	Bcer	V	Bcer	FALSE	NA	NA	NA
Bc_ctg3_101	B2	0	Bcer	S	Bcer	FALSE	NA	NA	NA
Bc_ctg3_102	B2	0.349665393	Bcer	K	Bcer	FALSE	NA	NA	NA
Bc_ctg3_103	B2	3.613209061	Bcer	K	Bcer	FALSE	NA	NA	NA
Bc_ctg3_104	B2	1.398661572	Bcer	L	Bcer	FALSE	NA	NA	NA
Bc_ctg3_105	B2	0.349665393	Bcer	L	Bcer	FALSE	NA	NA	NA
Bc_ctg3_106	B2	0	Bcer	S	Bcer	FALSE	NA	NA	NA
Bc_ctg3_107	B2	9.091300218	Bcer	V	Bcer	FALSE	NA	NA	NA
Bc_ctg3_108	B2	2.097992358	Bcer	V	Bcer	FALSE	NA	NA	NA
Bc_ctg3_109	B2	1.16555131	Bcer	V	Bcer	FALSE	NA	NA	NA
Bc_ctg3_11	B2	0.233110262	Bcer	NA	Bcer	FALSE	Bc_Ga0417192_03_rn01	Other	NA
Bc_ctg3_110	B2	5.478091157	Bcer	S	Bcer	FALSE	NA	NA	NA
Bc_ctg3_111	B2	1.16555131	Bcer	K	Bcer	FALSE	NA	NA	NA
Bc_ctg3_112	B2	210.3820114	Bcer	M	Bcer	FALSE	NA	NA	NA
Bc_ctg3_113	B2	10.25685153	Bcer	M	Bcer	FALSE	NA	NA	NA
Bc_ctg3_114	B2	13.40384006	Bcer	S	Bcer	FALSE	NA	NA	NA
Bc_ctg3_115	B2	587.6709705	Bcer	G	Bcer	FALSE	NA	NA	NA
Bc_ctg3_116	B2	239.4042391	Bcer	G	Bcer	FALSE	NA	NA	NA
Bc_ctg3_117	B2	563.8937238	Bcer	G	Bcer	FALSE	NA	NA	NA
Bc_ctg3_118	B2	18.76537609	Bcer	U	Bcer	FALSE	NA	NA	NA
Bc_ctg3_119	B2	1.631771834	Bcer	G	Bcer	FALSE	NA	NA	NA
Bc_ctg3_12	B2	1.864882096	Bcer	Q	Bcer	TRUE	Bc_Ga0417192_03_rn01	Ancillary biosynthesis	SMCOG1027
Bc_ctg3_120	B2	15.96805295	Bcer	S	Bcer	FALSE	NA	NA	NA
Bc_ctg3_121	B2	13.75350546	Bcer	G	Bcer	FALSE	NA	NA	NA
Bc_ctg3_122	B2	931.0423864	Bcer	C	Bcer	FALSE	NA	NA	NA
Bc_ctg3_123	B2	108.8624924	Bcer	G	Bcer	FALSE	NA	NA	NA
Bc_ctg3_124	B2	6.643642467	Bcer	NA	Bcer	FALSE	NA	NA	NA
Bc_ctg3_126	B2	5.944311681	Bcer	S	Bcer	FALSE	NA	NA	NA
Bc_ctg3_128	B2	2.797323144	Bcer	K	Bcer	FALSE	NA	NA	NA
Bc_ctg3_129	B2	6.060866812	Bcer	V	Bcer	FALSE	NA	NA	NA
Bc_ctg3_13	B2	0.582775655	Bcer	G	Bcer	FALSE	Bc_Ga0417192_03_rn01	Ancillary biosynthesis	SMCOG1235
Bc_ctg3_130	B2	4.195984716	Bcer	S	Bcer	FALSE	NA	NA	NA
Bc_ctg3_131	B2	10.60651692	Bcer	NA	Bcer	FALSE	NA	NA	NA
Bc_ctg3_132	B2	15.38527729	Bcer	U	Bcer	FALSE	NA	NA	NA
Bc_ctg3_133	B2	10.1402964	Bcer	NA	Bcer	FALSE	NA	NA	NA
Bc_ctg3_134	B2	29.25533788	Bcer	L	Bcer	FALSE	NA	NA	NA
Bc_ctg3_135	B2	2.680768013	Bcer	V	Bcer	FALSE	NA	NA	NA
Bc_ctg3_136	B2	1.282106441	Bcer	V	Bcer	FALSE	NA	NA	NA
Bc_ctg3_137	B2	0.815885917	Bcer	O	Bcer	FALSE	NA	NA	NA
Bc_ctg3_139	B2	9.674075873	Bcer	T	Bcer	FALSE	NA	NA	NA

Bc_ctg3_14	B2	10.72307205	Bcer	EGP	Bcer	FALSE	Bc_Ga0417192_03_rn01	Transport	SMCOG1020
Bc_ctg3_140	B2	32.51888155	Bcer	T	Bcer	FALSE	NA	NA	NA
Bc_ctg3_141	B2	0	Bcer	NA	Bcer	FALSE	NA	NA	NA
Bc_ctg3_142	B2	17.25015939	Bcer	S	Bcer	FALSE	NA	NA	NA
Bc_ctg3_143	B2	93.12754967	Bcer	S	Bcer	FALSE	NA	NA	NA
Bc_ctg3_144	B2	60.84177838	Bcer	G	Bcer	FALSE	NA	NA	NA
Bc_ctg3_145	B2	24.36002238	Bcer	S	Bcer	FALSE	NA	NA	NA
Bc_ctg3_146	B2	138.5840508	Bcer	Q	Bcer	TRUE	NA	NA	NA
Bc_ctg3_147	B2	102.2188499	Bcer	H	Bcer	FALSE	NA	NA	NA
Bc_ctg3_148	B2	278.7998733	Bcer	F	Bcer	FALSE	Bc_Ga0417192_03_rn02	Other	NA
Bc_ctg3_149	B2	409.8078406	Bcer	J	Bcer	FALSE	Bc_Ga0417192_03_rn02	Other	NA
Bc_ctg3_15	B2	0	Bcer	L	Bcer	FALSE	Bc_Ga0417192_03_rn01	Other	NA
Bc_ctg3_150	B2	46.03927674	Bcer	NA	Bcer	FALSE	Bc_Ga0417192_03_rn02	Ancillary biosynthesis	None
Bc_ctg3_151	B2	381.1352784	Bcer	NA	Bcer	FALSE	Bc_Ga0417192_03_rn02	Other	NA
Bc_ctg3_152	B2	54.19813591	Bcer	NA	Bcer	FALSE	Bc_Ga0417192_03_rn02	Other	NA
Bc_ctg3_153	B2	220.4057527	Bcer	T	Bcer	FALSE	Bc_Ga0417192_03_rn02	Other	SMCOG1226
Bc_ctg3_154	B2	163.6434039	Bcer	NA	Bcer	FALSE	Bc_Ga0417192_03_rn02	Other	NA
Bc_ctg3_155	B2	305.9572189	Bcer	EGP	Bcer	FALSE	Bc_Ga0417192_03_rn02	Transport	SMCOG1020
Bc_ctg3_156	B2	3.729764192	Bcer	NA	Bcer	FALSE	Bc_Ga0417192_03_rn02	Other	NA
Bc_ctg3_158	B2	18.53226583	Bcer	S	Bcer	FALSE	Bc_Ga0417192_03_rn02	Other	NA
Bc_ctg3_159	B2	51.40081277	Bcer	C	Bcer	FALSE	Bc_Ga0417192_03_rn02	Ancillary biosynthesis	None
Bc_ctg3_16	B2	0.699330786	Bcer	NA	Bcer	FALSE	Bc_Ga0417192_03_rn01	Other	NA
Bc_ctg3_160	B2	0.233110262	Bcer	S	Bcer	FALSE	Bc_Ga0417192_03_rn02	Core biosynthesis	None
Bc_ctg3_161	B2	44.05783952	Bcer	OS	Bcer	FALSE	Bc_Ga0417192_03_rn02	Ancillary biosynthesis	None
Bc_ctg3_162	B2	20.39714792	Bcer	O	Bcer	FALSE	Bc_Ga0417192_03_rn02	Other	NA
Bc_ctg3_163	B2	19.58126201	Bcer	E	Bcer	FALSE	Bc_Ga0417192_03_rn02	Ancillary biosynthesis	SMCOG1038
Bc_ctg3_164	B2	186.3716545	Bcer	E	Bcer	FALSE	Bc_Ga0417192_03_rn02	Ancillary biosynthesis	SMCOG1081
Bc_ctg3_165	B2	224.8348477	Bcer	E	Bcer	FALSE	Bc_Ga0417192_03_rn02	Other	NA
Bc_ctg3_166	B2	10.1402964	Bcer	E	Bcer	FALSE	NA	NA	NA
Bc_ctg3_167	B2	3.49665393	Bcer	S	Bcer	FALSE	NA	NA	NA
Bc_ctg3_168	B2	6.643642467	Bcer	C	Bcer	FALSE	NA	NA	NA
Bc_ctg3_169	B2	1.282106441	Bcer	P	Bcer	FALSE	NA	NA	NA
Bc_ctg3_17	B2	0.233110262	Bcer	K	Bcer	FALSE	Bc_Ga0417192_03_rn01	Other	NA
Bc_ctg3_170	B2	45.80616648	Bcer	C	Bcer	FALSE	NA	NA	NA
Bc_ctg3_171	B2	2.214547489	Bcer	L	Bcer	FALSE	NA	NA	NA
Bc_ctg3_172	B2	11.6555131	Bcer	L	Bcer	FALSE	NA	NA	NA
Bc_ctg3_173	B2	2.097992358	Bcer	NU	Bcer	FALSE	NA	NA	NA
Bc_ctg3_174	B2	20.74681332	Bcer	L	Bcer	FALSE	NA	NA	NA
Bc_ctg3_175	B2	24.36002238	Bcer	L	Bcer	FALSE	NA	NA	NA
Bc_ctg3_176	B2	15.15216703	Bcer	S	Bcer	FALSE	NA	NA	NA
Bc_ctg3_177	B2	15.38527729	Bcer	F	Bcer	FALSE	NA	NA	NA
Bc_ctg3_178	B2	9.790631004	Bcer	O	Bcer	FALSE	NA	NA	NA
Bc_ctg3_179	B2	8.625079694	Bcer	C	Bcer	FALSE	NA	NA	NA
Bc_ctg3_18	B2	4.079429585	Bcer	L	Bcer	FALSE	Bc_Ga0417192_03_rn01	Other	NA
Bc_ctg3_180	B2	0.815885917	Bcer	NA	Bcer	FALSE	NA	NA	NA
Bc_ctg3_181	B2	4211.486548	Bcer	C	Bcer	FALSE	NA	NA	NA
Bc_ctg3_182	B2	34.84998417	Bcer	NA	Bcer	FALSE	NA	NA	NA
Bc_ctg3_183	B2	37.29764192	Bcer	NA	Bcer	FALSE	NA	NA	NA
Bc_ctg3_184	B2	84.38591484	Bcer	K	Bcer	FALSE	NA	NA	NA
Bc_ctg3_185	B2	37.76386244	Bcer	NA	Bcer	FALSE	NA	NA	NA
Bc_ctg3_186	B2	290.1057211	Bcer	NA	Bcer	FALSE	NA	NA	NA
Bc_ctg3_187	B2	25.99179421	Bcer	K	Bcer	FALSE	NA	NA	NA
Bc_ctg3_188	B2	8.974745087	Bcer	NA	Bcer	FALSE	NA	NA	NA
Bc_ctg3_189	B2	0	Bcer	K	Bcer	FALSE	NA	NA	NA
Bc_ctg3_19	B2	0	Bcer	NA	Bcer	FALSE	Bc_Ga0417192_03_rn01	Other	NA
Bc_ctg3_190	B2	3.030433406	Bcer	NA	Bcer	FALSE	NA	NA	NA
Bc_ctg3_191	B2	6.527087336	Bcer	S	Bcer	FALSE	NA	NA	NA
Bc_ctg3_192	B2	1.16555131	Bcer	NA	Bcer	FALSE	NA	NA	NA
Bc_ctg3_193	B2	1.864882096	Bcer	NA	Bcer	FALSE	NA	NA	NA
Bc_ctg3_194	B2	3.263543668	Bcer	NA	Bcer	FALSE	NA	NA	NA
Bc_ctg3_195	B2	152.8037767	Bcer	T	Bcer	FALSE	NA	NA	NA
Bc_ctg3_196	B2	78.55815829	Bcer	L	Bcer	FALSE	NA	NA	NA

Bc_ctg3_197	B2	18.29915557	Bcer	NA	Bcer	FALSE	NA	NA	NA
Bc_ctg3_198	B2	2.447657751	Bcer	S	Bcer	FALSE	NA	NA	NA
Bc_ctg3_199	B2	2.913878275	Bcer	K	Bcer	FALSE	NA	NA	NA
Bc_ctg3_2	B2	103.7340666	Bcer	P	Bcer	FALSE	NA	NA	NA
Bc_ctg3_20	B2	0	Bcer	NA	Bcer	FALSE	Bc_Ga0417192_03_rn01	Other	NA
Bc_ctg3_200	B2	14.56939137	Bcer	NA	Bcer	FALSE	NA	NA	NA
Bc_ctg3_201	B2	19.69781714	Bcer	NA	Bcer	FALSE	NA	NA	NA
Bc_ctg3_202	B2	90.44678165	Bcer	P	Bcer	FALSE	NA	NA	NA
Bc_ctg3_203	B2	15.0356119	Bcer	NA	Bcer	FALSE	NA	NA	NA
Bc_ctg3_204	B2	8.15885917	Bcer	K	Bcer	FALSE	NA	NA	NA
Bc_ctg3_206	B2	0.466220524	Bcer	NA	Bcer	FALSE	NA	NA	NA
Bc_ctg3_207	B2	4.195984716	Bcer	Q	Bcer	TRUE	NA	NA	NA
Bc_ctg3_208	B2	41.26051637	Bcer	S	Bcer	FALSE	NA	NA	NA
Bc_ctg3_209	B2	2.447657751	Bcer	NA	Bcer	FALSE	NA	NA	NA
Bc_ctg3_21	B2	2.564212882	Bcer	M	Bcer	FALSE	Bc_Ga0417192_03_rn01	Core biosynthesis	SMCOG1010
Bc_ctg3_212	B2	5.944311681	Bcer	S	Bcer	FALSE	NA	NA	NA
Bc_ctg3_213	B2	1.981437227	Bcer	NA	Bcer	FALSE	NA	NA	NA
Bc_ctg3_214	B2	6.643642467	Bcer	E	Bcer	FALSE	NA	NA	NA
Bc_ctg3_215	B2	4.312539847	Bcer	E	Bcer	FALSE	NA	NA	NA
Bc_ctg3_216	B2	0.233110262	Bcer	L	Bcer	FALSE	NA	NA	NA
Bc_ctg3_217	B2	2.214547489	Bcer	K	Bcer	FALSE	NA	NA	NA
Bc_ctg3_218	B2	1.864882096	Bcer	S	Bcer	FALSE	NA	NA	NA
Bc_ctg3_219	B2	0	Bcer	S	Bcer	FALSE	NA	NA	NA
Bc_ctg3_22	B2	2.564212882	Bcer	M	Bcer	FALSE	Bc_Ga0417192_03_rn01	Core biosynthesis	SMCOG1010
Bc_ctg3_220	B2	6.060866812	Bcer	M	Bcer	FALSE	NA	NA	NA
Bc_ctg3_221	B2	0	Bcer	L	Bcer	FALSE	NA	NA	NA
Bc_ctg3_222	B2	0.349665393	Bcer	L	Bcer	FALSE	NA	NA	NA
Bc_ctg3_223	B2	5.478091157	Bcer	NA	Bcer	FALSE	NA	NA	NA
Bc_ctg3_224	B2	11.53895797	Bcer	C	Bcer	FALSE	NA	NA	NA
Bc_ctg3_225	B2	11.53895797	Bcer	K	Bcer	FALSE	NA	NA	NA
Bc_ctg3_226	B2	81.47203657	Bcer	GKT	Bcer	FALSE	NA	NA	NA
Bc_ctg3_227	B2	819.9653466	Bcer	G	Bcer	FALSE	NA	NA	NA
Bc_ctg3_228	B2	415.0528215	Bcer	G	Bcer	FALSE	NA	NA	NA
Bc_ctg3_229	B2	0.233110262	Bcer	NA	Bcer	FALSE	NA	NA	NA
Bc_ctg3_23	B2	1.515216703	Bcer	M	Bcer	FALSE	Bc_Ga0417192_03_rn01	Core biosynthesis	None
Bc_ctg3_230	B2	20.39714792	Bcer	P	Bcer	FALSE	NA	NA	NA
Bc_ctg3_24	B2	2.33110262	Bcer	M	Bcer	FALSE	Bc_Ga0417192_03_rn01	Ancillary biosynthesis	SMCOG1064
Bc_ctg3_25	B2	6.527087336	Bcer	I	Bcer	FALSE	Bc_Ga0417192_03_rn01	Core biosynthesis	SMCOG1022
Bc_ctg3_26	B2	4.195984716	Bcer	E	Bcer	FALSE	Bc_Ga0417192_03_rn01	Other	NA
Bc_ctg3_27	B2	2.680768013	Bcer	NA	Bcer	FALSE	Bc_Ga0417192_03_rn01	Other	NA
Bc_ctg3_28	B2	5.478091157	Bcer	K	Bcer	FALSE	Bc_Ga0417192_03_rn01	Other	NA
Bc_ctg3_29	B2	0.349665393	Bcer	I	Bcer	FALSE	Bc_Ga0417192_03_rn01	Ancillary biosynthesis	SMCOG1270
Bc_ctg3_3	B2	29.13878275	Bcer	E	Bcer	FALSE	NA	NA	NA
Bc_ctg3_30	B2	0.349665393	Bcer	IQ	Bcer	TRUE	Bc_Ga0417192_03_rn01	Ancillary biosynthesis	None
Bc_ctg3_31	B2	0.349665393	Bcer	IQ	Bcer	TRUE	Bc_Ga0417192_03_rn01	Ancillary biosynthesis	SMCOG1001
Bc_ctg3_32	B2	0.466220524	Bcer	I	Bcer	FALSE	Bc_Ga0417192_03_rn01	Ancillary biosynthesis	SMCOG1021
Bc_ctg3_33	B2	0	Bcer	S	Bcer	FALSE	Bc_Ga0417192_03_rn01	Other	NA
Bc_ctg3_34	B2	0.582775655	Bcer	V	Bcer	FALSE	Bc_Ga0417192_03_rn01	Transport	SMCOG1065
Bc_ctg3_35	B2	0.233110262	Bcer	V	Bcer	FALSE	Bc_Ga0417192_03_rn01	Transport	SMCOG1000
Bc_ctg3_36	B2	0.582775655	Bcer	S	Bcer	FALSE	Bc_Ga0417192_03_rn01	Other	NA
Bc_ctg3_37	B2	32.86854694	Bcer	S	Bcer	FALSE	Bc_Ga0417192_03_rn01	Other	NA
Bc_ctg3_38	B2	8.625079694	Bcer	NA	Bcer	FALSE	Bc_Ga0417192_03_rn01	Other	NA
Bc_ctg3_39	B2	5.244980895	Bcer	L	Bcer	FALSE	Bc_Ga0417192_03_rn01	Other	SMCOG1077
Bc_ctg3_4	B2	26.10834934	Bcer	H	Bcer	FALSE	NA	NA	NA
Bc_ctg3_40	B2	0.233110262	Bcer	NA	Bcer	FALSE	Bc_Ga0417192_03_rn01	Other	NA
Bc_ctg3_41	B2	8.858189956	Bcer	E	Bcer	FALSE	Bc_Ga0417192_03_rn01	Other	NA
Bc_ctg3_42	B2	6.760197598	Bcer	E	Bcer	FALSE	Bc_Ga0417192_03_rn01	Ancillary biosynthesis	SMCOG1153
Bc_ctg3_43	B2	7.809193777	Bcer	E	Bcer	FALSE	Bc_Ga0417192_03_rn01	Other	NA
Bc_ctg3_44	B2	0.932441048	Bcer	P	Bcer	FALSE	NA	NA	NA
Bc_ctg3_45	B2	0.349665393	Bcer	E	Bcer	FALSE	NA	NA	NA
Bc_ctg3_46	B2	0.349665393	Bcer	S	Bcer	FALSE	NA	NA	NA
Bc_ctg3_47	B2	0.116555131	Bcer	NA	Bcer	FALSE	NA	NA	NA

Bc_ctg3_48	B2	0	Bcer	NA	Bcer	FALSE	NA	NA	NA
Bc_ctg3_49	B2	0.466220524	Bcer	NA	Bcer	FALSE	NA	NA	NA
Bc_ctg3_5	B2	6.293977074	Bcer	K	Bcer	FALSE	NA	NA	NA
Bc_ctg3_50	B2	2.797323144	Bcer	V	Bcer	FALSE	NA	NA	NA
Bc_ctg3_51	B2	2.797323144	Bcer	V	Bcer	FALSE	NA	NA	NA
Bc_ctg3_52	B2	35.78242522	Bcer	K	Bcer	FALSE	NA	NA	NA
Bc_ctg3_53	B2	20.86336845	Bcer	S	Bcer	FALSE	NA	NA	NA
Bc_ctg3_54	B2	0	Bcer	NA	Bcer	FALSE	NA	NA	NA
Bc_ctg3_55	B2	0.233110262	Bcer	NA	Bcer	FALSE	NA	NA	NA
Bc_ctg3_56	B2	8.858189956	Bcer	NA	Bcer	FALSE	NA	NA	NA
Bc_ctg3_57	B2	1.515216703	Bcer	S	Bcer	FALSE	NA	NA	NA
Bc_ctg3_58	B2	1.398661572	Bcer	NA	Bcer	FALSE	NA	NA	NA
Bc_ctg3_59	B2	0	Bcer	NA	Bcer	FALSE	NA	NA	NA
Bc_ctg3_6	B2	0.582775655	Bcer	NA	Bcer	FALSE	NA	NA	NA
Bc_ctg3_60	B2	109.6783783	Bcer	K	Bcer	FALSE	NA	NA	NA
Bc_ctg3_61	B2	4.079429585	Bcer	S	Bcer	FALSE	NA	NA	NA
Bc_ctg3_62	B2	5.711201419	Bcer	NA	Bcer	FALSE	NA	NA	NA
Bc_ctg3_63	B2	1.631771834	Bcer	L	Bcer	FALSE	NA	NA	NA
Bc_ctg3_64	B2	28.43945196	Bcer	K	Bcer	FALSE	NA	NA	NA
Bc_ctg3_65	B2	5.711201419	Bcer	E	Bcer	FALSE	NA	NA	NA
Bc_ctg3_66	B2	2.564212882	Bcer	I	Bcer	FALSE	NA	NA	NA
Bc_ctg3_67	B2	0.116555131	Bcer	S	Bcer	FALSE	NA	NA	NA
Bc_ctg3_68	B2	1.282106441	Bcer	H	Bcer	FALSE	NA	NA	NA
Bc_ctg3_69	B2	1.048996179	Bcer	H	Bcer	FALSE	NA	NA	NA
Bc_ctg3_7	B2	15.73494268	Bcer	QT	Bcer	TRUE	Bc_Ga0417192_03_rn01	Other	NA
Bc_ctg3_70	B2	0	Bcer	L	Bcer	FALSE	NA	NA	NA
Bc_ctg3_71	B2	160.0301949	Bcer	I	Bcer	FALSE	NA	NA	NA
Bc_ctg3_72	B2	1.748326965	Bcer	L	Bcer	FALSE	NA	NA	NA
Bc_ctg3_73	B2	28.2063417	Bcer	K	Bcer	FALSE	NA	NA	NA
Bc_ctg3_74	B2	47.08827292	Bcer	S	Bcer	FALSE	NA	NA	NA
Bc_ctg3_75	B2	32.98510207	Bcer	K	Bcer	FALSE	NA	NA	NA
Bc_ctg3_76	B2	0	Bcer	L	Bcer	FALSE	NA	NA	NA
Bc_ctg3_77	B2	0	Bcer	L	Bcer	FALSE	NA	NA	NA
Bc_ctg3_78	B2	22.84480568	Bcer	D	Bcer	FALSE	NA	NA	NA
Bc_ctg3_79	B2	4.895315502	Bcer	M	Bcer	FALSE	NA	NA	NA
Bc_ctg3_8	B2	5.944311681	Bcer	EGP	Bcer	FALSE	Bc_Ga0417192_03_rn01	Transport	SMCOG1106
Bc_ctg3_80	B2	23.54413646	Bcer	I	Bcer	FALSE	NA	NA	NA
Bc_ctg3_81	B2	77.62571724	Bcer	M	Bcer	FALSE	NA	NA	NA
Bc_ctg3_82	B2	0.116555131	Bcer	NA	Bcer	FALSE	NA	NA	NA
Bc_ctg3_83	B2	0.349665393	Bcer	K	Bcer	FALSE	NA	NA	NA
Bc_ctg3_84	B2	21.56269923	Bcer	O	Bcer	FALSE	NA	NA	NA
Bc_ctg3_85	B2	0.932441048	Bcer	O	Bcer	FALSE	NA	NA	NA
Bc_ctg3_86	B2	0	Bcer	NA	Bcer	FALSE	NA	NA	NA
Bc_ctg3_87	B2	1.16555131	Bcer	NA	Bcer	FALSE	NA	NA	NA
Bc_ctg3_88	B2	8.858189956	Bcer	P	Bcer	FALSE	NA	NA	NA
Bc_ctg3_89	B2	2.797323144	Bcer	NA	Bcer	FALSE	NA	NA	NA
Bc_ctg3_9	B2	8.15885917	Bcer	E	Bcer	FALSE	Bc_Ga0417192_03_rn01	Ancillary biosynthesis	SMCOG1244
Bc_ctg3_90	B2	0.116555131	Bcer	NA	Bcer	FALSE	NA	NA	NA
Bc_ctg3_91	B2	142.3138149	Bcer	C	Bcer	FALSE	NA	NA	NA
Bc_ctg3_92	B2	15.96805295	Bcer	NA	Bcer	FALSE	NA	NA	NA
Bc_ctg3_93	B2	4.545650109	Bcer	NA	Bcer	FALSE	NA	NA	NA
Bc_ctg3_94	B2	1.748326965	Bcer	S	Bcer	FALSE	NA	NA	NA
Bc_ctg3_95	B2	7.109862991	Bcer	NA	Bcer	FALSE	NA	NA	NA
Bc_ctg3_96	B2	24.47657751	Bcer	I	Bcer	FALSE	NA	NA	NA
Bc_ctg3_97	B2	18.4157107	Bcer	T	Bcer	FALSE	NA	NA	NA
Bc_ctg3_98	B2	7.109862991	Bcer	T	Bcer	FALSE	NA	NA	NA
Bc_ctg3_99	B2	10.48996179	Bcer	V	Bcer	FALSE	NA	NA	NA
Bc_ctg1_1	B3	27.28365234	Bcer	E	Bcer	FALSE	NA	NA	NA
Bc_ctg1_10	B3	54.00359286	Bcer	K	Bcer	FALSE	NA	NA	NA
Bc_ctg1_100	B3	4.058725142	Bcer	S	Bcer	FALSE	NA	NA	NA
Bc_ctg1_1002	B3	19.72991388	Bcer	S	Bcer	FALSE	Bc_Ga0417192_01_rn09	Other	NA
Bc_ctg1_1003	B3	16.91135476	Bcer	S	Bcer	FALSE	Bc_Ga0417192_01_rn09	Other	NA

Bc_ctg1_1004	B3	70.68946289	Bcer	NA	Bcer	FALSE	Bc_Ga0417192_01_rn09	Other	NA
Bc_ctg1_1005	B3	7.779223189	Bcer	S	Bcer	FALSE	Bc_Ga0417192_01_rn09	Other	NA
Bc_ctg1_1006	B3	33.03351296	Bcer	S	Bcer	FALSE	Bc_Ga0417192_01_rn09	Other	NA
Bc_ctg1_1007	B3	484.9049121	Bcer	I	Bcer	FALSE	Bc_Ga0417192_01_rn09	Ancillary biosynthesis	SMCOG1124
Bc_ctg1_1008	B3	87.71356001	Bcer	F	Bcer	FALSE	Bc_Ga0417192_01_rn09	Other	NA
Bc_ctg1_1009	B3	175.42712	Bcer	S	Bcer	FALSE	Bc_Ga0417192_01_rn09	Other	NA
Bc_ctg1_101	B3	8.004707919	Bcer	S	Bcer	FALSE	NA	NA	NA
Bc_ctg1_1010	B3	0	Bcer	NA	Bcer	FALSE	NA	NA	NA
Bc_ctg1_1011	B3	8.004707919	Bcer	NA	Bcer	FALSE	NA	NA	NA
Bc_ctg1_1012	B3	10.71052468	Bcer	NA	Bcer	FALSE	NA	NA	NA
Bc_ctg1_1013	B3	1.578393111	Bcer	K	Bcer	FALSE	NA	NA	NA
Bc_ctg1_1014	B3	7.891965554	Bcer	IQ	Bcer	TRUE	NA	NA	NA
Bc_ctg1_1017	B3	0	Bcer	S	Bcer	FALSE	NA	NA	NA
Bc_ctg1_1018	B3	12.96537198	Bcer	E	Bcer	FALSE	NA	NA	NA
Bc_ctg1_1019	B3	19.95539861	Bcer	F	Bcer	FALSE	NA	NA	NA
Bc_ctg1_102	B3	0	Bcer	L	Bcer	FALSE	NA	NA	NA
Bc_ctg1_1020	B3	15.33296165	Bcer	S	Bcer	FALSE	NA	NA	NA
Bc_ctg1_1021	B3	55.58198597	Bcer	E	Bcer	FALSE	NA	NA	NA
Bc_ctg1_1022	B3	22.66121538	Bcer	G	Bcer	FALSE	NA	NA	NA
Bc_ctg1_1023	B3	11.49972124	Bcer	E	Bcer	FALSE	NA	NA	NA
Bc_ctg1_1024	B3	17.47506658	Bcer	E	Bcer	FALSE	NA	NA	NA
Bc_ctg1_1025	B3	15.78393111	Bcer	S	Bcer	FALSE	NA	NA	NA
Bc_ctg1_1026	B3	0.338227095	Bcer	NA	Bcer	FALSE	NA	NA	NA
Bc_ctg1_1027	B3	11.16149414	Bcer	S	Bcer	FALSE	NA	NA	NA
Bc_ctg1_1028	B3	102.2573251	Bcer	I	Bcer	FALSE	NA	NA	NA
Bc_ctg1_1029	B3	111.2767143	Bcer	V	Bcer	FALSE	NA	NA	NA
Bc_ctg1_103	B3	0	Bcer	L	Bcer	FALSE	NA	NA	NA
Bc_ctg1_1030	B3	2.705816761	Bcer	T	Bcer	FALSE	NA	NA	NA
Bc_ctg1_1031	B3	42.05290216	Bcer	C	Bcer	FALSE	NA	NA	NA
Bc_ctg1_1032	B3	20.6318528	Bcer	NA	Bcer	FALSE	NA	NA	NA
Bc_ctg1_1033	B3	11.16149414	Bcer	S	Bcer	FALSE	NA	NA	NA
Bc_ctg1_1034	B3	2153.040945	Bcer	G	Bcer	FALSE	NA	NA	NA
Bc_ctg1_1035	B3	291.8899831	Bcer	G	Bcer	FALSE	NA	NA	NA
Bc_ctg1_1036	B3	957.408164	Bcer	G	Bcer	FALSE	NA	NA	NA
Bc_ctg1_1037	B3	91.09583096	Bcer	K	Bcer	FALSE	NA	NA	NA
Bc_ctg1_1038	B3	194.9315492	Bcer	S	Bcer	FALSE	NA	NA	NA
Bc_ctg1_1039	B3	949.2907137	Bcer	K	Bcer	FALSE	NA	NA	NA
Bc_ctg1_104	B3	3.720498047	Bcer	S	Bcer	FALSE	NA	NA	NA
Bc_ctg1_1040	B3	1068.121167	Bcer	G	Bcer	FALSE	NA	NA	NA
Bc_ctg1_1041	B3	126.7224183	Bcer	G	Bcer	FALSE	NA	NA	NA
Bc_ctg1_1042	B3	3.833240412	Bcer	S	Bcer	FALSE	NA	NA	NA
Bc_ctg1_1043	B3	13.75456854	Bcer	D	Bcer	FALSE	NA	NA	NA
Bc_ctg1_1044	B3	94.36535955	Bcer	D	Bcer	FALSE	NA	NA	NA
Bc_ctg1_1046	B3	16.79861239	Bcer	S	Bcer	FALSE	NA	NA	NA
Bc_ctg1_1047	B3	165.5057919	Bcer	K	Bcer	FALSE	NA	NA	NA
Bc_ctg1_1048	B3	993.2602361	Bcer	O	Bcer	FALSE	NA	NA	NA
Bc_ctg1_1049	B3	282.5323668	Bcer	S	Bcer	FALSE	NA	NA	NA
Bc_ctg1_105	B3	1.127423651	Bcer	NA	Bcer	FALSE	NA	NA	NA
Bc_ctg1_1050	B3	19.27894442	Bcer	S	Bcer	FALSE	NA	NA	NA
Bc_ctg1_1051	B3	3.156786221	Bcer	EG	Bcer	FALSE	NA	NA	NA
Bc_ctg1_1052	B3	0.112742365	Bcer	S	Bcer	FALSE	NA	NA	NA
Bc_ctg1_1053	B3	0	Bcer	S	Bcer	FALSE	NA	NA	NA
Bc_ctg1_1054	B3	0.45096946	Bcer	IQ	Bcer	TRUE	NA	NA	NA
Bc_ctg1_1055	B3	0.112742365	Bcer	S	Bcer	FALSE	NA	NA	NA
Bc_ctg1_1057	B3	0	Bcer	S	Bcer	FALSE	NA	NA	NA
Bc_ctg1_1059	B3	0.563711825	Bcer	S	Bcer	FALSE	NA	NA	NA
Bc_ctg1_106	B3	0.22548473	Bcer	K	Bcer	FALSE	NA	NA	NA
Bc_ctg1_1060	B3	62.45927024	Bcer	K	Bcer	FALSE	NA	NA	NA
Bc_ctg1_1061	B3	34.27367898	Bcer	F	Bcer	FALSE	NA	NA	NA
Bc_ctg1_1062	B3	20.06814098	Bcer	T	Bcer	FALSE	NA	NA	NA
Bc_ctg1_1063	B3	0.112742365	Bcer	M	Bcer	FALSE	NA	NA	NA
Bc_ctg1_1064	B3	14.43102273	Bcer	K	Bcer	FALSE	NA	NA	NA

Bc_ctg1_1065	B3	46.22436967	Bcer	V	Bcer	FALSE	NA	NA	NA
Bc_ctg1_1066	B3	71.59140181	Bcer	S	Bcer	FALSE	NA	NA	NA
Bc_ctg1_1067	B3	1.691135476	Bcer	D	Bcer	FALSE	NA	NA	NA
Bc_ctg1_1068	B3	182.3044043	Bcer	K	Bcer	FALSE	NA	NA	NA
Bc_ctg1_1069	B3	0.67645419	Bcer	S	Bcer	FALSE	NA	NA	NA
Bc_ctg1_107	B3	8.004707919	Bcer	GIM	Bcer	FALSE	NA	NA	NA
Bc_ctg1_1070	B3	159.6431889	Bcer	S	Bcer	FALSE	NA	NA	NA
Bc_ctg1_1071	B3	85.12048561	Bcer	P	Bcer	FALSE	NA	NA	NA
Bc_ctg1_1072	B3	2875.155794	Bcer	F	Bcer	FALSE	NA	NA	NA
Bc_ctg1_1073	B3	2324.973052	Bcer	F	Bcer	FALSE	NA	NA	NA
Bc_ctg1_1074	B3	2835.583223	Bcer	G	Bcer	FALSE	NA	NA	NA
Bc_ctg1_1075	B3	85.00774325	Bcer	S	Bcer	FALSE	NA	NA	NA
Bc_ctg1_1076	B3	50.959549	Bcer	L	Bcer	FALSE	NA	NA	NA
Bc_ctg1_1077	B3	0.45096946	Bcer	S	Bcer	FALSE	NA	NA	NA
Bc_ctg1_1078	B3	504.5220836	Bcer	P	Bcer	FALSE	NA	NA	NA
Bc_ctg1_1079	B3	11.04875178	Bcer	S	Bcer	FALSE	NA	NA	NA
Bc_ctg1_108	B3	39.57257013	Bcer	K	Bcer	FALSE	NA	NA	NA
Bc_ctg1_1080	B3	226.0484419	Bcer	J	Bcer	FALSE	NA	NA	NA
Bc_ctg1_1081	B3	68.43461559	Bcer	L	Bcer	FALSE	NA	NA	NA
Bc_ctg1_1083	B3	80.04707919	Bcer	C	Bcer	FALSE	NA	NA	NA
Bc_ctg1_1084	B3	23.78863903	Bcer	S	Bcer	FALSE	NA	NA	NA
Bc_ctg1_1085	B3	4.847921697	Bcer	K	Bcer	FALSE	NA	NA	NA
Bc_ctg1_1086	B3	0.338227095	Bcer	S	Bcer	FALSE	NA	NA	NA
Bc_ctg1_1087	B3	157.1628569	Bcer	F	Bcer	FALSE	NA	NA	NA
Bc_ctg1_1088	B3	2.254847301	Bcer	S	Bcer	FALSE	NA	NA	NA
Bc_ctg1_1089	B3	17.36232422	Bcer	I	Bcer	FALSE	NA	NA	NA
Bc_ctg1_109	B3	3.156786221	Bcer	IQ	Bcer	TRUE	NA	NA	NA
Bc_ctg1_1090	B3	39.91079723	Bcer	S	Bcer	FALSE	NA	NA	NA
Bc_ctg1_1091	B3	26.71994052	Bcer	K	Bcer	FALSE	NA	NA	NA
Bc_ctg1_1092	B3	3.269528587	Bcer	K	Bcer	FALSE	NA	NA	NA
Bc_ctg1_1093	B3	157.2755992	Bcer	S	Bcer	FALSE	NA	NA	NA
Bc_ctg1_1094	B3	13.07811435	Bcer	S	Bcer	FALSE	NA	NA	NA
Bc_ctg1_1095	B3	31.68060458	Bcer	S	Bcer	FALSE	NA	NA	NA
Bc_ctg1_1096	B3	204.8528773	Bcer	H	Bcer	FALSE	NA	NA	NA
Bc_ctg1_1097	B3	140.9279563	Bcer	H	Bcer	FALSE	NA	NA	NA
Bc_ctg1_1098	B3	380.6182244	Bcer	H	Bcer	FALSE	NA	NA	NA
Bc_ctg1_1099	B3	185.91216	Bcer	H	Bcer	FALSE	NA	NA	NA
Bc_ctg1_11	B3	1.014681285	Bcer	S	Bcer	FALSE	NA	NA	NA
Bc_ctg1_110	B3	1.240166016	Bcer	K	Bcer	FALSE	NA	NA	NA
Bc_ctg1_1100	B3	0	Bcer	NA	Bcer	FALSE	NA	NA	NA
Bc_ctg1_1101	B3	3.607755682	Bcer	H	Bcer	FALSE	NA	NA	NA
Bc_ctg1_1102	B3	1.352908381	Bcer	H	Bcer	FALSE	NA	NA	NA
Bc_ctg1_1103	B3	1.465650746	Bcer	S	Bcer	FALSE	NA	NA	NA
Bc_ctg1_1104	B3	2.367589666	Bcer	H	Bcer	FALSE	NA	NA	NA
Bc_ctg1_1105	B3	1.352908381	Bcer	H	Bcer	FALSE	NA	NA	NA
Bc_ctg1_1106	B3	1.578393111	Bcer	H	Bcer	FALSE	NA	NA	NA
Bc_ctg1_1107	B3	0	Bcer	NA	Bcer	FALSE	NA	NA	NA
Bc_ctg1_1108	B3	18.82797496	Bcer	C	Bcer	FALSE	NA	NA	NA
Bc_ctg1_1109	B3	190.9855664	Bcer	F	Bcer	FALSE	NA	NA	NA
Bc_ctg1_111	B3	8.342935014	Bcer	S	Bcer	FALSE	NA	NA	NA
Bc_ctg1_1110	B3	22.43573065	Bcer	E	Bcer	FALSE	NA	NA	NA
Bc_ctg1_1111	B3	150.2855726	Bcer	K	Bcer	FALSE	NA	NA	NA
Bc_ctg1_1112	B3	4.509694602	Bcer	NA	Bcer	FALSE	NA	NA	NA
Bc_ctg1_1113	B3	35.06287553	Bcer	S	Bcer	FALSE	NA	NA	NA
Bc_ctg1_1114	B3	7.553738458	Bcer	E	Bcer	FALSE	NA	NA	NA
Bc_ctg1_1115	B3	4.847921697	Bcer	E	Bcer	FALSE	NA	NA	NA
Bc_ctg1_1116	B3	1.240166016	Bcer	E	Bcer	FALSE	NA	NA	NA
Bc_ctg1_1117	B3	0.22548473	Bcer	E	Bcer	FALSE	NA	NA	NA
Bc_ctg1_1118	B3	1.691135476	Bcer	E	Bcer	FALSE	NA	NA	NA
Bc_ctg1_1119	B3	0.90193892	Bcer	NA	Bcer	FALSE	NA	NA	NA
Bc_ctg1_112	B3	24.57783558	Bcer	L	Bcer	FALSE	Bc_Ga0417192_01_rn01	Other	NA
Bc_ctg1_1120	B3	122.8891779	Bcer	S	Bcer	FALSE	NA	NA	NA

Bc_ctg1_1121	B3	57.72409091	Bcer	S	Bcer	FALSE	NA	NA	NA
Bc_ctg1_1122	B3	4.960664062	Bcer	E	Bcer	FALSE	NA	NA	NA
Bc_ctg1_1123	B3	163.1382022	Bcer	E	Bcer	FALSE	NA	NA	NA
Bc_ctg1_1124	B3	103.8357182	Bcer	E	Bcer	FALSE	NA	NA	NA
Bc_ctg1_1125	B3	58.51328746	Bcer	S	Bcer	FALSE	NA	NA	NA
Bc_ctg1_1126	B3	94.81632901	Bcer	NA	Bcer	FALSE	NA	NA	NA
Bc_ctg1_1127	B3	1.916620206	Bcer	NA	Bcer	FALSE	NA	NA	NA
Bc_ctg1_1128	B3	57.16037908	Bcer	P	Bcer	FALSE	NA	NA	NA
Bc_ctg1_1129	B3	7.553738458	Bcer	S	Bcer	FALSE	NA	NA	NA
Bc_ctg1_113	B3	6.313572443	Bcer	E	Bcer	FALSE	Bc_Ga0417192_01_rn01	Other	NA
Bc_ctg1_1130	B3	16.79861239	Bcer	L	Bcer	FALSE	NA	NA	NA
Bc_ctg1_1132	B3	1804.892522	Bcer	E	Bcer	FALSE	NA	NA	NA
Bc_ctg1_1133	B3	100.2279625	Bcer	J	Bcer	FALSE	NA	NA	NA
Bc_ctg1_1134	B3	12.73988725	Bcer	NA	Bcer	FALSE	NA	NA	NA
Bc_ctg1_1135	B3	16.68587003	Bcer	S	Bcer	FALSE	NA	NA	NA
Bc_ctg1_1136	B3	41.26370561	Bcer	S	Bcer	FALSE	NA	NA	NA
Bc_ctg1_1137	B3	44.30774947	Bcer	E	Bcer	FALSE	NA	NA	NA
Bc_ctg1_1138	B3	30.66592329	Bcer	E	Bcer	FALSE	NA	NA	NA
Bc_ctg1_1139	B3	15.33296165	Bcer	ET	Bcer	FALSE	NA	NA	NA
Bc_ctg1_114	B3	1.578393111	Bcer	NA	Bcer	FALSE	Bc_Ga0417192_01_rn01	Other	NA
Bc_ctg1_1140	B3	71.25317471	Bcer	S	Bcer	FALSE	NA	NA	NA
Bc_ctg1_1141	B3	26.83268288	Bcer	S	Bcer	FALSE	NA	NA	NA
Bc_ctg1_1142	B3	27.39639471	Bcer	F	Bcer	FALSE	NA	NA	NA
Bc_ctg1_1143	B3	1.127423651	Bcer	S	Bcer	FALSE	NA	NA	NA
Bc_ctg1_1144	B3	303.0514773	Bcer	D	Bcer	FALSE	NA	NA	NA
Bc_ctg1_1145	B3	2996.466578	Bcer	C	Bcer	FALSE	NA	NA	NA
Bc_ctg1_1146	B3	1076.238617	Bcer	C	Bcer	FALSE	NA	NA	NA
Bc_ctg1_1147	B3	1207.357987	Bcer	C	Bcer	FALSE	NA	NA	NA
Bc_ctg1_1148	B3	1636.004459	Bcer	C	Bcer	FALSE	NA	NA	NA
Bc_ctg1_1149	B3	1143.207582	Bcer	C	Bcer	FALSE	NA	NA	NA
Bc_ctg1_1150	B3	3070.087343	Bcer	E	Bcer	FALSE	NA	NA	NA
Bc_ctg1_1151	B3	1731.497242	Bcer	C	Bcer	FALSE	NA	NA	NA
Bc_ctg1_1152	B3	31.45511985	Bcer	KT	Bcer	FALSE	NA	NA	NA
Bc_ctg1_1153	B3	2.480332031	Bcer	S	Bcer	FALSE	NA	NA	NA
Bc_ctg1_1154	B3	47.46453569	Bcer	C	Bcer	FALSE	NA	NA	NA
Bc_ctg1_1155	B3	0.338227095	Bcer	K	Bcer	FALSE	NA	NA	NA
Bc_ctg1_1156	B3	3.495013317	Bcer	NA	Bcer	FALSE	NA	NA	NA
Bc_ctg1_1157	B3	470.5866317	Bcer	KT	Bcer	FALSE	NA	NA	NA
Bc_ctg1_1158	B3	10.14681285	Bcer	M	Bcer	FALSE	NA	NA	NA
Bc_ctg1_1159	B3	107.1052468	Bcer	L	Bcer	FALSE	NA	NA	NA
Bc_ctg1_116	B3	7.779223189	Bcer	S	Bcer	FALSE	Bc_Ga0417192_01_rn01	Other	NA
Bc_ctg1_1160	B3	224.9210183	Bcer	K	Bcer	FALSE	NA	NA	NA
Bc_ctg1_1161	B3	134.5016415	Bcer	J	Bcer	FALSE	NA	NA	NA
Bc_ctg1_1162	B3	512.977761	Bcer	H	Bcer	FALSE	NA	NA	NA
Bc_ctg1_1163	B3	218.6074458	Bcer	H	Bcer	FALSE	NA	NA	NA
Bc_ctg1_1164	B3	35.73932972	Bcer	L	Bcer	FALSE	NA	NA	NA
Bc_ctg1_1165	B3	454.2389888	Bcer	L	Bcer	FALSE	NA	NA	NA
Bc_ctg1_1166	B3	293.0174068	Bcer	F	Bcer	FALSE	NA	NA	NA
Bc_ctg1_1167	B3	33.93545188	Bcer	K	Bcer	FALSE	NA	NA	NA
Bc_ctg1_1168	B3	793.0297958	Bcer	S	Bcer	FALSE	NA	NA	NA
Bc_ctg1_1169	B3	1771.295297	Bcer	I	Bcer	FALSE	NA	NA	NA
Bc_ctg1_117	B3	1.578393111	Bcer	NA	Bcer	FALSE	Bc_Ga0417192_01_rn01	Other	NA
Bc_ctg1_1170	B3	230.5581365	Bcer	I	Bcer	FALSE	NA	NA	NA
Bc_ctg1_1171	B3	0	Bcer	NA	Bcer	FALSE	NA	NA	NA
Bc_ctg1_1172	B3	0.45096946	Bcer	S	Bcer	FALSE	NA	NA	NA
Bc_ctg1_1173	B3	0.112742365	Bcer	S	Bcer	FALSE	NA	NA	NA
Bc_ctg1_1174	B3	0.90193892	Bcer	S	Bcer	FALSE	NA	NA	NA
Bc_ctg1_1175	B3	0.90193892	Bcer	S	Bcer	FALSE	NA	NA	NA
Bc_ctg1_1176	B3	0	Bcer	S	Bcer	FALSE	NA	NA	NA
Bc_ctg1_1177	B3	0.112742365	Bcer	S	Bcer	FALSE	NA	NA	NA
Bc_ctg1_1178	B3	0.112742365	Bcer	S	Bcer	FALSE	NA	NA	NA
Bc_ctg1_1179	B3	0.22548473	Bcer	S	Bcer	FALSE	NA	NA	NA

Bc_ctg1_118	B3	24.46509322	Bcer	EGP	Bcer	FALSE	Bc_Ga0417192_01_rn01	Transport	SMCOG1106
Bc_ctg1_1180	B3	0	Bcer	S	Bcer	FALSE	NA	NA	NA
Bc_ctg1_1181	B3	3.382270952	Bcer	J	Bcer	FALSE	NA	NA	NA
Bc_ctg1_1182	B3	1.916620206	Bcer	NA	Bcer	FALSE	NA	NA	NA
Bc_ctg1_1183	B3	792.5788263	Bcer	J	Bcer	FALSE	NA	NA	NA
Bc_ctg1_1184	B3	470.6993741	Bcer	E	Bcer	FALSE	NA	NA	NA
Bc_ctg1_1185	B3	26.94542525	Bcer	E	Bcer	FALSE	NA	NA	NA
Bc_ctg1_1186	B3	0.67645419	Bcer	S	Bcer	FALSE	NA	NA	NA
Bc_ctg1_1187	B3	108.3454128	Bcer	S	Bcer	FALSE	NA	NA	NA
Bc_ctg1_1188	B3	43.40581054	Bcer	NA	Bcer	FALSE	NA	NA	NA
Bc_ctg1_1189	B3	39.00885831	Bcer	S	Bcer	FALSE	NA	NA	NA
Bc_ctg1_119	B3	10.59778231	Bcer	K	Bcer	FALSE	Bc_Ga0417192_01_rn01	Other	NA
Bc_ctg1_1190	B3	27.7346218	Bcer	K	Bcer	FALSE	NA	NA	NA
Bc_ctg1_1191	B3	0.112742365	Bcer	L	Bcer	FALSE	NA	NA	NA
Bc_ctg1_1192	B3	5.186148792	Bcer	K	Bcer	FALSE	NA	NA	NA
Bc_ctg1_1193	B3	8.230192649	Bcer	S	Bcer	FALSE	NA	NA	NA
Bc_ctg1_1194	B3	21.75927646	Bcer	NU	Bcer	FALSE	NA	NA	NA
Bc_ctg1_1195	B3	59.52796875	Bcer	H	Bcer	FALSE	NA	NA	NA
Bc_ctg1_1196	B3	103.9484606	Bcer	P	Bcer	FALSE	NA	NA	NA
Bc_ctg1_1197	B3	0.338227095	Bcer	NA	Bcer	FALSE	NA	NA	NA
Bc_ctg1_1198	B3	34.61190607	Bcer	K	Bcer	FALSE	NA	NA	NA
Bc_ctg1_1199	B3	38.44514648	Bcer	K	Bcer	FALSE	NA	NA	NA
Bc_ctg1_120	B3	34.16093661	Bcer	K	Bcer	FALSE	Bc_Ga0417192_01_rn01	Other	NA
Bc_ctg1_1200	B3	4.058725142	Bcer	P	Bcer	FALSE	NA	NA	NA
Bc_ctg1_1201	B3	19.16620206	Bcer	P	Bcer	FALSE	NA	NA	NA
Bc_ctg1_1202	B3	5.073406427	Bcer	NA	Bcer	FALSE	NA	NA	NA
Bc_ctg1_1203	B3	0	Bcer	NA	Bcer	FALSE	NA	NA	NA
Bc_ctg1_1204	B3	0.563711825	Bcer	S	Bcer	FALSE	NA	NA	NA
Bc_ctg1_1205	B3	1.014681285	Bcer	S	Bcer	FALSE	NA	NA	NA
Bc_ctg1_1206	B3	1.352908381	Bcer	S	Bcer	FALSE	NA	NA	NA
Bc_ctg1_1207	B3	7.553738458	Bcer	S	Bcer	FALSE	NA	NA	NA
Bc_ctg1_1208	B3	6.426314808	Bcer	V	Bcer	FALSE	NA	NA	NA
Bc_ctg1_1209	B3	0.45096946	Bcer	K	Bcer	FALSE	NA	NA	NA
Bc_ctg1_121	B3	39.3470854	Bcer	C	Bcer	FALSE	Bc_Ga0417192_01_rn01	Ancillary biosynthesis	SMCOG1007
Bc_ctg1_1210	B3	2.705816761	Bcer	NA	Bcer	FALSE	NA	NA	NA
Bc_ctg1_1211	B3	0.563711825	Bcer	S	Bcer	FALSE	NA	NA	NA
Bc_ctg1_1212	B3	3331.424145	Bcer	E	Bcer	FALSE	NA	NA	NA
Bc_ctg1_1213	B3	3201.883167	Bcer	E	Bcer	FALSE	NA	NA	NA
Bc_ctg1_1214	B3	1174.09899	Bcer	E	Bcer	FALSE	NA	NA	NA
Bc_ctg1_1215	B3	1.127423651	Bcer	L	Bcer	FALSE	NA	NA	NA
Bc_ctg1_1216	B3	0.789196555	Bcer	S	Bcer	FALSE	NA	NA	NA
Bc_ctg1_1218	B3	1.240166016	Bcer	NA	Bcer	FALSE	NA	NA	NA
Bc_ctg1_1219	B3	23.11218484	Bcer	NA	Bcer	FALSE	NA	NA	NA
Bc_ctg1_122	B3	22.21024592	Bcer	S	Bcer	FALSE	Bc_Ga0417192_01_rn01	Core biosynthesis	None
Bc_ctg1_1220	B3	0.67645419	Bcer	S	Bcer	FALSE	NA	NA	NA
Bc_ctg1_1221	B3	7.553738458	Bcer	F	Bcer	FALSE	NA	NA	NA
Bc_ctg1_1222	B3	75.08641512	Bcer	O	Bcer	FALSE	NA	NA	NA
Bc_ctg1_1223	B3	0.22548473	Bcer	S	Bcer	FALSE	Bc_Ga0417192_01_rn10	Other	NA
Bc_ctg1_1224	B3	0	Bcer	U	Bcer	FALSE	Bc_Ga0417192_01_rn10	Other	NA
Bc_ctg1_1225	B3	0	Bcer	NA	Bcer	FALSE	Bc_Ga0417192_01_rn10	Other	NA
Bc_ctg1_1226	B3	0.112742365	Bcer	NU	Bcer	FALSE	Bc_Ga0417192_01_rn10	Other	NA
Bc_ctg1_1227	B3	0.338227095	Bcer	U	Bcer	FALSE	Bc_Ga0417192_01_rn10	Other	NA
Bc_ctg1_1228	B3	2.480332031	Bcer	NU	Bcer	FALSE	Bc_Ga0417192_01_rn10	Other	NA
Bc_ctg1_1229	B3	1.916620206	Bcer	NU	Bcer	FALSE	Bc_Ga0417192_01_rn10	Other	NA
Bc_ctg1_123	B3	25.70525923	Bcer	NT	Bcer	FALSE	Bc_Ga0417192_01_rn01	Other	SMCOG1061
Bc_ctg1_1230	B3	438.1168306	Bcer	K	Bcer	FALSE	Bc_Ga0417192_01_rn10	Other	NA
Bc_ctg1_1231	B3	5.073406427	Bcer	S	Bcer	FALSE	Bc_Ga0417192_01_rn10	Other	NA
Bc_ctg1_1232	B3	106.9925044	Bcer	U	Bcer	FALSE	Bc_Ga0417192_01_rn10	Transport	SMCOG1212
Bc_ctg1_1233	B3	0	Bcer	NA	Bcer	FALSE	Bc_Ga0417192_01_rn10	Other	NA
Bc_ctg1_1234	B3	0.67645419	Bcer	S	Bcer	FALSE	Bc_Ga0417192_01_rn10	Other	NA
Bc_ctg1_1235	B3	44.42049183	Bcer	NA	Bcer	FALSE	Bc_Ga0417192_01_rn10	Other	NA
Bc_ctg1_1236	B3	35.40110263	Bcer	NA	Bcer	FALSE	Bc_Ga0417192_01_rn10	Other	NA

Bc_ctg1_1237	B3	14.09279563	Bcer	NA	Bcer	FALSE	Bc_Ga0417192_01_rn10	Other	NA
Bc_ctg1_1238	B3	17.13683949	Bcer	S	Bcer	FALSE	Bc_Ga0417192_01_rn10	Other	NA
Bc_ctg1_1239	B3	80.72353338	Bcer	M	Bcer	FALSE	Bc_Ga0417192_01_rn10	Core biosynthesis	None
Bc_ctg1_124	B3	0	Bcer	NA	Bcer	FALSE	Bc_Ga0417192_01_rn01	Other	NA
Bc_ctg1_1240	B3	44.42049183	Bcer	E	Bcer	FALSE	Bc_Ga0417192_01_rn10	Other	NA
Bc_ctg1_1241	B3	17.58780895	Bcer	E	Bcer	FALSE	Bc_Ga0417192_01_rn10	Other	NA
Bc_ctg1_1242	B3	4.735179332	Bcer	E	Bcer	FALSE	Bc_Ga0417192_01_rn10	Core biosynthesis	SMCOG1168
Bc_ctg1_1243	B3	7.102768998	Bcer	E	Bcer	FALSE	Bc_Ga0417192_01_rn10	Ancillary biosynthesis	SMCOG1168
Bc_ctg1_1244	B3	10.37229758	Bcer	Q	Bcer	TRUE	Bc_Ga0417192_01_rn10	Other	NA
Bc_ctg1_1245	B3	109.2473517	Bcer	K	Bcer	FALSE	Bc_Ga0417192_01_rn10	Regulation	SMCOG1058
Bc_ctg1_1246	B3	91.09583096	Bcer	S	Bcer	FALSE	Bc_Ga0417192_01_rn10	Other	NA
Bc_ctg1_1247	B3	44.87146129	Bcer	S	Bcer	FALSE	Bc_Ga0417192_01_rn10	Ancillary biosynthesis	SMCOG1170
Bc_ctg1_1248	B3	24.12686612	Bcer	S	Bcer	FALSE	Bc_Ga0417192_01_rn10	Other	NA
Bc_ctg1_1249	B3	190.5345969	Bcer	G	Bcer	FALSE	Bc_Ga0417192_01_rn10	Other	SMCOG1108
Bc_ctg1_125	B3	0.45096946	Bcer	L	Bcer	FALSE	Bc_Ga0417192_01_rn01	Other	NA
Bc_ctg1_1250	B3	16.4603853	Bcer	S	Bcer	FALSE	Bc_Ga0417192_01_rn10	Other	NA
Bc_ctg1_1251	B3	50.39583718	Bcer	H	Bcer	FALSE	Bc_Ga0417192_01_rn10	Other	NA
Bc_ctg1_1252	B3	25.36703214	Bcer	J	Bcer	FALSE	Bc_Ga0417192_01_rn10	Other	NA
Bc_ctg1_1253	B3	17.92603604	Bcer	GJM	Bcer	FALSE	Bc_Ga0417192_01_rn10	Ancillary biosynthesis	SMCOG1064
Bc_ctg1_1254	B3	2.367589666	Bcer	P	Bcer	FALSE	NA	NA	NA
Bc_ctg1_1255	B3	3.833240412	Bcer	P	Bcer	FALSE	NA	NA	NA
Bc_ctg1_1256	B3	0.67645419	Bcer	P	Bcer	FALSE	NA	NA	NA
Bc_ctg1_1257	B3	2.142104936	Bcer	P	Bcer	FALSE	NA	NA	NA
Bc_ctg1_1258	B3	2.029362571	Bcer	P	Bcer	FALSE	NA	NA	NA
Bc_ctg1_1259	B3	786.2652539	Bcer	M	Bcer	FALSE	NA	NA	NA
Bc_ctg1_126	B3	11.6124636	Bcer	V	Bcer	FALSE	Bc_Ga0417192_01_rn01	Ancillary biosynthesis	SMCOG1053
Bc_ctg1_1260	B3	2.705816761	Bcer	EGP	Bcer	FALSE	NA	NA	NA
Bc_ctg1_1261	B3	279.0373535	Bcer	P	Bcer	FALSE	NA	NA	NA
Bc_ctg1_1262	B3	157.9520534	Bcer	S	Bcer	FALSE	NA	NA	NA
Bc_ctg1_1263	B3	1.465650746	Bcer	M	Bcer	FALSE	NA	NA	NA
Bc_ctg1_1264	B3	430.4503498	Bcer	I	Bcer	FALSE	NA	NA	NA
Bc_ctg1_1265	B3	12.85262962	Bcer	P	Bcer	FALSE	NA	NA	NA
Bc_ctg1_1266	B3	70.57672052	Bcer	P	Bcer	FALSE	NA	NA	NA
Bc_ctg1_1267	B3	63.69943625	Bcer	P	Bcer	FALSE	NA	NA	NA
Bc_ctg1_1268	B3	168.0988663	Bcer	S	Bcer	FALSE	NA	NA	NA
Bc_ctg1_127	B3	5.975345348	Bcer	V	Bcer	FALSE	Bc_Ga0417192_01_rn01	Ancillary biosynthesis	SMCOG1053
Bc_ctg1_1270	B3	64.15040571	Bcer	L	Bcer	FALSE	NA	NA	NA
Bc_ctg1_1271	B3	352.6581179	Bcer	JKL	Bcer	FALSE	NA	NA	NA
Bc_ctg1_1272	B3	0	Bcer	NA	Bcer	FALSE	NA	NA	NA
Bc_ctg1_1273	B3	229.0924858	Bcer	IM	Bcer	FALSE	NA	NA	NA
Bc_ctg1_1274	B3	55.8074707	Bcer	S	Bcer	FALSE	NA	NA	NA
Bc_ctg1_1275	B3	17.02409712	Bcer	S	Bcer	FALSE	NA	NA	NA
Bc_ctg1_1276	B3	19.16620206	Bcer	C	Bcer	FALSE	NA	NA	NA
Bc_ctg1_1277	B3	680.1746884	Bcer	K	Bcer	FALSE	NA	NA	NA
Bc_ctg1_1278	B3	1305.556587	Bcer	L	Bcer	FALSE	NA	NA	NA
Bc_ctg1_1279	B3	552.212104	Bcer	S	Bcer	FALSE	NA	NA	NA
Bc_ctg1_128	B3	0.563711825	Bcer	S	Bcer	FALSE	Bc_Ga0417192_01_rn01	Other	NA
Bc_ctg1_1280	B3	415.1173881	Bcer	K	Bcer	FALSE	NA	NA	NA
Bc_ctg1_1281	B3	70.91494762	Bcer	L	Bcer	FALSE	NA	NA	NA
Bc_ctg1_1282	B3	0.22548473	Bcer	S	Bcer	FALSE	NA	NA	NA
Bc_ctg1_1283	B3	618.2791299	Bcer	S	Bcer	FALSE	NA	NA	NA
Bc_ctg1_1284	B3	117.2520597	Bcer	F	Bcer	FALSE	NA	NA	NA
Bc_ctg1_1285	B3	8.004707919	Bcer	M	Bcer	FALSE	NA	NA	NA
Bc_ctg1_1286	B3	19.50442915	Bcer	S	Bcer	FALSE	NA	NA	NA
Bc_ctg1_1287	B3	225.8229572	Bcer	S	Bcer	FALSE	NA	NA	NA
Bc_ctg1_1288	B3	296.0614506	Bcer	T	Bcer	FALSE	NA	NA	NA
Bc_ctg1_1289	B3	1.127423651	Bcer	S	Bcer	FALSE	NA	NA	NA
Bc_ctg1_129	B3	0.112742365	Bcer	S	Bcer	FALSE	Bc_Ga0417192_01_rn01	Other	NA
Bc_ctg1_1290	B3	0	Bcer	S	Bcer	FALSE	NA	NA	NA
Bc_ctg1_1291	B3	1.014681285	Bcer	S	Bcer	FALSE	NA	NA	NA
Bc_ctg1_1292	B3	997.0934765	Bcer	S	Bcer	FALSE	NA	NA	NA
Bc_ctg1_1293	B3	394.9365048	Bcer	J	Bcer	FALSE	NA	NA	NA

Bc_ctg1_1294	B3	310.4924734	Bcer	J	Bcer	FALSE	NA	NA	NA
Bc_ctg1_1295	B3	137.432943	Bcer	J	Bcer	FALSE	NA	NA	NA
Bc_ctg1_1296	B3	291.7772408	Bcer	J	Bcer	FALSE	NA	NA	NA
Bc_ctg1_1297	B3	181.5152077	Bcer	O	Bcer	FALSE	NA	NA	NA
Bc_ctg1_1298	B3	2100.84123	Bcer	O	Bcer	FALSE	NA	NA	NA
Bc_ctg1_1299	B3	366.2999441	Bcer	O	Bcer	FALSE	NA	NA	NA
Bc_ctg1_13	B3	32.13157404	Bcer	Q	Bcer	TRUE	NA	NA	NA
Bc_ctg1_130	B3	1.465650746	Bcer	K	Bcer	FALSE	Bc_Ga0417192_01_rn01	Regulation	SMCOG1284
Bc_ctg1_1300	B3	257.1653347	Bcer	K	Bcer	FALSE	NA	NA	NA
Bc_ctg1_1301	B3	36.64126864	Bcer	H	Bcer	FALSE	NA	NA	NA
Bc_ctg1_1302	B3	7.215511363	Bcer	K	Bcer	FALSE	NA	NA	NA
Bc_ctg1_1303	B3	492.3459082	Bcer	M	Bcer	FALSE	NA	NA	NA
Bc_ctg1_1304	B3	0.338227095	Bcer	NA	Bcer	FALSE	NA	NA	NA
Bc_ctg1_1305	B3	3.495013317	Bcer	C	Bcer	FALSE	NA	NA	NA
Bc_ctg1_1306	B3	193.3531561	Bcer	J	Bcer	FALSE	NA	NA	NA
Bc_ctg1_1307	B3	52.31245738	Bcer	L	Bcer	FALSE	NA	NA	NA
Bc_ctg1_1308	B3	0.112742365	Bcer	S	Bcer	FALSE	NA	NA	NA
Bc_ctg1_1309	B3	14.09279563	Bcer	S	Bcer	FALSE	NA	NA	NA
Bc_ctg1_1310	B3	37.20498047	Bcer	F	Bcer	FALSE	NA	NA	NA
Bc_ctg1_1311	B3	3.720498047	Bcer	L	Bcer	FALSE	NA	NA	NA
Bc_ctg1_1312	B3	0	Bcer	E	Bcer	FALSE	NA	NA	NA
Bc_ctg1_1313	B3	219.9603542	Bcer	Q	Bcer	TRUE	NA	NA	NA
Bc_ctg1_1314	B3	153.2168741	Bcer	J	Bcer	FALSE	NA	NA	NA
Bc_ctg1_1315	B3	89.40469549	Bcer	H	Bcer	FALSE	NA	NA	NA
Bc_ctg1_1316	B3	123.9038592	Bcer	H	Bcer	FALSE	NA	NA	NA
Bc_ctg1_1317	B3	37.76869229	Bcer	J	Bcer	FALSE	NA	NA	NA
Bc_ctg1_1318	B3	184.8974787	Bcer	E	Bcer	FALSE	NA	NA	NA
Bc_ctg1_1319	B3	233.6021804	Bcer	S	Bcer	FALSE	NA	NA	NA
Bc_ctg1_1320	B3	73.73350674	Bcer	S	Bcer	FALSE	NA	NA	NA
Bc_ctg1_1321	B3	0.112742365	Bcer	NA	Bcer	FALSE	NA	NA	NA
Bc_ctg1_1322	B3	68.20913086	Bcer	I	Bcer	FALSE	NA	NA	NA
Bc_ctg1_1323	B3	2.029362571	Bcer	K	Bcer	FALSE	NA	NA	NA
Bc_ctg1_1324	B3	2.593074396	Bcer	NA	Bcer	FALSE	NA	NA	NA
Bc_ctg1_1325	B3	6.764541903	Bcer	K	Bcer	FALSE	NA	NA	NA
Bc_ctg1_1327	B3	3.382270952	Bcer	C	Bcer	FALSE	NA	NA	NA
Bc_ctg1_1328	B3	372.8390012	Bcer	S	Bcer	FALSE	NA	NA	NA
Bc_ctg1_1329	B3	0	Bcer	NA	Bcer	FALSE	NA	NA	NA
Bc_ctg1_133	B3	0.338227095	Bcer	NA	Bcer	FALSE	NA	NA	NA
Bc_ctg1_1330	B3	0.22548473	Bcer	P	Bcer	FALSE	NA	NA	NA
Bc_ctg1_1331	B3	9.357616299	Bcer	K	Bcer	FALSE	NA	NA	NA
Bc_ctg1_1332	B3	15.10747692	Bcer	I	Bcer	FALSE	NA	NA	NA
Bc_ctg1_1333	B3	22.66121538	Bcer	S	Bcer	FALSE	NA	NA	NA
Bc_ctg1_1334	B3	0.112742365	Bcer	NA	Bcer	FALSE	NA	NA	NA
Bc_ctg1_1335	B3	5.073406427	Bcer	G	Bcer	FALSE	NA	NA	NA
Bc_ctg1_1336	B3	8.568419744	Bcer	NA	Bcer	FALSE	NA	NA	NA
Bc_ctg1_1338	B3	3.833240412	Bcer	U	Bcer	FALSE	NA	NA	NA
Bc_ctg1_1339	B3	13.30359908	Bcer	O	Bcer	FALSE	NA	NA	NA
Bc_ctg1_134	B3	0.112742365	Bcer	NA	Bcer	FALSE	NA	NA	NA
Bc_ctg1_1340	B3	4.396952237	Bcer	E	Bcer	FALSE	NA	NA	NA
Bc_ctg1_1341	B3	0.789196555	Bcer	H	Bcer	FALSE	NA	NA	NA
Bc_ctg1_1342	B3	13.41634144	Bcer	S	Bcer	FALSE	NA	NA	NA
Bc_ctg1_1343	B3	1.127423651	Bcer	S	Bcer	FALSE	NA	NA	NA
Bc_ctg1_1344	B3	11.6124636	Bcer	K	Bcer	FALSE	NA	NA	NA
Bc_ctg1_1345	B3	0.90193892	Bcer	I	Bcer	FALSE	NA	NA	NA
Bc_ctg1_1346	B3	3.833240412	Bcer	K	Bcer	FALSE	NA	NA	NA
Bc_ctg1_1347	B3	2.029362571	Bcer	J	Bcer	FALSE	NA	NA	NA
Bc_ctg1_1348	B3	3.269528587	Bcer	C	Bcer	FALSE	NA	NA	NA
Bc_ctg1_1349	B3	20.29362571	Bcer	HP	Bcer	FALSE	NA	NA	NA
Bc_ctg1_135	B3	4.960664062	Bcer	T	Bcer	FALSE	NA	NA	NA
Bc_ctg1_1350	B3	27.50913707	Bcer	P	Bcer	FALSE	NA	NA	NA
Bc_ctg1_1351	B3	42.50387162	Bcer	P	Bcer	FALSE	NA	NA	NA
Bc_ctg1_1352	B3	0	Bcer	S	Bcer	FALSE	NA	NA	NA

Bc_ctg1_1353	B3	5315.577027	Bcer	C	Bcer	FALSE	NA	NA	NA
Bc_ctg1_1354	B3	530.3400852	Bcer	E	Bcer	FALSE	NA	NA	NA
Bc_ctg1_1355	B3	229.9944247	Bcer	E	Bcer	FALSE	NA	NA	NA
Bc_ctg1_1356	B3	342.6240474	Bcer	E	Bcer	FALSE	NA	NA	NA
Bc_ctg1_1357	B3	110.7130025	Bcer	Q	Bcer	TRUE	NA	NA	NA
Bc_ctg1_1358	B3	1.240166016	Bcer	S	Bcer	FALSE	NA	NA	NA
Bc_ctg1_1359	B3	352.6581179	Bcer	S	Bcer	FALSE	NA	NA	NA
Bc_ctg1_136	B3	12.96537198	Bcer	T	Bcer	FALSE	NA	NA	NA
Bc_ctg1_1360	B3	1.014681285	Bcer	M	Bcer	FALSE	NA	NA	NA
Bc_ctg1_1363	B3	193.8041255	Bcer	K	Bcer	FALSE	NA	NA	NA
Bc_ctg1_1364	B3	112.404138	Bcer	F	Bcer	FALSE	NA	NA	NA
Bc_ctg1_1365	B3	216.5780833	Bcer	O	Bcer	FALSE	NA	NA	NA
Bc_ctg1_1366	B3	38.55788885	Bcer	O	Bcer	FALSE	NA	NA	NA
Bc_ctg1_1367	B3	24.69057795	Bcer	S	Bcer	FALSE	NA	NA	NA
Bc_ctg1_1368	B3	148.3689524	Bcer	S	Bcer	FALSE	NA	NA	NA
Bc_ctg1_1369	B3	80.94901811	Bcer	S	Bcer	FALSE	NA	NA	NA
Bc_ctg1_137	B3	1.916620206	Bcer	NA	Bcer	FALSE	NA	NA	NA
Bc_ctg1_1370	B3	442.4010405	Bcer	L	Bcer	FALSE	NA	NA	NA
Bc_ctg1_1371	B3	348.7121351	Bcer	S	Bcer	FALSE	NA	NA	NA
Bc_ctg1_1372	B3	803.1766086	Bcer	J	Bcer	FALSE	Bc_Ga0417192_01_rn11	Other	NA
Bc_ctg1_1373	B3	2.818559126	Bcer	S	Bcer	FALSE	Bc_Ga0417192_01_rn11	Other	NA
Bc_ctg1_1374	B3	0.563711825	Bcer	S	Bcer	FALSE	Bc_Ga0417192_01_rn11	Other	NA
Bc_ctg1_1375	B3	0	Bcer	NA	Bcer	FALSE	Bc_Ga0417192_01_rn11	Other	NA
Bc_ctg1_1377	B3	8.230192649	Bcer	NA	Bcer	FALSE	Bc_Ga0417192_01_rn11	Other	NA
Bc_ctg1_1378	B3	95.04181374	Bcer	L	Bcer	FALSE	Bc_Ga0417192_01_rn11	Other	NA
Bc_ctg1_1379	B3	50.73406427	Bcer	S	Bcer	FALSE	Bc_Ga0417192_01_rn11	Other	SMCOG1272
Bc_ctg1_138	B3	78.24320135	Bcer	S	Bcer	FALSE	NA	NA	NA
Bc_ctg1_1380	B3	459.9888494	Bcer	J	Bcer	FALSE	Bc_Ga0417192_01_rn11	Other	NA
Bc_ctg1_1381	B3	394.9365048	Bcer	E	Bcer	FALSE	Bc_Ga0417192_01_rn11	Core biosynthesis	SMCOG1139
Bc_ctg1_1382	B3	88.61549893	Bcer	K	Bcer	FALSE	Bc_Ga0417192_01_rn11	Other	NA
Bc_ctg1_1383	B3	30.55318093	Bcer	L	Bcer	FALSE	Bc_Ga0417192_01_rn11	Other	NA
Bc_ctg1_1384	B3	26.83268288	Bcer	S	Bcer	FALSE	Bc_Ga0417192_01_rn11	Other	NA
Bc_ctg1_1385	B3	234.9550888	Bcer	H	Bcer	FALSE	Bc_Ga0417192_01_rn11	Ancillary biosynthesis	None
Bc_ctg1_1386	B3	428.4209872	Bcer	J	Bcer	FALSE	Bc_Ga0417192_01_rn11	Other	NA
Bc_ctg1_1387	B3	219.7348695	Bcer	J	Bcer	FALSE	Bc_Ga0417192_01_rn11	Other	SMCOG1162
Bc_ctg1_1388	B3	0	Bcer	NA	Bcer	FALSE	Bc_Ga0417192_01_rn11	Other	NA
Bc_ctg1_1389	B3	67.7581614	Bcer	J	Bcer	FALSE	Bc_Ga0417192_01_rn11	Other	NA
Bc_ctg1_139	B3	1.240166016	Bcer	S	Bcer	FALSE	NA	NA	NA
Bc_ctg1_1390	B3	624.0289906	Bcer	KT	Bcer	FALSE	Bc_Ga0417192_01_rn11	Other	NA
Bc_ctg1_1391	B3	198.7647896	Bcer	F	Bcer	FALSE	Bc_Ga0417192_01_rn11	Other	NA
Bc_ctg1_1392	B3	333.6046582	Bcer	L	Bcer	FALSE	Bc_Ga0417192_01_rn11	Other	NA
Bc_ctg1_1393	B3	7.328253728	Bcer	P	Bcer	FALSE	Bc_Ga0417192_01_rn11	Other	NA
Bc_ctg1_1394	B3	992.5837819	Bcer	U	Bcer	FALSE	Bc_Ga0417192_01_rn11	Other	NA
Bc_ctg1_1395	B3	16.79861239	Bcer	G	Bcer	FALSE	Bc_Ga0417192_01_rn11	Other	NA
Bc_ctg1_1396	B3	0	Bcer	S	Bcer	FALSE	Bc_Ga0417192_01_rn11	Core biosynthesis	SMCOG1086
Bc_ctg1_1397	B3	3.607755682	Bcer	S	Bcer	FALSE	Bc_Ga0417192_01_rn11	Other	NA
Bc_ctg1_1398	B3	0	Bcer	S	Bcer	FALSE	Bc_Ga0417192_01_rn11	Other	NA
Bc_ctg1_1399	B3	78.24320135	Bcer	U	Bcer	FALSE	Bc_Ga0417192_01_rn11	Other	NA
Bc_ctg1_14	B3	0.563711825	Bcer	F	Bcer	FALSE	NA	NA	NA
Bc_ctg1_140	B3	2.480332031	Bcer	S	Bcer	FALSE	NA	NA	NA
Bc_ctg1_1400	B3	214.323236	Bcer	F	Bcer	FALSE	Bc_Ga0417192_01_rn11	Other	NA
Bc_ctg1_1401	B3	178.1329368	Bcer	J	Bcer	FALSE	Bc_Ga0417192_01_rn11	Other	NA
Bc_ctg1_1402	B3	1.691135476	Bcer	S	Bcer	FALSE	Bc_Ga0417192_01_rn11	Other	NA
Bc_ctg1_1403	B3	83.76757723	Bcer	L	Bcer	FALSE	Bc_Ga0417192_01_rn11	Other	NA
Bc_ctg1_1404	B3	45.54791548	Bcer	L	Bcer	FALSE	Bc_Ga0417192_01_rn11	Other	NA
Bc_ctg1_1405	B3	16.57312766	Bcer	P	Bcer	FALSE	Bc_Ga0417192_01_rn11	Transport	SMCOG1033
Bc_ctg1_1406	B3	0.789196555	Bcer	S	Bcer	FALSE	Bc_Ga0417192_01_rn11	Other	NA
Bc_ctg1_1407	B3	24.12686612	Bcer	NA	Bcer	FALSE	Bc_Ga0417192_01_rn11	Other	NA
Bc_ctg1_1408	B3	1.352908381	Bcer	S	Bcer	FALSE	Bc_Ga0417192_01_rn11	Other	NA
Bc_ctg1_1409	B3	0.112742365	Bcer	M	Bcer	FALSE	NA	NA	NA
Bc_ctg1_141	B3	1.127423651	Bcer	S	Bcer	FALSE	NA	NA	NA
Bc_ctg1_1410	B3	4.509694602	Bcer	H	Bcer	FALSE	NA	NA	NA

Bc_ctg1_1411	B3	1.240166016	Bcer	H	Bcer	FALSE	NA	NA	NA
Bc_ctg1_1412	B3	1.803877841	Bcer	H	Bcer	FALSE	NA	NA	NA
Bc_ctg1_1413	B3	11.72520597	Bcer	E	Bcer	FALSE	NA	NA	NA
Bc_ctg1_1414	B3	34.95013317	Bcer	S	Bcer	FALSE	NA	NA	NA
Bc_ctg1_1415	B3	55.8074707	Bcer	S	Bcer	FALSE	NA	NA	NA
Bc_ctg1_1416	B3	82.30192649	Bcer	E	Bcer	FALSE	NA	NA	NA
Bc_ctg1_1417	B3	6.651799538	Bcer	V	Bcer	FALSE	NA	NA	NA
Bc_ctg1_1418	B3	51.07229137	Bcer	V	Bcer	FALSE	NA	NA	NA
Bc_ctg1_1419	B3	9.357616299	Bcer	V	Bcer	FALSE	NA	NA	NA
Bc_ctg1_142	B3	34.61190607	Bcer	T	Bcer	FALSE	NA	NA	NA
Bc_ctg1_1420	B3	19.16620206	Bcer	T	Bcer	FALSE	NA	NA	NA
Bc_ctg1_1421	B3	14.54376509	Bcer	T	Bcer	FALSE	NA	NA	NA
Bc_ctg1_1422	B3	393.0198846	Bcer	S	Bcer	FALSE	NA	NA	NA
Bc_ctg1_1423	B3	174.0742116	Bcer	T	Bcer	FALSE	NA	NA	NA
Bc_ctg1_1424	B3	975.4469424	Bcer	J	Bcer	FALSE	NA	NA	NA
Bc_ctg1_1425	B3	2072.317412	Bcer	J	Bcer	FALSE	NA	NA	NA
Bc_ctg1_1426	B3	467.2043608	Bcer	J	Bcer	FALSE	NA	NA	NA
Bc_ctg1_1427	B3	30.77866566	Bcer	J	Bcer	FALSE	NA	NA	NA
Bc_ctg1_1428	B3	6.313572443	Bcer	S	Bcer	FALSE	NA	NA	NA
Bc_ctg1_1429	B3	3.833240412	Bcer	M	Bcer	FALSE	NA	NA	NA
Bc_ctg1_143	B3	1.240166016	Bcer	V	Bcer	FALSE	NA	NA	NA
Bc_ctg1_1430	B3	603.171653	Bcer	D	Bcer	FALSE	NA	NA	NA
Bc_ctg1_1431	B3	494.0370437	Bcer	D	Bcer	FALSE	NA	NA	NA
Bc_ctg1_1432	B3	200.7941522	Bcer	M	Bcer	FALSE	NA	NA	NA
Bc_ctg1_1433	B3	545.5603045	Bcer	M	Bcer	FALSE	NA	NA	NA
Bc_ctg1_1434	B3	300.9093723	Bcer	D	Bcer	FALSE	NA	NA	NA
Bc_ctg1_1435	B3	26.60719815	Bcer	L	Bcer	FALSE	NA	NA	NA
Bc_ctg1_1437	B3	1.691135476	Bcer	E	Bcer	FALSE	NA	NA	NA
Bc_ctg1_1438	B3	24.57783558	Bcer	D	Bcer	FALSE	NA	NA	NA
Bc_ctg1_1439	B3	1.240166016	Bcer	NA	Bcer	FALSE	NA	NA	NA
Bc_ctg1_144	B3	0.45096946	Bcer	V	Bcer	FALSE	NA	NA	NA
Bc_ctg1_1440	B3	61.55733132	Bcer	H	Bcer	FALSE	NA	NA	NA
Bc_ctg1_1441	B3	700.0173446	Bcer	J	Bcer	FALSE	NA	NA	NA
Bc_ctg1_1442	B3	3.720498047	Bcer	S	Bcer	FALSE	NA	NA	NA
Bc_ctg1_1443	B3	0.112742365	Bcer	M	Bcer	FALSE	NA	NA	NA
Bc_ctg1_1444	B3	408.2401039	Bcer	H	Bcer	FALSE	NA	NA	NA
Bc_ctg1_1445	B3	141.153441	Bcer	H	Bcer	FALSE	NA	NA	NA
Bc_ctg1_1446	B3	56.70940962	Bcer	H	Bcer	FALSE	NA	NA	NA
Bc_ctg1_1447	B3	130.2174316	Bcer	H	Bcer	FALSE	NA	NA	NA
Bc_ctg1_1448	B3	36.41578391	Bcer	O	Bcer	FALSE	NA	NA	NA
Bc_ctg1_1449	B3	227.9650621	Bcer	H	Bcer	FALSE	NA	NA	NA
Bc_ctg1_145	B3	1.352908381	Bcer	T	Bcer	FALSE	NA	NA	NA
Bc_ctg1_1450	B3	14.54376509	Bcer	K	Bcer	FALSE	NA	NA	NA
Bc_ctg1_1451	B3	48.93018643	Bcer	O	Bcer	FALSE	NA	NA	NA
Bc_ctg1_1452	B3	248.4841726	Bcer	D	Bcer	FALSE	NA	NA	NA
Bc_ctg1_1453	B3	586.5985254	Bcer	O	Bcer	FALSE	NA	NA	NA
Bc_ctg1_1454	B3	31.22963512	Bcer	LO	Bcer	FALSE	NA	NA	NA
Bc_ctg1_1455	B3	2644.146688	Bcer	O	Bcer	FALSE	NA	NA	NA
Bc_ctg1_1456	B3	2376.721798	Bcer	D	Bcer	FALSE	NA	NA	NA
Bc_ctg1_1457	B3	233.0384686	Bcer	O	Bcer	FALSE	NA	NA	NA
Bc_ctg1_1458	B3	7.102768998	Bcer	NA	Bcer	FALSE	NA	NA	NA
Bc_ctg1_146	B3	1.352908381	Bcer	T	Bcer	FALSE	NA	NA	NA
Bc_ctg1_1460	B3	110.1492907	Bcer	S	Bcer	FALSE	NA	NA	NA
Bc_ctg1_1461	B3	0.112742365	Bcer	NA	Bcer	FALSE	NA	NA	NA
Bc_ctg1_1462	B3	0.90193892	Bcer	NA	Bcer	FALSE	NA	NA	NA
Bc_ctg1_1463	B3	106.4287926	Bcer	S	Bcer	FALSE	NA	NA	NA
Bc_ctg1_1464	B3	91.09583096	Bcer	F	Bcer	FALSE	NA	NA	NA
Bc_ctg1_1465	B3	36.19029918	Bcer	J	Bcer	FALSE	NA	NA	NA
Bc_ctg1_1466	B3	1.352908381	Bcer	S	Bcer	FALSE	NA	NA	NA
Bc_ctg1_1467	B3	88.16452947	Bcer	M	Bcer	FALSE	NA	NA	NA
Bc_ctg1_1468	B3	3.156786221	Bcer	EGP	Bcer	FALSE	NA	NA	NA
Bc_ctg1_1469	B3	3.495013317	Bcer	S	Bcer	FALSE	NA	NA	NA

Bc_ctg1_147	B3	3.044043856	Bcer	V	Bcer	FALSE	NA	NA	NA
Bc_ctg1_1470	B3	5.524375888	Bcer	K	Bcer	FALSE	NA	NA	NA
Bc_ctg1_1471	B3	0	Bcer	S	Bcer	FALSE	NA	NA	NA
Bc_ctg1_1472	B3	0.67645419	Bcer	K	Bcer	FALSE	NA	NA	NA
Bc_ctg1_1473	B3	6.088087713	Bcer	F	Bcer	FALSE	NA	NA	NA
Bc_ctg1_1474	B3	24.57783558	Bcer	F	Bcer	FALSE	NA	NA	NA
Bc_ctg1_1475	B3	1.352908381	Bcer	KT	Bcer	FALSE	NA	NA	NA
Bc_ctg1_1476	B3	1.127423651	Bcer	K	Bcer	FALSE	NA	NA	NA
Bc_ctg1_1477	B3	4.960664062	Bcer	EG	Bcer	FALSE	NA	NA	NA
Bc_ctg1_1478	B3	5.749860618	Bcer	G	Bcer	FALSE	NA	NA	NA
Bc_ctg1_1479	B3	106.8797621	Bcer	K	Bcer	FALSE	NA	NA	NA
Bc_ctg1_148	B3	3.269528587	Bcer	S	Bcer	FALSE	NA	NA	NA
Bc_ctg1_1480	B3	62.00830078	Bcer	CP	Bcer	FALSE	NA	NA	NA
Bc_ctg1_1481	B3	16.57312766	Bcer	S	Bcer	FALSE	NA	NA	NA
Bc_ctg1_1482	B3	78.35594371	Bcer	M	Bcer	FALSE	NA	NA	NA
Bc_ctg1_1483	B3	228.1905469	Bcer	N	Bcer	FALSE	NA	NA	NA
Bc_ctg1_1484	B3	219.0584153	Bcer	N	Bcer	FALSE	NA	NA	NA
Bc_ctg1_1485	B3	30.55318093	Bcer	M	Bcer	FALSE	NA	NA	NA
Bc_ctg1_1486	B3	33.93545188	Bcer	S	Bcer	FALSE	NA	NA	NA
Bc_ctg1_1487	B3	1572.64325	Bcer	C	Bcer	FALSE	NA	NA	NA
Bc_ctg1_1488	B3	3553.526604	Bcer	C	Bcer	FALSE	NA	NA	NA
Bc_ctg1_1489	B3	609.8234526	Bcer	C	Bcer	FALSE	NA	NA	NA
Bc_ctg1_149	B3	45.32243075	Bcer	T	Bcer	FALSE	NA	NA	NA
Bc_ctg1_1490	B3	120.9725577	Bcer	S	Bcer	FALSE	NA	NA	NA
Bc_ctg1_1491	B3	61.67007368	Bcer	L	Bcer	FALSE	NA	NA	NA
Bc_ctg1_1492	B3	90.30663441	Bcer	O	Bcer	FALSE	NA	NA	NA
Bc_ctg1_1493	B3	662.2486523	Bcer	C	Bcer	FALSE	NA	NA	NA
Bc_ctg1_1494	B3	606.4411816	Bcer	C	Bcer	FALSE	NA	NA	NA
Bc_ctg1_1495	B3	505.0857954	Bcer	I	Bcer	FALSE	NA	NA	NA
Bc_ctg1_1496	B3	386.2553427	Bcer	K	Bcer	FALSE	NA	NA	NA
Bc_ctg1_1497	B3	39.3470854	Bcer	IQ	Bcer	TRUE	NA	NA	NA
Bc_ctg1_1498	B3	0.112742365	Bcer	Q	Bcer	TRUE	NA	NA	NA
Bc_ctg1_1499	B3	51.74874556	Bcer	P	Bcer	FALSE	NA	NA	NA
Bc_ctg1_15	B3	0.22548473	Bcer	V	Bcer	FALSE	NA	NA	NA
Bc_ctg1_150	B3	6.200830078	Bcer	C	Bcer	FALSE	NA	NA	NA
Bc_ctg1_1500	B3	29.20027255	Bcer	P	Bcer	FALSE	NA	NA	NA
Bc_ctg1_1501	B3	2.029362571	Bcer	NA	Bcer	FALSE	NA	NA	NA
Bc_ctg1_1502	B3	80.94901811	Bcer	S	Bcer	FALSE	NA	NA	NA
Bc_ctg1_1503	B3	8.230192649	Bcer	NA	Bcer	FALSE	NA	NA	NA
Bc_ctg1_1504	B3	42.72935635	Bcer	S	Bcer	FALSE	NA	NA	NA
Bc_ctg1_1505	B3	29.87672674	Bcer	S	Bcer	FALSE	NA	NA	NA
Bc_ctg1_1506	B3	21.98476119	Bcer	S	Bcer	FALSE	NA	NA	NA
Bc_ctg1_1507	B3	2.254847301	Bcer	NA	Bcer	FALSE	NA	NA	NA
Bc_ctg1_1508	B3	23.5631543	Bcer	V	Bcer	FALSE	NA	NA	NA
Bc_ctg1_1509	B3	68.09638849	Bcer	T	Bcer	FALSE	NA	NA	NA
Bc_ctg1_151	B3	3.495013317	Bcer	EGP	Bcer	FALSE	NA	NA	NA
Bc_ctg1_1510	B3	42.50387162	Bcer	T	Bcer	FALSE	NA	NA	NA
Bc_ctg1_1511	B3	0.338227095	Bcer	NA	Bcer	FALSE	NA	NA	NA
Bc_ctg1_1512	B3	0.338227095	Bcer	S	Bcer	FALSE	NA	NA	NA
Bc_ctg1_1513	B3	70.91494762	Bcer	J	Bcer	FALSE	NA	NA	NA
Bc_ctg1_1514	B3	7.102768998	Bcer	C	Bcer	FALSE	NA	NA	NA
Bc_ctg1_1515	B3	4.847921697	Bcer	S	Bcer	FALSE	NA	NA	NA
Bc_ctg1_1516	B3	7.553738458	Bcer	HP	Bcer	FALSE	NA	NA	NA
Bc_ctg1_1517	B3	4.396952237	Bcer	P	Bcer	FALSE	NA	NA	NA
Bc_ctg1_1518	B3	6.426314808	Bcer	P	Bcer	FALSE	NA	NA	NA
Bc_ctg1_1519	B3	48.02824751	Bcer	M	Bcer	FALSE	NA	NA	NA
Bc_ctg1_1520	B3	14.205538	Bcer	M	Bcer	FALSE	NA	NA	NA
Bc_ctg1_1521	B3	24.46509322	Bcer	M	Bcer	FALSE	NA	NA	NA
Bc_ctg1_1522	B3	43.18032581	Bcer	E	Bcer	FALSE	NA	NA	NA
Bc_ctg1_1523	B3	7.553738458	Bcer	J	Bcer	FALSE	NA	NA	NA
Bc_ctg1_1525	B3	292.6791797	Bcer	L	Bcer	FALSE	NA	NA	NA
Bc_ctg1_1526	B3	108.5708975	Bcer	L	Bcer	FALSE	NA	NA	NA

Bc_ctg1_1527	B3	68.32187322	Bcer	S	Bcer	FALSE	NA	NA	NA
Bc_ctg1_1528	B3	29.20027255	Bcer	D	Bcer	FALSE	NA	NA	NA
Bc_ctg1_1529	B3	19.05345969	Bcer	L	Bcer	FALSE	NA	NA	NA
Bc_ctg1_153	B3	31.34237748	Bcer	EU	Bcer	FALSE	NA	NA	NA
Bc_ctg1_1530	B3	5.975345348	Bcer	NA	Bcer	FALSE	NA	NA	NA
Bc_ctg1_1531	B3	4.058725142	Bcer	NA	Bcer	FALSE	NA	NA	NA
Bc_ctg1_1533	B3	370.2459268	Bcer	J	Bcer	FALSE	NA	NA	NA
Bc_ctg1_1534	B3	1037.567986	Bcer	J	Bcer	FALSE	NA	NA	NA
Bc_ctg1_1535	B3	327.6293128	Bcer	J	Bcer	FALSE	NA	NA	NA
Bc_ctg1_1536	B3	98.64956942	Bcer	J	Bcer	FALSE	NA	NA	NA
Bc_ctg1_1538	B3	24.57783558	Bcer	S	Bcer	FALSE	NA	NA	NA
Bc_ctg1_1539	B3	16.23490057	Bcer	S	Bcer	FALSE	NA	NA	NA
Bc_ctg1_154	B3	1.352908381	Bcer	NA	Bcer	FALSE	NA	NA	NA
Bc_ctg1_1540	B3	4.058725142	Bcer	NA	Bcer	FALSE	NA	NA	NA
Bc_ctg1_1541	B3	8.906646839	Bcer	NA	Bcer	FALSE	NA	NA	NA
Bc_ctg1_1542	B3	276.3315367	Bcer	EGP	Bcer	FALSE	NA	NA	NA
Bc_ctg1_1543	B3	67.30719194	Bcer	V	Bcer	FALSE	NA	NA	NA
Bc_ctg1_1544	B3	14.65650746	Bcer	K	Bcer	FALSE	NA	NA	NA
Bc_ctg1_1545	B3	1102.169361	Bcer	G	Bcer	FALSE	NA	NA	NA
Bc_ctg1_1546	B3	148.7071795	Bcer	S	Bcer	FALSE	NA	NA	NA
Bc_ctg1_1547	B3	364.1578391	Bcer	J	Bcer	FALSE	NA	NA	NA
Bc_ctg1_1548	B3	234.729604	Bcer	J	Bcer	FALSE	NA	NA	NA
Bc_ctg1_1549	B3	825.837824	Bcer	J	Bcer	FALSE	NA	NA	NA
Bc_ctg1_1550	B3	2321.816266	Bcer	J	Bcer	FALSE	NA	NA	NA
Bc_ctg1_1551	B3	0.338227095	Bcer	S	Bcer	FALSE	NA	NA	NA
Bc_ctg1_1552	B3	1.014681285	Bcer	S	Bcer	FALSE	NA	NA	NA
Bc_ctg1_1553	B3	0.112742365	Bcer	L	Bcer	FALSE	NA	NA	NA
Bc_ctg1_1554	B3	0.45096946	Bcer	L	Bcer	FALSE	NA	NA	NA
Bc_ctg1_1555	B3	62.23378551	Bcer	L	Bcer	FALSE	NA	NA	NA
Bc_ctg1_1556	B3	47.12630859	Bcer	L	Bcer	FALSE	NA	NA	NA
Bc_ctg1_1557	B3	26.26897106	Bcer	K	Bcer	FALSE	NA	NA	NA
Bc_ctg1_1558	B3	244.5381898	Bcer	E	Bcer	FALSE	NA	NA	NA
Bc_ctg1_1559	B3	2317.306571	Bcer	G	Bcer	FALSE	NA	NA	NA
Bc_ctg1_156	B3	31.79334694	Bcer	E	Bcer	FALSE	NA	NA	NA
Bc_ctg1_1560	B3	10.25955522	Bcer	F	Bcer	FALSE	NA	NA	NA
Bc_ctg1_1561	B3	76.10109641	Bcer	P	Bcer	FALSE	NA	NA	NA
Bc_ctg1_1562	B3	200.5686674	Bcer	L	Bcer	FALSE	NA	NA	NA
Bc_ctg1_1563	B3	364.3833238	Bcer	L	Bcer	FALSE	NA	NA	NA
Bc_ctg1_1564	B3	124.0166016	Bcer	T	Bcer	FALSE	NA	NA	NA
Bc_ctg1_1565	B3	69.22381214	Bcer	T	Bcer	FALSE	NA	NA	NA
Bc_ctg1_1566	B3	0	Bcer	NA	Bcer	FALSE	NA	NA	NA
Bc_ctg1_1567	B3	0	Bcer	NA	Bcer	FALSE	NA	NA	NA
Bc_ctg1_1568	B3	61.33184659	Bcer	I	Bcer	FALSE	NA	NA	NA
Bc_ctg1_1569	B3	1963.859257	Bcer	C	Bcer	FALSE	NA	NA	NA
Bc_ctg1_157	B3	12.62714489	Bcer	S	Bcer	FALSE	NA	NA	NA
Bc_ctg1_1570	B3	2812.809266	Bcer	C	Bcer	FALSE	NA	NA	NA
Bc_ctg1_1571	B3	1986.520472	Bcer	C	Bcer	FALSE	NA	NA	NA
Bc_ctg1_1572	B3	6.651799538	Bcer	S	Bcer	FALSE	NA	NA	NA
Bc_ctg1_1573	B3	111.6149414	Bcer	S	Bcer	FALSE	NA	NA	NA
Bc_ctg1_1574	B3	1.465650746	Bcer	S	Bcer	FALSE	NA	NA	NA
Bc_ctg1_1575	B3	2384.613763	Bcer	G	Bcer	FALSE	NA	NA	NA
Bc_ctg1_1576	B3	721.2129092	Bcer	F	Bcer	FALSE	NA	NA	NA
Bc_ctg1_1577	B3	273.2874929	Bcer	I	Bcer	FALSE	NA	NA	NA
Bc_ctg1_1578	B3	257.6163041	Bcer	I	Bcer	FALSE	NA	NA	NA
Bc_ctg1_1579	B3	271.1453879	Bcer	K	Bcer	FALSE	NA	NA	NA
Bc_ctg1_158	B3	8.568419744	Bcer	S	Bcer	FALSE	NA	NA	NA
Bc_ctg1_1580	B3	933.619525	Bcer	C	Bcer	FALSE	NA	NA	NA
Bc_ctg1_1581	B3	193.9168679	Bcer	L	Bcer	FALSE	NA	NA	NA
Bc_ctg1_1582	B3	0	Bcer	S	Bcer	FALSE	NA	NA	NA
Bc_ctg1_1583	B3	0	Bcer	NA	Bcer	FALSE	NA	NA	NA
Bc_ctg1_1584	B3	132.4722789	Bcer	S	Bcer	FALSE	NA	NA	NA
Bc_ctg1_1585	B3	4.284209872	Bcer	S	Bcer	FALSE	NA	NA	NA

Bc_ctg1_1586	B3	7.328253728	Bcer	S	Bcer	FALSE	NA	NA	NA
Bc_ctg1_1587	B3	113.3060769	Bcer	K	Bcer	FALSE	NA	NA	NA
Bc_ctg1_1588	B3	0.22548473	Bcer	NA	Bcer	FALSE	NA	NA	NA
Bc_ctg1_1589	B3	123.6783745	Bcer	S	Bcer	FALSE	NA	NA	NA
Bc_ctg1_159	B3	0.22548473	Bcer	S	Bcer	FALSE	NA	NA	NA
Bc_ctg1_1590	B3	274.1894318	Bcer	E	Bcer	FALSE	NA	NA	NA
Bc_ctg1_1591	B3	5.862602983	Bcer	S	Bcer	FALSE	NA	NA	NA
Bc_ctg1_1592	B3	1.352908381	Bcer	NA	Bcer	FALSE	NA	NA	NA
Bc_ctg1_1593	B3	6.313572443	Bcer	S	Bcer	FALSE	NA	NA	NA
Bc_ctg1_1594	B3	0.22548473	Bcer	NA	Bcer	FALSE	NA	NA	NA
Bc_ctg1_1595	B3	2.367589666	Bcer	NA	Bcer	FALSE	NA	NA	NA
Bc_ctg1_1596	B3	5.186148792	Bcer	J	Bcer	FALSE	NA	NA	NA
Bc_ctg1_1597	B3	2.142104936	Bcer	G	Bcer	FALSE	NA	NA	NA
Bc_ctg1_1598	B3	1913.914389	Bcer	E	Bcer	FALSE	NA	NA	NA
Bc_ctg1_1599	B3	41.6019327	Bcer	IQ	Bcer	TRUE	NA	NA	NA
Bc_ctg1_16	B3	0.90193892	Bcer	U	Bcer	FALSE	NA	NA	NA
Bc_ctg1_160	B3	9.244873934	Bcer	Q	Bcer	TRUE	NA	NA	NA
Bc_ctg1_1600	B3	606.8921511	Bcer	T	Bcer	FALSE	NA	NA	NA
Bc_ctg1_1601	B3	15.44570401	Bcer	NA	Bcer	FALSE	NA	NA	NA
Bc_ctg1_1602	B3	0	Bcer	NA	Bcer	FALSE	NA	NA	NA
Bc_ctg1_1603	B3	82.07644176	Bcer	E	Bcer	FALSE	NA	NA	NA
Bc_ctg1_1604	B3	54.00359286	Bcer	E	Bcer	FALSE	NA	NA	NA
Bc_ctg1_1605	B3	17.02409712	Bcer	NA	Bcer	FALSE	NA	NA	NA
Bc_ctg1_1606	B3	0	Bcer	NA	Bcer	FALSE	NA	NA	NA
Bc_ctg1_1608	B3	310.7179581	Bcer	S	Bcer	FALSE	NA	NA	NA
Bc_ctg1_1609	B3	67.08170721	Bcer	S	Bcer	FALSE	NA	NA	NA
Bc_ctg1_161	B3	0.45096946	Bcer	E	Bcer	FALSE	NA	NA	NA
Bc_ctg1_1610	B3	0.112742365	Bcer	S	Bcer	FALSE	NA	NA	NA
Bc_ctg1_1611	B3	1.691135476	Bcer	F	Bcer	FALSE	NA	NA	NA
Bc_ctg1_1612	B3	266.8611781	Bcer	F	Bcer	FALSE	NA	NA	NA
Bc_ctg1_1613	B3	17.36232422	Bcer	L	Bcer	FALSE	NA	NA	NA
Bc_ctg1_1614	B3	448.8273553	Bcer	O	Bcer	FALSE	NA	NA	NA
Bc_ctg1_1615	B3	0.338227095	Bcer	S	Bcer	FALSE	NA	NA	NA
Bc_ctg1_1616	B3	0	Bcer	S	Bcer	FALSE	NA	NA	NA
Bc_ctg1_1617	B3	1289.208944	Bcer	G	Bcer	FALSE	NA	NA	NA
Bc_ctg1_1618	B3	14.09279563	Bcer	S	Bcer	FALSE	NA	NA	NA
Bc_ctg1_1619	B3	53.32713867	Bcer	S	Bcer	FALSE	NA	NA	NA
Bc_ctg1_162	B3	441.1608744	Bcer	L	Bcer	FALSE	NA	NA	NA
Bc_ctg1_1620	B3	5.749860618	Bcer	I	Bcer	FALSE	NA	NA	NA
Bc_ctg1_1621	B3	25.4797745	Bcer	S	Bcer	FALSE	NA	NA	NA
Bc_ctg1_1622	B3	238.111875	Bcer	H	Bcer	FALSE	NA	NA	NA
Bc_ctg1_1623	B3	104.2866877	Bcer	E	Bcer	FALSE	NA	NA	NA
Bc_ctg1_1624	B3	472.9542214	Bcer	D	Bcer	FALSE	NA	NA	NA
Bc_ctg1_1625	B3	1.352908381	Bcer	K	Bcer	FALSE	NA	NA	NA
Bc_ctg1_1626	B3	0.563711825	Bcer	EG	Bcer	FALSE	NA	NA	NA
Bc_ctg1_1627	B3	43.06758345	Bcer	K	Bcer	FALSE	NA	NA	NA
Bc_ctg1_1628	B3	22.66121538	Bcer	T	Bcer	FALSE	NA	NA	NA
Bc_ctg1_1629	B3	15.55844638	Bcer	E	Bcer	FALSE	NA	NA	NA
Bc_ctg1_163	B3	53.5526234	Bcer	V	Bcer	FALSE	NA	NA	NA
Bc_ctg1_1630	B3	2266.572507	Bcer	J	Bcer	FALSE	NA	NA	NA
Bc_ctg1_1631	B3	1.916620206	Bcer	S	Bcer	FALSE	NA	NA	NA
Bc_ctg1_1632	B3	0	Bcer	NA	Bcer	FALSE	NA	NA	NA
Bc_ctg1_1633	B3	173.6232422	Bcer	J	Bcer	FALSE	NA	NA	NA
Bc_ctg1_1634	B3	5.749860618	Bcer	NA	Bcer	FALSE	NA	NA	NA
Bc_ctg1_1635	B3	29.87672674	Bcer	K	Bcer	FALSE	NA	NA	NA
Bc_ctg1_1636	B3	13.30359908	Bcer	NA	Bcer	FALSE	NA	NA	NA
Bc_ctg1_1637	B3	202.2598029	Bcer	S	Bcer	FALSE	NA	NA	NA
Bc_ctg1_1638	B3	107.4434739	Bcer	K	Bcer	FALSE	NA	NA	NA
Bc_ctg1_1639	B3	394.4855353	Bcer	I	Bcer	FALSE	NA	NA	NA
Bc_ctg1_164	B3	155.2462367	Bcer	V	Bcer	FALSE	NA	NA	NA
Bc_ctg1_1640	B3	103.8357182	Bcer	K	Bcer	FALSE	NA	NA	NA
Bc_ctg1_1641	B3	83.5420925	Bcer	S	Bcer	FALSE	NA	NA	NA

Bc_ctg1_1642	B3	70.23849343	Bcer	BQ	Bcer	TRUE	NA	NA	NA
Bc_ctg1_1643	B3	5.073406427	Bcer	I	Bcer	FALSE	NA	NA	NA
Bc_ctg1_1644	B3	6.426314808	Bcer	NA	Bcer	FALSE	NA	NA	NA
Bc_ctg1_1645	B3	61.44458895	Bcer	T	Bcer	FALSE	NA	NA	NA
Bc_ctg1_1646	B3	45.54791548	Bcer	T	Bcer	FALSE	NA	NA	NA
Bc_ctg1_1647	B3	0	Bcer	S	Bcer	FALSE	NA	NA	NA
Bc_ctg1_1648	B3	119.1686799	Bcer	S	Bcer	FALSE	NA	NA	NA
Bc_ctg1_1649	B3	83.5420925	Bcer	M	Bcer	FALSE	NA	NA	NA
Bc_ctg1_165	B3	15.10747692	Bcer	K	Bcer	FALSE	NA	NA	NA
Bc_ctg1_1650	B3	11.49972124	Bcer	S	Bcer	FALSE	NA	NA	NA
Bc_ctg1_1651	B3	3.607755682	Bcer	S	Bcer	FALSE	NA	NA	NA
Bc_ctg1_1652	B3	16.68587003	Bcer	S	Bcer	FALSE	NA	NA	NA
Bc_ctg1_1653	B3	257.1653347	Bcer	K	Bcer	FALSE	NA	NA	NA
Bc_ctg1_1654	B3	2.818559126	Bcer	E	Bcer	FALSE	NA	NA	NA
Bc_ctg1_1655	B3	0.45096946	Bcer	E	Bcer	FALSE	NA	NA	NA
Bc_ctg1_1657	B3	133.9379297	Bcer	S	Bcer	FALSE	NA	NA	NA
Bc_ctg1_1658	B3	59.97893821	Bcer	O	Bcer	FALSE	NA	NA	NA
Bc_ctg1_1659	B3	53.89085049	Bcer	NA	Bcer	FALSE	NA	NA	NA
Bc_ctg1_166	B3	5.073406427	Bcer	G	Bcer	FALSE	NA	NA	NA
Bc_ctg1_1660	B3	799.3433682	Bcer	E	Bcer	FALSE	NA	NA	NA
Bc_ctg1_1661	B3	11.16149414	Bcer	S	Bcer	FALSE	NA	NA	NA
Bc_ctg1_1662	B3	8.906646839	Bcer	K	Bcer	FALSE	NA	NA	NA
Bc_ctg1_1663	B3	3.269528587	Bcer	J	Bcer	FALSE	NA	NA	NA
Bc_ctg1_1664	B3	2.029362571	Bcer	EG	Bcer	FALSE	NA	NA	NA
Bc_ctg1_1665	B3	0	Bcer	K	Bcer	FALSE	NA	NA	NA
Bc_ctg1_1666	B3	823.5829767	Bcer	M	Bcer	FALSE	NA	NA	NA
Bc_ctg1_1667	B3	470.361147	Bcer	H	Bcer	FALSE	NA	NA	NA
Bc_ctg1_1668	B3	524.5902246	Bcer	D	Bcer	FALSE	NA	NA	NA
Bc_ctg1_1669	B3	2.705816761	Bcer	M	Bcer	FALSE	Bc_Ga0417192_01_rn12	Other	NA
Bc_ctg1_167	B3	8.230192649	Bcer	S	Bcer	FALSE	NA	NA	NA
Bc_ctg1_1670	B3	0.112742365	Bcer	NA	Bcer	FALSE	Bc_Ga0417192_01_rn12	Other	NA
Bc_ctg1_1671	B3	217.4800222	Bcer	J	Bcer	FALSE	Bc_Ga0417192_01_rn12	Other	NA
Bc_ctg1_1672	B3	134.5016415	Bcer	S	Bcer	FALSE	Bc_Ga0417192_01_rn12	Other	NA
Bc_ctg1_1673	B3	32.5825435	Bcer	CO	Bcer	FALSE	Bc_Ga0417192_01_rn12	Other	NA
Bc_ctg1_1674	B3	1.691135476	Bcer	F	Bcer	FALSE	Bc_Ga0417192_01_rn12	Other	NA
Bc_ctg1_1675	B3	272.6110387	Bcer	G	Bcer	FALSE	Bc_Ga0417192_01_rn12	Ancillary biosynthesis	None
Bc_ctg1_1676	B3	10.37229758	Bcer	S	Bcer	FALSE	Bc_Ga0417192_01_rn12	Other	NA
Bc_ctg1_1677	B3	33.70996715	Bcer	S	Bcer	FALSE	Bc_Ga0417192_01_rn12	Other	NA
Bc_ctg1_1678	B3	45.54791548	Bcer	J	Bcer	FALSE	Bc_Ga0417192_01_rn12	Other	NA
Bc_ctg1_1679	B3	0	Bcer	S	Bcer	FALSE	Bc_Ga0417192_01_rn12	Other	NA
Bc_ctg1_168	B3	29.53849964	Bcer	T	Bcer	FALSE	NA	NA	NA
Bc_ctg1_1680	B3	97.29666104	Bcer	M	Bcer	FALSE	Bc_Ga0417192_01_rn12	Ancillary biosynthesis	None
Bc_ctg1_1681	B3	30.21495383	Bcer	G	Bcer	FALSE	Bc_Ga0417192_01_rn12	Core biosynthesis	SMCOG1297
Bc_ctg1_1682	B3	5.637118253	Bcer	J	Bcer	FALSE	Bc_Ga0417192_01_rn12	Other	NA
Bc_ctg1_1683	B3	281.4049432	Bcer	E	Bcer	FALSE	Bc_Ga0417192_01_rn12	Ancillary biosynthesis	SMCOG1247
Bc_ctg1_1684	B3	294.8212846	Bcer	K	Bcer	FALSE	Bc_Ga0417192_01_rn12	Other	NA
Bc_ctg1_1685	B3	39.3470854	Bcer	J	Bcer	FALSE	Bc_Ga0417192_01_rn12	Ancillary biosynthesis	SMCOG1154
Bc_ctg1_1686	B3	241.7196307	Bcer	S	Bcer	FALSE	Bc_Ga0417192_01_rn12	Core biosynthesis	SMCOG1086
Bc_ctg1_1687	B3	65.95428356	Bcer	S	Bcer	FALSE	Bc_Ga0417192_01_rn12	Other	NA
Bc_ctg1_1688	B3	107.6689586	Bcer	P	Bcer	FALSE	Bc_Ga0417192_01_rn12	Transport	SMCOG1005
Bc_ctg1_1689	B3	7.102768998	Bcer	K	Bcer	FALSE	Bc_Ga0417192_01_rn12	Regulation	SMCOG1058
Bc_ctg1_169	B3	7.891965554	Bcer	T	Bcer	FALSE	NA	NA	NA
Bc_ctg1_1690	B3	10.37229758	Bcer	S	Bcer	FALSE	Bc_Ga0417192_01_rn12	Other	NA
Bc_ctg1_1691	B3	0	Bcer	S	Bcer	FALSE	Bc_Ga0417192_01_rn12	Other	NA
Bc_ctg1_1693	B3	0	Bcer	S	Bcer	FALSE	Bc_Ga0417192_01_rn12	Other	NA
Bc_ctg1_1694	B3	531.4675089	Bcer	K	Bcer	FALSE	Bc_Ga0417192_01_rn12	Other	NA
Bc_ctg1_1695	B3	1.691135476	Bcer	IQ	Bcer	TRUE	Bc_Ga0417192_01_rn12	Ancillary biosynthesis	SMCOG1001
Bc_ctg1_1696	B3	0	Bcer	U	Bcer	FALSE	Bc_Ga0417192_01_rn12	Other	NA
Bc_ctg1_1697	B3	0.67645419	Bcer	S	Bcer	FALSE	Bc_Ga0417192_01_rn12	Other	NA
Bc_ctg1_1698	B3	2.593074396	Bcer	H	Bcer	FALSE	Bc_Ga0417192_01_rn12	Other	NA
Bc_ctg1_1699	B3	8.568419744	Bcer	H	Bcer	FALSE	Bc_Ga0417192_01_rn12	Other	NA
Bc_ctg1_17	B3	19.05345969	Bcer	M	Bcer	FALSE	NA	NA	NA

Bc_ctg1_170	B3	16.34764293	Bcer	S	Bcer	FALSE	NA	NA	NA
Bc_ctg1_1700	B3	31.90608931	Bcer	H	Bcer	FALSE	Bc_Ga0417192_01_rn12	Other	NA
Bc_ctg1_1701	B3	86.36065163	Bcer	H	Bcer	FALSE	Bc_Ga0417192_01_rn12	Other	NA
Bc_ctg1_1702	B3	12.17617543	Bcer	H	Bcer	FALSE	NA	NA	NA
Bc_ctg1_1703	B3	39.79805486	Bcer	H	Bcer	FALSE	NA	NA	NA
Bc_ctg1_1704	B3	41.48919034	Bcer	H	Bcer	FALSE	NA	NA	NA
Bc_ctg1_1705	B3	7.440996093	Bcer	Q	Bcer	TRUE	NA	NA	NA
Bc_ctg1_1706	B3	4.735179332	Bcer	NA	Bcer	FALSE	NA	NA	NA
Bc_ctg1_1707	B3	0.789196555	Bcer	NA	Bcer	FALSE	NA	NA	NA
Bc_ctg1_1708	B3	2.705816761	Bcer	P	Bcer	FALSE	NA	NA	NA
Bc_ctg1_1709	B3	0.789196555	Bcer	NA	Bcer	FALSE	NA	NA	NA
Bc_ctg1_171	B3	31.79334694	Bcer	NA	Bcer	FALSE	NA	NA	NA
Bc_ctg1_1710	B3	4.171467507	Bcer	E	Bcer	FALSE	NA	NA	NA
Bc_ctg1_1711	B3	0.22548473	Bcer	P	Bcer	FALSE	NA	NA	NA
Bc_ctg1_1712	B3	1.014681285	Bcer	E	Bcer	FALSE	NA	NA	NA
Bc_ctg1_1713	B3	3.945982777	Bcer	S	Bcer	FALSE	NA	NA	NA
Bc_ctg1_1714	B3	1.240166016	Bcer	NA	Bcer	FALSE	NA	NA	NA
Bc_ctg1_1715	B3	71.14043235	Bcer	S	Bcer	FALSE	NA	NA	NA
Bc_ctg1_1716	B3	0	Bcer	NA	Bcer	FALSE	NA	NA	NA
Bc_ctg1_1718	B3	0	Bcer	NA	Bcer	FALSE	NA	NA	NA
Bc_ctg1_1719	B3	705.8799476	Bcer	J	Bcer	FALSE	NA	NA	NA
Bc_ctg1_172	B3	40.69999378	Bcer	V	Bcer	FALSE	NA	NA	NA
Bc_ctg1_1720	B3	12.51440252	Bcer	EGP	Bcer	FALSE	NA	NA	NA
Bc_ctg1_1721	B3	16.68587003	Bcer	P	Bcer	FALSE	NA	NA	NA
Bc_ctg1_1722	B3	13.52908381	Bcer	P	Bcer	FALSE	NA	NA	NA
Bc_ctg1_1723	B3	12.73988725	Bcer	L	Bcer	FALSE	NA	NA	NA
Bc_ctg1_1724	B3	63.69943625	Bcer	V	Bcer	FALSE	NA	NA	NA
Bc_ctg1_1725	B3	13.07811435	Bcer	V	Bcer	FALSE	NA	NA	NA
Bc_ctg1_1726	B3	0.789196555	Bcer	S	Bcer	FALSE	NA	NA	NA
Bc_ctg1_1728	B3	0	Bcer	S	Bcer	FALSE	NA	NA	NA
Bc_ctg1_1729	B3	25.14154741	Bcer	S	Bcer	FALSE	NA	NA	NA
Bc_ctg1_173	B3	17.13683949	Bcer	S	Bcer	FALSE	NA	NA	NA
Bc_ctg1_1730	B3	17.81329368	Bcer	M	Bcer	FALSE	NA	NA	NA
Bc_ctg1_1731	B3	11.16149414	Bcer	V	Bcer	FALSE	NA	NA	NA
Bc_ctg1_1732	B3	3.720498047	Bcer	NA	Bcer	FALSE	NA	NA	NA
Bc_ctg1_1733	B3	4.396952237	Bcer	M	Bcer	FALSE	NA	NA	NA
Bc_ctg1_1734	B3	14.09279563	Bcer	S	Bcer	FALSE	NA	NA	NA
Bc_ctg1_1735	B3	185.1229634	Bcer	S	Bcer	FALSE	NA	NA	NA
Bc_ctg1_1736	B3	24.12686612	Bcer	I	Bcer	FALSE	NA	NA	NA
Bc_ctg1_1737	B3	26.60719815	Bcer	S	Bcer	FALSE	NA	NA	NA
Bc_ctg1_1738	B3	0.45096946	Bcer	I	Bcer	FALSE	NA	NA	NA
Bc_ctg1_1739	B3	0.789196555	Bcer	M	Bcer	FALSE	NA	NA	NA
Bc_ctg1_174	B3	1.803877841	Bcer	I	Bcer	FALSE	NA	NA	NA
Bc_ctg1_1740	B3	17.92603604	Bcer	H	Bcer	FALSE	NA	NA	NA
Bc_ctg1_1741	B3	20.29362571	Bcer	H	Bcer	FALSE	NA	NA	NA
Bc_ctg1_1742	B3	1.352908381	Bcer	NA	Bcer	FALSE	NA	NA	NA
Bc_ctg1_1743	B3	1299.581242	Bcer	H	Bcer	FALSE	NA	NA	NA
Bc_ctg1_1744	B3	4935.071545	Bcer	C	Bcer	FALSE	NA	NA	NA
Bc_ctg1_1745	B3	0	Bcer	S	Bcer	FALSE	NA	NA	NA
Bc_ctg1_1746	B3	0.338227095	Bcer	NA	Bcer	FALSE	NA	NA	NA
Bc_ctg1_1747	B3	7.666480824	Bcer	EG	Bcer	FALSE	NA	NA	NA
Bc_ctg1_1748	B3	2.931301491	Bcer	S	Bcer	FALSE	NA	NA	NA
Bc_ctg1_1749	B3	9.019389204	Bcer	S	Bcer	FALSE	NA	NA	NA
Bc_ctg1_175	B3	265.7337544	Bcer	S	Bcer	FALSE	NA	NA	NA
Bc_ctg1_1750	B3	0.563711825	Bcer	Q	Bcer	TRUE	NA	NA	NA
Bc_ctg1_1751	B3	1.578393111	Bcer	S	Bcer	FALSE	NA	NA	NA
Bc_ctg1_1752	B3	0.789196555	Bcer	S	Bcer	FALSE	NA	NA	NA
Bc_ctg1_1753	B3	0.45096946	Bcer	S	Bcer	FALSE	NA	NA	NA
Bc_ctg1_1754	B3	0.338227095	Bcer	NA	Bcer	FALSE	NA	NA	NA
Bc_ctg1_1755	B3	2.142104936	Bcer	NU	Bcer	FALSE	NA	NA	NA
Bc_ctg1_1756	B3	16.23490057	Bcer	T	Bcer	FALSE	NA	NA	NA
Bc_ctg1_1757	B3	6.990026633	Bcer	T	Bcer	FALSE	NA	NA	NA

Bc_ctg1_1758	B3	0.112742365	Bcer	S	Bcer	FALSE	NA	NA	NA
Bc_ctg1_1759	B3	103.8357182	Bcer	M	Bcer	FALSE	NA	NA	NA
Bc_ctg1_176	B3	97.4094034	Bcer	EH	Bcer	FALSE	NA	NA	NA
Bc_ctg1_1760	B3	4.284209872	Bcer	NA	Bcer	FALSE	NA	NA	NA
Bc_ctg1_1761	B3	3.495013317	Bcer	S	Bcer	FALSE	NA	NA	NA
Bc_ctg1_1762	B3	2.029362571	Bcer	P	Bcer	FALSE	NA	NA	NA
Bc_ctg1_1763	B3	1.916620206	Bcer	P	Bcer	FALSE	NA	NA	NA
Bc_ctg1_1764	B3	2.705816761	Bcer	P	Bcer	FALSE	NA	NA	NA
Bc_ctg1_1765	B3	0	Bcer	NA	Bcer	FALSE	NA	NA	NA
Bc_ctg1_1766	B3	13.8673109	Bcer	NA	Bcer	FALSE	NA	NA	NA
Bc_ctg1_1767	B3	7.328253728	Bcer	S	Bcer	FALSE	NA	NA	NA
Bc_ctg1_1768	B3	5.298891157	Bcer	S	Bcer	FALSE	NA	NA	NA
Bc_ctg1_1769	B3	118.0412562	Bcer	L	Bcer	FALSE	NA	NA	NA
Bc_ctg1_177	B3	35.96481445	Bcer	E	Bcer	FALSE	NA	NA	NA
Bc_ctg1_1770	B3	0.112742365	Bcer	NA	Bcer	FALSE	NA	NA	NA
Bc_ctg1_1772	B3	0.22548473	Bcer	NA	Bcer	FALSE	NA	NA	NA
Bc_ctg1_1773	B3	0	Bcer	NA	Bcer	FALSE	NA	NA	NA
Bc_ctg1_1774	B3	177.7947097	Bcer	T	Bcer	FALSE	NA	NA	NA
Bc_ctg1_1775	B3	1.352908381	Bcer	S	Bcer	FALSE	NA	NA	NA
Bc_ctg1_1776	B3	113.1933345	Bcer	P	Bcer	FALSE	NA	NA	NA
Bc_ctg1_1777	B3	409.7057546	Bcer	C	Bcer	FALSE	NA	NA	NA
Bc_ctg1_1778	B3	254.3467756	Bcer	C	Bcer	FALSE	NA	NA	NA
Bc_ctg1_1779	B3	0	Bcer	S	Bcer	FALSE	NA	NA	NA
Bc_ctg1_178	B3	0.112742365	Bcer	S	Bcer	FALSE	NA	NA	NA
Bc_ctg1_1780	B3	7.215511363	Bcer	S	Bcer	FALSE	NA	NA	NA
Bc_ctg1_1781	B3	0	Bcer	S	Bcer	FALSE	NA	NA	NA
Bc_ctg1_1782	B3	42.95484108	Bcer	S	Bcer	FALSE	NA	NA	NA
Bc_ctg1_1783	B3	12.17617543	Bcer	NA	Bcer	FALSE	NA	NA	NA
Bc_ctg1_1784	B3	235.8570277	Bcer	S	Bcer	FALSE	NA	NA	NA
Bc_ctg1_1785	B3	99.43876598	Bcer	S	Bcer	FALSE	NA	NA	NA
Bc_ctg1_1786	B3	29.42575728	Bcer	S	Bcer	FALSE	NA	NA	NA
Bc_ctg1_1787	B3	420.0780522	Bcer	KT	Bcer	FALSE	NA	NA	NA
Bc_ctg1_1788	B3	10.59778231	Bcer	S	Bcer	FALSE	NA	NA	NA
Bc_ctg1_179	B3	11.6124636	Bcer	G	Bcer	FALSE	NA	NA	NA
Bc_ctg1_1791	B3	0.112742365	Bcer	S	Bcer	FALSE	NA	NA	NA
Bc_ctg1_1792	B3	254.2340332	Bcer	P	Bcer	FALSE	NA	NA	NA
Bc_ctg1_1793	B3	14.43102273	Bcer	P	Bcer	FALSE	NA	NA	NA
Bc_ctg1_1794	B3	20.29362571	Bcer	S	Bcer	FALSE	NA	NA	NA
Bc_ctg1_1795	B3	83.76757723	Bcer	T	Bcer	FALSE	NA	NA	NA
Bc_ctg1_1796	B3	22.99944247	Bcer	T	Bcer	FALSE	NA	NA	NA
Bc_ctg1_1797	B3	33.82270952	Bcer	M	Bcer	FALSE	NA	NA	NA
Bc_ctg1_1798	B3	16.23490057	Bcer	NA	Bcer	FALSE	NA	NA	NA
Bc_ctg1_1799	B3	153.6678436	Bcer	NA	Bcer	FALSE	NA	NA	NA
Bc_ctg1_18	B3	26.38171342	Bcer	K	Bcer	FALSE	NA	NA	NA
Bc_ctg1_180	B3	1.352908381	Bcer	G	Bcer	FALSE	NA	NA	NA
Bc_ctg1_1800	B3	15.55844638	Bcer	NA	Bcer	FALSE	NA	NA	NA
Bc_ctg1_1801	B3	214.7742054	Bcer	F	Bcer	FALSE	NA	NA	NA
Bc_ctg1_1802	B3	144.0847425	Bcer	I	Bcer	FALSE	NA	NA	NA
Bc_ctg1_1803	B3	7.666480824	Bcer	S	Bcer	FALSE	NA	NA	NA
Bc_ctg1_1804	B3	13.07811435	Bcer	NA	Bcer	FALSE	NA	NA	NA
Bc_ctg1_1805	B3	15.33296165	Bcer	S	Bcer	FALSE	NA	NA	NA
Bc_ctg1_1806	B3	0.112742365	Bcer	NA	Bcer	FALSE	NA	NA	NA
Bc_ctg1_1807	B3	0.789196555	Bcer	NA	Bcer	FALSE	NA	NA	NA
Bc_ctg1_1808	B3	1.127423651	Bcer	S	Bcer	FALSE	NA	NA	NA
Bc_ctg1_1809	B3	0.22548473	Bcer	I	Bcer	FALSE	NA	NA	NA
Bc_ctg1_181	B3	1.465650746	Bcer	K	Bcer	FALSE	NA	NA	NA
Bc_ctg1_1810	B3	175.3143777	Bcer	V	Bcer	FALSE	NA	NA	NA
Bc_ctg1_1811	B3	39.3470854	Bcer	V	Bcer	FALSE	NA	NA	NA
Bc_ctg1_1812	B3	23.67589666	Bcer	K	Bcer	FALSE	NA	NA	NA
Bc_ctg1_1813	B3	17.58780895	Bcer	S	Bcer	FALSE	NA	NA	NA
Bc_ctg1_1814	B3	13.8673109	Bcer	S	Bcer	FALSE	NA	NA	NA
Bc_ctg1_1815	B3	2.480332031	Bcer	K	Bcer	FALSE	NA	NA	NA

Bc_ctg1_1816	B3	52.65068448	Bcer	K	Bcer	FALSE	NA	NA	NA
Bc_ctg1_1817	B3	79.48336736	Bcer	T	Bcer	FALSE	NA	NA	NA
Bc_ctg1_1818	B3	54.00359286	Bcer	V	Bcer	FALSE	NA	NA	NA
Bc_ctg1_1819	B3	49.83212535	Bcer	V	Bcer	FALSE	NA	NA	NA
Bc_ctg1_182	B3	7.102768998	Bcer	S	Bcer	FALSE	NA	NA	NA
Bc_ctg1_1820	B3	12.51440252	Bcer	V	Bcer	FALSE	NA	NA	NA
Bc_ctg1_1821	B3	54.45456232	Bcer	V	Bcer	FALSE	NA	NA	NA
Bc_ctg1_1823	B3	10.37229758	Bcer	EG	Bcer	FALSE	NA	NA	NA
Bc_ctg1_1824	B3	21.30830699	Bcer	K	Bcer	FALSE	NA	NA	NA
Bc_ctg1_1825	B3	17.47506658	Bcer	G	Bcer	FALSE	NA	NA	NA
Bc_ctg1_1826	B3	21.30830699	Bcer	S	Bcer	FALSE	NA	NA	NA
Bc_ctg1_1827	B3	27.39639471	Bcer	E	Bcer	FALSE	NA	NA	NA
Bc_ctg1_1828	B3	8.230192649	Bcer	S	Bcer	FALSE	NA	NA	NA
Bc_ctg1_1829	B3	2.593074396	Bcer	M	Bcer	FALSE	NA	NA	NA
Bc_ctg1_183	B3	25.36703214	Bcer	C	Bcer	FALSE	NA	NA	NA
Bc_ctg1_1830	B3	20.85733753	Bcer	T	Bcer	FALSE	NA	NA	NA
Bc_ctg1_1831	B3	8.906646839	Bcer	T	Bcer	FALSE	NA	NA	NA
Bc_ctg1_1832	B3	4.622436967	Bcer	H	Bcer	FALSE	NA	NA	NA
Bc_ctg1_1833	B3	5.524375888	Bcer	H	Bcer	FALSE	NA	NA	NA
Bc_ctg1_1834	B3	861.4644114	Bcer	H	Bcer	FALSE	NA	NA	NA
Bc_ctg1_1835	B3	46.33711204	Bcer	S	Bcer	FALSE	NA	NA	NA
Bc_ctg1_1836	B3	143.1828036	Bcer	H	Bcer	FALSE	Bc_Ga0417192_01_rn13	Other	NA
Bc_ctg1_1837	B3	109.2473517	Bcer	HQ	Bcer	TRUE	Bc_Ga0417192_01_rn13	Ancillary biosynthesis	SMCOG1018
Bc_ctg1_1838	B3	17.24958185	Bcer	H	Bcer	FALSE	Bc_Ga0417192_01_rn13	Ancillary biosynthesis	None
Bc_ctg1_1839	B3	0.789196555	Bcer	T	Bcer	FALSE	Bc_Ga0417192_01_rn13	Other	NA
Bc_ctg1_184	B3	0.45096946	Bcer	L	Bcer	FALSE	NA	NA	NA
Bc_ctg1_1840	B3	153.4423588	Bcer	K	Bcer	FALSE	Bc_Ga0417192_01_rn13	Regulation	SMCOG1255
Bc_ctg1_1841	B3	99.32602361	Bcer	S	Bcer	FALSE	Bc_Ga0417192_01_rn13	Other	NA
Bc_ctg1_1842	B3	0	Bcer	S	Bcer	FALSE	Bc_Ga0417192_01_rn13	Other	NA
Bc_ctg1_1843	B3	1.803877841	Bcer	K	Bcer	FALSE	Bc_Ga0417192_01_rn13	Regulation	SMCOG1171
Bc_ctg1_1844	B3	0	Bcer	I	Bcer	FALSE	Bc_Ga0417192_01_rn13	Other	NA
Bc_ctg1_1845	B3	0	Bcer	NA	Bcer	FALSE	Bc_Ga0417192_01_rn13	Other	NA
Bc_ctg1_1846	B3	746.6926837	Bcer	G	Bcer	FALSE	Bc_Ga0417192_01_rn13	Other	NA
Bc_ctg1_1847	B3	260.0966362	Bcer	G	Bcer	FALSE	Bc_Ga0417192_01_rn13	Core biosynthesis	SMCOG1045
Bc_ctg1_1848	B3	241.9451154	Bcer	G	Bcer	FALSE	Bc_Ga0417192_01_rn13	Ancillary biosynthesis	SMCOG1064
Bc_ctg1_1849	B3	214.6614631	Bcer	G	Bcer	FALSE	Bc_Ga0417192_01_rn13	Ancillary biosynthesis	SMCOG1064
Bc_ctg1_185	B3	39.91079723	Bcer	M	Bcer	FALSE	NA	NA	NA
Bc_ctg1_1850	B3	372.3880318	Bcer	G	Bcer	FALSE	Bc_Ga0417192_01_rn13	Core biosynthesis	SMCOG1297
Bc_ctg1_1852	B3	2.705816761	Bcer	O	Bcer	FALSE	Bc_Ga0417192_01_rn13	Ancillary biosynthesis	SMCOG1075
Bc_ctg1_1853	B3	6408.27603	Bcer	C	Bcer	FALSE	Bc_Ga0417192_01_rn13	Other	NA
Bc_ctg1_1855	B3	7.891965554	Bcer	S	Bcer	FALSE	Bc_Ga0417192_01_rn13	Other	NA
Bc_ctg1_1856	B3	0.45096946	Bcer	P	Bcer	FALSE	Bc_Ga0417192_01_rn13	Other	NA
Bc_ctg1_1857	B3	0.67645419	Bcer	S	Bcer	FALSE	Bc_Ga0417192_01_rn13	Other	NA
Bc_ctg1_1858	B3	1278.723904	Bcer	G	Bcer	FALSE	Bc_Ga0417192_01_rn13	Other	NA
Bc_ctg1_1859	B3	1.465650746	Bcer	S	Bcer	FALSE	Bc_Ga0417192_01_rn13	Other	NA
Bc_ctg1_186	B3	0.90193892	Bcer	NA	Bcer	FALSE	NA	NA	NA
Bc_ctg1_1860	B3	56.37118253	Bcer	J	Bcer	FALSE	Bc_Ga0417192_01_rn13	Other	NA
Bc_ctg1_1861	B3	100.4534473	Bcer	E	Bcer	FALSE	Bc_Ga0417192_01_rn13	Core biosynthesis	SMCOG1019
Bc_ctg1_1862	B3	3.720498047	Bcer	K	Bcer	FALSE	Bc_Ga0417192_01_rn13	Regulation	SMCOG1120
Bc_ctg1_1863	B3	112.0659109	Bcer	CH	Bcer	FALSE	Bc_Ga0417192_01_rn13	Ancillary biosynthesis	SMCOG1072
Bc_ctg1_1864	B3	0.22548473	Bcer	I	Bcer	FALSE	Bc_Ga0417192_01_rn13	Ancillary biosynthesis	SMCOG1262
Bc_ctg1_1865	B3	1.127423651	Bcer	S	Bcer	FALSE	Bc_Ga0417192_01_rn13	Other	NA
Bc_ctg1_1866	B3	46.4498544	Bcer	E	Bcer	FALSE	Bc_Ga0417192_01_rn13	Ancillary biosynthesis	SMCOG1019
Bc_ctg1_1867	B3	7.553738458	Bcer	P	Bcer	FALSE	Bc_Ga0417192_01_rn13	Other	NA
Bc_ctg1_1868	B3	0.112742365	Bcer	NA	Bcer	FALSE	Bc_Ga0417192_01_rn13	Other	NA
Bc_ctg1_1869	B3	48.93018643	Bcer	G	Bcer	FALSE	Bc_Ga0417192_01_rn13	Other	NA
Bc_ctg1_187	B3	110.4875178	Bcer	L	Bcer	FALSE	NA	NA	NA
Bc_ctg1_1870	B3	4.284209872	Bcer	L	Bcer	FALSE	Bc_Ga0417192_01_rn13	Other	NA
Bc_ctg1_1871	B3	51.86148792	Bcer	P	Bcer	FALSE	Bc_Ga0417192_01_rn13	Other	NA
Bc_ctg1_1872	B3	0.563711825	Bcer	NA	Bcer	FALSE	Bc_Ga0417192_01_rn13	Other	NA
Bc_ctg1_1873	B3	4.960664062	Bcer	L	Bcer	FALSE	Bc_Ga0417192_01_rn13	Other	NA
Bc_ctg1_1874	B3	531.4675089	Bcer	J	Bcer	FALSE	Bc_Ga0417192_01_rn13	Other	NA

Bc_ctg1_1875	B3	42.39112926	Bcer	Q	Bcer	TRUE	Bc_Ga0417192_01_rn13	Ancillary biosynthesis	SMCOG1144
Bc_ctg1_1876	B3	0.112742365	Bcer	S	Bcer	FALSE	Bc_Ga0417192_01_rn13	Other	NA
Bc_ctg1_1877	B3	13.52908381	Bcer	S	Bcer	FALSE	Bc_Ga0417192_01_rn13	Other	NA
Bc_ctg1_1878	B3	18.3770055	Bcer	S	Bcer	FALSE	Bc_Ga0417192_01_rn13	Other	NA
Bc_ctg1_1879	B3	304.9680975	Bcer	M	Bcer	FALSE	Bc_Ga0417192_01_rn13	Core biosynthesis	SMCOG1064
Bc_ctg1_188	B3	2.142104936	Bcer	NA	Bcer	FALSE	NA	NA	NA
Bc_ctg1_1880	B3	649.9597345	Bcer	G	Bcer	FALSE	Bc_Ga0417192_01_rn13	Other	NA
Bc_ctg1_1881	B3	0.789196555	Bcer	S	Bcer	FALSE	Bc_Ga0417192_01_rn13	Other	NA
Bc_ctg1_1882	B3	47.69002042	Bcer	NA	Bcer	FALSE	Bc_Ga0417192_01_rn13	Other	NA
Bc_ctg1_1883	B3	14.99473455	Bcer	S	Bcer	FALSE	Bc_Ga0417192_01_rn13	Other	NA
Bc_ctg1_1884	B3	5.186148792	Bcer	P	Bcer	FALSE	Bc_Ga0417192_01_rn13	Other	NA
Bc_ctg1_1885	B3	93.46342063	Bcer	H	Bcer	FALSE	Bc_Ga0417192_01_rn13	Ancillary biosynthesis	None
Bc_ctg1_1886	B3	1026.744719	Bcer	E	Bcer	FALSE	Bc_Ga0417192_01_rn13	Other	NA
Bc_ctg1_1887	B3	5.975345348	Bcer	S	Bcer	FALSE	Bc_Ga0417192_01_rn13	Other	NA
Bc_ctg1_1888	B3	14.31828036	Bcer	S	Bcer	FALSE	Bc_Ga0417192_01_rn13	Other	NA
Bc_ctg1_1889	B3	7.328253728	Bcer	F	Bcer	FALSE	Bc_Ga0417192_01_rn13	Other	NA
Bc_ctg1_189	B3	62.00830078	Bcer	M	Bcer	FALSE	NA	NA	NA
Bc_ctg1_1890	B3	28.86204545	Bcer	C	Bcer	FALSE	NA	NA	NA
Bc_ctg1_1891	B3	333.8301429	Bcer	C	Bcer	FALSE	NA	NA	NA
Bc_ctg1_1892	B3	55.24375888	Bcer	M	Bcer	FALSE	NA	NA	NA
Bc_ctg1_1893	B3	17.36232422	Bcer	K	Bcer	FALSE	NA	NA	NA
Bc_ctg1_1894	B3	73.28253728	Bcer	EGP	Bcer	FALSE	NA	NA	NA
Bc_ctg1_1895	B3	0.789196555	Bcer	K	Bcer	FALSE	NA	NA	NA
Bc_ctg1_1896	B3	2.480332031	Bcer	S	Bcer	FALSE	NA	NA	NA
Bc_ctg1_1897	B3	4.284209872	Bcer	S	Bcer	FALSE	NA	NA	NA
Bc_ctg1_1898	B3	0.22548473	Bcer	S	Bcer	FALSE	NA	NA	NA
Bc_ctg1_1899	B3	0.112742365	Bcer	NA	Bcer	FALSE	NA	NA	NA
Bc_ctg1_19	B3	6.539057173	Bcer	O	Bcer	FALSE	NA	NA	NA
Bc_ctg1_190	B3	77.34126243	Bcer	M	Bcer	FALSE	NA	NA	NA
Bc_ctg1_1900	B3	3.269528587	Bcer	NA	Bcer	FALSE	NA	NA	NA
Bc_ctg1_1901	B3	10.48503995	Bcer	J	Bcer	FALSE	NA	NA	NA
Bc_ctg1_1902	B3	133.0359908	Bcer	S	Bcer	FALSE	NA	NA	NA
Bc_ctg1_1903	B3	50.05761008	Bcer	E	Bcer	FALSE	NA	NA	NA
Bc_ctg1_1904	B3	8.906646839	Bcer	S	Bcer	FALSE	NA	NA	NA
Bc_ctg1_1905	B3	413.9899645	Bcer	C	Bcer	FALSE	NA	NA	NA
Bc_ctg1_1906	B3	10.82326704	Bcer	V	Bcer	FALSE	NA	NA	NA
Bc_ctg1_1907	B3	4.396952237	Bcer	S	Bcer	FALSE	NA	NA	NA
Bc_ctg1_1908	B3	14.54376509	Bcer	P	Bcer	FALSE	NA	NA	NA
Bc_ctg1_1909	B3	2.480332031	Bcer	NU	Bcer	FALSE	NA	NA	NA
Bc_ctg1_191	B3	2.029362571	Bcer	S	Bcer	FALSE	NA	NA	NA
Bc_ctg1_1910	B3	1.916620206	Bcer	S	Bcer	FALSE	NA	NA	NA
Bc_ctg1_1911	B3	2.705816761	Bcer	NA	Bcer	FALSE	NA	NA	NA
Bc_ctg1_1912	B3	0.112742365	Bcer	S	Bcer	FALSE	NA	NA	NA
Bc_ctg1_1913	B3	2.818559126	Bcer	J	Bcer	FALSE	NA	NA	NA
Bc_ctg1_1914	B3	2.367589666	Bcer	S	Bcer	FALSE	NA	NA	NA
Bc_ctg1_1915	B3	67.4199343	Bcer	O	Bcer	FALSE	NA	NA	NA
Bc_ctg1_1916	B3	10.14681285	Bcer	NA	Bcer	FALSE	NA	NA	NA
Bc_ctg1_1917	B3	11.16149414	Bcer	S	Bcer	FALSE	NA	NA	NA
Bc_ctg1_1918	B3	37.4304652	Bcer	I	Bcer	FALSE	NA	NA	NA
Bc_ctg1_1919	B3	2.029362571	Bcer	J	Bcer	FALSE	NA	NA	NA
Bc_ctg1_192	B3	43.06758345	Bcer	EGP	Bcer	FALSE	NA	NA	NA
Bc_ctg1_1920	B3	4.622436967	Bcer	G	Bcer	FALSE	NA	NA	NA
Bc_ctg1_1921	B3	3.269528587	Bcer	F	Bcer	FALSE	NA	NA	NA
Bc_ctg1_1922	B3	4.622436967	Bcer	J	Bcer	FALSE	NA	NA	NA
Bc_ctg1_1923	B3	2.705816761	Bcer	S	Bcer	FALSE	NA	NA	NA
Bc_ctg1_1924	B3	14.99473455	Bcer	S	Bcer	FALSE	NA	NA	NA
Bc_ctg1_1925	B3	3.495013317	Bcer	NA	Bcer	FALSE	NA	NA	NA
Bc_ctg1_1926	B3	106.2033079	Bcer	G	Bcer	FALSE	NA	NA	NA
Bc_ctg1_1927	B3	25.59251687	Bcer	K	Bcer	FALSE	NA	NA	NA
Bc_ctg1_1928	B3	82.52741122	Bcer	S	Bcer	FALSE	NA	NA	NA
Bc_ctg1_1929	B3	147.9179829	Bcer	G	Bcer	FALSE	NA	NA	NA
Bc_ctg1_193	B3	7.215511363	Bcer	NA	Bcer	FALSE	NA	NA	NA

Bc_ctg1_1930	B3	43.51855291	Bcer	S	Bcer	FALSE	NA	NA	NA
Bc_ctg1_1931	B3	2.480332031	Bcer	S	Bcer	FALSE	NA	NA	NA
Bc_ctg1_1932	B3	10.48503995	Bcer	S	Bcer	FALSE	NA	NA	NA
Bc_ctg1_1933	B3	0.67645419	Bcer	S	Bcer	FALSE	NA	NA	NA
Bc_ctg1_1934	B3	2.593074396	Bcer	K	Bcer	FALSE	NA	NA	NA
Bc_ctg1_1935	B3	60.3171653	Bcer	S	Bcer	FALSE	NA	NA	NA
Bc_ctg1_1936	B3	0	Bcer	NA	Bcer	FALSE	NA	NA	NA
Bc_ctg1_1937	B3	29.08753018	Bcer	G	Bcer	FALSE	NA	NA	NA
Bc_ctg1_1938	B3	15.89667347	Bcer	S	Bcer	FALSE	NA	NA	NA
Bc_ctg1_1939	B3	782.8829829	Bcer	H	Bcer	FALSE	NA	NA	NA
Bc_ctg1_194	B3	89.06646839	Bcer	I	Bcer	FALSE	NA	NA	NA
Bc_ctg1_1940	B3	0.338227095	Bcer	M	Bcer	FALSE	NA	NA	NA
Bc_ctg1_1941	B3	5.637118253	Bcer	S	Bcer	FALSE	NA	NA	NA
Bc_ctg1_1942	B3	9.019389204	Bcer	S	Bcer	FALSE	NA	NA	NA
Bc_ctg1_1943	B3	56.70940962	Bcer	F	Bcer	FALSE	NA	NA	NA
Bc_ctg1_1944	B3	1822.254846	Bcer	O	Bcer	FALSE	NA	NA	NA
Bc_ctg1_1945	B3	292.1154678	Bcer	C	Bcer	FALSE	NA	NA	NA
Bc_ctg1_1946	B3	1015.695967	Bcer	E	Bcer	FALSE	NA	NA	NA
Bc_ctg1_1947	B3	942.8643989	Bcer	O	Bcer	FALSE	NA	NA	NA
Bc_ctg1_1948	B3	463.3711204	Bcer	O	Bcer	FALSE	NA	NA	NA
Bc_ctg1_1949	B3	17.47506658	Bcer	P	Bcer	FALSE	NA	NA	NA
Bc_ctg1_195	B3	18.26426314	Bcer	K	Bcer	FALSE	NA	NA	NA
Bc_ctg1_1950	B3	111.8404261	Bcer	P	Bcer	FALSE	NA	NA	NA
Bc_ctg1_1951	B3	19.72991388	Bcer	P	Bcer	FALSE	NA	NA	NA
Bc_ctg1_1952	B3	40.47450905	Bcer	P	Bcer	FALSE	NA	NA	NA
Bc_ctg1_1953	B3	2.142104936	Bcer	NA	Bcer	FALSE	NA	NA	NA
Bc_ctg1_1954	B3	65.05234463	Bcer	CO	Bcer	FALSE	NA	NA	NA
Bc_ctg1_1955	B3	4.171467507	Bcer	L	Bcer	FALSE	NA	NA	NA
Bc_ctg1_1956	B3	1313.78678	Bcer	E	Bcer	FALSE	NA	NA	NA
Bc_ctg1_1957	B3	305.3063246	Bcer	P	Bcer	FALSE	NA	NA	NA
Bc_ctg1_1958	B3	1.465650746	Bcer	NA	Bcer	FALSE	NA	NA	NA
Bc_ctg1_1959	B3	20.74459517	Bcer	S	Bcer	FALSE	NA	NA	NA
Bc_ctg1_196	B3	40.92547851	Bcer	EGP	Bcer	FALSE	NA	NA	NA
Bc_ctg1_1960	B3	0.563711825	Bcer	NA	Bcer	FALSE	NA	NA	NA
Bc_ctg1_1961	B3	36.97949574	Bcer	S	Bcer	FALSE	NA	NA	NA
Bc_ctg1_1962	B3	17.24958185	Bcer	NA	Bcer	FALSE	NA	NA	NA
Bc_ctg1_1963	B3	11.6124636	Bcer	I	Bcer	FALSE	NA	NA	NA
Bc_ctg1_1964	B3	3620.382827	Bcer	S	Bcer	FALSE	NA	NA	NA
Bc_ctg1_1965	B3	5099.562656	Bcer	C	Bcer	FALSE	NA	NA	NA
Bc_ctg1_1966	B3	0.45096946	Bcer	M	Bcer	FALSE	NA	NA	NA
Bc_ctg1_1967	B3	0	Bcer	NA	Bcer	FALSE	NA	NA	NA
Bc_ctg1_1968	B3	9.132131569	Bcer	S	Bcer	FALSE	NA	NA	NA
Bc_ctg1_1969	B3	0	Bcer	NA	Bcer	FALSE	NA	NA	NA
Bc_ctg1_197	B3	151.638481	Bcer	V	Bcer	FALSE	NA	NA	NA
Bc_ctg1_1970	B3	5.073406427	Bcer	K	Bcer	FALSE	NA	NA	NA
Bc_ctg1_1971	B3	6.877284268	Bcer	S	Bcer	FALSE	NA	NA	NA
Bc_ctg1_1972	B3	1640.401412	Bcer	I	Bcer	FALSE	NA	NA	NA
Bc_ctg1_1973	B3	812.4214826	Bcer	I	Bcer	FALSE	NA	NA	NA
Bc_ctg1_1974	B3	1701.733258	Bcer	I	Bcer	FALSE	NA	NA	NA
Bc_ctg1_1975	B3	0	Bcer	S	Bcer	FALSE	NA	NA	NA
Bc_ctg1_1976	B3	29.9894691	Bcer	M	Bcer	FALSE	NA	NA	NA
Bc_ctg1_1977	B3	1762.163166	Bcer	E	Bcer	FALSE	NA	NA	NA
Bc_ctg1_1978	B3	0	Bcer	S	Bcer	FALSE	NA	NA	NA
Bc_ctg1_1979	B3	60.3171653	Bcer	S	Bcer	FALSE	NA	NA	NA
Bc_ctg1_1980	B3	150.5110573	Bcer	NT	Bcer	FALSE	NA	NA	NA
Bc_ctg1_1981	B3	45.43517312	Bcer	K	Bcer	FALSE	NA	NA	NA
Bc_ctg1_1983	B3	21.64653409	Bcer	EGP	Bcer	FALSE	NA	NA	NA
Bc_ctg1_1984	B3	241.9451154	Bcer	E	Bcer	FALSE	NA	NA	NA
Bc_ctg1_1985	B3	5.637118253	Bcer	NA	Bcer	FALSE	NA	NA	NA
Bc_ctg1_1986	B3	6.764541903	Bcer	NA	Bcer	FALSE	NA	NA	NA
Bc_ctg1_1987	B3	239.8030105	Bcer	U	Bcer	FALSE	NA	NA	NA
Bc_ctg1_1988	B3	35.1756179	Bcer	NA	Bcer	FALSE	NA	NA	NA

Bc_ctg1_1989	B3	97.63488813	Bcer	S	Bcer	FALSE	NA	NA	NA
Bc_ctg1_199	B3	22.54847301	Bcer	V	Bcer	FALSE	NA	NA	NA
Bc_ctg1_1990	B3	17.02409712	Bcer	J	Bcer	FALSE	NA	NA	NA
Bc_ctg1_1991	B3	50.73406427	Bcer	S	Bcer	FALSE	NA	NA	NA
Bc_ctg1_1992	B3	0.22548473	Bcer	NA	Bcer	FALSE	NA	NA	NA
Bc_ctg1_1993	B3	31.79334694	Bcer	S	Bcer	FALSE	NA	NA	NA
Bc_ctg1_1994	B3	11.49972124	Bcer	S	Bcer	FALSE	NA	NA	NA
Bc_ctg1_1995	B3	0.112742365	Bcer	K	Bcer	FALSE	NA	NA	NA
Bc_ctg1_1996	B3	43.29306818	Bcer	S	Bcer	FALSE	NA	NA	NA
Bc_ctg1_1997	B3	1.014681285	Bcer	S	Bcer	FALSE	NA	NA	NA
Bc_ctg1_1998	B3	1.578393111	Bcer	O	Bcer	FALSE	NA	NA	NA
Bc_ctg1_1999	B3	122.7764355	Bcer	T	Bcer	FALSE	NA	NA	NA
Bc_ctg1_2	B3	4.735179332	Bcer	S	Bcer	FALSE	NA	NA	NA
Bc_ctg1_20	B3	7.328253728	Bcer	S	Bcer	FALSE	NA	NA	NA
Bc_ctg1_200	B3	0.112742365	Bcer	NA	Bcer	FALSE	NA	NA	NA
Bc_ctg1_2000	B3	354.5747381	Bcer	T	Bcer	FALSE	NA	NA	NA
Bc_ctg1_2001	B3	13.30359908	Bcer	EG	Bcer	FALSE	NA	NA	NA
Bc_ctg1_2002	B3	3.833240412	Bcer	NA	Bcer	FALSE	NA	NA	NA
Bc_ctg1_2003	B3	4.735179332	Bcer	J	Bcer	FALSE	NA	NA	NA
Bc_ctg1_2004	B3	7.328253728	Bcer	H	Bcer	FALSE	NA	NA	NA
Bc_ctg1_2005	B3	1198.000371	Bcer	NT	Bcer	FALSE	NA	NA	NA
Bc_ctg1_2006	B3	160.4323855	Bcer	NT	Bcer	FALSE	NA	NA	NA
Bc_ctg1_2007	B3	11.38697887	Bcer	S	Bcer	FALSE	NA	NA	NA
Bc_ctg1_2008	B3	16.34764293	Bcer	E	Bcer	FALSE	NA	NA	NA
Bc_ctg1_2009	B3	50.28309481	Bcer	S	Bcer	FALSE	NA	NA	NA
Bc_ctg1_201	B3	0.45096946	Bcer	I	Bcer	FALSE	NA	NA	NA
Bc_ctg1_2010	B3	29.31301491	Bcer	K	Bcer	FALSE	NA	NA	NA
Bc_ctg1_2011	B3	0.67645419	Bcer	S	Bcer	FALSE	NA	NA	NA
Bc_ctg1_2012	B3	0.67645419	Bcer	S	Bcer	FALSE	NA	NA	NA
Bc_ctg1_2013	B3	4.622436967	Bcer	P	Bcer	FALSE	NA	NA	NA
Bc_ctg1_2014	B3	2.818559126	Bcer	P	Bcer	FALSE	NA	NA	NA
Bc_ctg1_2015	B3	97.7476305	Bcer	E	Bcer	FALSE	NA	NA	NA
Bc_ctg1_2016	B3	1519.090627	Bcer	P	Bcer	FALSE	NA	NA	NA
Bc_ctg1_2017	B3	1.803877841	Bcer	NA	Bcer	FALSE	NA	NA	NA
Bc_ctg1_2018	B3	1.127423651	Bcer	NA	Bcer	FALSE	NA	NA	NA
Bc_ctg1_2019	B3	4.284209872	Bcer	NA	Bcer	FALSE	NA	NA	NA
Bc_ctg1_202	B3	1.578393111	Bcer	NA	Bcer	FALSE	NA	NA	NA
Bc_ctg1_2020	B3	86.58613636	Bcer	S	Bcer	FALSE	NA	NA	NA
Bc_ctg1_2021	B3	95.38004083	Bcer	S	Bcer	FALSE	NA	NA	NA
Bc_ctg1_2022	B3	260.7730904	Bcer	P	Bcer	FALSE	NA	NA	NA
Bc_ctg1_2023	B3	6.200830078	Bcer	P	Bcer	FALSE	NA	NA	NA
Bc_ctg1_2024	B3	2.480332031	Bcer	P	Bcer	FALSE	NA	NA	NA
Bc_ctg1_2025	B3	643.4206774	Bcer	F	Bcer	FALSE	NA	NA	NA
Bc_ctg1_2026	B3	2.818559126	Bcer	F	Bcer	FALSE	NA	NA	NA
Bc_ctg1_2027	B3	157.9520534	Bcer	E	Bcer	FALSE	NA	NA	NA
Bc_ctg1_2028	B3	185.6866752	Bcer	E	Bcer	FALSE	NA	NA	NA
Bc_ctg1_2029	B3	5.862602983	Bcer	S	Bcer	FALSE	NA	NA	NA
Bc_ctg1_203	B3	1.465650746	Bcer	S	Bcer	FALSE	NA	NA	NA
Bc_ctg1_2030	B3	14.205538	Bcer	G	Bcer	FALSE	NA	NA	NA
Bc_ctg1_2031	B3	0.112742365	Bcer	M	Bcer	FALSE	NA	NA	NA
Bc_ctg1_2032	B3	12.96537198	Bcer	S	Bcer	FALSE	NA	NA	NA
Bc_ctg1_2033	B3	9.019389204	Bcer	EGP	Bcer	FALSE	NA	NA	NA
Bc_ctg1_2034	B3	8.906646839	Bcer	K	Bcer	FALSE	NA	NA	NA
Bc_ctg1_2035	B3	257.7290465	Bcer	S	Bcer	FALSE	NA	NA	NA
Bc_ctg1_2036	B3	980.858576	Bcer	C	Bcer	FALSE	NA	NA	NA
Bc_ctg1_2037	B3	69.90026633	Bcer	J	Bcer	FALSE	NA	NA	NA
Bc_ctg1_2038	B3	182.9808585	Bcer	M	Bcer	FALSE	NA	NA	NA
Bc_ctg1_2039	B3	117.9285138	Bcer	S	Bcer	FALSE	NA	NA	NA
Bc_ctg1_204	B3	1.014681285	Bcer	S	Bcer	FALSE	NA	NA	NA
Bc_ctg1_2040	B3	63.81217862	Bcer	NT	Bcer	FALSE	NA	NA	NA
Bc_ctg1_2041	B3	26.83268288	Bcer	L	Bcer	FALSE	NA	NA	NA
Bc_ctg1_2042	B3	0.789196555	Bcer	D	Bcer	FALSE	NA	NA	NA

Bc_ctg1_2043	B3	0.22548473	Bcer	D	Bcer	FALSE	NA	NA	NA
Bc_ctg1_2044	B3	1.803877841	Bcer	NA	Bcer	FALSE	NA	NA	NA
Bc_ctg1_2045	B3	1.240166016	Bcer	NA	Bcer	FALSE	NA	NA	NA
Bc_ctg1_2046	B3	0.563711825	Bcer	EGP	Bcer	FALSE	NA	NA	NA
Bc_ctg1_2047	B3	0.45096946	Bcer	S	Bcer	FALSE	NA	NA	NA
Bc_ctg1_2048	B3	2.705816761	Bcer	J	Bcer	FALSE	NA	NA	NA
Bc_ctg1_2049	B3	17.81329368	Bcer	S	Bcer	FALSE	NA	NA	NA
Bc_ctg1_205	B3	2.480332031	Bcer	NA	Bcer	FALSE	NA	NA	NA
Bc_ctg1_2050	B3	0.563711825	Bcer	S	Bcer	FALSE	NA	NA	NA
Bc_ctg1_2051	B3	0	Bcer	Q	Bcer	TRUE	NA	NA	NA
Bc_ctg1_2052	B3	5.186148792	Bcer	K	Bcer	FALSE	NA	NA	NA
Bc_ctg1_2053	B3	0.112742365	Bcer	S	Bcer	FALSE	NA	NA	NA
Bc_ctg1_2054	B3	7.102768998	Bcer	K	Bcer	FALSE	NA	NA	NA
Bc_ctg1_2055	B3	1.578393111	Bcer	P	Bcer	FALSE	NA	NA	NA
Bc_ctg1_2056	B3	3.607755682	Bcer	P	Bcer	FALSE	NA	NA	NA
Bc_ctg1_2057	B3	0.789196555	Bcer	F	Bcer	FALSE	NA	NA	NA
Bc_ctg1_2058	B3	1872.876168	Bcer	M	Bcer	FALSE	NA	NA	NA
Bc_ctg1_2059	B3	27.28365234	Bcer	P	Bcer	FALSE	NA	NA	NA
Bc_ctg1_206	B3	0	Bcer	NA	Bcer	FALSE	NA	NA	NA
Bc_ctg1_2060	B3	25.59251687	Bcer	P	Bcer	FALSE	NA	NA	NA
Bc_ctg1_2061	B3	23.90138139	Bcer	P	Bcer	FALSE	NA	NA	NA
Bc_ctg1_2062	B3	33.82270952	Bcer	P	Bcer	FALSE	NA	NA	NA
Bc_ctg1_2063	B3	2.818559126	Bcer	T	Bcer	FALSE	NA	NA	NA
Bc_ctg1_2064	B3	17.36232422	Bcer	S	Bcer	FALSE	NA	NA	NA
Bc_ctg1_2065	B3	35.51384499	Bcer	O	Bcer	FALSE	NA	NA	NA
Bc_ctg1_2066	B3	429.2101838	Bcer	K	Bcer	FALSE	NA	NA	NA
Bc_ctg1_2067	B3	131.9085671	Bcer	S	Bcer	FALSE	NA	NA	NA
Bc_ctg1_2068	B3	15.89667347	Bcer	U	Bcer	FALSE	NA	NA	NA
Bc_ctg1_2069	B3	93.57616299	Bcer	M	Bcer	FALSE	NA	NA	NA
Bc_ctg1_207	B3	58.73877219	Bcer	S	Bcer	FALSE	NA	NA	NA
Bc_ctg1_2070	B3	59.97893821	Bcer	S	Bcer	FALSE	NA	NA	NA
Bc_ctg1_2071	B3	0	Bcer	F	Bcer	FALSE	NA	NA	NA
Bc_ctg1_2072	B3	0	Bcer	F	Bcer	FALSE	NA	NA	NA
Bc_ctg1_2073	B3	6955.0765	Bcer	G	Bcer	FALSE	NA	NA	NA
Bc_ctg1_2074	B3	7519.577522	Bcer	G	Bcer	FALSE	NA	NA	NA
Bc_ctg1_2075	B3	3036.377376	Bcer	G	Bcer	FALSE	NA	NA	NA
Bc_ctg1_2076	B3	4615.334198	Bcer	F	Bcer	FALSE	NA	NA	NA
Bc_ctg1_2077	B3	7851.040075	Bcer	G	Bcer	FALSE	NA	NA	NA
Bc_ctg1_2078	B3	2751.928389	Bcer	K	Bcer	FALSE	NA	NA	NA
Bc_ctg1_2079	B3	47.80276278	Bcer	O	Bcer	FALSE	NA	NA	NA
Bc_ctg1_208	B3	7.891965554	Bcer	S	Bcer	FALSE	NA	NA	NA
Bc_ctg1_2080	B3	447.6999316	Bcer	K	Bcer	FALSE	NA	NA	NA
Bc_ctg1_2081	B3	0	Bcer	NA	Bcer	FALSE	NA	NA	NA
Bc_ctg1_2082	B3	2.931301491	Bcer	CP	Bcer	FALSE	NA	NA	NA
Bc_ctg1_2083	B3	1.465650746	Bcer	S	Bcer	FALSE	NA	NA	NA
Bc_ctg1_2084	B3	9.132131569	Bcer	T	Bcer	FALSE	NA	NA	NA
Bc_ctg1_2085	B3	30.55318093	Bcer	K	Bcer	FALSE	NA	NA	NA
Bc_ctg1_2087	B3	0	Bcer	NA	Bcer	FALSE	NA	NA	NA
Bc_ctg1_2088	B3	0.112742365	Bcer	S	Bcer	FALSE	NA	NA	NA
Bc_ctg1_2089	B3	0.67645419	Bcer	I	Bcer	FALSE	NA	NA	NA
Bc_ctg1_209	B3	19.84265625	Bcer	P	Bcer	FALSE	NA	NA	NA
Bc_ctg1_2090	B3	0	Bcer	S	Bcer	FALSE	NA	NA	NA
Bc_ctg1_2091	B3	0	Bcer	S	Bcer	FALSE	NA	NA	NA
Bc_ctg1_2092	B3	0.112742365	Bcer	S	Bcer	FALSE	NA	NA	NA
Bc_ctg1_2093	B3	637.445332	Bcer	OU	Bcer	FALSE	NA	NA	NA
Bc_ctg1_2094	B3	12.40166016	Bcer	G	Bcer	FALSE	NA	NA	NA
Bc_ctg1_2095	B3	172.8340456	Bcer	K	Bcer	FALSE	NA	NA	NA
Bc_ctg1_2096	B3	389.5248713	Bcer	S	Bcer	FALSE	NA	NA	NA
Bc_ctg1_2097	B3	274.4149165	Bcer	S	Bcer	FALSE	NA	NA	NA
Bc_ctg1_2098	B3	85.00774325	Bcer	F	Bcer	FALSE	NA	NA	NA
Bc_ctg1_2099	B3	5.186148792	Bcer	S	Bcer	FALSE	NA	NA	NA
Bc_ctg1_21	B3	1.691135476	Bcer	S	Bcer	FALSE	NA	NA	NA

Bc_ctg1_210	B3	7.215511363	Bcer	P	Bcer	FALSE	NA	NA	NA
Bc_ctg1_2100	B3	286.3656072	Bcer	C	Bcer	FALSE	NA	NA	NA
Bc_ctg1_2101	B3	211.8429039	Bcer	S	Bcer	FALSE	NA	NA	NA
Bc_ctg1_2102	B3	21.98476119	Bcer	S	Bcer	FALSE	NA	NA	NA
Bc_ctg1_2103	B3	21.53379172	Bcer	S	Bcer	FALSE	NA	NA	NA
Bc_ctg1_2104	B3	412.0733443	Bcer	M	Bcer	FALSE	NA	NA	NA
Bc_ctg1_2105	B3	343.0750169	Bcer	F	Bcer	FALSE	NA	NA	NA
Bc_ctg1_2106	B3	34.61190607	Bcer	S	Bcer	FALSE	NA	NA	NA
Bc_ctg1_2107	B3	467.7680726	Bcer	L	Bcer	FALSE	NA	NA	NA
Bc_ctg1_2108	B3	199.4412438	Bcer	L	Bcer	FALSE	NA	NA	NA
Bc_ctg1_2109	B3	2.705816761	Bcer	S	Bcer	FALSE	NA	NA	NA
Bc_ctg1_211	B3	11.6124636	Bcer	I	Bcer	FALSE	NA	NA	NA
Bc_ctg1_2110	B3	7.102768998	Bcer	NA	Bcer	FALSE	NA	NA	NA
Bc_ctg1_2111	B3	3.495013317	Bcer	K	Bcer	FALSE	NA	NA	NA
Bc_ctg1_2112	B3	2.593074396	Bcer	NA	Bcer	FALSE	NA	NA	NA
Bc_ctg1_2113	B3	4.396952237	Bcer	NA	Bcer	FALSE	NA	NA	NA
Bc_ctg1_2114	B3	3.495013317	Bcer	NA	Bcer	FALSE	NA	NA	NA
Bc_ctg1_2115	B3	0.45096946	Bcer	K	Bcer	FALSE	NA	NA	NA
Bc_ctg1_2116	B3	235.4060582	Bcer	S	Bcer	FALSE	NA	NA	NA
Bc_ctg1_2117	B3	5.411633522	Bcer	K	Bcer	FALSE	NA	NA	NA
Bc_ctg1_2118	B3	3.607755682	Bcer	K	Bcer	FALSE	NA	NA	NA
Bc_ctg1_2119	B3	6.764541903	Bcer	S	Bcer	FALSE	NA	NA	NA
Bc_ctg1_212	B3	349.614074	Bcer	I	Bcer	FALSE	NA	NA	NA
Bc_ctg1_2120	B3	5.862602983	Bcer	L	Bcer	FALSE	NA	NA	NA
Bc_ctg1_2121	B3	1.465650746	Bcer	S	Bcer	FALSE	NA	NA	NA
Bc_ctg1_2122	B3	11.16149414	Bcer	V	Bcer	FALSE	NA	NA	NA
Bc_ctg1_2123	B3	133.0359908	Bcer	O	Bcer	FALSE	NA	NA	NA
Bc_ctg1_2124	B3	129.4282351	Bcer	M	Bcer	FALSE	NA	NA	NA
Bc_ctg1_2125	B3	340.8201696	Bcer	D	Bcer	FALSE	NA	NA	NA
Bc_ctg1_2126	B3	96.95843394	Bcer	D	Bcer	FALSE	NA	NA	NA
Bc_ctg1_2127	B3	52.31245738	Bcer	C	Bcer	FALSE	NA	NA	NA
Bc_ctg1_2128	B3	239.6902681	Bcer	J	Bcer	FALSE	NA	NA	NA
Bc_ctg1_2129	B3	1439.832744	Bcer	U	Bcer	FALSE	NA	NA	NA
Bc_ctg1_213	B3	2.367589666	Bcer	H	Bcer	FALSE	NA	NA	NA
Bc_ctg1_2130	B3	1.691135476	Bcer	NA	Bcer	FALSE	NA	NA	NA
Bc_ctg1_2131	B3	2.142104936	Bcer	V	Bcer	FALSE	NA	NA	NA
Bc_ctg1_2132	B3	0.67645419	Bcer	NA	Bcer	FALSE	NA	NA	NA
Bc_ctg1_2133	B3	0.112742365	Bcer	NA	Bcer	FALSE	NA	NA	NA
Bc_ctg1_2134	B3	0.90193892	Bcer	NA	Bcer	FALSE	NA	NA	NA
Bc_ctg1_2135	B3	0.90193892	Bcer	KT	Bcer	FALSE	Bc_Ga0417192_01_rn14	Other	NA
Bc_ctg1_2136	B3	3.156786221	Bcer	NA	Bcer	FALSE	Bc_Ga0417192_01_rn14	Other	NA
Bc_ctg1_2137	B3	4600.903175	Bcer	J	Bcer	FALSE	Bc_Ga0417192_01_rn14	Other	NA
Bc_ctg1_2138	B3	0	Bcer	K	Bcer	FALSE	Bc_Ga0417192_01_rn14	Regulation	SMCOG1255
Bc_ctg1_2139	B3	0.789196555	Bcer	S	Bcer	FALSE	Bc_Ga0417192_01_rn14	Other	NA
Bc_ctg1_214	B3	0.563711825	Bcer	S	Bcer	FALSE	NA	NA	NA
Bc_ctg1_2140	B3	4.847921697	Bcer	L	Bcer	FALSE	Bc_Ga0417192_01_rn14	Other	NA
Bc_ctg1_2141	B3	1085.821718	Bcer	M	Bcer	FALSE	Bc_Ga0417192_01_rn14	Other	NA
Bc_ctg1_2142	B3	208.460633	Bcer	K	Bcer	FALSE	Bc_Ga0417192_01_rn14	Regulation	SMCOG1167
Bc_ctg1_2143	B3	43.40581054	Bcer	S	Bcer	FALSE	Bc_Ga0417192_01_rn14	Other	NA
Bc_ctg1_2144	B3	16.4603853	Bcer	S	Bcer	FALSE	Bc_Ga0417192_01_rn14	Other	NA
Bc_ctg1_2145	B3	92.11051225	Bcer	K	Bcer	FALSE	Bc_Ga0417192_01_rn14	Other	NA
Bc_ctg1_2146	B3	807.911788	Bcer	M	Bcer	FALSE	Bc_Ga0417192_01_rn14	Core biosynthesis	None
Bc_ctg1_2147	B3	4.058725142	Bcer	M	Bcer	FALSE	Bc_Ga0417192_01_rn14	Ancillary biosynthesis	SMCOG1152
Bc_ctg1_2148	B3	521.2079536	Bcer	M	Bcer	FALSE	Bc_Ga0417192_01_rn14	Core biosynthesis	None
Bc_ctg1_2149	B3	6.088087713	Bcer	G	Bcer	FALSE	Bc_Ga0417192_01_rn14	Ancillary biosynthesis	SMCOG1235
Bc_ctg1_215	B3	28.29833363	Bcer	L	Bcer	FALSE	NA	NA	NA
Bc_ctg1_2150	B3	119.9578764	Bcer	K	Bcer	FALSE	Bc_Ga0417192_01_rn14	Regulation	SMCOG1112
Bc_ctg1_2151	B3	0	Bcer	G	Bcer	FALSE	Bc_Ga0417192_01_rn14	Other	NA
Bc_ctg1_2152	B3	8.681162109	Bcer	P	Bcer	FALSE	Bc_Ga0417192_01_rn14	Other	NA
Bc_ctg1_2153	B3	11.16149414	Bcer	G	Bcer	FALSE	Bc_Ga0417192_01_rn14	Other	NA
Bc_ctg1_2154	B3	232.0237873	Bcer	G	Bcer	FALSE	Bc_Ga0417192_01_rn14	Other	NA
Bc_ctg1_2155	B3	13.41634144	Bcer	G	Bcer	FALSE	Bc_Ga0417192_01_rn14	Other	NA

Bc_ctg1_2156	B3	392.230688	Bcer	G	Bcer	FALSE	Bc_Ga0417192_01_rn14	Other	NA
Bc_ctg1_2157	B3	43.51855291	Bcer	G	Bcer	FALSE	Bc_Ga0417192_01_rn14	Other	NA
Bc_ctg1_2158	B3	1.465650746	Bcer	E	Bcer	FALSE	NA	NA	NA
Bc_ctg1_2159	B3	0.22548473	Bcer	E	Bcer	FALSE	NA	NA	NA
Bc_ctg1_216	B3	2.593074396	Bcer	NA	Bcer	FALSE	NA	NA	NA
Bc_ctg1_2160	B3	0.90193892	Bcer	G	Bcer	FALSE	NA	NA	NA
Bc_ctg1_2161	B3	13.64182617	Bcer	G	Bcer	FALSE	NA	NA	NA
Bc_ctg1_2162	B3	0	Bcer	G	Bcer	FALSE	NA	NA	NA
Bc_ctg1_2163	B3	5.073406427	Bcer	C	Bcer	FALSE	NA	NA	NA
Bc_ctg1_2164	B3	80.72353338	Bcer	I	Bcer	FALSE	NA	NA	NA
Bc_ctg1_2165	B3	44.1950071	Bcer	E	Bcer	FALSE	NA	NA	NA
Bc_ctg1_2166	B3	1.691135476	Bcer	S	Bcer	FALSE	NA	NA	NA
Bc_ctg1_2167	B3	18.48974787	Bcer	K	Bcer	FALSE	NA	NA	NA
Bc_ctg1_2168	B3	13.98005327	Bcer	G	Bcer	FALSE	NA	NA	NA
Bc_ctg1_2169	B3	0	Bcer	NA	Bcer	FALSE	NA	NA	NA
Bc_ctg1_217	B3	123.5656321	Bcer	I	Bcer	FALSE	NA	NA	NA
Bc_ctg1_2170	B3	0.338227095	Bcer	C	Bcer	FALSE	NA	NA	NA
Bc_ctg1_2171	B3	0.90193892	Bcer	C	Bcer	FALSE	NA	NA	NA
Bc_ctg1_2172	B3	0.22548473	Bcer	P	Bcer	FALSE	NA	NA	NA
Bc_ctg1_2173	B3	104.39943	Bcer	H	Bcer	FALSE	NA	NA	NA
Bc_ctg1_2174	B3	24.35235085	Bcer	NA	Bcer	FALSE	NA	NA	NA
Bc_ctg1_2175	B3	0.112742365	Bcer	L	Bcer	FALSE	NA	NA	NA
Bc_ctg1_2177	B3	221.6514897	Bcer	C	Bcer	FALSE	NA	NA	NA
Bc_ctg1_2178	B3	12.06343306	Bcer	S	Bcer	FALSE	NA	NA	NA
Bc_ctg1_2179	B3	45.09694602	Bcer	S	Bcer	FALSE	NA	NA	NA
Bc_ctg1_218	B3	0	Bcer	NA	Bcer	FALSE	NA	NA	NA
Bc_ctg1_2180	B3	40.02353959	Bcer	S	Bcer	FALSE	NA	NA	NA
Bc_ctg1_2181	B3	708.2475373	Bcer	M	Bcer	FALSE	NA	NA	NA
Bc_ctg1_2182	B3	39.91079723	Bcer	E	Bcer	FALSE	NA	NA	NA
Bc_ctg1_2184	B3	3888.709655	Bcer	MT	Bcer	FALSE	NA	NA	NA
Bc_ctg1_2185	B3	8.681162109	Bcer	F	Bcer	FALSE	NA	NA	NA
Bc_ctg1_2186	B3	24.23960849	Bcer	T	Bcer	FALSE	NA	NA	NA
Bc_ctg1_2187	B3	43.96952237	Bcer	M	Bcer	FALSE	NA	NA	NA
Bc_ctg1_2188	B3	0.338227095	Bcer	NA	Bcer	FALSE	NA	NA	NA
Bc_ctg1_2189	B3	28.29833363	Bcer	M	Bcer	FALSE	NA	NA	NA
Bc_ctg1_219	B3	21.30830699	Bcer	S	Bcer	FALSE	NA	NA	NA
Bc_ctg1_2190	B3	244.0872203	Bcer	T	Bcer	FALSE	NA	NA	NA
Bc_ctg1_2191	B3	41.71467507	Bcer	S	Bcer	FALSE	NA	NA	NA
Bc_ctg1_2192	B3	5.524375888	Bcer	S	Bcer	FALSE	NA	NA	NA
Bc_ctg1_2193	B3	2.593074396	Bcer	NA	Bcer	FALSE	NA	NA	NA
Bc_ctg1_2194	B3	0.112742365	Bcer	S	Bcer	FALSE	NA	NA	NA
Bc_ctg1_2195	B3	78.35594371	Bcer	S	Bcer	FALSE	NA	NA	NA
Bc_ctg1_2196	B3	234.1658922	Bcer	L	Bcer	FALSE	NA	NA	NA
Bc_ctg1_2197	B3	27.39639471	Bcer	S	Bcer	FALSE	NA	NA	NA
Bc_ctg1_2198	B3	0	Bcer	S	Bcer	FALSE	NA	NA	NA
Bc_ctg1_2199	B3	0	Bcer	S	Bcer	FALSE	NA	NA	NA
Bc_ctg1_22	B3	1.691135476	Bcer	S	Bcer	FALSE	NA	NA	NA
Bc_ctg1_220	B3	3.720498047	Bcer	J	Bcer	FALSE	NA	NA	NA
Bc_ctg1_2200	B3	1.691135476	Bcer	S	Bcer	FALSE	NA	NA	NA
Bc_ctg1_2201	B3	0.45096946	Bcer	K	Bcer	FALSE	NA	NA	NA
Bc_ctg1_2202	B3	0	Bcer	K	Bcer	FALSE	NA	NA	NA
Bc_ctg1_2203	B3	66.85622248	Bcer	S	Bcer	FALSE	NA	NA	NA
Bc_ctg1_2204	B3	152.878647	Bcer	V	Bcer	FALSE	Bc_Ga0417192_01_rn15	Other	NA
Bc_ctg1_2205	B3	49.38115589	Bcer	V	Bcer	FALSE	Bc_Ga0417192_01_rn15	Transport	SMCOG1000
Bc_ctg1_2206	B3	51.63600319	Bcer	M	Bcer	FALSE	Bc_Ga0417192_01_rn15	Transport	SMCOG1029
Bc_ctg1_2207	B3	0.563711825	Bcer	K	Bcer	FALSE	Bc_Ga0417192_01_rn15	Other	NA
Bc_ctg1_2208	B3	6.088087713	Bcer	S	Bcer	FALSE	Bc_Ga0417192_01_rn15	Other	NA
Bc_ctg1_2209	B3	22.66121538	Bcer	G	Bcer	FALSE	Bc_Ga0417192_01_rn15	Transport	SMCOG1106
Bc_ctg1_221	B3	0	Bcer	NA	Bcer	FALSE	NA	NA	NA
Bc_ctg1_2210	B3	20.51911044	Bcer	P	Bcer	FALSE	Bc_Ga0417192_01_rn15	Other	NA
Bc_ctg1_2211	B3	27.05816761	Bcer	S	Bcer	FALSE	Bc_Ga0417192_01_rn15	Other	NA
Bc_ctg1_2212	B3	37.76869229	Bcer	T	Bcer	FALSE	Bc_Ga0417192_01_rn15	Regulation	SMCOG1003

Bc_ctg1_2213	B3	8.906646839	Bcer	K	Bcer	FALSE	Bc_Ga0417192_01_rn15	Regulation	SMCOG1008
Bc_ctg1_2214	B3	27.84736417	Bcer	M	Bcer	FALSE	Bc_Ga0417192_01_rn15	Core biosynthesis	SMCOG1010
Bc_ctg1_2215	B3	61.33184659	Bcer	E	Bcer	FALSE	Bc_Ga0417192_01_rn15	Other	NA
Bc_ctg1_2216	B3	230.3326518	Bcer	K	Bcer	FALSE	Bc_Ga0417192_01_rn15	Other	NA
Bc_ctg1_2217	B3	198.7647896	Bcer	M	Bcer	FALSE	Bc_Ga0417192_01_rn15	Core biosynthesis	SMCOG1010
Bc_ctg1_2218	B3	231.7983025	Bcer	M	Bcer	FALSE	Bc_Ga0417192_01_rn15	Ancillary biosynthesis	SMCOG1152
Bc_ctg1_2219	B3	138.3348819	Bcer	S	Bcer	FALSE	Bc_Ga0417192_01_rn15	Core biosynthesis	None
Bc_ctg1_222	B3	2.480332031	Bcer	K	Bcer	FALSE	NA	NA	NA
Bc_ctg1_2220	B3	144.0847425	Bcer	M	Bcer	FALSE	Bc_Ga0417192_01_rn15	Other	NA
Bc_ctg1_2221	B3	65.95428356	Bcer	M	Bcer	FALSE	Bc_Ga0417192_01_rn15	Other	NA
Bc_ctg1_2222	B3	40.69999378	Bcer	M	Bcer	FALSE	Bc_Ga0417192_01_rn15	Core biosynthesis	None
Bc_ctg1_2223	B3	66.17976829	Bcer	M	Bcer	FALSE	Bc_Ga0417192_01_rn15	Core biosynthesis	SMCOG1045
Bc_ctg1_2224	B3	63.13572443	Bcer	M	Bcer	FALSE	Bc_Ga0417192_01_rn15	Core biosynthesis	SMCOG1045
Bc_ctg1_2225	B3	8.455677379	Bcer	S	Bcer	FALSE	Bc_Ga0417192_01_rn15	Other	NA
Bc_ctg1_2226	B3	14.88199219	Bcer	M	Bcer	FALSE	Bc_Ga0417192_01_rn15	Core biosynthesis	None
Bc_ctg1_2227	B3	64.26314808	Bcer	M	Bcer	FALSE	Bc_Ga0417192_01_rn15	Core biosynthesis	SMCOG1146
Bc_ctg1_2228	B3	47.46453569	Bcer	E	Bcer	FALSE	Bc_Ga0417192_01_rn15	Core biosynthesis	SMCOG1056
Bc_ctg1_2229	B3	33.48448242	Bcer	GM	Bcer	FALSE	Bc_Ga0417192_01_rn15	Core biosynthesis	SMCOG1010
Bc_ctg1_223	B3	3.945982777	Bcer	C	Bcer	FALSE	NA	NA	NA
Bc_ctg1_2230	B3	11.16149414	Bcer	M	Bcer	FALSE	Bc_Ga0417192_01_rn15	Core biosynthesis	SMCOG1064
Bc_ctg1_2231	B3	11.38697887	Bcer	GM	Bcer	FALSE	Bc_Ga0417192_01_rn15	Other	NA
Bc_ctg1_2232	B3	101.0171591	Bcer	D	Bcer	FALSE	Bc_Ga0417192_01_rn15	Ancillary biosynthesis	SMCOG1216
Bc_ctg1_2233	B3	66.40525302	Bcer	M	Bcer	FALSE	Bc_Ga0417192_01_rn15	Ancillary biosynthesis	SMCOG1216
Bc_ctg1_2234	B3	56.48392489	Bcer	D	Bcer	FALSE	Bc_Ga0417192_01_rn15	Ancillary biosynthesis	SMCOG1216
Bc_ctg1_2235	B3	122.4382084	Bcer	I	Bcer	FALSE	Bc_Ga0417192_01_rn15	Ancillary biosynthesis	SMCOG1270
Bc_ctg1_2236	B3	247.5822337	Bcer	D	Bcer	FALSE	Bc_Ga0417192_01_rn15	Other	NA
Bc_ctg1_2237	B3	0.90193892	Bcer	K	Bcer	FALSE	Bc_Ga0417192_01_rn15	Other	NA
Bc_ctg1_2238	B3	1.352908381	Bcer	M	Bcer	FALSE	Bc_Ga0417192_01_rn15	Other	NA
Bc_ctg1_2239	B3	12.51440252	Bcer	V	Bcer	FALSE	Bc_Ga0417192_01_rn15	Other	NA
Bc_ctg1_224	B3	7.666480824	Bcer	S	Bcer	FALSE	NA	NA	NA
Bc_ctg1_2240	B3	2.593074396	Bcer	V	Bcer	FALSE	Bc_Ga0417192_01_rn15	Transport	SMCOG1000
Bc_ctg1_2241	B3	14.65650746	Bcer	KTV	Bcer	FALSE	NA	NA	NA
Bc_ctg1_2242	B3	14.09279563	Bcer	D	Bcer	FALSE	NA	NA	NA
Bc_ctg1_2243	B3	943.7663378	Bcer	M	Bcer	FALSE	NA	NA	NA
Bc_ctg1_2244	B3	171.1429101	Bcer	S	Bcer	FALSE	NA	NA	NA
Bc_ctg1_2245	B3	10.37229758	Bcer	S	Bcer	FALSE	NA	NA	NA
Bc_ctg1_2246	B3	8.568419744	Bcer	C	Bcer	FALSE	NA	NA	NA
Bc_ctg1_2247	B3	3.607755682	Bcer	C	Bcer	FALSE	NA	NA	NA
Bc_ctg1_2248	B3	7.215511363	Bcer	CP	Bcer	FALSE	NA	NA	NA
Bc_ctg1_2249	B3	0.45096946	Bcer	C	Bcer	FALSE	NA	NA	NA
Bc_ctg1_225	B3	0.67645419	Bcer	G	Bcer	FALSE	NA	NA	NA
Bc_ctg1_2250	B3	1.916620206	Bcer	C	Bcer	FALSE	NA	NA	NA
Bc_ctg1_2251	B3	1.127423651	Bcer	C	Bcer	FALSE	NA	NA	NA
Bc_ctg1_2252	B3	2.029362571	Bcer	C	Bcer	FALSE	NA	NA	NA
Bc_ctg1_2253	B3	0.45096946	Bcer	C	Bcer	FALSE	NA	NA	NA
Bc_ctg1_2254	B3	0.45096946	Bcer	C	Bcer	FALSE	NA	NA	NA
Bc_ctg1_2255	B3	0.67645419	Bcer	C	Bcer	FALSE	NA	NA	NA
Bc_ctg1_2256	B3	0.112742365	Bcer	C	Bcer	FALSE	NA	NA	NA
Bc_ctg1_2257	B3	15.55844638	Bcer	T	Bcer	FALSE	NA	NA	NA
Bc_ctg1_2258	B3	24.91606268	Bcer	S	Bcer	FALSE	NA	NA	NA
Bc_ctg1_2259	B3	10.48503995	Bcer	S	Bcer	FALSE	NA	NA	NA
Bc_ctg1_226	B3	9.132131569	Bcer	NA	Bcer	FALSE	NA	NA	NA
Bc_ctg1_2260	B3	830.460261	Bcer	C	Bcer	FALSE	NA	NA	NA
Bc_ctg1_2261	B3	3797.613824	Bcer	C	Bcer	FALSE	NA	NA	NA
Bc_ctg1_2262	B3	1207.357987	Bcer	C	Bcer	FALSE	NA	NA	NA
Bc_ctg1_2263	B3	2223.166696	Bcer	C	Bcer	FALSE	NA	NA	NA
Bc_ctg1_2264	B3	417.7104625	Bcer	C	Bcer	FALSE	NA	NA	NA
Bc_ctg1_2265	B3	356.0403888	Bcer	C	Bcer	FALSE	NA	NA	NA
Bc_ctg1_2266	B3	96.73294921	Bcer	C	Bcer	FALSE	NA	NA	NA
Bc_ctg1_2267	B3	706.330917	Bcer	C	Bcer	FALSE	NA	NA	NA
Bc_ctg1_2268	B3	180.8387535	Bcer	S	Bcer	FALSE	NA	NA	NA
Bc_ctg1_2269	B3	70.80220525	Bcer	S	Bcer	FALSE	NA	NA	NA

Bc_ctg1_2270	B3	218.8329306	Bcer	F	Bcer	FALSE	NA	NA	NA
Bc_ctg1_2271	B3	1692.939354	Bcer	E	Bcer	FALSE	NA	NA	NA
Bc_ctg1_2272	B3	130.6684011	Bcer	S	Bcer	FALSE	NA	NA	NA
Bc_ctg1_2273	B3	70.35123579	Bcer	G	Bcer	FALSE	NA	NA	NA
Bc_ctg1_2274	B3	19.39168679	Bcer	T	Bcer	FALSE	NA	NA	NA
Bc_ctg1_2275	B3	178.809391	Bcer	G	Bcer	FALSE	NA	NA	NA
Bc_ctg1_2276	B3	0.338227095	Bcer	S	Bcer	FALSE	NA	NA	NA
Bc_ctg1_2277	B3	37.4304652	Bcer	S	Bcer	FALSE	NA	NA	NA
Bc_ctg1_2278	B3	13.52908381	Bcer	NA	Bcer	FALSE	NA	NA	NA
Bc_ctg1_2279	B3	0.112742365	Bcer	NA	Bcer	FALSE	NA	NA	NA
Bc_ctg1_2280	B3	37.0922381	Bcer	P	Bcer	FALSE	NA	NA	NA
Bc_ctg1_2281	B3	48.47921697	Bcer	J	Bcer	FALSE	NA	NA	NA
Bc_ctg1_2282	B3	10.03407049	Bcer	E	Bcer	FALSE	NA	NA	NA
Bc_ctg1_2283	B3	0.22548473	Bcer	S	Bcer	FALSE	NA	NA	NA
Bc_ctg1_2284	B3	45.66065785	Bcer	J	Bcer	FALSE	NA	NA	NA
Bc_ctg1_2285	B3	396.2894132	Bcer	J	Bcer	FALSE	NA	NA	NA
Bc_ctg1_2286	B3	63.02298206	Bcer	F	Bcer	FALSE	NA	NA	NA
Bc_ctg1_2287	B3	1414.465712	Bcer	J	Bcer	FALSE	NA	NA	NA
Bc_ctg1_2288	B3	683.8951864	Bcer	K	Bcer	FALSE	NA	NA	NA
Bc_ctg1_2289	B3	564.7265065	Bcer	G	Bcer	FALSE	NA	NA	NA
Bc_ctg1_229	B3	0.22548473	Bcer	NA	Bcer	FALSE	NA	NA	NA
Bc_ctg1_2290	B3	811.632286	Bcer	M	Bcer	FALSE	NA	NA	NA
Bc_ctg1_2291	B3	3513.164837	Bcer	G	Bcer	FALSE	NA	NA	NA
Bc_ctg1_2292	B3	74.74818803	Bcer	T	Bcer	FALSE	NA	NA	NA
Bc_ctg1_2293	B3	14.99473455	Bcer	S	Bcer	FALSE	NA	NA	NA
Bc_ctg1_2294	B3	710.1641575	Bcer	F	Bcer	FALSE	NA	NA	NA
Bc_ctg1_2295	B3	562.2461745	Bcer	K	Bcer	FALSE	NA	NA	NA
Bc_ctg1_2296	B3	14.09279563	Bcer	K	Bcer	FALSE	NA	NA	NA
Bc_ctg1_2297	B3	99.43876598	Bcer	I	Bcer	FALSE	NA	NA	NA
Bc_ctg1_2298	B3	83.76757723	Bcer	I	Bcer	FALSE	NA	NA	NA
Bc_ctg1_2299	B3	63.47395152	Bcer	I	Bcer	FALSE	NA	NA	NA
Bc_ctg1_23	B3	5.298891157	Bcer	E	Bcer	FALSE	NA	NA	NA
Bc_ctg1_230	B3	3.156786221	Bcer	NA	Bcer	FALSE	NA	NA	NA
Bc_ctg1_2300	B3	56.70940962	Bcer	I	Bcer	FALSE	NA	NA	NA
Bc_ctg1_2301	B3	406.0979989	Bcer	C	Bcer	FALSE	NA	NA	NA
Bc_ctg1_2302	B3	1.916620206	Bcer	I	Bcer	FALSE	NA	NA	NA
Bc_ctg1_2303	B3	2.142104936	Bcer	L	Bcer	FALSE	NA	NA	NA
Bc_ctg1_2304	B3	21.98476119	Bcer	T	Bcer	FALSE	NA	NA	NA
Bc_ctg1_2305	B3	0	Bcer	S	Bcer	FALSE	NA	NA	NA
Bc_ctg1_2306	B3	32.9207706	Bcer	K	Bcer	FALSE	NA	NA	NA
Bc_ctg1_2307	B3	528.7616921	Bcer	E	Bcer	FALSE	NA	NA	NA
Bc_ctg1_2308	B3	19.27894442	Bcer	T	Bcer	FALSE	NA	NA	NA
Bc_ctg1_2309	B3	42.95484108	Bcer	T	Bcer	FALSE	NA	NA	NA
Bc_ctg1_231	B3	9.357616299	Bcer	IQ	Bcer	TRUE	NA	NA	NA
Bc_ctg1_2310	B3	11.9506907	Bcer	V	Bcer	FALSE	NA	NA	NA
Bc_ctg1_2311	B3	15.89667347	Bcer	V	Bcer	FALSE	NA	NA	NA
Bc_ctg1_2312	B3	389.9758407	Bcer	E	Bcer	FALSE	NA	NA	NA
Bc_ctg1_2313	B3	464.2730593	Bcer	M	Bcer	FALSE	NA	NA	NA
Bc_ctg1_2314	B3	2.818559126	Bcer	S	Bcer	FALSE	NA	NA	NA
Bc_ctg1_2315	B3	12.96537198	Bcer	NA	Bcer	FALSE	NA	NA	NA
Bc_ctg1_2316	B3	0.112742365	Bcer	NA	Bcer	FALSE	NA	NA	NA
Bc_ctg1_2317	B3	1.352908381	Bcer	NA	Bcer	FALSE	NA	NA	NA
Bc_ctg1_2318	B3	0.112742365	Bcer	K	Bcer	FALSE	NA	NA	NA
Bc_ctg1_2319	B3	170.2409712	Bcer	J	Bcer	FALSE	NA	NA	NA
Bc_ctg1_232	B3	38.55788885	Bcer	S	Bcer	FALSE	NA	NA	NA
Bc_ctg1_2320	B3	19.39168679	Bcer	S	Bcer	FALSE	NA	NA	NA
Bc_ctg1_2321	B3	35.96481445	Bcer	V	Bcer	FALSE	NA	NA	NA
Bc_ctg1_2322	B3	0.45096946	Bcer	S	Bcer	FALSE	NA	NA	NA
Bc_ctg1_2323	B3	21.98476119	Bcer	G	Bcer	FALSE	NA	NA	NA
Bc_ctg1_2324	B3	333.6046582	Bcer	E	Bcer	FALSE	NA	NA	NA
Bc_ctg1_2325	B3	235.2933159	Bcer	E	Bcer	FALSE	NA	NA	NA
Bc_ctg1_2326	B3	6.088087713	Bcer	EGP	Bcer	FALSE	NA	NA	NA

Bc_ctg1_2327	B3	2.593074396	Bcer	K	Bcer	FALSE	NA	NA	NA
Bc_ctg1_2328	B3	141.9426376	Bcer	C	Bcer	FALSE	NA	NA	NA
Bc_ctg1_2329	B3	9.921328125	Bcer	M	Bcer	FALSE	NA	NA	NA
Bc_ctg1_233	B3	9.470358664	Bcer	V	Bcer	FALSE	NA	NA	NA
Bc_ctg1_2330	B3	75.98835404	Bcer	S	Bcer	FALSE	NA	NA	NA
Bc_ctg1_2331	B3	94.47810191	Bcer	S	Bcer	FALSE	NA	NA	NA
Bc_ctg1_2332	B3	108.7963823	Bcer	G	Bcer	FALSE	NA	NA	NA
Bc_ctg1_2333	B3	346.5700302	Bcer	S	Bcer	FALSE	NA	NA	NA
Bc_ctg1_2334	B3	63.24846679	Bcer	P	Bcer	FALSE	NA	NA	NA
Bc_ctg1_2335	B3	57.04763672	Bcer	HP	Bcer	FALSE	NA	NA	NA
Bc_ctg1_2336	B3	42.72935635	Bcer	P	Bcer	FALSE	NA	NA	NA
Bc_ctg1_2337	B3	35.96481445	Bcer	P	Bcer	FALSE	NA	NA	NA
Bc_ctg1_2338	B3	3.495013317	Bcer	S	Bcer	FALSE	NA	NA	NA
Bc_ctg1_2339	B3	0.338227095	Bcer	S	Bcer	FALSE	NA	NA	NA
Bc_ctg1_234	B3	0.22548473	Bcer	NA	Bcer	FALSE	NA	NA	NA
Bc_ctg1_2340	B3	52.98891157	Bcer	H	Bcer	FALSE	NA	NA	NA
Bc_ctg1_2341	B3	0	Bcer	NA	Bcer	FALSE	NA	NA	NA
Bc_ctg1_2343	B3	265.0573002	Bcer	C	Bcer	FALSE	NA	NA	NA
Bc_ctg1_2344	B3	544.5456232	Bcer	C	Bcer	FALSE	NA	NA	NA
Bc_ctg1_2345	B3	52.53794211	Bcer	M	Bcer	FALSE	NA	NA	NA
Bc_ctg1_2346	B3	0	Bcer	M	Bcer	FALSE	NA	NA	NA
Bc_ctg1_2347	B3	0.338227095	Bcer	S	Bcer	FALSE	NA	NA	NA
Bc_ctg1_2348	B3	40.02353959	Bcer	S	Bcer	FALSE	NA	NA	NA
Bc_ctg1_2349	B3	1.240166016	Bcer	S	Bcer	FALSE	NA	NA	NA
Bc_ctg1_235	B3	3.382270952	Bcer	NA	Bcer	FALSE	NA	NA	NA
Bc_ctg1_2350	B3	17.24958185	Bcer	P	Bcer	FALSE	NA	NA	NA
Bc_ctg1_2351	B3	34.04819425	Bcer	F	Bcer	FALSE	NA	NA	NA
Bc_ctg1_2352	B3	83.8803196	Bcer	S	Bcer	FALSE	Bc_Ga0417192_01_rn16	Ancillary biosynthesis	SMCOG1179
Bc_ctg1_2353	B3	29.53849964	Bcer	EGP	Bcer	FALSE	Bc_Ga0417192_01_rn16	Other	NA
Bc_ctg1_2354	B3	47.46453569	Bcer	L	Bcer	FALSE	Bc_Ga0417192_01_rn16	Other	NA
Bc_ctg1_2355	B3	49.26841353	Bcer	S	Bcer	FALSE	Bc_Ga0417192_01_rn16	Other	NA
Bc_ctg1_2356	B3	29.53849964	Bcer	V	Bcer	FALSE	Bc_Ga0417192_01_rn16	Transport	SMCOG1000
Bc_ctg1_2357	B3	25.8180016	Bcer	E	Bcer	FALSE	Bc_Ga0417192_01_rn16	Other	NA
Bc_ctg1_2358	B3	5.862602983	Bcer	K	Bcer	FALSE	Bc_Ga0417192_01_rn16	Regulation	SMCOG1078
Bc_ctg1_2359	B3	4.622436967	Bcer	S	Bcer	FALSE	Bc_Ga0417192_01_rn16	Other	NA
Bc_ctg1_236	B3	1.127423651	Bcer	K	Bcer	FALSE	NA	NA	NA
Bc_ctg1_2360	B3	46.11162731	Bcer	E	Bcer	FALSE	Bc_Ga0417192_01_rn16	Other	NA
Bc_ctg1_2361	B3	37.76869229	Bcer	E	Bcer	FALSE	Bc_Ga0417192_01_rn16	Other	NA
Bc_ctg1_2362	B3	60.09168057	Bcer	E	Bcer	FALSE	Bc_Ga0417192_01_rn16	Core biosynthesis	SMCOG1168
Bc_ctg1_2363	B3	21.87201882	Bcer	P	Bcer	FALSE	Bc_Ga0417192_01_rn16	Other	NA
Bc_ctg1_2364	B3	3.382270952	Bcer	S	Bcer	FALSE	Bc_Ga0417192_01_rn16	Ancillary biosynthesis	SMCOG1115
Bc_ctg1_2365	B3	11.16149414	Bcer	T	Bcer	FALSE	Bc_Ga0417192_01_rn16	Regulation	SMCOG1133
Bc_ctg1_2366	B3	0	Bcer	L	Bcer	FALSE	Bc_Ga0417192_01_rn16	Other	SMCOG1164
Bc_ctg1_2367	B3	0.112742365	Bcer	L	Bcer	FALSE	Bc_Ga0417192_01_rn16	Other	SMCOG1026
Bc_ctg1_2368	B3	4.058725142	Bcer	I	Bcer	FALSE	Bc_Ga0417192_01_rn16	Other	NA
Bc_ctg1_2369	B3	8.004707919	Bcer	K	Bcer	FALSE	Bc_Ga0417192_01_rn16	Regulation	SMCOG1016
Bc_ctg1_237	B3	116.3501207	Bcer	NA	Bcer	FALSE	NA	NA	NA
Bc_ctg1_2370	B3	12.17617543	Bcer	T	Bcer	FALSE	Bc_Ga0417192_01_rn16	Regulation	SMCOG1048
Bc_ctg1_2371	B3	481.9736106	Bcer	H	Bcer	FALSE	Bc_Ga0417192_01_rn16	Other	NA
Bc_ctg1_2372	B3	31.45511985	Bcer	T	Bcer	FALSE	Bc_Ga0417192_01_rn16	Ancillary biosynthesis	SMCOG1025
Bc_ctg1_2373	B3	1.691135476	Bcer	S	Bcer	FALSE	Bc_Ga0417192_01_rn16	Other	SMCOG1211
Bc_ctg1_2374	B3	564.7265065	Bcer	T	Bcer	FALSE	Bc_Ga0417192_01_rn16	Other	SMCOG1185
Bc_ctg1_2375	B3	258.180016	Bcer	KT	Bcer	FALSE	Bc_Ga0417192_01_rn16	Other	NA
Bc_ctg1_2376	B3	22.54847301	Bcer	G	Bcer	FALSE	Bc_Ga0417192_01_rn16	Transport	SMCOG1169
Bc_ctg1_2377	B3	139.8005327	Bcer	M	Bcer	FALSE	Bc_Ga0417192_01_rn16	Other	NA
Bc_ctg1_2378	B3	163.1382022	Bcer	M	Bcer	FALSE	Bc_Ga0417192_01_rn16	Core biosynthesis	SMCOG1045
Bc_ctg1_2379	B3	307.3356871	Bcer	M	Bcer	FALSE	Bc_Ga0417192_01_rn16	Other	NA
Bc_ctg1_238	B3	77.45400479	Bcer	S	Bcer	FALSE	NA	NA	NA
Bc_ctg1_2380	B3	33.25899769	Bcer	S	Bcer	FALSE	Bc_Ga0417192_01_rn16	Other	NA
Bc_ctg1_2381	B3	90.53211914	Bcer	NT	Bcer	FALSE	Bc_Ga0417192_01_rn16	Other	SMCOG1061
Bc_ctg1_2382	B3	22.88670011	Bcer	M	Bcer	FALSE	Bc_Ga0417192_01_rn16	Other	NA
Bc_ctg1_2383	B3	17.13683949	Bcer	I	Bcer	FALSE	Bc_Ga0417192_01_rn16	Other	NA

Bc_ctg1_2384	B3	0.112742365	Bcer	M	Bcer	FALSE	Bc_Ga0417192_01_rn16	Other	NA
Bc_ctg1_2385	B3	30.55318093	Bcer	M	Bcer	FALSE	Bc_Ga0417192_01_rn16	Core biosynthesis	SMCOG1123
Bc_ctg1_2386	B3	86.36065163	Bcer	S	Bcer	FALSE	Bc_Ga0417192_01_rn16	Core biosynthesis	None
Bc_ctg1_2387	B3	432.9306818	Bcer	S	Bcer	FALSE	Bc_Ga0417192_01_rn16	Other	NA
Bc_ctg1_2388	B3	296.6251625	Bcer	M	Bcer	FALSE	Bc_Ga0417192_01_rn16	Core biosynthesis	SMCOG1123
Bc_ctg1_239	B3	53.10165394	Bcer	T	Bcer	FALSE	NA	NA	NA
Bc_ctg1_2390	B3	0	Bcer	NA	Bcer	FALSE	Bc_Ga0417192_01_rn16	Other	NA
Bc_ctg1_2391	B3	2.029362571	Bcer	GM	Bcer	FALSE	Bc_Ga0417192_01_rn16	Ancillary biosynthesis	SMCOG1010
Bc_ctg1_2392	B3	20.6318528	Bcer	K	Bcer	FALSE	Bc_Ga0417192_01_rn16	Regulation	SMCOG1057
Bc_ctg1_2393	B3	166.2949885	Bcer	S	Bcer	FALSE	Bc_Ga0417192_01_rn16	Transport	SMCOG1000
Bc_ctg1_2394	B3	26.04348633	Bcer	K	Bcer	FALSE	Bc_Ga0417192_01_rn16	Regulation	SMCOG1057
Bc_ctg1_2395	B3	191.6620206	Bcer	V	Bcer	FALSE	Bc_Ga0417192_01_rn16	Transport	SMCOG1049
Bc_ctg1_2396	B3	64.60137517	Bcer	O	Bcer	FALSE	NA	NA	NA
Bc_ctg1_2397	B3	355.8149041	Bcer	S	Bcer	FALSE	NA	NA	NA
Bc_ctg1_2398	B3	1400.598401	Bcer	M	Bcer	FALSE	NA	NA	NA
Bc_ctg1_2399	B3	380.0545126	Bcer	S	Bcer	FALSE	NA	NA	NA
Bc_ctg1_24	B3	3.044043856	Bcer	E	Bcer	FALSE	NA	NA	NA
Bc_ctg1_240	B3	2.593074396	Bcer	K	Bcer	FALSE	NA	NA	NA
Bc_ctg1_2400	B3	20.85733753	Bcer	T	Bcer	FALSE	NA	NA	NA
Bc_ctg1_2401	B3	43.96952237	Bcer	T	Bcer	FALSE	NA	NA	NA
Bc_ctg1_2402	B3	4.396952237	Bcer	EGP	Bcer	FALSE	Bc_Ga0417192_01_rn17	Transport	SMCOG1169
Bc_ctg1_2403	B3	49.26841353	Bcer	M	Bcer	FALSE	Bc_Ga0417192_01_rn17	Other	NA
Bc_ctg1_2404	B3	297.5271014	Bcer	C	Bcer	FALSE	Bc_Ga0417192_01_rn17	Other	NA
Bc_ctg1_2405	B3	31173.71491	Bcer	P	Bcer	FALSE	Bc_Ga0417192_01_rn17	Other	NA
Bc_ctg1_2406	B3	14.88199219	Bcer	GM	Bcer	FALSE	Bc_Ga0417192_01_rn17	Ancillary biosynthesis	SMCOG1010
Bc_ctg1_2407	B3	11.6124636	Bcer	S	Bcer	FALSE	Bc_Ga0417192_01_rn17	Other	NA
Bc_ctg1_2408	B3	0	Bcer	NA	Bcer	FALSE	Bc_Ga0417192_01_rn17	Other	NA
Bc_ctg1_2409	B3	363.2559002	Bcer	M	Bcer	FALSE	Bc_Ga0417192_01_rn17	Core biosynthesis	SMCOG1010
Bc_ctg1_241	B3	18.03877841	Bcer	S	Bcer	FALSE	NA	NA	NA
Bc_ctg1_2410	B3	381.2946786	Bcer	S	Bcer	FALSE	Bc_Ga0417192_01_rn17	Other	NA
Bc_ctg1_2411	B3	22.99944247	Bcer	K	Bcer	FALSE	Bc_Ga0417192_01_rn17	Other	NA
Bc_ctg1_2412	B3	68.88558505	Bcer	JKL	Bcer	FALSE	Bc_Ga0417192_01_rn17	Other	SMCOG1122
Bc_ctg1_2413	B3	378.5888618	Bcer	F	Bcer	FALSE	Bc_Ga0417192_01_rn17	Other	NA
Bc_ctg1_2414	B3	48.25373224	Bcer	E	Bcer	FALSE	Bc_Ga0417192_01_rn17	Other	NA
Bc_ctg1_2415	B3	34.95013317	Bcer	C	Bcer	FALSE	Bc_Ga0417192_01_rn17	Ancillary biosynthesis	None
Bc_ctg1_2416	B3	72.38059836	Bcer	T	Bcer	FALSE	Bc_Ga0417192_01_rn17	Regulation	SMCOG1003
Bc_ctg1_2417	B3	13.41634144	Bcer	J	Bcer	FALSE	NA	NA	NA
Bc_ctg1_2418	B3	0.22548473	Bcer	S	Bcer	FALSE	NA	NA	NA
Bc_ctg1_2419	B3	204.5146502	Bcer	O	Bcer	FALSE	NA	NA	NA
Bc_ctg1_242	B3	8.793904474	Bcer	F	Bcer	FALSE	NA	NA	NA
Bc_ctg1_2420	B3	153.6678436	Bcer	S	Bcer	FALSE	NA	NA	NA
Bc_ctg1_2421	B3	134.8398686	Bcer	S	Bcer	FALSE	NA	NA	NA
Bc_ctg1_2422	B3	279.8265501	Bcer	S	Bcer	FALSE	NA	NA	NA
Bc_ctg1_2423	B3	518.6148792	Bcer	T	Bcer	FALSE	NA	NA	NA
Bc_ctg1_2424	B3	133.712445	Bcer	T	Bcer	FALSE	NA	NA	NA
Bc_ctg1_2425	B3	849.9646901	Bcer	F	Bcer	FALSE	NA	NA	NA
Bc_ctg1_2426	B3	808.9264692	Bcer	L	Bcer	FALSE	NA	NA	NA
Bc_ctg1_2427	B3	114.4335005	Bcer	J	Bcer	FALSE	NA	NA	NA
Bc_ctg1_2428	B3	890.4391992	Bcer	T	Bcer	FALSE	NA	NA	NA
Bc_ctg1_2429	B3	20.51911044	Bcer	S	Bcer	FALSE	NA	NA	NA
Bc_ctg1_243	B3	3.382270952	Bcer	S	Bcer	FALSE	NA	NA	NA
Bc_ctg1_2430	B3	281.5176855	Bcer	J	Bcer	FALSE	NA	NA	NA
Bc_ctg1_2431	B3	1722.252369	Bcer	L	Bcer	FALSE	NA	NA	NA
Bc_ctg1_2432	B3	676.679675	Bcer	J	Bcer	FALSE	NA	NA	NA
Bc_ctg1_2433	B3	333.2664311	Bcer	J	Bcer	FALSE	NA	NA	NA
Bc_ctg1_2434	B3	8.230192649	Bcer	S	Bcer	FALSE	NA	NA	NA
Bc_ctg1_2435	B3	95.26729847	Bcer	M	Bcer	FALSE	NA	NA	NA
Bc_ctg1_2436	B3	0.338227095	Bcer	S	Bcer	FALSE	NA	NA	NA
Bc_ctg1_2437	B3	1548.290899	Bcer	K	Bcer	FALSE	NA	NA	NA
Bc_ctg1_2438	B3	471.2630859	Bcer	D	Bcer	FALSE	NA	NA	NA
Bc_ctg1_2439	B3	305.6445517	Bcer	D	Bcer	FALSE	NA	NA	NA
Bc_ctg1_244	B3	2.367589666	Bcer	E	Bcer	FALSE	NA	NA	NA

Bc_ctg1_2440	B3	144.3102273	Bcer	J	Bcer	FALSE	NA	NA	NA
Bc_ctg1_2441	B3	476.4492347	Bcer	D	Bcer	FALSE	NA	NA	NA
Bc_ctg1_2442	B3	229.5434552	Bcer	S	Bcer	FALSE	NA	NA	NA
Bc_ctg1_2443	B3	313.5365172	Bcer	S	Bcer	FALSE	NA	NA	NA
Bc_ctg1_2444	B3	280.5030042	Bcer	U	Bcer	FALSE	NA	NA	NA
Bc_ctg1_2445	B3	26.60719815	Bcer	J	Bcer	FALSE	NA	NA	NA
Bc_ctg1_2446	B3	0.22548473	Bcer	J	Bcer	FALSE	NA	NA	NA
Bc_ctg1_2447	B3	319.6246049	Bcer	L	Bcer	FALSE	NA	NA	NA
Bc_ctg1_2448	B3	253.3320943	Bcer	L	Bcer	FALSE	NA	NA	NA
Bc_ctg1_2449	B3	9.470358664	Bcer	S	Bcer	FALSE	NA	NA	NA
Bc_ctg1_245	B3	8.117450284	Bcer	GK	Bcer	FALSE	NA	NA	NA
Bc_ctg1_2450	B3	351.3052095	Bcer	L	Bcer	FALSE	NA	NA	NA
Bc_ctg1_2451	B3	1081.988477	Bcer	L	Bcer	FALSE	NA	NA	NA
Bc_ctg1_2452	B3	2120.683887	Bcer	L	Bcer	FALSE	NA	NA	NA
Bc_ctg1_2454	B3	0.789196555	Bcer	S	Bcer	FALSE	NA	NA	NA
Bc_ctg1_2455	B3	1257.52834	Bcer	F	Bcer	FALSE	NA	NA	NA
Bc_ctg1_2456	B3	305.0808398	Bcer	M	Bcer	FALSE	NA	NA	NA
Bc_ctg1_2457	B3	838.9159384	Bcer	H	Bcer	FALSE	NA	NA	NA
Bc_ctg1_2458	B3	613.656693	Bcer	H	Bcer	FALSE	NA	NA	NA
Bc_ctg1_2459	B3	708.8112491	Bcer	J	Bcer	FALSE	NA	NA	NA
Bc_ctg1_246	B3	1.916620206	Bcer	G	Bcer	FALSE	NA	NA	NA
Bc_ctg1_2460	B3	221.6514897	Bcer	S	Bcer	FALSE	NA	NA	NA
Bc_ctg1_2461	B3	29.20027255	Bcer	F	Bcer	FALSE	NA	NA	NA
Bc_ctg1_2462	B3	18.03877841	Bcer	F	Bcer	FALSE	NA	NA	NA
Bc_ctg1_2463	B3	0.22548473	Bcer	Q	Bcer	TRUE	NA	NA	NA
Bc_ctg1_2464	B3	4.396952237	Bcer	FJ	Bcer	FALSE	NA	NA	NA
Bc_ctg1_2465	B3	619.9702654	Bcer	L	Bcer	FALSE	NA	NA	NA
Bc_ctg1_2466	B3	96.95843394	Bcer	S	Bcer	FALSE	NA	NA	NA
Bc_ctg1_2467	B3	433.0434242	Bcer	L	Bcer	FALSE	NA	NA	NA
Bc_ctg1_2468	B3	106.0905655	Bcer	S	Bcer	FALSE	NA	NA	NA
Bc_ctg1_2469	B3	0.22548473	Bcer	S	Bcer	FALSE	NA	NA	NA
Bc_ctg1_247	B3	2.705816761	Bcer	S	Bcer	FALSE	NA	NA	NA
Bc_ctg1_2471	B3	0	Bcer	S	Bcer	FALSE	NA	NA	NA
Bc_ctg1_2472	B3	67.98364613	Bcer	E	Bcer	FALSE	NA	NA	NA
Bc_ctg1_2473	B3	28.52381836	Bcer	F	Bcer	FALSE	NA	NA	NA
Bc_ctg1_2474	B3	176.4418013	Bcer	L	Bcer	FALSE	NA	NA	NA
Bc_ctg1_2475	B3	242.9597967	Bcer	S	Bcer	FALSE	NA	NA	NA
Bc_ctg1_2476	B3	52.53794211	Bcer	L	Bcer	FALSE	NA	NA	NA
Bc_ctg1_2477	B3	17.92603604	Bcer	S	Bcer	FALSE	NA	NA	NA
Bc_ctg1_2478	B3	1.803877841	Bcer	L	Bcer	FALSE	NA	NA	NA
Bc_ctg1_2479	B3	22.66121538	Bcer	H	Bcer	FALSE	NA	NA	NA
Bc_ctg1_248	B3	6.200830078	Bcer	J	Bcer	FALSE	NA	NA	NA
Bc_ctg1_2480	B3	735.0802201	Bcer	K	Bcer	FALSE	NA	NA	NA
Bc_ctg1_2481	B3	758.0796626	Bcer	J	Bcer	FALSE	NA	NA	NA
Bc_ctg1_2482	B3	33.93545188	Bcer	L	Bcer	FALSE	NA	NA	NA
Bc_ctg1_2483	B3	22.43573065	Bcer	J	Bcer	FALSE	NA	NA	NA
Bc_ctg1_2484	B3	91.20857333	Bcer	J	Bcer	FALSE	NA	NA	NA
Bc_ctg1_2485	B3	5.637118253	Bcer	S	Bcer	FALSE	NA	NA	NA
Bc_ctg1_2486	B3	0	Bcer	S	Bcer	FALSE	NA	NA	NA
Bc_ctg1_2488	B3	91.54680042	Bcer	I	Bcer	FALSE	NA	NA	NA
Bc_ctg1_2489	B3	366.6381711	Bcer	F	Bcer	FALSE	NA	NA	NA
Bc_ctg1_249	B3	45.66065785	Bcer	I	Bcer	FALSE	NA	NA	NA
Bc_ctg1_2490	B3	98.19859996	Bcer	J	Bcer	FALSE	NA	NA	NA
Bc_ctg1_2491	B3	674.199343	Bcer	D	Bcer	FALSE	NA	NA	NA
Bc_ctg1_2492	B3	1315.026946	Bcer	M	Bcer	FALSE	NA	NA	NA
Bc_ctg1_2493	B3	967.1040074	Bcer	F	Bcer	FALSE	NA	NA	NA
Bc_ctg1_2494	B3	86.92436345	Bcer	J	Bcer	FALSE	NA	NA	NA
Bc_ctg1_2495	B3	4.847921697	Bcer	S	Bcer	FALSE	NA	NA	NA
Bc_ctg1_2496	B3	566.8686115	Bcer	L	Bcer	FALSE	NA	NA	NA
Bc_ctg1_2497	B3	128.5262962	Bcer	K	Bcer	FALSE	NA	NA	NA
Bc_ctg1_2498	B3	130.4429164	Bcer	S	Bcer	FALSE	NA	NA	NA
Bc_ctg1_2499	B3	196.8481694	Bcer	S	Bcer	FALSE	NA	NA	NA

Bc_ctg1_25	B3	2.705816761	Bcer	K	Bcer	FALSE	NA	NA	NA
Bc_ctg1_250	B3	0.338227095	Bcer	L	Bcer	FALSE	NA	NA	NA
Bc_ctg1_2500	B3	26.49445579	Bcer	J	Bcer	FALSE	NA	NA	NA
Bc_ctg1_2501	B3	49.38115589	Bcer	S	Bcer	FALSE	NA	NA	NA
Bc_ctg1_2502	B3	168.324351	Bcer	S	Bcer	FALSE	NA	NA	NA
Bc_ctg1_2503	B3	104.8503995	Bcer	D	Bcer	FALSE	NA	NA	NA
Bc_ctg1_2504	B3	713.3209437	Bcer	J	Bcer	FALSE	NA	NA	NA
Bc_ctg1_2505	B3	7.440996093	Bcer	KT	Bcer	FALSE	NA	NA	NA
Bc_ctg1_2506	B3	1878.400544	Bcer	D	Bcer	FALSE	NA	NA	NA
Bc_ctg1_2507	B3	925.6148171	Bcer	F	Bcer	FALSE	NA	NA	NA
Bc_ctg1_2508	B3	5945.806848	Bcer	O	Bcer	FALSE	NA	NA	NA
Bc_ctg1_2509	B3	102.4828098	Bcer	F	Bcer	FALSE	NA	NA	NA
Bc_ctg1_251	B3	0.45096946	Bcer	L	Bcer	FALSE	NA	NA	NA
Bc_ctg1_2510	B3	202.59803	Bcer	O	Bcer	FALSE	NA	NA	NA
Bc_ctg1_2511	B3	629.2151394	Bcer	E	Bcer	FALSE	NA	NA	NA
Bc_ctg1_2512	B3	137.8839125	Bcer	EH	Bcer	FALSE	NA	NA	NA
Bc_ctg1_2513	B3	76.43932351	Bcer	EH	Bcer	FALSE	NA	NA	NA
Bc_ctg1_2514	B3	71.25317471	Bcer	EH	Bcer	FALSE	NA	NA	NA
Bc_ctg1_2515	B3	66.74348011	Bcer	H	Bcer	FALSE	NA	NA	NA
Bc_ctg1_2516	B3	24.46509322	Bcer	H	Bcer	FALSE	NA	NA	NA
Bc_ctg1_2517	B3	28.29833363	Bcer	H	Bcer	FALSE	NA	NA	NA
Bc_ctg1_2518	B3	0.338227095	Bcer	K	Bcer	FALSE	NA	NA	NA
Bc_ctg1_2519	B3	63.36120916	Bcer	J	Bcer	FALSE	NA	NA	NA
Bc_ctg1_252	B3	59.75345348	Bcer	S	Bcer	FALSE	NA	NA	NA
Bc_ctg1_2520	B3	693.3655451	Bcer	J	Bcer	FALSE	NA	NA	NA
Bc_ctg1_2523	B3	71.70414417	Bcer	K	Bcer	FALSE	NA	NA	NA
Bc_ctg1_2524	B3	142.2808647	Bcer	S	Bcer	FALSE	NA	NA	NA
Bc_ctg1_2525	B3	442.0628134	Bcer	E	Bcer	FALSE	NA	NA	NA
Bc_ctg1_2526	B3	1657.087282	Bcer	O	Bcer	FALSE	NA	NA	NA
Bc_ctg1_2527	B3	79.14514027	Bcer	O	Bcer	FALSE	NA	NA	NA
Bc_ctg1_2528	B3	86.92436345	Bcer	L	Bcer	FALSE	NA	NA	NA
Bc_ctg1_2529	B3	317.5952424	Bcer	S	Bcer	FALSE	NA	NA	NA
Bc_ctg1_253	B3	15.33296165	Bcer	NA	Bcer	FALSE	NA	NA	NA
Bc_ctg1_2530	B3	319.6246049	Bcer	I	Bcer	FALSE	NA	NA	NA
Bc_ctg1_2531	B3	40.92547851	Bcer	I	Bcer	FALSE	NA	NA	NA
Bc_ctg1_2532	B3	1010.058848	Bcer	J	Bcer	FALSE	NA	NA	NA
Bc_ctg1_2533	B3	70.23849343	Bcer	E	Bcer	FALSE	NA	NA	NA
Bc_ctg1_2534	B3	288.5077122	Bcer	J	Bcer	FALSE	NA	NA	NA
Bc_ctg1_2535	B3	25.02880504	Bcer	J	Bcer	FALSE	NA	NA	NA
Bc_ctg1_2536	B3	140.7024716	Bcer	J	Bcer	FALSE	NA	NA	NA
Bc_ctg1_2537	B3	124.9185405	Bcer	S	Bcer	FALSE	NA	NA	NA
Bc_ctg1_2538	B3	233.3766957	Bcer	K	Bcer	FALSE	NA	NA	NA
Bc_ctg1_2539	B3	6.088087713	Bcer	U	Bcer	FALSE	NA	NA	NA
Bc_ctg1_254	B3	3.720498047	Bcer	K	Bcer	FALSE	NA	NA	NA
Bc_ctg1_2540	B3	249.6115962	Bcer	K	Bcer	FALSE	NA	NA	NA
Bc_ctg1_2541	B3	5490.102209	Bcer	J	Bcer	FALSE	NA	NA	NA
Bc_ctg1_2542	B3	1707.595861	Bcer	J	Bcer	FALSE	NA	NA	NA
Bc_ctg1_2543	B3	2925.213404	Bcer	J	Bcer	FALSE	NA	NA	NA
Bc_ctg1_2544	B3	798.7796564	Bcer	J	Bcer	FALSE	NA	NA	NA
Bc_ctg1_2545	B3	185.6866752	Bcer	J	Bcer	FALSE	NA	NA	NA
Bc_ctg1_2546	B3	9465.510743	Bcer	K	Bcer	FALSE	NA	NA	NA
Bc_ctg1_2547	B3	10001.03698	Bcer	K	Bcer	FALSE	NA	NA	NA
Bc_ctg1_2548	B3	587.5004643	Bcer	J	Bcer	FALSE	NA	NA	NA
Bc_ctg1_2549	B3	2612.127856	Bcer	J	Bcer	FALSE	NA	NA	NA
Bc_ctg1_255	B3	0	Bcer	NA	Bcer	FALSE	NA	NA	NA
Bc_ctg1_2550	B3	1623.264572	Bcer	J	Bcer	FALSE	NA	NA	NA
Bc_ctg1_2551	B3	13105.51074	Bcer	J	Bcer	FALSE	NA	NA	NA
Bc_ctg1_2552	B3	14773.53403	Bcer	J	Bcer	FALSE	NA	NA	NA
Bc_ctg1_2553	B3	1645.700303	Bcer	J	Bcer	FALSE	NA	NA	NA
Bc_ctg1_2554	B3	3648.793903	Bcer	J	Bcer	FALSE	NA	NA	NA
Bc_ctg1_2555	B3	3429.960972	Bcer	J	Bcer	FALSE	NA	NA	NA
Bc_ctg1_2556	B3	1126.296227	Bcer	J	Bcer	FALSE	NA	NA	NA

Bc_ctg1_2557	B3	6104.322613	Bcer	J	Bcer	FALSE	NA	NA	NA
Bc_ctg1_2558	B3	2053.827664	Bcer	J	Bcer	FALSE	NA	NA	NA
Bc_ctg1_2559	B3	2939.644426	Bcer	J	Bcer	FALSE	NA	NA	NA
Bc_ctg1_256	B3	1.465650746	Bcer	Q	Bcer	TRUE	NA	NA	NA
Bc_ctg1_2560	B3	4063.460321	Bcer	J	Bcer	FALSE	NA	NA	NA
Bc_ctg1_2561	B3	2111.664497	Bcer	J	Bcer	FALSE	NA	NA	NA
Bc_ctg1_2562	B3	380.9564515	Bcer	J	Bcer	FALSE	NA	NA	NA
Bc_ctg1_2563	B3	1087.963823	Bcer	J	Bcer	FALSE	NA	NA	NA
Bc_ctg1_2564	B3	1823.495012	Bcer	J	Bcer	FALSE	NA	NA	NA
Bc_ctg1_2565	B3	2040.862292	Bcer	J	Bcer	FALSE	NA	NA	NA
Bc_ctg1_2566	B3	3612.716346	Bcer	J	Bcer	FALSE	NA	NA	NA
Bc_ctg1_2567	B3	476.3364923	Bcer	J	Bcer	FALSE	NA	NA	NA
Bc_ctg1_2568	B3	2424.186333	Bcer	J	Bcer	FALSE	NA	NA	NA
Bc_ctg1_2569	B3	3482.498914	Bcer	J	Bcer	FALSE	NA	NA	NA
Bc_ctg1_257	B3	0	Bcer	Q	Bcer	TRUE	Bc_Ga0417192_01_rn02	Other	NA
Bc_ctg1_2570	B3	1777.383385	Bcer	J	Bcer	FALSE	NA	NA	NA
Bc_ctg1_2571	B3	4053.989963	Bcer	J	Bcer	FALSE	NA	NA	NA
Bc_ctg1_2572	B3	155.4717214	Bcer	J	Bcer	FALSE	NA	NA	NA
Bc_ctg1_2573	B3	2343.011831	Bcer	J	Bcer	FALSE	NA	NA	NA
Bc_ctg1_2574	B3	9305.642069	Bcer	U	Bcer	FALSE	NA	NA	NA
Bc_ctg1_2575	B3	2735.242519	Bcer	F	Bcer	FALSE	NA	NA	NA
Bc_ctg1_2576	B3	4999.560178	Bcer	E	Bcer	FALSE	NA	NA	NA
Bc_ctg1_2577	B3	568.559747	Bcer	J	Bcer	FALSE	NA	NA	NA
Bc_ctg1_2578	B3	0.45096946	Bcer	J	Bcer	FALSE	NA	NA	NA
Bc_ctg1_2579	B3	3433.568728	Bcer	J	Bcer	FALSE	NA	NA	NA
Bc_ctg1_258	B3	16.34764293	Bcer	S	Bcer	FALSE	Bc_Ga0417192_01_rn02	Other	SMCOG1272
Bc_ctg1_2580	B3	1700.718577	Bcer	J	Bcer	FALSE	NA	NA	NA
Bc_ctg1_2581	B3	6784.046332	Bcer	K	Bcer	FALSE	NA	NA	NA
Bc_ctg1_2582	B3	977.2508203	Bcer	J	Bcer	FALSE	NA	NA	NA
Bc_ctg1_2583	B3	124.5803134	Bcer	P	Bcer	FALSE	NA	NA	NA
Bc_ctg1_2584	B3	73.62076438	Bcer	P	Bcer	FALSE	NA	NA	NA
Bc_ctg1_2585	B3	59.18974165	Bcer	P	Bcer	FALSE	NA	NA	NA
Bc_ctg1_2586	B3	100.2279625	Bcer	J	Bcer	FALSE	NA	NA	NA
Bc_ctg1_2587	B3	2393.97138	Bcer	J	Bcer	FALSE	NA	NA	NA
Bc_ctg1_2588	B3	842.4109517	Bcer	J	Bcer	FALSE	NA	NA	NA
Bc_ctg1_2589	B3	1.127423651	Bcer	S	Bcer	FALSE	NA	NA	NA
Bc_ctg1_259	B3	1.691135476	Bcer	NA	Bcer	FALSE	Bc_Ga0417192_01_rn02	Other	NA
Bc_ctg1_2590	B3	1.240166016	Bcer	M	Bcer	FALSE	NA	NA	NA
Bc_ctg1_2591	B3	453.1115651	Bcer	D	Bcer	FALSE	NA	NA	NA
Bc_ctg1_2592	B3	0.22548473	Bcer	S	Bcer	FALSE	NA	NA	NA
Bc_ctg1_2593	B3	64.37589044	Bcer	S	Bcer	FALSE	NA	NA	NA
Bc_ctg1_2594	B3	1.352908381	Bcer	G	Bcer	FALSE	NA	NA	NA
Bc_ctg1_2595	B3	0.112742365	Bcer	NA	Bcer	FALSE	NA	NA	NA
Bc_ctg1_2597	B3	0.67645419	Bcer	G	Bcer	FALSE	NA	NA	NA
Bc_ctg1_2598	B3	310.4924734	Bcer	E	Bcer	FALSE	NA	NA	NA
Bc_ctg1_2599	B3	137.9966548	Bcer	S	Bcer	FALSE	NA	NA	NA
Bc_ctg1_26	B3	159.7559313	Bcer	U	Bcer	FALSE	NA	NA	NA
Bc_ctg1_260	B3	0.563711825	Bcer	S	Bcer	FALSE	Bc_Ga0417192_01_rn02	Other	NA
Bc_ctg1_2600	B3	278.6991264	Bcer	S	Bcer	FALSE	NA	NA	NA
Bc_ctg1_2601	B3	549.2808025	Bcer	G	Bcer	FALSE	NA	NA	NA
Bc_ctg1_2602	B3	1080.410084	Bcer	M	Bcer	FALSE	NA	NA	NA
Bc_ctg1_2603	B3	9.244873934	Bcer	EU	Bcer	FALSE	NA	NA	NA
Bc_ctg1_2604	B3	26.71994052	Bcer	S	Bcer	FALSE	NA	NA	NA
Bc_ctg1_2605	B3	540.8251252	Bcer	S	Bcer	FALSE	NA	NA	NA
Bc_ctg1_2606	B3	72.15511363	Bcer	S	Bcer	FALSE	NA	NA	NA
Bc_ctg1_2607	B3	144.535712	Bcer	IQ	Bcer	TRUE	NA	NA	NA
Bc_ctg1_2608	B3	5.862602983	Bcer	P	Bcer	FALSE	NA	NA	NA
Bc_ctg1_2609	B3	8.117450284	Bcer	P	Bcer	FALSE	NA	NA	NA
Bc_ctg1_261	B3	34.38642134	Bcer	S	Bcer	FALSE	Bc_Ga0417192_01_rn02	Other	NA
Bc_ctg1_2610	B3	3.607755682	Bcer	P	Bcer	FALSE	NA	NA	NA
Bc_ctg1_2611	B3	0.112742365	Bcer	C	Bcer	FALSE	NA	NA	NA
Bc_ctg1_2612	B3	18.94071733	Bcer	NA	Bcer	FALSE	NA	NA	NA

Bc_ctg1_2613	B3	6.313572443	Bcer	C	Bcer	FALSE	NA	NA	NA
Bc_ctg1_2614	B3	3.833240412	Bcer	G	Bcer	FALSE	NA	NA	NA
Bc_ctg1_2615	B3	51.97423029	Bcer	S	Bcer	FALSE	NA	NA	NA
Bc_ctg1_2616	B3	14.88199219	Bcer	EGP	Bcer	FALSE	NA	NA	NA
Bc_ctg1_2617	B3	5.862602983	Bcer	NA	Bcer	FALSE	NA	NA	NA
Bc_ctg1_2618	B3	14.88199219	Bcer	NA	Bcer	FALSE	NA	NA	NA
Bc_ctg1_2619	B3	0	Bcer	S	Bcer	FALSE	NA	NA	NA
Bc_ctg1_262	B3	19.50442915	Bcer	S	Bcer	FALSE	Bc_Ga0417192_01_rn02	Other	NA
Bc_ctg1_2620	B3	3.269528587	Bcer	P	Bcer	FALSE	NA	NA	NA
Bc_ctg1_2621	B3	4.509694602	Bcer	EP	Bcer	FALSE	NA	NA	NA
Bc_ctg1_2622	B3	7.102768998	Bcer	P	Bcer	FALSE	NA	NA	NA
Bc_ctg1_2623	B3	6.651799538	Bcer	E	Bcer	FALSE	NA	NA	NA
Bc_ctg1_2624	B3	19.27894442	Bcer	E	Bcer	FALSE	NA	NA	NA
Bc_ctg1_2625	B3	0	Bcer	S	Bcer	FALSE	NA	NA	NA
Bc_ctg1_2626	B3	0	Bcer	Q	Bcer	TRUE	NA	NA	NA
Bc_ctg1_2627	B3	196.6226847	Bcer	E	Bcer	FALSE	NA	NA	NA
Bc_ctg1_2628	B3	236.4207395	Bcer	E	Bcer	FALSE	NA	NA	NA
Bc_ctg1_2629	B3	133.3742179	Bcer	S	Bcer	FALSE	NA	NA	NA
Bc_ctg1_263	B3	214.5487207	Bcer	S	Bcer	FALSE	Bc_Ga0417192_01_rn02	Other	SMCOG1273
Bc_ctg1_2630	B3	107.4434739	Bcer	E	Bcer	FALSE	NA	NA	NA
Bc_ctg1_2631	B3	4.735179332	Bcer	H	Bcer	FALSE	NA	NA	NA
Bc_ctg1_2632	B3	51.07229137	Bcer	U	Bcer	FALSE	NA	NA	NA
Bc_ctg1_2633	B3	77.56674716	Bcer	P	Bcer	FALSE	NA	NA	NA
Bc_ctg1_2634	B3	129.9919469	Bcer	P	Bcer	FALSE	NA	NA	NA
Bc_ctg1_2635	B3	45.77340021	Bcer	P	Bcer	FALSE	NA	NA	NA
Bc_ctg1_2638	B3	7.891965554	Bcer	K	Bcer	FALSE	NA	NA	NA
Bc_ctg1_2639	B3	2.593074396	Bcer	EG	Bcer	FALSE	NA	NA	NA
Bc_ctg1_264	B3	53.66536576	Bcer	K	Bcer	FALSE	Bc_Ga0417192_01_rn02	Regulation	SMCOG1171
Bc_ctg1_2642	B3	48.36647461	Bcer	I	Bcer	FALSE	NA	NA	NA
Bc_ctg1_2644	B3	13.41634144	Bcer	K	Bcer	FALSE	NA	NA	NA
Bc_ctg1_2645	B3	1.014681285	Bcer	NA	Bcer	FALSE	NA	NA	NA
Bc_ctg1_2646	B3	62.68475497	Bcer	S	Bcer	FALSE	NA	NA	NA
Bc_ctg1_2647	B3	0.112742365	Bcer	S	Bcer	FALSE	NA	NA	NA
Bc_ctg1_2648	B3	0	Bcer	NA	Bcer	FALSE	NA	NA	NA
Bc_ctg1_2649	B3	8.117450284	Bcer	L	Bcer	FALSE	NA	NA	NA
Bc_ctg1_265	B3	0.112742365	Bcer	NA	Bcer	FALSE	Bc_Ga0417192_01_rn02	Other	NA
Bc_ctg1_2650	B3	32.13157404	Bcer	S	Bcer	FALSE	NA	NA	NA
Bc_ctg1_2651	B3	49.26841353	Bcer	M	Bcer	FALSE	NA	NA	NA
Bc_ctg1_2652	B3	9.357616299	Bcer	C	Bcer	FALSE	NA	NA	NA
Bc_ctg1_2654	B3	22.09750355	Bcer	S	Bcer	FALSE	NA	NA	NA
Bc_ctg1_2655	B3	0	Bcer	NA	Bcer	FALSE	NA	NA	NA
Bc_ctg1_2656	B3	103.8357182	Bcer	E	Bcer	FALSE	NA	NA	NA
Bc_ctg1_2657	B3	4.284209872	Bcer	P	Bcer	FALSE	NA	NA	NA
Bc_ctg1_2658	B3	11.16149414	Bcer	EP	Bcer	FALSE	NA	NA	NA
Bc_ctg1_2659	B3	16.34764293	Bcer	P	Bcer	FALSE	NA	NA	NA
Bc_ctg1_266	B3	0.112742365	Bcer	JM	Bcer	FALSE	Bc_Ga0417192_01_rn02	Ancillary biosynthesis	SMCOG1064
Bc_ctg1_2660	B3	23.5631543	Bcer	E	Bcer	FALSE	NA	NA	NA
Bc_ctg1_2661	B3	209.1370872	Bcer	S	Bcer	FALSE	NA	NA	NA
Bc_ctg1_2663	B3	0	Bcer	NA	Bcer	FALSE	NA	NA	NA
Bc_ctg1_2664	B3	0	Bcer	NA	Bcer	FALSE	NA	NA	NA
Bc_ctg1_2665	B3	0	Bcer	NA	Bcer	FALSE	NA	NA	NA
Bc_ctg1_2667	B3	78.80691317	Bcer	E	Bcer	FALSE	NA	NA	NA
Bc_ctg1_2668	B3	41.48919034	Bcer	Q	Bcer	TRUE	NA	NA	NA
Bc_ctg1_2669	B3	113.3060769	Bcer	Q	Bcer	TRUE	NA	NA	NA
Bc_ctg1_267	B3	0.112742365	Bcer	GM	Bcer	FALSE	Bc_Ga0417192_01_rn02	Core biosynthesis	SMCOG1010
Bc_ctg1_2670	B3	5.637118253	Bcer	E	Bcer	FALSE	NA	NA	NA
Bc_ctg1_2671	B3	8.230192649	Bcer	EGP	Bcer	FALSE	NA	NA	NA
Bc_ctg1_2672	B3	199.8922132	Bcer	F	Bcer	FALSE	NA	NA	NA
Bc_ctg1_2673	B3	201.9215758	Bcer	M	Bcer	FALSE	NA	NA	NA
Bc_ctg1_2674	B3	1514.01722	Bcer	JKL	Bcer	FALSE	NA	NA	NA
Bc_ctg1_2675	B3	3.607755682	Bcer	L	Bcer	FALSE	NA	NA	NA
Bc_ctg1_2676	B3	0.45096946	Bcer	S	Bcer	FALSE	NA	NA	NA

Bc_ctg1_2677	B3	3.495013317	Bcer	I	Bcer	FALSE	NA	NA	NA
Bc_ctg1_2678	B3	23.11218484	Bcer	M	Bcer	FALSE	NA	NA	NA
Bc_ctg1_2679	B3	12.40166016	Bcer	E	Bcer	FALSE	NA	NA	NA
Bc_ctg1_268	B3	0	Bcer	H	Bcer	FALSE	Bc_Ga0417192_01_rn02	Other	NA
Bc_ctg1_2680	B3	420.3035369	Bcer	K	Bcer	FALSE	NA	NA	NA
Bc_ctg1_2681	B3	561.7952051	Bcer	L	Bcer	FALSE	NA	NA	NA
Bc_ctg1_2682	B3	143.1828036	Bcer	K	Bcer	FALSE	NA	NA	NA
Bc_ctg1_2683	B3	1.803877841	Bcer	S	Bcer	FALSE	NA	NA	NA
Bc_ctg1_2685	B3	6.764541903	Bcer	S	Bcer	FALSE	NA	NA	NA
Bc_ctg1_2686	B3	39.23434304	Bcer	O	Bcer	FALSE	NA	NA	NA
Bc_ctg1_2687	B3	9.695843394	Bcer	K	Bcer	FALSE	NA	NA	NA
Bc_ctg1_2688	B3	73.39527965	Bcer	O	Bcer	FALSE	NA	NA	NA
Bc_ctg1_2689	B3	206.5440128	Bcer	S	Bcer	FALSE	NA	NA	NA
Bc_ctg1_269	B3	0	Bcer	GM	Bcer	FALSE	Bc_Ga0417192_01_rn02	Core biosynthesis	SMCOG1010
Bc_ctg1_2690	B3	734.741993	Bcer	K	Bcer	FALSE	NA	NA	NA
Bc_ctg1_2691	B3	5.298891157	Bcer	S	Bcer	FALSE	NA	NA	NA
Bc_ctg1_2692	B3	112.6296227	Bcer	S	Bcer	FALSE	NA	NA	NA
Bc_ctg1_2693	B3	590.206281	Bcer	O	Bcer	FALSE	NA	NA	NA
Bc_ctg1_2694	B3	3409.667346	Bcer	O	Bcer	FALSE	NA	NA	NA
Bc_ctg1_2695	B3	49.0429288	Bcer	S	Bcer	FALSE	NA	NA	NA
Bc_ctg1_2696	B3	33.59722479	Bcer	S	Bcer	FALSE	NA	NA	NA
Bc_ctg1_2697	B3	1251.778479	Bcer	F	Bcer	FALSE	NA	NA	NA
Bc_ctg1_2698	B3	467.7680726	Bcer	S	Bcer	FALSE	NA	NA	NA
Bc_ctg1_2699	B3	3.720498047	Bcer	T	Bcer	FALSE	NA	NA	NA
Bc_ctg1_27	B3	72.49334073	Bcer	P	Bcer	FALSE	NA	NA	NA
Bc_ctg1_270	B3	1.465650746	Bcer	NA	Bcer	FALSE	Bc_Ga0417192_01_rn02	Other	NA
Bc_ctg1_2700	B3	26.38171342	Bcer	T	Bcer	FALSE	NA	NA	NA
Bc_ctg1_2702	B3	18.48974787	Bcer	S	Bcer	FALSE	NA	NA	NA
Bc_ctg1_2703	B3	22.66121538	Bcer	Q	Bcer	TRUE	NA	NA	NA
Bc_ctg1_2705	B3	10.93600941	Bcer	S	Bcer	FALSE	NA	NA	NA
Bc_ctg1_2707	B3	6.539057173	Bcer	V	Bcer	FALSE	NA	NA	NA
Bc_ctg1_2708	B3	2.593074396	Bcer	S	Bcer	FALSE	NA	NA	NA
Bc_ctg1_2709	B3	1.916620206	Bcer	V	Bcer	FALSE	NA	NA	NA
Bc_ctg1_271	B3	0	Bcer	S	Bcer	FALSE	Bc_Ga0417192_01_rn02	Other	NA
Bc_ctg1_2710	B3	4.058725142	Bcer	T	Bcer	FALSE	NA	NA	NA
Bc_ctg1_2711	B3	2.593074396	Bcer	T	Bcer	FALSE	NA	NA	NA
Bc_ctg1_2713	B3	0.67645419	Bcer	S	Bcer	FALSE	Bc_Ga0417192_01_rn18	Other	NA
Bc_ctg1_2714	B3	0.112742365	Bcer	Q	Bcer	TRUE	Bc_Ga0417192_01_rn18	Ancillary biosynthesis	SMCOG1089
Bc_ctg1_2715	B3	0.338227095	Bcer	Q	Bcer	TRUE	Bc_Ga0417192_01_rn18	Other	NA
Bc_ctg1_2716	B3	0.789196555	Bcer	G	Bcer	FALSE	Bc_Ga0417192_01_rn18	Other	NA
Bc_ctg1_2717	B3	3.156786221	Bcer	M	Bcer	FALSE	Bc_Ga0417192_01_rn18	Ancillary biosynthesis	SMCOG1010
Bc_ctg1_2718	B3	0.112742365	Bcer	M	Bcer	FALSE	Bc_Ga0417192_01_rn18	Other	NA
Bc_ctg1_2719	B3	2.254847301	Bcer	M	Bcer	FALSE	Bc_Ga0417192_01_rn18	Core biosynthesis	SMCOG1045
Bc_ctg1_272	B3	45.66065785	Bcer	EG	Bcer	FALSE	Bc_Ga0417192_01_rn02	Transport	SMCOG1166
Bc_ctg1_2721	B3	31.79334694	Bcer	EG	Bcer	FALSE	Bc_Ga0417192_01_rn18	Transport	SMCOG1166
Bc_ctg1_2722	B3	0.45096946	Bcer	G	Bcer	FALSE	Bc_Ga0417192_01_rn18	Other	NA
Bc_ctg1_2724	B3	192.2257324	Bcer	F	Bcer	FALSE	NA	NA	NA
Bc_ctg1_2725	B3	1122.462986	Bcer	F	Bcer	FALSE	NA	NA	NA
Bc_ctg1_2726	B3	1774.452084	Bcer	F	Bcer	FALSE	NA	NA	NA
Bc_ctg1_2727	B3	243.6362509	Bcer	F	Bcer	FALSE	NA	NA	NA
Bc_ctg1_2728	B3	90.6448615	Bcer	F	Bcer	FALSE	NA	NA	NA
Bc_ctg1_2729	B3	480.0569904	Bcer	F	Bcer	FALSE	NA	NA	NA
Bc_ctg1_273	B3	5.862602983	Bcer	S	Bcer	FALSE	Bc_Ga0417192_01_rn02	Other	SMCOG1286
Bc_ctg1_2730	B3	1888.434615	Bcer	F	Bcer	FALSE	NA	NA	NA
Bc_ctg1_2731	B3	1305.331103	Bcer	F	Bcer	FALSE	NA	NA	NA
Bc_ctg1_2732	B3	465.4004829	Bcer	F	Bcer	FALSE	NA	NA	NA
Bc_ctg1_2733	B3	173.0595304	Bcer	F	Bcer	FALSE	NA	NA	NA
Bc_ctg1_2734	B3	1115.585702	Bcer	F	Bcer	FALSE	NA	NA	NA
Bc_ctg1_2735	B3	520.08053	Bcer	F	Bcer	FALSE	NA	NA	NA
Bc_ctg1_2736	B3	3.044043856	Bcer	EGP	Bcer	FALSE	NA	NA	NA
Bc_ctg1_2737	B3	4.396952237	Bcer	K	Bcer	FALSE	NA	NA	NA
Bc_ctg1_2738	B3	9.132131569	Bcer	S	Bcer	FALSE	NA	NA	NA

Bc_ctg1_2739	B3	184.1082821	Bcer	O	Bcer	FALSE	NA	NA	NA
Bc_ctg1_274	B3	2.480332031	Bcer	S	Bcer	FALSE	Bc_Ga0417192_01_rn02	Other	NA
Bc_ctg1_2740	B3	0	Bcer	S	Bcer	FALSE	NA	NA	NA
Bc_ctg1_2741	B3	24.91606268	Bcer	I	Bcer	FALSE	NA	NA	NA
Bc_ctg1_2742	B3	183.2063432	Bcer	L	Bcer	FALSE	NA	NA	NA
Bc_ctg1_2743	B3	375.9957874	Bcer	L	Bcer	FALSE	NA	NA	NA
Bc_ctg1_2744	B3	221.9897168	Bcer	S	Bcer	FALSE	NA	NA	NA
Bc_ctg1_2745	B3	17.92603604	Bcer	S	Bcer	FALSE	NA	NA	NA
Bc_ctg1_2746	B3	8540.346895	Bcer	C	Bcer	FALSE	NA	NA	NA
Bc_ctg1_2747	B3	7.779223189	Bcer	Q	Bcer	TRUE	NA	NA	NA
Bc_ctg1_2748	B3	26.71994052	Bcer	P	Bcer	FALSE	NA	NA	NA
Bc_ctg1_2749	B3	5.524375888	Bcer	P	Bcer	FALSE	NA	NA	NA
Bc_ctg1_275	B3	52.19971502	Bcer	G	Bcer	FALSE	Bc_Ga0417192_01_rn02	Other	NA
Bc_ctg1_2750	B3	23.11218484	Bcer	M	Bcer	FALSE	NA	NA	NA
Bc_ctg1_2751	B3	0	Bcer	S	Bcer	FALSE	NA	NA	NA
Bc_ctg1_2752	B3	9.244873934	Bcer	D	Bcer	FALSE	NA	NA	NA
Bc_ctg1_2753	B3	22.43573065	Bcer	K	Bcer	FALSE	NA	NA	NA
Bc_ctg1_2754	B3	6.313572443	Bcer	S	Bcer	FALSE	NA	NA	NA
Bc_ctg1_2755	B3	143.5210307	Bcer	J	Bcer	FALSE	NA	NA	NA
Bc_ctg1_2756	B3	805.9951678	Bcer	J	Bcer	FALSE	NA	NA	NA
Bc_ctg1_2757	B3	1047.489314	Bcer	J	Bcer	FALSE	NA	NA	NA
Bc_ctg1_2758	B3	181.289723	Bcer	I	Bcer	FALSE	NA	NA	NA
Bc_ctg1_2759	B3	4.622436967	Bcer	S	Bcer	FALSE	NA	NA	NA
Bc_ctg1_276	B3	28.29833363	Bcer	G	Bcer	FALSE	Bc_Ga0417192_01_rn02	Ancillary biosynthesis	SMCOG1210
Bc_ctg1_2760	B3	0.112742365	Bcer	E	Bcer	FALSE	NA	NA	NA
Bc_ctg1_2761	B3	8.455677379	Bcer	KT	Bcer	FALSE	NA	NA	NA
Bc_ctg1_2762	B3	65.27782937	Bcer	C	Bcer	FALSE	NA	NA	NA
Bc_ctg1_2763	B3	7.328253728	Bcer	P	Bcer	FALSE	NA	NA	NA
Bc_ctg1_2764	B3	344.8788947	Bcer	E	Bcer	FALSE	NA	NA	NA
Bc_ctg1_2765	B3	47.35179332	Bcer	G	Bcer	FALSE	Bc_Ga0417192_01_rn19	Other	NA
Bc_ctg1_2766	B3	2.142104936	Bcer	NA	Bcer	FALSE	Bc_Ga0417192_01_rn19	Other	NA
Bc_ctg1_2767	B3	7.102768998	Bcer	NA	Bcer	FALSE	Bc_Ga0417192_01_rn19	Other	NA
Bc_ctg1_2768	B3	13.19085671	Bcer	F	Bcer	FALSE	Bc_Ga0417192_01_rn19	Other	NA
Bc_ctg1_2769	B3	66.96896484	Bcer	J	Bcer	FALSE	Bc_Ga0417192_01_rn19	Other	NA
Bc_ctg1_277	B3	11.49972124	Bcer	EG	Bcer	FALSE	Bc_Ga0417192_01_rn02	Other	NA
Bc_ctg1_2770	B3	63.24846679	Bcer	J	Bcer	FALSE	Bc_Ga0417192_01_rn19	Other	NA
Bc_ctg1_2771	B3	21.87201882	Bcer	V	Bcer	FALSE	Bc_Ga0417192_01_rn19	Transport	SMCOG1288
Bc_ctg1_2772	B3	43.96952237	Bcer	KLT	Bcer	FALSE	Bc_Ga0417192_01_rn19	Core biosynthesis	SMCOG1030
Bc_ctg1_278	B3	26.38171342	Bcer	G	Bcer	FALSE	Bc_Ga0417192_01_rn02	Other	NA
Bc_ctg1_2785	B3	2.480332031	Bcer	S	Bcer	FALSE	Bc_Ga0417192_01_rn19	Other	NA
Bc_ctg1_2786	B3	1.578393111	Bcer	S	Bcer	FALSE	Bc_Ga0417192_01_rn19	Other	NA
Bc_ctg1_2787	B3	15.44570401	Bcer	J	Bcer	FALSE	Bc_Ga0417192_01_rn19	Other	NA
Bc_ctg1_2788	B3	22.32298828	Bcer	M	Bcer	FALSE	Bc_Ga0417192_01_rn19	Ancillary biosynthesis	SMCOG1268
Bc_ctg1_2789	B3	7.553738458	Bcer	KT	Bcer	FALSE	Bc_Ga0417192_01_rn19	Other	NA
Bc_ctg1_279	B3	34.04819425	Bcer	G	Bcer	FALSE	Bc_Ga0417192_01_rn02	Ancillary biosynthesis	SMCOG1100
Bc_ctg1_2790	B3	6.426314808	Bcer	C	Bcer	FALSE	Bc_Ga0417192_01_rn19	Other	NA
Bc_ctg1_2791	B3	62.23378551	Bcer	S	Bcer	FALSE	Bc_Ga0417192_01_rn19	Other	NA
Bc_ctg1_2792	B3	10.14681285	Bcer	M	Bcer	FALSE	NA	NA	NA
Bc_ctg1_2795	B3	6792.614752	Bcer	O	Bcer	FALSE	NA	NA	NA
Bc_ctg1_2796	B3	3067.607011	Bcer	O	Bcer	FALSE	NA	NA	NA
Bc_ctg1_2797	B3	140.9279563	Bcer	F	Bcer	FALSE	NA	NA	NA
Bc_ctg1_2798	B3	203.3872266	Bcer	E	Bcer	FALSE	NA	NA	NA
Bc_ctg1_2799	B3	147.3542711	Bcer	G	Bcer	FALSE	NA	NA	NA
Bc_ctg1_28	B3	18.82797496	Bcer	K	Bcer	FALSE	NA	NA	NA
Bc_ctg1_280	B3	64.71411754	Bcer	G	Bcer	FALSE	NA	NA	NA
Bc_ctg1_2800	B3	0.112742365	Bcer	NA	Bcer	FALSE	NA	NA	NA
Bc_ctg1_2801	B3	19.50442915	Bcer	P	Bcer	FALSE	NA	NA	NA
Bc_ctg1_2802	B3	11.49972124	Bcer	P	Bcer	FALSE	NA	NA	NA
Bc_ctg1_2803	B3	138.673109	Bcer	P	Bcer	FALSE	NA	NA	NA
Bc_ctg1_2804	B3	148.5944371	Bcer	C	Bcer	FALSE	NA	NA	NA
Bc_ctg1_2805	B3	155.6972061	Bcer	NA	Bcer	FALSE	NA	NA	NA
Bc_ctg1_2806	B3	0.563711825	Bcer	G	Bcer	FALSE	NA	NA	NA

Bc_ctg1_2807	B3	0	Bcer	NA	Bcer	FALSE	NA	NA	NA
Bc_ctg1_2808	B3	0	Bcer	NA	Bcer	FALSE	NA	NA	NA
Bc_ctg1_2809	B3	1.803877841	Bcer	NA	Bcer	FALSE	NA	NA	NA
Bc_ctg1_281	B3	32.13157404	Bcer	G	Bcer	FALSE	NA	NA	NA
Bc_ctg1_2810	B3	2.142104936	Bcer	E	Bcer	FALSE	NA	NA	NA
Bc_ctg1_2811	B3	26.15622869	Bcer	T	Bcer	FALSE	NA	NA	NA
Bc_ctg1_2812	B3	6.764541903	Bcer	S	Bcer	FALSE	NA	NA	NA
Bc_ctg1_2813	B3	11.83794833	Bcer	S	Bcer	FALSE	NA	NA	NA
Bc_ctg1_2814	B3	0.67645419	Bcer	F	Bcer	FALSE	NA	NA	NA
Bc_ctg1_2815	B3	49.26841353	Bcer	S	Bcer	FALSE	NA	NA	NA
Bc_ctg1_2816	B3	3.833240412	Bcer	EGP	Bcer	FALSE	NA	NA	NA
Bc_ctg1_2817	B3	0.45096946	Bcer	CE	Bcer	FALSE	NA	NA	NA
Bc_ctg1_2818	B3	754.6973917	Bcer	I	Bcer	FALSE	NA	NA	NA
Bc_ctg1_2819	B3	2.029362571	Bcer	P	Bcer	FALSE	NA	NA	NA
Bc_ctg1_282	B3	0.112742365	Bcer	G	Bcer	FALSE	NA	NA	NA
Bc_ctg1_2820	B3	1.578393111	Bcer	ET	Bcer	FALSE	NA	NA	NA
Bc_ctg1_2821	B3	1.352908381	Bcer	E	Bcer	FALSE	NA	NA	NA
Bc_ctg1_2822	B3	59.97893821	Bcer	NT	Bcer	FALSE	NA	NA	NA
Bc_ctg1_2823	B3	37.54320756	Bcer	K	Bcer	FALSE	NA	NA	NA
Bc_ctg1_2824	B3	9435.070304	Bcer	E	Bcer	FALSE	NA	NA	NA
Bc_ctg1_2825	B3	4037.529577	Bcer	E	Bcer	FALSE	NA	NA	NA
Bc_ctg1_2826	B3	5483.788636	Bcer	E	Bcer	FALSE	NA	NA	NA
Bc_ctg1_2827	B3	3085.533047	Bcer	E	Bcer	FALSE	Bc_Ga0417192_01_rn20	Other	NA
Bc_ctg1_2828	B3	226.386669	Bcer	K	Bcer	FALSE	Bc_Ga0417192_01_rn20	Regulation	SMCOG1133
Bc_ctg1_2829	B3	26.15622869	Bcer	NA	Bcer	FALSE	Bc_Ga0417192_01_rn20	Other	NA
Bc_ctg1_283	B3	53.43988103	Bcer	C	Bcer	FALSE	NA	NA	NA
Bc_ctg1_2830	B3	0	Bcer	C	Bcer	FALSE	Bc_Ga0417192_01_rn20	Other	NA
Bc_ctg1_2831	B3	260.9985751	Bcer	G	Bcer	FALSE	Bc_Ga0417192_01_rn20	Ancillary biosynthesis	SMCOG1257
Bc_ctg1_2832	B3	251.077247	Bcer	G	Bcer	FALSE	Bc_Ga0417192_01_rn20	Ancillary biosynthesis	SMCOG1145
Bc_ctg1_2833	B3	118.2667409	Bcer	L	Bcer	FALSE	Bc_Ga0417192_01_rn20	Other	NA
Bc_ctg1_2834	B3	241.3814036	Bcer	S	Bcer	FALSE	Bc_Ga0417192_01_rn20	Other	NA
Bc_ctg1_2835	B3	197.4118812	Bcer	L	Bcer	FALSE	Bc_Ga0417192_01_rn20	Other	NA
Bc_ctg1_2836	B3	19.72991388	Bcer	H	Bcer	FALSE	Bc_Ga0417192_01_rn20	Other	NA
Bc_ctg1_2837	B3	6.088087713	Bcer	H	Bcer	FALSE	Bc_Ga0417192_01_rn20	Ancillary biosynthesis	SMCOG1280
Bc_ctg1_2838	B3	5.411633522	Bcer	S	Bcer	FALSE	Bc_Ga0417192_01_rn20	Other	NA
Bc_ctg1_2839	B3	78.46868608	Bcer	NT	Bcer	FALSE	Bc_Ga0417192_01_rn20	Other	SMCOG1061
Bc_ctg1_284	B3	10.93600941	Bcer	E	Bcer	FALSE	NA	NA	NA
Bc_ctg1_2840	B3	18.48974787	Bcer	Q	Bcer	TRUE	Bc_Ga0417192_01_rn20	Core biosynthesis	SMCOG1002
Bc_ctg1_2841	B3	16.1221582	Bcer	S	Bcer	FALSE	Bc_Ga0417192_01_rn20	Other	NA
Bc_ctg1_2842	B3	74.07173384	Bcer	P	Bcer	FALSE	Bc_Ga0417192_01_rn20	Transport	SMCOG1044
Bc_ctg1_2843	B3	66.06702592	Bcer	P	Bcer	FALSE	Bc_Ga0417192_01_rn20	Other	NA
Bc_ctg1_2844	B3	89.51743785	Bcer	P	Bcer	FALSE	Bc_Ga0417192_01_rn20	Transport	SMCOG1000
Bc_ctg1_2845	B3	5.073406427	Bcer	S	Bcer	FALSE	Bc_Ga0417192_01_rn20	Transport	SMCOG1000
Bc_ctg1_2846	B3	1.240166016	Bcer	G	Bcer	FALSE	Bc_Ga0417192_01_rn20	Other	NA
Bc_ctg1_2847	B3	4.396952237	Bcer	S	Bcer	FALSE	Bc_Ga0417192_01_rn20	Other	NA
Bc_ctg1_2848	B3	10.71052468	Bcer	CO	Bcer	FALSE	Bc_Ga0417192_01_rn20	Other	NA
Bc_ctg1_2849	B3	3.607755682	Bcer	NA	Bcer	FALSE	Bc_Ga0417192_01_rn20	Other	NA
Bc_ctg1_285	B3	7.891965554	Bcer	S	Bcer	FALSE	NA	NA	NA
Bc_ctg1_2852	B3	13.07811435	Bcer	K	Bcer	FALSE	Bc_Ga0417192_01_rn20	Regulation	SMCOG1057
Bc_ctg1_2853	B3	1.352908381	Bcer	G	Bcer	FALSE	Bc_Ga0417192_01_rn20	Transport	SMCOG1020
Bc_ctg1_2854	B3	1.578393111	Bcer	K	Bcer	FALSE	Bc_Ga0417192_01_rn20	Other	NA
Bc_ctg1_2855	B3	30.10221147	Bcer	S	Bcer	FALSE	Bc_Ga0417192_01_rn20	Transport	SMCOG1106
Bc_ctg1_2856	B3	19.95539861	Bcer	S	Bcer	FALSE	Bc_Ga0417192_01_rn20	Other	NA
Bc_ctg1_2857	B3	10.25955522	Bcer	S	Bcer	FALSE	Bc_Ga0417192_01_rn20	Other	NA
Bc_ctg1_2858	B3	175.7653471	Bcer	J	Bcer	FALSE	Bc_Ga0417192_01_rn20	Other	SMCOG1162
Bc_ctg1_2859	B3	0	Bcer	NU	Bcer	FALSE	Bc_Ga0417192_01_rn20	Other	NA
Bc_ctg1_286	B3	0.338227095	Bcer	S	Bcer	FALSE	NA	NA	NA
Bc_ctg1_2860	B3	1.352908381	Bcer	GK	Bcer	FALSE	Bc_Ga0417192_01_rn20	Other	SMCOG1108
Bc_ctg1_2861	B3	567.5450657	Bcer	T	Bcer	FALSE	Bc_Ga0417192_01_rn20	Other	SMCOG1296
Bc_ctg1_2862	B3	826.2887935	Bcer	T	Bcer	FALSE	Bc_Ga0417192_01_rn20	Other	SMCOG1296
Bc_ctg1_2863	B3	158.4030229	Bcer	T	Bcer	FALSE	NA	NA	NA
Bc_ctg1_2864	B3	405.7597718	Bcer	P	Bcer	FALSE	NA	NA	NA

Bc_ctg1_2865	B3	188.392492	Bcer	O	Bcer	FALSE	NA	NA	NA
Bc_ctg1_2866	B3	187.7160378	Bcer	P	Bcer	FALSE	NA	NA	NA
Bc_ctg1_2867	B3	983.0006809	Bcer	P	Bcer	FALSE	NA	NA	NA
Bc_ctg1_2868	B3	11.83794833	Bcer	S	Bcer	FALSE	NA	NA	NA
Bc_ctg1_2869	B3	29.76398437	Bcer	T	Bcer	FALSE	NA	NA	NA
Bc_ctg1_287	B3	0.112742365	Bcer	S	Bcer	FALSE	NA	NA	NA
Bc_ctg1_2870	B3	73.16979492	Bcer	NA	Bcer	FALSE	NA	NA	NA
Bc_ctg1_2871	B3	149.6091184	Bcer	S	Bcer	FALSE	NA	NA	NA
Bc_ctg1_2872	B3	67.08170721	Bcer	P	Bcer	FALSE	NA	NA	NA
Bc_ctg1_2873	B3	1.127423651	Bcer	EG	Bcer	FALSE	NA	NA	NA
Bc_ctg1_2874	B3	0.22548473	Bcer	EG	Bcer	FALSE	NA	NA	NA
Bc_ctg1_2875	B3	39.00885831	Bcer	S	Bcer	FALSE	NA	NA	NA
Bc_ctg1_2876	B3	9.357616299	Bcer	U	Bcer	FALSE	NA	NA	NA
Bc_ctg1_2877	B3	886.1549893	Bcer	S	Bcer	FALSE	NA	NA	NA
Bc_ctg1_2878	B3	20.6318528	Bcer	S	Bcer	FALSE	NA	NA	NA
Bc_ctg1_2879	B3	1.916620206	Bcer	S	Bcer	FALSE	NA	NA	NA
Bc_ctg1_288	B3	1.916620206	Bcer	S	Bcer	FALSE	NA	NA	NA
Bc_ctg1_2880	B3	0	Bcer	S	Bcer	FALSE	NA	NA	NA
Bc_ctg1_2881	B3	0.563711825	Bcer	S	Bcer	FALSE	NA	NA	NA
Bc_ctg1_2882	B3	0	Bcer	P	Bcer	FALSE	NA	NA	NA
Bc_ctg1_2883	B3	1.352908381	Bcer	S	Bcer	FALSE	NA	NA	NA
Bc_ctg1_2884	B3	106.6542773	Bcer	S	Bcer	FALSE	NA	NA	NA
Bc_ctg1_2885	B3	68.99832741	Bcer	NA	Bcer	FALSE	NA	NA	NA
Bc_ctg1_2886	B3	825.2741122	Bcer	C	Bcer	FALSE	NA	NA	NA
Bc_ctg1_2887	B3	11.27423651	Bcer	G	Bcer	FALSE	NA	NA	NA
Bc_ctg1_2888	B3	54.22907759	Bcer	L	Bcer	FALSE	NA	NA	NA
Bc_ctg1_2889	B3	122.5509508	Bcer	J	Bcer	FALSE	NA	NA	NA
Bc_ctg1_2890	B3	20.6318528	Bcer	J	Bcer	FALSE	NA	NA	NA
Bc_ctg1_2891	B3	20.51911044	Bcer	NA	Bcer	FALSE	NA	NA	NA
Bc_ctg1_2892	B3	14.54376509	Bcer	KT	Bcer	FALSE	NA	NA	NA
Bc_ctg1_2893	B3	0	Bcer	I	Bcer	FALSE	NA	NA	NA
Bc_ctg1_2894	B3	3.833240412	Bcer	E	Bcer	FALSE	Bc_Ga0417192_01_rn21	Ancillary biosynthesis	SMCOG1038
Bc_ctg1_2895	B3	5.749860618	Bcer	E	Bcer	FALSE	Bc_Ga0417192_01_rn21	Ancillary biosynthesis	SMCOG1247
Bc_ctg1_2896	B3	0.112742365	Bcer	K	Bcer	FALSE	Bc_Ga0417192_01_rn21	Other	NA
Bc_ctg1_2897	B3	0.338227095	Bcer	P	Bcer	FALSE	Bc_Ga0417192_01_rn21	Other	NA
Bc_ctg1_2898	B3	0.67645419	Bcer	S	Bcer	FALSE	Bc_Ga0417192_01_rn21	Other	NA
Bc_ctg1_2899	B3	17.24958185	Bcer	E	Bcer	FALSE	Bc_Ga0417192_01_rn21	Other	NA
Bc_ctg1_29	B3	8.793904474	Bcer	S	Bcer	FALSE	NA	NA	NA
Bc_ctg1_290	B3	411.058663	Bcer	E	Bcer	FALSE	NA	NA	NA
Bc_ctg1_2900	B3	148.7071795	Bcer	G	Bcer	FALSE	Bc_Ga0417192_01_rn21	Ancillary biosynthesis	SMCOG1145
Bc_ctg1_2901	B3	199.5539861	Bcer	V	Bcer	FALSE	Bc_Ga0417192_01_rn21	Ancillary biosynthesis	SMCOG1053
Bc_ctg1_2902	B3	0.45096946	Bcer	NA	Bcer	FALSE	Bc_Ga0417192_01_rn21	Other	NA
Bc_ctg1_2903	B3	0	Bcer	NA	Bcer	FALSE	Bc_Ga0417192_01_rn21	Other	NA
Bc_ctg1_2904	B3	0.45096946	Bcer	M	Bcer	FALSE	Bc_Ga0417192_01_rn21	Core biosynthesis	SMCOG1123
Bc_ctg1_2905	B3	0.90193892	Bcer	M	Bcer	FALSE	Bc_Ga0417192_01_rn21	Ancillary biosynthesis	SMCOG1152
Bc_ctg1_2906	B3	1.352908381	Bcer	M	Bcer	FALSE	Bc_Ga0417192_01_rn21	Core biosynthesis	SMCOG1010
Bc_ctg1_2907	B3	8.906646839	Bcer	M	Bcer	FALSE	Bc_Ga0417192_01_rn21	Core biosynthesis	SMCOG1123
Bc_ctg1_2908	B3	0.112742365	Bcer	NA	Bcer	FALSE	Bc_Ga0417192_01_rn21	Other	NA
Bc_ctg1_2909	B3	45002.12969	Bcer	C	Bcer	FALSE	Bc_Ga0417192_01_rn21	Other	NA
Bc_ctg1_291	B3	9.470358664	Bcer	S	Bcer	FALSE	NA	NA	NA
Bc_ctg1_2910	B3	13047.56117	Bcer	C	Bcer	FALSE	Bc_Ga0417192_01_rn21	Ancillary biosynthesis	None
Bc_ctg1_2911	B3	185.1229634	Bcer	M	Bcer	FALSE	Bc_Ga0417192_01_rn21	Core biosynthesis	None
Bc_ctg1_2913	B3	48.93018643	Bcer	J	Bcer	FALSE	Bc_Ga0417192_01_rn21	Other	NA
Bc_ctg1_2914	B3	16.00941584	Bcer	Q	Bcer	TRUE	Bc_Ga0417192_01_rn21	Other	NA
Bc_ctg1_2915	B3	21.53379172	Bcer	S	Bcer	FALSE	Bc_Ga0417192_01_rn21	Ancillary biosynthesis	SMCOG1010
Bc_ctg1_2916	B3	79.59610973	Bcer	S	Bcer	FALSE	Bc_Ga0417192_01_rn21	Other	NA
Bc_ctg1_2917	B3	27.17090998	Bcer	S	Bcer	FALSE	Bc_Ga0417192_01_rn21	Other	NA
Bc_ctg1_2919	B3	0	Bcer	NA	Bcer	FALSE	Bc_Ga0417192_01_rn21	Other	NA
Bc_ctg1_292	B3	9.019389204	Bcer	K	Bcer	FALSE	NA	NA	NA
Bc_ctg1_2920	B3	6.313572443	Bcer	S	Bcer	FALSE	Bc_Ga0417192_01_rn21	Other	NA
Bc_ctg1_2921	B3	55.46924361	Bcer	S	Bcer	FALSE	Bc_Ga0417192_01_rn21	Other	NA
Bc_ctg1_2922	B3	13.30359908	Bcer	L	Bcer	FALSE	Bc_Ga0417192_01_rn21	Other	NA

Bc_ctg1_2924	B3	0	Bcer	S	Bcer	FALSE	Bc_Ga0417192_01_rn21	Other	NA
Bc_ctg1_2925	B3	0	Bcer	S	Bcer	FALSE	Bc_Ga0417192_01_rn21	Other	NA
Bc_ctg1_2926	B3	84.44403142	Bcer	J	Bcer	FALSE	Bc_Ga0417192_01_rn21	Other	NA
Bc_ctg1_2927	B3	33.37174006	Bcer	V	Bcer	FALSE	NA	NA	NA
Bc_ctg1_2928	B3	94.59084428	Bcer	S	Bcer	FALSE	NA	NA	NA
Bc_ctg1_2929	B3	118.3794833	Bcer	E	Bcer	FALSE	NA	NA	NA
Bc_ctg1_293	B3	0.112742365	Bcer	NA	Bcer	FALSE	NA	NA	NA
Bc_ctg1_2930	B3	330.1096449	Bcer	H	Bcer	FALSE	NA	NA	NA
Bc_ctg1_2931	B3	10.93600941	Bcer	S	Bcer	FALSE	NA	NA	NA
Bc_ctg1_2932	B3	4.509694602	Bcer	S	Bcer	FALSE	NA	NA	NA
Bc_ctg1_2933	B3	8.455677379	Bcer	S	Bcer	FALSE	NA	NA	NA
Bc_ctg1_2934	B3	4.396952237	Bcer	P	Bcer	FALSE	NA	NA	NA
Bc_ctg1_2935	B3	24.80332031	Bcer	O	Bcer	FALSE	NA	NA	NA
Bc_ctg1_2936	B3	496.291891	Bcer	P	Bcer	FALSE	NA	NA	NA
Bc_ctg1_2937	B3	16.68587003	Bcer	K	Bcer	FALSE	NA	NA	NA
Bc_ctg1_2938	B3	9.921328125	Bcer	S	Bcer	FALSE	NA	NA	NA
Bc_ctg1_294	B3	12.73988725	Bcer	K	Bcer	FALSE	NA	NA	NA
Bc_ctg1_2940	B3	0.45096946	Bcer	NA	Bcer	FALSE	NA	NA	NA
Bc_ctg1_2942	B3	231.7983025	Bcer	K	Bcer	FALSE	NA	NA	NA
Bc_ctg1_2943	B3	0.45096946	Bcer	F	Bcer	FALSE	NA	NA	NA
Bc_ctg1_2944	B3	47.80276278	Bcer	M	Bcer	FALSE	NA	NA	NA
Bc_ctg1_2945	B3	97.63488813	Bcer	O	Bcer	FALSE	NA	NA	NA
Bc_ctg1_2946	B3	19.39168679	Bcer	C	Bcer	FALSE	NA	NA	NA
Bc_ctg1_2947	B3	24.91606268	Bcer	S	Bcer	FALSE	Bc_Ga0417192_01_rn22	Other	NA
Bc_ctg1_2948	B3	142.2808647	Bcer	J	Bcer	FALSE	Bc_Ga0417192_01_rn22	Other	NA
Bc_ctg1_2949	B3	46.11162731	Bcer	T	Bcer	FALSE	Bc_Ga0417192_01_rn22	Ancillary biosynthesis	SMCOG1025
Bc_ctg1_295	B3	22.77395774	Bcer	K	Bcer	FALSE	NA	NA	NA
Bc_ctg1_2951	B3	13.19085671	Bcer	T	Bcer	FALSE	Bc_Ga0417192_01_rn22	Other	NA
Bc_ctg1_2952	B3	8.342935014	Bcer	S	Bcer	FALSE	Bc_Ga0417192_01_rn22	Other	NA
Bc_ctg1_2953	B3	364.7215509	Bcer	E	Bcer	FALSE	Bc_Ga0417192_01_rn22	Transport	SMCOG1202
Bc_ctg1_2954	B3	24.57783558	Bcer	NA	Bcer	FALSE	Bc_Ga0417192_01_rn22	Other	NA
Bc_ctg1_2955	B3	48.81744407	Bcer	H	Bcer	FALSE	Bc_Ga0417192_01_rn22	Ancillary biosynthesis	SMCOG1012
Bc_ctg1_2956	B3	80.61079101	Bcer	T	Bcer	FALSE	Bc_Ga0417192_01_rn22	Regulation	SMCOG1048
Bc_ctg1_2957	B3	38.89611594	Bcer	KT	Bcer	FALSE	Bc_Ga0417192_01_rn22	Regulation	SMCOG1016
Bc_ctg1_2958	B3	2051.911044	Bcer	V	Bcer	FALSE	Bc_Ga0417192_01_rn22	Transport	SMCOG1000
Bc_ctg1_2959	B3	2870.533357	Bcer	V	Bcer	FALSE	Bc_Ga0417192_01_rn22	Other	NA
Bc_ctg1_296	B3	27.17090998	Bcer	S	Bcer	FALSE	NA	NA	NA
Bc_ctg1_2960	B3	1908.840983	Bcer	V	Bcer	FALSE	Bc_Ga0417192_01_rn22	Other	NA
Bc_ctg1_2961	B3	7.328253728	Bcer	NA	Bcer	FALSE	Bc_Ga0417192_01_rn22	Other	NA
Bc_ctg1_2962	B3	412.8625408	Bcer	Q	Bcer	TRUE	Bc_Ga0417192_01_rn22	Ancillary biosynthesis	SMCOG1004
Bc_ctg1_2963	B3	2277.283032	Bcer	Q	Bcer	TRUE	Bc_Ga0417192_01_rn22	Core biosynthesis	SMCOG1002
Bc_ctg1_2964	B3	2671.655825	Bcer	Q	Bcer	TRUE	Bc_Ga0417192_01_rn22	Core biosynthesis	SMCOG1209
Bc_ctg1_2965	B3	289.2969087	Bcer	I	Bcer	FALSE	Bc_Ga0417192_01_rn22	Core biosynthesis	SMCOG1021
Bc_ctg1_2966	B3	1365.76101	Bcer	Q	Bcer	TRUE	Bc_Ga0417192_01_rn22	Core biosynthesis	SMCOG1022
Bc_ctg1_2967	B3	1068.572136	Bcer	Q	Bcer	TRUE	Bc_Ga0417192_01_rn22	Core biosynthesis	SMCOG1022
Bc_ctg1_2968	B3	237.9991326	Bcer	Q	Bcer	TRUE	Bc_Ga0417192_01_rn22	Ancillary biosynthesis	SMCOG1222
Bc_ctg1_2969	B3	1362.491482	Bcer	Q	Bcer	TRUE	Bc_Ga0417192_01_rn22	Core biosynthesis	SMCOG1022
Bc_ctg1_297	B3	0	Bcer	K	Bcer	FALSE	NA	NA	NA
Bc_ctg1_2970	B3	335.408536	Bcer	Q	Bcer	TRUE	Bc_Ga0417192_01_rn22	Ancillary biosynthesis	SMCOG1042
Bc_ctg1_2971	B3	8.117450284	Bcer	M	Bcer	FALSE	Bc_Ga0417192_01_rn22	Other	NA
Bc_ctg1_2972	B3	1.014681285	Bcer	K	Bcer	FALSE	Bc_Ga0417192_01_rn22	Other	NA
Bc_ctg1_2973	B3	43.40581054	Bcer	M	Bcer	FALSE	Bc_Ga0417192_01_rn22	Other	NA
Bc_ctg1_2974	B3	267.3121475	Bcer	S	Bcer	FALSE	Bc_Ga0417192_01_rn22	Other	NA
Bc_ctg1_2975	B3	42.27838689	Bcer	OU	Bcer	FALSE	Bc_Ga0417192_01_rn22	Other	NA
Bc_ctg1_2976	B3	96.62020685	Bcer	S	Bcer	FALSE	Bc_Ga0417192_01_rn22	Other	NA
Bc_ctg1_2977	B3	139.8005327	Bcer	NT	Bcer	FALSE	Bc_Ga0417192_01_rn22	Other	SMCOG1061
Bc_ctg1_2978	B3	18.82797496	Bcer	T	Bcer	FALSE	Bc_Ga0417192_01_rn22	Other	NA
Bc_ctg1_2979	B3	9.921328125	Bcer	KT	Bcer	FALSE	Bc_Ga0417192_01_rn22	Regulation	SMCOG1008
Bc_ctg1_298	B3	3.044043856	Bcer	C	Bcer	FALSE	NA	NA	NA
Bc_ctg1_2980	B3	17.47506658	Bcer	C	Bcer	FALSE	Bc_Ga0417192_01_rn22	Other	NA
Bc_ctg1_2981	B3	0	Bcer	I	Bcer	FALSE	Bc_Ga0417192_01_rn22	Other	NA
Bc_ctg1_2982	B3	1.691135476	Bcer	C	Bcer	FALSE	Bc_Ga0417192_01_rn22	Other	NA

Bc_ctg1_2983	B3	66.63073775	Bcer	S	Bcer	FALSE	NA	NA	NA
Bc_ctg1_2984	B3	0	Bcer	S	Bcer	FALSE	NA	NA	NA
Bc_ctg1_2985	B3	0	Bcer	K	Bcer	FALSE	NA	NA	NA
Bc_ctg1_2986	B3	0.45096946	Bcer	P	Bcer	FALSE	NA	NA	NA
Bc_ctg1_2987	B3	0.563711825	Bcer	G	Bcer	FALSE	NA	NA	NA
Bc_ctg1_2988	B3	1.127423651	Bcer	P	Bcer	FALSE	NA	NA	NA
Bc_ctg1_2989	B3	11.6124636	Bcer	G	Bcer	FALSE	NA	NA	NA
Bc_ctg1_299	B3	1.240166016	Bcer	S	Bcer	FALSE	NA	NA	NA
Bc_ctg1_2990	B3	4.284209872	Bcer	T	Bcer	FALSE	NA	NA	NA
Bc_ctg1_2991	B3	3.156786221	Bcer	T	Bcer	FALSE	NA	NA	NA
Bc_ctg1_2992	B3	13.98005327	Bcer	T	Bcer	FALSE	NA	NA	NA
Bc_ctg1_2993	B3	0.789196555	Bcer	NA	Bcer	FALSE	NA	NA	NA
Bc_ctg1_2994	B3	4.171467507	Bcer	M	Bcer	FALSE	NA	NA	NA
Bc_ctg1_2995	B3	33.25899769	Bcer	NT	Bcer	FALSE	NA	NA	NA
Bc_ctg1_2996	B3	11.83794833	Bcer	T	Bcer	FALSE	NA	NA	NA
Bc_ctg1_2997	B3	14.54376509	Bcer	T	Bcer	FALSE	NA	NA	NA
Bc_ctg1_2998	B3	0.789196555	Bcer	C	Bcer	FALSE	NA	NA	NA
Bc_ctg1_2999	B3	1.127423651	Bcer	C	Bcer	FALSE	NA	NA	NA
Bc_ctg1_3	B3	12.28891779	Bcer	L	Bcer	FALSE	NA	NA	NA
Bc_ctg1_30	B3	71.14043235	Bcer	I	Bcer	FALSE	NA	NA	NA
Bc_ctg1_300	B3	6.426314808	Bcer	O	Bcer	FALSE	NA	NA	NA
Bc_ctg1_3000	B3	0.22548473	Bcer	NA	Bcer	FALSE	NA	NA	NA
Bc_ctg1_3001	B3	0.112742365	Bcer	EG	Bcer	FALSE	NA	NA	NA
Bc_ctg1_3002	B3	8.230192649	Bcer	K	Bcer	FALSE	NA	NA	NA
Bc_ctg1_3003	B3	23.5631543	Bcer	K	Bcer	FALSE	NA	NA	NA
Bc_ctg1_3004	B3	20.9700799	Bcer	T	Bcer	FALSE	NA	NA	NA
Bc_ctg1_3005	B3	28.52381836	Bcer	KT	Bcer	FALSE	NA	NA	NA
Bc_ctg1_3006	B3	0	Bcer	NA	Bcer	FALSE	NA	NA	NA
Bc_ctg1_3007	B3	4.960664062	Bcer	M	Bcer	FALSE	NA	NA	NA
Bc_ctg1_3008	B3	28.97478782	Bcer	C	Bcer	FALSE	NA	NA	NA
Bc_ctg1_3009	B3	1243.999256	Bcer	C	Bcer	FALSE	NA	NA	NA
Bc_ctg1_301	B3	23.90138139	Bcer	E	Bcer	FALSE	Bc_Ga0417192_01_rn03	Other	NA
Bc_ctg1_3010	B3	232.0237873	Bcer	S	Bcer	FALSE	NA	NA	NA
Bc_ctg1_3011	B3	73.62076438	Bcer	C	Bcer	FALSE	NA	NA	NA
Bc_ctg1_3012	B3	2213.019884	Bcer	E	Bcer	FALSE	NA	NA	NA
Bc_ctg1_3013	B3	819.8624787	Bcer	E	Bcer	FALSE	NA	NA	NA
Bc_ctg1_3014	B3	90.87034623	Bcer	K	Bcer	FALSE	NA	NA	NA
Bc_ctg1_3015	B3	1458.20975	Bcer	P	Bcer	FALSE	NA	NA	NA
Bc_ctg1_3016	B3	99.88973544	Bcer	H	Bcer	FALSE	NA	NA	NA
Bc_ctg1_3017	B3	386.7063121	Bcer	K	Bcer	FALSE	NA	NA	NA
Bc_ctg1_3019	B3	31.45511985	Bcer	E	Bcer	FALSE	NA	NA	NA
Bc_ctg1_302	B3	41.03822088	Bcer	S	Bcer	FALSE	Bc_Ga0417192_01_rn03	Other	NA
Bc_ctg1_3020	B3	9.132131569	Bcer	S	Bcer	FALSE	NA	NA	NA
Bc_ctg1_3021	B3	14.65650746	Bcer	NA	Bcer	FALSE	NA	NA	NA
Bc_ctg1_3022	B3	5.298891157	Bcer	NA	Bcer	FALSE	NA	NA	NA
Bc_ctg1_3023	B3	17.24958185	Bcer	V	Bcer	FALSE	NA	NA	NA
Bc_ctg1_3024	B3	23.45041193	Bcer	K	Bcer	FALSE	NA	NA	NA
Bc_ctg1_3025	B3	0.45096946	Bcer	F	Bcer	FALSE	NA	NA	NA
Bc_ctg1_3026	B3	2.593074396	Bcer	E	Bcer	FALSE	NA	NA	NA
Bc_ctg1_3027	B3	15.89667347	Bcer	S	Bcer	FALSE	NA	NA	NA
Bc_ctg1_3028	B3	275.4295978	Bcer	E	Bcer	FALSE	NA	NA	NA
Bc_ctg1_3029	B3	2780.903176	Bcer	C	Bcer	FALSE	NA	NA	NA
Bc_ctg1_303	B3	39.23434304	Bcer	S	Bcer	FALSE	Bc_Ga0417192_01_rn03	Ancillary biosynthesis	None
Bc_ctg1_3030	B3	253.3320943	Bcer	K	Bcer	FALSE	Bc_Ga0417192_01_rn23	Regulation	SMCOG1058
Bc_ctg1_3031	B3	0	Bcer	S	Bcer	FALSE	Bc_Ga0417192_01_rn23	Other	NA
Bc_ctg1_3032	B3	5011.736354	Bcer	E	Bcer	FALSE	Bc_Ga0417192_01_rn23	Transport	SMCOG1202
Bc_ctg1_3033	B3	228.8670011	Bcer	P	Bcer	FALSE	Bc_Ga0417192_01_rn23	Transport	SMCOG1033
Bc_ctg1_3034	B3	91.88502752	Bcer	P	Bcer	FALSE	Bc_Ga0417192_01_rn23	Transport	SMCOG1011
Bc_ctg1_3035	B3	69.22381214	Bcer	P	Bcer	FALSE	Bc_Ga0417192_01_rn23	Transport	SMCOG1011
Bc_ctg1_3036	B3	117.8157715	Bcer	HP	Bcer	FALSE	Bc_Ga0417192_01_rn23	Transport	SMCOG1000
Bc_ctg1_3037	B3	33.59722479	Bcer	Q	Bcer	TRUE	Bc_Ga0417192_01_rn23	Ancillary biosynthesis	SMCOG1248
Bc_ctg1_3038	B3	2251.803257	Bcer	H	Bcer	FALSE	Bc_Ga0417192_01_rn23	Ancillary biosynthesis	SMCOG1109

Bc_ctg1_3039	B3	2114.934026	Bcer	GM	Bcer	FALSE	Bc_Ga0417192_01_rn23	Core biosynthesis	SMCOG1010
Bc_ctg1_304	B3	4.735179332	Bcer	S	Bcer	FALSE	Bc_Ga0417192_01_rn03	Other	NA
Bc_ctg1_3040	B3	8.117450284	Bcer	F	Bcer	FALSE	Bc_Ga0417192_01_rn23	Other	NA
Bc_ctg1_3041	B3	2.254847301	Bcer	F	Bcer	FALSE	Bc_Ga0417192_01_rn23	Other	NA
Bc_ctg1_3042	B3	87.48807528	Bcer	P	Bcer	FALSE	Bc_Ga0417192_01_rn23	Other	NA
Bc_ctg1_3043	B3	26.60719815	Bcer	S	Bcer	FALSE	Bc_Ga0417192_01_rn23	Other	NA
Bc_ctg1_3044	B3	42.39112926	Bcer	I	Bcer	FALSE	Bc_Ga0417192_01_rn23	Other	NA
Bc_ctg1_3046	B3	51.07229137	Bcer	T	Bcer	FALSE	Bc_Ga0417192_01_rn23	Ancillary biosynthesis	SMCOG1025
Bc_ctg1_3047	B3	26.94542525	Bcer	E	Bcer	FALSE	Bc_Ga0417192_01_rn23	Ancillary biosynthesis	SMCOG1038
Bc_ctg1_3048	B3	28.86204545	Bcer	K	Bcer	FALSE	Bc_Ga0417192_01_rn23	Regulation	SMCOG1136
Bc_ctg1_3049	B3	4533.032272	Bcer	G	Bcer	FALSE	Bc_Ga0417192_01_rn23	Other	NA
Bc_ctg1_3050	B3	5417.045156	Bcer	G	Bcer	FALSE	Bc_Ga0417192_01_rn23	Core biosynthesis	SMCOG1257
Bc_ctg1_3051	B3	0.22548473	Bcer	S	Bcer	FALSE	Bc_Ga0417192_01_rn23	Other	NA
Bc_ctg1_3052	B3	0.22548473	Bcer	E	Bcer	FALSE	Bc_Ga0417192_01_rn23	Other	NA
Bc_ctg1_3053	B3	0	Bcer	EG	Bcer	FALSE	Bc_Ga0417192_01_rn23	Other	NA
Bc_ctg1_3054	B3	76.21383878	Bcer	S	Bcer	FALSE	Bc_Ga0417192_01_rn23	Ancillary biosynthesis	None
Bc_ctg1_3055	B3	4.735179332	Bcer	E	Bcer	FALSE	Bc_Ga0417192_01_rn23	Ancillary biosynthesis	SMCOG1153
Bc_ctg1_3056	B3	0	Bcer	E	Bcer	FALSE	Bc_Ga0417192_01_rn23	Transport	SMCOG1000
Bc_ctg1_3057	B3	0.45096946	Bcer	ET	Bcer	FALSE	Bc_Ga0417192_01_rn23	Ancillary biosynthesis	SMCOG1091
Bc_ctg1_3058	B3	0	Bcer	P	Bcer	FALSE	Bc_Ga0417192_01_rn23	Transport	SMCOG1096
Bc_ctg1_3059	B3	0.563711825	Bcer	P	Bcer	FALSE	Bc_Ga0417192_01_rn23	Transport	SMCOG1096
Bc_ctg1_306	B3	7.779223189	Bcer	S	Bcer	FALSE	Bc_Ga0417192_01_rn03	Other	NA
Bc_ctg1_3060	B3	177.0055131	Bcer	E	Bcer	FALSE	NA	NA	NA
Bc_ctg1_3061	B3	73.50802201	Bcer	O	Bcer	FALSE	NA	NA	NA
Bc_ctg1_3062	B3	8.681162109	Bcer	FG	Bcer	FALSE	NA	NA	NA
Bc_ctg1_3063	B3	1.916620206	Bcer	K	Bcer	FALSE	NA	NA	NA
Bc_ctg1_3064	B3	3.945982777	Bcer	NA	Bcer	FALSE	NA	NA	NA
Bc_ctg1_3065	B3	2.480332031	Bcer	K	Bcer	FALSE	NA	NA	NA
Bc_ctg1_3066	B3	0.67645419	Bcer	H	Bcer	FALSE	NA	NA	NA
Bc_ctg1_3067	B3	59.07699929	Bcer	T	Bcer	FALSE	NA	NA	NA
Bc_ctg1_3068	B3	28.86204545	Bcer	KT	Bcer	FALSE	NA	NA	NA
Bc_ctg1_3069	B3	1072.405376	Bcer	NA	Bcer	FALSE	NA	NA	NA
Bc_ctg1_3070	B3	11.38697887	Bcer	P	Bcer	FALSE	NA	NA	NA
Bc_ctg1_3071	B3	9.80858576	Bcer	T	Bcer	FALSE	NA	NA	NA
Bc_ctg1_3073	B3	166.1822461	Bcer	E	Bcer	FALSE	NA	NA	NA
Bc_ctg1_3074	B3	47.12630859	Bcer	EP	Bcer	FALSE	NA	NA	NA
Bc_ctg1_3075	B3	37.54320756	Bcer	P	Bcer	FALSE	NA	NA	NA
Bc_ctg1_3076	B3	23.5631543	Bcer	P	Bcer	FALSE	NA	NA	NA
Bc_ctg1_3077	B3	32.9207706	Bcer	P	Bcer	FALSE	NA	NA	NA
Bc_ctg1_3078	B3	35.40110263	Bcer	G	Bcer	FALSE	NA	NA	NA
Bc_ctg1_3079	B3	38.55788885	Bcer	K	Bcer	FALSE	NA	NA	NA
Bc_ctg1_308	B3	31.22963512	Bcer	IQ	Bcer	TRUE	Bc_Ga0417192_01_rn03	Ancillary biosynthesis	SMCOG1002
Bc_ctg1_3080	B3	370.3586692	Bcer	P	Bcer	FALSE	NA	NA	NA
Bc_ctg1_3081	B3	64.03766335	Bcer	K	Bcer	FALSE	NA	NA	NA
Bc_ctg1_3082	B3	73.28253728	Bcer	H	Bcer	FALSE	NA	NA	NA
Bc_ctg1_3083	B3	15.33296165	Bcer	G	Bcer	FALSE	NA	NA	NA
Bc_ctg1_3084	B3	130.1046893	Bcer	G	Bcer	FALSE	NA	NA	NA
Bc_ctg1_3085	B3	248.8223997	Bcer	G	Bcer	FALSE	NA	NA	NA
Bc_ctg1_3086	B3	545.109335	Bcer	G	Bcer	FALSE	NA	NA	NA
Bc_ctg1_3087	B3	626.7348073	Bcer	G	Bcer	FALSE	NA	NA	NA
Bc_ctg1_3088	B3	980.5203489	Bcer	V	Bcer	FALSE	NA	NA	NA
Bc_ctg1_3089	B3	0	Bcer	NA	Bcer	FALSE	NA	NA	NA
Bc_ctg1_309	B3	7.891965554	Bcer	S	Bcer	FALSE	Bc_Ga0417192_01_rn03	Other	NA
Bc_ctg1_3090	B3	0.22548473	Bcer	NA	Bcer	FALSE	NA	NA	NA
Bc_ctg1_3091	B3	45.43517312	Bcer	EGP	Bcer	FALSE	NA	NA	NA
Bc_ctg1_3092	B3	3609.446817	Bcer	E	Bcer	FALSE	NA	NA	NA
Bc_ctg1_3093	B3	1.803877841	Bcer	P	Bcer	FALSE	NA	NA	NA
Bc_ctg1_3094	B3	774.2018208	Bcer	M	Bcer	FALSE	NA	NA	NA
Bc_ctg1_3095	B3	157.501084	Bcer	S	Bcer	FALSE	NA	NA	NA
Bc_ctg1_3096	B3	2.931301491	Bcer	E	Bcer	FALSE	NA	NA	NA
Bc_ctg1_3097	B3	11.04875178	Bcer	C	Bcer	FALSE	NA	NA	NA
Bc_ctg1_3098	B3	5.411633522	Bcer	V	Bcer	FALSE	NA	NA	NA

Bc_ctg1_3099	B3	4.960664062	Bcer	NA	Bcer	FALSE	NA	NA	NA
Bc_ctg1_31	B3	29.42575728	Bcer	S	Bcer	FALSE	NA	NA	NA
Bc_ctg1_310	B3	4.396952237	Bcer	K	Bcer	FALSE	Bc_Ga0417192_01_rn03	Regulation	SMCOG1167
Bc_ctg1_3100	B3	1.014681285	Bcer	NA	Bcer	FALSE	NA	NA	NA
Bc_ctg1_3101	B3	12.17617543	Bcer	V	Bcer	FALSE	NA	NA	NA
Bc_ctg1_3102	B3	120.6343306	Bcer	NT	Bcer	FALSE	NA	NA	NA
Bc_ctg1_3103	B3	394.0345659	Bcer	S	Bcer	FALSE	NA	NA	NA
Bc_ctg1_3104	B3	0.67645419	Bcer	K	Bcer	FALSE	NA	NA	NA
Bc_ctg1_3105	B3	2.367589666	Bcer	NA	Bcer	FALSE	NA	NA	NA
Bc_ctg1_3106	B3	9.583101029	Bcer	M	Bcer	FALSE	NA	NA	NA
Bc_ctg1_3107	B3	1.803877841	Bcer	L	Bcer	FALSE	NA	NA	NA
Bc_ctg1_3108	B3	180.3877841	Bcer	E	Bcer	FALSE	NA	NA	NA
Bc_ctg1_3109	B3	1.465650746	Bcer	E	Bcer	FALSE	NA	NA	NA
Bc_ctg1_311	B3	0	Bcer	NA	Bcer	FALSE	Bc_Ga0417192_01_rn03	Other	NA
Bc_ctg1_3110	B3	71.59140181	Bcer	S	Bcer	FALSE	NA	NA	NA
Bc_ctg1_3111	B3	164.603853	Bcer	G	Bcer	FALSE	NA	NA	NA
Bc_ctg1_3112	B3	41.48919034	Bcer	E	Bcer	FALSE	NA	NA	NA
Bc_ctg1_3113	B3	44.75871893	Bcer	S	Bcer	FALSE	NA	NA	NA
Bc_ctg1_3114	B3	0.112742365	Bcer	K	Bcer	FALSE	NA	NA	NA
Bc_ctg1_3115	B3	1.240166016	Bcer	NA	Bcer	FALSE	NA	NA	NA
Bc_ctg1_3116	B3	19.27894442	Bcer	K	Bcer	FALSE	NA	NA	NA
Bc_ctg1_3117	B3	3.720498047	Bcer	S	Bcer	FALSE	NA	NA	NA
Bc_ctg1_3118	B3	15.55844638	Bcer	P	Bcer	FALSE	NA	NA	NA
Bc_ctg1_3119	B3	70.57672052	Bcer	C	Bcer	FALSE	NA	NA	NA
Bc_ctg1_312	B3	1.240166016	Bcer	K	Bcer	FALSE	Bc_Ga0417192_01_rn03	Regulation	SMCOG1058
Bc_ctg1_3120	B3	554.1287242	Bcer	C	Bcer	FALSE	NA	NA	NA
Bc_ctg1_3121	B3	2390.025397	Bcer	C	Bcer	FALSE	NA	NA	NA
Bc_ctg1_3122	B3	1137.570463	Bcer	C	Bcer	FALSE	NA	NA	NA
Bc_ctg1_3123	B3	68.66010032	Bcer	E	Bcer	FALSE	NA	NA	NA
Bc_ctg1_3124	B3	17.36232422	Bcer	F	Bcer	FALSE	NA	NA	NA
Bc_ctg1_3125	B3	1.465650746	Bcer	E	Bcer	FALSE	NA	NA	NA
Bc_ctg1_3126	B3	0	Bcer	S	Bcer	FALSE	NA	NA	NA
Bc_ctg1_3127	B3	9.244873934	Bcer	E	Bcer	FALSE	NA	NA	NA
Bc_ctg1_3128	B3	0	Bcer	EG	Bcer	FALSE	NA	NA	NA
Bc_ctg1_3129	B3	0	Bcer	E	Bcer	FALSE	NA	NA	NA
Bc_ctg1_3130	B3	0.112742365	Bcer	S	Bcer	FALSE	NA	NA	NA
Bc_ctg1_3131	B3	2529.487702	Bcer	P	Bcer	FALSE	NA	NA	NA
Bc_ctg1_3132	B3	1448.964876	Bcer	P	Bcer	FALSE	NA	NA	NA
Bc_ctg1_3133	B3	67.30719194	Bcer	P	Bcer	FALSE	NA	NA	NA
Bc_ctg1_3134	B3	1.916620206	Bcer	P	Bcer	FALSE	NA	NA	NA
Bc_ctg1_3135	B3	3.044043856	Bcer	P	Bcer	FALSE	NA	NA	NA
Bc_ctg1_3136	B3	3.495013317	Bcer	P	Bcer	FALSE	NA	NA	NA
Bc_ctg1_3137	B3	0.789196555	Bcer	S	Bcer	FALSE	NA	NA	NA
Bc_ctg1_3138	B3	14.88199219	Bcer	V	Bcer	FALSE	NA	NA	NA
Bc_ctg1_314	B3	0	Bcer	L	Bcer	FALSE	Bc_Ga0417192_01_rn03	Other	SMCOG1026
Bc_ctg1_3140	B3	11.04875178	Bcer	T	Bcer	FALSE	NA	NA	NA
Bc_ctg1_3141	B3	95.38004083	Bcer	S	Bcer	FALSE	NA	NA	NA
Bc_ctg1_3142	B3	64.93960227	Bcer	NA	Bcer	FALSE	NA	NA	NA
Bc_ctg1_3143	B3	463.0328933	Bcer	MT	Bcer	FALSE	NA	NA	NA
Bc_ctg1_3144	B3	1.578393111	Bcer	K	Bcer	FALSE	NA	NA	NA
Bc_ctg1_3145	B3	2.254847301	Bcer	P	Bcer	FALSE	NA	NA	NA
Bc_ctg1_3146	B3	1.578393111	Bcer	P	Bcer	FALSE	NA	NA	NA
Bc_ctg1_3147	B3	1.352908381	Bcer	P	Bcer	FALSE	NA	NA	NA
Bc_ctg1_3148	B3	1.803877841	Bcer	H	Bcer	FALSE	NA	NA	NA
Bc_ctg1_3149	B3	2.367589666	Bcer	E	Bcer	FALSE	NA	NA	NA
Bc_ctg1_315	B3	2.705816761	Bcer	M	Bcer	FALSE	Bc_Ga0417192_01_rn03	Core biosynthesis	SMCOG1123
Bc_ctg1_3150	B3	1.014681285	Bcer	H	Bcer	FALSE	NA	NA	NA
Bc_ctg1_3151	B3	3.720498047	Bcer	H	Bcer	FALSE	NA	NA	NA
Bc_ctg1_3152	B3	5.073406427	Bcer	H	Bcer	FALSE	NA	NA	NA
Bc_ctg1_3153	B3	1.465650746	Bcer	H	Bcer	FALSE	NA	NA	NA
Bc_ctg1_3154	B3	5.411633522	Bcer	NA	Bcer	FALSE	NA	NA	NA
Bc_ctg1_3155	B3	2.931301491	Bcer	NA	Bcer	FALSE	NA	NA	NA

Bc_ctg1_3157	B3	302.6005078	Bcer	P	Bcer	FALSE	NA	NA	NA
Bc_ctg1_3158	B3	352.996345	Bcer	P	Bcer	FALSE	NA	NA	NA
Bc_ctg1_3159	B3	74.5227033	Bcer	P	Bcer	FALSE	NA	NA	NA
Bc_ctg1_316	B3	0.45096946	Bcer	S	Bcer	FALSE	Bc_Ga0417192_01_rn03	Other	NA
Bc_ctg1_3160	B3	5.637118253	Bcer	T	Bcer	FALSE	NA	NA	NA
Bc_ctg1_3161	B3	0.789196555	Bcer	EGP	Bcer	FALSE	NA	NA	NA
Bc_ctg1_3162	B3	2.367589666	Bcer	K	Bcer	FALSE	NA	NA	NA
Bc_ctg1_3163	B3	32.2443164	Bcer	S	Bcer	FALSE	NA	NA	NA
Bc_ctg1_3164	B3	1.465650746	Bcer	NA	Bcer	FALSE	NA	NA	NA
Bc_ctg1_3165	B3	0.45096946	Bcer	V	Bcer	FALSE	NA	NA	NA
Bc_ctg1_3166	B3	0.563711825	Bcer	V	Bcer	FALSE	NA	NA	NA
Bc_ctg1_3167	B3	0.45096946	Bcer	V	Bcer	FALSE	NA	NA	NA
Bc_ctg1_3168	B3	0.338227095	Bcer	NA	Bcer	FALSE	NA	NA	NA
Bc_ctg1_3169	B3	13.8673109	Bcer	S	Bcer	FALSE	NA	NA	NA
Bc_ctg1_317	B3	0.112742365	Bcer	K	Bcer	FALSE	Bc_Ga0417192_01_rn03	Other	NA
Bc_ctg1_3170	B3	0.789196555	Bcer	IQ	Bcer	TRUE	NA	NA	NA
Bc_ctg1_3171	B3	1.578393111	Bcer	S	Bcer	FALSE	NA	NA	NA
Bc_ctg1_3172	B3	0.338227095	Bcer	NA	Bcer	FALSE	NA	NA	NA
Bc_ctg1_3173	B3	9.132131569	Bcer	S	Bcer	FALSE	NA	NA	NA
Bc_ctg1_3174	B3	29.31301491	Bcer	CO	Bcer	FALSE	NA	NA	NA
Bc_ctg1_3175	B3	26.15622869	Bcer	C	Bcer	FALSE	NA	NA	NA
Bc_ctg1_3176	B3	2.480332031	Bcer	S	Bcer	FALSE	NA	NA	NA
Bc_ctg1_3177	B3	34.8373908	Bcer	S	Bcer	FALSE	NA	NA	NA
Bc_ctg1_3178	B3	1.803877841	Bcer	E	Bcer	FALSE	NA	NA	NA
Bc_ctg1_3179	B3	0.45096946	Bcer	S	Bcer	FALSE	NA	NA	NA
Bc_ctg1_318	B3	20.85733753	Bcer	M	Bcer	FALSE	Bc_Ga0417192_01_rn03	Core biosynthesis	None
Bc_ctg1_3180	B3	0.45096946	Bcer	P	Bcer	FALSE	NA	NA	NA
Bc_ctg1_3181	B3	0.112742365	Bcer	NA	Bcer	FALSE	NA	NA	NA
Bc_ctg1_3182	B3	21.75927646	Bcer	C	Bcer	FALSE	NA	NA	NA
Bc_ctg1_3183	B3	0.45096946	Bcer	S	Bcer	FALSE	NA	NA	NA
Bc_ctg1_3184	B3	12.40166016	Bcer	S	Bcer	FALSE	NA	NA	NA
Bc_ctg1_3185	B3	7.779223189	Bcer	M	Bcer	FALSE	NA	NA	NA
Bc_ctg1_3186	B3	1.465650746	Bcer	NA	Bcer	FALSE	NA	NA	NA
Bc_ctg1_3187	B3	4.735179332	Bcer	P	Bcer	FALSE	NA	NA	NA
Bc_ctg1_3188	B3	22.21024592	Bcer	S	Bcer	FALSE	NA	NA	NA
Bc_ctg1_3189	B3	0.112742365	Bcer	OP	Bcer	FALSE	NA	NA	NA
Bc_ctg1_319	B3	7.102768998	Bcer	E	Bcer	FALSE	Bc_Ga0417192_01_rn03	Transport	SMCOG1116
Bc_ctg1_3190	B3	2.367589666	Bcer	P	Bcer	FALSE	NA	NA	NA
Bc_ctg1_3191	B3	0	Bcer	O	Bcer	FALSE	NA	NA	NA
Bc_ctg1_3192	B3	0	Bcer	P	Bcer	FALSE	NA	NA	NA
Bc_ctg1_3193	B3	0	Bcer	P	Bcer	FALSE	NA	NA	NA
Bc_ctg1_3194	B3	0	Bcer	O	Bcer	FALSE	NA	NA	NA
Bc_ctg1_3195	B3	17.92603604	Bcer	S	Bcer	FALSE	NA	NA	NA
Bc_ctg1_3196	B3	144.535712	Bcer	NA	Bcer	FALSE	NA	NA	NA
Bc_ctg1_3197	B3	493.8115589	Bcer	K	Bcer	FALSE	NA	NA	NA
Bc_ctg1_3198	B3	56.82215199	Bcer	C	Bcer	FALSE	NA	NA	NA
Bc_ctg1_32	B3	31.00415039	Bcer	J	Bcer	FALSE	NA	NA	NA
Bc_ctg1_320	B3	33.59722479	Bcer	S	Bcer	FALSE	Bc_Ga0417192_01_rn03	Other	NA
Bc_ctg1_3201	B3	6.877284268	Bcer	P	Bcer	FALSE	NA	NA	NA
Bc_ctg1_3202	B3	21.30830699	Bcer	EGP	Bcer	FALSE	NA	NA	NA
Bc_ctg1_3203	B3	125.3695099	Bcer	S	Bcer	FALSE	NA	NA	NA
Bc_ctg1_3204	B3	20.29362571	Bcer	K	Bcer	FALSE	NA	NA	NA
Bc_ctg1_3205	B3	3.269528587	Bcer	G	Bcer	FALSE	NA	NA	NA
Bc_ctg1_3206	B3	14.65650746	Bcer	G	Bcer	FALSE	NA	NA	NA
Bc_ctg1_3207	B3	43.18032581	Bcer	G	Bcer	FALSE	NA	NA	NA
Bc_ctg1_3208	B3	6.200830078	Bcer	NA	Bcer	FALSE	NA	NA	NA
Bc_ctg1_3209	B3	27.05816761	Bcer	S	Bcer	FALSE	NA	NA	NA
Bc_ctg1_321	B3	0.563711825	Bcer	NA	Bcer	FALSE	Bc_Ga0417192_01_rn03	Other	NA
Bc_ctg1_3210	B3	1853.484481	Bcer	M	Bcer	FALSE	NA	NA	NA
Bc_ctg1_3211	B3	91.09583096	Bcer	V	Bcer	FALSE	NA	NA	NA
Bc_ctg1_3212	B3	46.33711204	Bcer	V	Bcer	FALSE	NA	NA	NA
Bc_ctg1_3213	B3	52.76342684	Bcer	M	Bcer	FALSE	NA	NA	NA

Bc_ctg1_3214	B3	3.156786221	Bcer	S	Bcer	FALSE	NA	NA	NA
Bc_ctg1_3215	B3	0.67645419	Bcer	EP	Bcer	FALSE	NA	NA	NA
Bc_ctg1_3216	B3	1.916620206	Bcer	EP	Bcer	FALSE	NA	NA	NA
Bc_ctg1_3217	B3	22.09750355	Bcer	E	Bcer	FALSE	NA	NA	NA
Bc_ctg1_3218	B3	0.112742365	Bcer	P	Bcer	FALSE	NA	NA	NA
Bc_ctg1_322	B3	0.563711825	Bcer	NA	Bcer	FALSE	Bc_Ga0417192_01_rn03	Other	NA
Bc_ctg1_3220	B3	0	Bcer	S	Bcer	FALSE	NA	NA	NA
Bc_ctg1_3221	B3	0	Bcer	E	Bcer	FALSE	NA	NA	NA
Bc_ctg1_3222	B3	0.789196555	Bcer	S	Bcer	FALSE	NA	NA	NA
Bc_ctg1_3223	B3	0.563711825	Bcer	L	Bcer	FALSE	NA	NA	NA
Bc_ctg1_3224	B3	1.240166016	Bcer	S	Bcer	FALSE	NA	NA	NA
Bc_ctg1_3225	B3	0.90193892	Bcer	S	Bcer	FALSE	NA	NA	NA
Bc_ctg1_3226	B3	2.367589666	Bcer	S	Bcer	FALSE	NA	NA	NA
Bc_ctg1_3227	B3	0.45096946	Bcer	S	Bcer	FALSE	NA	NA	NA
Bc_ctg1_3228	B3	0.112742365	Bcer	NA	Bcer	FALSE	NA	NA	NA
Bc_ctg1_3229	B3	7.440996093	Bcer	S	Bcer	FALSE	NA	NA	NA
Bc_ctg1_323	B3	6.426314808	Bcer	K	Bcer	FALSE	Bc_Ga0417192_01_rn03	Other	NA
Bc_ctg1_3230	B3	6.426314808	Bcer	NA	Bcer	FALSE	NA	NA	NA
Bc_ctg1_3231	B3	7.215511363	Bcer	NA	Bcer	FALSE	NA	NA	NA
Bc_ctg1_3232	B3	0.789196555	Bcer	E	Bcer	FALSE	NA	NA	NA
Bc_ctg1_3233	B3	1.127423651	Bcer	P	Bcer	FALSE	NA	NA	NA
Bc_ctg1_3234	B3	6.088087713	Bcer	NA	Bcer	FALSE	NA	NA	NA
Bc_ctg1_3235	B3	37.54320756	Bcer	K	Bcer	FALSE	NA	NA	NA
Bc_ctg1_3236	B3	35.73932972	Bcer	G	Bcer	FALSE	NA	NA	NA
Bc_ctg1_3237	B3	447.4744469	Bcer	G	Bcer	FALSE	NA	NA	NA
Bc_ctg1_3238	B3	335.5212784	Bcer	S	Bcer	FALSE	NA	NA	NA
Bc_ctg1_3239	B3	0.22548473	Bcer	S	Bcer	FALSE	NA	NA	NA
Bc_ctg1_324	B3	1.352908381	Bcer	K	Bcer	FALSE	Bc_Ga0417192_01_rn03	Other	NA
Bc_ctg1_3241	B3	0.112742365	Bcer	NA	Bcer	FALSE	NA	NA	NA
Bc_ctg1_3242	B3	23.90138139	Bcer	EG	Bcer	FALSE	NA	NA	NA
Bc_ctg1_3243	B3	0.90193892	Bcer	K	Bcer	FALSE	NA	NA	NA
Bc_ctg1_3244	B3	0.112742365	Bcer	E	Bcer	FALSE	NA	NA	NA
Bc_ctg1_3245	B3	3.382270952	Bcer	S	Bcer	FALSE	NA	NA	NA
Bc_ctg1_3246	B3	0.112742365	Bcer	K	Bcer	FALSE	NA	NA	NA
Bc_ctg1_3247	B3	8.793904474	Bcer	P	Bcer	FALSE	NA	NA	NA
Bc_ctg1_3248	B3	3.269528587	Bcer	P	Bcer	FALSE	NA	NA	NA
Bc_ctg1_3249	B3	44.75871893	Bcer	K	Bcer	FALSE	NA	NA	NA
Bc_ctg1_325	B3	4.735179332	Bcer	V	Bcer	FALSE	Bc_Ga0417192_01_rn03	Transport	SMCOG1065
Bc_ctg1_3250	B3	45.32243075	Bcer	EGP	Bcer	FALSE	NA	NA	NA
Bc_ctg1_3251	B3	0.563711825	Bcer	NA	Bcer	FALSE	NA	NA	NA
Bc_ctg1_3252	B3	8.230192649	Bcer	T	Bcer	FALSE	NA	NA	NA
Bc_ctg1_3253	B3	18.60249023	Bcer	T	Bcer	FALSE	NA	NA	NA
Bc_ctg1_3254	B3	0.338227095	Bcer	M	Bcer	FALSE	NA	NA	NA
Bc_ctg1_3255	B3	1.240166016	Bcer	V	Bcer	FALSE	NA	NA	NA
Bc_ctg1_3256	B3	1.578393111	Bcer	V	Bcer	FALSE	NA	NA	NA
Bc_ctg1_3257	B3	0.789196555	Bcer	V	Bcer	FALSE	NA	NA	NA
Bc_ctg1_3258	B3	0.67645419	Bcer	V	Bcer	FALSE	NA	NA	NA
Bc_ctg1_3259	B3	5.073406427	Bcer	S	Bcer	FALSE	NA	NA	NA
Bc_ctg1_326	B3	2.705816761	Bcer	V	Bcer	FALSE	Bc_Ga0417192_01_rn03	Transport	SMCOG1000
Bc_ctg1_3260	B3	2.931301491	Bcer	K	Bcer	FALSE	NA	NA	NA
Bc_ctg1_3261	B3	0.338227095	Bcer	S	Bcer	FALSE	NA	NA	NA
Bc_ctg1_3262	B3	0.338227095	Bcer	S	Bcer	FALSE	NA	NA	NA
Bc_ctg1_3263	B3	3.156786221	Bcer	S	Bcer	FALSE	NA	NA	NA
Bc_ctg1_3264	B3	13.30359908	Bcer	S	Bcer	FALSE	NA	NA	NA
Bc_ctg1_3265	B3	40.58725142	Bcer	E	Bcer	FALSE	NA	NA	NA
Bc_ctg1_3266	B3	0	Bcer	NA	Bcer	FALSE	NA	NA	NA
Bc_ctg1_3267	B3	6.313572443	Bcer	S	Bcer	FALSE	NA	NA	NA
Bc_ctg1_3268	B3	46.33711204	Bcer	P	Bcer	FALSE	NA	NA	NA
Bc_ctg1_3269	B3	47.69002042	Bcer	U	Bcer	FALSE	NA	NA	NA
Bc_ctg1_327	B3	7.102768998	Bcer	S	Bcer	FALSE	Bc_Ga0417192_01_rn03	Other	NA
Bc_ctg1_3270	B3	32.2443164	Bcer	E	Bcer	FALSE	NA	NA	NA
Bc_ctg1_3271	B3	9.583101029	Bcer	S	Bcer	FALSE	NA	NA	NA

Bc_ctg1_3272	B3	348.9376198	Bcer	C	Bcer	FALSE	NA	NA	NA
Bc_ctg1_3273	B3	22.54847301	Bcer	V	Bcer	FALSE	NA	NA	NA
Bc_ctg1_3274	B3	24.91606268	Bcer	V	Bcer	FALSE	NA	NA	NA
Bc_ctg1_3275	B3	179.2603604	Bcer	ET	Bcer	FALSE	NA	NA	NA
Bc_ctg1_3276	B3	103.4974911	Bcer	P	Bcer	FALSE	NA	NA	NA
Bc_ctg1_3277	B3	168.8880628	Bcer	E	Bcer	FALSE	NA	NA	NA
Bc_ctg1_3278	B3	0.22548473	Bcer	S	Bcer	FALSE	NA	NA	NA
Bc_ctg1_3279	B3	0	Bcer	NA	Bcer	FALSE	NA	NA	NA
Bc_ctg1_328	B3	25.70525923	Bcer	K	Bcer	FALSE	Bc_Ga0417192_01_rn03	Other	NA
Bc_ctg1_3280	B3	0.22548473	Bcer	HJ	Bcer	FALSE	NA	NA	NA
Bc_ctg1_3281	B3	0.67645419	Bcer	HJ	Bcer	FALSE	NA	NA	NA
Bc_ctg1_3282	B3	8.568419744	Bcer	S	Bcer	FALSE	NA	NA	NA
Bc_ctg1_3283	B3	194.5933221	Bcer	S	Bcer	FALSE	NA	NA	NA
Bc_ctg1_3284	B3	5.524375888	Bcer	NA	Bcer	FALSE	NA	NA	NA
Bc_ctg1_3285	B3	4.847921697	Bcer	S	Bcer	FALSE	NA	NA	NA
Bc_ctg1_3286	B3	91.88502752	Bcer	L	Bcer	FALSE	NA	NA	NA
Bc_ctg1_3287	B3	219.9603542	Bcer	EH	Bcer	FALSE	NA	NA	NA
Bc_ctg1_3288	B3	85.7969398	Bcer	Q	Bcer	TRUE	NA	NA	NA
Bc_ctg1_3289	B3	2.593074396	Bcer	L	Bcer	FALSE	NA	NA	NA
Bc_ctg1_329	B3	500.3506161	Bcer	C	Bcer	FALSE	Bc_Ga0417192_01_rn03	Other	NA
Bc_ctg1_3290	B3	1.127423651	Bcer	S	Bcer	FALSE	NA	NA	NA
Bc_ctg1_3291	B3	77.67948952	Bcer	M	Bcer	FALSE	NA	NA	NA
Bc_ctg1_3292	B3	252.9938672	Bcer	M	Bcer	FALSE	NA	NA	NA
Bc_ctg1_3293	B3	186.2503871	Bcer	H	Bcer	FALSE	NA	NA	NA
Bc_ctg1_3294	B3	8.004707919	Bcer	IQ	Bcer	TRUE	NA	NA	NA
Bc_ctg1_3295	B3	0	Bcer	NA	Bcer	FALSE	NA	NA	NA
Bc_ctg1_3296	B3	46.4498544	Bcer	NA	Bcer	FALSE	NA	NA	NA
Bc_ctg1_3297	B3	60.09168057	Bcer	P	Bcer	FALSE	NA	NA	NA
Bc_ctg1_3298	B3	96.05649502	Bcer	S	Bcer	FALSE	NA	NA	NA
Bc_ctg1_3299	B3	39.6853125	Bcer	S	Bcer	FALSE	NA	NA	NA
Bc_ctg1_3300	B3	135.9672923	Bcer	G	Bcer	FALSE	NA	NA	NA
Bc_ctg1_3301	B3	67.7581614	Bcer	M	Bcer	FALSE	NA	NA	NA
Bc_ctg1_3302	B3	62.12104314	Bcer	I	Bcer	FALSE	NA	NA	NA
Bc_ctg1_3303	B3	3.720498047	Bcer	NA	Bcer	FALSE	NA	NA	NA
Bc_ctg1_3304	B3	1.352908381	Bcer	I	Bcer	FALSE	NA	NA	NA
Bc_ctg1_3305	B3	0.563711825	Bcer	NA	Bcer	FALSE	NA	NA	NA
Bc_ctg1_3306	B3	79.70885209	Bcer	G	Bcer	FALSE	NA	NA	NA
Bc_ctg1_3307	B3	72.04237127	Bcer	M	Bcer	FALSE	NA	NA	NA
Bc_ctg1_3308	B3	28.52381836	Bcer	L	Bcer	FALSE	NA	NA	NA
Bc_ctg1_331	B3	0.338227095	Bcer	S	Bcer	FALSE	NA	NA	NA
Bc_ctg1_3310	B3	0.789196555	Bcer	E	Bcer	FALSE	NA	NA	NA
Bc_ctg1_3311	B3	5.524375888	Bcer	E	Bcer	FALSE	NA	NA	NA
Bc_ctg1_3312	B3	8.681162109	Bcer	NA	Bcer	FALSE	NA	NA	NA
Bc_ctg1_3313	B3	88.16452947	Bcer	E	Bcer	FALSE	NA	NA	NA
Bc_ctg1_3314	B3	8.906646839	Bcer	P	Bcer	FALSE	NA	NA	NA
Bc_ctg1_3315	B3	16.23490057	Bcer	EP	Bcer	FALSE	NA	NA	NA
Bc_ctg1_3316	B3	29.20027255	Bcer	P	Bcer	FALSE	NA	NA	NA
Bc_ctg1_3317	B3	40.47450905	Bcer	E	Bcer	FALSE	NA	NA	NA
Bc_ctg1_3318	B3	0	Bcer	O	Bcer	FALSE	NA	NA	NA
Bc_ctg1_3319	B3	1.352908381	Bcer	S	Bcer	FALSE	NA	NA	NA
Bc_ctg1_332	B3	1.916620206	Bcer	M	Bcer	FALSE	NA	NA	NA
Bc_ctg1_3320	B3	0	Bcer	S	Bcer	FALSE	NA	NA	NA
Bc_ctg1_3321	B3	0.789196555	Bcer	S	Bcer	FALSE	NA	NA	NA
Bc_ctg1_3322	B3	1.240166016	Bcer	NA	Bcer	FALSE	NA	NA	NA
Bc_ctg1_3324	B3	0	Bcer	NA	Bcer	FALSE	NA	NA	NA
Bc_ctg1_3325	B3	1.352908381	Bcer	V	Bcer	FALSE	NA	NA	NA
Bc_ctg1_3326	B3	0	Bcer	EGP	Bcer	FALSE	NA	NA	NA
Bc_ctg1_3327	B3	3.720498047	Bcer	T	Bcer	FALSE	NA	NA	NA
Bc_ctg1_3328	B3	0	Bcer	T	Bcer	FALSE	NA	NA	NA
Bc_ctg1_3329	B3	1.352908381	Bcer	S	Bcer	FALSE	NA	NA	NA
Bc_ctg1_333	B3	0	Bcer	S	Bcer	FALSE	NA	NA	NA
Bc_ctg1_3330	B3	0	Bcer	NA	Bcer	FALSE	NA	NA	NA

Bc_ctg1_3331	B3	9.470358664	Bcer	L	Bcer	FALSE	NA	NA	NA
Bc_ctg1_3332	B3	10.82326704	Bcer	L	Bcer	FALSE	NA	NA	NA
Bc_ctg1_3333	B3	13.07811435	Bcer	S	Bcer	FALSE	NA	NA	NA
Bc_ctg1_3334	B3	23.90138139	Bcer	K	Bcer	FALSE	NA	NA	NA
Bc_ctg1_3335	B3	3.269528587	Bcer	S	Bcer	FALSE	NA	NA	NA
Bc_ctg1_3336	B3	2.142104936	Bcer	EG	Bcer	FALSE	NA	NA	NA
Bc_ctg1_3337	B3	16.23490057	Bcer	KT	Bcer	FALSE	NA	NA	NA
Bc_ctg1_3338	B3	0.338227095	Bcer	S	Bcer	FALSE	NA	NA	NA
Bc_ctg1_3339	B3	0.22548473	Bcer	S	Bcer	FALSE	NA	NA	NA
Bc_ctg1_334	B3	17.58780895	Bcer	S	Bcer	FALSE	NA	NA	NA
Bc_ctg1_3340	B3	0	Bcer	NA	Bcer	FALSE	NA	NA	NA
Bc_ctg1_3341	B3	6.651799538	Bcer	K	Bcer	FALSE	NA	NA	NA
Bc_ctg1_3342	B3	36.75401101	Bcer	EGP	Bcer	FALSE	NA	NA	NA
Bc_ctg1_3343	B3	6.313572443	Bcer	NA	Bcer	FALSE	NA	NA	NA
Bc_ctg1_3345	B3	23.11218484	Bcer	NA	Bcer	FALSE	NA	NA	NA
Bc_ctg1_3346	B3	8.568419744	Bcer	M	Bcer	FALSE	NA	NA	NA
Bc_ctg1_3347	B3	2.367589666	Bcer	NA	Bcer	FALSE	NA	NA	NA
Bc_ctg1_3348	B3	7.779223189	Bcer	G	Bcer	FALSE	NA	NA	NA
Bc_ctg1_335	B3	0	Bcer	NA	Bcer	FALSE	NA	NA	NA
Bc_ctg1_3350	B3	2.705816761	Bcer	NA	Bcer	FALSE	NA	NA	NA
Bc_ctg1_3351	B3	4.960664062	Bcer	S	Bcer	FALSE	NA	NA	NA
Bc_ctg1_3353	B3	17.70055131	Bcer	S	Bcer	FALSE	NA	NA	NA
Bc_ctg1_3354	B3	3.495013317	Bcer	T	Bcer	FALSE	NA	NA	NA
Bc_ctg1_3356	B3	1.803877841	Bcer	K	Bcer	FALSE	NA	NA	NA
Bc_ctg1_3357	B3	5.975345348	Bcer	I	Bcer	FALSE	NA	NA	NA
Bc_ctg1_3358	B3	3.945982777	Bcer	S	Bcer	FALSE	NA	NA	NA
Bc_ctg1_3359	B3	0.789196555	Bcer	EGP	Bcer	FALSE	NA	NA	NA
Bc_ctg1_336	B3	0.338227095	Bcer	NA	Bcer	FALSE	NA	NA	NA
Bc_ctg1_3360	B3	16.79861239	Bcer	K	Bcer	FALSE	NA	NA	NA
Bc_ctg1_3362	B3	0.338227095	Bcer	V	Bcer	FALSE	NA	NA	NA
Bc_ctg1_3363	B3	18.26426314	Bcer	G	Bcer	FALSE	NA	NA	NA
Bc_ctg1_3364	B3	4.284209872	Bcer	K	Bcer	FALSE	NA	NA	NA
Bc_ctg1_3365	B3	253.7830637	Bcer	G	Bcer	FALSE	NA	NA	NA
Bc_ctg1_3366	B3	5.186148792	Bcer	I	Bcer	FALSE	NA	NA	NA
Bc_ctg1_3367	B3	9.80858576	Bcer	K	Bcer	FALSE	NA	NA	NA
Bc_ctg1_3368	B3	1.240166016	Bcer	NA	Bcer	FALSE	NA	NA	NA
Bc_ctg1_337	B3	11.38697887	Bcer	V	Bcer	FALSE	NA	NA	NA
Bc_ctg1_3371	B3	5.186148792	Bcer	NA	Bcer	FALSE	NA	NA	NA
Bc_ctg1_3372	B3	0.45096946	Bcer	NA	Bcer	FALSE	NA	NA	NA
Bc_ctg1_3373	B3	9.80858576	Bcer	K	Bcer	FALSE	NA	NA	NA
Bc_ctg1_3374	B3	0	Bcer	NA	Bcer	FALSE	NA	NA	NA
Bc_ctg1_3375	B3	0.338227095	Bcer	NA	Bcer	FALSE	NA	NA	NA
Bc_ctg1_3376	B3	14.54376509	Bcer	S	Bcer	FALSE	NA	NA	NA
Bc_ctg1_3377	B3	0.112742365	Bcer	NA	Bcer	FALSE	NA	NA	NA
Bc_ctg1_3378	B3	17.92603604	Bcer	D	Bcer	FALSE	NA	NA	NA
Bc_ctg1_3379	B3	1.578393111	Bcer	NA	Bcer	FALSE	NA	NA	NA
Bc_ctg1_338	B3	2.029362571	Bcer	NA	Bcer	FALSE	NA	NA	NA
Bc_ctg1_3380	B3	1.014681285	Bcer	K	Bcer	FALSE	NA	NA	NA
Bc_ctg1_3381	B3	0.67645419	Bcer	S	Bcer	FALSE	NA	NA	NA
Bc_ctg1_3382	B3	0.563711825	Bcer	S	Bcer	FALSE	NA	NA	NA
Bc_ctg1_3383	B3	0.90193892	Bcer	NA	Bcer	FALSE	NA	NA	NA
Bc_ctg1_3384	B3	0.45096946	Bcer	NA	Bcer	FALSE	NA	NA	NA
Bc_ctg1_3385	B3	0.45096946	Bcer	S	Bcer	FALSE	NA	NA	NA
Bc_ctg1_3386	B3	0	Bcer	K	Bcer	FALSE	NA	NA	NA
Bc_ctg1_3387	B3	0.563711825	Bcer	S	Bcer	FALSE	NA	NA	NA
Bc_ctg1_3388	B3	0.45096946	Bcer	NA	Bcer	FALSE	NA	NA	NA
Bc_ctg1_3389	B3	15.44570401	Bcer	T	Bcer	FALSE	NA	NA	NA
Bc_ctg1_339	B3	14.88199219	Bcer	P	Bcer	FALSE	NA	NA	NA
Bc_ctg1_3390	B3	33.82270952	Bcer	T	Bcer	FALSE	NA	NA	NA
Bc_ctg1_3391	B3	41.15096324	Bcer	K	Bcer	FALSE	NA	NA	NA
Bc_ctg1_3392	B3	56.03295543	Bcer	S	Bcer	FALSE	NA	NA	NA
Bc_ctg1_3393	B3	28.0728489	Bcer	T	Bcer	FALSE	NA	NA	NA

Bc_ctg1_3394	B3	43.18032581	Bcer	NT	Bcer	FALSE	NA	NA	NA
Bc_ctg1_3395	B3	92.33599698	Bcer	T	Bcer	FALSE	NA	NA	NA
Bc_ctg1_3396	B3	0.112742365	Bcer	NA	Bcer	FALSE	NA	NA	NA
Bc_ctg1_3398	B3	2.367589666	Bcer	S	Bcer	FALSE	NA	NA	NA
Bc_ctg1_3399	B3	2.142104936	Bcer	NA	Bcer	FALSE	NA	NA	NA
Bc_ctg1_34	B3	0.563711825	Bcer	S	Bcer	FALSE	NA	NA	NA
Bc_ctg1_340	B3	3.833240412	Bcer	E	Bcer	FALSE	NA	NA	NA
Bc_ctg1_3400	B3	249.837081	Bcer	S	Bcer	FALSE	NA	NA	NA
Bc_ctg1_3402	B3	2.367589666	Bcer	NA	Bcer	FALSE	NA	NA	NA
Bc_ctg1_3403	B3	1.578393111	Bcer	P	Bcer	FALSE	NA	NA	NA
Bc_ctg1_3404	B3	18.15152077	Bcer	K	Bcer	FALSE	NA	NA	NA
Bc_ctg1_3405	B3	12.40166016	Bcer	C	Bcer	FALSE	NA	NA	NA
Bc_ctg1_3406	B3	29.08753018	Bcer	M	Bcer	FALSE	NA	NA	NA
Bc_ctg1_3407	B3	0.22548473	Bcer	S	Bcer	FALSE	NA	NA	NA
Bc_ctg1_3408	B3	25.02880504	Bcer	NA	Bcer	FALSE	NA	NA	NA
Bc_ctg1_3409	B3	17.47506658	Bcer	S	Bcer	FALSE	NA	NA	NA
Bc_ctg1_341	B3	4.171467507	Bcer	K	Bcer	FALSE	NA	NA	NA
Bc_ctg1_3410	B3	15.22021928	Bcer	L	Bcer	FALSE	NA	NA	NA
Bc_ctg1_3411	B3	28.0728489	Bcer	L	Bcer	FALSE	NA	NA	NA
Bc_ctg1_3412	B3	260.3221209	Bcer	L	Bcer	FALSE	NA	NA	NA
Bc_ctg1_3413	B3	32.35705877	Bcer	S	Bcer	FALSE	NA	NA	NA
Bc_ctg1_3414	B3	41.71467507	Bcer	E	Bcer	FALSE	NA	NA	NA
Bc_ctg1_3415	B3	64.71411754	Bcer	E	Bcer	FALSE	NA	NA	NA
Bc_ctg1_3416	B3	96.28197975	Bcer	E	Bcer	FALSE	NA	NA	NA
Bc_ctg1_3417	B3	9.132131569	Bcer	K	Bcer	FALSE	NA	NA	NA
Bc_ctg1_3418	B3	0.112742365	Bcer	NA	Bcer	FALSE	NA	NA	NA
Bc_ctg1_3419	B3	2.367589666	Bcer	S	Bcer	FALSE	NA	NA	NA
Bc_ctg1_342	B3	2.593074396	Bcer	S	Bcer	FALSE	NA	NA	NA
Bc_ctg1_3420	B3	0.67645419	Bcer	S	Bcer	FALSE	NA	NA	NA
Bc_ctg1_3421	B3	3.833240412	Bcer	S	Bcer	FALSE	NA	NA	NA
Bc_ctg1_3423	B3	2.705816761	Bcer	G	Bcer	FALSE	NA	NA	NA
Bc_ctg1_3424	B3	0.563711825	Bcer	K	Bcer	FALSE	NA	NA	NA
Bc_ctg1_3425	B3	135.7418075	Bcer	L	Bcer	FALSE	NA	NA	NA
Bc_ctg1_3426	B3	60.99361949	Bcer	K	Bcer	FALSE	NA	NA	NA
Bc_ctg1_3427	B3	47.46453569	Bcer	G	Bcer	FALSE	NA	NA	NA
Bc_ctg1_3428	B3	298.7672674	Bcer	C	Bcer	FALSE	NA	NA	NA
Bc_ctg1_3429	B3	419.8525675	Bcer	C	Bcer	FALSE	NA	NA	NA
Bc_ctg1_343	B3	4.622436967	Bcer	S	Bcer	FALSE	NA	NA	NA
Bc_ctg1_3430	B3	17.13683949	Bcer	K	Bcer	FALSE	NA	NA	NA
Bc_ctg1_3431	B3	30.21495383	Bcer	V	Bcer	FALSE	NA	NA	NA
Bc_ctg1_3432	B3	2.367589666	Bcer	NA	Bcer	FALSE	NA	NA	NA
Bc_ctg1_3433	B3	11.83794833	Bcer	L	Bcer	FALSE	NA	NA	NA
Bc_ctg1_3434	B3	1.578393111	Bcer	K	Bcer	FALSE	NA	NA	NA
Bc_ctg1_3435	B3	0.22548473	Bcer	K	Bcer	FALSE	NA	NA	NA
Bc_ctg1_3436	B3	13.19085671	Bcer	D	Bcer	FALSE	NA	NA	NA
Bc_ctg1_3437	B3	20.6318528	Bcer	L	Bcer	FALSE	NA	NA	NA
Bc_ctg1_3438	B3	3.720498047	Bcer	NA	Bcer	FALSE	NA	NA	NA
Bc_ctg1_3439	B3	714.5611097	Bcer	M	Bcer	FALSE	NA	NA	NA
Bc_ctg1_344	B3	1.916620206	Bcer	S	Bcer	FALSE	NA	NA	NA
Bc_ctg1_3440	B3	0.112742365	Bcer	NA	Bcer	FALSE	NA	NA	NA
Bc_ctg1_3441	B3	8.117450284	Bcer	S	Bcer	FALSE	NA	NA	NA
Bc_ctg1_3442	B3	191.6620206	Bcer	K	Bcer	FALSE	NA	NA	NA
Bc_ctg1_3443	B3	133.3742179	Bcer	S	Bcer	FALSE	NA	NA	NA
Bc_ctg1_3444	B3	66.63073775	Bcer	FG	Bcer	FALSE	NA	NA	NA
Bc_ctg1_3445	B3	36.41578391	Bcer	V	Bcer	FALSE	NA	NA	NA
Bc_ctg1_3446	B3	54.68004705	Bcer	U	Bcer	FALSE	NA	NA	NA
Bc_ctg1_3447	B3	41.9401598	Bcer	P	Bcer	FALSE	NA	NA	NA
Bc_ctg1_3448	B3	3.495013317	Bcer	NA	Bcer	FALSE	NA	NA	NA
Bc_ctg1_3449	B3	4.171467507	Bcer	NA	Bcer	FALSE	NA	NA	NA
Bc_ctg1_345	B3	24.23960849	Bcer	S	Bcer	FALSE	Bc_Ga0417192_01_rn04	Other	NA
Bc_ctg1_3450	B3	10.82326704	Bcer	NA	Bcer	FALSE	NA	NA	NA
Bc_ctg1_3451	B3	12.06343306	Bcer	IU	Bcer	FALSE	NA	NA	NA

Bc_ctg1_3452	B3	1.578393111	Bcer	NA	Bcer	FALSE	NA	NA	NA
Bc_ctg1_3453	B3	4.396952237	Bcer	K	Bcer	FALSE	NA	NA	NA
Bc_ctg1_3454	B3	0.112742365	Bcer	NA	Bcer	FALSE	NA	NA	NA
Bc_ctg1_3455	B3	18.26426314	Bcer	S	Bcer	FALSE	NA	NA	NA
Bc_ctg1_3456	B3	80.04707919	Bcer	S	Bcer	FALSE	NA	NA	NA
Bc_ctg1_3457	B3	36.75401101	Bcer	S	Bcer	FALSE	NA	NA	NA
Bc_ctg1_3458	B3	3.044043856	Bcer	NA	Bcer	FALSE	NA	NA	NA
Bc_ctg1_3459	B3	1.691135476	Bcer	S	Bcer	FALSE	NA	NA	NA
Bc_ctg1_346	B3	27.84736417	Bcer	C	Bcer	FALSE	Bc_Ga0417192_01_rn04	Ancillary biosynthesis	SMCOG1028
Bc_ctg1_3460	B3	2.142104936	Bcer	NA	Bcer	FALSE	NA	NA	NA
Bc_ctg1_3461	B3	1.352908381	Bcer	K	Bcer	FALSE	NA	NA	NA
Bc_ctg1_3462	B3	10.71052468	Bcer	M	Bcer	FALSE	NA	NA	NA
Bc_ctg1_3463	B3	86.58613636	Bcer	E	Bcer	FALSE	NA	NA	NA
Bc_ctg1_3464	B3	102.3700675	Bcer	IQ	Bcer	TRUE	NA	NA	NA
Bc_ctg1_3465	B3	38.33240412	Bcer	EG	Bcer	FALSE	NA	NA	NA
Bc_ctg1_3466	B3	2.480332031	Bcer	S	Bcer	FALSE	NA	NA	NA
Bc_ctg1_3467	B3	7.440996093	Bcer	S	Bcer	FALSE	NA	NA	NA
Bc_ctg1_3468	B3	100.1152202	Bcer	S	Bcer	FALSE	NA	NA	NA
Bc_ctg1_3469	B3	2.818559126	Bcer	M	Bcer	FALSE	NA	NA	NA
Bc_ctg1_347	B3	95.1545561	Bcer	E	Bcer	FALSE	Bc_Ga0417192_01_rn04	Ancillary biosynthesis	SMCOG1038
Bc_ctg1_3470	B3	116.8010902	Bcer	H	Bcer	FALSE	NA	NA	NA
Bc_ctg1_3471	B3	133.4869602	Bcer	H	Bcer	FALSE	NA	NA	NA
Bc_ctg1_3472	B3	309.5905344	Bcer	H	Bcer	FALSE	NA	NA	NA
Bc_ctg1_3473	B3	95.83101029	Bcer	S	Bcer	FALSE	NA	NA	NA
Bc_ctg1_3474	B3	12.73988725	Bcer	S	Bcer	FALSE	NA	NA	NA
Bc_ctg1_3475	B3	0.112742365	Bcer	NA	Bcer	FALSE	NA	NA	NA
Bc_ctg1_3476	B3	103.6102335	Bcer	NA	Bcer	FALSE	NA	NA	NA
Bc_ctg1_3477	B3	1.803877841	Bcer	K	Bcer	FALSE	NA	NA	NA
Bc_ctg1_3478	B3	6.200830078	Bcer	KT	Bcer	FALSE	NA	NA	NA
Bc_ctg1_3479	B3	74.40996093	Bcer	K	Bcer	FALSE	NA	NA	NA
Bc_ctg1_348	B3	51.2977761	Bcer	EGP	Bcer	FALSE	Bc_Ga0417192_01_rn04	Transport	SMCOG1005
Bc_ctg1_3480	B3	27.84736417	Bcer	S	Bcer	FALSE	NA	NA	NA
Bc_ctg1_3481	B3	1.014681285	Bcer	NA	Bcer	FALSE	NA	NA	NA
Bc_ctg1_3482	B3	7.553738458	Bcer	K	Bcer	FALSE	NA	NA	NA
Bc_ctg1_3483	B3	7.779223189	Bcer	S	Bcer	FALSE	NA	NA	NA
Bc_ctg1_3484	B3	1.578393111	Bcer	M	Bcer	FALSE	NA	NA	NA
Bc_ctg1_3485	B3	819.0732821	Bcer	K	Bcer	FALSE	NA	NA	NA
Bc_ctg1_3486	B3	0	Bcer	S	Bcer	FALSE	NA	NA	NA
Bc_ctg1_3487	B3	144.4229696	Bcer	S	Bcer	FALSE	NA	NA	NA
Bc_ctg1_3488	B3	360.7755682	Bcer	H	Bcer	FALSE	NA	NA	NA
Bc_ctg1_3489	B3	0.67645419	Bcer	NA	Bcer	FALSE	NA	NA	NA
Bc_ctg1_349	B3	0.338227095	Bcer	NA	Bcer	FALSE	Bc_Ga0417192_01_rn04	Other	NA
Bc_ctg1_3490	B3	43.74403764	Bcer	IQ	Bcer	TRUE	NA	NA	NA
Bc_ctg1_3491	B3	0	Bcer	M	Bcer	FALSE	NA	NA	NA
Bc_ctg1_3492	B3	3.495013317	Bcer	P	Bcer	FALSE	NA	NA	NA
Bc_ctg1_3493	B3	3.382270952	Bcer	O	Bcer	FALSE	NA	NA	NA
Bc_ctg1_3494	B3	3.607755682	Bcer	P	Bcer	FALSE	NA	NA	NA
Bc_ctg1_3495	B3	6.200830078	Bcer	P	Bcer	FALSE	NA	NA	NA
Bc_ctg1_3496	B3	842.1854669	Bcer	S	Bcer	FALSE	NA	NA	NA
Bc_ctg1_3497	B3	48.93018643	Bcer	S	Bcer	FALSE	NA	NA	NA
Bc_ctg1_3498	B3	2.029362571	Bcer	S	Bcer	FALSE	NA	NA	NA
Bc_ctg1_3499	B3	8.793904474	Bcer	S	Bcer	FALSE	NA	NA	NA
Bc_ctg1_35	B3	5.073406427	Bcer	S	Bcer	FALSE	NA	NA	NA
Bc_ctg1_350	B3	0.112742365	Bcer	S	Bcer	FALSE	Bc_Ga0417192_01_rn04	Other	NA
Bc_ctg1_3500	B3	4.284209872	Bcer	K	Bcer	FALSE	NA	NA	NA
Bc_ctg1_3501	B3	6.200830078	Bcer	U	Bcer	FALSE	NA	NA	NA
Bc_ctg1_3502	B3	6.651799538	Bcer	S	Bcer	FALSE	NA	NA	NA
Bc_ctg1_3503	B3	0.67645419	Bcer	K	Bcer	FALSE	NA	NA	NA
Bc_ctg1_3504	B3	60.3171653	Bcer	K	Bcer	FALSE	NA	NA	NA
Bc_ctg1_3505	B3	2.593074396	Bcer	E	Bcer	FALSE	NA	NA	NA
Bc_ctg1_3506	B3	16.1221582	Bcer	T	Bcer	FALSE	NA	NA	NA
Bc_ctg1_3507	B3	6.088087713	Bcer	T	Bcer	FALSE	NA	NA	NA

Bc_ctg1_3508	B3	25.36703214	Bcer	S	Bcer	FALSE	NA	NA	NA
Bc_ctg1_3509	B3	4.735179332	Bcer	NA	Bcer	FALSE	NA	NA	NA
Bc_ctg1_351	B3	0.112742365	Bcer	NA	Bcer	FALSE	Bc_Ga0417192_01_rn04	Other	NA
Bc_ctg1_3510	B3	2.029362571	Bcer	NA	Bcer	FALSE	NA	NA	NA
Bc_ctg1_3511	B3	12.28891779	Bcer	O	Bcer	FALSE	NA	NA	NA
Bc_ctg1_3512	B3	41.9401598	Bcer	NT	Bcer	FALSE	NA	NA	NA
Bc_ctg1_3513	B3	37.4304652	Bcer	D	Bcer	FALSE	NA	NA	NA
Bc_ctg1_3515	B3	2.931301491	Bcer	D	Bcer	FALSE	NA	NA	NA
Bc_ctg1_3516	B3	9.357616299	Bcer	C	Bcer	FALSE	NA	NA	NA
Bc_ctg1_3517	B3	15.22021928	Bcer	C	Bcer	FALSE	NA	NA	NA
Bc_ctg1_3518	B3	5.073406427	Bcer	S	Bcer	FALSE	NA	NA	NA
Bc_ctg1_3519	B3	0.90193892	Bcer	NA	Bcer	FALSE	NA	NA	NA
Bc_ctg1_352	B3	0.112742365	Bcer	NA	Bcer	FALSE	Bc_Ga0417192_01_rn04	Other	NA
Bc_ctg1_3520	B3	32.2443164	Bcer	K	Bcer	FALSE	NA	NA	NA
Bc_ctg1_3521	B3	0.112742365	Bcer	NA	Bcer	FALSE	NA	NA	NA
Bc_ctg1_3522	B3	1.916620206	Bcer	NA	Bcer	FALSE	NA	NA	NA
Bc_ctg1_3523	B3	14.65650746	Bcer	K	Bcer	FALSE	NA	NA	NA
Bc_ctg1_3524	B3	19.05345969	Bcer	S	Bcer	FALSE	NA	NA	NA
Bc_ctg1_3525	B3	16.68587003	Bcer	U	Bcer	FALSE	NA	NA	NA
Bc_ctg1_3526	B3	124.467571	Bcer	L	Bcer	FALSE	NA	NA	NA
Bc_ctg1_3527	B3	205.9803009	Bcer	L	Bcer	FALSE	NA	NA	NA
Bc_ctg1_3528	B3	2.367589666	Bcer	K	Bcer	FALSE	NA	NA	NA
Bc_ctg1_3529	B3	0	Bcer	S	Bcer	FALSE	NA	NA	NA
Bc_ctg1_353	B3	1.578393111	Bcer	NA	Bcer	FALSE	Bc_Ga0417192_01_rn04	Other	NA
Bc_ctg1_3530	B3	0.67645419	Bcer	S	Bcer	FALSE	NA	NA	NA
Bc_ctg1_3531	B3	0	Bcer	S	Bcer	FALSE	NA	NA	NA
Bc_ctg1_3532	B3	1.127423651	Bcer	S	Bcer	FALSE	NA	NA	NA
Bc_ctg1_3533	B3	0	Bcer	S	Bcer	FALSE	NA	NA	NA
Bc_ctg1_3534	B3	0.112742365	Bcer	S	Bcer	FALSE	NA	NA	NA
Bc_ctg1_3535	B3	0.112742365	Bcer	NA	Bcer	FALSE	NA	NA	NA
Bc_ctg1_3536	B3	28.86204545	Bcer	Q	Bcer	TRUE	NA	NA	NA
Bc_ctg1_3537	B3	9.357616299	Bcer	NA	Bcer	FALSE	NA	NA	NA
Bc_ctg1_3538	B3	2264.768629	Bcer	E	Bcer	FALSE	NA	NA	NA
Bc_ctg1_3539	B3	40.02353959	Bcer	S	Bcer	FALSE	NA	NA	NA
Bc_ctg1_354	B3	18.26426314	Bcer	IQ	Bcer	TRUE	Bc_Ga0417192_01_rn04	Ancillary biosynthesis	SMCOG1001
Bc_ctg1_3540	B3	0.45096946	Bcer	S	Bcer	FALSE	NA	NA	NA
Bc_ctg1_3541	B3	6.539057173	Bcer	E	Bcer	FALSE	NA	NA	NA
Bc_ctg1_3542	B3	125.5949947	Bcer	H	Bcer	FALSE	NA	NA	NA
Bc_ctg1_3543	B3	2165.217121	Bcer	P	Bcer	FALSE	NA	NA	NA
Bc_ctg1_3544	B3	4.735179332	Bcer	P	Bcer	FALSE	NA	NA	NA
Bc_ctg1_3545	B3	12.96537198	Bcer	G	Bcer	FALSE	NA	NA	NA
Bc_ctg1_3546	B3	28.97478782	Bcer	K	Bcer	FALSE	NA	NA	NA
Bc_ctg1_3547	B3	139.4623056	Bcer	S	Bcer	FALSE	NA	NA	NA
Bc_ctg1_3548	B3	56.48392489	Bcer	S	Bcer	FALSE	NA	NA	NA
Bc_ctg1_3549	B3	51.63600319	Bcer	M	Bcer	FALSE	Bc_Ga0417192_01_rn24	Other	NA
Bc_ctg1_355	B3	112.7423651	Bcer	S	Bcer	FALSE	Bc_Ga0417192_01_rn04	Core biosynthesis	None
Bc_ctg1_3551	B3	3.607755682	Bcer	S	Bcer	FALSE	Bc_Ga0417192_01_rn24	Other	NA
Bc_ctg1_3552	B3	9.921328125	Bcer	S	Bcer	FALSE	Bc_Ga0417192_01_rn24	Other	NA
Bc_ctg1_3554	B3	82.64015358	Bcer	S	Bcer	FALSE	Bc_Ga0417192_01_rn24	Ancillary biosynthesis	SMCOG1179
Bc_ctg1_3555	B3	4.058725142	Bcer	S	Bcer	FALSE	Bc_Ga0417192_01_rn24	Other	NA
Bc_ctg1_3556	B3	377.1232111	Bcer	O	Bcer	FALSE	Bc_Ga0417192_01_rn24	Other	SMCOG1223
Bc_ctg1_3557	B3	60.54265003	Bcer	S	Bcer	FALSE	Bc_Ga0417192_01_rn24	Other	NA
Bc_ctg1_3558	B3	0.789196555	Bcer	I	Bcer	FALSE	Bc_Ga0417192_01_rn24	Other	NA
Bc_ctg1_3559	B3	0.789196555	Bcer	GM	Bcer	FALSE	Bc_Ga0417192_01_rn24	Other	NA
Bc_ctg1_356	B3	31.79334694	Bcer	S	Bcer	FALSE	Bc_Ga0417192_01_rn04	Other	NA
Bc_ctg1_3560	B3	0	Bcer	S	Bcer	FALSE	Bc_Ga0417192_01_rn24	Other	NA
Bc_ctg1_3561	B3	113.0805921	Bcer	I	Bcer	FALSE	Bc_Ga0417192_01_rn24	Core biosynthesis	SMCOG1084
Bc_ctg1_3562	B3	380.6182244	Bcer	I	Bcer	FALSE	Bc_Ga0417192_01_rn24	Core biosynthesis	SMCOG1022
Bc_ctg1_3563	B3	1.127423651	Bcer	O	Bcer	FALSE	Bc_Ga0417192_01_rn24	Other	NA
Bc_ctg1_3564	B3	3.382270952	Bcer	S	Bcer	FALSE	Bc_Ga0417192_01_rn24	Other	NA
Bc_ctg1_3565	B3	143.1828036	Bcer	J	Bcer	FALSE	Bc_Ga0417192_01_rn24	Other	NA
Bc_ctg1_3566	B3	14.54376509	Bcer	S	Bcer	FALSE	Bc_Ga0417192_01_rn24	Other	NA

Bc_ctg1_3567	B3	661.0084863	Bcer	E	Bcer	FALSE	Bc_Ga0417192_01_rn24	Transport	SMCOG1068
Bc_ctg1_3568	B3	67.64541903	Bcer	E	Bcer	FALSE	Bc_Ga0417192_01_rn24	Other	NA
Bc_ctg1_3569	B3	569.5744282	Bcer	P	Bcer	FALSE	Bc_Ga0417192_01_rn24	Transport	SMCOG1118
Bc_ctg1_357	B3	56.1456978	Bcer	P	Bcer	FALSE	Bc_Ga0417192_01_rn04	Other	NA
Bc_ctg1_3570	B3	608.8087713	Bcer	EP	Bcer	FALSE	Bc_Ga0417192_01_rn24	Transport	SMCOG1085
Bc_ctg1_3571	B3	1217.843027	Bcer	P	Bcer	FALSE	Bc_Ga0417192_01_rn24	Transport	SMCOG1000
Bc_ctg1_3572	B3	849.7392054	Bcer	E	Bcer	FALSE	Bc_Ga0417192_01_rn24	Transport	SMCOG1000
Bc_ctg1_3573	B3	0.22548473	Bcer	L	Bcer	FALSE	NA	NA	NA
Bc_ctg1_3574	B3	0	Bcer	L	Bcer	FALSE	NA	NA	NA
Bc_ctg1_3575	B3	13.52908381	Bcer	V	Bcer	FALSE	NA	NA	NA
Bc_ctg1_3576	B3	1187.966301	Bcer	E	Bcer	FALSE	NA	NA	NA
Bc_ctg1_3577	B3	3.044043856	Bcer	NA	Bcer	FALSE	NA	NA	NA
Bc_ctg1_3578	B3	1249.298147	Bcer	K	Bcer	FALSE	NA	NA	NA
Bc_ctg1_3579	B3	1.127423651	Bcer	P	Bcer	FALSE	NA	NA	NA
Bc_ctg1_358	B3	2.142104936	Bcer	NA	Bcer	FALSE	Bc_Ga0417192_01_rn04	Other	NA
Bc_ctg1_3580	B3	145.0994238	Bcer	NOT	Bcer	FALSE	NA	NA	NA
Bc_ctg1_3581	B3	48.36647461	Bcer	I	Bcer	FALSE	NA	NA	NA
Bc_ctg1_3582	B3	4.171467507	Bcer	S	Bcer	FALSE	NA	NA	NA
Bc_ctg1_3583	B3	553.0013006	Bcer	E	Bcer	FALSE	NA	NA	NA
Bc_ctg1_3584	B3	33.37174006	Bcer	Q	Bcer	TRUE	Bc_Ga0417192_01_rn25	Other	NA
Bc_ctg1_3585	B3	13.8673109	Bcer	S	Bcer	FALSE	Bc_Ga0417192_01_rn25	Other	NA
Bc_ctg1_3586	B3	12.96537198	Bcer	S	Bcer	FALSE	Bc_Ga0417192_01_rn25	Other	NA
Bc_ctg1_3587	B3	33.70996715	Bcer	S	Bcer	FALSE	Bc_Ga0417192_01_rn25	Other	NA
Bc_ctg1_3588	B3	235.8570277	Bcer	S	Bcer	FALSE	Bc_Ga0417192_01_rn25	Other	NA
Bc_ctg1_3589	B3	234.1658922	Bcer	G	Bcer	FALSE	Bc_Ga0417192_01_rn25	Other	NA
Bc_ctg1_359	B3	1.014681285	Bcer	J	Bcer	FALSE	Bc_Ga0417192_01_rn04	Ancillary biosynthesis	SMCOG1111
Bc_ctg1_3590	B3	203.0489995	Bcer	J	Bcer	FALSE	Bc_Ga0417192_01_rn25	Ancillary biosynthesis	SMCOG1279
Bc_ctg1_3591	B3	1.127423651	Bcer	UW	Bcer	FALSE	Bc_Ga0417192_01_rn25	Other	NA
Bc_ctg1_3592	B3	3.945982777	Bcer	T	Bcer	FALSE	Bc_Ga0417192_01_rn25	Other	NA
Bc_ctg1_3593	B3	0.563711825	Bcer	D	Bcer	FALSE	Bc_Ga0417192_01_rn25	Other	NA
Bc_ctg1_3594	B3	0.112742365	Bcer	M	Bcer	FALSE	Bc_Ga0417192_01_rn25	Core biosynthesis	None
Bc_ctg1_3595	B3	0.112742365	Bcer	H	Bcer	FALSE	Bc_Ga0417192_01_rn25	Other	NA
Bc_ctg1_3596	B3	0	Bcer	S	Bcer	FALSE	Bc_Ga0417192_01_rn25	Other	NA
Bc_ctg1_3597	B3	0.67645419	Bcer	K	Bcer	FALSE	Bc_Ga0417192_01_rn25	Other	NA
Bc_ctg1_3598	B3	0.338227095	Bcer	M	Bcer	FALSE	Bc_Ga0417192_01_rn25	Core biosynthesis	None
Bc_ctg1_3599	B3	1.014681285	Bcer	H	Bcer	FALSE	Bc_Ga0417192_01_rn25	Other	NA
Bc_ctg1_36	B3	0.789196555	Bcer	K	Bcer	FALSE	NA	NA	NA
Bc_ctg1_360	B3	21.98476119	Bcer	P	Bcer	FALSE	Bc_Ga0417192_01_rn04	Transport	SMCOG1033
Bc_ctg1_3600	B3	2.480332031	Bcer	S	Bcer	FALSE	Bc_Ga0417192_01_rn25	Other	NA
Bc_ctg1_3601	B3	1.578393111	Bcer	S	Bcer	FALSE	Bc_Ga0417192_01_rn25	Other	NA
Bc_ctg1_3602	B3	1.465650746	Bcer	M	Bcer	FALSE	Bc_Ga0417192_01_rn25	Ancillary biosynthesis	SMCOG1064
Bc_ctg1_3603	B3	1.916620206	Bcer	M	Bcer	FALSE	Bc_Ga0417192_01_rn25	Core biosynthesis	None
Bc_ctg1_3604	B3	5.298891157	Bcer	M	Bcer	FALSE	Bc_Ga0417192_01_rn25	Core biosynthesis	SMCOG1010
Bc_ctg1_3605	B3	5.073406427	Bcer	M	Bcer	FALSE	Bc_Ga0417192_01_rn25	Core biosynthesis	SMCOG1010
Bc_ctg1_3606	B3	312.9728054	Bcer	I	Bcer	FALSE	Bc_Ga0417192_01_rn25	Ancillary biosynthesis	SMCOG1001
Bc_ctg1_3607	B3	0	Bcer	S	Bcer	FALSE	Bc_Ga0417192_01_rn25	Other	NA
Bc_ctg1_3608	B3	0	Bcer	S	Bcer	FALSE	Bc_Ga0417192_01_rn25	Other	NA
Bc_ctg1_3609	B3	0.563711825	Bcer	S	Bcer	FALSE	Bc_Ga0417192_01_rn25	Other	NA
Bc_ctg1_361	B3	6.990026633	Bcer	P	Bcer	FALSE	Bc_Ga0417192_01_rn04	Transport	SMCOG1011
Bc_ctg1_3610	B3	0.45096946	Bcer	NA	Bcer	FALSE	Bc_Ga0417192_01_rn25	Other	NA
Bc_ctg1_3611	B3	7.440996093	Bcer	S	Bcer	FALSE	Bc_Ga0417192_01_rn25	Other	NA
Bc_ctg1_3612	B3	150.8492844	Bcer	L	Bcer	FALSE	Bc_Ga0417192_01_rn25	Other	NA
Bc_ctg1_3613	B3	367.6528524	Bcer	S	Bcer	FALSE	Bc_Ga0417192_01_rn25	Other	NA
Bc_ctg1_3614	B3	397.5295792	Bcer	J	Bcer	FALSE	Bc_Ga0417192_01_rn25	Other	NA
Bc_ctg1_3615	B3	64.8268599	Bcer	P	Bcer	FALSE	Bc_Ga0417192_01_rn25	Ancillary biosynthesis	SMCOG1098
Bc_ctg1_3616	B3	105.7523384	Bcer	EG	Bcer	FALSE	Bc_Ga0417192_01_rn25	Transport	SMCOG1166
Bc_ctg1_3617	B3	0.45096946	Bcer	S	Bcer	FALSE	Bc_Ga0417192_01_rn25	Other	NA
Bc_ctg1_3618	B3	1.352908381	Bcer	N	Bcer	FALSE	NA	NA	NA
Bc_ctg1_3619	B3	101.5808709	Bcer	E	Bcer	FALSE	NA	NA	NA
Bc_ctg1_362	B3	10.03407049	Bcer	P	Bcer	FALSE	Bc_Ga0417192_01_rn04	Transport	SMCOG1011
Bc_ctg1_3620	B3	2.480332031	Bcer	EH	Bcer	FALSE	NA	NA	NA
Bc_ctg1_3621	B3	2.705816761	Bcer	EH	Bcer	FALSE	NA	NA	NA

Bc_ctg1_3622	B3	3.156786221	Bcer	E	Bcer	FALSE	NA	NA	NA
Bc_ctg1_3623	B3	2.705816761	Bcer	E	Bcer	FALSE	NA	NA	NA
Bc_ctg1_3624	B3	1.578393111	Bcer	E	Bcer	FALSE	NA	NA	NA
Bc_ctg1_3625	B3	4.847921697	Bcer	E	Bcer	FALSE	NA	NA	NA
Bc_ctg1_3626	B3	3.156786221	Bcer	E	Bcer	FALSE	Bc_Ga0417192_01_rn26	Other	NA
Bc_ctg1_3627	B3	2.254847301	Bcer	NA	Bcer	FALSE	Bc_Ga0417192_01_rn26	Other	NA
Bc_ctg1_3628	B3	4.509694602	Bcer	C	Bcer	FALSE	Bc_Ga0417192_01_rn26	Other	NA
Bc_ctg1_3629	B3	31.45511985	Bcer	S	Bcer	FALSE	Bc_Ga0417192_01_rn26	Other	NA
Bc_ctg1_363	B3	3.607755682	Bcer	S	Bcer	FALSE	Bc_Ga0417192_01_rn04	Other	NA
Bc_ctg1_3630	B3	16.57312766	Bcer	NA	Bcer	FALSE	Bc_Ga0417192_01_rn26	Other	NA
Bc_ctg1_3631	B3	0.45096946	Bcer	M	Bcer	FALSE	Bc_Ga0417192_01_rn26	Other	NA
Bc_ctg1_3632	B3	4.284209872	Bcer	S	Bcer	FALSE	Bc_Ga0417192_01_rn26	Other	NA
Bc_ctg1_3633	B3	0.112742365	Bcer	NA	Bcer	FALSE	Bc_Ga0417192_01_rn26	Other	NA
Bc_ctg1_3634	B3	9.470358664	Bcer	P	Bcer	FALSE	Bc_Ga0417192_01_rn26	Ancillary biosynthesis	SMCOG1175
Bc_ctg1_3635	B3	2.593074396	Bcer	S	Bcer	FALSE	Bc_Ga0417192_01_rn26	Other	NA
Bc_ctg1_3636	B3	3.833240412	Bcer	NA	Bcer	FALSE	Bc_Ga0417192_01_rn26	Other	NA
Bc_ctg1_3637	B3	1.803877841	Bcer	C	Bcer	FALSE	Bc_Ga0417192_01_rn26	Core biosynthesis	None
Bc_ctg1_3638	B3	0.563711825	Bcer	S	Bcer	FALSE	Bc_Ga0417192_01_rn26	Core biosynthesis	None
Bc_ctg1_3639	B3	1.127423651	Bcer	H	Bcer	FALSE	Bc_Ga0417192_01_rn26	Core biosynthesis	None
Bc_ctg1_364	B3	11.16149414	Bcer	S	Bcer	FALSE	Bc_Ga0417192_01_rn04	Other	NA
Bc_ctg1_3640	B3	1388.873195	Bcer	C	Bcer	FALSE	Bc_Ga0417192_01_rn26	Other	NA
Bc_ctg1_3641	B3	2511.336182	Bcer	C	Bcer	FALSE	Bc_Ga0417192_01_rn26	Other	NA
Bc_ctg1_3642	B3	0.338227095	Bcer	NA	Bcer	FALSE	Bc_Ga0417192_01_rn26	Other	NA
Bc_ctg1_3643	B3	0.563711825	Bcer	NA	Bcer	FALSE	Bc_Ga0417192_01_rn26	Other	NA
Bc_ctg1_3644	B3	1.578393111	Bcer	NA	Bcer	FALSE	Bc_Ga0417192_01_rn26	Other	NA
Bc_ctg1_3645	B3	2.931301491	Bcer	NA	Bcer	FALSE	Bc_Ga0417192_01_rn26	Other	NA
Bc_ctg1_3646	B3	117.7030291	Bcer	NA	Bcer	FALSE	Bc_Ga0417192_01_rn26	Other	NA
Bc_ctg1_3647	B3	0.112742365	Bcer	NA	Bcer	FALSE	NA	NA	NA
Bc_ctg1_3648	B3	3.720498047	Bcer	NA	Bcer	FALSE	NA	NA	NA
Bc_ctg1_3649	B3	60.42990767	Bcer	M	Bcer	FALSE	NA	NA	NA
Bc_ctg1_365	B3	45.32243075	Bcer	S	Bcer	FALSE	Bc_Ga0417192_01_rn04	Other	NA
Bc_ctg1_3650	B3	0.90193892	Bcer	U	Bcer	FALSE	NA	NA	NA
Bc_ctg1_3651	B3	0.789196555	Bcer	S	Bcer	FALSE	NA	NA	NA
Bc_ctg1_3652	B3	0.22548473	Bcer	M	Bcer	FALSE	NA	NA	NA
Bc_ctg1_3653	B3	9.583101029	Bcer	S	Bcer	FALSE	NA	NA	NA
Bc_ctg1_3654	B3	65.95428356	Bcer	K	Bcer	FALSE	NA	NA	NA
Bc_ctg1_3655	B3	0.22548473	Bcer	NA	Bcer	FALSE	NA	NA	NA
Bc_ctg1_3656	B3	21.30830699	Bcer	V	Bcer	FALSE	NA	NA	NA
Bc_ctg1_3657	B3	54.56730469	Bcer	C	Bcer	FALSE	NA	NA	NA
Bc_ctg1_3658	B3	60.88087713	Bcer	P	Bcer	FALSE	NA	NA	NA
Bc_ctg1_3659	B3	42.39112926	Bcer	E	Bcer	FALSE	NA	NA	NA
Bc_ctg1_366	B3	5.411633522	Bcer	S	Bcer	FALSE	Bc_Ga0417192_01_rn04	Other	NA
Bc_ctg1_3660	B3	40.36176669	Bcer	E	Bcer	FALSE	NA	NA	NA
Bc_ctg1_3661	B3	90.6448615	Bcer	P	Bcer	FALSE	NA	NA	NA
Bc_ctg1_3662	B3	9.357616299	Bcer	I	Bcer	FALSE	NA	NA	NA
Bc_ctg1_3663	B3	143.4082883	Bcer	S	Bcer	FALSE	NA	NA	NA
Bc_ctg1_3664	B3	0.67645419	Bcer	S	Bcer	FALSE	NA	NA	NA
Bc_ctg1_3665	B3	25.70525923	Bcer	J	Bcer	FALSE	NA	NA	NA
Bc_ctg1_3666	B3	45.88614258	Bcer	S	Bcer	FALSE	NA	NA	NA
Bc_ctg1_3667	B3	1.803877841	Bcer	J	Bcer	FALSE	NA	NA	NA
Bc_ctg1_3668	B3	48.36647461	Bcer	C	Bcer	FALSE	NA	NA	NA
Bc_ctg1_3669	B3	63.24846679	Bcer	C	Bcer	FALSE	NA	NA	NA
Bc_ctg1_367	B3	0.338227095	Bcer	S	Bcer	FALSE	Bc_Ga0417192_01_rn04	Other	NA
Bc_ctg1_3670	B3	3.720498047	Bcer	KT	Bcer	FALSE	NA	NA	NA
Bc_ctg1_3671	B3	13.75456854	Bcer	S	Bcer	FALSE	NA	NA	NA
Bc_ctg1_3672	B3	10.48503995	Bcer	T	Bcer	FALSE	NA	NA	NA
Bc_ctg1_3673	B3	42.61661399	Bcer	T	Bcer	FALSE	NA	NA	NA
Bc_ctg1_3674	B3	579.383014	Bcer	K	Bcer	FALSE	NA	NA	NA
Bc_ctg1_3675	B3	217.5927646	Bcer	C	Bcer	FALSE	NA	NA	NA
Bc_ctg1_3676	B3	870.3710582	Bcer	C	Bcer	FALSE	NA	NA	NA
Bc_ctg1_3677	B3	357.0550701	Bcer	S	Bcer	FALSE	NA	NA	NA
Bc_ctg1_3678	B3	36.52852628	Bcer	NOU	Bcer	FALSE	NA	NA	NA

Bc_ctg1_3679	B3	9.583101029	Bcer	S	Bcer	FALSE	NA	NA	NA
Bc_ctg1_368	B3	0	Bcer	S	Bcer	FALSE	Bc_Ga0417192_01_rn04	Other	NA
Bc_ctg1_3680	B3	363.2559002	Bcer	P	Bcer	FALSE	NA	NA	NA
Bc_ctg1_3681	B3	10.03407049	Bcer	P	Bcer	FALSE	NA	NA	NA
Bc_ctg1_3682	B3	0.563711825	Bcer	P	Bcer	FALSE	NA	NA	NA
Bc_ctg1_3683	B3	0.67645419	Bcer	S	Bcer	FALSE	NA	NA	NA
Bc_ctg1_3684	B3	313.5365172	Bcer	I	Bcer	FALSE	NA	NA	NA
Bc_ctg1_3685	B3	723.5804989	Bcer	S	Bcer	FALSE	NA	NA	NA
Bc_ctg1_3686	B3	213.5340394	Bcer	K	Bcer	FALSE	NA	NA	NA
Bc_ctg1_3687	B3	91.99776988	Bcer	S	Bcer	FALSE	NA	NA	NA
Bc_ctg1_3688	B3	135.5163228	Bcer	IQ	Bcer	TRUE	NA	NA	NA
Bc_ctg1_3689	B3	141.2661834	Bcer	I	Bcer	FALSE	NA	NA	NA
Bc_ctg1_369	B3	4.509694602	Bcer	K	Bcer	FALSE	Bc_Ga0417192_01_rn04	Regulation	SMCOG1014
Bc_ctg1_3690	B3	31.56786221	Bcer	E	Bcer	FALSE	NA	NA	NA
Bc_ctg1_3691	B3	86.92436345	Bcer	P	Bcer	FALSE	NA	NA	NA
Bc_ctg1_3694	B3	0	Bcer	P	Bcer	FALSE	NA	NA	NA
Bc_ctg1_3695	B3	0.112742365	Bcer	P	Bcer	FALSE	NA	NA	NA
Bc_ctg1_3696	B3	1.691135476	Bcer	P	Bcer	FALSE	NA	NA	NA
Bc_ctg1_3697	B3	0.112742365	Bcer	S	Bcer	FALSE	NA	NA	NA
Bc_ctg1_3698	B3	1.240166016	Bcer	E	Bcer	FALSE	NA	NA	NA
Bc_ctg1_3699	B3	4.058725142	Bcer	E	Bcer	FALSE	NA	NA	NA
Bc_ctg1_37	B3	0.22548473	Bcer	S	Bcer	FALSE	NA	NA	NA
Bc_ctg1_370	B3	2.367589666	Bcer	C	Bcer	FALSE	Bc_Ga0417192_01_rn04	Ancillary biosynthesis	SMCOG1028
Bc_ctg1_3700	B3	3.156786221	Bcer	K	Bcer	FALSE	NA	NA	NA
Bc_ctg1_3702	B3	91.77228515	Bcer	M	Bcer	FALSE	NA	NA	NA
Bc_ctg1_3703	B3	0.67645419	Bcer	E	Bcer	FALSE	NA	NA	NA
Bc_ctg1_3704	B3	4.622436967	Bcer	S	Bcer	FALSE	NA	NA	NA
Bc_ctg1_3705	B3	0.563711825	Bcer	S	Bcer	FALSE	NA	NA	NA
Bc_ctg1_3706	B3	1.240166016	Bcer	S	Bcer	FALSE	NA	NA	NA
Bc_ctg1_3707	B3	28.41107599	Bcer	T	Bcer	FALSE	NA	NA	NA
Bc_ctg1_3708	B3	15.33296165	Bcer	K	Bcer	FALSE	NA	NA	NA
Bc_ctg1_3709	B3	904.4192524	Bcer	E	Bcer	FALSE	NA	NA	NA
Bc_ctg1_371	B3	4.735179332	Bcer	G	Bcer	FALSE	Bc_Ga0417192_01_rn04	Ancillary biosynthesis	SMCOG1101
Bc_ctg1_3710	B3	86.69887872	Bcer	S	Bcer	FALSE	NA	NA	NA
Bc_ctg1_3711	B3	0	Bcer	NA	Bcer	FALSE	NA	NA	NA
Bc_ctg1_3712	B3	103.7229758	Bcer	T	Bcer	FALSE	NA	NA	NA
Bc_ctg1_3713	B3	57.61134854	Bcer	F	Bcer	FALSE	NA	NA	NA
Bc_ctg1_3714	B3	54.79278942	Bcer	H	Bcer	FALSE	NA	NA	NA
Bc_ctg1_3715	B3	48.25373224	Bcer	H	Bcer	FALSE	NA	NA	NA
Bc_ctg1_3716	B3	44.75871893	Bcer	S	Bcer	FALSE	NA	NA	NA
Bc_ctg1_3717	B3	3.156786221	Bcer	S	Bcer	FALSE	NA	NA	NA
Bc_ctg1_3718	B3	0.112742365	Bcer	NA	Bcer	FALSE	NA	NA	NA
Bc_ctg1_3719	B3	36.19029918	Bcer	G	Bcer	FALSE	NA	NA	NA
Bc_ctg1_372	B3	1.803877841	Bcer	S	Bcer	FALSE	Bc_Ga0417192_01_rn04	Other	NA
Bc_ctg1_3720	B3	80.94901811	Bcer	K	Bcer	FALSE	NA	NA	NA
Bc_ctg1_3721	B3	4.284209872	Bcer	NA	Bcer	FALSE	NA	NA	NA
Bc_ctg1_3722	B3	17.36232422	Bcer	NA	Bcer	FALSE	NA	NA	NA
Bc_ctg1_3723	B3	71.47865944	Bcer	F	Bcer	FALSE	NA	NA	NA
Bc_ctg1_3724	B3	829.1073526	Bcer	F	Bcer	FALSE	NA	NA	NA
Bc_ctg1_3725	B3	140.9279563	Bcer	F	Bcer	FALSE	NA	NA	NA
Bc_ctg1_3726	B3	345.5553489	Bcer	L	Bcer	FALSE	NA	NA	NA
Bc_ctg1_3727	B3	120.747073	Bcer	F	Bcer	FALSE	NA	NA	NA
Bc_ctg1_3728	B3	6.313572443	Bcer	K	Bcer	FALSE	NA	NA	NA
Bc_ctg1_3729	B3	19.05345969	Bcer	V	Bcer	FALSE	NA	NA	NA
Bc_ctg1_373	B3	5.862602983	Bcer	L	Bcer	FALSE	Bc_Ga0417192_01_rn04	Other	NA
Bc_ctg1_3730	B3	11.16149414	Bcer	NA	Bcer	FALSE	NA	NA	NA
Bc_ctg1_3731	B3	53.89085049	Bcer	V	Bcer	FALSE	NA	NA	NA
Bc_ctg1_3732	B3	79.14514027	Bcer	S	Bcer	FALSE	NA	NA	NA
Bc_ctg1_3733	B3	36.41578391	Bcer	S	Bcer	FALSE	NA	NA	NA
Bc_ctg1_3734	B3	0	Bcer	NA	Bcer	FALSE	NA	NA	NA
Bc_ctg1_3735	B3	5.073406427	Bcer	K	Bcer	FALSE	NA	NA	NA
Bc_ctg1_3736	B3	2.029362571	Bcer	S	Bcer	FALSE	NA	NA	NA

Bc_ctg1_3737	B3	3.720498047	Bcer	Q	Bcer	TRUE	NA	NA	NA
Bc_ctg1_3738	B3	42.72935635	Bcer	S	Bcer	FALSE	NA	NA	NA
Bc_ctg1_3739	B3	7.328253728	Bcer	V	Bcer	FALSE	NA	NA	NA
Bc_ctg1_374	B3	0.338227095	Bcer	NA	Bcer	FALSE	Bc_Ga0417192_01_rn04	Other	NA
Bc_ctg1_3740	B3	8.681162109	Bcer	S	Bcer	FALSE	NA	NA	NA
Bc_ctg1_3741	B3	250.2880504	Bcer	M	Bcer	FALSE	NA	NA	NA
Bc_ctg1_3742	B3	80.04707919	Bcer	IQ	Bcer	TRUE	NA	NA	NA
Bc_ctg1_3743	B3	543.869169	Bcer	M	Bcer	FALSE	NA	NA	NA
Bc_ctg1_3744	B3	829.6710644	Bcer	Q	Bcer	TRUE	NA	NA	NA
Bc_ctg1_3745	B3	2.142104936	Bcer	NA	Bcer	FALSE	NA	NA	NA
Bc_ctg1_3746	B3	1.803877841	Bcer	E	Bcer	FALSE	NA	NA	NA
Bc_ctg1_3747	B3	0.563711825	Bcer	NA	Bcer	FALSE	NA	NA	NA
Bc_ctg1_3748	B3	4.735179332	Bcer	C	Bcer	FALSE	NA	NA	NA
Bc_ctg1_3749	B3	14.09279563	Bcer	NA	Bcer	FALSE	NA	NA	NA
Bc_ctg1_375	B3	6.313572443	Bcer	G	Bcer	FALSE	Bc_Ga0417192_01_rn04	Core biosynthesis	SMCOG1257
Bc_ctg1_3750	B3	0.22548473	Bcer	NA	Bcer	FALSE	NA	NA	NA
Bc_ctg1_3751	B3	3.382270952	Bcer	S	Bcer	FALSE	NA	NA	NA
Bc_ctg1_3752	B3	1.465650746	Bcer	NA	Bcer	FALSE	NA	NA	NA
Bc_ctg1_3754	B3	1.127423651	Bcer	NA	Bcer	FALSE	NA	NA	NA
Bc_ctg1_3755	B3	10.71052468	Bcer	V	Bcer	FALSE	NA	NA	NA
Bc_ctg1_3756	B3	242.8470543	Bcer	V	Bcer	FALSE	NA	NA	NA
Bc_ctg1_3757	B3	66.74348011	Bcer	CO	Bcer	FALSE	NA	NA	NA
Bc_ctg1_3758	B3	5.073406427	Bcer	S	Bcer	FALSE	NA	NA	NA
Bc_ctg1_3759	B3	22.09750355	Bcer	K	Bcer	FALSE	NA	NA	NA
Bc_ctg1_376	B3	13.41634144	Bcer	Q	Bcer	TRUE	Bc_Ga0417192_01_rn04	Other	NA
Bc_ctg1_3761	B3	9.244873934	Bcer	C	Bcer	FALSE	NA	NA	NA
Bc_ctg1_3762	B3	272.1600692	Bcer	P	Bcer	FALSE	NA	NA	NA
Bc_ctg1_3763	B3	0.45096946	Bcer	NA	Bcer	FALSE	NA	NA	NA
Bc_ctg1_3764	B3	0.563711825	Bcer	S	Bcer	FALSE	NA	NA	NA
Bc_ctg1_3766	B3	0.563711825	Bcer	S	Bcer	FALSE	NA	NA	NA
Bc_ctg1_3767	B3	3.044043856	Bcer	S	Bcer	FALSE	NA	NA	NA
Bc_ctg1_3768	B3	12.96537198	Bcer	E	Bcer	FALSE	NA	NA	NA
Bc_ctg1_3769	B3	13.8673109	Bcer	E	Bcer	FALSE	NA	NA	NA
Bc_ctg1_377	B3	106.4287926	Bcer	E	Bcer	FALSE	Bc_Ga0417192_01_rn04	Other	NA
Bc_ctg1_3770	B3	2.029362571	Bcer	E	Bcer	FALSE	NA	NA	NA
Bc_ctg1_3771	B3	5.073406427	Bcer	EH	Bcer	FALSE	NA	NA	NA
Bc_ctg1_3772	B3	17.47506658	Bcer	E	Bcer	FALSE	NA	NA	NA
Bc_ctg1_3773	B3	1.803877841	Bcer	CE	Bcer	FALSE	NA	NA	NA
Bc_ctg1_3774	B3	3.382270952	Bcer	E	Bcer	FALSE	NA	NA	NA
Bc_ctg1_3775	B3	3.044043856	Bcer	E	Bcer	FALSE	NA	NA	NA
Bc_ctg1_3776	B3	7.891965554	Bcer	E	Bcer	FALSE	NA	NA	NA
Bc_ctg1_3777	B3	4.622436967	Bcer	E	Bcer	FALSE	NA	NA	NA
Bc_ctg1_3778	B3	8.004707919	Bcer	E	Bcer	FALSE	NA	NA	NA
Bc_ctg1_3779	B3	1.803877841	Bcer	E	Bcer	FALSE	NA	NA	NA
Bc_ctg1_378	B3	0.112742365	Bcer	E	Bcer	FALSE	Bc_Ga0417192_01_rn04	Other	NA
Bc_ctg1_3780	B3	1.014681285	Bcer	E	Bcer	FALSE	NA	NA	NA
Bc_ctg1_3781	B3	3.044043856	Bcer	E	Bcer	FALSE	NA	NA	NA
Bc_ctg1_3782	B3	2.705816761	Bcer	E	Bcer	FALSE	NA	NA	NA
Bc_ctg1_3783	B3	0.563711825	Bcer	E	Bcer	FALSE	NA	NA	NA
Bc_ctg1_3784	B3	0.22548473	Bcer	E	Bcer	FALSE	NA	NA	NA
Bc_ctg1_3785	B3	6.539057173	Bcer	E	Bcer	FALSE	NA	NA	NA
Bc_ctg1_3786	B3	1.127423651	Bcer	NA	Bcer	FALSE	NA	NA	NA
Bc_ctg1_3787	B3	107.4434739	Bcer	CH	Bcer	FALSE	NA	NA	NA
Bc_ctg1_3788	B3	95.83101029	Bcer	I	Bcer	FALSE	NA	NA	NA
Bc_ctg1_3789	B3	165.0548224	Bcer	M	Bcer	FALSE	NA	NA	NA
Bc_ctg1_379	B3	4.284209872	Bcer	K	Bcer	FALSE	Bc_Ga0417192_01_rn04	Regulation	SMCOG1171
Bc_ctg1_3790	B3	0	Bcer	S	Bcer	FALSE	NA	NA	NA
Bc_ctg1_3791	B3	64.03766335	Bcer	E	Bcer	FALSE	NA	NA	NA
Bc_ctg1_3792	B3	0	Bcer	S	Bcer	FALSE	NA	NA	NA
Bc_ctg1_3793	B3	22.21024592	Bcer	EH	Bcer	FALSE	NA	NA	NA
Bc_ctg1_3794	B3	71.59140181	Bcer	P	Bcer	FALSE	NA	NA	NA
Bc_ctg1_3795	B3	53.10165394	Bcer	P	Bcer	FALSE	NA	NA	NA

Bc_ctg1_3796	B3	198.877532	Bcer	P	Bcer	FALSE	NA	NA	NA
Bc_ctg1_3797	B3	18.15152077	Bcer	S	Bcer	FALSE	NA	NA	NA
Bc_ctg1_3798	B3	12.51440252	Bcer	H	Bcer	FALSE	NA	NA	NA
Bc_ctg1_3799	B3	23.2249272	Bcer	S	Bcer	FALSE	NA	NA	NA
Bc_ctg1_38	B3	0.112742365	Bcer	S	Bcer	FALSE	NA	NA	NA
Bc_ctg1_380	B3	2.367589666	Bcer	E	Bcer	FALSE	Bc_Ga0417192_01_rn04	Other	NA
Bc_ctg1_3800	B3	21.75927646	Bcer	H	Bcer	FALSE	NA	NA	NA
Bc_ctg1_3801	B3	25.70525923	Bcer	S	Bcer	FALSE	NA	NA	NA
Bc_ctg1_3802	B3	0.112742365	Bcer	NA	Bcer	FALSE	NA	NA	NA
Bc_ctg1_3803	B3	252.6556401	Bcer	D	Bcer	FALSE	NA	NA	NA
Bc_ctg1_3804	B3	794.6081889	Bcer	C	Bcer	FALSE	NA	NA	NA
Bc_ctg1_3805	B3	3.495013317	Bcer	S	Bcer	FALSE	NA	NA	NA
Bc_ctg1_3806	B3	41.15096324	Bcer	C	Bcer	FALSE	NA	NA	NA
Bc_ctg1_3807	B3	54.56730469	Bcer	NA	Bcer	FALSE	NA	NA	NA
Bc_ctg1_3808	B3	202.9362571	Bcer	KT	Bcer	FALSE	NA	NA	NA
Bc_ctg1_3809	B3	27.17090998	Bcer	S	Bcer	FALSE	NA	NA	NA
Bc_ctg1_381	B3	0	Bcer	S	Bcer	FALSE	Bc_Ga0417192_01_rn04	Other	NA
Bc_ctg1_3810	B3	54.34181995	Bcer	T	Bcer	FALSE	NA	NA	NA
Bc_ctg1_3811	B3	49.49389826	Bcer	KT	Bcer	FALSE	NA	NA	NA
Bc_ctg1_3812	B3	13.30359908	Bcer	NA	Bcer	FALSE	NA	NA	NA
Bc_ctg1_3813	B3	11.9506907	Bcer	E	Bcer	FALSE	NA	NA	NA
Bc_ctg1_3814	B3	48.93018643	Bcer	NA	Bcer	FALSE	NA	NA	NA
Bc_ctg1_3815	B3	17.36232422	Bcer	Q	Bcer	TRUE	NA	NA	NA
Bc_ctg1_3816	B3	13.75456854	Bcer	NA	Bcer	FALSE	NA	NA	NA
Bc_ctg1_3818	B3	8.793904474	Bcer	S	Bcer	FALSE	NA	NA	NA
Bc_ctg1_3819	B3	2.593074396	Bcer	NA	Bcer	FALSE	NA	NA	NA
Bc_ctg1_382	B3	0	Bcer	E	Bcer	FALSE	Bc_Ga0417192_01_rn04	Other	NA
Bc_ctg1_3820	B3	437.7786035	Bcer	C	Bcer	FALSE	NA	NA	NA
Bc_ctg1_3821	B3	13.64182617	Bcer	T	Bcer	FALSE	NA	NA	NA
Bc_ctg1_3822	B3	2.254847301	Bcer	NA	Bcer	FALSE	NA	NA	NA
Bc_ctg1_3823	B3	149.158149	Bcer	S	Bcer	FALSE	NA	NA	NA
Bc_ctg1_3824	B3	585.1328746	Bcer	P	Bcer	FALSE	NA	NA	NA
Bc_ctg1_3825	B3	295.9487083	Bcer	P	Bcer	FALSE	NA	NA	NA
Bc_ctg1_3826	B3	21.98476119	Bcer	M	Bcer	FALSE	NA	NA	NA
Bc_ctg1_3827	B3	0	Bcer	NA	Bcer	FALSE	NA	NA	NA
Bc_ctg1_3828	B3	0.112742365	Bcer	NA	Bcer	FALSE	NA	NA	NA
Bc_ctg1_3829	B3	38.44514648	Bcer	T	Bcer	FALSE	NA	NA	NA
Bc_ctg1_383	B3	93.68890536	Bcer	V	Bcer	FALSE	Bc_Ga0417192_01_rn04	Ancillary biosynthesis	SMCOG1053
Bc_ctg1_3830	B3	4.847921697	Bcer	S	Bcer	FALSE	NA	NA	NA
Bc_ctg1_3831	B3	18.94071733	Bcer	S	Bcer	FALSE	NA	NA	NA
Bc_ctg1_3832	B3	673.6356312	Bcer	L	Bcer	FALSE	NA	NA	NA
Bc_ctg1_3833	B3	200.9068945	Bcer	T	Bcer	FALSE	NA	NA	NA
Bc_ctg1_3834	B3	310.7179581	Bcer	F	Bcer	FALSE	NA	NA	NA
Bc_ctg1_3835	B3	21.75927646	Bcer	S	Bcer	FALSE	NA	NA	NA
Bc_ctg1_3836	B3	219.5093848	Bcer	S	Bcer	FALSE	NA	NA	NA
Bc_ctg1_3837	B3	3.945982777	Bcer	NA	Bcer	FALSE	NA	NA	NA
Bc_ctg1_3838	B3	10.37229758	Bcer	S	Bcer	FALSE	NA	NA	NA
Bc_ctg1_3839	B3	2.029362571	Bcer	P	Bcer	FALSE	NA	NA	NA
Bc_ctg1_384	B3	1.803877841	Bcer	NA	Bcer	FALSE	NA	NA	NA
Bc_ctg1_3840	B3	0.45096946	Bcer	M	Bcer	FALSE	NA	NA	NA
Bc_ctg1_3841	B3	0.789196555	Bcer	S	Bcer	FALSE	NA	NA	NA
Bc_ctg1_3842	B3	0.45096946	Bcer	S	Bcer	FALSE	NA	NA	NA
Bc_ctg1_3843	B3	151.1875115	Bcer	J	Bcer	FALSE	NA	NA	NA
Bc_ctg1_3844	B3	32.69528587	Bcer	CO	Bcer	FALSE	NA	NA	NA
Bc_ctg1_3845	B3	43.96952237	Bcer	O	Bcer	FALSE	NA	NA	NA
Bc_ctg1_3846	B3	67.30719194	Bcer	O	Bcer	FALSE	NA	NA	NA
Bc_ctg1_3847	B3	157.2755992	Bcer	T	Bcer	FALSE	NA	NA	NA
Bc_ctg1_3848	B3	330.3351296	Bcer	T	Bcer	FALSE	NA	NA	NA
Bc_ctg1_3849	B3	4.058725142	Bcer	S	Bcer	FALSE	NA	NA	NA
Bc_ctg1_385	B3	3.044043856	Bcer	G	Bcer	FALSE	NA	NA	NA
Bc_ctg1_3850	B3	2.254847301	Bcer	M	Bcer	FALSE	NA	NA	NA
Bc_ctg1_3851	B3	104.6249148	Bcer	M	Bcer	FALSE	NA	NA	NA

Bc_ctg1_3852	B3	103.8357182	Bcer	U	Bcer	FALSE	NA	NA	NA
Bc_ctg1_3853	B3	100.0024778	Bcer	C	Bcer	FALSE	NA	NA	NA
Bc_ctg1_3854	B3	29.42575728	Bcer	S	Bcer	FALSE	NA	NA	NA
Bc_ctg1_3855	B3	52.65068448	Bcer	L	Bcer	FALSE	NA	NA	NA
Bc_ctg1_3856	B3	56.03295543	Bcer	S	Bcer	FALSE	NA	NA	NA
Bc_ctg1_3857	B3	44.64597656	Bcer	M	Bcer	FALSE	NA	NA	NA
Bc_ctg1_3858	B3	129.6537198	Bcer	S	Bcer	FALSE	NA	NA	NA
Bc_ctg1_3859	B3	188.1670073	Bcer	K	Bcer	FALSE	NA	NA	NA
Bc_ctg1_386	B3	9.695843394	Bcer	K	Bcer	FALSE	NA	NA	NA
Bc_ctg1_3860	B3	161.1088397	Bcer	NOT	Bcer	FALSE	NA	NA	NA
Bc_ctg1_3861	B3	2181.001052	Bcer	E	Bcer	FALSE	NA	NA	NA
Bc_ctg1_3862	B3	0	Bcer	NA	Bcer	FALSE	NA	NA	NA
Bc_ctg1_3863	B3	2.705816761	Bcer	S	Bcer	FALSE	NA	NA	NA
Bc_ctg1_3864	B3	6.764541903	Bcer	M	Bcer	FALSE	NA	NA	NA
Bc_ctg1_3865	B3	17.13683949	Bcer	O	Bcer	FALSE	NA	NA	NA
Bc_ctg1_3866	B3	148.7071795	Bcer	EJ	Bcer	FALSE	NA	NA	NA
Bc_ctg1_3867	B3	0	Bcer	S	Bcer	FALSE	NA	NA	NA
Bc_ctg1_3868	B3	67.7581614	Bcer	F	Bcer	FALSE	NA	NA	NA
Bc_ctg1_3869	B3	1377.373474	Bcer	J	Bcer	FALSE	NA	NA	NA
Bc_ctg1_387	B3	3.495013317	Bcer	S	Bcer	FALSE	NA	NA	NA
Bc_ctg1_3870	B3	44.75871893	Bcer	C	Bcer	FALSE	NA	NA	NA
Bc_ctg1_3872	B3	0	Bcer	NA	Bcer	FALSE	NA	NA	NA
Bc_ctg1_3873	B3	0.112742365	Bcer	S	Bcer	FALSE	NA	NA	NA
Bc_ctg1_3875	B3	645.3372976	Bcer	S	Bcer	FALSE	NA	NA	NA
Bc_ctg1_3876	B3	597.8727619	Bcer	I	Bcer	FALSE	NA	NA	NA
Bc_ctg1_3877	B3	0.90193892	Bcer	S	Bcer	FALSE	NA	NA	NA
Bc_ctg1_3878	B3	0.90193892	Bcer	S	Bcer	FALSE	NA	NA	NA
Bc_ctg1_3879	B3	6.088087713	Bcer	NA	Bcer	FALSE	NA	NA	NA
Bc_ctg1_388	B3	71.70414417	Bcer	C	Bcer	FALSE	NA	NA	NA
Bc_ctg1_3880	B3	10.03407049	Bcer	S	Bcer	FALSE	NA	NA	NA
Bc_ctg1_3881	B3	899.0076189	Bcer	L	Bcer	FALSE	NA	NA	NA
Bc_ctg1_3882	B3	191.774763	Bcer	H	Bcer	FALSE	NA	NA	NA
Bc_ctg1_3883	B3	66.74348011	Bcer	H	Bcer	FALSE	NA	NA	NA
Bc_ctg1_3884	B3	80.27256392	Bcer	H	Bcer	FALSE	NA	NA	NA
Bc_ctg1_3885	B3	110.0365483	Bcer	H	Bcer	FALSE	NA	NA	NA
Bc_ctg1_3886	B3	1492.596171	Bcer	F	Bcer	FALSE	NA	NA	NA
Bc_ctg1_3887	B3	69.6747816	Bcer	E	Bcer	FALSE	NA	NA	NA
Bc_ctg1_3888	B3	174.6379235	Bcer	E	Bcer	FALSE	NA	NA	NA
Bc_ctg1_3889	B3	349.0503622	Bcer	E	Bcer	FALSE	NA	NA	NA
Bc_ctg1_389	B3	10.82326704	Bcer	NA	Bcer	FALSE	NA	NA	NA
Bc_ctg1_3890	B3	139.2368208	Bcer	S	Bcer	FALSE	NA	NA	NA
Bc_ctg1_3891	B3	106.0905655	Bcer	S	Bcer	FALSE	Bc_Ga0417192_01_rn27	Other	NA
Bc_ctg1_3892	B3	12.73988725	Bcer	S	Bcer	FALSE	Bc_Ga0417192_01_rn27	Other	NA
Bc_ctg1_3893	B3	26.49445579	Bcer	C	Bcer	FALSE	Bc_Ga0417192_01_rn27	Other	NA
Bc_ctg1_3894	B3	48.36647461	Bcer	C	Bcer	FALSE	Bc_Ga0417192_01_rn27	Other	NA
Bc_ctg1_3895	B3	26.26897106	Bcer	C	Bcer	FALSE	Bc_Ga0417192_01_rn27	Other	NA
Bc_ctg1_3896	B3	21.08282226	Bcer	S	Bcer	FALSE	Bc_Ga0417192_01_rn27	Other	NA
Bc_ctg1_3897	B3	17.81329368	Bcer	S	Bcer	FALSE	Bc_Ga0417192_01_rn27	Other	NA
Bc_ctg1_3898	B3	6.313572443	Bcer	S	Bcer	FALSE	Bc_Ga0417192_01_rn27	Other	NA
Bc_ctg1_3899	B3	315.7913645	Bcer	S	Bcer	FALSE	Bc_Ga0417192_01_rn27	Other	NA
Bc_ctg1_39	B3	2.367589666	Bcer	J	Bcer	FALSE	NA	NA	NA
Bc_ctg1_390	B3	2.931301491	Bcer	NA	Bcer	FALSE	NA	NA	NA
Bc_ctg1_3900	B3	1.240166016	Bcer	L	Bcer	FALSE	Bc_Ga0417192_01_rn27	Other	NA
Bc_ctg1_3901	B3	24.69057795	Bcer	S	Bcer	FALSE	Bc_Ga0417192_01_rn27	Other	NA
Bc_ctg1_3902	B3	2.593074396	Bcer	S	Bcer	FALSE	Bc_Ga0417192_01_rn27	Other	NA
Bc_ctg1_3903	B3	118.4922257	Bcer	E	Bcer	FALSE	Bc_Ga0417192_01_rn27	Other	NA
Bc_ctg1_3904	B3	63.02298206	Bcer	G	Bcer	FALSE	Bc_Ga0417192_01_rn27	Other	NA
Bc_ctg1_3905	B3	76.32658114	Bcer	S	Bcer	FALSE	Bc_Ga0417192_01_rn27	Core biosynthesis	None
Bc_ctg1_3906	B3	145.8886204	Bcer	M	Bcer	FALSE	Bc_Ga0417192_01_rn27	Core biosynthesis	SMCOG1045
Bc_ctg1_3907	B3	201.4706063	Bcer	J	Bcer	FALSE	Bc_Ga0417192_01_rn27	Other	NA
Bc_ctg1_3908	B3	159.0794771	Bcer	K	Bcer	FALSE	Bc_Ga0417192_01_rn27	Other	NA
Bc_ctg1_3909	B3	0	Bcer	C	Bcer	FALSE	Bc_Ga0417192_01_rn27	Other	NA

Bc_ctg1_391	B3	3.945982777	Bcer	S	Bcer	FALSE	NA	NA	NA
Bc_ctg1_3910	B3	81.06176047	Bcer	H	Bcer	FALSE	Bc_Ga0417192_01_rn27	Other	NA
Bc_ctg1_3911	B3	158.4030229	Bcer	H	Bcer	FALSE	Bc_Ga0417192_01_rn27	Other	NA
Bc_ctg1_3912	B3	19.95539861	Bcer	H	Bcer	FALSE	Bc_Ga0417192_01_rn27	Other	NA
Bc_ctg1_3913	B3	311.7326394	Bcer	L	Bcer	FALSE	Bc_Ga0417192_01_rn27	Other	SMCOG1194
Bc_ctg1_3914	B3	35.96481445	Bcer	S	Bcer	FALSE	Bc_Ga0417192_01_rn27	Other	NA
Bc_ctg1_3915	B3	83.65483487	Bcer	S	Bcer	FALSE	Bc_Ga0417192_01_rn27	Other	NA
Bc_ctg1_3916	B3	919.6394717	Bcer	E	Bcer	FALSE	Bc_Ga0417192_01_rn27	Core biosynthesis	SMCOG1019
Bc_ctg1_3917	B3	64.37589044	Bcer	L	Bcer	FALSE	Bc_Ga0417192_01_rn27	Other	NA
Bc_ctg1_3918	B3	57.83683327	Bcer	L	Bcer	FALSE	Bc_Ga0417192_01_rn27	Other	NA
Bc_ctg1_3919	B3	19.50442915	Bcer	NA	Bcer	FALSE	Bc_Ga0417192_01_rn27	Other	NA
Bc_ctg1_392	B3	36.97949574	Bcer	NA	Bcer	FALSE	NA	NA	NA
Bc_ctg1_3920	B3	389.0739018	Bcer	M	Bcer	FALSE	Bc_Ga0417192_01_rn27	Other	NA
Bc_ctg1_3921	B3	25.02880504	Bcer	L	Bcer	FALSE	Bc_Ga0417192_01_rn27	Other	NA
Bc_ctg1_3922	B3	3.607755682	Bcer	S	Bcer	FALSE	Bc_Ga0417192_01_rn27	Other	NA
Bc_ctg1_3924	B3	2.480332031	Bcer	NA	Bcer	FALSE	Bc_Ga0417192_01_rn27	Other	NA
Bc_ctg1_3925	B3	3.495013317	Bcer	S	Bcer	FALSE	Bc_Ga0417192_01_rn27	Other	NA
Bc_ctg1_3926	B3	13.41634144	Bcer	S	Bcer	FALSE	Bc_Ga0417192_01_rn27	Other	NA
Bc_ctg1_3927	B3	1.240166016	Bcer	NA	Bcer	FALSE	Bc_Ga0417192_01_rn27	Other	NA
Bc_ctg1_3928	B3	0.22548473	Bcer	S	Bcer	FALSE	Bc_Ga0417192_01_rn27	Other	NA
Bc_ctg1_3929	B3	5.524375888	Bcer	S	Bcer	FALSE	Bc_Ga0417192_01_rn27	Other	NA
Bc_ctg1_393	B3	76.89029297	Bcer	S	Bcer	FALSE	NA	NA	NA
Bc_ctg1_3930	B3	293.9193457	Bcer	D	Bcer	FALSE	NA	NA	NA
Bc_ctg1_3931	B3	128.9772656	Bcer	L	Bcer	FALSE	NA	NA	NA
Bc_ctg1_3932	B3	11.04875178	Bcer	S	Bcer	FALSE	NA	NA	NA
Bc_ctg1_3933	B3	117.7030291	Bcer	KL	Bcer	FALSE	NA	NA	NA
Bc_ctg1_3934	B3	443.9794336	Bcer	E	Bcer	FALSE	NA	NA	NA
Bc_ctg1_3935	B3	0.338227095	Bcer	L	Bcer	FALSE	NA	NA	NA
Bc_ctg1_3937	B3	1.352908381	Bcer	J	Bcer	FALSE	NA	NA	NA
Bc_ctg1_3938	B3	9.244873934	Bcer	E	Bcer	FALSE	NA	NA	NA
Bc_ctg1_3939	B3	101.919098	Bcer	F	Bcer	FALSE	NA	NA	NA
Bc_ctg1_394	B3	45.09694602	Bcer	NA	Bcer	FALSE	NA	NA	NA
Bc_ctg1_3940	B3	601.2550328	Bcer	F	Bcer	FALSE	NA	NA	NA
Bc_ctg1_3941	B3	0	Bcer	NA	Bcer	FALSE	NA	NA	NA
Bc_ctg1_3943	B3	8.004707919	Bcer	P	Bcer	FALSE	NA	NA	NA
Bc_ctg1_3945	B3	274.865886	Bcer	S	Bcer	FALSE	NA	NA	NA
Bc_ctg1_3946	B3	0	Bcer	S	Bcer	FALSE	NA	NA	NA
Bc_ctg1_3947	B3	34.38642134	Bcer	K	Bcer	FALSE	NA	NA	NA
Bc_ctg1_3948	B3	41.6019327	Bcer	S	Bcer	FALSE	Bc_Ga0417192_01_rn28	Other	NA
Bc_ctg1_3949	B3	226.2739267	Bcer	P	Bcer	FALSE	Bc_Ga0417192_01_rn28	Other	NA
Bc_ctg1_395	B3	2.593074396	Bcer	CH	Bcer	FALSE	NA	NA	NA
Bc_ctg1_3950	B3	8.004707919	Bcer	NA	Bcer	FALSE	Bc_Ga0417192_01_rn28	Other	NA
Bc_ctg1_3951	B3	11.6124636	Bcer	NA	Bcer	FALSE	Bc_Ga0417192_01_rn28	Other	NA
Bc_ctg1_3952	B3	34.04819425	Bcer	E	Bcer	FALSE	Bc_Ga0417192_01_rn28	Other	NA
Bc_ctg1_3953	B3	3.720498047	Bcer	S	Bcer	FALSE	Bc_Ga0417192_01_rn28	Other	NA
Bc_ctg1_3954	B3	287.2675462	Bcer	P	Bcer	FALSE	Bc_Ga0417192_01_rn28	Other	NA
Bc_ctg1_3955	B3	98.53682706	Bcer	L	Bcer	FALSE	Bc_Ga0417192_01_rn28	Other	NA
Bc_ctg1_3956	B3	0	Bcer	NA	Bcer	FALSE	Bc_Ga0417192_01_rn28	Other	NA
Bc_ctg1_3957	B3	1.803877841	Bcer	I	Bcer	FALSE	Bc_Ga0417192_01_rn28	Ancillary biosynthesis	SMCOG1088
Bc_ctg1_3958	B3	1.014681285	Bcer	M	Bcer	FALSE	Bc_Ga0417192_01_rn28	Ancillary biosynthesis	SMCOG1216
Bc_ctg1_3959	B3	0.789196555	Bcer	D	Bcer	FALSE	Bc_Ga0417192_01_rn28	Other	NA
Bc_ctg1_396	B3	0.90193892	Bcer	K	Bcer	FALSE	NA	NA	NA
Bc_ctg1_3960	B3	0.22548473	Bcer	M	Bcer	FALSE	Bc_Ga0417192_01_rn28	Core biosynthesis	SMCOG1146
Bc_ctg1_3961	B3	4.622436967	Bcer	S	Bcer	FALSE	Bc_Ga0417192_01_rn28	Core biosynthesis	None
Bc_ctg1_3962	B3	1.803877841	Bcer	M	Bcer	FALSE	Bc_Ga0417192_01_rn28	Core biosynthesis	None
Bc_ctg1_3963	B3	0.67645419	Bcer	M	Bcer	FALSE	Bc_Ga0417192_01_rn28	Core biosynthesis	None
Bc_ctg1_3964	B3	0.67645419	Bcer	M	Bcer	FALSE	Bc_Ga0417192_01_rn28	Core biosynthesis	SMCOG1045
Bc_ctg1_3965	B3	0.789196555	Bcer	M	Bcer	FALSE	Bc_Ga0417192_01_rn28	Other	NA
Bc_ctg1_3966	B3	8.230192649	Bcer	M	Bcer	FALSE	NA	NA	NA
Bc_ctg1_3967	B3	0.789196555	Bcer	K	Bcer	FALSE	NA	NA	NA
Bc_ctg1_3968	B3	26.71994052	Bcer	Q	Bcer	TRUE	NA	NA	NA
Bc_ctg1_3969	B3	6.313572443	Bcer	NA	Bcer	FALSE	NA	NA	NA

Bc_ctg1_397	B3	6.651799538	Bcer	C	Bcer	FALSE	NA	NA	NA
Bc_ctg1_3970	B3	15.33296165	Bcer	EG	Bcer	FALSE	NA	NA	NA
Bc_ctg1_3971	B3	5.298891157	Bcer	L	Bcer	FALSE	NA	NA	NA
Bc_ctg1_3972	B3	59.86619584	Bcer	G	Bcer	FALSE	NA	NA	NA
Bc_ctg1_3973	B3	3.269528587	Bcer	S	Bcer	FALSE	NA	NA	NA
Bc_ctg1_3974	B3	4.847921697	Bcer	L	Bcer	FALSE	NA	NA	NA
Bc_ctg1_3975	B3	0.563711825	Bcer	S	Bcer	FALSE	NA	NA	NA
Bc_ctg1_3976	B3	0.789196555	Bcer	S	Bcer	FALSE	NA	NA	NA
Bc_ctg1_3977	B3	6.877284268	Bcer	K	Bcer	FALSE	NA	NA	NA
Bc_ctg1_3978	B3	1.240166016	Bcer	S	Bcer	FALSE	NA	NA	NA
Bc_ctg1_3979	B3	19.72991388	Bcer	E	Bcer	FALSE	NA	NA	NA
Bc_ctg1_398	B3	102.9337793	Bcer	S	Bcer	FALSE	NA	NA	NA
Bc_ctg1_3980	B3	2.705816761	Bcer	NA	Bcer	FALSE	NA	NA	NA
Bc_ctg1_3981	B3	7.553738458	Bcer	I	Bcer	FALSE	NA	NA	NA
Bc_ctg1_3982	B3	0.563711825	Bcer	S	Bcer	FALSE	NA	NA	NA
Bc_ctg1_3983	B3	1.803877841	Bcer	S	Bcer	FALSE	NA	NA	NA
Bc_ctg1_3984	B3	73.39527965	Bcer	E	Bcer	FALSE	NA	NA	NA
Bc_ctg1_3985	B3	0	Bcer	S	Bcer	FALSE	NA	NA	NA
Bc_ctg1_3986	B3	62.79749733	Bcer	P	Bcer	FALSE	NA	NA	NA
Bc_ctg1_3987	B3	9.80858576	Bcer	KT	Bcer	FALSE	NA	NA	NA
Bc_ctg1_3988	B3	44.1950071	Bcer	V	Bcer	FALSE	NA	NA	NA
Bc_ctg1_3989	B3	0.45096946	Bcer	S	Bcer	FALSE	NA	NA	NA
Bc_ctg1_399	B3	11.38697887	Bcer	S	Bcer	FALSE	NA	NA	NA
Bc_ctg1_3990	B3	17.58780895	Bcer	S	Bcer	FALSE	NA	NA	NA
Bc_ctg1_3991	B3	1.578393111	Bcer	S	Bcer	FALSE	NA	NA	NA
Bc_ctg1_3992	B3	33.14625533	Bcer	C	Bcer	FALSE	NA	NA	NA
Bc_ctg1_3993	B3	4.171467507	Bcer	K	Bcer	FALSE	NA	NA	NA
Bc_ctg1_3994	B3	4.171467507	Bcer	NA	Bcer	FALSE	NA	NA	NA
Bc_ctg1_3995	B3	6.877284268	Bcer	S	Bcer	FALSE	NA	NA	NA
Bc_ctg1_3996	B3	4.622436967	Bcer	G	Bcer	FALSE	NA	NA	NA
Bc_ctg1_3997	B3	1.465650746	Bcer	K	Bcer	FALSE	NA	NA	NA
Bc_ctg1_3998	B3	1.014681285	Bcer	EGP	Bcer	FALSE	NA	NA	NA
Bc_ctg1_3999	B3	0.22548473	Bcer	E	Bcer	FALSE	NA	NA	NA
Bc_ctg1_4	B3	1.014681285	Bcer	F	Bcer	FALSE	NA	NA	NA
Bc_ctg1_40	B3	0	Bcer	L	Bcer	FALSE	NA	NA	NA
Bc_ctg1_400	B3	7.440996093	Bcer	NA	Bcer	FALSE	NA	NA	NA
Bc_ctg1_4001	B3	0	Bcer	NA	Bcer	FALSE	NA	NA	NA
Bc_ctg1_4002	B3	2.029362571	Bcer	S	Bcer	FALSE	NA	NA	NA
Bc_ctg1_4003	B3	37.88143466	Bcer	S	Bcer	FALSE	NA	NA	NA
Bc_ctg1_4004	B3	51.2977761	Bcer	N	Bcer	FALSE	NA	NA	NA
Bc_ctg1_4005	B3	65.39057173	Bcer	N	Bcer	FALSE	NA	NA	NA
Bc_ctg1_4006	B3	71.36591708	Bcer	T	Bcer	FALSE	NA	NA	NA
Bc_ctg1_4007	B3	671.8317533	Bcer	NT	Bcer	FALSE	NA	NA	NA
Bc_ctg1_4008	B3	148.1434677	Bcer	N	Bcer	FALSE	NA	NA	NA
Bc_ctg1_4009	B3	46.7880815	Bcer	NA	Bcer	FALSE	NA	NA	NA
Bc_ctg1_401	B3	10.14681285	Bcer	K	Bcer	FALSE	NA	NA	NA
Bc_ctg1_4010	B3	83.31660777	Bcer	NA	Bcer	FALSE	NA	NA	NA
Bc_ctg1_4011	B3	37.65594993	Bcer	NT	Bcer	FALSE	NA	NA	NA
Bc_ctg1_4012	B3	33.70996715	Bcer	NA	Bcer	FALSE	NA	NA	NA
Bc_ctg1_4013	B3	46.7880815	Bcer	S	Bcer	FALSE	NA	NA	NA
Bc_ctg1_4014	B3	39.91079723	Bcer	NA	Bcer	FALSE	NA	NA	NA
Bc_ctg1_4015	B3	355.476677	Bcer	N	Bcer	FALSE	NA	NA	NA
Bc_ctg1_4016	B3	246.45481	Bcer	N	Bcer	FALSE	NA	NA	NA
Bc_ctg1_4017	B3	508.4680664	Bcer	N	Bcer	FALSE	NA	NA	NA
Bc_ctg1_4018	B3	183.9955398	Bcer	N	Bcer	FALSE	NA	NA	NA
Bc_ctg1_4019	B3	117.1393173	Bcer	NA	Bcer	FALSE	NA	NA	NA
Bc_ctg1_402	B3	1.916620206	Bcer	S	Bcer	FALSE	NA	NA	NA
Bc_ctg1_4020	B3	68.09638849	Bcer	N	Bcer	FALSE	NA	NA	NA
Bc_ctg1_4021	B3	57.04763672	Bcer	N	Bcer	FALSE	NA	NA	NA
Bc_ctg1_4022	B3	32.80802823	Bcer	N	Bcer	FALSE	NA	NA	NA
Bc_ctg1_4023	B3	295.2722541	Bcer	N	Bcer	FALSE	NA	NA	NA
Bc_ctg1_4024	B3	261.6750293	Bcer	N	Bcer	FALSE	NA	NA	NA

Bc_ctg1_4025	B3	54.34181995	Bcer	N	Bcer	FALSE	NA	NA	NA
Bc_ctg1_4026	B3	202.59803	Bcer	NU	Bcer	FALSE	NA	NA	NA
Bc_ctg1_4027	B3	71.59140181	Bcer	NA	Bcer	FALSE	NA	NA	NA
Bc_ctg1_4028	B3	172.1575914	Bcer	N	Bcer	FALSE	NA	NA	NA
Bc_ctg1_4029	B3	158.0647958	Bcer	N	Bcer	FALSE	NA	NA	NA
Bc_ctg1_403	B3	1.352908381	Bcer	S	Bcer	FALSE	NA	NA	NA
Bc_ctg1_4030	B3	423.122096	Bcer	N	Bcer	FALSE	NA	NA	NA
Bc_ctg1_4031	B3	91.99776988	Bcer	S	Bcer	FALSE	NA	NA	NA
Bc_ctg1_4032	B3	203.3872266	Bcer	NA	Bcer	FALSE	NA	NA	NA
Bc_ctg1_4033	B3	556.9472834	Bcer	T	Bcer	FALSE	NA	NA	NA
Bc_ctg1_4034	B3	42.39112926	Bcer	K	Bcer	FALSE	NA	NA	NA
Bc_ctg1_4035	B3	53.43988103	Bcer	N	Bcer	FALSE	NA	NA	NA
Bc_ctg1_4036	B3	15392.82784	Bcer	N	Bcer	FALSE	NA	NA	NA
Bc_ctg1_4037	B3	84.10580433	Bcer	M	Bcer	FALSE	NA	NA	NA
Bc_ctg1_4038	B3	3.945982777	Bcer	N	Bcer	FALSE	NA	NA	NA
Bc_ctg1_4039	B3	128.4135538	Bcer	N	Bcer	FALSE	NA	NA	NA
Bc_ctg1_404	B3	2.142104936	Bcer	C	Bcer	FALSE	NA	NA	NA
Bc_ctg1_4040	B3	52.87616921	Bcer	N	Bcer	FALSE	NA	NA	NA
Bc_ctg1_4041	B3	20.51911044	Bcer	N	Bcer	FALSE	NA	NA	NA
Bc_ctg1_4042	B3	69.6747816	Bcer	N	Bcer	FALSE	NA	NA	NA
Bc_ctg1_4043	B3	12.28891779	Bcer	N	Bcer	FALSE	NA	NA	NA
Bc_ctg1_4044	B3	67.64541903	Bcer	N	Bcer	FALSE	NA	NA	NA
Bc_ctg1_4045	B3	107.1052468	Bcer	N	Bcer	FALSE	NA	NA	NA
Bc_ctg1_4046	B3	207.5586941	Bcer	N	Bcer	FALSE	NA	NA	NA
Bc_ctg1_4047	B3	257.1653347	Bcer	N	Bcer	FALSE	NA	NA	NA
Bc_ctg1_4048	B3	177.3437402	Bcer	N	Bcer	FALSE	NA	NA	NA
Bc_ctg1_4049	B3	44.75871893	Bcer	C	Bcer	FALSE	NA	NA	NA
Bc_ctg1_405	B3	8.455677379	Bcer	S	Bcer	FALSE	NA	NA	NA
Bc_ctg1_4050	B3	2.705816761	Bcer	S	Bcer	FALSE	NA	NA	NA
Bc_ctg1_4051	B3	11.38697887	Bcer	K	Bcer	FALSE	NA	NA	NA
Bc_ctg1_4052	B3	9.583101029	Bcer	E	Bcer	FALSE	NA	NA	NA
Bc_ctg1_4053	B3	4.960664062	Bcer	S	Bcer	FALSE	NA	NA	NA
Bc_ctg1_4054	B3	0.67645419	Bcer	NA	Bcer	FALSE	NA	NA	NA
Bc_ctg1_4055	B3	1.691135476	Bcer	NA	Bcer	FALSE	NA	NA	NA
Bc_ctg1_4056	B3	4.847921697	Bcer	Q	Bcer	TRUE	NA	NA	NA
Bc_ctg1_4057	B3	1.916620206	Bcer	S	Bcer	FALSE	NA	NA	NA
Bc_ctg1_4058	B3	0.22548473	Bcer	S	Bcer	FALSE	NA	NA	NA
Bc_ctg1_4059	B3	15.33296165	Bcer	K	Bcer	FALSE	NA	NA	NA
Bc_ctg1_406	B3	24.80332031	Bcer	J	Bcer	FALSE	NA	NA	NA
Bc_ctg1_4060	B3	1.014681285	Bcer	EGP	Bcer	FALSE	NA	NA	NA
Bc_ctg1_4061	B3	0.112742365	Bcer	S	Bcer	FALSE	NA	NA	NA
Bc_ctg1_4062	B3	1.240166016	Bcer	K	Bcer	FALSE	NA	NA	NA
Bc_ctg1_4063	B3	0.112742365	Bcer	P	Bcer	FALSE	NA	NA	NA
Bc_ctg1_4064	B3	0.112742365	Bcer	P	Bcer	FALSE	NA	NA	NA
Bc_ctg1_4065	B3	1.578393111	Bcer	P	Bcer	FALSE	NA	NA	NA
Bc_ctg1_4066	B3	0.90193892	Bcer	J	Bcer	FALSE	NA	NA	NA
Bc_ctg1_4067	B3	0.563711825	Bcer	NA	Bcer	FALSE	NA	NA	NA
Bc_ctg1_4068	B3	220.7495508	Bcer	V	Bcer	FALSE	NA	NA	NA
Bc_ctg1_4069	B3	64.60137517	Bcer	S	Bcer	FALSE	NA	NA	NA
Bc_ctg1_407	B3	0	Bcer	NA	Bcer	FALSE	NA	NA	NA
Bc_ctg1_4070	B3	10.37229758	Bcer	NA	Bcer	FALSE	NA	NA	NA
Bc_ctg1_4071	B3	1.127423651	Bcer	V	Bcer	FALSE	NA	NA	NA
Bc_ctg1_4072	B3	0.90193892	Bcer	G	Bcer	FALSE	NA	NA	NA
Bc_ctg1_4073	B3	1438.930805	Bcer	NA	Bcer	FALSE	NA	NA	NA
Bc_ctg1_4074	B3	2.367589666	Bcer	S	Bcer	FALSE	NA	NA	NA
Bc_ctg1_4075	B3	0.45096946	Bcer	NA	Bcer	FALSE	NA	NA	NA
Bc_ctg1_4076	B3	0.22548473	Bcer	K	Bcer	FALSE	NA	NA	NA
Bc_ctg1_4077	B3	13.98005327	Bcer	T	Bcer	FALSE	NA	NA	NA
Bc_ctg1_4078	B3	52.08697265	Bcer	E	Bcer	FALSE	NA	NA	NA
Bc_ctg1_4079	B3	0.112742365	Bcer	K	Bcer	FALSE	NA	NA	NA
Bc_ctg1_408	B3	0	Bcer	I	Bcer	FALSE	NA	NA	NA
Bc_ctg1_4080	B3	0.112742365	Bcer	S	Bcer	FALSE	NA	NA	NA

Bc_ctg1_4081	B3	0.338227095	Bcer	S	Bcer	FALSE	NA	NA	NA	
Bc_ctg1_4082	B3	49.0429288	Bcer	P	Bcer	FALSE	NA	NA	NA	
Bc_ctg1_4083	B3	1.240166016	Bcer	S	Bcer	FALSE	NA	NA	NA	
Bc_ctg1_4084	B3	3.044043856	Bcer	K	Bcer	FALSE	NA	NA	NA	
Bc_ctg1_4085	B3	3.945982777	Bcer	EG	Bcer	FALSE	NA	NA	NA	
Bc_ctg1_4086	B3	0.112742365	Bcer	NA	Bcer	FALSE	NA	NA	NA	
Bc_ctg1_4087	B3	1.578393111	Bcer	S	Bcer	FALSE	NA	NA	NA	
Bc_ctg1_4088	B3	0	Bcer	S	Bcer	FALSE	NA	NA	NA	
Bc_ctg1_4089	B3	0.45096946	Bcer	S	Bcer	FALSE	NA	NA	NA	
Bc_ctg1_409	B3	0.90193892	Bcer	NA	Bcer	FALSE	NA	NA	NA	
Bc_ctg1_4090	B3	1.127423651	Bcer	M	Bcer	FALSE	NA	NA	NA	
Bc_ctg1_4091	B3	56.48392489	Bcer	C	Bcer	FALSE	NA	NA	NA	
Bc_ctg1_4092	B3	4.735179332	Bcer	K	Bcer	FALSE	NA	NA	NA	
Bc_ctg1_4093	B3	0.22548473	Bcer	IQ	Bcer	TRUE	NA	NA	NA	
Bc_ctg1_4094	B3	429.4356685	Bcer	C	Bcer	FALSE	NA	NA	NA	
Bc_ctg1_4095	B3	20.74459517	Bcer	S	Bcer	FALSE	NA	NA	NA	
Bc_ctg1_4096	B3	13.19085671	Bcer	K	Bcer	FALSE	NA	NA	NA	
Bc_ctg1_4097	B3	25.8180016	Bcer	V	Bcer	FALSE	NA	NA	NA	
Bc_ctg1_4098	B3	17.70055131	Bcer	E	Bcer	FALSE	NA	NA	NA	
Bc_ctg1_4099	B3	3.269528587	Bcer	NA	Bcer	FALSE	NA	NA	NA	
Bc_ctg1_41	B3	0	Bcer	L	Bcer	FALSE	NA	NA	NA	
Bc_ctg1_410	B3	10.14681285	Bcer	P	Bcer	FALSE	NA	NA	NA	
Bc_ctg1_4100	B3	13.8673109	Bcer	K	Bcer	FALSE	NA	NA	NA	
Bc_ctg1_4101	B3	7.553738458	Bcer	O	Bcer	FALSE	NA	NA	NA	
Bc_ctg1_4102	B3	1.014681285	Bcer	O	Bcer	FALSE	NA	NA	NA	
Bc_ctg1_4103	B3	2.593074396	Bcer	CO	Bcer	FALSE	NA	NA	NA	
Bc_ctg1_4104	B3	2.029362571	Bcer	M	Bcer	FALSE	NA	NA	NA	
Bc_ctg1_4105	B3	0	Bcer	NA	Bcer	FALSE	NA	NA	NA	
Bc_ctg1_4106	B3	40.13628196	Bcer	K	Bcer	FALSE	NA	NA	NA	
Bc_ctg1_4107	B3	12.17617543	Bcer	K	Bcer	FALSE	NA	NA	NA	
Bc_ctg1_4108	B3	10.03407049	Bcer	E	Bcer	FALSE	NA	NA	NA	
Bc_ctg1_4109	B3	50.62132191	Bcer	S	Bcer	FALSE	NA	NA	NA	
Bc_ctg1_411	B3	3.945982777	Bcer	S	Bcer	FALSE	NA	NA	NA	
Bc_ctg1_4110	B3	12.85262962	Bcer	NA	Bcer	FALSE	NA	NA	NA	
Bc_ctg1_4111	B3	4.284209872	Bcer	NA	Bcer	FALSE	NA	NA	NA	
Bc_ctg1_4112	B3	1.352908381	Bcer	NA	Bcer	FALSE	NA	NA	NA	
Bc_ctg1_4113	B3	3.044043856	Bcer	K	Bcer	FALSE	NA	NA	NA	
Bc_ctg1_4114	B3	2.480332031	Bcer	K	Bcer	FALSE	NA	NA	NA	
Bc_ctg1_4115	B3	4.622436967	Bcer	T	Bcer	FALSE	NA	NA	NA	
Bc_ctg1_4116	B3	38.67063121	Bcer	V	Bcer	FALSE	NA	NA	NA	
Bc_ctg1_4117	B3	41.48919034	Bcer	V	Bcer	FALSE	NA	NA	NA	
Bc_ctg1_4118	B3	63.81217862	Bcer	V	Bcer	FALSE	NA	NA	NA	
Bc_ctg1_4119	B3	0	Bcer	S	Bcer	FALSE	NA	NA	NA	
Bc_ctg1_412	B3	0.67645419	Bcer	NA	Bcer	FALSE	NA	NA	NA	
Bc_ctg1_4120	B3	1.127423651	Bcer	I	Bcer	FALSE	NA	NA	NA	
Bc_ctg1_4121	B3	28.0728489	Bcer	F	Bcer	FALSE	NA	NA	NA	
Bc_ctg1_4122	B3	300.3456605	Bcer	C	Bcer	FALSE	NA	NA	NA	
Bc_ctg1_4123	B3	449.6165518	Bcer	E	Bcer	FALSE	NA	NA	NA	
Bc_ctg1_4124	B3	444.0921759	Bcer	C	Bcer	FALSE	NA	NA	NA	
Bc_ctg1_4125	B3	18.3770055	Bcer	T	Bcer	FALSE	NA	NA	NA	
Bc_ctg1_4126	B3	11.72520597	Bcer	T	Bcer	FALSE	NA	NA	NA	
Bc_ctg1_4127	B3	72.60608309	Bcer	L	Bcer	FALSE	NA	NA	NA	
Bc_ctg1_4128	B3	0	Bcer	NA	Bcer	FALSE	NA	NA	NA	
Bc_ctg1_413	B3	1.127423651	Bcer	U	Bcer	FALSE	NA	NA	NA	
Bc_ctg1_4130	B3	76.10109641	Bcer	F	Bcer	FALSE	NA	NA	NA	
Bc_ctg1_4131	B3	0.338227095	Bcer	K	Bcer	FALSE	NA	NA	NA	
Bc_ctg1_4132	B3	10.59778231	Bcer	E	Bcer	FALSE	NA	NA	NA	
Bc_ctg1_4133	B3	17.81329368	Bcer	E	Bcer	FALSE	NA	NA	NA	
Bc_ctg1_4134	B3	9.132131569	Bcer	S	Bcer	FALSE	NA	NA	NA	
Bc_ctg1_4135	B3	5.524375888	Bcer	S	Bcer	FALSE	Bc_Ga0417192_01_rn29	Other	NA	
Bc_ctg1_4136	B3	15.33296165	Bcer	S	Bcer	FALSE	Bc_Ga0417192_01_rn29	Other	NA	
Bc_ctg1_4137	B3	0	Bcer	NA	Bcer	FALSE	Bc_Ga0417192_01_rn29	Other	NA	

Bc_ctg1_4138	B3	29.08753018	Bcer	S	Bcer	FALSE	Bc_Ga0417192_01_rn29	Other	NA
Bc_ctg1_4139	B3	17.36232422	Bcer	K	Bcer	FALSE	Bc_Ga0417192_01_rn29	Regulation	SMCOG1278
Bc_ctg1_414	B3	13.07811435	Bcer	S	Bcer	FALSE	NA	NA	NA
Bc_ctg1_4140	B3	135.7418075	Bcer	EGP	Bcer	FALSE	Bc_Ga0417192_01_rn29	Transport	SMCOG1005
Bc_ctg1_4141	B3	8.004707919	Bcer	S	Bcer	FALSE	Bc_Ga0417192_01_rn29	Other	NA
Bc_ctg1_4142	B3	13.07811435	Bcer	EGP	Bcer	FALSE	Bc_Ga0417192_01_rn29	Transport	SMCOG1202
Bc_ctg1_4143	B3	4.847921697	Bcer	I	Bcer	FALSE	Bc_Ga0417192_01_rn29	Core biosynthesis	SMCOG1084
Bc_ctg1_4144	B3	67.08170721	Bcer	S	Bcer	FALSE	Bc_Ga0417192_01_rn29	Other	NA
Bc_ctg1_4145	B3	12.28891779	Bcer	P	Bcer	FALSE	Bc_Ga0417192_01_rn29	Transport	SMCOG1106
Bc_ctg1_4146	B3	72.49334073	Bcer	E	Bcer	FALSE	Bc_Ga0417192_01_rn29	Ancillary biosynthesis	SMCOG1081
Bc_ctg1_4147	B3	9.132131569	Bcer	S	Bcer	FALSE	Bc_Ga0417192_01_rn29	Other	NA
Bc_ctg1_4148	B3	14.99473455	Bcer	S	Bcer	FALSE	Bc_Ga0417192_01_rn29	Other	NA
Bc_ctg1_4149	B3	17.81329368	Bcer	NA	Bcer	FALSE	Bc_Ga0417192_01_rn29	Other	NA
Bc_ctg1_415	B3	21.30830699	Bcer	S	Bcer	FALSE	NA	NA	NA
Bc_ctg1_4150	B3	4.735179332	Bcer	S	Bcer	FALSE	Bc_Ga0417192_01_rn29	Other	NA
Bc_ctg1_4151	B3	8.004707919	Bcer	G	Bcer	FALSE	Bc_Ga0417192_01_rn29	Ancillary biosynthesis	SMCOG1235
Bc_ctg1_4152	B3	6.539057173	Bcer	S	Bcer	FALSE	Bc_Ga0417192_01_rn29	Other	NA
Bc_ctg1_4153	B3	2.931301491	Bcer	S	Bcer	FALSE	NA	NA	NA
Bc_ctg1_4154	B3	42.27838689	Bcer	S	Bcer	FALSE	NA	NA	NA
Bc_ctg1_4155	B3	0.22548473	Bcer	DZ	Bcer	FALSE	NA	NA	NA
Bc_ctg1_4156	B3	0.112742365	Bcer	NA	Bcer	FALSE	NA	NA	NA
Bc_ctg1_4158	B3	30.77866566	Bcer	O	Bcer	FALSE	NA	NA	NA
Bc_ctg1_4159	B3	0.67645419	Bcer	IQ	Bcer	TRUE	NA	NA	NA
Bc_ctg1_416	B3	0.563711825	Bcer	P	Bcer	FALSE	Bc_Ga0417192_01_rn05	Other	NA
Bc_ctg1_4160	B3	0.112742365	Bcer	E	Bcer	FALSE	NA	NA	NA
Bc_ctg1_4161	B3	1.803877841	Bcer	E	Bcer	FALSE	NA	NA	NA
Bc_ctg1_4162	B3	0.338227095	Bcer	E	Bcer	FALSE	NA	NA	NA
Bc_ctg1_4163	B3	0.563711825	Bcer	EH	Bcer	FALSE	NA	NA	NA
Bc_ctg1_4164	B3	4.284209872	Bcer	E	Bcer	FALSE	NA	NA	NA
Bc_ctg1_4165	B3	9.695843394	Bcer	E	Bcer	FALSE	NA	NA	NA
Bc_ctg1_4166	B3	0	Bcer	NA	Bcer	FALSE	NA	NA	NA
Bc_ctg1_4167	B3	0.90193892	Bcer	M	Bcer	FALSE	NA	NA	NA
Bc_ctg1_4168	B3	0.112742365	Bcer	S	Bcer	FALSE	NA	NA	NA
Bc_ctg1_4169	B3	0.45096946	Bcer	K	Bcer	FALSE	NA	NA	NA
Bc_ctg1_417	B3	0.338227095	Bcer	E	Bcer	FALSE	Bc_Ga0417192_01_rn05	Other	NA
Bc_ctg1_4170	B3	16.68587003	Bcer	EGP	Bcer	FALSE	NA	NA	NA
Bc_ctg1_4171	B3	10.14681285	Bcer	S	Bcer	FALSE	NA	NA	NA
Bc_ctg1_4172	B3	49.49389826	Bcer	I	Bcer	FALSE	NA	NA	NA
Bc_ctg1_4173	B3	34.04819425	Bcer	K	Bcer	FALSE	NA	NA	NA
Bc_ctg1_4174	B3	422.8966113	Bcer	C	Bcer	FALSE	NA	NA	NA
Bc_ctg1_4175	B3	1.352908381	Bcer	K	Bcer	FALSE	NA	NA	NA
Bc_ctg1_4176	B3	0.22548473	Bcer	S	Bcer	FALSE	NA	NA	NA
Bc_ctg1_4177	B3	206.0930433	Bcer	F	Bcer	FALSE	NA	NA	NA
Bc_ctg1_4178	B3	2.480332031	Bcer	EGP	Bcer	FALSE	NA	NA	NA
Bc_ctg1_4179	B3	3.833240412	Bcer	K	Bcer	FALSE	NA	NA	NA
Bc_ctg1_418	B3	4.058725142	Bcer	S	Bcer	FALSE	Bc_Ga0417192_01_rn05	Other	NA
Bc_ctg1_4180	B3	66.17976829	Bcer	E	Bcer	FALSE	NA	NA	NA
Bc_ctg1_4181	B3	4.509694602	Bcer	J	Bcer	FALSE	NA	NA	NA
Bc_ctg1_4182	B3	1.352908381	Bcer	T	Bcer	FALSE	NA	NA	NA
Bc_ctg1_4183	B3	1.127423651	Bcer	T	Bcer	FALSE	NA	NA	NA
Bc_ctg1_4184	B3	1.916620206	Bcer	M	Bcer	FALSE	NA	NA	NA
Bc_ctg1_4185	B3	168.4370934	Bcer	P	Bcer	FALSE	NA	NA	NA
Bc_ctg1_4186	B3	50.73406427	Bcer	S	Bcer	FALSE	NA	NA	NA
Bc_ctg1_4187	B3	0.67645419	Bcer	P	Bcer	FALSE	NA	NA	NA
Bc_ctg1_4188	B3	133.712445	Bcer	H	Bcer	FALSE	NA	NA	NA
Bc_ctg1_419	B3	0.112742365	Bcer	S	Bcer	FALSE	Bc_Ga0417192_01_rn05	Other	NA
Bc_ctg1_4190	B3	126.7224183	Bcer	E	Bcer	FALSE	NA	NA	NA
Bc_ctg1_4191	B3	1601.73078	Bcer	O	Bcer	FALSE	NA	NA	NA
Bc_ctg1_4192	B3	2572.104316	Bcer	E	Bcer	FALSE	NA	NA	NA
Bc_ctg1_4193	B3	310.9434428	Bcer	E	Bcer	FALSE	NA	NA	NA
Bc_ctg1_4194	B3	18.60249023	Bcer	O	Bcer	FALSE	NA	NA	NA
Bc_ctg1_4195	B3	9.357616299	Bcer	E	Bcer	FALSE	NA	NA	NA

Bc_ctg1_4196	B3	12.96537198	Bcer	S	Bcer	FALSE	NA	NA	NA
Bc_ctg1_4197	B3	6.764541903	Bcer	K	Bcer	FALSE	NA	NA	NA
Bc_ctg1_4198	B3	10.93600941	Bcer	K	Bcer	FALSE	NA	NA	NA
Bc_ctg1_4199	B3	0	Bcer	S	Bcer	FALSE	NA	NA	NA
Bc_ctg1_42	B3	32.5825435	Bcer	S	Bcer	FALSE	NA	NA	NA
Bc_ctg1_420	B3	0.112742365	Bcer	JM	Bcer	FALSE	Bc_Ga0417192_01_rn05	Ancillary biosynthesis	SMCOG1064
Bc_ctg1_4200	B3	0.112742365	Bcer	S	Bcer	FALSE	NA	NA	NA
Bc_ctg1_4201	B3	12.06343306	Bcer	K	Bcer	FALSE	NA	NA	NA
Bc_ctg1_4202	B3	26.15622869	Bcer	K	Bcer	FALSE	NA	NA	NA
Bc_ctg1_4203	B3	83.76757723	Bcer	F	Bcer	FALSE	NA	NA	NA
Bc_ctg1_4204	B3	85.90968217	Bcer	F	Bcer	FALSE	NA	NA	NA
Bc_ctg1_4205	B3	101.4681285	Bcer	F	Bcer	FALSE	NA	NA	NA
Bc_ctg1_4206	B3	19.50442915	Bcer	F	Bcer	FALSE	NA	NA	NA
Bc_ctg1_4207	B3	7.440996093	Bcer	O	Bcer	FALSE	NA	NA	NA
Bc_ctg1_4208	B3	7.553738458	Bcer	S	Bcer	FALSE	NA	NA	NA
Bc_ctg1_4209	B3	3.833240412	Bcer	NA	Bcer	FALSE	NA	NA	NA
Bc_ctg1_421	B3	0	Bcer	M	Bcer	FALSE	Bc_Ga0417192_01_rn05	Other	NA
Bc_ctg1_4210	B3	45.32243075	Bcer	O	Bcer	FALSE	NA	NA	NA
Bc_ctg1_4211	B3	58.62602983	Bcer	S	Bcer	FALSE	NA	NA	NA
Bc_ctg1_4212	B3	103.2720064	Bcer	E	Bcer	FALSE	NA	NA	NA
Bc_ctg1_4213	B3	141.6044105	Bcer	Q	Bcer	TRUE	NA	NA	NA
Bc_ctg1_4214	B3	0	Bcer	O	Bcer	FALSE	NA	NA	NA
Bc_ctg1_4215	B3	12.40166016	Bcer	MT	Bcer	FALSE	NA	NA	NA
Bc_ctg1_4216	B3	0.338227095	Bcer	NA	Bcer	FALSE	NA	NA	NA
Bc_ctg1_4217	B3	63.81217862	Bcer	L	Bcer	FALSE	NA	NA	NA
Bc_ctg1_4218	B3	4.622436967	Bcer	S	Bcer	FALSE	NA	NA	NA
Bc_ctg1_4219	B3	26.04348633	Bcer	P	Bcer	FALSE	NA	NA	NA
Bc_ctg1_422	B3	0	Bcer	GM	Bcer	FALSE	Bc_Ga0417192_01_rn05	Core biosynthesis	SMCOG1010
Bc_ctg1_4220	B3	3.382270952	Bcer	I	Bcer	FALSE	NA	NA	NA
Bc_ctg1_4221	B3	258.6309854	Bcer	E	Bcer	FALSE	NA	NA	NA
Bc_ctg1_4222	B3	7.666480824	Bcer	E	Bcer	FALSE	NA	NA	NA
Bc_ctg1_4223	B3	4.960664062	Bcer	P	Bcer	FALSE	NA	NA	NA
Bc_ctg1_4224	B3	2.367589666	Bcer	S	Bcer	FALSE	NA	NA	NA
Bc_ctg1_4225	B3	14.205538	Bcer	EG	Bcer	FALSE	NA	NA	NA
Bc_ctg1_4226	B3	3.607755682	Bcer	K	Bcer	FALSE	NA	NA	NA
Bc_ctg1_4227	B3	1.127423651	Bcer	K	Bcer	FALSE	NA	NA	NA
Bc_ctg1_4228	B3	2.593074396	Bcer	Q	Bcer	TRUE	NA	NA	NA
Bc_ctg1_4229	B3	3.156786221	Bcer	S	Bcer	FALSE	NA	NA	NA
Bc_ctg1_423	B3	1.240166016	Bcer	GM	Bcer	FALSE	Bc_Ga0417192_01_rn05	Core biosynthesis	SMCOG1010
Bc_ctg1_4231	B3	2.254847301	Bcer	J	Bcer	FALSE	NA	NA	NA
Bc_ctg1_4232	B3	8.004707919	Bcer	S	Bcer	FALSE	NA	NA	NA
Bc_ctg1_4233	B3	24.12686612	Bcer	C	Bcer	FALSE	NA	NA	NA
Bc_ctg1_4234	B3	0	Bcer	E	Bcer	FALSE	NA	NA	NA
Bc_ctg1_4235	B3	12.51440252	Bcer	S	Bcer	FALSE	NA	NA	NA
Bc_ctg1_4236	B3	5.637118253	Bcer	E	Bcer	FALSE	NA	NA	NA
Bc_ctg1_4237	B3	1.803877841	Bcer	E	Bcer	FALSE	NA	NA	NA
Bc_ctg1_4238	B3	0.338227095	Bcer	E	Bcer	FALSE	NA	NA	NA
Bc_ctg1_4239	B3	1.578393111	Bcer	E	Bcer	FALSE	NA	NA	NA
Bc_ctg1_424	B3	6.764541903	Bcer	K	Bcer	FALSE	Bc_Ga0417192_01_rn05	Other	NA
Bc_ctg1_4240	B3	2.480332031	Bcer	E	Bcer	FALSE	NA	NA	NA
Bc_ctg1_4241	B3	7.328253728	Bcer	K	Bcer	FALSE	NA	NA	NA
Bc_ctg1_4242	B3	1.691135476	Bcer	S	Bcer	FALSE	NA	NA	NA
Bc_ctg1_4243	B3	0.789196555	Bcer	S	Bcer	FALSE	Bc_Ga0417192_01_rn30	Other	NA
Bc_ctg1_4244	B3	0.112742365	Bcer	S	Bcer	FALSE	Bc_Ga0417192_01_rn30	Other	NA
Bc_ctg1_4245	B3	49.60664062	Bcer	K	Bcer	FALSE	Bc_Ga0417192_01_rn30	Regulation	SMCOG1135
Bc_ctg1_4246	B3	41.9401598	Bcer	C	Bcer	FALSE	Bc_Ga0417192_01_rn30	Other	SMCOG1251
Bc_ctg1_4247	B3	45.09694602	Bcer	C	Bcer	FALSE	Bc_Ga0417192_01_rn30	Other	NA
Bc_ctg1_4248	B3	25.25428977	Bcer	C	Bcer	FALSE	Bc_Ga0417192_01_rn30	Other	NA
Bc_ctg1_4249	B3	24.23960849	Bcer	V	Bcer	FALSE	Bc_Ga0417192_01_rn30	Transport	SMCOG1288
Bc_ctg1_425	B3	0	Bcer	NA	Bcer	FALSE	Bc_Ga0417192_01_rn05	Other	NA
Bc_ctg1_4250	B3	32.2443164	Bcer	V	Bcer	FALSE	Bc_Ga0417192_01_rn30	Transport	SMCOG1288
Bc_ctg1_4251	B3	0.22548473	Bcer	E	Bcer	FALSE	Bc_Ga0417192_01_rn30	Other	NA

Bc_ctg1_4252	B3	3.945982777	Bcer	MQ	Bcer	TRUE	Bc_Ga0417192_01_rn30	Core biosynthesis	None
Bc_ctg1_4253	B3	2.142104936	Bcer	E	Bcer	FALSE	Bc_Ga0417192_01_rn30	Core biosynthesis	SMCOG1056
Bc_ctg1_4254	B3	2.705816761	Bcer	S	Bcer	FALSE	Bc_Ga0417192_01_rn30	Ancillary biosynthesis	SMCOG1156
Bc_ctg1_4256	B3	2.593074396	Bcer	NA	Bcer	FALSE	Bc_Ga0417192_01_rn30	Other	NA
Bc_ctg1_4257	B3	22.43573065	Bcer	P	Bcer	FALSE	Bc_Ga0417192_01_rn30	Other	NA
Bc_ctg1_4258	B3	29.9894691	Bcer	EG	Bcer	FALSE	Bc_Ga0417192_01_rn30	Other	NA
Bc_ctg1_4259	B3	33.70996715	Bcer	V	Bcer	FALSE	Bc_Ga0417192_01_rn30	Other	NA
Bc_ctg1_426	B3	35.40110263	Bcer	NT	Bcer	FALSE	Bc_Ga0417192_01_rn05	Other	SMCOG1061
Bc_ctg1_4260	B3	33.70996715	Bcer	M	Bcer	FALSE	Bc_Ga0417192_01_rn30	Other	NA
Bc_ctg1_4261	B3	519.1785911	Bcer	S	Bcer	FALSE	Bc_Ga0417192_01_rn30	Ancillary biosynthesis	None
Bc_ctg1_4262	B3	1.014681285	Bcer	NA	Bcer	FALSE	Bc_Ga0417192_01_rn30	Other	NA
Bc_ctg1_4263	B3	2630.617604	Bcer	M	Bcer	FALSE	Bc_Ga0417192_01_rn30	Other	NA
Bc_ctg1_4264	B3	20.18088334	Bcer	S	Bcer	FALSE	Bc_Ga0417192_01_rn30	Ancillary biosynthesis	SMCOG1036
Bc_ctg1_4265	B3	17.58780895	Bcer	V	Bcer	FALSE	NA	NA	NA
Bc_ctg1_4266	B3	12.96537198	Bcer	T	Bcer	FALSE	NA	NA	NA
Bc_ctg1_4267	B3	9.921328125	Bcer	T	Bcer	FALSE	NA	NA	NA
Bc_ctg1_4268	B3	0.67645419	Bcer	NA	Bcer	FALSE	NA	NA	NA
Bc_ctg1_4269	B3	95.38004083	Bcer	S	Bcer	FALSE	NA	NA	NA
Bc_ctg1_427	B3	0.22548473	Bcer	NA	Bcer	FALSE	Bc_Ga0417192_01_rn05	Other	NA
Bc_ctg1_4270	B3	22.88670011	Bcer	S	Bcer	FALSE	NA	NA	NA
Bc_ctg1_4271	B3	70.0130087	Bcer	G	Bcer	FALSE	NA	NA	NA
Bc_ctg1_4272	B3	0.45096946	Bcer	G	Bcer	FALSE	NA	NA	NA
Bc_ctg1_4273	B3	2.705816761	Bcer	S	Bcer	FALSE	NA	NA	NA
Bc_ctg1_4274	B3	35.85207209	Bcer	I	Bcer	FALSE	NA	NA	NA
Bc_ctg1_4275	B3	2.593074396	Bcer	S	Bcer	FALSE	NA	NA	NA
Bc_ctg1_4276	B3	89.51743785	Bcer	E	Bcer	FALSE	NA	NA	NA
Bc_ctg1_4277	B3	112.2913956	Bcer	E	Bcer	FALSE	NA	NA	NA
Bc_ctg1_4278	B3	121.1980424	Bcer	E	Bcer	FALSE	NA	NA	NA
Bc_ctg1_4279	B3	0.563711825	Bcer	S	Bcer	FALSE	NA	NA	NA
Bc_ctg1_428	B3	1.578393111	Bcer	NA	Bcer	FALSE	Bc_Ga0417192_01_rn05	Other	NA
Bc_ctg1_4280	B3	4.058725142	Bcer	S	Bcer	FALSE	NA	NA	NA
Bc_ctg1_4281	B3	122.8891779	Bcer	K	Bcer	FALSE	NA	NA	NA
Bc_ctg1_4282	B3	8.117450284	Bcer	S	Bcer	FALSE	NA	NA	NA
Bc_ctg1_4283	B3	5.298891157	Bcer	T	Bcer	FALSE	Bc_Ga0417192_01_rn31	Other	NA
Bc_ctg1_4284	B3	12.40166016	Bcer	T	Bcer	FALSE	Bc_Ga0417192_01_rn31	Regulation	SMCOG1003
Bc_ctg1_4285	B3	165.9567614	Bcer	G	Bcer	FALSE	Bc_Ga0417192_01_rn31	Ancillary biosynthesis	SMCOG1235
Bc_ctg1_4286	B3	91.20857333	Bcer	L	Bcer	FALSE	Bc_Ga0417192_01_rn31	Other	NA
Bc_ctg1_4287	B3	12.62714489	Bcer	S	Bcer	FALSE	Bc_Ga0417192_01_rn31	Other	NA
Bc_ctg1_4288	B3	3.945982777	Bcer	M	Bcer	FALSE	Bc_Ga0417192_01_rn31	Other	NA
Bc_ctg1_4289	B3	30.3276962	Bcer	Q	Bcer	TRUE	Bc_Ga0417192_01_rn31	Core biosynthesis	None
Bc_ctg1_429	B3	0.112742365	Bcer	NA	Bcer	FALSE	Bc_Ga0417192_01_rn05	Other	NA
Bc_ctg1_4290	B3	28.29833363	Bcer	Q	Bcer	TRUE	Bc_Ga0417192_01_rn31	Core biosynthesis	None
Bc_ctg1_4291	B3	11.6124636	Bcer	IQ	Bcer	TRUE	Bc_Ga0417192_01_rn31	Ancillary biosynthesis	SMCOG1002
Bc_ctg1_4292	B3	1.240166016	Bcer	IQ	Bcer	TRUE	Bc_Ga0417192_01_rn31	Other	NA
Bc_ctg1_4293	B3	15.22021928	Bcer	IQ	Bcer	TRUE	Bc_Ga0417192_01_rn31	Other	NA
Bc_ctg1_4294	B3	13.07811435	Bcer	G	Bcer	FALSE	Bc_Ga0417192_01_rn31	Other	NA
Bc_ctg1_4295	B3	0	Bcer	S	Bcer	FALSE	Bc_Ga0417192_01_rn31	Other	NA
Bc_ctg1_4296	B3	0	Bcer	EGP	Bcer	FALSE	Bc_Ga0417192_01_rn31	Other	NA
Bc_ctg1_4297	B3	3.156786221	Bcer	S	Bcer	FALSE	NA	NA	NA
Bc_ctg1_4298	B3	39.23434304	Bcer	K	Bcer	FALSE	NA	NA	NA
Bc_ctg1_4299	B3	0.22548473	Bcer	S	Bcer	FALSE	NA	NA	NA
Bc_ctg1_43	B3	8.793904474	Bcer	K	Bcer	FALSE	NA	NA	NA
Bc_ctg1_430	B3	0.112742365	Bcer	Q	Bcer	TRUE	Bc_Ga0417192_01_rn05	Other	NA
Bc_ctg1_4300	B3	1.240166016	Bcer	S	Bcer	FALSE	NA	NA	NA
Bc_ctg1_4302	B3	44.1950071	Bcer	S	Bcer	FALSE	NA	NA	NA
Bc_ctg1_4303	B3	374.6428791	Bcer	D	Bcer	FALSE	NA	NA	NA
Bc_ctg1_4304	B3	2.029362571	Bcer	S	Bcer	FALSE	NA	NA	NA
Bc_ctg1_4305	B3	16.4603853	Bcer	F	Bcer	FALSE	NA	NA	NA
Bc_ctg1_4306	B3	93.57616299	Bcer	H	Bcer	FALSE	NA	NA	NA
Bc_ctg1_4307	B3	15.22021928	Bcer	V	Bcer	FALSE	NA	NA	NA
Bc_ctg1_4308	B3	26.60719815	Bcer	K	Bcer	FALSE	NA	NA	NA
Bc_ctg1_4309	B3	41.9401598	Bcer	O	Bcer	FALSE	NA	NA	NA

Bc_ctg1_431	B3	6.088087713	Bcer	S	Bcer	FALSE	NA	NA	NA
Bc_ctg1_4310	B3	5.862602983	Bcer	K	Bcer	FALSE	NA	NA	NA
Bc_ctg1_4311	B3	1.803877841	Bcer	C	Bcer	FALSE	NA	NA	NA
Bc_ctg1_4312	B3	1.127423651	Bcer	EGP	Bcer	FALSE	NA	NA	NA
Bc_ctg1_4313	B3	19.95539861	Bcer	NA	Bcer	FALSE	NA	NA	NA
Bc_ctg1_4314	B3	27.96010653	Bcer	S	Bcer	FALSE	NA	NA	NA
Bc_ctg1_4315	B3	3.833240412	Bcer	S	Bcer	FALSE	NA	NA	NA
Bc_ctg1_4316	B3	59.52796875	Bcer	S	Bcer	FALSE	NA	NA	NA
Bc_ctg1_4317	B3	32.2443164	Bcer	NT	Bcer	FALSE	NA	NA	NA
Bc_ctg1_4318	B3	15.55844638	Bcer	NA	Bcer	FALSE	NA	NA	NA
Bc_ctg1_4319	B3	8.906646839	Bcer	S	Bcer	FALSE	NA	NA	NA
Bc_ctg1_432	B3	2.367589666	Bcer	O	Bcer	FALSE	NA	NA	NA
Bc_ctg1_4320	B3	0	Bcer	S	Bcer	FALSE	NA	NA	NA
Bc_ctg1_4321	B3	153.3296165	Bcer	P	Bcer	FALSE	NA	NA	NA
Bc_ctg1_4322	B3	200.7941522	Bcer	S	Bcer	FALSE	NA	NA	NA
Bc_ctg1_4323	B3	1.803877841	Bcer	S	Bcer	FALSE	NA	NA	NA
Bc_ctg1_4326	B3	41.37644797	Bcer	C	Bcer	FALSE	NA	NA	NA
Bc_ctg1_4328	B3	1.916620206	Bcer	E	Bcer	FALSE	NA	NA	NA
Bc_ctg1_4329	B3	13.8673109	Bcer	J	Bcer	FALSE	NA	NA	NA
Bc_ctg1_433	B3	10.48503995	Bcer	G	Bcer	FALSE	NA	NA	NA
Bc_ctg1_4330	B3	290.1988476	Bcer	S	Bcer	FALSE	NA	NA	NA
Bc_ctg1_4331	B3	31.34237748	Bcer	S	Bcer	FALSE	NA	NA	NA
Bc_ctg1_4332	B3	3.833240412	Bcer	K	Bcer	FALSE	NA	NA	NA
Bc_ctg1_4333	B3	73.50802201	Bcer	P	Bcer	FALSE	NA	NA	NA
Bc_ctg1_4335	B3	29.53849964	Bcer	GM	Bcer	FALSE	NA	NA	NA
Bc_ctg1_4336	B3	16.4603853	Bcer	C	Bcer	FALSE	NA	NA	NA
Bc_ctg1_4337	B3	5.862602983	Bcer	L	Bcer	FALSE	NA	NA	NA
Bc_ctg1_4338	B3	28.63656072	Bcer	NU	Bcer	FALSE	NA	NA	NA
Bc_ctg1_4339	B3	6.539057173	Bcer	E	Bcer	FALSE	NA	NA	NA
Bc_ctg1_4340	B3	10.03407049	Bcer	H	Bcer	FALSE	NA	NA	NA
Bc_ctg1_4341	B3	3.156786221	Bcer	S	Bcer	FALSE	NA	NA	NA
Bc_ctg1_4342	B3	10.59778231	Bcer	G	Bcer	FALSE	NA	NA	NA
Bc_ctg1_4343	B3	0.90193892	Bcer	NA	Bcer	FALSE	NA	NA	NA
Bc_ctg1_4344	B3	9.695843394	Bcer	M	Bcer	FALSE	NA	NA	NA
Bc_ctg1_4345	B3	15.22021928	Bcer	F	Bcer	FALSE	NA	NA	NA
Bc_ctg1_4346	B3	45.54791548	Bcer	S	Bcer	FALSE	NA	NA	NA
Bc_ctg1_4347	B3	8.004707919	Bcer	S	Bcer	FALSE	NA	NA	NA
Bc_ctg1_4348	B3	77.90497425	Bcer	P	Bcer	FALSE	NA	NA	NA
Bc_ctg1_4349	B3	34.8373908	Bcer	S	Bcer	FALSE	NA	NA	NA
Bc_ctg1_435	B3	7.553738458	Bcer	T	Bcer	FALSE	NA	NA	NA
Bc_ctg1_4350	B3	48.25373224	Bcer	S	Bcer	FALSE	NA	NA	NA
Bc_ctg1_4351	B3	3.495013317	Bcer	I	Bcer	FALSE	NA	NA	NA
Bc_ctg1_4352	B3	0	Bcer	I	Bcer	FALSE	NA	NA	NA
Bc_ctg1_4353	B3	0	Bcer	M	Bcer	FALSE	NA	NA	NA
Bc_ctg1_4355	B3	1.352908381	Bcer	GM	Bcer	FALSE	NA	NA	NA
Bc_ctg1_4356	B3	12.73988725	Bcer	S	Bcer	FALSE	NA	NA	NA
Bc_ctg1_4357	B3	57.38586381	Bcer	S	Bcer	FALSE	NA	NA	NA
Bc_ctg1_4358	B3	0.112742365	Bcer	NA	Bcer	FALSE	NA	NA	NA
Bc_ctg1_4359	B3	1.352908381	Bcer	K	Bcer	FALSE	NA	NA	NA
Bc_ctg1_436	B3	19.39168679	Bcer	S	Bcer	FALSE	NA	NA	NA
Bc_ctg1_4360	B3	1.803877841	Bcer	S	Bcer	FALSE	NA	NA	NA
Bc_ctg1_4361	B3	304.8553551	Bcer	S	Bcer	FALSE	NA	NA	NA
Bc_ctg1_4362	B3	0	Bcer	L	Bcer	FALSE	NA	NA	NA
Bc_ctg1_4364	B3	104.9631419	Bcer	E	Bcer	FALSE	NA	NA	NA
Bc_ctg1_4365	B3	0	Bcer	NA	Bcer	FALSE	NA	NA	NA
Bc_ctg1_4367	B3	6.764541903	Bcer	J	Bcer	FALSE	NA	NA	NA
Bc_ctg1_4368	B3	4.847921697	Bcer	S	Bcer	FALSE	NA	NA	NA
Bc_ctg1_437	B3	9.132131569	Bcer	K	Bcer	FALSE	NA	NA	NA
Bc_ctg1_4370	B3	12.40166016	Bcer	M	Bcer	FALSE	NA	NA	NA
Bc_ctg1_4371	B3	2.029362571	Bcer	J	Bcer	FALSE	NA	NA	NA
Bc_ctg1_4372	B3	11.49972124	Bcer	M	Bcer	FALSE	NA	NA	NA
Bc_ctg1_4373	B3	3.720498047	Bcer	F	Bcer	FALSE	NA	NA	NA

Bc_ctg1_4374	B3	25.36703214	Bcer	J	Bcer	FALSE	NA	NA	NA
Bc_ctg1_4375	B3	7.891965554	Bcer	G	Bcer	FALSE	NA	NA	NA
Bc_ctg1_4376	B3	127.849842	Bcer	OU	Bcer	FALSE	NA	NA	NA
Bc_ctg1_4377	B3	419.1761133	Bcer	O	Bcer	FALSE	NA	NA	NA
Bc_ctg1_4378	B3	1.240166016	Bcer	M	Bcer	FALSE	NA	NA	NA
Bc_ctg1_4379	B3	18.48974787	Bcer	G	Bcer	FALSE	NA	NA	NA
Bc_ctg1_438	B3	6.990026633	Bcer	S	Bcer	FALSE	NA	NA	NA
Bc_ctg1_4380	B3	10.59778231	Bcer	S	Bcer	FALSE	NA	NA	NA
Bc_ctg1_4381	B3	8.906646839	Bcer	K	Bcer	FALSE	NA	NA	NA
Bc_ctg1_4382	B3	2.931301491	Bcer	EGP	Bcer	FALSE	NA	NA	NA
Bc_ctg1_4383	B3	0.563711825	Bcer	S	Bcer	FALSE	NA	NA	NA
Bc_ctg1_4384	B3	134.2761568	Bcer	E	Bcer	FALSE	NA	NA	NA
Bc_ctg1_4385	B3	8.906646839	Bcer	S	Bcer	FALSE	NA	NA	NA
Bc_ctg1_4386	B3	16.4603853	Bcer	K	Bcer	FALSE	NA	NA	NA
Bc_ctg1_4387	B3	13.8673109	Bcer	S	Bcer	FALSE	NA	NA	NA
Bc_ctg1_4388	B3	152.7659046	Bcer	CG	Bcer	FALSE	NA	NA	NA
Bc_ctg1_4389	B3	33.59722479	Bcer	S	Bcer	FALSE	NA	NA	NA
Bc_ctg1_439	B3	2.593074396	Bcer	M	Bcer	FALSE	NA	NA	NA
Bc_ctg1_4390	B3	13.52908381	Bcer	K	Bcer	FALSE	NA	NA	NA
Bc_ctg1_4391	B3	0.67645419	Bcer	K	Bcer	FALSE	NA	NA	NA
Bc_ctg1_4392	B3	14.76924982	Bcer	T	Bcer	FALSE	NA	NA	NA
Bc_ctg1_4393	B3	7.553738458	Bcer	S	Bcer	FALSE	NA	NA	NA
Bc_ctg1_4394	B3	11.6124636	Bcer	J	Bcer	FALSE	NA	NA	NA
Bc_ctg1_4395	B3	19.05345969	Bcer	J	Bcer	FALSE	NA	NA	NA
Bc_ctg1_4396	B3	8.455677379	Bcer	M	Bcer	FALSE	NA	NA	NA
Bc_ctg1_4397	B3	4.622436967	Bcer	S	Bcer	FALSE	NA	NA	NA
Bc_ctg1_4398	B3	0.45096946	Bcer	K	Bcer	FALSE	NA	NA	NA
Bc_ctg1_4399	B3	0.563711825	Bcer	NA	Bcer	FALSE	NA	NA	NA
Bc_ctg1_44	B3	52.53794211	Bcer	GT	Bcer	FALSE	NA	NA	NA
Bc_ctg1_4400	B3	1.465650746	Bcer	S	Bcer	FALSE	NA	NA	NA
Bc_ctg1_4401	B3	1.691135476	Bcer	NA	Bcer	FALSE	NA	NA	NA
Bc_ctg1_4402	B3	0.112742365	Bcer	NA	Bcer	FALSE	NA	NA	NA
Bc_ctg1_4403	B3	2.029362571	Bcer	NA	Bcer	FALSE	NA	NA	NA
Bc_ctg1_4404	B3	96.73294921	Bcer	NA	Bcer	FALSE	NA	NA	NA
Bc_ctg1_4405	B3	9.019389204	Bcer	NA	Bcer	FALSE	NA	NA	NA
Bc_ctg1_4406	B3	0.22548473	Bcer	NA	Bcer	FALSE	NA	NA	NA
Bc_ctg1_4407	B3	3.044043856	Bcer	NA	Bcer	FALSE	NA	NA	NA
Bc_ctg1_4408	B3	0	Bcer	NA	Bcer	FALSE	NA	NA	NA
Bc_ctg1_441	B3	1.916620206	Bcer	M	Bcer	FALSE	NA	NA	NA
Bc_ctg1_4411	B3	0	Bcer	NA	Bcer	FALSE	NA	NA	NA
Bc_ctg1_4414	B3	0	Bcer	NA	Bcer	FALSE	NA	NA	NA
Bc_ctg1_4416	B3	0.45096946	Bcer	NA	Bcer	FALSE	NA	NA	NA
Bc_ctg1_4418	B3	0	Bcer	NA	Bcer	FALSE	NA	NA	NA
Bc_ctg1_4419	B3	1.127423651	Bcer	D	Bcer	FALSE	NA	NA	NA
Bc_ctg1_442	B3	15.55844638	Bcer	NA	Bcer	FALSE	NA	NA	NA
Bc_ctg1_4420	B3	0	Bcer	NA	Bcer	FALSE	NA	NA	NA
Bc_ctg1_4422	B3	1.127423651	Bcer	NA	Bcer	FALSE	NA	NA	NA
Bc_ctg1_4423	B3	0	Bcer	NA	Bcer	FALSE	NA	NA	NA
Bc_ctg1_4424	B3	0.45096946	Bcer	NA	Bcer	FALSE	NA	NA	NA
Bc_ctg1_4425	B3	1.014681285	Bcer	NA	Bcer	FALSE	NA	NA	NA
Bc_ctg1_4426	B3	0	Bcer	S	Bcer	FALSE	NA	NA	NA
Bc_ctg1_4427	B3	0	Bcer	L	Bcer	FALSE	NA	NA	NA
Bc_ctg1_4428	B3	11.72520597	Bcer	S	Bcer	FALSE	NA	NA	NA
Bc_ctg1_4429	B3	0.112742365	Bcer	S	Bcer	FALSE	NA	NA	NA
Bc_ctg1_443	B3	23.90138139	Bcer	M	Bcer	FALSE	NA	NA	NA
Bc_ctg1_4430	B3	311.9581241	Bcer	S	Bcer	FALSE	NA	NA	NA
Bc_ctg1_4431	B3	19.16620206	Bcer	NA	Bcer	FALSE	NA	NA	NA
Bc_ctg1_4433	B3	0	Bcer	NA	Bcer	FALSE	NA	NA	NA
Bc_ctg1_4434	B3	7.891965554	Bcer	Q	Bcer	TRUE	NA	NA	NA
Bc_ctg1_4435	B3	1446.259059	Bcer	F	Bcer	FALSE	NA	NA	NA
Bc_ctg1_4436	B3	35.40110263	Bcer	NA	Bcer	FALSE	NA	NA	NA
Bc_ctg1_4437	B3	90.30663441	Bcer	L	Bcer	FALSE	NA	NA	NA

Bc_ctg1_4438	B3	1.352908381	Bcer	G	Bcer	FALSE	NA	NA	NA
Bc_ctg1_4439	B3	8.793904474	Bcer	E	Bcer	FALSE	NA	NA	NA
Bc_ctg1_444	B3	3.269528587	Bcer	NA	Bcer	FALSE	NA	NA	NA
Bc_ctg1_4440	B3	40.69999378	Bcer	NA	Bcer	FALSE	NA	NA	NA
Bc_ctg1_4441	B3	1.127423651	Bcer	C	Bcer	FALSE	NA	NA	NA
Bc_ctg1_4442	B3	0.45096946	Bcer	K	Bcer	FALSE	NA	NA	NA
Bc_ctg1_4443	B3	5.524375888	Bcer	S	Bcer	FALSE	NA	NA	NA
Bc_ctg1_4444	B3	0.112742365	Bcer	NA	Bcer	FALSE	NA	NA	NA
Bc_ctg1_4445	B3	99.88973544	Bcer	S	Bcer	FALSE	NA	NA	NA
Bc_ctg1_4446	B3	5.411633522	Bcer	S	Bcer	FALSE	NA	NA	NA
Bc_ctg1_4447	B3	21.19556463	Bcer	O	Bcer	FALSE	NA	NA	NA
Bc_ctg1_4448	B3	2.593074396	Bcer	E	Bcer	FALSE	NA	NA	NA
Bc_ctg1_4449	B3	0.112742365	Bcer	S	Bcer	FALSE	NA	NA	NA
Bc_ctg1_445	B3	491.1057422	Bcer	NA	Bcer	FALSE	NA	NA	NA
Bc_ctg1_4450	B3	0.45096946	Bcer	S	Bcer	FALSE	NA	NA	NA
Bc_ctg1_4451	B3	522.2226349	Bcer	C	Bcer	FALSE	NA	NA	NA
Bc_ctg1_4452	B3	226.386669	Bcer	C	Bcer	FALSE	NA	NA	NA
Bc_ctg1_4453	B3	90.75760387	Bcer	C	Bcer	FALSE	NA	NA	NA
Bc_ctg1_4454	B3	126.947903	Bcer	C	Bcer	FALSE	NA	NA	NA
Bc_ctg1_4455	B3	28.41107599	Bcer	K	Bcer	FALSE	NA	NA	NA
Bc_ctg1_4456	B3	69.56203924	Bcer	H	Bcer	FALSE	NA	NA	NA
Bc_ctg1_4457	B3	75.87561168	Bcer	H	Bcer	FALSE	NA	NA	NA
Bc_ctg1_4458	B3	120.1833611	Bcer	H	Bcer	FALSE	NA	NA	NA
Bc_ctg1_4459	B3	24.12686612	Bcer	H	Bcer	FALSE	NA	NA	NA
Bc_ctg1_446	B3	22.54847301	Bcer	T	Bcer	FALSE	NA	NA	NA
Bc_ctg1_4460	B3	1.691135476	Bcer	H	Bcer	FALSE	NA	NA	NA
Bc_ctg1_4461	B3	100.5661896	Bcer	P	Bcer	FALSE	NA	NA	NA
Bc_ctg1_4462	B3	2.029362571	Bcer	S	Bcer	FALSE	NA	NA	NA
Bc_ctg1_4463	B3	1.352908381	Bcer	NA	Bcer	FALSE	NA	NA	NA
Bc_ctg1_4464	B3	12.85262962	Bcer	H	Bcer	FALSE	NA	NA	NA
Bc_ctg1_4465	B3	30.10221147	Bcer	S	Bcer	FALSE	NA	NA	NA
Bc_ctg1_4466	B3	93.91439009	Bcer	H	Bcer	FALSE	NA	NA	NA
Bc_ctg1_4467	B3	74.29721857	Bcer	P	Bcer	FALSE	NA	NA	NA
Bc_ctg1_4468	B3	606.8921511	Bcer	C	Bcer	FALSE	NA	NA	NA
Bc_ctg1_4469	B3	8.342935014	Bcer	D	Bcer	FALSE	NA	NA	NA
Bc_ctg1_447	B3	1.691135476	Bcer	S	Bcer	FALSE	NA	NA	NA
Bc_ctg1_4470	B3	2.029362571	Bcer	M	Bcer	FALSE	NA	NA	NA
Bc_ctg1_4472	B3	0.112742365	Bcer	NA	Bcer	FALSE	NA	NA	NA
Bc_ctg1_4473	B3	13.30359908	Bcer	Q	Bcer	TRUE	NA	NA	NA
Bc_ctg1_4474	B3	5.073406427	Bcer	S	Bcer	FALSE	NA	NA	NA
Bc_ctg1_4475	B3	0.22548473	Bcer	NA	Bcer	FALSE	NA	NA	NA
Bc_ctg1_4476	B3	19.50442915	Bcer	S	Bcer	FALSE	NA	NA	NA
Bc_ctg1_4477	B3	0	Bcer	NA	Bcer	FALSE	NA	NA	NA
Bc_ctg1_448	B3	0.67645419	Bcer	S	Bcer	FALSE	NA	NA	NA
Bc_ctg1_4480	B3	28.41107599	Bcer	S	Bcer	FALSE	NA	NA	NA
Bc_ctg1_4481	B3	223.3426252	Bcer	S	Bcer	FALSE	NA	NA	NA
Bc_ctg1_4482	B3	293.693861	Bcer	S	Bcer	FALSE	NA	NA	NA
Bc_ctg1_4483	B3	0.90193892	Bcer	S	Bcer	FALSE	NA	NA	NA
Bc_ctg1_4484	B3	0	Bcer	L	Bcer	FALSE	NA	NA	NA
Bc_ctg1_4485	B3	0.45096946	Bcer	NA	Bcer	FALSE	NA	NA	NA
Bc_ctg1_4486	B3	86.1351669	Bcer	C	Bcer	FALSE	NA	NA	NA
Bc_ctg1_4487	B3	17.81329368	Bcer	S	Bcer	FALSE	NA	NA	NA
Bc_ctg1_4488	B3	190.872824	Bcer	S	Bcer	FALSE	NA	NA	NA
Bc_ctg1_4489	B3	24.35235085	Bcer	J	Bcer	FALSE	NA	NA	NA
Bc_ctg1_449	B3	4.847921697	Bcer	S	Bcer	FALSE	NA	NA	NA
Bc_ctg1_4490	B3	36.30304155	Bcer	J	Bcer	FALSE	NA	NA	NA
Bc_ctg1_4491	B3	1.127423651	Bcer	Q	Bcer	TRUE	NA	NA	NA
Bc_ctg1_4492	B3	34.04819425	Bcer	H	Bcer	FALSE	NA	NA	NA
Bc_ctg1_4493	B3	229.5434552	Bcer	J	Bcer	FALSE	NA	NA	NA
Bc_ctg1_4494	B3	3.382270952	Bcer	S	Bcer	FALSE	NA	NA	NA
Bc_ctg1_4495	B3	4.735179332	Bcer	K	Bcer	FALSE	NA	NA	NA
Bc_ctg1_4496	B3	11.38697887	Bcer	E	Bcer	FALSE	NA	NA	NA

Bc_ctg1_4498	B3	15.22021928	Bcer	J	Bcer	FALSE	NA	NA	NA
Bc_ctg1_4499	B3	21.53379172	Bcer	S	Bcer	FALSE	NA	NA	NA
Bc_ctg1_45	B3	1.916620206	Bcer	E	Bcer	FALSE	NA	NA	NA
Bc_ctg1_450	B3	33.59722479	Bcer	S	Bcer	FALSE	NA	NA	NA
Bc_ctg1_4500	B3	17.58780895	Bcer	T	Bcer	FALSE	NA	NA	NA
Bc_ctg1_4501	B3	5.524375888	Bcer	NA	Bcer	FALSE	NA	NA	NA
Bc_ctg1_4502	B3	297.1888743	Bcer	D	Bcer	FALSE	NA	NA	NA
Bc_ctg1_4503	B3	113.644304	Bcer	S	Bcer	FALSE	NA	NA	NA
Bc_ctg1_4504	B3	134.6143839	Bcer	KLT	Bcer	FALSE	NA	NA	NA
Bc_ctg1_4505	B3	179.1476181	Bcer	NU	Bcer	FALSE	NA	NA	NA
Bc_ctg1_4506	B3	3.495013317	Bcer	S	Bcer	FALSE	NA	NA	NA
Bc_ctg1_4507	B3	64.15040571	Bcer	MS	Bcer	FALSE	NA	NA	NA
Bc_ctg1_4508	B3	35.73932972	Bcer	S	Bcer	FALSE	NA	NA	NA
Bc_ctg1_4509	B3	10.48503995	Bcer	S	Bcer	FALSE	NA	NA	NA
Bc_ctg1_451	B3	39.45982777	Bcer	V	Bcer	FALSE	NA	NA	NA
Bc_ctg1_4510	B3	10.14681285	Bcer	I	Bcer	FALSE	NA	NA	NA
Bc_ctg1_4511	B3	55.69472834	Bcer	S	Bcer	FALSE	NA	NA	NA
Bc_ctg1_4512	B3	64.71411754	Bcer	U	Bcer	FALSE	NA	NA	NA
Bc_ctg1_4513	B3	2.480332031	Bcer	K	Bcer	FALSE	NA	NA	NA
Bc_ctg1_4514	B3	12.06343306	Bcer	EG	Bcer	FALSE	NA	NA	NA
Bc_ctg1_4515	B3	14.43102273	Bcer	C	Bcer	FALSE	NA	NA	NA
Bc_ctg1_4516	B3	4.735179332	Bcer	M	Bcer	FALSE	NA	NA	NA
Bc_ctg1_4517	B3	114.5462429	Bcer	S	Bcer	FALSE	NA	NA	NA
Bc_ctg1_4518	B3	28.29833363	Bcer	K	Bcer	FALSE	NA	NA	NA
Bc_ctg1_4519	B3	6.651799538	Bcer	S	Bcer	FALSE	NA	NA	NA
Bc_ctg1_452	B3	2.142104936	Bcer	NA	Bcer	FALSE	NA	NA	NA
Bc_ctg1_4520	B3	2.254847301	Bcer	K	Bcer	FALSE	NA	NA	NA
Bc_ctg1_4521	B3	37.76869229	Bcer	E	Bcer	FALSE	NA	NA	NA
Bc_ctg1_4522	B3	17.13683949	Bcer	K	Bcer	FALSE	NA	NA	NA
Bc_ctg1_4523	B3	8.568419744	Bcer	V	Bcer	FALSE	NA	NA	NA
Bc_ctg1_4524	B3	7.891965554	Bcer	U	Bcer	FALSE	NA	NA	NA
Bc_ctg1_4525	B3	268.2140865	Bcer	S	Bcer	FALSE	NA	NA	NA
Bc_ctg1_4526	B3	3.607755682	Bcer	S	Bcer	FALSE	NA	NA	NA
Bc_ctg1_4527	B3	1.465650746	Bcer	E	Bcer	FALSE	NA	NA	NA
Bc_ctg1_4528	B3	6.651799538	Bcer	S	Bcer	FALSE	NA	NA	NA
Bc_ctg1_4529	B3	101.2426438	Bcer	V	Bcer	FALSE	NA	NA	NA
Bc_ctg1_453	B3	77.00303533	Bcer	S	Bcer	FALSE	NA	NA	NA
Bc_ctg1_4530	B3	91.88502752	Bcer	F	Bcer	FALSE	NA	NA	NA
Bc_ctg1_4531	B3	22.77395774	Bcer	H	Bcer	FALSE	NA	NA	NA
Bc_ctg1_4532	B3	461.2290154	Bcer	M	Bcer	FALSE	NA	NA	NA
Bc_ctg1_4533	B3	98.19859996	Bcer	I	Bcer	FALSE	NA	NA	NA
Bc_ctg1_4534	B3	43.63129528	Bcer	I	Bcer	FALSE	NA	NA	NA
Bc_ctg1_4535	B3	81.06176047	Bcer	S	Bcer	FALSE	NA	NA	NA
Bc_ctg1_4536	B3	11.38697887	Bcer	U	Bcer	FALSE	NA	NA	NA
Bc_ctg1_4537	B3	104.1739453	Bcer	U	Bcer	FALSE	NA	NA	NA
Bc_ctg1_4538	B3	5.975345348	Bcer	S	Bcer	FALSE	NA	NA	NA
Bc_ctg1_4539	B3	157.6138263	Bcer	S	Bcer	FALSE	NA	NA	NA
Bc_ctg1_454	B3	1.240166016	Bcer	NA	Bcer	FALSE	NA	NA	NA
Bc_ctg1_4540	B3	20.18088334	Bcer	S	Bcer	FALSE	NA	NA	NA
Bc_ctg1_4541	B3	45.09694602	Bcer	S	Bcer	FALSE	NA	NA	NA
Bc_ctg1_4542	B3	0	Bcer	O	Bcer	FALSE	NA	NA	NA
Bc_ctg1_4543	B3	1023.362448	Bcer	E	Bcer	FALSE	NA	NA	NA
Bc_ctg1_4544	B3	1.014681285	Bcer	K	Bcer	FALSE	NA	NA	NA
Bc_ctg1_4545	B3	7.440996093	Bcer	NA	Bcer	FALSE	NA	NA	NA
Bc_ctg1_4546	B3	0.22548473	Bcer	M	Bcer	FALSE	NA	NA	NA
Bc_ctg1_4547	B3	105.4141113	Bcer	P	Bcer	FALSE	NA	NA	NA
Bc_ctg1_4548	B3	33.48448242	Bcer	E	Bcer	FALSE	NA	NA	NA
Bc_ctg1_4549	B3	85.00774325	Bcer	C	Bcer	FALSE	NA	NA	NA
Bc_ctg1_455	B3	0.22548473	Bcer	NA	Bcer	FALSE	NA	NA	NA
Bc_ctg1_4550	B3	82.75289595	Bcer	NA	Bcer	FALSE	NA	NA	NA
Bc_ctg1_4551	B3	27.96010653	Bcer	Q	Bcer	TRUE	NA	NA	NA
Bc_ctg1_4552	B3	0.112742365	Bcer	F	Bcer	FALSE	NA	NA	NA

Bc_ctg1_4553	B3	24.57783558	Bcer	M	Bcer	FALSE	NA	NA	NA
Bc_ctg1_4554	B3	6.313572443	Bcer	T	Bcer	FALSE	NA	NA	NA
Bc_ctg1_4555	B3	5.186148792	Bcer	K	Bcer	FALSE	NA	NA	NA
Bc_ctg1_4556	B3	61.89555841	Bcer	K	Bcer	FALSE	NA	NA	NA
Bc_ctg1_4557	B3	47.01356623	Bcer	S	Bcer	FALSE	NA	NA	NA
Bc_ctg1_4558	B3	4964.722787	Bcer	C	Bcer	FALSE	NA	NA	NA
Bc_ctg1_4559	B3	3.720498047	Bcer	EP	Bcer	FALSE	NA	NA	NA
Bc_ctg1_456	B3	3.044043856	Bcer	L	Bcer	FALSE	NA	NA	NA
Bc_ctg1_4560	B3	0.789196555	Bcer	EP	Bcer	FALSE	NA	NA	NA
Bc_ctg1_4562	B3	99.32602361	Bcer	G	Bcer	FALSE	NA	NA	NA
Bc_ctg1_4563	B3	77.00303533	Bcer	EG	Bcer	FALSE	NA	NA	NA
Bc_ctg1_4564	B3	72.04237127	Bcer	G	Bcer	FALSE	NA	NA	NA
Bc_ctg1_4565	B3	2.029362571	Bcer	S	Bcer	FALSE	NA	NA	NA
Bc_ctg1_4566	B3	0.45096946	Bcer	M	Bcer	FALSE	NA	NA	NA
Bc_ctg1_4567	B3	0.112742365	Bcer	O	Bcer	FALSE	NA	NA	NA
Bc_ctg1_4568	B3	15.55844638	Bcer	S	Bcer	FALSE	NA	NA	NA
Bc_ctg1_4569	B3	44.75871893	Bcer	EGP	Bcer	FALSE	NA	NA	NA
Bc_ctg1_457	B3	335.8595055	Bcer	K	Bcer	FALSE	NA	NA	NA
Bc_ctg1_4570	B3	23.2249272	Bcer	S	Bcer	FALSE	NA	NA	NA
Bc_ctg1_4571	B3	6.088087713	Bcer	E	Bcer	FALSE	NA	NA	NA
Bc_ctg1_4572	B3	18.03877841	Bcer	EM	Bcer	FALSE	NA	NA	NA
Bc_ctg1_4573	B3	28.29833363	Bcer	E	Bcer	FALSE	NA	NA	NA
Bc_ctg1_4574	B3	27.05816761	Bcer	NA	Bcer	FALSE	NA	NA	NA
Bc_ctg1_4575	B3	2.705816761	Bcer	V	Bcer	FALSE	NA	NA	NA
Bc_ctg1_4576	B3	2.029362571	Bcer	NA	Bcer	FALSE	NA	NA	NA
Bc_ctg1_4577	B3	70.68946289	Bcer	NA	Bcer	FALSE	NA	NA	NA
Bc_ctg1_4578	B3	0.67645419	Bcer	K	Bcer	FALSE	NA	NA	NA
Bc_ctg1_4579	B3	45.20968839	Bcer	S	Bcer	FALSE	NA	NA	NA
Bc_ctg1_458	B3	69.33655451	Bcer	NA	Bcer	FALSE	NA	NA	NA
Bc_ctg1_4580	B3	84.55677379	Bcer	C	Bcer	FALSE	NA	NA	NA
Bc_ctg1_4581	B3	75.08641512	Bcer	S	Bcer	FALSE	NA	NA	NA
Bc_ctg1_4582	B3	127.9625843	Bcer	T	Bcer	FALSE	NA	NA	NA
Bc_ctg1_4583	B3	0.22548473	Bcer	O	Bcer	FALSE	NA	NA	NA
Bc_ctg1_4584	B3	5.411633522	Bcer	H	Bcer	FALSE	NA	NA	NA
Bc_ctg1_4585	B3	2.367589666	Bcer	I	Bcer	FALSE	NA	NA	NA
Bc_ctg1_4586	B3	2.367589666	Bcer	I	Bcer	FALSE	NA	NA	NA
Bc_ctg1_4587	B3	3.495013317	Bcer	E	Bcer	FALSE	NA	NA	NA
Bc_ctg1_4588	B3	20.29362571	Bcer	K	Bcer	FALSE	NA	NA	NA
Bc_ctg1_4589	B3	40.81273615	Bcer	KT	Bcer	FALSE	NA	NA	NA
Bc_ctg1_459	B3	9.470358664	Bcer	C	Bcer	FALSE	NA	NA	NA
Bc_ctg1_4590	B3	14.76924982	Bcer	E	Bcer	FALSE	NA	NA	NA
Bc_ctg1_4591	B3	1.465650746	Bcer	S	Bcer	FALSE	NA	NA	NA
Bc_ctg1_4592	B3	2.142104936	Bcer	S	Bcer	FALSE	NA	NA	NA
Bc_ctg1_4593	B3	0	Bcer	NA	Bcer	FALSE	NA	NA	NA
Bc_ctg1_4596	B3	9.357616299	Bcer	NA	Bcer	FALSE	NA	NA	NA
Bc_ctg1_4597	B3	53.77810813	Bcer	KLT	Bcer	FALSE	NA	NA	NA
Bc_ctg1_4598	B3	73.73350674	Bcer	S	Bcer	FALSE	NA	NA	NA
Bc_ctg1_4599	B3	20.18088334	Bcer	I	Bcer	FALSE	NA	NA	NA
Bc_ctg1_46	B3	2.367589666	Bcer	S	Bcer	FALSE	NA	NA	NA
Bc_ctg1_460	B3	6.200830078	Bcer	S	Bcer	FALSE	NA	NA	NA
Bc_ctg1_4600	B3	4.847921697	Bcer	P	Bcer	FALSE	NA	NA	NA
Bc_ctg1_4602	B3	78.24320135	Bcer	CO	Bcer	FALSE	NA	NA	NA
Bc_ctg1_4603	B3	19.84265625	Bcer	J	Bcer	FALSE	NA	NA	NA
Bc_ctg1_4604	B3	0	Bcer	NA	Bcer	FALSE	NA	NA	NA
Bc_ctg1_4605	B3	0	Bcer	NA	Bcer	FALSE	NA	NA	NA
Bc_ctg1_4606	B3	0.789196555	Bcer	NA	Bcer	FALSE	NA	NA	NA
Bc_ctg1_4607	B3	0.563711825	Bcer	NA	Bcer	FALSE	NA	NA	NA
Bc_ctg1_4608	B3	0.338227095	Bcer	NA	Bcer	FALSE	NA	NA	NA
Bc_ctg1_4609	B3	0	Bcer	NA	Bcer	FALSE	NA	NA	NA
Bc_ctg1_461	B3	0	Bcer	F	Bcer	FALSE	NA	NA	NA
Bc_ctg1_4610	B3	8.681162109	Bcer	M	Bcer	FALSE	NA	NA	NA
Bc_ctg1_4611	B3	35.06287553	Bcer	M	Bcer	FALSE	NA	NA	NA

Bc_ctg1_4612	B3	0	Bcer	NA	Bcer	FALSE	NA	NA	NA
Bc_ctg1_4613	B3	0.112742365	Bcer	NA	Bcer	FALSE	NA	NA	NA
Bc_ctg1_4614	B3	0.338227095	Bcer	S	Bcer	FALSE	NA	NA	NA
Bc_ctg1_4616	B3	18.3770055	Bcer	NA	Bcer	FALSE	NA	NA	NA
Bc_ctg1_4617	B3	0.338227095	Bcer	NA	Bcer	FALSE	NA	NA	NA
Bc_ctg1_4618	B3	17.92603604	Bcer	IQ	Bcer	TRUE	NA	NA	NA
Bc_ctg1_4619	B3	0.112742365	Bcer	NA	Bcer	FALSE	NA	NA	NA
Bc_ctg1_462	B3	0	Bcer	S	Bcer	FALSE	NA	NA	NA
Bc_ctg1_4620	B3	2.480332031	Bcer	S	Bcer	FALSE	NA	NA	NA
Bc_ctg1_4621	B3	0	Bcer	S	Bcer	FALSE	NA	NA	NA
Bc_ctg1_4622	B3	624.47996	Bcer	M	Bcer	FALSE	NA	NA	NA
Bc_ctg1_4623	B3	0	Bcer	NA	Bcer	FALSE	NA	NA	NA
Bc_ctg1_4624	B3	1.578393111	Bcer	EGP	Bcer	FALSE	NA	NA	NA
Bc_ctg1_4626	B3	5.073406427	Bcer	C	Bcer	FALSE	NA	NA	NA
Bc_ctg1_4627	B3	23.78863903	Bcer	S	Bcer	FALSE	Bc_Ga0417192_01_rn32	Other	NA
Bc_ctg1_4628	B3	8.117450284	Bcer	G	Bcer	FALSE	Bc_Ga0417192_01_rn32	Ancillary biosynthesis	SMCOG1231
Bc_ctg1_4629	B3	107.6689586	Bcer	C	Bcer	FALSE	Bc_Ga0417192_01_rn32	Ancillary biosynthesis	SMCOG1006
Bc_ctg1_463	B3	4.847921697	Bcer	S	Bcer	FALSE	NA	NA	NA
Bc_ctg1_4630	B3	58.51328746	Bcer	I	Bcer	FALSE	Bc_Ga0417192_01_rn32	Ancillary biosynthesis	SMCOG1100
Bc_ctg1_4631	B3	152.6531623	Bcer	C	Bcer	FALSE	Bc_Ga0417192_01_rn32	Ancillary biosynthesis	SMCOG1017
Bc_ctg1_4632	B3	6.990026633	Bcer	E	Bcer	FALSE	Bc_Ga0417192_01_rn32	Transport	SMCOG1116
Bc_ctg1_4633	B3	192.5639595	Bcer	I	Bcer	FALSE	Bc_Ga0417192_01_rn32	Ancillary biosynthesis	SMCOG1023
Bc_ctg1_4634	B3	1.803877841	Bcer	NA	Bcer	FALSE	Bc_Ga0417192_01_rn32	Other	NA
Bc_ctg1_4635	B3	1.352908381	Bcer	S	Bcer	FALSE	Bc_Ga0417192_01_rn32	Other	SMCOG1300
Bc_ctg1_4636	B3	30.44043856	Bcer	L	Bcer	FALSE	Bc_Ga0417192_01_rn32	Other	SMCOG1230
Bc_ctg1_4637	B3	119.2814222	Bcer	L	Bcer	FALSE	Bc_Ga0417192_01_rn32	Other	SMCOG1218
Bc_ctg1_4639	B3	35.06287553	Bcer	K	Bcer	FALSE	Bc_Ga0417192_01_rn32	Regulation	SMCOG1058
Bc_ctg1_464	B3	13.41634144	Bcer	M	Bcer	FALSE	NA	NA	NA
Bc_ctg1_4640	B3	0.563711825	Bcer	NA	Bcer	FALSE	Bc_Ga0417192_01_rn32	Other	NA
Bc_ctg1_4641	B3	1218.632224	Bcer	P	Bcer	FALSE	Bc_Ga0417192_01_rn32	Transport	SMCOG1137
Bc_ctg1_4642	B3	26.94542525	Bcer	IQ	Bcer	TRUE	Bc_Ga0417192_01_rn32	Ancillary biosynthesis	SMCOG1001
Bc_ctg1_4643	B3	62.68475497	Bcer	HQ	Bcer	TRUE	Bc_Ga0417192_01_rn32	Ancillary biosynthesis	SMCOG1018
Bc_ctg1_4644	B3	92.44873934	Bcer	Q	Bcer	TRUE	Bc_Ga0417192_01_rn32	Core biosynthesis	SMCOG1002
Bc_ctg1_4645	B3	29.87672674	Bcer	Q	Bcer	TRUE	Bc_Ga0417192_01_rn32	Ancillary biosynthesis	SMCOG1027
Bc_ctg1_4646	B3	476.9002042	Bcer	Q	Bcer	TRUE	Bc_Ga0417192_01_rn32	Core biosynthesis	SMCOG1127
Bc_ctg1_4647	B3	5.749860618	Bcer	S	Bcer	FALSE	Bc_Ga0417192_01_rn32	Ancillary biosynthesis	SMCOG1009
Bc_ctg1_4648	B3	26.49445579	Bcer	P	Bcer	FALSE	Bc_Ga0417192_01_rn32	Transport	SMCOG1005
Bc_ctg1_4649	B3	10.37229758	Bcer	H	Bcer	FALSE	Bc_Ga0417192_01_rn32	Ancillary biosynthesis	SMCOG1012
Bc_ctg1_465	B3	62.9102397	Bcer	IQ	Bcer	TRUE	Bc_Ga0417192_01_rn06	Other	NA
Bc_ctg1_4650	B3	20.40636807	Bcer	H	Bcer	FALSE	Bc_Ga0417192_01_rn32	Ancillary biosynthesis	SMCOG1259
Bc_ctg1_4651	B3	19.84265625	Bcer	L	Bcer	FALSE	Bc_Ga0417192_01_rn32	Other	SMCOG1132
Bc_ctg1_4652	B3	24.12686612	Bcer	S	Bcer	FALSE	Bc_Ga0417192_01_rn32	Other	SMCOG1267
Bc_ctg1_4653	B3	32.46980113	Bcer	S	Bcer	FALSE	Bc_Ga0417192_01_rn32	Other	SMCOG1250
Bc_ctg1_4654	B3	0.112742365	Bcer	O	Bcer	FALSE	Bc_Ga0417192_01_rn32	Ancillary biosynthesis	SMCOG1075
Bc_ctg1_4655	B3	20.9700799	Bcer	E	Bcer	FALSE	Bc_Ga0417192_01_rn32	Ancillary biosynthesis	SMCOG1153
Bc_ctg1_4656	B3	4.058725142	Bcer	NA	Bcer	FALSE	Bc_Ga0417192_01_rn32	Other	SMCOG1289
Bc_ctg1_4657	B3	0.45096946	Bcer	NA	Bcer	FALSE	Bc_Ga0417192_01_rn32	Other	NA
Bc_ctg1_4658	B3	1.014681285	Bcer	G	Bcer	FALSE	Bc_Ga0417192_01_rn32	Ancillary biosynthesis	SMCOG1258
Bc_ctg1_4659	B3	41.82741743	Bcer	J	Bcer	FALSE	Bc_Ga0417192_01_rn32	Ancillary biosynthesis	SMCOG1154
Bc_ctg1_466	B3	25.14154741	Bcer	S	Bcer	FALSE	Bc_Ga0417192_01_rn06	Other	NA
Bc_ctg1_4660	B3	69.33655451	Bcer	J	Bcer	FALSE	Bc_Ga0417192_01_rn32	Other	SMCOG1162
Bc_ctg1_4661	B3	3.833240412	Bcer	V	Bcer	FALSE	Bc_Ga0417192_01_rn32	Transport	SMCOG1000
Bc_ctg1_4662	B3	28.86204545	Bcer	V	Bcer	FALSE	Bc_Ga0417192_01_rn32	Transport	SMCOG1243
Bc_ctg1_4663	B3	5.637118253	Bcer	G	Bcer	FALSE	Bc_Ga0417192_01_rn32	Transport	SMCOG1020
Bc_ctg1_4664	B3	1.803877841	Bcer	P	Bcer	FALSE	Bc_Ga0417192_01_rn32	Transport	SMCOG1118
Bc_ctg1_4665	B3	17.58780895	Bcer	P	Bcer	FALSE	Bc_Ga0417192_01_rn32	Transport	SMCOG1085
Bc_ctg1_4666	B3	3.495013317	Bcer	EH	Bcer	FALSE	NA	NA	NA
Bc_ctg1_4667	B3	0.90193892	Bcer	P	Bcer	FALSE	NA	NA	NA
Bc_ctg1_4668	B3	17.47506658	Bcer	S	Bcer	FALSE	NA	NA	NA
Bc_ctg1_4669	B3	14.54376509	Bcer	F	Bcer	FALSE	NA	NA	NA
Bc_ctg1_467	B3	1.127423651	Bcer	E	Bcer	FALSE	Bc_Ga0417192_01_rn06	Ancillary biosynthesis	SMCOG1063
Bc_ctg1_4670	B3	5.637118253	Bcer	S	Bcer	FALSE	NA	NA	NA

Bc_ctg1_4671	B3	3.156786221	Bcer	NA	Bcer	FALSE	NA	NA	NA
Bc_ctg1_4673	B3	0	Bcer	K	Bcer	FALSE	NA	NA	NA
Bc_ctg1_4674	B3	0	Bcer	S	Bcer	FALSE	NA	NA	NA
Bc_ctg1_4675	B3	4.622436967	Bcer	H	Bcer	FALSE	NA	NA	NA
Bc_ctg1_4676	B3	1.465650746	Bcer	K	Bcer	FALSE	NA	NA	NA
Bc_ctg1_4677	B3	1.916620206	Bcer	K	Bcer	FALSE	NA	NA	NA
Bc_ctg1_4678	B3	4.847921697	Bcer	S	Bcer	FALSE	NA	NA	NA
Bc_ctg1_4679	B3	14.54376509	Bcer	K	Bcer	FALSE	NA	NA	NA
Bc_ctg1_468	B3	82.41466885	Bcer	V	Bcer	FALSE	Bc_Ga0417192_01_rn06	Other	NA
Bc_ctg1_4680	B3	20.74459517	Bcer	S	Bcer	FALSE	NA	NA	NA
Bc_ctg1_4682	B3	8.117450284	Bcer	V	Bcer	FALSE	NA	NA	NA
Bc_ctg1_4683	B3	1.127423651	Bcer	K	Bcer	FALSE	NA	NA	NA
Bc_ctg1_4684	B3	4.509694602	Bcer	K	Bcer	FALSE	NA	NA	NA
Bc_ctg1_4685	B3	7.440996093	Bcer	J	Bcer	FALSE	NA	NA	NA
Bc_ctg1_4686	B3	2.029362571	Bcer	EG	Bcer	FALSE	NA	NA	NA
Bc_ctg1_4687	B3	3.382270952	Bcer	K	Bcer	FALSE	NA	NA	NA
Bc_ctg1_4688	B3	11.6124636	Bcer	NA	Bcer	FALSE	NA	NA	NA
Bc_ctg1_4689	B3	0	Bcer	NA	Bcer	FALSE	NA	NA	NA
Bc_ctg1_469	B3	6.764541903	Bcer	F	Bcer	FALSE	Bc_Ga0417192_01_rn06	Other	NA
Bc_ctg1_4690	B3	88.61549893	Bcer	H	Bcer	FALSE	NA	NA	NA
Bc_ctg1_4691	B3	1.240166016	Bcer	NA	Bcer	FALSE	NA	NA	NA
Bc_ctg1_4692	B3	117.9285138	Bcer	J	Bcer	FALSE	NA	NA	NA
Bc_ctg1_4693	B3	23.2249272	Bcer	S	Bcer	FALSE	NA	NA	NA
Bc_ctg1_4694	B3	28.74930309	Bcer	K	Bcer	FALSE	NA	NA	NA
Bc_ctg1_4695	B3	3.945982777	Bcer	S	Bcer	FALSE	NA	NA	NA
Bc_ctg1_4696	B3	4.622436967	Bcer	F	Bcer	FALSE	NA	NA	NA
Bc_ctg1_4697	B3	5.298891157	Bcer	NA	Bcer	FALSE	NA	NA	NA
Bc_ctg1_4698	B3	7.553738458	Bcer	K	Bcer	FALSE	NA	NA	NA
Bc_ctg1_4699	B3	6.200830078	Bcer	E	Bcer	FALSE	NA	NA	NA
Bc_ctg1_47	B3	0.563711825	Bcer	K	Bcer	FALSE	NA	NA	NA
Bc_ctg1_470	B3	9.357616299	Bcer	H	Bcer	FALSE	Bc_Ga0417192_01_rn06	Other	NA
Bc_ctg1_4700	B3	1.014681285	Bcer	NA	Bcer	FALSE	NA	NA	NA
Bc_ctg1_4701	B3	3.269528587	Bcer	S	Bcer	FALSE	NA	NA	NA
Bc_ctg1_4702	B3	11.72520597	Bcer	K	Bcer	FALSE	NA	NA	NA
Bc_ctg1_4703	B3	7.553738458	Bcer	K	Bcer	FALSE	NA	NA	NA
Bc_ctg1_4704	B3	1.803877841	Bcer	S	Bcer	FALSE	NA	NA	NA
Bc_ctg1_4705	B3	68.32187322	Bcer	S	Bcer	FALSE	NA	NA	NA
Bc_ctg1_4706	B3	45.77340021	Bcer	S	Bcer	FALSE	NA	NA	NA
Bc_ctg1_4707	B3	69.44929687	Bcer	V	Bcer	FALSE	NA	NA	NA
Bc_ctg1_4708	B3	87.03710582	Bcer	V	Bcer	FALSE	NA	NA	NA
Bc_ctg1_4709	B3	1.465650746	Bcer	NA	Bcer	FALSE	NA	NA	NA
Bc_ctg1_471	B3	1.240166016	Bcer	S	Bcer	FALSE	Bc_Ga0417192_01_rn06	Other	NA
Bc_ctg1_4710	B3	0.563711825	Bcer	S	Bcer	FALSE	NA	NA	NA
Bc_ctg1_4711	B3	13.8673109	Bcer	NA	Bcer	FALSE	NA	NA	NA
Bc_ctg1_4712	B3	5.749860618	Bcer	NA	Bcer	FALSE	NA	NA	NA
Bc_ctg1_4713	B3	13.8673109	Bcer	E	Bcer	FALSE	NA	NA	NA
Bc_ctg1_4714	B3	6.088087713	Bcer	K	Bcer	FALSE	NA	NA	NA
Bc_ctg1_4715	B3	0.45096946	Bcer	NA	Bcer	FALSE	NA	NA	NA
Bc_ctg1_4716	B3	2.818559126	Bcer	M	Bcer	FALSE	NA	NA	NA
Bc_ctg1_4717	B3	0.67645419	Bcer	M	Bcer	FALSE	NA	NA	NA
Bc_ctg1_4719	B3	0.22548473	Bcer	G	Bcer	FALSE	NA	NA	NA
Bc_ctg1_472	B3	95.38004083	Bcer	C	Bcer	FALSE	Bc_Ga0417192_01_rn06	Ancillary biosynthesis	SMCOG1017
Bc_ctg1_4720	B3	0.563711825	Bcer	NA	Bcer	FALSE	NA	NA	NA
Bc_ctg1_4721	B3	2.818559126	Bcer	H	Bcer	FALSE	NA	NA	NA
Bc_ctg1_4722	B3	1.916620206	Bcer	K	Bcer	FALSE	NA	NA	NA
Bc_ctg1_4724	B3	25.93074396	Bcer	S	Bcer	FALSE	NA	NA	NA
Bc_ctg1_4725	B3	186.0249023	Bcer	P	Bcer	FALSE	NA	NA	NA
Bc_ctg1_4726	B3	345.893576	Bcer	S	Bcer	FALSE	NA	NA	NA
Bc_ctg1_4727	B3	1.240166016	Bcer	T	Bcer	FALSE	NA	NA	NA
Bc_ctg1_4728	B3	0	Bcer	G	Bcer	FALSE	NA	NA	NA
Bc_ctg1_4729	B3	1.014681285	Bcer	G	Bcer	FALSE	NA	NA	NA
Bc_ctg1_473	B3	40.02353959	Bcer	IQ	Bcer	TRUE	Bc_Ga0417192_01_rn06	Ancillary biosynthesis	SMCOG1001

Bc_ctg1_4730	B3	0	Bcer	NA	Bcer	FALSE	NA	NA	NA
Bc_ctg1_4731	B3	0.45096946	Bcer	O	Bcer	FALSE	NA	NA	NA
Bc_ctg1_4732	B3	2.480332031	Bcer	G	Bcer	FALSE	NA	NA	NA
Bc_ctg1_4733	B3	2.931301491	Bcer	I	Bcer	FALSE	NA	NA	NA
Bc_ctg1_4734	B3	12.51440252	Bcer	E	Bcer	FALSE	NA	NA	NA
Bc_ctg1_4735	B3	7.215511363	Bcer	E	Bcer	FALSE	NA	NA	NA
Bc_ctg1_4736	B3	2.480332031	Bcer	K	Bcer	FALSE	NA	NA	NA
Bc_ctg1_4737	B3	0.22548473	Bcer	EGP	Bcer	FALSE	NA	NA	NA
Bc_ctg1_4738	B3	2.705816761	Bcer	S	Bcer	FALSE	NA	NA	NA
Bc_ctg1_4739	B3	4.284209872	Bcer	S	Bcer	FALSE	NA	NA	NA
Bc_ctg1_4741	B3	142.05538	Bcer	L	Bcer	FALSE	NA	NA	NA
Bc_ctg1_4742	B3	3.382270952	Bcer	NA	Bcer	FALSE	NA	NA	NA
Bc_ctg1_4743	B3	7.553738458	Bcer	K	Bcer	FALSE	NA	NA	NA
Bc_ctg1_4744	B3	3.833240412	Bcer	EGP	Bcer	FALSE	NA	NA	NA
Bc_ctg1_4746	B3	0.338227095	Bcer	NA	Bcer	FALSE	NA	NA	NA
Bc_ctg1_4747	B3	0.45096946	Bcer	S	Bcer	FALSE	NA	NA	NA
Bc_ctg1_4748	B3	0.45096946	Bcer	NA	Bcer	FALSE	NA	NA	NA
Bc_ctg1_4749	B3	0	Bcer	NA	Bcer	FALSE	NA	NA	NA
Bc_ctg1_475	B3	4.396952237	Bcer	I	Bcer	FALSE	Bc_Ga0417192_01_rn06	Core biosynthesis	None
Bc_ctg1_4750	B3	0.67645419	Bcer	NA	Bcer	FALSE	NA	NA	NA
Bc_ctg1_4751	B3	3.833240412	Bcer	NA	Bcer	FALSE	NA	NA	NA
Bc_ctg1_4754	B3	0	Bcer	E	Bcer	FALSE	NA	NA	NA
Bc_ctg1_4755	B3	2.818559126	Bcer	N	Bcer	FALSE	NA	NA	NA
Bc_ctg1_4756	B3	0	Bcer	NA	Bcer	FALSE	NA	NA	NA
Bc_ctg1_4758	B3	0.22548473	Bcer	NA	Bcer	FALSE	NA	NA	NA
Bc_ctg1_4759	B3	3.833240412	Bcer	NA	Bcer	FALSE	NA	NA	NA
Bc_ctg1_476	B3	22.54847301	Bcer	S	Bcer	FALSE	Bc_Ga0417192_01_rn06	Other	NA
Bc_ctg1_4760	B3	0.45096946	Bcer	S	Bcer	FALSE	NA	NA	NA
Bc_ctg1_4761	B3	0	Bcer	NA	Bcer	FALSE	NA	NA	NA
Bc_ctg1_4762	B3	0.112742365	Bcer	NA	Bcer	FALSE	NA	NA	NA
Bc_ctg1_4763	B3	4.284209872	Bcer	NA	Bcer	FALSE	NA	NA	NA
Bc_ctg1_4765	B3	2.254847301	Bcer	Q	Bcer	TRUE	NA	NA	NA
Bc_ctg1_4766	B3	4.509694602	Bcer	S	Bcer	FALSE	NA	NA	NA
Bc_ctg1_4767	B3	9.244873934	Bcer	Q	Bcer	TRUE	NA	NA	NA
Bc_ctg1_4768	B3	0.22548473	Bcer	GH	Bcer	FALSE	NA	NA	NA
Bc_ctg1_4769	B3	257.9545312	Bcer	K	Bcer	FALSE	NA	NA	NA
Bc_ctg1_477	B3	54.00359286	Bcer	S	Bcer	FALSE	Bc_Ga0417192_01_rn06	Transport	SMCOG1166
Bc_ctg1_4770	B3	242.734312	Bcer	G	Bcer	FALSE	Bc_Ga0417192_01_rn33	Ancillary biosynthesis	SMCOG1101
Bc_ctg1_4771	B3	157.6138263	Bcer	S	Bcer	FALSE	Bc_Ga0417192_01_rn33	Other	NA
Bc_ctg1_4773	B3	1.127423651	Bcer	NA	Bcer	FALSE	Bc_Ga0417192_01_rn33	Other	NA
Bc_ctg1_4774	B3	1.014681285	Bcer	S	Bcer	FALSE	Bc_Ga0417192_01_rn33	Other	NA
Bc_ctg1_4775	B3	940.3840669	Bcer	E	Bcer	FALSE	Bc_Ga0417192_01_rn33	Ancillary biosynthesis	SMCOG1247
Bc_ctg1_4776	B3	0.67645419	Bcer	S	Bcer	FALSE	Bc_Ga0417192_01_rn33	Other	NA
Bc_ctg1_4777	B3	0.789196555	Bcer	NA	Bcer	FALSE	Bc_Ga0417192_01_rn33	Other	NA
Bc_ctg1_4778	B3	7.666480824	Bcer	E	Bcer	FALSE	Bc_Ga0417192_01_rn33	Other	NA
Bc_ctg1_4779	B3	0.563711825	Bcer	KT	Bcer	FALSE	Bc_Ga0417192_01_rn33	Other	NA
Bc_ctg1_478	B3	0	Bcer	S	Bcer	FALSE	Bc_Ga0417192_01_rn06	Other	NA
Bc_ctg1_4781	B3	2.818559126	Bcer	K	Bcer	FALSE	Bc_Ga0417192_01_rn33	Other	NA
Bc_ctg1_4782	B3	2.142104936	Bcer	K	Bcer	FALSE	Bc_Ga0417192_01_rn33	Other	NA
Bc_ctg1_4783	B3	3.945982777	Bcer	NA	Bcer	FALSE	Bc_Ga0417192_01_rn33	Other	NA
Bc_ctg1_4784	B3	1.691135476	Bcer	S	Bcer	FALSE	Bc_Ga0417192_01_rn33	Other	NA
Bc_ctg1_4785	B3	12.96537198	Bcer	S	Bcer	FALSE	Bc_Ga0417192_01_rn33	Ancillary biosynthesis	SMCOG1156
Bc_ctg1_4786	B3	0.112742365	Bcer	M	Bcer	FALSE	Bc_Ga0417192_01_rn33	Other	NA
Bc_ctg1_4787	B3	1.465650746	Bcer	I	Bcer	FALSE	Bc_Ga0417192_01_rn33	Ancillary biosynthesis	SMCOG1088
Bc_ctg1_4788	B3	22.77395774	Bcer	E	Bcer	FALSE	Bc_Ga0417192_01_rn33	Other	NA
Bc_ctg1_4789	B3	4.171467507	Bcer	EGP	Bcer	FALSE	Bc_Ga0417192_01_rn33	Transport	SMCOG1020
Bc_ctg1_479	B3	6.990026633	Bcer	NA	Bcer	FALSE	Bc_Ga0417192_01_rn06	Other	NA
Bc_ctg1_4790	B3	13.07811435	Bcer	Q	Bcer	TRUE	Bc_Ga0417192_01_rn33	Core biosynthesis	SMCOG1002
Bc_ctg1_4791	B3	16.34764293	Bcer	Q	Bcer	TRUE	Bc_Ga0417192_01_rn33	Core biosynthesis	SMCOG1127
Bc_ctg1_4792	B3	73.95899147	Bcer	Q	Bcer	TRUE	Bc_Ga0417192_01_rn33	Core biosynthesis	SMCOG1127
Bc_ctg1_4793	B3	1.803877841	Bcer	H	Bcer	FALSE	Bc_Ga0417192_01_rn33	Ancillary biosynthesis	SMCOG1012
Bc_ctg1_4794	B3	4.960664062	Bcer	Q	Bcer	TRUE	Bc_Ga0417192_01_rn33	Ancillary biosynthesis	SMCOG1004

Bc_ctg1_4795	B3	8.455677379	Bcer	P	Bcer	FALSE	Bc_Ga0417192_01_rn33	Other	NA
Bc_ctg1_4796	B3	5.186148792	Bcer	E	Bcer	FALSE	Bc_Ga0417192_01_rn33	Ancillary biosynthesis	SMCOG1153
Bc_ctg1_4798	B3	2.705816761	Bcer	E	Bcer	FALSE	Bc_Ga0417192_01_rn33	Other	NA
Bc_ctg1_4799	B3	23.33766957	Bcer	T	Bcer	FALSE	Bc_Ga0417192_01_rn33	Other	NA
Bc_ctg1_48	B3	0.789196555	Bcer	IQ	Bcer	TRUE	NA	NA	NA
Bc_ctg1_480	B3	2.705816761	Bcer	NA	Bcer	FALSE	Bc_Ga0417192_01_rn06	Other	NA
Bc_ctg1_4800	B3	14.99473455	Bcer	T	Bcer	FALSE	Bc_Ga0417192_01_rn33	Regulation	SMCOG1008
Bc_ctg1_4801	B3	0.67645419	Bcer	EG	Bcer	FALSE	Bc_Ga0417192_01_rn33	Other	NA
Bc_ctg1_4802	B3	0.22548473	Bcer	S	Bcer	FALSE	Bc_Ga0417192_01_rn33	Ancillary biosynthesis	SMCOG1038
Bc_ctg1_4803	B3	0.112742365	Bcer	S	Bcer	FALSE	Bc_Ga0417192_01_rn33	Other	NA
Bc_ctg1_4804	B3	0.563711825	Bcer	E	Bcer	FALSE	Bc_Ga0417192_01_rn33	Other	NA
Bc_ctg1_4805	B3	1.691135476	Bcer	E	Bcer	FALSE	Bc_Ga0417192_01_rn33	Ancillary biosynthesis	SMCOG1038
Bc_ctg1_4806	B3	1.465650746	Bcer	L	Bcer	FALSE	Bc_Ga0417192_01_rn33	Other	NA
Bc_ctg1_4807	B3	7.215511363	Bcer	K	Bcer	FALSE	NA	NA	NA
Bc_ctg1_4808	B3	827.07799	Bcer	S	Bcer	FALSE	NA	NA	NA
Bc_ctg1_4809	B3	728.3156782	Bcer	E	Bcer	FALSE	NA	NA	NA
Bc_ctg1_481	B3	39.79805486	Bcer	D	Bcer	FALSE	Bc_Ga0417192_01_rn06	Other	SMCOG1157
Bc_ctg1_4810	B3	978.3782439	Bcer	E	Bcer	FALSE	NA	NA	NA
Bc_ctg1_4811	B3	82.75289595	Bcer	E	Bcer	FALSE	NA	NA	NA
Bc_ctg1_4812	B3	71.59140181	Bcer	T	Bcer	FALSE	NA	NA	NA
Bc_ctg1_4813	B3	0.338227095	Bcer	P	Bcer	FALSE	NA	NA	NA
Bc_ctg1_4814	B3	1.127423651	Bcer	E	Bcer	FALSE	NA	NA	NA
Bc_ctg1_4815	B3	1.014681285	Bcer	S	Bcer	FALSE	NA	NA	NA
Bc_ctg1_4817	B3	1.916620206	Bcer	M	Bcer	FALSE	NA	NA	NA
Bc_ctg1_4818	B3	35.28836026	Bcer	J	Bcer	FALSE	NA	NA	NA
Bc_ctg1_4819	B3	0.112742365	Bcer	NA	Bcer	FALSE	NA	NA	NA
Bc_ctg1_482	B3	1.916620206	Bcer	H	Bcer	FALSE	Bc_Ga0417192_01_rn06	Other	NA
Bc_ctg1_4820	B3	19.72991388	Bcer	M	Bcer	FALSE	NA	NA	NA
Bc_ctg1_4821	B3	2.818559126	Bcer	K	Bcer	FALSE	NA	NA	NA
Bc_ctg1_4822	B3	1.691135476	Bcer	NA	Bcer	FALSE	NA	NA	NA
Bc_ctg1_4823	B3	3.833240412	Bcer	G	Bcer	FALSE	NA	NA	NA
Bc_ctg1_4824	B3	4.735179332	Bcer	K	Bcer	FALSE	NA	NA	NA
Bc_ctg1_4825	B3	12.06343306	Bcer	E	Bcer	FALSE	NA	NA	NA
Bc_ctg1_4826	B3	17.92603604	Bcer	T	Bcer	FALSE	NA	NA	NA
Bc_ctg1_4827	B3	4.960664062	Bcer	T	Bcer	FALSE	NA	NA	NA
Bc_ctg1_4828	B3	2.931301491	Bcer	O	Bcer	FALSE	NA	NA	NA
Bc_ctg1_4829	B3	1.240166016	Bcer	S	Bcer	FALSE	NA	NA	NA
Bc_ctg1_483	B3	4.622436967	Bcer	H	Bcer	FALSE	Bc_Ga0417192_01_rn06	Other	NA
Bc_ctg1_4830	B3	3.495013317	Bcer	S	Bcer	FALSE	NA	NA	NA
Bc_ctg1_4834	B3	7.328253728	Bcer	KT	Bcer	FALSE	NA	NA	NA
Bc_ctg1_4835	B3	7.779223189	Bcer	NA	Bcer	FALSE	NA	NA	NA
Bc_ctg1_4836	B3	1.691135476	Bcer	NA	Bcer	FALSE	NA	NA	NA
Bc_ctg1_4837	B3	83.42935014	Bcer	NA	Bcer	FALSE	NA	NA	NA
Bc_ctg1_4838	B3	0	Bcer	NA	Bcer	FALSE	NA	NA	NA
Bc_ctg1_4839	B3	2.705816761	Bcer	NA	Bcer	FALSE	NA	NA	NA
Bc_ctg1_484	B3	26.04348633	Bcer	H	Bcer	FALSE	Bc_Ga0417192_01_rn06	Other	NA
Bc_ctg1_4840	B3	2.254847301	Bcer	S	Bcer	FALSE	NA	NA	NA
Bc_ctg1_4841	B3	0.112742365	Bcer	NA	Bcer	FALSE	NA	NA	NA
Bc_ctg1_4842	B3	19.16620206	Bcer	E	Bcer	FALSE	NA	NA	NA
Bc_ctg1_4843	B3	20.74459517	Bcer	E	Bcer	FALSE	NA	NA	NA
Bc_ctg1_4844	B3	38.55788885	Bcer	E	Bcer	FALSE	NA	NA	NA
Bc_ctg1_4845	B3	0.112742365	Bcer	NA	Bcer	FALSE	NA	NA	NA
Bc_ctg1_4847	B3	0	Bcer	S	Bcer	FALSE	NA	NA	NA
Bc_ctg1_4848	B3	12.40166016	Bcer	L	Bcer	FALSE	NA	NA	NA
Bc_ctg1_4849	B3	0.90193892	Bcer	S	Bcer	FALSE	NA	NA	NA
Bc_ctg1_485	B3	12.51440252	Bcer	H	Bcer	FALSE	Bc_Ga0417192_01_rn06	Ancillary biosynthesis	None
Bc_ctg1_4850	B3	11.04875178	Bcer	P	Bcer	FALSE	NA	NA	NA
Bc_ctg1_4851	B3	3.044043856	Bcer	D	Bcer	FALSE	NA	NA	NA
Bc_ctg1_4852	B3	31.11689275	Bcer	P	Bcer	FALSE	NA	NA	NA
Bc_ctg1_4853	B3	36.64126864	Bcer	G	Bcer	FALSE	NA	NA	NA
Bc_ctg1_4855	B3	1.240166016	Bcer	M	Bcer	FALSE	NA	NA	NA
Bc_ctg1_4856	B3	0.338227095	Bcer	K	Bcer	FALSE	NA	NA	NA

Bc_ctg1_4857	B3	0.112742365	Bcer	NA	Bcer	FALSE	NA	NA	NA
Bc_ctg1_4858	B3	51.63600319	Bcer	M	Bcer	FALSE	NA	NA	NA
Bc_ctg1_4859	B3	1.127423651	Bcer	M	Bcer	FALSE	NA	NA	NA
Bc_ctg1_486	B3	7.891965554	Bcer	P	Bcer	FALSE	Bc_Ga0417192_01_rn06	Other	NA
Bc_ctg1_4860	B3	0.338227095	Bcer	V	Bcer	FALSE	NA	NA	NA
Bc_ctg1_4861	B3	0.67645419	Bcer	J	Bcer	FALSE	NA	NA	NA
Bc_ctg1_4862	B3	4.284209872	Bcer	S	Bcer	FALSE	Bc_Ga0417192_01_rn34	Other	NA
Bc_ctg1_4863	B3	0	Bcer	NA	Bcer	FALSE	Bc_Ga0417192_01_rn34	Other	NA
Bc_ctg1_4864	B3	4.847921697	Bcer	V	Bcer	FALSE	Bc_Ga0417192_01_rn34	Transport	SMCOG1000
Bc_ctg1_4865	B3	14.43102273	Bcer	V	Bcer	FALSE	Bc_Ga0417192_01_rn34	Transport	SMCOG1065
Bc_ctg1_4866	B3	11.72520597	Bcer	K	Bcer	FALSE	Bc_Ga0417192_01_rn34	Regulation	SMCOG1057
Bc_ctg1_4869	B3	0.112742365	Bcer	NA	Bcer	FALSE	Bc_Ga0417192_01_rn34	Other	NA
Bc_ctg1_487	B3	96.39472212	Bcer	H	Bcer	FALSE	Bc_Ga0417192_01_rn06	Ancillary biosynthesis	None
Bc_ctg1_4870	B3	5.524375888	Bcer	S	Bcer	FALSE	Bc_Ga0417192_01_rn34	Other	NA
Bc_ctg1_4871	B3	26.94542525	Bcer	I	Bcer	FALSE	Bc_Ga0417192_01_rn34	Ancillary biosynthesis	SMCOG1006
Bc_ctg1_4872	B3	33.70996715	Bcer	I	Bcer	FALSE	Bc_Ga0417192_01_rn34	Ancillary biosynthesis	SMCOG1090
Bc_ctg1_4873	B3	0.112742365	Bcer	I	Bcer	FALSE	Bc_Ga0417192_01_rn34	Other	NA
Bc_ctg1_4874	B3	11.72520597	Bcer	E	Bcer	FALSE	Bc_Ga0417192_01_rn34	Core biosynthesis	SMCOG1271
Bc_ctg1_4875	B3	10.59778231	Bcer	I	Bcer	FALSE	Bc_Ga0417192_01_rn34	Ancillary biosynthesis	SMCOG1023
Bc_ctg1_4876	B3	23.2249272	Bcer	I	Bcer	FALSE	Bc_Ga0417192_01_rn34	Ancillary biosynthesis	SMCOG1059
Bc_ctg1_4877	B3	41.37644797	Bcer	I	Bcer	FALSE	Bc_Ga0417192_01_rn34	Core biosynthesis	SMCOG1002
Bc_ctg1_4878	B3	0	Bcer	NA	Bcer	FALSE	Bc_Ga0417192_01_rn34	Other	NA
Bc_ctg1_4879	B3	0.112742365	Bcer	NA	Bcer	FALSE	Bc_Ga0417192_01_rn34	Other	NA
Bc_ctg1_488	B3	61.55733132	Bcer	C	Bcer	FALSE	Bc_Ga0417192_01_rn06	Other	NA
Bc_ctg1_4880	B3	1.240166016	Bcer	J	Bcer	FALSE	Bc_Ga0417192_01_rn34	Ancillary biosynthesis	SMCOG1111
Bc_ctg1_4881	B3	0.338227095	Bcer	NA	Bcer	FALSE	Bc_Ga0417192_01_rn34	Other	NA
Bc_ctg1_4884	B3	5.637118253	Bcer	M	Bcer	FALSE	Bc_Ga0417192_01_rn34	Other	NA
Bc_ctg1_4885	B3	21.19556463	Bcer	T	Bcer	FALSE	Bc_Ga0417192_01_rn34	Regulation	SMCOG1003
Bc_ctg1_4886	B3	11.49972124	Bcer	K	Bcer	FALSE	Bc_Ga0417192_01_rn34	Regulation	SMCOG1008
Bc_ctg1_4887	B3	0.45096946	Bcer	H	Bcer	FALSE	Bc_Ga0417192_01_rn34	Other	NA
Bc_ctg1_4888	B3	1.691135476	Bcer	H	Bcer	FALSE	Bc_Ga0417192_01_rn34	Other	NA
Bc_ctg1_4889	B3	7.891965554	Bcer	S	Bcer	FALSE	Bc_Ga0417192_01_rn34	Other	NA
Bc_ctg1_489	B3	16.23490057	Bcer	S	Bcer	FALSE	Bc_Ga0417192_01_rn06	Other	NA
Bc_ctg1_4890	B3	17.81329368	Bcer	F	Bcer	FALSE	Bc_Ga0417192_01_rn34	Other	NA
Bc_ctg1_4891	B3	26.49445579	Bcer	K	Bcer	FALSE	Bc_Ga0417192_01_rn34	Other	NA
Bc_ctg1_4892	B3	27.84736417	Bcer	NA	Bcer	FALSE	NA	NA	NA
Bc_ctg1_4893	B3	21.64653409	Bcer	E	Bcer	FALSE	NA	NA	NA
Bc_ctg1_4894	B3	0.22548473	Bcer	K	Bcer	FALSE	NA	NA	NA
Bc_ctg1_4895	B3	0.563711825	Bcer	M	Bcer	FALSE	NA	NA	NA
Bc_ctg1_4896	B3	0	Bcer	M	Bcer	FALSE	NA	NA	NA
Bc_ctg1_4897	B3	0.67645419	Bcer	M	Bcer	FALSE	NA	NA	NA
Bc_ctg1_4898	B3	11.04875178	Bcer	S	Bcer	FALSE	NA	NA	NA
Bc_ctg1_4899	B3	8.230192649	Bcer	S	Bcer	FALSE	NA	NA	NA
Bc_ctg1_49	B3	1.803877841	Bcer	S	Bcer	FALSE	NA	NA	NA
Bc_ctg1_490	B3	22.21024592	Bcer	S	Bcer	FALSE	NA	NA	NA
Bc_ctg1_4900	B3	92.11051225	Bcer	L	Bcer	FALSE	NA	NA	NA
Bc_ctg1_4901	B3	32.5825435	Bcer	K	Bcer	FALSE	NA	NA	NA
Bc_ctg1_4902	B3	1.803877841	Bcer	IQ	Bcer	TRUE	NA	NA	NA
Bc_ctg1_4903	B3	0.45096946	Bcer	K	Bcer	FALSE	NA	NA	NA
Bc_ctg1_4904	B3	15.44570401	Bcer	Q	Bcer	TRUE	NA	NA	NA
Bc_ctg1_4906	B3	1.916620206	Bcer	E	Bcer	FALSE	NA	NA	NA
Bc_ctg1_4907	B3	2.367589666	Bcer	M	Bcer	FALSE	NA	NA	NA
Bc_ctg1_4908	B3	4.509694602	Bcer	S	Bcer	FALSE	NA	NA	NA
Bc_ctg1_4909	B3	1.691135476	Bcer	K	Bcer	FALSE	Bc_Ga0417192_01_rn35	Other	NA
Bc_ctg1_491	B3	394.0345659	Bcer	C	Bcer	FALSE	NA	NA	NA
Bc_ctg1_4910	B3	49.26841353	Bcer	E	Bcer	FALSE	Bc_Ga0417192_01_rn35	Other	NA
Bc_ctg1_4911	B3	0.45096946	Bcer	S	Bcer	FALSE	Bc_Ga0417192_01_rn35	Other	NA
Bc_ctg1_4912	B3	6.764541903	Bcer	K	Bcer	FALSE	Bc_Ga0417192_01_rn35	Ancillary biosynthesis	SMCOG1019
Bc_ctg1_4913	B3	0.67645419	Bcer	NA	Bcer	FALSE	Bc_Ga0417192_01_rn35	Other	NA
Bc_ctg1_4914	B3	1.240166016	Bcer	Q	Bcer	TRUE	Bc_Ga0417192_01_rn35	Other	NA
Bc_ctg1_4915	B3	0	Bcer	S	Bcer	FALSE	Bc_Ga0417192_01_rn35	Other	NA
Bc_ctg1_4916	B3	0.112742365	Bcer	NA	Bcer	FALSE	Bc_Ga0417192_01_rn35	Other	NA

Bc_ctg1_4917	B3	2.705816761	Bcer	E	Bcer	FALSE	Bc_Ga0417192_01_rn35	Ancillary biosynthesis	SMCOG1040
Bc_ctg1_4918	B3	6.313572443	Bcer	GM	Bcer	FALSE	Bc_Ga0417192_01_rn35	Core biosynthesis	SMCOG1010
Bc_ctg1_4919	B3	1.465650746	Bcer	S	Bcer	FALSE	Bc_Ga0417192_01_rn35	Other	NA
Bc_ctg1_492	B3	0.22548473	Bcer	EG	Bcer	FALSE	NA	NA	NA
Bc_ctg1_4920	B3	5.411633522	Bcer	Q	Bcer	TRUE	Bc_Ga0417192_01_rn35	Ancillary biosynthesis	SMCOG1248
Bc_ctg1_4921	B3	20.51911044	Bcer	S	Bcer	FALSE	Bc_Ga0417192_01_rn35	Other	NA
Bc_ctg1_4922	B3	4.622436967	Bcer	Q	Bcer	TRUE	Bc_Ga0417192_01_rn35	Other	NA
Bc_ctg1_4923	B3	5.073406427	Bcer	G	Bcer	FALSE	Bc_Ga0417192_01_rn35	Other	NA
Bc_ctg1_4924	B3	0.112742365	Bcer	NA	Bcer	FALSE	Bc_Ga0417192_01_rn35	Other	NA
Bc_ctg1_4925	B3	6.651799538	Bcer	J	Bcer	FALSE	Bc_Ga0417192_01_rn35	Other	NA
Bc_ctg1_4926	B3	0	Bcer	M	Bcer	FALSE	Bc_Ga0417192_01_rn35	Other	NA
Bc_ctg1_4927	B3	12.51440252	Bcer	C	Bcer	FALSE	Bc_Ga0417192_01_rn35	Other	NA
Bc_ctg1_4928	B3	12.17617543	Bcer	T	Bcer	FALSE	Bc_Ga0417192_01_rn35	Regulation	SMCOG1008
Bc_ctg1_4929	B3	22.66121538	Bcer	T	Bcer	FALSE	Bc_Ga0417192_01_rn35	Regulation	SMCOG1003
Bc_ctg1_493	B3	0.338227095	Bcer	E	Bcer	FALSE	NA	NA	NA
Bc_ctg1_4930	B3	0	Bcer	NA	Bcer	FALSE	Bc_Ga0417192_01_rn35	Other	NA
Bc_ctg1_4931	B3	8.004707919	Bcer	NA	Bcer	FALSE	NA	NA	NA
Bc_ctg1_4932	B3	29.08753018	Bcer	S	Bcer	FALSE	NA	NA	NA
Bc_ctg1_4933	B3	9.921328125	Bcer	J	Bcer	FALSE	NA	NA	NA
Bc_ctg1_4934	B3	0	Bcer	NA	Bcer	FALSE	NA	NA	NA
Bc_ctg1_4935	B3	2.029362571	Bcer	V	Bcer	FALSE	NA	NA	NA
Bc_ctg1_4936	B3	6.651799538	Bcer	V	Bcer	FALSE	NA	NA	NA
Bc_ctg1_4937	B3	2.818559126	Bcer	S	Bcer	FALSE	NA	NA	NA
Bc_ctg1_4938	B3	192.5639595	Bcer	NA	Bcer	FALSE	NA	NA	NA
Bc_ctg1_4939	B3	0.338227095	Bcer	D	Bcer	FALSE	NA	NA	NA
Bc_ctg1_494	B3	0	Bcer	S	Bcer	FALSE	NA	NA	NA
Bc_ctg1_4940	B3	2.593074396	Bcer	E	Bcer	FALSE	NA	NA	NA
Bc_ctg1_4941	B3	11.38697887	Bcer	K	Bcer	FALSE	Bc_Ga0417192_01_rn36	Ancillary biosynthesis	SMCOG1019
Bc_ctg1_4942	B3	7.779223189	Bcer	F	Bcer	FALSE	Bc_Ga0417192_01_rn36	Ancillary biosynthesis	None
Bc_ctg1_4943	B3	1.578393111	Bcer	K	Bcer	FALSE	Bc_Ga0417192_01_rn36	Other	NA
Bc_ctg1_4944	B3	84.33128906	Bcer	S	Bcer	FALSE	Bc_Ga0417192_01_rn36	Other	NA
Bc_ctg1_4945	B3	0.563711825	Bcer	NA	Bcer	FALSE	Bc_Ga0417192_01_rn36	Core biosynthesis	None
Bc_ctg1_4946	B3	4.847921697	Bcer	NA	Bcer	FALSE	Bc_Ga0417192_01_rn36	Other	NA
Bc_ctg1_4947	B3	0	Bcer	S	Bcer	FALSE	Bc_Ga0417192_01_rn36	Other	NA
Bc_ctg1_4948	B3	18.48974787	Bcer	L	Bcer	FALSE	Bc_Ga0417192_01_rn36	Other	SMCOG1077
Bc_ctg1_4949	B3	2.705816761	Bcer	NA	Bcer	FALSE	Bc_Ga0417192_01_rn36	Other	NA
Bc_ctg1_495	B3	110.1492907	Bcer	E	Bcer	FALSE	NA	NA	NA
Bc_ctg1_4950	B3	5.298891157	Bcer	NA	Bcer	FALSE	NA	NA	NA
Bc_ctg1_4951	B3	0	Bcer	NA	Bcer	FALSE	NA	NA	NA
Bc_ctg1_4953	B3	12.51440252	Bcer	K	Bcer	FALSE	NA	NA	NA
Bc_ctg1_4954	B3	0	Bcer	K	Bcer	FALSE	NA	NA	NA
Bc_ctg1_4955	B3	0	Bcer	NA	Bcer	FALSE	NA	NA	NA
Bc_ctg1_4956	B3	0	Bcer	NA	Bcer	FALSE	NA	NA	NA
Bc_ctg1_4957	B3	1.916620206	Bcer	L	Bcer	FALSE	NA	NA	NA
Bc_ctg1_4958	B3	0.112742365	Bcer	K	Bcer	FALSE	NA	NA	NA
Bc_ctg1_4959	B3	0.112742365	Bcer	NA	Bcer	FALSE	NA	NA	NA
Bc_ctg1_4961	B3	0	Bcer	NA	Bcer	FALSE	NA	NA	NA
Bc_ctg1_4962	B3	0.112742365	Bcer	S	Bcer	FALSE	NA	NA	NA
Bc_ctg1_4963	B3	7.553738458	Bcer	NA	Bcer	FALSE	NA	NA	NA
Bc_ctg1_4964	B3	0	Bcer	NA	Bcer	FALSE	NA	NA	NA
Bc_ctg1_4965	B3	4.960664062	Bcer	K	Bcer	FALSE	NA	NA	NA
Bc_ctg1_4966	B3	7.328253728	Bcer	NA	Bcer	FALSE	NA	NA	NA
Bc_ctg1_4967	B3	0.112742365	Bcer	NA	Bcer	FALSE	NA	NA	NA
Bc_ctg1_497	B3	4.396952237	Bcer	L	Bcer	FALSE	NA	NA	NA
Bc_ctg1_4970	B3	3.269528587	Bcer	S	Bcer	FALSE	NA	NA	NA
Bc_ctg1_4971	B3	3.382270952	Bcer	K	Bcer	FALSE	NA	NA	NA
Bc_ctg1_4972	B3	248.3714302	Bcer	L	Bcer	FALSE	NA	NA	NA
Bc_ctg1_4973	B3	64.26314808	Bcer	NA	Bcer	FALSE	NA	NA	NA
Bc_ctg1_4974	B3	0	Bcer	NA	Bcer	FALSE	NA	NA	NA
Bc_ctg1_4976	B3	0	Bcer	NA	Bcer	FALSE	NA	NA	NA
Bc_ctg1_4977	B3	0	Bcer	S	Bcer	FALSE	NA	NA	NA
Bc_ctg1_4978	B3	0.67645419	Bcer	S	Bcer	FALSE	NA	NA	NA

Bc_ctg1_4979	B3	0.112742365	Bcer	S	Bcer	FALSE	NA	NA	NA
Bc_ctg1_498	B3	0.789196555	Bcer	S	Bcer	FALSE	NA	NA	NA
Bc_ctg1_4980	B3	0.563711825	Bcer	OU	Bcer	FALSE	NA	NA	NA
Bc_ctg1_4981	B3	1.127423651	Bcer	S	Bcer	FALSE	NA	NA	NA
Bc_ctg1_4982	B3	0	Bcer	NA	Bcer	FALSE	NA	NA	NA
Bc_ctg1_4983	B3	0	Bcer	NA	Bcer	FALSE	NA	NA	NA
Bc_ctg1_4984	B3	0	Bcer	NA	Bcer	FALSE	NA	NA	NA
Bc_ctg1_4985	B3	0	Bcer	NA	Bcer	FALSE	NA	NA	NA
Bc_ctg1_4986	B3	0	Bcer	NA	Bcer	FALSE	NA	NA	NA
Bc_ctg1_4987	B3	0	Bcer	NA	Bcer	FALSE	NA	NA	NA
Bc_ctg1_4988	B3	0.112742365	Bcer	E	Bcer	FALSE	NA	NA	NA
Bc_ctg1_4989	B3	0.112742365	Bcer	S	Bcer	FALSE	NA	NA	NA
Bc_ctg1_499	B3	4.622436967	Bcer	E	Bcer	FALSE	NA	NA	NA
Bc_ctg1_4990	B3	0.22548473	Bcer	M	Bcer	FALSE	NA	NA	NA
Bc_ctg1_4993	B3	0	Bcer	S	Bcer	FALSE	NA	NA	NA
Bc_ctg1_4994	B3	0	Bcer	Q	Bcer	TRUE	NA	NA	NA
Bc_ctg1_4995	B3	3.495013317	Bcer	NA	Bcer	FALSE	NA	NA	NA
Bc_ctg1_4996	B3	58.62602983	Bcer	T	Bcer	FALSE	NA	NA	NA
Bc_ctg1_4997	B3	2.254847301	Bcer	S	Bcer	FALSE	NA	NA	NA
Bc_ctg1_4998	B3	43.18032581	Bcer	S	Bcer	FALSE	NA	NA	NA
Bc_ctg1_4999	B3	4.735179332	Bcer	S	Bcer	FALSE	NA	NA	NA
Bc_ctg1_50	B3	1.127423651	Bcer	S	Bcer	FALSE	NA	NA	NA
Bc_ctg1_5000	B3	2.367589666	Bcer	NA	Bcer	FALSE	NA	NA	NA
Bc_ctg1_5001	B3	0.112742365	Bcer	NA	Bcer	FALSE	NA	NA	NA
Bc_ctg1_5002	B3	12.85262962	Bcer	S	Bcer	FALSE	NA	NA	NA
Bc_ctg1_5003	B3	0.789196555	Bcer	NA	Bcer	FALSE	NA	NA	NA
Bc_ctg1_5004	B3	375.8830451	Bcer	C	Bcer	FALSE	NA	NA	NA
Bc_ctg1_5005	B3	25.14154741	Bcer	EGP	Bcer	FALSE	NA	NA	NA
Bc_ctg1_5006	B3	10.03407049	Bcer	S	Bcer	FALSE	NA	NA	NA
Bc_ctg1_5007	B3	0.112742365	Bcer	S	Bcer	FALSE	NA	NA	NA
Bc_ctg1_5008	B3	4.284209872	Bcer	K	Bcer	FALSE	NA	NA	NA
Bc_ctg1_5009	B3	9.357616299	Bcer	Q	Bcer	TRUE	NA	NA	NA
Bc_ctg1_501	B3	0	Bcer	NA	Bcer	FALSE	NA	NA	NA
Bc_ctg1_5010	B3	0.90193892	Bcer	C	Bcer	FALSE	NA	NA	NA
Bc_ctg1_5011	B3	27.39639471	Bcer	S	Bcer	FALSE	NA	NA	NA
Bc_ctg1_5012	B3	45.99888494	Bcer	T	Bcer	FALSE	NA	NA	NA
Bc_ctg1_5013	B3	69.6747816	Bcer	M	Bcer	FALSE	NA	NA	NA
Bc_ctg1_5014	B3	0.90193892	Bcer	U	Bcer	FALSE	NA	NA	NA
Bc_ctg1_5015	B3	2.931301491	Bcer	CG	Bcer	FALSE	NA	NA	NA
Bc_ctg1_5016	B3	2.142104936	Bcer	M	Bcer	FALSE	NA	NA	NA
Bc_ctg1_5017	B3	1.803877841	Bcer	S	Bcer	FALSE	NA	NA	NA
Bc_ctg1_5018	B3	3.720498047	Bcer	P	Bcer	FALSE	NA	NA	NA
Bc_ctg1_5019	B3	0.789196555	Bcer	P	Bcer	FALSE	NA	NA	NA
Bc_ctg1_502	B3	0	Bcer	Q	Bcer	TRUE	NA	NA	NA
Bc_ctg1_5021	B3	39.00885831	Bcer	T	Bcer	FALSE	NA	NA	NA
Bc_ctg1_5023	B3	4.509694602	Bcer	NA	Bcer	FALSE	NA	NA	NA
Bc_ctg1_5024	B3	0.112742365	Bcer	NA	Bcer	FALSE	NA	NA	NA
Bc_ctg1_5025	B3	0.67645419	Bcer	NA	Bcer	FALSE	NA	NA	NA
Bc_ctg1_5026	B3	0	Bcer	NA	Bcer	FALSE	NA	NA	NA
Bc_ctg1_5028	B3	0	Bcer	F	Bcer	FALSE	NA	NA	NA
Bc_ctg1_503	B3	4.960664062	Bcer	E	Bcer	FALSE	NA	NA	NA
Bc_ctg1_5033	B3	0.789196555	Bcer	D	Bcer	FALSE	NA	NA	NA
Bc_ctg1_5034	B3	0	Bcer	NA	Bcer	FALSE	NA	NA	NA
Bc_ctg1_5035	B3	0.22548473	Bcer	NA	Bcer	FALSE	NA	NA	NA
Bc_ctg1_5036	B3	14.99473455	Bcer	F	Bcer	FALSE	NA	NA	NA
Bc_ctg1_5037	B3	0.112742365	Bcer	NA	Bcer	FALSE	NA	NA	NA
Bc_ctg1_5038	B3	13.41634144	Bcer	KLT	Bcer	FALSE	NA	NA	NA
Bc_ctg1_5039	B3	0.45096946	Bcer	S	Bcer	FALSE	NA	NA	NA
Bc_ctg1_504	B3	1.352908381	Bcer	L	Bcer	FALSE	NA	NA	NA
Bc_ctg1_5040	B3	8.681162109	Bcer	C	Bcer	FALSE	NA	NA	NA
Bc_ctg1_5041	B3	117.8157715	Bcer	I	Bcer	FALSE	NA	NA	NA
Bc_ctg1_5042	B3	16.00941584	Bcer	M	Bcer	FALSE	NA	NA	NA

Bc_ctg1_5043	B3	4.509694602	Bcer	EGP	Bcer	FALSE	NA	NA	NA
Bc_ctg1_5044	B3	5.975345348	Bcer	V	Bcer	FALSE	NA	NA	NA
Bc_ctg1_5045	B3	3.044043856	Bcer	V	Bcer	FALSE	NA	NA	NA
Bc_ctg1_5046	B3	1.691135476	Bcer	S	Bcer	FALSE	NA	NA	NA
Bc_ctg1_5047	B3	1.352908381	Bcer	S	Bcer	FALSE	NA	NA	NA
Bc_ctg1_5048	B3	73.95899147	Bcer	S	Bcer	FALSE	NA	NA	NA
Bc_ctg1_5049	B3	6.764541903	Bcer	K	Bcer	FALSE	NA	NA	NA
Bc_ctg1_505	B3	0	Bcer	L	Bcer	FALSE	NA	NA	NA
Bc_ctg1_5050	B3	7.440996093	Bcer	NA	Bcer	FALSE	NA	NA	NA
Bc_ctg1_5051	B3	114.4335005	Bcer	S	Bcer	FALSE	NA	NA	NA
Bc_ctg1_5052	B3	44.64597656	Bcer	KQ	Bcer	TRUE	NA	NA	NA
Bc_ctg1_5053	B3	0.338227095	Bcer	EGP	Bcer	FALSE	NA	NA	NA
Bc_ctg1_5054	B3	2.480332031	Bcer	K	Bcer	FALSE	NA	NA	NA
Bc_ctg1_5055	B3	2.480332031	Bcer	M	Bcer	FALSE	NA	NA	NA
Bc_ctg1_5056	B3	6.200830078	Bcer	NA	Bcer	FALSE	NA	NA	NA
Bc_ctg1_5058	B3	5.862602983	Bcer	K	Bcer	FALSE	NA	NA	NA
Bc_ctg1_5059	B3	11.72520597	Bcer	C	Bcer	FALSE	NA	NA	NA
Bc_ctg1_506	B3	6.088087713	Bcer	E	Bcer	FALSE	NA	NA	NA
Bc_ctg1_5060	B3	2.593074396	Bcer	S	Bcer	FALSE	NA	NA	NA
Bc_ctg1_5061	B3	0	Bcer	NA	Bcer	FALSE	NA	NA	NA
Bc_ctg1_5062	B3	11.16149414	Bcer	FG	Bcer	FALSE	NA	NA	NA
Bc_ctg1_5063	B3	6.539057173	Bcer	K	Bcer	FALSE	NA	NA	NA
Bc_ctg1_5064	B3	0.45096946	Bcer	EG	Bcer	FALSE	Bc_Ga0417192_01_rn37	Other	NA
Bc_ctg1_5065	B3	3.382270952	Bcer	G	Bcer	FALSE	Bc_Ga0417192_01_rn37	Other	NA
Bc_ctg1_5066	B3	0	Bcer	F	Bcer	FALSE	Bc_Ga0417192_01_rn37	Other	NA
Bc_ctg1_5067	B3	11.04875178	Bcer	K	Bcer	FALSE	Bc_Ga0417192_01_rn37	Other	NA
Bc_ctg1_5068	B3	1.465650746	Bcer	S	Bcer	FALSE	Bc_Ga0417192_01_rn37	Other	NA
Bc_ctg1_5069	B3	10.59778231	Bcer	F	Bcer	FALSE	Bc_Ga0417192_01_rn37	Other	NA
Bc_ctg1_507	B3	1301.38512	Bcer	E	Bcer	FALSE	NA	NA	NA
Bc_ctg1_5070	B3	0	Bcer	NA	Bcer	FALSE	Bc_Ga0417192_01_rn37	Core biosynthesis	None
Bc_ctg1_5071	B3	2.142104936	Bcer	V	Bcer	FALSE	Bc_Ga0417192_01_rn37	Other	NA
Bc_ctg1_5072	B3	3.720498047	Bcer	V	Bcer	FALSE	Bc_Ga0417192_01_rn37	Other	NA
Bc_ctg1_5073	B3	1.803877841	Bcer	V	Bcer	FALSE	Bc_Ga0417192_01_rn37	Other	NA
Bc_ctg1_5075	B3	2.705816761	Bcer	P	Bcer	FALSE	Bc_Ga0417192_01_rn37	Transport	SMCOG1137
Bc_ctg1_5076	B3	0.563711825	Bcer	P	Bcer	FALSE	Bc_Ga0417192_01_rn37	Other	NA
Bc_ctg1_5077	B3	1.578393111	Bcer	K	Bcer	FALSE	Bc_Ga0417192_01_rn37	Other	NA
Bc_ctg1_5078	B3	0	Bcer	F	Bcer	FALSE	Bc_Ga0417192_01_rn37	Other	NA
Bc_ctg1_5079	B3	5.862602983	Bcer	L	Bcer	FALSE	Bc_Ga0417192_01_rn37	Other	NA
Bc_ctg1_508	B3	3.382270952	Bcer	K	Bcer	FALSE	NA	NA	NA
Bc_ctg1_5080	B3	10.37229758	Bcer	M	Bcer	FALSE	NA	NA	NA
Bc_ctg1_5081	B3	10.93600941	Bcer	S	Bcer	FALSE	NA	NA	NA
Bc_ctg1_5082	B3	21.87201882	Bcer	S	Bcer	FALSE	NA	NA	NA
Bc_ctg1_5083	B3	1.240166016	Bcer	L	Bcer	FALSE	NA	NA	NA
Bc_ctg1_5084	B3	9.80858576	Bcer	J	Bcer	FALSE	NA	NA	NA
Bc_ctg1_5085	B3	30.44043856	Bcer	S	Bcer	FALSE	NA	NA	NA
Bc_ctg1_5086	B3	16.4603853	Bcer	P	Bcer	FALSE	NA	NA	NA
Bc_ctg1_5087	B3	9.019389204	Bcer	NA	Bcer	FALSE	NA	NA	NA
Bc_ctg1_5088	B3	2.480332031	Bcer	S	Bcer	FALSE	NA	NA	NA
Bc_ctg1_5089	B3	2.593074396	Bcer	S	Bcer	FALSE	NA	NA	NA
Bc_ctg1_509	B3	2.029362571	Bcer	NA	Bcer	FALSE	NA	NA	NA
Bc_ctg1_5090	B3	36.30304155	Bcer	S	Bcer	FALSE	NA	NA	NA
Bc_ctg1_5091	B3	8.004707919	Bcer	K	Bcer	FALSE	NA	NA	NA
Bc_ctg1_5092	B3	8.681162109	Bcer	S	Bcer	FALSE	NA	NA	NA
Bc_ctg1_5093	B3	11.83794833	Bcer	J	Bcer	FALSE	NA	NA	NA
Bc_ctg1_5094	B3	16.34764293	Bcer	CH	Bcer	FALSE	NA	NA	NA
Bc_ctg1_5095	B3	0	Bcer	V	Bcer	FALSE	NA	NA	NA
Bc_ctg1_5096	B3	0.112742365	Bcer	NA	Bcer	FALSE	NA	NA	NA
Bc_ctg1_5097	B3	68.09638849	Bcer	L	Bcer	FALSE	NA	NA	NA
Bc_ctg1_5098	B3	6.764541903	Bcer	K	Bcer	FALSE	NA	NA	NA
Bc_ctg1_5099	B3	1.240166016	Bcer	NA	Bcer	FALSE	NA	NA	NA
Bc_ctg1_51	B3	1.352908381	Bcer	S	Bcer	FALSE	NA	NA	NA
Bc_ctg1_510	B3	0.112742365	Bcer	K	Bcer	FALSE	NA	NA	NA

Bc_ctg1_5100	B3	3.945982777	Bcer	G	Bcer	FALSE	NA	NA	NA
Bc_ctg1_5101	B3	4.509694602	Bcer	NA	Bcer	FALSE	NA	NA	NA
Bc_ctg1_5102	B3	0.338227095	Bcer	NA	Bcer	FALSE	NA	NA	NA
Bc_ctg1_5103	B3	2.931301491	Bcer	V	Bcer	FALSE	Bc_Ga0417192_01_rn38	Other	NA
Bc_ctg1_5104	B3	2.705816761	Bcer	CG	Bcer	FALSE	Bc_Ga0417192_01_rn38	Ancillary biosynthesis	SMCOG1062
Bc_ctg1_5105	B3	3.495013317	Bcer	KLT	Bcer	FALSE	Bc_Ga0417192_01_rn38	Other	NA
Bc_ctg1_5106	B3	3.044043856	Bcer	L	Bcer	FALSE	Bc_Ga0417192_01_rn38	Other	NA
Bc_ctg1_5107	B3	37.65594993	Bcer	E	Bcer	FALSE	Bc_Ga0417192_01_rn38	Ancillary biosynthesis	SMCOG1103
Bc_ctg1_5108	B3	11.04875178	Bcer	S	Bcer	FALSE	Bc_Ga0417192_01_rn38	Other	NA
Bc_ctg1_5109	B3	0.338227095	Bcer	NA	Bcer	FALSE	Bc_Ga0417192_01_rn38	Other	NA
Bc_ctg1_511	B3	35.85207209	Bcer	S	Bcer	FALSE	NA	NA	NA
Bc_ctg1_5110	B3	4.509694602	Bcer	Q	Bcer	TRUE	Bc_Ga0417192_01_rn38	Other	NA
Bc_ctg1_5111	B3	4.171467507	Bcer	S	Bcer	FALSE	Bc_Ga0417192_01_rn38	Other	NA
Bc_ctg1_5112	B3	4.396952237	Bcer	K	Bcer	FALSE	Bc_Ga0417192_01_rn38	Other	NA
Bc_ctg1_5113	B3	7.102768998	Bcer	S	Bcer	FALSE	Bc_Ga0417192_01_rn38	Ancillary biosynthesis	SMCOG1115
Bc_ctg1_5114	B3	1.465650746	Bcer	T	Bcer	FALSE	Bc_Ga0417192_01_rn38	Regulation	SMCOG1008
Bc_ctg1_5115	B3	4.509694602	Bcer	T	Bcer	FALSE	Bc_Ga0417192_01_rn38	Regulation	SMCOG1003
Bc_ctg1_5116	B3	0.112742365	Bcer	S	Bcer	FALSE	Bc_Ga0417192_01_rn38	Other	NA
Bc_ctg1_5117	B3	0.67645419	Bcer	NA	Bcer	FALSE	Bc_Ga0417192_01_rn38	Other	NA
Bc_ctg1_5118	B3	1.916620206	Bcer	V	Bcer	FALSE	Bc_Ga0417192_01_rn38	Transport	SMCOG1000
Bc_ctg1_5119	B3	0.789196555	Bcer	V	Bcer	FALSE	Bc_Ga0417192_01_rn38	Other	NA
Bc_ctg1_512	B3	34.38642134	Bcer	V	Bcer	FALSE	NA	NA	NA
Bc_ctg1_5120	B3	4.960664062	Bcer	S	Bcer	FALSE	Bc_Ga0417192_01_rn38	Other	NA
Bc_ctg1_5121	B3	30.44043856	Bcer	I	Bcer	FALSE	Bc_Ga0417192_01_rn38	Ancillary biosynthesis	SMCOG1066
Bc_ctg1_5122	B3	5.749860618	Bcer	E	Bcer	FALSE	Bc_Ga0417192_01_rn38	Ancillary biosynthesis	SMCOG1180
Bc_ctg1_5123	B3	13.19085671	Bcer	Q	Bcer	TRUE	Bc_Ga0417192_01_rn38	Core biosynthesis	SMCOG1002
Bc_ctg1_5124	B3	1.014681285	Bcer	C	Bcer	FALSE	Bc_Ga0417192_01_rn38	Ancillary biosynthesis	None
Bc_ctg1_5125	B3	25.93074396	Bcer	G	Bcer	FALSE	Bc_Ga0417192_01_rn38	Core biosynthesis	SMCOG1297
Bc_ctg1_5126	B3	2115.046768	Bcer	E	Bcer	FALSE	Bc_Ga0417192_01_rn38	Other	NA
Bc_ctg1_5127	B3	44.08226474	Bcer	F	Bcer	FALSE	Bc_Ga0417192_01_rn38	Ancillary biosynthesis	SMCOG1283
Bc_ctg1_5128	B3	5.298891157	Bcer	S	Bcer	FALSE	Bc_Ga0417192_01_rn38	Other	NA
Bc_ctg1_5129	B3	2.367589666	Bcer	L	Bcer	FALSE	Bc_Ga0417192_01_rn38	Other	NA
Bc_ctg1_513	B3	12.62714489	Bcer	K	Bcer	FALSE	NA	NA	NA
Bc_ctg1_5130	B3	0	Bcer	E	Bcer	FALSE	Bc_Ga0417192_01_rn38	Other	NA
Bc_ctg1_5131	B3	171.706622	Bcer	U	Bcer	FALSE	Bc_Ga0417192_01_rn38	Other	NA
Bc_ctg1_5132	B3	0.90193892	Bcer	EG	Bcer	FALSE	Bc_Ga0417192_01_rn38	Transport	SMCOG1166
Bc_ctg1_5133	B3	3.044043856	Bcer	K	Bcer	FALSE	Bc_Ga0417192_01_rn38	Ancillary biosynthesis	SMCOG1019
Bc_ctg1_5134	B3	8.793904474	Bcer	C	Bcer	FALSE	Bc_Ga0417192_01_rn38	Ancillary biosynthesis	SMCOG1028
Bc_ctg1_5135	B3	1.240166016	Bcer	I	Bcer	FALSE	Bc_Ga0417192_01_rn38	Ancillary biosynthesis	SMCOG1036
Bc_ctg1_5136	B3	0	Bcer	NA	Bcer	FALSE	Bc_Ga0417192_01_rn38	Other	NA
Bc_ctg1_5137	B3	0.112742365	Bcer	NU	Bcer	FALSE	Bc_Ga0417192_01_rn38	Other	NA
Bc_ctg1_5138	B3	5.186148792	Bcer	NA	Bcer	FALSE	Bc_Ga0417192_01_rn38	Other	NA
Bc_ctg1_5139	B3	4.735179332	Bcer	S	Bcer	FALSE	Bc_Ga0417192_01_rn38	Other	NA
Bc_ctg1_514	B3	5.862602983	Bcer	K	Bcer	FALSE	NA	NA	NA
Bc_ctg1_5140	B3	6.539057173	Bcer	S	Bcer	FALSE	NA	NA	NA
Bc_ctg1_5141	B3	15.89667347	Bcer	J	Bcer	FALSE	NA	NA	NA
Bc_ctg1_5142	B3	4.058725142	Bcer	NA	Bcer	FALSE	NA	NA	NA
Bc_ctg1_5143	B3	0.22548473	Bcer	S	Bcer	FALSE	NA	NA	NA
Bc_ctg1_5144	B3	0	Bcer	H	Bcer	FALSE	NA	NA	NA
Bc_ctg1_5145	B3	0.112742365	Bcer	M	Bcer	FALSE	NA	NA	NA
Bc_ctg1_5147	B3	5.298891157	Bcer	C	Bcer	FALSE	NA	NA	NA
Bc_ctg1_5148	B3	5.637118253	Bcer	V	Bcer	FALSE	NA	NA	NA
Bc_ctg1_5149	B3	53.5526234	Bcer	E	Bcer	FALSE	NA	NA	NA
Bc_ctg1_515	B3	1.240166016	Bcer	S	Bcer	FALSE	NA	NA	NA
Bc_ctg1_5150	B3	29.87672674	Bcer	S	Bcer	FALSE	NA	NA	NA
Bc_ctg1_5151	B3	51.52326083	Bcer	E	Bcer	FALSE	NA	NA	NA
Bc_ctg1_5152	B3	8.681162109	Bcer	K	Bcer	FALSE	NA	NA	NA
Bc_ctg1_5153	B3	0.563711825	Bcer	F	Bcer	FALSE	NA	NA	NA
Bc_ctg1_5154	B3	0	Bcer	Q	Bcer	TRUE	NA	NA	NA
Bc_ctg1_5155	B3	0.45096946	Bcer	Q	Bcer	TRUE	NA	NA	NA
Bc_ctg1_5156	B3	2.367589666	Bcer	G	Bcer	FALSE	NA	NA	NA
Bc_ctg1_5157	B3	0	Bcer	S	Bcer	FALSE	NA	NA	NA

Bc_ctg1_5158	B3	19.27894442	Bcer	KQ	Bcer	TRUE	NA	NA	NA
Bc_ctg1_5159	B3	0.563711825	Bcer	NA	Bcer	FALSE	NA	NA	NA
Bc_ctg1_516	B3	16.4603853	Bcer	S	Bcer	FALSE	NA	NA	NA
Bc_ctg1_5160	B3	1.014681285	Bcer	F	Bcer	FALSE	NA	NA	NA
Bc_ctg1_5161	B3	1.691135476	Bcer	K	Bcer	FALSE	NA	NA	NA
Bc_ctg1_5162	B3	3.382270952	Bcer	K	Bcer	FALSE	NA	NA	NA
Bc_ctg1_5163	B3	3.720498047	Bcer	NA	Bcer	FALSE	NA	NA	NA
Bc_ctg1_5164	B3	0.45096946	Bcer	Q	Bcer	TRUE	NA	NA	NA
Bc_ctg1_5165	B3	14.09279563	Bcer	K	Bcer	FALSE	NA	NA	NA
Bc_ctg1_5166	B3	6.877284268	Bcer	S	Bcer	FALSE	NA	NA	NA
Bc_ctg1_5167	B3	0.45096946	Bcer	NA	Bcer	FALSE	NA	NA	NA
Bc_ctg1_5168	B3	0.112742365	Bcer	C	Bcer	FALSE	NA	NA	NA
Bc_ctg1_5169	B3	0.112742365	Bcer	C	Bcer	FALSE	NA	NA	NA
Bc_ctg1_517	B3	333.7174006	Bcer	L	Bcer	FALSE	NA	NA	NA
Bc_ctg1_5170	B3	0	Bcer	C	Bcer	FALSE	NA	NA	NA
Bc_ctg1_5171	B3	0	Bcer	C	Bcer	FALSE	NA	NA	NA
Bc_ctg1_5173	B3	11.16149414	Bcer	S	Bcer	FALSE	NA	NA	NA
Bc_ctg1_5174	B3	5.411633522	Bcer	S	Bcer	FALSE	NA	NA	NA
Bc_ctg1_5175	B3	2.931301491	Bcer	S	Bcer	FALSE	NA	NA	NA
Bc_ctg1_5176	B3	0.22548473	Bcer	S	Bcer	FALSE	NA	NA	NA
Bc_ctg1_5177	B3	0.45096946	Bcer	L	Bcer	FALSE	NA	NA	NA
Bc_ctg1_5178	B3	0.563711825	Bcer	S	Bcer	FALSE	NA	NA	NA
Bc_ctg1_5179	B3	2.029362571	Bcer	T	Bcer	FALSE	NA	NA	NA
Bc_ctg1_518	B3	165.6185343	Bcer	L	Bcer	FALSE	NA	NA	NA
Bc_ctg1_5180	B3	1.014681285	Bcer	FG	Bcer	FALSE	NA	NA	NA
Bc_ctg1_5182	B3	0.45096946	Bcer	P	Bcer	FALSE	NA	NA	NA
Bc_ctg1_5183	B3	0.338227095	Bcer	E	Bcer	FALSE	NA	NA	NA
Bc_ctg1_5184	B3	0	Bcer	E	Bcer	FALSE	NA	NA	NA
Bc_ctg1_5185	B3	3.833240412	Bcer	OU	Bcer	FALSE	NA	NA	NA
Bc_ctg1_5186	B3	6.764541903	Bcer	K	Bcer	FALSE	NA	NA	NA
Bc_ctg1_5187	B3	0	Bcer	C	Bcer	FALSE	NA	NA	NA
Bc_ctg1_5188	B3	0	Bcer	G	Bcer	FALSE	NA	NA	NA
Bc_ctg1_5189	B3	17.36232422	Bcer	S	Bcer	FALSE	NA	NA	NA
Bc_ctg1_519	B3	44.1950071	Bcer	S	Bcer	FALSE	NA	NA	NA
Bc_ctg1_5190	B3	635.4159694	Bcer	S	Bcer	FALSE	NA	NA	NA
Bc_ctg1_5191	B3	11.27423651	Bcer	CG	Bcer	FALSE	NA	NA	NA
Bc_ctg1_5192	B3	2.818559126	Bcer	S	Bcer	FALSE	NA	NA	NA
Bc_ctg1_5193	B3	3.720498047	Bcer	F	Bcer	FALSE	NA	NA	NA
Bc_ctg1_5194	B3	3.044043856	Bcer	T	Bcer	FALSE	NA	NA	NA
Bc_ctg1_5195	B3	7.102768998	Bcer	Q	Bcer	TRUE	NA	NA	NA
Bc_ctg1_5196	B3	3.833240412	Bcer	S	Bcer	FALSE	NA	NA	NA
Bc_ctg1_5197	B3	4.622436967	Bcer	K	Bcer	FALSE	NA	NA	NA
Bc_ctg1_5198	B3	4.960664062	Bcer	K	Bcer	FALSE	NA	NA	NA
Bc_ctg1_5199	B3	2.705816761	Bcer	NA	Bcer	FALSE	NA	NA	NA
Bc_ctg1_520	B3	474.081645	Bcer	O	Bcer	FALSE	NA	NA	NA
Bc_ctg1_5200	B3	11.27423651	Bcer	M	Bcer	FALSE	NA	NA	NA
Bc_ctg1_5201	B3	6.313572443	Bcer	C	Bcer	FALSE	NA	NA	NA
Bc_ctg1_5202	B3	0.112742365	Bcer	K	Bcer	FALSE	NA	NA	NA
Bc_ctg1_5203	B3	3.720498047	Bcer	S	Bcer	FALSE	NA	NA	NA
Bc_ctg1_5204	B3	5.637118253	Bcer	NA	Bcer	FALSE	NA	NA	NA
Bc_ctg1_5205	B3	34.04819425	Bcer	L	Bcer	FALSE	NA	NA	NA
Bc_ctg1_5206	B3	0.112742365	Bcer	S	Bcer	FALSE	NA	NA	NA
Bc_ctg1_5207	B3	17.24958185	Bcer	S	Bcer	FALSE	NA	NA	NA
Bc_ctg1_5208	B3	79.59610973	Bcer	L	Bcer	FALSE	NA	NA	NA
Bc_ctg1_521	B3	57.38586381	Bcer	T	Bcer	FALSE	NA	NA	NA
Bc_ctg1_5210	B3	0.22548473	Bcer	M	Bcer	FALSE	NA	NA	NA
Bc_ctg1_5211	B3	2.931301491	Bcer	NA	Bcer	FALSE	NA	NA	NA
Bc_ctg1_5212	B3	3.044043856	Bcer	NA	Bcer	FALSE	NA	NA	NA
Bc_ctg1_5213	B3	0	Bcer	NA	Bcer	FALSE	NA	NA	NA
Bc_ctg1_5214	B3	1.352908381	Bcer	S	Bcer	FALSE	NA	NA	NA
Bc_ctg1_5215	B3	0	Bcer	S	Bcer	FALSE	NA	NA	NA
Bc_ctg1_5216	B3	4.847921697	Bcer	E	Bcer	FALSE	NA	NA	NA

Bc_ctg1_5217	B3	357.1678125	Bcer	C	Bcer	FALSE	NA	NA	NA
Bc_ctg1_5218	B3	32.13157404	Bcer	S	Bcer	FALSE	NA	NA	NA
Bc_ctg1_5219	B3	14.43102273	Bcer	K	Bcer	FALSE	NA	NA	NA
Bc_ctg1_522	B3	2.593074396	Bcer	C	Bcer	FALSE	NA	NA	NA
Bc_ctg1_5220	B3	8.004707919	Bcer	H	Bcer	FALSE	NA	NA	NA
Bc_ctg1_5221	B3	0.22548473	Bcer	S	Bcer	FALSE	NA	NA	NA
Bc_ctg1_5222	B3	1.014681285	Bcer	E	Bcer	FALSE	NA	NA	NA
Bc_ctg1_5223	B3	2.705816761	Bcer	C	Bcer	FALSE	NA	NA	NA
Bc_ctg1_5224	B3	1.127423651	Bcer	E	Bcer	FALSE	NA	NA	NA
Bc_ctg1_5225	B3	0.112742365	Bcer	E	Bcer	FALSE	NA	NA	NA
Bc_ctg1_5226	B3	0.22548473	Bcer	E	Bcer	FALSE	NA	NA	NA
Bc_ctg1_5227	B3	0	Bcer	E	Bcer	FALSE	NA	NA	NA
Bc_ctg1_5228	B3	10.37229758	Bcer	KT	Bcer	FALSE	NA	NA	NA
Bc_ctg1_5229	B3	0.90193892	Bcer	C	Bcer	FALSE	NA	NA	NA
Bc_ctg1_523	B3	81.73821466	Bcer	F	Bcer	FALSE	NA	NA	NA
Bc_ctg1_5230	B3	1.916620206	Bcer	C	Bcer	FALSE	NA	NA	NA
Bc_ctg1_5231	B3	6.426314808	Bcer	C	Bcer	FALSE	NA	NA	NA
Bc_ctg1_5232	B3	11.16149414	Bcer	S	Bcer	FALSE	NA	NA	NA
Bc_ctg1_5233	B3	52.65068448	Bcer	S	Bcer	FALSE	NA	NA	NA
Bc_ctg1_5234	B3	50.959549	Bcer	NA	Bcer	FALSE	NA	NA	NA
Bc_ctg1_5236	B3	69.78752397	Bcer	S	Bcer	FALSE	NA	NA	NA
Bc_ctg1_5238	B3	17.36232422	Bcer	S	Bcer	FALSE	NA	NA	NA
Bc_ctg1_5239	B3	0	Bcer	NA	Bcer	FALSE	NA	NA	NA
Bc_ctg1_524	B3	117.8157715	Bcer	I	Bcer	FALSE	NA	NA	NA
Bc_ctg1_5240	B3	36.41578391	Bcer	M	Bcer	FALSE	NA	NA	NA
Bc_ctg1_5241	B3	20.40636807	Bcer	E	Bcer	FALSE	NA	NA	NA
Bc_ctg1_5242	B3	11.04875178	Bcer	M	Bcer	FALSE	NA	NA	NA
Bc_ctg1_5243	B3	7.440996093	Bcer	M	Bcer	FALSE	NA	NA	NA
Bc_ctg1_5244	B3	7.102768998	Bcer	S	Bcer	FALSE	NA	NA	NA
Bc_ctg1_5245	B3	0.563711825	Bcer	D	Bcer	FALSE	NA	NA	NA
Bc_ctg1_5246	B3	2.705816761	Bcer	C	Bcer	FALSE	NA	NA	NA
Bc_ctg1_5247	B3	5.749860618	Bcer	K	Bcer	FALSE	NA	NA	NA
Bc_ctg1_5248	B3	1.578393111	Bcer	S	Bcer	FALSE	NA	NA	NA
Bc_ctg1_5249	B3	0.90193892	Bcer	NA	Bcer	FALSE	NA	NA	NA
Bc_ctg1_525	B3	40.69999378	Bcer	S	Bcer	FALSE	NA	NA	NA
Bc_ctg1_5250	B3	0.789196555	Bcer	NA	Bcer	FALSE	NA	NA	NA
Bc_ctg1_5251	B3	25.70525923	Bcer	Q	Bcer	TRUE	NA	NA	NA
Bc_ctg1_5252	B3	0.563711825	Bcer	S	Bcer	FALSE	NA	NA	NA
Bc_ctg1_5253	B3	27.28365234	Bcer	M	Bcer	FALSE	NA	NA	NA
Bc_ctg1_5254	B3	5.073406427	Bcer	T	Bcer	FALSE	NA	NA	NA
Bc_ctg1_5255	B3	2.254847301	Bcer	T	Bcer	FALSE	NA	NA	NA
Bc_ctg1_5256	B3	0	Bcer	NA	Bcer	FALSE	NA	NA	NA
Bc_ctg1_5257	B3	0.67645419	Bcer	K	Bcer	FALSE	NA	NA	NA
Bc_ctg1_5258	B3	15.33296165	Bcer	S	Bcer	FALSE	NA	NA	NA
Bc_ctg1_5259	B3	14.54376509	Bcer	F	Bcer	FALSE	NA	NA	NA
Bc_ctg1_526	B3	75.08641512	Bcer	S	Bcer	FALSE	NA	NA	NA
Bc_ctg1_5260	B3	7.328253728	Bcer	F	Bcer	FALSE	NA	NA	NA
Bc_ctg1_5261	B3	13.19085671	Bcer	S	Bcer	FALSE	NA	NA	NA
Bc_ctg1_5262	B3	12.17617543	Bcer	M	Bcer	FALSE	NA	NA	NA
Bc_ctg1_5263	B3	28.0728489	Bcer	E	Bcer	FALSE	NA	NA	NA
Bc_ctg1_5264	B3	44.30774947	Bcer	S	Bcer	FALSE	NA	NA	NA
Bc_ctg1_5265	B3	1.240166016	Bcer	S	Bcer	FALSE	NA	NA	NA
Bc_ctg1_5267	B3	0	Bcer	S	Bcer	FALSE	NA	NA	NA
Bc_ctg1_5268	B3	14.65650746	Bcer	K	Bcer	FALSE	NA	NA	NA
Bc_ctg1_5269	B3	9.80858576	Bcer	K	Bcer	FALSE	NA	NA	NA
Bc_ctg1_527	B3	1.465650746	Bcer	M	Bcer	FALSE	NA	NA	NA
Bc_ctg1_5270	B3	0.112742365	Bcer	NA	Bcer	FALSE	NA	NA	NA
Bc_ctg1_5271	B3	76.10109641	Bcer	NA	Bcer	FALSE	NA	NA	NA
Bc_ctg1_5272	B3	187.9415225	Bcer	EGP	Bcer	FALSE	NA	NA	NA
Bc_ctg1_5273	B3	90.75760387	Bcer	M	Bcer	FALSE	NA	NA	NA
Bc_ctg1_5274	B3	176.7800284	Bcer	M	Bcer	FALSE	NA	NA	NA
Bc_ctg1_5275	B3	314.2129714	Bcer	K	Bcer	FALSE	NA	NA	NA

Bc_ctg1_5276	B3	8.681162109	Bcer	S	Bcer	FALSE	NA	NA	NA
Bc_ctg1_5277	B3	0.563711825	Bcer	EGP	Bcer	FALSE	NA	NA	NA
Bc_ctg1_5278	B3	12.85262962	Bcer	G	Bcer	FALSE	NA	NA	NA
Bc_ctg1_5279	B3	9.244873934	Bcer	NA	Bcer	FALSE	NA	NA	NA
Bc_ctg1_5280	B3	0.112742365	Bcer	NA	Bcer	FALSE	NA	NA	NA
Bc_ctg1_5281	B3	46.33711204	Bcer	NA	Bcer	FALSE	NA	NA	NA
Bc_ctg1_5282	B3	0	Bcer	S	Bcer	FALSE	NA	NA	NA
Bc_ctg1_5283	B3	1.352908381	Bcer	F	Bcer	FALSE	NA	NA	NA
Bc_ctg1_5284	B3	0.112742365	Bcer	S	Bcer	FALSE	NA	NA	NA
Bc_ctg1_5285	B3	27.39639471	Bcer	Q	Bcer	TRUE	NA	NA	NA
Bc_ctg1_5286	B3	0.22548473	Bcer	NA	Bcer	FALSE	NA	NA	NA
Bc_ctg1_5289	B3	0.67645419	Bcer	NA	Bcer	FALSE	NA	NA	NA
Bc_ctg1_5290	B3	26.83268288	Bcer	EGP	Bcer	FALSE	NA	NA	NA
Bc_ctg1_5291	B3	0.112742365	Bcer	NA	Bcer	FALSE	NA	NA	NA
Bc_ctg1_5292	B3	5.862602983	Bcer	NA	Bcer	FALSE	NA	NA	NA
Bc_ctg1_5293	B3	276.6697638	Bcer	E	Bcer	FALSE	NA	NA	NA
Bc_ctg1_5294	B3	14.205538	Bcer	I	Bcer	FALSE	NA	NA	NA
Bc_ctg1_5295	B3	29.42575728	Bcer	H	Bcer	FALSE	NA	NA	NA
Bc_ctg1_5296	B3	0.67645419	Bcer	J	Bcer	FALSE	NA	NA	NA
Bc_ctg1_5297	B3	3.156786221	Bcer	O	Bcer	FALSE	NA	NA	NA
Bc_ctg1_5298	B3	3.833240412	Bcer	NA	Bcer	FALSE	NA	NA	NA
Bc_ctg1_5299	B3	1.803877841	Bcer	V	Bcer	FALSE	NA	NA	NA
Bc_ctg1_53	B3	0.22548473	Bcer	E	Bcer	FALSE	NA	NA	NA
Bc_ctg1_5300	B3	0	Bcer	K	Bcer	FALSE	NA	NA	NA
Bc_ctg1_5301	B3	0.112742365	Bcer	NA	Bcer	FALSE	NA	NA	NA
Bc_ctg1_5302	B3	0.338227095	Bcer	S	Bcer	FALSE	NA	NA	NA
Bc_ctg1_5304	B3	0	Bcer	NA	Bcer	FALSE	NA	NA	NA
Bc_ctg1_5305	B3	0	Bcer	NA	Bcer	FALSE	NA	NA	NA
Bc_ctg1_5307	B3	0	Bcer	S	Bcer	FALSE	NA	NA	NA
Bc_ctg1_5309	B3	65.72879883	Bcer	S	Bcer	FALSE	NA	NA	NA
Bc_ctg1_5310	B3	63.02298206	Bcer	S	Bcer	FALSE	NA	NA	NA
Bc_ctg1_5311	B3	9.695843394	Bcer	C	Bcer	FALSE	NA	NA	NA
Bc_ctg1_5312	B3	11.16149414	Bcer	P	Bcer	FALSE	NA	NA	NA
Bc_ctg1_5313	B3	6.877284268	Bcer	P	Bcer	FALSE	NA	NA	NA
Bc_ctg1_5314	B3	2.254847301	Bcer	P	Bcer	FALSE	NA	NA	NA
Bc_ctg1_5315	B3	5.298891157	Bcer	S	Bcer	FALSE	NA	NA	NA
Bc_ctg1_5317	B3	18.15152077	Bcer	NA	Bcer	FALSE	NA	NA	NA
Bc_ctg1_5318	B3	9.244873934	Bcer	S	Bcer	FALSE	NA	NA	NA
Bc_ctg1_5319	B3	11.04875178	Bcer	H	Bcer	FALSE	NA	NA	NA
Bc_ctg1_532	B3	14.76924982	Bcer	L	Bcer	FALSE	NA	NA	NA
Bc_ctg1_5320	B3	20.06814098	Bcer	V	Bcer	FALSE	NA	NA	NA
Bc_ctg1_5321	B3	0.112742365	Bcer	K	Bcer	FALSE	NA	NA	NA
Bc_ctg1_5322	B3	26.94542525	Bcer	EG	Bcer	FALSE	NA	NA	NA
Bc_ctg1_5323	B3	0.22548473	Bcer	NA	Bcer	FALSE	NA	NA	NA
Bc_ctg1_5324	B3	9.695843394	Bcer	K	Bcer	FALSE	NA	NA	NA
Bc_ctg1_5325	B3	4.171467507	Bcer	J	Bcer	FALSE	NA	NA	NA
Bc_ctg1_5326	B3	2.142104936	Bcer	S	Bcer	FALSE	NA	NA	NA
Bc_ctg1_5327	B3	12.06343306	Bcer	L	Bcer	FALSE	NA	NA	NA
Bc_ctg1_5328	B3	0	Bcer	E	Bcer	FALSE	NA	NA	NA
Bc_ctg1_5329	B3	0	Bcer	E	Bcer	FALSE	NA	NA	NA
Bc_ctg1_533	B3	0.338227095	Bcer	CO	Bcer	FALSE	NA	NA	NA
Bc_ctg1_5330	B3	1.127423651	Bcer	E	Bcer	FALSE	NA	NA	NA
Bc_ctg1_5331	B3	1.014681285	Bcer	S	Bcer	FALSE	NA	NA	NA
Bc_ctg1_5332	B3	25.93074396	Bcer	S	Bcer	FALSE	NA	NA	NA
Bc_ctg1_5333	B3	148.0307253	Bcer	G	Bcer	FALSE	NA	NA	NA
Bc_ctg1_5334	B3	0.112742365	Bcer	S	Bcer	FALSE	NA	NA	NA
Bc_ctg1_5335	B3	2.480332031	Bcer	P	Bcer	FALSE	NA	NA	NA
Bc_ctg1_5336	B3	1383.010592	Bcer	M	Bcer	FALSE	NA	NA	NA
Bc_ctg1_5337	B3	680.061946	Bcer	S	Bcer	FALSE	NA	NA	NA
Bc_ctg1_5338	B3	14.99473455	Bcer	S	Bcer	FALSE	NA	NA	NA
Bc_ctg1_5339	B3	72.04237127	Bcer	K	Bcer	FALSE	NA	NA	NA
Bc_ctg1_534	B3	0.67645419	Bcer	NA	Bcer	FALSE	NA	NA	NA

Bc_ctg1_5340	B3	61.21910422	Bcer	S	Bcer	FALSE	NA	NA	NA
Bc_ctg1_5341	B3	184.1082821	Bcer	E	Bcer	FALSE	NA	NA	NA
Bc_ctg1_5342	B3	63.02298206	Bcer	E	Bcer	FALSE	NA	NA	NA
Bc_ctg1_5343	B3	86.24790926	Bcer	E	Bcer	FALSE	NA	NA	NA
Bc_ctg1_5344	B3	36.52852628	Bcer	E	Bcer	FALSE	NA	NA	NA
Bc_ctg1_5345	B3	161.5598091	Bcer	E	Bcer	FALSE	NA	NA	NA
Bc_ctg1_5346	B3	77.79223189	Bcer	K	Bcer	FALSE	NA	NA	NA
Bc_ctg1_5347	B3	0.338227095	Bcer	S	Bcer	FALSE	NA	NA	NA
Bc_ctg1_5348	B3	16.1221582	Bcer	Q	Bcer	TRUE	NA	NA	NA
Bc_ctg1_5349	B3	5.411633522	Bcer	J	Bcer	FALSE	NA	NA	NA
Bc_ctg1_535	B3	0.90193892	Bcer	V	Bcer	FALSE	NA	NA	NA
Bc_ctg1_5350	B3	4.622436967	Bcer	S	Bcer	FALSE	NA	NA	NA
Bc_ctg1_5351	B3	0	Bcer	S	Bcer	FALSE	NA	NA	NA
Bc_ctg1_5352	B3	0.563711825	Bcer	NA	Bcer	FALSE	NA	NA	NA
Bc_ctg1_5353	B3	3.269528587	Bcer	S	Bcer	FALSE	NA	NA	NA
Bc_ctg1_5354	B3	18.15152077	Bcer	M	Bcer	FALSE	NA	NA	NA
Bc_ctg1_5355	B3	0	Bcer	S	Bcer	FALSE	NA	NA	NA
Bc_ctg1_5356	B3	5.524375888	Bcer	E	Bcer	FALSE	NA	NA	NA
Bc_ctg1_5357	B3	2.142104936	Bcer	C	Bcer	FALSE	NA	NA	NA
Bc_ctg1_5358	B3	1.014681285	Bcer	K	Bcer	FALSE	NA	NA	NA
Bc_ctg1_5359	B3	2.593074396	Bcer	NA	Bcer	FALSE	NA	NA	NA
Bc_ctg1_536	B3	8585.894811	Bcer	C	Bcer	FALSE	NA	NA	NA
Bc_ctg1_5360	B3	6.539057173	Bcer	NA	Bcer	FALSE	NA	NA	NA
Bc_ctg1_5361	B3	53.43988103	Bcer	S	Bcer	FALSE	NA	NA	NA
Bc_ctg1_5362	B3	1033.283776	Bcer	S	Bcer	FALSE	NA	NA	NA
Bc_ctg1_5363	B3	1.916620206	Bcer	G	Bcer	FALSE	NA	NA	NA
Bc_ctg1_5364	B3	0	Bcer	G	Bcer	FALSE	NA	NA	NA
Bc_ctg1_5365	B3	0	Bcer	G	Bcer	FALSE	NA	NA	NA
Bc_ctg1_5366	B3	0.22548473	Bcer	G	Bcer	FALSE	NA	NA	NA
Bc_ctg1_5367	B3	0	Bcer	K	Bcer	FALSE	NA	NA	NA
Bc_ctg1_5368	B3	0.112742365	Bcer	G	Bcer	FALSE	NA	NA	NA
Bc_ctg1_5369	B3	0.112742365	Bcer	G	Bcer	FALSE	NA	NA	NA
Bc_ctg1_537	B3	0	Bcer	S	Bcer	FALSE	NA	NA	NA
Bc_ctg1_5370	B3	0.112742365	Bcer	G	Bcer	FALSE	NA	NA	NA
Bc_ctg1_5371	B3	1.465650746	Bcer	E	Bcer	FALSE	NA	NA	NA
Bc_ctg1_5372	B3	2.142104936	Bcer	S	Bcer	FALSE	NA	NA	NA
Bc_ctg1_5373	B3	8.004707919	Bcer	EGP	Bcer	FALSE	NA	NA	NA
Bc_ctg1_5374	B3	5.073406427	Bcer	K	Bcer	FALSE	NA	NA	NA
Bc_ctg1_5375	B3	0.112742365	Bcer	F	Bcer	FALSE	NA	NA	NA
Bc_ctg1_5376	B3	27.05816761	Bcer	E	Bcer	FALSE	NA	NA	NA
Bc_ctg1_5377	B3	12.06343306	Bcer	E	Bcer	FALSE	NA	NA	NA
Bc_ctg1_5378	B3	4.058725142	Bcer	E	Bcer	FALSE	NA	NA	NA
Bc_ctg1_5379	B3	27.17090998	Bcer	E	Bcer	FALSE	NA	NA	NA
Bc_ctg1_538	B3	3.720498047	Bcer	G	Bcer	FALSE	NA	NA	NA
Bc_ctg1_5380	B3	8.568419744	Bcer	K	Bcer	FALSE	NA	NA	NA
Bc_ctg1_5381	B3	3.833240412	Bcer	E	Bcer	FALSE	NA	NA	NA
Bc_ctg1_5382	B3	4.622436967	Bcer	E	Bcer	FALSE	NA	NA	NA
Bc_ctg1_5383	B3	0	Bcer	E	Bcer	FALSE	NA	NA	NA
Bc_ctg1_5384	B3	214.2104936	Bcer	I	Bcer	FALSE	NA	NA	NA
Bc_ctg1_5385	B3	15.22021928	Bcer	V	Bcer	FALSE	NA	NA	NA
Bc_ctg1_5386	B3	0.67645419	Bcer	V	Bcer	FALSE	NA	NA	NA
Bc_ctg1_5387	B3	1.465650746	Bcer	P	Bcer	FALSE	NA	NA	NA
Bc_ctg1_5388	B3	5.073406427	Bcer	S	Bcer	FALSE	NA	NA	NA
Bc_ctg1_5389	B3	1.465650746	Bcer	K	Bcer	FALSE	NA	NA	NA
Bc_ctg1_539	B3	1.465650746	Bcer	NA	Bcer	FALSE	NA	NA	NA
Bc_ctg1_5390	B3	11.16149414	Bcer	L	Bcer	FALSE	NA	NA	NA
Bc_ctg1_5391	B3	0.45096946	Bcer	NA	Bcer	FALSE	NA	NA	NA
Bc_ctg1_5392	B3	15.33296165	Bcer	S	Bcer	FALSE	NA	NA	NA
Bc_ctg1_5393	B3	29.65124201	Bcer	F	Bcer	FALSE	NA	NA	NA
Bc_ctg1_5394	B3	16.91135476	Bcer	J	Bcer	FALSE	NA	NA	NA
Bc_ctg1_5395	B3	22.09750355	Bcer	E	Bcer	FALSE	NA	NA	NA
Bc_ctg1_5397	B3	0.112742365	Bcer	NA	Bcer	FALSE	NA	NA	NA

Bc_ctg1_5398	B3	9.019389204	Bcer	S	Bcer	FALSE	NA	NA	NA
Bc_ctg1_5399	B3	3.382270952	Bcer	K	Bcer	FALSE	NA	NA	NA
Bc_ctg1_54	B3	0.338227095	Bcer	P	Bcer	FALSE	NA	NA	NA
Bc_ctg1_5400	B3	0.563711825	Bcer	S	Bcer	FALSE	NA	NA	NA
Bc_ctg1_5401	B3	1.352908381	Bcer	S	Bcer	FALSE	NA	NA	NA
Bc_ctg1_5402	B3	3.720498047	Bcer	NA	Bcer	FALSE	NA	NA	NA
Bc_ctg1_5403	B3	629.4406241	Bcer	EGP	Bcer	FALSE	NA	NA	NA
Bc_ctg1_5404	B3	1.578393111	Bcer	E	Bcer	FALSE	NA	NA	NA
Bc_ctg1_5405	B3	4.509694602	Bcer	C	Bcer	FALSE	NA	NA	NA
Bc_ctg1_5406	B3	0.22548473	Bcer	S	Bcer	FALSE	NA	NA	NA
Bc_ctg1_5407	B3	0.112742365	Bcer	NA	Bcer	FALSE	NA	NA	NA
Bc_ctg1_5408	B3	8.230192649	Bcer	F	Bcer	FALSE	NA	NA	NA
Bc_ctg1_5409	B3	30.89140802	Bcer	V	Bcer	FALSE	NA	NA	NA
Bc_ctg1_541	B3	0.22548473	Bcer	O	Bcer	FALSE	NA	NA	NA
Bc_ctg1_5410	B3	0.563711825	Bcer	NA	Bcer	FALSE	NA	NA	NA
Bc_ctg1_5411	B3	5.749860618	Bcer	S	Bcer	FALSE	NA	NA	NA
Bc_ctg1_5412	B3	19.39168679	Bcer	C	Bcer	FALSE	NA	NA	NA
Bc_ctg1_5413	B3	7.553738458	Bcer	K	Bcer	FALSE	NA	NA	NA
Bc_ctg1_5414	B3	38.55788885	Bcer	J	Bcer	FALSE	NA	NA	NA
Bc_ctg1_5415	B3	0	Bcer	F	Bcer	FALSE	NA	NA	NA
Bc_ctg1_5416	B3	2.367589666	Bcer	S	Bcer	FALSE	NA	NA	NA
Bc_ctg1_5417	B3	11.72520597	Bcer	S	Bcer	FALSE	NA	NA	NA
Bc_ctg1_5418	B3	11.9506907	Bcer	V	Bcer	FALSE	NA	NA	NA
Bc_ctg1_5419	B3	6.539057173	Bcer	K	Bcer	FALSE	NA	NA	NA
Bc_ctg1_542	B3	40.13628196	Bcer	EGP	Bcer	FALSE	NA	NA	NA
Bc_ctg1_5421	B3	10.37229758	Bcer	GM	Bcer	FALSE	NA	NA	NA
Bc_ctg1_5422	B3	9.80858576	Bcer	V	Bcer	FALSE	NA	NA	NA
Bc_ctg1_5423	B3	5.298891157	Bcer	K	Bcer	FALSE	NA	NA	NA
Bc_ctg1_5424	B3	5.186148792	Bcer	K	Bcer	FALSE	NA	NA	NA
Bc_ctg1_5425	B3	2.142104936	Bcer	NA	Bcer	FALSE	NA	NA	NA
Bc_ctg1_5426	B3	0.45096946	Bcer	L	Bcer	FALSE	NA	NA	NA
Bc_ctg1_5427	B3	1.240166016	Bcer	EG	Bcer	FALSE	NA	NA	NA
Bc_ctg1_5428	B3	20.06814098	Bcer	K	Bcer	FALSE	NA	NA	NA
Bc_ctg1_5429	B3	17.36232422	Bcer	S	Bcer	FALSE	NA	NA	NA
Bc_ctg1_543	B3	17.92603604	Bcer	NA	Bcer	FALSE	NA	NA	NA
Bc_ctg1_5430	B3	1.127423651	Bcer	S	Bcer	FALSE	NA	NA	NA
Bc_ctg1_5431	B3	18.3770055	Bcer	S	Bcer	FALSE	NA	NA	NA
Bc_ctg1_5432	B3	10.25955522	Bcer	F	Bcer	FALSE	NA	NA	NA
Bc_ctg1_5433	B3	10.14681285	Bcer	T	Bcer	FALSE	NA	NA	NA
Bc_ctg1_5434	B3	4.960664062	Bcer	S	Bcer	FALSE	NA	NA	NA
Bc_ctg1_5435	B3	7.215511363	Bcer	E	Bcer	FALSE	NA	NA	NA
Bc_ctg1_5436	B3	10.03407049	Bcer	S	Bcer	FALSE	NA	NA	NA
Bc_ctg1_5437	B3	0.338227095	Bcer	J	Bcer	FALSE	NA	NA	NA
Bc_ctg1_544	B3	42.50387162	Bcer	J	Bcer	FALSE	NA	NA	NA
Bc_ctg1_545	B3	2.593074396	Bcer	F	Bcer	FALSE	NA	NA	NA
Bc_ctg1_546	B3	4.396952237	Bcer	NA	Bcer	FALSE	NA	NA	NA
Bc_ctg1_547	B3	0.90193892	Bcer	I	Bcer	FALSE	NA	NA	NA
Bc_ctg1_548	B3	1.127423651	Bcer	IQ	Bcer	TRUE	NA	NA	NA
Bc_ctg1_549	B3	4.509694602	Bcer	S	Bcer	FALSE	NA	NA	NA
Bc_ctg1_55	B3	2.142104936	Bcer	S	Bcer	FALSE	NA	NA	NA
Bc_ctg1_550	B3	10.82326704	Bcer	IQ	Bcer	TRUE	NA	NA	NA
Bc_ctg1_551	B3	2.480332031	Bcer	S	Bcer	FALSE	NA	NA	NA
Bc_ctg1_552	B3	2.142104936	Bcer	V	Bcer	FALSE	NA	NA	NA
Bc_ctg1_553	B3	2.593074396	Bcer	K	Bcer	FALSE	NA	NA	NA
Bc_ctg1_554	B3	1.691135476	Bcer	NA	Bcer	FALSE	NA	NA	NA
Bc_ctg1_555	B3	0.67645419	Bcer	K	Bcer	FALSE	NA	NA	NA
Bc_ctg1_556	B3	2.931301491	Bcer	NA	Bcer	FALSE	NA	NA	NA
Bc_ctg1_557	B3	16.00941584	Bcer	S	Bcer	FALSE	NA	NA	NA
Bc_ctg1_558	B3	5.862602983	Bcer	V	Bcer	FALSE	NA	NA	NA
Bc_ctg1_559	B3	7.666480824	Bcer	T	Bcer	FALSE	NA	NA	NA
Bc_ctg1_56	B3	0	Bcer	L	Bcer	FALSE	NA	NA	NA
Bc_ctg1_560	B3	26.04348633	Bcer	S	Bcer	FALSE	NA	NA	NA

Bc_ctg1_561	B3	2.367589666	Bcer	E	Bcer	FALSE	NA	NA	NA
Bc_ctg1_562	B3	33.70996715	Bcer	T	Bcer	FALSE	NA	NA	NA
Bc_ctg1_563	B3	16.1221582	Bcer	S	Bcer	FALSE	NA	NA	NA
Bc_ctg1_564	B3	12.28891779	Bcer	E	Bcer	FALSE	NA	NA	NA
Bc_ctg1_565	B3	4.735179332	Bcer	E	Bcer	FALSE	NA	NA	NA
Bc_ctg1_566	B3	1.127423651	Bcer	O	Bcer	FALSE	NA	NA	NA
Bc_ctg1_567	B3	0.112742365	Bcer	E	Bcer	FALSE	NA	NA	NA
Bc_ctg1_568	B3	59.86619584	Bcer	C	Bcer	FALSE	NA	NA	NA
Bc_ctg1_569	B3	199.8922132	Bcer	K	Bcer	FALSE	NA	NA	NA
Bc_ctg1_57	B3	14.65650746	Bcer	L	Bcer	FALSE	NA	NA	NA
Bc_ctg1_570	B3	177.569225	Bcer	E	Bcer	FALSE	NA	NA	NA
Bc_ctg1_571	B3	187.152326	Bcer	Q	Bcer	TRUE	NA	NA	NA
Bc_ctg1_572	B3	210.940965	Bcer	E	Bcer	FALSE	NA	NA	NA
Bc_ctg1_573	B3	154.9080096	Bcer	E	Bcer	FALSE	NA	NA	NA
Bc_ctg1_574	B3	819.2987668	Bcer	K	Bcer	FALSE	NA	NA	NA
Bc_ctg1_575	B3	3.156786221	Bcer	Q	Bcer	TRUE	NA	NA	NA
Bc_ctg1_576	B3	3.044043856	Bcer	S	Bcer	FALSE	NA	NA	NA
Bc_ctg1_577	B3	0.563711825	Bcer	F	Bcer	FALSE	NA	NA	NA
Bc_ctg1_578	B3	29.20027255	Bcer	NA	Bcer	FALSE	NA	NA	NA
Bc_ctg1_579	B3	9.921328125	Bcer	S	Bcer	FALSE	NA	NA	NA
Bc_ctg1_58	B3	14.99473455	Bcer	L	Bcer	FALSE	NA	NA	NA
Bc_ctg1_580	B3	158.0647958	Bcer	O	Bcer	FALSE	NA	NA	NA
Bc_ctg1_581	B3	52.42519975	Bcer	O	Bcer	FALSE	NA	NA	NA
Bc_ctg1_582	B3	0	Bcer	NA	Bcer	FALSE	NA	NA	NA
Bc_ctg1_583	B3	57.72409091	Bcer	EG	Bcer	FALSE	NA	NA	NA
Bc_ctg1_584	B3	25.02880504	Bcer	E	Bcer	FALSE	NA	NA	NA
Bc_ctg1_585	B3	21.53379172	Bcer	S	Bcer	FALSE	NA	NA	NA
Bc_ctg1_586	B3	16.79861239	Bcer	V	Bcer	FALSE	NA	NA	NA
Bc_ctg1_588	B3	5.298891157	Bcer	V	Bcer	FALSE	NA	NA	NA
Bc_ctg1_589	B3	1.578393111	Bcer	NA	Bcer	FALSE	NA	NA	NA
Bc_ctg1_59	B3	3.044043856	Bcer	NA	Bcer	FALSE	NA	NA	NA
Bc_ctg1_590	B3	31.68060458	Bcer	K	Bcer	FALSE	NA	NA	NA
Bc_ctg1_591	B3	5.637118253	Bcer	S	Bcer	FALSE	NA	NA	NA
Bc_ctg1_592	B3	18.7152326	Bcer	M	Bcer	FALSE	NA	NA	NA
Bc_ctg1_593	B3	0.563711825	Bcer	F	Bcer	FALSE	NA	NA	NA
Bc_ctg1_594	B3	0.338227095	Bcer	S	Bcer	FALSE	NA	NA	NA
Bc_ctg1_595	B3	14.99473455	Bcer	S	Bcer	FALSE	NA	NA	NA
Bc_ctg1_596	B3	0	Bcer	NA	Bcer	FALSE	NA	NA	NA
Bc_ctg1_597	B3	11.6124636	Bcer	NA	Bcer	FALSE	NA	NA	NA
Bc_ctg1_598	B3	7.779223189	Bcer	S	Bcer	FALSE	NA	NA	NA
Bc_ctg1_599	B3	1.014681285	Bcer	M	Bcer	FALSE	NA	NA	NA
Bc_ctg1_6	B3	3.044043856	Bcer	NA	Bcer	FALSE	NA	NA	NA
Bc_ctg1_60	B3	13.07811435	Bcer	E	Bcer	FALSE	NA	NA	NA
Bc_ctg1_600	B3	26.04348633	Bcer	V	Bcer	FALSE	NA	NA	NA
Bc_ctg1_601	B3	14.99473455	Bcer	V	Bcer	FALSE	NA	NA	NA
Bc_ctg1_602	B3	82.30192649	Bcer	S	Bcer	FALSE	NA	NA	NA
Bc_ctg1_603	B3	36.64126864	Bcer	S	Bcer	FALSE	NA	NA	NA
Bc_ctg1_604	B3	2671.204855	Bcer	G	Bcer	FALSE	NA	NA	NA
Bc_ctg1_605	B3	4.960664062	Bcer	P	Bcer	FALSE	NA	NA	NA
Bc_ctg1_606	B3	2.818559126	Bcer	P	Bcer	FALSE	NA	NA	NA
Bc_ctg1_607	B3	1.352908381	Bcer	P	Bcer	FALSE	NA	NA	NA
Bc_ctg1_608	B3	1.916620206	Bcer	P	Bcer	FALSE	NA	NA	NA
Bc_ctg1_609	B3	12.51440252	Bcer	F	Bcer	FALSE	NA	NA	NA
Bc_ctg1_61	B3	16.23490057	Bcer	K	Bcer	FALSE	NA	NA	NA
Bc_ctg1_610	B3	6.990026633	Bcer	S	Bcer	FALSE	NA	NA	NA
Bc_ctg1_611	B3	8.906646839	Bcer	L	Bcer	FALSE	NA	NA	NA
Bc_ctg1_612	B3	386.368085	Bcer	K	Bcer	FALSE	NA	NA	NA
Bc_ctg1_613	B3	6.877284268	Bcer	S	Bcer	FALSE	NA	NA	NA
Bc_ctg1_614	B3	6.313572443	Bcer	S	Bcer	FALSE	NA	NA	NA
Bc_ctg1_615	B3	0	Bcer	K	Bcer	FALSE	NA	NA	NA
Bc_ctg1_616	B3	2.931301491	Bcer	S	Bcer	FALSE	NA	NA	NA
Bc_ctg1_617	B3	45.32243075	Bcer	S	Bcer	FALSE	NA	NA	NA

Bc_ctg1_618	B3	43.40581054	Bcer	M	Bcer	FALSE	NA	NA	NA
Bc_ctg1_619	B3	306.6592329	Bcer	M	Bcer	FALSE	NA	NA	NA
Bc_ctg1_62	B3	1.127423651	Bcer	L	Bcer	FALSE	NA	NA	NA
Bc_ctg1_620	B3	1.127423651	Bcer	S	Bcer	FALSE	NA	NA	NA
Bc_ctg1_621	B3	3.833240412	Bcer	L	Bcer	FALSE	NA	NA	NA
Bc_ctg1_622	B3	0.22548473	Bcer	NA	Bcer	FALSE	NA	NA	NA
Bc_ctg1_623	B3	1.465650746	Bcer	NA	Bcer	FALSE	NA	NA	NA
Bc_ctg1_624	B3	0.112742365	Bcer	NA	Bcer	FALSE	NA	NA	NA
Bc_ctg1_625	B3	69.44929687	Bcer	K	Bcer	FALSE	NA	NA	NA
Bc_ctg1_626	B3	2423.960849	Bcer	E	Bcer	FALSE	NA	NA	NA
Bc_ctg1_627	B3	206.9949822	Bcer	K	Bcer	FALSE	NA	NA	NA
Bc_ctg1_628	B3	64.60137517	Bcer	P	Bcer	FALSE	NA	NA	NA
Bc_ctg1_629	B3	74.07173384	Bcer	S	Bcer	FALSE	NA	NA	NA
Bc_ctg1_63	B3	25.02880504	Bcer	V	Bcer	FALSE	NA	NA	NA
Bc_ctg1_630	B3	19.72991388	Bcer	S	Bcer	FALSE	NA	NA	NA
Bc_ctg1_631	B3	0	Bcer	NA	Bcer	FALSE	NA	NA	NA
Bc_ctg1_632	B3	2.480332031	Bcer	O	Bcer	FALSE	NA	NA	NA
Bc_ctg1_633	B3	89.06646839	Bcer	L	Bcer	FALSE	NA	NA	NA
Bc_ctg1_634	B3	1.803877841	Bcer	Q	Bcer	TRUE	NA	NA	NA
Bc_ctg1_635	B3	207.7841788	Bcer	J	Bcer	FALSE	NA	NA	NA
Bc_ctg1_636	B3	132.9232484	Bcer	J	Bcer	FALSE	NA	NA	NA
Bc_ctg1_637	B3	0	Bcer	NA	Bcer	FALSE	NA	NA	NA
Bc_ctg1_638	B3	132.4722789	Bcer	T	Bcer	FALSE	NA	NA	NA
Bc_ctg1_639	B3	4947.473206	Bcer	GT	Bcer	FALSE	NA	NA	NA
Bc_ctg1_64	B3	9.132131569	Bcer	S	Bcer	FALSE	NA	NA	NA
Bc_ctg1_640	B3	1140.952734	Bcer	G	Bcer	FALSE	NA	NA	NA
Bc_ctg1_641	B3	1871.636002	Bcer	K	Bcer	FALSE	NA	NA	NA
Bc_ctg1_642	B3	158.2902805	Bcer	C	Bcer	FALSE	NA	NA	NA
Bc_ctg1_643	B3	136.5310041	Bcer	S	Bcer	FALSE	NA	NA	NA
Bc_ctg1_645	B3	73.50802201	Bcer	NA	Bcer	FALSE	NA	NA	NA
Bc_ctg1_646	B3	0.45096946	Bcer	E	Bcer	FALSE	NA	NA	NA
Bc_ctg1_647	B3	0.112742365	Bcer	G	Bcer	FALSE	NA	NA	NA
Bc_ctg1_648	B3	1.803877841	Bcer	S	Bcer	FALSE	NA	NA	NA
Bc_ctg1_649	B3	2.254847301	Bcer	P	Bcer	FALSE	NA	NA	NA
Bc_ctg1_65	B3	82.52741122	Bcer	F	Bcer	FALSE	NA	NA	NA
Bc_ctg1_650	B3	264.9445579	Bcer	L	Bcer	FALSE	NA	NA	NA
Bc_ctg1_651	B3	4.735179332	Bcer	M	Bcer	FALSE	NA	NA	NA
Bc_ctg1_652	B3	155.8099485	Bcer	P	Bcer	FALSE	NA	NA	NA
Bc_ctg1_653	B3	23.67589666	Bcer	P	Bcer	FALSE	NA	NA	NA
Bc_ctg1_654	B3	22.32298828	Bcer	S	Bcer	FALSE	NA	NA	NA
Bc_ctg1_655	B3	0	Bcer	NA	Bcer	FALSE	NA	NA	NA
Bc_ctg1_656	B3	50.50857954	Bcer	S	Bcer	FALSE	NA	NA	NA
Bc_ctg1_657	B3	24.91606268	Bcer	HP	Bcer	FALSE	NA	NA	NA
Bc_ctg1_658	B3	32.35705877	Bcer	P	Bcer	FALSE	NA	NA	NA
Bc_ctg1_659	B3	65.165087	Bcer	P	Bcer	FALSE	NA	NA	NA
Bc_ctg1_66	B3	7.891965554	Bcer	I	Bcer	FALSE	NA	NA	NA
Bc_ctg1_660	B3	223.906337	Bcer	P	Bcer	FALSE	NA	NA	NA
Bc_ctg1_661	B3	8.793904474	Bcer	L	Bcer	FALSE	NA	NA	NA
Bc_ctg1_662	B3	9.921328125	Bcer	K	Bcer	FALSE	Bc_Ga0417192_01_rn07	Regulation	SMCOG1287
Bc_ctg1_663	B3	5.298891157	Bcer	L	Bcer	FALSE	Bc_Ga0417192_01_rn07	Other	NA
Bc_ctg1_664	B3	21.98476119	Bcer	L	Bcer	FALSE	Bc_Ga0417192_01_rn07	Other	NA
Bc_ctg1_665	B3	275.4295978	Bcer	E	Bcer	FALSE	Bc_Ga0417192_01_rn07	Other	NA
Bc_ctg1_666	B3	1.014681285	Bcer	S	Bcer	FALSE	Bc_Ga0417192_01_rn07	Other	NA
Bc_ctg1_667	B3	13.07811435	Bcer	NA	Bcer	FALSE	Bc_Ga0417192_01_rn07	Other	NA
Bc_ctg1_668	B3	9.583101029	Bcer	G	Bcer	FALSE	Bc_Ga0417192_01_rn07	Ancillary biosynthesis	SMCOG1101
Bc_ctg1_669	B3	15.55844638	Bcer	S	Bcer	FALSE	Bc_Ga0417192_01_rn07	Ancillary biosynthesis	SMCOG1262
Bc_ctg1_67	B3	2.367589666	Bcer	Q	Bcer	TRUE	NA	NA	NA
Bc_ctg1_670	B3	153.7805859	Bcer	T	Bcer	FALSE	Bc_Ga0417192_01_rn07	Ancillary biosynthesis	SMCOG1025
Bc_ctg1_671	B3	1.014681285	Bcer	M	Bcer	FALSE	Bc_Ga0417192_01_rn07	Core biosynthesis	None
Bc_ctg1_672	B3	0.22548473	Bcer	NA	Bcer	FALSE	Bc_Ga0417192_01_rn07	Other	NA
Bc_ctg1_673	B3	0.112742365	Bcer	GM	Bcer	FALSE	Bc_Ga0417192_01_rn07	Core biosynthesis	SMCOG1010
Bc_ctg1_674	B3	0.112742365	Bcer	M	Bcer	FALSE	Bc_Ga0417192_01_rn07	Core biosynthesis	SMCOG1010

Bc_ctg1_675	B3	0.22548473	Bcer	S	Bcer	FALSE	Bc_Ga0417192_01_rn07	Other	NA
Bc_ctg1_676	B3	1.691135476	Bcer	S	Bcer	FALSE	Bc_Ga0417192_01_rn07	Other	NA
Bc_ctg1_677	B3	4.284209872	Bcer	M	Bcer	FALSE	Bc_Ga0417192_01_rn07	Other	NA
Bc_ctg1_678	B3	2.254847301	Bcer	S	Bcer	FALSE	Bc_Ga0417192_01_rn07	Other	NA
Bc_ctg1_679	B3	10.71052468	Bcer	K	Bcer	FALSE	Bc_Ga0417192_01_rn07	Ancillary biosynthesis	SMCOG1019
Bc_ctg1_68	B3	1.240166016	Bcer	Q	Bcer	TRUE	NA	NA	NA
Bc_ctg1_680	B3	0.563711825	Bcer	NA	Bcer	FALSE	Bc_Ga0417192_01_rn07	Other	NA
Bc_ctg1_681	B3	27.50913707	Bcer	K	Bcer	FALSE	Bc_Ga0417192_01_rn07	Regulation	SMCOG1136
Bc_ctg1_682	B3	60.76813476	Bcer	G	Bcer	FALSE	Bc_Ga0417192_01_rn07	Other	NA
Bc_ctg1_683	B3	49.49389826	Bcer	U	Bcer	FALSE	Bc_Ga0417192_01_rn07	Other	NA
Bc_ctg1_684	B3	46.90082386	Bcer	O	Bcer	FALSE	NA	NA	NA
Bc_ctg1_685	B3	536.5409153	Bcer	M	Bcer	FALSE	NA	NA	NA
Bc_ctg1_686	B3	9.80858576	Bcer	S	Bcer	FALSE	NA	NA	NA
Bc_ctg1_687	B3	17.36232422	Bcer	M	Bcer	FALSE	NA	NA	NA
Bc_ctg1_688	B3	11.72520597	Bcer	S	Bcer	FALSE	NA	NA	NA
Bc_ctg1_69	B3	0.22548473	Bcer	NA	Bcer	FALSE	NA	NA	NA
Bc_ctg1_690	B3	173.6232422	Bcer	L	Bcer	FALSE	NA	NA	NA
Bc_ctg1_691	B3	186.7013565	Bcer	L	Bcer	FALSE	NA	NA	NA
Bc_ctg1_692	B3	2.367589666	Bcer	S	Bcer	FALSE	NA	NA	NA
Bc_ctg1_693	B3	96.28197975	Bcer	S	Bcer	FALSE	NA	NA	NA
Bc_ctg1_694	B3	452.8860804	Bcer	J	Bcer	FALSE	NA	NA	NA
Bc_ctg1_695	B3	900.9242391	Bcer	C	Bcer	FALSE	NA	NA	NA
Bc_ctg1_696	B3	1931.727683	Bcer	C	Bcer	FALSE	NA	NA	NA
Bc_ctg1_697	B3	17.92603604	Bcer	E	Bcer	FALSE	NA	NA	NA
Bc_ctg1_698	B3	48.59195934	Bcer	S	Bcer	FALSE	NA	NA	NA
Bc_ctg1_699	B3	108.9091246	Bcer	S	Bcer	FALSE	NA	NA	NA
Bc_ctg1_7	B3	0.789196555	Bcer	S	Bcer	FALSE	NA	NA	NA
Bc_ctg1_70	B3	6.764541903	Bcer	S	Bcer	FALSE	NA	NA	NA
Bc_ctg1_700	B3	3375.50641	Bcer	S	Bcer	FALSE	NA	NA	NA
Bc_ctg1_701	B3	499.7869043	Bcer	L	Bcer	FALSE	NA	NA	NA
Bc_ctg1_702	B3	366.0744593	Bcer	S	Bcer	FALSE	NA	NA	NA
Bc_ctg1_703	B3	119.0559375	Bcer	I	Bcer	FALSE	NA	NA	NA
Bc_ctg1_704	B3	335.9722479	Bcer	S	Bcer	FALSE	NA	NA	NA
Bc_ctg1_705	B3	198.0883354	Bcer	S	Bcer	FALSE	NA	NA	NA
Bc_ctg1_706	B3	0.338227095	Bcer	S	Bcer	FALSE	NA	NA	NA
Bc_ctg1_707	B3	19.16620206	Bcer	IQ	Bcer	TRUE	NA	NA	NA
Bc_ctg1_708	B3	191.6620206	Bcer	S	Bcer	FALSE	NA	NA	NA
Bc_ctg1_709	B3	135.6290652	Bcer	S	Bcer	FALSE	NA	NA	NA
Bc_ctg1_71	B3	6.426314808	Bcer	K	Bcer	FALSE	NA	NA	NA
Bc_ctg1_710	B3	399.333457	Bcer	S	Bcer	FALSE	NA	NA	NA
Bc_ctg1_711	B3	322.7813911	Bcer	S	Bcer	FALSE	NA	NA	NA
Bc_ctg1_712	B3	406.6617107	Bcer	S	Bcer	FALSE	NA	NA	NA
Bc_ctg1_713	B3	1398.907266	Bcer	S	Bcer	FALSE	NA	NA	NA
Bc_ctg1_714	B3	413.0880255	Bcer	K	Bcer	FALSE	NA	NA	NA
Bc_ctg1_715	B3	76.10109641	Bcer	NA	Bcer	FALSE	NA	NA	NA
Bc_ctg1_716	B3	163.0254599	Bcer	D	Bcer	FALSE	NA	NA	NA
Bc_ctg1_717	B3	0.67645419	Bcer	S	Bcer	FALSE	NA	NA	NA
Bc_ctg1_718	B3	2.367589666	Bcer	OU	Bcer	FALSE	NA	NA	NA
Bc_ctg1_719	B3	1076.351359	Bcer	S	Bcer	FALSE	NA	NA	NA
Bc_ctg1_72	B3	0.563711825	Bcer	K	Bcer	FALSE	NA	NA	NA
Bc_ctg1_720	B3	858.7585946	Bcer	E	Bcer	FALSE	NA	NA	NA
Bc_ctg1_721	B3	1424.499782	Bcer	E	Bcer	FALSE	NA	NA	NA
Bc_ctg1_722	B3	1208.259926	Bcer	E	Bcer	FALSE	NA	NA	NA
Bc_ctg1_723	B3	2.254847301	Bcer	H	Bcer	FALSE	NA	NA	NA
Bc_ctg1_724	B3	14.31828036	Bcer	NA	Bcer	FALSE	NA	NA	NA
Bc_ctg1_726	B3	65.165087	Bcer	T	Bcer	FALSE	NA	NA	NA
Bc_ctg1_727	B3	58.85151456	Bcer	NA	Bcer	FALSE	NA	NA	NA
Bc_ctg1_728	B3	1.352908381	Bcer	NA	Bcer	FALSE	NA	NA	NA
Bc_ctg1_729	B3	0.22548473	Bcer	L	Bcer	FALSE	NA	NA	NA
Bc_ctg1_73	B3	2.705816761	Bcer	P	Bcer	FALSE	NA	NA	NA
Bc_ctg1_731	B3	0	Bcer	M	Bcer	FALSE	NA	NA	NA
Bc_ctg1_732	B3	0	Bcer	S	Bcer	FALSE	NA	NA	NA

Bc_ctg1_733	B3	87.60081765	Bcer	S	Bcer	FALSE	NA	NA	NA
Bc_ctg1_734	B3	0	Bcer	NA	Bcer	FALSE	NA	NA	NA
Bc_ctg1_735	B3	0.90193892	Bcer	U	Bcer	FALSE	NA	NA	NA
Bc_ctg1_736	B3	0	Bcer	S	Bcer	FALSE	NA	NA	NA
Bc_ctg1_737	B3	3.495013317	Bcer	D	Bcer	FALSE	NA	NA	NA
Bc_ctg1_739	B3	0	Bcer	S	Bcer	FALSE	NA	NA	NA
Bc_ctg1_74	B3	7.328253728	Bcer	S	Bcer	FALSE	NA	NA	NA
Bc_ctg1_740	B3	0.22548473	Bcer	S	Bcer	FALSE	NA	NA	NA
Bc_ctg1_741	B3	0	Bcer	NA	Bcer	FALSE	NA	NA	NA
Bc_ctg1_742	B3	0	Bcer	S	Bcer	FALSE	NA	NA	NA
Bc_ctg1_743	B3	0	Bcer	S	Bcer	FALSE	NA	NA	NA
Bc_ctg1_745	B3	0.112742365	Bcer	S	Bcer	FALSE	NA	NA	NA
Bc_ctg1_746	B3	0.112742365	Bcer	OU	Bcer	FALSE	NA	NA	NA
Bc_ctg1_747	B3	0.22548473	Bcer	S	Bcer	FALSE	NA	NA	NA
Bc_ctg1_748	B3	0	Bcer	S	Bcer	FALSE	NA	NA	NA
Bc_ctg1_749	B3	0.45096946	Bcer	S	Bcer	FALSE	NA	NA	NA
Bc_ctg1_75	B3	0.22548473	Bcer	NA	Bcer	FALSE	NA	NA	NA
Bc_ctg1_750	B3	1.240166016	Bcer	V	Bcer	FALSE	NA	NA	NA
Bc_ctg1_751	B3	0.45096946	Bcer	NA	Bcer	FALSE	NA	NA	NA
Bc_ctg1_752	B3	2100.953973	Bcer	NA	Bcer	FALSE	NA	NA	NA
Bc_ctg1_753	B3	0.22548473	Bcer	NA	Bcer	FALSE	NA	NA	NA
Bc_ctg1_754	B3	0.563711825	Bcer	NA	Bcer	FALSE	NA	NA	NA
Bc_ctg1_755	B3	0.22548473	Bcer	L	Bcer	FALSE	NA	NA	NA
Bc_ctg1_756	B3	0	Bcer	S	Bcer	FALSE	NA	NA	NA
Bc_ctg1_757	B3	50.84680664	Bcer	S	Bcer	FALSE	NA	NA	NA
Bc_ctg1_758	B3	58.4005451	Bcer	S	Bcer	FALSE	NA	NA	NA
Bc_ctg1_759	B3	0	Bcer	NA	Bcer	FALSE	NA	NA	NA
Bc_ctg1_76	B3	0.112742365	Bcer	S	Bcer	FALSE	NA	NA	NA
Bc_ctg1_760	B3	0.22548473	Bcer	NA	Bcer	FALSE	NA	NA	NA
Bc_ctg1_761	B3	0	Bcer	NA	Bcer	FALSE	NA	NA	NA
Bc_ctg1_762	B3	0.67645419	Bcer	L	Bcer	FALSE	NA	NA	NA
Bc_ctg1_763	B3	1.465650746	Bcer	L	Bcer	FALSE	NA	NA	NA
Bc_ctg1_764	B3	0	Bcer	NA	Bcer	FALSE	NA	NA	NA
Bc_ctg1_765	B3	0.67645419	Bcer	NA	Bcer	FALSE	NA	NA	NA
Bc_ctg1_766	B3	0.112742365	Bcer	NA	Bcer	FALSE	NA	NA	NA
Bc_ctg1_767	B3	0.45096946	Bcer	NA	Bcer	FALSE	NA	NA	NA
Bc_ctg1_768	B3	0.338227095	Bcer	NA	Bcer	FALSE	NA	NA	NA
Bc_ctg1_769	B3	0.112742365	Bcer	K	Bcer	FALSE	NA	NA	NA
Bc_ctg1_77	B3	50.73406427	Bcer	G	Bcer	FALSE	NA	NA	NA
Bc_ctg1_770	B3	15.44570401	Bcer	K	Bcer	FALSE	NA	NA	NA
Bc_ctg1_771	B3	1.127423651	Bcer	E	Bcer	FALSE	NA	NA	NA
Bc_ctg1_772	B3	0.90193892	Bcer	NA	Bcer	FALSE	NA	NA	NA
Bc_ctg1_773	B3	1.691135476	Bcer	NA	Bcer	FALSE	NA	NA	NA
Bc_ctg1_774	B3	8.004707919	Bcer	L	Bcer	FALSE	NA	NA	NA
Bc_ctg1_775	B3	0	Bcer	H	Bcer	FALSE	NA	NA	NA
Bc_ctg1_776	B3	0	Bcer	E	Bcer	FALSE	NA	NA	NA
Bc_ctg1_778	B3	228.3032892	Bcer	S	Bcer	FALSE	NA	NA	NA
Bc_ctg1_779	B3	29.31301491	Bcer	G	Bcer	FALSE	NA	NA	NA
Bc_ctg1_78	B3	0	Bcer	S	Bcer	FALSE	NA	NA	NA
Bc_ctg1_780	B3	1455.165706	Bcer	J	Bcer	FALSE	NA	NA	NA
Bc_ctg1_781	B3	512.977761	Bcer	J	Bcer	FALSE	NA	NA	NA
Bc_ctg1_782	B3	219.5093848	Bcer	H	Bcer	FALSE	NA	NA	NA
Bc_ctg1_783	B3	85.7969398	Bcer	J	Bcer	FALSE	NA	NA	NA
Bc_ctg1_784	B3	347.3592267	Bcer	J	Bcer	FALSE	NA	NA	NA
Bc_ctg1_785	B3	144.3102273	Bcer	S	Bcer	FALSE	NA	NA	NA
Bc_ctg1_786	B3	2487.4348	Bcer	J	Bcer	FALSE	NA	NA	NA
Bc_ctg1_787	B3	206.0930433	Bcer	J	Bcer	FALSE	NA	NA	NA
Bc_ctg1_788	B3	70.57672052	Bcer	K	Bcer	FALSE	NA	NA	NA
Bc_ctg1_789	B3	698.5516939	Bcer	K	Bcer	FALSE	NA	NA	NA
Bc_ctg1_79	B3	10.25955522	Bcer	I	Bcer	FALSE	NA	NA	NA
Bc_ctg1_790	B3	413.0880255	Bcer	S	Bcer	FALSE	NA	NA	NA
Bc_ctg1_791	B3	410.8331782	Bcer	L	Bcer	FALSE	NA	NA	NA

Bc_ctg1_792	B3	419.6270827	Bcer	J	Bcer	FALSE	NA	NA	NA
Bc_ctg1_793	B3	387.608251	Bcer	M	Bcer	FALSE	NA	NA	NA
Bc_ctg1_794	B3	275.7678249	Bcer	I	Bcer	FALSE	NA	NA	NA
Bc_ctg1_795	B3	124.0166016	Bcer	S	Bcer	FALSE	NA	NA	NA
Bc_ctg1_796	B3	116.5756055	Bcer	I	Bcer	FALSE	NA	NA	NA
Bc_ctg1_797	B3	432.7051971	Bcer	J	Bcer	FALSE	NA	NA	NA
Bc_ctg1_798	B3	466.8661337	Bcer	F	Bcer	FALSE	NA	NA	NA
Bc_ctg1_799	B3	1657.650993	Bcer	J	Bcer	FALSE	NA	NA	NA
Bc_ctg1_8	B3	1.691135476	Bcer	NA	Bcer	FALSE	NA	NA	NA
Bc_ctg1_80	B3	9.80858576	Bcer	T	Bcer	FALSE	NA	NA	NA
Bc_ctg1_800	B3	4191.873875	Bcer	J	Bcer	FALSE	NA	NA	NA
Bc_ctg1_801	B3	649.2832803	Bcer	K	Bcer	FALSE	NA	NA	NA
Bc_ctg1_802	B3	684.0079288	Bcer	O	Bcer	FALSE	NA	NA	NA
Bc_ctg1_803	B3	62.9102397	Bcer	O	Bcer	FALSE	NA	NA	NA
Bc_ctg1_804	B3	166.1822461	Bcer	L	Bcer	FALSE	NA	NA	NA
Bc_ctg1_805	B3	320.9775133	Bcer	J	Bcer	FALSE	NA	NA	NA
Bc_ctg1_806	B3	1942.776435	Bcer	L	Bcer	FALSE	NA	NA	NA
Bc_ctg1_807	B3	0.338227095	Bcer	LU	Bcer	FALSE	NA	NA	NA
Bc_ctg1_808	B3	1929.923805	Bcer	C	Bcer	FALSE	NA	NA	NA
Bc_ctg1_809	B3	2249.999379	Bcer	C	Bcer	FALSE	NA	NA	NA
Bc_ctg1_81	B3	31.45511985	Bcer	L	Bcer	FALSE	NA	NA	NA
Bc_ctg1_810	B3	17.02409712	Bcer	L	Bcer	FALSE	NA	NA	NA
Bc_ctg1_811	B3	131.1193706	Bcer	S	Bcer	FALSE	NA	NA	NA
Bc_ctg1_812	B3	97.7476305	Bcer	U	Bcer	FALSE	NA	NA	NA
Bc_ctg1_813	B3	2670.753886	Bcer	J	Bcer	FALSE	NA	NA	NA
Bc_ctg1_814	B3	61.89555841	Bcer	J	Bcer	FALSE	NA	NA	NA
Bc_ctg1_815	B3	32.5825435	Bcer	J	Bcer	FALSE	NA	NA	NA
Bc_ctg1_816	B3	156.0354332	Bcer	S	Bcer	FALSE	NA	NA	NA
Bc_ctg1_817	B3	715.1248215	Bcer	J	Bcer	FALSE	NA	NA	NA
Bc_ctg1_818	B3	1596.319147	Bcer	U	Bcer	FALSE	NA	NA	NA
Bc_ctg1_819	B3	118.0412562	Bcer	S	Bcer	FALSE	NA	NA	NA
Bc_ctg1_82	B3	15.89667347	Bcer	K	Bcer	FALSE	NA	NA	NA
Bc_ctg1_820	B3	144.3102273	Bcer	U	Bcer	FALSE	NA	NA	NA
Bc_ctg1_821	B3	198.0883354	Bcer	D	Bcer	FALSE	NA	NA	NA
Bc_ctg1_822	B3	290.4243324	Bcer	J	Bcer	FALSE	NA	NA	NA
Bc_ctg1_823	B3	106.9925044	Bcer	IQ	Bcer	TRUE	NA	NA	NA
Bc_ctg1_824	B3	214.4359783	Bcer	IQ	Bcer	TRUE	NA	NA	NA
Bc_ctg1_825	B3	174.8634082	Bcer	I	Bcer	FALSE	NA	NA	NA
Bc_ctg1_826	B3	112.8551074	Bcer	I	Bcer	FALSE	NA	NA	NA
Bc_ctg1_827	B3	47.57727805	Bcer	K	Bcer	FALSE	NA	NA	NA
Bc_ctg1_828	B3	106.2033079	Bcer	L	Bcer	FALSE	NA	NA	NA
Bc_ctg1_829	B3	1289.998141	Bcer	S	Bcer	FALSE	NA	NA	NA
Bc_ctg1_83	B3	0.789196555	Bcer	EGP	Bcer	FALSE	NA	NA	NA
Bc_ctg1_830	B3	195.6080034	Bcer	S	Bcer	FALSE	NA	NA	NA
Bc_ctg1_831	B3	16.23490057	Bcer	J	Bcer	FALSE	NA	NA	NA
Bc_ctg1_832	B3	17.24958185	Bcer	H	Bcer	FALSE	NA	NA	NA
Bc_ctg1_833	B3	242.0578578	Bcer	G	Bcer	FALSE	NA	NA	NA
Bc_ctg1_834	B3	119.8451341	Bcer	S	Bcer	FALSE	NA	NA	NA
Bc_ctg1_835	B3	786.8289657	Bcer	KLT	Bcer	FALSE	NA	NA	NA
Bc_ctg1_836	B3	147.3542711	Bcer	T	Bcer	FALSE	NA	NA	NA
Bc_ctg1_837	B3	438.1168306	Bcer	J	Bcer	FALSE	NA	NA	NA
Bc_ctg1_838	B3	234.729604	Bcer	J	Bcer	FALSE	NA	NA	NA
Bc_ctg1_839	B3	118.604968	Bcer	J	Bcer	FALSE	NA	NA	NA
Bc_ctg1_84	B3	1.240166016	Bcer	E	Bcer	FALSE	NA	NA	NA
Bc_ctg1_840	B3	33.48448242	Bcer	J	Bcer	FALSE	NA	NA	NA
Bc_ctg1_841	B3	108.4581552	Bcer	L	Bcer	FALSE	NA	NA	NA
Bc_ctg1_842	B3	44.08226474	Bcer	H	Bcer	FALSE	NA	NA	NA
Bc_ctg1_843	B3	40.47450905	Bcer	K	Bcer	FALSE	NA	NA	NA
Bc_ctg1_844	B3	134.2761568	Bcer	F	Bcer	FALSE	NA	NA	NA
Bc_ctg1_845	B3	43.63129528	Bcer	S	Bcer	FALSE	NA	NA	NA
Bc_ctg1_846	B3	142.05538	Bcer	S	Bcer	FALSE	NA	NA	NA
Bc_ctg1_847	B3	13.19085671	Bcer	P	Bcer	FALSE	NA	NA	NA

Bc_ctg1_848	B3	100.2279625	Bcer	K	Bcer	FALSE	NA	NA	NA
Bc_ctg1_849	B3	8.681162109	Bcer	S	Bcer	FALSE	NA	NA	NA
Bc_ctg1_85	B3	0.112742365	Bcer	I	Bcer	FALSE	NA	NA	NA
Bc_ctg1_850	B3	1.352908381	Bcer	E	Bcer	FALSE	NA	NA	NA
Bc_ctg1_851	B3	0.22548473	Bcer	S	Bcer	FALSE	NA	NA	NA
Bc_ctg1_852	B3	0.90193892	Bcer	S	Bcer	FALSE	NA	NA	NA
Bc_ctg1_853	B3	0	Bcer	S	Bcer	FALSE	NA	NA	NA
Bc_ctg1_855	B3	1.127423651	Bcer	C	Bcer	FALSE	NA	NA	NA
Bc_ctg1_856	B3	14.31828036	Bcer	S	Bcer	FALSE	NA	NA	NA
Bc_ctg1_857	B3	23.5631543	Bcer	S	Bcer	FALSE	NA	NA	NA
Bc_ctg1_858	B3	218.8329306	Bcer	F	Bcer	FALSE	NA	NA	NA
Bc_ctg1_859	B3	213.6467818	Bcer	F	Bcer	FALSE	NA	NA	NA
Bc_ctg1_86	B3	0.563711825	Bcer	P	Bcer	FALSE	NA	NA	NA
Bc_ctg1_860	B3	431.2395463	Bcer	F	Bcer	FALSE	NA	NA	NA
Bc_ctg1_861	B3	254.1212908	Bcer	C	Bcer	FALSE	NA	NA	NA
Bc_ctg1_862	B3	1633.749612	Bcer	F	Bcer	FALSE	NA	NA	NA
Bc_ctg1_863	B3	314.5511985	Bcer	F	Bcer	FALSE	NA	NA	NA
Bc_ctg1_864	B3	151.1875115	Bcer	F	Bcer	FALSE	NA	NA	NA
Bc_ctg1_865	B3	57.94957564	Bcer	F	Bcer	FALSE	NA	NA	NA
Bc_ctg1_866	B3	113.7570463	Bcer	F	Bcer	FALSE	NA	NA	NA
Bc_ctg1_867	B3	186.8140989	Bcer	F	Bcer	FALSE	NA	NA	NA
Bc_ctg1_868	B3	334.9575666	Bcer	J	Bcer	FALSE	NA	NA	NA
Bc_ctg1_869	B3	101.4681285	Bcer	MU	Bcer	FALSE	NA	NA	NA
Bc_ctg1_87	B3	1.803877841	Bcer	S	Bcer	FALSE	NA	NA	NA
Bc_ctg1_870	B3	0.112742365	Bcer	T	Bcer	FALSE	NA	NA	NA
Bc_ctg1_871	B3	1123.364925	Bcer	J	Bcer	FALSE	NA	NA	NA
Bc_ctg1_872	B3	501.252555	Bcer	D	Bcer	FALSE	NA	NA	NA
Bc_ctg1_873	B3	650.0724769	Bcer	S	Bcer	FALSE	NA	NA	NA
Bc_ctg1_874	B3	66.40525302	Bcer	S	Bcer	FALSE	NA	NA	NA
Bc_ctg1_875	B3	192.9021866	Bcer	D	Bcer	FALSE	NA	NA	NA
Bc_ctg1_876	B3	358.6334632	Bcer	S	Bcer	FALSE	Bc_Ga0417192_01_rn08	Other	NA
Bc_ctg1_877	B3	347.8101962	Bcer	S	Bcer	FALSE	Bc_Ga0417192_01_rn08	Other	NA
Bc_ctg1_878	B3	0	Bcer	S	Bcer	FALSE	Bc_Ga0417192_01_rn08	Other	NA
Bc_ctg1_879	B3	0	Bcer	K	Bcer	FALSE	Bc_Ga0417192_01_rn08	Other	NA
Bc_ctg1_88	B3	1.127423651	Bcer	CP	Bcer	FALSE	NA	NA	NA
Bc_ctg1_880	B3	1.803877841	Bcer	K	Bcer	FALSE	Bc_Ga0417192_01_rn08	Other	NA
Bc_ctg1_881	B3	2.480332031	Bcer	M	Bcer	FALSE	Bc_Ga0417192_01_rn08	Other	NA
Bc_ctg1_882	B3	1259.670445	Bcer	D	Bcer	FALSE	Bc_Ga0417192_01_rn08	Other	NA
Bc_ctg1_883	B3	1161.697329	Bcer	D	Bcer	FALSE	Bc_Ga0417192_01_rn08	Other	NA
Bc_ctg1_884	B3	21.19556463	Bcer	D	Bcer	FALSE	Bc_Ga0417192_01_rn08	Other	NA
Bc_ctg1_885	B3	74.1844762	Bcer	M	Bcer	FALSE	Bc_Ga0417192_01_rn08	Other	NA
Bc_ctg1_886	B3	40.36176669	Bcer	M	Bcer	FALSE	Bc_Ga0417192_01_rn08	Core biosynthesis	SMCOG1062
Bc_ctg1_887	B3	47.23905096	Bcer	D	Bcer	FALSE	Bc_Ga0417192_01_rn08	Other	NA
Bc_ctg1_888	B3	455.1409277	Bcer	M	Bcer	FALSE	Bc_Ga0417192_01_rn08	Other	NA
Bc_ctg1_889	B3	224.2445641	Bcer	M	Bcer	FALSE	Bc_Ga0417192_01_rn08	Core biosynthesis	None
Bc_ctg1_89	B3	0.22548473	Bcer	S	Bcer	FALSE	NA	NA	NA
Bc_ctg1_890	B3	205.6420739	Bcer	M	Bcer	FALSE	Bc_Ga0417192_01_rn08	Other	NA
Bc_ctg1_891	B3	344.0896981	Bcer	M	Bcer	FALSE	Bc_Ga0417192_01_rn08	Other	NA
Bc_ctg1_892	B3	583.4417391	Bcer	M	Bcer	FALSE	Bc_Ga0417192_01_rn08	Other	NA
Bc_ctg1_893	B3	358.5207209	Bcer	D	Bcer	FALSE	Bc_Ga0417192_01_rn08	Other	NA
Bc_ctg1_894	B3	411.7351172	Bcer	J	Bcer	FALSE	Bc_Ga0417192_01_rn08	Other	NA
Bc_ctg1_895	B3	174.8634082	Bcer	S	Bcer	FALSE	Bc_Ga0417192_01_rn08	Other	NA
Bc_ctg1_896	B3	13.75456854	Bcer	H	Bcer	FALSE	NA	NA	NA
Bc_ctg1_897	B3	272.1600692	Bcer	K	Bcer	FALSE	NA	NA	NA
Bc_ctg1_898	B3	10.14681285	Bcer	S	Bcer	FALSE	NA	NA	NA
Bc_ctg1_899	B3	43.85678001	Bcer	J	Bcer	FALSE	NA	NA	NA
Bc_ctg1_9	B3	0	Bcer	NA	Bcer	FALSE	NA	NA	NA
Bc_ctg1_90	B3	0	Bcer	EGP	Bcer	FALSE	NA	NA	NA
Bc_ctg1_900	B3	751.2023783	Bcer	S	Bcer	FALSE	NA	NA	NA
Bc_ctg1_901	B3	78.69417081	Bcer	S	Bcer	FALSE	NA	NA	NA
Bc_ctg1_902	B3	25.36703214	Bcer	T	Bcer	FALSE	NA	NA	NA
Bc_ctg1_903	B3	21.87201882	Bcer	S	Bcer	FALSE	NA	NA	NA

Bc_ctg1_904	B3	0.90193892	Bcer	S	Bcer	FALSE	NA	NA	NA
Bc_ctg1_905	B3	58.062318	Bcer	H	Bcer	FALSE	NA	NA	NA
Bc_ctg1_906	B3	29.08753018	Bcer	L	Bcer	FALSE	NA	NA	NA
Bc_ctg1_907	B3	96.05649502	Bcer	S	Bcer	FALSE	NA	NA	NA
Bc_ctg1_908	B3	65.84154119	Bcer	S	Bcer	FALSE	NA	NA	NA
Bc_ctg1_909	B3	89.85566495	Bcer	S	Bcer	FALSE	NA	NA	NA
Bc_ctg1_91	B3	3.156786221	Bcer	K	Bcer	FALSE	NA	NA	NA
Bc_ctg1_910	B3	28.63656072	Bcer	G	Bcer	FALSE	NA	NA	NA
Bc_ctg1_911	B3	0.112742365	Bcer	S	Bcer	FALSE	NA	NA	NA
Bc_ctg1_912	B3	0.22548473	Bcer	S	Bcer	FALSE	NA	NA	NA
Bc_ctg1_913	B3	89.51743785	Bcer	S	Bcer	FALSE	NA	NA	NA
Bc_ctg1_914	B3	6.764541903	Bcer	S	Bcer	FALSE	NA	NA	NA
Bc_ctg1_915	B3	10.93600941	Bcer	S	Bcer	FALSE	NA	NA	NA
Bc_ctg1_916	B3	44.98420366	Bcer	S	Bcer	FALSE	NA	NA	NA
Bc_ctg1_917	B3	26.83268288	Bcer	C	Bcer	FALSE	NA	NA	NA
Bc_ctg1_918	B3	89.85566495	Bcer	C	Bcer	FALSE	NA	NA	NA
Bc_ctg1_919	B3	328.5312518	Bcer	C	Bcer	FALSE	NA	NA	NA
Bc_ctg1_92	B3	0.338227095	Bcer	S	Bcer	FALSE	NA	NA	NA
Bc_ctg1_920	B3	212.7448429	Bcer	C	Bcer	FALSE	NA	NA	NA
Bc_ctg1_921	B3	247.9204607	Bcer	O	Bcer	FALSE	NA	NA	NA
Bc_ctg1_922	B3	192.6767019	Bcer	O	Bcer	FALSE	NA	NA	NA
Bc_ctg1_923	B3	0.338227095	Bcer	L	Bcer	FALSE	NA	NA	NA
Bc_ctg1_924	B3	1.691135476	Bcer	L	Bcer	FALSE	NA	NA	NA
Bc_ctg1_925	B3	3972.36449	Bcer	C	Bcer	FALSE	NA	NA	NA
Bc_ctg1_926	B3	133.5997026	Bcer	D	Bcer	FALSE	NA	NA	NA
Bc_ctg1_927	B3	200.3431827	Bcer	S	Bcer	FALSE	NA	NA	NA
Bc_ctg1_928	B3	128.8645233	Bcer	S	Bcer	FALSE	NA	NA	NA
Bc_ctg1_929	B3	292.0027255	Bcer	T	Bcer	FALSE	NA	NA	NA
Bc_ctg1_93	B3	9.80858576	Bcer	G	Bcer	FALSE	NA	NA	NA
Bc_ctg1_930	B3	191.4365359	Bcer	S	Bcer	FALSE	NA	NA	NA
Bc_ctg1_931	B3	8.793904474	Bcer	S	Bcer	FALSE	NA	NA	NA
Bc_ctg1_932	B3	1.014681285	Bcer	NA	Bcer	FALSE	NA	NA	NA
Bc_ctg1_933	B3	0.90193892	Bcer	S	Bcer	FALSE	NA	NA	NA
Bc_ctg1_934	B3	2620.92176	Bcer	T	Bcer	FALSE	NA	NA	NA
Bc_ctg1_935	B3	30.44043856	Bcer	NA	Bcer	FALSE	NA	NA	NA
Bc_ctg1_936	B3	46.67533913	Bcer	G	Bcer	FALSE	NA	NA	NA
Bc_ctg1_937	B3	0.67645419	Bcer	NA	Bcer	FALSE	NA	NA	NA
Bc_ctg1_938	B3	34.61190607	Bcer	S	Bcer	FALSE	NA	NA	NA
Bc_ctg1_939	B3	3.495013317	Bcer	S	Bcer	FALSE	NA	NA	NA
Bc_ctg1_94	B3	3.945982777	Bcer	K	Bcer	FALSE	NA	NA	NA
Bc_ctg1_940	B3	396.0639284	Bcer	E	Bcer	FALSE	NA	NA	NA
Bc_ctg1_941	B3	1.465650746	Bcer	H	Bcer	FALSE	NA	NA	NA
Bc_ctg1_942	B3	0.338227095	Bcer	S	Bcer	FALSE	NA	NA	NA
Bc_ctg1_943	B3	0.22548473	Bcer	S	Bcer	FALSE	NA	NA	NA
Bc_ctg1_944	B3	0.338227095	Bcer	S	Bcer	FALSE	NA	NA	NA
Bc_ctg1_945	B3	5.186148792	Bcer	S	Bcer	FALSE	NA	NA	NA
Bc_ctg1_946	B3	4.960664062	Bcer	S	Bcer	FALSE	NA	NA	NA
Bc_ctg1_947	B3	0.112742365	Bcer	NA	Bcer	FALSE	NA	NA	NA
Bc_ctg1_948	B3	2800.407606	Bcer	C	Bcer	FALSE	NA	NA	NA
Bc_ctg1_949	B3	3524.777301	Bcer	C	Bcer	FALSE	NA	NA	NA
Bc_ctg1_95	B3	37.0922381	Bcer	H	Bcer	FALSE	NA	NA	NA
Bc_ctg1_950	B3	1510.18398	Bcer	C	Bcer	FALSE	NA	NA	NA
Bc_ctg1_951	B3	2629.49018	Bcer	C	Bcer	FALSE	NA	NA	NA
Bc_ctg1_953	B3	156.260918	Bcer	J	Bcer	FALSE	NA	NA	NA
Bc_ctg1_954	B3	55.8074707	Bcer	S	Bcer	FALSE	NA	NA	NA
Bc_ctg1_955	B3	31.00415039	Bcer	S	Bcer	FALSE	NA	NA	NA
Bc_ctg1_956	B3	1547.614445	Bcer	S	Bcer	FALSE	NA	NA	NA
Bc_ctg1_957	B3	79.48336736	Bcer	P	Bcer	FALSE	NA	NA	NA
Bc_ctg1_958	B3	56.37118253	Bcer	C	Bcer	FALSE	NA	NA	NA
Bc_ctg1_959	B3	117.364802	Bcer	E	Bcer	FALSE	NA	NA	NA
Bc_ctg1_96	B3	1.465650746	Bcer	T	Bcer	FALSE	NA	NA	NA
Bc_ctg1_960	B3	403.955894	Bcer	E	Bcer	FALSE	NA	NA	NA

Bc_ctg1_961	B3	83.09112304	Bcer	K	Bcer	FALSE	NA	NA	NA
Bc_ctg1_962	B3	45.43517312	Bcer	S	Bcer	FALSE	NA	NA	NA
Bc_ctg1_963	B3	3.269528587	Bcer	S	Bcer	FALSE	NA	NA	NA
Bc_ctg1_964	B3	0.112742365	Bcer	S	Bcer	FALSE	NA	NA	NA
Bc_ctg1_966	B3	14.65650746	Bcer	S	Bcer	FALSE	NA	NA	NA
Bc_ctg1_967	B3	1.916620206	Bcer	O	Bcer	FALSE	NA	NA	NA
Bc_ctg1_968	B3	5.862602983	Bcer	S	Bcer	FALSE	NA	NA	NA
Bc_ctg1_969	B3	75.08641512	Bcer	T	Bcer	FALSE	NA	NA	NA
Bc_ctg1_97	B3	1.803877841	Bcer	P	Bcer	FALSE	NA	NA	NA
Bc_ctg1_970	B3	111.1639719	Bcer	IQ	Bcer	TRUE	NA	NA	NA
Bc_ctg1_971	B3	1.578393111	Bcer	S	Bcer	FALSE	NA	NA	NA
Bc_ctg1_972	B3	0	Bcer	NA	Bcer	FALSE	NA	NA	NA
Bc_ctg1_973	B3	192.11299	Bcer	P	Bcer	FALSE	NA	NA	NA
Bc_ctg1_974	B3	35.62658736	Bcer	FP	Bcer	FALSE	NA	NA	NA
Bc_ctg1_975	B3	67.53267667	Bcer	S	Bcer	FALSE	NA	NA	NA
Bc_ctg1_976	B3	39.6853125	Bcer	L	Bcer	FALSE	NA	NA	NA
Bc_ctg1_977	B3	1.014681285	Bcer	T	Bcer	FALSE	NA	NA	NA
Bc_ctg1_978	B3	0.22548473	Bcer	S	Bcer	FALSE	Bc_Ga0417192_01_rn09	Other	NA
Bc_ctg1_979	B3	36.64126864	Bcer	NA	Bcer	FALSE	Bc_Ga0417192_01_rn09	Other	NA
Bc_ctg1_98	B3	0.67645419	Bcer	E	Bcer	FALSE	NA	NA	NA
Bc_ctg1_980	B3	71.14043235	Bcer	P	Bcer	FALSE	Bc_Ga0417192_01_rn09	Transport	SMCOG1005
Bc_ctg1_981	B3	15.89667347	Bcer	K	Bcer	FALSE	Bc_Ga0417192_01_rn09	Regulation	SMCOG1278
Bc_ctg1_982	B3	2797.250819	Bcer	E	Bcer	FALSE	Bc_Ga0417192_01_rn09	Other	NA
Bc_ctg1_983	B3	5.862602983	Bcer	S	Bcer	FALSE	Bc_Ga0417192_01_rn09	Other	NA
Bc_ctg1_984	B3	3.269528587	Bcer	M	Bcer	FALSE	Bc_Ga0417192_01_rn09	Other	NA
Bc_ctg1_985	B3	1.014681285	Bcer	S	Bcer	FALSE	Bc_Ga0417192_01_rn09	Other	NA
Bc_ctg1_986	B3	64.26314808	Bcer	T	Bcer	FALSE	Bc_Ga0417192_01_rn09	Regulation	SMCOG1003
Bc_ctg1_987	B3	28.86204545	Bcer	NA	Bcer	FALSE	Bc_Ga0417192_01_rn09	Other	NA
Bc_ctg1_988	B3	92.22325461	Bcer	E	Bcer	FALSE	Bc_Ga0417192_01_rn09	Core biosynthesis	SMCOG1019
Bc_ctg1_989	B3	89.40469549	Bcer	K	Bcer	FALSE	Bc_Ga0417192_01_rn09	Regulation	SMCOG1078
Bc_ctg1_99	B3	0.67645419	Bcer	K	Bcer	FALSE	NA	NA	NA
Bc_ctg1_990	B3	417.2594931	Bcer	P	Bcer	FALSE	Bc_Ga0417192_01_rn09	Transport	SMCOG1069
Bc_ctg1_991	B3	800.1325648	Bcer	P	Bcer	FALSE	Bc_Ga0417192_01_rn09	Transport	SMCOG1067
Bc_ctg1_992	B3	786.2652539	Bcer	G	Bcer	FALSE	Bc_Ga0417192_01_rn09	Ancillary biosynthesis	SMCOG1143
Bc_ctg1_993	B3	45.99888494	Bcer	G	Bcer	FALSE	Bc_Ga0417192_01_rn09	Core biosynthesis	SMCOG1257
Bc_ctg1_994	B3	0	Bcer	L	Bcer	FALSE	Bc_Ga0417192_01_rn09	Other	SMCOG1026
Bc_ctg1_996	B3	188.1670073	Bcer	G	Bcer	FALSE	Bc_Ga0417192_01_rn09	Core biosynthesis	SMCOG1257
Bc_ctg1_997	B3	459.53788	Bcer	P	Bcer	FALSE	Bc_Ga0417192_01_rn09	Transport	SMCOG1000
Bc_ctg1_998	B3	166.2949885	Bcer	S	Bcer	FALSE	Bc_Ga0417192_01_rn09	Ancillary biosynthesis	SMCOG1001
Bc_ctg2_1	B3	1.803877841	Bcer	M	Bcer	FALSE	Bc_Ga0417192_02_rn01	Core biosynthesis	None
Bc_ctg2_10	B3	0.90193892	Bcer	S	Bcer	FALSE	Bc_Ga0417192_02_rn01	Other	NA
Bc_ctg2_100	B3	12.28891779	Bcer	K	Bcer	FALSE	Bc_Ga0417192_02_rn02	Ancillary biosynthesis	SMCOG1019
Bc_ctg2_101	B3	29.65124201	Bcer	NA	Bcer	FALSE	Bc_Ga0417192_02_rn02	Other	NA
Bc_ctg2_102	B3	175.6526048	Bcer	E	Bcer	FALSE	Bc_Ga0417192_02_rn02	Transport	SMCOG1202
Bc_ctg2_103	B3	1.691135476	Bcer	M	Bcer	FALSE	Bc_Ga0417192_02_rn02	Ancillary biosynthesis	SMCOG1064
Bc_ctg2_104	B3	0.789196555	Bcer	M	Bcer	FALSE	Bc_Ga0417192_02_rn02	Ancillary biosynthesis	SMCOG1152
Bc_ctg2_105	B3	0.338227095	Bcer	M	Bcer	FALSE	Bc_Ga0417192_02_rn02	Core biosynthesis	SMCOG1010
Bc_ctg2_106	B3	0.338227095	Bcer	M	Bcer	FALSE	Bc_Ga0417192_02_rn02	Core biosynthesis	SMCOG1045
Bc_ctg2_107	B3	0	Bcer	NA	Bcer	FALSE	Bc_Ga0417192_02_rn02	Other	NA
Bc_ctg2_108	B3	0.338227095	Bcer	NA	Bcer	FALSE	Bc_Ga0417192_02_rn02	Other	NA
Bc_ctg2_109	B3	0.67645419	Bcer	S	Bcer	FALSE	Bc_Ga0417192_02_rn02	Other	NA
Bc_ctg2_11	B3	0.22548473	Bcer	NA	Bcer	FALSE	Bc_Ga0417192_02_rn01	Other	NA
Bc_ctg2_110	B3	0.563711825	Bcer	M	Bcer	FALSE	Bc_Ga0417192_02_rn02	Core biosynthesis	SMCOG1045
Bc_ctg2_111	B3	0.67645419	Bcer	S	Bcer	FALSE	Bc_Ga0417192_02_rn02	Ancillary biosynthesis	None
Bc_ctg2_116	B3	2.029362571	Bcer	NA	Bcer	FALSE	Bc_Ga0417192_02_rn02	Other	NA
Bc_ctg2_118	B3	5.073406427	Bcer	K	Bcer	FALSE	Bc_Ga0417192_02_rn02	Other	NA
Bc_ctg2_119	B3	11.04875178	Bcer	E	Bcer	FALSE	Bc_Ga0417192_02_rn02	Ancillary biosynthesis	SMCOG1158
Bc_ctg2_12	B3	0	Bcer	NA	Bcer	FALSE	Bc_Ga0417192_02_rn01	Other	NA
Bc_ctg2_120	B3	8.681162109	Bcer	E	Bcer	FALSE	Bc_Ga0417192_02_rn02	Ancillary biosynthesis	SMCOG1081
Bc_ctg2_121	B3	11.9506907	Bcer	K	Bcer	FALSE	Bc_Ga0417192_02_rn02	Other	NA
Bc_ctg2_122	B3	0.22548473	Bcer	NA	Bcer	FALSE	Bc_Ga0417192_02_rn02	Other	NA
Bc_ctg2_123	B3	1.803877841	Bcer	Q	Bcer	TRUE	Bc_Ga0417192_02_rn02	Ancillary biosynthesis	SMCOG1004

Bc_ctg2_124	B3	22.54847301	Bcer	Q	Bcer	TRUE	Bc_Ga0417192_02_rn02	Core biosynthesis	SMCOG1127
Bc_ctg2_125	B3	8.117450284	Bcer	Q	Bcer	TRUE	Bc_Ga0417192_02_rn02	Core biosynthesis	SMCOG1127
Bc_ctg2_126	B3	17.02409712	Bcer	IQ	Bcer	TRUE	Bc_Ga0417192_02_rn02	Core biosynthesis	SMCOG1127
Bc_ctg2_127	B3	2.029362571	Bcer	S	Bcer	FALSE	Bc_Ga0417192_02_rn02	Other	NA
Bc_ctg2_128	B3	60.54265003	Bcer	EGP	Bcer	FALSE	Bc_Ga0417192_02_rn02	Transport	SMCOG1202
Bc_ctg2_129	B3	76.10109641	Bcer	K	Bcer	FALSE	Bc_Ga0417192_02_rn02	Other	NA
Bc_ctg2_130	B3	18.82797496	Bcer	EG	Bcer	FALSE	Bc_Ga0417192_02_rn02	Transport	SMCOG1166
Bc_ctg2_131	B3	6.088087713	Bcer	K	Bcer	FALSE	Bc_Ga0417192_02_rn02	Regulation	SMCOG1071
Bc_ctg2_132	B3	11.04875178	Bcer	EG	Bcer	FALSE	Bc_Ga0417192_02_rn02	Transport	SMCOG1166
Bc_ctg2_133	B3	1.127423651	Bcer	NA	Bcer	FALSE	Bc_Ga0417192_02_rn02	Other	NA
Bc_ctg2_134	B3	5.186148792	Bcer	C	Bcer	FALSE	Bc_Ga0417192_02_rn02	Ancillary biosynthesis	None
Bc_ctg2_135	B3	0.338227095	Bcer	K	Bcer	FALSE	Bc_Ga0417192_02_rn02	Other	NA
Bc_ctg2_136	B3	0.789196555	Bcer	K	Bcer	FALSE	Bc_Ga0417192_02_rn02	Other	NA
Bc_ctg2_137	B3	8.568419744	Bcer	K	Bcer	FALSE	Bc_Ga0417192_02_rn02	Other	NA
Bc_ctg2_139	B3	0.112742365	Bcer	NA	Bcer	FALSE	Bc_Ga0417192_02_rn02	Other	NA
Bc_ctg2_14	B3	33.59722479	Bcer	S	Bcer	FALSE	Bc_Ga0417192_02_rn01	Other	NA
Bc_ctg2_140	B3	0.112742365	Bcer	L	Bcer	FALSE	Bc_Ga0417192_02_rn02	Other	SMCOG1132
Bc_ctg2_141	B3	0.67645419	Bcer	L	Bcer	FALSE	Bc_Ga0417192_02_rn02	Other	NA
Bc_ctg2_142	B3	0	Bcer	L	Bcer	FALSE	Bc_Ga0417192_02_rn02	Other	NA
Bc_ctg2_143	B3	16.79861239	Bcer	V	Bcer	FALSE	Bc_Ga0417192_02_rn02	Ancillary biosynthesis	SMCOG1053
Bc_ctg2_144	B3	0.112742365	Bcer	NA	Bcer	FALSE	Bc_Ga0417192_02_rn02	Other	NA
Bc_ctg2_145	B3	2.593074396	Bcer	L	Bcer	FALSE	NA	NA	NA
Bc_ctg2_146	B3	1.240166016	Bcer	S	Bcer	FALSE	NA	NA	NA
Bc_ctg2_147	B3	4.284209872	Bcer	NA	Bcer	FALSE	NA	NA	NA
Bc_ctg2_148	B3	39.23434304	Bcer	NA	Bcer	FALSE	NA	NA	NA
Bc_ctg2_149	B3	1.240166016	Bcer	CO	Bcer	FALSE	NA	NA	NA
Bc_ctg2_15	B3	67.7581614	Bcer	P	Bcer	FALSE	Bc_Ga0417192_02_rn01	Ancillary biosynthesis	SMCOG1170
Bc_ctg2_150	B3	0	Bcer	P	Bcer	FALSE	NA	NA	NA
Bc_ctg2_152	B3	0	Bcer	NA	Bcer	FALSE	NA	NA	NA
Bc_ctg2_153	B3	4.284209872	Bcer	L	Bcer	FALSE	NA	NA	NA
Bc_ctg2_154	B3	3.607755682	Bcer	NA	Bcer	FALSE	NA	NA	NA
Bc_ctg2_156	B3	0.112742365	Bcer	NA	Bcer	FALSE	NA	NA	NA
Bc_ctg2_157	B3	0.22548473	Bcer	NA	Bcer	FALSE	NA	NA	NA
Bc_ctg2_158	B3	0	Bcer	L	Bcer	FALSE	NA	NA	NA
Bc_ctg2_159	B3	0.45096946	Bcer	NA	Bcer	FALSE	NA	NA	NA
Bc_ctg2_16	B3	12.17617543	Bcer	NA	Bcer	FALSE	Bc_Ga0417192_02_rn01	Other	NA
Bc_ctg2_160	B3	0.112742365	Bcer	S	Bcer	FALSE	NA	NA	NA
Bc_ctg2_161	B3	0.22548473	Bcer	L	Bcer	FALSE	NA	NA	NA
Bc_ctg2_162	B3	0	Bcer	S	Bcer	FALSE	NA	NA	NA
Bc_ctg2_166	B3	0	Bcer	NA	Bcer	FALSE	NA	NA	NA
Bc_ctg2_167	B3	0	Bcer	S	Bcer	FALSE	NA	NA	NA
Bc_ctg2_169	B3	27.62187944	Bcer	G	Bcer	FALSE	NA	NA	NA
Bc_ctg2_17	B3	0.22548473	Bcer	V	Bcer	FALSE	NA	NA	NA
Bc_ctg2_170	B3	2.367589666	Bcer	K	Bcer	FALSE	NA	NA	NA
Bc_ctg2_171	B3	2.931301491	Bcer	NA	Bcer	FALSE	NA	NA	NA
Bc_ctg2_172	B3	2.480332031	Bcer	O	Bcer	FALSE	NA	NA	NA
Bc_ctg2_173	B3	5.073406427	Bcer	K	Bcer	FALSE	NA	NA	NA
Bc_ctg2_174	B3	35.1756179	Bcer	V	Bcer	FALSE	NA	NA	NA
Bc_ctg2_176	B3	1.127423651	Bcer	NA	Bcer	FALSE	NA	NA	NA
Bc_ctg2_177	B3	0.338227095	Bcer	NA	Bcer	FALSE	NA	NA	NA
Bc_ctg2_178	B3	14.54376509	Bcer	V	Bcer	FALSE	NA	NA	NA
Bc_ctg2_179	B3	1.465650746	Bcer	H	Bcer	FALSE	NA	NA	NA
Bc_ctg2_18	B3	11.27423651	Bcer	V	Bcer	FALSE	NA	NA	NA
Bc_ctg2_180	B3	0.338227095	Bcer	NA	Bcer	FALSE	NA	NA	NA
Bc_ctg2_181	B3	1.014681285	Bcer	S	Bcer	FALSE	NA	NA	NA
Bc_ctg2_182	B3	0.112742365	Bcer	NA	Bcer	FALSE	NA	NA	NA
Bc_ctg2_184	B3	0	Bcer	NA	Bcer	FALSE	NA	NA	NA
Bc_ctg2_185	B3	0.22548473	Bcer	L	Bcer	FALSE	NA	NA	NA
Bc_ctg2_186	B3	0.789196555	Bcer	L	Bcer	FALSE	NA	NA	NA
Bc_ctg2_187	B3	0.22548473	Bcer	S	Bcer	FALSE	NA	NA	NA
Bc_ctg2_188	B3	2.931301491	Bcer	NA	Bcer	FALSE	NA	NA	NA
Bc_ctg2_189	B3	3.833240412	Bcer	L	Bcer	FALSE	NA	NA	NA

Bc_ctg2_19	B3	5.975345348	Bcer	T	Bcer	FALSE	NA	NA	NA
Bc_ctg2_190	B3	0.90193892	Bcer	NA	Bcer	FALSE	NA	NA	NA
Bc_ctg2_191	B3	18.03877841	Bcer	NA	Bcer	FALSE	NA	NA	NA
Bc_ctg2_192	B3	67.19444957	Bcer	S	Bcer	FALSE	NA	NA	NA
Bc_ctg2_193	B3	2.818559126	Bcer	S	Bcer	FALSE	NA	NA	NA
Bc_ctg2_194	B3	0	Bcer	CO	Bcer	FALSE	NA	NA	NA
Bc_ctg2_195	B3	0	Bcer	P	Bcer	FALSE	NA	NA	NA
Bc_ctg2_196	B3	1.465650746	Bcer	NA	Bcer	FALSE	NA	NA	NA
Bc_ctg2_197	B3	1.691135476	Bcer	V	Bcer	FALSE	NA	NA	NA
Bc_ctg2_198	B3	0	Bcer	S	Bcer	FALSE	NA	NA	NA
Bc_ctg2_199	B3	0.22548473	Bcer	NA	Bcer	FALSE	NA	NA	NA
Bc_ctg2_2	B3	0.112742365	Bcer	M	Bcer	FALSE	Bc_Ga0417192_02_rn01	Core biosynthesis	None
Bc_ctg2_20	B3	13.98005327	Bcer	T	Bcer	FALSE	NA	NA	NA
Bc_ctg2_200	B3	1.916620206	Bcer	NA	Bcer	FALSE	NA	NA	NA
Bc_ctg2_201	B3	5.186148792	Bcer	M	Bcer	FALSE	NA	NA	NA
Bc_ctg2_202	B3	2.254847301	Bcer	NA	Bcer	FALSE	NA	NA	NA
Bc_ctg2_203	B3	0.45096946	Bcer	U	Bcer	FALSE	NA	NA	NA
Bc_ctg2_204	B3	0	Bcer	NA	Bcer	FALSE	NA	NA	NA
Bc_ctg2_205	B3	0	Bcer	NA	Bcer	FALSE	NA	NA	NA
Bc_ctg2_206	B3	0.112742365	Bcer	NA	Bcer	FALSE	NA	NA	NA
Bc_ctg2_207	B3	0	Bcer	NA	Bcer	FALSE	NA	NA	NA
Bc_ctg2_208	B3	0.112742365	Bcer	NA	Bcer	FALSE	NA	NA	NA
Bc_ctg2_209	B3	0	Bcer	NA	Bcer	FALSE	NA	NA	NA
Bc_ctg2_21	B3	6.764541903	Bcer	T	Bcer	FALSE	NA	NA	NA
Bc_ctg2_210	B3	0	Bcer	L	Bcer	FALSE	NA	NA	NA
Bc_ctg2_211	B3	0.338227095	Bcer	NA	Bcer	FALSE	NA	NA	NA
Bc_ctg2_212	B3	1.014681285	Bcer	S	Bcer	FALSE	NA	NA	NA
Bc_ctg2_213	B3	0	Bcer	NA	Bcer	FALSE	NA	NA	NA
Bc_ctg2_214	B3	46.11162731	Bcer	S	Bcer	FALSE	NA	NA	NA
Bc_ctg2_215	B3	3.044043856	Bcer	M	Bcer	FALSE	NA	NA	NA
Bc_ctg2_216	B3	0.789196555	Bcer	S	Bcer	FALSE	NA	NA	NA
Bc_ctg2_217	B3	2.931301491	Bcer	S	Bcer	FALSE	NA	NA	NA
Bc_ctg2_218	B3	0	Bcer	S	Bcer	FALSE	NA	NA	NA
Bc_ctg2_22	B3	4.396952237	Bcer	T	Bcer	FALSE	NA	NA	NA
Bc_ctg2_220	B3	0	Bcer	NA	Bcer	FALSE	NA	NA	NA
Bc_ctg2_221	B3	0.789196555	Bcer	NA	Bcer	FALSE	NA	NA	NA
Bc_ctg2_223	B3	2.705816761	Bcer	NA	Bcer	FALSE	NA	NA	NA
Bc_ctg2_224	B3	1.465650746	Bcer	NA	Bcer	FALSE	NA	NA	NA
Bc_ctg2_225	B3	0.338227095	Bcer	NA	Bcer	FALSE	NA	NA	NA
Bc_ctg2_226	B3	0	Bcer	NA	Bcer	FALSE	NA	NA	NA
Bc_ctg2_227	B3	0.22548473	Bcer	NA	Bcer	FALSE	NA	NA	NA
Bc_ctg2_228	B3	0.22548473	Bcer	NA	Bcer	FALSE	NA	NA	NA
Bc_ctg2_229	B3	0.112742365	Bcer	NA	Bcer	FALSE	NA	NA	NA
Bc_ctg2_23	B3	2.367589666	Bcer	S	Bcer	FALSE	NA	NA	NA
Bc_ctg2_230	B3	1.578393111	Bcer	NA	Bcer	FALSE	NA	NA	NA
Bc_ctg2_231	B3	0.45096946	Bcer	NA	Bcer	FALSE	NA	NA	NA
Bc_ctg2_232	B3	1.240166016	Bcer	U	Bcer	FALSE	NA	NA	NA
Bc_ctg2_233	B3	0.22548473	Bcer	D	Bcer	FALSE	NA	NA	NA
Bc_ctg2_234	B3	0.112742365	Bcer	U	Bcer	FALSE	NA	NA	NA
Bc_ctg2_235	B3	0.112742365	Bcer	NA	Bcer	FALSE	NA	NA	NA
Bc_ctg2_236	B3	2.254847301	Bcer	M	Bcer	FALSE	NA	NA	NA
Bc_ctg2_237	B3	30.66592329	Bcer	GM	Bcer	FALSE	NA	NA	NA
Bc_ctg2_238	B3	0.112742365	Bcer	NA	Bcer	FALSE	NA	NA	NA
Bc_ctg2_239	B3	1.014681285	Bcer	NA	Bcer	FALSE	NA	NA	NA
Bc_ctg2_24	B3	5.073406427	Bcer	V	Bcer	FALSE	NA	NA	NA
Bc_ctg2_240	B3	2.931301491	Bcer	D	Bcer	FALSE	NA	NA	NA
Bc_ctg2_241	B3	1.240166016	Bcer	NA	Bcer	FALSE	NA	NA	NA
Bc_ctg2_242	B3	0	Bcer	NA	Bcer	FALSE	NA	NA	NA
Bc_ctg2_243	B3	78.24320135	Bcer	NA	Bcer	FALSE	NA	NA	NA
Bc_ctg2_244	B3	182.8681161	Bcer	D	Bcer	FALSE	NA	NA	NA
Bc_ctg2_245	B3	28.41107599	Bcer	NA	Bcer	FALSE	NA	NA	NA
Bc_ctg2_246	B3	7.328253728	Bcer	L	Bcer	FALSE	NA	NA	NA

Bc_ctg2_247	B3	12.40166016	Bcer	NA	Bcer	FALSE	NA	NA	NA
Bc_ctg2_248	B3	0.22548473	Bcer	NA	Bcer	FALSE	NA	NA	NA
Bc_ctg2_249	B3	16.23490057	Bcer	U	Bcer	FALSE	NA	NA	NA
Bc_ctg2_25	B3	30.77866566	Bcer	V	Bcer	FALSE	NA	NA	NA
Bc_ctg2_250	B3	2.367589666	Bcer	J	Bcer	FALSE	NA	NA	NA
Bc_ctg2_251	B3	4.284209872	Bcer	NA	Bcer	FALSE	NA	NA	NA
Bc_ctg2_252	B3	15.10747692	Bcer	NA	Bcer	FALSE	NA	NA	NA
Bc_ctg2_253	B3	0	Bcer	NA	Bcer	FALSE	NA	NA	NA
Bc_ctg2_254	B3	1.240166016	Bcer	NA	Bcer	FALSE	NA	NA	NA
Bc_ctg2_255	B3	40.02353959	Bcer	S	Bcer	FALSE	NA	NA	NA
Bc_ctg2_256	B3	1.691135476	Bcer	NA	Bcer	FALSE	NA	NA	NA
Bc_ctg2_257	B3	0	Bcer	NA	Bcer	FALSE	NA	NA	NA
Bc_ctg2_259	B3	0	Bcer	NA	Bcer	FALSE	NA	NA	NA
Bc_ctg2_26	B3	12.62714489	Bcer	NA	Bcer	FALSE	NA	NA	NA
Bc_ctg2_260	B3	6.200830078	Bcer	NA	Bcer	FALSE	NA	NA	NA
Bc_ctg2_261	B3	6.088087713	Bcer	NA	Bcer	FALSE	NA	NA	NA
Bc_ctg2_262	B3	53.43988103	Bcer	K	Bcer	FALSE	NA	NA	NA
Bc_ctg2_263	B3	15.89667347	Bcer	S	Bcer	FALSE	NA	NA	NA
Bc_ctg2_264	B3	286.0273801	Bcer	S	Bcer	FALSE	NA	NA	NA
Bc_ctg2_265	B3	604.8627885	Bcer	E	Bcer	FALSE	NA	NA	NA
Bc_ctg2_266	B3	740.604596	Bcer	E	Bcer	FALSE	NA	NA	NA
Bc_ctg2_267	B3	0	Bcer	NA	Bcer	FALSE	NA	NA	NA
Bc_ctg2_268	B3	0	Bcer	L	Bcer	FALSE	NA	NA	NA
Bc_ctg2_269	B3	0	Bcer	NA	Bcer	FALSE	NA	NA	NA
Bc_ctg2_27	B3	3.495013317	Bcer	NA	Bcer	FALSE	NA	NA	NA
Bc_ctg2_270	B3	52.65068448	Bcer	S	Bcer	FALSE	NA	NA	NA
Bc_ctg2_271	B3	23.2249272	Bcer	S	Bcer	FALSE	NA	NA	NA
Bc_ctg2_272	B3	5.524375888	Bcer	EH	Bcer	FALSE	NA	NA	NA
Bc_ctg2_273	B3	6.088087713	Bcer	NA	Bcer	FALSE	NA	NA	NA
Bc_ctg2_275	B3	16.4603853	Bcer	S	Bcer	FALSE	NA	NA	NA
Bc_ctg2_276	B3	4.735179332	Bcer	L	Bcer	FALSE	NA	NA	NA
Bc_ctg2_278	B3	29.9894691	Bcer	NA	Bcer	FALSE	NA	NA	NA
Bc_ctg2_279	B3	94.92907137	Bcer	K	Bcer	FALSE	NA	NA	NA
Bc_ctg2_28	B3	3.156786221	Bcer	EGP	Bcer	FALSE	NA	NA	NA
Bc_ctg2_280	B3	2.367589666	Bcer	S	Bcer	FALSE	NA	NA	NA
Bc_ctg2_281	B3	7.215511363	Bcer	O	Bcer	FALSE	NA	NA	NA
Bc_ctg2_282	B3	0.563711825	Bcer	S	Bcer	FALSE	NA	NA	NA
Bc_ctg2_283	B3	32.80802823	Bcer	S	Bcer	FALSE	NA	NA	NA
Bc_ctg2_284	B3	43.40581054	Bcer	S	Bcer	FALSE	NA	NA	NA
Bc_ctg2_285	B3	2.705816761	Bcer	NA	Bcer	FALSE	NA	NA	NA
Bc_ctg2_286	B3	4.284209872	Bcer	S	Bcer	FALSE	NA	NA	NA
Bc_ctg2_287	B3	9.80858576	Bcer	NT	Bcer	FALSE	NA	NA	NA
Bc_ctg2_288	B3	0.789196555	Bcer	S	Bcer	FALSE	NA	NA	NA
Bc_ctg2_289	B3	93.68890536	Bcer	NA	Bcer	FALSE	NA	NA	NA
Bc_ctg2_29	B3	1.803877841	Bcer	M	Bcer	FALSE	NA	NA	NA
Bc_ctg2_290	B3	0	Bcer	NA	Bcer	FALSE	NA	NA	NA
Bc_ctg2_291	B3	17.24958185	Bcer	K	Bcer	FALSE	NA	NA	NA
Bc_ctg2_292	B3	0.45096946	Bcer	S	Bcer	FALSE	NA	NA	NA
Bc_ctg2_293	B3	4.058725142	Bcer	O	Bcer	FALSE	NA	NA	NA
Bc_ctg2_294	B3	0.563711825	Bcer	S	Bcer	FALSE	NA	NA	NA
Bc_ctg2_295	B3	3.269528587	Bcer	S	Bcer	FALSE	NA	NA	NA
Bc_ctg2_296	B3	0.112742365	Bcer	NA	Bcer	FALSE	NA	NA	NA
Bc_ctg2_297	B3	3.495013317	Bcer	V	Bcer	FALSE	NA	NA	NA
Bc_ctg2_298	B3	2.029362571	Bcer	NA	Bcer	FALSE	NA	NA	NA
Bc_ctg2_299	B3	0.90193892	Bcer	S	Bcer	FALSE	NA	NA	NA
Bc_ctg2_3	B3	0.112742365	Bcer	M	Bcer	FALSE	Bc_Ga0417192_02_rn01	Core biosynthesis	SMCOG1010
Bc_ctg2_30	B3	2.254847301	Bcer	S	Bcer	FALSE	NA	NA	NA
Bc_ctg2_300	B3	11.49972124	Bcer	L	Bcer	FALSE	NA	NA	NA
Bc_ctg2_303	B3	3.382270952	Bcer	O	Bcer	FALSE	NA	NA	NA
Bc_ctg2_304	B3	0	Bcer	NA	Bcer	FALSE	NA	NA	NA
Bc_ctg2_305	B3	1.127423651	Bcer	DZ	Bcer	FALSE	NA	NA	NA
Bc_ctg2_307	B3	0.112742365	Bcer	O	Bcer	FALSE	NA	NA	NA

Bc_ctg2_309	B3	0	Bcer	NA	Bcer	FALSE	NA	NA	NA
Bc_ctg2_31	B3	2.142104936	Bcer	NA	Bcer	FALSE	NA	NA	NA
Bc_ctg2_310	B3	0.67645419	Bcer	GM	Bcer	FALSE	NA	NA	NA
Bc_ctg2_311	B3	3.044043856	Bcer	E	Bcer	FALSE	NA	NA	NA
Bc_ctg2_312	B3	4.284209872	Bcer	K	Bcer	FALSE	NA	NA	NA
Bc_ctg2_313	B3	0.22548473	Bcer	K	Bcer	FALSE	NA	NA	NA
Bc_ctg2_314	B3	1.691135476	Bcer	L	Bcer	FALSE	NA	NA	NA
Bc_ctg2_315	B3	0.22548473	Bcer	S	Bcer	FALSE	NA	NA	NA
Bc_ctg2_316	B3	550.2954838	Bcer	M	Bcer	FALSE	NA	NA	NA
Bc_ctg2_317	B3	60.54265003	Bcer	M	Bcer	FALSE	NA	NA	NA
Bc_ctg2_318	B3	81.85095703	Bcer	S	Bcer	FALSE	NA	NA	NA
Bc_ctg2_319	B3	3.044043856	Bcer	S	Bcer	FALSE	NA	NA	NA
Bc_ctg2_32	B3	0.112742365	Bcer	C	Bcer	FALSE	NA	NA	NA
Bc_ctg2_320	B3	100.7916744	Bcer	S	Bcer	FALSE	NA	NA	NA
Bc_ctg2_322	B3	0	Bcer	NA	Bcer	FALSE	NA	NA	NA
Bc_ctg2_323	B3	15.22021928	Bcer	NA	Bcer	FALSE	NA	NA	NA
Bc_ctg2_324	B3	0.112742365	Bcer	NA	Bcer	FALSE	NA	NA	NA
Bc_ctg2_325	B3	0	Bcer	NA	Bcer	FALSE	NA	NA	NA
Bc_ctg2_326	B3	0	Bcer	L	Bcer	FALSE	NA	NA	NA
Bc_ctg2_327	B3	0	Bcer	NA	Bcer	FALSE	NA	NA	NA
Bc_ctg2_328	B3	0.338227095	Bcer	H	Bcer	FALSE	NA	NA	NA
Bc_ctg2_329	B3	0	Bcer	NA	Bcer	FALSE	NA	NA	NA
Bc_ctg2_33	B3	3.156786221	Bcer	IQ	Bcer	TRUE	NA	NA	NA
Bc_ctg2_330	B3	0.563711825	Bcer	T	Bcer	FALSE	NA	NA	NA
Bc_ctg2_331	B3	6.200830078	Bcer	T	Bcer	FALSE	NA	NA	NA
Bc_ctg2_332	B3	26.60719815	Bcer	M	Bcer	FALSE	NA	NA	NA
Bc_ctg2_333	B3	14.76924982	Bcer	K	Bcer	FALSE	NA	NA	NA
Bc_ctg2_334	B3	0.112742365	Bcer	E	Bcer	FALSE	NA	NA	NA
Bc_ctg2_335	B3	2.367589666	Bcer	NA	Bcer	FALSE	NA	NA	NA
Bc_ctg2_336	B3	10.14681285	Bcer	G	Bcer	FALSE	NA	NA	NA
Bc_ctg2_337	B3	4.622436967	Bcer	K	Bcer	FALSE	NA	NA	NA
Bc_ctg2_338	B3	15.67118874	Bcer	E	Bcer	FALSE	NA	NA	NA
Bc_ctg2_339	B3	0.112742365	Bcer	K	Bcer	FALSE	NA	NA	NA
Bc_ctg2_34	B3	0	Bcer	S	Bcer	FALSE	NA	NA	NA
Bc_ctg2_340	B3	0.789196555	Bcer	S	Bcer	FALSE	NA	NA	NA
Bc_ctg2_341	B3	0	Bcer	K	Bcer	FALSE	NA	NA	NA
Bc_ctg2_342	B3	1.916620206	Bcer	E	Bcer	FALSE	NA	NA	NA
Bc_ctg2_343	B3	0.338227095	Bcer	K	Bcer	FALSE	NA	NA	NA
Bc_ctg2_344	B3	9.019389204	Bcer	S	Bcer	FALSE	NA	NA	NA
Bc_ctg2_345	B3	46.22436967	Bcer	K	Bcer	FALSE	NA	NA	NA
Bc_ctg2_346	B3	1.578393111	Bcer	L	Bcer	FALSE	NA	NA	NA
Bc_ctg2_347	B3	4.396952237	Bcer	L	Bcer	FALSE	NA	NA	NA
Bc_ctg2_348	B3	2.142104936	Bcer	NA	Bcer	FALSE	NA	NA	NA
Bc_ctg2_349	B3	15.33296165	Bcer	L	Bcer	FALSE	NA	NA	NA
Bc_ctg2_35	B3	1.578393111	Bcer	NA	Bcer	FALSE	NA	NA	NA
Bc_ctg2_350	B3	6.764541903	Bcer	L	Bcer	FALSE	NA	NA	NA
Bc_ctg2_351	B3	0.67645419	Bcer	K	Bcer	FALSE	NA	NA	NA
Bc_ctg2_352	B3	2.142104936	Bcer	NA	Bcer	FALSE	NA	NA	NA
Bc_ctg2_353	B3	13.07811435	Bcer	NA	Bcer	FALSE	NA	NA	NA
Bc_ctg2_354	B3	13.52908381	Bcer	S	Bcer	FALSE	NA	NA	NA
Bc_ctg2_355	B3	9.357616299	Bcer	NA	Bcer	FALSE	NA	NA	NA
Bc_ctg2_356	B3	3.269528587	Bcer	NA	Bcer	FALSE	NA	NA	NA
Bc_ctg2_359	B3	4.847921697	Bcer	CH	Bcer	FALSE	NA	NA	NA
Bc_ctg2_36	B3	0	Bcer	NA	Bcer	FALSE	NA	NA	NA
Bc_ctg2_360	B3	10.93600941	Bcer	L	Bcer	FALSE	NA	NA	NA
Bc_ctg2_361	B3	2.254847301	Bcer	NA	Bcer	FALSE	NA	NA	NA
Bc_ctg2_362	B3	6.313572443	Bcer	KT	Bcer	FALSE	NA	NA	NA
Bc_ctg2_363	B3	16.57312766	Bcer	K	Bcer	FALSE	NA	NA	NA
Bc_ctg2_364	B3	8.793904474	Bcer	P	Bcer	FALSE	NA	NA	NA
Bc_ctg2_365	B3	2.593074396	Bcer	NA	Bcer	FALSE	NA	NA	NA
Bc_ctg2_366	B3	21.87201882	Bcer	S	Bcer	FALSE	NA	NA	NA
Bc_ctg2_367	B3	7.440996093	Bcer	L	Bcer	FALSE	NA	NA	NA

Bc_ctg2_368	B3	14.65650746	Bcer	S	Bcer	FALSE	NA	NA	NA
Bc_ctg2_369	B3	0.67645419	Bcer	NA	Bcer	FALSE	NA	NA	NA
Bc_ctg2_37	B3	1.014681285	Bcer	NA	Bcer	FALSE	NA	NA	NA
Bc_ctg2_370	B3	0.789196555	Bcer	P	Bcer	FALSE	NA	NA	NA
Bc_ctg2_371	B3	0.112742365	Bcer	NA	Bcer	FALSE	NA	NA	NA
Bc_ctg2_372	B3	18.7152326	Bcer	S	Bcer	FALSE	NA	NA	NA
Bc_ctg2_373	B3	12.85262962	Bcer	NA	Bcer	FALSE	NA	NA	NA
Bc_ctg2_374	B3	0	Bcer	NA	Bcer	FALSE	NA	NA	NA
Bc_ctg2_375	B3	1.240166016	Bcer	NA	Bcer	FALSE	NA	NA	NA
Bc_ctg2_376	B3	47.35179332	Bcer	LO	Bcer	FALSE	NA	NA	NA
Bc_ctg2_377	B3	24.91606268	Bcer	L	Bcer	FALSE	NA	NA	NA
Bc_ctg2_378	B3	34.27367898	Bcer	V	Bcer	FALSE	NA	NA	NA
Bc_ctg2_379	B3	23.2249272	Bcer	V	Bcer	FALSE	NA	NA	NA
Bc_ctg2_38	B3	3.607755682	Bcer	NA	Bcer	FALSE	NA	NA	NA
Bc_ctg2_380	B3	6.313572443	Bcer	NA	Bcer	FALSE	NA	NA	NA
Bc_ctg2_381	B3	29.87672674	Bcer	NA	Bcer	FALSE	NA	NA	NA
Bc_ctg2_382	B3	0.563711825	Bcer	NA	Bcer	FALSE	NA	NA	NA
Bc_ctg2_383	B3	0.45096946	Bcer	NA	Bcer	FALSE	NA	NA	NA
Bc_ctg2_384	B3	116.8010902	Bcer	D	Bcer	FALSE	NA	NA	NA
Bc_ctg2_385	B3	21.08282226	Bcer	NA	Bcer	FALSE	NA	NA	NA
Bc_ctg2_386	B3	116.9138326	Bcer	NA	Bcer	FALSE	NA	NA	NA
Bc_ctg2_387	B3	1.240166016	Bcer	NA	Bcer	FALSE	NA	NA	NA
Bc_ctg2_388	B3	5.975345348	Bcer	NA	Bcer	FALSE	NA	NA	NA
Bc_ctg2_389	B3	28.74930309	Bcer	NA	Bcer	FALSE	Bc_Ga0417192_02_rn03	Other	NA
Bc_ctg2_39	B3	34.95013317	Bcer	NA	Bcer	FALSE	NA	NA	NA
Bc_ctg2_390	B3	59.52796875	Bcer	NA	Bcer	FALSE	Bc_Ga0417192_02_rn03	Other	NA
Bc_ctg2_391	B3	185.4611905	Bcer	NA	Bcer	FALSE	Bc_Ga0417192_02_rn03	Other	NA
Bc_ctg2_392	B3	101.1299015	Bcer	T	Bcer	FALSE	Bc_Ga0417192_02_rn03	Other	NA
Bc_ctg2_393	B3	46.56259677	Bcer	NA	Bcer	FALSE	Bc_Ga0417192_02_rn03	Other	NA
Bc_ctg2_394	B3	100.3407049	Bcer	L	Bcer	FALSE	Bc_Ga0417192_02_rn03	Other	SMCOG1077
Bc_ctg2_395	B3	13.41634144	Bcer	K	Bcer	FALSE	Bc_Ga0417192_02_rn03	Other	SMCOG1148
Bc_ctg2_396	B3	0.112742365	Bcer	K	Bcer	FALSE	Bc_Ga0417192_02_rn03	Other	NA
Bc_ctg2_397	B3	0.563711825	Bcer	NA	Bcer	FALSE	Bc_Ga0417192_02_rn03	Other	NA
Bc_ctg2_398	B3	41.37644797	Bcer	EGP	Bcer	FALSE	Bc_Ga0417192_02_rn03	Transport	SMCOG1005
Bc_ctg2_399	B3	19.50442915	Bcer	UW	Bcer	FALSE	Bc_Ga0417192_02_rn03	Other	NA
Bc_ctg2_4	B3	0.112742365	Bcer	M	Bcer	FALSE	Bc_Ga0417192_02_rn01	Core biosynthesis	SMCOG1010
Bc_ctg2_40	B3	25.4797745	Bcer	L	Bcer	FALSE	NA	NA	NA
Bc_ctg2_400	B3	0.112742365	Bcer	NA	Bcer	FALSE	Bc_Ga0417192_02_rn03	Other	NA
Bc_ctg2_401	B3	1.916620206	Bcer	S	Bcer	FALSE	Bc_Ga0417192_02_rn03	Other	NA
Bc_ctg2_402	B3	1.240166016	Bcer	NA	Bcer	FALSE	Bc_Ga0417192_02_rn03	Other	NA
Bc_ctg2_403	B3	49.26841353	Bcer	NA	Bcer	FALSE	Bc_Ga0417192_02_rn03	Other	NA
Bc_ctg2_404	B3	0.789196555	Bcer	CO	Bcer	FALSE	Bc_Ga0417192_02_rn03	Other	NA
Bc_ctg2_405	B3	16.91135476	Bcer	NA	Bcer	FALSE	Bc_Ga0417192_02_rn03	Other	NA
Bc_ctg2_406	B3	27.17090998	Bcer	E	Bcer	FALSE	Bc_Ga0417192_02_rn03	Ancillary biosynthesis	SMCOG1180
Bc_ctg2_407	B3	0.45096946	Bcer	C	Bcer	FALSE	Bc_Ga0417192_02_rn03	Ancillary biosynthesis	SMCOG1039
Bc_ctg2_408	B3	175.7653471	Bcer	Q	Bcer	TRUE	Bc_Ga0417192_02_rn03	Core biosynthesis	SMCOG1127
Bc_ctg2_409	B3	0.112742365	Bcer	NA	Bcer	FALSE	Bc_Ga0417192_02_rn03	Other	NA
Bc_ctg2_41	B3	0.112742365	Bcer	NA	Bcer	FALSE	NA	NA	NA
Bc_ctg2_410	B3	4.622436967	Bcer	NA	Bcer	FALSE	Bc_Ga0417192_02_rn03	Other	NA
Bc_ctg2_411	B3	15.22021928	Bcer	NA	Bcer	FALSE	Bc_Ga0417192_02_rn03	Other	NA
Bc_ctg2_412	B3	29.87672674	Bcer	NA	Bcer	FALSE	Bc_Ga0417192_02_rn03	Other	NA
Bc_ctg2_413	B3	6.200830078	Bcer	NA	Bcer	FALSE	Bc_Ga0417192_02_rn03	Other	NA
Bc_ctg2_414	B3	0	Bcer	L	Bcer	FALSE	Bc_Ga0417192_02_rn03	Other	NA
Bc_ctg2_415	B3	1.240166016	Bcer	L	Bcer	FALSE	Bc_Ga0417192_02_rn03	Other	NA
Bc_ctg2_416	B3	2.254847301	Bcer	G	Bcer	FALSE	Bc_Ga0417192_02_rn03	Ancillary biosynthesis	SMCOG1101
Bc_ctg2_417	B3	0.90193892	Bcer	NA	Bcer	FALSE	Bc_Ga0417192_02_rn03	Other	NA
Bc_ctg2_418	B3	5.975345348	Bcer	NA	Bcer	FALSE	Bc_Ga0417192_02_rn03	Other	NA
Bc_ctg2_419	B3	7.666480824	Bcer	ST	Bcer	FALSE	Bc_Ga0417192_02_rn03	Other	NA
Bc_ctg2_42	B3	0	Bcer	NA	Bcer	FALSE	NA	NA	NA
Bc_ctg2_420	B3	26.83268288	Bcer	M	Bcer	FALSE	Bc_Ga0417192_02_rn03	Other	NA
Bc_ctg2_421	B3	1.352908381	Bcer	S	Bcer	FALSE	Bc_Ga0417192_02_rn03	Other	NA
Bc_ctg2_422	B3	12.17617543	Bcer	Q	Bcer	TRUE	Bc_Ga0417192_02_rn03	Core biosynthesis	SMCOG1022

Bc_ctg2_423	B3	28.41107599	Bcer	Q	Bcer	TRUE	Bc_Ga0417192_02_rn03	Core biosynthesis	SMCOG1127
Bc_ctg2_424	B3	13.98005327	Bcer	Q	Bcer	TRUE	Bc_Ga0417192_02_rn03	Core biosynthesis	SMCOG1127
Bc_ctg2_425	B3	0.789196555	Bcer	IQ	Bcer	TRUE	Bc_Ga0417192_02_rn03	Ancillary biosynthesis	SMCOG1147
Bc_ctg2_426	B3	13.41634144	Bcer	I	Bcer	FALSE	Bc_Ga0417192_02_rn03	Ancillary biosynthesis	SMCOG1006
Bc_ctg2_427	B3	14.54376509	Bcer	J	Bcer	FALSE	Bc_Ga0417192_02_rn03	Other	NA
Bc_ctg2_428	B3	13.75456854	Bcer	I	Bcer	FALSE	Bc_Ga0417192_02_rn03	Ancillary biosynthesis	SMCOG1021
Bc_ctg2_429	B3	5.975345348	Bcer	I	Bcer	FALSE	Bc_Ga0417192_02_rn03	Ancillary biosynthesis	SMCOG1095
Bc_ctg2_430	B3	0	Bcer	IQ	Bcer	TRUE	Bc_Ga0417192_02_rn03	Ancillary biosynthesis	SMCOG1147
Bc_ctg2_431	B3	4.171467507	Bcer	I	Bcer	FALSE	Bc_Ga0417192_02_rn03	Ancillary biosynthesis	SMCOG1006
Bc_ctg2_432	B3	5.975345348	Bcer	Q	Bcer	TRUE	Bc_Ga0417192_02_rn03	Core biosynthesis	SMCOG1002
Bc_ctg2_433	B3	42.50387162	Bcer	Q	Bcer	TRUE	Bc_Ga0417192_02_rn03	Core biosynthesis	SMCOG1127
Bc_ctg2_434	B3	7.891965554	Bcer	C	Bcer	FALSE	Bc_Ga0417192_02_rn03	Ancillary biosynthesis	SMCOG1083
Bc_ctg2_435	B3	19.39168679	Bcer	V	Bcer	FALSE	Bc_Ga0417192_02_rn03	Ancillary biosynthesis	SMCOG1053
Bc_ctg2_436	B3	4.735179332	Bcer	Q	Bcer	TRUE	Bc_Ga0417192_02_rn03	Ancillary biosynthesis	SMCOG1256
Bc_ctg2_437	B3	18.60249023	Bcer	Q	Bcer	TRUE	Bc_Ga0417192_02_rn03	Core biosynthesis	SMCOG1002
Bc_ctg2_438	B3	3.269528587	Bcer	Q	Bcer	TRUE	Bc_Ga0417192_02_rn03	Ancillary biosynthesis	SMCOG1004
Bc_ctg2_439	B3	29.76398437	Bcer	Q	Bcer	TRUE	Bc_Ga0417192_02_rn03	Core biosynthesis	SMCOG1127
Bc_ctg2_440	B3	2.480332031	Bcer	H	Bcer	FALSE	Bc_Ga0417192_02_rn03	Ancillary biosynthesis	SMCOG1012
Bc_ctg2_441	B3	17.70055131	Bcer	K	Bcer	FALSE	Bc_Ga0417192_02_rn03	Regulation	SMCOG1078
Bc_ctg2_442	B3	3.833240412	Bcer	E	Bcer	FALSE	Bc_Ga0417192_02_rn03	Ancillary biosynthesis	SMCOG1056
Bc_ctg2_443	B3	4.058725142	Bcer	S	Bcer	FALSE	Bc_Ga0417192_02_rn03	Ancillary biosynthesis	SMCOG1179
Bc_ctg2_444	B3	6.313572443	Bcer	S	Bcer	FALSE	Bc_Ga0417192_02_rn03	Ancillary biosynthesis	SMCOG1079
Bc_ctg2_445	B3	0.45096946	Bcer	G	Bcer	FALSE	Bc_Ga0417192_02_rn03	Other	NA
Bc_ctg2_446	B3	2.931301491	Bcer	E	Bcer	FALSE	Bc_Ga0417192_02_rn03	Ancillary biosynthesis	SMCOG1114
Bc_ctg2_447	B3	2.254847301	Bcer	E	Bcer	FALSE	Bc_Ga0417192_02_rn03	Ancillary biosynthesis	SMCOG1081
Bc_ctg2_448	B3	6.088087713	Bcer	E	Bcer	FALSE	Bc_Ga0417192_02_rn03	Ancillary biosynthesis	SMCOG1158
Bc_ctg2_449	B3	3.833240412	Bcer	K	Bcer	FALSE	Bc_Ga0417192_02_rn03	Other	NA
Bc_ctg2_45	B3	0	Bcer	NA	Bcer	FALSE	NA	NA	NA
Bc_ctg2_450	B3	0.789196555	Bcer	NA	Bcer	FALSE	Bc_Ga0417192_02_rn03	Other	NA
Bc_ctg2_451	B3	73.62076438	Bcer	NA	Bcer	FALSE	Bc_Ga0417192_02_rn03	Other	NA
Bc_ctg2_453	B3	0.45096946	Bcer	NA	Bcer	FALSE	Bc_Ga0417192_02_rn03	Other	NA
Bc_ctg2_454	B3	0	Bcer	L	Bcer	FALSE	Bc_Ga0417192_02_rn03	Other	NA
Bc_ctg2_455	B3	0	Bcer	NA	Bcer	FALSE	Bc_Ga0417192_02_rn03	Other	NA
Bc_ctg2_456	B3	0.90193892	Bcer	NA	Bcer	FALSE	Bc_Ga0417192_02_rn03	Other	NA
Bc_ctg2_457	B3	5.298891157	Bcer	NA	Bcer	FALSE	Bc_Ga0417192_02_rn03	Other	NA
Bc_ctg2_458	B3	34.61190607	Bcer	S	Bcer	FALSE	Bc_Ga0417192_02_rn03	Other	NA
Bc_ctg2_459	B3	0.112742365	Bcer	E	Bcer	FALSE	Bc_Ga0417192_02_rn03	Other	NA
Bc_ctg2_46	B3	0	Bcer	NA	Bcer	FALSE	NA	NA	NA
Bc_ctg2_460	B3	0	Bcer	G	Bcer	FALSE	Bc_Ga0417192_02_rn03	Ancillary biosynthesis	SMCOG1101
Bc_ctg2_462	B3	0.67645419	Bcer	A	Bcer	FALSE	Bc_Ga0417192_02_rn03	Other	NA
Bc_ctg2_464	B3	0.789196555	Bcer	NA	Bcer	FALSE	NA	NA	NA
Bc_ctg2_465	B3	0.789196555	Bcer	NA	Bcer	FALSE	NA	NA	NA
Bc_ctg2_466	B3	3.156786221	Bcer	NA	Bcer	FALSE	NA	NA	NA
Bc_ctg2_467	B3	2.142104936	Bcer	NA	Bcer	FALSE	NA	NA	NA
Bc_ctg2_468	B3	0	Bcer	NA	Bcer	FALSE	NA	NA	NA
Bc_ctg2_469	B3	0	Bcer	L	Bcer	FALSE	NA	NA	NA
Bc_ctg2_47	B3	4.735179332	Bcer	V	Bcer	FALSE	NA	NA	NA
Bc_ctg2_471	B3	11.83794833	Bcer	NA	Bcer	FALSE	NA	NA	NA
Bc_ctg2_472	B3	2.705816761	Bcer	S	Bcer	FALSE	NA	NA	NA
Bc_ctg2_473	B3	38.67063121	Bcer	S	Bcer	FALSE	NA	NA	NA
Bc_ctg2_474	B3	9.132131569	Bcer	NA	Bcer	FALSE	NA	NA	NA
Bc_ctg2_475	B3	0	Bcer	L	Bcer	FALSE	NA	NA	NA
Bc_ctg2_476	B3	3.833240412	Bcer	L	Bcer	FALSE	NA	NA	NA
Bc_ctg2_477	B3	0	Bcer	NA	Bcer	FALSE	NA	NA	NA
Bc_ctg2_478	B3	0	Bcer	L	Bcer	FALSE	NA	NA	NA
Bc_ctg2_479	B3	0.45096946	Bcer	L	Bcer	FALSE	NA	NA	NA
Bc_ctg2_48	B3	6.313572443	Bcer	M	Bcer	FALSE	NA	NA	NA
Bc_ctg2_480	B3	4.171467507	Bcer	L	Bcer	FALSE	NA	NA	NA
Bc_ctg2_481	B3	0.563711825	Bcer	L	Bcer	FALSE	NA	NA	NA
Bc_ctg2_482	B3	0.563711825	Bcer	NA	Bcer	FALSE	NA	NA	NA
Bc_ctg2_483	B3	0.789196555	Bcer	NA	Bcer	FALSE	NA	NA	NA
Bc_ctg2_484	B3	2.254847301	Bcer	NA	Bcer	FALSE	NA	NA	NA

Bc_ctg2_485	B3	0.338227095	Bcer	O	Bcer	FALSE	NA	NA	NA
Bc_ctg2_486	B3	0.563711825	Bcer	K	Bcer	FALSE	NA	NA	NA
Bc_ctg2_487	B3	24.57783558	Bcer	S	Bcer	FALSE	NA	NA	NA
Bc_ctg2_488	B3	0	Bcer	NA	Bcer	FALSE	NA	NA	NA
Bc_ctg2_489	B3	0	Bcer	NA	Bcer	FALSE	NA	NA	NA
Bc_ctg2_49	B3	0.112742365	Bcer	E	Bcer	FALSE	NA	NA	NA
Bc_ctg2_490	B3	1.240166016	Bcer	S	Bcer	FALSE	NA	NA	NA
Bc_ctg2_491	B3	20.74459517	Bcer	NA	Bcer	FALSE	Bc_Ga0417192_02_rn04	Other	NA
Bc_ctg2_492	B3	3.269528587	Bcer	NA	Bcer	FALSE	Bc_Ga0417192_02_rn04	Other	NA
Bc_ctg2_493	B3	1.352908381	Bcer	NA	Bcer	FALSE	Bc_Ga0417192_02_rn04	Other	NA
Bc_ctg2_494	B3	6.426314808	Bcer	NA	Bcer	FALSE	Bc_Ga0417192_02_rn04	Other	NA
Bc_ctg2_495	B3	50.05761008	Bcer	L	Bcer	FALSE	Bc_Ga0417192_02_rn04	Other	NA
Bc_ctg2_496	B3	90.30663441	Bcer	L	Bcer	FALSE	Bc_Ga0417192_02_rn04	Other	NA
Bc_ctg2_497	B3	65.84154119	Bcer	K	Bcer	FALSE	Bc_Ga0417192_02_rn04	Other	NA
Bc_ctg2_498	B3	24.23960849	Bcer	EGP	Bcer	FALSE	Bc_Ga0417192_02_rn04	Transport	SMCOG1020
Bc_ctg2_499	B3	27.50913707	Bcer	KLT	Bcer	FALSE	Bc_Ga0417192_02_rn04	Core biosynthesis	SMCOG1070
Bc_ctg2_5	B3	1.014681285	Bcer	E	Bcer	FALSE	Bc_Ga0417192_02_rn01	Core biosynthesis	SMCOG1056
Bc_ctg2_50	B3	0	Bcer	NA	Bcer	FALSE	NA	NA	NA
Bc_ctg2_500	B3	2.818559126	Bcer	J	Bcer	FALSE	Bc_Ga0417192_02_rn04	Other	NA
Bc_ctg2_504	B3	0.45096946	Bcer	NA	Bcer	FALSE	Bc_Ga0417192_02_rn04	Other	NA
Bc_ctg2_505	B3	76.21383878	Bcer	S	Bcer	FALSE	Bc_Ga0417192_02_rn04	Other	NA
Bc_ctg2_506	B3	25.59251687	Bcer	L	Bcer	FALSE	Bc_Ga0417192_02_rn04	Other	SMCOG1077
Bc_ctg2_507	B3	1.691135476	Bcer	K	Bcer	FALSE	Bc_Ga0417192_02_rn04	Regulation	SMCOG1058
Bc_ctg2_508	B3	18.7152326	Bcer	L	Bcer	FALSE	Bc_Ga0417192_02_rn04	Other	NA
Bc_ctg2_509	B3	2.029362571	Bcer	NA	Bcer	FALSE	Bc_Ga0417192_02_rn04	Other	NA
Bc_ctg2_51	B3	2.818559126	Bcer	L	Bcer	FALSE	NA	NA	NA
Bc_ctg2_510	B3	4.396952237	Bcer	S	Bcer	FALSE	Bc_Ga0417192_02_rn04	Other	NA
Bc_ctg2_511	B3	0.45096946	Bcer	NA	Bcer	FALSE	NA	NA	NA
Bc_ctg2_512	B3	2.254847301	Bcer	NA	Bcer	FALSE	NA	NA	NA
Bc_ctg2_513	B3	1.578393111	Bcer	M	Bcer	FALSE	NA	NA	NA
Bc_ctg2_52	B3	0.90193892	Bcer	M	Bcer	FALSE	NA	NA	NA
Bc_ctg2_53	B3	10.03407049	Bcer	M	Bcer	FALSE	NA	NA	NA
Bc_ctg2_54	B3	0.789196555	Bcer	M	Bcer	FALSE	NA	NA	NA
Bc_ctg2_55	B3	1.465650746	Bcer	NA	Bcer	FALSE	NA	NA	NA
Bc_ctg2_56	B3	0.789196555	Bcer	V	Bcer	FALSE	NA	NA	NA
Bc_ctg2_57	B3	6.200830078	Bcer	CH	Bcer	FALSE	NA	NA	NA
Bc_ctg2_58	B3	5.524375888	Bcer	E	Bcer	FALSE	NA	NA	NA
Bc_ctg2_59	B3	2.593074396	Bcer	S	Bcer	FALSE	NA	NA	NA
Bc_ctg2_6	B3	0	Bcer	M	Bcer	FALSE	Bc_Ga0417192_02_rn01	Other	NA
Bc_ctg2_60	B3	5.186148792	Bcer	K	Bcer	FALSE	NA	NA	NA
Bc_ctg2_61	B3	14.09279563	Bcer	D	Bcer	FALSE	NA	NA	NA
Bc_ctg2_62	B3	1.127423651	Bcer	NA	Bcer	FALSE	NA	NA	NA
Bc_ctg2_63	B3	2.818559126	Bcer	K	Bcer	FALSE	NA	NA	NA
Bc_ctg2_64	B3	6.426314808	Bcer	E	Bcer	FALSE	NA	NA	NA
Bc_ctg2_65	B3	4.396952237	Bcer	L	Bcer	FALSE	NA	NA	NA
Bc_ctg2_66	B3	28.41107599	Bcer	NA	Bcer	FALSE	NA	NA	NA
Bc_ctg2_67	B3	0.45096946	Bcer	NA	Bcer	FALSE	NA	NA	NA
Bc_ctg2_68	B3	0	Bcer	NA	Bcer	FALSE	NA	NA	NA
Bc_ctg2_69	B3	0	Bcer	NA	Bcer	FALSE	NA	NA	NA
Bc_ctg2_7	B3	0.789196555	Bcer	M	Bcer	FALSE	Bc_Ga0417192_02_rn01	Core biosynthesis	None
Bc_ctg2_70	B3	0	Bcer	M	Bcer	FALSE	NA	NA	NA
Bc_ctg2_71	B3	1.240166016	Bcer	NA	Bcer	FALSE	NA	NA	NA
Bc_ctg2_72	B3	10.71052468	Bcer	NA	Bcer	FALSE	NA	NA	NA
Bc_ctg2_73	B3	27.62187944	Bcer	S	Bcer	FALSE	NA	NA	NA
Bc_ctg2_74	B3	40.24902432	Bcer	S	Bcer	FALSE	NA	NA	NA
Bc_ctg2_75	B3	1.803877841	Bcer	NA	Bcer	FALSE	NA	NA	NA
Bc_ctg2_76	B3	3.269528587	Bcer	NA	Bcer	FALSE	NA	NA	NA
Bc_ctg2_77	B3	14.76924982	Bcer	KT	Bcer	FALSE	NA	NA	NA
Bc_ctg2_78	B3	2.818559126	Bcer	K	Bcer	FALSE	NA	NA	NA
Bc_ctg2_79	B3	1.916620206	Bcer	NA	Bcer	FALSE	NA	NA	NA
Bc_ctg2_8	B3	0.338227095	Bcer	J	Bcer	FALSE	Bc_Ga0417192_02_rn01	Other	NA
Bc_ctg2_80	B3	9.583101029	Bcer	K	Bcer	FALSE	NA	NA	NA

Bc_ctg2_81	B3	36.64126864	Bcer	M	Bcer	FALSE	NA	NA	NA
Bc_ctg2_82	B3	63.13572443	Bcer	S	Bcer	FALSE	NA	NA	NA
Bc_ctg2_83	B3	1.127423651	Bcer	K	Bcer	FALSE	NA	NA	NA
Bc_ctg2_84	B3	1.240166016	Bcer	E	Bcer	FALSE	NA	NA	NA
Bc_ctg2_86	B3	6.313572443	Bcer	S	Bcer	FALSE	NA	NA	NA
Bc_ctg2_87	B3	0.67645419	Bcer	K	Bcer	FALSE	NA	NA	NA
Bc_ctg2_88	B3	193.014929	Bcer	M	Bcer	FALSE	NA	NA	NA
Bc_ctg2_89	B3	6.990026633	Bcer	S	Bcer	FALSE	NA	NA	NA
Bc_ctg2_9	B3	3.044043856	Bcer	M	Bcer	FALSE	Bc_Ga0417192_02_rn01	Other	NA
Bc_ctg2_90	B3	5.637118253	Bcer	NA	Bcer	FALSE	NA	NA	NA
Bc_ctg2_91	B3	9.244873934	Bcer	V	Bcer	FALSE	NA	NA	NA
Bc_ctg2_93	B3	1.352908381	Bcer	I	Bcer	FALSE	NA	NA	NA
Bc_ctg2_94	B3	1.465650746	Bcer	NA	Bcer	FALSE	NA	NA	NA
Bc_ctg2_95	B3	1032.945549	Bcer	S	Bcer	FALSE	NA	NA	NA
Bc_ctg2_96	B3	6.764541903	Bcer	IQ	Bcer	TRUE	NA	NA	NA
Bc_ctg2_98	B3	0.112742365	Bcer	NA	Bcer	FALSE	Bc_Ga0417192_02_rn02	Other	NA
Bc_ctg2_99	B3	2.480332031	Bcer	EG	Bcer	FALSE	Bc_Ga0417192_02_rn02	Transport	SMCOG1166
Bc_ctg3_1	B3	15.89667347	Bcer	P	Bcer	FALSE	NA	NA	NA
Bc_ctg3_10	B3	20.06814098	Bcer	EGP	Bcer	FALSE	Bc_Ga0417192_03_rn01	Transport	SMCOG1106
Bc_ctg3_100	B3	2.029362571	Bcer	V	Bcer	FALSE	NA	NA	NA
Bc_ctg3_101	B3	0.112742365	Bcer	S	Bcer	FALSE	NA	NA	NA
Bc_ctg3_102	B3	0.563711825	Bcer	K	Bcer	FALSE	NA	NA	NA
Bc_ctg3_103	B3	4.960664062	Bcer	K	Bcer	FALSE	NA	NA	NA
Bc_ctg3_104	B3	0.45096946	Bcer	L	Bcer	FALSE	NA	NA	NA
Bc_ctg3_105	B3	0.563711825	Bcer	L	Bcer	FALSE	NA	NA	NA
Bc_ctg3_106	B3	0.789196555	Bcer	S	Bcer	FALSE	NA	NA	NA
Bc_ctg3_107	B3	8.455677379	Bcer	V	Bcer	FALSE	NA	NA	NA
Bc_ctg3_108	B3	2.142104936	Bcer	V	Bcer	FALSE	NA	NA	NA
Bc_ctg3_109	B3	1.014681285	Bcer	V	Bcer	FALSE	NA	NA	NA
Bc_ctg3_11	B3	1.352908381	Bcer	NA	Bcer	FALSE	Bc_Ga0417192_03_rn01	Other	NA
Bc_ctg3_110	B3	6.764541903	Bcer	S	Bcer	FALSE	NA	NA	NA
Bc_ctg3_111	B3	2.818559126	Bcer	K	Bcer	FALSE	NA	NA	NA
Bc_ctg3_112	B3	178.0201944	Bcer	M	Bcer	FALSE	NA	NA	NA
Bc_ctg3_113	B3	13.30359908	Bcer	M	Bcer	FALSE	NA	NA	NA
Bc_ctg3_114	B3	12.73988725	Bcer	S	Bcer	FALSE	NA	NA	NA
Bc_ctg3_115	B3	681.8658238	Bcer	G	Bcer	FALSE	NA	NA	NA
Bc_ctg3_116	B3	224.6955335	Bcer	G	Bcer	FALSE	NA	NA	NA
Bc_ctg3_117	B3	622.1123704	Bcer	G	Bcer	FALSE	NA	NA	NA
Bc_ctg3_118	B3	21.53379172	Bcer	U	Bcer	FALSE	NA	NA	NA
Bc_ctg3_119	B3	1.465650746	Bcer	G	Bcer	FALSE	NA	NA	NA
Bc_ctg3_12	B3	2.367589666	Bcer	Q	Bcer	TRUE	Bc_Ga0417192_03_rn01	Ancillary biosynthesis	SMCOG1027
Bc_ctg3_120	B3	15.55844638	Bcer	S	Bcer	FALSE	NA	NA	NA
Bc_ctg3_121	B3	17.58780895	Bcer	G	Bcer	FALSE	NA	NA	NA
Bc_ctg3_122	B3	1436.224988	Bcer	C	Bcer	FALSE	NA	NA	NA
Bc_ctg3_123	B3	154.3442978	Bcer	G	Bcer	FALSE	NA	NA	NA
Bc_ctg3_124	B3	5.524375888	Bcer	NA	Bcer	FALSE	NA	NA	NA
Bc_ctg3_126	B3	5.298891157	Bcer	S	Bcer	FALSE	NA	NA	NA
Bc_ctg3_128	B3	4.622436967	Bcer	K	Bcer	FALSE	NA	NA	NA
Bc_ctg3_129	B3	4.960664062	Bcer	V	Bcer	FALSE	NA	NA	NA
Bc_ctg3_13	B3	3.156786221	Bcer	G	Bcer	FALSE	Bc_Ga0417192_03_rn01	Ancillary biosynthesis	SMCOG1235
Bc_ctg3_130	B3	3.720498047	Bcer	S	Bcer	FALSE	NA	NA	NA
Bc_ctg3_131	B3	11.16149414	Bcer	NA	Bcer	FALSE	NA	NA	NA
Bc_ctg3_132	B3	14.99473455	Bcer	U	Bcer	FALSE	NA	NA	NA
Bc_ctg3_133	B3	15.44570401	Bcer	NA	Bcer	FALSE	NA	NA	NA
Bc_ctg3_134	B3	32.01883167	Bcer	L	Bcer	FALSE	NA	NA	NA
Bc_ctg3_135	B3	1.916620206	Bcer	V	Bcer	FALSE	NA	NA	NA
Bc_ctg3_136	B3	0.789196555	Bcer	V	Bcer	FALSE	NA	NA	NA
Bc_ctg3_137	B3	0.90193892	Bcer	O	Bcer	FALSE	NA	NA	NA
Bc_ctg3_139	B3	8.004707919	Bcer	T	Bcer	FALSE	NA	NA	NA
Bc_ctg3_14	B3	14.205538	Bcer	EGP	Bcer	FALSE	Bc_Ga0417192_03_rn01	Transport	SMCOG1020
Bc_ctg3_140	B3	26.71994052	Bcer	T	Bcer	FALSE	NA	NA	NA
Bc_ctg3_141	B3	0	Bcer	NA	Bcer	FALSE	NA	NA	NA

Bc_ctg3_142	B3	23.78863903	Bcer	S	Bcer	FALSE	NA	NA	NA
Bc_ctg3_143	B3	110.3747754	Bcer	S	Bcer	FALSE	NA	NA	NA
Bc_ctg3_144	B3	60.6553924	Bcer	G	Bcer	FALSE	NA	NA	NA
Bc_ctg3_145	B3	13.8673109	Bcer	S	Bcer	FALSE	NA	NA	NA
Bc_ctg3_146	B3	80.15982155	Bcer	Q	Bcer	TRUE	NA	NA	NA
Bc_ctg3_147	B3	59.30248402	Bcer	H	Bcer	FALSE	NA	NA	NA
Bc_ctg3_148	B3	156.260918	Bcer	F	Bcer	FALSE	Bc_Ga0417192_03_rn02	Other	NA
Bc_ctg3_149	B3	238.5628444	Bcer	J	Bcer	FALSE	Bc_Ga0417192_03_rn02	Other	NA
Bc_ctg3_15	B3	0	Bcer	L	Bcer	FALSE	Bc_Ga0417192_03_rn01	Other	NA
Bc_ctg3_150	B3	29.31301491	Bcer	NA	Bcer	FALSE	Bc_Ga0417192_03_rn02	Ancillary biosynthesis	None
Bc_ctg3_151	B3	222.1024592	Bcer	NA	Bcer	FALSE	Bc_Ga0417192_03_rn02	Other	NA
Bc_ctg3_152	B3	29.42575728	Bcer	NA	Bcer	FALSE	Bc_Ga0417192_03_rn02	Other	NA
Bc_ctg3_153	B3	121.1980424	Bcer	T	Bcer	FALSE	Bc_Ga0417192_03_rn02	Other	SMCOG1226
Bc_ctg3_154	B3	91.09583096	Bcer	NA	Bcer	FALSE	Bc_Ga0417192_03_rn02	Other	NA
Bc_ctg3_155	B3	151.4129963	Bcer	EGP	Bcer	FALSE	Bc_Ga0417192_03_rn02	Transport	SMCOG1020
Bc_ctg3_156	B3	5.637118253	Bcer	NA	Bcer	FALSE	Bc_Ga0417192_03_rn02	Other	NA
Bc_ctg3_158	B3	13.07811435	Bcer	S	Bcer	FALSE	Bc_Ga0417192_03_rn02	Other	NA
Bc_ctg3_159	B3	49.71938299	Bcer	C	Bcer	FALSE	Bc_Ga0417192_03_rn02	Ancillary biosynthesis	None
Bc_ctg3_16	B3	0.338227095	Bcer	NA	Bcer	FALSE	Bc_Ga0417192_03_rn01	Other	NA
Bc_ctg3_160	B3	0	Bcer	S	Bcer	FALSE	Bc_Ga0417192_03_rn02	Core biosynthesis	None
Bc_ctg3_161	B3	36.52852628	Bcer	OS	Bcer	FALSE	Bc_Ga0417192_03_rn02	Ancillary biosynthesis	None
Bc_ctg3_162	B3	14.65650746	Bcer	O	Bcer	FALSE	Bc_Ga0417192_03_rn02	Other	NA
Bc_ctg3_163	B3	22.66121538	Bcer	E	Bcer	FALSE	Bc_Ga0417192_03_rn02	Ancillary biosynthesis	SMCOG1038
Bc_ctg3_164	B3	358.7462056	Bcer	E	Bcer	FALSE	Bc_Ga0417192_03_rn02	Ancillary biosynthesis	SMCOG1081
Bc_ctg3_165	B3	351.4179519	Bcer	E	Bcer	FALSE	Bc_Ga0417192_03_rn02	Other	NA
Bc_ctg3_166	B3	9.357616299	Bcer	E	Bcer	FALSE	NA	NA	NA
Bc_ctg3_167	B3	5.411633522	Bcer	S	Bcer	FALSE	NA	NA	NA
Bc_ctg3_168	B3	6.313572443	Bcer	C	Bcer	FALSE	NA	NA	NA
Bc_ctg3_169	B3	1.465650746	Bcer	P	Bcer	FALSE	NA	NA	NA
Bc_ctg3_17	B3	0	Bcer	K	Bcer	FALSE	Bc_Ga0417192_03_rn01	Other	NA
Bc_ctg3_170	B3	49.49389826	Bcer	C	Bcer	FALSE	NA	NA	NA
Bc_ctg3_171	B3	1.691135476	Bcer	L	Bcer	FALSE	NA	NA	NA
Bc_ctg3_172	B3	12.17617543	Bcer	L	Bcer	FALSE	NA	NA	NA
Bc_ctg3_173	B3	2.480332031	Bcer	NU	Bcer	FALSE	NA	NA	NA
Bc_ctg3_174	B3	31.45511985	Bcer	L	Bcer	FALSE	NA	NA	NA
Bc_ctg3_175	B3	35.51384499	Bcer	L	Bcer	FALSE	NA	NA	NA
Bc_ctg3_176	B3	23.45041193	Bcer	S	Bcer	FALSE	NA	NA	NA
Bc_ctg3_177	B3	14.54376509	Bcer	F	Bcer	FALSE	NA	NA	NA
Bc_ctg3_178	B3	7.891965554	Bcer	O	Bcer	FALSE	NA	NA	NA
Bc_ctg3_179	B3	10.59778231	Bcer	C	Bcer	FALSE	NA	NA	NA
Bc_ctg3_18	B3	3.607755682	Bcer	L	Bcer	FALSE	Bc_Ga0417192_03_rn01	Other	NA
Bc_ctg3_180	B3	0.67645419	Bcer	NA	Bcer	FALSE	NA	NA	NA
Bc_ctg3_181	B3	2704.463853	Bcer	C	Bcer	FALSE	NA	NA	NA
Bc_ctg3_182	B3	23.2249272	Bcer	NA	Bcer	FALSE	NA	NA	NA
Bc_ctg3_183	B3	22.43573065	Bcer	NA	Bcer	FALSE	NA	NA	NA
Bc_ctg3_184	B3	94.25261718	Bcer	K	Bcer	FALSE	NA	NA	NA
Bc_ctg3_185	B3	33.48448242	Bcer	NA	Bcer	FALSE	NA	NA	NA
Bc_ctg3_186	B3	289.4096511	Bcer	NA	Bcer	FALSE	NA	NA	NA
Bc_ctg3_187	B3	32.5825435	Bcer	K	Bcer	FALSE	NA	NA	NA
Bc_ctg3_188	B3	9.80858576	Bcer	NA	Bcer	FALSE	NA	NA	NA
Bc_ctg3_189	B3	0.90193892	Bcer	K	Bcer	FALSE	NA	NA	NA
Bc_ctg3_19	B3	0	Bcer	NA	Bcer	FALSE	Bc_Ga0417192_03_rn01	Other	NA
Bc_ctg3_190	B3	3.607755682	Bcer	NA	Bcer	FALSE	NA	NA	NA
Bc_ctg3_191	B3	9.357616299	Bcer	S	Bcer	FALSE	NA	NA	NA
Bc_ctg3_192	B3	0.789196555	Bcer	NA	Bcer	FALSE	NA	NA	NA
Bc_ctg3_193	B3	2.142104936	Bcer	NA	Bcer	FALSE	NA	NA	NA
Bc_ctg3_194	B3	6.764541903	Bcer	NA	Bcer	FALSE	NA	NA	NA
Bc_ctg3_195	B3	149.158149	Bcer	T	Bcer	FALSE	NA	NA	NA
Bc_ctg3_196	B3	80.15982155	Bcer	L	Bcer	FALSE	NA	NA	NA
Bc_ctg3_197	B3	17.47506658	Bcer	NA	Bcer	FALSE	NA	NA	NA
Bc_ctg3_198	B3	3.382270952	Bcer	S	Bcer	FALSE	NA	NA	NA
Bc_ctg3_199	B3	2.593074396	Bcer	K	Bcer	FALSE	NA	NA	NA

Bc_ctg3_2	B3	96.39472212	Bcer	P	Bcer	FALSE	NA	NA	NA
Bc_ctg3_20	B3	0	Bcer	NA	Bcer	FALSE	Bc_Ga0417192_03_rn01	Other	NA
Bc_ctg3_200	B3	12.28891779	Bcer	NA	Bcer	FALSE	NA	NA	NA
Bc_ctg3_201	B3	21.75927646	Bcer	NA	Bcer	FALSE	NA	NA	NA
Bc_ctg3_202	B3	80.38530628	Bcer	P	Bcer	FALSE	NA	NA	NA
Bc_ctg3_203	B3	16.4603853	Bcer	NA	Bcer	FALSE	NA	NA	NA
Bc_ctg3_204	B3	5.524375888	Bcer	K	Bcer	FALSE	NA	NA	NA
Bc_ctg3_206	B3	0	Bcer	NA	Bcer	FALSE	NA	NA	NA
Bc_ctg3_207	B3	4.622436967	Bcer	Q	Bcer	TRUE	NA	NA	NA
Bc_ctg3_208	B3	29.76398437	Bcer	S	Bcer	FALSE	NA	NA	NA
Bc_ctg3_209	B3	2.593074396	Bcer	NA	Bcer	FALSE	NA	NA	NA
Bc_ctg3_21	B3	1.916620206	Bcer	M	Bcer	FALSE	Bc_Ga0417192_03_rn01	Core biosynthesis	SMCOG1010
Bc_ctg3_212	B3	6.426314808	Bcer	S	Bcer	FALSE	NA	NA	NA
Bc_ctg3_213	B3	2.367589666	Bcer	NA	Bcer	FALSE	NA	NA	NA
Bc_ctg3_214	B3	4.622436967	Bcer	E	Bcer	FALSE	NA	NA	NA
Bc_ctg3_215	B3	7.553738458	Bcer	E	Bcer	FALSE	NA	NA	NA
Bc_ctg3_216	B3	0	Bcer	L	Bcer	FALSE	NA	NA	NA
Bc_ctg3_217	B3	2.593074396	Bcer	K	Bcer	FALSE	NA	NA	NA
Bc_ctg3_218	B3	1.691135476	Bcer	S	Bcer	FALSE	NA	NA	NA
Bc_ctg3_219	B3	0.338227095	Bcer	S	Bcer	FALSE	NA	NA	NA
Bc_ctg3_22	B3	5.862602983	Bcer	M	Bcer	FALSE	Bc_Ga0417192_03_rn01	Core biosynthesis	SMCOG1010
Bc_ctg3_220	B3	3.156786221	Bcer	M	Bcer	FALSE	NA	NA	NA
Bc_ctg3_221	B3	0	Bcer	L	Bcer	FALSE	NA	NA	NA
Bc_ctg3_222	B3	0.338227095	Bcer	L	Bcer	FALSE	NA	NA	NA
Bc_ctg3_223	B3	8.455677379	Bcer	NA	Bcer	FALSE	NA	NA	NA
Bc_ctg3_224	B3	14.88199219	Bcer	C	Bcer	FALSE	NA	NA	NA
Bc_ctg3_225	B3	11.83794833	Bcer	K	Bcer	FALSE	NA	NA	NA
Bc_ctg3_226	B3	83.99306196	Bcer	GKT	Bcer	FALSE	NA	NA	NA
Bc_ctg3_227	B3	1166.432509	Bcer	G	Bcer	FALSE	NA	NA	NA
Bc_ctg3_228	B3	603.6226225	Bcer	G	Bcer	FALSE	NA	NA	NA
Bc_ctg3_229	B3	0.112742365	Bcer	NA	Bcer	FALSE	NA	NA	NA
Bc_ctg3_23	B3	1.352908381	Bcer	M	Bcer	FALSE	Bc_Ga0417192_03_rn01	Core biosynthesis	None
Bc_ctg3_230	B3	23.5631543	Bcer	P	Bcer	FALSE	NA	NA	NA
Bc_ctg3_24	B3	0.90193892	Bcer	M	Bcer	FALSE	Bc_Ga0417192_03_rn01	Ancillary biosynthesis	SMCOG1064
Bc_ctg3_25	B3	8.342935014	Bcer	I	Bcer	FALSE	Bc_Ga0417192_03_rn01	Core biosynthesis	SMCOG1022
Bc_ctg3_26	B3	3.044043856	Bcer	E	Bcer	FALSE	Bc_Ga0417192_03_rn01	Other	NA
Bc_ctg3_27	B3	0.789196555	Bcer	NA	Bcer	FALSE	Bc_Ga0417192_03_rn01	Other	NA
Bc_ctg3_28	B3	5.186148792	Bcer	K	Bcer	FALSE	Bc_Ga0417192_03_rn01	Other	NA
Bc_ctg3_29	B3	0.90193892	Bcer	I	Bcer	FALSE	Bc_Ga0417192_03_rn01	Ancillary biosynthesis	SMCOG1270
Bc_ctg3_3	B3	29.87672674	Bcer	E	Bcer	FALSE	NA	NA	NA
Bc_ctg3_30	B3	0.22548473	Bcer	IQ	Bcer	TRUE	Bc_Ga0417192_03_rn01	Ancillary biosynthesis	None
Bc_ctg3_31	B3	0.563711825	Bcer	IQ	Bcer	TRUE	Bc_Ga0417192_03_rn01	Ancillary biosynthesis	SMCOG1001
Bc_ctg3_32	B3	2.480332031	Bcer	I	Bcer	FALSE	Bc_Ga0417192_03_rn01	Ancillary biosynthesis	SMCOG1021
Bc_ctg3_33	B3	0.90193892	Bcer	S	Bcer	FALSE	Bc_Ga0417192_03_rn01	Other	NA
Bc_ctg3_34	B3	2.254847301	Bcer	V	Bcer	FALSE	Bc_Ga0417192_03_rn01	Transport	SMCOG1065
Bc_ctg3_35	B3	0.67645419	Bcer	V	Bcer	FALSE	Bc_Ga0417192_03_rn01	Transport	SMCOG1000
Bc_ctg3_36	B3	1.127423651	Bcer	S	Bcer	FALSE	Bc_Ga0417192_03_rn01	Other	NA
Bc_ctg3_37	B3	33.70996715	Bcer	S	Bcer	FALSE	Bc_Ga0417192_03_rn01	Other	NA
Bc_ctg3_38	B3	10.14681285	Bcer	NA	Bcer	FALSE	Bc_Ga0417192_03_rn01	Other	NA
Bc_ctg3_39	B3	6.764541903	Bcer	L	Bcer	FALSE	Bc_Ga0417192_03_rn01	Other	SMCOG1077
Bc_ctg3_4	B3	32.35705877	Bcer	H	Bcer	FALSE	NA	NA	NA
Bc_ctg3_40	B3	0	Bcer	NA	Bcer	FALSE	Bc_Ga0417192_03_rn01	Other	NA
Bc_ctg3_41	B3	9.583101029	Bcer	E	Bcer	FALSE	Bc_Ga0417192_03_rn01	Other	NA
Bc_ctg3_42	B3	8.004707919	Bcer	E	Bcer	FALSE	Bc_Ga0417192_03_rn01	Ancillary biosynthesis	SMCOG1153
Bc_ctg3_43	B3	7.102768998	Bcer	E	Bcer	FALSE	Bc_Ga0417192_03_rn01	Other	NA
Bc_ctg3_44	B3	0.67645419	Bcer	P	Bcer	FALSE	NA	NA	NA
Bc_ctg3_45	B3	0.22548473	Bcer	E	Bcer	FALSE	NA	NA	NA
Bc_ctg3_46	B3	2.367589666	Bcer	S	Bcer	FALSE	NA	NA	NA
Bc_ctg3_47	B3	0.112742365	Bcer	NA	Bcer	FALSE	NA	NA	NA
Bc_ctg3_48	B3	0	Bcer	NA	Bcer	FALSE	NA	NA	NA
Bc_ctg3_49	B3	2.254847301	Bcer	NA	Bcer	FALSE	NA	NA	NA
Bc_ctg3_5	B3	9.019389204	Bcer	K	Bcer	FALSE	NA	NA	NA

Bc_ctg3_50	B3	2.254847301	Bcer	V	Bcer	FALSE	NA	NA	NA
Bc_ctg3_51	B3	4.735179332	Bcer	V	Bcer	FALSE	NA	NA	NA
Bc_ctg3_52	B3	26.15622869	Bcer	K	Bcer	FALSE	NA	NA	NA
Bc_ctg3_53	B3	18.3770055	Bcer	S	Bcer	FALSE	NA	NA	NA
Bc_ctg3_54	B3	0	Bcer	NA	Bcer	FALSE	NA	NA	NA
Bc_ctg3_55	B3	0.338227095	Bcer	NA	Bcer	FALSE	NA	NA	NA
Bc_ctg3_56	B3	11.16149414	Bcer	NA	Bcer	FALSE	NA	NA	NA
Bc_ctg3_57	B3	1.352908381	Bcer	S	Bcer	FALSE	NA	NA	NA
Bc_ctg3_58	B3	2.818559126	Bcer	NA	Bcer	FALSE	NA	NA	NA
Bc_ctg3_59	B3	0	Bcer	NA	Bcer	FALSE	NA	NA	NA
Bc_ctg3_6	B3	1.578393111	Bcer	NA	Bcer	FALSE	NA	NA	NA
Bc_ctg3_60	B3	147.4670135	Bcer	K	Bcer	FALSE	NA	NA	NA
Bc_ctg3_61	B3	4.847921697	Bcer	S	Bcer	FALSE	NA	NA	NA
Bc_ctg3_62	B3	4.847921697	Bcer	NA	Bcer	FALSE	NA	NA	NA
Bc_ctg3_63	B3	0.90193892	Bcer	L	Bcer	FALSE	NA	NA	NA
Bc_ctg3_64	B3	22.99944247	Bcer	K	Bcer	FALSE	NA	NA	NA
Bc_ctg3_65	B3	5.975345348	Bcer	E	Bcer	FALSE	NA	NA	NA
Bc_ctg3_66	B3	2.480332031	Bcer	I	Bcer	FALSE	NA	NA	NA
Bc_ctg3_67	B3	0.67645419	Bcer	S	Bcer	FALSE	NA	NA	NA
Bc_ctg3_68	B3	1.578393111	Bcer	H	Bcer	FALSE	NA	NA	NA
Bc_ctg3_69	B3	1.014681285	Bcer	H	Bcer	FALSE	NA	NA	NA
Bc_ctg3_7	B3	18.3770055	Bcer	QT	Bcer	TRUE	Bc_Ga0417192_03_rn01	Other	NA
Bc_ctg3_70	B3	0	Bcer	L	Bcer	FALSE	NA	NA	NA
Bc_ctg3_71	B3	169.1135476	Bcer	I	Bcer	FALSE	NA	NA	NA
Bc_ctg3_72	B3	4.396952237	Bcer	L	Bcer	FALSE	NA	NA	NA
Bc_ctg3_73	B3	42.61661399	Bcer	K	Bcer	FALSE	NA	NA	NA
Bc_ctg3_74	B3	133.4869602	Bcer	S	Bcer	FALSE	NA	NA	NA
Bc_ctg3_75	B3	96.84569158	Bcer	K	Bcer	FALSE	NA	NA	NA
Bc_ctg3_76	B3	0	Bcer	L	Bcer	FALSE	NA	NA	NA
Bc_ctg3_77	B3	0	Bcer	L	Bcer	FALSE	NA	NA	NA
Bc_ctg3_78	B3	22.66121538	Bcer	D	Bcer	FALSE	NA	NA	NA
Bc_ctg3_79	B3	4.735179332	Bcer	M	Bcer	FALSE	NA	NA	NA
Bc_ctg3_8	B3	9.583101029	Bcer	EGP	Bcer	FALSE	Bc_Ga0417192_03_rn01	Transport	SMCOG1106
Bc_ctg3_80	B3	14.31828036	Bcer	I	Bcer	FALSE	NA	NA	NA
Bc_ctg3_81	B3	80.27256392	Bcer	M	Bcer	FALSE	NA	NA	NA
Bc_ctg3_82	B3	0.563711825	Bcer	NA	Bcer	FALSE	NA	NA	NA
Bc_ctg3_83	B3	1.240166016	Bcer	K	Bcer	FALSE	NA	NA	NA
Bc_ctg3_84	B3	26.04348633	Bcer	O	Bcer	FALSE	NA	NA	NA
Bc_ctg3_85	B3	0.90193892	Bcer	O	Bcer	FALSE	NA	NA	NA
Bc_ctg3_86	B3	0.112742365	Bcer	NA	Bcer	FALSE	NA	NA	NA
Bc_ctg3_87	B3	1.691135476	Bcer	NA	Bcer	FALSE	NA	NA	NA
Bc_ctg3_88	B3	8.342935014	Bcer	P	Bcer	FALSE	NA	NA	NA
Bc_ctg3_89	B3	3.945982777	Bcer	NA	Bcer	FALSE	NA	NA	NA
Bc_ctg3_9	B3	7.666480824	Bcer	E	Bcer	FALSE	Bc_Ga0417192_03_rn01	Ancillary biosynthesis	SMCOG1244
Bc_ctg3_90	B3	0.22548473	Bcer	NA	Bcer	FALSE	NA	NA	NA
Bc_ctg3_91	B3	141.3789258	Bcer	C	Bcer	FALSE	NA	NA	NA
Bc_ctg3_92	B3	26.60719815	Bcer	NA	Bcer	FALSE	NA	NA	NA
Bc_ctg3_93	B3	5.298891157	Bcer	NA	Bcer	FALSE	NA	NA	NA
Bc_ctg3_94	B3	1.578393111	Bcer	S	Bcer	FALSE	NA	NA	NA
Bc_ctg3_95	B3	7.666480824	Bcer	NA	Bcer	FALSE	NA	NA	NA
Bc_ctg3_96	B3	32.2443164	Bcer	I	Bcer	FALSE	NA	NA	NA
Bc_ctg3_97	B3	26.15622869	Bcer	T	Bcer	FALSE	NA	NA	NA
Bc_ctg3_98	B3	4.509694602	Bcer	T	Bcer	FALSE	NA	NA	NA
Bc_ctg3_99	B3	11.04875178	Bcer	V	Bcer	FALSE	NA	NA	NA
Bc_ctg1_1	B4	26.11305295	Bcer	E	Bcer	FALSE	NA	NA	NA
Bc_ctg1_10	B4	34.81740394	Bcer	K	Bcer	FALSE	NA	NA	NA
Bc_ctg1_100	B4	15.62319407	Bcer	S	Bcer	FALSE	NA	NA	NA
Bc_ctg1_1002	B4	15.84638256	Bcer	S	Bcer	FALSE	Bc_Ga0417192_01_rn09	Other	NA
Bc_ctg1_1003	B4	13.83768618	Bcer	S	Bcer	FALSE	Bc_Ga0417192_01_rn09	Other	NA
Bc_ctg1_1004	B4	54.12320804	Bcer	NA	Bcer	FALSE	Bc_Ga0417192_01_rn09	Other	NA
Bc_ctg1_1005	B4	6.360871873	Bcer	S	Bcer	FALSE	Bc_Ga0417192_01_rn09	Other	NA
Bc_ctg1_1006	B4	39.72755065	Bcer	S	Bcer	FALSE	Bc_Ga0417192_01_rn09	Other	NA

Bc_ctg1_1007	B4	404.1943496	Bcer	I	Bcer	FALSE	Bc_Ga0417192_01_rn09	Ancillary biosynthesis	SMCOG1124
Bc_ctg1_1008	B4	74.65654883	Bcer	F	Bcer	FALSE	Bc_Ga0417192_01_rn09	Other	NA
Bc_ctg1_1009	B4	181.8986167	Bcer	S	Bcer	FALSE	Bc_Ga0417192_01_rn09	Other	NA
Bc_ctg1_101	B4	7.476814307	Bcer	S	Bcer	FALSE	NA	NA	NA
Bc_ctg1_1010	B4	0	Bcer	NA	Bcer	FALSE	NA	NA	NA
Bc_ctg1_1011	B4	9.039133715	Bcer	NA	Bcer	FALSE	NA	NA	NA
Bc_ctg1_1012	B4	11.71739556	Bcer	NA	Bcer	FALSE	NA	NA	NA
Bc_ctg1_1013	B4	1.227536677	Bcer	K	Bcer	FALSE	NA	NA	NA
Bc_ctg1_1014	B4	5.691306413	Bcer	IQ	Bcer	TRUE	NA	NA	NA
Bc_ctg1_1017	B4	0	Bcer	S	Bcer	FALSE	NA	NA	NA
Bc_ctg1_1018	B4	11.94058404	Bcer	E	Bcer	FALSE	NA	NA	NA
Bc_ctg1_1019	B4	16.73913651	Bcer	F	Bcer	FALSE	NA	NA	NA
Bc_ctg1_102	B4	0	Bcer	L	Bcer	FALSE	NA	NA	NA
Bc_ctg1_1020	B4	15.9579768	Bcer	S	Bcer	FALSE	NA	NA	NA
Bc_ctg1_1021	B4	69.41161939	Bcer	E	Bcer	FALSE	NA	NA	NA
Bc_ctg1_1022	B4	31.91595361	Bcer	G	Bcer	FALSE	NA	NA	NA
Bc_ctg1_1023	B4	12.05217829	Bcer	E	Bcer	FALSE	NA	NA	NA
Bc_ctg1_1024	B4	21.42609473	Bcer	E	Bcer	FALSE	NA	NA	NA
Bc_ctg1_1025	B4	17.18551348	Bcer	S	Bcer	FALSE	NA	NA	NA
Bc_ctg1_1026	B4	1.004348191	Bcer	NA	Bcer	FALSE	NA	NA	NA
Bc_ctg1_1027	B4	9.597104932	Bcer	S	Bcer	FALSE	NA	NA	NA
Bc_ctg1_1028	B4	100.7696018	Bcer	I	Bcer	FALSE	NA	NA	NA
Bc_ctg1_1029	B4	109.8087355	Bcer	V	Bcer	FALSE	NA	NA	NA
Bc_ctg1_103	B4	0	Bcer	L	Bcer	FALSE	NA	NA	NA
Bc_ctg1_1030	B4	3.571015789	Bcer	T	Bcer	FALSE	NA	NA	NA
Bc_ctg1_1031	B4	33.58986726	Bcer	C	Bcer	FALSE	NA	NA	NA
Bc_ctg1_1032	B4	28.45653206	Bcer	NA	Bcer	FALSE	NA	NA	NA
Bc_ctg1_1033	B4	6.91884309	Bcer	S	Bcer	FALSE	NA	NA	NA
Bc_ctg1_1034	B4	2305.871851	Bcer	G	Bcer	FALSE	NA	NA	NA
Bc_ctg1_1035	B4	322.6189576	Bcer	G	Bcer	FALSE	NA	NA	NA
Bc_ctg1_1036	B4	990.3989101	Bcer	G	Bcer	FALSE	NA	NA	NA
Bc_ctg1_1037	B4	96.08264356	Bcer	K	Bcer	FALSE	NA	NA	NA
Bc_ctg1_1038	B4	191.1609389	Bcer	S	Bcer	FALSE	NA	NA	NA
Bc_ctg1_1039	B4	916.1887382	Bcer	K	Bcer	FALSE	NA	NA	NA
Bc_ctg1_104	B4	3.013044572	Bcer	S	Bcer	FALSE	NA	NA	NA
Bc_ctg1_1040	B4	1064.609082	Bcer	G	Bcer	FALSE	NA	NA	NA
Bc_ctg1_1041	B4	125.9899008	Bcer	G	Bcer	FALSE	NA	NA	NA
Bc_ctg1_1042	B4	2.901450328	Bcer	S	Bcer	FALSE	NA	NA	NA
Bc_ctg1_1043	B4	7.588408551	Bcer	D	Bcer	FALSE	NA	NA	NA
Bc_ctg1_1044	B4	109.3623585	Bcer	D	Bcer	FALSE	NA	NA	NA
Bc_ctg1_1046	B4	17.85507894	Bcer	S	Bcer	FALSE	NA	NA	NA
Bc_ctg1_1047	B4	190.3797792	Bcer	K	Bcer	FALSE	NA	NA	NA
Bc_ctg1_1048	B4	927.5713511	Bcer	O	Bcer	FALSE	NA	NA	NA
Bc_ctg1_1049	B4	288.8059019	Bcer	S	Bcer	FALSE	NA	NA	NA
Bc_ctg1_105	B4	1.227536677	Bcer	NA	Bcer	FALSE	NA	NA	NA
Bc_ctg1_1050	B4	21.53768897	Bcer	S	Bcer	FALSE	NA	NA	NA
Bc_ctg1_1051	B4	3.347827302	Bcer	EG	Bcer	FALSE	NA	NA	NA
Bc_ctg1_1052	B4	0.781159704	Bcer	S	Bcer	FALSE	NA	NA	NA
Bc_ctg1_1053	B4	0.111594243	Bcer	S	Bcer	FALSE	NA	NA	NA
Bc_ctg1_1054	B4	0.446376974	Bcer	IQ	Bcer	TRUE	NA	NA	NA
Bc_ctg1_1055	B4	0	Bcer	S	Bcer	FALSE	NA	NA	NA
Bc_ctg1_1057	B4	0	Bcer	S	Bcer	FALSE	NA	NA	NA
Bc_ctg1_1059	B4	1.673913651	Bcer	S	Bcer	FALSE	NA	NA	NA
Bc_ctg1_106	B4	0.446376974	Bcer	K	Bcer	FALSE	NA	NA	NA
Bc_ctg1_1060	B4	78.89713008	Bcer	K	Bcer	FALSE	NA	NA	NA
Bc_ctg1_1061	B4	40.50871035	Bcer	F	Bcer	FALSE	NA	NA	NA
Bc_ctg1_1062	B4	29.68406874	Bcer	T	Bcer	FALSE	NA	NA	NA
Bc_ctg1_1063	B4	0	Bcer	M	Bcer	FALSE	NA	NA	NA
Bc_ctg1_1064	B4	22.8768199	Bcer	K	Bcer	FALSE	NA	NA	NA
Bc_ctg1_1065	B4	71.30872153	Bcer	V	Bcer	FALSE	NA	NA	NA
Bc_ctg1_1066	B4	93.85075869	Bcer	S	Bcer	FALSE	NA	NA	NA
Bc_ctg1_1067	B4	1.450725164	Bcer	D	Bcer	FALSE	NA	NA	NA

Bc_ctg1_1068	B4	130.9000475	Bcer	K	Bcer	FALSE	NA	NA	NA
Bc_ctg1_1069	B4	0.781159704	Bcer	S	Bcer	FALSE	NA	NA	NA
Bc_ctg1_107	B4	11.49420707	Bcer	GIM	Bcer	FALSE	NA	NA	NA
Bc_ctg1_1070	B4	165.7174514	Bcer	S	Bcer	FALSE	NA	NA	NA
Bc_ctg1_1071	B4	96.08264356	Bcer	P	Bcer	FALSE	NA	NA	NA
Bc_ctg1_1072	B4	2505.17917	Bcer	F	Bcer	FALSE	NA	NA	NA
Bc_ctg1_1073	B4	1904.690546	Bcer	F	Bcer	FALSE	NA	NA	NA
Bc_ctg1_1074	B4	2199.968914	Bcer	G	Bcer	FALSE	NA	NA	NA
Bc_ctg1_1075	B4	84.92321922	Bcer	S	Bcer	FALSE	NA	NA	NA
Bc_ctg1_1076	B4	62.04639933	Bcer	L	Bcer	FALSE	NA	NA	NA
Bc_ctg1_1077	B4	0.892753947	Bcer	S	Bcer	FALSE	NA	NA	NA
Bc_ctg1_1078	B4	539.669761	Bcer	P	Bcer	FALSE	NA	NA	NA
Bc_ctg1_1079	B4	16.29275954	Bcer	S	Bcer	FALSE	NA	NA	NA
Bc_ctg1_108	B4	34.48262121	Bcer	K	Bcer	FALSE	NA	NA	NA
Bc_ctg1_1080	B4	207.5652927	Bcer	J	Bcer	FALSE	NA	NA	NA
Bc_ctg1_1081	B4	104.7869945	Bcer	L	Bcer	FALSE	NA	NA	NA
Bc_ctg1_1083	B4	94.52032415	Bcer	C	Bcer	FALSE	NA	NA	NA
Bc_ctg1_1084	B4	24.10435657	Bcer	S	Bcer	FALSE	NA	NA	NA
Bc_ctg1_1085	B4	6.026089143	Bcer	K	Bcer	FALSE	NA	NA	NA
Bc_ctg1_1086	B4	0	Bcer	S	Bcer	FALSE	NA	NA	NA
Bc_ctg1_1087	B4	131.4580187	Bcer	F	Bcer	FALSE	NA	NA	NA
Bc_ctg1_1088	B4	1.450725164	Bcer	S	Bcer	FALSE	NA	NA	NA
Bc_ctg1_1089	B4	25.66667598	Bcer	I	Bcer	FALSE	NA	NA	NA
Bc_ctg1_109	B4	1.897102138	Bcer	IQ	Bcer	TRUE	NA	NA	NA
Bc_ctg1_1090	B4	43.85653765	Bcer	S	Bcer	FALSE	NA	NA	NA
Bc_ctg1_1091	B4	26.11305295	Bcer	K	Bcer	FALSE	NA	NA	NA
Bc_ctg1_1092	B4	4.910146709	Bcer	K	Bcer	FALSE	NA	NA	NA
Bc_ctg1_1093	B4	161.8116529	Bcer	S	Bcer	FALSE	NA	NA	NA
Bc_ctg1_1094	B4	9.597104932	Bcer	S	Bcer	FALSE	NA	NA	NA
Bc_ctg1_1095	B4	59.36813748	Bcer	S	Bcer	FALSE	NA	NA	NA
Bc_ctg1_1096	B4	333.5551935	Bcer	H	Bcer	FALSE	NA	NA	NA
Bc_ctg1_1097	B4	205.8913791	Bcer	H	Bcer	FALSE	NA	NA	NA
Bc_ctg1_1098	B4	513.8914908	Bcer	H	Bcer	FALSE	NA	NA	NA
Bc_ctg1_1099	B4	245.1725527	Bcer	H	Bcer	FALSE	NA	NA	NA
Bc_ctg1_11	B4	2.343479111	Bcer	S	Bcer	FALSE	NA	NA	NA
Bc_ctg1_110	B4	2.343479111	Bcer	K	Bcer	FALSE	NA	NA	NA
Bc_ctg1_1100	B4	0	Bcer	NA	Bcer	FALSE	NA	NA	NA
Bc_ctg1_1101	B4	3.013044572	Bcer	H	Bcer	FALSE	NA	NA	NA
Bc_ctg1_1102	B4	1.897102138	Bcer	H	Bcer	FALSE	NA	NA	NA
Bc_ctg1_1103	B4	0.557971217	Bcer	S	Bcer	FALSE	NA	NA	NA
Bc_ctg1_1104	B4	2.231884868	Bcer	H	Bcer	FALSE	NA	NA	NA
Bc_ctg1_1105	B4	1.227536677	Bcer	H	Bcer	FALSE	NA	NA	NA
Bc_ctg1_1106	B4	1.562319407	Bcer	H	Bcer	FALSE	NA	NA	NA
Bc_ctg1_1107	B4	0	Bcer	NA	Bcer	FALSE	NA	NA	NA
Bc_ctg1_1108	B4	18.5246444	Bcer	C	Bcer	FALSE	NA	NA	NA
Bc_ctg1_1109	B4	170.8507866	Bcer	F	Bcer	FALSE	NA	NA	NA
Bc_ctg1_111	B4	13.05652648	Bcer	S	Bcer	FALSE	NA	NA	NA
Bc_ctg1_1110	B4	24.10435657	Bcer	E	Bcer	FALSE	NA	NA	NA
Bc_ctg1_1111	B4	125.7667123	Bcer	K	Bcer	FALSE	NA	NA	NA
Bc_ctg1_1112	B4	5.9144949	Bcer	NA	Bcer	FALSE	NA	NA	NA
Bc_ctg1_1113	B4	41.73624703	Bcer	S	Bcer	FALSE	NA	NA	NA
Bc_ctg1_1114	B4	7.030437334	Bcer	E	Bcer	FALSE	NA	NA	NA
Bc_ctg1_1115	B4	8.927539471	Bcer	E	Bcer	FALSE	NA	NA	NA
Bc_ctg1_1116	B4	4.240581249	Bcer	E	Bcer	FALSE	NA	NA	NA
Bc_ctg1_1117	B4	4.575363979	Bcer	E	Bcer	FALSE	NA	NA	NA
Bc_ctg1_1118	B4	2.008696381	Bcer	E	Bcer	FALSE	NA	NA	NA
Bc_ctg1_1119	B4	0.892753947	Bcer	NA	Bcer	FALSE	NA	NA	NA
Bc_ctg1_112	B4	25.10870476	Bcer	L	Bcer	FALSE	Bc_Ga0417192_01_rn01	Other	NA
Bc_ctg1_1120	B4	119.4058404	Bcer	S	Bcer	FALSE	NA	NA	NA
Bc_ctg1_1121	B4	63.05074752	Bcer	S	Bcer	FALSE	NA	NA	NA
Bc_ctg1_1122	B4	4.686958222	Bcer	E	Bcer	FALSE	NA	NA	NA
Bc_ctg1_1123	B4	218.724717	Bcer	E	Bcer	FALSE	NA	NA	NA

Bc_ctg1_1124	B4	104.1174291	Bcer	E	Bcer	FALSE	NA	NA	NA
Bc_ctg1_1125	B4	66.73335755	Bcer	S	Bcer	FALSE	NA	NA	NA
Bc_ctg1_1126	B4	81.68698616	Bcer	NA	Bcer	FALSE	NA	NA	NA
Bc_ctg1_1127	B4	2.901450328	Bcer	NA	Bcer	FALSE	NA	NA	NA
Bc_ctg1_1128	B4	105.45656	Bcer	P	Bcer	FALSE	NA	NA	NA
Bc_ctg1_1129	B4	14.95362861	Bcer	S	Bcer	FALSE	NA	NA	NA
Bc_ctg1_113	B4	6.472466117	Bcer	E	Bcer	FALSE	Bc_Ga0417192_01_rn01	Other	NA
Bc_ctg1_1130	B4	16.962325	Bcer	L	Bcer	FALSE	NA	NA	NA
Bc_ctg1_1132	B4	1763.30064	Bcer	E	Bcer	FALSE	NA	NA	NA
Bc_ctg1_1133	B4	113.3797513	Bcer	J	Bcer	FALSE	NA	NA	NA
Bc_ctg1_1134	B4	11.8289898	Bcer	NA	Bcer	FALSE	NA	NA	NA
Bc_ctg1_1135	B4	19.75218108	Bcer	S	Bcer	FALSE	NA	NA	NA
Bc_ctg1_1136	B4	59.47973173	Bcer	S	Bcer	FALSE	NA	NA	NA
Bc_ctg1_1137	B4	65.39422663	Bcer	E	Bcer	FALSE	NA	NA	NA
Bc_ctg1_1138	B4	37.16088305	Bcer	E	Bcer	FALSE	NA	NA	NA
Bc_ctg1_1139	B4	30.57682269	Bcer	ET	Bcer	FALSE	NA	NA	NA
Bc_ctg1_114	B4	1.785507894	Bcer	NA	Bcer	FALSE	Bc_Ga0417192_01_rn01	Other	NA
Bc_ctg1_1140	B4	64.16668995	Bcer	S	Bcer	FALSE	NA	NA	NA
Bc_ctg1_1141	B4	27.89856085	Bcer	S	Bcer	FALSE	NA	NA	NA
Bc_ctg1_1142	B4	26.00145871	Bcer	F	Bcer	FALSE	NA	NA	NA
Bc_ctg1_1143	B4	0.557971217	Bcer	S	Bcer	FALSE	NA	NA	NA
Bc_ctg1_1144	B4	348.2856336	Bcer	D	Bcer	FALSE	NA	NA	NA
Bc_ctg1_1145	B4	2878.461914	Bcer	C	Bcer	FALSE	NA	NA	NA
Bc_ctg1_1146	B4	1252.645382	Bcer	C	Bcer	FALSE	NA	NA	NA
Bc_ctg1_1147	B4	1409.993265	Bcer	C	Bcer	FALSE	NA	NA	NA
Bc_ctg1_1148	B4	1668.445533	Bcer	C	Bcer	FALSE	NA	NA	NA
Bc_ctg1_1149	B4	1074.875752	Bcer	C	Bcer	FALSE	NA	NA	NA
Bc_ctg1_1150	B4	3078.438798	Bcer	E	Bcer	FALSE	NA	NA	NA
Bc_ctg1_1151	B4	1833.828202	Bcer	C	Bcer	FALSE	NA	NA	NA
Bc_ctg1_1152	B4	36.2681291	Bcer	KT	Bcer	FALSE	NA	NA	NA
Bc_ctg1_1153	B4	2.120290624	Bcer	S	Bcer	FALSE	NA	NA	NA
Bc_ctg1_1154	B4	51.55654045	Bcer	C	Bcer	FALSE	NA	NA	NA
Bc_ctg1_1155	B4	0	Bcer	K	Bcer	FALSE	NA	NA	NA
Bc_ctg1_1156	B4	6.026089143	Bcer	NA	Bcer	FALSE	NA	NA	NA
Bc_ctg1_1157	B4	490.4566997	Bcer	KT	Bcer	FALSE	NA	NA	NA
Bc_ctg1_1158	B4	6.360871873	Bcer	M	Bcer	FALSE	NA	NA	NA
Bc_ctg1_1159	B4	79.23191281	Bcer	L	Bcer	FALSE	NA	NA	NA
Bc_ctg1_116	B4	5.691306413	Bcer	S	Bcer	FALSE	Bc_Ga0417192_01_rn01	Other	NA
Bc_ctg1_1160	B4	216.8276149	Bcer	K	Bcer	FALSE	NA	NA	NA
Bc_ctg1_1161	B4	140.0507755	Bcer	J	Bcer	FALSE	NA	NA	NA
Bc_ctg1_1162	B4	536.5451222	Bcer	H	Bcer	FALSE	NA	NA	NA
Bc_ctg1_1163	B4	237.3609557	Bcer	H	Bcer	FALSE	NA	NA	NA
Bc_ctg1_1164	B4	53.2304541	Bcer	L	Bcer	FALSE	NA	NA	NA
Bc_ctg1_1165	B4	468.0262568	Bcer	L	Bcer	FALSE	NA	NA	NA
Bc_ctg1_1166	B4	302.1972111	Bcer	F	Bcer	FALSE	NA	NA	NA
Bc_ctg1_1167	B4	32.80870756	Bcer	K	Bcer	FALSE	NA	NA	NA
Bc_ctg1_1168	B4	783.6147771	Bcer	S	Bcer	FALSE	NA	NA	NA
Bc_ctg1_1169	B4	1974.660137	Bcer	I	Bcer	FALSE	NA	NA	NA
Bc_ctg1_117	B4	2.678261841	Bcer	NA	Bcer	FALSE	Bc_Ga0417192_01_rn01	Other	NA
Bc_ctg1_1170	B4	239.9276233	Bcer	I	Bcer	FALSE	NA	NA	NA
Bc_ctg1_1171	B4	0	Bcer	NA	Bcer	FALSE	NA	NA	NA
Bc_ctg1_1172	B4	0	Bcer	S	Bcer	FALSE	NA	NA	NA
Bc_ctg1_1173	B4	0.66956546	Bcer	S	Bcer	FALSE	NA	NA	NA
Bc_ctg1_1174	B4	0.446376974	Bcer	S	Bcer	FALSE	NA	NA	NA
Bc_ctg1_1175	B4	1.004348191	Bcer	S	Bcer	FALSE	NA	NA	NA
Bc_ctg1_1176	B4	0.66956546	Bcer	S	Bcer	FALSE	NA	NA	NA
Bc_ctg1_1177	B4	0	Bcer	S	Bcer	FALSE	NA	NA	NA
Bc_ctg1_1178	B4	0.223188487	Bcer	S	Bcer	FALSE	NA	NA	NA
Bc_ctg1_1179	B4	0.781159704	Bcer	S	Bcer	FALSE	NA	NA	NA
Bc_ctg1_118	B4	35.93334637	Bcer	EGP	Bcer	FALSE	Bc_Ga0417192_01_rn01	Transport	SMCOG1106
Bc_ctg1_1180	B4	0	Bcer	S	Bcer	FALSE	NA	NA	NA
Bc_ctg1_1181	B4	1.673913651	Bcer	J	Bcer	FALSE	NA	NA	NA

Bc_ctg1_1182	B4	2.901450328	Bcer	NA	Bcer	FALSE	NA	NA	NA
Bc_ctg1_1183	B4	886.9510465	Bcer	J	Bcer	FALSE	NA	NA	NA
Bc_ctg1_1184	B4	591.2263015	Bcer	E	Bcer	FALSE	NA	NA	NA
Bc_ctg1_1185	B4	33.36667877	Bcer	E	Bcer	FALSE	NA	NA	NA
Bc_ctg1_1186	B4	1.227536677	Bcer	S	Bcer	FALSE	NA	NA	NA
Bc_ctg1_1187	B4	123.2000447	Bcer	S	Bcer	FALSE	NA	NA	NA
Bc_ctg1_1188	B4	55.35074472	Bcer	NA	Bcer	FALSE	NA	NA	NA
Bc_ctg1_1189	B4	26.44783568	Bcer	S	Bcer	FALSE	NA	NA	NA
Bc_ctg1_119	B4	12.72174375	Bcer	K	Bcer	FALSE	Bc_Ga0417192_01_rn01	Other	NA
Bc_ctg1_1190	B4	28.56812631	Bcer	K	Bcer	FALSE	NA	NA	NA
Bc_ctg1_1191	B4	0.223188487	Bcer	L	Bcer	FALSE	NA	NA	NA
Bc_ctg1_1192	B4	2.120290624	Bcer	K	Bcer	FALSE	NA	NA	NA
Bc_ctg1_1193	B4	6.026089143	Bcer	S	Bcer	FALSE	NA	NA	NA
Bc_ctg1_1194	B4	18.41305016	Bcer	NU	Bcer	FALSE	NA	NA	NA
Bc_ctg1_1195	B4	57.13625262	Bcer	H	Bcer	FALSE	NA	NA	NA
Bc_ctg1_1196	B4	75.66089702	Bcer	P	Bcer	FALSE	NA	NA	NA
Bc_ctg1_1197	B4	0.33478273	Bcer	NA	Bcer	FALSE	NA	NA	NA
Bc_ctg1_1198	B4	32.9203018	Bcer	K	Bcer	FALSE	NA	NA	NA
Bc_ctg1_1199	B4	30.80001118	Bcer	K	Bcer	FALSE	NA	NA	NA
Bc_ctg1_120	B4	34.25943272	Bcer	K	Bcer	FALSE	Bc_Ga0417192_01_rn01	Other	NA
Bc_ctg1_1200	B4	4.686958222	Bcer	P	Bcer	FALSE	NA	NA	NA
Bc_ctg1_1201	B4	25.88986447	Bcer	P	Bcer	FALSE	NA	NA	NA
Bc_ctg1_1202	B4	3.682610032	Bcer	NA	Bcer	FALSE	NA	NA	NA
Bc_ctg1_1203	B4	0	Bcer	NA	Bcer	FALSE	NA	NA	NA
Bc_ctg1_1204	B4	1.450725164	Bcer	S	Bcer	FALSE	NA	NA	NA
Bc_ctg1_1205	B4	0.66956546	Bcer	S	Bcer	FALSE	NA	NA	NA
Bc_ctg1_1206	B4	2.566667598	Bcer	S	Bcer	FALSE	NA	NA	NA
Bc_ctg1_1207	B4	4.686958222	Bcer	S	Bcer	FALSE	NA	NA	NA
Bc_ctg1_1208	B4	6.24927763	Bcer	V	Bcer	FALSE	NA	NA	NA
Bc_ctg1_1209	B4	0.33478273	Bcer	K	Bcer	FALSE	NA	NA	NA
Bc_ctg1_121	B4	29.79566299	Bcer	C	Bcer	FALSE	Bc_Ga0417192_01_rn01	Ancillary biosynthesis	SMCOG1007
Bc_ctg1_1210	B4	3.459421545	Bcer	NA	Bcer	FALSE	NA	NA	NA
Bc_ctg1_1211	B4	1.115942434	Bcer	S	Bcer	FALSE	NA	NA	NA
Bc_ctg1_1212	B4	3619.336096	Bcer	E	Bcer	FALSE	NA	NA	NA
Bc_ctg1_1213	B4	3360.437451	Bcer	E	Bcer	FALSE	NA	NA	NA
Bc_ctg1_1214	B4	1465.678793	Bcer	E	Bcer	FALSE	NA	NA	NA
Bc_ctg1_1215	B4	2.566667598	Bcer	L	Bcer	FALSE	NA	NA	NA
Bc_ctg1_1216	B4	1.897102138	Bcer	S	Bcer	FALSE	NA	NA	NA
Bc_ctg1_1218	B4	4.240581249	Bcer	NA	Bcer	FALSE	NA	NA	NA
Bc_ctg1_1219	B4	19.52899259	Bcer	NA	Bcer	FALSE	NA	NA	NA
Bc_ctg1_122	B4	19.52899259	Bcer	S	Bcer	FALSE	Bc_Ga0417192_01_rn01	Core biosynthesis	None
Bc_ctg1_1220	B4	0.446376974	Bcer	S	Bcer	FALSE	NA	NA	NA
Bc_ctg1_1221	B4	8.146379768	Bcer	F	Bcer	FALSE	NA	NA	NA
Bc_ctg1_1222	B4	67.40292301	Bcer	O	Bcer	FALSE	NA	NA	NA
Bc_ctg1_1223	B4	0.111594243	Bcer	S	Bcer	FALSE	Bc_Ga0417192_01_rn10	Other	NA
Bc_ctg1_1224	B4	0.223188487	Bcer	U	Bcer	FALSE	Bc_Ga0417192_01_rn10	Other	NA
Bc_ctg1_1225	B4	0.111594243	Bcer	NA	Bcer	FALSE	Bc_Ga0417192_01_rn10	Other	NA
Bc_ctg1_1226	B4	0.446376974	Bcer	NU	Bcer	FALSE	Bc_Ga0417192_01_rn10	Other	NA
Bc_ctg1_1227	B4	0.557971217	Bcer	U	Bcer	FALSE	Bc_Ga0417192_01_rn10	Other	NA
Bc_ctg1_1228	B4	1.673913651	Bcer	NU	Bcer	FALSE	Bc_Ga0417192_01_rn10	Other	NA
Bc_ctg1_1229	B4	3.459421545	Bcer	NU	Bcer	FALSE	Bc_Ga0417192_01_rn10	Other	NA
Bc_ctg1_123	B4	12.72174375	Bcer	NT	Bcer	FALSE	Bc_Ga0417192_01_rn01	Other	SMCOG1061
Bc_ctg1_1230	B4	346.5001257	Bcer	K	Bcer	FALSE	Bc_Ga0417192_01_rn10	Other	NA
Bc_ctg1_1231	B4	3.682610032	Bcer	S	Bcer	FALSE	Bc_Ga0417192_01_rn10	Other	NA
Bc_ctg1_1232	B4	87.71307531	Bcer	U	Bcer	FALSE	Bc_Ga0417192_01_rn10	Transport	SMCOG1212
Bc_ctg1_1233	B4	0.111594243	Bcer	NA	Bcer	FALSE	Bc_Ga0417192_01_rn10	Other	NA
Bc_ctg1_1234	B4	0.66956546	Bcer	S	Bcer	FALSE	Bc_Ga0417192_01_rn10	Other	NA
Bc_ctg1_1235	B4	35.4869694	Bcer	NA	Bcer	FALSE	Bc_Ga0417192_01_rn10	Other	NA
Bc_ctg1_1236	B4	43.63334917	Bcer	NA	Bcer	FALSE	Bc_Ga0417192_01_rn10	Other	NA
Bc_ctg1_1237	B4	18.30145592	Bcer	NA	Bcer	FALSE	Bc_Ga0417192_01_rn10	Other	NA
Bc_ctg1_1238	B4	18.74783289	Bcer	S	Bcer	FALSE	Bc_Ga0417192_01_rn10	Other	NA
Bc_ctg1_1239	B4	95.85945507	Bcer	M	Bcer	FALSE	Bc_Ga0417192_01_rn10	Core biosynthesis	None

Bc_ctg1_124	B4	0	Bcer	NA	Bcer	FALSE	Bc_Ga0417192_01_rn01	Other	NA
Bc_ctg1_1240	B4	53.67683107	Bcer	E	Bcer	FALSE	Bc_Ga0417192_01_rn10	Other	NA
Bc_ctg1_1241	B4	17.96667319	Bcer	E	Bcer	FALSE	Bc_Ga0417192_01_rn10	Other	NA
Bc_ctg1_1242	B4	9.150727958	Bcer	E	Bcer	FALSE	Bc_Ga0417192_01_rn10	Core biosynthesis	SMCOG1168
Bc_ctg1_1243	B4	10.60145312	Bcer	E	Bcer	FALSE	Bc_Ga0417192_01_rn10	Ancillary biosynthesis	SMCOG1168
Bc_ctg1_1244	B4	7.811597037	Bcer	Q	Bcer	TRUE	Bc_Ga0417192_01_rn10	Other	NA
Bc_ctg1_1245	B4	105.1217773	Bcer	K	Bcer	FALSE	Bc_Ga0417192_01_rn10	Regulation	SMCOG1058
Bc_ctg1_1246	B4	119.9638116	Bcer	S	Bcer	FALSE	Bc_Ga0417192_01_rn10	Other	NA
Bc_ctg1_1247	B4	53.45364258	Bcer	S	Bcer	FALSE	Bc_Ga0417192_01_rn10	Ancillary biosynthesis	SMCOG1170
Bc_ctg1_1248	B4	17.52029621	Bcer	S	Bcer	FALSE	Bc_Ga0417192_01_rn10	Other	NA
Bc_ctg1_1249	B4	205.7797848	Bcer	G	Bcer	FALSE	Bc_Ga0417192_01_rn10	Other	SMCOG1108
Bc_ctg1_125	B4	0	Bcer	L	Bcer	FALSE	Bc_Ga0417192_01_rn01	Other	NA
Bc_ctg1_1250	B4	17.85507894	Bcer	S	Bcer	FALSE	Bc_Ga0417192_01_rn10	Other	NA
Bc_ctg1_1251	B4	59.47973173	Bcer	H	Bcer	FALSE	Bc_Ga0417192_01_rn10	Other	NA
Bc_ctg1_1252	B4	44.30291463	Bcer	J	Bcer	FALSE	Bc_Ga0417192_01_rn10	Other	NA
Bc_ctg1_1253	B4	17.52029621	Bcer	GJM	Bcer	FALSE	Bc_Ga0417192_01_rn10	Ancillary biosynthesis	SMCOG1064
Bc_ctg1_1254	B4	0.557971217	Bcer	P	Bcer	FALSE	NA	NA	NA
Bc_ctg1_1255	B4	3.905798519	Bcer	P	Bcer	FALSE	NA	NA	NA
Bc_ctg1_1256	B4	3.013044572	Bcer	P	Bcer	FALSE	NA	NA	NA
Bc_ctg1_1257	B4	1.562319407	Bcer	P	Bcer	FALSE	NA	NA	NA
Bc_ctg1_1258	B4	1.227536677	Bcer	P	Bcer	FALSE	NA	NA	NA
Bc_ctg1_1259	B4	862.2887187	Bcer	M	Bcer	FALSE	NA	NA	NA
Bc_ctg1_126	B4	4.463769736	Bcer	V	Bcer	FALSE	Bc_Ga0417192_01_rn01	Ancillary biosynthesis	SMCOG1053
Bc_ctg1_1260	B4	3.347827302	Bcer	EGP	Bcer	FALSE	NA	NA	NA
Bc_ctg1_1261	B4	252.5377728	Bcer	P	Bcer	FALSE	NA	NA	NA
Bc_ctg1_1262	B4	151.0986056	Bcer	S	Bcer	FALSE	NA	NA	NA
Bc_ctg1_1263	B4	0.781159704	Bcer	M	Bcer	FALSE	NA	NA	NA
Bc_ctg1_1264	B4	473.8291574	Bcer	I	Bcer	FALSE	NA	NA	NA
Bc_ctg1_1265	B4	20.64493503	Bcer	P	Bcer	FALSE	NA	NA	NA
Bc_ctg1_1266	B4	52.22610591	Bcer	P	Bcer	FALSE	NA	NA	NA
Bc_ctg1_1267	B4	58.14060081	Bcer	P	Bcer	FALSE	NA	NA	NA
Bc_ctg1_1268	B4	191.9420986	Bcer	S	Bcer	FALSE	NA	NA	NA
Bc_ctg1_127	B4	2.678261841	Bcer	V	Bcer	FALSE	Bc_Ga0417192_01_rn01	Ancillary biosynthesis	SMCOG1053
Bc_ctg1_1270	B4	74.65654883	Bcer	L	Bcer	FALSE	NA	NA	NA
Bc_ctg1_1271	B4	264.7015453	Bcer	JKL	Bcer	FALSE	NA	NA	NA
Bc_ctg1_1272	B4	0	Bcer	NA	Bcer	FALSE	NA	NA	NA
Bc_ctg1_1273	B4	230.3305184	Bcer	IM	Bcer	FALSE	NA	NA	NA
Bc_ctg1_1274	B4	60.37248567	Bcer	S	Bcer	FALSE	NA	NA	NA
Bc_ctg1_1275	B4	16.73913651	Bcer	S	Bcer	FALSE	NA	NA	NA
Bc_ctg1_1276	B4	15.1768171	Bcer	C	Bcer	FALSE	NA	NA	NA
Bc_ctg1_1277	B4	908.3771412	Bcer	K	Bcer	FALSE	NA	NA	NA
Bc_ctg1_1278	B4	1546.138242	Bcer	L	Bcer	FALSE	NA	NA	NA
Bc_ctg1_1279	B4	569.5770183	Bcer	S	Bcer	FALSE	NA	NA	NA
Bc_ctg1_128	B4	0.557971217	Bcer	S	Bcer	FALSE	Bc_Ga0417192_01_rn01	Other	NA
Bc_ctg1_1280	B4	460.4378482	Bcer	K	Bcer	FALSE	NA	NA	NA
Bc_ctg1_1281	B4	96.1942378	Bcer	L	Bcer	FALSE	NA	NA	NA
Bc_ctg1_1282	B4	0	Bcer	S	Bcer	FALSE	NA	NA	NA
Bc_ctg1_1283	B4	666.8871985	Bcer	S	Bcer	FALSE	NA	NA	NA
Bc_ctg1_1284	B4	99.31887662	Bcer	F	Bcer	FALSE	NA	NA	NA
Bc_ctg1_1285	B4	9.150727958	Bcer	M	Bcer	FALSE	NA	NA	NA
Bc_ctg1_1286	B4	27.89856085	Bcer	S	Bcer	FALSE	NA	NA	NA
Bc_ctg1_1287	B4	230.1073299	Bcer	S	Bcer	FALSE	NA	NA	NA
Bc_ctg1_1288	B4	265.1479223	Bcer	T	Bcer	FALSE	NA	NA	NA
Bc_ctg1_1289	B4	1.897102138	Bcer	S	Bcer	FALSE	NA	NA	NA
Bc_ctg1_129	B4	0.111594243	Bcer	S	Bcer	FALSE	Bc_Ga0417192_01_rn01	Other	NA
Bc_ctg1_1290	B4	0	Bcer	S	Bcer	FALSE	NA	NA	NA
Bc_ctg1_1291	B4	1.673913651	Bcer	S	Bcer	FALSE	NA	NA	NA
Bc_ctg1_1292	B4	937.9496157	Bcer	S	Bcer	FALSE	NA	NA	NA
Bc_ctg1_1293	B4	365.4711471	Bcer	J	Bcer	FALSE	NA	NA	NA
Bc_ctg1_1294	B4	318.8247534	Bcer	J	Bcer	FALSE	NA	NA	NA
Bc_ctg1_1295	B4	147.0812128	Bcer	J	Bcer	FALSE	NA	NA	NA
Bc_ctg1_1296	B4	288.5827134	Bcer	J	Bcer	FALSE	NA	NA	NA

Bc_ctg1_1297	B4	202.208769	Bcer	O	Bcer	FALSE	NA	NA	NA
Bc_ctg1_1298	B4	2037.152913	Bcer	O	Bcer	FALSE	NA	NA	NA
Bc_ctg1_1299	B4	324.7392483	Bcer	O	Bcer	FALSE	NA	NA	NA
Bc_ctg1_13	B4	35.93334637	Bcer	Q	Bcer	TRUE	NA	NA	NA
Bc_ctg1_130	B4	1.897102138	Bcer	K	Bcer	FALSE	Bc_Ga0417192_01_rn01	Regulation	SMCOG1284
Bc_ctg1_1300	B4	231.3348665	Bcer	K	Bcer	FALSE	NA	NA	NA
Bc_ctg1_1301	B4	47.3159592	Bcer	H	Bcer	FALSE	NA	NA	NA
Bc_ctg1_1302	B4	7.25362582	Bcer	K	Bcer	FALSE	NA	NA	NA
Bc_ctg1_1303	B4	561.20745	Bcer	M	Bcer	FALSE	NA	NA	NA
Bc_ctg1_1304	B4	0.557971217	Bcer	NA	Bcer	FALSE	NA	NA	NA
Bc_ctg1_1305	B4	3.794204275	Bcer	C	Bcer	FALSE	NA	NA	NA
Bc_ctg1_1306	B4	270.950823	Bcer	J	Bcer	FALSE	NA	NA	NA
Bc_ctg1_1307	B4	61.48842811	Bcer	L	Bcer	FALSE	NA	NA	NA
Bc_ctg1_1308	B4	0.223188487	Bcer	S	Bcer	FALSE	NA	NA	NA
Bc_ctg1_1309	B4	12.38696102	Bcer	S	Bcer	FALSE	NA	NA	NA
Bc_ctg1_1310	B4	41.17827581	Bcer	F	Bcer	FALSE	NA	NA	NA
Bc_ctg1_1311	B4	3.236233058	Bcer	L	Bcer	FALSE	NA	NA	NA
Bc_ctg1_1312	B4	0	Bcer	E	Bcer	FALSE	NA	NA	NA
Bc_ctg1_1313	B4	234.0131284	Bcer	Q	Bcer	TRUE	NA	NA	NA
Bc_ctg1_1314	B4	144.5145452	Bcer	J	Bcer	FALSE	NA	NA	NA
Bc_ctg1_1315	B4	110.1435182	Bcer	H	Bcer	FALSE	NA	NA	NA
Bc_ctg1_1316	B4	131.9043957	Bcer	H	Bcer	FALSE	NA	NA	NA
Bc_ctg1_1317	B4	33.7014615	Bcer	J	Bcer	FALSE	NA	NA	NA
Bc_ctg1_1318	B4	182.1218052	Bcer	E	Bcer	FALSE	NA	NA	NA
Bc_ctg1_1319	B4	269.611692	Bcer	S	Bcer	FALSE	NA	NA	NA
Bc_ctg1_1320	B4	85.92756741	Bcer	S	Bcer	FALSE	NA	NA	NA
Bc_ctg1_1321	B4	0	Bcer	NA	Bcer	FALSE	NA	NA	NA
Bc_ctg1_1322	B4	72.87104093	Bcer	I	Bcer	FALSE	NA	NA	NA
Bc_ctg1_1323	B4	1.897102138	Bcer	K	Bcer	FALSE	NA	NA	NA
Bc_ctg1_1324	B4	3.124638815	Bcer	NA	Bcer	FALSE	NA	NA	NA
Bc_ctg1_1325	B4	10.60145312	Bcer	K	Bcer	FALSE	NA	NA	NA
Bc_ctg1_1327	B4	5.468117926	Bcer	C	Bcer	FALSE	NA	NA	NA
Bc_ctg1_1328	B4	420.9334861	Bcer	S	Bcer	FALSE	NA	NA	NA
Bc_ctg1_1329	B4	0	Bcer	NA	Bcer	FALSE	NA	NA	NA
Bc_ctg1_133	B4	0	Bcer	NA	Bcer	FALSE	NA	NA	NA
Bc_ctg1_1330	B4	0.781159704	Bcer	P	Bcer	FALSE	NA	NA	NA
Bc_ctg1_1331	B4	7.923191281	Bcer	K	Bcer	FALSE	NA	NA	NA
Bc_ctg1_1332	B4	10.26667039	Bcer	I	Bcer	FALSE	NA	NA	NA
Bc_ctg1_1333	B4	11.94058404	Bcer	S	Bcer	FALSE	NA	NA	NA
Bc_ctg1_1334	B4	0.781159704	Bcer	NA	Bcer	FALSE	NA	NA	NA
Bc_ctg1_1335	B4	6.137683386	Bcer	G	Bcer	FALSE	NA	NA	NA
Bc_ctg1_1336	B4	5.57971217	Bcer	NA	Bcer	FALSE	NA	NA	NA
Bc_ctg1_1338	B4	1.450725164	Bcer	U	Bcer	FALSE	NA	NA	NA
Bc_ctg1_1339	B4	19.41739835	Bcer	O	Bcer	FALSE	NA	NA	NA
Bc_ctg1_134	B4	0.111594243	Bcer	NA	Bcer	FALSE	NA	NA	NA
Bc_ctg1_1340	B4	3.124638815	Bcer	E	Bcer	FALSE	NA	NA	NA
Bc_ctg1_1341	B4	0.892753947	Bcer	H	Bcer	FALSE	NA	NA	NA
Bc_ctg1_1342	B4	16.962325	Bcer	S	Bcer	FALSE	NA	NA	NA
Bc_ctg1_1343	B4	0.781159704	Bcer	S	Bcer	FALSE	NA	NA	NA
Bc_ctg1_1344	B4	11.71739556	Bcer	K	Bcer	FALSE	NA	NA	NA
Bc_ctg1_1345	B4	0.892753947	Bcer	I	Bcer	FALSE	NA	NA	NA
Bc_ctg1_1346	B4	4.686958222	Bcer	K	Bcer	FALSE	NA	NA	NA
Bc_ctg1_1347	B4	1.562319407	Bcer	J	Bcer	FALSE	NA	NA	NA
Bc_ctg1_1348	B4	1.450725164	Bcer	C	Bcer	FALSE	NA	NA	NA
Bc_ctg1_1349	B4	9.931887662	Bcer	HP	Bcer	FALSE	NA	NA	NA
Bc_ctg1_135	B4	3.794204275	Bcer	T	Bcer	FALSE	NA	NA	NA
Bc_ctg1_1350	B4	14.17246891	Bcer	P	Bcer	FALSE	NA	NA	NA
Bc_ctg1_1351	B4	18.63623865	Bcer	P	Bcer	FALSE	NA	NA	NA
Bc_ctg1_1352	B4	0	Bcer	S	Bcer	FALSE	NA	NA	NA
Bc_ctg1_1353	B4	3997.863769	Bcer	C	Bcer	FALSE	NA	NA	NA
Bc_ctg1_1354	B4	481.6407545	Bcer	E	Bcer	FALSE	NA	NA	NA
Bc_ctg1_1355	B4	256.2203828	Bcer	E	Bcer	FALSE	NA	NA	NA

Bc_ctg1_1356	B4	340.0276596	Bcer	E	Bcer	FALSE	NA	NA	NA
Bc_ctg1_1357	B4	101.9971385	Bcer	Q	Bcer	TRUE	NA	NA	NA
Bc_ctg1_1358	B4	1.227536677	Bcer	S	Bcer	FALSE	NA	NA	NA
Bc_ctg1_1359	B4	332.5508453	Bcer	S	Bcer	FALSE	NA	NA	NA
Bc_ctg1_136	B4	10.04348191	Bcer	T	Bcer	FALSE	NA	NA	NA
Bc_ctg1_1360	B4	0.33478273	Bcer	M	Bcer	FALSE	NA	NA	NA
Bc_ctg1_1363	B4	189.4870253	Bcer	K	Bcer	FALSE	NA	NA	NA
Bc_ctg1_1364	B4	126.7710605	Bcer	F	Bcer	FALSE	NA	NA	NA
Bc_ctg1_1365	B4	283.4493782	Bcer	O	Bcer	FALSE	NA	NA	NA
Bc_ctg1_1366	B4	39.95073913	Bcer	O	Bcer	FALSE	NA	NA	NA
Bc_ctg1_1367	B4	16.29275954	Bcer	S	Bcer	FALSE	NA	NA	NA
Bc_ctg1_1368	B4	173.8638312	Bcer	S	Bcer	FALSE	NA	NA	NA
Bc_ctg1_1369	B4	128.8913511	Bcer	S	Bcer	FALSE	NA	NA	NA
Bc_ctg1_137	B4	1.227536677	Bcer	NA	Bcer	FALSE	NA	NA	NA
Bc_ctg1_1370	B4	366.6986838	Bcer	L	Bcer	FALSE	NA	NA	NA
Bc_ctg1_1371	B4	262.0232835	Bcer	S	Bcer	FALSE	NA	NA	NA
Bc_ctg1_1372	B4	853.5843677	Bcer	J	Bcer	FALSE	Bc_Ga0417192_01_rn11	Other	NA
Bc_ctg1_1373	B4	4.240581249	Bcer	S	Bcer	FALSE	Bc_Ga0417192_01_rn11	Other	NA
Bc_ctg1_1374	B4	0.446376974	Bcer	S	Bcer	FALSE	Bc_Ga0417192_01_rn11	Other	NA
Bc_ctg1_1375	B4	0	Bcer	NA	Bcer	FALSE	Bc_Ga0417192_01_rn11	Other	NA
Bc_ctg1_1377	B4	7.588408551	Bcer	NA	Bcer	FALSE	Bc_Ga0417192_01_rn11	Other	NA
Bc_ctg1_1378	B4	110.7014894	Bcer	L	Bcer	FALSE	Bc_Ga0417192_01_rn11	Other	NA
Bc_ctg1_1379	B4	45.75363979	Bcer	S	Bcer	FALSE	Bc_Ga0417192_01_rn11	Other	SMCOG1272
Bc_ctg1_138	B4	81.57539192	Bcer	S	Bcer	FALSE	NA	NA	NA
Bc_ctg1_1380	B4	458.7639346	Bcer	J	Bcer	FALSE	Bc_Ga0417192_01_rn11	Other	NA
Bc_ctg1_1381	B4	426.1784155	Bcer	E	Bcer	FALSE	Bc_Ga0417192_01_rn11	Core biosynthesis	SMCOG1139
Bc_ctg1_1382	B4	95.85945507	Bcer	K	Bcer	FALSE	Bc_Ga0417192_01_rn11	Other	NA
Bc_ctg1_1383	B4	39.39276792	Bcer	L	Bcer	FALSE	Bc_Ga0417192_01_rn11	Other	NA
Bc_ctg1_1384	B4	26.2246472	Bcer	S	Bcer	FALSE	Bc_Ga0417192_01_rn11	Other	NA
Bc_ctg1_1385	B4	192.7232583	Bcer	H	Bcer	FALSE	Bc_Ga0417192_01_rn11	Ancillary biosynthesis	None
Bc_ctg1_1386	B4	489.4523515	Bcer	J	Bcer	FALSE	Bc_Ga0417192_01_rn11	Other	NA
Bc_ctg1_1387	B4	262.6928489	Bcer	J	Bcer	FALSE	Bc_Ga0417192_01_rn11	Other	SMCOG1162
Bc_ctg1_1388	B4	0.33478273	Bcer	NA	Bcer	FALSE	Bc_Ga0417192_01_rn11	Other	NA
Bc_ctg1_1389	B4	65.8406036	Bcer	J	Bcer	FALSE	Bc_Ga0417192_01_rn11	Other	NA
Bc_ctg1_139	B4	2.008696381	Bcer	S	Bcer	FALSE	NA	NA	NA
Bc_ctg1_1390	B4	607.072684	Bcer	KT	Bcer	FALSE	Bc_Ga0417192_01_rn11	Other	NA
Bc_ctg1_1391	B4	184.8000671	Bcer	F	Bcer	FALSE	Bc_Ga0417192_01_rn11	Other	NA
Bc_ctg1_1392	B4	369.2653514	Bcer	L	Bcer	FALSE	Bc_Ga0417192_01_rn11	Other	NA
Bc_ctg1_1393	B4	8.592756741	Bcer	P	Bcer	FALSE	Bc_Ga0417192_01_rn11	Other	NA
Bc_ctg1_1394	B4	1044.410524	Bcer	U	Bcer	FALSE	Bc_Ga0417192_01_rn11	Other	NA
Bc_ctg1_1395	B4	17.85507894	Bcer	G	Bcer	FALSE	Bc_Ga0417192_01_rn11	Other	NA
Bc_ctg1_1396	B4	0	Bcer	S	Bcer	FALSE	Bc_Ga0417192_01_rn11	Core biosynthesis	SMCOG1086
Bc_ctg1_1397	B4	3.459421545	Bcer	S	Bcer	FALSE	Bc_Ga0417192_01_rn11	Other	NA
Bc_ctg1_1398	B4	0	Bcer	S	Bcer	FALSE	Bc_Ga0417192_01_rn11	Other	NA
Bc_ctg1_1399	B4	92.51162777	Bcer	U	Bcer	FALSE	Bc_Ga0417192_01_rn11	Other	NA
Bc_ctg1_14	B4	0.892753947	Bcer	F	Bcer	FALSE	NA	NA	NA
Bc_ctg1_140	B4	3.794204275	Bcer	S	Bcer	FALSE	NA	NA	NA
Bc_ctg1_1400	B4	293.0464831	Bcer	F	Bcer	FALSE	Bc_Ga0417192_01_rn11	Other	NA
Bc_ctg1_1401	B4	208.9044236	Bcer	J	Bcer	FALSE	Bc_Ga0417192_01_rn11	Other	NA
Bc_ctg1_1402	B4	1.227536677	Bcer	S	Bcer	FALSE	Bc_Ga0417192_01_rn11	Other	NA
Bc_ctg1_1403	B4	107.3536621	Bcer	L	Bcer	FALSE	Bc_Ga0417192_01_rn11	Other	NA
Bc_ctg1_1404	B4	41.06668157	Bcer	L	Bcer	FALSE	Bc_Ga0417192_01_rn11	Other	NA
Bc_ctg1_1405	B4	7.811597037	Bcer	P	Bcer	FALSE	Bc_Ga0417192_01_rn11	Transport	SMCOG1033
Bc_ctg1_1406	B4	0.223188487	Bcer	S	Bcer	FALSE	Bc_Ga0417192_01_rn11	Other	NA
Bc_ctg1_1407	B4	21.091312	Bcer	NA	Bcer	FALSE	Bc_Ga0417192_01_rn11	Other	NA
Bc_ctg1_1408	B4	0.892753947	Bcer	S	Bcer	FALSE	Bc_Ga0417192_01_rn11	Other	NA
Bc_ctg1_1409	B4	0.781159704	Bcer	M	Bcer	FALSE	NA	NA	NA
Bc_ctg1_141	B4	0.781159704	Bcer	S	Bcer	FALSE	NA	NA	NA
Bc_ctg1_1410	B4	3.124638815	Bcer	H	Bcer	FALSE	NA	NA	NA
Bc_ctg1_1411	B4	2.231884868	Bcer	H	Bcer	FALSE	NA	NA	NA
Bc_ctg1_1412	B4	3.347827302	Bcer	H	Bcer	FALSE	NA	NA	NA
Bc_ctg1_1413	B4	12.27536677	Bcer	E	Bcer	FALSE	NA	NA	NA

Bc_ctg1_1414	B4	45.97682828	Bcer	S	Bcer	FALSE	NA	NA	NA
Bc_ctg1_1415	B4	71.19712728	Bcer	S	Bcer	FALSE	NA	NA	NA
Bc_ctg1_1416	B4	87.93626379	Bcer	E	Bcer	FALSE	NA	NA	NA
Bc_ctg1_1417	B4	10.15507615	Bcer	V	Bcer	FALSE	NA	NA	NA
Bc_ctg1_1418	B4	55.35074472	Bcer	V	Bcer	FALSE	NA	NA	NA
Bc_ctg1_1419	B4	12.94493223	Bcer	V	Bcer	FALSE	NA	NA	NA
Bc_ctg1_142	B4	35.37537515	Bcer	T	Bcer	FALSE	NA	NA	NA
Bc_ctg1_1420	B4	26.78261841	Bcer	T	Bcer	FALSE	NA	NA	NA
Bc_ctg1_1421	B4	15.51159983	Bcer	T	Bcer	FALSE	NA	NA	NA
Bc_ctg1_1422	B4	438.1189996	Bcer	S	Bcer	FALSE	NA	NA	NA
Bc_ctg1_1423	B4	166.4986111	Bcer	T	Bcer	FALSE	NA	NA	NA
Bc_ctg1_1424	B4	1260.233791	Bcer	J	Bcer	FALSE	NA	NA	NA
Bc_ctg1_1425	B4	1889.625323	Bcer	J	Bcer	FALSE	NA	NA	NA
Bc_ctg1_1426	B4	513.7798966	Bcer	J	Bcer	FALSE	NA	NA	NA
Bc_ctg1_1427	B4	31.46957664	Bcer	J	Bcer	FALSE	NA	NA	NA
Bc_ctg1_1428	B4	7.365220064	Bcer	S	Bcer	FALSE	NA	NA	NA
Bc_ctg1_1429	B4	4.798552466	Bcer	M	Bcer	FALSE	NA	NA	NA
Bc_ctg1_143	B4	1.339130921	Bcer	V	Bcer	FALSE	NA	NA	NA
Bc_ctg1_1430	B4	733.2857733	Bcer	D	Bcer	FALSE	NA	NA	NA
Bc_ctg1_1431	B4	542.9059941	Bcer	D	Bcer	FALSE	NA	NA	NA
Bc_ctg1_1432	B4	251.6450188	Bcer	M	Bcer	FALSE	NA	NA	NA
Bc_ctg1_1433	B4	602.943697	Bcer	M	Bcer	FALSE	NA	NA	NA
Bc_ctg1_1434	B4	349.847953	Bcer	D	Bcer	FALSE	NA	NA	NA
Bc_ctg1_1435	B4	29.68406874	Bcer	L	Bcer	FALSE	NA	NA	NA
Bc_ctg1_1437	B4	3.236233058	Bcer	E	Bcer	FALSE	NA	NA	NA
Bc_ctg1_1438	B4	24.10435657	Bcer	D	Bcer	FALSE	NA	NA	NA
Bc_ctg1_1439	B4	1.004348191	Bcer	NA	Bcer	FALSE	NA	NA	NA
Bc_ctg1_144	B4	0.33478273	Bcer	V	Bcer	FALSE	NA	NA	NA
Bc_ctg1_1440	B4	64.16668995	Bcer	H	Bcer	FALSE	NA	NA	NA
Bc_ctg1_1441	B4	815.8655134	Bcer	J	Bcer	FALSE	NA	NA	NA
Bc_ctg1_1442	B4	5.133335196	Bcer	S	Bcer	FALSE	NA	NA	NA
Bc_ctg1_1443	B4	0.223188487	Bcer	M	Bcer	FALSE	NA	NA	NA
Bc_ctg1_1444	B4	431.6465334	Bcer	H	Bcer	FALSE	NA	NA	NA
Bc_ctg1_1445	B4	180.7826743	Bcer	H	Bcer	FALSE	NA	NA	NA
Bc_ctg1_1446	B4	62.82755903	Bcer	H	Bcer	FALSE	NA	NA	NA
Bc_ctg1_1447	B4	142.7290373	Bcer	H	Bcer	FALSE	NA	NA	NA
Bc_ctg1_1448	B4	47.42755344	Bcer	O	Bcer	FALSE	NA	NA	NA
Bc_ctg1_1449	B4	228.9913874	Bcer	H	Bcer	FALSE	NA	NA	NA
Bc_ctg1_145	B4	0.892753947	Bcer	T	Bcer	FALSE	NA	NA	NA
Bc_ctg1_1450	B4	12.72174375	Bcer	K	Bcer	FALSE	NA	NA	NA
Bc_ctg1_1451	B4	40.39711611	Bcer	O	Bcer	FALSE	NA	NA	NA
Bc_ctg1_1452	B4	265.929082	Bcer	D	Bcer	FALSE	NA	NA	NA
Bc_ctg1_1453	B4	730.7191057	Bcer	O	Bcer	FALSE	NA	NA	NA
Bc_ctg1_1454	B4	42.62900098	Bcer	LO	Bcer	FALSE	NA	NA	NA
Bc_ctg1_1455	B4	2734.50534	Bcer	O	Bcer	FALSE	NA	NA	NA
Bc_ctg1_1456	B4	2555.284985	Bcer	D	Bcer	FALSE	NA	NA	NA
Bc_ctg1_1457	B4	271.1740114	Bcer	O	Bcer	FALSE	NA	NA	NA
Bc_ctg1_1458	B4	8.034785524	Bcer	NA	Bcer	FALSE	NA	NA	NA
Bc_ctg1_146	B4	0.111594243	Bcer	T	Bcer	FALSE	NA	NA	NA
Bc_ctg1_1460	B4	98.31452843	Bcer	S	Bcer	FALSE	NA	NA	NA
Bc_ctg1_1461	B4	1.897102138	Bcer	NA	Bcer	FALSE	NA	NA	NA
Bc_ctg1_1462	B4	1.115942434	Bcer	NA	Bcer	FALSE	NA	NA	NA
Bc_ctg1_1463	B4	81.68698616	Bcer	S	Bcer	FALSE	NA	NA	NA
Bc_ctg1_1464	B4	103.8942406	Bcer	F	Bcer	FALSE	NA	NA	NA
Bc_ctg1_1465	B4	45.86523403	Bcer	J	Bcer	FALSE	NA	NA	NA
Bc_ctg1_1466	B4	2.901450328	Bcer	S	Bcer	FALSE	NA	NA	NA
Bc_ctg1_1467	B4	94.85510688	Bcer	M	Bcer	FALSE	NA	NA	NA
Bc_ctg1_1468	B4	2.566667598	Bcer	EGP	Bcer	FALSE	NA	NA	NA
Bc_ctg1_1469	B4	5.244929439	Bcer	S	Bcer	FALSE	NA	NA	NA
Bc_ctg1_147	B4	2.455073355	Bcer	V	Bcer	FALSE	NA	NA	NA
Bc_ctg1_1470	B4	3.124638815	Bcer	K	Bcer	FALSE	NA	NA	NA
Bc_ctg1_1471	B4	0	Bcer	S	Bcer	FALSE	NA	NA	NA

Bc_ctg1_1472	B4	0.892753947	Bcer	K	Bcer	FALSE	NA	NA	NA
Bc_ctg1_1473	B4	9.373916445	Bcer	F	Bcer	FALSE	NA	NA	NA
Bc_ctg1_1474	B4	23.88116809	Bcer	F	Bcer	FALSE	NA	NA	NA
Bc_ctg1_1475	B4	1.227536677	Bcer	KT	Bcer	FALSE	NA	NA	NA
Bc_ctg1_1476	B4	2.566667598	Bcer	K	Bcer	FALSE	NA	NA	NA
Bc_ctg1_1477	B4	4.240581249	Bcer	EG	Bcer	FALSE	NA	NA	NA
Bc_ctg1_1478	B4	6.91884309	Bcer	G	Bcer	FALSE	NA	NA	NA
Bc_ctg1_1479	B4	71.19712728	Bcer	K	Bcer	FALSE	NA	NA	NA
Bc_ctg1_148	B4	2.343479111	Bcer	S	Bcer	FALSE	NA	NA	NA
Bc_ctg1_1480	B4	48.65509012	Bcer	CP	Bcer	FALSE	NA	NA	NA
Bc_ctg1_1481	B4	13.16812072	Bcer	S	Bcer	FALSE	NA	NA	NA
Bc_ctg1_1482	B4	74.3217661	Bcer	M	Bcer	FALSE	NA	NA	NA
Bc_ctg1_1483	B4	230.6653011	Bcer	N	Bcer	FALSE	NA	NA	NA
Bc_ctg1_1484	B4	246.8464664	Bcer	N	Bcer	FALSE	NA	NA	NA
Bc_ctg1_1485	B4	28.79131479	Bcer	M	Bcer	FALSE	NA	NA	NA
Bc_ctg1_1486	B4	39.50436216	Bcer	S	Bcer	FALSE	NA	NA	NA
Bc_ctg1_1487	B4	1686.5238	Bcer	C	Bcer	FALSE	NA	NA	NA
Bc_ctg1_1488	B4	3816.746312	Bcer	C	Bcer	FALSE	NA	NA	NA
Bc_ctg1_1489	B4	669.7886488	Bcer	C	Bcer	FALSE	NA	NA	NA
Bc_ctg1_149	B4	41.06668157	Bcer	T	Bcer	FALSE	NA	NA	NA
Bc_ctg1_1490	B4	97.31018024	Bcer	S	Bcer	FALSE	NA	NA	NA
Bc_ctg1_1491	B4	71.30872153	Bcer	L	Bcer	FALSE	NA	NA	NA
Bc_ctg1_1492	B4	108.0232276	Bcer	O	Bcer	FALSE	NA	NA	NA
Bc_ctg1_1493	B4	672.2437222	Bcer	C	Bcer	FALSE	NA	NA	NA
Bc_ctg1_1494	B4	526.7248288	Bcer	C	Bcer	FALSE	NA	NA	NA
Bc_ctg1_1495	B4	494.4740925	Bcer	I	Bcer	FALSE	NA	NA	NA
Bc_ctg1_1496	B4	369.3769456	Bcer	K	Bcer	FALSE	NA	NA	NA
Bc_ctg1_1497	B4	48.98987285	Bcer	IQ	Bcer	TRUE	NA	NA	NA
Bc_ctg1_1498	B4	0.111594243	Bcer	Q	Bcer	TRUE	NA	NA	NA
Bc_ctg1_1499	B4	41.84784127	Bcer	P	Bcer	FALSE	NA	NA	NA
Bc_ctg1_15	B4	0	Bcer	V	Bcer	FALSE	NA	NA	NA
Bc_ctg1_150	B4	12.16377253	Bcer	C	Bcer	FALSE	NA	NA	NA
Bc_ctg1_1500	B4	24.99711052	Bcer	P	Bcer	FALSE	NA	NA	NA
Bc_ctg1_1501	B4	3.794204275	Bcer	NA	Bcer	FALSE	NA	NA	NA
Bc_ctg1_1502	B4	76.66524521	Bcer	S	Bcer	FALSE	NA	NA	NA
Bc_ctg1_1503	B4	7.923191281	Bcer	NA	Bcer	FALSE	NA	NA	NA
Bc_ctg1_1504	B4	22.65363141	Bcer	S	Bcer	FALSE	NA	NA	NA
Bc_ctg1_1505	B4	20.42174654	Bcer	S	Bcer	FALSE	NA	NA	NA
Bc_ctg1_1506	B4	20.19855805	Bcer	S	Bcer	FALSE	NA	NA	NA
Bc_ctg1_1507	B4	1.115942434	Bcer	NA	Bcer	FALSE	NA	NA	NA
Bc_ctg1_1508	B4	16.40435378	Bcer	V	Bcer	FALSE	NA	NA	NA
Bc_ctg1_1509	B4	59.144949	Bcer	T	Bcer	FALSE	NA	NA	NA
Bc_ctg1_151	B4	5.57971217	Bcer	EGP	Bcer	FALSE	NA	NA	NA
Bc_ctg1_1510	B4	52.11451166	Bcer	T	Bcer	FALSE	NA	NA	NA
Bc_ctg1_1511	B4	1.673913651	Bcer	NA	Bcer	FALSE	NA	NA	NA
Bc_ctg1_1512	B4	0.446376974	Bcer	S	Bcer	FALSE	NA	NA	NA
Bc_ctg1_1513	B4	67.84929998	Bcer	J	Bcer	FALSE	NA	NA	NA
Bc_ctg1_1514	B4	6.24927763	Bcer	C	Bcer	FALSE	NA	NA	NA
Bc_ctg1_1515	B4	2.231884868	Bcer	S	Bcer	FALSE	NA	NA	NA
Bc_ctg1_1516	B4	4.128987005	Bcer	HP	Bcer	FALSE	NA	NA	NA
Bc_ctg1_1517	B4	2.901450328	Bcer	P	Bcer	FALSE	NA	NA	NA
Bc_ctg1_1518	B4	0.892753947	Bcer	P	Bcer	FALSE	NA	NA	NA
Bc_ctg1_1519	B4	11.71739556	Bcer	M	Bcer	FALSE	NA	NA	NA
Bc_ctg1_1520	B4	4.798552466	Bcer	M	Bcer	FALSE	NA	NA	NA
Bc_ctg1_1521	B4	7.923191281	Bcer	M	Bcer	FALSE	NA	NA	NA
Bc_ctg1_1522	B4	46.42320525	Bcer	E	Bcer	FALSE	NA	NA	NA
Bc_ctg1_1523	B4	7.588408551	Bcer	J	Bcer	FALSE	NA	NA	NA
Bc_ctg1_1525	B4	300.9696744	Bcer	L	Bcer	FALSE	NA	NA	NA
Bc_ctg1_1526	B4	111.3710549	Bcer	L	Bcer	FALSE	NA	NA	NA
Bc_ctg1_1527	B4	78.4507531	Bcer	S	Bcer	FALSE	NA	NA	NA
Bc_ctg1_1528	B4	32.47392483	Bcer	D	Bcer	FALSE	NA	NA	NA
Bc_ctg1_1529	B4	24.77392203	Bcer	L	Bcer	FALSE	NA	NA	NA

Bc_ctg1_153	B4	39.95073913	Bcer	EU	Bcer	FALSE	NA	NA	NA
Bc_ctg1_1530	B4	6.137683386	Bcer	NA	Bcer	FALSE	NA	NA	NA
Bc_ctg1_1531	B4	4.575363979	Bcer	NA	Bcer	FALSE	NA	NA	NA
Bc_ctg1_1533	B4	425.1740673	Bcer	J	Bcer	FALSE	NA	NA	NA
Bc_ctg1_1534	B4	1198.075797	Bcer	J	Bcer	FALSE	NA	NA	NA
Bc_ctg1_1535	B4	369.6001341	Bcer	J	Bcer	FALSE	NA	NA	NA
Bc_ctg1_1536	B4	90.2797429	Bcer	J	Bcer	FALSE	NA	NA	NA
Bc_ctg1_1538	B4	28.56812631	Bcer	S	Bcer	FALSE	NA	NA	NA
Bc_ctg1_1539	B4	14.50725164	Bcer	S	Bcer	FALSE	NA	NA	NA
Bc_ctg1_154	B4	1.004348191	Bcer	NA	Bcer	FALSE	NA	NA	NA
Bc_ctg1_1540	B4	6.026089143	Bcer	NA	Bcer	FALSE	NA	NA	NA
Bc_ctg1_1541	B4	6.472466117	Bcer	NA	Bcer	FALSE	NA	NA	NA
Bc_ctg1_1542	B4	319.0479419	Bcer	EGP	Bcer	FALSE	NA	NA	NA
Bc_ctg1_1543	B4	57.02465837	Bcer	V	Bcer	FALSE	NA	NA	NA
Bc_ctg1_1544	B4	12.83333799	Bcer	K	Bcer	FALSE	NA	NA	NA
Bc_ctg1_1545	B4	1182.787386	Bcer	G	Bcer	FALSE	NA	NA	NA
Bc_ctg1_1546	B4	176.3189046	Bcer	S	Bcer	FALSE	NA	NA	NA
Bc_ctg1_1547	B4	424.3929076	Bcer	J	Bcer	FALSE	NA	NA	NA
Bc_ctg1_1548	B4	226.2015314	Bcer	J	Bcer	FALSE	NA	NA	NA
Bc_ctg1_1549	B4	768.9959312	Bcer	J	Bcer	FALSE	NA	NA	NA
Bc_ctg1_1550	B4	2852.57205	Bcer	J	Bcer	FALSE	NA	NA	NA
Bc_ctg1_1551	B4	0.33478273	Bcer	S	Bcer	FALSE	NA	NA	NA
Bc_ctg1_1552	B4	0.892753947	Bcer	S	Bcer	FALSE	NA	NA	NA
Bc_ctg1_1553	B4	0.223188487	Bcer	L	Bcer	FALSE	NA	NA	NA
Bc_ctg1_1554	B4	1.227536677	Bcer	L	Bcer	FALSE	NA	NA	NA
Bc_ctg1_1555	B4	70.30437334	Bcer	L	Bcer	FALSE	NA	NA	NA
Bc_ctg1_1556	B4	47.76233617	Bcer	L	Bcer	FALSE	NA	NA	NA
Bc_ctg1_1557	B4	22.09566019	Bcer	K	Bcer	FALSE	NA	NA	NA
Bc_ctg1_1558	B4	219.5058767	Bcer	E	Bcer	FALSE	NA	NA	NA
Bc_ctg1_1559	B4	1899.445617	Bcer	G	Bcer	FALSE	NA	NA	NA
Bc_ctg1_156	B4	45.86523403	Bcer	E	Bcer	FALSE	NA	NA	NA
Bc_ctg1_1560	B4	15.62319407	Bcer	F	Bcer	FALSE	NA	NA	NA
Bc_ctg1_1561	B4	90.16814866	Bcer	P	Bcer	FALSE	NA	NA	NA
Bc_ctg1_1562	B4	208.0116697	Bcer	L	Bcer	FALSE	NA	NA	NA
Bc_ctg1_1563	B4	397.3871007	Bcer	L	Bcer	FALSE	NA	NA	NA
Bc_ctg1_1564	B4	150.4290401	Bcer	T	Bcer	FALSE	NA	NA	NA
Bc_ctg1_1565	B4	87.82466955	Bcer	T	Bcer	FALSE	NA	NA	NA
Bc_ctg1_1566	B4	0	Bcer	NA	Bcer	FALSE	NA	NA	NA
Bc_ctg1_1567	B4	0	Bcer	NA	Bcer	FALSE	NA	NA	NA
Bc_ctg1_1568	B4	46.64639374	Bcer	I	Bcer	FALSE	NA	NA	NA
Bc_ctg1_1569	B4	1735.402079	Bcer	C	Bcer	FALSE	NA	NA	NA
Bc_ctg1_157	B4	15.73478832	Bcer	S	Bcer	FALSE	NA	NA	NA
Bc_ctg1_1570	B4	2551.825564	Bcer	C	Bcer	FALSE	NA	NA	NA
Bc_ctg1_1571	B4	1681.836842	Bcer	C	Bcer	FALSE	NA	NA	NA
Bc_ctg1_1572	B4	5.57971217	Bcer	S	Bcer	FALSE	NA	NA	NA
Bc_ctg1_1573	B4	121.7493195	Bcer	S	Bcer	FALSE	NA	NA	NA
Bc_ctg1_1574	B4	3.682610032	Bcer	S	Bcer	FALSE	NA	NA	NA
Bc_ctg1_1575	B4	2687.412569	Bcer	G	Bcer	FALSE	NA	NA	NA
Bc_ctg1_1576	B4	878.469884	Bcer	F	Bcer	FALSE	NA	NA	NA
Bc_ctg1_1577	B4	323.0653346	Bcer	I	Bcer	FALSE	NA	NA	NA
Bc_ctg1_1578	B4	316.5928685	Bcer	I	Bcer	FALSE	NA	NA	NA
Bc_ctg1_1579	B4	358.552304	Bcer	K	Bcer	FALSE	NA	NA	NA
Bc_ctg1_158	B4	6.695654603	Bcer	S	Bcer	FALSE	NA	NA	NA
Bc_ctg1_1580	B4	1092.061266	Bcer	C	Bcer	FALSE	NA	NA	NA
Bc_ctg1_1581	B4	236.3566075	Bcer	L	Bcer	FALSE	NA	NA	NA
Bc_ctg1_1582	B4	0	Bcer	S	Bcer	FALSE	NA	NA	NA
Bc_ctg1_1583	B4	0.557971217	Bcer	NA	Bcer	FALSE	NA	NA	NA
Bc_ctg1_1584	B4	141.6130949	Bcer	S	Bcer	FALSE	NA	NA	NA
Bc_ctg1_1585	B4	2.120290624	Bcer	S	Bcer	FALSE	NA	NA	NA
Bc_ctg1_1586	B4	7.030437334	Bcer	S	Bcer	FALSE	NA	NA	NA
Bc_ctg1_1587	B4	111.9290261	Bcer	K	Bcer	FALSE	NA	NA	NA
Bc_ctg1_1588	B4	0.111594243	Bcer	NA	Bcer	FALSE	NA	NA	NA

Bc_ctg1_1589	B4	114.9420707	Bcer	S	Bcer	FALSE	NA	NA	NA
Bc_ctg1_159	B4	0	Bcer	S	Bcer	FALSE	NA	NA	NA
Bc_ctg1_1590	B4	282.6682185	Bcer	E	Bcer	FALSE	NA	NA	NA
Bc_ctg1_1591	B4	3.013044572	Bcer	S	Bcer	FALSE	NA	NA	NA
Bc_ctg1_1592	B4	1.562319407	Bcer	NA	Bcer	FALSE	NA	NA	NA
Bc_ctg1_1593	B4	5.244929439	Bcer	S	Bcer	FALSE	NA	NA	NA
Bc_ctg1_1594	B4	0	Bcer	NA	Bcer	FALSE	NA	NA	NA
Bc_ctg1_1595	B4	2.008696381	Bcer	NA	Bcer	FALSE	NA	NA	NA
Bc_ctg1_1596	B4	6.58406036	Bcer	J	Bcer	FALSE	NA	NA	NA
Bc_ctg1_1597	B4	2.678261841	Bcer	G	Bcer	FALSE	NA	NA	NA
Bc_ctg1_1598	B4	1859.718066	Bcer	E	Bcer	FALSE	NA	NA	NA
Bc_ctg1_1599	B4	54.0116138	Bcer	IQ	Bcer	TRUE	NA	NA	NA
Bc_ctg1_16	B4	1.673913651	Bcer	U	Bcer	FALSE	NA	NA	NA
Bc_ctg1_160	B4	5.244929439	Bcer	Q	Bcer	TRUE	NA	NA	NA
Bc_ctg1_1600	B4	539.7813553	Bcer	T	Bcer	FALSE	NA	NA	NA
Bc_ctg1_1601	B4	18.63623865	Bcer	NA	Bcer	FALSE	NA	NA	NA
Bc_ctg1_1602	B4	0.66956546	Bcer	NA	Bcer	FALSE	NA	NA	NA
Bc_ctg1_1603	B4	126.4362778	Bcer	E	Bcer	FALSE	NA	NA	NA
Bc_ctg1_1604	B4	100.881196	Bcer	E	Bcer	FALSE	NA	NA	NA
Bc_ctg1_1605	B4	11.38261283	Bcer	NA	Bcer	FALSE	NA	NA	NA
Bc_ctg1_1606	B4	0.557971217	Bcer	NA	Bcer	FALSE	NA	NA	NA
Bc_ctg1_1608	B4	390.1334749	Bcer	S	Bcer	FALSE	NA	NA	NA
Bc_ctg1_1609	B4	70.9739388	Bcer	S	Bcer	FALSE	NA	NA	NA
Bc_ctg1_161	B4	0.446376974	Bcer	E	Bcer	FALSE	NA	NA	NA
Bc_ctg1_1610	B4	0.33478273	Bcer	S	Bcer	FALSE	NA	NA	NA
Bc_ctg1_1611	B4	1.227536677	Bcer	F	Bcer	FALSE	NA	NA	NA
Bc_ctg1_1612	B4	264.5899511	Bcer	F	Bcer	FALSE	NA	NA	NA
Bc_ctg1_1613	B4	14.84203437	Bcer	L	Bcer	FALSE	NA	NA	NA
Bc_ctg1_1614	B4	403.3015956	Bcer	O	Bcer	FALSE	NA	NA	NA
Bc_ctg1_1615	B4	0.111594243	Bcer	S	Bcer	FALSE	NA	NA	NA
Bc_ctg1_1616	B4	0	Bcer	S	Bcer	FALSE	NA	NA	NA
Bc_ctg1_1617	B4	988.055431	Bcer	G	Bcer	FALSE	NA	NA	NA
Bc_ctg1_1618	B4	18.74783289	Bcer	S	Bcer	FALSE	NA	NA	NA
Bc_ctg1_1619	B4	45.08407433	Bcer	S	Bcer	FALSE	NA	NA	NA
Bc_ctg1_162	B4	418.14363	Bcer	L	Bcer	FALSE	NA	NA	NA
Bc_ctg1_1620	B4	5.9144949	Bcer	I	Bcer	FALSE	NA	NA	NA
Bc_ctg1_1621	B4	22.76522565	Bcer	S	Bcer	FALSE	NA	NA	NA
Bc_ctg1_1622	B4	258.0058907	Bcer	H	Bcer	FALSE	NA	NA	NA
Bc_ctg1_1623	B4	125.9899008	Bcer	E	Bcer	FALSE	NA	NA	NA
Bc_ctg1_1624	B4	426.7363867	Bcer	D	Bcer	FALSE	NA	NA	NA
Bc_ctg1_1625	B4	2.343479111	Bcer	K	Bcer	FALSE	NA	NA	NA
Bc_ctg1_1626	B4	0.66956546	Bcer	EG	Bcer	FALSE	NA	NA	NA
Bc_ctg1_1627	B4	45.75363979	Bcer	K	Bcer	FALSE	NA	NA	NA
Bc_ctg1_1628	B4	29.79566299	Bcer	T	Bcer	FALSE	NA	NA	NA
Bc_ctg1_1629	B4	16.40435378	Bcer	E	Bcer	FALSE	NA	NA	NA
Bc_ctg1_163	B4	60.59567416	Bcer	V	Bcer	FALSE	NA	NA	NA
Bc_ctg1_1630	B4	1909.600693	Bcer	J	Bcer	FALSE	NA	NA	NA
Bc_ctg1_1631	B4	2.120290624	Bcer	S	Bcer	FALSE	NA	NA	NA
Bc_ctg1_1632	B4	0.223188487	Bcer	NA	Bcer	FALSE	NA	NA	NA
Bc_ctg1_1633	B4	167.7261478	Bcer	J	Bcer	FALSE	NA	NA	NA
Bc_ctg1_1634	B4	3.905798519	Bcer	NA	Bcer	FALSE	NA	NA	NA
Bc_ctg1_1635	B4	35.26378091	Bcer	K	Bcer	FALSE	NA	NA	NA
Bc_ctg1_1636	B4	17.7434847	Bcer	NA	Bcer	FALSE	NA	NA	NA
Bc_ctg1_1637	B4	203.6594942	Bcer	S	Bcer	FALSE	NA	NA	NA
Bc_ctg1_1638	B4	110.478301	Bcer	K	Bcer	FALSE	NA	NA	NA
Bc_ctg1_1639	B4	407.6537711	Bcer	I	Bcer	FALSE	NA	NA	NA
Bc_ctg1_164	B4	171.8551348	Bcer	V	Bcer	FALSE	NA	NA	NA
Bc_ctg1_1640	B4	102.3319212	Bcer	K	Bcer	FALSE	NA	NA	NA
Bc_ctg1_1641	B4	101.8855442	Bcer	S	Bcer	FALSE	NA	NA	NA
Bc_ctg1_1642	B4	100.1000363	Bcer	BQ	Bcer	TRUE	NA	NA	NA
Bc_ctg1_1643	B4	3.347827302	Bcer	I	Bcer	FALSE	NA	NA	NA
Bc_ctg1_1644	B4	6.026089143	Bcer	NA	Bcer	FALSE	NA	NA	NA

Bc_ctg1_1645	B4	61.15364538	Bcer	T	Bcer	FALSE	NA	NA	NA
Bc_ctg1_1646	B4	68.74205393	Bcer	T	Bcer	FALSE	NA	NA	NA
Bc_ctg1_1647	B4	0.33478273	Bcer	S	Bcer	FALSE	NA	NA	NA
Bc_ctg1_1648	B4	118.4014922	Bcer	S	Bcer	FALSE	NA	NA	NA
Bc_ctg1_1649	B4	65.95219784	Bcer	M	Bcer	FALSE	NA	NA	NA
Bc_ctg1_165	B4	17.63189046	Bcer	K	Bcer	FALSE	NA	NA	NA
Bc_ctg1_1650	B4	10.26667039	Bcer	S	Bcer	FALSE	NA	NA	NA
Bc_ctg1_1651	B4	1.673913651	Bcer	S	Bcer	FALSE	NA	NA	NA
Bc_ctg1_1652	B4	9.820293418	Bcer	S	Bcer	FALSE	NA	NA	NA
Bc_ctg1_1653	B4	240.5971888	Bcer	K	Bcer	FALSE	NA	NA	NA
Bc_ctg1_1654	B4	2.343479111	Bcer	E	Bcer	FALSE	NA	NA	NA
Bc_ctg1_1655	B4	0.223188487	Bcer	E	Bcer	FALSE	NA	NA	NA
Bc_ctg1_1657	B4	147.5275898	Bcer	S	Bcer	FALSE	NA	NA	NA
Bc_ctg1_1658	B4	59.70292021	Bcer	O	Bcer	FALSE	NA	NA	NA
Bc_ctg1_1659	B4	64.27828419	Bcer	NA	Bcer	FALSE	NA	NA	NA
Bc_ctg1_166	B4	2.789856085	Bcer	G	Bcer	FALSE	NA	NA	NA
Bc_ctg1_1660	B4	822.2263853	Bcer	E	Bcer	FALSE	NA	NA	NA
Bc_ctg1_1661	B4	12.38696102	Bcer	S	Bcer	FALSE	NA	NA	NA
Bc_ctg1_1662	B4	6.026089143	Bcer	K	Bcer	FALSE	NA	NA	NA
Bc_ctg1_1663	B4	4.798552466	Bcer	J	Bcer	FALSE	NA	NA	NA
Bc_ctg1_1664	B4	3.013044572	Bcer	EG	Bcer	FALSE	NA	NA	NA
Bc_ctg1_1665	B4	0.446376974	Bcer	K	Bcer	FALSE	NA	NA	NA
Bc_ctg1_1666	B4	883.6032192	Bcer	M	Bcer	FALSE	NA	NA	NA
Bc_ctg1_1667	B4	615.3306581	Bcer	H	Bcer	FALSE	NA	NA	NA
Bc_ctg1_1668	B4	695.3437306	Bcer	D	Bcer	FALSE	NA	NA	NA
Bc_ctg1_1669	B4	2.231884868	Bcer	M	Bcer	FALSE	Bc_Ga0417192_01_rn12	Other	NA
Bc_ctg1_167	B4	4.575363979	Bcer	S	Bcer	FALSE	NA	NA	NA
Bc_ctg1_1670	B4	0	Bcer	NA	Bcer	FALSE	Bc_Ga0417192_01_rn12	Other	NA
Bc_ctg1_1671	B4	228.9913874	Bcer	J	Bcer	FALSE	Bc_Ga0417192_01_rn12	Other	NA
Bc_ctg1_1672	B4	163.8203493	Bcer	S	Bcer	FALSE	Bc_Ga0417192_01_rn12	Other	NA
Bc_ctg1_1673	B4	36.04494062	Bcer	CO	Bcer	FALSE	Bc_Ga0417192_01_rn12	Other	NA
Bc_ctg1_1674	B4	3.347827302	Bcer	F	Bcer	FALSE	Bc_Ga0417192_01_rn12	Other	NA
Bc_ctg1_1675	B4	326.5247562	Bcer	G	Bcer	FALSE	Bc_Ga0417192_01_rn12	Ancillary biosynthesis	None
Bc_ctg1_1676	B4	10.37826464	Bcer	S	Bcer	FALSE	Bc_Ga0417192_01_rn12	Other	NA
Bc_ctg1_1677	B4	28.90290904	Bcer	S	Bcer	FALSE	Bc_Ga0417192_01_rn12	Other	NA
Bc_ctg1_1678	B4	40.17392762	Bcer	J	Bcer	FALSE	Bc_Ga0417192_01_rn12	Other	NA
Bc_ctg1_1679	B4	0	Bcer	S	Bcer	FALSE	Bc_Ga0417192_01_rn12	Other	NA
Bc_ctg1_168	B4	31.91595361	Bcer	T	Bcer	FALSE	NA	NA	NA
Bc_ctg1_1680	B4	139.2696158	Bcer	M	Bcer	FALSE	Bc_Ga0417192_01_rn12	Ancillary biosynthesis	None
Bc_ctg1_1681	B4	22.31884868	Bcer	G	Bcer	FALSE	Bc_Ga0417192_01_rn12	Core biosynthesis	SMCOG1297
Bc_ctg1_1682	B4	4.798552466	Bcer	J	Bcer	FALSE	Bc_Ga0417192_01_rn12	Other	NA
Bc_ctg1_1683	B4	310.9015621	Bcer	E	Bcer	FALSE	Bc_Ga0417192_01_rn12	Ancillary biosynthesis	SMCOG1247
Bc_ctg1_1684	B4	241.6015369	Bcer	K	Bcer	FALSE	Bc_Ga0417192_01_rn12	Other	NA
Bc_ctg1_1685	B4	53.56523683	Bcer	J	Bcer	FALSE	Bc_Ga0417192_01_rn12	Ancillary biosynthesis	SMCOG1154
Bc_ctg1_1686	B4	320.05229	Bcer	S	Bcer	FALSE	Bc_Ga0417192_01_rn12	Core biosynthesis	SMCOG1086
Bc_ctg1_1687	B4	76.55365097	Bcer	S	Bcer	FALSE	Bc_Ga0417192_01_rn12	Other	NA
Bc_ctg1_1688	B4	100.7696018	Bcer	P	Bcer	FALSE	Bc_Ga0417192_01_rn12	Transport	SMCOG1005
Bc_ctg1_1689	B4	6.137683386	Bcer	K	Bcer	FALSE	Bc_Ga0417192_01_rn12	Regulation	SMCOG1058
Bc_ctg1_169	B4	10.60145312	Bcer	T	Bcer	FALSE	NA	NA	NA
Bc_ctg1_1690	B4	8.704350984	Bcer	S	Bcer	FALSE	Bc_Ga0417192_01_rn12	Other	NA
Bc_ctg1_1691	B4	0	Bcer	S	Bcer	FALSE	Bc_Ga0417192_01_rn12	Other	NA
Bc_ctg1_1693	B4	0	Bcer	S	Bcer	FALSE	Bc_Ga0417192_01_rn12	Other	NA
Bc_ctg1_1694	B4	412.5639178	Bcer	K	Bcer	FALSE	Bc_Ga0417192_01_rn12	Other	NA
Bc_ctg1_1695	B4	1.115942434	Bcer	IQ	Bcer	TRUE	Bc_Ga0417192_01_rn12	Ancillary biosynthesis	SMCOG1001
Bc_ctg1_1696	B4	0.111594243	Bcer	U	Bcer	FALSE	Bc_Ga0417192_01_rn12	Other	NA
Bc_ctg1_1697	B4	0	Bcer	S	Bcer	FALSE	Bc_Ga0417192_01_rn12	Other	NA
Bc_ctg1_1698	B4	3.124638815	Bcer	H	Bcer	FALSE	Bc_Ga0417192_01_rn12	Other	NA
Bc_ctg1_1699	B4	11.60580131	Bcer	H	Bcer	FALSE	Bc_Ga0417192_01_rn12	Other	NA
Bc_ctg1_17	B4	12.27536677	Bcer	M	Bcer	FALSE	NA	NA	NA
Bc_ctg1_170	B4	16.73913651	Bcer	S	Bcer	FALSE	NA	NA	NA
Bc_ctg1_1700	B4	39.28117367	Bcer	H	Bcer	FALSE	Bc_Ga0417192_01_rn12	Other	NA
Bc_ctg1_1701	B4	105.010183	Bcer	H	Bcer	FALSE	Bc_Ga0417192_01_rn12	Other	NA

Bc_ctg1_1702	B4	17.85507894	Bcer	H	Bcer	FALSE	NA	NA	NA
Bc_ctg1_1703	B4	48.43190163	Bcer	H	Bcer	FALSE	NA	NA	NA
Bc_ctg1_1704	B4	50.32900377	Bcer	H	Bcer	FALSE	NA	NA	NA
Bc_ctg1_1705	B4	8.704350984	Bcer	Q	Bcer	TRUE	NA	NA	NA
Bc_ctg1_1706	B4	6.137683386	Bcer	NA	Bcer	FALSE	NA	NA	NA
Bc_ctg1_1707	B4	1.562319407	Bcer	NA	Bcer	FALSE	NA	NA	NA
Bc_ctg1_1708	B4	4.352175492	Bcer	P	Bcer	FALSE	NA	NA	NA
Bc_ctg1_1709	B4	1.562319407	Bcer	NA	Bcer	FALSE	NA	NA	NA
Bc_ctg1_171	B4	32.69711331	Bcer	NA	Bcer	FALSE	NA	NA	NA
Bc_ctg1_1710	B4	6.58406036	Bcer	E	Bcer	FALSE	NA	NA	NA
Bc_ctg1_1711	B4	1.004348191	Bcer	P	Bcer	FALSE	NA	NA	NA
Bc_ctg1_1712	B4	0.781159704	Bcer	E	Bcer	FALSE	NA	NA	NA
Bc_ctg1_1713	B4	2.901450328	Bcer	S	Bcer	FALSE	NA	NA	NA
Bc_ctg1_1714	B4	1.897102138	Bcer	NA	Bcer	FALSE	NA	NA	NA
Bc_ctg1_1715	B4	68.29567696	Bcer	S	Bcer	FALSE	NA	NA	NA
Bc_ctg1_1716	B4	0	Bcer	NA	Bcer	FALSE	NA	NA	NA
Bc_ctg1_1718	B4	0.223188487	Bcer	NA	Bcer	FALSE	NA	NA	NA
Bc_ctg1_1719	B4	815.3075422	Bcer	J	Bcer	FALSE	NA	NA	NA
Bc_ctg1_172	B4	49.21306134	Bcer	V	Bcer	FALSE	NA	NA	NA
Bc_ctg1_1720	B4	18.18986167	Bcer	EGP	Bcer	FALSE	NA	NA	NA
Bc_ctg1_1721	B4	15.73478832	Bcer	P	Bcer	FALSE	NA	NA	NA
Bc_ctg1_1722	B4	12.6101495	Bcer	P	Bcer	FALSE	NA	NA	NA
Bc_ctg1_1723	B4	10.93623585	Bcer	L	Bcer	FALSE	NA	NA	NA
Bc_ctg1_1724	B4	91.84206231	Bcer	V	Bcer	FALSE	NA	NA	NA
Bc_ctg1_1725	B4	17.40870197	Bcer	V	Bcer	FALSE	NA	NA	NA
Bc_ctg1_1726	B4	1.897102138	Bcer	S	Bcer	FALSE	NA	NA	NA
Bc_ctg1_1728	B4	0	Bcer	S	Bcer	FALSE	NA	NA	NA
Bc_ctg1_1729	B4	26.89421266	Bcer	S	Bcer	FALSE	NA	NA	NA
Bc_ctg1_173	B4	14.17246891	Bcer	S	Bcer	FALSE	NA	NA	NA
Bc_ctg1_1730	B4	26.11305295	Bcer	M	Bcer	FALSE	NA	NA	NA
Bc_ctg1_1731	B4	11.71739556	Bcer	V	Bcer	FALSE	NA	NA	NA
Bc_ctg1_1732	B4	4.128987005	Bcer	NA	Bcer	FALSE	NA	NA	NA
Bc_ctg1_1733	B4	4.352175492	Bcer	M	Bcer	FALSE	NA	NA	NA
Bc_ctg1_1734	B4	10.26667039	Bcer	S	Bcer	FALSE	NA	NA	NA
Bc_ctg1_1735	B4	242.9406679	Bcer	S	Bcer	FALSE	NA	NA	NA
Bc_ctg1_1736	B4	36.60291183	Bcer	I	Bcer	FALSE	NA	NA	NA
Bc_ctg1_1737	B4	28.79131479	Bcer	S	Bcer	FALSE	NA	NA	NA
Bc_ctg1_1738	B4	0.223188487	Bcer	I	Bcer	FALSE	NA	NA	NA
Bc_ctg1_1739	B4	0.33478273	Bcer	M	Bcer	FALSE	NA	NA	NA
Bc_ctg1_174	B4	0	Bcer	I	Bcer	FALSE	NA	NA	NA
Bc_ctg1_1740	B4	29.5724745	Bcer	H	Bcer	FALSE	NA	NA	NA
Bc_ctg1_1741	B4	24.21595082	Bcer	H	Bcer	FALSE	NA	NA	NA
Bc_ctg1_1742	B4	1.115942434	Bcer	NA	Bcer	FALSE	NA	NA	NA
Bc_ctg1_1743	B4	1497.594746	Bcer	H	Bcer	FALSE	NA	NA	NA
Bc_ctg1_1744	B4	4571.569775	Bcer	C	Bcer	FALSE	NA	NA	NA
Bc_ctg1_1745	B4	0.223188487	Bcer	S	Bcer	FALSE	NA	NA	NA
Bc_ctg1_1746	B4	0	Bcer	NA	Bcer	FALSE	NA	NA	NA
Bc_ctg1_1747	B4	6.360871873	Bcer	EG	Bcer	FALSE	NA	NA	NA
Bc_ctg1_1748	B4	3.794204275	Bcer	S	Bcer	FALSE	NA	NA	NA
Bc_ctg1_1749	B4	10.04348191	Bcer	S	Bcer	FALSE	NA	NA	NA
Bc_ctg1_175	B4	340.1392539	Bcer	S	Bcer	FALSE	NA	NA	NA
Bc_ctg1_1750	B4	1.785507894	Bcer	Q	Bcer	TRUE	NA	NA	NA
Bc_ctg1_1751	B4	1.450725164	Bcer	S	Bcer	FALSE	NA	NA	NA
Bc_ctg1_1752	B4	1.115942434	Bcer	S	Bcer	FALSE	NA	NA	NA
Bc_ctg1_1753	B4	0.111594243	Bcer	S	Bcer	FALSE	NA	NA	NA
Bc_ctg1_1754	B4	0	Bcer	NA	Bcer	FALSE	NA	NA	NA
Bc_ctg1_1755	B4	1.562319407	Bcer	NU	Bcer	FALSE	NA	NA	NA
Bc_ctg1_1756	B4	18.5246444	Bcer	T	Bcer	FALSE	NA	NA	NA
Bc_ctg1_1757	B4	2.901450328	Bcer	T	Bcer	FALSE	NA	NA	NA
Bc_ctg1_1758	B4	0.557971217	Bcer	S	Bcer	FALSE	NA	NA	NA
Bc_ctg1_1759	B4	124.2043929	Bcer	M	Bcer	FALSE	NA	NA	NA
Bc_ctg1_176	B4	144.2913567	Bcer	EH	Bcer	FALSE	NA	NA	NA

Bc_ctg1_1760	B4	4.686958222	Bcer	NA	Bcer	FALSE	NA	NA	NA
Bc_ctg1_1761	B4	6.137683386	Bcer	S	Bcer	FALSE	NA	NA	NA
Bc_ctg1_1762	B4	2.343479111	Bcer	P	Bcer	FALSE	NA	NA	NA
Bc_ctg1_1763	B4	3.459421545	Bcer	P	Bcer	FALSE	NA	NA	NA
Bc_ctg1_1764	B4	4.686958222	Bcer	P	Bcer	FALSE	NA	NA	NA
Bc_ctg1_1765	B4	0	Bcer	NA	Bcer	FALSE	NA	NA	NA
Bc_ctg1_1766	B4	11.8289898	Bcer	NA	Bcer	FALSE	NA	NA	NA
Bc_ctg1_1767	B4	7.25362582	Bcer	S	Bcer	FALSE	NA	NA	NA
Bc_ctg1_1768	B4	8.146379768	Bcer	S	Bcer	FALSE	NA	NA	NA
Bc_ctg1_1769	B4	144.6261394	Bcer	L	Bcer	FALSE	NA	NA	NA
Bc_ctg1_177	B4	55.46233897	Bcer	E	Bcer	FALSE	NA	NA	NA
Bc_ctg1_1770	B4	0.557971217	Bcer	NA	Bcer	FALSE	NA	NA	NA
Bc_ctg1_1772	B4	0	Bcer	NA	Bcer	FALSE	NA	NA	NA
Bc_ctg1_1773	B4	0	Bcer	NA	Bcer	FALSE	NA	NA	NA
Bc_ctg1_1774	B4	138.0420791	Bcer	T	Bcer	FALSE	NA	NA	NA
Bc_ctg1_1775	B4	0.781159704	Bcer	S	Bcer	FALSE	NA	NA	NA
Bc_ctg1_1776	B4	113.8261283	Bcer	P	Bcer	FALSE	NA	NA	NA
Bc_ctg1_1777	B4	479.8552466	Bcer	C	Bcer	FALSE	NA	NA	NA
Bc_ctg1_1778	B4	273.6290848	Bcer	C	Bcer	FALSE	NA	NA	NA
Bc_ctg1_1779	B4	0	Bcer	S	Bcer	FALSE	NA	NA	NA
Bc_ctg1_178	B4	0	Bcer	S	Bcer	FALSE	NA	NA	NA
Bc_ctg1_1780	B4	11.0478301	Bcer	S	Bcer	FALSE	NA	NA	NA
Bc_ctg1_1781	B4	0.111594243	Bcer	S	Bcer	FALSE	NA	NA	NA
Bc_ctg1_1782	B4	49.99422104	Bcer	S	Bcer	FALSE	NA	NA	NA
Bc_ctg1_1783	B4	14.61884588	Bcer	NA	Bcer	FALSE	NA	NA	NA
Bc_ctg1_1784	B4	262.246472	Bcer	S	Bcer	FALSE	NA	NA	NA
Bc_ctg1_1785	B4	109.0275758	Bcer	S	Bcer	FALSE	NA	NA	NA
Bc_ctg1_1786	B4	36.37972335	Bcer	S	Bcer	FALSE	NA	NA	NA
Bc_ctg1_1787	B4	400.5117395	Bcer	KT	Bcer	FALSE	NA	NA	NA
Bc_ctg1_1788	B4	14.50725164	Bcer	S	Bcer	FALSE	NA	NA	NA
Bc_ctg1_179	B4	10.71304737	Bcer	G	Bcer	FALSE	NA	NA	NA
Bc_ctg1_1791	B4	0	Bcer	S	Bcer	FALSE	NA	NA	NA
Bc_ctg1_1792	B4	271.7319827	Bcer	P	Bcer	FALSE	NA	NA	NA
Bc_ctg1_1793	B4	15.62319407	Bcer	P	Bcer	FALSE	NA	NA	NA
Bc_ctg1_1794	B4	21.98406595	Bcer	S	Bcer	FALSE	NA	NA	NA
Bc_ctg1_1795	B4	85.92756741	Bcer	T	Bcer	FALSE	NA	NA	NA
Bc_ctg1_1796	B4	29.90725723	Bcer	T	Bcer	FALSE	NA	NA	NA
Bc_ctg1_1797	B4	43.85653765	Bcer	M	Bcer	FALSE	NA	NA	NA
Bc_ctg1_1798	B4	29.68406874	Bcer	NA	Bcer	FALSE	NA	NA	NA
Bc_ctg1_1799	B4	253.4305267	Bcer	NA	Bcer	FALSE	NA	NA	NA
Bc_ctg1_18	B4	22.8768199	Bcer	K	Bcer	FALSE	NA	NA	NA
Bc_ctg1_180	B4	1.339130921	Bcer	G	Bcer	FALSE	NA	NA	NA
Bc_ctg1_1800	B4	25.77827022	Bcer	NA	Bcer	FALSE	NA	NA	NA
Bc_ctg1_1801	B4	197.7449993	Bcer	F	Bcer	FALSE	NA	NA	NA
Bc_ctg1_1802	B4	154.5580271	Bcer	I	Bcer	FALSE	NA	NA	NA
Bc_ctg1_1803	B4	10.82464161	Bcer	S	Bcer	FALSE	NA	NA	NA
Bc_ctg1_1804	B4	11.49420707	Bcer	NA	Bcer	FALSE	NA	NA	NA
Bc_ctg1_1805	B4	14.3956574	Bcer	S	Bcer	FALSE	NA	NA	NA
Bc_ctg1_1806	B4	0.33478273	Bcer	NA	Bcer	FALSE	NA	NA	NA
Bc_ctg1_1807	B4	0.557971217	Bcer	NA	Bcer	FALSE	NA	NA	NA
Bc_ctg1_1808	B4	1.673913651	Bcer	S	Bcer	FALSE	NA	NA	NA
Bc_ctg1_1809	B4	1.227536677	Bcer	I	Bcer	FALSE	NA	NA	NA
Bc_ctg1_181	B4	1.785507894	Bcer	K	Bcer	FALSE	NA	NA	NA
Bc_ctg1_1810	B4	245.3957412	Bcer	V	Bcer	FALSE	NA	NA	NA
Bc_ctg1_1811	B4	66.28698057	Bcer	V	Bcer	FALSE	NA	NA	NA
Bc_ctg1_1812	B4	23.21160263	Bcer	K	Bcer	FALSE	NA	NA	NA
Bc_ctg1_1813	B4	19.52899259	Bcer	S	Bcer	FALSE	NA	NA	NA
Bc_ctg1_1814	B4	9.931887662	Bcer	S	Bcer	FALSE	NA	NA	NA
Bc_ctg1_1815	B4	2.008696381	Bcer	K	Bcer	FALSE	NA	NA	NA
Bc_ctg1_1816	B4	56.35509291	Bcer	K	Bcer	FALSE	NA	NA	NA
Bc_ctg1_1817	B4	102.5551097	Bcer	T	Bcer	FALSE	NA	NA	NA
Bc_ctg1_1818	B4	60.26089143	Bcer	V	Bcer	FALSE	NA	NA	NA

Bc_ctg1_1819	B4	40.84349308	Bcer	V	Bcer	FALSE	NA	NA	NA
Bc_ctg1_182	B4	6.360871873	Bcer	S	Bcer	FALSE	NA	NA	NA
Bc_ctg1_1820	B4	18.85942713	Bcer	V	Bcer	FALSE	NA	NA	NA
Bc_ctg1_1821	B4	58.81016627	Bcer	V	Bcer	FALSE	NA	NA	NA
Bc_ctg1_1823	B4	8.257974011	Bcer	EG	Bcer	FALSE	NA	NA	NA
Bc_ctg1_1824	B4	21.091312	Bcer	K	Bcer	FALSE	NA	NA	NA
Bc_ctg1_1825	B4	21.42609473	Bcer	G	Bcer	FALSE	NA	NA	NA
Bc_ctg1_1826	B4	18.18986167	Bcer	S	Bcer	FALSE	NA	NA	NA
Bc_ctg1_1827	B4	19.19420986	Bcer	E	Bcer	FALSE	NA	NA	NA
Bc_ctg1_1828	B4	8.034785524	Bcer	S	Bcer	FALSE	NA	NA	NA
Bc_ctg1_1829	B4	2.789856085	Bcer	M	Bcer	FALSE	NA	NA	NA
Bc_ctg1_183	B4	22.98841414	Bcer	C	Bcer	FALSE	NA	NA	NA
Bc_ctg1_1830	B4	26.67102417	Bcer	T	Bcer	FALSE	NA	NA	NA
Bc_ctg1_1831	B4	10.37826464	Bcer	T	Bcer	FALSE	NA	NA	NA
Bc_ctg1_1832	B4	7.923191281	Bcer	H	Bcer	FALSE	NA	NA	NA
Bc_ctg1_1833	B4	10.37826464	Bcer	H	Bcer	FALSE	NA	NA	NA
Bc_ctg1_1834	B4	893.3119183	Bcer	H	Bcer	FALSE	NA	NA	NA
Bc_ctg1_1835	B4	59.59132597	Bcer	S	Bcer	FALSE	NA	NA	NA
Bc_ctg1_1836	B4	163.4855666	Bcer	H	Bcer	FALSE	Bc_Ga0417192_01_rn13	Other	NA
Bc_ctg1_1837	B4	142.9522258	Bcer	HQ	Bcer	TRUE	Bc_Ga0417192_01_rn13	Ancillary biosynthesis	SMCOG1018
Bc_ctg1_1838	B4	19.52899259	Bcer	H	Bcer	FALSE	Bc_Ga0417192_01_rn13	Ancillary biosynthesis	None
Bc_ctg1_1839	B4	0.557971217	Bcer	T	Bcer	FALSE	Bc_Ga0417192_01_rn13	Other	NA
Bc_ctg1_184	B4	0.111594243	Bcer	L	Bcer	FALSE	NA	NA	NA
Bc_ctg1_1840	B4	163.0391896	Bcer	K	Bcer	FALSE	Bc_Ga0417192_01_rn13	Regulation	SMCOG1255
Bc_ctg1_1841	B4	97.08699175	Bcer	S	Bcer	FALSE	Bc_Ga0417192_01_rn13	Other	NA
Bc_ctg1_1842	B4	0.223188487	Bcer	S	Bcer	FALSE	Bc_Ga0417192_01_rn13	Other	NA
Bc_ctg1_1843	B4	3.682610032	Bcer	K	Bcer	FALSE	Bc_Ga0417192_01_rn13	Regulation	SMCOG1171
Bc_ctg1_1844	B4	0	Bcer	I	Bcer	FALSE	Bc_Ga0417192_01_rn13	Other	NA
Bc_ctg1_1845	B4	0	Bcer	NA	Bcer	FALSE	Bc_Ga0417192_01_rn13	Other	NA
Bc_ctg1_1846	B4	793.7698532	Bcer	G	Bcer	FALSE	Bc_Ga0417192_01_rn13	Other	NA
Bc_ctg1_1847	B4	351.6334609	Bcer	G	Bcer	FALSE	Bc_Ga0417192_01_rn13	Core biosynthesis	SMCOG1045
Bc_ctg1_1848	B4	380.53637	Bcer	G	Bcer	FALSE	Bc_Ga0417192_01_rn13	Ancillary biosynthesis	SMCOG1064
Bc_ctg1_1849	B4	365.1363644	Bcer	G	Bcer	FALSE	Bc_Ga0417192_01_rn13	Ancillary biosynthesis	SMCOG1064
Bc_ctg1_185	B4	30.13044572	Bcer	M	Bcer	FALSE	NA	NA	NA
Bc_ctg1_1850	B4	535.987151	Bcer	G	Bcer	FALSE	Bc_Ga0417192_01_rn13	Core biosynthesis	SMCOG1297
Bc_ctg1_1852	B4	6.137683386	Bcer	O	Bcer	FALSE	Bc_Ga0417192_01_rn13	Ancillary biosynthesis	SMCOG1075
Bc_ctg1_1853	B4	5518.44693	Bcer	C	Bcer	FALSE	Bc_Ga0417192_01_rn13	Other	NA
Bc_ctg1_1855	B4	12.94493223	Bcer	S	Bcer	FALSE	Bc_Ga0417192_01_rn13	Other	NA
Bc_ctg1_1856	B4	0.446376974	Bcer	P	Bcer	FALSE	Bc_Ga0417192_01_rn13	Other	NA
Bc_ctg1_1857	B4	2.343479111	Bcer	S	Bcer	FALSE	Bc_Ga0417192_01_rn13	Other	NA
Bc_ctg1_1858	B4	1366.136728	Bcer	G	Bcer	FALSE	Bc_Ga0417192_01_rn13	Other	NA
Bc_ctg1_1859	B4	2.789856085	Bcer	S	Bcer	FALSE	Bc_Ga0417192_01_rn13	Other	NA
Bc_ctg1_186	B4	1.339130921	Bcer	NA	Bcer	FALSE	NA	NA	NA
Bc_ctg1_1860	B4	56.35509291	Bcer	J	Bcer	FALSE	Bc_Ga0417192_01_rn13	Other	NA
Bc_ctg1_1861	B4	138.488456	Bcer	E	Bcer	FALSE	Bc_Ga0417192_01_rn13	Core biosynthesis	SMCOG1019
Bc_ctg1_1862	B4	4.240581249	Bcer	K	Bcer	FALSE	Bc_Ga0417192_01_rn13	Regulation	SMCOG1120
Bc_ctg1_1863	B4	144.5145452	Bcer	CH	Bcer	FALSE	Bc_Ga0417192_01_rn13	Ancillary biosynthesis	SMCOG1072
Bc_ctg1_1864	B4	0.223188487	Bcer	I	Bcer	FALSE	Bc_Ga0417192_01_rn13	Ancillary biosynthesis	SMCOG1262
Bc_ctg1_1865	B4	0.892753947	Bcer	S	Bcer	FALSE	Bc_Ga0417192_01_rn13	Other	NA
Bc_ctg1_1866	B4	60.59567416	Bcer	E	Bcer	FALSE	Bc_Ga0417192_01_rn13	Ancillary biosynthesis	SMCOG1019
Bc_ctg1_1867	B4	6.360871873	Bcer	P	Bcer	FALSE	Bc_Ga0417192_01_rn13	Other	NA
Bc_ctg1_1868	B4	0.781159704	Bcer	NA	Bcer	FALSE	Bc_Ga0417192_01_rn13	Other	NA
Bc_ctg1_1869	B4	43.74494341	Bcer	G	Bcer	FALSE	Bc_Ga0417192_01_rn13	Other	NA
Bc_ctg1_187	B4	115.8348246	Bcer	L	Bcer	FALSE	NA	NA	NA
Bc_ctg1_1870	B4	7.811597037	Bcer	L	Bcer	FALSE	Bc_Ga0417192_01_rn13	Other	NA
Bc_ctg1_1871	B4	50.44059801	Bcer	P	Bcer	FALSE	Bc_Ga0417192_01_rn13	Other	NA
Bc_ctg1_1872	B4	0.892753947	Bcer	NA	Bcer	FALSE	Bc_Ga0417192_01_rn13	Other	NA
Bc_ctg1_1873	B4	6.695654603	Bcer	L	Bcer	FALSE	Bc_Ga0417192_01_rn13	Other	NA
Bc_ctg1_1874	B4	609.7509459	Bcer	J	Bcer	FALSE	Bc_Ga0417192_01_rn13	Other	NA
Bc_ctg1_1875	B4	37.04928881	Bcer	Q	Bcer	TRUE	Bc_Ga0417192_01_rn13	Ancillary biosynthesis	SMCOG1144
Bc_ctg1_1876	B4	0	Bcer	S	Bcer	FALSE	Bc_Ga0417192_01_rn13	Other	NA
Bc_ctg1_1877	B4	12.16377253	Bcer	S	Bcer	FALSE	Bc_Ga0417192_01_rn13	Other	NA

Bc_ctg1_1878	B4	15.28841134	Bcer	S	Bcer	FALSE	Bc_Ga0417192_01_rn13	Other	NA
Bc_ctg1_1879	B4	279.9899567	Bcer	M	Bcer	FALSE	Bc_Ga0417192_01_rn13	Core biosynthesis	SMCOG1064
Bc_ctg1_188	B4	1.562319407	Bcer	NA	Bcer	FALSE	NA	NA	NA
Bc_ctg1_1880	B4	665.6596618	Bcer	G	Bcer	FALSE	Bc_Ga0417192_01_rn13	Other	NA
Bc_ctg1_1881	B4	0.446376974	Bcer	S	Bcer	FALSE	Bc_Ga0417192_01_rn13	Other	NA
Bc_ctg1_1882	B4	32.69711331	Bcer	NA	Bcer	FALSE	Bc_Ga0417192_01_rn13	Other	NA
Bc_ctg1_1883	B4	8.257974011	Bcer	S	Bcer	FALSE	Bc_Ga0417192_01_rn13	Other	NA
Bc_ctg1_1884	B4	3.905798519	Bcer	P	Bcer	FALSE	Bc_Ga0417192_01_rn13	Other	NA
Bc_ctg1_1885	B4	69.85799636	Bcer	H	Bcer	FALSE	Bc_Ga0417192_01_rn13	Ancillary biosynthesis	None
Bc_ctg1_1886	B4	1145.403314	Bcer	E	Bcer	FALSE	Bc_Ga0417192_01_rn13	Other	NA
Bc_ctg1_1887	B4	6.24927763	Bcer	S	Bcer	FALSE	Bc_Ga0417192_01_rn13	Other	NA
Bc_ctg1_1888	B4	14.95362861	Bcer	S	Bcer	FALSE	Bc_Ga0417192_01_rn13	Other	NA
Bc_ctg1_1889	B4	6.807248847	Bcer	F	Bcer	FALSE	Bc_Ga0417192_01_rn13	Other	NA
Bc_ctg1_189	B4	63.05074752	Bcer	M	Bcer	FALSE	NA	NA	NA
Bc_ctg1_1890	B4	26.2246472	Bcer	C	Bcer	FALSE	NA	NA	NA
Bc_ctg1_1891	B4	393.4813022	Bcer	C	Bcer	FALSE	NA	NA	NA
Bc_ctg1_1892	B4	38.053637	Bcer	M	Bcer	FALSE	NA	NA	NA
Bc_ctg1_1893	B4	19.41739835	Bcer	K	Bcer	FALSE	NA	NA	NA
Bc_ctg1_1894	B4	83.02611708	Bcer	EGP	Bcer	FALSE	NA	NA	NA
Bc_ctg1_1895	B4	0.66956546	Bcer	K	Bcer	FALSE	NA	NA	NA
Bc_ctg1_1896	B4	4.128987005	Bcer	S	Bcer	FALSE	NA	NA	NA
Bc_ctg1_1897	B4	9.708699175	Bcer	S	Bcer	FALSE	NA	NA	NA
Bc_ctg1_1898	B4	0.446376974	Bcer	S	Bcer	FALSE	NA	NA	NA
Bc_ctg1_1899	B4	0	Bcer	NA	Bcer	FALSE	NA	NA	NA
Bc_ctg1_19	B4	5.356523683	Bcer	O	Bcer	FALSE	NA	NA	NA
Bc_ctg1_190	B4	88.7174235	Bcer	M	Bcer	FALSE	NA	NA	NA
Bc_ctg1_1900	B4	5.691306413	Bcer	NA	Bcer	FALSE	NA	NA	NA
Bc_ctg1_1901	B4	10.26667039	Bcer	J	Bcer	FALSE	NA	NA	NA
Bc_ctg1_1902	B4	126.4362778	Bcer	S	Bcer	FALSE	NA	NA	NA
Bc_ctg1_1903	B4	52.22610591	Bcer	E	Bcer	FALSE	NA	NA	NA
Bc_ctg1_1904	B4	7.365220064	Bcer	S	Bcer	FALSE	NA	NA	NA
Bc_ctg1_1905	B4	435.7755204	Bcer	C	Bcer	FALSE	NA	NA	NA
Bc_ctg1_1906	B4	12.94493223	Bcer	V	Bcer	FALSE	NA	NA	NA
Bc_ctg1_1907	B4	5.57971217	Bcer	S	Bcer	FALSE	NA	NA	NA
Bc_ctg1_1908	B4	12.94493223	Bcer	P	Bcer	FALSE	NA	NA	NA
Bc_ctg1_1909	B4	3.124638815	Bcer	NU	Bcer	FALSE	NA	NA	NA
Bc_ctg1_191	B4	2.566667598	Bcer	S	Bcer	FALSE	NA	NA	NA
Bc_ctg1_1910	B4	1.115942434	Bcer	S	Bcer	FALSE	NA	NA	NA
Bc_ctg1_1911	B4	1.897102138	Bcer	NA	Bcer	FALSE	NA	NA	NA
Bc_ctg1_1912	B4	0.66956546	Bcer	S	Bcer	FALSE	NA	NA	NA
Bc_ctg1_1913	B4	1.673913651	Bcer	J	Bcer	FALSE	NA	NA	NA
Bc_ctg1_1914	B4	2.343479111	Bcer	S	Bcer	FALSE	NA	NA	NA
Bc_ctg1_1915	B4	77.78118764	Bcer	O	Bcer	FALSE	NA	NA	NA
Bc_ctg1_1916	B4	13.94928042	Bcer	NA	Bcer	FALSE	NA	NA	NA
Bc_ctg1_1917	B4	12.83333799	Bcer	S	Bcer	FALSE	NA	NA	NA
Bc_ctg1_1918	B4	47.20436495	Bcer	I	Bcer	FALSE	NA	NA	NA
Bc_ctg1_1919	B4	2.566667598	Bcer	J	Bcer	FALSE	NA	NA	NA
Bc_ctg1_192	B4	55.57393321	Bcer	EGP	Bcer	FALSE	NA	NA	NA
Bc_ctg1_1920	B4	6.472466117	Bcer	G	Bcer	FALSE	NA	NA	NA
Bc_ctg1_1921	B4	5.9144949	Bcer	F	Bcer	FALSE	NA	NA	NA
Bc_ctg1_1922	B4	4.575363979	Bcer	J	Bcer	FALSE	NA	NA	NA
Bc_ctg1_1923	B4	4.352175492	Bcer	S	Bcer	FALSE	NA	NA	NA
Bc_ctg1_1924	B4	15.28841134	Bcer	S	Bcer	FALSE	NA	NA	NA
Bc_ctg1_1925	B4	2.901450328	Bcer	NA	Bcer	FALSE	NA	NA	NA
Bc_ctg1_1926	B4	109.2507643	Bcer	G	Bcer	FALSE	NA	NA	NA
Bc_ctg1_1927	B4	20.86812351	Bcer	K	Bcer	FALSE	NA	NA	NA
Bc_ctg1_1928	B4	72.42466396	Bcer	S	Bcer	FALSE	NA	NA	NA
Bc_ctg1_1929	B4	156.343535	Bcer	G	Bcer	FALSE	NA	NA	NA
Bc_ctg1_193	B4	8.592756741	Bcer	NA	Bcer	FALSE	NA	NA	NA
Bc_ctg1_1930	B4	36.82610032	Bcer	S	Bcer	FALSE	NA	NA	NA
Bc_ctg1_1931	B4	0.66956546	Bcer	S	Bcer	FALSE	NA	NA	NA
Bc_ctg1_1932	B4	13.39130921	Bcer	S	Bcer	FALSE	NA	NA	NA

Bc_ctg1_1933	B4	0.111594243	Bcer	S	Bcer	FALSE	NA	NA	NA
Bc_ctg1_1934	B4	4.463769736	Bcer	K	Bcer	FALSE	NA	NA	NA
Bc_ctg1_1935	B4	71.7550985	Bcer	S	Bcer	FALSE	NA	NA	NA
Bc_ctg1_1936	B4	0.223188487	Bcer	NA	Bcer	FALSE	NA	NA	NA
Bc_ctg1_1937	B4	33.14349029	Bcer	G	Bcer	FALSE	NA	NA	NA
Bc_ctg1_1938	B4	24.55073355	Bcer	S	Bcer	FALSE	NA	NA	NA
Bc_ctg1_1939	B4	801.1350733	Bcer	H	Bcer	FALSE	NA	NA	NA
Bc_ctg1_194	B4	72.64785245	Bcer	I	Bcer	FALSE	NA	NA	NA
Bc_ctg1_1940	B4	0.111594243	Bcer	M	Bcer	FALSE	NA	NA	NA
Bc_ctg1_1941	B4	6.472466117	Bcer	S	Bcer	FALSE	NA	NA	NA
Bc_ctg1_1942	B4	12.72174375	Bcer	S	Bcer	FALSE	NA	NA	NA
Bc_ctg1_1943	B4	68.4072712	Bcer	F	Bcer	FALSE	NA	NA	NA
Bc_ctg1_1944	B4	1610.974498	Bcer	O	Bcer	FALSE	NA	NA	NA
Bc_ctg1_1945	B4	233.2319687	Bcer	C	Bcer	FALSE	NA	NA	NA
Bc_ctg1_1946	B4	973.7713678	Bcer	E	Bcer	FALSE	NA	NA	NA
Bc_ctg1_1947	B4	1033.2511	Bcer	O	Bcer	FALSE	NA	NA	NA
Bc_ctg1_1948	B4	521.9262763	Bcer	O	Bcer	FALSE	NA	NA	NA
Bc_ctg1_1949	B4	14.84203437	Bcer	P	Bcer	FALSE	NA	NA	NA
Bc_ctg1_195	B4	16.85073075	Bcer	K	Bcer	FALSE	NA	NA	NA
Bc_ctg1_1950	B4	108.4696046	Bcer	P	Bcer	FALSE	NA	NA	NA
Bc_ctg1_1951	B4	23.10000838	Bcer	P	Bcer	FALSE	NA	NA	NA
Bc_ctg1_1952	B4	33.92464999	Bcer	P	Bcer	FALSE	NA	NA	NA
Bc_ctg1_1953	B4	1.673913651	Bcer	NA	Bcer	FALSE	NA	NA	NA
Bc_ctg1_1954	B4	45.19566857	Bcer	CO	Bcer	FALSE	NA	NA	NA
Bc_ctg1_1955	B4	6.026089143	Bcer	L	Bcer	FALSE	NA	NA	NA
Bc_ctg1_1956	B4	1119.848232	Bcer	E	Bcer	FALSE	NA	NA	NA
Bc_ctg1_1957	B4	282.3334358	Bcer	P	Bcer	FALSE	NA	NA	NA
Bc_ctg1_1958	B4	1.339130921	Bcer	NA	Bcer	FALSE	NA	NA	NA
Bc_ctg1_1959	B4	20.97971776	Bcer	S	Bcer	FALSE	NA	NA	NA
Bc_ctg1_196	B4	34.92899818	Bcer	EGP	Bcer	FALSE	NA	NA	NA
Bc_ctg1_1960	B4	0.223188487	Bcer	NA	Bcer	FALSE	NA	NA	NA
Bc_ctg1_1961	B4	43.29856644	Bcer	S	Bcer	FALSE	NA	NA	NA
Bc_ctg1_1962	B4	14.17246891	Bcer	NA	Bcer	FALSE	NA	NA	NA
Bc_ctg1_1963	B4	14.3956574	Bcer	I	Bcer	FALSE	NA	NA	NA
Bc_ctg1_1964	B4	2898.660472	Bcer	S	Bcer	FALSE	NA	NA	NA
Bc_ctg1_1965	B4	3653.37234	Bcer	C	Bcer	FALSE	NA	NA	NA
Bc_ctg1_1966	B4	0	Bcer	M	Bcer	FALSE	NA	NA	NA
Bc_ctg1_1967	B4	0	Bcer	NA	Bcer	FALSE	NA	NA	NA
Bc_ctg1_1968	B4	5.468117926	Bcer	S	Bcer	FALSE	NA	NA	NA
Bc_ctg1_1969	B4	0	Bcer	NA	Bcer	FALSE	NA	NA	NA
Bc_ctg1_197	B4	161.7000587	Bcer	V	Bcer	FALSE	NA	NA	NA
Bc_ctg1_1970	B4	6.472466117	Bcer	K	Bcer	FALSE	NA	NA	NA
Bc_ctg1_1971	B4	8.927539471	Bcer	S	Bcer	FALSE	NA	NA	NA
Bc_ctg1_1972	B4	1901.789096	Bcer	I	Bcer	FALSE	NA	NA	NA
Bc_ctg1_1973	B4	960.3800586	Bcer	I	Bcer	FALSE	NA	NA	NA
Bc_ctg1_1974	B4	2013.718122	Bcer	I	Bcer	FALSE	NA	NA	NA
Bc_ctg1_1975	B4	0	Bcer	S	Bcer	FALSE	NA	NA	NA
Bc_ctg1_1976	B4	27.45218387	Bcer	M	Bcer	FALSE	NA	NA	NA
Bc_ctg1_1977	B4	1363.57006	Bcer	E	Bcer	FALSE	NA	NA	NA
Bc_ctg1_1978	B4	0.33478273	Bcer	S	Bcer	FALSE	NA	NA	NA
Bc_ctg1_1979	B4	58.14060081	Bcer	S	Bcer	FALSE	NA	NA	NA
Bc_ctg1_1980	B4	115.9464189	Bcer	NT	Bcer	FALSE	NA	NA	NA
Bc_ctg1_1981	B4	42.07102976	Bcer	K	Bcer	FALSE	NA	NA	NA
Bc_ctg1_1983	B4	6.026089143	Bcer	EGP	Bcer	FALSE	NA	NA	NA
Bc_ctg1_1984	B4	270.504446	Bcer	E	Bcer	FALSE	NA	NA	NA
Bc_ctg1_1985	B4	9.820293418	Bcer	NA	Bcer	FALSE	NA	NA	NA
Bc_ctg1_1986	B4	3.682610032	Bcer	NA	Bcer	FALSE	NA	NA	NA
Bc_ctg1_1987	B4	258.2290792	Bcer	U	Bcer	FALSE	NA	NA	NA
Bc_ctg1_1988	B4	37.27247729	Bcer	NA	Bcer	FALSE	NA	NA	NA
Bc_ctg1_1989	B4	123.7580159	Bcer	S	Bcer	FALSE	NA	NA	NA
Bc_ctg1_199	B4	43.74494341	Bcer	V	Bcer	FALSE	NA	NA	NA
Bc_ctg1_1990	B4	13.61449769	Bcer	J	Bcer	FALSE	NA	NA	NA

Bc_ctg1_1991	B4	53.11885985	Bcer	S	Bcer	FALSE	NA	NA	NA
Bc_ctg1_1992	B4	1.785507894	Bcer	NA	Bcer	FALSE	NA	NA	NA
Bc_ctg1_1993	B4	31.24638815	Bcer	S	Bcer	FALSE	NA	NA	NA
Bc_ctg1_1994	B4	10.48985888	Bcer	S	Bcer	FALSE	NA	NA	NA
Bc_ctg1_1995	B4	0	Bcer	K	Bcer	FALSE	NA	NA	NA
Bc_ctg1_1996	B4	40.73189884	Bcer	S	Bcer	FALSE	NA	NA	NA
Bc_ctg1_1997	B4	0.223188487	Bcer	S	Bcer	FALSE	NA	NA	NA
Bc_ctg1_1998	B4	2.008696381	Bcer	O	Bcer	FALSE	NA	NA	NA
Bc_ctg1_1999	B4	125.5435238	Bcer	T	Bcer	FALSE	NA	NA	NA
Bc_ctg1_2	B4	4.128987005	Bcer	S	Bcer	FALSE	NA	NA	NA
Bc_ctg1_20	B4	3.794204275	Bcer	S	Bcer	FALSE	NA	NA	NA
Bc_ctg1_200	B4	0	Bcer	NA	Bcer	FALSE	NA	NA	NA
Bc_ctg1_2000	B4	356.9899846	Bcer	T	Bcer	FALSE	NA	NA	NA
Bc_ctg1_2001	B4	17.63189046	Bcer	EG	Bcer	FALSE	NA	NA	NA
Bc_ctg1_2002	B4	3.347827302	Bcer	NA	Bcer	FALSE	NA	NA	NA
Bc_ctg1_2003	B4	5.802900656	Bcer	J	Bcer	FALSE	NA	NA	NA
Bc_ctg1_2004	B4	3.571015789	Bcer	H	Bcer	FALSE	NA	NA	NA
Bc_ctg1_2005	B4	803.9249294	Bcer	NT	Bcer	FALSE	NA	NA	NA
Bc_ctg1_2006	B4	120.1870001	Bcer	NT	Bcer	FALSE	NA	NA	NA
Bc_ctg1_2007	B4	8.481162498	Bcer	S	Bcer	FALSE	NA	NA	NA
Bc_ctg1_2008	B4	10.60145312	Bcer	E	Bcer	FALSE	NA	NA	NA
Bc_ctg1_2009	B4	38.27682548	Bcer	S	Bcer	FALSE	NA	NA	NA
Bc_ctg1_201	B4	0.33478273	Bcer	I	Bcer	FALSE	NA	NA	NA
Bc_ctg1_2010	B4	31.58117088	Bcer	K	Bcer	FALSE	NA	NA	NA
Bc_ctg1_2011	B4	0.111594243	Bcer	S	Bcer	FALSE	NA	NA	NA
Bc_ctg1_2012	B4	0.66956546	Bcer	S	Bcer	FALSE	NA	NA	NA
Bc_ctg1_2013	B4	5.021740953	Bcer	P	Bcer	FALSE	NA	NA	NA
Bc_ctg1_2014	B4	2.789856085	Bcer	P	Bcer	FALSE	NA	NA	NA
Bc_ctg1_2015	B4	95.63626659	Bcer	E	Bcer	FALSE	NA	NA	NA
Bc_ctg1_2016	B4	1364.686002	Bcer	P	Bcer	FALSE	NA	NA	NA
Bc_ctg1_2017	B4	2.343479111	Bcer	NA	Bcer	FALSE	NA	NA	NA
Bc_ctg1_2018	B4	0.33478273	Bcer	NA	Bcer	FALSE	NA	NA	NA
Bc_ctg1_2019	B4	3.905798519	Bcer	NA	Bcer	FALSE	NA	NA	NA
Bc_ctg1_202	B4	0.781159704	Bcer	NA	Bcer	FALSE	NA	NA	NA
Bc_ctg1_2020	B4	126.8826547	Bcer	S	Bcer	FALSE	NA	NA	NA
Bc_ctg1_2021	B4	125.5435238	Bcer	S	Bcer	FALSE	NA	NA	NA
Bc_ctg1_2022	B4	282.1102473	Bcer	P	Bcer	FALSE	NA	NA	NA
Bc_ctg1_2023	B4	6.137683386	Bcer	P	Bcer	FALSE	NA	NA	NA
Bc_ctg1_2024	B4	3.124638815	Bcer	P	Bcer	FALSE	NA	NA	NA
Bc_ctg1_2025	B4	587.320503	Bcer	F	Bcer	FALSE	NA	NA	NA
Bc_ctg1_2026	B4	3.459421545	Bcer	F	Bcer	FALSE	NA	NA	NA
Bc_ctg1_2027	B4	254.4348749	Bcer	E	Bcer	FALSE	NA	NA	NA
Bc_ctg1_2028	B4	180.2247031	Bcer	E	Bcer	FALSE	NA	NA	NA
Bc_ctg1_2029	B4	7.365220064	Bcer	S	Bcer	FALSE	NA	NA	NA
Bc_ctg1_203	B4	1.673913651	Bcer	S	Bcer	FALSE	NA	NA	NA
Bc_ctg1_2030	B4	15.28841134	Bcer	G	Bcer	FALSE	NA	NA	NA
Bc_ctg1_2031	B4	0	Bcer	M	Bcer	FALSE	NA	NA	NA
Bc_ctg1_2032	B4	10.82464161	Bcer	S	Bcer	FALSE	NA	NA	NA
Bc_ctg1_2033	B4	10.37826464	Bcer	EGP	Bcer	FALSE	NA	NA	NA
Bc_ctg1_2034	B4	7.142031577	Bcer	K	Bcer	FALSE	NA	NA	NA
Bc_ctg1_2035	B4	241.0435657	Bcer	S	Bcer	FALSE	NA	NA	NA
Bc_ctg1_2036	B4	1003.455437	Bcer	C	Bcer	FALSE	NA	NA	NA
Bc_ctg1_2037	B4	97.53336872	Bcer	J	Bcer	FALSE	NA	NA	NA
Bc_ctg1_2038	B4	184.5768786	Bcer	M	Bcer	FALSE	NA	NA	NA
Bc_ctg1_2039	B4	102.7782982	Bcer	S	Bcer	FALSE	NA	NA	NA
Bc_ctg1_204	B4	3.905798519	Bcer	S	Bcer	FALSE	NA	NA	NA
Bc_ctg1_2040	B4	40.84349308	Bcer	NT	Bcer	FALSE	NA	NA	NA
Bc_ctg1_2041	B4	21.42609473	Bcer	L	Bcer	FALSE	NA	NA	NA
Bc_ctg1_2042	B4	1.339130921	Bcer	D	Bcer	FALSE	NA	NA	NA
Bc_ctg1_2043	B4	0.33478273	Bcer	D	Bcer	FALSE	NA	NA	NA
Bc_ctg1_2044	B4	2.120290624	Bcer	NA	Bcer	FALSE	NA	NA	NA
Bc_ctg1_2045	B4	1.450725164	Bcer	NA	Bcer	FALSE	NA	NA	NA

Bc_ctg1_2046	B4	1.562319407	Bcer	EGP	Bcer	FALSE	NA	NA	NA
Bc_ctg1_2047	B4	0	Bcer	S	Bcer	FALSE	NA	NA	NA
Bc_ctg1_2048	B4	6.137683386	Bcer	J	Bcer	FALSE	NA	NA	NA
Bc_ctg1_2049	B4	13.05652648	Bcer	S	Bcer	FALSE	NA	NA	NA
Bc_ctg1_205	B4	1.785507894	Bcer	NA	Bcer	FALSE	NA	NA	NA
Bc_ctg1_2050	B4	0.223188487	Bcer	S	Bcer	FALSE	NA	NA	NA
Bc_ctg1_2051	B4	0.33478273	Bcer	Q	Bcer	TRUE	NA	NA	NA
Bc_ctg1_2052	B4	5.021740953	Bcer	K	Bcer	FALSE	NA	NA	NA
Bc_ctg1_2053	B4	0.33478273	Bcer	S	Bcer	FALSE	NA	NA	NA
Bc_ctg1_2054	B4	7.588408551	Bcer	K	Bcer	FALSE	NA	NA	NA
Bc_ctg1_2055	B4	4.128987005	Bcer	P	Bcer	FALSE	NA	NA	NA
Bc_ctg1_2056	B4	3.124638815	Bcer	P	Bcer	FALSE	NA	NA	NA
Bc_ctg1_2057	B4	1.897102138	Bcer	F	Bcer	FALSE	NA	NA	NA
Bc_ctg1_2058	B4	1468.24546	Bcer	M	Bcer	FALSE	NA	NA	NA
Bc_ctg1_2059	B4	30.01885147	Bcer	P	Bcer	FALSE	NA	NA	NA
Bc_ctg1_206	B4	0.111594243	Bcer	NA	Bcer	FALSE	NA	NA	NA
Bc_ctg1_2060	B4	30.3536342	Bcer	P	Bcer	FALSE	NA	NA	NA
Bc_ctg1_2061	B4	26.78261841	Bcer	P	Bcer	FALSE	NA	NA	NA
Bc_ctg1_2062	B4	20.42174654	Bcer	P	Bcer	FALSE	NA	NA	NA
Bc_ctg1_2063	B4	4.128987005	Bcer	T	Bcer	FALSE	NA	NA	NA
Bc_ctg1_2064	B4	20.19855805	Bcer	S	Bcer	FALSE	NA	NA	NA
Bc_ctg1_2065	B4	35.93334637	Bcer	O	Bcer	FALSE	NA	NA	NA
Bc_ctg1_2066	B4	548.1509235	Bcer	K	Bcer	FALSE	NA	NA	NA
Bc_ctg1_2067	B4	139.0464273	Bcer	S	Bcer	FALSE	NA	NA	NA
Bc_ctg1_2068	B4	17.96667319	Bcer	U	Bcer	FALSE	NA	NA	NA
Bc_ctg1_2069	B4	108.6927931	Bcer	M	Bcer	FALSE	NA	NA	NA
Bc_ctg1_207	B4	52.89567137	Bcer	S	Bcer	FALSE	NA	NA	NA
Bc_ctg1_2070	B4	53.56523683	Bcer	S	Bcer	FALSE	NA	NA	NA
Bc_ctg1_2071	B4	0.33478273	Bcer	F	Bcer	FALSE	NA	NA	NA
Bc_ctg1_2072	B4	0	Bcer	F	Bcer	FALSE	NA	NA	NA
Bc_ctg1_2073	B4	7585.060723	Bcer	G	Bcer	FALSE	NA	NA	NA
Bc_ctg1_2074	B4	9193.803336	Bcer	G	Bcer	FALSE	NA	NA	NA
Bc_ctg1_2075	B4	3601.927394	Bcer	G	Bcer	FALSE	NA	NA	NA
Bc_ctg1_2076	B4	5276.956987	Bcer	F	Bcer	FALSE	NA	NA	NA
Bc_ctg1_2077	B4	8093.149313	Bcer	G	Bcer	FALSE	NA	NA	NA
Bc_ctg1_2078	B4	3282.098292	Bcer	K	Bcer	FALSE	NA	NA	NA
Bc_ctg1_2079	B4	43.07537795	Bcer	O	Bcer	FALSE	NA	NA	NA
Bc_ctg1_208	B4	7.030437334	Bcer	S	Bcer	FALSE	NA	NA	NA
Bc_ctg1_2080	B4	456.8668324	Bcer	K	Bcer	FALSE	NA	NA	NA
Bc_ctg1_2081	B4	0	Bcer	NA	Bcer	FALSE	NA	NA	NA
Bc_ctg1_2082	B4	3.459421545	Bcer	CP	Bcer	FALSE	NA	NA	NA
Bc_ctg1_2083	B4	2.231884868	Bcer	S	Bcer	FALSE	NA	NA	NA
Bc_ctg1_2084	B4	8.369568254	Bcer	T	Bcer	FALSE	NA	NA	NA
Bc_ctg1_2085	B4	30.80001118	Bcer	K	Bcer	FALSE	NA	NA	NA
Bc_ctg1_2087	B4	0	Bcer	NA	Bcer	FALSE	NA	NA	NA
Bc_ctg1_2088	B4	0	Bcer	S	Bcer	FALSE	NA	NA	NA
Bc_ctg1_2089	B4	0	Bcer	I	Bcer	FALSE	NA	NA	NA
Bc_ctg1_209	B4	14.3956574	Bcer	P	Bcer	FALSE	NA	NA	NA
Bc_ctg1_2090	B4	0	Bcer	S	Bcer	FALSE	NA	NA	NA
Bc_ctg1_2091	B4	0	Bcer	S	Bcer	FALSE	NA	NA	NA
Bc_ctg1_2092	B4	0.223188487	Bcer	S	Bcer	FALSE	NA	NA	NA
Bc_ctg1_2093	B4	696.5712672	Bcer	OU	Bcer	FALSE	NA	NA	NA
Bc_ctg1_2094	B4	16.51594802	Bcer	G	Bcer	FALSE	NA	NA	NA
Bc_ctg1_2095	B4	183.6841246	Bcer	K	Bcer	FALSE	NA	NA	NA
Bc_ctg1_2096	B4	380.6479642	Bcer	S	Bcer	FALSE	NA	NA	NA
Bc_ctg1_2097	B4	293.0464831	Bcer	S	Bcer	FALSE	NA	NA	NA
Bc_ctg1_2098	B4	84.92321922	Bcer	F	Bcer	FALSE	NA	NA	NA
Bc_ctg1_2099	B4	6.695654603	Bcer	S	Bcer	FALSE	NA	NA	NA
Bc_ctg1_21	B4	2.231884868	Bcer	S	Bcer	FALSE	NA	NA	NA
Bc_ctg1_210	B4	4.910146709	Bcer	P	Bcer	FALSE	NA	NA	NA
Bc_ctg1_2100	B4	375.6262233	Bcer	C	Bcer	FALSE	NA	NA	NA
Bc_ctg1_2101	B4	214.1493531	Bcer	S	Bcer	FALSE	NA	NA	NA

Bc_ctg1_2102	B4	21.76087746	Bcer	S	Bcer	FALSE	NA	NA	NA
Bc_ctg1_2103	B4	21.091312	Bcer	S	Bcer	FALSE	NA	NA	NA
Bc_ctg1_2104	B4	427.1827637	Bcer	M	Bcer	FALSE	NA	NA	NA
Bc_ctg1_2105	B4	421.379863	Bcer	F	Bcer	FALSE	NA	NA	NA
Bc_ctg1_2106	B4	27.45218387	Bcer	S	Bcer	FALSE	NA	NA	NA
Bc_ctg1_2107	B4	617.3393544	Bcer	L	Bcer	FALSE	NA	NA	NA
Bc_ctg1_2108	B4	239.2580578	Bcer	L	Bcer	FALSE	NA	NA	NA
Bc_ctg1_2109	B4	3.236233058	Bcer	S	Bcer	FALSE	NA	NA	NA
Bc_ctg1_211	B4	10.48985888	Bcer	I	Bcer	FALSE	NA	NA	NA
Bc_ctg1_2110	B4	6.91884309	Bcer	NA	Bcer	FALSE	NA	NA	NA
Bc_ctg1_2111	B4	4.017392762	Bcer	K	Bcer	FALSE	NA	NA	NA
Bc_ctg1_2112	B4	2.566667598	Bcer	NA	Bcer	FALSE	NA	NA	NA
Bc_ctg1_2113	B4	4.128987005	Bcer	NA	Bcer	FALSE	NA	NA	NA
Bc_ctg1_2114	B4	2.901450328	Bcer	NA	Bcer	FALSE	NA	NA	NA
Bc_ctg1_2115	B4	0.111594243	Bcer	K	Bcer	FALSE	NA	NA	NA
Bc_ctg1_2116	B4	362.0117256	Bcer	S	Bcer	FALSE	NA	NA	NA
Bc_ctg1_2117	B4	8.257974011	Bcer	K	Bcer	FALSE	NA	NA	NA
Bc_ctg1_2118	B4	4.352175492	Bcer	K	Bcer	FALSE	NA	NA	NA
Bc_ctg1_2119	B4	9.039133715	Bcer	S	Bcer	FALSE	NA	NA	NA
Bc_ctg1_212	B4	325.520408	Bcer	I	Bcer	FALSE	NA	NA	NA
Bc_ctg1_2120	B4	8.146379768	Bcer	L	Bcer	FALSE	NA	NA	NA
Bc_ctg1_2121	B4	0.892753947	Bcer	S	Bcer	FALSE	NA	NA	NA
Bc_ctg1_2122	B4	8.034785524	Bcer	V	Bcer	FALSE	NA	NA	NA
Bc_ctg1_2123	B4	161.1420875	Bcer	O	Bcer	FALSE	NA	NA	NA
Bc_ctg1_2124	B4	111.8174319	Bcer	M	Bcer	FALSE	NA	NA	NA
Bc_ctg1_2125	B4	345.0494006	Bcer	D	Bcer	FALSE	NA	NA	NA
Bc_ctg1_2126	B4	99.43047086	Bcer	D	Bcer	FALSE	NA	NA	NA
Bc_ctg1_2127	B4	68.74205393	Bcer	C	Bcer	FALSE	NA	NA	NA
Bc_ctg1_2128	B4	255.2160346	Bcer	J	Bcer	FALSE	NA	NA	NA
Bc_ctg1_2129	B4	1607.403482	Bcer	U	Bcer	FALSE	NA	NA	NA
Bc_ctg1_213	B4	5.021740953	Bcer	H	Bcer	FALSE	NA	NA	NA
Bc_ctg1_2130	B4	1.227536677	Bcer	NA	Bcer	FALSE	NA	NA	NA
Bc_ctg1_2131	B4	3.013044572	Bcer	V	Bcer	FALSE	NA	NA	NA
Bc_ctg1_2132	B4	1.004348191	Bcer	NA	Bcer	FALSE	NA	NA	NA
Bc_ctg1_2133	B4	0.111594243	Bcer	NA	Bcer	FALSE	NA	NA	NA
Bc_ctg1_2134	B4	1.115942434	Bcer	NA	Bcer	FALSE	NA	NA	NA
Bc_ctg1_2135	B4	0.557971217	Bcer	KT	Bcer	FALSE	Bc_Ga0417192_01_rn14	Other	NA
Bc_ctg1_2136	B4	2.343479111	Bcer	NA	Bcer	FALSE	Bc_Ga0417192_01_rn14	Other	NA
Bc_ctg1_2137	B4	3538.430269	Bcer	J	Bcer	FALSE	Bc_Ga0417192_01_rn14	Other	NA
Bc_ctg1_2138	B4	0.223188487	Bcer	K	Bcer	FALSE	Bc_Ga0417192_01_rn14	Regulation	SMCOG1255
Bc_ctg1_2139	B4	2.231884868	Bcer	S	Bcer	FALSE	Bc_Ga0417192_01_rn14	Other	NA
Bc_ctg1_214	B4	0.892753947	Bcer	S	Bcer	FALSE	NA	NA	NA
Bc_ctg1_2140	B4	6.807248847	Bcer	L	Bcer	FALSE	Bc_Ga0417192_01_rn14	Other	NA
Bc_ctg1_2141	B4	1137.368529	Bcer	M	Bcer	FALSE	Bc_Ga0417192_01_rn14	Other	NA
Bc_ctg1_2142	B4	170.7391924	Bcer	K	Bcer	FALSE	Bc_Ga0417192_01_rn14	Regulation	SMCOG1167
Bc_ctg1_2143	B4	43.52175492	Bcer	S	Bcer	FALSE	Bc_Ga0417192_01_rn14	Other	NA
Bc_ctg1_2144	B4	20.86812351	Bcer	S	Bcer	FALSE	Bc_Ga0417192_01_rn14	Other	NA
Bc_ctg1_2145	B4	99.76525359	Bcer	K	Bcer	FALSE	Bc_Ga0417192_01_rn14	Other	NA
Bc_ctg1_2146	B4	882.3756825	Bcer	M	Bcer	FALSE	Bc_Ga0417192_01_rn14	Core biosynthesis	None
Bc_ctg1_2147	B4	4.463769736	Bcer	M	Bcer	FALSE	Bc_Ga0417192_01_rn14	Ancillary biosynthesis	SMCOG1152
Bc_ctg1_2148	B4	581.5176023	Bcer	M	Bcer	FALSE	Bc_Ga0417192_01_rn14	Core biosynthesis	None
Bc_ctg1_2149	B4	6.472466117	Bcer	G	Bcer	FALSE	Bc_Ga0417192_01_rn14	Ancillary biosynthesis	SMCOG1235
Bc_ctg1_215	B4	25.44348749	Bcer	L	Bcer	FALSE	NA	NA	NA
Bc_ctg1_2150	B4	115.1652592	Bcer	K	Bcer	FALSE	Bc_Ga0417192_01_rn14	Regulation	SMCOG1112
Bc_ctg1_2151	B4	0	Bcer	G	Bcer	FALSE	Bc_Ga0417192_01_rn14	Other	NA
Bc_ctg1_2152	B4	11.71739556	Bcer	P	Bcer	FALSE	Bc_Ga0417192_01_rn14	Other	NA
Bc_ctg1_2153	B4	10.48985888	Bcer	G	Bcer	FALSE	Bc_Ga0417192_01_rn14	Other	NA
Bc_ctg1_2154	B4	177.881224	Bcer	G	Bcer	FALSE	Bc_Ga0417192_01_rn14	Other	NA
Bc_ctg1_2155	B4	15.73478832	Bcer	G	Bcer	FALSE	Bc_Ga0417192_01_rn14	Other	NA
Bc_ctg1_2156	B4	333.4435993	Bcer	G	Bcer	FALSE	Bc_Ga0417192_01_rn14	Other	NA
Bc_ctg1_2157	B4	42.51740673	Bcer	G	Bcer	FALSE	Bc_Ga0417192_01_rn14	Other	NA
Bc_ctg1_2158	B4	0.781159704	Bcer	E	Bcer	FALSE	NA	NA	NA

Bc_ctg1_2159	B4	0.446376974	Bcer	E	Bcer	FALSE	NA	NA	NA
Bc_ctg1_216	B4	2.789856085	Bcer	NA	Bcer	FALSE	NA	NA	NA
Bc_ctg1_2160	B4	0.892753947	Bcer	G	Bcer	FALSE	NA	NA	NA
Bc_ctg1_2161	B4	19.52899259	Bcer	G	Bcer	FALSE	NA	NA	NA
Bc_ctg1_2162	B4	0	Bcer	G	Bcer	FALSE	NA	NA	NA
Bc_ctg1_2163	B4	4.240581249	Bcer	C	Bcer	FALSE	NA	NA	NA
Bc_ctg1_2164	B4	92.51162777	Bcer	I	Bcer	FALSE	NA	NA	NA
Bc_ctg1_2165	B4	43.07537795	Bcer	E	Bcer	FALSE	NA	NA	NA
Bc_ctg1_2166	B4	4.017392762	Bcer	S	Bcer	FALSE	NA	NA	NA
Bc_ctg1_2167	B4	15.06522286	Bcer	K	Bcer	FALSE	NA	NA	NA
Bc_ctg1_2168	B4	13.50290345	Bcer	G	Bcer	FALSE	NA	NA	NA
Bc_ctg1_2169	B4	0	Bcer	NA	Bcer	FALSE	NA	NA	NA
Bc_ctg1_217	B4	117.0623613	Bcer	I	Bcer	FALSE	NA	NA	NA
Bc_ctg1_2170	B4	0.446376974	Bcer	C	Bcer	FALSE	NA	NA	NA
Bc_ctg1_2171	B4	0.33478273	Bcer	C	Bcer	FALSE	NA	NA	NA
Bc_ctg1_2172	B4	0.111594243	Bcer	P	Bcer	FALSE	NA	NA	NA
Bc_ctg1_2173	B4	144.2913567	Bcer	H	Bcer	FALSE	NA	NA	NA
Bc_ctg1_2174	B4	18.07826743	Bcer	NA	Bcer	FALSE	NA	NA	NA
Bc_ctg1_2175	B4	0	Bcer	L	Bcer	FALSE	NA	NA	NA
Bc_ctg1_2177	B4	226.4247198	Bcer	C	Bcer	FALSE	NA	NA	NA
Bc_ctg1_2178	B4	8.481162498	Bcer	S	Bcer	FALSE	NA	NA	NA
Bc_ctg1_2179	B4	43.52175492	Bcer	S	Bcer	FALSE	NA	NA	NA
Bc_ctg1_218	B4	0.557971217	Bcer	NA	Bcer	FALSE	NA	NA	NA
Bc_ctg1_2180	B4	35.82175213	Bcer	S	Bcer	FALSE	NA	NA	NA
Bc_ctg1_2181	B4	611.0900768	Bcer	M	Bcer	FALSE	NA	NA	NA
Bc_ctg1_2182	B4	53.11885985	Bcer	E	Bcer	FALSE	NA	NA	NA
Bc_ctg1_2184	B4	4069.395679	Bcer	MT	Bcer	FALSE	NA	NA	NA
Bc_ctg1_2185	B4	7.25362582	Bcer	F	Bcer	FALSE	NA	NA	NA
Bc_ctg1_2186	B4	20.08696381	Bcer	T	Bcer	FALSE	NA	NA	NA
Bc_ctg1_2187	B4	43.9681319	Bcer	M	Bcer	FALSE	NA	NA	NA
Bc_ctg1_2188	B4	0.446376974	Bcer	NA	Bcer	FALSE	NA	NA	NA
Bc_ctg1_2189	B4	25.88986447	Bcer	M	Bcer	FALSE	NA	NA	NA
Bc_ctg1_219	B4	29.34928601	Bcer	S	Bcer	FALSE	NA	NA	NA
Bc_ctg1_2190	B4	221.5145731	Bcer	T	Bcer	FALSE	NA	NA	NA
Bc_ctg1_2191	B4	27.89856085	Bcer	S	Bcer	FALSE	NA	NA	NA
Bc_ctg1_2192	B4	1.673913651	Bcer	S	Bcer	FALSE	NA	NA	NA
Bc_ctg1_2193	B4	1.450725164	Bcer	NA	Bcer	FALSE	NA	NA	NA
Bc_ctg1_2194	B4	0	Bcer	S	Bcer	FALSE	NA	NA	NA
Bc_ctg1_2195	B4	100.5464133	Bcer	S	Bcer	FALSE	NA	NA	NA
Bc_ctg1_2196	B4	292.4885119	Bcer	L	Bcer	FALSE	NA	NA	NA
Bc_ctg1_2197	B4	31.58117088	Bcer	S	Bcer	FALSE	NA	NA	NA
Bc_ctg1_2198	B4	0	Bcer	S	Bcer	FALSE	NA	NA	NA
Bc_ctg1_2199	B4	0	Bcer	S	Bcer	FALSE	NA	NA	NA
Bc_ctg1_22	B4	4.240581249	Bcer	S	Bcer	FALSE	NA	NA	NA
Bc_ctg1_220	B4	3.682610032	Bcer	J	Bcer	FALSE	NA	NA	NA
Bc_ctg1_2200	B4	5.021740953	Bcer	S	Bcer	FALSE	NA	NA	NA
Bc_ctg1_2201	B4	0.66956546	Bcer	K	Bcer	FALSE	NA	NA	NA
Bc_ctg1_2202	B4	0	Bcer	K	Bcer	FALSE	NA	NA	NA
Bc_ctg1_2203	B4	61.15364538	Bcer	S	Bcer	FALSE	NA	NA	NA
Bc_ctg1_2204	B4	147.5275898	Bcer	V	Bcer	FALSE	Bc_Ga0417192_01_rn15	Other	NA
Bc_ctg1_2205	B4	57.91741232	Bcer	V	Bcer	FALSE	Bc_Ga0417192_01_rn15	Transport	SMCOG1000
Bc_ctg1_2206	B4	46.75798798	Bcer	M	Bcer	FALSE	Bc_Ga0417192_01_rn15	Transport	SMCOG1029
Bc_ctg1_2207	B4	0.111594243	Bcer	K	Bcer	FALSE	Bc_Ga0417192_01_rn15	Other	NA
Bc_ctg1_2208	B4	2.678261841	Bcer	S	Bcer	FALSE	Bc_Ga0417192_01_rn15	Other	NA
Bc_ctg1_2209	B4	35.82175213	Bcer	G	Bcer	FALSE	Bc_Ga0417192_01_rn15	Transport	SMCOG1106
Bc_ctg1_221	B4	0.33478273	Bcer	NA	Bcer	FALSE	NA	NA	NA
Bc_ctg1_2210	B4	27.45218387	Bcer	P	Bcer	FALSE	Bc_Ga0417192_01_rn15	Other	NA
Bc_ctg1_2211	B4	31.58117088	Bcer	S	Bcer	FALSE	Bc_Ga0417192_01_rn15	Other	NA
Bc_ctg1_2212	B4	40.73189884	Bcer	T	Bcer	FALSE	Bc_Ga0417192_01_rn15	Regulation	SMCOG1003
Bc_ctg1_2213	B4	16.18116529	Bcer	K	Bcer	FALSE	Bc_Ga0417192_01_rn15	Regulation	SMCOG1008
Bc_ctg1_2214	B4	25.44348749	Bcer	M	Bcer	FALSE	Bc_Ga0417192_01_rn15	Core biosynthesis	SMCOG1010
Bc_ctg1_2215	B4	35.82175213	Bcer	E	Bcer	FALSE	Bc_Ga0417192_01_rn15	Other	NA

Bc_ctg1_2216	B4	204.3290596	Bcer	K	Bcer	FALSE	Bc_Ga0417192_01_rn15	Other	NA
Bc_ctg1_2217	B4	206.2261618	Bcer	M	Bcer	FALSE	Bc_Ga0417192_01_rn15	Core biosynthesis	SMCOG1010
Bc_ctg1_2218	B4	246.9580606	Bcer	M	Bcer	FALSE	Bc_Ga0417192_01_rn15	Ancillary biosynthesis	SMCOG1152
Bc_ctg1_2219	B4	133.4667151	Bcer	S	Bcer	FALSE	Bc_Ga0417192_01_rn15	Core biosynthesis	None
Bc_ctg1_222	B4	1.450725164	Bcer	K	Bcer	FALSE	NA	NA	NA
Bc_ctg1_2220	B4	158.2406371	Bcer	M	Bcer	FALSE	Bc_Ga0417192_01_rn15	Other	NA
Bc_ctg1_2221	B4	73.76379488	Bcer	M	Bcer	FALSE	Bc_Ga0417192_01_rn15	Other	NA
Bc_ctg1_2222	B4	51.11016347	Bcer	M	Bcer	FALSE	Bc_Ga0417192_01_rn15	Core biosynthesis	None
Bc_ctg1_2223	B4	77.78118764	Bcer	M	Bcer	FALSE	Bc_Ga0417192_01_rn15	Core biosynthesis	SMCOG1045
Bc_ctg1_2224	B4	59.25654324	Bcer	M	Bcer	FALSE	Bc_Ga0417192_01_rn15	Core biosynthesis	SMCOG1045
Bc_ctg1_2225	B4	8.704350984	Bcer	S	Bcer	FALSE	Bc_Ga0417192_01_rn15	Other	NA
Bc_ctg1_2226	B4	15.28841134	Bcer	M	Bcer	FALSE	Bc_Ga0417192_01_rn15	Core biosynthesis	None
Bc_ctg1_2227	B4	67.06814028	Bcer	M	Bcer	FALSE	Bc_Ga0417192_01_rn15	Core biosynthesis	SMCOG1146
Bc_ctg1_2228	B4	48.0971189	Bcer	E	Bcer	FALSE	Bc_Ga0417192_01_rn15	Core biosynthesis	SMCOG1056
Bc_ctg1_2229	B4	26.2246472	Bcer	GM	Bcer	FALSE	Bc_Ga0417192_01_rn15	Core biosynthesis	SMCOG1010
Bc_ctg1_223	B4	1.673913651	Bcer	C	Bcer	FALSE	NA	NA	NA
Bc_ctg1_2230	B4	8.815945228	Bcer	M	Bcer	FALSE	Bc_Ga0417192_01_rn15	Core biosynthesis	SMCOG1064
Bc_ctg1_2231	B4	6.472466117	Bcer	GM	Bcer	FALSE	Bc_Ga0417192_01_rn15	Other	NA
Bc_ctg1_2232	B4	84.70003073	Bcer	D	Bcer	FALSE	Bc_Ga0417192_01_rn15	Ancillary biosynthesis	SMCOG1216
Bc_ctg1_2233	B4	50.55219226	Bcer	M	Bcer	FALSE	Bc_Ga0417192_01_rn15	Ancillary biosynthesis	SMCOG1216
Bc_ctg1_2234	B4	81.12901495	Bcer	D	Bcer	FALSE	Bc_Ga0417192_01_rn15	Ancillary biosynthesis	SMCOG1216
Bc_ctg1_2235	B4	132.0159899	Bcer	I	Bcer	FALSE	Bc_Ga0417192_01_rn15	Ancillary biosynthesis	SMCOG1270
Bc_ctg1_2236	B4	294.0508313	Bcer	D	Bcer	FALSE	Bc_Ga0417192_01_rn15	Other	NA
Bc_ctg1_2237	B4	0.557971217	Bcer	K	Bcer	FALSE	Bc_Ga0417192_01_rn15	Other	NA
Bc_ctg1_2238	B4	0.557971217	Bcer	M	Bcer	FALSE	Bc_Ga0417192_01_rn15	Other	NA
Bc_ctg1_2239	B4	10.04348191	Bcer	V	Bcer	FALSE	Bc_Ga0417192_01_rn15	Other	NA
Bc_ctg1_224	B4	6.137683386	Bcer	S	Bcer	FALSE	NA	NA	NA
Bc_ctg1_2240	B4	2.901450328	Bcer	V	Bcer	FALSE	Bc_Ga0417192_01_rn15	Transport	SMCOG1000
Bc_ctg1_2241	B4	12.83333799	Bcer	KTV	Bcer	FALSE	NA	NA	NA
Bc_ctg1_2242	B4	15.1768171	Bcer	D	Bcer	FALSE	NA	NA	NA
Bc_ctg1_2243	B4	830.7075478	Bcer	M	Bcer	FALSE	NA	NA	NA
Bc_ctg1_2244	B4	196.6290569	Bcer	S	Bcer	FALSE	NA	NA	NA
Bc_ctg1_2245	B4	18.63623865	Bcer	S	Bcer	FALSE	NA	NA	NA
Bc_ctg1_2246	B4	9.373916445	Bcer	C	Bcer	FALSE	NA	NA	NA
Bc_ctg1_2247	B4	8.481162498	Bcer	C	Bcer	FALSE	NA	NA	NA
Bc_ctg1_2248	B4	12.16377253	Bcer	CP	Bcer	FALSE	NA	NA	NA
Bc_ctg1_2249	B4	0.66956546	Bcer	C	Bcer	FALSE	NA	NA	NA
Bc_ctg1_225	B4	0.446376974	Bcer	G	Bcer	FALSE	NA	NA	NA
Bc_ctg1_2250	B4	2.231884868	Bcer	C	Bcer	FALSE	NA	NA	NA
Bc_ctg1_2251	B4	1.673913651	Bcer	C	Bcer	FALSE	NA	NA	NA
Bc_ctg1_2252	B4	3.571015789	Bcer	C	Bcer	FALSE	NA	NA	NA
Bc_ctg1_2253	B4	4.910146709	Bcer	C	Bcer	FALSE	NA	NA	NA
Bc_ctg1_2254	B4	0.892753947	Bcer	C	Bcer	FALSE	NA	NA	NA
Bc_ctg1_2255	B4	2.566667598	Bcer	C	Bcer	FALSE	NA	NA	NA
Bc_ctg1_2256	B4	0.557971217	Bcer	C	Bcer	FALSE	NA	NA	NA
Bc_ctg1_2257	B4	16.06957105	Bcer	T	Bcer	FALSE	NA	NA	NA
Bc_ctg1_2258	B4	19.19420986	Bcer	S	Bcer	FALSE	NA	NA	NA
Bc_ctg1_2259	B4	11.15942434	Bcer	S	Bcer	FALSE	NA	NA	NA
Bc_ctg1_226	B4	10.26667039	Bcer	NA	Bcer	FALSE	NA	NA	NA
Bc_ctg1_2260	B4	818.9901522	Bcer	C	Bcer	FALSE	NA	NA	NA
Bc_ctg1_2261	B4	4151.305854	Bcer	C	Bcer	FALSE	NA	NA	NA
Bc_ctg1_2262	B4	1362.230929	Bcer	C	Bcer	FALSE	NA	NA	NA
Bc_ctg1_2263	B4	2430.968998	Bcer	C	Bcer	FALSE	NA	NA	NA
Bc_ctg1_2264	B4	447.7161045	Bcer	C	Bcer	FALSE	NA	NA	NA
Bc_ctg1_2265	B4	422.4958055	Bcer	C	Bcer	FALSE	NA	NA	NA
Bc_ctg1_2266	B4	133.9130921	Bcer	C	Bcer	FALSE	NA	NA	NA
Bc_ctg1_2267	B4	726.1437417	Bcer	C	Bcer	FALSE	NA	NA	NA
Bc_ctg1_2268	B4	207.4536985	Bcer	S	Bcer	FALSE	NA	NA	NA
Bc_ctg1_2269	B4	68.51886544	Bcer	S	Bcer	FALSE	NA	NA	NA
Bc_ctg1_2270	B4	199.1957245	Bcer	F	Bcer	FALSE	NA	NA	NA
Bc_ctg1_2271	B4	1972.539846	Bcer	E	Bcer	FALSE	NA	NA	NA
Bc_ctg1_2272	B4	169.9580327	Bcer	S	Bcer	FALSE	NA	NA	NA

Bc_ctg1_2273	B4	90.16814866	Bcer	G	Bcer	FALSE	NA	NA	NA	
Bc_ctg1_2274	B4	26.2246472	Bcer	T	Bcer	FALSE	NA	NA	NA	
Bc_ctg1_2275	B4	184.2420958	Bcer	G	Bcer	FALSE	NA	NA	NA	
Bc_ctg1_2276	B4	0.892753947	Bcer	S	Bcer	FALSE	NA	NA	NA	
Bc_ctg1_2277	B4	31.69276512	Bcer	S	Bcer	FALSE	NA	NA	NA	
Bc_ctg1_2278	B4	16.40435378	Bcer	NA	Bcer	FALSE	NA	NA	NA	
Bc_ctg1_2279	B4	0.557971217	Bcer	NA	Bcer	FALSE	NA	NA	NA	
Bc_ctg1_2280	B4	54.0116138	Bcer	P	Bcer	FALSE	NA	NA	NA	
Bc_ctg1_2281	B4	59.70292021	Bcer	J	Bcer	FALSE	NA	NA	NA	
Bc_ctg1_2282	B4	8.257974011	Bcer	E	Bcer	FALSE	NA	NA	NA	
Bc_ctg1_2283	B4	0.33478273	Bcer	S	Bcer	FALSE	NA	NA	NA	
Bc_ctg1_2284	B4	56.91306413	Bcer	J	Bcer	FALSE	NA	NA	NA	
Bc_ctg1_2285	B4	464.5668352	Bcer	J	Bcer	FALSE	NA	NA	NA	
Bc_ctg1_2286	B4	69.41161939	Bcer	F	Bcer	FALSE	NA	NA	NA	
Bc_ctg1_2287	B4	1102.104748	Bcer	J	Bcer	FALSE	NA	NA	NA	
Bc_ctg1_2288	B4	751.2524465	Bcer	K	Bcer	FALSE	NA	NA	NA	
Bc_ctg1_2289	B4	685.4118429	Bcer	G	Bcer	FALSE	NA	NA	NA	
Bc_ctg1_229	B4	0.223188487	Bcer	NA	Bcer	FALSE	NA	NA	NA	
Bc_ctg1_2290	B4	943.0829509	Bcer	M	Bcer	FALSE	NA	NA	NA	
Bc_ctg1_2291	B4	4522.914685	Bcer	G	Bcer	FALSE	NA	NA	NA	
Bc_ctg1_2292	B4	91.39568534	Bcer	T	Bcer	FALSE	NA	NA	NA	
Bc_ctg1_2293	B4	18.07826743	Bcer	S	Bcer	FALSE	NA	NA	NA	
Bc_ctg1_2294	B4	776.3611513	Bcer	F	Bcer	FALSE	NA	NA	NA	
Bc_ctg1_2295	B4	495.1436579	Bcer	K	Bcer	FALSE	NA	NA	NA	
Bc_ctg1_2296	B4	12.27536677	Bcer	K	Bcer	FALSE	NA	NA	NA	
Bc_ctg1_2297	B4	95.63626659	Bcer	I	Bcer	FALSE	NA	NA	NA	
Bc_ctg1_2298	B4	86.82032136	Bcer	I	Bcer	FALSE	NA	NA	NA	
Bc_ctg1_2299	B4	69.07683666	Bcer	I	Bcer	FALSE	NA	NA	NA	
Bc_ctg1_23	B4	6.695654603	Bcer	E	Bcer	FALSE	NA	NA	NA	
Bc_ctg1_230	B4	1.004348191	Bcer	NA	Bcer	FALSE	NA	NA	NA	
Bc_ctg1_2300	B4	67.17973452	Bcer	I	Bcer	FALSE	NA	NA	NA	
Bc_ctg1_2301	B4	415.3537739	Bcer	C	Bcer	FALSE	NA	NA	NA	
Bc_ctg1_2302	B4	0.781159704	Bcer	I	Bcer	FALSE	NA	NA	NA	
Bc_ctg1_2303	B4	2.343479111	Bcer	L	Bcer	FALSE	NA	NA	NA	
Bc_ctg1_2304	B4	22.8768199	Bcer	T	Bcer	FALSE	NA	NA	NA	
Bc_ctg1_2305	B4	0.111594243	Bcer	S	Bcer	FALSE	NA	NA	NA	
Bc_ctg1_2306	B4	41.28987005	Bcer	K	Bcer	FALSE	NA	NA	NA	
Bc_ctg1_2307	B4	707.6190973	Bcer	E	Bcer	FALSE	NA	NA	NA	
Bc_ctg1_2308	B4	14.28406315	Bcer	T	Bcer	FALSE	NA	NA	NA	
Bc_ctg1_2309	B4	40.06233338	Bcer	T	Bcer	FALSE	NA	NA	NA	
Bc_ctg1_231	B4	7.25362582	Bcer	IQ	Bcer	TRUE	NA	NA	NA	
Bc_ctg1_2310	B4	6.24927763	Bcer	V	Bcer	FALSE	NA	NA	NA	
Bc_ctg1_2311	B4	7.700002794	Bcer	V	Bcer	FALSE	NA	NA	NA	
Bc_ctg1_2312	B4	444.8146542	Bcer	E	Bcer	FALSE	NA	NA	NA	
Bc_ctg1_2313	B4	455.8624843	Bcer	M	Bcer	FALSE	NA	NA	NA	
Bc_ctg1_2314	B4	4.128987005	Bcer	S	Bcer	FALSE	NA	NA	NA	
Bc_ctg1_2315	B4	14.50725164	Bcer	NA	Bcer	FALSE	NA	NA	NA	
Bc_ctg1_2316	B4	0.66956546	Bcer	NA	Bcer	FALSE	NA	NA	NA	
Bc_ctg1_2317	B4	0	Bcer	NA	Bcer	FALSE	NA	NA	NA	
Bc_ctg1_2318	B4	0	Bcer	K	Bcer	FALSE	NA	NA	NA	
Bc_ctg1_2319	B4	180.1131088	Bcer	J	Bcer	FALSE	NA	NA	NA	
Bc_ctg1_232	B4	30.3536342	Bcer	S	Bcer	FALSE	NA	NA	NA	
Bc_ctg1_2320	B4	12.72174375	Bcer	S	Bcer	FALSE	NA	NA	NA	
Bc_ctg1_2321	B4	41.62465278	Bcer	V	Bcer	FALSE	NA	NA	NA	
Bc_ctg1_2322	B4	0.111594243	Bcer	S	Bcer	FALSE	NA	NA	NA	
Bc_ctg1_2323	B4	28.01015509	Bcer	G	Bcer	FALSE	NA	NA	NA	
Bc_ctg1_2324	B4	334.6711359	Bcer	E	Bcer	FALSE	NA	NA	NA	
Bc_ctg1_2325	B4	262.1348777	Bcer	E	Bcer	FALSE	NA	NA	NA	
Bc_ctg1_2326	B4	6.807248847	Bcer	EGP	Bcer	FALSE	NA	NA	NA	
Bc_ctg1_2327	B4	3.347827302	Bcer	K	Bcer	FALSE	NA	NA	NA	
Bc_ctg1_2328	B4	138.0420791	Bcer	C	Bcer	FALSE	NA	NA	NA	
Bc_ctg1_2329	B4	12.49855526	Bcer	M	Bcer	FALSE	NA	NA	NA	

Bc_ctg1_233	B4	6.360871873	Bcer	V	Bcer	FALSE	NA	NA	NA
Bc_ctg1_2330	B4	96.52902053	Bcer	S	Bcer	FALSE	NA	NA	NA
Bc_ctg1_2331	B4	115.7232304	Bcer	S	Bcer	FALSE	NA	NA	NA
Bc_ctg1_2332	B4	137.9304848	Bcer	G	Bcer	FALSE	NA	NA	NA
Bc_ctg1_2333	B4	363.3508565	Bcer	S	Bcer	FALSE	NA	NA	NA
Bc_ctg1_2334	B4	33.7014615	Bcer	P	Bcer	FALSE	NA	NA	NA
Bc_ctg1_2335	B4	49.99422104	Bcer	HP	Bcer	FALSE	NA	NA	NA
Bc_ctg1_2336	B4	43.63334917	Bcer	P	Bcer	FALSE	NA	NA	NA
Bc_ctg1_2337	B4	41.4014643	Bcer	P	Bcer	FALSE	NA	NA	NA
Bc_ctg1_2338	B4	3.236233058	Bcer	S	Bcer	FALSE	NA	NA	NA
Bc_ctg1_2339	B4	0.33478273	Bcer	S	Bcer	FALSE	NA	NA	NA
Bc_ctg1_234	B4	0	Bcer	NA	Bcer	FALSE	NA	NA	NA
Bc_ctg1_2340	B4	50.32900377	Bcer	H	Bcer	FALSE	NA	NA	NA
Bc_ctg1_2341	B4	0.111594243	Bcer	NA	Bcer	FALSE	NA	NA	NA
Bc_ctg1_2343	B4	309.4508369	Bcer	C	Bcer	FALSE	NA	NA	NA
Bc_ctg1_2344	B4	658.1828475	Bcer	C	Bcer	FALSE	NA	NA	NA
Bc_ctg1_2345	B4	43.29856644	Bcer	M	Bcer	FALSE	NA	NA	NA
Bc_ctg1_2346	B4	0.111594243	Bcer	M	Bcer	FALSE	NA	NA	NA
Bc_ctg1_2347	B4	0.446376974	Bcer	S	Bcer	FALSE	NA	NA	NA
Bc_ctg1_2348	B4	41.17827581	Bcer	S	Bcer	FALSE	NA	NA	NA
Bc_ctg1_2349	B4	1.450725164	Bcer	S	Bcer	FALSE	NA	NA	NA
Bc_ctg1_235	B4	2.343479111	Bcer	NA	Bcer	FALSE	NA	NA	NA
Bc_ctg1_2350	B4	20.86812351	Bcer	P	Bcer	FALSE	NA	NA	NA
Bc_ctg1_2351	B4	39.83914489	Bcer	F	Bcer	FALSE	NA	NA	NA
Bc_ctg1_2352	B4	91.84206231	Bcer	S	Bcer	FALSE	Bc_Ga0417192_01_rn16	Ancillary biosynthesis	SMCOG1179
Bc_ctg1_2353	B4	34.25943272	Bcer	EGP	Bcer	FALSE	Bc_Ga0417192_01_rn16	Other	NA
Bc_ctg1_2354	B4	46.53479949	Bcer	L	Bcer	FALSE	Bc_Ga0417192_01_rn16	Other	NA
Bc_ctg1_2355	B4	61.37683386	Bcer	S	Bcer	FALSE	Bc_Ga0417192_01_rn16	Other	NA
Bc_ctg1_2356	B4	39.6159564	Bcer	V	Bcer	FALSE	Bc_Ga0417192_01_rn16	Transport	SMCOG1000
Bc_ctg1_2357	B4	30.24203996	Bcer	E	Bcer	FALSE	Bc_Ga0417192_01_rn16	Other	NA
Bc_ctg1_2358	B4	6.91884309	Bcer	K	Bcer	FALSE	Bc_Ga0417192_01_rn16	Regulation	SMCOG1078
Bc_ctg1_2359	B4	2.789856085	Bcer	S	Bcer	FALSE	Bc_Ga0417192_01_rn16	Other	NA
Bc_ctg1_236	B4	2.343479111	Bcer	K	Bcer	FALSE	NA	NA	NA
Bc_ctg1_2360	B4	61.7116166	Bcer	E	Bcer	FALSE	Bc_Ga0417192_01_rn16	Other	NA
Bc_ctg1_2361	B4	42.182624	Bcer	E	Bcer	FALSE	Bc_Ga0417192_01_rn16	Other	NA
Bc_ctg1_2362	B4	82.91452284	Bcer	E	Bcer	FALSE	Bc_Ga0417192_01_rn16	Core biosynthesis	SMCOG1168
Bc_ctg1_2363	B4	22.43044292	Bcer	P	Bcer	FALSE	Bc_Ga0417192_01_rn16	Other	NA
Bc_ctg1_2364	B4	1.339130921	Bcer	S	Bcer	FALSE	Bc_Ga0417192_01_rn16	Ancillary biosynthesis	SMCOG1115
Bc_ctg1_2365	B4	11.49420707	Bcer	T	Bcer	FALSE	Bc_Ga0417192_01_rn16	Regulation	SMCOG1133
Bc_ctg1_2366	B4	0	Bcer	L	Bcer	FALSE	Bc_Ga0417192_01_rn16	Other	SMCOG1164
Bc_ctg1_2367	B4	0.66956546	Bcer	L	Bcer	FALSE	Bc_Ga0417192_01_rn16	Other	SMCOG1026
Bc_ctg1_2368	B4	2.455073355	Bcer	I	Bcer	FALSE	Bc_Ga0417192_01_rn16	Other	NA
Bc_ctg1_2369	B4	4.352175492	Bcer	K	Bcer	FALSE	Bc_Ga0417192_01_rn16	Regulation	SMCOG1016
Bc_ctg1_237	B4	134.2478748	Bcer	NA	Bcer	FALSE	NA	NA	NA
Bc_ctg1_2370	B4	9.597104932	Bcer	T	Bcer	FALSE	Bc_Ga0417192_01_rn16	Regulation	SMCOG1048
Bc_ctg1_2371	B4	533.9784546	Bcer	H	Bcer	FALSE	Bc_Ga0417192_01_rn16	Other	NA
Bc_ctg1_2372	B4	38.053637	Bcer	T	Bcer	FALSE	Bc_Ga0417192_01_rn16	Ancillary biosynthesis	SMCOG1025
Bc_ctg1_2373	B4	4.686958222	Bcer	S	Bcer	FALSE	Bc_Ga0417192_01_rn16	Other	SMCOG1211
Bc_ctg1_2374	B4	689.3176414	Bcer	T	Bcer	FALSE	Bc_Ga0417192_01_rn16	Other	SMCOG1185
Bc_ctg1_2375	B4	268.8305323	Bcer	KT	Bcer	FALSE	Bc_Ga0417192_01_rn16	Other	NA
Bc_ctg1_2376	B4	35.93334637	Bcer	G	Bcer	FALSE	Bc_Ga0417192_01_rn16	Transport	SMCOG1169
Bc_ctg1_2377	B4	144.6261394	Bcer	M	Bcer	FALSE	Bc_Ga0417192_01_rn16	Other	NA
Bc_ctg1_2378	B4	156.2319407	Bcer	M	Bcer	FALSE	Bc_Ga0417192_01_rn16	Core biosynthesis	SMCOG1045
Bc_ctg1_2379	B4	261.353718	Bcer	M	Bcer	FALSE	Bc_Ga0417192_01_rn16	Other	NA
Bc_ctg1_238	B4	68.85364817	Bcer	S	Bcer	FALSE	NA	NA	NA
Bc_ctg1_2380	B4	30.91160542	Bcer	S	Bcer	FALSE	Bc_Ga0417192_01_rn16	Other	NA
Bc_ctg1_2381	B4	70.86234455	Bcer	NT	Bcer	FALSE	Bc_Ga0417192_01_rn16	Other	SMCOG1061
Bc_ctg1_2382	B4	26.55942993	Bcer	M	Bcer	FALSE	Bc_Ga0417192_01_rn16	Other	NA
Bc_ctg1_2383	B4	17.85507894	Bcer	I	Bcer	FALSE	Bc_Ga0417192_01_rn16	Other	NA
Bc_ctg1_2384	B4	0.33478273	Bcer	M	Bcer	FALSE	Bc_Ga0417192_01_rn16	Other	NA
Bc_ctg1_2385	B4	29.5724745	Bcer	M	Bcer	FALSE	Bc_Ga0417192_01_rn16	Core biosynthesis	SMCOG1123
Bc_ctg1_2386	B4	96.52902053	Bcer	S	Bcer	FALSE	Bc_Ga0417192_01_rn16	Core biosynthesis	None

Bc_ctg1_2387	B4	550.0480257	Bcer	S	Bcer	FALSE	Bc_Ga0417192_01_rn16	Other	NA
Bc_ctg1_2388	B4	325.6320022	Bcer	M	Bcer	FALSE	Bc_Ga0417192_01_rn16	Core biosynthesis	SMCOG1123
Bc_ctg1_239	B4	46.75798798	Bcer	T	Bcer	FALSE	NA	NA	NA
Bc_ctg1_2390	B4	0	Bcer	NA	Bcer	FALSE	Bc_Ga0417192_01_rn16	Other	NA
Bc_ctg1_2391	B4	3.013044572	Bcer	GM	Bcer	FALSE	Bc_Ga0417192_01_rn16	Ancillary biosynthesis	SMCOG1010
Bc_ctg1_2392	B4	19.41739835	Bcer	K	Bcer	FALSE	Bc_Ga0417192_01_rn16	Regulation	SMCOG1057
Bc_ctg1_2393	B4	171.9667291	Bcer	S	Bcer	FALSE	Bc_Ga0417192_01_rn16	Transport	SMCOG1000
Bc_ctg1_2394	B4	19.52899259	Bcer	K	Bcer	FALSE	Bc_Ga0417192_01_rn16	Regulation	SMCOG1057
Bc_ctg1_2395	B4	188.1478944	Bcer	V	Bcer	FALSE	Bc_Ga0417192_01_rn16	Transport	SMCOG1049
Bc_ctg1_2396	B4	71.7550985	Bcer	O	Bcer	FALSE	NA	NA	NA
Bc_ctg1_2397	B4	403.4131899	Bcer	S	Bcer	FALSE	NA	NA	NA
Bc_ctg1_2398	B4	1284.226553	Bcer	M	Bcer	FALSE	NA	NA	NA
Bc_ctg1_2399	B4	414.3494257	Bcer	S	Bcer	FALSE	NA	NA	NA
Bc_ctg1_24	B4	4.686958222	Bcer	E	Bcer	FALSE	NA	NA	NA
Bc_ctg1_240	B4	1.450725164	Bcer	K	Bcer	FALSE	NA	NA	NA
Bc_ctg1_2400	B4	23.10000838	Bcer	T	Bcer	FALSE	NA	NA	NA
Bc_ctg1_2401	B4	38.16523124	Bcer	T	Bcer	FALSE	NA	NA	NA
Bc_ctg1_2402	B4	8.927539471	Bcer	EGP	Bcer	FALSE	Bc_Ga0417192_01_rn17	Transport	SMCOG1169
Bc_ctg1_2403	B4	63.83190722	Bcer	M	Bcer	FALSE	Bc_Ga0417192_01_rn17	Other	NA
Bc_ctg1_2404	B4	392.1421713	Bcer	C	Bcer	FALSE	Bc_Ga0417192_01_rn17	Other	NA
Bc_ctg1_2405	B4	30248.73561	Bcer	P	Bcer	FALSE	Bc_Ga0417192_01_rn17	Other	NA
Bc_ctg1_2406	B4	10.93623585	Bcer	GM	Bcer	FALSE	Bc_Ga0417192_01_rn17	Ancillary biosynthesis	SMCOG1010
Bc_ctg1_2407	B4	11.60580131	Bcer	S	Bcer	FALSE	Bc_Ga0417192_01_rn17	Other	NA
Bc_ctg1_2408	B4	0.223188487	Bcer	NA	Bcer	FALSE	Bc_Ga0417192_01_rn17	Other	NA
Bc_ctg1_2409	B4	444.4798714	Bcer	M	Bcer	FALSE	Bc_Ga0417192_01_rn17	Core biosynthesis	SMCOG1010
Bc_ctg1_241	B4	16.06957105	Bcer	S	Bcer	FALSE	NA	NA	NA
Bc_ctg1_2410	B4	452.8494397	Bcer	S	Bcer	FALSE	Bc_Ga0417192_01_rn17	Other	NA
Bc_ctg1_2411	B4	22.76522565	Bcer	K	Bcer	FALSE	Bc_Ga0417192_01_rn17	Other	NA
Bc_ctg1_2412	B4	78.00437613	Bcer	JKL	Bcer	FALSE	Bc_Ga0417192_01_rn17	Other	SMCOG1122
Bc_ctg1_2413	B4	368.4841917	Bcer	F	Bcer	FALSE	Bc_Ga0417192_01_rn17	Other	NA
Bc_ctg1_2414	B4	52.33770015	Bcer	E	Bcer	FALSE	Bc_Ga0417192_01_rn17	Other	NA
Bc_ctg1_2415	B4	32.80870756	Bcer	C	Bcer	FALSE	Bc_Ga0417192_01_rn17	Ancillary biosynthesis	None
Bc_ctg1_2416	B4	83.13771133	Bcer	T	Bcer	FALSE	Bc_Ga0417192_01_rn17	Regulation	SMCOG1003
Bc_ctg1_2417	B4	16.962325	Bcer	J	Bcer	FALSE	NA	NA	NA
Bc_ctg1_2418	B4	0.892753947	Bcer	S	Bcer	FALSE	NA	NA	NA
Bc_ctg1_2419	B4	243.6102333	Bcer	O	Bcer	FALSE	NA	NA	NA
Bc_ctg1_242	B4	7.142031577	Bcer	F	Bcer	FALSE	NA	NA	NA
Bc_ctg1_2420	B4	194.6203605	Bcer	S	Bcer	FALSE	NA	NA	NA
Bc_ctg1_2421	B4	131.7928014	Bcer	S	Bcer	FALSE	NA	NA	NA
Bc_ctg1_2422	B4	327.9754813	Bcer	S	Bcer	FALSE	NA	NA	NA
Bc_ctg1_2423	B4	545.5842559	Bcer	T	Bcer	FALSE	NA	NA	NA
Bc_ctg1_2424	B4	144.2913567	Bcer	T	Bcer	FALSE	NA	NA	NA
Bc_ctg1_2425	B4	853.0263965	Bcer	F	Bcer	FALSE	NA	NA	NA
Bc_ctg1_2426	B4	940.7394718	Bcer	L	Bcer	FALSE	NA	NA	NA
Bc_ctg1_2427	B4	133.3551209	Bcer	J	Bcer	FALSE	NA	NA	NA
Bc_ctg1_2428	B4	990.6220986	Bcer	T	Bcer	FALSE	NA	NA	NA
Bc_ctg1_2429	B4	18.18986167	Bcer	S	Bcer	FALSE	NA	NA	NA
Bc_ctg1_243	B4	2.231884868	Bcer	S	Bcer	FALSE	NA	NA	NA
Bc_ctg1_2430	B4	387.0088361	Bcer	J	Bcer	FALSE	NA	NA	NA
Bc_ctg1_2431	B4	1489.894744	Bcer	L	Bcer	FALSE	NA	NA	NA
Bc_ctg1_2432	B4	584.9770239	Bcer	J	Bcer	FALSE	NA	NA	NA
Bc_ctg1_2433	B4	384.1073858	Bcer	J	Bcer	FALSE	NA	NA	NA
Bc_ctg1_2434	B4	13.94928042	Bcer	S	Bcer	FALSE	NA	NA	NA
Bc_ctg1_2435	B4	109.2507643	Bcer	M	Bcer	FALSE	NA	NA	NA
Bc_ctg1_2436	B4	0.66956546	Bcer	S	Bcer	FALSE	NA	NA	NA
Bc_ctg1_2437	B4	1449.497627	Bcer	K	Bcer	FALSE	NA	NA	NA
Bc_ctg1_2438	B4	467.9146625	Bcer	D	Bcer	FALSE	NA	NA	NA
Bc_ctg1_2439	B4	302.3088053	Bcer	D	Bcer	FALSE	NA	NA	NA
Bc_ctg1_244	B4	2.008696381	Bcer	E	Bcer	FALSE	NA	NA	NA
Bc_ctg1_2440	B4	149.3130977	Bcer	J	Bcer	FALSE	NA	NA	NA
Bc_ctg1_2441	B4	441.8016096	Bcer	D	Bcer	FALSE	NA	NA	NA
Bc_ctg1_2442	B4	220.063848	Bcer	S	Bcer	FALSE	NA	NA	NA

Bc_ctg1_2443	B4	316.0348973	Bcer	S	Bcer	FALSE	NA	NA	NA
Bc_ctg1_2444	B4	266.8218359	Bcer	U	Bcer	FALSE	NA	NA	NA
Bc_ctg1_2445	B4	23.21160263	Bcer	J	Bcer	FALSE	NA	NA	NA
Bc_ctg1_2446	B4	0.892753947	Bcer	J	Bcer	FALSE	NA	NA	NA
Bc_ctg1_2447	B4	371.2740478	Bcer	L	Bcer	FALSE	NA	NA	NA
Bc_ctg1_2448	B4	269.7232863	Bcer	L	Bcer	FALSE	NA	NA	NA
Bc_ctg1_2449	B4	11.0478301	Bcer	S	Bcer	FALSE	NA	NA	NA
Bc_ctg1_245	B4	7.588408551	Bcer	GK	Bcer	FALSE	NA	NA	NA
Bc_ctg1_2450	B4	359.445058	Bcer	L	Bcer	FALSE	NA	NA	NA
Bc_ctg1_2451	B4	1316.365695	Bcer	L	Bcer	FALSE	NA	NA	NA
Bc_ctg1_2452	B4	2183.56456	Bcer	L	Bcer	FALSE	NA	NA	NA
Bc_ctg1_2454	B4	1.115942434	Bcer	S	Bcer	FALSE	NA	NA	NA
Bc_ctg1_2455	B4	1548.481721	Bcer	F	Bcer	FALSE	NA	NA	NA
Bc_ctg1_2456	B4	297.5102529	Bcer	M	Bcer	FALSE	NA	NA	NA
Bc_ctg1_2457	B4	972.9902081	Bcer	H	Bcer	FALSE	NA	NA	NA
Bc_ctg1_2458	B4	635.1944334	Bcer	H	Bcer	FALSE	NA	NA	NA
Bc_ctg1_2459	B4	680.390102	Bcer	J	Bcer	FALSE	NA	NA	NA
Bc_ctg1_246	B4	3.459421545	Bcer	G	Bcer	FALSE	NA	NA	NA
Bc_ctg1_2460	B4	185.3580383	Bcer	S	Bcer	FALSE	NA	NA	NA
Bc_ctg1_2461	B4	27.67537236	Bcer	F	Bcer	FALSE	NA	NA	NA
Bc_ctg1_2462	B4	18.41305016	Bcer	F	Bcer	FALSE	NA	NA	NA
Bc_ctg1_2463	B4	0	Bcer	Q	Bcer	TRUE	NA	NA	NA
Bc_ctg1_2464	B4	6.24927763	Bcer	FJ	Bcer	FALSE	NA	NA	NA
Bc_ctg1_2465	B4	667.8915467	Bcer	L	Bcer	FALSE	NA	NA	NA
Bc_ctg1_2466	B4	131.6812072	Bcer	S	Bcer	FALSE	NA	NA	NA
Bc_ctg1_2467	B4	443.363929	Bcer	L	Bcer	FALSE	NA	NA	NA
Bc_ctg1_2468	B4	117.6203325	Bcer	S	Bcer	FALSE	NA	NA	NA
Bc_ctg1_2469	B4	0.557971217	Bcer	S	Bcer	FALSE	NA	NA	NA
Bc_ctg1_247	B4	2.231884868	Bcer	S	Bcer	FALSE	NA	NA	NA
Bc_ctg1_2471	B4	0	Bcer	S	Bcer	FALSE	NA	NA	NA
Bc_ctg1_2472	B4	73.76379488	Bcer	E	Bcer	FALSE	NA	NA	NA
Bc_ctg1_2473	B4	32.58551907	Bcer	F	Bcer	FALSE	NA	NA	NA
Bc_ctg1_2474	B4	193.8392008	Bcer	L	Bcer	FALSE	NA	NA	NA
Bc_ctg1_2475	B4	272.5131424	Bcer	S	Bcer	FALSE	NA	NA	NA
Bc_ctg1_2476	B4	59.9261087	Bcer	L	Bcer	FALSE	NA	NA	NA
Bc_ctg1_2477	B4	21.8724717	Bcer	S	Bcer	FALSE	NA	NA	NA
Bc_ctg1_2478	B4	2.789856085	Bcer	L	Bcer	FALSE	NA	NA	NA
Bc_ctg1_2479	B4	27.89856085	Bcer	H	Bcer	FALSE	NA	NA	NA
Bc_ctg1_248	B4	8.481162498	Bcer	J	Bcer	FALSE	NA	NA	NA
Bc_ctg1_2480	B4	649.0321196	Bcer	K	Bcer	FALSE	NA	NA	NA
Bc_ctg1_2481	B4	816.8698616	Bcer	J	Bcer	FALSE	NA	NA	NA
Bc_ctg1_2482	B4	44.86088584	Bcer	L	Bcer	FALSE	NA	NA	NA
Bc_ctg1_2483	B4	24.10435657	Bcer	J	Bcer	FALSE	NA	NA	NA
Bc_ctg1_2484	B4	98.09133994	Bcer	J	Bcer	FALSE	NA	NA	NA
Bc_ctg1_2485	B4	6.24927763	Bcer	S	Bcer	FALSE	NA	NA	NA
Bc_ctg1_2486	B4	0.223188487	Bcer	S	Bcer	FALSE	NA	NA	NA
Bc_ctg1_2488	B4	99.87684783	Bcer	I	Bcer	FALSE	NA	NA	NA
Bc_ctg1_2489	B4	360.6725946	Bcer	F	Bcer	FALSE	NA	NA	NA
Bc_ctg1_249	B4	46.08842252	Bcer	I	Bcer	FALSE	NA	NA	NA
Bc_ctg1_2490	B4	98.98409389	Bcer	J	Bcer	FALSE	NA	NA	NA
Bc_ctg1_2491	B4	541.7900517	Bcer	D	Bcer	FALSE	NA	NA	NA
Bc_ctg1_2492	B4	1502.839676	Bcer	M	Bcer	FALSE	NA	NA	NA
Bc_ctg1_2493	B4	1145.738097	Bcer	F	Bcer	FALSE	NA	NA	NA
Bc_ctg1_2494	B4	115.8348246	Bcer	J	Bcer	FALSE	NA	NA	NA
Bc_ctg1_2495	B4	8.257974011	Bcer	S	Bcer	FALSE	NA	NA	NA
Bc_ctg1_2496	B4	572.9248456	Bcer	L	Bcer	FALSE	NA	NA	NA
Bc_ctg1_2497	B4	107.6884449	Bcer	K	Bcer	FALSE	NA	NA	NA
Bc_ctg1_2498	B4	155.6739695	Bcer	S	Bcer	FALSE	NA	NA	NA
Bc_ctg1_2499	B4	194.9551432	Bcer	S	Bcer	FALSE	NA	NA	NA
Bc_ctg1_25	B4	2.789856085	Bcer	K	Bcer	FALSE	NA	NA	NA
Bc_ctg1_250	B4	0.111594243	Bcer	L	Bcer	FALSE	NA	NA	NA
Bc_ctg1_2500	B4	27.45218387	Bcer	J	Bcer	FALSE	NA	NA	NA

Bc_ctg1_2501	B4	60.48407992	Bcer	S	Bcer	FALSE	NA	NA	NA
Bc_ctg1_2502	B4	183.1261534	Bcer	S	Bcer	FALSE	NA	NA	NA
Bc_ctg1_2503	B4	107.6884449	Bcer	D	Bcer	FALSE	NA	NA	NA
Bc_ctg1_2504	B4	696.2364845	Bcer	J	Bcer	FALSE	NA	NA	NA
Bc_ctg1_2505	B4	6.24927763	Bcer	KT	Bcer	FALSE	NA	NA	NA
Bc_ctg1_2506	B4	2181.779053	Bcer	D	Bcer	FALSE	NA	NA	NA
Bc_ctg1_2507	B4	1048.874294	Bcer	F	Bcer	FALSE	NA	NA	NA
Bc_ctg1_2508	B4	7257.531619	Bcer	O	Bcer	FALSE	NA	NA	NA
Bc_ctg1_2509	B4	122.5304792	Bcer	F	Bcer	FALSE	NA	NA	NA
Bc_ctg1_251	B4	0.557971217	Bcer	L	Bcer	FALSE	NA	NA	NA
Bc_ctg1_2510	B4	239.4812463	Bcer	O	Bcer	FALSE	NA	NA	NA
Bc_ctg1_2511	B4	593.6813748	Bcer	E	Bcer	FALSE	NA	NA	NA
Bc_ctg1_2512	B4	154.7812156	Bcer	EH	Bcer	FALSE	NA	NA	NA
Bc_ctg1_2513	B4	94.52032415	Bcer	EH	Bcer	FALSE	NA	NA	NA
Bc_ctg1_2514	B4	88.94061198	Bcer	EH	Bcer	FALSE	NA	NA	NA
Bc_ctg1_2515	B4	78.89713008	Bcer	H	Bcer	FALSE	NA	NA	NA
Bc_ctg1_2516	B4	24.66232779	Bcer	H	Bcer	FALSE	NA	NA	NA
Bc_ctg1_2517	B4	36.49131759	Bcer	H	Bcer	FALSE	NA	NA	NA
Bc_ctg1_2518	B4	1.339130921	Bcer	K	Bcer	FALSE	NA	NA	NA
Bc_ctg1_2519	B4	72.42466396	Bcer	J	Bcer	FALSE	NA	NA	NA
Bc_ctg1_252	B4	53.90001956	Bcer	S	Bcer	FALSE	NA	NA	NA
Bc_ctg1_2520	B4	757.3901299	Bcer	J	Bcer	FALSE	NA	NA	NA
Bc_ctg1_2523	B4	82.24495738	Bcer	K	Bcer	FALSE	NA	NA	NA
Bc_ctg1_2524	B4	129.0029454	Bcer	S	Bcer	FALSE	NA	NA	NA
Bc_ctg1_2525	B4	404.9755093	Bcer	E	Bcer	FALSE	NA	NA	NA
Bc_ctg1_2526	B4	1541.897661	Bcer	O	Bcer	FALSE	NA	NA	NA
Bc_ctg1_2527	B4	80.79423221	Bcer	O	Bcer	FALSE	NA	NA	NA
Bc_ctg1_2528	B4	92.95800474	Bcer	L	Bcer	FALSE	NA	NA	NA
Bc_ctg1_2529	B4	334.8943244	Bcer	S	Bcer	FALSE	NA	NA	NA
Bc_ctg1_253	B4	18.07826743	Bcer	NA	Bcer	FALSE	NA	NA	NA
Bc_ctg1_2530	B4	335.4522956	Bcer	I	Bcer	FALSE	NA	NA	NA
Bc_ctg1_2531	B4	57.13625262	Bcer	I	Bcer	FALSE	NA	NA	NA
Bc_ctg1_2532	B4	1203.878698	Bcer	J	Bcer	FALSE	NA	NA	NA
Bc_ctg1_2533	B4	66.73335755	Bcer	E	Bcer	FALSE	NA	NA	NA
Bc_ctg1_2534	B4	280.5479279	Bcer	J	Bcer	FALSE	NA	NA	NA
Bc_ctg1_2535	B4	41.95943551	Bcer	J	Bcer	FALSE	NA	NA	NA
Bc_ctg1_2536	B4	128.6681626	Bcer	J	Bcer	FALSE	NA	NA	NA
Bc_ctg1_2537	B4	117.5087383	Bcer	S	Bcer	FALSE	NA	NA	NA
Bc_ctg1_2538	B4	221.7377616	Bcer	K	Bcer	FALSE	NA	NA	NA
Bc_ctg1_2539	B4	10.82464161	Bcer	U	Bcer	FALSE	NA	NA	NA
Bc_ctg1_254	B4	4.128987005	Bcer	K	Bcer	FALSE	NA	NA	NA
Bc_ctg1_2540	B4	264.1435741	Bcer	K	Bcer	FALSE	NA	NA	NA
Bc_ctg1_2541	B4	4259.217487	Bcer	J	Bcer	FALSE	NA	NA	NA
Bc_ctg1_2542	B4	1768.545569	Bcer	J	Bcer	FALSE	NA	NA	NA
Bc_ctg1_2543	B4	2442.909582	Bcer	J	Bcer	FALSE	NA	NA	NA
Bc_ctg1_2544	B4	849.6785692	Bcer	J	Bcer	FALSE	NA	NA	NA
Bc_ctg1_2545	B4	219.7290652	Bcer	J	Bcer	FALSE	NA	NA	NA
Bc_ctg1_2546	B4	11276.37511	Bcer	K	Bcer	FALSE	NA	NA	NA
Bc_ctg1_2547	B4	10693.40678	Bcer	K	Bcer	FALSE	NA	NA	NA
Bc_ctg1_2548	B4	629.503127	Bcer	J	Bcer	FALSE	NA	NA	NA
Bc_ctg1_2549	B4	2775.683616	Bcer	J	Bcer	FALSE	NA	NA	NA
Bc_ctg1_255	B4	0.33478273	Bcer	NA	Bcer	FALSE	NA	NA	NA
Bc_ctg1_2550	B4	1951.448534	Bcer	J	Bcer	FALSE	NA	NA	NA
Bc_ctg1_2551	B4	15053.72865	Bcer	J	Bcer	FALSE	NA	NA	NA
Bc_ctg1_2552	B4	16325.45665	Bcer	J	Bcer	FALSE	NA	NA	NA
Bc_ctg1_2553	B4	1673.35568	Bcer	J	Bcer	FALSE	NA	NA	NA
Bc_ctg1_2554	B4	3766.19412	Bcer	J	Bcer	FALSE	NA	NA	NA
Bc_ctg1_2555	B4	3750.459332	Bcer	J	Bcer	FALSE	NA	NA	NA
Bc_ctg1_2556	B4	1135.583021	Bcer	J	Bcer	FALSE	NA	NA	NA
Bc_ctg1_2557	B4	6501.592214	Bcer	J	Bcer	FALSE	NA	NA	NA
Bc_ctg1_2558	B4	2208.226888	Bcer	J	Bcer	FALSE	NA	NA	NA
Bc_ctg1_2559	B4	2761.84593	Bcer	J	Bcer	FALSE	NA	NA	NA

Bc_ctg1_256	B4	3.124638815	Bcer	Q	Bcer	TRUE	NA	NA	NA
Bc_ctg1_2560	B4	4288.789962	Bcer	J	Bcer	FALSE	NA	NA	NA
Bc_ctg1_2561	B4	2339.908095	Bcer	J	Bcer	FALSE	NA	NA	NA
Bc_ctg1_2562	B4	429.0798658	Bcer	J	Bcer	FALSE	NA	NA	NA
Bc_ctg1_2563	B4	1227.983054	Bcer	J	Bcer	FALSE	NA	NA	NA
Bc_ctg1_2564	B4	2010.147106	Bcer	J	Bcer	FALSE	NA	NA	NA
Bc_ctg1_2565	B4	2034.809434	Bcer	J	Bcer	FALSE	NA	NA	NA
Bc_ctg1_2566	B4	3810.943412	Bcer	J	Bcer	FALSE	NA	NA	NA
Bc_ctg1_2567	B4	576.9422383	Bcer	J	Bcer	FALSE	NA	NA	NA
Bc_ctg1_2568	B4	2404.409568	Bcer	J	Bcer	FALSE	NA	NA	NA
Bc_ctg1_2569	B4	3513.879536	Bcer	J	Bcer	FALSE	NA	NA	NA
Bc_ctg1_257	B4	0.111594243	Bcer	Q	Bcer	TRUE	Bc_Ga0417192_01_rn02	Other	NA
Bc_ctg1_2570	B4	1737.187587	Bcer	J	Bcer	FALSE	NA	NA	NA
Bc_ctg1_2571	B4	3957.355059	Bcer	J	Bcer	FALSE	NA	NA	NA
Bc_ctg1_2572	B4	181.0058628	Bcer	J	Bcer	FALSE	NA	NA	NA
Bc_ctg1_2573	B4	2576.041514	Bcer	J	Bcer	FALSE	NA	NA	NA
Bc_ctg1_2574	B4	9846.406471	Bcer	U	Bcer	FALSE	NA	NA	NA
Bc_ctg1_2575	B4	3125.754757	Bcer	F	Bcer	FALSE	NA	NA	NA
Bc_ctg1_2576	B4	5247.272918	Bcer	E	Bcer	FALSE	NA	NA	NA
Bc_ctg1_2577	B4	546.8117926	Bcer	J	Bcer	FALSE	NA	NA	NA
Bc_ctg1_2578	B4	0.223188487	Bcer	J	Bcer	FALSE	NA	NA	NA
Bc_ctg1_2579	B4	3527.159251	Bcer	J	Bcer	FALSE	NA	NA	NA
Bc_ctg1_258	B4	13.94928042	Bcer	S	Bcer	FALSE	Bc_Ga0417192_01_rn02	Other	SMCOG1272
Bc_ctg1_2580	B4	2013.160151	Bcer	J	Bcer	FALSE	NA	NA	NA
Bc_ctg1_2581	B4	6954.888031	Bcer	K	Bcer	FALSE	NA	NA	NA
Bc_ctg1_2582	B4	1018.074282	Bcer	J	Bcer	FALSE	NA	NA	NA
Bc_ctg1_2583	B4	165.1594802	Bcer	P	Bcer	FALSE	NA	NA	NA
Bc_ctg1_2584	B4	89.05220623	Bcer	P	Bcer	FALSE	NA	NA	NA
Bc_ctg1_2585	B4	61.04205113	Bcer	P	Bcer	FALSE	NA	NA	NA
Bc_ctg1_2586	B4	80.79423221	Bcer	J	Bcer	FALSE	NA	NA	NA
Bc_ctg1_2587	B4	2266.813866	Bcer	J	Bcer	FALSE	NA	NA	NA
Bc_ctg1_2588	B4	921.0988849	Bcer	J	Bcer	FALSE	NA	NA	NA
Bc_ctg1_2589	B4	1.004348191	Bcer	S	Bcer	FALSE	NA	NA	NA
Bc_ctg1_259	B4	1.004348191	Bcer	NA	Bcer	FALSE	Bc_Ga0417192_01_rn02	Other	NA
Bc_ctg1_2590	B4	2.789856085	Bcer	M	Bcer	FALSE	NA	NA	NA
Bc_ctg1_2591	B4	521.2567109	Bcer	D	Bcer	FALSE	NA	NA	NA
Bc_ctg1_2592	B4	0.223188487	Bcer	S	Bcer	FALSE	NA	NA	NA
Bc_ctg1_2593	B4	70.19277909	Bcer	S	Bcer	FALSE	NA	NA	NA
Bc_ctg1_2594	B4	0.66956546	Bcer	G	Bcer	FALSE	NA	NA	NA
Bc_ctg1_2595	B4	0.33478273	Bcer	NA	Bcer	FALSE	NA	NA	NA
Bc_ctg1_2597	B4	1.673913651	Bcer	G	Bcer	FALSE	NA	NA	NA
Bc_ctg1_2598	B4	281.3290876	Bcer	E	Bcer	FALSE	NA	NA	NA
Bc_ctg1_2599	B4	165.2710745	Bcer	S	Bcer	FALSE	NA	NA	NA
Bc_ctg1_26	B4	137.5957021	Bcer	U	Bcer	FALSE	NA	NA	NA
Bc_ctg1_260	B4	1.115942434	Bcer	S	Bcer	FALSE	Bc_Ga0417192_01_rn02	Other	NA
Bc_ctg1_2600	B4	339.1349057	Bcer	S	Bcer	FALSE	NA	NA	NA
Bc_ctg1_2601	B4	632.0697946	Bcer	G	Bcer	FALSE	NA	NA	NA
Bc_ctg1_2602	B4	1158.906218	Bcer	M	Bcer	FALSE	NA	NA	NA
Bc_ctg1_2603	B4	9.373916445	Bcer	EU	Bcer	FALSE	NA	NA	NA
Bc_ctg1_2604	B4	28.79131479	Bcer	S	Bcer	FALSE	NA	NA	NA
Bc_ctg1_2605	B4	348.3972279	Bcer	S	Bcer	FALSE	NA	NA	NA
Bc_ctg1_2606	B4	68.07248847	Bcer	S	Bcer	FALSE	NA	NA	NA
Bc_ctg1_2607	B4	140.2739639	Bcer	IQ	Bcer	TRUE	NA	NA	NA
Bc_ctg1_2608	B4	3.571015789	Bcer	P	Bcer	FALSE	NA	NA	NA
Bc_ctg1_2609	B4	5.691306413	Bcer	P	Bcer	FALSE	NA	NA	NA
Bc_ctg1_261	B4	21.53768897	Bcer	S	Bcer	FALSE	Bc_Ga0417192_01_rn02	Other	NA
Bc_ctg1_2610	B4	3.347827302	Bcer	P	Bcer	FALSE	NA	NA	NA
Bc_ctg1_2611	B4	0.33478273	Bcer	C	Bcer	FALSE	NA	NA	NA
Bc_ctg1_2612	B4	21.091312	Bcer	NA	Bcer	FALSE	NA	NA	NA
Bc_ctg1_2613	B4	8.257974011	Bcer	C	Bcer	FALSE	NA	NA	NA
Bc_ctg1_2614	B4	2.789856085	Bcer	G	Bcer	FALSE	NA	NA	NA
Bc_ctg1_2615	B4	57.91741232	Bcer	S	Bcer	FALSE	NA	NA	NA

Bc_ctg1_2616	B4	16.18116529	Bcer	EGP	Bcer	FALSE	NA	NA	NA
Bc_ctg1_2617	B4	3.124638815	Bcer	NA	Bcer	FALSE	NA	NA	NA
Bc_ctg1_2618	B4	20.19855805	Bcer	NA	Bcer	FALSE	NA	NA	NA
Bc_ctg1_2619	B4	0.223188487	Bcer	S	Bcer	FALSE	NA	NA	NA
Bc_ctg1_262	B4	19.52899259	Bcer	S	Bcer	FALSE	Bc_Ga0417192_01_rn02	Other	NA
Bc_ctg1_2620	B4	2.008696381	Bcer	P	Bcer	FALSE	NA	NA	NA
Bc_ctg1_2621	B4	2.455073355	Bcer	EP	Bcer	FALSE	NA	NA	NA
Bc_ctg1_2622	B4	3.794204275	Bcer	P	Bcer	FALSE	NA	NA	NA
Bc_ctg1_2623	B4	3.682610032	Bcer	E	Bcer	FALSE	NA	NA	NA
Bc_ctg1_2624	B4	12.05217829	Bcer	E	Bcer	FALSE	NA	NA	NA
Bc_ctg1_2625	B4	0	Bcer	S	Bcer	FALSE	NA	NA	NA
Bc_ctg1_2626	B4	0.111594243	Bcer	Q	Bcer	TRUE	NA	NA	NA
Bc_ctg1_2627	B4	166.2754227	Bcer	E	Bcer	FALSE	NA	NA	NA
Bc_ctg1_2628	B4	170.6275981	Bcer	E	Bcer	FALSE	NA	NA	NA
Bc_ctg1_2629	B4	132.5739611	Bcer	S	Bcer	FALSE	NA	NA	NA
Bc_ctg1_263	B4	237.3609557	Bcer	S	Bcer	FALSE	Bc_Ga0417192_01_rn02	Other	SMCOG1273
Bc_ctg1_2630	B4	105.1217773	Bcer	E	Bcer	FALSE	NA	NA	NA
Bc_ctg1_2631	B4	4.352175492	Bcer	H	Bcer	FALSE	NA	NA	NA
Bc_ctg1_2632	B4	58.81016627	Bcer	U	Bcer	FALSE	NA	NA	NA
Bc_ctg1_2633	B4	73.98698337	Bcer	P	Bcer	FALSE	NA	NA	NA
Bc_ctg1_2634	B4	106.1261255	Bcer	P	Bcer	FALSE	NA	NA	NA
Bc_ctg1_2635	B4	56.68987564	Bcer	P	Bcer	FALSE	NA	NA	NA
Bc_ctg1_2638	B4	7.25362582	Bcer	K	Bcer	FALSE	NA	NA	NA
Bc_ctg1_2639	B4	2.343479111	Bcer	EG	Bcer	FALSE	NA	NA	NA
Bc_ctg1_264	B4	56.91306413	Bcer	K	Bcer	FALSE	Bc_Ga0417192_01_rn02	Regulation	SMCOG1171
Bc_ctg1_2642	B4	45.97682828	Bcer	I	Bcer	FALSE	NA	NA	NA
Bc_ctg1_2644	B4	17.52029621	Bcer	K	Bcer	FALSE	NA	NA	NA
Bc_ctg1_2645	B4	1.450725164	Bcer	NA	Bcer	FALSE	NA	NA	NA
Bc_ctg1_2646	B4	64.72466117	Bcer	S	Bcer	FALSE	NA	NA	NA
Bc_ctg1_2647	B4	0	Bcer	S	Bcer	FALSE	NA	NA	NA
Bc_ctg1_2648	B4	0	Bcer	NA	Bcer	FALSE	NA	NA	NA
Bc_ctg1_2649	B4	6.026089143	Bcer	L	Bcer	FALSE	NA	NA	NA
Bc_ctg1_265	B4	0	Bcer	NA	Bcer	FALSE	Bc_Ga0417192_01_rn02	Other	NA
Bc_ctg1_2650	B4	29.01450328	Bcer	S	Bcer	FALSE	NA	NA	NA
Bc_ctg1_2651	B4	56.13190443	Bcer	M	Bcer	FALSE	NA	NA	NA
Bc_ctg1_2652	B4	11.38261283	Bcer	C	Bcer	FALSE	NA	NA	NA
Bc_ctg1_2654	B4	16.29275954	Bcer	S	Bcer	FALSE	NA	NA	NA
Bc_ctg1_2655	B4	0.33478273	Bcer	NA	Bcer	FALSE	NA	NA	NA
Bc_ctg1_2656	B4	99.31887662	Bcer	E	Bcer	FALSE	NA	NA	NA
Bc_ctg1_2657	B4	9.931887662	Bcer	P	Bcer	FALSE	NA	NA	NA
Bc_ctg1_2658	B4	12.05217829	Bcer	EP	Bcer	FALSE	NA	NA	NA
Bc_ctg1_2659	B4	26.11305295	Bcer	P	Bcer	FALSE	NA	NA	NA
Bc_ctg1_266	B4	0	Bcer	JM	Bcer	FALSE	Bc_Ga0417192_01_rn02	Ancillary biosynthesis	SMCOG1064
Bc_ctg1_2660	B4	28.45653206	Bcer	E	Bcer	FALSE	NA	NA	NA
Bc_ctg1_2661	B4	204.3290596	Bcer	S	Bcer	FALSE	NA	NA	NA
Bc_ctg1_2663	B4	0	Bcer	NA	Bcer	FALSE	NA	NA	NA
Bc_ctg1_2664	B4	0	Bcer	NA	Bcer	FALSE	NA	NA	NA
Bc_ctg1_2665	B4	0	Bcer	NA	Bcer	FALSE	NA	NA	NA
Bc_ctg1_2667	B4	98.64931116	Bcer	E	Bcer	FALSE	NA	NA	NA
Bc_ctg1_2668	B4	50.10581528	Bcer	Q	Bcer	TRUE	NA	NA	NA
Bc_ctg1_2669	B4	134.4710633	Bcer	Q	Bcer	TRUE	NA	NA	NA
Bc_ctg1_267	B4	0.223188487	Bcer	GM	Bcer	FALSE	Bc_Ga0417192_01_rn02	Core biosynthesis	SMCOG1010
Bc_ctg1_2670	B4	3.347827302	Bcer	E	Bcer	FALSE	NA	NA	NA
Bc_ctg1_2671	B4	8.369568254	Bcer	EGP	Bcer	FALSE	NA	NA	NA
Bc_ctg1_2672	B4	226.2015314	Bcer	F	Bcer	FALSE	NA	NA	NA
Bc_ctg1_2673	B4	225.3087774	Bcer	M	Bcer	FALSE	NA	NA	NA
Bc_ctg1_2674	B4	1718.774537	Bcer	JKL	Bcer	FALSE	NA	NA	NA
Bc_ctg1_2675	B4	3.794204275	Bcer	L	Bcer	FALSE	NA	NA	NA
Bc_ctg1_2676	B4	0.111594243	Bcer	S	Bcer	FALSE	NA	NA	NA
Bc_ctg1_2677	B4	5.691306413	Bcer	I	Bcer	FALSE	NA	NA	NA
Bc_ctg1_2678	B4	33.7014615	Bcer	M	Bcer	FALSE	NA	NA	NA
Bc_ctg1_2679	B4	18.63623865	Bcer	E	Bcer	FALSE	NA	NA	NA

Bc_ctg1_268	B4	0	Bcer	H	Bcer	FALSE	Bc_Ga0417192_01_rn02	Other	NA
Bc_ctg1_2680	B4	508.2001844	Bcer	K	Bcer	FALSE	NA	NA	NA
Bc_ctg1_2681	B4	586.874126	Bcer	L	Bcer	FALSE	NA	NA	NA
Bc_ctg1_2682	B4	177.3232527	Bcer	K	Bcer	FALSE	NA	NA	NA
Bc_ctg1_2683	B4	4.240581249	Bcer	S	Bcer	FALSE	NA	NA	NA
Bc_ctg1_2685	B4	12.72174375	Bcer	S	Bcer	FALSE	NA	NA	NA
Bc_ctg1_2686	B4	33.36667877	Bcer	O	Bcer	FALSE	NA	NA	NA
Bc_ctg1_2687	B4	10.93623585	Bcer	K	Bcer	FALSE	NA	NA	NA
Bc_ctg1_2688	B4	76.44205672	Bcer	O	Bcer	FALSE	NA	NA	NA
Bc_ctg1_2689	B4	236.8029845	Bcer	S	Bcer	FALSE	NA	NA	NA
Bc_ctg1_269	B4	0	Bcer	GM	Bcer	FALSE	Bc_Ga0417192_01_rn02	Core biosynthesis	SMCOG1010
Bc_ctg1_2690	B4	783.2799944	Bcer	K	Bcer	FALSE	NA	NA	NA
Bc_ctg1_2691	B4	6.472466117	Bcer	S	Bcer	FALSE	NA	NA	NA
Bc_ctg1_2692	B4	109.585547	Bcer	S	Bcer	FALSE	NA	NA	NA
Bc_ctg1_2693	B4	393.4813022	Bcer	O	Bcer	FALSE	NA	NA	NA
Bc_ctg1_2694	B4	3071.073578	Bcer	O	Bcer	FALSE	NA	NA	NA
Bc_ctg1_2695	B4	55.90871594	Bcer	S	Bcer	FALSE	NA	NA	NA
Bc_ctg1_2696	B4	31.02319966	Bcer	S	Bcer	FALSE	NA	NA	NA
Bc_ctg1_2697	B4	1385.554126	Bcer	F	Bcer	FALSE	NA	NA	NA
Bc_ctg1_2698	B4	521.1451166	Bcer	S	Bcer	FALSE	NA	NA	NA
Bc_ctg1_2699	B4	4.128987005	Bcer	T	Bcer	FALSE	NA	NA	NA
Bc_ctg1_27	B4	77.89278189	Bcer	P	Bcer	FALSE	NA	NA	NA
Bc_ctg1_270	B4	1.004348191	Bcer	NA	Bcer	FALSE	Bc_Ga0417192_01_rn02	Other	NA
Bc_ctg1_2700	B4	30.46522845	Bcer	T	Bcer	FALSE	NA	NA	NA
Bc_ctg1_2702	B4	18.63623865	Bcer	S	Bcer	FALSE	NA	NA	NA
Bc_ctg1_2703	B4	26.55942993	Bcer	Q	Bcer	TRUE	NA	NA	NA
Bc_ctg1_2705	B4	12.72174375	Bcer	S	Bcer	FALSE	NA	NA	NA
Bc_ctg1_2707	B4	6.58406036	Bcer	V	Bcer	FALSE	NA	NA	NA
Bc_ctg1_2708	B4	3.236233058	Bcer	S	Bcer	FALSE	NA	NA	NA
Bc_ctg1_2709	B4	2.901450328	Bcer	V	Bcer	FALSE	NA	NA	NA
Bc_ctg1_271	B4	0	Bcer	S	Bcer	FALSE	Bc_Ga0417192_01_rn02	Other	NA
Bc_ctg1_2710	B4	3.124638815	Bcer	T	Bcer	FALSE	NA	NA	NA
Bc_ctg1_2711	B4	2.008696381	Bcer	T	Bcer	FALSE	NA	NA	NA
Bc_ctg1_2713	B4	0.33478273	Bcer	S	Bcer	FALSE	Bc_Ga0417192_01_rn18	Other	NA
Bc_ctg1_2714	B4	0.446376974	Bcer	Q	Bcer	TRUE	Bc_Ga0417192_01_rn18	Ancillary biosynthesis	SMCOG1089
Bc_ctg1_2715	B4	0	Bcer	Q	Bcer	TRUE	Bc_Ga0417192_01_rn18	Other	NA
Bc_ctg1_2716	B4	0.446376974	Bcer	G	Bcer	FALSE	Bc_Ga0417192_01_rn18	Other	NA
Bc_ctg1_2717	B4	3.571015789	Bcer	M	Bcer	FALSE	Bc_Ga0417192_01_rn18	Ancillary biosynthesis	SMCOG1010
Bc_ctg1_2718	B4	0.781159704	Bcer	M	Bcer	FALSE	Bc_Ga0417192_01_rn18	Other	NA
Bc_ctg1_2719	B4	2.566667598	Bcer	M	Bcer	FALSE	Bc_Ga0417192_01_rn18	Core biosynthesis	SMCOG1045
Bc_ctg1_272	B4	43.9681319	Bcer	EG	Bcer	FALSE	Bc_Ga0417192_01_rn02	Transport	SMCOG1166
Bc_ctg1_2721	B4	25.77827022	Bcer	EG	Bcer	FALSE	Bc_Ga0417192_01_rn18	Transport	SMCOG1166
Bc_ctg1_2722	B4	0.223188487	Bcer	G	Bcer	FALSE	Bc_Ga0417192_01_rn18	Other	NA
Bc_ctg1_2724	B4	270.058069	Bcer	F	Bcer	FALSE	NA	NA	NA
Bc_ctg1_2725	B4	1270.723649	Bcer	F	Bcer	FALSE	NA	NA	NA
Bc_ctg1_2726	B4	1785.284706	Bcer	F	Bcer	FALSE	NA	NA	NA
Bc_ctg1_2727	B4	266.4870532	Bcer	F	Bcer	FALSE	NA	NA	NA
Bc_ctg1_2728	B4	88.82901774	Bcer	F	Bcer	FALSE	NA	NA	NA
Bc_ctg1_2729	B4	468.2494453	Bcer	F	Bcer	FALSE	NA	NA	NA
Bc_ctg1_273	B4	4.575363979	Bcer	S	Bcer	FALSE	Bc_Ga0417192_01_rn02	Other	SMCOG1286
Bc_ctg1_2730	B4	2064.270314	Bcer	F	Bcer	FALSE	NA	NA	NA
Bc_ctg1_2731	B4	1543.013603	Bcer	F	Bcer	FALSE	NA	NA	NA
Bc_ctg1_2732	B4	541.5668632	Bcer	F	Bcer	FALSE	NA	NA	NA
Bc_ctg1_2733	B4	209.6855833	Bcer	F	Bcer	FALSE	NA	NA	NA
Bc_ctg1_2734	B4	1244.499002	Bcer	F	Bcer	FALSE	NA	NA	NA
Bc_ctg1_2735	B4	575.3799189	Bcer	F	Bcer	FALSE	NA	NA	NA
Bc_ctg1_2736	B4	5.244929439	Bcer	EGP	Bcer	FALSE	NA	NA	NA
Bc_ctg1_2737	B4	6.58406036	Bcer	K	Bcer	FALSE	NA	NA	NA
Bc_ctg1_2738	B4	8.034785524	Bcer	S	Bcer	FALSE	NA	NA	NA
Bc_ctg1_2739	B4	168.1725248	Bcer	O	Bcer	FALSE	NA	NA	NA
Bc_ctg1_274	B4	4.240581249	Bcer	S	Bcer	FALSE	Bc_Ga0417192_01_rn02	Other	NA
Bc_ctg1_2740	B4	0.111594243	Bcer	S	Bcer	FALSE	NA	NA	NA

Bc_ctg1_2741	B4	21.98406595	Bcer	I	Bcer	FALSE	NA	NA	NA
Bc_ctg1_2742	B4	231.3348665	Bcer	L	Bcer	FALSE	NA	NA	NA
Bc_ctg1_2743	B4	376.6305714	Bcer	L	Bcer	FALSE	NA	NA	NA
Bc_ctg1_2744	B4	225.1971832	Bcer	S	Bcer	FALSE	NA	NA	NA
Bc_ctg1_2745	B4	18.5246444	Bcer	S	Bcer	FALSE	NA	NA	NA
Bc_ctg1_2746	B4	8023.514506	Bcer	C	Bcer	FALSE	NA	NA	NA
Bc_ctg1_2747	B4	5.356523683	Bcer	Q	Bcer	TRUE	NA	NA	NA
Bc_ctg1_2748	B4	28.01015509	Bcer	P	Bcer	FALSE	NA	NA	NA
Bc_ctg1_2749	B4	9.931887662	Bcer	P	Bcer	FALSE	NA	NA	NA
Bc_ctg1_275	B4	61.48842811	Bcer	G	Bcer	FALSE	Bc_Ga0417192_01_rn02	Other	NA
Bc_ctg1_2750	B4	21.42609473	Bcer	M	Bcer	FALSE	NA	NA	NA
Bc_ctg1_2751	B4	0.446376974	Bcer	S	Bcer	FALSE	NA	NA	NA
Bc_ctg1_2752	B4	8.034785524	Bcer	D	Bcer	FALSE	NA	NA	NA
Bc_ctg1_2753	B4	22.98841414	Bcer	K	Bcer	FALSE	NA	NA	NA
Bc_ctg1_2754	B4	4.352175492	Bcer	S	Bcer	FALSE	NA	NA	NA
Bc_ctg1_2755	B4	162.2580299	Bcer	J	Bcer	FALSE	NA	NA	NA
Bc_ctg1_2756	B4	986.1583288	Bcer	J	Bcer	FALSE	NA	NA	NA
Bc_ctg1_2757	B4	1222.514936	Bcer	J	Bcer	FALSE	NA	NA	NA
Bc_ctg1_2758	B4	201.5392036	Bcer	I	Bcer	FALSE	NA	NA	NA
Bc_ctg1_2759	B4	5.244929439	Bcer	S	Bcer	FALSE	NA	NA	NA
Bc_ctg1_276	B4	38.50001397	Bcer	G	Bcer	FALSE	Bc_Ga0417192_01_rn02	Ancillary biosynthesis	SMCOG1210
Bc_ctg1_2760	B4	0.446376974	Bcer	E	Bcer	FALSE	NA	NA	NA
Bc_ctg1_2761	B4	8.257974011	Bcer	KT	Bcer	FALSE	NA	NA	NA
Bc_ctg1_2762	B4	74.76814307	Bcer	C	Bcer	FALSE	NA	NA	NA
Bc_ctg1_2763	B4	9.820293418	Bcer	P	Bcer	FALSE	NA	NA	NA
Bc_ctg1_2764	B4	327.8638871	Bcer	E	Bcer	FALSE	NA	NA	NA
Bc_ctg1_2765	B4	48.65509012	Bcer	G	Bcer	FALSE	Bc_Ga0417192_01_rn19	Other	NA
Bc_ctg1_2766	B4	0.892753947	Bcer	NA	Bcer	FALSE	Bc_Ga0417192_01_rn19	Other	NA
Bc_ctg1_2767	B4	6.472466117	Bcer	NA	Bcer	FALSE	Bc_Ga0417192_01_rn19	Other	NA
Bc_ctg1_2768	B4	14.73044013	Bcer	F	Bcer	FALSE	Bc_Ga0417192_01_rn19	Other	NA
Bc_ctg1_2769	B4	71.53191001	Bcer	J	Bcer	FALSE	Bc_Ga0417192_01_rn19	Other	NA
Bc_ctg1_277	B4	17.29710773	Bcer	EG	Bcer	FALSE	Bc_Ga0417192_01_rn02	Other	NA
Bc_ctg1_2770	B4	75.32611429	Bcer	J	Bcer	FALSE	Bc_Ga0417192_01_rn19	Other	NA
Bc_ctg1_2771	B4	20.97971776	Bcer	V	Bcer	FALSE	Bc_Ga0417192_01_rn19	Transport	SMCOG1288
Bc_ctg1_2772	B4	45.41885706	Bcer	KLT	Bcer	FALSE	Bc_Ga0417192_01_rn19	Core biosynthesis	SMCOG1030
Bc_ctg1_278	B4	42.51740673	Bcer	G	Bcer	FALSE	Bc_Ga0417192_01_rn02	Other	NA
Bc_ctg1_2785	B4	1.785507894	Bcer	S	Bcer	FALSE	Bc_Ga0417192_01_rn19	Other	NA
Bc_ctg1_2786	B4	2.120290624	Bcer	S	Bcer	FALSE	Bc_Ga0417192_01_rn19	Other	NA
Bc_ctg1_2787	B4	20.3101523	Bcer	J	Bcer	FALSE	Bc_Ga0417192_01_rn19	Other	NA
Bc_ctg1_2788	B4	25.77827022	Bcer	M	Bcer	FALSE	Bc_Ga0417192_01_rn19	Ancillary biosynthesis	SMCOG1268
Bc_ctg1_2789	B4	7.700002794	Bcer	KT	Bcer	FALSE	Bc_Ga0417192_01_rn19	Other	NA
Bc_ctg1_279	B4	48.98987285	Bcer	G	Bcer	FALSE	Bc_Ga0417192_01_rn02	Ancillary biosynthesis	SMCOG1100
Bc_ctg1_2790	B4	7.142031577	Bcer	C	Bcer	FALSE	Bc_Ga0417192_01_rn19	Other	NA
Bc_ctg1_2791	B4	61.15364538	Bcer	S	Bcer	FALSE	Bc_Ga0417192_01_rn19	Other	NA
Bc_ctg1_2792	B4	9.150727958	Bcer	M	Bcer	FALSE	NA	NA	NA
Bc_ctg1_2795	B4	6409.192181	Bcer	O	Bcer	FALSE	NA	NA	NA
Bc_ctg1_2796	B4	2834.493782	Bcer	O	Bcer	FALSE	NA	NA	NA
Bc_ctg1_2797	B4	131.6812072	Bcer	F	Bcer	FALSE	NA	NA	NA
Bc_ctg1_2798	B4	171.7435406	Bcer	E	Bcer	FALSE	NA	NA	NA
Bc_ctg1_2799	B4	113.6029398	Bcer	G	Bcer	FALSE	NA	NA	NA
Bc_ctg1_28	B4	15.84638256	Bcer	K	Bcer	FALSE	NA	NA	NA
Bc_ctg1_280	B4	108.3580103	Bcer	G	Bcer	FALSE	NA	NA	NA
Bc_ctg1_2800	B4	0.223188487	Bcer	NA	Bcer	FALSE	NA	NA	NA
Bc_ctg1_2801	B4	12.72174375	Bcer	P	Bcer	FALSE	NA	NA	NA
Bc_ctg1_2802	B4	8.257974011	Bcer	P	Bcer	FALSE	NA	NA	NA
Bc_ctg1_2803	B4	88.04785804	Bcer	P	Bcer	FALSE	NA	NA	NA
Bc_ctg1_2804	B4	99.31887662	Bcer	C	Bcer	FALSE	NA	NA	NA
Bc_ctg1_2805	B4	116.5043901	Bcer	NA	Bcer	FALSE	NA	NA	NA
Bc_ctg1_2806	B4	1.339130921	Bcer	G	Bcer	FALSE	NA	NA	NA
Bc_ctg1_2807	B4	0.223188487	Bcer	NA	Bcer	FALSE	NA	NA	NA
Bc_ctg1_2808	B4	0	Bcer	NA	Bcer	FALSE	NA	NA	NA
Bc_ctg1_2809	B4	2.901450328	Bcer	NA	Bcer	FALSE	NA	NA	NA

Bc_ctg1_281	B4	55.35074472	Bcer	G	Bcer	FALSE	NA	NA	NA
Bc_ctg1_2810	B4	2.231884868	Bcer	E	Bcer	FALSE	NA	NA	NA
Bc_ctg1_2811	B4	23.76957384	Bcer	T	Bcer	FALSE	NA	NA	NA
Bc_ctg1_2812	B4	7.588408551	Bcer	S	Bcer	FALSE	NA	NA	NA
Bc_ctg1_2813	B4	11.49420707	Bcer	S	Bcer	FALSE	NA	NA	NA
Bc_ctg1_2814	B4	1.115942434	Bcer	F	Bcer	FALSE	NA	NA	NA
Bc_ctg1_2815	B4	49.8826268	Bcer	S	Bcer	FALSE	NA	NA	NA
Bc_ctg1_2816	B4	3.571015789	Bcer	EGP	Bcer	FALSE	NA	NA	NA
Bc_ctg1_2817	B4	0.892753947	Bcer	CE	Bcer	FALSE	NA	NA	NA
Bc_ctg1_2818	B4	686.9741623	Bcer	I	Bcer	FALSE	NA	NA	NA
Bc_ctg1_2819	B4	1.339130921	Bcer	P	Bcer	FALSE	NA	NA	NA
Bc_ctg1_282	B4	0	Bcer	G	Bcer	FALSE	NA	NA	NA
Bc_ctg1_2820	B4	2.455073355	Bcer	ET	Bcer	FALSE	NA	NA	NA
Bc_ctg1_2821	B4	1.562319407	Bcer	E	Bcer	FALSE	NA	NA	NA
Bc_ctg1_2822	B4	48.0971189	Bcer	NT	Bcer	FALSE	NA	NA	NA
Bc_ctg1_2823	B4	31.35798239	Bcer	K	Bcer	FALSE	NA	NA	NA
Bc_ctg1_2824	B4	7347.922956	Bcer	E	Bcer	FALSE	NA	NA	NA
Bc_ctg1_2825	B4	3760.614408	Bcer	E	Bcer	FALSE	NA	NA	NA
Bc_ctg1_2826	B4	5516.661422	Bcer	E	Bcer	FALSE	NA	NA	NA
Bc_ctg1_2827	B4	2933.031499	Bcer	E	Bcer	FALSE	Bc_Ga0417192_01_rn20	Other	NA
Bc_ctg1_2828	B4	217.3855861	Bcer	K	Bcer	FALSE	Bc_Ga0417192_01_rn20	Regulation	SMCOG1133
Bc_ctg1_2829	B4	26.89421266	Bcer	NA	Bcer	FALSE	Bc_Ga0417192_01_rn20	Other	NA
Bc_ctg1_283	B4	65.17103814	Bcer	C	Bcer	FALSE	NA	NA	NA
Bc_ctg1_2830	B4	0	Bcer	C	Bcer	FALSE	Bc_Ga0417192_01_rn20	Other	NA
Bc_ctg1_2831	B4	198.972536	Bcer	G	Bcer	FALSE	Bc_Ga0417192_01_rn20	Ancillary biosynthesis	SMCOG1257
Bc_ctg1_2832	B4	231.2232723	Bcer	G	Bcer	FALSE	Bc_Ga0417192_01_rn20	Ancillary biosynthesis	SMCOG1145
Bc_ctg1_2833	B4	110.7014894	Bcer	L	Bcer	FALSE	Bc_Ga0417192_01_rn20	Other	NA
Bc_ctg1_2834	B4	249.1899455	Bcer	S	Bcer	FALSE	Bc_Ga0417192_01_rn20	Other	NA
Bc_ctg1_2835	B4	244.5029873	Bcer	L	Bcer	FALSE	Bc_Ga0417192_01_rn20	Other	NA
Bc_ctg1_2836	B4	17.18551348	Bcer	H	Bcer	FALSE	Bc_Ga0417192_01_rn20	Other	NA
Bc_ctg1_2837	B4	8.704350984	Bcer	H	Bcer	FALSE	Bc_Ga0417192_01_rn20	Ancillary biosynthesis	SMCOG1280
Bc_ctg1_2838	B4	14.06087467	Bcer	S	Bcer	FALSE	Bc_Ga0417192_01_rn20	Other	NA
Bc_ctg1_2839	B4	54.45799077	Bcer	NT	Bcer	FALSE	Bc_Ga0417192_01_rn20	Other	SMCOG1061
Bc_ctg1_284	B4	9.373916445	Bcer	E	Bcer	FALSE	NA	NA	NA
Bc_ctg1_2840	B4	15.73478832	Bcer	Q	Bcer	TRUE	Bc_Ga0417192_01_rn20	Core biosynthesis	SMCOG1002
Bc_ctg1_2841	B4	25.55508174	Bcer	S	Bcer	FALSE	Bc_Ga0417192_01_rn20	Other	NA
Bc_ctg1_2842	B4	84.81162498	Bcer	P	Bcer	FALSE	Bc_Ga0417192_01_rn20	Transport	SMCOG1044
Bc_ctg1_2843	B4	80.57104373	Bcer	P	Bcer	FALSE	Bc_Ga0417192_01_rn20	Other	NA
Bc_ctg1_2844	B4	99.87684783	Bcer	P	Bcer	FALSE	Bc_Ga0417192_01_rn20	Transport	SMCOG1000
Bc_ctg1_2845	B4	5.57971217	Bcer	S	Bcer	FALSE	Bc_Ga0417192_01_rn20	Transport	SMCOG1000
Bc_ctg1_2846	B4	3.013044572	Bcer	G	Bcer	FALSE	Bc_Ga0417192_01_rn20	Other	NA
Bc_ctg1_2847	B4	6.137683386	Bcer	S	Bcer	FALSE	Bc_Ga0417192_01_rn20	Other	NA
Bc_ctg1_2848	B4	11.71739556	Bcer	CO	Bcer	FALSE	Bc_Ga0417192_01_rn20	Other	NA
Bc_ctg1_2849	B4	3.905798519	Bcer	NA	Bcer	FALSE	Bc_Ga0417192_01_rn20	Other	NA
Bc_ctg1_285	B4	7.811597037	Bcer	S	Bcer	FALSE	NA	NA	NA
Bc_ctg1_2852	B4	9.485510688	Bcer	K	Bcer	FALSE	Bc_Ga0417192_01_rn20	Regulation	SMCOG1057
Bc_ctg1_2853	B4	0.446376974	Bcer	G	Bcer	FALSE	Bc_Ga0417192_01_rn20	Transport	SMCOG1020
Bc_ctg1_2854	B4	1.450725164	Bcer	K	Bcer	FALSE	Bc_Ga0417192_01_rn20	Other	NA
Bc_ctg1_2855	B4	31.69276512	Bcer	S	Bcer	FALSE	Bc_Ga0417192_01_rn20	Transport	SMCOG1106
Bc_ctg1_2856	B4	27.67537236	Bcer	S	Bcer	FALSE	Bc_Ga0417192_01_rn20	Other	NA
Bc_ctg1_2857	B4	11.0478301	Bcer	S	Bcer	FALSE	Bc_Ga0417192_01_rn20	Other	NA
Bc_ctg1_2858	B4	204.3290596	Bcer	J	Bcer	FALSE	Bc_Ga0417192_01_rn20	Other	SMCOG1162
Bc_ctg1_2859	B4	0.111594243	Bcer	NU	Bcer	FALSE	Bc_Ga0417192_01_rn20	Other	NA
Bc_ctg1_286	B4	0.892753947	Bcer	S	Bcer	FALSE	NA	NA	NA
Bc_ctg1_2860	B4	1.785507894	Bcer	GK	Bcer	FALSE	Bc_Ga0417192_01_rn20	Other	SMCOG1108
Bc_ctg1_2861	B4	460.4378482	Bcer	T	Bcer	FALSE	Bc_Ga0417192_01_rn20	Other	SMCOG1296
Bc_ctg1_2862	B4	771.1162218	Bcer	T	Bcer	FALSE	Bc_Ga0417192_01_rn20	Other	SMCOG1296
Bc_ctg1_2863	B4	164.0435378	Bcer	T	Bcer	FALSE	NA	NA	NA
Bc_ctg1_2864	B4	381.0943412	Bcer	P	Bcer	FALSE	NA	NA	NA
Bc_ctg1_2865	B4	204.1058712	Bcer	O	Bcer	FALSE	NA	NA	NA
Bc_ctg1_2866	B4	179.8899203	Bcer	P	Bcer	FALSE	NA	NA	NA
Bc_ctg1_2867	B4	908.1539527	Bcer	P	Bcer	FALSE	NA	NA	NA

Bc_ctg1_2868	B4	11.49420707	Bcer	S	Bcer	FALSE	NA	NA	NA
Bc_ctg1_2869	B4	33.14349029	Bcer	T	Bcer	FALSE	NA	NA	NA
Bc_ctg1_287	B4	1.004348191	Bcer	S	Bcer	FALSE	NA	NA	NA
Bc_ctg1_2870	B4	96.86380326	Bcer	NA	Bcer	FALSE	NA	NA	NA
Bc_ctg1_2871	B4	161.7000587	Bcer	S	Bcer	FALSE	NA	NA	NA
Bc_ctg1_2872	B4	97.64496297	Bcer	P	Bcer	FALSE	NA	NA	NA
Bc_ctg1_2873	B4	0.66956546	Bcer	EG	Bcer	FALSE	NA	NA	NA
Bc_ctg1_2874	B4	0.781159704	Bcer	EG	Bcer	FALSE	NA	NA	NA
Bc_ctg1_2875	B4	45.30726282	Bcer	S	Bcer	FALSE	NA	NA	NA
Bc_ctg1_2876	B4	6.360871873	Bcer	U	Bcer	FALSE	NA	NA	NA
Bc_ctg1_2877	B4	868.3148078	Bcer	S	Bcer	FALSE	NA	NA	NA
Bc_ctg1_2878	B4	25.22029901	Bcer	S	Bcer	FALSE	NA	NA	NA
Bc_ctg1_2879	B4	0.781159704	Bcer	S	Bcer	FALSE	NA	NA	NA
Bc_ctg1_288	B4	2.343479111	Bcer	S	Bcer	FALSE	NA	NA	NA
Bc_ctg1_2880	B4	0	Bcer	S	Bcer	FALSE	NA	NA	NA
Bc_ctg1_2881	B4	0.557971217	Bcer	S	Bcer	FALSE	NA	NA	NA
Bc_ctg1_2882	B4	1.115942434	Bcer	P	Bcer	FALSE	NA	NA	NA
Bc_ctg1_2883	B4	1.562319407	Bcer	S	Bcer	FALSE	NA	NA	NA
Bc_ctg1_2884	B4	112.0406204	Bcer	S	Bcer	FALSE	NA	NA	NA
Bc_ctg1_2885	B4	67.84929998	Bcer	NA	Bcer	FALSE	NA	NA	NA
Bc_ctg1_2886	B4	1280.320754	Bcer	C	Bcer	FALSE	NA	NA	NA
Bc_ctg1_2887	B4	17.40870197	Bcer	G	Bcer	FALSE	NA	NA	NA
Bc_ctg1_2888	B4	46.86958222	Bcer	L	Bcer	FALSE	NA	NA	NA
Bc_ctg1_2889	B4	127.8870029	Bcer	J	Bcer	FALSE	NA	NA	NA
Bc_ctg1_2890	B4	14.3956574	Bcer	J	Bcer	FALSE	NA	NA	NA
Bc_ctg1_2891	B4	21.42609473	Bcer	NA	Bcer	FALSE	NA	NA	NA
Bc_ctg1_2892	B4	15.73478832	Bcer	KT	Bcer	FALSE	NA	NA	NA
Bc_ctg1_2893	B4	0.111594243	Bcer	I	Bcer	FALSE	NA	NA	NA
Bc_ctg1_2894	B4	5.468117926	Bcer	E	Bcer	FALSE	Bc_Ga0417192_01_rn21	Ancillary biosynthesis	SMCOG1038
Bc_ctg1_2895	B4	5.691306413	Bcer	E	Bcer	FALSE	Bc_Ga0417192_01_rn21	Ancillary biosynthesis	SMCOG1247
Bc_ctg1_2896	B4	0.223188487	Bcer	K	Bcer	FALSE	Bc_Ga0417192_01_rn21	Other	NA
Bc_ctg1_2897	B4	0	Bcer	P	Bcer	FALSE	Bc_Ga0417192_01_rn21	Other	NA
Bc_ctg1_2898	B4	0.111594243	Bcer	S	Bcer	FALSE	Bc_Ga0417192_01_rn21	Other	NA
Bc_ctg1_2899	B4	13.50290345	Bcer	E	Bcer	FALSE	Bc_Ga0417192_01_rn21	Other	NA
Bc_ctg1_29	B4	5.57971217	Bcer	S	Bcer	FALSE	NA	NA	NA
Bc_ctg1_290	B4	436.6682744	Bcer	E	Bcer	FALSE	NA	NA	NA
Bc_ctg1_2900	B4	133.1319324	Bcer	G	Bcer	FALSE	Bc_Ga0417192_01_rn21	Ancillary biosynthesis	SMCOG1145
Bc_ctg1_2901	B4	226.7595026	Bcer	V	Bcer	FALSE	Bc_Ga0417192_01_rn21	Ancillary biosynthesis	SMCOG1053
Bc_ctg1_2902	B4	0.223188487	Bcer	NA	Bcer	FALSE	Bc_Ga0417192_01_rn21	Other	NA
Bc_ctg1_2903	B4	0	Bcer	NA	Bcer	FALSE	Bc_Ga0417192_01_rn21	Other	NA
Bc_ctg1_2904	B4	0.557971217	Bcer	M	Bcer	FALSE	Bc_Ga0417192_01_rn21	Core biosynthesis	SMCOG1123
Bc_ctg1_2905	B4	0.892753947	Bcer	M	Bcer	FALSE	Bc_Ga0417192_01_rn21	Ancillary biosynthesis	SMCOG1152
Bc_ctg1_2906	B4	2.455073355	Bcer	M	Bcer	FALSE	Bc_Ga0417192_01_rn21	Core biosynthesis	SMCOG1010
Bc_ctg1_2907	B4	10.48985888	Bcer	M	Bcer	FALSE	Bc_Ga0417192_01_rn21	Core biosynthesis	SMCOG1123
Bc_ctg1_2908	B4	0	Bcer	NA	Bcer	FALSE	Bc_Ga0417192_01_rn21	Other	NA
Bc_ctg1_2909	B4	48283.70448	Bcer	C	Bcer	FALSE	Bc_Ga0417192_01_rn21	Other	NA
Bc_ctg1_291	B4	8.592756741	Bcer	S	Bcer	FALSE	NA	NA	NA
Bc_ctg1_2910	B4	14517.29512	Bcer	C	Bcer	FALSE	Bc_Ga0417192_01_rn21	Ancillary biosynthesis	None
Bc_ctg1_2911	B4	203.2131172	Bcer	M	Bcer	FALSE	Bc_Ga0417192_01_rn21	Core biosynthesis	None
Bc_ctg1_2913	B4	41.06668157	Bcer	J	Bcer	FALSE	Bc_Ga0417192_01_rn21	Other	NA
Bc_ctg1_2914	B4	20.75652927	Bcer	Q	Bcer	TRUE	Bc_Ga0417192_01_rn21	Other	NA
Bc_ctg1_2915	B4	25.66667598	Bcer	S	Bcer	FALSE	Bc_Ga0417192_01_rn21	Ancillary biosynthesis	SMCOG1010
Bc_ctg1_2916	B4	77.55799916	Bcer	S	Bcer	FALSE	Bc_Ga0417192_01_rn21	Other	NA
Bc_ctg1_2917	B4	27.45218387	Bcer	S	Bcer	FALSE	Bc_Ga0417192_01_rn21	Other	NA
Bc_ctg1_2919	B4	0	Bcer	NA	Bcer	FALSE	Bc_Ga0417192_01_rn21	Other	NA
Bc_ctg1_292	B4	8.034785524	Bcer	K	Bcer	FALSE	NA	NA	NA
Bc_ctg1_2920	B4	7.030437334	Bcer	S	Bcer	FALSE	Bc_Ga0417192_01_rn21	Other	NA
Bc_ctg1_2921	B4	80.68263797	Bcer	S	Bcer	FALSE	Bc_Ga0417192_01_rn21	Other	NA
Bc_ctg1_2922	B4	14.84203437	Bcer	L	Bcer	FALSE	Bc_Ga0417192_01_rn21	Other	NA
Bc_ctg1_2924	B4	0.33478273	Bcer	S	Bcer	FALSE	Bc_Ga0417192_01_rn21	Other	NA
Bc_ctg1_2925	B4	0	Bcer	S	Bcer	FALSE	Bc_Ga0417192_01_rn21	Other	NA
Bc_ctg1_2926	B4	86.37394438	Bcer	J	Bcer	FALSE	Bc_Ga0417192_01_rn21	Other	NA

Bc_ctg1_2927	B4	33.92464999	Bcer	V	Bcer	FALSE	NA	NA	NA
Bc_ctg1_2928	B4	91.84206231	Bcer	S	Bcer	FALSE	NA	NA	NA
Bc_ctg1_2929	B4	134.9174403	Bcer	E	Bcer	FALSE	NA	NA	NA
Bc_ctg1_293	B4	0	Bcer	NA	Bcer	FALSE	NA	NA	NA
Bc_ctg1_2930	B4	412.6755121	Bcer	H	Bcer	FALSE	NA	NA	NA
Bc_ctg1_2931	B4	10.71304737	Bcer	S	Bcer	FALSE	NA	NA	NA
Bc_ctg1_2932	B4	4.798552466	Bcer	S	Bcer	FALSE	NA	NA	NA
Bc_ctg1_2933	B4	8.592756741	Bcer	S	Bcer	FALSE	NA	NA	NA
Bc_ctg1_2934	B4	3.794204275	Bcer	P	Bcer	FALSE	NA	NA	NA
Bc_ctg1_2935	B4	25.66667598	Bcer	O	Bcer	FALSE	NA	NA	NA
Bc_ctg1_2936	B4	483.4262624	Bcer	P	Bcer	FALSE	NA	NA	NA
Bc_ctg1_2937	B4	21.42609473	Bcer	K	Bcer	FALSE	NA	NA	NA
Bc_ctg1_2938	B4	6.807248847	Bcer	S	Bcer	FALSE	NA	NA	NA
Bc_ctg1_294	B4	8.481162498	Bcer	K	Bcer	FALSE	NA	NA	NA
Bc_ctg1_2940	B4	0.446376974	Bcer	NA	Bcer	FALSE	NA	NA	NA
Bc_ctg1_2942	B4	281.6638703	Bcer	K	Bcer	FALSE	NA	NA	NA
Bc_ctg1_2943	B4	0.223188487	Bcer	F	Bcer	FALSE	NA	NA	NA
Bc_ctg1_2944	B4	51.66813469	Bcer	M	Bcer	FALSE	NA	NA	NA
Bc_ctg1_2945	B4	115.8348246	Bcer	O	Bcer	FALSE	NA	NA	NA
Bc_ctg1_2946	B4	19.08261562	Bcer	C	Bcer	FALSE	NA	NA	NA
Bc_ctg1_2947	B4	22.8768199	Bcer	S	Bcer	FALSE	Bc_Ga0417192_01_rn22	Other	NA
Bc_ctg1_2948	B4	146.9696185	Bcer	J	Bcer	FALSE	Bc_Ga0417192_01_rn22	Other	NA
Bc_ctg1_2949	B4	48.32030739	Bcer	T	Bcer	FALSE	Bc_Ga0417192_01_rn22	Ancillary biosynthesis	SMCOG1025
Bc_ctg1_295	B4	18.63623865	Bcer	K	Bcer	FALSE	NA	NA	NA
Bc_ctg1_2951	B4	11.94058404	Bcer	T	Bcer	FALSE	Bc_Ga0417192_01_rn22	Other	NA
Bc_ctg1_2952	B4	9.262322201	Bcer	S	Bcer	FALSE	Bc_Ga0417192_01_rn22	Other	NA
Bc_ctg1_2953	B4	360.0030292	Bcer	E	Bcer	FALSE	Bc_Ga0417192_01_rn22	Transport	SMCOG1202
Bc_ctg1_2954	B4	23.43479111	Bcer	NA	Bcer	FALSE	Bc_Ga0417192_01_rn22	Other	NA
Bc_ctg1_2955	B4	58.92176051	Bcer	H	Bcer	FALSE	Bc_Ga0417192_01_rn22	Ancillary biosynthesis	SMCOG1012
Bc_ctg1_2956	B4	91.17249685	Bcer	T	Bcer	FALSE	Bc_Ga0417192_01_rn22	Regulation	SMCOG1048
Bc_ctg1_2957	B4	43.9681319	Bcer	KT	Bcer	FALSE	Bc_Ga0417192_01_rn22	Regulation	SMCOG1016
Bc_ctg1_2958	B4	2032.577549	Bcer	V	Bcer	FALSE	Bc_Ga0417192_01_rn22	Transport	SMCOG1000
Bc_ctg1_2959	B4	2846.657555	Bcer	V	Bcer	FALSE	Bc_Ga0417192_01_rn22	Other	NA
Bc_ctg1_296	B4	21.98406595	Bcer	S	Bcer	FALSE	NA	NA	NA
Bc_ctg1_2960	B4	1947.207953	Bcer	V	Bcer	FALSE	Bc_Ga0417192_01_rn22	Other	NA
Bc_ctg1_2961	B4	13.16812072	Bcer	NA	Bcer	FALSE	Bc_Ga0417192_01_rn22	Other	NA
Bc_ctg1_2962	B4	401.0697107	Bcer	Q	Bcer	TRUE	Bc_Ga0417192_01_rn22	Ancillary biosynthesis	SMCOG1004
Bc_ctg1_2963	B4	2257.551544	Bcer	Q	Bcer	TRUE	Bc_Ga0417192_01_rn22	Core biosynthesis	SMCOG1002
Bc_ctg1_2964	B4	2552.383535	Bcer	Q	Bcer	TRUE	Bc_Ga0417192_01_rn22	Core biosynthesis	SMCOG1209
Bc_ctg1_2965	B4	278.76242	Bcer	I	Bcer	FALSE	Bc_Ga0417192_01_rn22	Core biosynthesis	SMCOG1021
Bc_ctg1_2966	B4	1187.36275	Bcer	Q	Bcer	TRUE	Bc_Ga0417192_01_rn22	Core biosynthesis	SMCOG1022
Bc_ctg1_2967	B4	831.265519	Bcer	Q	Bcer	TRUE	Bc_Ga0417192_01_rn22	Core biosynthesis	SMCOG1022
Bc_ctg1_2968	B4	176.98847	Bcer	Q	Bcer	TRUE	Bc_Ga0417192_01_rn22	Ancillary biosynthesis	SMCOG1222
Bc_ctg1_2969	B4	1158.348246	Bcer	Q	Bcer	TRUE	Bc_Ga0417192_01_rn22	Core biosynthesis	SMCOG1022
Bc_ctg1_297	B4	0	Bcer	K	Bcer	FALSE	NA	NA	NA
Bc_ctg1_2970	B4	276.6421294	Bcer	Q	Bcer	TRUE	Bc_Ga0417192_01_rn22	Ancillary biosynthesis	SMCOG1042
Bc_ctg1_2971	B4	3.571015789	Bcer	M	Bcer	FALSE	Bc_Ga0417192_01_rn22	Other	NA
Bc_ctg1_2972	B4	1.450725164	Bcer	K	Bcer	FALSE	Bc_Ga0417192_01_rn22	Other	NA
Bc_ctg1_2973	B4	45.5304513	Bcer	M	Bcer	FALSE	Bc_Ga0417192_01_rn22	Other	NA
Bc_ctg1_2974	B4	250.1942937	Bcer	S	Bcer	FALSE	Bc_Ga0417192_01_rn22	Other	NA
Bc_ctg1_2975	B4	29.68406874	Bcer	OU	Bcer	FALSE	Bc_Ga0417192_01_rn22	Other	NA
Bc_ctg1_2976	B4	96.64061478	Bcer	S	Bcer	FALSE	Bc_Ga0417192_01_rn22	Other	NA
Bc_ctg1_2977	B4	89.72177169	Bcer	NT	Bcer	FALSE	Bc_Ga0417192_01_rn22	Other	SMCOG1061
Bc_ctg1_2978	B4	16.962325	Bcer	T	Bcer	FALSE	Bc_Ga0417192_01_rn22	Other	NA
Bc_ctg1_2979	B4	9.820293418	Bcer	KT	Bcer	FALSE	Bc_Ga0417192_01_rn22	Regulation	SMCOG1008
Bc_ctg1_298	B4	5.468117926	Bcer	C	Bcer	FALSE	NA	NA	NA
Bc_ctg1_2980	B4	16.73913651	Bcer	C	Bcer	FALSE	Bc_Ga0417192_01_rn22	Other	NA
Bc_ctg1_2981	B4	0.223188487	Bcer	I	Bcer	FALSE	Bc_Ga0417192_01_rn22	Other	NA
Bc_ctg1_2982	B4	1.115942434	Bcer	C	Bcer	FALSE	Bc_Ga0417192_01_rn22	Other	NA
Bc_ctg1_2983	B4	69.96959061	Bcer	S	Bcer	FALSE	NA	NA	NA
Bc_ctg1_2984	B4	0.223188487	Bcer	S	Bcer	FALSE	NA	NA	NA
Bc_ctg1_2985	B4	0.111594243	Bcer	K	Bcer	FALSE	NA	NA	NA

Bc_ctg1_2986	B4	0.781159704	Bcer	P	Bcer	FALSE	NA	NA	NA
Bc_ctg1_2987	B4	0.111594243	Bcer	G	Bcer	FALSE	NA	NA	NA
Bc_ctg1_2988	B4	0.446376974	Bcer	P	Bcer	FALSE	NA	NA	NA
Bc_ctg1_2989	B4	13.27971496	Bcer	G	Bcer	FALSE	NA	NA	NA
Bc_ctg1_299	B4	0.781159704	Bcer	S	Bcer	FALSE	NA	NA	NA
Bc_ctg1_2990	B4	6.026089143	Bcer	T	Bcer	FALSE	NA	NA	NA
Bc_ctg1_2991	B4	4.240581249	Bcer	T	Bcer	FALSE	NA	NA	NA
Bc_ctg1_2992	B4	15.51159983	Bcer	T	Bcer	FALSE	NA	NA	NA
Bc_ctg1_2993	B4	0.223188487	Bcer	NA	Bcer	FALSE	NA	NA	NA
Bc_ctg1_2994	B4	2.231884868	Bcer	M	Bcer	FALSE	NA	NA	NA
Bc_ctg1_2995	B4	21.8724717	Bcer	NT	Bcer	FALSE	NA	NA	NA
Bc_ctg1_2996	B4	7.142031577	Bcer	T	Bcer	FALSE	NA	NA	NA
Bc_ctg1_2997	B4	10.71304737	Bcer	T	Bcer	FALSE	NA	NA	NA
Bc_ctg1_2998	B4	0.66956546	Bcer	C	Bcer	FALSE	NA	NA	NA
Bc_ctg1_2999	B4	2.120290624	Bcer	C	Bcer	FALSE	NA	NA	NA
Bc_ctg1_3	B4	19.30580411	Bcer	L	Bcer	FALSE	NA	NA	NA
Bc_ctg1_30	B4	62.15799357	Bcer	I	Bcer	FALSE	NA	NA	NA
Bc_ctg1_300	B4	8.815945228	Bcer	O	Bcer	FALSE	NA	NA	NA
Bc_ctg1_3000	B4	0	Bcer	NA	Bcer	FALSE	NA	NA	NA
Bc_ctg1_3001	B4	0.33478273	Bcer	EG	Bcer	FALSE	NA	NA	NA
Bc_ctg1_3002	B4	6.026089143	Bcer	K	Bcer	FALSE	NA	NA	NA
Bc_ctg1_3003	B4	24.32754506	Bcer	K	Bcer	FALSE	NA	NA	NA
Bc_ctg1_3004	B4	27.22899539	Bcer	T	Bcer	FALSE	NA	NA	NA
Bc_ctg1_3005	B4	29.01450328	Bcer	KT	Bcer	FALSE	NA	NA	NA
Bc_ctg1_3006	B4	0.111594243	Bcer	NA	Bcer	FALSE	NA	NA	NA
Bc_ctg1_3007	B4	4.798552466	Bcer	M	Bcer	FALSE	NA	NA	NA
Bc_ctg1_3008	B4	28.23334358	Bcer	C	Bcer	FALSE	NA	NA	NA
Bc_ctg1_3009	B4	1524.823742	Bcer	C	Bcer	FALSE	NA	NA	NA
Bc_ctg1_301	B4	23.43479111	Bcer	E	Bcer	FALSE	Bc_Ga0417192_01_rn03	Other	NA
Bc_ctg1_3010	B4	222.0725443	Bcer	S	Bcer	FALSE	NA	NA	NA
Bc_ctg1_3011	B4	89.27539471	Bcer	C	Bcer	FALSE	NA	NA	NA
Bc_ctg1_3012	B4	1880.251407	Bcer	E	Bcer	FALSE	NA	NA	NA
Bc_ctg1_3013	B4	805.2640603	Bcer	E	Bcer	FALSE	NA	NA	NA
Bc_ctg1_3014	B4	95.4130781	Bcer	K	Bcer	FALSE	NA	NA	NA
Bc_ctg1_3015	B4	1883.48764	Bcer	P	Bcer	FALSE	NA	NA	NA
Bc_ctg1_3016	B4	131.7928014	Bcer	H	Bcer	FALSE	NA	NA	NA
Bc_ctg1_3017	B4	392.5885482	Bcer	K	Bcer	FALSE	NA	NA	NA
Bc_ctg1_3019	B4	17.18551348	Bcer	E	Bcer	FALSE	NA	NA	NA
Bc_ctg1_302	B4	31.80435937	Bcer	S	Bcer	FALSE	Bc_Ga0417192_01_rn03	Other	NA
Bc_ctg1_3020	B4	13.83768618	Bcer	S	Bcer	FALSE	NA	NA	NA
Bc_ctg1_3021	B4	18.41305016	Bcer	NA	Bcer	FALSE	NA	NA	NA
Bc_ctg1_3022	B4	4.017392762	Bcer	NA	Bcer	FALSE	NA	NA	NA
Bc_ctg1_3023	B4	14.84203437	Bcer	V	Bcer	FALSE	NA	NA	NA
Bc_ctg1_3024	B4	27.45218387	Bcer	K	Bcer	FALSE	NA	NA	NA
Bc_ctg1_3025	B4	0.66956546	Bcer	F	Bcer	FALSE	NA	NA	NA
Bc_ctg1_3026	B4	5.468117926	Bcer	E	Bcer	FALSE	NA	NA	NA
Bc_ctg1_3027	B4	26.2246472	Bcer	S	Bcer	FALSE	NA	NA	NA
Bc_ctg1_3028	B4	303.8711248	Bcer	E	Bcer	FALSE	NA	NA	NA
Bc_ctg1_3029	B4	2467.57191	Bcer	C	Bcer	FALSE	NA	NA	NA
Bc_ctg1_303	B4	46.53479949	Bcer	S	Bcer	FALSE	Bc_Ga0417192_01_rn03	Ancillary biosynthesis	None
Bc_ctg1_3030	B4	232.3392147	Bcer	K	Bcer	FALSE	Bc_Ga0417192_01_rn23	Regulation	SMCOG1058
Bc_ctg1_3031	B4	0.33478273	Bcer	S	Bcer	FALSE	Bc_Ga0417192_01_rn23	Other	NA
Bc_ctg1_3032	B4	4540.658169	Bcer	E	Bcer	FALSE	Bc_Ga0417192_01_rn23	Transport	SMCOG1202
Bc_ctg1_3033	B4	174.5333967	Bcer	P	Bcer	FALSE	Bc_Ga0417192_01_rn23	Transport	SMCOG1033
Bc_ctg1_3034	B4	100.5464133	Bcer	P	Bcer	FALSE	Bc_Ga0417192_01_rn23	Transport	SMCOG1011
Bc_ctg1_3035	B4	72.31306972	Bcer	P	Bcer	FALSE	Bc_Ga0417192_01_rn23	Transport	SMCOG1011
Bc_ctg1_3036	B4	104.4522118	Bcer	HP	Bcer	FALSE	Bc_Ga0417192_01_rn23	Transport	SMCOG1000
Bc_ctg1_3037	B4	36.04494062	Bcer	Q	Bcer	TRUE	Bc_Ga0417192_01_rn23	Ancillary biosynthesis	SMCOG1248
Bc_ctg1_3038	B4	2290.248657	Bcer	H	Bcer	FALSE	Bc_Ga0417192_01_rn23	Ancillary biosynthesis	SMCOG1109
Bc_ctg1_3039	B4	2119.286276	Bcer	GM	Bcer	FALSE	Bc_Ga0417192_01_rn23	Core biosynthesis	SMCOG1010
Bc_ctg1_304	B4	5.021740953	Bcer	S	Bcer	FALSE	Bc_Ga0417192_01_rn03	Other	NA
Bc_ctg1_3040	B4	7.030437334	Bcer	F	Bcer	FALSE	Bc_Ga0417192_01_rn23	Other	NA

Bc_ctg1_3041	B4	0.66956546	Bcer	F	Bcer	FALSE	Bc_Ga0417192_01_rn23	Other	NA
Bc_ctg1_3042	B4	116.5043901	Bcer	P	Bcer	FALSE	Bc_Ga0417192_01_rn23	Other	NA
Bc_ctg1_3043	B4	36.49131759	Bcer	S	Bcer	FALSE	Bc_Ga0417192_01_rn23	Other	NA
Bc_ctg1_3044	B4	50.44059801	Bcer	I	Bcer	FALSE	Bc_Ga0417192_01_rn23	Other	NA
Bc_ctg1_3046	B4	39.95073913	Bcer	T	Bcer	FALSE	Bc_Ga0417192_01_rn23	Ancillary biosynthesis	SMCOG1025
Bc_ctg1_3047	B4	20.64493503	Bcer	E	Bcer	FALSE	Bc_Ga0417192_01_rn23	Ancillary biosynthesis	SMCOG1038
Bc_ctg1_3048	B4	30.24203996	Bcer	K	Bcer	FALSE	Bc_Ga0417192_01_rn23	Regulation	SMCOG1136
Bc_ctg1_3049	B4	4326.062439	Bcer	G	Bcer	FALSE	Bc_Ga0417192_01_rn23	Other	NA
Bc_ctg1_3050	B4	4363.446511	Bcer	G	Bcer	FALSE	Bc_Ga0417192_01_rn23	Core biosynthesis	SMCOG1257
Bc_ctg1_3051	B4	0.781159704	Bcer	S	Bcer	FALSE	Bc_Ga0417192_01_rn23	Other	NA
Bc_ctg1_3052	B4	0.33478273	Bcer	E	Bcer	FALSE	Bc_Ga0417192_01_rn23	Other	NA
Bc_ctg1_3053	B4	0.111594243	Bcer	EG	Bcer	FALSE	Bc_Ga0417192_01_rn23	Other	NA
Bc_ctg1_3054	B4	84.03046527	Bcer	S	Bcer	FALSE	Bc_Ga0417192_01_rn23	Ancillary biosynthesis	None
Bc_ctg1_3055	B4	7.030437334	Bcer	E	Bcer	FALSE	Bc_Ga0417192_01_rn23	Ancillary biosynthesis	SMCOG1153
Bc_ctg1_3056	B4	0	Bcer	E	Bcer	FALSE	Bc_Ga0417192_01_rn23	Transport	SMCOG1000
Bc_ctg1_3057	B4	0.892753947	Bcer	ET	Bcer	FALSE	Bc_Ga0417192_01_rn23	Ancillary biosynthesis	SMCOG1091
Bc_ctg1_3058	B4	0	Bcer	P	Bcer	FALSE	Bc_Ga0417192_01_rn23	Transport	SMCOG1096
Bc_ctg1_3059	B4	0.111594243	Bcer	P	Bcer	FALSE	Bc_Ga0417192_01_rn23	Transport	SMCOG1096
Bc_ctg1_306	B4	5.9144949	Bcer	S	Bcer	FALSE	Bc_Ga0417192_01_rn03	Other	NA
Bc_ctg1_3060	B4	199.1957245	Bcer	E	Bcer	FALSE	NA	NA	NA
Bc_ctg1_3061	B4	55.57393321	Bcer	O	Bcer	FALSE	NA	NA	NA
Bc_ctg1_3062	B4	5.57971217	Bcer	FG	Bcer	FALSE	NA	NA	NA
Bc_ctg1_3063	B4	0.223188487	Bcer	K	Bcer	FALSE	NA	NA	NA
Bc_ctg1_3064	B4	3.905798519	Bcer	NA	Bcer	FALSE	NA	NA	NA
Bc_ctg1_3065	B4	2.789856085	Bcer	K	Bcer	FALSE	NA	NA	NA
Bc_ctg1_3066	B4	1.339130921	Bcer	H	Bcer	FALSE	NA	NA	NA
Bc_ctg1_3067	B4	27.56377812	Bcer	T	Bcer	FALSE	NA	NA	NA
Bc_ctg1_3068	B4	12.94493223	Bcer	KT	Bcer	FALSE	NA	NA	NA
Bc_ctg1_3069	B4	154.5580271	Bcer	NA	Bcer	FALSE	NA	NA	NA
Bc_ctg1_3070	B4	6.026089143	Bcer	P	Bcer	FALSE	NA	NA	NA
Bc_ctg1_3071	B4	14.17246891	Bcer	T	Bcer	FALSE	NA	NA	NA
Bc_ctg1_3073	B4	145.6304876	Bcer	E	Bcer	FALSE	NA	NA	NA
Bc_ctg1_3074	B4	42.40581249	Bcer	EP	Bcer	FALSE	NA	NA	NA
Bc_ctg1_3075	B4	33.92464999	Bcer	P	Bcer	FALSE	NA	NA	NA
Bc_ctg1_3076	B4	24.4391393	Bcer	P	Bcer	FALSE	NA	NA	NA
Bc_ctg1_3077	B4	35.82175213	Bcer	P	Bcer	FALSE	NA	NA	NA
Bc_ctg1_3078	B4	29.12609753	Bcer	G	Bcer	FALSE	NA	NA	NA
Bc_ctg1_3079	B4	45.64204555	Bcer	K	Bcer	FALSE	NA	NA	NA
Bc_ctg1_308	B4	40.62030459	Bcer	IQ	Bcer	TRUE	Bc_Ga0417192_01_rn03	Ancillary biosynthesis	SMCOG1002
Bc_ctg1_3080	B4	458.0943691	Bcer	P	Bcer	FALSE	NA	NA	NA
Bc_ctg1_3081	B4	60.26089143	Bcer	K	Bcer	FALSE	NA	NA	NA
Bc_ctg1_3082	B4	61.48842811	Bcer	H	Bcer	FALSE	NA	NA	NA
Bc_ctg1_3083	B4	14.28406315	Bcer	G	Bcer	FALSE	NA	NA	NA
Bc_ctg1_3084	B4	106.2377197	Bcer	G	Bcer	FALSE	NA	NA	NA
Bc_ctg1_3085	B4	264.3667626	Bcer	G	Bcer	FALSE	NA	NA	NA
Bc_ctg1_3086	B4	701.9277909	Bcer	G	Bcer	FALSE	NA	NA	NA
Bc_ctg1_3087	B4	759.5104205	Bcer	G	Bcer	FALSE	NA	NA	NA
Bc_ctg1_3088	B4	924.6699007	Bcer	V	Bcer	FALSE	NA	NA	NA
Bc_ctg1_3089	B4	0	Bcer	NA	Bcer	FALSE	NA	NA	NA
Bc_ctg1_309	B4	10.15507615	Bcer	S	Bcer	FALSE	Bc_Ga0417192_01_rn03	Other	NA
Bc_ctg1_3090	B4	0	Bcer	NA	Bcer	FALSE	NA	NA	NA
Bc_ctg1_3091	B4	66.17538633	Bcer	EGP	Bcer	FALSE	NA	NA	NA
Bc_ctg1_3092	B4	3550.482448	Bcer	E	Bcer	FALSE	NA	NA	NA
Bc_ctg1_3093	B4	4.798552466	Bcer	P	Bcer	FALSE	NA	NA	NA
Bc_ctg1_3094	B4	821.1104429	Bcer	M	Bcer	FALSE	NA	NA	NA
Bc_ctg1_3095	B4	174.9797736	Bcer	S	Bcer	FALSE	NA	NA	NA
Bc_ctg1_3096	B4	2.455073355	Bcer	E	Bcer	FALSE	NA	NA	NA
Bc_ctg1_3097	B4	6.026089143	Bcer	C	Bcer	FALSE	NA	NA	NA
Bc_ctg1_3098	B4	5.356523683	Bcer	V	Bcer	FALSE	NA	NA	NA
Bc_ctg1_3099	B4	4.017392762	Bcer	NA	Bcer	FALSE	NA	NA	NA
Bc_ctg1_31	B4	23.21160263	Bcer	S	Bcer	FALSE	NA	NA	NA
Bc_ctg1_310	B4	2.566667598	Bcer	K	Bcer	FALSE	Bc_Ga0417192_01_rn03	Regulation	SMCOG1167

Bc_ctg1_3100	B4	0.111594243	Bcer	NA	Bcer	FALSE	NA	NA	NA
Bc_ctg1_3101	B4	5.57971217	Bcer	V	Bcer	FALSE	NA	NA	NA
Bc_ctg1_3102	B4	91.39568534	Bcer	NT	Bcer	FALSE	NA	NA	NA
Bc_ctg1_3103	B4	358.2175213	Bcer	S	Bcer	FALSE	NA	NA	NA
Bc_ctg1_3104	B4	0.111594243	Bcer	K	Bcer	FALSE	NA	NA	NA
Bc_ctg1_3105	B4	2.343479111	Bcer	NA	Bcer	FALSE	NA	NA	NA
Bc_ctg1_3106	B4	8.927539471	Bcer	M	Bcer	FALSE	NA	NA	NA
Bc_ctg1_3107	B4	2.231884868	Bcer	L	Bcer	FALSE	NA	NA	NA
Bc_ctg1_3108	B4	251.1986419	Bcer	E	Bcer	FALSE	NA	NA	NA
Bc_ctg1_3109	B4	2.120290624	Bcer	E	Bcer	FALSE	NA	NA	NA
Bc_ctg1_311	B4	0.111594243	Bcer	NA	Bcer	FALSE	Bc_Ga0417192_01_rn03	Other	NA
Bc_ctg1_3110	B4	83.5840883	Bcer	S	Bcer	FALSE	NA	NA	NA
Bc_ctg1_3111	B4	152.5493307	Bcer	G	Bcer	FALSE	NA	NA	NA
Bc_ctg1_3112	B4	38.72320246	Bcer	E	Bcer	FALSE	NA	NA	NA
Bc_ctg1_3113	B4	41.73624703	Bcer	S	Bcer	FALSE	NA	NA	NA
Bc_ctg1_3114	B4	0.33478273	Bcer	K	Bcer	FALSE	NA	NA	NA
Bc_ctg1_3115	B4	3.459421545	Bcer	NA	Bcer	FALSE	NA	NA	NA
Bc_ctg1_3116	B4	13.83768618	Bcer	K	Bcer	FALSE	NA	NA	NA
Bc_ctg1_3117	B4	3.124638815	Bcer	S	Bcer	FALSE	NA	NA	NA
Bc_ctg1_3118	B4	20.53334078	Bcer	P	Bcer	FALSE	NA	NA	NA
Bc_ctg1_3119	B4	73.20582366	Bcer	C	Bcer	FALSE	NA	NA	NA
Bc_ctg1_312	B4	0.33478273	Bcer	K	Bcer	FALSE	Bc_Ga0417192_01_rn03	Regulation	SMCOG1058
Bc_ctg1_3120	B4	633.5205197	Bcer	C	Bcer	FALSE	NA	NA	NA
Bc_ctg1_3121	B4	2718.770552	Bcer	C	Bcer	FALSE	NA	NA	NA
Bc_ctg1_3122	B4	1161.919262	Bcer	C	Bcer	FALSE	NA	NA	NA
Bc_ctg1_3123	B4	73.87538912	Bcer	E	Bcer	FALSE	NA	NA	NA
Bc_ctg1_3124	B4	17.85507894	Bcer	F	Bcer	FALSE	NA	NA	NA
Bc_ctg1_3125	B4	1.785507894	Bcer	E	Bcer	FALSE	NA	NA	NA
Bc_ctg1_3126	B4	0	Bcer	S	Bcer	FALSE	NA	NA	NA
Bc_ctg1_3127	B4	12.27536677	Bcer	E	Bcer	FALSE	NA	NA	NA
Bc_ctg1_3128	B4	0.446376974	Bcer	EG	Bcer	FALSE	NA	NA	NA
Bc_ctg1_3129	B4	0.223188487	Bcer	E	Bcer	FALSE	NA	NA	NA
Bc_ctg1_3130	B4	1.004348191	Bcer	S	Bcer	FALSE	NA	NA	NA
Bc_ctg1_3131	B4	2988.27065	Bcer	P	Bcer	FALSE	NA	NA	NA
Bc_ctg1_3132	B4	1594.235361	Bcer	P	Bcer	FALSE	NA	NA	NA
Bc_ctg1_3133	B4	97.86815145	Bcer	P	Bcer	FALSE	NA	NA	NA
Bc_ctg1_3134	B4	2.120290624	Bcer	P	Bcer	FALSE	NA	NA	NA
Bc_ctg1_3135	B4	2.678261841	Bcer	P	Bcer	FALSE	NA	NA	NA
Bc_ctg1_3136	B4	5.802900656	Bcer	P	Bcer	FALSE	NA	NA	NA
Bc_ctg1_3137	B4	0.781159704	Bcer	S	Bcer	FALSE	NA	NA	NA
Bc_ctg1_3138	B4	19.30580411	Bcer	V	Bcer	FALSE	NA	NA	NA
Bc_ctg1_314	B4	0.111594243	Bcer	L	Bcer	FALSE	Bc_Ga0417192_01_rn03	Other	SMCOG1026
Bc_ctg1_3140	B4	10.48985888	Bcer	T	Bcer	FALSE	NA	NA	NA
Bc_ctg1_3141	B4	88.49423501	Bcer	S	Bcer	FALSE	NA	NA	NA
Bc_ctg1_3142	B4	67.06814028	Bcer	NA	Bcer	FALSE	NA	NA	NA
Bc_ctg1_3143	B4	400.4001453	Bcer	MT	Bcer	FALSE	NA	NA	NA
Bc_ctg1_3144	B4	2.678261841	Bcer	K	Bcer	FALSE	NA	NA	NA
Bc_ctg1_3145	B4	2.455073355	Bcer	P	Bcer	FALSE	NA	NA	NA
Bc_ctg1_3146	B4	0.892753947	Bcer	P	Bcer	FALSE	NA	NA	NA
Bc_ctg1_3147	B4	1.450725164	Bcer	P	Bcer	FALSE	NA	NA	NA
Bc_ctg1_3148	B4	1.115942434	Bcer	H	Bcer	FALSE	NA	NA	NA
Bc_ctg1_3149	B4	3.124638815	Bcer	E	Bcer	FALSE	NA	NA	NA
Bc_ctg1_315	B4	2.008696381	Bcer	M	Bcer	FALSE	Bc_Ga0417192_01_rn03	Core biosynthesis	SMCOG1123
Bc_ctg1_3150	B4	0.223188487	Bcer	H	Bcer	FALSE	NA	NA	NA
Bc_ctg1_3151	B4	3.459421545	Bcer	H	Bcer	FALSE	NA	NA	NA
Bc_ctg1_3152	B4	3.905798519	Bcer	H	Bcer	FALSE	NA	NA	NA
Bc_ctg1_3153	B4	4.798552466	Bcer	H	Bcer	FALSE	NA	NA	NA
Bc_ctg1_3154	B4	6.24927763	Bcer	NA	Bcer	FALSE	NA	NA	NA
Bc_ctg1_3155	B4	6.472466117	Bcer	NA	Bcer	FALSE	NA	NA	NA
Bc_ctg1_3157	B4	334.8943244	Bcer	P	Bcer	FALSE	NA	NA	NA
Bc_ctg1_3158	B4	386.8972418	Bcer	P	Bcer	FALSE	NA	NA	NA
Bc_ctg1_3159	B4	71.42031577	Bcer	P	Bcer	FALSE	NA	NA	NA

Bc_ctg1_316	B4	0.781159704	Bcer	S	Bcer	FALSE	Bc_Ga0417192_01_rn03	Other	NA
Bc_ctg1_3160	B4	8.704350984	Bcer	T	Bcer	FALSE	NA	NA	NA
Bc_ctg1_3161	B4	0.111594243	Bcer	EGP	Bcer	FALSE	NA	NA	NA
Bc_ctg1_3162	B4	4.686958222	Bcer	K	Bcer	FALSE	NA	NA	NA
Bc_ctg1_3163	B4	43.18697219	Bcer	S	Bcer	FALSE	NA	NA	NA
Bc_ctg1_3164	B4	2.008696381	Bcer	NA	Bcer	FALSE	NA	NA	NA
Bc_ctg1_3165	B4	0.33478273	Bcer	V	Bcer	FALSE	NA	NA	NA
Bc_ctg1_3166	B4	1.339130921	Bcer	V	Bcer	FALSE	NA	NA	NA
Bc_ctg1_3167	B4	2.008696381	Bcer	V	Bcer	FALSE	NA	NA	NA
Bc_ctg1_3168	B4	0.557971217	Bcer	NA	Bcer	FALSE	NA	NA	NA
Bc_ctg1_3169	B4	10.60145312	Bcer	S	Bcer	FALSE	NA	NA	NA
Bc_ctg1_317	B4	0	Bcer	K	Bcer	FALSE	Bc_Ga0417192_01_rn03	Other	NA
Bc_ctg1_3170	B4	0.223188487	Bcer	IQ	Bcer	TRUE	NA	NA	NA
Bc_ctg1_3171	B4	1.673913651	Bcer	S	Bcer	FALSE	NA	NA	NA
Bc_ctg1_3172	B4	0.111594243	Bcer	NA	Bcer	FALSE	NA	NA	NA
Bc_ctg1_3173	B4	9.485510688	Bcer	S	Bcer	FALSE	NA	NA	NA
Bc_ctg1_3174	B4	30.80001118	Bcer	CO	Bcer	FALSE	NA	NA	NA
Bc_ctg1_3175	B4	32.9203018	Bcer	C	Bcer	FALSE	NA	NA	NA
Bc_ctg1_3176	B4	1.450725164	Bcer	S	Bcer	FALSE	NA	NA	NA
Bc_ctg1_3177	B4	40.84349308	Bcer	S	Bcer	FALSE	NA	NA	NA
Bc_ctg1_3178	B4	1.339130921	Bcer	E	Bcer	FALSE	NA	NA	NA
Bc_ctg1_3179	B4	0.66956546	Bcer	S	Bcer	FALSE	NA	NA	NA
Bc_ctg1_318	B4	19.08261562	Bcer	M	Bcer	FALSE	Bc_Ga0417192_01_rn03	Core biosynthesis	None
Bc_ctg1_3180	B4	0.446376974	Bcer	P	Bcer	FALSE	NA	NA	NA
Bc_ctg1_3181	B4	0.446376974	Bcer	NA	Bcer	FALSE	NA	NA	NA
Bc_ctg1_3182	B4	12.72174375	Bcer	C	Bcer	FALSE	NA	NA	NA
Bc_ctg1_3183	B4	0.892753947	Bcer	S	Bcer	FALSE	NA	NA	NA
Bc_ctg1_3184	B4	10.48985888	Bcer	S	Bcer	FALSE	NA	NA	NA
Bc_ctg1_3185	B4	4.910146709	Bcer	M	Bcer	FALSE	NA	NA	NA
Bc_ctg1_3186	B4	0.557971217	Bcer	NA	Bcer	FALSE	NA	NA	NA
Bc_ctg1_3187	B4	4.128987005	Bcer	P	Bcer	FALSE	NA	NA	NA
Bc_ctg1_3188	B4	18.5246444	Bcer	S	Bcer	FALSE	NA	NA	NA
Bc_ctg1_3189	B4	0.111594243	Bcer	OP	Bcer	FALSE	NA	NA	NA
Bc_ctg1_319	B4	8.592756741	Bcer	E	Bcer	FALSE	Bc_Ga0417192_01_rn03	Transport	SMCOG1116
Bc_ctg1_3190	B4	1.562319407	Bcer	P	Bcer	FALSE	NA	NA	NA
Bc_ctg1_3191	B4	0	Bcer	O	Bcer	FALSE	NA	NA	NA
Bc_ctg1_3192	B4	0	Bcer	P	Bcer	FALSE	NA	NA	NA
Bc_ctg1_3193	B4	0	Bcer	P	Bcer	FALSE	NA	NA	NA
Bc_ctg1_3194	B4	0	Bcer	O	Bcer	FALSE	NA	NA	NA
Bc_ctg1_3195	B4	14.84203437	Bcer	S	Bcer	FALSE	NA	NA	NA
Bc_ctg1_3196	B4	121.0797541	Bcer	NA	Bcer	FALSE	NA	NA	NA
Bc_ctg1_3197	B4	508.0885902	Bcer	K	Bcer	FALSE	NA	NA	NA
Bc_ctg1_3198	B4	75.43770853	Bcer	C	Bcer	FALSE	NA	NA	NA
Bc_ctg1_32	B4	23.6579796	Bcer	J	Bcer	FALSE	NA	NA	NA
Bc_ctg1_320	B4	45.5304513	Bcer	S	Bcer	FALSE	Bc_Ga0417192_01_rn03	Other	NA
Bc_ctg1_3201	B4	5.691306413	Bcer	P	Bcer	FALSE	NA	NA	NA
Bc_ctg1_3202	B4	26.55942993	Bcer	EGP	Bcer	FALSE	NA	NA	NA
Bc_ctg1_3203	B4	116.9507671	Bcer	S	Bcer	FALSE	NA	NA	NA
Bc_ctg1_3204	B4	25.10870476	Bcer	K	Bcer	FALSE	NA	NA	NA
Bc_ctg1_3205	B4	5.133335196	Bcer	G	Bcer	FALSE	NA	NA	NA
Bc_ctg1_3206	B4	9.039133715	Bcer	G	Bcer	FALSE	NA	NA	NA
Bc_ctg1_3207	B4	33.47827302	Bcer	G	Bcer	FALSE	NA	NA	NA
Bc_ctg1_3208	B4	4.463769736	Bcer	NA	Bcer	FALSE	NA	NA	NA
Bc_ctg1_3209	B4	19.75218108	Bcer	S	Bcer	FALSE	NA	NA	NA
Bc_ctg1_321	B4	0.223188487	Bcer	NA	Bcer	FALSE	Bc_Ga0417192_01_rn03	Other	NA
Bc_ctg1_3210	B4	2194.054419	Bcer	M	Bcer	FALSE	NA	NA	NA
Bc_ctg1_3211	B4	114.4956937	Bcer	V	Bcer	FALSE	NA	NA	NA
Bc_ctg1_3212	B4	66.73335755	Bcer	V	Bcer	FALSE	NA	NA	NA
Bc_ctg1_3213	B4	65.8406036	Bcer	M	Bcer	FALSE	NA	NA	NA
Bc_ctg1_3214	B4	6.807248847	Bcer	S	Bcer	FALSE	NA	NA	NA
Bc_ctg1_3215	B4	0.781159704	Bcer	EP	Bcer	FALSE	NA	NA	NA
Bc_ctg1_3216	B4	2.343479111	Bcer	EP	Bcer	FALSE	NA	NA	NA

Bc_ctg1_3217	B4	34.59421545	Bcer	E	Bcer	FALSE	NA	NA	NA
Bc_ctg1_3218	B4	0	Bcer	P	Bcer	FALSE	NA	NA	NA
Bc_ctg1_322	B4	0.111594243	Bcer	NA	Bcer	FALSE	Bc_Ga0417192_01_rn03	Other	NA
Bc_ctg1_3220	B4	0	Bcer	S	Bcer	FALSE	NA	NA	NA
Bc_ctg1_3221	B4	0.111594243	Bcer	E	Bcer	FALSE	NA	NA	NA
Bc_ctg1_3222	B4	0.33478273	Bcer	S	Bcer	FALSE	NA	NA	NA
Bc_ctg1_3223	B4	0.892753947	Bcer	L	Bcer	FALSE	NA	NA	NA
Bc_ctg1_3224	B4	0.33478273	Bcer	S	Bcer	FALSE	NA	NA	NA
Bc_ctg1_3225	B4	1.339130921	Bcer	S	Bcer	FALSE	NA	NA	NA
Bc_ctg1_3226	B4	2.678261841	Bcer	S	Bcer	FALSE	NA	NA	NA
Bc_ctg1_3227	B4	1.673913651	Bcer	S	Bcer	FALSE	NA	NA	NA
Bc_ctg1_3228	B4	0	Bcer	NA	Bcer	FALSE	NA	NA	NA
Bc_ctg1_3229	B4	3.347827302	Bcer	S	Bcer	FALSE	NA	NA	NA
Bc_ctg1_323	B4	7.365220064	Bcer	K	Bcer	FALSE	Bc_Ga0417192_01_rn03	Other	NA
Bc_ctg1_3230	B4	6.026089143	Bcer	NA	Bcer	FALSE	NA	NA	NA
Bc_ctg1_3231	B4	11.0478301	Bcer	NA	Bcer	FALSE	NA	NA	NA
Bc_ctg1_3232	B4	2.455073355	Bcer	E	Bcer	FALSE	NA	NA	NA
Bc_ctg1_3233	B4	0.111594243	Bcer	P	Bcer	FALSE	NA	NA	NA
Bc_ctg1_3234	B4	6.695654603	Bcer	NA	Bcer	FALSE	NA	NA	NA
Bc_ctg1_3235	B4	46.75798798	Bcer	K	Bcer	FALSE	NA	NA	NA
Bc_ctg1_3236	B4	41.4014643	Bcer	G	Bcer	FALSE	NA	NA	NA
Bc_ctg1_3237	B4	323.0653346	Bcer	G	Bcer	FALSE	NA	NA	NA
Bc_ctg1_3238	B4	232.7855917	Bcer	S	Bcer	FALSE	NA	NA	NA
Bc_ctg1_3239	B4	0	Bcer	S	Bcer	FALSE	NA	NA	NA
Bc_ctg1_324	B4	0.33478273	Bcer	K	Bcer	FALSE	Bc_Ga0417192_01_rn03	Other	NA
Bc_ctg1_3241	B4	0	Bcer	NA	Bcer	FALSE	NA	NA	NA
Bc_ctg1_3242	B4	12.49855526	Bcer	EG	Bcer	FALSE	NA	NA	NA
Bc_ctg1_3243	B4	1.115942434	Bcer	K	Bcer	FALSE	NA	NA	NA
Bc_ctg1_3244	B4	0.33478273	Bcer	E	Bcer	FALSE	NA	NA	NA
Bc_ctg1_3245	B4	1.785507894	Bcer	S	Bcer	FALSE	NA	NA	NA
Bc_ctg1_3246	B4	0	Bcer	K	Bcer	FALSE	NA	NA	NA
Bc_ctg1_3247	B4	4.575363979	Bcer	P	Bcer	FALSE	NA	NA	NA
Bc_ctg1_3248	B4	2.789856085	Bcer	P	Bcer	FALSE	NA	NA	NA
Bc_ctg1_3249	B4	36.2681291	Bcer	K	Bcer	FALSE	NA	NA	NA
Bc_ctg1_325	B4	4.128987005	Bcer	V	Bcer	FALSE	Bc_Ga0417192_01_rn03	Transport	SMCOG1065
Bc_ctg1_3250	B4	48.87827861	Bcer	EGP	Bcer	FALSE	NA	NA	NA
Bc_ctg1_3251	B4	0.892753947	Bcer	NA	Bcer	FALSE	NA	NA	NA
Bc_ctg1_3252	B4	11.8289898	Bcer	T	Bcer	FALSE	NA	NA	NA
Bc_ctg1_3253	B4	19.30580411	Bcer	T	Bcer	FALSE	NA	NA	NA
Bc_ctg1_3254	B4	0.557971217	Bcer	M	Bcer	FALSE	NA	NA	NA
Bc_ctg1_3255	B4	0.66956546	Bcer	V	Bcer	FALSE	NA	NA	NA
Bc_ctg1_3256	B4	1.339130921	Bcer	V	Bcer	FALSE	NA	NA	NA
Bc_ctg1_3257	B4	0.223188487	Bcer	V	Bcer	FALSE	NA	NA	NA
Bc_ctg1_3258	B4	0.557971217	Bcer	V	Bcer	FALSE	NA	NA	NA
Bc_ctg1_3259	B4	3.459421545	Bcer	S	Bcer	FALSE	NA	NA	NA
Bc_ctg1_326	B4	4.352175492	Bcer	V	Bcer	FALSE	Bc_Ga0417192_01_rn03	Transport	SMCOG1000
Bc_ctg1_3260	B4	1.897102138	Bcer	K	Bcer	FALSE	NA	NA	NA
Bc_ctg1_3261	B4	0.33478273	Bcer	S	Bcer	FALSE	NA	NA	NA
Bc_ctg1_3262	B4	1.004348191	Bcer	S	Bcer	FALSE	NA	NA	NA
Bc_ctg1_3263	B4	1.339130921	Bcer	S	Bcer	FALSE	NA	NA	NA
Bc_ctg1_3264	B4	10.26667039	Bcer	S	Bcer	FALSE	NA	NA	NA
Bc_ctg1_3265	B4	51.66813469	Bcer	E	Bcer	FALSE	NA	NA	NA
Bc_ctg1_3266	B4	0	Bcer	NA	Bcer	FALSE	NA	NA	NA
Bc_ctg1_3267	B4	9.931887662	Bcer	S	Bcer	FALSE	NA	NA	NA
Bc_ctg1_3268	B4	51.55654045	Bcer	P	Bcer	FALSE	NA	NA	NA
Bc_ctg1_3269	B4	51.33335196	Bcer	U	Bcer	FALSE	NA	NA	NA
Bc_ctg1_327	B4	9.039133715	Bcer	S	Bcer	FALSE	Bc_Ga0417192_01_rn03	Other	NA
Bc_ctg1_3270	B4	30.68841693	Bcer	E	Bcer	FALSE	NA	NA	NA
Bc_ctg1_3271	B4	8.146379768	Bcer	S	Bcer	FALSE	NA	NA	NA
Bc_ctg1_3272	B4	419.9291379	Bcer	C	Bcer	FALSE	NA	NA	NA
Bc_ctg1_3273	B4	19.86377532	Bcer	V	Bcer	FALSE	NA	NA	NA
Bc_ctg1_3274	B4	24.4391393	Bcer	V	Bcer	FALSE	NA	NA	NA

Bc_ctg1_3275	B4	172.859483	Bcer	ET	Bcer	FALSE	NA	NA	NA
Bc_ctg1_3276	B4	106.7956909	Bcer	P	Bcer	FALSE	NA	NA	NA
Bc_ctg1_3277	B4	181.3406455	Bcer	E	Bcer	FALSE	NA	NA	NA
Bc_ctg1_3278	B4	0.33478273	Bcer	S	Bcer	FALSE	NA	NA	NA
Bc_ctg1_3279	B4	0	Bcer	NA	Bcer	FALSE	NA	NA	NA
Bc_ctg1_328	B4	22.31884868	Bcer	K	Bcer	FALSE	Bc_Ga0417192_01_rn03	Other	NA
Bc_ctg1_3280	B4	0.111594243	Bcer	HJ	Bcer	FALSE	NA	NA	NA
Bc_ctg1_3281	B4	0.33478273	Bcer	HJ	Bcer	FALSE	NA	NA	NA
Bc_ctg1_3282	B4	9.485510688	Bcer	S	Bcer	FALSE	NA	NA	NA
Bc_ctg1_3283	B4	130.0072936	Bcer	S	Bcer	FALSE	NA	NA	NA
Bc_ctg1_3284	B4	6.58406036	Bcer	NA	Bcer	FALSE	NA	NA	NA
Bc_ctg1_3285	B4	4.910146709	Bcer	S	Bcer	FALSE	NA	NA	NA
Bc_ctg1_3286	B4	145.4072991	Bcer	L	Bcer	FALSE	NA	NA	NA
Bc_ctg1_3287	B4	403.3015956	Bcer	EH	Bcer	FALSE	NA	NA	NA
Bc_ctg1_3288	B4	148.4203437	Bcer	Q	Bcer	TRUE	NA	NA	NA
Bc_ctg1_3289	B4	3.571015789	Bcer	L	Bcer	FALSE	NA	NA	NA
Bc_ctg1_329	B4	482.3103199	Bcer	C	Bcer	FALSE	Bc_Ga0417192_01_rn03	Other	NA
Bc_ctg1_3290	B4	1.562319407	Bcer	S	Bcer	FALSE	NA	NA	NA
Bc_ctg1_3291	B4	60.03770294	Bcer	M	Bcer	FALSE	NA	NA	NA
Bc_ctg1_3292	B4	230.5537068	Bcer	M	Bcer	FALSE	NA	NA	NA
Bc_ctg1_3293	B4	203.5478999	Bcer	H	Bcer	FALSE	NA	NA	NA
Bc_ctg1_3294	B4	8.927539471	Bcer	IQ	Bcer	TRUE	NA	NA	NA
Bc_ctg1_3295	B4	0	Bcer	NA	Bcer	FALSE	NA	NA	NA
Bc_ctg1_3296	B4	52.67248288	Bcer	NA	Bcer	FALSE	NA	NA	NA
Bc_ctg1_3297	B4	83.69568254	Bcer	P	Bcer	FALSE	NA	NA	NA
Bc_ctg1_3298	B4	118.289898	Bcer	S	Bcer	FALSE	NA	NA	NA
Bc_ctg1_3299	B4	49.65943831	Bcer	S	Bcer	FALSE	NA	NA	NA
Bc_ctg1_3300	B4	141.8362833	Bcer	G	Bcer	FALSE	NA	NA	NA
Bc_ctg1_3301	B4	69.1884309	Bcer	M	Bcer	FALSE	NA	NA	NA
Bc_ctg1_3302	B4	54.90436775	Bcer	I	Bcer	FALSE	NA	NA	NA
Bc_ctg1_3303	B4	2.566667598	Bcer	NA	Bcer	FALSE	NA	NA	NA
Bc_ctg1_3304	B4	1.897102138	Bcer	I	Bcer	FALSE	NA	NA	NA
Bc_ctg1_3305	B4	0.223188487	Bcer	NA	Bcer	FALSE	NA	NA	NA
Bc_ctg1_3306	B4	83.13771133	Bcer	G	Bcer	FALSE	NA	NA	NA
Bc_ctg1_3307	B4	85.59278468	Bcer	M	Bcer	FALSE	NA	NA	NA
Bc_ctg1_3308	B4	34.48262121	Bcer	L	Bcer	FALSE	NA	NA	NA
Bc_ctg1_331	B4	1.339130921	Bcer	S	Bcer	FALSE	NA	NA	NA
Bc_ctg1_3310	B4	0.446376974	Bcer	E	Bcer	FALSE	NA	NA	NA
Bc_ctg1_3311	B4	4.575363979	Bcer	E	Bcer	FALSE	NA	NA	NA
Bc_ctg1_3312	B4	9.373916445	Bcer	NA	Bcer	FALSE	NA	NA	NA
Bc_ctg1_3313	B4	82.35655162	Bcer	E	Bcer	FALSE	NA	NA	NA
Bc_ctg1_3314	B4	12.6101495	Bcer	P	Bcer	FALSE	NA	NA	NA
Bc_ctg1_3315	B4	18.5246444	Bcer	EP	Bcer	FALSE	NA	NA	NA
Bc_ctg1_3316	B4	36.60291183	Bcer	P	Bcer	FALSE	NA	NA	NA
Bc_ctg1_3317	B4	47.76233617	Bcer	E	Bcer	FALSE	NA	NA	NA
Bc_ctg1_3318	B4	0.223188487	Bcer	O	Bcer	FALSE	NA	NA	NA
Bc_ctg1_3319	B4	0.66956546	Bcer	S	Bcer	FALSE	NA	NA	NA
Bc_ctg1_332	B4	2.120290624	Bcer	M	Bcer	FALSE	NA	NA	NA
Bc_ctg1_3320	B4	0.33478273	Bcer	S	Bcer	FALSE	NA	NA	NA
Bc_ctg1_3321	B4	0	Bcer	S	Bcer	FALSE	NA	NA	NA
Bc_ctg1_3322	B4	0.892753947	Bcer	NA	Bcer	FALSE	NA	NA	NA
Bc_ctg1_3324	B4	0	Bcer	NA	Bcer	FALSE	NA	NA	NA
Bc_ctg1_3325	B4	1.004348191	Bcer	V	Bcer	FALSE	NA	NA	NA
Bc_ctg1_3326	B4	0.781159704	Bcer	EGP	Bcer	FALSE	NA	NA	NA
Bc_ctg1_3327	B4	4.686958222	Bcer	T	Bcer	FALSE	NA	NA	NA
Bc_ctg1_3328	B4	2.343479111	Bcer	T	Bcer	FALSE	NA	NA	NA
Bc_ctg1_3329	B4	2.008696381	Bcer	S	Bcer	FALSE	NA	NA	NA
Bc_ctg1_333	B4	0	Bcer	S	Bcer	FALSE	NA	NA	NA
Bc_ctg1_3330	B4	0	Bcer	NA	Bcer	FALSE	NA	NA	NA
Bc_ctg1_3331	B4	11.0478301	Bcer	L	Bcer	FALSE	NA	NA	NA
Bc_ctg1_3332	B4	8.704350984	Bcer	L	Bcer	FALSE	NA	NA	NA
Bc_ctg1_3333	B4	19.30580411	Bcer	S	Bcer	FALSE	NA	NA	NA

Bc_ctg1_3334	B4	23.43479111	Bcer	K	Bcer	FALSE	NA	NA	NA
Bc_ctg1_3335	B4	2.343479111	Bcer	S	Bcer	FALSE	NA	NA	NA
Bc_ctg1_3336	B4	1.227536677	Bcer	EG	Bcer	FALSE	NA	NA	NA
Bc_ctg1_3337	B4	17.63189046	Bcer	KT	Bcer	FALSE	NA	NA	NA
Bc_ctg1_3338	B4	0.446376974	Bcer	S	Bcer	FALSE	NA	NA	NA
Bc_ctg1_3339	B4	0.892753947	Bcer	S	Bcer	FALSE	NA	NA	NA
Bc_ctg1_334	B4	13.83768618	Bcer	S	Bcer	FALSE	NA	NA	NA
Bc_ctg1_3340	B4	0.111594243	Bcer	NA	Bcer	FALSE	NA	NA	NA
Bc_ctg1_3341	B4	6.695654603	Bcer	K	Bcer	FALSE	NA	NA	NA
Bc_ctg1_3342	B4	41.06668157	Bcer	EGP	Bcer	FALSE	NA	NA	NA
Bc_ctg1_3343	B4	5.9144949	Bcer	NA	Bcer	FALSE	NA	NA	NA
Bc_ctg1_3345	B4	14.3956574	Bcer	NA	Bcer	FALSE	NA	NA	NA
Bc_ctg1_3346	B4	3.459421545	Bcer	M	Bcer	FALSE	NA	NA	NA
Bc_ctg1_3347	B4	2.008696381	Bcer	NA	Bcer	FALSE	NA	NA	NA
Bc_ctg1_3348	B4	1.785507894	Bcer	G	Bcer	FALSE	NA	NA	NA
Bc_ctg1_335	B4	0.223188487	Bcer	NA	Bcer	FALSE	NA	NA	NA
Bc_ctg1_3350	B4	0.781159704	Bcer	NA	Bcer	FALSE	NA	NA	NA
Bc_ctg1_3351	B4	3.571015789	Bcer	S	Bcer	FALSE	NA	NA	NA
Bc_ctg1_3353	B4	13.72609194	Bcer	S	Bcer	FALSE	NA	NA	NA
Bc_ctg1_3354	B4	1.673913651	Bcer	T	Bcer	FALSE	NA	NA	NA
Bc_ctg1_3356	B4	3.347827302	Bcer	K	Bcer	FALSE	NA	NA	NA
Bc_ctg1_3357	B4	5.133335196	Bcer	I	Bcer	FALSE	NA	NA	NA
Bc_ctg1_3358	B4	1.785507894	Bcer	S	Bcer	FALSE	NA	NA	NA
Bc_ctg1_3359	B4	0.223188487	Bcer	EGP	Bcer	FALSE	NA	NA	NA
Bc_ctg1_336	B4	0	Bcer	NA	Bcer	FALSE	NA	NA	NA
Bc_ctg1_3360	B4	16.62754227	Bcer	K	Bcer	FALSE	NA	NA	NA
Bc_ctg1_3362	B4	0	Bcer	V	Bcer	FALSE	NA	NA	NA
Bc_ctg1_3363	B4	21.53768897	Bcer	G	Bcer	FALSE	NA	NA	NA
Bc_ctg1_3364	B4	5.244929439	Bcer	K	Bcer	FALSE	NA	NA	NA
Bc_ctg1_3365	B4	315.2537376	Bcer	G	Bcer	FALSE	NA	NA	NA
Bc_ctg1_3366	B4	8.257974011	Bcer	I	Bcer	FALSE	NA	NA	NA
Bc_ctg1_3367	B4	13.27971496	Bcer	K	Bcer	FALSE	NA	NA	NA
Bc_ctg1_3368	B4	0.892753947	Bcer	NA	Bcer	FALSE	NA	NA	NA
Bc_ctg1_337	B4	9.597104932	Bcer	V	Bcer	FALSE	NA	NA	NA
Bc_ctg1_3371	B4	2.678261841	Bcer	NA	Bcer	FALSE	NA	NA	NA
Bc_ctg1_3372	B4	0.111594243	Bcer	NA	Bcer	FALSE	NA	NA	NA
Bc_ctg1_3373	B4	9.262322201	Bcer	K	Bcer	FALSE	NA	NA	NA
Bc_ctg1_3374	B4	0.781159704	Bcer	NA	Bcer	FALSE	NA	NA	NA
Bc_ctg1_3375	B4	0.223188487	Bcer	NA	Bcer	FALSE	NA	NA	NA
Bc_ctg1_3376	B4	11.8289898	Bcer	S	Bcer	FALSE	NA	NA	NA
Bc_ctg1_3377	B4	0.446376974	Bcer	NA	Bcer	FALSE	NA	NA	NA
Bc_ctg1_3378	B4	13.94928042	Bcer	D	Bcer	FALSE	NA	NA	NA
Bc_ctg1_3379	B4	0.33478273	Bcer	NA	Bcer	FALSE	NA	NA	NA
Bc_ctg1_338	B4	3.571015789	Bcer	NA	Bcer	FALSE	NA	NA	NA
Bc_ctg1_3380	B4	0	Bcer	K	Bcer	FALSE	NA	NA	NA
Bc_ctg1_3381	B4	2.678261841	Bcer	S	Bcer	FALSE	NA	NA	NA
Bc_ctg1_3382	B4	0.446376974	Bcer	S	Bcer	FALSE	NA	NA	NA
Bc_ctg1_3383	B4	0.446376974	Bcer	NA	Bcer	FALSE	NA	NA	NA
Bc_ctg1_3384	B4	0.557971217	Bcer	NA	Bcer	FALSE	NA	NA	NA
Bc_ctg1_3385	B4	1.227536677	Bcer	S	Bcer	FALSE	NA	NA	NA
Bc_ctg1_3386	B4	0.33478273	Bcer	K	Bcer	FALSE	NA	NA	NA
Bc_ctg1_3387	B4	1.004348191	Bcer	S	Bcer	FALSE	NA	NA	NA
Bc_ctg1_3388	B4	1.115942434	Bcer	NA	Bcer	FALSE	NA	NA	NA
Bc_ctg1_3389	B4	14.17246891	Bcer	T	Bcer	FALSE	NA	NA	NA
Bc_ctg1_339	B4	12.05217829	Bcer	P	Bcer	FALSE	NA	NA	NA
Bc_ctg1_3390	B4	32.25073634	Bcer	T	Bcer	FALSE	NA	NA	NA
Bc_ctg1_3391	B4	36.49131759	Bcer	K	Bcer	FALSE	NA	NA	NA
Bc_ctg1_3392	B4	61.93480508	Bcer	S	Bcer	FALSE	NA	NA	NA
Bc_ctg1_3393	B4	37.27247729	Bcer	T	Bcer	FALSE	NA	NA	NA
Bc_ctg1_3394	B4	37.38407154	Bcer	NT	Bcer	FALSE	NA	NA	NA
Bc_ctg1_3395	B4	104.2290233	Bcer	T	Bcer	FALSE	NA	NA	NA
Bc_ctg1_3396	B4	1.115942434	Bcer	NA	Bcer	FALSE	NA	NA	NA

Bc_ctg1_3398	B4	3.013044572	Bcer	S	Bcer	FALSE	NA	NA	NA
Bc_ctg1_3399	B4	3.013044572	Bcer	NA	Bcer	FALSE	NA	NA	NA
Bc_ctg1_34	B4	1.339130921	Bcer	S	Bcer	FALSE	NA	NA	NA
Bc_ctg1_340	B4	2.901450328	Bcer	E	Bcer	FALSE	NA	NA	NA
Bc_ctg1_3400	B4	251.0870476	Bcer	S	Bcer	FALSE	NA	NA	NA
Bc_ctg1_3402	B4	1.897102138	Bcer	NA	Bcer	FALSE	NA	NA	NA
Bc_ctg1_3403	B4	1.227536677	Bcer	P	Bcer	FALSE	NA	NA	NA
Bc_ctg1_3404	B4	17.29710773	Bcer	K	Bcer	FALSE	NA	NA	NA
Bc_ctg1_3405	B4	13.05652648	Bcer	C	Bcer	FALSE	NA	NA	NA
Bc_ctg1_3406	B4	30.57682269	Bcer	M	Bcer	FALSE	NA	NA	NA
Bc_ctg1_3407	B4	2.566667598	Bcer	S	Bcer	FALSE	NA	NA	NA
Bc_ctg1_3408	B4	22.65363141	Bcer	NA	Bcer	FALSE	NA	NA	NA
Bc_ctg1_3409	B4	28.12174933	Bcer	S	Bcer	FALSE	NA	NA	NA
Bc_ctg1_341	B4	2.008696381	Bcer	K	Bcer	FALSE	NA	NA	NA
Bc_ctg1_3410	B4	18.85942713	Bcer	L	Bcer	FALSE	NA	NA	NA
Bc_ctg1_3411	B4	29.12609753	Bcer	L	Bcer	FALSE	NA	NA	NA
Bc_ctg1_3412	B4	255.1044404	Bcer	L	Bcer	FALSE	NA	NA	NA
Bc_ctg1_3413	B4	34.59421545	Bcer	S	Bcer	FALSE	NA	NA	NA
Bc_ctg1_3414	B4	57.13625262	Bcer	E	Bcer	FALSE	NA	NA	NA
Bc_ctg1_3415	B4	75.43770853	Bcer	E	Bcer	FALSE	NA	NA	NA
Bc_ctg1_3416	B4	105.7913427	Bcer	E	Bcer	FALSE	NA	NA	NA
Bc_ctg1_3417	B4	4.128987005	Bcer	K	Bcer	FALSE	NA	NA	NA
Bc_ctg1_3418	B4	0.223188487	Bcer	NA	Bcer	FALSE	NA	NA	NA
Bc_ctg1_3419	B4	1.339130921	Bcer	S	Bcer	FALSE	NA	NA	NA
Bc_ctg1_342	B4	1.673913651	Bcer	S	Bcer	FALSE	NA	NA	NA
Bc_ctg1_3420	B4	0.446376974	Bcer	S	Bcer	FALSE	NA	NA	NA
Bc_ctg1_3421	B4	1.004348191	Bcer	S	Bcer	FALSE	NA	NA	NA
Bc_ctg1_3423	B4	0.446376974	Bcer	G	Bcer	FALSE	NA	NA	NA
Bc_ctg1_3424	B4	1.673913651	Bcer	K	Bcer	FALSE	NA	NA	NA
Bc_ctg1_3425	B4	174.8681794	Bcer	L	Bcer	FALSE	NA	NA	NA
Bc_ctg1_3426	B4	70.41596758	Bcer	K	Bcer	FALSE	NA	NA	NA
Bc_ctg1_3427	B4	54.7927735	Bcer	G	Bcer	FALSE	NA	NA	NA
Bc_ctg1_3428	B4	239.3696521	Bcer	C	Bcer	FALSE	NA	NA	NA
Bc_ctg1_3429	B4	320.7218555	Bcer	C	Bcer	FALSE	NA	NA	NA
Bc_ctg1_343	B4	3.013044572	Bcer	S	Bcer	FALSE	NA	NA	NA
Bc_ctg1_3430	B4	27.67537236	Bcer	K	Bcer	FALSE	NA	NA	NA
Bc_ctg1_3431	B4	43.9681319	Bcer	V	Bcer	FALSE	NA	NA	NA
Bc_ctg1_3432	B4	1.785507894	Bcer	NA	Bcer	FALSE	NA	NA	NA
Bc_ctg1_3433	B4	14.17246891	Bcer	L	Bcer	FALSE	NA	NA	NA
Bc_ctg1_3434	B4	1.339130921	Bcer	K	Bcer	FALSE	NA	NA	NA
Bc_ctg1_3435	B4	0.557971217	Bcer	K	Bcer	FALSE	NA	NA	NA
Bc_ctg1_3436	B4	10.48985888	Bcer	D	Bcer	FALSE	NA	NA	NA
Bc_ctg1_3437	B4	28.12174933	Bcer	L	Bcer	FALSE	NA	NA	NA
Bc_ctg1_3438	B4	3.571015789	Bcer	NA	Bcer	FALSE	NA	NA	NA
Bc_ctg1_3439	B4	662.3118345	Bcer	M	Bcer	FALSE	NA	NA	NA
Bc_ctg1_344	B4	0.557971217	Bcer	S	Bcer	FALSE	NA	NA	NA
Bc_ctg1_3440	B4	0.223188487	Bcer	NA	Bcer	FALSE	NA	NA	NA
Bc_ctg1_3441	B4	6.24927763	Bcer	S	Bcer	FALSE	NA	NA	NA
Bc_ctg1_3442	B4	132.9087439	Bcer	K	Bcer	FALSE	NA	NA	NA
Bc_ctg1_3443	B4	105.45656	Bcer	S	Bcer	FALSE	NA	NA	NA
Bc_ctg1_3444	B4	67.51451725	Bcer	FG	Bcer	FALSE	NA	NA	NA
Bc_ctg1_3445	B4	42.51740673	Bcer	V	Bcer	FALSE	NA	NA	NA
Bc_ctg1_3446	B4	55.12755624	Bcer	U	Bcer	FALSE	NA	NA	NA
Bc_ctg1_3447	B4	54.23480229	Bcer	P	Bcer	FALSE	NA	NA	NA
Bc_ctg1_3448	B4	3.794204275	Bcer	NA	Bcer	FALSE	NA	NA	NA
Bc_ctg1_3449	B4	6.360871873	Bcer	NA	Bcer	FALSE	NA	NA	NA
Bc_ctg1_345	B4	22.20725443	Bcer	S	Bcer	FALSE	Bc_Ga0417192_01_rn04	Other	NA
Bc_ctg1_3450	B4	10.82464161	Bcer	NA	Bcer	FALSE	NA	NA	NA
Bc_ctg1_3451	B4	8.704350984	Bcer	IU	Bcer	FALSE	NA	NA	NA
Bc_ctg1_3452	B4	1.004348191	Bcer	NA	Bcer	FALSE	NA	NA	NA
Bc_ctg1_3453	B4	4.463769736	Bcer	K	Bcer	FALSE	NA	NA	NA
Bc_ctg1_3454	B4	0.111594243	Bcer	NA	Bcer	FALSE	NA	NA	NA

Bc_ctg1_3455	B4	17.40870197	Bcer	S	Bcer	FALSE	NA	NA	NA
Bc_ctg1_3456	B4	83.47249406	Bcer	S	Bcer	FALSE	NA	NA	NA
Bc_ctg1_3457	B4	37.94204275	Bcer	S	Bcer	FALSE	NA	NA	NA
Bc_ctg1_3458	B4	3.682610032	Bcer	NA	Bcer	FALSE	NA	NA	NA
Bc_ctg1_3459	B4	0.781159704	Bcer	S	Bcer	FALSE	NA	NA	NA
Bc_ctg1_346	B4	23.32319687	Bcer	C	Bcer	FALSE	Bc_Ga0417192_01_rn04	Ancillary biosynthesis	SMCOG1028
Bc_ctg1_3460	B4	3.682610032	Bcer	NA	Bcer	FALSE	NA	NA	NA
Bc_ctg1_3461	B4	0.892753947	Bcer	K	Bcer	FALSE	NA	NA	NA
Bc_ctg1_3462	B4	10.15507615	Bcer	M	Bcer	FALSE	NA	NA	NA
Bc_ctg1_3463	B4	71.97828699	Bcer	E	Bcer	FALSE	NA	NA	NA
Bc_ctg1_3464	B4	81.46379768	Bcer	IQ	Bcer	TRUE	NA	NA	NA
Bc_ctg1_3465	B4	36.71450608	Bcer	EG	Bcer	FALSE	NA	NA	NA
Bc_ctg1_3466	B4	2.008696381	Bcer	S	Bcer	FALSE	NA	NA	NA
Bc_ctg1_3467	B4	4.910146709	Bcer	S	Bcer	FALSE	NA	NA	NA
Bc_ctg1_3468	B4	95.74786083	Bcer	S	Bcer	FALSE	NA	NA	NA
Bc_ctg1_3469	B4	3.236233058	Bcer	M	Bcer	FALSE	NA	NA	NA
Bc_ctg1_347	B4	116.9507671	Bcer	E	Bcer	FALSE	Bc_Ga0417192_01_rn04	Ancillary biosynthesis	SMCOG1038
Bc_ctg1_3470	B4	120.2985944	Bcer	H	Bcer	FALSE	NA	NA	NA
Bc_ctg1_3471	B4	150.4290401	Bcer	H	Bcer	FALSE	NA	NA	NA
Bc_ctg1_3472	B4	334.8943244	Bcer	H	Bcer	FALSE	NA	NA	NA
Bc_ctg1_3473	B4	118.9594635	Bcer	S	Bcer	FALSE	NA	NA	NA
Bc_ctg1_3474	B4	11.49420707	Bcer	S	Bcer	FALSE	NA	NA	NA
Bc_ctg1_3475	B4	0.33478273	Bcer	NA	Bcer	FALSE	NA	NA	NA
Bc_ctg1_3476	B4	117.1739556	Bcer	NA	Bcer	FALSE	NA	NA	NA
Bc_ctg1_3477	B4	0.557971217	Bcer	K	Bcer	FALSE	NA	NA	NA
Bc_ctg1_3478	B4	8.704350984	Bcer	KT	Bcer	FALSE	NA	NA	NA
Bc_ctg1_3479	B4	102.2203269	Bcer	K	Bcer	FALSE	NA	NA	NA
Bc_ctg1_348	B4	64.61306692	Bcer	EGP	Bcer	FALSE	Bc_Ga0417192_01_rn04	Transport	SMCOG1005
Bc_ctg1_3480	B4	33.36667877	Bcer	S	Bcer	FALSE	NA	NA	NA
Bc_ctg1_3481	B4	1.897102138	Bcer	NA	Bcer	FALSE	NA	NA	NA
Bc_ctg1_3482	B4	7.25362582	Bcer	K	Bcer	FALSE	NA	NA	NA
Bc_ctg1_3483	B4	5.021740953	Bcer	S	Bcer	FALSE	NA	NA	NA
Bc_ctg1_3484	B4	2.120290624	Bcer	M	Bcer	FALSE	NA	NA	NA
Bc_ctg1_3485	B4	665.6596618	Bcer	K	Bcer	FALSE	NA	NA	NA
Bc_ctg1_3486	B4	0	Bcer	S	Bcer	FALSE	NA	NA	NA
Bc_ctg1_3487	B4	177.3232527	Bcer	S	Bcer	FALSE	NA	NA	NA
Bc_ctg1_3488	B4	374.1754981	Bcer	H	Bcer	FALSE	NA	NA	NA
Bc_ctg1_3489	B4	0.557971217	Bcer	NA	Bcer	FALSE	NA	NA	NA
Bc_ctg1_349	B4	0.557971217	Bcer	NA	Bcer	FALSE	Bc_Ga0417192_01_rn04	Other	NA
Bc_ctg1_3490	B4	58.92176051	Bcer	IQ	Bcer	TRUE	NA	NA	NA
Bc_ctg1_3491	B4	0	Bcer	M	Bcer	FALSE	NA	NA	NA
Bc_ctg1_3492	B4	1.897102138	Bcer	P	Bcer	FALSE	NA	NA	NA
Bc_ctg1_3493	B4	2.343479111	Bcer	O	Bcer	FALSE	NA	NA	NA
Bc_ctg1_3494	B4	4.910146709	Bcer	P	Bcer	FALSE	NA	NA	NA
Bc_ctg1_3495	B4	6.807248847	Bcer	P	Bcer	FALSE	NA	NA	NA
Bc_ctg1_3496	B4	726.3669302	Bcer	S	Bcer	FALSE	NA	NA	NA
Bc_ctg1_3497	B4	60.37248567	Bcer	S	Bcer	FALSE	NA	NA	NA
Bc_ctg1_3498	B4	1.450725164	Bcer	S	Bcer	FALSE	NA	NA	NA
Bc_ctg1_3499	B4	9.262322201	Bcer	S	Bcer	FALSE	NA	NA	NA
Bc_ctg1_35	B4	6.24927763	Bcer	S	Bcer	FALSE	NA	NA	NA
Bc_ctg1_350	B4	0	Bcer	S	Bcer	FALSE	Bc_Ga0417192_01_rn04	Other	NA
Bc_ctg1_3500	B4	2.566667598	Bcer	K	Bcer	FALSE	NA	NA	NA
Bc_ctg1_3501	B4	8.815945228	Bcer	U	Bcer	FALSE	NA	NA	NA
Bc_ctg1_3502	B4	5.133335196	Bcer	S	Bcer	FALSE	NA	NA	NA
Bc_ctg1_3503	B4	2.120290624	Bcer	K	Bcer	FALSE	NA	NA	NA
Bc_ctg1_3504	B4	68.07248847	Bcer	K	Bcer	FALSE	NA	NA	NA
Bc_ctg1_3505	B4	1.785507894	Bcer	E	Bcer	FALSE	NA	NA	NA
Bc_ctg1_3506	B4	16.18116529	Bcer	T	Bcer	FALSE	NA	NA	NA
Bc_ctg1_3507	B4	4.352175492	Bcer	T	Bcer	FALSE	NA	NA	NA
Bc_ctg1_3508	B4	29.01450328	Bcer	S	Bcer	FALSE	NA	NA	NA
Bc_ctg1_3509	B4	8.146379768	Bcer	NA	Bcer	FALSE	NA	NA	NA
Bc_ctg1_351	B4	0	Bcer	NA	Bcer	FALSE	Bc_Ga0417192_01_rn04	Other	NA

Bc_ctg1_3510	B4	3.124638815	Bcer	NA	Bcer	FALSE	NA	NA	NA
Bc_ctg1_3511	B4	14.28406315	Bcer	O	Bcer	FALSE	NA	NA	NA
Bc_ctg1_3512	B4	30.13044572	Bcer	NT	Bcer	FALSE	NA	NA	NA
Bc_ctg1_3513	B4	37.27247729	Bcer	D	Bcer	FALSE	NA	NA	NA
Bc_ctg1_3515	B4	1.673913651	Bcer	D	Bcer	FALSE	NA	NA	NA
Bc_ctg1_3516	B4	10.71304737	Bcer	C	Bcer	FALSE	NA	NA	NA
Bc_ctg1_3517	B4	21.42609473	Bcer	C	Bcer	FALSE	NA	NA	NA
Bc_ctg1_3518	B4	7.365220064	Bcer	S	Bcer	FALSE	NA	NA	NA
Bc_ctg1_3519	B4	1.339130921	Bcer	NA	Bcer	FALSE	NA	NA	NA
Bc_ctg1_352	B4	0	Bcer	NA	Bcer	FALSE	Bc_Ga0417192_01_rn04	Other	NA
Bc_ctg1_3520	B4	87.37829258	Bcer	K	Bcer	FALSE	NA	NA	NA
Bc_ctg1_3521	B4	0.111594243	Bcer	NA	Bcer	FALSE	NA	NA	NA
Bc_ctg1_3522	B4	2.678261841	Bcer	NA	Bcer	FALSE	NA	NA	NA
Bc_ctg1_3523	B4	10.04348191	Bcer	K	Bcer	FALSE	NA	NA	NA
Bc_ctg1_3524	B4	23.21160263	Bcer	S	Bcer	FALSE	NA	NA	NA
Bc_ctg1_3525	B4	29.23769177	Bcer	U	Bcer	FALSE	NA	NA	NA
Bc_ctg1_3526	B4	127.440626	Bcer	L	Bcer	FALSE	NA	NA	NA
Bc_ctg1_3527	B4	249.413134	Bcer	L	Bcer	FALSE	NA	NA	NA
Bc_ctg1_3528	B4	4.798552466	Bcer	K	Bcer	FALSE	NA	NA	NA
Bc_ctg1_3529	B4	0.111594243	Bcer	S	Bcer	FALSE	NA	NA	NA
Bc_ctg1_353	B4	3.682610032	Bcer	NA	Bcer	FALSE	Bc_Ga0417192_01_rn04	Other	NA
Bc_ctg1_3530	B4	1.673913651	Bcer	S	Bcer	FALSE	NA	NA	NA
Bc_ctg1_3531	B4	0	Bcer	S	Bcer	FALSE	NA	NA	NA
Bc_ctg1_3532	B4	2.008696381	Bcer	S	Bcer	FALSE	NA	NA	NA
Bc_ctg1_3533	B4	0.111594243	Bcer	S	Bcer	FALSE	NA	NA	NA
Bc_ctg1_3534	B4	0.111594243	Bcer	S	Bcer	FALSE	NA	NA	NA
Bc_ctg1_3535	B4	0.33478273	Bcer	NA	Bcer	FALSE	NA	NA	NA
Bc_ctg1_3536	B4	37.04928881	Bcer	Q	Bcer	TRUE	NA	NA	NA
Bc_ctg1_3537	B4	7.365220064	Bcer	NA	Bcer	FALSE	NA	NA	NA
Bc_ctg1_3538	B4	2056.570311	Bcer	E	Bcer	FALSE	NA	NA	NA
Bc_ctg1_3539	B4	31.91595361	Bcer	S	Bcer	FALSE	NA	NA	NA
Bc_ctg1_354	B4	18.30145592	Bcer	IQ	Bcer	TRUE	Bc_Ga0417192_01_rn04	Ancillary biosynthesis	SMCOG1001
Bc_ctg1_3540	B4	0.446376974	Bcer	S	Bcer	FALSE	NA	NA	NA
Bc_ctg1_3541	B4	5.57971217	Bcer	E	Bcer	FALSE	NA	NA	NA
Bc_ctg1_3542	B4	131.0116417	Bcer	H	Bcer	FALSE	NA	NA	NA
Bc_ctg1_3543	B4	1594.793332	Bcer	P	Bcer	FALSE	NA	NA	NA
Bc_ctg1_3544	B4	7.030437334	Bcer	P	Bcer	FALSE	NA	NA	NA
Bc_ctg1_3545	B4	14.3956574	Bcer	G	Bcer	FALSE	NA	NA	NA
Bc_ctg1_3546	B4	33.14349029	Bcer	K	Bcer	FALSE	NA	NA	NA
Bc_ctg1_3547	B4	134.0246863	Bcer	S	Bcer	FALSE	NA	NA	NA
Bc_ctg1_3548	B4	62.26958781	Bcer	S	Bcer	FALSE	NA	NA	NA
Bc_ctg1_3549	B4	76.66524521	Bcer	M	Bcer	FALSE	Bc_Ga0417192_01_rn24	Other	NA
Bc_ctg1_355	B4	100.3232248	Bcer	S	Bcer	FALSE	Bc_Ga0417192_01_rn04	Core biosynthesis	None
Bc_ctg1_3551	B4	3.682610032	Bcer	S	Bcer	FALSE	Bc_Ga0417192_01_rn24	Other	NA
Bc_ctg1_3552	B4	16.40435378	Bcer	S	Bcer	FALSE	Bc_Ga0417192_01_rn24	Other	NA
Bc_ctg1_3554	B4	122.0841023	Bcer	S	Bcer	FALSE	Bc_Ga0417192_01_rn24	Ancillary biosynthesis	SMCOG1179
Bc_ctg1_3555	B4	2.231884868	Bcer	S	Bcer	FALSE	Bc_Ga0417192_01_rn24	Other	NA
Bc_ctg1_3556	B4	414.2378315	Bcer	O	Bcer	FALSE	Bc_Ga0417192_01_rn24	Other	SMCOG1223
Bc_ctg1_3557	B4	90.94930836	Bcer	S	Bcer	FALSE	Bc_Ga0417192_01_rn24	Other	NA
Bc_ctg1_3558	B4	0.223188487	Bcer	I	Bcer	FALSE	Bc_Ga0417192_01_rn24	Other	NA
Bc_ctg1_3559	B4	0.223188487	Bcer	GM	Bcer	FALSE	Bc_Ga0417192_01_rn24	Other	NA
Bc_ctg1_356	B4	31.80435937	Bcer	S	Bcer	FALSE	Bc_Ga0417192_01_rn04	Other	NA
Bc_ctg1_3560	B4	0.111594243	Bcer	S	Bcer	FALSE	Bc_Ga0417192_01_rn24	Other	NA
Bc_ctg1_3561	B4	100.5464133	Bcer	I	Bcer	FALSE	Bc_Ga0417192_01_rn24	Core biosynthesis	SMCOG1084
Bc_ctg1_3562	B4	343.4870812	Bcer	I	Bcer	FALSE	Bc_Ga0417192_01_rn24	Core biosynthesis	SMCOG1022
Bc_ctg1_3563	B4	1.897102138	Bcer	O	Bcer	FALSE	Bc_Ga0417192_01_rn24	Other	NA
Bc_ctg1_3564	B4	3.459421545	Bcer	S	Bcer	FALSE	Bc_Ga0417192_01_rn24	Other	NA
Bc_ctg1_3565	B4	147.4159955	Bcer	J	Bcer	FALSE	Bc_Ga0417192_01_rn24	Other	NA
Bc_ctg1_3566	B4	8.704350984	Bcer	S	Bcer	FALSE	Bc_Ga0417192_01_rn24	Other	NA
Bc_ctg1_3567	B4	548.5973005	Bcer	E	Bcer	FALSE	Bc_Ga0417192_01_rn24	Transport	SMCOG1068
Bc_ctg1_3568	B4	54.12320804	Bcer	E	Bcer	FALSE	Bc_Ga0417192_01_rn24	Other	NA
Bc_ctg1_3569	B4	500.0538046	Bcer	P	Bcer	FALSE	Bc_Ga0417192_01_rn24	Transport	SMCOG1118

Bc_ctg1_357	B4	55.46233897	Bcer	P	Bcer	FALSE	Bc_Ga0417192_01_rn04	Other	NA
Bc_ctg1_3570	B4	609.1929747	Bcer	EP	Bcer	FALSE	Bc_Ga0417192_01_rn24	Transport	SMCOG1085
Bc_ctg1_3571	B4	1261.01495	Bcer	P	Bcer	FALSE	Bc_Ga0417192_01_rn24	Transport	SMCOG1000
Bc_ctg1_3572	B4	853.8075562	Bcer	E	Bcer	FALSE	Bc_Ga0417192_01_rn24	Transport	SMCOG1000
Bc_ctg1_3573	B4	0.33478273	Bcer	L	Bcer	FALSE	NA	NA	NA
Bc_ctg1_3574	B4	0	Bcer	L	Bcer	FALSE	NA	NA	NA
Bc_ctg1_3575	B4	13.94928042	Bcer	V	Bcer	FALSE	NA	NA	NA
Bc_ctg1_3576	B4	770.1118736	Bcer	E	Bcer	FALSE	NA	NA	NA
Bc_ctg1_3577	B4	4.240581249	Bcer	NA	Bcer	FALSE	NA	NA	NA
Bc_ctg1_3578	B4	1039.611971	Bcer	K	Bcer	FALSE	NA	NA	NA
Bc_ctg1_3579	B4	0.892753947	Bcer	P	Bcer	FALSE	NA	NA	NA
Bc_ctg1_358	B4	0.781159704	Bcer	NA	Bcer	FALSE	Bc_Ga0417192_01_rn04	Other	NA
Bc_ctg1_3580	B4	141.0551236	Bcer	NOT	Bcer	FALSE	NA	NA	NA
Bc_ctg1_3581	B4	67.06814028	Bcer	I	Bcer	FALSE	NA	NA	NA
Bc_ctg1_3582	B4	5.468117926	Bcer	S	Bcer	FALSE	NA	NA	NA
Bc_ctg1_3583	B4	633.9668967	Bcer	E	Bcer	FALSE	NA	NA	NA
Bc_ctg1_3584	B4	40.73189884	Bcer	Q	Bcer	TRUE	Bc_Ga0417192_01_rn25	Other	NA
Bc_ctg1_3585	B4	16.40435378	Bcer	S	Bcer	FALSE	Bc_Ga0417192_01_rn25	Other	NA
Bc_ctg1_3586	B4	16.85073075	Bcer	S	Bcer	FALSE	Bc_Ga0417192_01_rn25	Other	NA
Bc_ctg1_3587	B4	44.52610311	Bcer	S	Bcer	FALSE	Bc_Ga0417192_01_rn25	Other	NA
Bc_ctg1_3588	B4	252.7609613	Bcer	S	Bcer	FALSE	Bc_Ga0417192_01_rn25	Other	NA
Bc_ctg1_3589	B4	318.3783764	Bcer	G	Bcer	FALSE	Bc_Ga0417192_01_rn25	Other	NA
Bc_ctg1_359	B4	0.781159704	Bcer	J	Bcer	FALSE	Bc_Ga0417192_01_rn04	Ancillary biosynthesis	SMCOG1111
Bc_ctg1_3590	B4	235.3522593	Bcer	J	Bcer	FALSE	Bc_Ga0417192_01_rn25	Ancillary biosynthesis	SMCOG1279
Bc_ctg1_3591	B4	1.227536677	Bcer	UW	Bcer	FALSE	Bc_Ga0417192_01_rn25	Other	NA
Bc_ctg1_3592	B4	4.017392762	Bcer	T	Bcer	FALSE	Bc_Ga0417192_01_rn25	Other	NA
Bc_ctg1_3593	B4	0.446376974	Bcer	D	Bcer	FALSE	Bc_Ga0417192_01_rn25	Other	NA
Bc_ctg1_3594	B4	0.33478273	Bcer	M	Bcer	FALSE	Bc_Ga0417192_01_rn25	Core biosynthesis	None
Bc_ctg1_3595	B4	0.223188487	Bcer	H	Bcer	FALSE	Bc_Ga0417192_01_rn25	Other	NA
Bc_ctg1_3596	B4	0.111594243	Bcer	S	Bcer	FALSE	Bc_Ga0417192_01_rn25	Other	NA
Bc_ctg1_3597	B4	0	Bcer	K	Bcer	FALSE	Bc_Ga0417192_01_rn25	Other	NA
Bc_ctg1_3598	B4	1.785507894	Bcer	M	Bcer	FALSE	Bc_Ga0417192_01_rn25	Core biosynthesis	None
Bc_ctg1_3599	B4	1.115942434	Bcer	H	Bcer	FALSE	Bc_Ga0417192_01_rn25	Other	NA
Bc_ctg1_36	B4	1.115942434	Bcer	K	Bcer	FALSE	NA	NA	NA
Bc_ctg1_360	B4	9.708699175	Bcer	P	Bcer	FALSE	Bc_Ga0417192_01_rn04	Transport	SMCOG1033
Bc_ctg1_3600	B4	3.013044572	Bcer	S	Bcer	FALSE	Bc_Ga0417192_01_rn25	Other	NA
Bc_ctg1_3601	B4	0.223188487	Bcer	S	Bcer	FALSE	Bc_Ga0417192_01_rn25	Other	NA
Bc_ctg1_3602	B4	0.781159704	Bcer	M	Bcer	FALSE	Bc_Ga0417192_01_rn25	Ancillary biosynthesis	SMCOG1064
Bc_ctg1_3603	B4	1.450725164	Bcer	M	Bcer	FALSE	Bc_Ga0417192_01_rn25	Core biosynthesis	None
Bc_ctg1_3604	B4	8.481162498	Bcer	M	Bcer	FALSE	Bc_Ga0417192_01_rn25	Core biosynthesis	SMCOG1010
Bc_ctg1_3605	B4	10.04348191	Bcer	M	Bcer	FALSE	Bc_Ga0417192_01_rn25	Core biosynthesis	SMCOG1010
Bc_ctg1_3606	B4	269.5000978	Bcer	I	Bcer	FALSE	Bc_Ga0417192_01_rn25	Ancillary biosynthesis	SMCOG1001
Bc_ctg1_3607	B4	0.111594243	Bcer	S	Bcer	FALSE	Bc_Ga0417192_01_rn25	Other	NA
Bc_ctg1_3608	B4	0	Bcer	S	Bcer	FALSE	Bc_Ga0417192_01_rn25	Other	NA
Bc_ctg1_3609	B4	0.33478273	Bcer	S	Bcer	FALSE	Bc_Ga0417192_01_rn25	Other	NA
Bc_ctg1_361	B4	4.128987005	Bcer	P	Bcer	FALSE	Bc_Ga0417192_01_rn04	Transport	SMCOG1011
Bc_ctg1_3610	B4	0.557971217	Bcer	NA	Bcer	FALSE	Bc_Ga0417192_01_rn25	Other	NA
Bc_ctg1_3611	B4	3.794204275	Bcer	S	Bcer	FALSE	Bc_Ga0417192_01_rn25	Other	NA
Bc_ctg1_3612	B4	144.0681682	Bcer	L	Bcer	FALSE	Bc_Ga0417192_01_rn25	Other	NA
Bc_ctg1_3613	B4	380.2015872	Bcer	S	Bcer	FALSE	Bc_Ga0417192_01_rn25	Other	NA
Bc_ctg1_3614	B4	371.7204247	Bcer	J	Bcer	FALSE	Bc_Ga0417192_01_rn25	Other	NA
Bc_ctg1_3615	B4	58.25219505	Bcer	P	Bcer	FALSE	Bc_Ga0417192_01_rn25	Ancillary biosynthesis	SMCOG1098
Bc_ctg1_3616	B4	115.8348246	Bcer	EG	Bcer	FALSE	Bc_Ga0417192_01_rn25	Transport	SMCOG1166
Bc_ctg1_3617	B4	1.227536677	Bcer	S	Bcer	FALSE	Bc_Ga0417192_01_rn25	Other	NA
Bc_ctg1_3618	B4	1.450725164	Bcer	N	Bcer	FALSE	NA	NA	NA
Bc_ctg1_3619	B4	82.35655162	Bcer	E	Bcer	FALSE	NA	NA	NA
Bc_ctg1_362	B4	7.030437334	Bcer	P	Bcer	FALSE	Bc_Ga0417192_01_rn04	Transport	SMCOG1011
Bc_ctg1_3620	B4	7.25362582	Bcer	EH	Bcer	FALSE	NA	NA	NA
Bc_ctg1_3621	B4	2.455073355	Bcer	EH	Bcer	FALSE	NA	NA	NA
Bc_ctg1_3622	B4	3.459421545	Bcer	E	Bcer	FALSE	NA	NA	NA
Bc_ctg1_3623	B4	2.789856085	Bcer	E	Bcer	FALSE	NA	NA	NA
Bc_ctg1_3624	B4	2.120290624	Bcer	E	Bcer	FALSE	NA	NA	NA

Bc_ctg1_3625	B4	9.039133715	Bcer	E	Bcer	FALSE	NA	NA	NA
Bc_ctg1_3626	B4	7.142031577	Bcer	E	Bcer	FALSE	Bc_Ga0417192_01_rn26	Other	NA
Bc_ctg1_3627	B4	2.343479111	Bcer	NA	Bcer	FALSE	Bc_Ga0417192_01_rn26	Other	NA
Bc_ctg1_3628	B4	5.468117926	Bcer	C	Bcer	FALSE	Bc_Ga0417192_01_rn26	Other	NA
Bc_ctg1_3629	B4	36.82610032	Bcer	S	Bcer	FALSE	Bc_Ga0417192_01_rn26	Other	NA
Bc_ctg1_363	B4	4.463769736	Bcer	S	Bcer	FALSE	Bc_Ga0417192_01_rn04	Other	NA
Bc_ctg1_3630	B4	13.27971496	Bcer	NA	Bcer	FALSE	Bc_Ga0417192_01_rn26	Other	NA
Bc_ctg1_3631	B4	0.66956546	Bcer	M	Bcer	FALSE	Bc_Ga0417192_01_rn26	Other	NA
Bc_ctg1_3632	B4	1.450725164	Bcer	S	Bcer	FALSE	Bc_Ga0417192_01_rn26	Other	NA
Bc_ctg1_3633	B4	0	Bcer	NA	Bcer	FALSE	Bc_Ga0417192_01_rn26	Other	NA
Bc_ctg1_3634	B4	10.48985888	Bcer	P	Bcer	FALSE	Bc_Ga0417192_01_rn26	Ancillary biosynthesis	SMCOG1175
Bc_ctg1_3635	B4	1.227536677	Bcer	S	Bcer	FALSE	Bc_Ga0417192_01_rn26	Other	NA
Bc_ctg1_3636	B4	2.678261841	Bcer	NA	Bcer	FALSE	Bc_Ga0417192_01_rn26	Other	NA
Bc_ctg1_3637	B4	2.343479111	Bcer	C	Bcer	FALSE	Bc_Ga0417192_01_rn26	Core biosynthesis	None
Bc_ctg1_3638	B4	1.450725164	Bcer	S	Bcer	FALSE	Bc_Ga0417192_01_rn26	Core biosynthesis	None
Bc_ctg1_3639	B4	2.231884868	Bcer	H	Bcer	FALSE	Bc_Ga0417192_01_rn26	Core biosynthesis	None
Bc_ctg1_364	B4	13.16812072	Bcer	S	Bcer	FALSE	Bc_Ga0417192_01_rn04	Other	NA
Bc_ctg1_3640	B4	1693.777426	Bcer	C	Bcer	FALSE	Bc_Ga0417192_01_rn26	Other	NA
Bc_ctg1_3641	B4	2919.082219	Bcer	C	Bcer	FALSE	Bc_Ga0417192_01_rn26	Other	NA
Bc_ctg1_3642	B4	0.33478273	Bcer	NA	Bcer	FALSE	Bc_Ga0417192_01_rn26	Other	NA
Bc_ctg1_3643	B4	0.892753947	Bcer	NA	Bcer	FALSE	Bc_Ga0417192_01_rn26	Other	NA
Bc_ctg1_3644	B4	1.004348191	Bcer	NA	Bcer	FALSE	Bc_Ga0417192_01_rn26	Other	NA
Bc_ctg1_3645	B4	3.794204275	Bcer	NA	Bcer	FALSE	Bc_Ga0417192_01_rn26	Other	NA
Bc_ctg1_3646	B4	125.4319296	Bcer	NA	Bcer	FALSE	Bc_Ga0417192_01_rn26	Other	NA
Bc_ctg1_3647	B4	0.892753947	Bcer	NA	Bcer	FALSE	NA	NA	NA
Bc_ctg1_3648	B4	3.236233058	Bcer	NA	Bcer	FALSE	NA	NA	NA
Bc_ctg1_3649	B4	72.98263518	Bcer	M	Bcer	FALSE	NA	NA	NA
Bc_ctg1_365	B4	46.64639374	Bcer	S	Bcer	FALSE	Bc_Ga0417192_01_rn04	Other	NA
Bc_ctg1_3650	B4	0.111594243	Bcer	U	Bcer	FALSE	NA	NA	NA
Bc_ctg1_3651	B4	1.004348191	Bcer	S	Bcer	FALSE	NA	NA	NA
Bc_ctg1_3652	B4	1.673913651	Bcer	M	Bcer	FALSE	NA	NA	NA
Bc_ctg1_3653	B4	5.021740953	Bcer	S	Bcer	FALSE	NA	NA	NA
Bc_ctg1_3654	B4	64.27828419	Bcer	K	Bcer	FALSE	NA	NA	NA
Bc_ctg1_3655	B4	0	Bcer	NA	Bcer	FALSE	NA	NA	NA
Bc_ctg1_3656	B4	20.64493503	Bcer	V	Bcer	FALSE	NA	NA	NA
Bc_ctg1_3657	B4	58.14060081	Bcer	C	Bcer	FALSE	NA	NA	NA
Bc_ctg1_3658	B4	60.14929719	Bcer	P	Bcer	FALSE	NA	NA	NA
Bc_ctg1_3659	B4	44.07972614	Bcer	E	Bcer	FALSE	NA	NA	NA
Bc_ctg1_366	B4	5.57971217	Bcer	S	Bcer	FALSE	Bc_Ga0417192_01_rn04	Other	NA
Bc_ctg1_3660	B4	33.03189604	Bcer	E	Bcer	FALSE	NA	NA	NA
Bc_ctg1_3661	B4	70.86234455	Bcer	P	Bcer	FALSE	NA	NA	NA
Bc_ctg1_3662	B4	9.931887662	Bcer	I	Bcer	FALSE	NA	NA	NA
Bc_ctg1_3663	B4	156.9015062	Bcer	S	Bcer	FALSE	NA	NA	NA
Bc_ctg1_3664	B4	1.004348191	Bcer	S	Bcer	FALSE	NA	NA	NA
Bc_ctg1_3665	B4	33.25508453	Bcer	J	Bcer	FALSE	NA	NA	NA
Bc_ctg1_3666	B4	48.20871314	Bcer	S	Bcer	FALSE	NA	NA	NA
Bc_ctg1_3667	B4	1.004348191	Bcer	J	Bcer	FALSE	NA	NA	NA
Bc_ctg1_3668	B4	61.93480508	Bcer	C	Bcer	FALSE	NA	NA	NA
Bc_ctg1_3669	B4	76.8884337	Bcer	C	Bcer	FALSE	NA	NA	NA
Bc_ctg1_367	B4	0.111594243	Bcer	S	Bcer	FALSE	Bc_Ga0417192_01_rn04	Other	NA
Bc_ctg1_3670	B4	3.794204275	Bcer	KT	Bcer	FALSE	NA	NA	NA
Bc_ctg1_3671	B4	19.86377532	Bcer	S	Bcer	FALSE	NA	NA	NA
Bc_ctg1_3672	B4	10.60145312	Bcer	T	Bcer	FALSE	NA	NA	NA
Bc_ctg1_3673	B4	39.95073913	Bcer	T	Bcer	FALSE	NA	NA	NA
Bc_ctg1_3674	B4	586.9857202	Bcer	K	Bcer	FALSE	NA	NA	NA
Bc_ctg1_3675	B4	218.3899343	Bcer	C	Bcer	FALSE	NA	NA	NA
Bc_ctg1_3676	B4	932.593092	Bcer	C	Bcer	FALSE	NA	NA	NA
Bc_ctg1_3677	B4	362.0117256	Bcer	S	Bcer	FALSE	NA	NA	NA
Bc_ctg1_3678	B4	34.03624423	Bcer	NOU	Bcer	FALSE	NA	NA	NA
Bc_ctg1_3679	B4	12.72174375	Bcer	S	Bcer	FALSE	NA	NA	NA
Bc_ctg1_368	B4	0.446376974	Bcer	S	Bcer	FALSE	Bc_Ga0417192_01_rn04	Other	NA
Bc_ctg1_3680	B4	348.7320106	Bcer	P	Bcer	FALSE	NA	NA	NA

Bc_ctg1_3681	B4	10.82464161	Bcer	P	Bcer	FALSE	NA	NA	NA
Bc_ctg1_3682	B4	0.446376974	Bcer	P	Bcer	FALSE	NA	NA	NA
Bc_ctg1_3683	B4	1.115942434	Bcer	S	Bcer	FALSE	NA	NA	NA
Bc_ctg1_3684	B4	327.6406986	Bcer	I	Bcer	FALSE	NA	NA	NA
Bc_ctg1_3685	B4	781.271298	Bcer	S	Bcer	FALSE	NA	NA	NA
Bc_ctg1_3686	B4	259.1218332	Bcer	K	Bcer	FALSE	NA	NA	NA
Bc_ctg1_3687	B4	82.69133435	Bcer	S	Bcer	FALSE	NA	NA	NA
Bc_ctg1_3688	B4	146.7464301	Bcer	IQ	Bcer	TRUE	NA	NA	NA
Bc_ctg1_3689	B4	168.5073075	Bcer	I	Bcer	FALSE	NA	NA	NA
Bc_ctg1_369	B4	2.120290624	Bcer	K	Bcer	FALSE	Bc_Ga0417192_01_rn04	Regulation	SMCOG1014
Bc_ctg1_3690	B4	35.4869694	Bcer	E	Bcer	FALSE	NA	NA	NA
Bc_ctg1_3691	B4	91.39568534	Bcer	P	Bcer	FALSE	NA	NA	NA
Bc_ctg1_3694	B4	0	Bcer	P	Bcer	FALSE	NA	NA	NA
Bc_ctg1_3695	B4	1.227536677	Bcer	P	Bcer	FALSE	NA	NA	NA
Bc_ctg1_3696	B4	2.343479111	Bcer	P	Bcer	FALSE	NA	NA	NA
Bc_ctg1_3697	B4	0.446376974	Bcer	S	Bcer	FALSE	NA	NA	NA
Bc_ctg1_3698	B4	1.450725164	Bcer	E	Bcer	FALSE	NA	NA	NA
Bc_ctg1_3699	B4	3.571015789	Bcer	E	Bcer	FALSE	NA	NA	NA
Bc_ctg1_37	B4	1.004348191	Bcer	S	Bcer	FALSE	NA	NA	NA
Bc_ctg1_370	B4	1.004348191	Bcer	C	Bcer	FALSE	Bc_Ga0417192_01_rn04	Ancillary biosynthesis	SMCOG1028
Bc_ctg1_3700	B4	3.013044572	Bcer	K	Bcer	FALSE	NA	NA	NA
Bc_ctg1_3702	B4	37.71885427	Bcer	M	Bcer	FALSE	NA	NA	NA
Bc_ctg1_3703	B4	1.227536677	Bcer	E	Bcer	FALSE	NA	NA	NA
Bc_ctg1_3704	B4	3.347827302	Bcer	S	Bcer	FALSE	NA	NA	NA
Bc_ctg1_3705	B4	1.562319407	Bcer	S	Bcer	FALSE	NA	NA	NA
Bc_ctg1_3706	B4	1.004348191	Bcer	S	Bcer	FALSE	NA	NA	NA
Bc_ctg1_3707	B4	31.80435937	Bcer	T	Bcer	FALSE	NA	NA	NA
Bc_ctg1_3708	B4	14.61884588	Bcer	K	Bcer	FALSE	NA	NA	NA
Bc_ctg1_3709	B4	898.1104708	Bcer	E	Bcer	FALSE	NA	NA	NA
Bc_ctg1_371	B4	8.927539471	Bcer	G	Bcer	FALSE	Bc_Ga0417192_01_rn04	Ancillary biosynthesis	SMCOG1101
Bc_ctg1_3710	B4	103.6710521	Bcer	S	Bcer	FALSE	NA	NA	NA
Bc_ctg1_3711	B4	0	Bcer	NA	Bcer	FALSE	NA	NA	NA
Bc_ctg1_3712	B4	112.0406204	Bcer	T	Bcer	FALSE	NA	NA	NA
Bc_ctg1_3713	B4	67.62611149	Bcer	F	Bcer	FALSE	NA	NA	NA
Bc_ctg1_3714	B4	63.83190722	Bcer	H	Bcer	FALSE	NA	NA	NA
Bc_ctg1_3715	B4	59.36813748	Bcer	H	Bcer	FALSE	NA	NA	NA
Bc_ctg1_3716	B4	44.97248009	Bcer	S	Bcer	FALSE	NA	NA	NA
Bc_ctg1_3717	B4	3.124638815	Bcer	S	Bcer	FALSE	NA	NA	NA
Bc_ctg1_3718	B4	0.781159704	Bcer	NA	Bcer	FALSE	NA	NA	NA
Bc_ctg1_3719	B4	39.50436216	Bcer	G	Bcer	FALSE	NA	NA	NA
Bc_ctg1_372	B4	3.905798519	Bcer	S	Bcer	FALSE	Bc_Ga0417192_01_rn04	Other	NA
Bc_ctg1_3720	B4	87.93626379	Bcer	K	Bcer	FALSE	NA	NA	NA
Bc_ctg1_3721	B4	3.682610032	Bcer	NA	Bcer	FALSE	NA	NA	NA
Bc_ctg1_3722	B4	15.40000559	Bcer	NA	Bcer	FALSE	NA	NA	NA
Bc_ctg1_3723	B4	99.5420651	Bcer	F	Bcer	FALSE	NA	NA	NA
Bc_ctg1_3724	B4	1170.735207	Bcer	F	Bcer	FALSE	NA	NA	NA
Bc_ctg1_3725	B4	180.0015146	Bcer	F	Bcer	FALSE	NA	NA	NA
Bc_ctg1_3726	B4	425.955227	Bcer	L	Bcer	FALSE	NA	NA	NA
Bc_ctg1_3727	B4	134.0246863	Bcer	F	Bcer	FALSE	NA	NA	NA
Bc_ctg1_3728	B4	6.24927763	Bcer	K	Bcer	FALSE	NA	NA	NA
Bc_ctg1_3729	B4	23.76957384	Bcer	V	Bcer	FALSE	NA	NA	NA
Bc_ctg1_373	B4	5.021740953	Bcer	L	Bcer	FALSE	Bc_Ga0417192_01_rn04	Other	NA
Bc_ctg1_3730	B4	13.94928042	Bcer	NA	Bcer	FALSE	NA	NA	NA
Bc_ctg1_3731	B4	52.00291742	Bcer	V	Bcer	FALSE	NA	NA	NA
Bc_ctg1_3732	B4	74.21017185	Bcer	S	Bcer	FALSE	NA	NA	NA
Bc_ctg1_3733	B4	38.27682548	Bcer	S	Bcer	FALSE	NA	NA	NA
Bc_ctg1_3734	B4	0	Bcer	NA	Bcer	FALSE	NA	NA	NA
Bc_ctg1_3735	B4	4.686958222	Bcer	K	Bcer	FALSE	NA	NA	NA
Bc_ctg1_3736	B4	3.013044572	Bcer	S	Bcer	FALSE	NA	NA	NA
Bc_ctg1_3737	B4	3.459421545	Bcer	Q	Bcer	TRUE	NA	NA	NA
Bc_ctg1_3738	B4	32.58551907	Bcer	S	Bcer	FALSE	NA	NA	NA
Bc_ctg1_3739	B4	7.811597037	Bcer	V	Bcer	FALSE	NA	NA	NA

Bc_ctg1_374	B4	0.557971217	Bcer	NA	Bcer	FALSE	Bc_Ga0417192_01_rn04	Other	NA
Bc_ctg1_3740	B4	17.63189046	Bcer	S	Bcer	FALSE	NA	NA	NA
Bc_ctg1_3741	B4	236.3566075	Bcer	M	Bcer	FALSE	NA	NA	NA
Bc_ctg1_3742	B4	89.61017744	Bcer	IQ	Bcer	TRUE	NA	NA	NA
Bc_ctg1_3743	B4	562.3233924	Bcer	M	Bcer	FALSE	NA	NA	NA
Bc_ctg1_3744	B4	938.2843984	Bcer	Q	Bcer	TRUE	NA	NA	NA
Bc_ctg1_3745	B4	2.678261841	Bcer	NA	Bcer	FALSE	NA	NA	NA
Bc_ctg1_3746	B4	1.450725164	Bcer	E	Bcer	FALSE	NA	NA	NA
Bc_ctg1_3747	B4	0.111594243	Bcer	NA	Bcer	FALSE	NA	NA	NA
Bc_ctg1_3748	B4	2.678261841	Bcer	C	Bcer	FALSE	NA	NA	NA
Bc_ctg1_3749	B4	5.356523683	Bcer	NA	Bcer	FALSE	NA	NA	NA
Bc_ctg1_375	B4	4.910146709	Bcer	G	Bcer	FALSE	Bc_Ga0417192_01_rn04	Core biosynthesis	SMCOG1257
Bc_ctg1_3750	B4	0	Bcer	NA	Bcer	FALSE	NA	NA	NA
Bc_ctg1_3751	B4	5.133335196	Bcer	S	Bcer	FALSE	NA	NA	NA
Bc_ctg1_3752	B4	1.673913651	Bcer	NA	Bcer	FALSE	NA	NA	NA
Bc_ctg1_3754	B4	0.892753947	Bcer	NA	Bcer	FALSE	NA	NA	NA
Bc_ctg1_3755	B4	14.28406315	Bcer	V	Bcer	FALSE	NA	NA	NA
Bc_ctg1_3756	B4	216.3812379	Bcer	V	Bcer	FALSE	NA	NA	NA
Bc_ctg1_3757	B4	62.15799357	Bcer	CO	Bcer	FALSE	NA	NA	NA
Bc_ctg1_3758	B4	5.356523683	Bcer	S	Bcer	FALSE	NA	NA	NA
Bc_ctg1_3759	B4	14.84203437	Bcer	K	Bcer	FALSE	NA	NA	NA
Bc_ctg1_376	B4	10.82464161	Bcer	Q	Bcer	TRUE	Bc_Ga0417192_01_rn04	Other	NA
Bc_ctg1_3761	B4	11.27101858	Bcer	C	Bcer	FALSE	NA	NA	NA
Bc_ctg1_3762	B4	296.0595277	Bcer	P	Bcer	FALSE	NA	NA	NA
Bc_ctg1_3763	B4	0.66956546	Bcer	NA	Bcer	FALSE	NA	NA	NA
Bc_ctg1_3764	B4	0.892753947	Bcer	S	Bcer	FALSE	NA	NA	NA
Bc_ctg1_3766	B4	0.66956546	Bcer	S	Bcer	FALSE	NA	NA	NA
Bc_ctg1_3767	B4	2.008696381	Bcer	S	Bcer	FALSE	NA	NA	NA
Bc_ctg1_3768	B4	12.49855526	Bcer	E	Bcer	FALSE	NA	NA	NA
Bc_ctg1_3769	B4	9.262322201	Bcer	E	Bcer	FALSE	NA	NA	NA
Bc_ctg1_377	B4	116.1696074	Bcer	E	Bcer	FALSE	Bc_Ga0417192_01_rn04	Other	NA
Bc_ctg1_3770	B4	2.343479111	Bcer	E	Bcer	FALSE	NA	NA	NA
Bc_ctg1_3771	B4	5.9144949	Bcer	EH	Bcer	FALSE	NA	NA	NA
Bc_ctg1_3772	B4	10.93623585	Bcer	E	Bcer	FALSE	NA	NA	NA
Bc_ctg1_3773	B4	2.566667598	Bcer	CE	Bcer	FALSE	NA	NA	NA
Bc_ctg1_3774	B4	2.231884868	Bcer	E	Bcer	FALSE	NA	NA	NA
Bc_ctg1_3775	B4	1.785507894	Bcer	E	Bcer	FALSE	NA	NA	NA
Bc_ctg1_3776	B4	7.700002794	Bcer	E	Bcer	FALSE	NA	NA	NA
Bc_ctg1_3777	B4	5.9144949	Bcer	E	Bcer	FALSE	NA	NA	NA
Bc_ctg1_3778	B4	6.695654603	Bcer	E	Bcer	FALSE	NA	NA	NA
Bc_ctg1_3779	B4	1.785507894	Bcer	E	Bcer	FALSE	NA	NA	NA
Bc_ctg1_378	B4	0.33478273	Bcer	E	Bcer	FALSE	Bc_Ga0417192_01_rn04	Other	NA
Bc_ctg1_3780	B4	1.897102138	Bcer	E	Bcer	FALSE	NA	NA	NA
Bc_ctg1_3781	B4	0.66956546	Bcer	E	Bcer	FALSE	NA	NA	NA
Bc_ctg1_3782	B4	1.897102138	Bcer	E	Bcer	FALSE	NA	NA	NA
Bc_ctg1_3783	B4	0.781159704	Bcer	E	Bcer	FALSE	NA	NA	NA
Bc_ctg1_3784	B4	0.223188487	Bcer	E	Bcer	FALSE	NA	NA	NA
Bc_ctg1_3785	B4	8.481162498	Bcer	E	Bcer	FALSE	NA	NA	NA
Bc_ctg1_3786	B4	1.562319407	Bcer	NA	Bcer	FALSE	NA	NA	NA
Bc_ctg1_3787	B4	149.5362861	Bcer	CH	Bcer	FALSE	NA	NA	NA
Bc_ctg1_3788	B4	105.3449658	Bcer	I	Bcer	FALSE	NA	NA	NA
Bc_ctg1_3789	B4	170.2928154	Bcer	M	Bcer	FALSE	NA	NA	NA
Bc_ctg1_379	B4	1.673913651	Bcer	K	Bcer	FALSE	Bc_Ga0417192_01_rn04	Regulation	SMCOG1171
Bc_ctg1_3790	B4	0.781159704	Bcer	S	Bcer	FALSE	NA	NA	NA
Bc_ctg1_3791	B4	103.4478636	Bcer	E	Bcer	FALSE	NA	NA	NA
Bc_ctg1_3792	B4	0.33478273	Bcer	S	Bcer	FALSE	NA	NA	NA
Bc_ctg1_3793	B4	24.88551628	Bcer	EH	Bcer	FALSE	NA	NA	NA
Bc_ctg1_3794	B4	68.63045969	Bcer	P	Bcer	FALSE	NA	NA	NA
Bc_ctg1_3795	B4	42.07102976	Bcer	P	Bcer	FALSE	NA	NA	NA
Bc_ctg1_3796	B4	170.6275981	Bcer	P	Bcer	FALSE	NA	NA	NA
Bc_ctg1_3797	B4	16.29275954	Bcer	S	Bcer	FALSE	NA	NA	NA
Bc_ctg1_3798	B4	13.50290345	Bcer	H	Bcer	FALSE	NA	NA	NA

Bc_ctg1_3799	B4	21.76087746	Bcer	S	Bcer	FALSE	NA	NA	NA
Bc_ctg1_38	B4	0.223188487	Bcer	S	Bcer	FALSE	NA	NA	NA
Bc_ctg1_380	B4	1.450725164	Bcer	E	Bcer	FALSE	Bc_Ga0417192_01_rn04	Other	NA
Bc_ctg1_3800	B4	15.62319407	Bcer	H	Bcer	FALSE	NA	NA	NA
Bc_ctg1_3801	B4	21.53768897	Bcer	S	Bcer	FALSE	NA	NA	NA
Bc_ctg1_3802	B4	0.557971217	Bcer	NA	Bcer	FALSE	NA	NA	NA
Bc_ctg1_3803	B4	278.76242	Bcer	D	Bcer	FALSE	NA	NA	NA
Bc_ctg1_3804	B4	744.5567919	Bcer	C	Bcer	FALSE	NA	NA	NA
Bc_ctg1_3805	B4	3.013044572	Bcer	S	Bcer	FALSE	NA	NA	NA
Bc_ctg1_3806	B4	48.0971189	Bcer	C	Bcer	FALSE	NA	NA	NA
Bc_ctg1_3807	B4	33.03189604	Bcer	NA	Bcer	FALSE	NA	NA	NA
Bc_ctg1_3808	B4	134.0246863	Bcer	KT	Bcer	FALSE	NA	NA	NA
Bc_ctg1_3809	B4	25.33189325	Bcer	S	Bcer	FALSE	NA	NA	NA
Bc_ctg1_381	B4	0	Bcer	S	Bcer	FALSE	Bc_Ga0417192_01_rn04	Other	NA
Bc_ctg1_3810	B4	51.4449462	Bcer	T	Bcer	FALSE	NA	NA	NA
Bc_ctg1_3811	B4	50.21740953	Bcer	KT	Bcer	FALSE	NA	NA	NA
Bc_ctg1_3812	B4	16.85073075	Bcer	NA	Bcer	FALSE	NA	NA	NA
Bc_ctg1_3813	B4	11.0478301	Bcer	E	Bcer	FALSE	NA	NA	NA
Bc_ctg1_3814	B4	36.04494062	Bcer	NA	Bcer	FALSE	NA	NA	NA
Bc_ctg1_3815	B4	20.08696381	Bcer	Q	Bcer	TRUE	NA	NA	NA
Bc_ctg1_3816	B4	13.05652648	Bcer	NA	Bcer	FALSE	NA	NA	NA
Bc_ctg1_3818	B4	9.150727958	Bcer	S	Bcer	FALSE	NA	NA	NA
Bc_ctg1_3819	B4	1.673913651	Bcer	NA	Bcer	FALSE	NA	NA	NA
Bc_ctg1_382	B4	1.004348191	Bcer	E	Bcer	FALSE	Bc_Ga0417192_01_rn04	Other	NA
Bc_ctg1_3820	B4	446.7117563	Bcer	C	Bcer	FALSE	NA	NA	NA
Bc_ctg1_3821	B4	11.71739556	Bcer	T	Bcer	FALSE	NA	NA	NA
Bc_ctg1_3822	B4	1.339130921	Bcer	NA	Bcer	FALSE	NA	NA	NA
Bc_ctg1_3823	B4	177.434847	Bcer	S	Bcer	FALSE	NA	NA	NA
Bc_ctg1_3824	B4	618.0089199	Bcer	P	Bcer	FALSE	NA	NA	NA
Bc_ctg1_3825	B4	303.6479363	Bcer	P	Bcer	FALSE	NA	NA	NA
Bc_ctg1_3826	B4	26.78261841	Bcer	M	Bcer	FALSE	NA	NA	NA
Bc_ctg1_3827	B4	0.111594243	Bcer	NA	Bcer	FALSE	NA	NA	NA
Bc_ctg1_3828	B4	0.111594243	Bcer	NA	Bcer	FALSE	NA	NA	NA
Bc_ctg1_3829	B4	35.15218667	Bcer	T	Bcer	FALSE	NA	NA	NA
Bc_ctg1_383	B4	91.61887382	Bcer	V	Bcer	FALSE	Bc_Ga0417192_01_rn04	Ancillary biosynthesis	SMCOG1053
Bc_ctg1_3830	B4	7.142031577	Bcer	S	Bcer	FALSE	NA	NA	NA
Bc_ctg1_3831	B4	24.10435657	Bcer	S	Bcer	FALSE	NA	NA	NA
Bc_ctg1_3832	B4	735.9640352	Bcer	L	Bcer	FALSE	NA	NA	NA
Bc_ctg1_3833	B4	211.359497	Bcer	T	Bcer	FALSE	NA	NA	NA
Bc_ctg1_3834	B4	309.6740254	Bcer	F	Bcer	FALSE	NA	NA	NA
Bc_ctg1_3835	B4	29.23769177	Bcer	S	Bcer	FALSE	NA	NA	NA
Bc_ctg1_3836	B4	268.3841554	Bcer	S	Bcer	FALSE	NA	NA	NA
Bc_ctg1_3837	B4	2.789856085	Bcer	NA	Bcer	FALSE	NA	NA	NA
Bc_ctg1_3838	B4	7.923191281	Bcer	S	Bcer	FALSE	NA	NA	NA
Bc_ctg1_3839	B4	0.66956546	Bcer	P	Bcer	FALSE	NA	NA	NA
Bc_ctg1_384	B4	1.339130921	Bcer	NA	Bcer	FALSE	NA	NA	NA
Bc_ctg1_3840	B4	0.223188487	Bcer	M	Bcer	FALSE	NA	NA	NA
Bc_ctg1_3841	B4	0.892753947	Bcer	S	Bcer	FALSE	NA	NA	NA
Bc_ctg1_3842	B4	1.004348191	Bcer	S	Bcer	FALSE	NA	NA	NA
Bc_ctg1_3843	B4	133.3551209	Bcer	J	Bcer	FALSE	NA	NA	NA
Bc_ctg1_3844	B4	33.7014615	Bcer	CO	Bcer	FALSE	NA	NA	NA
Bc_ctg1_3845	B4	38.27682548	Bcer	O	Bcer	FALSE	NA	NA	NA
Bc_ctg1_3846	B4	61.60002235	Bcer	O	Bcer	FALSE	NA	NA	NA
Bc_ctg1_3847	B4	155.897158	Bcer	T	Bcer	FALSE	NA	NA	NA
Bc_ctg1_3848	B4	353.3073746	Bcer	T	Bcer	FALSE	NA	NA	NA
Bc_ctg1_3849	B4	6.24927763	Bcer	S	Bcer	FALSE	NA	NA	NA
Bc_ctg1_385	B4	2.789856085	Bcer	G	Bcer	FALSE	NA	NA	NA
Bc_ctg1_3850	B4	2.008696381	Bcer	M	Bcer	FALSE	NA	NA	NA
Bc_ctg1_3851	B4	102.3319212	Bcer	M	Bcer	FALSE	NA	NA	NA
Bc_ctg1_3852	B4	123.3116389	Bcer	U	Bcer	FALSE	NA	NA	NA
Bc_ctg1_3853	B4	97.9797457	Bcer	C	Bcer	FALSE	NA	NA	NA
Bc_ctg1_3854	B4	21.76087746	Bcer	S	Bcer	FALSE	NA	NA	NA

Bc_ctg1_3855	B4	42.40581249	Bcer	L	Bcer	FALSE	NA	NA	NA
Bc_ctg1_3856	B4	43.07537795	Bcer	S	Bcer	FALSE	NA	NA	NA
Bc_ctg1_3857	B4	44.30291463	Bcer	M	Bcer	FALSE	NA	NA	NA
Bc_ctg1_3858	B4	120.8565656	Bcer	S	Bcer	FALSE	NA	NA	NA
Bc_ctg1_3859	B4	180.3362973	Bcer	K	Bcer	FALSE	NA	NA	NA
Bc_ctg1_386	B4	8.927539471	Bcer	K	Bcer	FALSE	NA	NA	NA
Bc_ctg1_3860	B4	151.5449825	Bcer	NOT	Bcer	FALSE	NA	NA	NA
Bc_ctg1_3861	B4	2113.148593	Bcer	E	Bcer	FALSE	NA	NA	NA
Bc_ctg1_3862	B4	0.223188487	Bcer	NA	Bcer	FALSE	NA	NA	NA
Bc_ctg1_3863	B4	3.347827302	Bcer	S	Bcer	FALSE	NA	NA	NA
Bc_ctg1_3864	B4	7.25362582	Bcer	M	Bcer	FALSE	NA	NA	NA
Bc_ctg1_3865	B4	17.18551348	Bcer	O	Bcer	FALSE	NA	NA	NA
Bc_ctg1_3866	B4	156.5667235	Bcer	EJ	Bcer	FALSE	NA	NA	NA
Bc_ctg1_3867	B4	0	Bcer	S	Bcer	FALSE	NA	NA	NA
Bc_ctg1_3868	B4	85.25800195	Bcer	F	Bcer	FALSE	NA	NA	NA
Bc_ctg1_3869	B4	1422.938197	Bcer	J	Bcer	FALSE	NA	NA	NA
Bc_ctg1_387	B4	2.789856085	Bcer	S	Bcer	FALSE	NA	NA	NA
Bc_ctg1_3870	B4	44.30291463	Bcer	C	Bcer	FALSE	NA	NA	NA
Bc_ctg1_3872	B4	0.111594243	Bcer	NA	Bcer	FALSE	NA	NA	NA
Bc_ctg1_3873	B4	0.781159704	Bcer	S	Bcer	FALSE	NA	NA	NA
Bc_ctg1_3875	B4	522.1494648	Bcer	S	Bcer	FALSE	NA	NA	NA
Bc_ctg1_3876	B4	557.1900573	Bcer	I	Bcer	FALSE	NA	NA	NA
Bc_ctg1_3877	B4	0.446376974	Bcer	S	Bcer	FALSE	NA	NA	NA
Bc_ctg1_3878	B4	0.781159704	Bcer	S	Bcer	FALSE	NA	NA	NA
Bc_ctg1_3879	B4	7.030437334	Bcer	NA	Bcer	FALSE	NA	NA	NA
Bc_ctg1_388	B4	50.32900377	Bcer	C	Bcer	FALSE	NA	NA	NA
Bc_ctg1_3880	B4	8.369568254	Bcer	S	Bcer	FALSE	NA	NA	NA
Bc_ctg1_3881	B4	888.8481486	Bcer	L	Bcer	FALSE	NA	NA	NA
Bc_ctg1_3882	B4	203.101523	Bcer	H	Bcer	FALSE	NA	NA	NA
Bc_ctg1_3883	B4	69.30002515	Bcer	H	Bcer	FALSE	NA	NA	NA
Bc_ctg1_3884	B4	74.65654883	Bcer	H	Bcer	FALSE	NA	NA	NA
Bc_ctg1_3885	B4	136.5913539	Bcer	H	Bcer	FALSE	NA	NA	NA
Bc_ctg1_3886	B4	1423.27298	Bcer	F	Bcer	FALSE	NA	NA	NA
Bc_ctg1_3887	B4	96.08264356	Bcer	E	Bcer	FALSE	NA	NA	NA
Bc_ctg1_3888	B4	159.6913623	Bcer	E	Bcer	FALSE	NA	NA	NA
Bc_ctg1_3889	B4	367.0334665	Bcer	E	Bcer	FALSE	NA	NA	NA
Bc_ctg1_389	B4	11.49420707	Bcer	NA	Bcer	FALSE	NA	NA	NA
Bc_ctg1_3890	B4	167.9493363	Bcer	S	Bcer	FALSE	NA	NA	NA
Bc_ctg1_3891	B4	114.0493167	Bcer	S	Bcer	FALSE	Bc_Ga0417192_01_rn27	Other	NA
Bc_ctg1_3892	B4	14.17246891	Bcer	S	Bcer	FALSE	Bc_Ga0417192_01_rn27	Other	NA
Bc_ctg1_3893	B4	30.68841693	Bcer	C	Bcer	FALSE	Bc_Ga0417192_01_rn27	Other	NA
Bc_ctg1_3894	B4	57.91741232	Bcer	C	Bcer	FALSE	Bc_Ga0417192_01_rn27	Other	NA
Bc_ctg1_3895	B4	22.76522565	Bcer	C	Bcer	FALSE	Bc_Ga0417192_01_rn27	Other	NA
Bc_ctg1_3896	B4	19.75218108	Bcer	S	Bcer	FALSE	Bc_Ga0417192_01_rn27	Other	NA
Bc_ctg1_3897	B4	16.73913651	Bcer	S	Bcer	FALSE	Bc_Ga0417192_01_rn27	Other	NA
Bc_ctg1_3898	B4	2.901450328	Bcer	S	Bcer	FALSE	Bc_Ga0417192_01_rn27	Other	NA
Bc_ctg1_3899	B4	296.7290932	Bcer	S	Bcer	FALSE	Bc_Ga0417192_01_rn27	Other	NA
Bc_ctg1_39	B4	2.789856085	Bcer	J	Bcer	FALSE	NA	NA	NA
Bc_ctg1_390	B4	4.686958222	Bcer	NA	Bcer	FALSE	NA	NA	NA
Bc_ctg1_3900	B4	0.781159704	Bcer	L	Bcer	FALSE	Bc_Ga0417192_01_rn27	Other	NA
Bc_ctg1_3901	B4	29.68406874	Bcer	S	Bcer	FALSE	Bc_Ga0417192_01_rn27	Other	NA
Bc_ctg1_3902	B4	3.571015789	Bcer	S	Bcer	FALSE	Bc_Ga0417192_01_rn27	Other	NA
Bc_ctg1_3903	B4	125.2087411	Bcer	E	Bcer	FALSE	Bc_Ga0417192_01_rn27	Other	NA
Bc_ctg1_3904	B4	67.17973452	Bcer	G	Bcer	FALSE	Bc_Ga0417192_01_rn27	Other	NA
Bc_ctg1_3905	B4	92.40003353	Bcer	S	Bcer	FALSE	Bc_Ga0417192_01_rn27	Core biosynthesis	None
Bc_ctg1_3906	B4	153.3304904	Bcer	M	Bcer	FALSE	Bc_Ga0417192_01_rn27	Core biosynthesis	SMCOG1045
Bc_ctg1_3907	B4	208.9044236	Bcer	J	Bcer	FALSE	Bc_Ga0417192_01_rn27	Other	NA
Bc_ctg1_3908	B4	159.579768	Bcer	K	Bcer	FALSE	Bc_Ga0417192_01_rn27	Other	NA
Bc_ctg1_3909	B4	0.33478273	Bcer	C	Bcer	FALSE	Bc_Ga0417192_01_rn27	Other	NA
Bc_ctg1_391	B4	4.686958222	Bcer	S	Bcer	FALSE	NA	NA	NA
Bc_ctg1_3910	B4	76.8884337	Bcer	H	Bcer	FALSE	Bc_Ga0417192_01_rn27	Other	NA
Bc_ctg1_3911	B4	152.214548	Bcer	H	Bcer	FALSE	Bc_Ga0417192_01_rn27	Other	NA

Bc_ctg1_3912	B4	19.64058684	Bcer	H	Bcer	FALSE	Bc_Ga0417192_01_rn27	Other	NA
Bc_ctg1_3913	B4	393.2581137	Bcer	L	Bcer	FALSE	Bc_Ga0417192_01_rn27	Other	SMCOG1194
Bc_ctg1_3914	B4	38.053637	Bcer	S	Bcer	FALSE	Bc_Ga0417192_01_rn27	Other	NA
Bc_ctg1_3915	B4	112.1522146	Bcer	S	Bcer	FALSE	Bc_Ga0417192_01_rn27	Other	NA
Bc_ctg1_3916	B4	1074.987347	Bcer	E	Bcer	FALSE	Bc_Ga0417192_01_rn27	Core biosynthesis	SMCOG1019
Bc_ctg1_3917	B4	53.78842531	Bcer	L	Bcer	FALSE	Bc_Ga0417192_01_rn27	Other	NA
Bc_ctg1_3918	B4	56.35509291	Bcer	L	Bcer	FALSE	Bc_Ga0417192_01_rn27	Other	NA
Bc_ctg1_3919	B4	26.33624144	Bcer	NA	Bcer	FALSE	Bc_Ga0417192_01_rn27	Other	NA
Bc_ctg1_392	B4	30.3536342	Bcer	NA	Bcer	FALSE	NA	NA	NA
Bc_ctg1_3920	B4	405.0871035	Bcer	M	Bcer	FALSE	Bc_Ga0417192_01_rn27	Other	NA
Bc_ctg1_3921	B4	24.4391393	Bcer	L	Bcer	FALSE	Bc_Ga0417192_01_rn27	Other	NA
Bc_ctg1_3922	B4	4.352175492	Bcer	S	Bcer	FALSE	Bc_Ga0417192_01_rn27	Other	NA
Bc_ctg1_3924	B4	2.008696381	Bcer	NA	Bcer	FALSE	Bc_Ga0417192_01_rn27	Other	NA
Bc_ctg1_3925	B4	4.575363979	Bcer	S	Bcer	FALSE	Bc_Ga0417192_01_rn27	Other	NA
Bc_ctg1_3926	B4	7.142031577	Bcer	S	Bcer	FALSE	Bc_Ga0417192_01_rn27	Other	NA
Bc_ctg1_3927	B4	1.115942434	Bcer	NA	Bcer	FALSE	Bc_Ga0417192_01_rn27	Other	NA
Bc_ctg1_3928	B4	0.446376974	Bcer	S	Bcer	FALSE	Bc_Ga0417192_01_rn27	Other	NA
Bc_ctg1_3929	B4	7.030437334	Bcer	S	Bcer	FALSE	Bc_Ga0417192_01_rn27	Other	NA
Bc_ctg1_393	B4	75.88408551	Bcer	S	Bcer	FALSE	NA	NA	NA
Bc_ctg1_3930	B4	304.6522845	Bcer	D	Bcer	FALSE	NA	NA	NA
Bc_ctg1_3931	B4	127.5522202	Bcer	L	Bcer	FALSE	NA	NA	NA
Bc_ctg1_3932	B4	10.04348191	Bcer	S	Bcer	FALSE	NA	NA	NA
Bc_ctg1_3933	B4	103.7826464	Bcer	KL	Bcer	FALSE	NA	NA	NA
Bc_ctg1_3934	B4	557.7480285	Bcer	E	Bcer	FALSE	NA	NA	NA
Bc_ctg1_3935	B4	0.557971217	Bcer	L	Bcer	FALSE	NA	NA	NA
Bc_ctg1_3937	B4	1.115942434	Bcer	J	Bcer	FALSE	NA	NA	NA
Bc_ctg1_3938	B4	12.49855526	Bcer	E	Bcer	FALSE	NA	NA	NA
Bc_ctg1_3939	B4	117.5087383	Bcer	F	Bcer	FALSE	NA	NA	NA
Bc_ctg1_394	B4	44.86088584	Bcer	NA	Bcer	FALSE	NA	NA	NA
Bc_ctg1_3940	B4	645.9074807	Bcer	F	Bcer	FALSE	NA	NA	NA
Bc_ctg1_3941	B4	0	Bcer	NA	Bcer	FALSE	NA	NA	NA
Bc_ctg1_3943	B4	4.910146709	Bcer	P	Bcer	FALSE	NA	NA	NA
Bc_ctg1_3945	B4	248.1855973	Bcer	S	Bcer	FALSE	NA	NA	NA
Bc_ctg1_3946	B4	0	Bcer	S	Bcer	FALSE	NA	NA	NA
Bc_ctg1_3947	B4	41.28987005	Bcer	K	Bcer	FALSE	NA	NA	NA
Bc_ctg1_3948	B4	37.83044851	Bcer	S	Bcer	FALSE	Bc_Ga0417192_01_rn28	Other	NA
Bc_ctg1_3949	B4	199.0841302	Bcer	P	Bcer	FALSE	Bc_Ga0417192_01_rn28	Other	NA
Bc_ctg1_395	B4	3.013044572	Bcer	CH	Bcer	FALSE	NA	NA	NA
Bc_ctg1_3950	B4	3.905798519	Bcer	NA	Bcer	FALSE	Bc_Ga0417192_01_rn28	Other	NA
Bc_ctg1_3951	B4	9.373916445	Bcer	NA	Bcer	FALSE	Bc_Ga0417192_01_rn28	Other	NA
Bc_ctg1_3952	B4	30.68841693	Bcer	E	Bcer	FALSE	Bc_Ga0417192_01_rn28	Other	NA
Bc_ctg1_3953	B4	4.910146709	Bcer	S	Bcer	FALSE	Bc_Ga0417192_01_rn28	Other	NA
Bc_ctg1_3954	B4	309.2276484	Bcer	P	Bcer	FALSE	Bc_Ga0417192_01_rn28	Other	NA
Bc_ctg1_3955	B4	106.4609082	Bcer	L	Bcer	FALSE	Bc_Ga0417192_01_rn28	Other	NA
Bc_ctg1_3956	B4	0	Bcer	NA	Bcer	FALSE	Bc_Ga0417192_01_rn28	Other	NA
Bc_ctg1_3957	B4	1.227536677	Bcer	I	Bcer	FALSE	Bc_Ga0417192_01_rn28	Ancillary biosynthesis	SMCOG1088
Bc_ctg1_3958	B4	1.115942434	Bcer	M	Bcer	FALSE	Bc_Ga0417192_01_rn28	Ancillary biosynthesis	SMCOG1216
Bc_ctg1_3959	B4	1.004348191	Bcer	D	Bcer	FALSE	Bc_Ga0417192_01_rn28	Other	NA
Bc_ctg1_396	B4	1.115942434	Bcer	K	Bcer	FALSE	NA	NA	NA
Bc_ctg1_3960	B4	0.33478273	Bcer	M	Bcer	FALSE	Bc_Ga0417192_01_rn28	Core biosynthesis	SMCOG1146
Bc_ctg1_3961	B4	3.236233058	Bcer	S	Bcer	FALSE	Bc_Ga0417192_01_rn28	Core biosynthesis	None
Bc_ctg1_3962	B4	2.455073355	Bcer	M	Bcer	FALSE	Bc_Ga0417192_01_rn28	Core biosynthesis	None
Bc_ctg1_3963	B4	2.566667598	Bcer	M	Bcer	FALSE	Bc_Ga0417192_01_rn28	Core biosynthesis	None
Bc_ctg1_3964	B4	0.223188487	Bcer	M	Bcer	FALSE	Bc_Ga0417192_01_rn28	Core biosynthesis	SMCOG1045
Bc_ctg1_3965	B4	0.223188487	Bcer	M	Bcer	FALSE	Bc_Ga0417192_01_rn28	Other	NA
Bc_ctg1_3966	B4	7.476814307	Bcer	M	Bcer	FALSE	NA	NA	NA
Bc_ctg1_3967	B4	2.901450328	Bcer	K	Bcer	FALSE	NA	NA	NA
Bc_ctg1_3968	B4	20.3101523	Bcer	Q	Bcer	TRUE	NA	NA	NA
Bc_ctg1_3969	B4	6.137683386	Bcer	NA	Bcer	FALSE	NA	NA	NA
Bc_ctg1_397	B4	6.360871873	Bcer	C	Bcer	FALSE	NA	NA	NA
Bc_ctg1_3970	B4	18.18986167	Bcer	EG	Bcer	FALSE	NA	NA	NA
Bc_ctg1_3971	B4	7.700002794	Bcer	L	Bcer	FALSE	NA	NA	NA

Bc_ctg1_3972	B4	55.35074472	Bcer	G	Bcer	FALSE	NA	NA	NA
Bc_ctg1_3973	B4	2.789856085	Bcer	S	Bcer	FALSE	NA	NA	NA
Bc_ctg1_3974	B4	3.682610032	Bcer	L	Bcer	FALSE	NA	NA	NA
Bc_ctg1_3975	B4	1.339130921	Bcer	S	Bcer	FALSE	NA	NA	NA
Bc_ctg1_3976	B4	0.66956546	Bcer	S	Bcer	FALSE	NA	NA	NA
Bc_ctg1_3977	B4	9.708699175	Bcer	K	Bcer	FALSE	NA	NA	NA
Bc_ctg1_3978	B4	0.781159704	Bcer	S	Bcer	FALSE	NA	NA	NA
Bc_ctg1_3979	B4	18.18986167	Bcer	E	Bcer	FALSE	NA	NA	NA
Bc_ctg1_398	B4	135.0290345	Bcer	S	Bcer	FALSE	NA	NA	NA
Bc_ctg1_3980	B4	2.678261841	Bcer	NA	Bcer	FALSE	NA	NA	NA
Bc_ctg1_3981	B4	3.794204275	Bcer	I	Bcer	FALSE	NA	NA	NA
Bc_ctg1_3982	B4	0	Bcer	S	Bcer	FALSE	NA	NA	NA
Bc_ctg1_3983	B4	2.789856085	Bcer	S	Bcer	FALSE	NA	NA	NA
Bc_ctg1_3984	B4	65.8406036	Bcer	E	Bcer	FALSE	NA	NA	NA
Bc_ctg1_3985	B4	0	Bcer	S	Bcer	FALSE	NA	NA	NA
Bc_ctg1_3986	B4	45.19566857	Bcer	P	Bcer	FALSE	NA	NA	NA
Bc_ctg1_3987	B4	10.04348191	Bcer	KT	Bcer	FALSE	NA	NA	NA
Bc_ctg1_3988	B4	48.32030739	Bcer	V	Bcer	FALSE	NA	NA	NA
Bc_ctg1_3989	B4	0.33478273	Bcer	S	Bcer	FALSE	NA	NA	NA
Bc_ctg1_399	B4	9.931887662	Bcer	S	Bcer	FALSE	NA	NA	NA
Bc_ctg1_3990	B4	12.27536677	Bcer	S	Bcer	FALSE	NA	NA	NA
Bc_ctg1_3991	B4	1.115942434	Bcer	S	Bcer	FALSE	NA	NA	NA
Bc_ctg1_3992	B4	28.90290904	Bcer	C	Bcer	FALSE	NA	NA	NA
Bc_ctg1_3993	B4	4.910146709	Bcer	K	Bcer	FALSE	NA	NA	NA
Bc_ctg1_3994	B4	3.459421545	Bcer	NA	Bcer	FALSE	NA	NA	NA
Bc_ctg1_3995	B4	4.352175492	Bcer	S	Bcer	FALSE	NA	NA	NA
Bc_ctg1_3996	B4	3.794204275	Bcer	G	Bcer	FALSE	NA	NA	NA
Bc_ctg1_3997	B4	1.785507894	Bcer	K	Bcer	FALSE	NA	NA	NA
Bc_ctg1_3998	B4	1.673913651	Bcer	EGP	Bcer	FALSE	NA	NA	NA
Bc_ctg1_3999	B4	0.223188487	Bcer	E	Bcer	FALSE	NA	NA	NA
Bc_ctg1_4	B4	0.446376974	Bcer	F	Bcer	FALSE	NA	NA	NA
Bc_ctg1_40	B4	0	Bcer	L	Bcer	FALSE	NA	NA	NA
Bc_ctg1_400	B4	6.137683386	Bcer	NA	Bcer	FALSE	NA	NA	NA
Bc_ctg1_4001	B4	0.223188487	Bcer	NA	Bcer	FALSE	NA	NA	NA
Bc_ctg1_4002	B4	1.897102138	Bcer	S	Bcer	FALSE	NA	NA	NA
Bc_ctg1_4003	B4	40.84349308	Bcer	S	Bcer	FALSE	NA	NA	NA
Bc_ctg1_4004	B4	37.71885427	Bcer	N	Bcer	FALSE	NA	NA	NA
Bc_ctg1_4005	B4	51.22175772	Bcer	N	Bcer	FALSE	NA	NA	NA
Bc_ctg1_4006	B4	59.144949	Bcer	T	Bcer	FALSE	NA	NA	NA
Bc_ctg1_4007	B4	519.3596087	Bcer	NT	Bcer	FALSE	NA	NA	NA
Bc_ctg1_4008	B4	115.8348246	Bcer	N	Bcer	FALSE	NA	NA	NA
Bc_ctg1_4009	B4	36.2681291	Bcer	NA	Bcer	FALSE	NA	NA	NA
Bc_ctg1_401	B4	7.811597037	Bcer	K	Bcer	FALSE	NA	NA	NA
Bc_ctg1_4010	B4	62.38118206	Bcer	NA	Bcer	FALSE	NA	NA	NA
Bc_ctg1_4011	B4	35.71015789	Bcer	NT	Bcer	FALSE	NA	NA	NA
Bc_ctg1_4012	B4	31.02319966	Bcer	NA	Bcer	FALSE	NA	NA	NA
Bc_ctg1_4013	B4	41.4014643	Bcer	S	Bcer	FALSE	NA	NA	NA
Bc_ctg1_4014	B4	36.49131759	Bcer	NA	Bcer	FALSE	NA	NA	NA
Bc_ctg1_4015	B4	342.2595445	Bcer	N	Bcer	FALSE	NA	NA	NA
Bc_ctg1_4016	B4	175.9841218	Bcer	N	Bcer	FALSE	NA	NA	NA
Bc_ctg1_4017	B4	385.3349224	Bcer	N	Bcer	FALSE	NA	NA	NA
Bc_ctg1_4018	B4	135.252223	Bcer	N	Bcer	FALSE	NA	NA	NA
Bc_ctg1_4019	B4	90.39133715	Bcer	NA	Bcer	FALSE	NA	NA	NA
Bc_ctg1_402	B4	0.111594243	Bcer	S	Bcer	FALSE	NA	NA	NA
Bc_ctg1_4020	B4	60.7072684	Bcer	N	Bcer	FALSE	NA	NA	NA
Bc_ctg1_4021	B4	57.3594411	Bcer	N	Bcer	FALSE	NA	NA	NA
Bc_ctg1_4022	B4	29.12609753	Bcer	N	Bcer	FALSE	NA	NA	NA
Bc_ctg1_4023	B4	268.0493726	Bcer	N	Bcer	FALSE	NA	NA	NA
Bc_ctg1_4024	B4	211.2479027	Bcer	N	Bcer	FALSE	NA	NA	NA
Bc_ctg1_4025	B4	45.08407433	Bcer	N	Bcer	FALSE	NA	NA	NA
Bc_ctg1_4026	B4	160.2493335	Bcer	NU	Bcer	FALSE	NA	NA	NA
Bc_ctg1_4027	B4	77.33481067	Bcer	NA	Bcer	FALSE	NA	NA	NA

Bc_ctg1_4028	B4	160.8073047	Bcer	N	Bcer	FALSE	NA	NA	NA
Bc_ctg1_4029	B4	151.0986056	Bcer	N	Bcer	FALSE	NA	NA	NA
Bc_ctg1_403	B4	1.785507894	Bcer	S	Bcer	FALSE	NA	NA	NA
Bc_ctg1_4030	B4	414.5726142	Bcer	N	Bcer	FALSE	NA	NA	NA
Bc_ctg1_4031	B4	124.5391756	Bcer	S	Bcer	FALSE	NA	NA	NA
Bc_ctg1_4032	B4	194.397172	Bcer	NA	Bcer	FALSE	NA	NA	NA
Bc_ctg1_4033	B4	514.2262735	Bcer	T	Bcer	FALSE	NA	NA	NA
Bc_ctg1_4034	B4	33.25508453	Bcer	K	Bcer	FALSE	NA	NA	NA
Bc_ctg1_4035	B4	50.55219226	Bcer	N	Bcer	FALSE	NA	NA	NA
Bc_ctg1_4036	B4	13053.29024	Bcer	N	Bcer	FALSE	NA	NA	NA
Bc_ctg1_4037	B4	75.1029258	Bcer	M	Bcer	FALSE	NA	NA	NA
Bc_ctg1_4038	B4	3.682610032	Bcer	N	Bcer	FALSE	NA	NA	NA
Bc_ctg1_4039	B4	125.6551181	Bcer	N	Bcer	FALSE	NA	NA	NA
Bc_ctg1_404	B4	2.566667598	Bcer	C	Bcer	FALSE	NA	NA	NA
Bc_ctg1_4040	B4	58.81016627	Bcer	N	Bcer	FALSE	NA	NA	NA
Bc_ctg1_4041	B4	20.64493503	Bcer	N	Bcer	FALSE	NA	NA	NA
Bc_ctg1_4042	B4	81.79858041	Bcer	N	Bcer	FALSE	NA	NA	NA
Bc_ctg1_4043	B4	14.61884588	Bcer	N	Bcer	FALSE	NA	NA	NA
Bc_ctg1_4044	B4	73.09422942	Bcer	N	Bcer	FALSE	NA	NA	NA
Bc_ctg1_4045	B4	120.0754059	Bcer	N	Bcer	FALSE	NA	NA	NA
Bc_ctg1_4046	B4	182.3449937	Bcer	N	Bcer	FALSE	NA	NA	NA
Bc_ctg1_4047	B4	180.0015146	Bcer	N	Bcer	FALSE	NA	NA	NA
Bc_ctg1_4048	B4	141.0551236	Bcer	N	Bcer	FALSE	NA	NA	NA
Bc_ctg1_4049	B4	26.2246472	Bcer	C	Bcer	FALSE	NA	NA	NA
Bc_ctg1_405	B4	7.476814307	Bcer	S	Bcer	FALSE	NA	NA	NA
Bc_ctg1_4050	B4	2.566667598	Bcer	S	Bcer	FALSE	NA	NA	NA
Bc_ctg1_4051	B4	11.8289898	Bcer	K	Bcer	FALSE	NA	NA	NA
Bc_ctg1_4052	B4	15.62319407	Bcer	E	Bcer	FALSE	NA	NA	NA
Bc_ctg1_4053	B4	6.026089143	Bcer	S	Bcer	FALSE	NA	NA	NA
Bc_ctg1_4054	B4	0.33478273	Bcer	NA	Bcer	FALSE	NA	NA	NA
Bc_ctg1_4055	B4	1.004348191	Bcer	NA	Bcer	FALSE	NA	NA	NA
Bc_ctg1_4056	B4	5.244929439	Bcer	Q	Bcer	TRUE	NA	NA	NA
Bc_ctg1_4057	B4	1.115942434	Bcer	S	Bcer	FALSE	NA	NA	NA
Bc_ctg1_4058	B4	0.892753947	Bcer	S	Bcer	FALSE	NA	NA	NA
Bc_ctg1_4059	B4	12.6101495	Bcer	K	Bcer	FALSE	NA	NA	NA
Bc_ctg1_406	B4	17.40870197	Bcer	J	Bcer	FALSE	NA	NA	NA
Bc_ctg1_4060	B4	3.013044572	Bcer	EGP	Bcer	FALSE	NA	NA	NA
Bc_ctg1_4061	B4	0.66956546	Bcer	S	Bcer	FALSE	NA	NA	NA
Bc_ctg1_4062	B4	1.785507894	Bcer	K	Bcer	FALSE	NA	NA	NA
Bc_ctg1_4063	B4	0	Bcer	P	Bcer	FALSE	NA	NA	NA
Bc_ctg1_4064	B4	0.781159704	Bcer	P	Bcer	FALSE	NA	NA	NA
Bc_ctg1_4065	B4	0.557971217	Bcer	P	Bcer	FALSE	NA	NA	NA
Bc_ctg1_4066	B4	1.115942434	Bcer	J	Bcer	FALSE	NA	NA	NA
Bc_ctg1_4067	B4	0.111594243	Bcer	NA	Bcer	FALSE	NA	NA	NA
Bc_ctg1_4068	B4	240.4855945	Bcer	V	Bcer	FALSE	NA	NA	NA
Bc_ctg1_4069	B4	59.47973173	Bcer	S	Bcer	FALSE	NA	NA	NA
Bc_ctg1_407	B4	0	Bcer	NA	Bcer	FALSE	NA	NA	NA
Bc_ctg1_4070	B4	16.962325	Bcer	NA	Bcer	FALSE	NA	NA	NA
Bc_ctg1_4071	B4	1.450725164	Bcer	V	Bcer	FALSE	NA	NA	NA
Bc_ctg1_4072	B4	0.223188487	Bcer	G	Bcer	FALSE	NA	NA	NA
Bc_ctg1_4073	B4	1303.197574	Bcer	NA	Bcer	FALSE	NA	NA	NA
Bc_ctg1_4074	B4	2.343479111	Bcer	S	Bcer	FALSE	NA	NA	NA
Bc_ctg1_4075	B4	1.004348191	Bcer	NA	Bcer	FALSE	NA	NA	NA
Bc_ctg1_4076	B4	1.450725164	Bcer	K	Bcer	FALSE	NA	NA	NA
Bc_ctg1_4077	B4	11.38261283	Bcer	T	Bcer	FALSE	NA	NA	NA
Bc_ctg1_4078	B4	50.32900377	Bcer	E	Bcer	FALSE	NA	NA	NA
Bc_ctg1_4079	B4	0.33478273	Bcer	K	Bcer	FALSE	NA	NA	NA
Bc_ctg1_408	B4	0.223188487	Bcer	I	Bcer	FALSE	NA	NA	NA
Bc_ctg1_4080	B4	1.115942434	Bcer	S	Bcer	FALSE	NA	NA	NA
Bc_ctg1_4081	B4	0.223188487	Bcer	S	Bcer	FALSE	NA	NA	NA
Bc_ctg1_4082	B4	68.96524242	Bcer	P	Bcer	FALSE	NA	NA	NA
Bc_ctg1_4083	B4	1.562319407	Bcer	S	Bcer	FALSE	NA	NA	NA

Bc_ctg1_4084	B4	3.571015789	Bcer	K	Bcer	FALSE	NA	NA	NA
Bc_ctg1_4085	B4	7.142031577	Bcer	EG	Bcer	FALSE	NA	NA	NA
Bc_ctg1_4086	B4	0.33478273	Bcer	NA	Bcer	FALSE	NA	NA	NA
Bc_ctg1_4087	B4	0.781159704	Bcer	S	Bcer	FALSE	NA	NA	NA
Bc_ctg1_4088	B4	0.111594243	Bcer	S	Bcer	FALSE	NA	NA	NA
Bc_ctg1_4089	B4	0.33478273	Bcer	S	Bcer	FALSE	NA	NA	NA
Bc_ctg1_409	B4	0.66956546	Bcer	NA	Bcer	FALSE	NA	NA	NA
Bc_ctg1_4090	B4	0.223188487	Bcer	M	Bcer	FALSE	NA	NA	NA
Bc_ctg1_4091	B4	59.47973173	Bcer	C	Bcer	FALSE	NA	NA	NA
Bc_ctg1_4092	B4	3.459421545	Bcer	K	Bcer	FALSE	NA	NA	NA
Bc_ctg1_4093	B4	0.66956546	Bcer	IQ	Bcer	TRUE	NA	NA	NA
Bc_ctg1_4094	B4	245.6189297	Bcer	C	Bcer	FALSE	NA	NA	NA
Bc_ctg1_4095	B4	15.51159983	Bcer	S	Bcer	FALSE	NA	NA	NA
Bc_ctg1_4096	B4	9.485510688	Bcer	K	Bcer	FALSE	NA	NA	NA
Bc_ctg1_4097	B4	28.12174933	Bcer	V	Bcer	FALSE	NA	NA	NA
Bc_ctg1_4098	B4	13.39130921	Bcer	E	Bcer	FALSE	NA	NA	NA
Bc_ctg1_4099	B4	2.343479111	Bcer	NA	Bcer	FALSE	NA	NA	NA
Bc_ctg1_41	B4	0.446376974	Bcer	L	Bcer	FALSE	NA	NA	NA
Bc_ctg1_410	B4	6.360871873	Bcer	P	Bcer	FALSE	NA	NA	NA
Bc_ctg1_4100	B4	8.927539471	Bcer	K	Bcer	FALSE	NA	NA	NA
Bc_ctg1_4101	B4	8.815945228	Bcer	O	Bcer	FALSE	NA	NA	NA
Bc_ctg1_4102	B4	1.785507894	Bcer	O	Bcer	FALSE	NA	NA	NA
Bc_ctg1_4103	B4	1.004348191	Bcer	CO	Bcer	FALSE	NA	NA	NA
Bc_ctg1_4104	B4	3.459421545	Bcer	M	Bcer	FALSE	NA	NA	NA
Bc_ctg1_4105	B4	0.33478273	Bcer	NA	Bcer	FALSE	NA	NA	NA
Bc_ctg1_4106	B4	42.85218946	Bcer	K	Bcer	FALSE	NA	NA	NA
Bc_ctg1_4107	B4	7.25362582	Bcer	K	Bcer	FALSE	NA	NA	NA
Bc_ctg1_4108	B4	13.94928042	Bcer	E	Bcer	FALSE	NA	NA	NA
Bc_ctg1_4109	B4	48.43190163	Bcer	S	Bcer	FALSE	NA	NA	NA
Bc_ctg1_411	B4	4.128987005	Bcer	S	Bcer	FALSE	NA	NA	NA
Bc_ctg1_4110	B4	8.146379768	Bcer	NA	Bcer	FALSE	NA	NA	NA
Bc_ctg1_4111	B4	5.802900656	Bcer	NA	Bcer	FALSE	NA	NA	NA
Bc_ctg1_4112	B4	2.008696381	Bcer	NA	Bcer	FALSE	NA	NA	NA
Bc_ctg1_4113	B4	1.450725164	Bcer	K	Bcer	FALSE	NA	NA	NA
Bc_ctg1_4114	B4	2.120290624	Bcer	K	Bcer	FALSE	NA	NA	NA
Bc_ctg1_4115	B4	5.133335196	Bcer	T	Bcer	FALSE	NA	NA	NA
Bc_ctg1_4116	B4	31.13479391	Bcer	V	Bcer	FALSE	NA	NA	NA
Bc_ctg1_4117	B4	28.23334358	Bcer	V	Bcer	FALSE	NA	NA	NA
Bc_ctg1_4118	B4	57.91741232	Bcer	V	Bcer	FALSE	NA	NA	NA
Bc_ctg1_4119	B4	0.111594243	Bcer	S	Bcer	FALSE	NA	NA	NA
Bc_ctg1_412	B4	0.223188487	Bcer	NA	Bcer	FALSE	NA	NA	NA
Bc_ctg1_4120	B4	1.897102138	Bcer	I	Bcer	FALSE	NA	NA	NA
Bc_ctg1_4121	B4	26.55942993	Bcer	F	Bcer	FALSE	NA	NA	NA
Bc_ctg1_4122	B4	279.7667682	Bcer	C	Bcer	FALSE	NA	NA	NA
Bc_ctg1_4123	B4	397.7218834	Bcer	E	Bcer	FALSE	NA	NA	NA
Bc_ctg1_4124	B4	423.1653709	Bcer	C	Bcer	FALSE	NA	NA	NA
Bc_ctg1_4125	B4	14.50725164	Bcer	T	Bcer	FALSE	NA	NA	NA
Bc_ctg1_4126	B4	10.37826464	Bcer	T	Bcer	FALSE	NA	NA	NA
Bc_ctg1_4127	B4	67.29132876	Bcer	L	Bcer	FALSE	NA	NA	NA
Bc_ctg1_4128	B4	0	Bcer	NA	Bcer	FALSE	NA	NA	NA
Bc_ctg1_413	B4	0.557971217	Bcer	U	Bcer	FALSE	NA	NA	NA
Bc_ctg1_4130	B4	76.66524521	Bcer	F	Bcer	FALSE	NA	NA	NA
Bc_ctg1_4131	B4	0.892753947	Bcer	K	Bcer	FALSE	NA	NA	NA
Bc_ctg1_4132	B4	11.0478301	Bcer	E	Bcer	FALSE	NA	NA	NA
Bc_ctg1_4133	B4	19.30580411	Bcer	E	Bcer	FALSE	NA	NA	NA
Bc_ctg1_4134	B4	8.592756741	Bcer	S	Bcer	FALSE	NA	NA	NA
Bc_ctg1_4135	B4	2.901450328	Bcer	S	Bcer	FALSE	Bc_Ga0417192_01_rn29	Other	NA
Bc_ctg1_4136	B4	12.05217829	Bcer	S	Bcer	FALSE	Bc_Ga0417192_01_rn29	Other	NA
Bc_ctg1_4137	B4	0.223188487	Bcer	NA	Bcer	FALSE	Bc_Ga0417192_01_rn29	Other	NA
Bc_ctg1_4138	B4	28.34493782	Bcer	S	Bcer	FALSE	Bc_Ga0417192_01_rn29	Other	NA
Bc_ctg1_4139	B4	21.42609473	Bcer	K	Bcer	FALSE	Bc_Ga0417192_01_rn29	Regulation	SMCOG1278
Bc_ctg1_414	B4	21.64928322	Bcer	S	Bcer	FALSE	NA	NA	NA

Bc_ctg1_4140	B4	116.0580131	Bcer	EGP	Bcer	FALSE	Bc_Ga0417192_01_rn29	Transport	SMCOG1005
Bc_ctg1_4141	B4	6.695654603	Bcer	S	Bcer	FALSE	Bc_Ga0417192_01_rn29	Other	NA
Bc_ctg1_4142	B4	14.17246891	Bcer	EGP	Bcer	FALSE	Bc_Ga0417192_01_rn29	Transport	SMCOG1202
Bc_ctg1_4143	B4	5.356523683	Bcer	I	Bcer	FALSE	Bc_Ga0417192_01_rn29	Core biosynthesis	SMCOG1084
Bc_ctg1_4144	B4	62.71596479	Bcer	S	Bcer	FALSE	Bc_Ga0417192_01_rn29	Other	NA
Bc_ctg1_4145	B4	15.28841134	Bcer	P	Bcer	FALSE	Bc_Ga0417192_01_rn29	Transport	SMCOG1106
Bc_ctg1_4146	B4	63.05074752	Bcer	E	Bcer	FALSE	Bc_Ga0417192_01_rn29	Ancillary biosynthesis	SMCOG1081
Bc_ctg1_4147	B4	8.704350984	Bcer	S	Bcer	FALSE	Bc_Ga0417192_01_rn29	Other	NA
Bc_ctg1_4148	B4	17.52029621	Bcer	S	Bcer	FALSE	Bc_Ga0417192_01_rn29	Other	NA
Bc_ctg1_4149	B4	16.29275954	Bcer	NA	Bcer	FALSE	Bc_Ga0417192_01_rn29	Other	NA
Bc_ctg1_415	B4	28.34493782	Bcer	S	Bcer	FALSE	NA	NA	NA
Bc_ctg1_4150	B4	3.794204275	Bcer	S	Bcer	FALSE	Bc_Ga0417192_01_rn29	Other	NA
Bc_ctg1_4151	B4	6.026089143	Bcer	G	Bcer	FALSE	Bc_Ga0417192_01_rn29	Ancillary biosynthesis	SMCOG1235
Bc_ctg1_4152	B4	4.910146709	Bcer	S	Bcer	FALSE	Bc_Ga0417192_01_rn29	Other	NA
Bc_ctg1_4153	B4	2.120290624	Bcer	S	Bcer	FALSE	NA	NA	NA
Bc_ctg1_4154	B4	35.04059242	Bcer	S	Bcer	FALSE	NA	NA	NA
Bc_ctg1_4155	B4	2.901450328	Bcer	DZ	Bcer	FALSE	NA	NA	NA
Bc_ctg1_4156	B4	0	Bcer	NA	Bcer	FALSE	NA	NA	NA
Bc_ctg1_4158	B4	23.21160263	Bcer	O	Bcer	FALSE	NA	NA	NA
Bc_ctg1_4159	B4	0.111594243	Bcer	IQ	Bcer	TRUE	NA	NA	NA
Bc_ctg1_416	B4	1.897102138	Bcer	P	Bcer	FALSE	Bc_Ga0417192_01_rn05	Other	NA
Bc_ctg1_4160	B4	0.33478273	Bcer	E	Bcer	FALSE	NA	NA	NA
Bc_ctg1_4161	B4	1.562319407	Bcer	E	Bcer	FALSE	NA	NA	NA
Bc_ctg1_4162	B4	0.111594243	Bcer	E	Bcer	FALSE	NA	NA	NA
Bc_ctg1_4163	B4	0.111594243	Bcer	EH	Bcer	FALSE	NA	NA	NA
Bc_ctg1_4164	B4	5.57971217	Bcer	E	Bcer	FALSE	NA	NA	NA
Bc_ctg1_4165	B4	6.472466117	Bcer	E	Bcer	FALSE	NA	NA	NA
Bc_ctg1_4166	B4	0.111594243	Bcer	NA	Bcer	FALSE	NA	NA	NA
Bc_ctg1_4167	B4	1.450725164	Bcer	M	Bcer	FALSE	NA	NA	NA
Bc_ctg1_4168	B4	1.227536677	Bcer	S	Bcer	FALSE	NA	NA	NA
Bc_ctg1_4169	B4	0.111594243	Bcer	K	Bcer	FALSE	NA	NA	NA
Bc_ctg1_417	B4	0.781159704	Bcer	E	Bcer	FALSE	Bc_Ga0417192_01_rn05	Other	NA
Bc_ctg1_4170	B4	14.84203437	Bcer	EGP	Bcer	FALSE	NA	NA	NA
Bc_ctg1_4171	B4	8.592756741	Bcer	S	Bcer	FALSE	NA	NA	NA
Bc_ctg1_4172	B4	46.98117647	Bcer	I	Bcer	FALSE	NA	NA	NA
Bc_ctg1_4173	B4	45.08407433	Bcer	K	Bcer	FALSE	NA	NA	NA
Bc_ctg1_4174	B4	391.5842001	Bcer	C	Bcer	FALSE	NA	NA	NA
Bc_ctg1_4175	B4	1.785507894	Bcer	K	Bcer	FALSE	NA	NA	NA
Bc_ctg1_4176	B4	0.111594243	Bcer	S	Bcer	FALSE	NA	NA	NA
Bc_ctg1_4177	B4	108.9159815	Bcer	F	Bcer	FALSE	NA	NA	NA
Bc_ctg1_4178	B4	1.673913651	Bcer	EGP	Bcer	FALSE	NA	NA	NA
Bc_ctg1_4179	B4	9.931887662	Bcer	K	Bcer	FALSE	NA	NA	NA
Bc_ctg1_418	B4	2.343479111	Bcer	S	Bcer	FALSE	Bc_Ga0417192_01_rn05	Other	NA
Bc_ctg1_4180	B4	66.06379209	Bcer	E	Bcer	FALSE	NA	NA	NA
Bc_ctg1_4181	B4	4.128987005	Bcer	J	Bcer	FALSE	NA	NA	NA
Bc_ctg1_4182	B4	1.785507894	Bcer	T	Bcer	FALSE	NA	NA	NA
Bc_ctg1_4183	B4	2.678261841	Bcer	T	Bcer	FALSE	NA	NA	NA
Bc_ctg1_4184	B4	1.004348191	Bcer	M	Bcer	FALSE	NA	NA	NA
Bc_ctg1_4185	B4	206.784133	Bcer	P	Bcer	FALSE	NA	NA	NA
Bc_ctg1_4186	B4	43.9681319	Bcer	S	Bcer	FALSE	NA	NA	NA
Bc_ctg1_4187	B4	0.111594243	Bcer	P	Bcer	FALSE	NA	NA	NA
Bc_ctg1_4188	B4	118.8478692	Bcer	H	Bcer	FALSE	NA	NA	NA
Bc_ctg1_419	B4	1.339130921	Bcer	S	Bcer	FALSE	Bc_Ga0417192_01_rn05	Other	NA
Bc_ctg1_4190	B4	160.026145	Bcer	E	Bcer	FALSE	NA	NA	NA
Bc_ctg1_4191	B4	1434.767187	Bcer	O	Bcer	FALSE	NA	NA	NA
Bc_ctg1_4192	B4	2165.709481	Bcer	E	Bcer	FALSE	NA	NA	NA
Bc_ctg1_4193	B4	311.2363448	Bcer	E	Bcer	FALSE	NA	NA	NA
Bc_ctg1_4194	B4	19.86377532	Bcer	O	Bcer	FALSE	NA	NA	NA
Bc_ctg1_4195	B4	7.030437334	Bcer	E	Bcer	FALSE	NA	NA	NA
Bc_ctg1_4196	B4	11.27101858	Bcer	S	Bcer	FALSE	NA	NA	NA
Bc_ctg1_4197	B4	8.815945228	Bcer	K	Bcer	FALSE	NA	NA	NA
Bc_ctg1_4198	B4	13.94928042	Bcer	K	Bcer	FALSE	NA	NA	NA

Bc_ctg1_4199	B4	0	Bcer	S	Bcer	FALSE	NA	NA	NA
Bc_ctg1_42	B4	375.961006	Bcer	S	Bcer	FALSE	NA	NA	NA
Bc_ctg1_420	B4	0	Bcer	JM	Bcer	FALSE	Bc_Ga0417192_01_rn05	Ancillary biosynthesis	SMCOG1064
Bc_ctg1_4200	B4	0.111594243	Bcer	S	Bcer	FALSE	NA	NA	NA
Bc_ctg1_4201	B4	10.60145312	Bcer	K	Bcer	FALSE	NA	NA	NA
Bc_ctg1_4202	B4	30.01885147	Bcer	K	Bcer	FALSE	NA	NA	NA
Bc_ctg1_4203	B4	68.07248847	Bcer	F	Bcer	FALSE	NA	NA	NA
Bc_ctg1_4204	B4	48.98987285	Bcer	F	Bcer	FALSE	NA	NA	NA
Bc_ctg1_4205	B4	49.99422104	Bcer	F	Bcer	FALSE	NA	NA	NA
Bc_ctg1_4206	B4	13.39130921	Bcer	F	Bcer	FALSE	NA	NA	NA
Bc_ctg1_4207	B4	7.700002794	Bcer	O	Bcer	FALSE	NA	NA	NA
Bc_ctg1_4208	B4	3.794204275	Bcer	S	Bcer	FALSE	NA	NA	NA
Bc_ctg1_4209	B4	2.120290624	Bcer	NA	Bcer	FALSE	NA	NA	NA
Bc_ctg1_421	B4	0.223188487	Bcer	M	Bcer	FALSE	Bc_Ga0417192_01_rn05	Other	NA
Bc_ctg1_4210	B4	7.700002794	Bcer	O	Bcer	FALSE	NA	NA	NA
Bc_ctg1_4211	B4	10.37826464	Bcer	S	Bcer	FALSE	NA	NA	NA
Bc_ctg1_4212	B4	89.72177169	Bcer	E	Bcer	FALSE	NA	NA	NA
Bc_ctg1_4213	B4	9.485510688	Bcer	Q	Bcer	TRUE	NA	NA	NA
Bc_ctg1_4214	B4	0	Bcer	O	Bcer	FALSE	NA	NA	NA
Bc_ctg1_4215	B4	10.71304737	Bcer	MT	Bcer	FALSE	NA	NA	NA
Bc_ctg1_4216	B4	0.223188487	Bcer	NA	Bcer	FALSE	NA	NA	NA
Bc_ctg1_4217	B4	51.66813469	Bcer	L	Bcer	FALSE	NA	NA	NA
Bc_ctg1_4218	B4	7.25362582	Bcer	S	Bcer	FALSE	NA	NA	NA
Bc_ctg1_4219	B4	31.13479391	Bcer	P	Bcer	FALSE	NA	NA	NA
Bc_ctg1_422	B4	0.111594243	Bcer	GM	Bcer	FALSE	Bc_Ga0417192_01_rn05	Core biosynthesis	SMCOG1010
Bc_ctg1_4220	B4	4.463769736	Bcer	I	Bcer	FALSE	NA	NA	NA
Bc_ctg1_4221	B4	220.5102249	Bcer	E	Bcer	FALSE	NA	NA	NA
Bc_ctg1_4222	B4	7.811597037	Bcer	E	Bcer	FALSE	NA	NA	NA
Bc_ctg1_4223	B4	9.039133715	Bcer	P	Bcer	FALSE	NA	NA	NA
Bc_ctg1_4224	B4	3.236233058	Bcer	S	Bcer	FALSE	NA	NA	NA
Bc_ctg1_4225	B4	11.8289898	Bcer	EG	Bcer	FALSE	NA	NA	NA
Bc_ctg1_4226	B4	4.575363979	Bcer	K	Bcer	FALSE	NA	NA	NA
Bc_ctg1_4227	B4	1.450725164	Bcer	K	Bcer	FALSE	NA	NA	NA
Bc_ctg1_4228	B4	1.339130921	Bcer	Q	Bcer	TRUE	NA	NA	NA
Bc_ctg1_4229	B4	1.562319407	Bcer	S	Bcer	FALSE	NA	NA	NA
Bc_ctg1_423	B4	0.66956546	Bcer	GM	Bcer	FALSE	Bc_Ga0417192_01_rn05	Core biosynthesis	SMCOG1010
Bc_ctg1_4231	B4	1.562319407	Bcer	J	Bcer	FALSE	NA	NA	NA
Bc_ctg1_4232	B4	6.58406036	Bcer	S	Bcer	FALSE	NA	NA	NA
Bc_ctg1_4233	B4	23.43479111	Bcer	C	Bcer	FALSE	NA	NA	NA
Bc_ctg1_4234	B4	0.446376974	Bcer	E	Bcer	FALSE	NA	NA	NA
Bc_ctg1_4235	B4	9.708699175	Bcer	S	Bcer	FALSE	NA	NA	NA
Bc_ctg1_4236	B4	3.682610032	Bcer	E	Bcer	FALSE	NA	NA	NA
Bc_ctg1_4237	B4	0.446376974	Bcer	E	Bcer	FALSE	NA	NA	NA
Bc_ctg1_4238	B4	0	Bcer	E	Bcer	FALSE	NA	NA	NA
Bc_ctg1_4239	B4	0.781159704	Bcer	E	Bcer	FALSE	NA	NA	NA
Bc_ctg1_424	B4	2.343479111	Bcer	K	Bcer	FALSE	Bc_Ga0417192_01_rn05	Other	NA
Bc_ctg1_4240	B4	4.240581249	Bcer	E	Bcer	FALSE	NA	NA	NA
Bc_ctg1_4241	B4	5.802900656	Bcer	K	Bcer	FALSE	NA	NA	NA
Bc_ctg1_4242	B4	1.339130921	Bcer	S	Bcer	FALSE	NA	NA	NA
Bc_ctg1_4243	B4	0.557971217	Bcer	S	Bcer	FALSE	Bc_Ga0417192_01_rn30	Other	NA
Bc_ctg1_4244	B4	0.223188487	Bcer	S	Bcer	FALSE	Bc_Ga0417192_01_rn30	Other	NA
Bc_ctg1_4245	B4	41.4014643	Bcer	K	Bcer	FALSE	Bc_Ga0417192_01_rn30	Regulation	SMCOG1135
Bc_ctg1_4246	B4	37.27247729	Bcer	C	Bcer	FALSE	Bc_Ga0417192_01_rn30	Other	SMCOG1251
Bc_ctg1_4247	B4	53.90001956	Bcer	C	Bcer	FALSE	Bc_Ga0417192_01_rn30	Other	NA
Bc_ctg1_4248	B4	28.23334358	Bcer	C	Bcer	FALSE	Bc_Ga0417192_01_rn30	Other	NA
Bc_ctg1_4249	B4	24.21595082	Bcer	V	Bcer	FALSE	Bc_Ga0417192_01_rn30	Transport	SMCOG1288
Bc_ctg1_425	B4	0	Bcer	NA	Bcer	FALSE	Bc_Ga0417192_01_rn05	Other	NA
Bc_ctg1_4250	B4	28.56812631	Bcer	V	Bcer	FALSE	Bc_Ga0417192_01_rn30	Transport	SMCOG1288
Bc_ctg1_4251	B4	0	Bcer	E	Bcer	FALSE	Bc_Ga0417192_01_rn30	Other	NA
Bc_ctg1_4252	B4	1.562319407	Bcer	MQ	Bcer	TRUE	Bc_Ga0417192_01_rn30	Core biosynthesis	None
Bc_ctg1_4253	B4	3.459421545	Bcer	E	Bcer	FALSE	Bc_Ga0417192_01_rn30	Core biosynthesis	SMCOG1056
Bc_ctg1_4254	B4	1.227536677	Bcer	S	Bcer	FALSE	Bc_Ga0417192_01_rn30	Ancillary biosynthesis	SMCOG1156

Bc_ctg1_4256	B4	5.244929439	Bcer	NA	Bcer	FALSE	Bc_Ga0417192_01_rn30	Other	NA
Bc_ctg1_4257	B4	19.41739835	Bcer	P	Bcer	FALSE	Bc_Ga0417192_01_rn30	Other	NA
Bc_ctg1_4258	B4	26.00145871	Bcer	EG	Bcer	FALSE	Bc_Ga0417192_01_rn30	Other	NA
Bc_ctg1_4259	B4	38.61160821	Bcer	V	Bcer	FALSE	Bc_Ga0417192_01_rn30	Other	NA
Bc_ctg1_426	B4	21.64928322	Bcer	NT	Bcer	FALSE	Bc_Ga0417192_01_rn05	Other	SMCOG1061
Bc_ctg1_4260	B4	27.34058963	Bcer	M	Bcer	FALSE	Bc_Ga0417192_01_rn30	Other	NA
Bc_ctg1_4261	B4	522.707436	Bcer	S	Bcer	FALSE	Bc_Ga0417192_01_rn30	Ancillary biosynthesis	None
Bc_ctg1_4262	B4	1.897102138	Bcer	NA	Bcer	FALSE	Bc_Ga0417192_01_rn30	Other	NA
Bc_ctg1_4263	B4	2326.182003	Bcer	M	Bcer	FALSE	Bc_Ga0417192_01_rn30	Other	NA
Bc_ctg1_4264	B4	12.27536677	Bcer	S	Bcer	FALSE	Bc_Ga0417192_01_rn30	Ancillary biosynthesis	SMCOG1036
Bc_ctg1_4265	B4	20.53334078	Bcer	V	Bcer	FALSE	NA	NA	NA
Bc_ctg1_4266	B4	9.262322201	Bcer	T	Bcer	FALSE	NA	NA	NA
Bc_ctg1_4267	B4	6.695654603	Bcer	T	Bcer	FALSE	NA	NA	NA
Bc_ctg1_4268	B4	1.004348191	Bcer	NA	Bcer	FALSE	NA	NA	NA
Bc_ctg1_4269	B4	102.4435154	Bcer	S	Bcer	FALSE	NA	NA	NA
Bc_ctg1_427	B4	0.557971217	Bcer	NA	Bcer	FALSE	Bc_Ga0417192_01_rn05	Other	NA
Bc_ctg1_4270	B4	29.79566299	Bcer	S	Bcer	FALSE	NA	NA	NA
Bc_ctg1_4271	B4	64.83625541	Bcer	G	Bcer	FALSE	NA	NA	NA
Bc_ctg1_4272	B4	0.66956546	Bcer	G	Bcer	FALSE	NA	NA	NA
Bc_ctg1_4273	B4	2.231884868	Bcer	S	Bcer	FALSE	NA	NA	NA
Bc_ctg1_4274	B4	36.15653486	Bcer	I	Bcer	FALSE	NA	NA	NA
Bc_ctg1_4275	B4	3.013044572	Bcer	S	Bcer	FALSE	NA	NA	NA
Bc_ctg1_4276	B4	74.54495459	Bcer	E	Bcer	FALSE	NA	NA	NA
Bc_ctg1_4277	B4	116.8391728	Bcer	E	Bcer	FALSE	NA	NA	NA
Bc_ctg1_4278	B4	124.7623641	Bcer	E	Bcer	FALSE	NA	NA	NA
Bc_ctg1_4279	B4	1.115942434	Bcer	S	Bcer	FALSE	NA	NA	NA
Bc_ctg1_428	B4	1.004348191	Bcer	NA	Bcer	FALSE	Bc_Ga0417192_01_rn05	Other	NA
Bc_ctg1_4280	B4	3.347827302	Bcer	S	Bcer	FALSE	NA	NA	NA
Bc_ctg1_4281	B4	116.2812016	Bcer	K	Bcer	FALSE	NA	NA	NA
Bc_ctg1_4282	B4	11.27101858	Bcer	S	Bcer	FALSE	NA	NA	NA
Bc_ctg1_4283	B4	6.026089143	Bcer	T	Bcer	FALSE	Bc_Ga0417192_01_rn31	Other	NA
Bc_ctg1_4284	B4	15.28841134	Bcer	T	Bcer	FALSE	Bc_Ga0417192_01_rn31	Regulation	SMCOG1003
Bc_ctg1_4285	B4	147.5275898	Bcer	G	Bcer	FALSE	Bc_Ga0417192_01_rn31	Ancillary biosynthesis	SMCOG1235
Bc_ctg1_4286	B4	81.12901495	Bcer	L	Bcer	FALSE	Bc_Ga0417192_01_rn31	Other	NA
Bc_ctg1_4287	B4	17.63189046	Bcer	S	Bcer	FALSE	Bc_Ga0417192_01_rn31	Other	NA
Bc_ctg1_4288	B4	1.673913651	Bcer	M	Bcer	FALSE	Bc_Ga0417192_01_rn31	Other	NA
Bc_ctg1_4289	B4	12.6101495	Bcer	Q	Bcer	TRUE	Bc_Ga0417192_01_rn31	Core biosynthesis	None
Bc_ctg1_429	B4	0.111594243	Bcer	NA	Bcer	FALSE	Bc_Ga0417192_01_rn05	Other	NA
Bc_ctg1_4290	B4	7.476814307	Bcer	Q	Bcer	TRUE	Bc_Ga0417192_01_rn31	Core biosynthesis	None
Bc_ctg1_4291	B4	4.910146709	Bcer	IQ	Bcer	TRUE	Bc_Ga0417192_01_rn31	Ancillary biosynthesis	SMCOG1002
Bc_ctg1_4292	B4	0.111594243	Bcer	IQ	Bcer	TRUE	Bc_Ga0417192_01_rn31	Other	NA
Bc_ctg1_4293	B4	4.910146709	Bcer	IQ	Bcer	TRUE	Bc_Ga0417192_01_rn31	Other	NA
Bc_ctg1_4294	B4	3.459421545	Bcer	G	Bcer	FALSE	Bc_Ga0417192_01_rn31	Other	NA
Bc_ctg1_4295	B4	0	Bcer	S	Bcer	FALSE	Bc_Ga0417192_01_rn31	Other	NA
Bc_ctg1_4296	B4	0.223188487	Bcer	EGP	Bcer	FALSE	Bc_Ga0417192_01_rn31	Other	NA
Bc_ctg1_4297	B4	3.682610032	Bcer	S	Bcer	FALSE	NA	NA	NA
Bc_ctg1_4298	B4	53.00726561	Bcer	K	Bcer	FALSE	NA	NA	NA
Bc_ctg1_4299	B4	0.33478273	Bcer	S	Bcer	FALSE	NA	NA	NA
Bc_ctg1_43	B4	39.05798519	Bcer	K	Bcer	FALSE	NA	NA	NA
Bc_ctg1_430	B4	0	Bcer	Q	Bcer	TRUE	Bc_Ga0417192_01_rn05	Other	NA
Bc_ctg1_4300	B4	1.562319407	Bcer	S	Bcer	FALSE	NA	NA	NA
Bc_ctg1_4302	B4	43.07537795	Bcer	S	Bcer	FALSE	NA	NA	NA
Bc_ctg1_4303	B4	383.5494145	Bcer	D	Bcer	FALSE	NA	NA	NA
Bc_ctg1_4304	B4	2.678261841	Bcer	S	Bcer	FALSE	NA	NA	NA
Bc_ctg1_4305	B4	13.83768618	Bcer	F	Bcer	FALSE	NA	NA	NA
Bc_ctg1_4306	B4	80.12466675	Bcer	H	Bcer	FALSE	NA	NA	NA
Bc_ctg1_4307	B4	16.40435378	Bcer	V	Bcer	FALSE	NA	NA	NA
Bc_ctg1_4308	B4	15.84638256	Bcer	K	Bcer	FALSE	NA	NA	NA
Bc_ctg1_4309	B4	33.7014615	Bcer	O	Bcer	FALSE	NA	NA	NA
Bc_ctg1_431	B4	4.240581249	Bcer	S	Bcer	FALSE	NA	NA	NA
Bc_ctg1_4310	B4	4.463769736	Bcer	K	Bcer	FALSE	NA	NA	NA
Bc_ctg1_4311	B4	0.781159704	Bcer	C	Bcer	FALSE	NA	NA	NA

Bc_ctg1_4312	B4	2.008696381	Bcer	EGP	Bcer	FALSE	NA	NA	NA
Bc_ctg1_4313	B4	22.98841414	Bcer	NA	Bcer	FALSE	NA	NA	NA
Bc_ctg1_4314	B4	22.09566019	Bcer	S	Bcer	FALSE	NA	NA	NA
Bc_ctg1_4315	B4	3.571015789	Bcer	S	Bcer	FALSE	NA	NA	NA
Bc_ctg1_4316	B4	50.21740953	Bcer	S	Bcer	FALSE	NA	NA	NA
Bc_ctg1_4317	B4	28.56812631	Bcer	NT	Bcer	FALSE	NA	NA	NA
Bc_ctg1_4318	B4	12.72174375	Bcer	NA	Bcer	FALSE	NA	NA	NA
Bc_ctg1_4319	B4	10.15507615	Bcer	S	Bcer	FALSE	NA	NA	NA
Bc_ctg1_432	B4	2.901450328	Bcer	O	Bcer	FALSE	NA	NA	NA
Bc_ctg1_4320	B4	0	Bcer	S	Bcer	FALSE	NA	NA	NA
Bc_ctg1_4321	B4	129.8956993	Bcer	P	Bcer	FALSE	NA	NA	NA
Bc_ctg1_4322	B4	144.7377337	Bcer	S	Bcer	FALSE	NA	NA	NA
Bc_ctg1_4323	B4	4.352175492	Bcer	S	Bcer	FALSE	NA	NA	NA
Bc_ctg1_4326	B4	48.32030739	Bcer	C	Bcer	FALSE	NA	NA	NA
Bc_ctg1_4328	B4	3.905798519	Bcer	E	Bcer	FALSE	NA	NA	NA
Bc_ctg1_4329	B4	10.04348191	Bcer	J	Bcer	FALSE	NA	NA	NA
Bc_ctg1_433	B4	9.262322201	Bcer	G	Bcer	FALSE	NA	NA	NA
Bc_ctg1_4330	B4	255.7740059	Bcer	S	Bcer	FALSE	NA	NA	NA
Bc_ctg1_4331	B4	30.80001118	Bcer	S	Bcer	FALSE	NA	NA	NA
Bc_ctg1_4332	B4	2.231884868	Bcer	K	Bcer	FALSE	NA	NA	NA
Bc_ctg1_4333	B4	26.78261841	Bcer	P	Bcer	FALSE	NA	NA	NA
Bc_ctg1_4335	B4	30.3536342	Bcer	GM	Bcer	FALSE	NA	NA	NA
Bc_ctg1_4336	B4	13.83768618	Bcer	C	Bcer	FALSE	NA	NA	NA
Bc_ctg1_4337	B4	2.901450328	Bcer	L	Bcer	FALSE	NA	NA	NA
Bc_ctg1_4338	B4	29.12609753	Bcer	NU	Bcer	FALSE	NA	NA	NA
Bc_ctg1_4339	B4	4.240581249	Bcer	E	Bcer	FALSE	NA	NA	NA
Bc_ctg1_4340	B4	5.468117926	Bcer	H	Bcer	FALSE	NA	NA	NA
Bc_ctg1_4341	B4	4.352175492	Bcer	S	Bcer	FALSE	NA	NA	NA
Bc_ctg1_4342	B4	7.365220064	Bcer	G	Bcer	FALSE	NA	NA	NA
Bc_ctg1_4343	B4	2.566667598	Bcer	NA	Bcer	FALSE	NA	NA	NA
Bc_ctg1_4344	B4	7.030437334	Bcer	M	Bcer	FALSE	NA	NA	NA
Bc_ctg1_4345	B4	16.73913651	Bcer	F	Bcer	FALSE	NA	NA	NA
Bc_ctg1_4346	B4	45.19566857	Bcer	S	Bcer	FALSE	NA	NA	NA
Bc_ctg1_4347	B4	6.695654603	Bcer	S	Bcer	FALSE	NA	NA	NA
Bc_ctg1_4348	B4	84.92321922	Bcer	P	Bcer	FALSE	NA	NA	NA
Bc_ctg1_4349	B4	46.53479949	Bcer	S	Bcer	FALSE	NA	NA	NA
Bc_ctg1_435	B4	12.27536677	Bcer	T	Bcer	FALSE	NA	NA	NA
Bc_ctg1_4350	B4	52.33770015	Bcer	S	Bcer	FALSE	NA	NA	NA
Bc_ctg1_4351	B4	4.575363979	Bcer	I	Bcer	FALSE	NA	NA	NA
Bc_ctg1_4352	B4	0.111594243	Bcer	I	Bcer	FALSE	NA	NA	NA
Bc_ctg1_4353	B4	0.111594243	Bcer	M	Bcer	FALSE	NA	NA	NA
Bc_ctg1_4355	B4	1.785507894	Bcer	GM	Bcer	FALSE	NA	NA	NA
Bc_ctg1_4356	B4	10.26667039	Bcer	S	Bcer	FALSE	NA	NA	NA
Bc_ctg1_4357	B4	49.43624982	Bcer	S	Bcer	FALSE	NA	NA	NA
Bc_ctg1_4358	B4	0.111594243	Bcer	NA	Bcer	FALSE	NA	NA	NA
Bc_ctg1_4359	B4	2.008696381	Bcer	K	Bcer	FALSE	NA	NA	NA
Bc_ctg1_436	B4	21.20290624	Bcer	S	Bcer	FALSE	NA	NA	NA
Bc_ctg1_4360	B4	1.897102138	Bcer	S	Bcer	FALSE	NA	NA	NA
Bc_ctg1_4361	B4	246.4000894	Bcer	S	Bcer	FALSE	NA	NA	NA
Bc_ctg1_4362	B4	0	Bcer	L	Bcer	FALSE	NA	NA	NA
Bc_ctg1_4364	B4	87.37829258	Bcer	E	Bcer	FALSE	NA	NA	NA
Bc_ctg1_4365	B4	0	Bcer	NA	Bcer	FALSE	NA	NA	NA
Bc_ctg1_4367	B4	7.030437334	Bcer	J	Bcer	FALSE	NA	NA	NA
Bc_ctg1_4368	B4	7.25362582	Bcer	S	Bcer	FALSE	NA	NA	NA
Bc_ctg1_437	B4	8.704350984	Bcer	K	Bcer	FALSE	NA	NA	NA
Bc_ctg1_4370	B4	15.40000559	Bcer	M	Bcer	FALSE	NA	NA	NA
Bc_ctg1_4371	B4	4.352175492	Bcer	J	Bcer	FALSE	NA	NA	NA
Bc_ctg1_4372	B4	13.72609194	Bcer	M	Bcer	FALSE	NA	NA	NA
Bc_ctg1_4373	B4	5.802900656	Bcer	F	Bcer	FALSE	NA	NA	NA
Bc_ctg1_4374	B4	27.45218387	Bcer	J	Bcer	FALSE	NA	NA	NA
Bc_ctg1_4375	B4	9.708699175	Bcer	G	Bcer	FALSE	NA	NA	NA
Bc_ctg1_4376	B4	96.08264356	Bcer	OU	Bcer	FALSE	NA	NA	NA

Bc_ctg1_4377	B4	256.1087886	Bcer	O	Bcer	FALSE	NA	NA	NA
Bc_ctg1_4378	B4	1.115942434	Bcer	M	Bcer	FALSE	NA	NA	NA
Bc_ctg1_4379	B4	16.40435378	Bcer	G	Bcer	FALSE	NA	NA	NA
Bc_ctg1_438	B4	4.017392762	Bcer	S	Bcer	FALSE	NA	NA	NA
Bc_ctg1_4380	B4	7.811597037	Bcer	S	Bcer	FALSE	NA	NA	NA
Bc_ctg1_4381	B4	5.802900656	Bcer	K	Bcer	FALSE	NA	NA	NA
Bc_ctg1_4382	B4	3.459421545	Bcer	EGP	Bcer	FALSE	NA	NA	NA
Bc_ctg1_4383	B4	1.450725164	Bcer	S	Bcer	FALSE	NA	NA	NA
Bc_ctg1_4384	B4	111.3710549	Bcer	E	Bcer	FALSE	NA	NA	NA
Bc_ctg1_4385	B4	8.815945228	Bcer	S	Bcer	FALSE	NA	NA	NA
Bc_ctg1_4386	B4	13.16812072	Bcer	K	Bcer	FALSE	NA	NA	NA
Bc_ctg1_4387	B4	12.94493223	Bcer	S	Bcer	FALSE	NA	NA	NA
Bc_ctg1_4388	B4	131.7928014	Bcer	CG	Bcer	FALSE	NA	NA	NA
Bc_ctg1_4389	B4	30.80001118	Bcer	S	Bcer	FALSE	NA	NA	NA
Bc_ctg1_439	B4	2.455073355	Bcer	M	Bcer	FALSE	NA	NA	NA
Bc_ctg1_4390	B4	13.83768618	Bcer	K	Bcer	FALSE	NA	NA	NA
Bc_ctg1_4391	B4	0.33478273	Bcer	K	Bcer	FALSE	NA	NA	NA
Bc_ctg1_4392	B4	14.50725164	Bcer	T	Bcer	FALSE	NA	NA	NA
Bc_ctg1_4393	B4	8.369568254	Bcer	S	Bcer	FALSE	NA	NA	NA
Bc_ctg1_4394	B4	16.06957105	Bcer	J	Bcer	FALSE	NA	NA	NA
Bc_ctg1_4395	B4	20.64493503	Bcer	J	Bcer	FALSE	NA	NA	NA
Bc_ctg1_4396	B4	6.137683386	Bcer	M	Bcer	FALSE	NA	NA	NA
Bc_ctg1_4397	B4	4.240581249	Bcer	S	Bcer	FALSE	NA	NA	NA
Bc_ctg1_4398	B4	1.450725164	Bcer	K	Bcer	FALSE	NA	NA	NA
Bc_ctg1_4399	B4	0	Bcer	NA	Bcer	FALSE	NA	NA	NA
Bc_ctg1_44	B4	59.25654324	Bcer	GT	Bcer	FALSE	NA	NA	NA
Bc_ctg1_4400	B4	0.892753947	Bcer	S	Bcer	FALSE	NA	NA	NA
Bc_ctg1_4401	B4	0.892753947	Bcer	NA	Bcer	FALSE	NA	NA	NA
Bc_ctg1_4402	B4	0.446376974	Bcer	NA	Bcer	FALSE	NA	NA	NA
Bc_ctg1_4403	B4	1.115942434	Bcer	NA	Bcer	FALSE	NA	NA	NA
Bc_ctg1_4404	B4	95.18988961	Bcer	NA	Bcer	FALSE	NA	NA	NA
Bc_ctg1_4405	B4	9.039133715	Bcer	NA	Bcer	FALSE	NA	NA	NA
Bc_ctg1_4406	B4	0	Bcer	NA	Bcer	FALSE	NA	NA	NA
Bc_ctg1_4407	B4	1.450725164	Bcer	NA	Bcer	FALSE	NA	NA	NA
Bc_ctg1_4408	B4	0	Bcer	NA	Bcer	FALSE	NA	NA	NA
Bc_ctg1_441	B4	8.815945228	Bcer	M	Bcer	FALSE	NA	NA	NA
Bc_ctg1_4411	B4	0	Bcer	NA	Bcer	FALSE	NA	NA	NA
Bc_ctg1_4414	B4	0	Bcer	NA	Bcer	FALSE	NA	NA	NA
Bc_ctg1_4416	B4	0.111594243	Bcer	NA	Bcer	FALSE	NA	NA	NA
Bc_ctg1_4418	B4	0	Bcer	NA	Bcer	FALSE	NA	NA	NA
Bc_ctg1_4419	B4	0.781159704	Bcer	D	Bcer	FALSE	NA	NA	NA
Bc_ctg1_442	B4	11.49420707	Bcer	NA	Bcer	FALSE	NA	NA	NA
Bc_ctg1_4420	B4	0	Bcer	NA	Bcer	FALSE	NA	NA	NA
Bc_ctg1_4422	B4	1.450725164	Bcer	NA	Bcer	FALSE	NA	NA	NA
Bc_ctg1_4423	B4	0	Bcer	NA	Bcer	FALSE	NA	NA	NA
Bc_ctg1_4424	B4	0.66956546	Bcer	NA	Bcer	FALSE	NA	NA	NA
Bc_ctg1_4425	B4	1.227536677	Bcer	NA	Bcer	FALSE	NA	NA	NA
Bc_ctg1_4426	B4	0	Bcer	S	Bcer	FALSE	NA	NA	NA
Bc_ctg1_4427	B4	0	Bcer	L	Bcer	FALSE	NA	NA	NA
Bc_ctg1_4428	B4	9.039133715	Bcer	S	Bcer	FALSE	NA	NA	NA
Bc_ctg1_4429	B4	0.111594243	Bcer	S	Bcer	FALSE	NA	NA	NA
Bc_ctg1_443	B4	18.63623865	Bcer	M	Bcer	FALSE	NA	NA	NA
Bc_ctg1_4430	B4	286.3508285	Bcer	S	Bcer	FALSE	NA	NA	NA
Bc_ctg1_4431	B4	16.18116529	Bcer	NA	Bcer	FALSE	NA	NA	NA
Bc_ctg1_4433	B4	0	Bcer	NA	Bcer	FALSE	NA	NA	NA
Bc_ctg1_4434	B4	9.039133715	Bcer	Q	Bcer	TRUE	NA	NA	NA
Bc_ctg1_4435	B4	1630.949867	Bcer	F	Bcer	FALSE	NA	NA	NA
Bc_ctg1_4436	B4	24.55073355	Bcer	NA	Bcer	FALSE	NA	NA	NA
Bc_ctg1_4437	B4	86.26235014	Bcer	L	Bcer	FALSE	NA	NA	NA
Bc_ctg1_4438	B4	3.905798519	Bcer	G	Bcer	FALSE	NA	NA	NA
Bc_ctg1_4439	B4	9.262322201	Bcer	E	Bcer	FALSE	NA	NA	NA
Bc_ctg1_444	B4	2.566667598	Bcer	NA	Bcer	FALSE	NA	NA	NA

Bc_ctg1_4440	B4	43.29856644	Bcer	NA	Bcer	FALSE	NA	NA	NA
Bc_ctg1_4441	B4	0.33478273	Bcer	C	Bcer	FALSE	NA	NA	NA
Bc_ctg1_4442	B4	0.223188487	Bcer	K	Bcer	FALSE	NA	NA	NA
Bc_ctg1_4443	B4	7.142031577	Bcer	S	Bcer	FALSE	NA	NA	NA
Bc_ctg1_4444	B4	0	Bcer	NA	Bcer	FALSE	NA	NA	NA
Bc_ctg1_4445	B4	107.3536621	Bcer	S	Bcer	FALSE	NA	NA	NA
Bc_ctg1_4446	B4	5.133335196	Bcer	S	Bcer	FALSE	NA	NA	NA
Bc_ctg1_4447	B4	20.3101523	Bcer	O	Bcer	FALSE	NA	NA	NA
Bc_ctg1_4448	B4	3.124638815	Bcer	E	Bcer	FALSE	NA	NA	NA
Bc_ctg1_4449	B4	0.66956546	Bcer	S	Bcer	FALSE	NA	NA	NA
Bc_ctg1_445	B4	557.7480285	Bcer	NA	Bcer	FALSE	NA	NA	NA
Bc_ctg1_4450	B4	0.33478273	Bcer	S	Bcer	FALSE	NA	NA	NA
Bc_ctg1_4451	B4	633.2973312	Bcer	C	Bcer	FALSE	NA	NA	NA
Bc_ctg1_4452	B4	305.5450384	Bcer	C	Bcer	FALSE	NA	NA	NA
Bc_ctg1_4453	B4	112.7101858	Bcer	C	Bcer	FALSE	NA	NA	NA
Bc_ctg1_4454	B4	171.6319463	Bcer	C	Bcer	FALSE	NA	NA	NA
Bc_ctg1_4455	B4	26.44783568	Bcer	K	Bcer	FALSE	NA	NA	NA
Bc_ctg1_4456	B4	85.25800195	Bcer	H	Bcer	FALSE	NA	NA	NA
Bc_ctg1_4457	B4	89.4985832	Bcer	H	Bcer	FALSE	NA	NA	NA
Bc_ctg1_4458	B4	132.7971496	Bcer	H	Bcer	FALSE	NA	NA	NA
Bc_ctg1_4459	B4	30.3536342	Bcer	H	Bcer	FALSE	NA	NA	NA
Bc_ctg1_446	B4	23.6579796	Bcer	T	Bcer	FALSE	NA	NA	NA
Bc_ctg1_4460	B4	3.013044572	Bcer	H	Bcer	FALSE	NA	NA	NA
Bc_ctg1_4461	B4	114.4956937	Bcer	P	Bcer	FALSE	NA	NA	NA
Bc_ctg1_4462	B4	4.686958222	Bcer	S	Bcer	FALSE	NA	NA	NA
Bc_ctg1_4463	B4	0.66956546	Bcer	NA	Bcer	FALSE	NA	NA	NA
Bc_ctg1_4464	B4	6.807248847	Bcer	H	Bcer	FALSE	NA	NA	NA
Bc_ctg1_4465	B4	30.24203996	Bcer	S	Bcer	FALSE	NA	NA	NA
Bc_ctg1_4466	B4	84.14205952	Bcer	H	Bcer	FALSE	NA	NA	NA
Bc_ctg1_4467	B4	64.72466117	Bcer	P	Bcer	FALSE	NA	NA	NA
Bc_ctg1_4468	B4	601.0465949	Bcer	C	Bcer	FALSE	NA	NA	NA
Bc_ctg1_4469	B4	7.923191281	Bcer	D	Bcer	FALSE	NA	NA	NA
Bc_ctg1_447	B4	0.557971217	Bcer	S	Bcer	FALSE	NA	NA	NA
Bc_ctg1_4470	B4	0.781159704	Bcer	M	Bcer	FALSE	NA	NA	NA
Bc_ctg1_4472	B4	0.33478273	Bcer	NA	Bcer	FALSE	NA	NA	NA
Bc_ctg1_4473	B4	9.485510688	Bcer	Q	Bcer	TRUE	NA	NA	NA
Bc_ctg1_4474	B4	8.257974011	Bcer	S	Bcer	FALSE	NA	NA	NA
Bc_ctg1_4475	B4	0.223188487	Bcer	NA	Bcer	FALSE	NA	NA	NA
Bc_ctg1_4476	B4	11.60580131	Bcer	S	Bcer	FALSE	NA	NA	NA
Bc_ctg1_4477	B4	0.111594243	Bcer	NA	Bcer	FALSE	NA	NA	NA
Bc_ctg1_448	B4	0.223188487	Bcer	S	Bcer	FALSE	NA	NA	NA
Bc_ctg1_4480	B4	28.01015509	Bcer	S	Bcer	FALSE	NA	NA	NA
Bc_ctg1_4481	B4	218.1667458	Bcer	S	Bcer	FALSE	NA	NA	NA
Bc_ctg1_4482	B4	272.5131424	Bcer	S	Bcer	FALSE	NA	NA	NA
Bc_ctg1_4483	B4	1.562319407	Bcer	S	Bcer	FALSE	NA	NA	NA
Bc_ctg1_4484	B4	0.111594243	Bcer	L	Bcer	FALSE	NA	NA	NA
Bc_ctg1_4485	B4	0	Bcer	NA	Bcer	FALSE	NA	NA	NA
Bc_ctg1_4486	B4	86.70872711	Bcer	C	Bcer	FALSE	NA	NA	NA
Bc_ctg1_4487	B4	16.29275954	Bcer	S	Bcer	FALSE	NA	NA	NA
Bc_ctg1_4488	B4	165.8290457	Bcer	S	Bcer	FALSE	NA	NA	NA
Bc_ctg1_4489	B4	24.66232779	Bcer	J	Bcer	FALSE	NA	NA	NA
Bc_ctg1_449	B4	2.901450328	Bcer	S	Bcer	FALSE	NA	NA	NA
Bc_ctg1_4490	B4	41.51305854	Bcer	J	Bcer	FALSE	NA	NA	NA
Bc_ctg1_4491	B4	1.785507894	Bcer	Q	Bcer	TRUE	NA	NA	NA
Bc_ctg1_4492	B4	42.182624	Bcer	H	Bcer	FALSE	NA	NA	NA
Bc_ctg1_4493	B4	212.5870337	Bcer	J	Bcer	FALSE	NA	NA	NA
Bc_ctg1_4494	B4	5.57971217	Bcer	S	Bcer	FALSE	NA	NA	NA
Bc_ctg1_4495	B4	4.017392762	Bcer	K	Bcer	FALSE	NA	NA	NA
Bc_ctg1_4496	B4	7.365220064	Bcer	E	Bcer	FALSE	NA	NA	NA
Bc_ctg1_4498	B4	17.63189046	Bcer	J	Bcer	FALSE	NA	NA	NA
Bc_ctg1_4499	B4	18.97102138	Bcer	S	Bcer	FALSE	NA	NA	NA
Bc_ctg1_45	B4	1.450725164	Bcer	E	Bcer	FALSE	NA	NA	NA

Bc_ctg1_450	B4	37.38407154	Bcer	S	Bcer	FALSE	NA	NA	NA
Bc_ctg1_4500	B4	17.96667319	Bcer	T	Bcer	FALSE	NA	NA	NA
Bc_ctg1_4501	B4	4.686958222	Bcer	NA	Bcer	FALSE	NA	NA	NA
Bc_ctg1_4502	B4	244.7261758	Bcer	D	Bcer	FALSE	NA	NA	NA
Bc_ctg1_4503	B4	78.00437613	Bcer	S	Bcer	FALSE	NA	NA	NA
Bc_ctg1_4504	B4	107.5768506	Bcer	KLT	Bcer	FALSE	NA	NA	NA
Bc_ctg1_4505	B4	163.8203493	Bcer	NU	Bcer	FALSE	NA	NA	NA
Bc_ctg1_4506	B4	5.468117926	Bcer	S	Bcer	FALSE	NA	NA	NA
Bc_ctg1_4507	B4	70.75075031	Bcer	MS	Bcer	FALSE	NA	NA	NA
Bc_ctg1_4508	B4	35.26378091	Bcer	S	Bcer	FALSE	NA	NA	NA
Bc_ctg1_4509	B4	8.815945228	Bcer	S	Bcer	FALSE	NA	NA	NA
Bc_ctg1_451	B4	46.64639374	Bcer	V	Bcer	FALSE	NA	NA	NA
Bc_ctg1_4510	B4	5.468117926	Bcer	I	Bcer	FALSE	NA	NA	NA
Bc_ctg1_4511	B4	52.33770015	Bcer	S	Bcer	FALSE	NA	NA	NA
Bc_ctg1_4512	B4	65.28263238	Bcer	U	Bcer	FALSE	NA	NA	NA
Bc_ctg1_4513	B4	1.227536677	Bcer	K	Bcer	FALSE	NA	NA	NA
Bc_ctg1_4514	B4	12.16377253	Bcer	EG	Bcer	FALSE	NA	NA	NA
Bc_ctg1_4515	B4	18.07826743	Bcer	C	Bcer	FALSE	NA	NA	NA
Bc_ctg1_4516	B4	3.459421545	Bcer	M	Bcer	FALSE	NA	NA	NA
Bc_ctg1_4517	B4	73.65220064	Bcer	S	Bcer	FALSE	NA	NA	NA
Bc_ctg1_4518	B4	30.68841693	Bcer	K	Bcer	FALSE	NA	NA	NA
Bc_ctg1_4519	B4	6.807248847	Bcer	S	Bcer	FALSE	NA	NA	NA
Bc_ctg1_452	B4	3.124638815	Bcer	NA	Bcer	FALSE	NA	NA	NA
Bc_ctg1_4520	B4	1.339130921	Bcer	K	Bcer	FALSE	NA	NA	NA
Bc_ctg1_4521	B4	31.58117088	Bcer	E	Bcer	FALSE	NA	NA	NA
Bc_ctg1_4522	B4	11.38261283	Bcer	K	Bcer	FALSE	NA	NA	NA
Bc_ctg1_4523	B4	9.262322201	Bcer	V	Bcer	FALSE	NA	NA	NA
Bc_ctg1_4524	B4	11.27101858	Bcer	U	Bcer	FALSE	NA	NA	NA
Bc_ctg1_4525	B4	239.5928406	Bcer	S	Bcer	FALSE	NA	NA	NA
Bc_ctg1_4526	B4	2.008696381	Bcer	S	Bcer	FALSE	NA	NA	NA
Bc_ctg1_4527	B4	1.339130921	Bcer	E	Bcer	FALSE	NA	NA	NA
Bc_ctg1_4528	B4	6.472466117	Bcer	S	Bcer	FALSE	NA	NA	NA
Bc_ctg1_4529	B4	98.87249964	Bcer	V	Bcer	FALSE	NA	NA	NA
Bc_ctg1_453	B4	69.74640212	Bcer	S	Bcer	FALSE	NA	NA	NA
Bc_ctg1_4530	B4	96.75220902	Bcer	F	Bcer	FALSE	NA	NA	NA
Bc_ctg1_4531	B4	23.32319687	Bcer	H	Bcer	FALSE	NA	NA	NA
Bc_ctg1_4532	B4	375.961006	Bcer	M	Bcer	FALSE	NA	NA	NA
Bc_ctg1_4533	B4	75.88408551	Bcer	I	Bcer	FALSE	NA	NA	NA
Bc_ctg1_4534	B4	43.85653765	Bcer	I	Bcer	FALSE	NA	NA	NA
Bc_ctg1_4535	B4	104.6754003	Bcer	S	Bcer	FALSE	NA	NA	NA
Bc_ctg1_4536	B4	10.04348191	Bcer	U	Bcer	FALSE	NA	NA	NA
Bc_ctg1_4537	B4	107.0188794	Bcer	U	Bcer	FALSE	NA	NA	NA
Bc_ctg1_4538	B4	4.686958222	Bcer	S	Bcer	FALSE	NA	NA	NA
Bc_ctg1_4539	B4	159.0217968	Bcer	S	Bcer	FALSE	NA	NA	NA
Bc_ctg1_454	B4	3.347827302	Bcer	NA	Bcer	FALSE	NA	NA	NA
Bc_ctg1_4540	B4	21.53768897	Bcer	S	Bcer	FALSE	NA	NA	NA
Bc_ctg1_4541	B4	37.49566578	Bcer	S	Bcer	FALSE	NA	NA	NA
Bc_ctg1_4542	B4	0.223188487	Bcer	O	Bcer	FALSE	NA	NA	NA
Bc_ctg1_4543	B4	910.6090261	Bcer	E	Bcer	FALSE	NA	NA	NA
Bc_ctg1_4544	B4	1.115942434	Bcer	K	Bcer	FALSE	NA	NA	NA
Bc_ctg1_4545	B4	6.026089143	Bcer	NA	Bcer	FALSE	NA	NA	NA
Bc_ctg1_4546	B4	0.33478273	Bcer	M	Bcer	FALSE	NA	NA	NA
Bc_ctg1_4547	B4	69.30002515	Bcer	P	Bcer	FALSE	NA	NA	NA
Bc_ctg1_4548	B4	30.13044572	Bcer	E	Bcer	FALSE	NA	NA	NA
Bc_ctg1_4549	B4	76.55365097	Bcer	C	Bcer	FALSE	NA	NA	NA
Bc_ctg1_455	B4	0.111594243	Bcer	NA	Bcer	FALSE	NA	NA	NA
Bc_ctg1_4550	B4	75.54930278	Bcer	NA	Bcer	FALSE	NA	NA	NA
Bc_ctg1_4551	B4	25.66667598	Bcer	Q	Bcer	TRUE	NA	NA	NA
Bc_ctg1_4552	B4	0.446376974	Bcer	F	Bcer	FALSE	NA	NA	NA
Bc_ctg1_4553	B4	21.76087746	Bcer	M	Bcer	FALSE	NA	NA	NA
Bc_ctg1_4554	B4	5.468117926	Bcer	T	Bcer	FALSE	NA	NA	NA
Bc_ctg1_4555	B4	9.485510688	Bcer	K	Bcer	FALSE	NA	NA	NA

Bc_ctg1_4556	B4	63.72031298	Bcer	K	Bcer	FALSE	NA	NA	NA
Bc_ctg1_4557	B4	50.55219226	Bcer	S	Bcer	FALSE	NA	NA	NA
Bc_ctg1_4558	B4	4195.162392	Bcer	C	Bcer	FALSE	NA	NA	NA
Bc_ctg1_4559	B4	2.678261841	Bcer	EP	Bcer	FALSE	NA	NA	NA
Bc_ctg1_456	B4	2.008696381	Bcer	L	Bcer	FALSE	NA	NA	NA
Bc_ctg1_4560	B4	1.562319407	Bcer	EP	Bcer	FALSE	NA	NA	NA
Bc_ctg1_4562	B4	134.359469	Bcer	G	Bcer	FALSE	NA	NA	NA
Bc_ctg1_4563	B4	97.19858599	Bcer	EG	Bcer	FALSE	NA	NA	NA
Bc_ctg1_4564	B4	86.1507559	Bcer	G	Bcer	FALSE	NA	NA	NA
Bc_ctg1_4565	B4	2.566667598	Bcer	S	Bcer	FALSE	NA	NA	NA
Bc_ctg1_4566	B4	0	Bcer	M	Bcer	FALSE	NA	NA	NA
Bc_ctg1_4567	B4	0.892753947	Bcer	O	Bcer	FALSE	NA	NA	NA
Bc_ctg1_4568	B4	17.40870197	Bcer	S	Bcer	FALSE	NA	NA	NA
Bc_ctg1_4569	B4	60.14929719	Bcer	EGP	Bcer	FALSE	NA	NA	NA
Bc_ctg1_457	B4	402.1856532	Bcer	K	Bcer	FALSE	NA	NA	NA
Bc_ctg1_4570	B4	26.00145871	Bcer	S	Bcer	FALSE	NA	NA	NA
Bc_ctg1_4571	B4	9.597104932	Bcer	E	Bcer	FALSE	NA	NA	NA
Bc_ctg1_4572	B4	20.19855805	Bcer	EM	Bcer	FALSE	NA	NA	NA
Bc_ctg1_4573	B4	39.16957943	Bcer	E	Bcer	FALSE	NA	NA	NA
Bc_ctg1_4574	B4	25.77827022	Bcer	NA	Bcer	FALSE	NA	NA	NA
Bc_ctg1_4575	B4	2.455073355	Bcer	V	Bcer	FALSE	NA	NA	NA
Bc_ctg1_4576	B4	1.004348191	Bcer	NA	Bcer	FALSE	NA	NA	NA
Bc_ctg1_4577	B4	58.92176051	Bcer	NA	Bcer	FALSE	NA	NA	NA
Bc_ctg1_4578	B4	0.446376974	Bcer	K	Bcer	FALSE	NA	NA	NA
Bc_ctg1_4579	B4	55.57393321	Bcer	S	Bcer	FALSE	NA	NA	NA
Bc_ctg1_458	B4	45.97682828	Bcer	NA	Bcer	FALSE	NA	NA	NA
Bc_ctg1_4580	B4	99.5420651	Bcer	C	Bcer	FALSE	NA	NA	NA
Bc_ctg1_4581	B4	75.32611429	Bcer	S	Bcer	FALSE	NA	NA	NA
Bc_ctg1_4582	B4	119.7406232	Bcer	T	Bcer	FALSE	NA	NA	NA
Bc_ctg1_4583	B4	0.223188487	Bcer	O	Bcer	FALSE	NA	NA	NA
Bc_ctg1_4584	B4	5.57971217	Bcer	H	Bcer	FALSE	NA	NA	NA
Bc_ctg1_4585	B4	2.455073355	Bcer	I	Bcer	FALSE	NA	NA	NA
Bc_ctg1_4586	B4	3.124638815	Bcer	I	Bcer	FALSE	NA	NA	NA
Bc_ctg1_4587	B4	4.017392762	Bcer	E	Bcer	FALSE	NA	NA	NA
Bc_ctg1_4588	B4	18.07826743	Bcer	K	Bcer	FALSE	NA	NA	NA
Bc_ctg1_4589	B4	37.38407154	Bcer	KT	Bcer	FALSE	NA	NA	NA
Bc_ctg1_459	B4	6.472466117	Bcer	C	Bcer	FALSE	NA	NA	NA
Bc_ctg1_4590	B4	9.262322201	Bcer	E	Bcer	FALSE	NA	NA	NA
Bc_ctg1_4591	B4	1.450725164	Bcer	S	Bcer	FALSE	NA	NA	NA
Bc_ctg1_4592	B4	3.124638815	Bcer	S	Bcer	FALSE	NA	NA	NA
Bc_ctg1_4593	B4	0	Bcer	NA	Bcer	FALSE	NA	NA	NA
Bc_ctg1_4596	B4	5.691306413	Bcer	NA	Bcer	FALSE	NA	NA	NA
Bc_ctg1_4597	B4	49.99422104	Bcer	KLT	Bcer	FALSE	NA	NA	NA
Bc_ctg1_4598	B4	76.8884337	Bcer	S	Bcer	FALSE	NA	NA	NA
Bc_ctg1_4599	B4	20.97971776	Bcer	I	Bcer	FALSE	NA	NA	NA
Bc_ctg1_46	B4	1.897102138	Bcer	S	Bcer	FALSE	NA	NA	NA
Bc_ctg1_460	B4	7.811597037	Bcer	S	Bcer	FALSE	NA	NA	NA
Bc_ctg1_4600	B4	0.557971217	Bcer	P	Bcer	FALSE	NA	NA	NA
Bc_ctg1_4602	B4	59.70292021	Bcer	CO	Bcer	FALSE	NA	NA	NA
Bc_ctg1_4603	B4	18.85942713	Bcer	J	Bcer	FALSE	NA	NA	NA
Bc_ctg1_4604	B4	0	Bcer	NA	Bcer	FALSE	NA	NA	NA
Bc_ctg1_4605	B4	0	Bcer	NA	Bcer	FALSE	NA	NA	NA
Bc_ctg1_4606	B4	0.223188487	Bcer	NA	Bcer	FALSE	NA	NA	NA
Bc_ctg1_4607	B4	0	Bcer	NA	Bcer	FALSE	NA	NA	NA
Bc_ctg1_4608	B4	0.446376974	Bcer	NA	Bcer	FALSE	NA	NA	NA
Bc_ctg1_4609	B4	0	Bcer	NA	Bcer	FALSE	NA	NA	NA
Bc_ctg1_461	B4	0.33478273	Bcer	F	Bcer	FALSE	NA	NA	NA
Bc_ctg1_4610	B4	12.6101495	Bcer	M	Bcer	FALSE	NA	NA	NA
Bc_ctg1_4611	B4	53.11885985	Bcer	M	Bcer	FALSE	NA	NA	NA
Bc_ctg1_4612	B4	0.223188487	Bcer	NA	Bcer	FALSE	NA	NA	NA
Bc_ctg1_4613	B4	0.223188487	Bcer	NA	Bcer	FALSE	NA	NA	NA
Bc_ctg1_4614	B4	0.223188487	Bcer	S	Bcer	FALSE	NA	NA	NA

Bc_ctg1_4616	B4	13.05652648	Bcer	NA	Bcer	FALSE	NA	NA	NA
Bc_ctg1_4617	B4	0.223188487	Bcer	NA	Bcer	FALSE	NA	NA	NA
Bc_ctg1_4618	B4	12.83333799	Bcer	IQ	Bcer	TRUE	NA	NA	NA
Bc_ctg1_4619	B4	0.66956546	Bcer	NA	Bcer	FALSE	NA	NA	NA
Bc_ctg1_462	B4	0.223188487	Bcer	S	Bcer	FALSE	NA	NA	NA
Bc_ctg1_4620	B4	1.673913651	Bcer	S	Bcer	FALSE	NA	NA	NA
Bc_ctg1_4621	B4	0	Bcer	S	Bcer	FALSE	NA	NA	NA
Bc_ctg1_4622	B4	651.8219756	Bcer	M	Bcer	FALSE	NA	NA	NA
Bc_ctg1_4623	B4	0	Bcer	NA	Bcer	FALSE	NA	NA	NA
Bc_ctg1_4624	B4	5.021740953	Bcer	EGP	Bcer	FALSE	NA	NA	NA
Bc_ctg1_4626	B4	6.58406036	Bcer	C	Bcer	FALSE	NA	NA	NA
Bc_ctg1_4627	B4	19.08261562	Bcer	S	Bcer	FALSE	Bc_Ga0417192_01_rn32	Other	NA
Bc_ctg1_4628	B4	8.704350984	Bcer	G	Bcer	FALSE	Bc_Ga0417192_01_rn32	Ancillary biosynthesis	SMCOG1231
Bc_ctg1_4629	B4	103.6710521	Bcer	C	Bcer	FALSE	Bc_Ga0417192_01_rn32	Ancillary biosynthesis	SMCOG1006
Bc_ctg1_463	B4	3.794204275	Bcer	S	Bcer	FALSE	NA	NA	NA
Bc_ctg1_4630	B4	60.26089143	Bcer	I	Bcer	FALSE	Bc_Ga0417192_01_rn32	Ancillary biosynthesis	SMCOG1100
Bc_ctg1_4631	B4	157.2362889	Bcer	C	Bcer	FALSE	Bc_Ga0417192_01_rn32	Ancillary biosynthesis	SMCOG1017
Bc_ctg1_4632	B4	4.463769736	Bcer	E	Bcer	FALSE	Bc_Ga0417192_01_rn32	Transport	SMCOG1116
Bc_ctg1_4633	B4	223.5232695	Bcer	I	Bcer	FALSE	Bc_Ga0417192_01_rn32	Ancillary biosynthesis	SMCOG1023
Bc_ctg1_4634	B4	2.231884868	Bcer	NA	Bcer	FALSE	Bc_Ga0417192_01_rn32	Other	NA
Bc_ctg1_4635	B4	1.673913651	Bcer	S	Bcer	FALSE	Bc_Ga0417192_01_rn32	Other	SMCOG1300
Bc_ctg1_4636	B4	38.50001397	Bcer	L	Bcer	FALSE	Bc_Ga0417192_01_rn32	Other	SMCOG1230
Bc_ctg1_4637	B4	97.9797457	Bcer	L	Bcer	FALSE	Bc_Ga0417192_01_rn32	Other	SMCOG1218
Bc_ctg1_4639	B4	41.06668157	Bcer	K	Bcer	FALSE	Bc_Ga0417192_01_rn32	Regulation	SMCOG1058
Bc_ctg1_464	B4	14.3956574	Bcer	M	Bcer	FALSE	NA	NA	NA
Bc_ctg1_4640	B4	0.223188487	Bcer	NA	Bcer	FALSE	Bc_Ga0417192_01_rn32	Other	NA
Bc_ctg1_4641	B4	1140.939544	Bcer	P	Bcer	FALSE	Bc_Ga0417192_01_rn32	Transport	SMCOG1137
Bc_ctg1_4642	B4	10.15507615	Bcer	IQ	Bcer	TRUE	Bc_Ga0417192_01_rn32	Ancillary biosynthesis	SMCOG1001
Bc_ctg1_4643	B4	17.18551348	Bcer	HQ	Bcer	TRUE	Bc_Ga0417192_01_rn32	Ancillary biosynthesis	SMCOG1018
Bc_ctg1_4644	B4	34.92899818	Bcer	Q	Bcer	TRUE	Bc_Ga0417192_01_rn32	Core biosynthesis	SMCOG1002
Bc_ctg1_4645	B4	9.262322201	Bcer	Q	Bcer	TRUE	Bc_Ga0417192_01_rn32	Ancillary biosynthesis	SMCOG1027
Bc_ctg1_4646	B4	114.9420707	Bcer	Q	Bcer	TRUE	Bc_Ga0417192_01_rn32	Core biosynthesis	SMCOG1127
Bc_ctg1_4647	B4	0.781159704	Bcer	S	Bcer	FALSE	Bc_Ga0417192_01_rn32	Ancillary biosynthesis	SMCOG1009
Bc_ctg1_4648	B4	8.704350984	Bcer	P	Bcer	FALSE	Bc_Ga0417192_01_rn32	Transport	SMCOG1005
Bc_ctg1_4649	B4	5.57971217	Bcer	H	Bcer	FALSE	Bc_Ga0417192_01_rn32	Ancillary biosynthesis	SMCOG1012
Bc_ctg1_465	B4	57.02465837	Bcer	IQ	Bcer	TRUE	Bc_Ga0417192_01_rn06	Other	NA
Bc_ctg1_4650	B4	9.373916445	Bcer	H	Bcer	FALSE	Bc_Ga0417192_01_rn32	Ancillary biosynthesis	SMCOG1259
Bc_ctg1_4651	B4	20.53334078	Bcer	L	Bcer	FALSE	Bc_Ga0417192_01_rn32	Other	SMCOG1132
Bc_ctg1_4652	B4	19.75218108	Bcer	S	Bcer	FALSE	Bc_Ga0417192_01_rn32	Other	SMCOG1267
Bc_ctg1_4653	B4	30.24203996	Bcer	S	Bcer	FALSE	Bc_Ga0417192_01_rn32	Other	SMCOG1250
Bc_ctg1_4654	B4	0.111594243	Bcer	O	Bcer	FALSE	Bc_Ga0417192_01_rn32	Ancillary biosynthesis	SMCOG1075
Bc_ctg1_4655	B4	33.7014615	Bcer	E	Bcer	FALSE	Bc_Ga0417192_01_rn32	Ancillary biosynthesis	SMCOG1153
Bc_ctg1_4656	B4	4.240581249	Bcer	NA	Bcer	FALSE	Bc_Ga0417192_01_rn32	Other	SMCOG1289
Bc_ctg1_4657	B4	0	Bcer	NA	Bcer	FALSE	Bc_Ga0417192_01_rn32	Other	NA
Bc_ctg1_4658	B4	2.008696381	Bcer	G	Bcer	FALSE	Bc_Ga0417192_01_rn32	Ancillary biosynthesis	SMCOG1258
Bc_ctg1_4659	B4	36.37972335	Bcer	J	Bcer	FALSE	Bc_Ga0417192_01_rn32	Ancillary biosynthesis	SMCOG1154
Bc_ctg1_466	B4	20.97971776	Bcer	S	Bcer	FALSE	Bc_Ga0417192_01_rn06	Other	NA
Bc_ctg1_4660	B4	92.17684504	Bcer	J	Bcer	FALSE	Bc_Ga0417192_01_rn32	Other	SMCOG1162
Bc_ctg1_4661	B4	4.352175492	Bcer	V	Bcer	FALSE	Bc_Ga0417192_01_rn32	Transport	SMCOG1000
Bc_ctg1_4662	B4	27.0058069	Bcer	V	Bcer	FALSE	Bc_Ga0417192_01_rn32	Transport	SMCOG1243
Bc_ctg1_4663	B4	7.811597037	Bcer	G	Bcer	FALSE	Bc_Ga0417192_01_rn32	Transport	SMCOG1020
Bc_ctg1_4664	B4	4.128987005	Bcer	P	Bcer	FALSE	Bc_Ga0417192_01_rn32	Transport	SMCOG1118
Bc_ctg1_4665	B4	28.12174933	Bcer	P	Bcer	FALSE	Bc_Ga0417192_01_rn32	Transport	SMCOG1085
Bc_ctg1_4666	B4	4.352175492	Bcer	EH	Bcer	FALSE	NA	NA	NA
Bc_ctg1_4667	B4	0.66956546	Bcer	P	Bcer	FALSE	NA	NA	NA
Bc_ctg1_4668	B4	24.32754506	Bcer	S	Bcer	FALSE	NA	NA	NA
Bc_ctg1_4669	B4	13.83768618	Bcer	F	Bcer	FALSE	NA	NA	NA
Bc_ctg1_467	B4	2.231884868	Bcer	E	Bcer	FALSE	Bc_Ga0417192_01_rn06	Ancillary biosynthesis	SMCOG1063
Bc_ctg1_4670	B4	7.700002794	Bcer	S	Bcer	FALSE	NA	NA	NA
Bc_ctg1_4671	B4	2.678261841	Bcer	NA	Bcer	FALSE	NA	NA	NA
Bc_ctg1_4673	B4	0	Bcer	K	Bcer	FALSE	NA	NA	NA
Bc_ctg1_4674	B4	0.111594243	Bcer	S	Bcer	FALSE	NA	NA	NA

Bc_ctg1_4675	B4	3.682610032	Bcer	H	Bcer	FALSE	NA	NA	NA
Bc_ctg1_4676	B4	1.227536677	Bcer	K	Bcer	FALSE	NA	NA	NA
Bc_ctg1_4677	B4	1.339130921	Bcer	K	Bcer	FALSE	NA	NA	NA
Bc_ctg1_4678	B4	2.678261841	Bcer	S	Bcer	FALSE	NA	NA	NA
Bc_ctg1_4679	B4	14.3956574	Bcer	K	Bcer	FALSE	NA	NA	NA
Bc_ctg1_468	B4	95.63626659	Bcer	V	Bcer	FALSE	Bc_Ga0417192_01_rn06	Other	NA
Bc_ctg1_4680	B4	19.30580411	Bcer	S	Bcer	FALSE	NA	NA	NA
Bc_ctg1_4682	B4	9.485510688	Bcer	V	Bcer	FALSE	NA	NA	NA
Bc_ctg1_4683	B4	0.33478273	Bcer	K	Bcer	FALSE	NA	NA	NA
Bc_ctg1_4684	B4	3.459421545	Bcer	K	Bcer	FALSE	NA	NA	NA
Bc_ctg1_4685	B4	4.128987005	Bcer	J	Bcer	FALSE	NA	NA	NA
Bc_ctg1_4686	B4	2.566667598	Bcer	EG	Bcer	FALSE	NA	NA	NA
Bc_ctg1_4687	B4	1.785507894	Bcer	K	Bcer	FALSE	NA	NA	NA
Bc_ctg1_4688	B4	11.49420707	Bcer	NA	Bcer	FALSE	NA	NA	NA
Bc_ctg1_4689	B4	0	Bcer	NA	Bcer	FALSE	NA	NA	NA
Bc_ctg1_469	B4	5.57971217	Bcer	F	Bcer	FALSE	Bc_Ga0417192_01_rn06	Other	NA
Bc_ctg1_4690	B4	104.7869945	Bcer	H	Bcer	FALSE	NA	NA	NA
Bc_ctg1_4691	B4	0.892753947	Bcer	NA	Bcer	FALSE	NA	NA	NA
Bc_ctg1_4692	B4	82.24495738	Bcer	J	Bcer	FALSE	NA	NA	NA
Bc_ctg1_4693	B4	19.52899259	Bcer	S	Bcer	FALSE	NA	NA	NA
Bc_ctg1_4694	B4	34.81740394	Bcer	K	Bcer	FALSE	NA	NA	NA
Bc_ctg1_4695	B4	2.678261841	Bcer	S	Bcer	FALSE	NA	NA	NA
Bc_ctg1_4696	B4	5.021740953	Bcer	F	Bcer	FALSE	NA	NA	NA
Bc_ctg1_4697	B4	1.339130921	Bcer	NA	Bcer	FALSE	NA	NA	NA
Bc_ctg1_4698	B4	6.472466117	Bcer	K	Bcer	FALSE	NA	NA	NA
Bc_ctg1_4699	B4	4.017392762	Bcer	E	Bcer	FALSE	NA	NA	NA
Bc_ctg1_47	B4	2.008696381	Bcer	K	Bcer	FALSE	NA	NA	NA
Bc_ctg1_470	B4	7.811597037	Bcer	H	Bcer	FALSE	Bc_Ga0417192_01_rn06	Other	NA
Bc_ctg1_4700	B4	2.231884868	Bcer	NA	Bcer	FALSE	NA	NA	NA
Bc_ctg1_4701	B4	10.04348191	Bcer	S	Bcer	FALSE	NA	NA	NA
Bc_ctg1_4702	B4	18.74783289	Bcer	K	Bcer	FALSE	NA	NA	NA
Bc_ctg1_4703	B4	8.815945228	Bcer	K	Bcer	FALSE	NA	NA	NA
Bc_ctg1_4704	B4	1.004348191	Bcer	S	Bcer	FALSE	NA	NA	NA
Bc_ctg1_4705	B4	64.50147268	Bcer	S	Bcer	FALSE	NA	NA	NA
Bc_ctg1_4706	B4	47.3159592	Bcer	S	Bcer	FALSE	NA	NA	NA
Bc_ctg1_4707	B4	74.65654883	Bcer	V	Bcer	FALSE	NA	NA	NA
Bc_ctg1_4708	B4	84.365248	Bcer	V	Bcer	FALSE	NA	NA	NA
Bc_ctg1_4709	B4	0.66956546	Bcer	NA	Bcer	FALSE	NA	NA	NA
Bc_ctg1_471	B4	1.227536677	Bcer	S	Bcer	FALSE	Bc_Ga0417192_01_rn06	Other	NA
Bc_ctg1_4710	B4	0.892753947	Bcer	S	Bcer	FALSE	NA	NA	NA
Bc_ctg1_4711	B4	8.146379768	Bcer	NA	Bcer	FALSE	NA	NA	NA
Bc_ctg1_4712	B4	7.030437334	Bcer	NA	Bcer	FALSE	NA	NA	NA
Bc_ctg1_4713	B4	9.597104932	Bcer	E	Bcer	FALSE	NA	NA	NA
Bc_ctg1_4714	B4	7.365220064	Bcer	K	Bcer	FALSE	NA	NA	NA
Bc_ctg1_4715	B4	0.33478273	Bcer	NA	Bcer	FALSE	NA	NA	NA
Bc_ctg1_4716	B4	4.128987005	Bcer	M	Bcer	FALSE	NA	NA	NA
Bc_ctg1_4717	B4	2.120290624	Bcer	M	Bcer	FALSE	NA	NA	NA
Bc_ctg1_4719	B4	0	Bcer	G	Bcer	FALSE	NA	NA	NA
Bc_ctg1_472	B4	107.8000391	Bcer	C	Bcer	FALSE	Bc_Ga0417192_01_rn06	Ancillary biosynthesis	SMCOG1017
Bc_ctg1_4720	B4	0.33478273	Bcer	NA	Bcer	FALSE	NA	NA	NA
Bc_ctg1_4721	B4	3.571015789	Bcer	H	Bcer	FALSE	NA	NA	NA
Bc_ctg1_4722	B4	5.021740953	Bcer	K	Bcer	FALSE	NA	NA	NA
Bc_ctg1_4724	B4	22.31884868	Bcer	S	Bcer	FALSE	NA	NA	NA
Bc_ctg1_4725	B4	138.1536733	Bcer	P	Bcer	FALSE	NA	NA	NA
Bc_ctg1_4726	B4	263.0276317	Bcer	S	Bcer	FALSE	NA	NA	NA
Bc_ctg1_4727	B4	1.004348191	Bcer	T	Bcer	FALSE	NA	NA	NA
Bc_ctg1_4728	B4	0	Bcer	G	Bcer	FALSE	NA	NA	NA
Bc_ctg1_4729	B4	0.66956546	Bcer	G	Bcer	FALSE	NA	NA	NA
Bc_ctg1_473	B4	36.04494062	Bcer	IQ	Bcer	TRUE	Bc_Ga0417192_01_rn06	Ancillary biosynthesis	SMCOG1001
Bc_ctg1_4730	B4	0	Bcer	NA	Bcer	FALSE	NA	NA	NA
Bc_ctg1_4731	B4	1.004348191	Bcer	O	Bcer	FALSE	NA	NA	NA
Bc_ctg1_4732	B4	1.227536677	Bcer	G	Bcer	FALSE	NA	NA	NA

Bc_ctg1_4733	B4	2.231884868	Bcer	I	Bcer	FALSE	NA	NA	NA
Bc_ctg1_4734	B4	9.373916445	Bcer	E	Bcer	FALSE	NA	NA	NA
Bc_ctg1_4735	B4	5.133335196	Bcer	E	Bcer	FALSE	NA	NA	NA
Bc_ctg1_4736	B4	2.455073355	Bcer	K	Bcer	FALSE	NA	NA	NA
Bc_ctg1_4737	B4	0.223188487	Bcer	EGP	Bcer	FALSE	NA	NA	NA
Bc_ctg1_4738	B4	3.794204275	Bcer	S	Bcer	FALSE	NA	NA	NA
Bc_ctg1_4739	B4	1.673913651	Bcer	S	Bcer	FALSE	NA	NA	NA
Bc_ctg1_4741	B4	129.6725108	Bcer	L	Bcer	FALSE	NA	NA	NA
Bc_ctg1_4742	B4	2.231884868	Bcer	NA	Bcer	FALSE	NA	NA	NA
Bc_ctg1_4743	B4	7.700002794	Bcer	K	Bcer	FALSE	NA	NA	NA
Bc_ctg1_4744	B4	5.57971217	Bcer	EGP	Bcer	FALSE	NA	NA	NA
Bc_ctg1_4746	B4	0	Bcer	NA	Bcer	FALSE	NA	NA	NA
Bc_ctg1_4747	B4	0.892753947	Bcer	S	Bcer	FALSE	NA	NA	NA
Bc_ctg1_4748	B4	0	Bcer	NA	Bcer	FALSE	NA	NA	NA
Bc_ctg1_4749	B4	0.446376974	Bcer	NA	Bcer	FALSE	NA	NA	NA
Bc_ctg1_475	B4	5.021740953	Bcer	I	Bcer	FALSE	Bc_Ga0417192_01_rn06	Core biosynthesis	None
Bc_ctg1_4750	B4	0.66956546	Bcer	NA	Bcer	FALSE	NA	NA	NA
Bc_ctg1_4751	B4	1.450725164	Bcer	NA	Bcer	FALSE	NA	NA	NA
Bc_ctg1_4754	B4	0.111594243	Bcer	E	Bcer	FALSE	NA	NA	NA
Bc_ctg1_4755	B4	2.455073355	Bcer	N	Bcer	FALSE	NA	NA	NA
Bc_ctg1_4756	B4	0	Bcer	NA	Bcer	FALSE	NA	NA	NA
Bc_ctg1_4758	B4	0.892753947	Bcer	NA	Bcer	FALSE	NA	NA	NA
Bc_ctg1_4759	B4	2.789856085	Bcer	NA	Bcer	FALSE	NA	NA	NA
Bc_ctg1_476	B4	22.09566019	Bcer	S	Bcer	FALSE	Bc_Ga0417192_01_rn06	Other	NA
Bc_ctg1_4760	B4	0.557971217	Bcer	S	Bcer	FALSE	NA	NA	NA
Bc_ctg1_4761	B4	0.33478273	Bcer	NA	Bcer	FALSE	NA	NA	NA
Bc_ctg1_4762	B4	1.450725164	Bcer	NA	Bcer	FALSE	NA	NA	NA
Bc_ctg1_4763	B4	4.463769736	Bcer	NA	Bcer	FALSE	NA	NA	NA
Bc_ctg1_4765	B4	1.785507894	Bcer	Q	Bcer	TRUE	NA	NA	NA
Bc_ctg1_4766	B4	6.24927763	Bcer	S	Bcer	FALSE	NA	NA	NA
Bc_ctg1_4767	B4	8.034785524	Bcer	Q	Bcer	TRUE	NA	NA	NA
Bc_ctg1_4768	B4	1.450725164	Bcer	GH	Bcer	FALSE	NA	NA	NA
Bc_ctg1_4769	B4	244.5029873	Bcer	K	Bcer	FALSE	NA	NA	NA
Bc_ctg1_477	B4	46.75798798	Bcer	S	Bcer	FALSE	Bc_Ga0417192_01_rn06	Transport	SMCOG1166
Bc_ctg1_4770	B4	225.1971832	Bcer	G	Bcer	FALSE	Bc_Ga0417192_01_rn33	Ancillary biosynthesis	SMCOG1101
Bc_ctg1_4771	B4	138.7116445	Bcer	S	Bcer	FALSE	Bc_Ga0417192_01_rn33	Other	NA
Bc_ctg1_4773	B4	2.566667598	Bcer	NA	Bcer	FALSE	Bc_Ga0417192_01_rn33	Other	NA
Bc_ctg1_4774	B4	1.115942434	Bcer	S	Bcer	FALSE	Bc_Ga0417192_01_rn33	Other	NA
Bc_ctg1_4775	B4	865.8597345	Bcer	E	Bcer	FALSE	Bc_Ga0417192_01_rn33	Ancillary biosynthesis	SMCOG1247
Bc_ctg1_4776	B4	0	Bcer	S	Bcer	FALSE	Bc_Ga0417192_01_rn33	Other	NA
Bc_ctg1_4777	B4	1.227536677	Bcer	NA	Bcer	FALSE	Bc_Ga0417192_01_rn33	Other	NA
Bc_ctg1_4778	B4	7.365220064	Bcer	E	Bcer	FALSE	Bc_Ga0417192_01_rn33	Other	NA
Bc_ctg1_4779	B4	0.223188487	Bcer	KT	Bcer	FALSE	Bc_Ga0417192_01_rn33	Other	NA
Bc_ctg1_478	B4	0.223188487	Bcer	S	Bcer	FALSE	Bc_Ga0417192_01_rn06	Other	NA
Bc_ctg1_4781	B4	4.463769736	Bcer	K	Bcer	FALSE	Bc_Ga0417192_01_rn33	Other	NA
Bc_ctg1_4782	B4	0.111594243	Bcer	K	Bcer	FALSE	Bc_Ga0417192_01_rn33	Other	NA
Bc_ctg1_4783	B4	3.124638815	Bcer	NA	Bcer	FALSE	Bc_Ga0417192_01_rn33	Other	NA
Bc_ctg1_4784	B4	2.008696381	Bcer	S	Bcer	FALSE	Bc_Ga0417192_01_rn33	Other	NA
Bc_ctg1_4785	B4	14.3956574	Bcer	S	Bcer	FALSE	Bc_Ga0417192_01_rn33	Ancillary biosynthesis	SMCOG1156
Bc_ctg1_4786	B4	1.004348191	Bcer	M	Bcer	FALSE	Bc_Ga0417192_01_rn33	Other	NA
Bc_ctg1_4787	B4	4.463769736	Bcer	I	Bcer	FALSE	Bc_Ga0417192_01_rn33	Ancillary biosynthesis	SMCOG1088
Bc_ctg1_4788	B4	24.66232779	Bcer	E	Bcer	FALSE	Bc_Ga0417192_01_rn33	Other	NA
Bc_ctg1_4789	B4	2.008696381	Bcer	EGP	Bcer	FALSE	Bc_Ga0417192_01_rn33	Transport	SMCOG1020
Bc_ctg1_479	B4	3.905798519	Bcer	NA	Bcer	FALSE	Bc_Ga0417192_01_rn06	Other	NA
Bc_ctg1_4790	B4	9.820293418	Bcer	Q	Bcer	TRUE	Bc_Ga0417192_01_rn33	Core biosynthesis	SMCOG1002
Bc_ctg1_4791	B4	10.60145312	Bcer	Q	Bcer	TRUE	Bc_Ga0417192_01_rn33	Core biosynthesis	SMCOG1127
Bc_ctg1_4792	B4	88.27104652	Bcer	Q	Bcer	TRUE	Bc_Ga0417192_01_rn33	Core biosynthesis	SMCOG1127
Bc_ctg1_4793	B4	1.785507894	Bcer	H	Bcer	FALSE	Bc_Ga0417192_01_rn33	Ancillary biosynthesis	SMCOG1012
Bc_ctg1_4794	B4	2.678261841	Bcer	Q	Bcer	TRUE	Bc_Ga0417192_01_rn33	Ancillary biosynthesis	SMCOG1004
Bc_ctg1_4795	B4	5.691306413	Bcer	P	Bcer	FALSE	Bc_Ga0417192_01_rn33	Other	NA
Bc_ctg1_4796	B4	3.347827302	Bcer	E	Bcer	FALSE	Bc_Ga0417192_01_rn33	Ancillary biosynthesis	SMCOG1153
Bc_ctg1_4798	B4	3.013044572	Bcer	E	Bcer	FALSE	Bc_Ga0417192_01_rn33	Other	NA

Bc_ctg1_4799	B4	21.53768897	Bcer	T	Bcer	FALSE	Bc_Ga0417192_01_rn33	Other	NA
Bc_ctg1_48	B4	2.455073355	Bcer	IQ	Bcer	TRUE	NA	NA	NA
Bc_ctg1_480	B4	2.789856085	Bcer	NA	Bcer	FALSE	Bc_Ga0417192_01_rn06	Other	NA
Bc_ctg1_4800	B4	20.64493503	Bcer	T	Bcer	FALSE	Bc_Ga0417192_01_rn33	Regulation	SMCOG1008
Bc_ctg1_4801	B4	0.33478273	Bcer	EG	Bcer	FALSE	Bc_Ga0417192_01_rn33	Other	NA
Bc_ctg1_4802	B4	0.557971217	Bcer	S	Bcer	FALSE	Bc_Ga0417192_01_rn33	Ancillary biosynthesis	SMCOG1038
Bc_ctg1_4803	B4	0.223188487	Bcer	S	Bcer	FALSE	Bc_Ga0417192_01_rn33	Other	NA
Bc_ctg1_4804	B4	0.223188487	Bcer	E	Bcer	FALSE	Bc_Ga0417192_01_rn33	Other	NA
Bc_ctg1_4805	B4	2.120290624	Bcer	E	Bcer	FALSE	Bc_Ga0417192_01_rn33	Ancillary biosynthesis	SMCOG1038
Bc_ctg1_4806	B4	3.013044572	Bcer	L	Bcer	FALSE	Bc_Ga0417192_01_rn33	Other	NA
Bc_ctg1_4807	B4	7.811597037	Bcer	K	Bcer	FALSE	NA	NA	NA
Bc_ctg1_4808	B4	856.0394411	Bcer	S	Bcer	FALSE	NA	NA	NA
Bc_ctg1_4809	B4	905.8104736	Bcer	E	Bcer	FALSE	NA	NA	NA
Bc_ctg1_481	B4	30.68841693	Bcer	D	Bcer	FALSE	Bc_Ga0417192_01_rn06	Other	SMCOG1157
Bc_ctg1_4810	B4	1005.687321	Bcer	E	Bcer	FALSE	NA	NA	NA
Bc_ctg1_4811	B4	76.55365097	Bcer	E	Bcer	FALSE	NA	NA	NA
Bc_ctg1_4812	B4	55.57393321	Bcer	T	Bcer	FALSE	NA	NA	NA
Bc_ctg1_4813	B4	1.227536677	Bcer	P	Bcer	FALSE	NA	NA	NA
Bc_ctg1_4814	B4	0.223188487	Bcer	E	Bcer	FALSE	NA	NA	NA
Bc_ctg1_4815	B4	1.450725164	Bcer	S	Bcer	FALSE	NA	NA	NA
Bc_ctg1_4817	B4	2.901450328	Bcer	M	Bcer	FALSE	NA	NA	NA
Bc_ctg1_4818	B4	38.16523124	Bcer	J	Bcer	FALSE	NA	NA	NA
Bc_ctg1_4819	B4	0.33478273	Bcer	NA	Bcer	FALSE	NA	NA	NA
Bc_ctg1_482	B4	1.115942434	Bcer	H	Bcer	FALSE	Bc_Ga0417192_01_rn06	Other	NA
Bc_ctg1_4820	B4	29.12609753	Bcer	M	Bcer	FALSE	NA	NA	NA
Bc_ctg1_4821	B4	2.901450328	Bcer	K	Bcer	FALSE	NA	NA	NA
Bc_ctg1_4822	B4	1.339130921	Bcer	NA	Bcer	FALSE	NA	NA	NA
Bc_ctg1_4823	B4	2.789856085	Bcer	G	Bcer	FALSE	NA	NA	NA
Bc_ctg1_4824	B4	1.897102138	Bcer	K	Bcer	FALSE	NA	NA	NA
Bc_ctg1_4825	B4	15.73478832	Bcer	E	Bcer	FALSE	NA	NA	NA
Bc_ctg1_4826	B4	18.63623865	Bcer	T	Bcer	FALSE	NA	NA	NA
Bc_ctg1_4827	B4	6.807248847	Bcer	T	Bcer	FALSE	NA	NA	NA
Bc_ctg1_4828	B4	2.120290624	Bcer	O	Bcer	FALSE	NA	NA	NA
Bc_ctg1_4829	B4	1.339130921	Bcer	S	Bcer	FALSE	NA	NA	NA
Bc_ctg1_483	B4	4.686958222	Bcer	H	Bcer	FALSE	Bc_Ga0417192_01_rn06	Other	NA
Bc_ctg1_4830	B4	5.244929439	Bcer	S	Bcer	FALSE	NA	NA	NA
Bc_ctg1_4834	B4	7.588408551	Bcer	KT	Bcer	FALSE	NA	NA	NA
Bc_ctg1_4835	B4	6.695654603	Bcer	NA	Bcer	FALSE	NA	NA	NA
Bc_ctg1_4836	B4	1.115942434	Bcer	NA	Bcer	FALSE	NA	NA	NA
Bc_ctg1_4837	B4	65.72900936	Bcer	NA	Bcer	FALSE	NA	NA	NA
Bc_ctg1_4838	B4	0.223188487	Bcer	NA	Bcer	FALSE	NA	NA	NA
Bc_ctg1_4839	B4	2.566667598	Bcer	NA	Bcer	FALSE	NA	NA	NA
Bc_ctg1_484	B4	26.67102417	Bcer	H	Bcer	FALSE	Bc_Ga0417192_01_rn06	Other	NA
Bc_ctg1_4840	B4	1.897102138	Bcer	S	Bcer	FALSE	NA	NA	NA
Bc_ctg1_4841	B4	0	Bcer	NA	Bcer	FALSE	NA	NA	NA
Bc_ctg1_4842	B4	27.22899539	Bcer	E	Bcer	FALSE	NA	NA	NA
Bc_ctg1_4843	B4	32.25073634	Bcer	E	Bcer	FALSE	NA	NA	NA
Bc_ctg1_4844	B4	52.56088864	Bcer	E	Bcer	FALSE	NA	NA	NA
Bc_ctg1_4845	B4	0	Bcer	NA	Bcer	FALSE	NA	NA	NA
Bc_ctg1_4847	B4	0	Bcer	S	Bcer	FALSE	NA	NA	NA
Bc_ctg1_4848	B4	7.588408551	Bcer	L	Bcer	FALSE	NA	NA	NA
Bc_ctg1_4849	B4	1.897102138	Bcer	S	Bcer	FALSE	NA	NA	NA
Bc_ctg1_485	B4	11.8289898	Bcer	H	Bcer	FALSE	Bc_Ga0417192_01_rn06	Ancillary biosynthesis	None
Bc_ctg1_4850	B4	12.38696102	Bcer	P	Bcer	FALSE	NA	NA	NA
Bc_ctg1_4851	B4	7.476814307	Bcer	D	Bcer	FALSE	NA	NA	NA
Bc_ctg1_4852	B4	41.51305854	Bcer	P	Bcer	FALSE	NA	NA	NA
Bc_ctg1_4853	B4	60.37248567	Bcer	G	Bcer	FALSE	NA	NA	NA
Bc_ctg1_4855	B4	0.446376974	Bcer	M	Bcer	FALSE	NA	NA	NA
Bc_ctg1_4856	B4	0	Bcer	K	Bcer	FALSE	NA	NA	NA
Bc_ctg1_4857	B4	0.223188487	Bcer	NA	Bcer	FALSE	NA	NA	NA
Bc_ctg1_4858	B4	52.89567137	Bcer	M	Bcer	FALSE	NA	NA	NA
Bc_ctg1_4859	B4	1.339130921	Bcer	M	Bcer	FALSE	NA	NA	NA

Bc_ctg1_486	B4	9.820293418	Bcer	P	Bcer	FALSE	Bc_Ga0417192_01_rn06	Other	NA
Bc_ctg1_4860	B4	0.223188487	Bcer	V	Bcer	FALSE	NA	NA	NA
Bc_ctg1_4861	B4	0.66956546	Bcer	J	Bcer	FALSE	NA	NA	NA
Bc_ctg1_4862	B4	4.240581249	Bcer	S	Bcer	FALSE	Bc_Ga0417192_01_rn34	Other	NA
Bc_ctg1_4863	B4	0	Bcer	NA	Bcer	FALSE	Bc_Ga0417192_01_rn34	Other	NA
Bc_ctg1_4864	B4	7.811597037	Bcer	V	Bcer	FALSE	Bc_Ga0417192_01_rn34	Transport	SMCOG1000
Bc_ctg1_4865	B4	23.43479111	Bcer	V	Bcer	FALSE	Bc_Ga0417192_01_rn34	Transport	SMCOG1065
Bc_ctg1_4866	B4	17.96667319	Bcer	K	Bcer	FALSE	Bc_Ga0417192_01_rn34	Regulation	SMCOG1057
Bc_ctg1_4869	B4	0	Bcer	NA	Bcer	FALSE	Bc_Ga0417192_01_rn34	Other	NA
Bc_ctg1_487	B4	83.47249406	Bcer	H	Bcer	FALSE	Bc_Ga0417192_01_rn06	Ancillary biosynthesis	None
Bc_ctg1_4870	B4	5.021740953	Bcer	S	Bcer	FALSE	Bc_Ga0417192_01_rn34	Other	NA
Bc_ctg1_4871	B4	23.88116809	Bcer	I	Bcer	FALSE	Bc_Ga0417192_01_rn34	Ancillary biosynthesis	SMCOG1006
Bc_ctg1_4872	B4	27.89856085	Bcer	I	Bcer	FALSE	Bc_Ga0417192_01_rn34	Ancillary biosynthesis	SMCOG1090
Bc_ctg1_4873	B4	0.111594243	Bcer	I	Bcer	FALSE	Bc_Ga0417192_01_rn34	Other	NA
Bc_ctg1_4874	B4	14.50725164	Bcer	E	Bcer	FALSE	Bc_Ga0417192_01_rn34	Core biosynthesis	SMCOG1271
Bc_ctg1_4875	B4	8.481162498	Bcer	I	Bcer	FALSE	Bc_Ga0417192_01_rn34	Ancillary biosynthesis	SMCOG1023
Bc_ctg1_4876	B4	26.2246472	Bcer	I	Bcer	FALSE	Bc_Ga0417192_01_rn34	Ancillary biosynthesis	SMCOG1059
Bc_ctg1_4877	B4	36.60291183	Bcer	I	Bcer	FALSE	Bc_Ga0417192_01_rn34	Core biosynthesis	SMCOG1002
Bc_ctg1_4878	B4	0.557971217	Bcer	NA	Bcer	FALSE	Bc_Ga0417192_01_rn34	Other	NA
Bc_ctg1_4879	B4	0	Bcer	NA	Bcer	FALSE	Bc_Ga0417192_01_rn34	Other	NA
Bc_ctg1_488	B4	54.7927735	Bcer	C	Bcer	FALSE	Bc_Ga0417192_01_rn06	Other	NA
Bc_ctg1_4880	B4	1.562319407	Bcer	J	Bcer	FALSE	Bc_Ga0417192_01_rn34	Ancillary biosynthesis	SMCOG1111
Bc_ctg1_4881	B4	0.111594243	Bcer	NA	Bcer	FALSE	Bc_Ga0417192_01_rn34	Other	NA
Bc_ctg1_4884	B4	4.017392762	Bcer	M	Bcer	FALSE	Bc_Ga0417192_01_rn34	Other	NA
Bc_ctg1_4885	B4	19.86377532	Bcer	T	Bcer	FALSE	Bc_Ga0417192_01_rn34	Regulation	SMCOG1003
Bc_ctg1_4886	B4	7.030437334	Bcer	K	Bcer	FALSE	Bc_Ga0417192_01_rn34	Regulation	SMCOG1008
Bc_ctg1_4887	B4	1.450725164	Bcer	H	Bcer	FALSE	Bc_Ga0417192_01_rn34	Other	NA
Bc_ctg1_4888	B4	1.339130921	Bcer	H	Bcer	FALSE	Bc_Ga0417192_01_rn34	Other	NA
Bc_ctg1_4889	B4	8.034785524	Bcer	S	Bcer	FALSE	Bc_Ga0417192_01_rn34	Other	NA
Bc_ctg1_489	B4	19.52899259	Bcer	S	Bcer	FALSE	Bc_Ga0417192_01_rn06	Other	NA
Bc_ctg1_4890	B4	12.05217829	Bcer	F	Bcer	FALSE	Bc_Ga0417192_01_rn34	Other	NA
Bc_ctg1_4891	B4	22.31884868	Bcer	K	Bcer	FALSE	Bc_Ga0417192_01_rn34	Other	NA
Bc_ctg1_4892	B4	22.98841414	Bcer	NA	Bcer	FALSE	NA	NA	NA
Bc_ctg1_4893	B4	23.88116809	Bcer	E	Bcer	FALSE	NA	NA	NA
Bc_ctg1_4894	B4	0.223188487	Bcer	K	Bcer	FALSE	NA	NA	NA
Bc_ctg1_4895	B4	0.223188487	Bcer	M	Bcer	FALSE	NA	NA	NA
Bc_ctg1_4896	B4	0	Bcer	M	Bcer	FALSE	NA	NA	NA
Bc_ctg1_4897	B4	0.33478273	Bcer	M	Bcer	FALSE	NA	NA	NA
Bc_ctg1_4898	B4	9.485510688	Bcer	S	Bcer	FALSE	NA	NA	NA
Bc_ctg1_4899	B4	7.700002794	Bcer	S	Bcer	FALSE	NA	NA	NA
Bc_ctg1_49	B4	1.562319407	Bcer	S	Bcer	FALSE	NA	NA	NA
Bc_ctg1_490	B4	26.00145871	Bcer	S	Bcer	FALSE	NA	NA	NA
Bc_ctg1_4900	B4	98.64931116	Bcer	L	Bcer	FALSE	NA	NA	NA
Bc_ctg1_4901	B4	29.01450328	Bcer	K	Bcer	FALSE	NA	NA	NA
Bc_ctg1_4902	B4	1.785507894	Bcer	IQ	Bcer	TRUE	NA	NA	NA
Bc_ctg1_4903	B4	0.111594243	Bcer	K	Bcer	FALSE	NA	NA	NA
Bc_ctg1_4904	B4	8.815945228	Bcer	Q	Bcer	TRUE	NA	NA	NA
Bc_ctg1_4906	B4	2.231884868	Bcer	E	Bcer	FALSE	NA	NA	NA
Bc_ctg1_4907	B4	3.013044572	Bcer	M	Bcer	FALSE	NA	NA	NA
Bc_ctg1_4908	B4	4.240581249	Bcer	S	Bcer	FALSE	NA	NA	NA
Bc_ctg1_4909	B4	3.459421545	Bcer	K	Bcer	FALSE	Bc_Ga0417192_01_rn35	Other	NA
Bc_ctg1_491	B4	397.2755065	Bcer	C	Bcer	FALSE	NA	NA	NA
Bc_ctg1_4910	B4	47.3159592	Bcer	E	Bcer	FALSE	Bc_Ga0417192_01_rn35	Other	NA
Bc_ctg1_4911	B4	0	Bcer	S	Bcer	FALSE	Bc_Ga0417192_01_rn35	Other	NA
Bc_ctg1_4912	B4	5.691306413	Bcer	K	Bcer	FALSE	Bc_Ga0417192_01_rn35	Ancillary biosynthesis	SMCOG1019
Bc_ctg1_4913	B4	1.450725164	Bcer	NA	Bcer	FALSE	Bc_Ga0417192_01_rn35	Other	NA
Bc_ctg1_4914	B4	0.223188487	Bcer	Q	Bcer	TRUE	Bc_Ga0417192_01_rn35	Other	NA
Bc_ctg1_4915	B4	0	Bcer	S	Bcer	FALSE	Bc_Ga0417192_01_rn35	Other	NA
Bc_ctg1_4916	B4	0	Bcer	NA	Bcer	FALSE	Bc_Ga0417192_01_rn35	Other	NA
Bc_ctg1_4917	B4	2.343479111	Bcer	E	Bcer	FALSE	Bc_Ga0417192_01_rn35	Ancillary biosynthesis	SMCOG1040
Bc_ctg1_4918	B4	7.365220064	Bcer	GM	Bcer	FALSE	Bc_Ga0417192_01_rn35	Core biosynthesis	SMCOG1010
Bc_ctg1_4919	B4	1.339130921	Bcer	S	Bcer	FALSE	Bc_Ga0417192_01_rn35	Other	NA

Bc_ctg1_492	B4	0	Bcer	EG	Bcer	FALSE	NA	NA	NA
Bc_ctg1_4920	B4	3.905798519	Bcer	Q	Bcer	TRUE	Bc_Ga0417192_01_rn35	Ancillary biosynthesis	SMCOG1248
Bc_ctg1_4921	B4	17.96667319	Bcer	S	Bcer	FALSE	Bc_Ga0417192_01_rn35	Other	NA
Bc_ctg1_4922	B4	7.030437334	Bcer	Q	Bcer	TRUE	Bc_Ga0417192_01_rn35	Other	NA
Bc_ctg1_4923	B4	9.039133715	Bcer	G	Bcer	FALSE	Bc_Ga0417192_01_rn35	Other	NA
Bc_ctg1_4924	B4	0	Bcer	NA	Bcer	FALSE	Bc_Ga0417192_01_rn35	Other	NA
Bc_ctg1_4925	B4	4.128987005	Bcer	J	Bcer	FALSE	Bc_Ga0417192_01_rn35	Other	NA
Bc_ctg1_4926	B4	0.111594243	Bcer	M	Bcer	FALSE	Bc_Ga0417192_01_rn35	Other	NA
Bc_ctg1_4927	B4	11.49420707	Bcer	C	Bcer	FALSE	Bc_Ga0417192_01_rn35	Other	NA
Bc_ctg1_4928	B4	10.04348191	Bcer	T	Bcer	FALSE	Bc_Ga0417192_01_rn35	Regulation	SMCOG1008
Bc_ctg1_4929	B4	21.98406595	Bcer	T	Bcer	FALSE	Bc_Ga0417192_01_rn35	Regulation	SMCOG1003
Bc_ctg1_493	B4	0	Bcer	E	Bcer	FALSE	NA	NA	NA
Bc_ctg1_4930	B4	0	Bcer	NA	Bcer	FALSE	Bc_Ga0417192_01_rn35	Other	NA
Bc_ctg1_4931	B4	4.686958222	Bcer	NA	Bcer	FALSE	NA	NA	NA
Bc_ctg1_4932	B4	16.962325	Bcer	S	Bcer	FALSE	NA	NA	NA
Bc_ctg1_4933	B4	7.030437334	Bcer	J	Bcer	FALSE	NA	NA	NA
Bc_ctg1_4934	B4	0	Bcer	NA	Bcer	FALSE	NA	NA	NA
Bc_ctg1_4935	B4	2.901450328	Bcer	V	Bcer	FALSE	NA	NA	NA
Bc_ctg1_4936	B4	6.91884309	Bcer	V	Bcer	FALSE	NA	NA	NA
Bc_ctg1_4937	B4	1.785507894	Bcer	S	Bcer	FALSE	NA	NA	NA
Bc_ctg1_4938	B4	99.20728237	Bcer	NA	Bcer	FALSE	NA	NA	NA
Bc_ctg1_4939	B4	0.111594243	Bcer	D	Bcer	FALSE	NA	NA	NA
Bc_ctg1_494	B4	1.339130921	Bcer	S	Bcer	FALSE	NA	NA	NA
Bc_ctg1_4940	B4	3.347827302	Bcer	E	Bcer	FALSE	NA	NA	NA
Bc_ctg1_4941	B4	12.27536677	Bcer	K	Bcer	FALSE	Bc_Ga0417192_01_rn36	Ancillary biosynthesis	SMCOG1019
Bc_ctg1_4942	B4	8.034785524	Bcer	F	Bcer	FALSE	Bc_Ga0417192_01_rn36	Ancillary biosynthesis	None
Bc_ctg1_4943	B4	2.678261841	Bcer	K	Bcer	FALSE	Bc_Ga0417192_01_rn36	Other	NA
Bc_ctg1_4944	B4	53.11885985	Bcer	S	Bcer	FALSE	Bc_Ga0417192_01_rn36	Other	NA
Bc_ctg1_4945	B4	0.446376974	Bcer	NA	Bcer	FALSE	Bc_Ga0417192_01_rn36	Core biosynthesis	None
Bc_ctg1_4946	B4	4.910146709	Bcer	NA	Bcer	FALSE	Bc_Ga0417192_01_rn36	Other	NA
Bc_ctg1_4947	B4	1.004348191	Bcer	S	Bcer	FALSE	Bc_Ga0417192_01_rn36	Other	NA
Bc_ctg1_4948	B4	19.41739835	Bcer	L	Bcer	FALSE	Bc_Ga0417192_01_rn36	Other	SMCOG1077
Bc_ctg1_4949	B4	2.008696381	Bcer	NA	Bcer	FALSE	Bc_Ga0417192_01_rn36	Other	NA
Bc_ctg1_495	B4	128.4449741	Bcer	E	Bcer	FALSE	NA	NA	NA
Bc_ctg1_4950	B4	4.017392762	Bcer	NA	Bcer	FALSE	NA	NA	NA
Bc_ctg1_4951	B4	0.111594243	Bcer	NA	Bcer	FALSE	NA	NA	NA
Bc_ctg1_4953	B4	13.16812072	Bcer	K	Bcer	FALSE	NA	NA	NA
Bc_ctg1_4954	B4	0	Bcer	K	Bcer	FALSE	NA	NA	NA
Bc_ctg1_4955	B4	0.33478273	Bcer	NA	Bcer	FALSE	NA	NA	NA
Bc_ctg1_4956	B4	0	Bcer	NA	Bcer	FALSE	NA	NA	NA
Bc_ctg1_4957	B4	0.892753947	Bcer	L	Bcer	FALSE	NA	NA	NA
Bc_ctg1_4958	B4	0.446376974	Bcer	K	Bcer	FALSE	NA	NA	NA
Bc_ctg1_4959	B4	0	Bcer	NA	Bcer	FALSE	NA	NA	NA
Bc_ctg1_4961	B4	0	Bcer	NA	Bcer	FALSE	NA	NA	NA
Bc_ctg1_4962	B4	0.33478273	Bcer	S	Bcer	FALSE	NA	NA	NA
Bc_ctg1_4963	B4	5.691306413	Bcer	NA	Bcer	FALSE	NA	NA	NA
Bc_ctg1_4964	B4	0	Bcer	NA	Bcer	FALSE	NA	NA	NA
Bc_ctg1_4965	B4	3.571015789	Bcer	K	Bcer	FALSE	NA	NA	NA
Bc_ctg1_4966	B4	4.017392762	Bcer	NA	Bcer	FALSE	NA	NA	NA
Bc_ctg1_4967	B4	0	Bcer	NA	Bcer	FALSE	NA	NA	NA
Bc_ctg1_497	B4	3.905798519	Bcer	L	Bcer	FALSE	NA	NA	NA
Bc_ctg1_4970	B4	4.686958222	Bcer	S	Bcer	FALSE	NA	NA	NA
Bc_ctg1_4971	B4	3.236233058	Bcer	K	Bcer	FALSE	NA	NA	NA
Bc_ctg1_4972	B4	268.6073438	Bcer	L	Bcer	FALSE	NA	NA	NA
Bc_ctg1_4973	B4	68.29567696	Bcer	NA	Bcer	FALSE	NA	NA	NA
Bc_ctg1_4974	B4	0	Bcer	NA	Bcer	FALSE	NA	NA	NA
Bc_ctg1_4976	B4	0.223188487	Bcer	NA	Bcer	FALSE	NA	NA	NA
Bc_ctg1_4977	B4	0	Bcer	S	Bcer	FALSE	NA	NA	NA
Bc_ctg1_4978	B4	0	Bcer	S	Bcer	FALSE	NA	NA	NA
Bc_ctg1_4979	B4	0.111594243	Bcer	S	Bcer	FALSE	NA	NA	NA
Bc_ctg1_498	B4	1.450725164	Bcer	S	Bcer	FALSE	NA	NA	NA
Bc_ctg1_4980	B4	0.223188487	Bcer	OU	Bcer	FALSE	NA	NA	NA

Bc_ctg1_4981	B4	1.562319407	Bcer	S	Bcer	FALSE	NA	NA	NA
Bc_ctg1_4982	B4	0	Bcer	NA	Bcer	FALSE	NA	NA	NA
Bc_ctg1_4983	B4	0.223188487	Bcer	NA	Bcer	FALSE	NA	NA	NA
Bc_ctg1_4984	B4	0	Bcer	NA	Bcer	FALSE	NA	NA	NA
Bc_ctg1_4985	B4	0	Bcer	NA	Bcer	FALSE	NA	NA	NA
Bc_ctg1_4986	B4	0.111594243	Bcer	NA	Bcer	FALSE	NA	NA	NA
Bc_ctg1_4987	B4	0	Bcer	NA	Bcer	FALSE	NA	NA	NA
Bc_ctg1_4988	B4	0	Bcer	E	Bcer	FALSE	NA	NA	NA
Bc_ctg1_4989	B4	0	Bcer	S	Bcer	FALSE	NA	NA	NA
Bc_ctg1_499	B4	4.463769736	Bcer	E	Bcer	FALSE	NA	NA	NA
Bc_ctg1_4990	B4	0.223188487	Bcer	M	Bcer	FALSE	NA	NA	NA
Bc_ctg1_4993	B4	0	Bcer	S	Bcer	FALSE	NA	NA	NA
Bc_ctg1_4994	B4	1.450725164	Bcer	Q	Bcer	TRUE	NA	NA	NA
Bc_ctg1_4995	B4	4.575363979	Bcer	NA	Bcer	FALSE	NA	NA	NA
Bc_ctg1_4996	B4	46.31161101	Bcer	T	Bcer	FALSE	NA	NA	NA
Bc_ctg1_4997	B4	2.343479111	Bcer	S	Bcer	FALSE	NA	NA	NA
Bc_ctg1_4998	B4	53.2304541	Bcer	S	Bcer	FALSE	NA	NA	NA
Bc_ctg1_4999	B4	7.365220064	Bcer	S	Bcer	FALSE	NA	NA	NA
Bc_ctg1_50	B4	0	Bcer	S	Bcer	FALSE	NA	NA	NA
Bc_ctg1_5000	B4	4.463769736	Bcer	NA	Bcer	FALSE	NA	NA	NA
Bc_ctg1_5001	B4	0.111594243	Bcer	NA	Bcer	FALSE	NA	NA	NA
Bc_ctg1_5002	B4	14.06087467	Bcer	S	Bcer	FALSE	NA	NA	NA
Bc_ctg1_5003	B4	0.223188487	Bcer	NA	Bcer	FALSE	NA	NA	NA
Bc_ctg1_5004	B4	353.4189688	Bcer	C	Bcer	FALSE	NA	NA	NA
Bc_ctg1_5005	B4	19.19420986	Bcer	EGP	Bcer	FALSE	NA	NA	NA
Bc_ctg1_5006	B4	8.704350984	Bcer	S	Bcer	FALSE	NA	NA	NA
Bc_ctg1_5007	B4	0.111594243	Bcer	S	Bcer	FALSE	NA	NA	NA
Bc_ctg1_5008	B4	2.455073355	Bcer	K	Bcer	FALSE	NA	NA	NA
Bc_ctg1_5009	B4	9.150727958	Bcer	Q	Bcer	TRUE	NA	NA	NA
Bc_ctg1_501	B4	0	Bcer	NA	Bcer	FALSE	NA	NA	NA
Bc_ctg1_5010	B4	0.781159704	Bcer	C	Bcer	FALSE	NA	NA	NA
Bc_ctg1_5011	B4	32.25073634	Bcer	S	Bcer	FALSE	NA	NA	NA
Bc_ctg1_5012	B4	37.49566578	Bcer	T	Bcer	FALSE	NA	NA	NA
Bc_ctg1_5013	B4	85.92756741	Bcer	M	Bcer	FALSE	NA	NA	NA
Bc_ctg1_5014	B4	3.013044572	Bcer	U	Bcer	FALSE	NA	NA	NA
Bc_ctg1_5015	B4	2.008696381	Bcer	CG	Bcer	FALSE	NA	NA	NA
Bc_ctg1_5016	B4	1.227536677	Bcer	M	Bcer	FALSE	NA	NA	NA
Bc_ctg1_5017	B4	1.562319407	Bcer	S	Bcer	FALSE	NA	NA	NA
Bc_ctg1_5018	B4	2.455073355	Bcer	P	Bcer	FALSE	NA	NA	NA
Bc_ctg1_5019	B4	1.897102138	Bcer	P	Bcer	FALSE	NA	NA	NA
Bc_ctg1_502	B4	0	Bcer	Q	Bcer	TRUE	NA	NA	NA
Bc_ctg1_5021	B4	31.35798239	Bcer	T	Bcer	FALSE	NA	NA	NA
Bc_ctg1_5023	B4	3.682610032	Bcer	NA	Bcer	FALSE	NA	NA	NA
Bc_ctg1_5024	B4	0.111594243	Bcer	NA	Bcer	FALSE	NA	NA	NA
Bc_ctg1_5025	B4	0.66956546	Bcer	NA	Bcer	FALSE	NA	NA	NA
Bc_ctg1_5026	B4	0	Bcer	NA	Bcer	FALSE	NA	NA	NA
Bc_ctg1_5028	B4	0.111594243	Bcer	F	Bcer	FALSE	NA	NA	NA
Bc_ctg1_503	B4	5.9144949	Bcer	E	Bcer	FALSE	NA	NA	NA
Bc_ctg1_5033	B4	2.120290624	Bcer	D	Bcer	FALSE	NA	NA	NA
Bc_ctg1_5034	B4	0.557971217	Bcer	NA	Bcer	FALSE	NA	NA	NA
Bc_ctg1_5035	B4	0	Bcer	NA	Bcer	FALSE	NA	NA	NA
Bc_ctg1_5036	B4	11.49420707	Bcer	F	Bcer	FALSE	NA	NA	NA
Bc_ctg1_5037	B4	0.111594243	Bcer	NA	Bcer	FALSE	NA	NA	NA
Bc_ctg1_5038	B4	11.38261283	Bcer	KLT	Bcer	FALSE	NA	NA	NA
Bc_ctg1_5039	B4	0.892753947	Bcer	S	Bcer	FALSE	NA	NA	NA
Bc_ctg1_504	B4	0.33478273	Bcer	L	Bcer	FALSE	NA	NA	NA
Bc_ctg1_5040	B4	6.91884309	Bcer	C	Bcer	FALSE	NA	NA	NA
Bc_ctg1_5041	B4	106.6840967	Bcer	I	Bcer	FALSE	NA	NA	NA
Bc_ctg1_5042	B4	10.26667039	Bcer	M	Bcer	FALSE	NA	NA	NA
Bc_ctg1_5043	B4	4.686958222	Bcer	EGP	Bcer	FALSE	NA	NA	NA
Bc_ctg1_5044	B4	7.365220064	Bcer	V	Bcer	FALSE	NA	NA	NA
Bc_ctg1_5045	B4	3.794204275	Bcer	V	Bcer	FALSE	NA	NA	NA

Bc_ctg1_5046	B4	0.557971217	Bcer	S	Bcer	FALSE	NA	NA	NA
Bc_ctg1_5047	B4	1.562319407	Bcer	S	Bcer	FALSE	NA	NA	NA
Bc_ctg1_5048	B4	57.3594411	Bcer	S	Bcer	FALSE	NA	NA	NA
Bc_ctg1_5049	B4	7.030437334	Bcer	K	Bcer	FALSE	NA	NA	NA
Bc_ctg1_505	B4	0	Bcer	L	Bcer	FALSE	NA	NA	NA
Bc_ctg1_5050	B4	7.811597037	Bcer	NA	Bcer	FALSE	NA	NA	NA
Bc_ctg1_5051	B4	57.58262959	Bcer	S	Bcer	FALSE	NA	NA	NA
Bc_ctg1_5052	B4	41.17827581	Bcer	KQ	Bcer	TRUE	NA	NA	NA
Bc_ctg1_5053	B4	0.111594243	Bcer	EGP	Bcer	FALSE	NA	NA	NA
Bc_ctg1_5054	B4	1.673913651	Bcer	K	Bcer	FALSE	NA	NA	NA
Bc_ctg1_5055	B4	2.789856085	Bcer	M	Bcer	FALSE	NA	NA	NA
Bc_ctg1_5056	B4	9.708699175	Bcer	NA	Bcer	FALSE	NA	NA	NA
Bc_ctg1_5058	B4	4.017392762	Bcer	K	Bcer	FALSE	NA	NA	NA
Bc_ctg1_5059	B4	8.927539471	Bcer	C	Bcer	FALSE	NA	NA	NA
Bc_ctg1_506	B4	6.026089143	Bcer	E	Bcer	FALSE	NA	NA	NA
Bc_ctg1_5060	B4	2.231884868	Bcer	S	Bcer	FALSE	NA	NA	NA
Bc_ctg1_5061	B4	0	Bcer	NA	Bcer	FALSE	NA	NA	NA
Bc_ctg1_5062	B4	9.708699175	Bcer	FG	Bcer	FALSE	NA	NA	NA
Bc_ctg1_5063	B4	6.24927763	Bcer	K	Bcer	FALSE	NA	NA	NA
Bc_ctg1_5064	B4	0.33478273	Bcer	EG	Bcer	FALSE	Bc_Ga0417192_01_rn37	Other	NA
Bc_ctg1_5065	B4	2.901450328	Bcer	G	Bcer	FALSE	Bc_Ga0417192_01_rn37	Other	NA
Bc_ctg1_5066	B4	0	Bcer	F	Bcer	FALSE	Bc_Ga0417192_01_rn37	Other	NA
Bc_ctg1_5067	B4	9.820293418	Bcer	K	Bcer	FALSE	Bc_Ga0417192_01_rn37	Other	NA
Bc_ctg1_5068	B4	2.231884868	Bcer	S	Bcer	FALSE	Bc_Ga0417192_01_rn37	Other	NA
Bc_ctg1_5069	B4	10.26667039	Bcer	F	Bcer	FALSE	Bc_Ga0417192_01_rn37	Other	NA
Bc_ctg1_507	B4	991.5148525	Bcer	E	Bcer	FALSE	NA	NA	NA
Bc_ctg1_5070	B4	0	Bcer	NA	Bcer	FALSE	Bc_Ga0417192_01_rn37	Core biosynthesis	None
Bc_ctg1_5071	B4	1.562319407	Bcer	V	Bcer	FALSE	Bc_Ga0417192_01_rn37	Other	NA
Bc_ctg1_5072	B4	3.905798519	Bcer	V	Bcer	FALSE	Bc_Ga0417192_01_rn37	Other	NA
Bc_ctg1_5073	B4	3.124638815	Bcer	V	Bcer	FALSE	Bc_Ga0417192_01_rn37	Other	NA
Bc_ctg1_5075	B4	4.240581249	Bcer	P	Bcer	FALSE	Bc_Ga0417192_01_rn37	Transport	SMCOG1137
Bc_ctg1_5076	B4	0.33478273	Bcer	P	Bcer	FALSE	Bc_Ga0417192_01_rn37	Other	NA
Bc_ctg1_5077	B4	0.223188487	Bcer	K	Bcer	FALSE	Bc_Ga0417192_01_rn37	Other	NA
Bc_ctg1_5078	B4	0.223188487	Bcer	F	Bcer	FALSE	Bc_Ga0417192_01_rn37	Other	NA
Bc_ctg1_5079	B4	9.262322201	Bcer	L	Bcer	FALSE	Bc_Ga0417192_01_rn37	Other	NA
Bc_ctg1_508	B4	2.008696381	Bcer	K	Bcer	FALSE	NA	NA	NA
Bc_ctg1_5080	B4	8.481162498	Bcer	M	Bcer	FALSE	NA	NA	NA
Bc_ctg1_5081	B4	10.93623585	Bcer	S	Bcer	FALSE	NA	NA	NA
Bc_ctg1_5082	B4	24.21595082	Bcer	S	Bcer	FALSE	NA	NA	NA
Bc_ctg1_5083	B4	0.33478273	Bcer	L	Bcer	FALSE	NA	NA	NA
Bc_ctg1_5084	B4	4.798552466	Bcer	J	Bcer	FALSE	NA	NA	NA
Bc_ctg1_5085	B4	38.8347967	Bcer	S	Bcer	FALSE	NA	NA	NA
Bc_ctg1_5086	B4	21.8724717	Bcer	P	Bcer	FALSE	NA	NA	NA
Bc_ctg1_5087	B4	5.9144949	Bcer	NA	Bcer	FALSE	NA	NA	NA
Bc_ctg1_5088	B4	1.897102138	Bcer	S	Bcer	FALSE	NA	NA	NA
Bc_ctg1_5089	B4	4.352175492	Bcer	S	Bcer	FALSE	NA	NA	NA
Bc_ctg1_509	B4	1.562319407	Bcer	NA	Bcer	FALSE	NA	NA	NA
Bc_ctg1_5090	B4	34.59421545	Bcer	S	Bcer	FALSE	NA	NA	NA
Bc_ctg1_5091	B4	7.365220064	Bcer	K	Bcer	FALSE	NA	NA	NA
Bc_ctg1_5092	B4	12.49855526	Bcer	S	Bcer	FALSE	NA	NA	NA
Bc_ctg1_5093	B4	10.60145312	Bcer	J	Bcer	FALSE	NA	NA	NA
Bc_ctg1_5094	B4	16.18116529	Bcer	CH	Bcer	FALSE	NA	NA	NA
Bc_ctg1_5095	B4	0	Bcer	V	Bcer	FALSE	NA	NA	NA
Bc_ctg1_5096	B4	0	Bcer	NA	Bcer	FALSE	NA	NA	NA
Bc_ctg1_5097	B4	39.95073913	Bcer	L	Bcer	FALSE	NA	NA	NA
Bc_ctg1_5098	B4	7.25362582	Bcer	K	Bcer	FALSE	NA	NA	NA
Bc_ctg1_5099	B4	0.33478273	Bcer	NA	Bcer	FALSE	NA	NA	NA
Bc_ctg1_51	B4	0.892753947	Bcer	S	Bcer	FALSE	NA	NA	NA
Bc_ctg1_510	B4	0.781159704	Bcer	K	Bcer	FALSE	NA	NA	NA
Bc_ctg1_5100	B4	2.678261841	Bcer	G	Bcer	FALSE	NA	NA	NA
Bc_ctg1_5101	B4	5.356523683	Bcer	NA	Bcer	FALSE	NA	NA	NA
Bc_ctg1_5102	B4	0.223188487	Bcer	NA	Bcer	FALSE	NA	NA	NA

Bc_ctg1_5103	B4	4.352175492	Bcer	V	Bcer	FALSE	Bc_Ga0417192_01_rn38	Other	NA
Bc_ctg1_5104	B4	3.124638815	Bcer	CG	Bcer	FALSE	Bc_Ga0417192_01_rn38	Ancillary biosynthesis	SMCOG1062
Bc_ctg1_5105	B4	6.91884309	Bcer	KLT	Bcer	FALSE	Bc_Ga0417192_01_rn38	Other	NA
Bc_ctg1_5106	B4	2.231884868	Bcer	L	Bcer	FALSE	Bc_Ga0417192_01_rn38	Other	NA
Bc_ctg1_5107	B4	40.06233338	Bcer	E	Bcer	FALSE	Bc_Ga0417192_01_rn38	Ancillary biosynthesis	SMCOG1103
Bc_ctg1_5108	B4	5.691306413	Bcer	S	Bcer	FALSE	Bc_Ga0417192_01_rn38	Other	NA
Bc_ctg1_5109	B4	0.33478273	Bcer	NA	Bcer	FALSE	Bc_Ga0417192_01_rn38	Other	NA
Bc_ctg1_511	B4	28.34493782	Bcer	S	Bcer	FALSE	NA	NA	NA
Bc_ctg1_5110	B4	5.021740953	Bcer	Q	Bcer	TRUE	Bc_Ga0417192_01_rn38	Other	NA
Bc_ctg1_5111	B4	3.459421545	Bcer	S	Bcer	FALSE	Bc_Ga0417192_01_rn38	Other	NA
Bc_ctg1_5112	B4	2.231884868	Bcer	K	Bcer	FALSE	Bc_Ga0417192_01_rn38	Other	NA
Bc_ctg1_5113	B4	7.25362582	Bcer	S	Bcer	FALSE	Bc_Ga0417192_01_rn38	Ancillary biosynthesis	SMCOG1115
Bc_ctg1_5114	B4	0.892753947	Bcer	T	Bcer	FALSE	Bc_Ga0417192_01_rn38	Regulation	SMCOG1008
Bc_ctg1_5115	B4	1.785507894	Bcer	T	Bcer	FALSE	Bc_Ga0417192_01_rn38	Regulation	SMCOG1003
Bc_ctg1_5116	B4	0.223188487	Bcer	S	Bcer	FALSE	Bc_Ga0417192_01_rn38	Other	NA
Bc_ctg1_5117	B4	0.446376974	Bcer	NA	Bcer	FALSE	Bc_Ga0417192_01_rn38	Other	NA
Bc_ctg1_5118	B4	4.575363979	Bcer	V	Bcer	FALSE	Bc_Ga0417192_01_rn38	Transport	SMCOG1000
Bc_ctg1_5119	B4	1.115942434	Bcer	V	Bcer	FALSE	Bc_Ga0417192_01_rn38	Other	NA
Bc_ctg1_512	B4	22.98841414	Bcer	V	Bcer	FALSE	NA	NA	NA
Bc_ctg1_5120	B4	6.026089143	Bcer	S	Bcer	FALSE	Bc_Ga0417192_01_rn38	Other	NA
Bc_ctg1_5121	B4	20.75652927	Bcer	I	Bcer	FALSE	Bc_Ga0417192_01_rn38	Ancillary biosynthesis	SMCOG1066
Bc_ctg1_5122	B4	7.365220064	Bcer	E	Bcer	FALSE	Bc_Ga0417192_01_rn38	Ancillary biosynthesis	SMCOG1180
Bc_ctg1_5123	B4	15.9579768	Bcer	Q	Bcer	TRUE	Bc_Ga0417192_01_rn38	Core biosynthesis	SMCOG1002
Bc_ctg1_5124	B4	1.673913651	Bcer	C	Bcer	FALSE	Bc_Ga0417192_01_rn38	Ancillary biosynthesis	None
Bc_ctg1_5125	B4	14.95362861	Bcer	G	Bcer	FALSE	Bc_Ga0417192_01_rn38	Core biosynthesis	SMCOG1297
Bc_ctg1_5126	B4	2527.386424	Bcer	E	Bcer	FALSE	Bc_Ga0417192_01_rn38	Other	NA
Bc_ctg1_5127	B4	37.27247729	Bcer	F	Bcer	FALSE	Bc_Ga0417192_01_rn38	Ancillary biosynthesis	SMCOG1283
Bc_ctg1_5128	B4	5.244929439	Bcer	S	Bcer	FALSE	Bc_Ga0417192_01_rn38	Other	NA
Bc_ctg1_5129	B4	2.678261841	Bcer	L	Bcer	FALSE	Bc_Ga0417192_01_rn38	Other	NA
Bc_ctg1_513	B4	12.27536677	Bcer	K	Bcer	FALSE	NA	NA	NA
Bc_ctg1_5130	B4	0	Bcer	E	Bcer	FALSE	Bc_Ga0417192_01_rn38	Other	NA
Bc_ctg1_5131	B4	172.7478888	Bcer	U	Bcer	FALSE	Bc_Ga0417192_01_rn38	Other	NA
Bc_ctg1_5132	B4	0.66956546	Bcer	EG	Bcer	FALSE	Bc_Ga0417192_01_rn38	Transport	SMCOG1166
Bc_ctg1_5133	B4	2.231884868	Bcer	K	Bcer	FALSE	Bc_Ga0417192_01_rn38	Ancillary biosynthesis	SMCOG1019
Bc_ctg1_5134	B4	4.352175492	Bcer	C	Bcer	FALSE	Bc_Ga0417192_01_rn38	Ancillary biosynthesis	SMCOG1028
Bc_ctg1_5135	B4	1.673913651	Bcer	I	Bcer	FALSE	Bc_Ga0417192_01_rn38	Ancillary biosynthesis	SMCOG1036
Bc_ctg1_5136	B4	0.111594243	Bcer	NA	Bcer	FALSE	Bc_Ga0417192_01_rn38	Other	NA
Bc_ctg1_5137	B4	0.66956546	Bcer	NU	Bcer	FALSE	Bc_Ga0417192_01_rn38	Other	NA
Bc_ctg1_5138	B4	2.120290624	Bcer	NA	Bcer	FALSE	Bc_Ga0417192_01_rn38	Other	NA
Bc_ctg1_5139	B4	7.811597037	Bcer	S	Bcer	FALSE	Bc_Ga0417192_01_rn38	Other	NA
Bc_ctg1_514	B4	4.910146709	Bcer	K	Bcer	FALSE	NA	NA	NA
Bc_ctg1_5140	B4	9.373916445	Bcer	S	Bcer	FALSE	NA	NA	NA
Bc_ctg1_5141	B4	12.16377253	Bcer	J	Bcer	FALSE	NA	NA	NA
Bc_ctg1_5142	B4	2.789856085	Bcer	NA	Bcer	FALSE	NA	NA	NA
Bc_ctg1_5143	B4	0	Bcer	S	Bcer	FALSE	NA	NA	NA
Bc_ctg1_5144	B4	0.557971217	Bcer	H	Bcer	FALSE	NA	NA	NA
Bc_ctg1_5145	B4	0	Bcer	M	Bcer	FALSE	NA	NA	NA
Bc_ctg1_5147	B4	3.905798519	Bcer	C	Bcer	FALSE	NA	NA	NA
Bc_ctg1_5148	B4	5.244929439	Bcer	V	Bcer	FALSE	NA	NA	NA
Bc_ctg1_5149	B4	44.7492916	Bcer	E	Bcer	FALSE	NA	NA	NA
Bc_ctg1_515	B4	0.892753947	Bcer	S	Bcer	FALSE	NA	NA	NA
Bc_ctg1_5150	B4	30.13044572	Bcer	S	Bcer	FALSE	NA	NA	NA
Bc_ctg1_5151	B4	56.46668716	Bcer	E	Bcer	FALSE	NA	NA	NA
Bc_ctg1_5152	B4	11.27101858	Bcer	K	Bcer	FALSE	NA	NA	NA
Bc_ctg1_5153	B4	0.557971217	Bcer	F	Bcer	FALSE	NA	NA	NA
Bc_ctg1_5154	B4	0.223188487	Bcer	Q	Bcer	TRUE	NA	NA	NA
Bc_ctg1_5155	B4	0.111594243	Bcer	Q	Bcer	TRUE	NA	NA	NA
Bc_ctg1_5156	B4	5.244929439	Bcer	G	Bcer	FALSE	NA	NA	NA
Bc_ctg1_5157	B4	0.111594243	Bcer	S	Bcer	FALSE	NA	NA	NA
Bc_ctg1_5158	B4	25.33189325	Bcer	KQ	Bcer	TRUE	NA	NA	NA
Bc_ctg1_5159	B4	0.557971217	Bcer	NA	Bcer	FALSE	NA	NA	NA
Bc_ctg1_516	B4	17.7434847	Bcer	S	Bcer	FALSE	NA	NA	NA

Bc_ctg1_5160	B4	1.004348191	Bcer	F	Bcer	FALSE	NA	NA	NA
Bc_ctg1_5161	B4	2.120290624	Bcer	K	Bcer	FALSE	NA	NA	NA
Bc_ctg1_5162	B4	9.597104932	Bcer	K	Bcer	FALSE	NA	NA	NA
Bc_ctg1_5163	B4	1.227536677	Bcer	NA	Bcer	FALSE	NA	NA	NA
Bc_ctg1_5164	B4	0.446376974	Bcer	Q	Bcer	TRUE	NA	NA	NA
Bc_ctg1_5165	B4	7.588408551	Bcer	K	Bcer	FALSE	NA	NA	NA
Bc_ctg1_5166	B4	3.682610032	Bcer	S	Bcer	FALSE	NA	NA	NA
Bc_ctg1_5167	B4	0.557971217	Bcer	NA	Bcer	FALSE	NA	NA	NA
Bc_ctg1_5168	B4	1.339130921	Bcer	C	Bcer	FALSE	NA	NA	NA
Bc_ctg1_5169	B4	0	Bcer	C	Bcer	FALSE	NA	NA	NA
Bc_ctg1_517	B4	365.6943356	Bcer	L	Bcer	FALSE	NA	NA	NA
Bc_ctg1_5170	B4	0.33478273	Bcer	C	Bcer	FALSE	NA	NA	NA
Bc_ctg1_5171	B4	0	Bcer	C	Bcer	FALSE	NA	NA	NA
Bc_ctg1_5173	B4	12.16377253	Bcer	S	Bcer	FALSE	NA	NA	NA
Bc_ctg1_5174	B4	6.58406036	Bcer	S	Bcer	FALSE	NA	NA	NA
Bc_ctg1_5175	B4	3.236233058	Bcer	S	Bcer	FALSE	NA	NA	NA
Bc_ctg1_5176	B4	0.446376974	Bcer	S	Bcer	FALSE	NA	NA	NA
Bc_ctg1_5177	B4	0.446376974	Bcer	L	Bcer	FALSE	NA	NA	NA
Bc_ctg1_5178	B4	0.557971217	Bcer	S	Bcer	FALSE	NA	NA	NA
Bc_ctg1_5179	B4	2.566667598	Bcer	T	Bcer	FALSE	NA	NA	NA
Bc_ctg1_518	B4	186.5855749	Bcer	L	Bcer	FALSE	NA	NA	NA
Bc_ctg1_5180	B4	0.781159704	Bcer	FG	Bcer	FALSE	NA	NA	NA
Bc_ctg1_5182	B4	0.781159704	Bcer	P	Bcer	FALSE	NA	NA	NA
Bc_ctg1_5183	B4	0	Bcer	E	Bcer	FALSE	NA	NA	NA
Bc_ctg1_5184	B4	0	Bcer	E	Bcer	FALSE	NA	NA	NA
Bc_ctg1_5185	B4	2.789856085	Bcer	OU	Bcer	FALSE	NA	NA	NA
Bc_ctg1_5186	B4	4.128987005	Bcer	K	Bcer	FALSE	NA	NA	NA
Bc_ctg1_5187	B4	0	Bcer	C	Bcer	FALSE	NA	NA	NA
Bc_ctg1_5188	B4	0.111594243	Bcer	G	Bcer	FALSE	NA	NA	NA
Bc_ctg1_5189	B4	11.49420707	Bcer	S	Bcer	FALSE	NA	NA	NA
Bc_ctg1_519	B4	56.91306413	Bcer	S	Bcer	FALSE	NA	NA	NA
Bc_ctg1_5190	B4	526.2784518	Bcer	S	Bcer	FALSE	NA	NA	NA
Bc_ctg1_5191	B4	9.708699175	Bcer	CG	Bcer	FALSE	NA	NA	NA
Bc_ctg1_5192	B4	1.450725164	Bcer	S	Bcer	FALSE	NA	NA	NA
Bc_ctg1_5193	B4	4.575363979	Bcer	F	Bcer	FALSE	NA	NA	NA
Bc_ctg1_5194	B4	2.901450328	Bcer	T	Bcer	FALSE	NA	NA	NA
Bc_ctg1_5195	B4	5.021740953	Bcer	Q	Bcer	TRUE	NA	NA	NA
Bc_ctg1_5196	B4	2.120290624	Bcer	S	Bcer	FALSE	NA	NA	NA
Bc_ctg1_5197	B4	4.798552466	Bcer	K	Bcer	FALSE	NA	NA	NA
Bc_ctg1_5198	B4	4.128987005	Bcer	K	Bcer	FALSE	NA	NA	NA
Bc_ctg1_5199	B4	3.682610032	Bcer	NA	Bcer	FALSE	NA	NA	NA
Bc_ctg1_520	B4	596.3596367	Bcer	O	Bcer	FALSE	NA	NA	NA
Bc_ctg1_5200	B4	8.034785524	Bcer	M	Bcer	FALSE	NA	NA	NA
Bc_ctg1_5201	B4	3.682610032	Bcer	C	Bcer	FALSE	NA	NA	NA
Bc_ctg1_5202	B4	0.66956546	Bcer	K	Bcer	FALSE	NA	NA	NA
Bc_ctg1_5203	B4	2.008696381	Bcer	S	Bcer	FALSE	NA	NA	NA
Bc_ctg1_5204	B4	6.91884309	Bcer	NA	Bcer	FALSE	NA	NA	NA
Bc_ctg1_5205	B4	21.53768897	Bcer	L	Bcer	FALSE	NA	NA	NA
Bc_ctg1_5206	B4	0	Bcer	S	Bcer	FALSE	NA	NA	NA
Bc_ctg1_5207	B4	10.15507615	Bcer	S	Bcer	FALSE	NA	NA	NA
Bc_ctg1_5208	B4	64.38987844	Bcer	L	Bcer	FALSE	NA	NA	NA
Bc_ctg1_521	B4	53.2304541	Bcer	T	Bcer	FALSE	NA	NA	NA
Bc_ctg1_5210	B4	0.111594243	Bcer	M	Bcer	FALSE	NA	NA	NA
Bc_ctg1_5211	B4	4.128987005	Bcer	NA	Bcer	FALSE	NA	NA	NA
Bc_ctg1_5212	B4	1.785507894	Bcer	NA	Bcer	FALSE	NA	NA	NA
Bc_ctg1_5213	B4	0	Bcer	NA	Bcer	FALSE	NA	NA	NA
Bc_ctg1_5214	B4	1.785507894	Bcer	S	Bcer	FALSE	NA	NA	NA
Bc_ctg1_5215	B4	0.557971217	Bcer	S	Bcer	FALSE	NA	NA	NA
Bc_ctg1_5216	B4	3.124638815	Bcer	E	Bcer	FALSE	NA	NA	NA
Bc_ctg1_5217	B4	369.1537571	Bcer	C	Bcer	FALSE	NA	NA	NA
Bc_ctg1_5218	B4	27.45218387	Bcer	S	Bcer	FALSE	NA	NA	NA
Bc_ctg1_5219	B4	15.28841134	Bcer	K	Bcer	FALSE	NA	NA	NA

Bc_ctg1_522	B4	2.901450328	Bcer	C	Bcer	FALSE	NA	NA	NA
Bc_ctg1_5220	B4	7.25362582	Bcer	H	Bcer	FALSE	NA	NA	NA
Bc_ctg1_5221	B4	0.223188487	Bcer	S	Bcer	FALSE	NA	NA	NA
Bc_ctg1_5222	B4	1.004348191	Bcer	E	Bcer	FALSE	NA	NA	NA
Bc_ctg1_5223	B4	3.794204275	Bcer	C	Bcer	FALSE	NA	NA	NA
Bc_ctg1_5224	B4	1.562319407	Bcer	E	Bcer	FALSE	NA	NA	NA
Bc_ctg1_5225	B4	0.223188487	Bcer	E	Bcer	FALSE	NA	NA	NA
Bc_ctg1_5226	B4	1.004348191	Bcer	E	Bcer	FALSE	NA	NA	NA
Bc_ctg1_5227	B4	0.111594243	Bcer	E	Bcer	FALSE	NA	NA	NA
Bc_ctg1_5228	B4	10.26667039	Bcer	KT	Bcer	FALSE	NA	NA	NA
Bc_ctg1_5229	B4	2.120290624	Bcer	C	Bcer	FALSE	NA	NA	NA
Bc_ctg1_523	B4	107.5768506	Bcer	F	Bcer	FALSE	NA	NA	NA
Bc_ctg1_5230	B4	2.901450328	Bcer	C	Bcer	FALSE	NA	NA	NA
Bc_ctg1_5231	B4	10.60145312	Bcer	C	Bcer	FALSE	NA	NA	NA
Bc_ctg1_5232	B4	7.25362582	Bcer	S	Bcer	FALSE	NA	NA	NA
Bc_ctg1_5233	B4	27.89856085	Bcer	S	Bcer	FALSE	NA	NA	NA
Bc_ctg1_5234	B4	28.45653206	Bcer	NA	Bcer	FALSE	NA	NA	NA
Bc_ctg1_5236	B4	64.05509571	Bcer	S	Bcer	FALSE	NA	NA	NA
Bc_ctg1_5238	B4	12.16377253	Bcer	S	Bcer	FALSE	NA	NA	NA
Bc_ctg1_5239	B4	0	Bcer	NA	Bcer	FALSE	NA	NA	NA
Bc_ctg1_524	B4	87.04350984	Bcer	I	Bcer	FALSE	NA	NA	NA
Bc_ctg1_5240	B4	34.48262121	Bcer	M	Bcer	FALSE	NA	NA	NA
Bc_ctg1_5241	B4	17.40870197	Bcer	E	Bcer	FALSE	NA	NA	NA
Bc_ctg1_5242	B4	8.704350984	Bcer	M	Bcer	FALSE	NA	NA	NA
Bc_ctg1_5243	B4	2.343479111	Bcer	M	Bcer	FALSE	NA	NA	NA
Bc_ctg1_5244	B4	3.236233058	Bcer	S	Bcer	FALSE	NA	NA	NA
Bc_ctg1_5245	B4	1.897102138	Bcer	D	Bcer	FALSE	NA	NA	NA
Bc_ctg1_5246	B4	2.678261841	Bcer	C	Bcer	FALSE	NA	NA	NA
Bc_ctg1_5247	B4	7.476814307	Bcer	K	Bcer	FALSE	NA	NA	NA
Bc_ctg1_5248	B4	2.678261841	Bcer	S	Bcer	FALSE	NA	NA	NA
Bc_ctg1_5249	B4	0.223188487	Bcer	NA	Bcer	FALSE	NA	NA	NA
Bc_ctg1_525	B4	31.24638815	Bcer	S	Bcer	FALSE	NA	NA	NA
Bc_ctg1_5250	B4	0.781159704	Bcer	NA	Bcer	FALSE	NA	NA	NA
Bc_ctg1_5251	B4	22.20725443	Bcer	Q	Bcer	TRUE	NA	NA	NA
Bc_ctg1_5252	B4	1.339130921	Bcer	S	Bcer	FALSE	NA	NA	NA
Bc_ctg1_5253	B4	25.88986447	Bcer	M	Bcer	FALSE	NA	NA	NA
Bc_ctg1_5254	B4	8.034785524	Bcer	T	Bcer	FALSE	NA	NA	NA
Bc_ctg1_5255	B4	3.013044572	Bcer	T	Bcer	FALSE	NA	NA	NA
Bc_ctg1_5256	B4	0	Bcer	NA	Bcer	FALSE	NA	NA	NA
Bc_ctg1_5257	B4	0.781159704	Bcer	K	Bcer	FALSE	NA	NA	NA
Bc_ctg1_5258	B4	11.8289898	Bcer	S	Bcer	FALSE	NA	NA	NA
Bc_ctg1_5259	B4	10.82464161	Bcer	F	Bcer	FALSE	NA	NA	NA
Bc_ctg1_526	B4	67.06814028	Bcer	S	Bcer	FALSE	NA	NA	NA
Bc_ctg1_5260	B4	5.57971217	Bcer	F	Bcer	FALSE	NA	NA	NA
Bc_ctg1_5261	B4	12.83333799	Bcer	S	Bcer	FALSE	NA	NA	NA
Bc_ctg1_5262	B4	9.150727958	Bcer	M	Bcer	FALSE	NA	NA	NA
Bc_ctg1_5263	B4	25.44348749	Bcer	E	Bcer	FALSE	NA	NA	NA
Bc_ctg1_5264	B4	29.79566299	Bcer	S	Bcer	FALSE	NA	NA	NA
Bc_ctg1_5265	B4	1.785507894	Bcer	S	Bcer	FALSE	NA	NA	NA
Bc_ctg1_5267	B4	0	Bcer	S	Bcer	FALSE	NA	NA	NA
Bc_ctg1_5268	B4	11.49420707	Bcer	K	Bcer	FALSE	NA	NA	NA
Bc_ctg1_5269	B4	8.592756741	Bcer	K	Bcer	FALSE	NA	NA	NA
Bc_ctg1_527	B4	1.897102138	Bcer	M	Bcer	FALSE	NA	NA	NA
Bc_ctg1_5270	B4	0	Bcer	NA	Bcer	FALSE	NA	NA	NA
Bc_ctg1_5271	B4	56.68987564	Bcer	NA	Bcer	FALSE	NA	NA	NA
Bc_ctg1_5272	B4	364.3552047	Bcer	EGP	Bcer	FALSE	NA	NA	NA
Bc_ctg1_5273	B4	122.6420735	Bcer	M	Bcer	FALSE	NA	NA	NA
Bc_ctg1_5274	B4	181.8986167	Bcer	M	Bcer	FALSE	NA	NA	NA
Bc_ctg1_5275	B4	329.3146122	Bcer	K	Bcer	FALSE	NA	NA	NA
Bc_ctg1_5276	B4	8.146379768	Bcer	S	Bcer	FALSE	NA	NA	NA
Bc_ctg1_5277	B4	0.446376974	Bcer	EGP	Bcer	FALSE	NA	NA	NA
Bc_ctg1_5278	B4	10.82464161	Bcer	G	Bcer	FALSE	NA	NA	NA

Bc_ctg1_5279	B4	5.802900656	Bcer	NA	Bcer	FALSE	NA	NA	NA
Bc_ctg1_5280	B4	0	Bcer	NA	Bcer	FALSE	NA	NA	NA
Bc_ctg1_5281	B4	41.95943551	Bcer	NA	Bcer	FALSE	NA	NA	NA
Bc_ctg1_5282	B4	0.446376974	Bcer	S	Bcer	FALSE	NA	NA	NA
Bc_ctg1_5283	B4	1.227536677	Bcer	F	Bcer	FALSE	NA	NA	NA
Bc_ctg1_5284	B4	0.111594243	Bcer	S	Bcer	FALSE	NA	NA	NA
Bc_ctg1_5285	B4	33.81305575	Bcer	Q	Bcer	TRUE	NA	NA	NA
Bc_ctg1_5286	B4	0.111594243	Bcer	NA	Bcer	FALSE	NA	NA	NA
Bc_ctg1_5289	B4	0.33478273	Bcer	NA	Bcer	FALSE	NA	NA	NA
Bc_ctg1_5290	B4	26.33624144	Bcer	EGP	Bcer	FALSE	NA	NA	NA
Bc_ctg1_5291	B4	0.66956546	Bcer	NA	Bcer	FALSE	NA	NA	NA
Bc_ctg1_5292	B4	5.133335196	Bcer	NA	Bcer	FALSE	NA	NA	NA
Bc_ctg1_5293	B4	276.1957524	Bcer	E	Bcer	FALSE	NA	NA	NA
Bc_ctg1_5294	B4	11.94058404	Bcer	I	Bcer	FALSE	NA	NA	NA
Bc_ctg1_5295	B4	29.5724745	Bcer	H	Bcer	FALSE	NA	NA	NA
Bc_ctg1_5296	B4	1.562319407	Bcer	J	Bcer	FALSE	NA	NA	NA
Bc_ctg1_5297	B4	5.468117926	Bcer	O	Bcer	FALSE	NA	NA	NA
Bc_ctg1_5298	B4	3.124638815	Bcer	NA	Bcer	FALSE	NA	NA	NA
Bc_ctg1_5299	B4	2.008696381	Bcer	V	Bcer	FALSE	NA	NA	NA
Bc_ctg1_53	B4	0.33478273	Bcer	E	Bcer	FALSE	NA	NA	NA
Bc_ctg1_5300	B4	0.33478273	Bcer	K	Bcer	FALSE	NA	NA	NA
Bc_ctg1_5301	B4	0.446376974	Bcer	NA	Bcer	FALSE	NA	NA	NA
Bc_ctg1_5302	B4	0	Bcer	S	Bcer	FALSE	NA	NA	NA
Bc_ctg1_5304	B4	0	Bcer	NA	Bcer	FALSE	NA	NA	NA
Bc_ctg1_5305	B4	0	Bcer	NA	Bcer	FALSE	NA	NA	NA
Bc_ctg1_5307	B4	0	Bcer	S	Bcer	FALSE	NA	NA	NA
Bc_ctg1_5309	B4	61.93480508	Bcer	S	Bcer	FALSE	NA	NA	NA
Bc_ctg1_5310	B4	55.90871594	Bcer	S	Bcer	FALSE	NA	NA	NA
Bc_ctg1_5311	B4	7.25362582	Bcer	C	Bcer	FALSE	NA	NA	NA
Bc_ctg1_5312	B4	6.24927763	Bcer	P	Bcer	FALSE	NA	NA	NA
Bc_ctg1_5313	B4	5.802900656	Bcer	P	Bcer	FALSE	NA	NA	NA
Bc_ctg1_5314	B4	1.227536677	Bcer	P	Bcer	FALSE	NA	NA	NA
Bc_ctg1_5315	B4	4.128987005	Bcer	S	Bcer	FALSE	NA	NA	NA
Bc_ctg1_5317	B4	21.53768897	Bcer	NA	Bcer	FALSE	NA	NA	NA
Bc_ctg1_5318	B4	10.82464161	Bcer	S	Bcer	FALSE	NA	NA	NA
Bc_ctg1_5319	B4	12.05217829	Bcer	H	Bcer	FALSE	NA	NA	NA
Bc_ctg1_532	B4	15.51159983	Bcer	L	Bcer	FALSE	NA	NA	NA
Bc_ctg1_5320	B4	13.50290345	Bcer	V	Bcer	FALSE	NA	NA	NA
Bc_ctg1_5321	B4	0	Bcer	K	Bcer	FALSE	NA	NA	NA
Bc_ctg1_5322	B4	24.21595082	Bcer	EG	Bcer	FALSE	NA	NA	NA
Bc_ctg1_5323	B4	0.446376974	Bcer	NA	Bcer	FALSE	NA	NA	NA
Bc_ctg1_5324	B4	8.704350984	Bcer	K	Bcer	FALSE	NA	NA	NA
Bc_ctg1_5325	B4	5.244929439	Bcer	J	Bcer	FALSE	NA	NA	NA
Bc_ctg1_5326	B4	2.789856085	Bcer	S	Bcer	FALSE	NA	NA	NA
Bc_ctg1_5327	B4	5.468117926	Bcer	L	Bcer	FALSE	NA	NA	NA
Bc_ctg1_5328	B4	0.223188487	Bcer	E	Bcer	FALSE	NA	NA	NA
Bc_ctg1_5329	B4	0.111594243	Bcer	E	Bcer	FALSE	NA	NA	NA
Bc_ctg1_533	B4	0.781159704	Bcer	CO	Bcer	FALSE	NA	NA	NA
Bc_ctg1_5330	B4	1.227536677	Bcer	E	Bcer	FALSE	NA	NA	NA
Bc_ctg1_5331	B4	0.66956546	Bcer	S	Bcer	FALSE	NA	NA	NA
Bc_ctg1_5332	B4	24.4391393	Bcer	S	Bcer	FALSE	NA	NA	NA
Bc_ctg1_5333	B4	152.660925	Bcer	G	Bcer	FALSE	NA	NA	NA
Bc_ctg1_5334	B4	0.557971217	Bcer	S	Bcer	FALSE	NA	NA	NA
Bc_ctg1_5335	B4	1.004348191	Bcer	P	Bcer	FALSE	NA	NA	NA
Bc_ctg1_5336	B4	1519.24403	Bcer	M	Bcer	FALSE	NA	NA	NA
Bc_ctg1_5337	B4	704.1596758	Bcer	S	Bcer	FALSE	NA	NA	NA
Bc_ctg1_5338	B4	13.61449769	Bcer	S	Bcer	FALSE	NA	NA	NA
Bc_ctg1_5339	B4	60.93045689	Bcer	K	Bcer	FALSE	NA	NA	NA
Bc_ctg1_534	B4	2.008696381	Bcer	NA	Bcer	FALSE	NA	NA	NA
Bc_ctg1_5340	B4	53.90001956	Bcer	S	Bcer	FALSE	NA	NA	NA
Bc_ctg1_5341	B4	184.1305016	Bcer	E	Bcer	FALSE	NA	NA	NA
Bc_ctg1_5342	B4	60.7072684	Bcer	E	Bcer	FALSE	NA	NA	NA

Bc_ctg1_5343	B4	86.37394438	Bcer	E	Bcer	FALSE	NA	NA	NA
Bc_ctg1_5344	B4	44.86088584	Bcer	E	Bcer	FALSE	NA	NA	NA
Bc_ctg1_5345	B4	151.6565768	Bcer	E	Bcer	FALSE	NA	NA	NA
Bc_ctg1_5346	B4	78.33915886	Bcer	K	Bcer	FALSE	NA	NA	NA
Bc_ctg1_5347	B4	0.111594243	Bcer	S	Bcer	FALSE	NA	NA	NA
Bc_ctg1_5348	B4	8.257974011	Bcer	Q	Bcer	TRUE	NA	NA	NA
Bc_ctg1_5349	B4	5.021740953	Bcer	J	Bcer	FALSE	NA	NA	NA
Bc_ctg1_535	B4	0.66956546	Bcer	V	Bcer	FALSE	NA	NA	NA
Bc_ctg1_5350	B4	4.240581249	Bcer	S	Bcer	FALSE	NA	NA	NA
Bc_ctg1_5351	B4	0	Bcer	S	Bcer	FALSE	NA	NA	NA
Bc_ctg1_5352	B4	2.231884868	Bcer	NA	Bcer	FALSE	NA	NA	NA
Bc_ctg1_5353	B4	1.562319407	Bcer	S	Bcer	FALSE	NA	NA	NA
Bc_ctg1_5354	B4	18.07826743	Bcer	M	Bcer	FALSE	NA	NA	NA
Bc_ctg1_5355	B4	0	Bcer	S	Bcer	FALSE	NA	NA	NA
Bc_ctg1_5356	B4	3.236233058	Bcer	E	Bcer	FALSE	NA	NA	NA
Bc_ctg1_5357	B4	1.897102138	Bcer	C	Bcer	FALSE	NA	NA	NA
Bc_ctg1_5358	B4	0.781159704	Bcer	K	Bcer	FALSE	NA	NA	NA
Bc_ctg1_5359	B4	4.017392762	Bcer	NA	Bcer	FALSE	NA	NA	NA
Bc_ctg1_536	B4	7830.902841	Bcer	C	Bcer	FALSE	NA	NA	NA
Bc_ctg1_5360	B4	5.9144949	Bcer	NA	Bcer	FALSE	NA	NA	NA
Bc_ctg1_5361	B4	53.45364258	Bcer	S	Bcer	FALSE	NA	NA	NA
Bc_ctg1_5362	B4	1091.168512	Bcer	S	Bcer	FALSE	NA	NA	NA
Bc_ctg1_5363	B4	1.227536677	Bcer	G	Bcer	FALSE	NA	NA	NA
Bc_ctg1_5364	B4	0.111594243	Bcer	G	Bcer	FALSE	NA	NA	NA
Bc_ctg1_5365	B4	0.33478273	Bcer	G	Bcer	FALSE	NA	NA	NA
Bc_ctg1_5366	B4	0.111594243	Bcer	G	Bcer	FALSE	NA	NA	NA
Bc_ctg1_5367	B4	0.111594243	Bcer	K	Bcer	FALSE	NA	NA	NA
Bc_ctg1_5368	B4	0	Bcer	G	Bcer	FALSE	NA	NA	NA
Bc_ctg1_5369	B4	0	Bcer	G	Bcer	FALSE	NA	NA	NA
Bc_ctg1_537	B4	0	Bcer	S	Bcer	FALSE	NA	NA	NA
Bc_ctg1_5370	B4	0.557971217	Bcer	G	Bcer	FALSE	NA	NA	NA
Bc_ctg1_5371	B4	0.66956546	Bcer	E	Bcer	FALSE	NA	NA	NA
Bc_ctg1_5372	B4	2.008696381	Bcer	S	Bcer	FALSE	NA	NA	NA
Bc_ctg1_5373	B4	5.57971217	Bcer	EGP	Bcer	FALSE	NA	NA	NA
Bc_ctg1_5374	B4	5.468117926	Bcer	K	Bcer	FALSE	NA	NA	NA
Bc_ctg1_5375	B4	0.111594243	Bcer	F	Bcer	FALSE	NA	NA	NA
Bc_ctg1_5376	B4	28.12174933	Bcer	E	Bcer	FALSE	NA	NA	NA
Bc_ctg1_5377	B4	16.62754227	Bcer	E	Bcer	FALSE	NA	NA	NA
Bc_ctg1_5378	B4	3.571015789	Bcer	E	Bcer	FALSE	NA	NA	NA
Bc_ctg1_5379	B4	26.44783568	Bcer	E	Bcer	FALSE	NA	NA	NA
Bc_ctg1_538	B4	2.789856085	Bcer	G	Bcer	FALSE	NA	NA	NA
Bc_ctg1_5380	B4	9.708699175	Bcer	K	Bcer	FALSE	NA	NA	NA
Bc_ctg1_5381	B4	4.240581249	Bcer	E	Bcer	FALSE	NA	NA	NA
Bc_ctg1_5382	B4	4.575363979	Bcer	E	Bcer	FALSE	NA	NA	NA
Bc_ctg1_5383	B4	0	Bcer	E	Bcer	FALSE	NA	NA	NA
Bc_ctg1_5384	B4	182.5681822	Bcer	I	Bcer	FALSE	NA	NA	NA
Bc_ctg1_5385	B4	24.32754506	Bcer	V	Bcer	FALSE	NA	NA	NA
Bc_ctg1_5386	B4	2.120290624	Bcer	V	Bcer	FALSE	NA	NA	NA
Bc_ctg1_5387	B4	1.339130921	Bcer	P	Bcer	FALSE	NA	NA	NA
Bc_ctg1_5388	B4	6.472466117	Bcer	S	Bcer	FALSE	NA	NA	NA
Bc_ctg1_5389	B4	1.562319407	Bcer	K	Bcer	FALSE	NA	NA	NA
Bc_ctg1_539	B4	2.455073355	Bcer	NA	Bcer	FALSE	NA	NA	NA
Bc_ctg1_5390	B4	10.82464161	Bcer	L	Bcer	FALSE	NA	NA	NA
Bc_ctg1_5391	B4	1.673913651	Bcer	NA	Bcer	FALSE	NA	NA	NA
Bc_ctg1_5392	B4	17.29710773	Bcer	S	Bcer	FALSE	NA	NA	NA
Bc_ctg1_5393	B4	29.79566299	Bcer	F	Bcer	FALSE	NA	NA	NA
Bc_ctg1_5394	B4	18.41305016	Bcer	J	Bcer	FALSE	NA	NA	NA
Bc_ctg1_5395	B4	22.09566019	Bcer	E	Bcer	FALSE	NA	NA	NA
Bc_ctg1_5397	B4	0	Bcer	NA	Bcer	FALSE	NA	NA	NA
Bc_ctg1_5398	B4	5.468117926	Bcer	S	Bcer	FALSE	NA	NA	NA
Bc_ctg1_5399	B4	2.789856085	Bcer	K	Bcer	FALSE	NA	NA	NA
Bc_ctg1_54	B4	0	Bcer	P	Bcer	FALSE	NA	NA	NA

Bc_ctg1_5400	B4	0.781159704	Bcer	S	Bcer	FALSE	NA	NA	NA
Bc_ctg1_5401	B4	1.673913651	Bcer	S	Bcer	FALSE	NA	NA	NA
Bc_ctg1_5402	B4	2.566667598	Bcer	NA	Bcer	FALSE	NA	NA	NA
Bc_ctg1_5403	B4	775.2452088	Bcer	EGP	Bcer	FALSE	NA	NA	NA
Bc_ctg1_5404	B4	1.227536677	Bcer	E	Bcer	FALSE	NA	NA	NA
Bc_ctg1_5405	B4	2.789856085	Bcer	C	Bcer	FALSE	NA	NA	NA
Bc_ctg1_5406	B4	1.673913651	Bcer	S	Bcer	FALSE	NA	NA	NA
Bc_ctg1_5407	B4	0.111594243	Bcer	NA	Bcer	FALSE	NA	NA	NA
Bc_ctg1_5408	B4	9.820293418	Bcer	F	Bcer	FALSE	NA	NA	NA
Bc_ctg1_5409	B4	18.18986167	Bcer	V	Bcer	FALSE	NA	NA	NA
Bc_ctg1_541	B4	0.223188487	Bcer	O	Bcer	FALSE	NA	NA	NA
Bc_ctg1_5410	B4	0.66956546	Bcer	NA	Bcer	FALSE	NA	NA	NA
Bc_ctg1_5411	B4	6.91884309	Bcer	S	Bcer	FALSE	NA	NA	NA
Bc_ctg1_5412	B4	15.06522286	Bcer	C	Bcer	FALSE	NA	NA	NA
Bc_ctg1_5413	B4	7.923191281	Bcer	K	Bcer	FALSE	NA	NA	NA
Bc_ctg1_5414	B4	40.28552186	Bcer	J	Bcer	FALSE	NA	NA	NA
Bc_ctg1_5415	B4	0	Bcer	F	Bcer	FALSE	NA	NA	NA
Bc_ctg1_5416	B4	2.343479111	Bcer	S	Bcer	FALSE	NA	NA	NA
Bc_ctg1_5417	B4	11.94058404	Bcer	S	Bcer	FALSE	NA	NA	NA
Bc_ctg1_5418	B4	17.85507894	Bcer	V	Bcer	FALSE	NA	NA	NA
Bc_ctg1_5419	B4	6.137683386	Bcer	K	Bcer	FALSE	NA	NA	NA
Bc_ctg1_542	B4	37.04928881	Bcer	EGP	Bcer	FALSE	NA	NA	NA
Bc_ctg1_5421	B4	13.72609194	Bcer	GM	Bcer	FALSE	NA	NA	NA
Bc_ctg1_5422	B4	7.923191281	Bcer	V	Bcer	FALSE	NA	NA	NA
Bc_ctg1_5423	B4	6.137683386	Bcer	K	Bcer	FALSE	NA	NA	NA
Bc_ctg1_5424	B4	4.910146709	Bcer	K	Bcer	FALSE	NA	NA	NA
Bc_ctg1_5425	B4	3.013044572	Bcer	NA	Bcer	FALSE	NA	NA	NA
Bc_ctg1_5426	B4	0	Bcer	L	Bcer	FALSE	NA	NA	NA
Bc_ctg1_5427	B4	0.557971217	Bcer	EG	Bcer	FALSE	NA	NA	NA
Bc_ctg1_5428	B4	25.33189325	Bcer	K	Bcer	FALSE	NA	NA	NA
Bc_ctg1_5429	B4	12.49855526	Bcer	S	Bcer	FALSE	NA	NA	NA
Bc_ctg1_543	B4	13.72609194	Bcer	NA	Bcer	FALSE	NA	NA	NA
Bc_ctg1_5430	B4	2.455073355	Bcer	S	Bcer	FALSE	NA	NA	NA
Bc_ctg1_5431	B4	16.18116529	Bcer	S	Bcer	FALSE	NA	NA	NA
Bc_ctg1_5432	B4	9.485510688	Bcer	F	Bcer	FALSE	NA	NA	NA
Bc_ctg1_5433	B4	10.71304737	Bcer	T	Bcer	FALSE	NA	NA	NA
Bc_ctg1_5434	B4	8.704350984	Bcer	S	Bcer	FALSE	NA	NA	NA
Bc_ctg1_5435	B4	6.807248847	Bcer	E	Bcer	FALSE	NA	NA	NA
Bc_ctg1_5436	B4	5.802900656	Bcer	S	Bcer	FALSE	NA	NA	NA
Bc_ctg1_5437	B4	1.004348191	Bcer	J	Bcer	FALSE	NA	NA	NA
Bc_ctg1_544	B4	40.62030459	Bcer	J	Bcer	FALSE	NA	NA	NA
Bc_ctg1_545	B4	2.901450328	Bcer	F	Bcer	FALSE	NA	NA	NA
Bc_ctg1_546	B4	5.133335196	Bcer	NA	Bcer	FALSE	NA	NA	NA
Bc_ctg1_547	B4	4.686958222	Bcer	I	Bcer	FALSE	NA	NA	NA
Bc_ctg1_548	B4	0.33478273	Bcer	IQ	Bcer	TRUE	NA	NA	NA
Bc_ctg1_549	B4	4.017392762	Bcer	S	Bcer	FALSE	NA	NA	NA
Bc_ctg1_55	B4	1.450725164	Bcer	S	Bcer	FALSE	NA	NA	NA
Bc_ctg1_550	B4	14.17246891	Bcer	IQ	Bcer	TRUE	NA	NA	NA
Bc_ctg1_551	B4	1.227536677	Bcer	S	Bcer	FALSE	NA	NA	NA
Bc_ctg1_552	B4	3.124638815	Bcer	V	Bcer	FALSE	NA	NA	NA
Bc_ctg1_553	B4	5.468117926	Bcer	K	Bcer	FALSE	NA	NA	NA
Bc_ctg1_554	B4	1.450725164	Bcer	NA	Bcer	FALSE	NA	NA	NA
Bc_ctg1_555	B4	2.343479111	Bcer	K	Bcer	FALSE	NA	NA	NA
Bc_ctg1_556	B4	1.004348191	Bcer	NA	Bcer	FALSE	NA	NA	NA
Bc_ctg1_557	B4	22.43044292	Bcer	S	Bcer	FALSE	NA	NA	NA
Bc_ctg1_558	B4	3.571015789	Bcer	V	Bcer	FALSE	NA	NA	NA
Bc_ctg1_559	B4	7.700002794	Bcer	T	Bcer	FALSE	NA	NA	NA
Bc_ctg1_56	B4	0.111594243	Bcer	L	Bcer	FALSE	NA	NA	NA
Bc_ctg1_560	B4	23.43479111	Bcer	S	Bcer	FALSE	NA	NA	NA
Bc_ctg1_561	B4	2.231884868	Bcer	E	Bcer	FALSE	NA	NA	NA
Bc_ctg1_562	B4	38.94639094	Bcer	T	Bcer	FALSE	NA	NA	NA
Bc_ctg1_563	B4	13.39130921	Bcer	S	Bcer	FALSE	NA	NA	NA

Bc_ctg1_564	B4	11.38261283	Bcer	E	Bcer	FALSE	NA	NA	NA
Bc_ctg1_565	B4	3.013044572	Bcer	E	Bcer	FALSE	NA	NA	NA
Bc_ctg1_566	B4	1.562319407	Bcer	O	Bcer	FALSE	NA	NA	NA
Bc_ctg1_567	B4	0.111594243	Bcer	E	Bcer	FALSE	NA	NA	NA
Bc_ctg1_568	B4	74.99133156	Bcer	C	Bcer	FALSE	NA	NA	NA
Bc_ctg1_569	B4	139.0464273	Bcer	K	Bcer	FALSE	NA	NA	NA
Bc_ctg1_57	B4	15.62319407	Bcer	L	Bcer	FALSE	NA	NA	NA
Bc_ctg1_570	B4	81.79858041	Bcer	E	Bcer	FALSE	NA	NA	NA
Bc_ctg1_571	B4	114.607288	Bcer	Q	Bcer	TRUE	NA	NA	NA
Bc_ctg1_572	B4	151.321794	Bcer	E	Bcer	FALSE	NA	NA	NA
Bc_ctg1_573	B4	137.7072963	Bcer	E	Bcer	FALSE	NA	NA	NA
Bc_ctg1_574	B4	693.0002515	Bcer	K	Bcer	FALSE	NA	NA	NA
Bc_ctg1_575	B4	3.347827302	Bcer	Q	Bcer	TRUE	NA	NA	NA
Bc_ctg1_576	B4	2.231884868	Bcer	S	Bcer	FALSE	NA	NA	NA
Bc_ctg1_577	B4	0.446376974	Bcer	F	Bcer	FALSE	NA	NA	NA
Bc_ctg1_578	B4	15.51159983	Bcer	NA	Bcer	FALSE	NA	NA	NA
Bc_ctg1_579	B4	10.60145312	Bcer	S	Bcer	FALSE	NA	NA	NA
Bc_ctg1_58	B4	16.62754227	Bcer	L	Bcer	FALSE	NA	NA	NA
Bc_ctg1_580	B4	143.3986028	Bcer	O	Bcer	FALSE	NA	NA	NA
Bc_ctg1_581	B4	58.69857202	Bcer	O	Bcer	FALSE	NA	NA	NA
Bc_ctg1_582	B4	0	Bcer	NA	Bcer	FALSE	NA	NA	NA
Bc_ctg1_583	B4	48.76668436	Bcer	EG	Bcer	FALSE	NA	NA	NA
Bc_ctg1_584	B4	21.091312	Bcer	E	Bcer	FALSE	NA	NA	NA
Bc_ctg1_585	B4	22.8768199	Bcer	S	Bcer	FALSE	NA	NA	NA
Bc_ctg1_586	B4	20.08696381	Bcer	V	Bcer	FALSE	NA	NA	NA
Bc_ctg1_588	B4	3.905798519	Bcer	V	Bcer	FALSE	NA	NA	NA
Bc_ctg1_589	B4	1.004348191	Bcer	NA	Bcer	FALSE	NA	NA	NA
Bc_ctg1_59	B4	2.008696381	Bcer	NA	Bcer	FALSE	NA	NA	NA
Bc_ctg1_590	B4	33.14349029	Bcer	K	Bcer	FALSE	NA	NA	NA
Bc_ctg1_591	B4	4.128987005	Bcer	S	Bcer	FALSE	NA	NA	NA
Bc_ctg1_592	B4	26.78261841	Bcer	M	Bcer	FALSE	NA	NA	NA
Bc_ctg1_593	B4	1.785507894	Bcer	F	Bcer	FALSE	NA	NA	NA
Bc_ctg1_594	B4	0.223188487	Bcer	S	Bcer	FALSE	NA	NA	NA
Bc_ctg1_595	B4	14.3956574	Bcer	S	Bcer	FALSE	NA	NA	NA
Bc_ctg1_596	B4	0	Bcer	NA	Bcer	FALSE	NA	NA	NA
Bc_ctg1_597	B4	10.26667039	Bcer	NA	Bcer	FALSE	NA	NA	NA
Bc_ctg1_598	B4	7.811597037	Bcer	S	Bcer	FALSE	NA	NA	NA
Bc_ctg1_599	B4	0.33478273	Bcer	M	Bcer	FALSE	NA	NA	NA
Bc_ctg1_6	B4	2.678261841	Bcer	NA	Bcer	FALSE	NA	NA	NA
Bc_ctg1_60	B4	11.60580131	Bcer	E	Bcer	FALSE	NA	NA	NA
Bc_ctg1_600	B4	20.75652927	Bcer	V	Bcer	FALSE	NA	NA	NA
Bc_ctg1_601	B4	11.27101858	Bcer	V	Bcer	FALSE	NA	NA	NA
Bc_ctg1_602	B4	73.42901215	Bcer	S	Bcer	FALSE	NA	NA	NA
Bc_ctg1_603	B4	36.60291183	Bcer	S	Bcer	FALSE	NA	NA	NA
Bc_ctg1_604	B4	3400.722973	Bcer	G	Bcer	FALSE	NA	NA	NA
Bc_ctg1_605	B4	4.910146709	Bcer	P	Bcer	FALSE	NA	NA	NA
Bc_ctg1_606	B4	1.450725164	Bcer	P	Bcer	FALSE	NA	NA	NA
Bc_ctg1_607	B4	4.575363979	Bcer	P	Bcer	FALSE	NA	NA	NA
Bc_ctg1_608	B4	1.562319407	Bcer	P	Bcer	FALSE	NA	NA	NA
Bc_ctg1_609	B4	20.42174654	Bcer	F	Bcer	FALSE	NA	NA	NA
Bc_ctg1_61	B4	21.20290624	Bcer	K	Bcer	FALSE	NA	NA	NA
Bc_ctg1_610	B4	8.369568254	Bcer	S	Bcer	FALSE	NA	NA	NA
Bc_ctg1_611	B4	12.83333799	Bcer	L	Bcer	FALSE	NA	NA	NA
Bc_ctg1_612	B4	416.3581221	Bcer	K	Bcer	FALSE	NA	NA	NA
Bc_ctg1_613	B4	5.133335196	Bcer	S	Bcer	FALSE	NA	NA	NA
Bc_ctg1_614	B4	8.704350984	Bcer	S	Bcer	FALSE	NA	NA	NA
Bc_ctg1_615	B4	0	Bcer	K	Bcer	FALSE	NA	NA	NA
Bc_ctg1_616	B4	2.678261841	Bcer	S	Bcer	FALSE	NA	NA	NA
Bc_ctg1_617	B4	33.58986726	Bcer	S	Bcer	FALSE	NA	NA	NA
Bc_ctg1_618	B4	44.41450887	Bcer	M	Bcer	FALSE	NA	NA	NA
Bc_ctg1_619	B4	323.0653346	Bcer	M	Bcer	FALSE	NA	NA	NA
Bc_ctg1_62	B4	0.223188487	Bcer	L	Bcer	FALSE	NA	NA	NA

Bc_ctg1_620	B4	0.892753947	Bcer	S	Bcer	FALSE	NA	NA	NA
Bc_ctg1_621	B4	2.901450328	Bcer	L	Bcer	FALSE	NA	NA	NA
Bc_ctg1_622	B4	0	Bcer	NA	Bcer	FALSE	NA	NA	NA
Bc_ctg1_623	B4	2.566667598	Bcer	NA	Bcer	FALSE	NA	NA	NA
Bc_ctg1_624	B4	0	Bcer	NA	Bcer	FALSE	NA	NA	NA
Bc_ctg1_625	B4	51.55654045	Bcer	K	Bcer	FALSE	NA	NA	NA
Bc_ctg1_626	B4	3169.499701	Bcer	E	Bcer	FALSE	NA	NA	NA
Bc_ctg1_627	B4	297.5102529	Bcer	K	Bcer	FALSE	NA	NA	NA
Bc_ctg1_628	B4	86.26235014	Bcer	P	Bcer	FALSE	NA	NA	NA
Bc_ctg1_629	B4	83.47249406	Bcer	S	Bcer	FALSE	NA	NA	NA
Bc_ctg1_63	B4	11.49420707	Bcer	V	Bcer	FALSE	NA	NA	NA
Bc_ctg1_630	B4	15.9579768	Bcer	S	Bcer	FALSE	NA	NA	NA
Bc_ctg1_631	B4	0.111594243	Bcer	NA	Bcer	FALSE	NA	NA	NA
Bc_ctg1_632	B4	2.566667598	Bcer	O	Bcer	FALSE	NA	NA	NA
Bc_ctg1_633	B4	105.010183	Bcer	L	Bcer	FALSE	NA	NA	NA
Bc_ctg1_634	B4	2.231884868	Bcer	Q	Bcer	TRUE	NA	NA	NA
Bc_ctg1_635	B4	188.9290541	Bcer	J	Bcer	FALSE	NA	NA	NA
Bc_ctg1_636	B4	121.6377253	Bcer	J	Bcer	FALSE	NA	NA	NA
Bc_ctg1_637	B4	0	Bcer	NA	Bcer	FALSE	NA	NA	NA
Bc_ctg1_638	B4	118.0667095	Bcer	T	Bcer	FALSE	NA	NA	NA
Bc_ctg1_639	B4	3913.944898	Bcer	GT	Bcer	FALSE	NA	NA	NA
Bc_ctg1_64	B4	5.021740953	Bcer	S	Bcer	FALSE	NA	NA	NA
Bc_ctg1_640	B4	1068.403286	Bcer	G	Bcer	FALSE	NA	NA	NA
Bc_ctg1_641	B4	1600.26145	Bcer	K	Bcer	FALSE	NA	NA	NA
Bc_ctg1_642	B4	174.9797736	Bcer	C	Bcer	FALSE	NA	NA	NA
Bc_ctg1_643	B4	136.8145424	Bcer	S	Bcer	FALSE	NA	NA	NA
Bc_ctg1_645	B4	79.23191281	Bcer	NA	Bcer	FALSE	NA	NA	NA
Bc_ctg1_646	B4	1.004348191	Bcer	E	Bcer	FALSE	NA	NA	NA
Bc_ctg1_647	B4	0.111594243	Bcer	G	Bcer	FALSE	NA	NA	NA
Bc_ctg1_648	B4	0.781159704	Bcer	S	Bcer	FALSE	NA	NA	NA
Bc_ctg1_649	B4	4.798552466	Bcer	P	Bcer	FALSE	NA	NA	NA
Bc_ctg1_65	B4	76.77683945	Bcer	F	Bcer	FALSE	NA	NA	NA
Bc_ctg1_650	B4	280.9943049	Bcer	L	Bcer	FALSE	NA	NA	NA
Bc_ctg1_651	B4	4.463769736	Bcer	M	Bcer	FALSE	NA	NA	NA
Bc_ctg1_652	B4	39.05798519	Bcer	P	Bcer	FALSE	NA	NA	NA
Bc_ctg1_653	B4	10.37826464	Bcer	P	Bcer	FALSE	NA	NA	NA
Bc_ctg1_654	B4	18.97102138	Bcer	S	Bcer	FALSE	NA	NA	NA
Bc_ctg1_655	B4	0	Bcer	NA	Bcer	FALSE	NA	NA	NA
Bc_ctg1_656	B4	17.18551348	Bcer	S	Bcer	FALSE	NA	NA	NA
Bc_ctg1_657	B4	10.37826464	Bcer	HP	Bcer	FALSE	NA	NA	NA
Bc_ctg1_658	B4	18.97102138	Bcer	P	Bcer	FALSE	NA	NA	NA
Bc_ctg1_659	B4	26.11305295	Bcer	P	Bcer	FALSE	NA	NA	NA
Bc_ctg1_66	B4	10.82464161	Bcer	I	Bcer	FALSE	NA	NA	NA
Bc_ctg1_660	B4	99.09568813	Bcer	P	Bcer	FALSE	NA	NA	NA
Bc_ctg1_661	B4	13.94928042	Bcer	L	Bcer	FALSE	NA	NA	NA
Bc_ctg1_662	B4	10.26667039	Bcer	K	Bcer	FALSE	Bc_Ga0417192_01_rn07	Regulation	SMCOG1287
Bc_ctg1_663	B4	8.592756741	Bcer	L	Bcer	FALSE	Bc_Ga0417192_01_rn07	Other	NA
Bc_ctg1_664	B4	20.19855805	Bcer	L	Bcer	FALSE	Bc_Ga0417192_01_rn07	Other	NA
Bc_ctg1_665	B4	274.2986503	Bcer	E	Bcer	FALSE	Bc_Ga0417192_01_rn07	Other	NA
Bc_ctg1_666	B4	0.781159704	Bcer	S	Bcer	FALSE	Bc_Ga0417192_01_rn07	Other	NA
Bc_ctg1_667	B4	14.3956574	Bcer	NA	Bcer	FALSE	Bc_Ga0417192_01_rn07	Other	NA
Bc_ctg1_668	B4	9.820293418	Bcer	G	Bcer	FALSE	Bc_Ga0417192_01_rn07	Ancillary biosynthesis	SMCOG1101
Bc_ctg1_669	B4	13.61449769	Bcer	S	Bcer	FALSE	Bc_Ga0417192_01_rn07	Ancillary biosynthesis	SMCOG1262
Bc_ctg1_67	B4	1.673913651	Bcer	Q	Bcer	TRUE	NA	NA	NA
Bc_ctg1_670	B4	166.8333939	Bcer	T	Bcer	FALSE	Bc_Ga0417192_01_rn07	Ancillary biosynthesis	SMCOG1025
Bc_ctg1_671	B4	0.66956546	Bcer	M	Bcer	FALSE	Bc_Ga0417192_01_rn07	Core biosynthesis	None
Bc_ctg1_672	B4	0	Bcer	NA	Bcer	FALSE	Bc_Ga0417192_01_rn07	Other	NA
Bc_ctg1_673	B4	0.223188487	Bcer	GM	Bcer	FALSE	Bc_Ga0417192_01_rn07	Core biosynthesis	SMCOG1010
Bc_ctg1_674	B4	0.33478273	Bcer	M	Bcer	FALSE	Bc_Ga0417192_01_rn07	Core biosynthesis	SMCOG1010
Bc_ctg1_675	B4	0.781159704	Bcer	S	Bcer	FALSE	Bc_Ga0417192_01_rn07	Other	NA
Bc_ctg1_676	B4	2.008696381	Bcer	S	Bcer	FALSE	Bc_Ga0417192_01_rn07	Other	NA
Bc_ctg1_677	B4	3.794204275	Bcer	M	Bcer	FALSE	Bc_Ga0417192_01_rn07	Other	NA

Bc_ctg1_678	B4	1.897102138	Bcer	S	Bcer	FALSE	Bc_Ga0417192_01_rn07	Other	NA
Bc_ctg1_679	B4	11.60580131	Bcer	K	Bcer	FALSE	Bc_Ga0417192_01_rn07	Ancillary biosynthesis	SMCOG1019
Bc_ctg1_68	B4	2.566667598	Bcer	Q	Bcer	TRUE	NA	NA	NA
Bc_ctg1_680	B4	0.557971217	Bcer	NA	Bcer	FALSE	Bc_Ga0417192_01_rn07	Other	NA
Bc_ctg1_681	B4	33.36667877	Bcer	K	Bcer	FALSE	Bc_Ga0417192_01_rn07	Regulation	SMCOG1136
Bc_ctg1_682	B4	85.70437892	Bcer	G	Bcer	FALSE	Bc_Ga0417192_01_rn07	Other	NA
Bc_ctg1_683	B4	57.80581808	Bcer	U	Bcer	FALSE	Bc_Ga0417192_01_rn07	Other	NA
Bc_ctg1_684	B4	45.75363979	Bcer	O	Bcer	FALSE	NA	NA	NA
Bc_ctg1_685	B4	425.955227	Bcer	M	Bcer	FALSE	NA	NA	NA
Bc_ctg1_686	B4	10.71304737	Bcer	S	Bcer	FALSE	NA	NA	NA
Bc_ctg1_687	B4	19.86377532	Bcer	M	Bcer	FALSE	NA	NA	NA
Bc_ctg1_688	B4	15.28841134	Bcer	S	Bcer	FALSE	NA	NA	NA
Bc_ctg1_69	B4	1.115942434	Bcer	NA	Bcer	FALSE	NA	NA	NA
Bc_ctg1_690	B4	171.6319463	Bcer	L	Bcer	FALSE	NA	NA	NA
Bc_ctg1_691	B4	199.6421014	Bcer	L	Bcer	FALSE	NA	NA	NA
Bc_ctg1_692	B4	2.455073355	Bcer	S	Bcer	FALSE	NA	NA	NA
Bc_ctg1_693	B4	127.2174375	Bcer	S	Bcer	FALSE	NA	NA	NA
Bc_ctg1_694	B4	558.5291882	Bcer	J	Bcer	FALSE	NA	NA	NA
Bc_ctg1_695	B4	1042.625016	Bcer	C	Bcer	FALSE	NA	NA	NA
Bc_ctg1_696	B4	2254.650093	Bcer	C	Bcer	FALSE	NA	NA	NA
Bc_ctg1_697	B4	25.66667598	Bcer	E	Bcer	FALSE	NA	NA	NA
Bc_ctg1_698	B4	44.52610311	Bcer	S	Bcer	FALSE	NA	NA	NA
Bc_ctg1_699	B4	97.75655721	Bcer	S	Bcer	FALSE	NA	NA	NA
Bc_ctg1_7	B4	0.557971217	Bcer	S	Bcer	FALSE	NA	NA	NA
Bc_ctg1_70	B4	5.133335196	Bcer	S	Bcer	FALSE	NA	NA	NA
Bc_ctg1_700	B4	2918.07787	Bcer	S	Bcer	FALSE	NA	NA	NA
Bc_ctg1_701	B4	547.5929523	Bcer	L	Bcer	FALSE	NA	NA	NA
Bc_ctg1_702	B4	379.4204275	Bcer	S	Bcer	FALSE	NA	NA	NA
Bc_ctg1_703	B4	117.1739556	Bcer	I	Bcer	FALSE	NA	NA	NA
Bc_ctg1_704	B4	334.0015705	Bcer	S	Bcer	FALSE	NA	NA	NA
Bc_ctg1_705	B4	209.0160179	Bcer	S	Bcer	FALSE	NA	NA	NA
Bc_ctg1_706	B4	1.227536677	Bcer	S	Bcer	FALSE	NA	NA	NA
Bc_ctg1_707	B4	23.10000838	Bcer	IQ	Bcer	TRUE	NA	NA	NA
Bc_ctg1_708	B4	197.4102166	Bcer	S	Bcer	FALSE	NA	NA	NA
Bc_ctg1_709	B4	147.192807	Bcer	S	Bcer	FALSE	NA	NA	NA
Bc_ctg1_71	B4	6.807248847	Bcer	K	Bcer	FALSE	NA	NA	NA
Bc_ctg1_710	B4	359.2218695	Bcer	S	Bcer	FALSE	NA	NA	NA
Bc_ctg1_711	B4	335.2291071	Bcer	S	Bcer	FALSE	NA	NA	NA
Bc_ctg1_712	B4	406.5378287	Bcer	S	Bcer	FALSE	NA	NA	NA
Bc_ctg1_713	B4	1174.529412	Bcer	S	Bcer	FALSE	NA	NA	NA
Bc_ctg1_714	B4	373.2827441	Bcer	K	Bcer	FALSE	NA	NA	NA
Bc_ctg1_715	B4	63.38553025	Bcer	NA	Bcer	FALSE	NA	NA	NA
Bc_ctg1_716	B4	153.4420847	Bcer	D	Bcer	FALSE	NA	NA	NA
Bc_ctg1_717	B4	0.223188487	Bcer	S	Bcer	FALSE	NA	NA	NA
Bc_ctg1_718	B4	5.021740953	Bcer	OU	Bcer	FALSE	NA	NA	NA
Bc_ctg1_719	B4	1264.809155	Bcer	S	Bcer	FALSE	NA	NA	NA
Bc_ctg1_72	B4	1.115942434	Bcer	K	Bcer	FALSE	NA	NA	NA
Bc_ctg1_720	B4	1087.151119	Bcer	E	Bcer	FALSE	NA	NA	NA
Bc_ctg1_721	B4	1645.568713	Bcer	E	Bcer	FALSE	NA	NA	NA
Bc_ctg1_722	B4	1408.096163	Bcer	E	Bcer	FALSE	NA	NA	NA
Bc_ctg1_723	B4	4.575363979	Bcer	H	Bcer	FALSE	NA	NA	NA
Bc_ctg1_724	B4	9.262322201	Bcer	NA	Bcer	FALSE	NA	NA	NA
Bc_ctg1_726	B4	68.07248847	Bcer	T	Bcer	FALSE	NA	NA	NA
Bc_ctg1_727	B4	45.86523403	Bcer	NA	Bcer	FALSE	NA	NA	NA
Bc_ctg1_728	B4	1.227536677	Bcer	NA	Bcer	FALSE	NA	NA	NA
Bc_ctg1_729	B4	0	Bcer	L	Bcer	FALSE	NA	NA	NA
Bc_ctg1_73	B4	2.901450328	Bcer	P	Bcer	FALSE	NA	NA	NA
Bc_ctg1_731	B4	0.557971217	Bcer	M	Bcer	FALSE	NA	NA	NA
Bc_ctg1_732	B4	0	Bcer	S	Bcer	FALSE	NA	NA	NA
Bc_ctg1_733	B4	54.23480229	Bcer	S	Bcer	FALSE	NA	NA	NA
Bc_ctg1_734	B4	0	Bcer	NA	Bcer	FALSE	NA	NA	NA
Bc_ctg1_735	B4	1.115942434	Bcer	U	Bcer	FALSE	NA	NA	NA

Bc_ctg1_736	B4	0	Bcer	S	Bcer	FALSE	NA	NA	NA
Bc_ctg1_737	B4	3.236233058	Bcer	D	Bcer	FALSE	NA	NA	NA
Bc_ctg1_739	B4	0	Bcer	S	Bcer	FALSE	NA	NA	NA
Bc_ctg1_74	B4	9.262322201	Bcer	S	Bcer	FALSE	NA	NA	NA
Bc_ctg1_740	B4	0	Bcer	S	Bcer	FALSE	NA	NA	NA
Bc_ctg1_741	B4	0	Bcer	NA	Bcer	FALSE	NA	NA	NA
Bc_ctg1_742	B4	0	Bcer	S	Bcer	FALSE	NA	NA	NA
Bc_ctg1_743	B4	0	Bcer	S	Bcer	FALSE	NA	NA	NA
Bc_ctg1_745	B4	0	Bcer	S	Bcer	FALSE	NA	NA	NA
Bc_ctg1_746	B4	0.111594243	Bcer	OU	Bcer	FALSE	NA	NA	NA
Bc_ctg1_747	B4	0.892753947	Bcer	S	Bcer	FALSE	NA	NA	NA
Bc_ctg1_748	B4	0.33478273	Bcer	S	Bcer	FALSE	NA	NA	NA
Bc_ctg1_749	B4	0.223188487	Bcer	S	Bcer	FALSE	NA	NA	NA
Bc_ctg1_75	B4	0.111594243	Bcer	NA	Bcer	FALSE	NA	NA	NA
Bc_ctg1_750	B4	1.004348191	Bcer	V	Bcer	FALSE	NA	NA	NA
Bc_ctg1_751	B4	0.33478273	Bcer	NA	Bcer	FALSE	NA	NA	NA
Bc_ctg1_752	B4	1699.691921	Bcer	NA	Bcer	FALSE	NA	NA	NA
Bc_ctg1_753	B4	0	Bcer	NA	Bcer	FALSE	NA	NA	NA
Bc_ctg1_754	B4	0.223188487	Bcer	NA	Bcer	FALSE	NA	NA	NA
Bc_ctg1_755	B4	0	Bcer	L	Bcer	FALSE	NA	NA	NA
Bc_ctg1_756	B4	0	Bcer	S	Bcer	FALSE	NA	NA	NA
Bc_ctg1_757	B4	69.96959061	Bcer	S	Bcer	FALSE	NA	NA	NA
Bc_ctg1_758	B4	59.03335475	Bcer	S	Bcer	FALSE	NA	NA	NA
Bc_ctg1_759	B4	0	Bcer	NA	Bcer	FALSE	NA	NA	NA
Bc_ctg1_76	B4	1.004348191	Bcer	S	Bcer	FALSE	NA	NA	NA
Bc_ctg1_760	B4	0.111594243	Bcer	NA	Bcer	FALSE	NA	NA	NA
Bc_ctg1_761	B4	0.223188487	Bcer	NA	Bcer	FALSE	NA	NA	NA
Bc_ctg1_762	B4	0.557971217	Bcer	L	Bcer	FALSE	NA	NA	NA
Bc_ctg1_763	B4	0.557971217	Bcer	L	Bcer	FALSE	NA	NA	NA
Bc_ctg1_764	B4	0.446376974	Bcer	NA	Bcer	FALSE	NA	NA	NA
Bc_ctg1_765	B4	0.446376974	Bcer	NA	Bcer	FALSE	NA	NA	NA
Bc_ctg1_766	B4	0.223188487	Bcer	NA	Bcer	FALSE	NA	NA	NA
Bc_ctg1_767	B4	0.557971217	Bcer	NA	Bcer	FALSE	NA	NA	NA
Bc_ctg1_768	B4	0	Bcer	NA	Bcer	FALSE	NA	NA	NA
Bc_ctg1_769	B4	0	Bcer	K	Bcer	FALSE	NA	NA	NA
Bc_ctg1_77	B4	61.82321084	Bcer	G	Bcer	FALSE	NA	NA	NA
Bc_ctg1_770	B4	14.50725164	Bcer	K	Bcer	FALSE	NA	NA	NA
Bc_ctg1_771	B4	0.111594243	Bcer	E	Bcer	FALSE	NA	NA	NA
Bc_ctg1_772	B4	0.223188487	Bcer	NA	Bcer	FALSE	NA	NA	NA
Bc_ctg1_773	B4	1.897102138	Bcer	NA	Bcer	FALSE	NA	NA	NA
Bc_ctg1_774	B4	6.137683386	Bcer	L	Bcer	FALSE	NA	NA	NA
Bc_ctg1_775	B4	0.111594243	Bcer	H	Bcer	FALSE	NA	NA	NA
Bc_ctg1_776	B4	0.111594243	Bcer	E	Bcer	FALSE	NA	NA	NA
Bc_ctg1_778	B4	241.8247254	Bcer	S	Bcer	FALSE	NA	NA	NA
Bc_ctg1_779	B4	25.33189325	Bcer	G	Bcer	FALSE	NA	NA	NA
Bc_ctg1_78	B4	0	Bcer	S	Bcer	FALSE	NA	NA	NA
Bc_ctg1_780	B4	1390.464273	Bcer	J	Bcer	FALSE	NA	NA	NA
Bc_ctg1_781	B4	498.7146737	Bcer	J	Bcer	FALSE	NA	NA	NA
Bc_ctg1_782	B4	229.8841414	Bcer	H	Bcer	FALSE	NA	NA	NA
Bc_ctg1_783	B4	80.79423221	Bcer	J	Bcer	FALSE	NA	NA	NA
Bc_ctg1_784	B4	324.7392483	Bcer	J	Bcer	FALSE	NA	NA	NA
Bc_ctg1_785	B4	146.9696185	Bcer	S	Bcer	FALSE	NA	NA	NA
Bc_ctg1_786	B4	2728.367657	Bcer	J	Bcer	FALSE	NA	NA	NA
Bc_ctg1_787	B4	222.0725443	Bcer	J	Bcer	FALSE	NA	NA	NA
Bc_ctg1_788	B4	65.61741511	Bcer	K	Bcer	FALSE	NA	NA	NA
Bc_ctg1_789	B4	811.5133379	Bcer	K	Bcer	FALSE	NA	NA	NA
Bc_ctg1_79	B4	10.04348191	Bcer	I	Bcer	FALSE	NA	NA	NA
Bc_ctg1_790	B4	433.2088528	Bcer	S	Bcer	FALSE	NA	NA	NA
Bc_ctg1_791	B4	488.3364091	Bcer	L	Bcer	FALSE	NA	NA	NA
Bc_ctg1_792	B4	419.2595724	Bcer	J	Bcer	FALSE	NA	NA	NA
Bc_ctg1_793	B4	364.1320162	Bcer	M	Bcer	FALSE	NA	NA	NA
Bc_ctg1_794	B4	285.4580746	Bcer	I	Bcer	FALSE	NA	NA	NA

Bc_ctg1_795	B4	114.2725052	Bcer	S	Bcer	FALSE	NA	NA	NA
Bc_ctg1_796	B4	113.4913455	Bcer	I	Bcer	FALSE	NA	NA	NA
Bc_ctg1_797	B4	385.3349224	Bcer	J	Bcer	FALSE	NA	NA	NA
Bc_ctg1_798	B4	437.6726226	Bcer	F	Bcer	FALSE	NA	NA	NA
Bc_ctg1_799	B4	1482.752712	Bcer	J	Bcer	FALSE	NA	NA	NA
Bc_ctg1_8	B4	1.450725164	Bcer	NA	Bcer	FALSE	NA	NA	NA
Bc_ctg1_80	B4	10.04348191	Bcer	T	Bcer	FALSE	NA	NA	NA
Bc_ctg1_800	B4	3782.375285	Bcer	J	Bcer	FALSE	NA	NA	NA
Bc_ctg1_801	B4	682.1756098	Bcer	K	Bcer	FALSE	NA	NA	NA
Bc_ctg1_802	B4	618.0089199	Bcer	O	Bcer	FALSE	NA	NA	NA
Bc_ctg1_803	B4	66.28698057	Bcer	O	Bcer	FALSE	NA	NA	NA
Bc_ctg1_804	B4	177.434847	Bcer	L	Bcer	FALSE	NA	NA	NA
Bc_ctg1_805	B4	341.8131675	Bcer	J	Bcer	FALSE	NA	NA	NA
Bc_ctg1_806	B4	2279.981987	Bcer	L	Bcer	FALSE	NA	NA	NA
Bc_ctg1_807	B4	2.120290624	Bcer	LU	Bcer	FALSE	NA	NA	NA
Bc_ctg1_808	B4	2050.544222	Bcer	C	Bcer	FALSE	NA	NA	NA
Bc_ctg1_809	B4	2511.540042	Bcer	C	Bcer	FALSE	NA	NA	NA
Bc_ctg1_81	B4	42.29421825	Bcer	L	Bcer	FALSE	NA	NA	NA
Bc_ctg1_810	B4	12.27536677	Bcer	L	Bcer	FALSE	NA	NA	NA
Bc_ctg1_811	B4	130.230482	Bcer	S	Bcer	FALSE	NA	NA	NA
Bc_ctg1_812	B4	95.18988961	Bcer	U	Bcer	FALSE	NA	NA	NA
Bc_ctg1_813	B4	1799.34558	Bcer	J	Bcer	FALSE	NA	NA	NA
Bc_ctg1_814	B4	75.99567975	Bcer	J	Bcer	FALSE	NA	NA	NA
Bc_ctg1_815	B4	41.17827581	Bcer	J	Bcer	FALSE	NA	NA	NA
Bc_ctg1_816	B4	143.2870085	Bcer	S	Bcer	FALSE	NA	NA	NA
Bc_ctg1_817	B4	552.6146933	Bcer	J	Bcer	FALSE	NA	NA	NA
Bc_ctg1_818	B4	1479.628073	Bcer	U	Bcer	FALSE	NA	NA	NA
Bc_ctg1_819	B4	106.6840967	Bcer	S	Bcer	FALSE	NA	NA	NA
Bc_ctg1_82	B4	14.84203437	Bcer	K	Bcer	FALSE	NA	NA	NA
Bc_ctg1_820	B4	150.5406343	Bcer	U	Bcer	FALSE	NA	NA	NA
Bc_ctg1_821	B4	170.0696269	Bcer	D	Bcer	FALSE	NA	NA	NA
Bc_ctg1_822	B4	346.0537488	Bcer	J	Bcer	FALSE	NA	NA	NA
Bc_ctg1_823	B4	116.7275786	Bcer	IQ	Bcer	TRUE	NA	NA	NA
Bc_ctg1_824	B4	216.7160207	Bcer	IQ	Bcer	TRUE	NA	NA	NA
Bc_ctg1_825	B4	180.8942685	Bcer	I	Bcer	FALSE	NA	NA	NA
Bc_ctg1_826	B4	119.0710577	Bcer	I	Bcer	FALSE	NA	NA	NA
Bc_ctg1_827	B4	48.65509012	Bcer	K	Bcer	FALSE	NA	NA	NA
Bc_ctg1_828	B4	118.6246807	Bcer	L	Bcer	FALSE	NA	NA	NA
Bc_ctg1_829	B4	1160.580131	Bcer	S	Bcer	FALSE	NA	NA	NA
Bc_ctg1_83	B4	0.33478273	Bcer	EGP	Bcer	FALSE	NA	NA	NA
Bc_ctg1_830	B4	159.4681738	Bcer	S	Bcer	FALSE	NA	NA	NA
Bc_ctg1_831	B4	33.14349029	Bcer	J	Bcer	FALSE	NA	NA	NA
Bc_ctg1_832	B4	14.06087467	Bcer	H	Bcer	FALSE	NA	NA	NA
Bc_ctg1_833	B4	266.5986475	Bcer	G	Bcer	FALSE	NA	NA	NA
Bc_ctg1_834	B4	138.488456	Bcer	S	Bcer	FALSE	NA	NA	NA
Bc_ctg1_835	B4	674.3640128	Bcer	KLT	Bcer	FALSE	NA	NA	NA
Bc_ctg1_836	B4	160.1377393	Bcer	T	Bcer	FALSE	NA	NA	NA
Bc_ctg1_837	B4	496.9291658	Bcer	J	Bcer	FALSE	NA	NA	NA
Bc_ctg1_838	B4	283.0030012	Bcer	J	Bcer	FALSE	NA	NA	NA
Bc_ctg1_839	B4	138.2652676	Bcer	J	Bcer	FALSE	NA	NA	NA
Bc_ctg1_84	B4	2.343479111	Bcer	E	Bcer	FALSE	NA	NA	NA
Bc_ctg1_840	B4	37.16088305	Bcer	J	Bcer	FALSE	NA	NA	NA
Bc_ctg1_841	B4	94.07394718	Bcer	L	Bcer	FALSE	NA	NA	NA
Bc_ctg1_842	B4	50.77538074	Bcer	H	Bcer	FALSE	NA	NA	NA
Bc_ctg1_843	B4	41.73624703	Bcer	K	Bcer	FALSE	NA	NA	NA
Bc_ctg1_844	B4	148.6435322	Bcer	F	Bcer	FALSE	NA	NA	NA
Bc_ctg1_845	B4	51.55654045	Bcer	S	Bcer	FALSE	NA	NA	NA
Bc_ctg1_846	B4	134.2478748	Bcer	S	Bcer	FALSE	NA	NA	NA
Bc_ctg1_847	B4	22.65363141	Bcer	P	Bcer	FALSE	NA	NA	NA
Bc_ctg1_848	B4	101.2159788	Bcer	K	Bcer	FALSE	NA	NA	NA
Bc_ctg1_849	B4	5.021740953	Bcer	S	Bcer	FALSE	NA	NA	NA
Bc_ctg1_85	B4	0.111594243	Bcer	I	Bcer	FALSE	NA	NA	NA

Bc_ctg1_850	B4	1.115942434	Bcer	E	Bcer	FALSE	NA	NA	NA
Bc_ctg1_851	B4	0.223188487	Bcer	S	Bcer	FALSE	NA	NA	NA
Bc_ctg1_852	B4	1.339130921	Bcer	S	Bcer	FALSE	NA	NA	NA
Bc_ctg1_853	B4	0	Bcer	S	Bcer	FALSE	NA	NA	NA
Bc_ctg1_855	B4	0.33478273	Bcer	C	Bcer	FALSE	NA	NA	NA
Bc_ctg1_856	B4	11.49420707	Bcer	S	Bcer	FALSE	NA	NA	NA
Bc_ctg1_857	B4	25.10870476	Bcer	S	Bcer	FALSE	NA	NA	NA
Bc_ctg1_858	B4	199.9768842	Bcer	F	Bcer	FALSE	NA	NA	NA
Bc_ctg1_859	B4	252.5377728	Bcer	F	Bcer	FALSE	NA	NA	NA
Bc_ctg1_86	B4	0	Bcer	P	Bcer	FALSE	NA	NA	NA
Bc_ctg1_860	B4	434.213201	Bcer	F	Bcer	FALSE	NA	NA	NA
Bc_ctg1_861	B4	256.778354	Bcer	C	Bcer	FALSE	NA	NA	NA
Bc_ctg1_862	B4	1487.997641	Bcer	F	Bcer	FALSE	NA	NA	NA
Bc_ctg1_863	B4	294.7203968	Bcer	F	Bcer	FALSE	NA	NA	NA
Bc_ctg1_864	B4	190.4913735	Bcer	F	Bcer	FALSE	NA	NA	NA
Bc_ctg1_865	B4	71.19712728	Bcer	F	Bcer	FALSE	NA	NA	NA
Bc_ctg1_866	B4	161.1420875	Bcer	F	Bcer	FALSE	NA	NA	NA
Bc_ctg1_867	B4	166.7217996	Bcer	F	Bcer	FALSE	NA	NA	NA
Bc_ctg1_868	B4	364.3552047	Bcer	J	Bcer	FALSE	NA	NA	NA
Bc_ctg1_869	B4	118.9594635	Bcer	MU	Bcer	FALSE	NA	NA	NA
Bc_ctg1_87	B4	4.686958222	Bcer	S	Bcer	FALSE	NA	NA	NA
Bc_ctg1_870	B4	0.33478273	Bcer	T	Bcer	FALSE	NA	NA	NA
Bc_ctg1_871	B4	1131.677222	Bcer	J	Bcer	FALSE	NA	NA	NA
Bc_ctg1_872	B4	488.8943803	Bcer	D	Bcer	FALSE	NA	NA	NA
Bc_ctg1_873	B4	763.3046248	Bcer	S	Bcer	FALSE	NA	NA	NA
Bc_ctg1_874	B4	116.2812016	Bcer	S	Bcer	FALSE	NA	NA	NA
Bc_ctg1_875	B4	175.6493391	Bcer	D	Bcer	FALSE	NA	NA	NA
Bc_ctg1_876	B4	327.4175101	Bcer	S	Bcer	FALSE	Bc_Ga0417192_01_rn08	Other	NA
Bc_ctg1_877	B4	390.0218807	Bcer	S	Bcer	FALSE	Bc_Ga0417192_01_rn08	Other	NA
Bc_ctg1_878	B4	0.111594243	Bcer	S	Bcer	FALSE	Bc_Ga0417192_01_rn08	Other	NA
Bc_ctg1_879	B4	0	Bcer	K	Bcer	FALSE	Bc_Ga0417192_01_rn08	Other	NA
Bc_ctg1_88	B4	0.781159704	Bcer	CP	Bcer	FALSE	NA	NA	NA
Bc_ctg1_880	B4	2.231884868	Bcer	K	Bcer	FALSE	Bc_Ga0417192_01_rn08	Other	NA
Bc_ctg1_881	B4	1.673913651	Bcer	M	Bcer	FALSE	Bc_Ga0417192_01_rn08	Other	NA
Bc_ctg1_882	B4	1339.577298	Bcer	D	Bcer	FALSE	Bc_Ga0417192_01_rn08	Other	NA
Bc_ctg1_883	B4	1432.312114	Bcer	D	Bcer	FALSE	Bc_Ga0417192_01_rn08	Other	NA
Bc_ctg1_884	B4	26.2246472	Bcer	D	Bcer	FALSE	Bc_Ga0417192_01_rn08	Other	NA
Bc_ctg1_885	B4	90.72611988	Bcer	M	Bcer	FALSE	Bc_Ga0417192_01_rn08	Other	NA
Bc_ctg1_886	B4	44.52610311	Bcer	M	Bcer	FALSE	Bc_Ga0417192_01_rn08	Core biosynthesis	SMCOG1062
Bc_ctg1_887	B4	56.80146989	Bcer	D	Bcer	FALSE	Bc_Ga0417192_01_rn08	Other	NA
Bc_ctg1_888	B4	489.1175688	Bcer	M	Bcer	FALSE	Bc_Ga0417192_01_rn08	Other	NA
Bc_ctg1_889	B4	258.8986447	Bcer	M	Bcer	FALSE	Bc_Ga0417192_01_rn08	Core biosynthesis	None
Bc_ctg1_89	B4	0.111594243	Bcer	S	Bcer	FALSE	NA	NA	NA
Bc_ctg1_890	B4	232.450809	Bcer	M	Bcer	FALSE	Bc_Ga0417192_01_rn08	Other	NA
Bc_ctg1_891	B4	397.9450719	Bcer	M	Bcer	FALSE	Bc_Ga0417192_01_rn08	Other	NA
Bc_ctg1_892	B4	633.5205197	Bcer	M	Bcer	FALSE	Bc_Ga0417192_01_rn08	Other	NA
Bc_ctg1_893	B4	329.203018	Bcer	D	Bcer	FALSE	Bc_Ga0417192_01_rn08	Other	NA
Bc_ctg1_894	B4	486.8856839	Bcer	J	Bcer	FALSE	Bc_Ga0417192_01_rn08	Other	NA
Bc_ctg1_895	B4	237.8073327	Bcer	S	Bcer	FALSE	Bc_Ga0417192_01_rn08	Other	NA
Bc_ctg1_896	B4	19.30580411	Bcer	H	Bcer	FALSE	NA	NA	NA
Bc_ctg1_897	B4	180.0015146	Bcer	K	Bcer	FALSE	NA	NA	NA
Bc_ctg1_898	B4	6.026089143	Bcer	S	Bcer	FALSE	NA	NA	NA
Bc_ctg1_899	B4	71.08553304	Bcer	J	Bcer	FALSE	NA	NA	NA
Bc_ctg1_9	B4	0	Bcer	NA	Bcer	FALSE	NA	NA	NA
Bc_ctg1_90	B4	0	Bcer	EGP	Bcer	FALSE	NA	NA	NA
Bc_ctg1_900	B4	638.7654492	Bcer	S	Bcer	FALSE	NA	NA	NA
Bc_ctg1_901	B4	85.92756741	Bcer	S	Bcer	FALSE	NA	NA	NA
Bc_ctg1_902	B4	32.47392483	Bcer	T	Bcer	FALSE	NA	NA	NA
Bc_ctg1_903	B4	25.44348749	Bcer	S	Bcer	FALSE	NA	NA	NA
Bc_ctg1_904	B4	0.33478273	Bcer	S	Bcer	FALSE	NA	NA	NA
Bc_ctg1_905	B4	65.95219784	Bcer	H	Bcer	FALSE	NA	NA	NA
Bc_ctg1_906	B4	32.02754785	Bcer	L	Bcer	FALSE	NA	NA	NA

Bc_ctg1_907	B4	71.53191001	Bcer	S	Bcer	FALSE	NA	NA	NA
Bc_ctg1_908	B4	57.80581808	Bcer	S	Bcer	FALSE	NA	NA	NA
Bc_ctg1_909	B4	103.6710521	Bcer	S	Bcer	FALSE	NA	NA	NA
Bc_ctg1_91	B4	4.575363979	Bcer	K	Bcer	FALSE	NA	NA	NA
Bc_ctg1_910	B4	30.91160542	Bcer	G	Bcer	FALSE	NA	NA	NA
Bc_ctg1_911	B4	0.446376974	Bcer	S	Bcer	FALSE	NA	NA	NA
Bc_ctg1_912	B4	0.111594243	Bcer	S	Bcer	FALSE	NA	NA	NA
Bc_ctg1_913	B4	107.1304737	Bcer	S	Bcer	FALSE	NA	NA	NA
Bc_ctg1_914	B4	7.923191281	Bcer	S	Bcer	FALSE	NA	NA	NA
Bc_ctg1_915	B4	16.62754227	Bcer	S	Bcer	FALSE	NA	NA	NA
Bc_ctg1_916	B4	44.41450887	Bcer	S	Bcer	FALSE	NA	NA	NA
Bc_ctg1_917	B4	37.83044851	Bcer	C	Bcer	FALSE	NA	NA	NA
Bc_ctg1_918	B4	103.0014866	Bcer	C	Bcer	FALSE	NA	NA	NA
Bc_ctg1_919	B4	379.643616	Bcer	C	Bcer	FALSE	NA	NA	NA
Bc_ctg1_92	B4	0.892753947	Bcer	S	Bcer	FALSE	NA	NA	NA
Bc_ctg1_920	B4	246.5116837	Bcer	C	Bcer	FALSE	NA	NA	NA
Bc_ctg1_921	B4	268.8305323	Bcer	O	Bcer	FALSE	NA	NA	NA
Bc_ctg1_922	B4	205.5565963	Bcer	O	Bcer	FALSE	NA	NA	NA
Bc_ctg1_923	B4	0.557971217	Bcer	L	Bcer	FALSE	NA	NA	NA
Bc_ctg1_924	B4	2.343479111	Bcer	L	Bcer	FALSE	NA	NA	NA
Bc_ctg1_925	B4	3927.336208	Bcer	C	Bcer	FALSE	NA	NA	NA
Bc_ctg1_926	B4	143.06382	Bcer	D	Bcer	FALSE	NA	NA	NA
Bc_ctg1_927	B4	175.8725276	Bcer	S	Bcer	FALSE	NA	NA	NA
Bc_ctg1_928	B4	117.7319268	Bcer	S	Bcer	FALSE	NA	NA	NA
Bc_ctg1_929	B4	295.0551795	Bcer	T	Bcer	FALSE	NA	NA	NA
Bc_ctg1_93	B4	27.34058963	Bcer	G	Bcer	FALSE	NA	NA	NA
Bc_ctg1_930	B4	173.0826715	Bcer	S	Bcer	FALSE	NA	NA	NA
Bc_ctg1_931	B4	9.262322201	Bcer	S	Bcer	FALSE	NA	NA	NA
Bc_ctg1_932	B4	1.115942434	Bcer	NA	Bcer	FALSE	NA	NA	NA
Bc_ctg1_933	B4	2.789856085	Bcer	S	Bcer	FALSE	NA	NA	NA
Bc_ctg1_934	B4	2774.567673	Bcer	T	Bcer	FALSE	NA	NA	NA
Bc_ctg1_935	B4	44.97248009	Bcer	NA	Bcer	FALSE	NA	NA	NA
Bc_ctg1_936	B4	61.93480508	Bcer	G	Bcer	FALSE	NA	NA	NA
Bc_ctg1_937	B4	1.115942434	Bcer	NA	Bcer	FALSE	NA	NA	NA
Bc_ctg1_938	B4	35.04059242	Bcer	S	Bcer	FALSE	NA	NA	NA
Bc_ctg1_939	B4	3.794204275	Bcer	S	Bcer	FALSE	NA	NA	NA
Bc_ctg1_94	B4	5.133335196	Bcer	K	Bcer	FALSE	NA	NA	NA
Bc_ctg1_940	B4	451.175526	Bcer	E	Bcer	FALSE	NA	NA	NA
Bc_ctg1_941	B4	2.120290624	Bcer	H	Bcer	FALSE	NA	NA	NA
Bc_ctg1_942	B4	1.785507894	Bcer	S	Bcer	FALSE	NA	NA	NA
Bc_ctg1_943	B4	1.785507894	Bcer	S	Bcer	FALSE	NA	NA	NA
Bc_ctg1_944	B4	0.111594243	Bcer	S	Bcer	FALSE	NA	NA	NA
Bc_ctg1_945	B4	4.686958222	Bcer	S	Bcer	FALSE	NA	NA	NA
Bc_ctg1_946	B4	6.24927763	Bcer	S	Bcer	FALSE	NA	NA	NA
Bc_ctg1_947	B4	0.111594243	Bcer	NA	Bcer	FALSE	NA	NA	NA
Bc_ctg1_948	B4	3151.309839	Bcer	C	Bcer	FALSE	NA	NA	NA
Bc_ctg1_949	B4	3432.19255	Bcer	C	Bcer	FALSE	NA	NA	NA
Bc_ctg1_95	B4	35.59856364	Bcer	H	Bcer	FALSE	NA	NA	NA
Bc_ctg1_950	B4	1756.047014	Bcer	C	Bcer	FALSE	NA	NA	NA
Bc_ctg1_951	B4	2800.345944	Bcer	C	Bcer	FALSE	NA	NA	NA
Bc_ctg1_953	B4	164.0435378	Bcer	J	Bcer	FALSE	NA	NA	NA
Bc_ctg1_954	B4	48.32030739	Bcer	S	Bcer	FALSE	NA	NA	NA
Bc_ctg1_955	B4	41.73624703	Bcer	S	Bcer	FALSE	NA	NA	NA
Bc_ctg1_956	B4	1786.17746	Bcer	S	Bcer	FALSE	NA	NA	NA
Bc_ctg1_957	B4	88.7174235	Bcer	P	Bcer	FALSE	NA	NA	NA
Bc_ctg1_958	B4	67.51451725	Bcer	C	Bcer	FALSE	NA	NA	NA
Bc_ctg1_959	B4	150.8754171	Bcer	E	Bcer	FALSE	NA	NA	NA
Bc_ctg1_96	B4	2.566667598	Bcer	T	Bcer	FALSE	NA	NA	NA
Bc_ctg1_960	B4	424.3929076	Bcer	E	Bcer	FALSE	NA	NA	NA
Bc_ctg1_961	B4	124.2043929	Bcer	K	Bcer	FALSE	NA	NA	NA
Bc_ctg1_962	B4	42.62900098	Bcer	S	Bcer	FALSE	NA	NA	NA
Bc_ctg1_963	B4	2.231884868	Bcer	S	Bcer	FALSE	NA	NA	NA

Bc_ctg1_964	B4	1.227536677	Bcer	S	Bcer	FALSE	NA	NA	NA
Bc_ctg1_966	B4	16.73913651	Bcer	S	Bcer	FALSE	NA	NA	NA
Bc_ctg1_967	B4	5.468117926	Bcer	O	Bcer	FALSE	NA	NA	NA
Bc_ctg1_968	B4	5.356523683	Bcer	S	Bcer	FALSE	NA	NA	NA
Bc_ctg1_969	B4	72.75944669	Bcer	T	Bcer	FALSE	NA	NA	NA
Bc_ctg1_97	B4	1.227536677	Bcer	P	Bcer	FALSE	NA	NA	NA
Bc_ctg1_970	B4	116.3927959	Bcer	IQ	Bcer	TRUE	NA	NA	NA
Bc_ctg1_971	B4	1.450725164	Bcer	S	Bcer	FALSE	NA	NA	NA
Bc_ctg1_972	B4	0.33478273	Bcer	NA	Bcer	FALSE	NA	NA	NA
Bc_ctg1_973	B4	183.9073131	Bcer	P	Bcer	FALSE	NA	NA	NA
Bc_ctg1_974	B4	22.09566019	Bcer	FP	Bcer	FALSE	NA	NA	NA
Bc_ctg1_975	B4	59.144949	Bcer	S	Bcer	FALSE	NA	NA	NA
Bc_ctg1_976	B4	35.4869694	Bcer	L	Bcer	FALSE	NA	NA	NA
Bc_ctg1_977	B4	0.892753947	Bcer	T	Bcer	FALSE	NA	NA	NA
Bc_ctg1_978	B4	0	Bcer	S	Bcer	FALSE	Bc_Ga0417192_01_rn09	Other	NA
Bc_ctg1_979	B4	39.16957943	Bcer	NA	Bcer	FALSE	Bc_Ga0417192_01_rn09	Other	NA
Bc_ctg1_98	B4	0.111594243	Bcer	E	Bcer	FALSE	NA	NA	NA
Bc_ctg1_980	B4	59.03335475	Bcer	P	Bcer	FALSE	Bc_Ga0417192_01_rn09	Transport	SMCOG1005
Bc_ctg1_981	B4	16.29275954	Bcer	K	Bcer	FALSE	Bc_Ga0417192_01_rn09	Regulation	SMCOG1278
Bc_ctg1_982	B4	2409.654498	Bcer	E	Bcer	FALSE	Bc_Ga0417192_01_rn09	Other	NA
Bc_ctg1_983	B4	7.476814307	Bcer	S	Bcer	FALSE	Bc_Ga0417192_01_rn09	Other	NA
Bc_ctg1_984	B4	3.459421545	Bcer	M	Bcer	FALSE	Bc_Ga0417192_01_rn09	Other	NA
Bc_ctg1_985	B4	1.339130921	Bcer	S	Bcer	FALSE	Bc_Ga0417192_01_rn09	Other	NA
Bc_ctg1_986	B4	66.17538633	Bcer	T	Bcer	FALSE	Bc_Ga0417192_01_rn09	Regulation	SMCOG1003
Bc_ctg1_987	B4	29.79566299	Bcer	NA	Bcer	FALSE	Bc_Ga0417192_01_rn09	Other	NA
Bc_ctg1_988	B4	96.97539751	Bcer	E	Bcer	FALSE	Bc_Ga0417192_01_rn09	Core biosynthesis	SMCOG1019
Bc_ctg1_989	B4	91.17249685	Bcer	K	Bcer	FALSE	Bc_Ga0417192_01_rn09	Regulation	SMCOG1078
Bc_ctg1_99	B4	0.557971217	Bcer	K	Bcer	FALSE	NA	NA	NA
Bc_ctg1_990	B4	313.0218527	Bcer	P	Bcer	FALSE	Bc_Ga0417192_01_rn09	Transport	SMCOG1069
Bc_ctg1_991	B4	630.0610982	Bcer	P	Bcer	FALSE	Bc_Ga0417192_01_rn09	Transport	SMCOG1067
Bc_ctg1_992	B4	621.8031242	Bcer	G	Bcer	FALSE	Bc_Ga0417192_01_rn09	Ancillary biosynthesis	SMCOG1143
Bc_ctg1_993	B4	46.98117647	Bcer	G	Bcer	FALSE	Bc_Ga0417192_01_rn09	Core biosynthesis	SMCOG1257
Bc_ctg1_994	B4	0.111594243	Bcer	L	Bcer	FALSE	Bc_Ga0417192_01_rn09	Other	SMCOG1026
Bc_ctg1_996	B4	156.1203465	Bcer	G	Bcer	FALSE	Bc_Ga0417192_01_rn09	Core biosynthesis	SMCOG1257
Bc_ctg1_997	B4	299.5189493	Bcer	P	Bcer	FALSE	Bc_Ga0417192_01_rn09	Transport	SMCOG1000
Bc_ctg1_998	B4	207.5652927	Bcer	S	Bcer	FALSE	Bc_Ga0417192_01_rn09	Ancillary biosynthesis	SMCOG1001
Bc_ctg2_1	B4	1.227536677	Bcer	M	Bcer	FALSE	Bc_Ga0417192_02_rn01	Core biosynthesis	None
Bc_ctg2_10	B4	0.781159704	Bcer	S	Bcer	FALSE	Bc_Ga0417192_02_rn01	Other	NA
Bc_ctg2_100	B4	19.86377532	Bcer	K	Bcer	FALSE	Bc_Ga0417192_02_rn02	Ancillary biosynthesis	SMCOG1019
Bc_ctg2_101	B4	20.97971776	Bcer	NA	Bcer	FALSE	Bc_Ga0417192_02_rn02	Other	NA
Bc_ctg2_102	B4	140.4971524	Bcer	E	Bcer	FALSE	Bc_Ga0417192_02_rn02	Transport	SMCOG1202
Bc_ctg2_103	B4	1.450725164	Bcer	M	Bcer	FALSE	Bc_Ga0417192_02_rn02	Ancillary biosynthesis	SMCOG1064
Bc_ctg2_104	B4	1.004348191	Bcer	M	Bcer	FALSE	Bc_Ga0417192_02_rn02	Ancillary biosynthesis	SMCOG1152
Bc_ctg2_105	B4	0.446376974	Bcer	M	Bcer	FALSE	Bc_Ga0417192_02_rn02	Core biosynthesis	SMCOG1010
Bc_ctg2_106	B4	0.446376974	Bcer	M	Bcer	FALSE	Bc_Ga0417192_02_rn02	Core biosynthesis	SMCOG1045
Bc_ctg2_107	B4	0	Bcer	NA	Bcer	FALSE	Bc_Ga0417192_02_rn02	Other	NA
Bc_ctg2_108	B4	0	Bcer	NA	Bcer	FALSE	Bc_Ga0417192_02_rn02	Other	NA
Bc_ctg2_109	B4	0.557971217	Bcer	S	Bcer	FALSE	Bc_Ga0417192_02_rn02	Other	NA
Bc_ctg2_11	B4	0.223188487	Bcer	NA	Bcer	FALSE	Bc_Ga0417192_02_rn01	Other	NA
Bc_ctg2_110	B4	0.223188487	Bcer	M	Bcer	FALSE	Bc_Ga0417192_02_rn02	Core biosynthesis	SMCOG1045
Bc_ctg2_111	B4	0.781159704	Bcer	S	Bcer	FALSE	Bc_Ga0417192_02_rn02	Ancillary biosynthesis	None
Bc_ctg2_116	B4	0.892753947	Bcer	NA	Bcer	FALSE	Bc_Ga0417192_02_rn02	Other	NA
Bc_ctg2_118	B4	3.013044572	Bcer	K	Bcer	FALSE	Bc_Ga0417192_02_rn02	Other	NA
Bc_ctg2_119	B4	18.85942713	Bcer	E	Bcer	FALSE	Bc_Ga0417192_02_rn02	Ancillary biosynthesis	SMCOG1158
Bc_ctg2_12	B4	0	Bcer	NA	Bcer	FALSE	Bc_Ga0417192_02_rn01	Other	NA
Bc_ctg2_120	B4	7.923191281	Bcer	E	Bcer	FALSE	Bc_Ga0417192_02_rn02	Ancillary biosynthesis	SMCOG1081
Bc_ctg2_121	B4	12.05217829	Bcer	K	Bcer	FALSE	Bc_Ga0417192_02_rn02	Other	NA
Bc_ctg2_122	B4	0.111594243	Bcer	NA	Bcer	FALSE	Bc_Ga0417192_02_rn02	Other	NA
Bc_ctg2_123	B4	1.785507894	Bcer	Q	Bcer	TRUE	Bc_Ga0417192_02_rn02	Ancillary biosynthesis	SMCOG1004
Bc_ctg2_124	B4	26.44783568	Bcer	Q	Bcer	TRUE	Bc_Ga0417192_02_rn02	Core biosynthesis	SMCOG1127
Bc_ctg2_125	B4	5.356523683	Bcer	Q	Bcer	TRUE	Bc_Ga0417192_02_rn02	Core biosynthesis	SMCOG1127
Bc_ctg2_126	B4	18.74783289	Bcer	IQ	Bcer	TRUE	Bc_Ga0417192_02_rn02	Core biosynthesis	SMCOG1127

Bc_ctg2_127	B4	3.571015789	Bcer	S	Bcer	FALSE	Bc_Ga0417192_02_rn02	Other	NA
Bc_ctg2_128	B4	67.84929998	Bcer	EGP	Bcer	FALSE	Bc_Ga0417192_02_rn02	Transport	SMCOG1202
Bc_ctg2_129	B4	77.33481067	Bcer	K	Bcer	FALSE	Bc_Ga0417192_02_rn02	Other	NA
Bc_ctg2_130	B4	22.65363141	Bcer	EG	Bcer	FALSE	Bc_Ga0417192_02_rn02	Transport	SMCOG1166
Bc_ctg2_131	B4	7.365220064	Bcer	K	Bcer	FALSE	Bc_Ga0417192_02_rn02	Regulation	SMCOG1071
Bc_ctg2_132	B4	10.37826464	Bcer	EG	Bcer	FALSE	Bc_Ga0417192_02_rn02	Transport	SMCOG1166
Bc_ctg2_133	B4	2.008696381	Bcer	NA	Bcer	FALSE	Bc_Ga0417192_02_rn02	Other	NA
Bc_ctg2_134	B4	4.575363979	Bcer	C	Bcer	FALSE	Bc_Ga0417192_02_rn02	Ancillary biosynthesis	None
Bc_ctg2_135	B4	0.557971217	Bcer	K	Bcer	FALSE	Bc_Ga0417192_02_rn02	Other	NA
Bc_ctg2_136	B4	0.66956546	Bcer	K	Bcer	FALSE	Bc_Ga0417192_02_rn02	Other	NA
Bc_ctg2_137	B4	6.807248847	Bcer	K	Bcer	FALSE	Bc_Ga0417192_02_rn02	Other	NA
Bc_ctg2_139	B4	0.557971217	Bcer	NA	Bcer	FALSE	Bc_Ga0417192_02_rn02	Other	NA
Bc_ctg2_14	B4	37.94204275	Bcer	S	Bcer	FALSE	Bc_Ga0417192_02_rn01	Other	NA
Bc_ctg2_140	B4	0	Bcer	L	Bcer	FALSE	Bc_Ga0417192_02_rn02	Other	SMCOG1132
Bc_ctg2_141	B4	0.66956546	Bcer	L	Bcer	FALSE	Bc_Ga0417192_02_rn02	Other	NA
Bc_ctg2_142	B4	0	Bcer	L	Bcer	FALSE	Bc_Ga0417192_02_rn02	Other	NA
Bc_ctg2_143	B4	16.18116529	Bcer	V	Bcer	FALSE	Bc_Ga0417192_02_rn02	Ancillary biosynthesis	SMCOG1053
Bc_ctg2_144	B4	0	Bcer	NA	Bcer	FALSE	Bc_Ga0417192_02_rn02	Other	NA
Bc_ctg2_145	B4	9.039133715	Bcer	L	Bcer	FALSE	NA	NA	NA
Bc_ctg2_146	B4	0.33478273	Bcer	S	Bcer	FALSE	NA	NA	NA
Bc_ctg2_147	B4	2.901450328	Bcer	NA	Bcer	FALSE	NA	NA	NA
Bc_ctg2_148	B4	36.93769456	Bcer	NA	Bcer	FALSE	NA	NA	NA
Bc_ctg2_149	B4	0.66956546	Bcer	CO	Bcer	FALSE	NA	NA	NA
Bc_ctg2_15	B4	60.59567416	Bcer	P	Bcer	FALSE	Bc_Ga0417192_02_rn01	Ancillary biosynthesis	SMCOG1170
Bc_ctg2_150	B4	0	Bcer	P	Bcer	FALSE	NA	NA	NA
Bc_ctg2_152	B4	0	Bcer	NA	Bcer	FALSE	NA	NA	NA
Bc_ctg2_153	B4	3.236233058	Bcer	L	Bcer	FALSE	NA	NA	NA
Bc_ctg2_154	B4	2.901450328	Bcer	NA	Bcer	FALSE	NA	NA	NA
Bc_ctg2_156	B4	0.557971217	Bcer	NA	Bcer	FALSE	NA	NA	NA
Bc_ctg2_157	B4	0.557971217	Bcer	NA	Bcer	FALSE	NA	NA	NA
Bc_ctg2_158	B4	0	Bcer	L	Bcer	FALSE	NA	NA	NA
Bc_ctg2_159	B4	0.111594243	Bcer	NA	Bcer	FALSE	NA	NA	NA
Bc_ctg2_16	B4	10.26667039	Bcer	NA	Bcer	FALSE	Bc_Ga0417192_02_rn01	Other	NA
Bc_ctg2_160	B4	0.111594243	Bcer	S	Bcer	FALSE	NA	NA	NA
Bc_ctg2_161	B4	0	Bcer	L	Bcer	FALSE	NA	NA	NA
Bc_ctg2_162	B4	0.111594243	Bcer	S	Bcer	FALSE	NA	NA	NA
Bc_ctg2_166	B4	0	Bcer	NA	Bcer	FALSE	NA	NA	NA
Bc_ctg2_167	B4	0	Bcer	S	Bcer	FALSE	NA	NA	NA
Bc_ctg2_169	B4	19.41739835	Bcer	G	Bcer	FALSE	NA	NA	NA
Bc_ctg2_17	B4	0.892753947	Bcer	V	Bcer	FALSE	NA	NA	NA
Bc_ctg2_170	B4	2.455073355	Bcer	K	Bcer	FALSE	NA	NA	NA
Bc_ctg2_171	B4	1.115942434	Bcer	NA	Bcer	FALSE	NA	NA	NA
Bc_ctg2_172	B4	3.013044572	Bcer	O	Bcer	FALSE	NA	NA	NA
Bc_ctg2_173	B4	6.24927763	Bcer	K	Bcer	FALSE	NA	NA	NA
Bc_ctg2_174	B4	36.49131759	Bcer	V	Bcer	FALSE	NA	NA	NA
Bc_ctg2_176	B4	2.678261841	Bcer	NA	Bcer	FALSE	NA	NA	NA
Bc_ctg2_177	B4	0.111594243	Bcer	NA	Bcer	FALSE	NA	NA	NA
Bc_ctg2_178	B4	16.73913651	Bcer	V	Bcer	FALSE	NA	NA	NA
Bc_ctg2_179	B4	0.223188487	Bcer	H	Bcer	FALSE	NA	NA	NA
Bc_ctg2_18	B4	11.94058404	Bcer	V	Bcer	FALSE	NA	NA	NA
Bc_ctg2_180	B4	0.557971217	Bcer	NA	Bcer	FALSE	NA	NA	NA
Bc_ctg2_181	B4	1.339130921	Bcer	S	Bcer	FALSE	NA	NA	NA
Bc_ctg2_182	B4	0.66956546	Bcer	NA	Bcer	FALSE	NA	NA	NA
Bc_ctg2_184	B4	0	Bcer	NA	Bcer	FALSE	NA	NA	NA
Bc_ctg2_185	B4	0.66956546	Bcer	L	Bcer	FALSE	NA	NA	NA
Bc_ctg2_186	B4	2.231884868	Bcer	L	Bcer	FALSE	NA	NA	NA
Bc_ctg2_187	B4	0	Bcer	S	Bcer	FALSE	NA	NA	NA
Bc_ctg2_188	B4	2.008696381	Bcer	NA	Bcer	FALSE	NA	NA	NA
Bc_ctg2_189	B4	4.240581249	Bcer	L	Bcer	FALSE	NA	NA	NA
Bc_ctg2_19	B4	7.030437334	Bcer	T	Bcer	FALSE	NA	NA	NA
Bc_ctg2_190	B4	1.004348191	Bcer	NA	Bcer	FALSE	NA	NA	NA
Bc_ctg2_191	B4	9.485510688	Bcer	NA	Bcer	FALSE	NA	NA	NA

Bc_ctg2_192	B4	69.85799636	Bcer	S	Bcer	FALSE	NA	NA	NA
Bc_ctg2_193	B4	1.673913651	Bcer	S	Bcer	FALSE	NA	NA	NA
Bc_ctg2_194	B4	0	Bcer	CO	Bcer	FALSE	NA	NA	NA
Bc_ctg2_195	B4	0.557971217	Bcer	P	Bcer	FALSE	NA	NA	NA
Bc_ctg2_196	B4	4.463769736	Bcer	NA	Bcer	FALSE	NA	NA	NA
Bc_ctg2_197	B4	2.231884868	Bcer	V	Bcer	FALSE	NA	NA	NA
Bc_ctg2_198	B4	0.111594243	Bcer	S	Bcer	FALSE	NA	NA	NA
Bc_ctg2_199	B4	0.111594243	Bcer	NA	Bcer	FALSE	NA	NA	NA
Bc_ctg2_2	B4	0.111594243	Bcer	M	Bcer	FALSE	Bc_Ga0417192_02_rn01	Core biosynthesis	None
Bc_ctg2_20	B4	15.40000559	Bcer	T	Bcer	FALSE	NA	NA	NA
Bc_ctg2_200	B4	1.673913651	Bcer	NA	Bcer	FALSE	NA	NA	NA
Bc_ctg2_201	B4	6.026089143	Bcer	M	Bcer	FALSE	NA	NA	NA
Bc_ctg2_202	B4	2.678261841	Bcer	NA	Bcer	FALSE	NA	NA	NA
Bc_ctg2_203	B4	0.33478273	Bcer	U	Bcer	FALSE	NA	NA	NA
Bc_ctg2_204	B4	0	Bcer	NA	Bcer	FALSE	NA	NA	NA
Bc_ctg2_205	B4	0	Bcer	NA	Bcer	FALSE	NA	NA	NA
Bc_ctg2_206	B4	0	Bcer	NA	Bcer	FALSE	NA	NA	NA
Bc_ctg2_207	B4	0	Bcer	NA	Bcer	FALSE	NA	NA	NA
Bc_ctg2_208	B4	0.446376974	Bcer	NA	Bcer	FALSE	NA	NA	NA
Bc_ctg2_209	B4	0	Bcer	NA	Bcer	FALSE	NA	NA	NA
Bc_ctg2_21	B4	10.26667039	Bcer	T	Bcer	FALSE	NA	NA	NA
Bc_ctg2_210	B4	0	Bcer	L	Bcer	FALSE	NA	NA	NA
Bc_ctg2_211	B4	0	Bcer	NA	Bcer	FALSE	NA	NA	NA
Bc_ctg2_212	B4	0.446376974	Bcer	S	Bcer	FALSE	NA	NA	NA
Bc_ctg2_213	B4	0	Bcer	NA	Bcer	FALSE	NA	NA	NA
Bc_ctg2_214	B4	22.09566019	Bcer	S	Bcer	FALSE	NA	NA	NA
Bc_ctg2_215	B4	3.013044572	Bcer	M	Bcer	FALSE	NA	NA	NA
Bc_ctg2_216	B4	0.33478273	Bcer	S	Bcer	FALSE	NA	NA	NA
Bc_ctg2_217	B4	3.459421545	Bcer	S	Bcer	FALSE	NA	NA	NA
Bc_ctg2_218	B4	0.446376974	Bcer	S	Bcer	FALSE	NA	NA	NA
Bc_ctg2_22	B4	4.352175492	Bcer	T	Bcer	FALSE	NA	NA	NA
Bc_ctg2_220	B4	0.223188487	Bcer	NA	Bcer	FALSE	NA	NA	NA
Bc_ctg2_221	B4	0.781159704	Bcer	NA	Bcer	FALSE	NA	NA	NA
Bc_ctg2_223	B4	1.339130921	Bcer	NA	Bcer	FALSE	NA	NA	NA
Bc_ctg2_224	B4	1.339130921	Bcer	NA	Bcer	FALSE	NA	NA	NA
Bc_ctg2_225	B4	1.227536677	Bcer	NA	Bcer	FALSE	NA	NA	NA
Bc_ctg2_226	B4	0	Bcer	NA	Bcer	FALSE	NA	NA	NA
Bc_ctg2_227	B4	0.446376974	Bcer	NA	Bcer	FALSE	NA	NA	NA
Bc_ctg2_228	B4	0.223188487	Bcer	NA	Bcer	FALSE	NA	NA	NA
Bc_ctg2_229	B4	0.223188487	Bcer	NA	Bcer	FALSE	NA	NA	NA
Bc_ctg2_23	B4	2.343479111	Bcer	S	Bcer	FALSE	NA	NA	NA
Bc_ctg2_230	B4	0.557971217	Bcer	NA	Bcer	FALSE	NA	NA	NA
Bc_ctg2_231	B4	0	Bcer	NA	Bcer	FALSE	NA	NA	NA
Bc_ctg2_232	B4	0.223188487	Bcer	U	Bcer	FALSE	NA	NA	NA
Bc_ctg2_233	B4	0.111594243	Bcer	D	Bcer	FALSE	NA	NA	NA
Bc_ctg2_234	B4	0	Bcer	U	Bcer	FALSE	NA	NA	NA
Bc_ctg2_235	B4	0	Bcer	NA	Bcer	FALSE	NA	NA	NA
Bc_ctg2_236	B4	2.901450328	Bcer	M	Bcer	FALSE	NA	NA	NA
Bc_ctg2_237	B4	18.41305016	Bcer	GM	Bcer	FALSE	NA	NA	NA
Bc_ctg2_238	B4	0	Bcer	NA	Bcer	FALSE	NA	NA	NA
Bc_ctg2_239	B4	0	Bcer	NA	Bcer	FALSE	NA	NA	NA
Bc_ctg2_24	B4	6.24927763	Bcer	V	Bcer	FALSE	NA	NA	NA
Bc_ctg2_240	B4	1.227536677	Bcer	D	Bcer	FALSE	NA	NA	NA
Bc_ctg2_241	B4	0.33478273	Bcer	NA	Bcer	FALSE	NA	NA	NA
Bc_ctg2_242	B4	0	Bcer	NA	Bcer	FALSE	NA	NA	NA
Bc_ctg2_243	B4	85.25800195	Bcer	NA	Bcer	FALSE	NA	NA	NA
Bc_ctg2_244	B4	177.2116585	Bcer	D	Bcer	FALSE	NA	NA	NA
Bc_ctg2_245	B4	19.75218108	Bcer	NA	Bcer	FALSE	NA	NA	NA
Bc_ctg2_246	B4	9.373916445	Bcer	L	Bcer	FALSE	NA	NA	NA
Bc_ctg2_247	B4	11.27101858	Bcer	NA	Bcer	FALSE	NA	NA	NA
Bc_ctg2_248	B4	0.111594243	Bcer	NA	Bcer	FALSE	NA	NA	NA
Bc_ctg2_249	B4	18.41305016	Bcer	U	Bcer	FALSE	NA	NA	NA

Bc_ctg2_25	B4	40.62030459	Bcer	V	Bcer	FALSE	NA	NA	NA
Bc_ctg2_250	B4	0.66956546	Bcer	J	Bcer	FALSE	NA	NA	NA
Bc_ctg2_251	B4	3.571015789	Bcer	NA	Bcer	FALSE	NA	NA	NA
Bc_ctg2_252	B4	7.25362582	Bcer	NA	Bcer	FALSE	NA	NA	NA
Bc_ctg2_253	B4	0	Bcer	NA	Bcer	FALSE	NA	NA	NA
Bc_ctg2_254	B4	1.450725164	Bcer	NA	Bcer	FALSE	NA	NA	NA
Bc_ctg2_255	B4	37.71885427	Bcer	S	Bcer	FALSE	NA	NA	NA
Bc_ctg2_256	B4	1.450725164	Bcer	NA	Bcer	FALSE	NA	NA	NA
Bc_ctg2_257	B4	0	Bcer	NA	Bcer	FALSE	NA	NA	NA
Bc_ctg2_259	B4	0	Bcer	NA	Bcer	FALSE	NA	NA	NA
Bc_ctg2_26	B4	9.597104932	Bcer	NA	Bcer	FALSE	NA	NA	NA
Bc_ctg2_260	B4	6.137683386	Bcer	NA	Bcer	FALSE	NA	NA	NA
Bc_ctg2_261	B4	4.798552466	Bcer	NA	Bcer	FALSE	NA	NA	NA
Bc_ctg2_262	B4	63.60871873	Bcer	K	Bcer	FALSE	NA	NA	NA
Bc_ctg2_263	B4	14.3956574	Bcer	S	Bcer	FALSE	NA	NA	NA
Bc_ctg2_264	B4	392.8117367	Bcer	S	Bcer	FALSE	NA	NA	NA
Bc_ctg2_265	B4	643.4524074	Bcer	E	Bcer	FALSE	NA	NA	NA
Bc_ctg2_266	B4	714.5379404	Bcer	E	Bcer	FALSE	NA	NA	NA
Bc_ctg2_267	B4	0	Bcer	NA	Bcer	FALSE	NA	NA	NA
Bc_ctg2_268	B4	0	Bcer	L	Bcer	FALSE	NA	NA	NA
Bc_ctg2_269	B4	0	Bcer	NA	Bcer	FALSE	NA	NA	NA
Bc_ctg2_27	B4	2.343479111	Bcer	NA	Bcer	FALSE	NA	NA	NA
Bc_ctg2_270	B4	63.05074752	Bcer	S	Bcer	FALSE	NA	NA	NA
Bc_ctg2_271	B4	31.46957664	Bcer	S	Bcer	FALSE	NA	NA	NA
Bc_ctg2_272	B4	5.9144949	Bcer	EH	Bcer	FALSE	NA	NA	NA
Bc_ctg2_273	B4	7.811597037	Bcer	NA	Bcer	FALSE	NA	NA	NA
Bc_ctg2_275	B4	18.18986167	Bcer	S	Bcer	FALSE	NA	NA	NA
Bc_ctg2_276	B4	3.236233058	Bcer	L	Bcer	FALSE	NA	NA	NA
Bc_ctg2_278	B4	30.24203996	Bcer	NA	Bcer	FALSE	NA	NA	NA
Bc_ctg2_279	B4	101.1043845	Bcer	K	Bcer	FALSE	NA	NA	NA
Bc_ctg2_28	B4	3.794204275	Bcer	EGP	Bcer	FALSE	NA	NA	NA
Bc_ctg2_280	B4	0.557971217	Bcer	S	Bcer	FALSE	NA	NA	NA
Bc_ctg2_281	B4	13.61449769	Bcer	O	Bcer	FALSE	NA	NA	NA
Bc_ctg2_282	B4	0.33478273	Bcer	S	Bcer	FALSE	NA	NA	NA
Bc_ctg2_283	B4	41.28987005	Bcer	S	Bcer	FALSE	NA	NA	NA
Bc_ctg2_284	B4	46.98117647	Bcer	S	Bcer	FALSE	NA	NA	NA
Bc_ctg2_285	B4	3.682610032	Bcer	NA	Bcer	FALSE	NA	NA	NA
Bc_ctg2_286	B4	3.459421545	Bcer	S	Bcer	FALSE	NA	NA	NA
Bc_ctg2_287	B4	9.150727958	Bcer	NT	Bcer	FALSE	NA	NA	NA
Bc_ctg2_288	B4	0.33478273	Bcer	S	Bcer	FALSE	NA	NA	NA
Bc_ctg2_289	B4	43.07537795	Bcer	NA	Bcer	FALSE	NA	NA	NA
Bc_ctg2_29	B4	2.008696381	Bcer	M	Bcer	FALSE	NA	NA	NA
Bc_ctg2_290	B4	0	Bcer	NA	Bcer	FALSE	NA	NA	NA
Bc_ctg2_291	B4	18.07826743	Bcer	K	Bcer	FALSE	NA	NA	NA
Bc_ctg2_292	B4	0.111594243	Bcer	S	Bcer	FALSE	NA	NA	NA
Bc_ctg2_293	B4	3.905798519	Bcer	O	Bcer	FALSE	NA	NA	NA
Bc_ctg2_294	B4	1.450725164	Bcer	S	Bcer	FALSE	NA	NA	NA
Bc_ctg2_295	B4	3.347827302	Bcer	S	Bcer	FALSE	NA	NA	NA
Bc_ctg2_296	B4	0	Bcer	NA	Bcer	FALSE	NA	NA	NA
Bc_ctg2_297	B4	4.463769736	Bcer	V	Bcer	FALSE	NA	NA	NA
Bc_ctg2_298	B4	1.339130921	Bcer	NA	Bcer	FALSE	NA	NA	NA
Bc_ctg2_299	B4	3.013044572	Bcer	S	Bcer	FALSE	NA	NA	NA
Bc_ctg2_3	B4	0	Bcer	M	Bcer	FALSE	Bc_Ga0417192_02_rn01	Core biosynthesis	SMCOG1010
Bc_ctg2_30	B4	2.678261841	Bcer	S	Bcer	FALSE	NA	NA	NA
Bc_ctg2_300	B4	15.9579768	Bcer	L	Bcer	FALSE	NA	NA	NA
Bc_ctg2_303	B4	3.682610032	Bcer	O	Bcer	FALSE	NA	NA	NA
Bc_ctg2_304	B4	0	Bcer	NA	Bcer	FALSE	NA	NA	NA
Bc_ctg2_305	B4	1.562319407	Bcer	DZ	Bcer	FALSE	NA	NA	NA
Bc_ctg2_307	B4	0.223188487	Bcer	O	Bcer	FALSE	NA	NA	NA
Bc_ctg2_309	B4	0.111594243	Bcer	NA	Bcer	FALSE	NA	NA	NA
Bc_ctg2_31	B4	3.124638815	Bcer	NA	Bcer	FALSE	NA	NA	NA
Bc_ctg2_310	B4	0.892753947	Bcer	GM	Bcer	FALSE	NA	NA	NA

Bc_ctg2_311	B4	5.021740953	Bcer	E	Bcer	FALSE	NA	NA	NA
Bc_ctg2_312	B4	3.571015789	Bcer	K	Bcer	FALSE	NA	NA	NA
Bc_ctg2_313	B4	0.446376974	Bcer	K	Bcer	FALSE	NA	NA	NA
Bc_ctg2_314	B4	3.236233058	Bcer	L	Bcer	FALSE	NA	NA	NA
Bc_ctg2_315	B4	0.33478273	Bcer	S	Bcer	FALSE	NA	NA	NA
Bc_ctg2_316	B4	591.2263015	Bcer	M	Bcer	FALSE	NA	NA	NA
Bc_ctg2_317	B4	64.94784965	Bcer	M	Bcer	FALSE	NA	NA	NA
Bc_ctg2_318	B4	70.86234455	Bcer	S	Bcer	FALSE	NA	NA	NA
Bc_ctg2_319	B4	6.58406036	Bcer	S	Bcer	FALSE	NA	NA	NA
Bc_ctg2_32	B4	0.223188487	Bcer	C	Bcer	FALSE	NA	NA	NA
Bc_ctg2_320	B4	108.0232276	Bcer	S	Bcer	FALSE	NA	NA	NA
Bc_ctg2_322	B4	0	Bcer	NA	Bcer	FALSE	NA	NA	NA
Bc_ctg2_323	B4	11.49420707	Bcer	NA	Bcer	FALSE	NA	NA	NA
Bc_ctg2_324	B4	0	Bcer	NA	Bcer	FALSE	NA	NA	NA
Bc_ctg2_325	B4	0	Bcer	NA	Bcer	FALSE	NA	NA	NA
Bc_ctg2_326	B4	0	Bcer	L	Bcer	FALSE	NA	NA	NA
Bc_ctg2_327	B4	0.446376974	Bcer	NA	Bcer	FALSE	NA	NA	NA
Bc_ctg2_328	B4	0.446376974	Bcer	H	Bcer	FALSE	NA	NA	NA
Bc_ctg2_329	B4	0	Bcer	NA	Bcer	FALSE	NA	NA	NA
Bc_ctg2_33	B4	4.017392762	Bcer	IQ	Bcer	TRUE	NA	NA	NA
Bc_ctg2_330	B4	0.446376974	Bcer	T	Bcer	FALSE	NA	NA	NA
Bc_ctg2_331	B4	5.356523683	Bcer	T	Bcer	FALSE	NA	NA	NA
Bc_ctg2_332	B4	35.37537515	Bcer	M	Bcer	FALSE	NA	NA	NA
Bc_ctg2_333	B4	9.262322201	Bcer	K	Bcer	FALSE	NA	NA	NA
Bc_ctg2_334	B4	0.223188487	Bcer	E	Bcer	FALSE	NA	NA	NA
Bc_ctg2_335	B4	2.343479111	Bcer	NA	Bcer	FALSE	NA	NA	NA
Bc_ctg2_336	B4	7.25362582	Bcer	G	Bcer	FALSE	NA	NA	NA
Bc_ctg2_337	B4	4.910146709	Bcer	K	Bcer	FALSE	NA	NA	NA
Bc_ctg2_338	B4	15.84638256	Bcer	E	Bcer	FALSE	NA	NA	NA
Bc_ctg2_339	B4	0.781159704	Bcer	K	Bcer	FALSE	NA	NA	NA
Bc_ctg2_34	B4	0.33478273	Bcer	S	Bcer	FALSE	NA	NA	NA
Bc_ctg2_340	B4	2.231884868	Bcer	S	Bcer	FALSE	NA	NA	NA
Bc_ctg2_341	B4	0	Bcer	K	Bcer	FALSE	NA	NA	NA
Bc_ctg2_342	B4	1.785507894	Bcer	E	Bcer	FALSE	NA	NA	NA
Bc_ctg2_343	B4	0.446376974	Bcer	K	Bcer	FALSE	NA	NA	NA
Bc_ctg2_344	B4	8.481162498	Bcer	S	Bcer	FALSE	NA	NA	NA
Bc_ctg2_345	B4	47.76233617	Bcer	K	Bcer	FALSE	NA	NA	NA
Bc_ctg2_346	B4	2.231884868	Bcer	L	Bcer	FALSE	NA	NA	NA
Bc_ctg2_347	B4	6.91884309	Bcer	L	Bcer	FALSE	NA	NA	NA
Bc_ctg2_348	B4	3.013044572	Bcer	NA	Bcer	FALSE	NA	NA	NA
Bc_ctg2_349	B4	14.73044013	Bcer	L	Bcer	FALSE	NA	NA	NA
Bc_ctg2_35	B4	0.781159704	Bcer	NA	Bcer	FALSE	NA	NA	NA
Bc_ctg2_350	B4	7.923191281	Bcer	L	Bcer	FALSE	NA	NA	NA
Bc_ctg2_351	B4	0.446376974	Bcer	K	Bcer	FALSE	NA	NA	NA
Bc_ctg2_352	B4	1.673913651	Bcer	NA	Bcer	FALSE	NA	NA	NA
Bc_ctg2_353	B4	10.60145312	Bcer	NA	Bcer	FALSE	NA	NA	NA
Bc_ctg2_354	B4	10.37826464	Bcer	S	Bcer	FALSE	NA	NA	NA
Bc_ctg2_355	B4	9.708699175	Bcer	NA	Bcer	FALSE	NA	NA	NA
Bc_ctg2_356	B4	1.339130921	Bcer	NA	Bcer	FALSE	NA	NA	NA
Bc_ctg2_359	B4	6.695654603	Bcer	CH	Bcer	FALSE	NA	NA	NA
Bc_ctg2_36	B4	0	Bcer	NA	Bcer	FALSE	NA	NA	NA
Bc_ctg2_360	B4	4.575363979	Bcer	L	Bcer	FALSE	NA	NA	NA
Bc_ctg2_361	B4	1.227536677	Bcer	NA	Bcer	FALSE	NA	NA	NA
Bc_ctg2_362	B4	9.597104932	Bcer	KT	Bcer	FALSE	NA	NA	NA
Bc_ctg2_363	B4	18.18986167	Bcer	K	Bcer	FALSE	NA	NA	NA
Bc_ctg2_364	B4	11.15942434	Bcer	P	Bcer	FALSE	NA	NA	NA
Bc_ctg2_365	B4	1.227536677	Bcer	NA	Bcer	FALSE	NA	NA	NA
Bc_ctg2_366	B4	13.83768618	Bcer	S	Bcer	FALSE	NA	NA	NA
Bc_ctg2_367	B4	8.481162498	Bcer	L	Bcer	FALSE	NA	NA	NA
Bc_ctg2_368	B4	14.3956574	Bcer	S	Bcer	FALSE	NA	NA	NA
Bc_ctg2_369	B4	0.111594243	Bcer	NA	Bcer	FALSE	NA	NA	NA
Bc_ctg2_37	B4	1.227536677	Bcer	NA	Bcer	FALSE	NA	NA	NA

Bc_ctg2_370	B4	1.227536677	Bcer	P	Bcer	FALSE	NA	NA	NA
Bc_ctg2_371	B4	0.223188487	Bcer	NA	Bcer	FALSE	NA	NA	NA
Bc_ctg2_372	B4	17.29710773	Bcer	S	Bcer	FALSE	NA	NA	NA
Bc_ctg2_373	B4	15.51159983	Bcer	NA	Bcer	FALSE	NA	NA	NA
Bc_ctg2_374	B4	0	Bcer	NA	Bcer	FALSE	NA	NA	NA
Bc_ctg2_375	B4	2.120290624	Bcer	NA	Bcer	FALSE	NA	NA	NA
Bc_ctg2_376	B4	44.52610311	Bcer	LO	Bcer	FALSE	NA	NA	NA
Bc_ctg2_377	B4	18.85942713	Bcer	L	Bcer	FALSE	NA	NA	NA
Bc_ctg2_378	B4	35.15218667	Bcer	V	Bcer	FALSE	NA	NA	NA
Bc_ctg2_379	B4	19.19420986	Bcer	V	Bcer	FALSE	NA	NA	NA
Bc_ctg2_38	B4	1.897102138	Bcer	NA	Bcer	FALSE	NA	NA	NA
Bc_ctg2_380	B4	6.695654603	Bcer	NA	Bcer	FALSE	NA	NA	NA
Bc_ctg2_381	B4	30.46522845	Bcer	NA	Bcer	FALSE	NA	NA	NA
Bc_ctg2_382	B4	1.562319407	Bcer	NA	Bcer	FALSE	NA	NA	NA
Bc_ctg2_383	B4	0.111594243	Bcer	NA	Bcer	FALSE	NA	NA	NA
Bc_ctg2_384	B4	95.4130781	Bcer	D	Bcer	FALSE	NA	NA	NA
Bc_ctg2_385	B4	27.0058069	Bcer	NA	Bcer	FALSE	NA	NA	NA
Bc_ctg2_386	B4	113.0449686	Bcer	NA	Bcer	FALSE	NA	NA	NA
Bc_ctg2_387	B4	1.897102138	Bcer	NA	Bcer	FALSE	NA	NA	NA
Bc_ctg2_388	B4	6.137683386	Bcer	NA	Bcer	FALSE	NA	NA	NA
Bc_ctg2_389	B4	32.25073634	Bcer	NA	Bcer	FALSE	Bc_Ga0417192_02_rn03	Other	NA
Bc_ctg2_39	B4	36.2681291	Bcer	NA	Bcer	FALSE	NA	NA	NA
Bc_ctg2_390	B4	61.48842811	Bcer	NA	Bcer	FALSE	Bc_Ga0417192_02_rn03	Other	NA
Bc_ctg2_391	B4	201.5392036	Bcer	NA	Bcer	FALSE	Bc_Ga0417192_02_rn03	Other	NA
Bc_ctg2_392	B4	115.1652592	Bcer	T	Bcer	FALSE	Bc_Ga0417192_02_rn03	Other	NA
Bc_ctg2_393	B4	47.98552466	Bcer	NA	Bcer	FALSE	Bc_Ga0417192_02_rn03	Other	NA
Bc_ctg2_394	B4	114.3840995	Bcer	L	Bcer	FALSE	Bc_Ga0417192_02_rn03	Other	SMCOG1077
Bc_ctg2_395	B4	8.592756741	Bcer	K	Bcer	FALSE	Bc_Ga0417192_02_rn03	Other	SMCOG1148
Bc_ctg2_396	B4	0.446376974	Bcer	K	Bcer	FALSE	Bc_Ga0417192_02_rn03	Other	NA
Bc_ctg2_397	B4	0.111594243	Bcer	NA	Bcer	FALSE	Bc_Ga0417192_02_rn03	Other	NA
Bc_ctg2_398	B4	44.07972614	Bcer	EGP	Bcer	FALSE	Bc_Ga0417192_02_rn03	Transport	SMCOG1005
Bc_ctg2_399	B4	24.21595082	Bcer	UW	Bcer	FALSE	Bc_Ga0417192_02_rn03	Other	NA
Bc_ctg2_4	B4	1.004348191	Bcer	M	Bcer	FALSE	Bc_Ga0417192_02_rn01	Core biosynthesis	SMCOG1010
Bc_ctg2_40	B4	25.55508174	Bcer	L	Bcer	FALSE	NA	NA	NA
Bc_ctg2_400	B4	0	Bcer	NA	Bcer	FALSE	Bc_Ga0417192_02_rn03	Other	NA
Bc_ctg2_401	B4	1.897102138	Bcer	S	Bcer	FALSE	Bc_Ga0417192_02_rn03	Other	NA
Bc_ctg2_402	B4	0.66956546	Bcer	NA	Bcer	FALSE	Bc_Ga0417192_02_rn03	Other	NA
Bc_ctg2_403	B4	36.93769456	Bcer	NA	Bcer	FALSE	Bc_Ga0417192_02_rn03	Other	NA
Bc_ctg2_404	B4	1.785507894	Bcer	CO	Bcer	FALSE	Bc_Ga0417192_02_rn03	Other	NA
Bc_ctg2_405	B4	16.06957105	Bcer	NA	Bcer	FALSE	Bc_Ga0417192_02_rn03	Other	NA
Bc_ctg2_406	B4	28.01015509	Bcer	E	Bcer	FALSE	Bc_Ga0417192_02_rn03	Ancillary biosynthesis	SMCOG1180
Bc_ctg2_407	B4	0	Bcer	C	Bcer	FALSE	Bc_Ga0417192_02_rn03	Ancillary biosynthesis	SMCOG1039
Bc_ctg2_408	B4	154.8928098	Bcer	Q	Bcer	TRUE	Bc_Ga0417192_02_rn03	Core biosynthesis	SMCOG1127
Bc_ctg2_409	B4	0.223188487	Bcer	NA	Bcer	FALSE	Bc_Ga0417192_02_rn03	Other	NA
Bc_ctg2_41	B4	0	Bcer	NA	Bcer	FALSE	NA	NA	NA
Bc_ctg2_410	B4	4.686958222	Bcer	NA	Bcer	FALSE	Bc_Ga0417192_02_rn03	Other	NA
Bc_ctg2_411	B4	14.84203437	Bcer	NA	Bcer	FALSE	Bc_Ga0417192_02_rn03	Other	NA
Bc_ctg2_412	B4	30.46522845	Bcer	NA	Bcer	FALSE	Bc_Ga0417192_02_rn03	Other	NA
Bc_ctg2_413	B4	4.463769736	Bcer	NA	Bcer	FALSE	Bc_Ga0417192_02_rn03	Other	NA
Bc_ctg2_414	B4	0.66956546	Bcer	L	Bcer	FALSE	Bc_Ga0417192_02_rn03	Other	NA
Bc_ctg2_415	B4	2.343479111	Bcer	L	Bcer	FALSE	Bc_Ga0417192_02_rn03	Other	NA
Bc_ctg2_416	B4	2.901450328	Bcer	G	Bcer	FALSE	Bc_Ga0417192_02_rn03	Ancillary biosynthesis	SMCOG1101
Bc_ctg2_417	B4	0.223188487	Bcer	NA	Bcer	FALSE	Bc_Ga0417192_02_rn03	Other	NA
Bc_ctg2_418	B4	2.343479111	Bcer	NA	Bcer	FALSE	Bc_Ga0417192_02_rn03	Other	NA
Bc_ctg2_419	B4	6.91884309	Bcer	ST	Bcer	FALSE	Bc_Ga0417192_02_rn03	Other	NA
Bc_ctg2_42	B4	0.223188487	Bcer	NA	Bcer	FALSE	NA	NA	NA
Bc_ctg2_420	B4	23.99276233	Bcer	M	Bcer	FALSE	Bc_Ga0417192_02_rn03	Other	NA
Bc_ctg2_421	B4	1.450725164	Bcer	S	Bcer	FALSE	Bc_Ga0417192_02_rn03	Other	NA
Bc_ctg2_422	B4	8.034785524	Bcer	Q	Bcer	TRUE	Bc_Ga0417192_02_rn03	Core biosynthesis	SMCOG1022
Bc_ctg2_423	B4	27.56377812	Bcer	Q	Bcer	TRUE	Bc_Ga0417192_02_rn03	Core biosynthesis	SMCOG1127
Bc_ctg2_424	B4	10.04348191	Bcer	Q	Bcer	TRUE	Bc_Ga0417192_02_rn03	Core biosynthesis	SMCOG1127
Bc_ctg2_425	B4	0.892753947	Bcer	IQ	Bcer	TRUE	Bc_Ga0417192_02_rn03	Ancillary biosynthesis	SMCOG1147

Bc_ctg2_426	B4	17.52029621	Bcer	I	Bcer	FALSE	Bc_Ga0417192_02_rn03	Ancillary biosynthesis	SMCOG1006
Bc_ctg2_427	B4	19.97536957	Bcer	J	Bcer	FALSE	Bc_Ga0417192_02_rn03	Other	NA
Bc_ctg2_428	B4	14.95362861	Bcer	I	Bcer	FALSE	Bc_Ga0417192_02_rn03	Ancillary biosynthesis	SMCOG1021
Bc_ctg2_429	B4	7.811597037	Bcer	I	Bcer	FALSE	Bc_Ga0417192_02_rn03	Ancillary biosynthesis	SMCOG1095
Bc_ctg2_430	B4	0.33478273	Bcer	IQ	Bcer	TRUE	Bc_Ga0417192_02_rn03	Ancillary biosynthesis	SMCOG1147
Bc_ctg2_431	B4	5.691306413	Bcer	I	Bcer	FALSE	Bc_Ga0417192_02_rn03	Ancillary biosynthesis	SMCOG1006
Bc_ctg2_432	B4	6.360871873	Bcer	Q	Bcer	TRUE	Bc_Ga0417192_02_rn03	Core biosynthesis	SMCOG1002
Bc_ctg2_433	B4	48.87827861	Bcer	Q	Bcer	TRUE	Bc_Ga0417192_02_rn03	Core biosynthesis	SMCOG1127
Bc_ctg2_434	B4	7.588408551	Bcer	C	Bcer	FALSE	Bc_Ga0417192_02_rn03	Ancillary biosynthesis	SMCOG1083
Bc_ctg2_435	B4	16.40435378	Bcer	V	Bcer	FALSE	Bc_Ga0417192_02_rn03	Ancillary biosynthesis	SMCOG1053
Bc_ctg2_436	B4	3.124638815	Bcer	Q	Bcer	TRUE	Bc_Ga0417192_02_rn03	Ancillary biosynthesis	SMCOG1256
Bc_ctg2_437	B4	15.40000559	Bcer	Q	Bcer	TRUE	Bc_Ga0417192_02_rn03	Core biosynthesis	SMCOG1002
Bc_ctg2_438	B4	3.347827302	Bcer	Q	Bcer	TRUE	Bc_Ga0417192_02_rn03	Ancillary biosynthesis	SMCOG1004
Bc_ctg2_439	B4	35.82175213	Bcer	Q	Bcer	TRUE	Bc_Ga0417192_02_rn03	Core biosynthesis	SMCOG1127
Bc_ctg2_440	B4	1.785507894	Bcer	H	Bcer	FALSE	Bc_Ga0417192_02_rn03	Ancillary biosynthesis	SMCOG1012
Bc_ctg2_441	B4	16.40435378	Bcer	K	Bcer	FALSE	Bc_Ga0417192_02_rn03	Regulation	SMCOG1078
Bc_ctg2_442	B4	1.785507894	Bcer	E	Bcer	FALSE	Bc_Ga0417192_02_rn03	Ancillary biosynthesis	SMCOG1056
Bc_ctg2_443	B4	5.691306413	Bcer	S	Bcer	FALSE	Bc_Ga0417192_02_rn03	Ancillary biosynthesis	SMCOG1179
Bc_ctg2_444	B4	6.472466117	Bcer	S	Bcer	FALSE	Bc_Ga0417192_02_rn03	Ancillary biosynthesis	SMCOG1079
Bc_ctg2_445	B4	2.455073355	Bcer	G	Bcer	FALSE	Bc_Ga0417192_02_rn03	Other	NA
Bc_ctg2_446	B4	2.789856085	Bcer	E	Bcer	FALSE	Bc_Ga0417192_02_rn03	Ancillary biosynthesis	SMCOG1114
Bc_ctg2_447	B4	2.455073355	Bcer	E	Bcer	FALSE	Bc_Ga0417192_02_rn03	Ancillary biosynthesis	SMCOG1081
Bc_ctg2_448	B4	6.24927763	Bcer	E	Bcer	FALSE	Bc_Ga0417192_02_rn03	Ancillary biosynthesis	SMCOG1158
Bc_ctg2_449	B4	4.463769736	Bcer	K	Bcer	FALSE	Bc_Ga0417192_02_rn03	Other	NA
Bc_ctg2_45	B4	0	Bcer	NA	Bcer	FALSE	NA	NA	NA
Bc_ctg2_450	B4	0.33478273	Bcer	NA	Bcer	FALSE	Bc_Ga0417192_02_rn03	Other	NA
Bc_ctg2_451	B4	54.34639653	Bcer	NA	Bcer	FALSE	Bc_Ga0417192_02_rn03	Other	NA
Bc_ctg2_453	B4	0.66956546	Bcer	NA	Bcer	FALSE	Bc_Ga0417192_02_rn03	Other	NA
Bc_ctg2_454	B4	0	Bcer	L	Bcer	FALSE	Bc_Ga0417192_02_rn03	Other	NA
Bc_ctg2_455	B4	0	Bcer	NA	Bcer	FALSE	Bc_Ga0417192_02_rn03	Other	NA
Bc_ctg2_456	B4	1.673913651	Bcer	NA	Bcer	FALSE	Bc_Ga0417192_02_rn03	Other	NA
Bc_ctg2_457	B4	8.481162498	Bcer	NA	Bcer	FALSE	Bc_Ga0417192_02_rn03	Other	NA
Bc_ctg2_458	B4	30.46522845	Bcer	S	Bcer	FALSE	Bc_Ga0417192_02_rn03	Other	NA
Bc_ctg2_459	B4	0.557971217	Bcer	E	Bcer	FALSE	Bc_Ga0417192_02_rn03	Other	NA
Bc_ctg2_46	B4	0	Bcer	NA	Bcer	FALSE	NA	NA	NA
Bc_ctg2_460	B4	0.111594243	Bcer	G	Bcer	FALSE	Bc_Ga0417192_02_rn03	Ancillary biosynthesis	SMCOG1101
Bc_ctg2_462	B4	0.892753947	Bcer	A	Bcer	FALSE	Bc_Ga0417192_02_rn03	Other	NA
Bc_ctg2_464	B4	0	Bcer	NA	Bcer	FALSE	NA	NA	NA
Bc_ctg2_465	B4	1.897102138	Bcer	NA	Bcer	FALSE	NA	NA	NA
Bc_ctg2_466	B4	4.575363979	Bcer	NA	Bcer	FALSE	NA	NA	NA
Bc_ctg2_467	B4	3.571015789	Bcer	NA	Bcer	FALSE	NA	NA	NA
Bc_ctg2_468	B4	0	Bcer	NA	Bcer	FALSE	NA	NA	NA
Bc_ctg2_469	B4	0.781159704	Bcer	L	Bcer	FALSE	NA	NA	NA
Bc_ctg2_47	B4	9.150727958	Bcer	V	Bcer	FALSE	NA	NA	NA
Bc_ctg2_471	B4	10.48985888	Bcer	NA	Bcer	FALSE	NA	NA	NA
Bc_ctg2_472	B4	3.347827302	Bcer	S	Bcer	FALSE	NA	NA	NA
Bc_ctg2_473	B4	39.05798519	Bcer	S	Bcer	FALSE	NA	NA	NA
Bc_ctg2_474	B4	8.815945228	Bcer	NA	Bcer	FALSE	NA	NA	NA
Bc_ctg2_475	B4	0	Bcer	L	Bcer	FALSE	NA	NA	NA
Bc_ctg2_476	B4	4.686958222	Bcer	L	Bcer	FALSE	NA	NA	NA
Bc_ctg2_477	B4	0	Bcer	NA	Bcer	FALSE	NA	NA	NA
Bc_ctg2_478	B4	0	Bcer	L	Bcer	FALSE	NA	NA	NA
Bc_ctg2_479	B4	0.446376974	Bcer	L	Bcer	FALSE	NA	NA	NA
Bc_ctg2_48	B4	9.262322201	Bcer	M	Bcer	FALSE	NA	NA	NA
Bc_ctg2_480	B4	0.781159704	Bcer	L	Bcer	FALSE	NA	NA	NA
Bc_ctg2_481	B4	0.33478273	Bcer	L	Bcer	FALSE	NA	NA	NA
Bc_ctg2_482	B4	0.223188487	Bcer	NA	Bcer	FALSE	NA	NA	NA
Bc_ctg2_483	B4	0.66956546	Bcer	NA	Bcer	FALSE	NA	NA	NA
Bc_ctg2_484	B4	2.343479111	Bcer	NA	Bcer	FALSE	NA	NA	NA
Bc_ctg2_485	B4	0.892753947	Bcer	O	Bcer	FALSE	NA	NA	NA
Bc_ctg2_486	B4	0.557971217	Bcer	K	Bcer	FALSE	NA	NA	NA
Bc_ctg2_487	B4	26.2246472	Bcer	S	Bcer	FALSE	NA	NA	NA

Bc_ctg2_488	B4	0.111594243	Bcer	NA	Bcer	FALSE	NA	NA	NA
Bc_ctg2_489	B4	0.223188487	Bcer	NA	Bcer	FALSE	NA	NA	NA
[truncated: 656,264 more chars]
